# Supplementary material for: Lifestyle elements and risk of metabolic syndrome in adults
Source: PLoS One. 2022 Sep 30;17(9):e0275510. doi: 10.1371/journal.pone.0275510 (PMC9524662; doi:10.1371/journal.pone.0275510)
Supplement: S1 File — (PDF) [file pone.0275510.s001.pdf]

var 5	var 6	var 7	var 8	var 9	var 10	var 11	var 12	var 13
0	1	1	trzy i więce	2	60	8,57143	0	8,57143
0	0	1	dwa czynni	3	240	34,2857	17,1429	17,1429
0	0	1	dwa czynni	3	90	12,8571	12,8571	0
0	0	1	dwa czynni	2	0	0	0	0
0	0	1	trzy i więce	3	20	2,85714	0	2,85714
0	1	1	trzy i więce	3	0	0	0	0
0	1	1	trzy i więce	3	0	0	0	0
1	1	1	trzy i więce	3	180	25,7143	0	25,7143
0	0	1	dwa czynni	1	180	25,7143	0	25,7143
0	1	1	trzy i więce	2	120	17,1429	0	17,1429
1	0	1	trzy i więce	3	0	0	0	0
0	1	1	trzy i więce	3	0	0	0	0
0	0	0	metabolicz	3	240	34,2857	0	34,2857
0	1	0	jeden czyn	2	0	0	0	0
1	1	1	trzy i więce	3	140	20	20	0
0	1	0	jeden czyn	3	240	34,2857	34,2857	0
1	1	1	trzy i więce	3	600	85,7143	0	85,7143
0	0	1	dwa czynni	2	0	0	0	0
0	1	1	dwa czynni	2	780	111,429	111,429	0
0	0	1	dwa czynni	3	360	51,4286	21,4286	30
0	1	1	trzy i więce	3	120	17,1429	17,1429	0
0	0	1	dwa czynni	2	150	21,4286	0	21,4286
0	1	1	trzy i więce	3	0	0	0	0
1	1	1	trzy i więce	1	900	128,571	0	128,571
0	0	1	jeden czyn	2	360	51,4286	0	51,4286
1	1	1	trzy i więce	1	180	25,7143	0	25,7143
0	0	1	trzy i więce	1	480	68,5714	60	8,57143
0	1	1	trzy i więce	3	0	0	0	0
0	0	0	metabolicz	3	0	0	0	0
0	0	0	metabolicz	2	0	0	0	0
0	0	1	jeden czyn	1	1260	180	0	180
0	0	1	jeden czyn	3	960	137,143	17,1429	120
0	0	1	trzy i więce	2	240	34,2857	0	34,2857
0	1	1	trzy i więce	3	0	0	0	0
0	1	1	trzy i więce	3	0	0	0	0
1	1	1	trzy i więce	2	450	64,2857	12,8571	51,4286
0	1	1	trzy i więce	2	210	30	0	30
0	0	1	jeden czyn	3	300	42,8571	0	42,8571
0	1	1	trzy i więce	3	360	51,4286	0	51,4286
0	1	1	trzy i więce	1	420	60	60	0
0	1	1	dwa czynni	3	30	4,28571	0	4,28571
1	1	1	trzy i więce	2	0	0	0	0
1	0	1	trzy i więce	3	120	17,1429	0	17,1429
1	0	1	trzy i więce	2	120	17,1429	0	17,1429
1	1	1	trzy i więce	2	0	0	0	0
1	1	0	trzy i więce	3	345	49,2857	15	34,2857
0	1	1	trzy i więce	2	30	4,28571	4,28571	0

1	0	1	trzy i więc	1	60	8,57143	0	8,57143
1	0	1	trzy i więc	2	0	0	0	0
1	0	1	dwa czynni	3	495	70,7143	45	25,7143
1	1	1	trzy i więc	1	480	68,5714	0	68,5714
0	1	1	trzy i więc	3	420	60	51,4286	8,57143
0	0	1	dwa czynni	2	180	25,7143	0	25,7143
0	1	1	trzy i więc	3	210	30	10	20
1	0	1	trzy i więc	3	0	0	0	0
0	0	0	dwa czynni	3	60	8,57143	0	8,57143
0	1	1	trzy i więc	2	180	25,7143	0	25,7143
1	0	1	trzy i więc	2	280	40	0	40
0	0	1	dwa czynni	3	0	0	0	0
1	0	1	trzy i więc	2	0	0	0	0
0	1	0	jeden czyn	2	210	30	30	0
0	0	1	dwa czynni	2	0	0	0	0
0	0	1	dwa czynni	2	60	8,57143	0	8,57143
0	0	1	dwa czynni	3	120	17,1429	17,1429	0
0	0	0	jeden czyn	3	120	17,1429	0	17,1429
0	1	1	dwa czynni	3	0	0	0	0
1	0	0	jeden czyn	1	60	8,57143	0	8,57143
0	0	0	jeden czyn	3	120	17,1429	0	17,1429
0	1	1	trzy i więc	2	720	102,857	60	42,8571
0	1	1	dwa czynni	2	360	51,4286	42,8571	8,57143
0	1	0	dwa czynni	1	980	140	20	120
0	1	0	dwa czynni	3	210	30	0	30
1	1	1	trzy i więc	3	300	42,8571	0	42,8571
0	1	1	trzy i więc	2	180	25,7143	0	25,7143
0	1	1	trzy i więc	2	0	0	0	0
1	0	1	trzy i więc	2	420	60	0	60
1	1	1	trzy i więc	1	90	12,8571	0	12,8571
0	0	1	dwa czynni	2	0	0	0	0
0	1	1	trzy i więc	3	30	4,28571	0	4,28571
1	1	1	trzy i więc	2	0	0	0	0
0	1	1	trzy i więc	2	360	51,4286	0	51,4286
0	1	1	trzy i więc	1	60	8,57143	4,28571	4,28571
0	1	1	trzy i więc	3	420	60	0	60
1	1	1	trzy i więc	3	90	12,8571	0	12,8571
0	1	1	trzy i więc	2	0	0	0	0
0	0	1	dwa czynni	3	0	0	0	0
0	0	1	trzy i więc	3	720	102,857	0	102,857
0	0	1	jeden czyn	3	600	85,7143	0	85,7143
0	1	1	trzy i więc	1	0	0	0	0
0	0	1	jeden czyn	1	0	0	0	0
1	1	1	trzy i więc	2	0	0	0	0
1	1	1	trzy i więc	2	0	0	0	0
0	0	1	dwa czynni	2	0	0	0	0
0	0	1	dwa czynni	2	60	8,57143	0	8,57143
1	1	1	trzy i więc	1	240	34,2857	0	34,2857

0	1	1	trzy i więce	2	120	17,1429	0	17,1429
0	0	0	metabolicz	3	0	0	0	0
1	0	1	trzy i więce	1	840	120	0	120
1	0	1	trzy i więce	3	370	52,8571	10	42,8571
0	0	1	jeden czyn	3	210	30	0	30
0	0	1	dwa czynni	3	0	0	0	0
1	1	1	trzy i więce	1	240	34,2857	0	34,2857
0	1	1	trzy i więce	2	210	30	0	30
0	1	1	trzy i więce	3	600	85,7143	17,1429	68,5714
0	0	1	jeden czyn	3	0	0	0	0
1	0	1	trzy i więce	3	840	120	60	60
0	0	1	dwa czynni	1	0	0	0	0
0	0	1	dwa czynni	2	120	17,1429	0	17,1429
0	0	0	jeden czyn	3	0	0	0	0
0	0	1	jeden czyn	1	0	0	0	0
0	0	0	dwa czynni	3	0	0	0	0
0	1	1	trzy i więce	2	720	102,857	0	102,857
0	1	1	trzy i więce	3	720	102,857	0	102,857
0	1	1	trzy i więce	3	90	12,8571	12,8571	0
1	1	1	trzy i więce	3	30	4,28571	4,28571	0
1	0	1	trzy i więce	3	340	48,5714	5,71429	42,8571
0	0	1	dwa czynni	2	0	0	0	0
0	1	1	dwa czynni	2	480	68,5714	25,7143	42,8571
1	1	1	trzy i więce	3	180	25,7143	0	25,7143
1	1	1	trzy i więce	3	0	0	0	0
0	1	1	trzy i więce	2	0	0	0	0
0	0	1	jeden czyn	2	170	24,2857	17,1429	7,14286
1	1	1	trzy i więce	3	0	0	0	0
0	1	1	trzy i więce	1	210	30	0	30
1	1	1	trzy i więce	2	240	34,2857	8,57143	25,7143
0	0	1	dwa czynni	3	0	0	0	0
1	0	1	trzy i więce	1	0	0	0	0
1	1	1	trzy i więce	3	0	0	0	0
1	0	1	trzy i więce	2	330	47,1429	21,4286	25,7143
0	1	0	dwa czynni	3	0	0	0	0
0	0	0	jeden czyn	2	40	5,71429	0	5,71429
0	1	1	trzy i więce	2	70	10	10	0
0	1	1	trzy i więce	3	150	21,4286	0	21,4286
0	1	1	trzy i więce	3	240	34,2857	17,1429	17,1429
1	1	1	trzy i więce	3	360	51,4286	0	51,4286
0	0	1	dwa czynni	3	120	17,1429	0	17,1429
1	0	0	trzy i więce	1	0	0	0	0
0	1	0	dwa czynni	3	0	0	0	0
1	1	1	trzy i więce	3	540	77,1429	77,1429	0
0	0	0	metabolicz	2	135	19,2857	15	4,28571
1	0	1	trzy i więce	3	0	0	0	0
0	1	1	trzy i więce	2	0	0	0	0
0	0	1	dwa czynni	3	0	0	0	0

1	*1*	*1*	trzy i więce	3	*0*	*0*	*0*	*0*
0	*1*	*1*	trzy i więce	3	*0*	*0*	*0*	*0*
0	*1*	*1*	trzy i więce	2	*240*	*34,2857*	*0*	*34,2857*
0	*1*	*1*	trzy i więce	2	*90*	*12,8571*	*0*	*12,8571*
0	*0*	*1*	dwa czynni	2	*0*	*0*	*0*	*0*
0	*0*	*1*	trzy i więce	2	*840*	*120*	*0*	*120*
0	*0*	*1*	trzy i więce	3	*360*	*51,4286*	*0*	*51,4286*
0	*0*	*1*	jeden czyn	1	*110*	*15,7143*	*7,14286*	*8,57143*
0	*0*	*1*	dwa czynni	2	*0*	*0*	*0*	*0*
0	*1*	*1*	dwa czynni	2	*180*	*25,7143*	*25,7143*	*0*
0	*0*	*1*	jeden czyn	2	*0*	*0*	*0*	*0*
0	*0*	*1*	dwa czynni	3	*60*	*8,57143*	*0*	*8,57143*
0	*0*	*1*	dwa czynni	2	*40*	*5,71429*	*0*	*5,71429*
0	*0*	*1*	dwa czynni	1	*0*	*0*	*0*	*0*
0	*0*	*1*	dwa czynni	1	*315*	*45*	*0*	*45*
0	*0*	*1*	dwa czynni	1	*60*	*8,57143*	*0*	*8,57143*
0	*1*	*1*	trzy i więce	1	*30*	*4,28571*	*0*	*4,28571*
0	*1*	*1*	dwa czynni	1	*420*	*60*	*0*	*60*
0	*0*	*1*	dwa czynni	3	*300*	*42,8571*	*0*	*42,8571*
0	*0*	*1*	jeden czyn	2	*840*	*120*	*60*	*60*
0	*0*	*1*	trzy i więce	3	*0*	*0*	*0*	*0*
1	*0*	*1*	trzy i więce	3	*360*	*51,4286*	*0*	*51,4286*
0	*0*	*1*	dwa czynni	2	*120*	*17,1429*	*0*	*17,1429*
0	*0*	*1*	jeden czyn	3	*0*	*0*	*0*	*0*
1	*1*	*1*	trzy i więce	1	*600*	*85,7143*	*60*	*25,7143*
1	*0*	*1*	trzy i więce	2	*0*	*0*	*0*	*0*
1	*1*	*1*	trzy i więce	2	*90*	*12,8571*	*0*	*12,8571*
0	*1*	*1*	trzy i więce	1	*380*	*54,2857*	*2,85714*	*51,4286*
1	*1*	*1*	trzy i więce	3	*0*	*0*	*0*	*0*
0	*0*	*0*	metabolicz	2	*60*	*8,57143*	*0*	*8,57143*
1	*1*	*1*	trzy i więce	1	*80*	*11,4286*	*2,85714*	*8,57143*
0	*0*	*1*	dwa czynni	2	*0*	*0*	*0*	*0*
1	*0*	*1*	trzy i więce	1	*420*	*60*	*8,57143*	*51,4286*
0	*0*	*1*	jeden czyn	2	*200*	*28,5714*	*11,4286*	*17,1429*
0	*0*	*1*	trzy i więce	3	*150*	*21,4286*	*0*	*21,4286*
0	*0*	*1*	jeden czyn	1	*0*	*0*	*0*	*0*
1	*1*	*1*	trzy i więce	3	*120*	*17,1429*	*0*	*17,1429*
0	*1*	*1*	trzy i więce	3	*0*	*0*	*0*	*0*
0	*0*	*1*	dwa czynni	3	*0*	*0*	*0*	*0*
0	*1*	*1*	trzy i więce	3	*0*	*0*	*0*	*0*
0	*0*	*1*	trzy i więce	2	*180*	*25,7143*	*0*	*25,7143*
0	*1*	*1*	dwa czynni	2	*360*	*51,4286*	*51,4286*	*0*
0	*1*	*1*	trzy i więce	1	*360*	*51,4286*	*0*	*51,4286*
0	*0*	*1*	jeden czyn	3	*180*	*25,7143*	*25,7143*	*0*
0	*0*	*1*	dwa czynni	2	*130*	*18,5714*	*10*	*8,57143*
0	*1*	*1*	trzy i więce	3	*240*	*34,2857*	*0*	*34,2857*
0	*0*	*1*	dwa czynni	3	*0*	*0*	*0*	*0*
0	*0*	*1*	dwa czynni	2	*0*	*0*	*0*	*0*

0	1	1	trzy i więc	3	420	60	0	60
1	0	1	trzy i więc	3	60	8,57143	0	8,57143
0	0	0	metabolicz	2	0	0	0	0
0	1	1	trzy i więc	3	60	8,57143	0	8,57143
0	1	1	dwa czynni	3	0	0	0	0
0	0	1	dwa czynni	2	0	0	0	0
0	1	0	jeden czyn	3	480	68,5714	0	68,5714
0	0	1	dwa czynni	3	140	20	20	0
1	1	1	trzy i więc	1	420	60	0	60
1	0	1	trzy i więc	3	0	0	0	0
0	1	1	dwa czynni	3	840	120	0	120
0	1	1	trzy i więc	1	180	25,7143	0	25,7143
0	0	0	jeden czyn	2	75	10,7143	2,14286	8,57143
1	0	0	dwa czynni	1	0	0	0	0
1	1	1	trzy i więc	2	1260	180	0	180
1	0	1	trzy i więc	3	150	21,4286	21,4286	0
0	0	1	dwa czynni	2	120	17,1429	0	17,1429
1	0	1	trzy i więc	3	60	8,57143	0	8,57143
0	1	1	trzy i więc	3	0	0	0	0
0	0	1	jeden czyn	2	0	0	0	0
1	1	1	trzy i więc	2	420	60	8,57143	51,4286
0	1	1	trzy i więc	3	240	34,2857	34,2857	0
0	0	1	trzy i więc	3	0	0	0	0
1	1	1	trzy i więc	3	40	5,71429	0	5,71429
0	1	1	trzy i więc	2	60	8,57143	0	8,57143
0	0	1	jeden czyn	1	0	0	0	0
0	0	1	jeden czyn	2	135	19,2857	0	19,2857
0	0	0	metabolicz	2	840	120	0	120
1	0	1	trzy i więc	3	120	17,1429	17,1429	0
1	1	1	trzy i więc	3	150	21,4286	4,28571	17,1429
0	1	1	trzy i więc	3	0	0	0	0
0	0	0	metabolicz	2	360	51,4286	51,4286	0
1	1	0	trzy i więc	2	0	0	0	0
0	0	1	dwa czynni	2	60	8,57143	8,57143	0
0	0	1	trzy i więc	1	240	34,2857	0	34,2857
0	0	1	dwa czynni	2	360	51,4286	0	51,4286
0	1	1	dwa czynni	1	450	64,2857	64,2857	0
1	1	1	trzy i więc	2	195	27,8571	10,7143	17,1429
0	0	0	jeden czyn	3	0	0	0	0
1	1	1	trzy i więc	2	120	17,1429	0	17,1429
1	1	1	trzy i więc	2	0	0	0	0
0	0	1	dwa czynni	3	0	0	0	0
0	0	1	trzy i więc	3	0	0	0	0
1	1	1	trzy i więc	3	60	8,57143	0	8,57143
0	0	0	metabolicz	1	0	0	0	0
1	0	1	trzy i więc	2	210	30	0	30
0	1	0	dwa czynni	3	225	32,1429	0	32,1429
1	1	0	trzy i więc	3	120	17,1429	0	17,1429

0	0	1	dwa czynni	2	0	0	0	0
0	0	0	jeden czyn	3	0	0	0	0
1	1	1	trzy i więce	3	0	0	0	0
0	1	1	trzy i więce	3	210	30	0	30
0	0	0	jeden czyn	3	180	25,7143	0	25,7143
0	1	1	trzy i więce	1	600	85,7143	0	85,7143
0	0	1	trzy i więce	2	0	0	0	0
0	1	1	trzy i więce	1	0	0	0	0
0	0	1	dwa czynni	3	0	0	0	0
1	0	1	trzy i więce	3	480	68,5714	34,2857	34,2857
1	1	1	trzy i więce	3	0	0	0	0
0	1	1	dwa czynni	3	420	60	0	60
1	1	1	trzy i więce	3	240	34,2857	0	34,2857
1	1	1	trzy i więce	1	210	30	0	30
0	1	0	jeden czyn	2	420	60	60	0
0	1	1	trzy i więce	2	360	51,4286	17,1429	34,2857
1	0	1	trzy i więce	2	60	8,57143	0	8,57143
0	0	1	dwa czynni	3	210	30	0	30
1	0	1	trzy i więce	3	0	0	0	0
0	1	1	trzy i więce	3	720	102,857	0	102,857
0	0	0	dwa czynni	3	420	60	0	60
1	1	1	trzy i więce	3	0	0	0	0
0	1	1	trzy i więce	2	0	0	0	0
0	0	1	dwa czynni	2	180	25,7143	0	25,7143
1	1	1	trzy i więce	2	300	42,8571	17,1429	25,7143
0	1	1	trzy i więce	3	0	0	0	0
0	1	1	trzy i więce	3	1050	150	60	90
0	0	1	dwa czynni	1	60	8,57143	0	8,57143
0	1	1	trzy i więce	2	0	0	0	0
0	0	0	jeden czyn	2	300	42,8571	25,7143	17,1429
0	0	1	jeden czyn	3	420	60	0	60
0	1	0	dwa czynni	3	0	0	0	0
0	1	1	trzy i więce	3	60	8,57143	0	8,57143
0	0	1	trzy i więce	3	840	120	0	120
0	0	1	trzy i więce	1	0	0	0	0
1	0	1	trzy i więce	3	210	30	4,28571	25,7143
0	1	1	trzy i więce	2	0	0	0	0
0	1	1	dwa czynni	2	90	12,8571	12,8571	0
0	0	1	dwa czynni	1	0	0	0	0
1	1	1	trzy i więce	3	0	0	0	0
0	1	1	trzy i więce	3	90	12,8571	0	12,8571
0	0	1	dwa czynni	2	120	17,1429	0	17,1429
0	0	1	dwa czynni	2	0	0	0	0
0	1	1	trzy i więce	1	360	51,4286	0	51,4286
0	0	1	dwa czynni	2	60	8,57143	0	8,57143
0	1	1	trzy i więce	2	50	7,14286	0	7,14286
1	1	0	trzy i więce	3	1680	240	120	120
0	0	1	jeden czyn	2	480	68,5714	51,4286	17,1429

1	1	1	trzy i więce	1	360	51,4286	0	51,4286
0	0	0	jeden czyn	3	840	120	0	120
0	1	1	trzy i więce	2	690	98,5714	72,8571	25,7143
0	1	1	dwa czynni	3	450	64,2857	30	34,2857
0	0	1	dwa czynni	2	45	6,42857	6,42857	0
0	1	1	trzy i więce	2	300	42,8571	0	42,8571
0	0	1	dwa czynni	1	0	0	0	0
0	1	0	trzy i więce	3	330	47,1429	30	17,1429
0	1	1	dwa czynni	3	0	0	0	0
0	0	1	jeden czyn	1	385	55	15	40
1	1	1	trzy i więce	3	0	0	0	0
1	0	1	trzy i więce	2	0	0	0	0
0	0	1	dwa czynni	3	0	0	0	0
0	0	1	jeden czyn	2	0	0	0	0
1	1	1	trzy i więce	2	0	0	0	0
0	0	1	dwa czynni	3	0	0	0	0
0	1	1	trzy i więce	3	360	51,4286	0	51,4286
0	1	1	trzy i więce	3	100	14,2857	0	14,2857
0	0	0	jeden czyn	3	200	28,5714	20	8,57143
0	1	1	trzy i więce	3	250	35,7143	0	35,7143
0	1	1	trzy i więce	2	0	0	0	0
1	1	0	trzy i więce	1	180	25,7143	0	25,7143
0	0	1	dwa czynni	3	120	17,1429	0	17,1429
0	0	1	jeden czyn	2	90	12,8571	12,8571	0
0	1	1	trzy i więce	2	0	0	0	0
0	1	1	trzy i więce	1	240	34,2857	21,4286	12,8571
1	1	1	trzy i więce	3	0	0	0	0
0	1	1	trzy i więce	2	0	0	0	0
1	1	1	trzy i więce	3	260	37,1429	20	17,1429
0	1	1	trzy i więce	2	0	0	0	0
1	0	1	trzy i więce	3	30	4,28571	0	4,28571
0	1	1	trzy i więce	3	1020	145,714	25,7143	120
0	1	1	dwa czynni	1	0	0	0	0
1	0	1	trzy i więce	1	120	17,1429	0	17,1429
0	0	1	dwa czynni	3	60	8,57143	0	8,57143
1	1	1	trzy i więce	1	240	34,2857	8,57143	25,7143
0	0	1	jeden czyn	1	60	8,57143	0	8,57143
1	0	0	trzy i więce	1	300	42,8571	0	42,8571
0	1	1	trzy i więce	2	300	42,8571	12,8571	30
0	1	1	dwa czynni	2	535	76,4286	25	51,4286
1	1	1	trzy i więce	3	420	60	0	60
0	1	1	trzy i więce	3	270	38,5714	21,4286	17,1429
1	0	1	trzy i więce	3	560	80	20	60
0	1	1	trzy i więce	3	0	0	0	0
0	1	1	trzy i więce	3	0	0	0	0
0	0	1	jeden czyn	3	180	25,7143	0	25,7143
0	0	1	dwa czynni	1	0	0	0	0
0	0	0	jeden czyn	2	0	0	0	0

1	0	1	trzy i więce	2	60	8,57143	0	8,57143
0	0	1	dwa czynni	2	60	8,57143	8,57143	0
0	0	1	jeden czyn	2	0	0	0	0
0	0	1	dwa czynni	2	0	0	0	0
0	1	1	dwa czynni	1	0	0	0	0
0	1	1	trzy i więce	3	80	11,4286	0	11,4286
0	0	1	dwa czynni	1	60	8,57143	0	8,57143
0	0	1	dwa czynni	2	120	17,1429	0	17,1429
0	0	0	metabolicz	2	150	21,4286	8,57143	12,8571
0	1	1	trzy i więce	3	0	0	0	0
1	0	1	trzy i więce	3	300	42,8571	0	42,8571
0	1	1	trzy i więce	3	180	25,7143	0	25,7143
0	0	1	trzy i więce	2	450	64,2857	30	34,2857
0	1	1	trzy i więce	3	20	2,85714	0	2,85714
1	1	1	trzy i więce	3	0	0	0	0
0	1	1	trzy i więce	1	0	0	0	0
0	1	1	trzy i więce	3	1080	154,286	154,286	0
1	1	1	trzy i więce	3	420	60	0	60
0	1	1	trzy i więce	3	120	17,1429	0	17,1429
0	0	1	dwa czynni	3	420	60	0	60
0	0	0	jeden czyn	3	60	8,57143	0	8,57143
0	0	1	dwa czynni	3	0	0	0	0
0	1	1	trzy i więce	3	70	10	10	0
1	1	1	trzy i więce	3	30	4,28571	0	4,28571
0	0	1	dwa czynni	3	0	0	0	0
0	0	1	dwa czynni	1	0	0	0	0
0	1	1	trzy i więce	3	420	60	0	60
1	1	1	trzy i więce	3	0	0	0	0
0	1	1	trzy i więce	3	40	5,71429	0	5,71429
0	1	1	trzy i więce	1	0	0	0	0
0	1	1	trzy i więce	3	240	34,2857	0	34,2857
0	0	1	dwa czynni	3	0	0	0	0
0	0	1	trzy i więce	3	420	60	0	60
0	1	0	dwa czynni	3	630	90	0	90
0	1	1	trzy i więce	3	1260	180	0	180
1	1	1	trzy i więce	3	0	0	0	0
0	1	1	trzy i więce	3	120	17,1429	0	17,1429
0	1	1	trzy i więce	2	0	0	0	0
0	1	1	trzy i więce	3	120	17,1429	0	17,1429
1	0	1	trzy i więce	1	0	0	0	0
1	1	1	trzy i więce	3	240	34,2857	0	34,2857
0	0	1	jeden czyn	3	120	17,1429	0	17,1429
1	1	1	trzy i więce	3	120	17,1429	0	17,1429
0	0	1	dwa czynni	3	0	0	0	0
0	0	1	trzy i więce	3	480	68,5714	17,1429	51,4286
0	0	1	jeden czyn	1	2280	325,714	265,714	60
0	0	0	jeden czyn	3	240	34,2857	0	34,2857
0	0	1	dwa czynni	2	120	17,1429	0	17,1429

1	0	1	dwa czynni	2	0	0	0	0
0	0	1	dwa czynni	3	360	51,4286	25,7143	25,7143
0	0	1	dwa czynni	2	60	8,57143	0	8,57143
1	1	1	trzy i więc	1	300	42,8571	8,57143	34,2857
0	1	1	trzy i więc	2	100	14,2857	14,2857	0
0	1	1	trzy i więc	1	120	17,1429	0	17,1429
0	1	1	dwa czynni	1	630	90	60	30
0	1	1	trzy i więc	2	360	51,4286	0	51,4286
0	0	0	metabolicz	3	165	23,5714	15	8,57143
0	0	1	dwa czynni	1	420	60	0	60
1	0	1	trzy i więc	3	420	60	0	60
0	0	1	dwa czynni	3	600	85,7143	60	25,7143
0	1	0	dwa czynni	3	420	60	0	60
1	1	1	trzy i więc	3	300	42,8571	0	42,8571
1	0	1	trzy i więc	3	10	1,42857	1,42857	0
0	0	0	metabolicz	1	1680	240	60	180
0	1	1	dwa czynni	2	50	7,14286	0	7,14286
0	0	1	jeden czyn	1	60	8,57143	0	8,57143
0	1	1	trzy i więc	3	180	25,7143	0	25,7143
0	1	1	trzy i więc	2	90	12,8571	0	12,8571
0	0	1	trzy i więc	3	0	0	0	0
0	1	1	trzy i więc	2	0	0	0	0
0	0	1	jeden czyn	3	500	71,4286	11,4286	60
1	1	1	trzy i więc	2	180	25,7143	0	25,7143
1	1	1	trzy i więc	1	80	11,4286	11,4286	0
1	0	1	trzy i więc	3	720	102,857	0	102,857
0	0	1	dwa czynni	3	0	0	0	0
0	0	1	dwa czynni	3	255	36,4286	6,42857	30
0	0	0	jeden czyn	3	420	60	0	60
1	0	1	trzy i więc	3	0	0	0	0
0	1	1	trzy i więc	1	0	0	0	0
0	0	1	dwa czynni	2	630	90	30	60
0	0	0	jeden czyn	3	60	8,57143	8,57143	0
0	1	1	trzy i więc	3	0	0	0	0
0	0	1	jeden czyn	1	0	0	0	0
0	1	1	trzy i więc	2	1260	180	0	180
1	1	1	trzy i więc	2	0	0	0	0
0	0	0	metabolicz	3	735	105	53,5714	51,4286
0	0	1	trzy i więc	1	0	0	0	0
0	0	1	jeden czyn	3	840	120	0	120
0	1	1	trzy i więc	2	600	85,7143	60	25,7143
0	1	1	trzy i więc	3	1440	205,714	128,571	77,1429
0	1	1	trzy i więc	3	0	0	0	0
1	0	1	trzy i więc	3	0	0	0	0
0	0	1	dwa czynni	1	120	17,1429	17,1429	0
0	0	1	trzy i więc	3	360	51,4286	0	51,4286
0	0	1	dwa czynni	3	420	60	60	0
0	0	0	jeden czyn	2	0	0	0	0

0	0	1	trzy i więce	2	240	34,2857	0	34,2857
1	0	1	trzy i więce	3	0	0	0	0
1	0	1	trzy i więce	2	240	34,2857	0	34,2857
0	1	0	dwa czynni	1	0	0	0	0
0	0	0	metabolicz	2	840	120	0	120
0	1	1	trzy i więce	3	420	60	0	60
0	1	0	dwa czynni	3	0	0	0	0
1	0	1	trzy i więce	3	150	21,4286	0	21,4286
0	0	1	dwa czynni	3	80	11,4286	0	11,4286
0	0	1	dwa czynni	3	0	0	0	0
0	1	1	trzy i więce	2	60	8,57143	0	8,57143
0	1	1	dwa czynni	3	210	30	0	30
0	1	1	trzy i więce	3	150	21,4286	0	21,4286
1	0	1	trzy i więce	3	50	7,14286	0	7,14286
0	1	1	trzy i więce	2	20	2,85714	2,85714	0
0	0	1	trzy i więce	3	0	0	0	0
0	1	1	trzy i więce	1	0	0	0	0
0	1	1	trzy i więce	3	210	30	0	30
0	1	1	trzy i więce	2	560	80	20	60
1	1	1	trzy i więce	1	0	0	0	0
1	1	1	trzy i więce	3	180	25,7143	4,28571	21,4286
1	1	0	dwa czynni	1	60	8,57143	0	8,57143
1	1	1	trzy i więce	3	45	6,42857	0	6,42857
1	0	1	trzy i więce	3	840	120	0	120
0	1	1	dwa czynni	2	180	25,7143	0	25,7143
0	0	1	trzy i więce	2	0	0	0	0
0	0	1	dwa czynni	2	0	0	0	0
0	1	1	dwa czynni	2	0	0	0	0
0	0	0	jeden czyn	2	330	47,1429	12,8571	34,2857
0	1	1	trzy i więce	3	300	42,8571	12,8571	30
0	0	1	dwa czynni	1	60	8,57143	0	8,57143
0	0	1	dwa czynni	3	30	4,28571	0	4,28571
0	0	0	jeden czyn	2	20	2,85714	0	2,85714
0	1	1	trzy i więce	3	130	18,5714	12,8571	5,71429
0	0	1	jeden czyn	2	130	18,5714	10	8,57143
1	1	0	trzy i więce	3	0	0	0	0
0	0	1	jeden czyn	3	360	51,4286	34,2857	17,1429
0	0	1	dwa czynni	3	0	0	0	0
1	1	1	trzy i więce	1	0	0	0	0
0	0	1	jeden czyn	1	0	0	0	0
0	1	1	trzy i więce	3	30	4,28571	0	4,28571
0	1	1	trzy i więce	1	430	61,4286	1,42857	60
0	1	1	dwa czynni	1	120	17,1429	17,1429	0
0	1	1	trzy i więce	1	210	30	0	30
0	1	1	trzy i więce	2	120	17,1429	0	17,1429
1	1	1	trzy i więce	2	120	17,1429	0	17,1429
0	0	1	dwa czynni	2	50	7,14286	0	7,14286
1	0	1	trzy i więce	3	0	0	0	0

1	0	1	trzy i więce	2	0	0	0	0
0	0	1	dwa czynni	2	120	17,1429	0	17,1429
0	1	1	dwa czynni	3	360	51,4286	0	51,4286
0	1	1	trzy i więce	1	420	60	0	60
0	0	1	trzy i więce	3	150	21,4286	12,8571	8,57143
0	1	1	trzy i więce	1	210	30	0	30
0	0	0	jeden czyn	3	60	8,57143	0	8,57143
0	1	1	trzy i więce	3	0	0	0	0
1	1	1	trzy i więce	1	0	0	0	0
0	0	1	jeden czyn	3	180	25,7143	25,7143	0
0	0	1	dwa czynni	3	120	17,1429	0	17,1429
1	0	1	trzy i więce	1	0	0	0	0
0	1	1	dwa czynni	2	60	8,57143	0	8,57143
0	0	1	dwa czynni	2	360	51,4286	0	51,4286
0	0	1	dwa czynni	1	360	51,4286	0	51,4286
0	0	1	dwa czynni	2	405	57,8571	6,42857	51,4286
1	1	1	trzy i więce	3	0	0	0	0
0	1	1	trzy i więce	3	0	0	0	0
0	0	1	jeden czyn	1	840	120	0	120
1	1	0	trzy i więce	3	0	0	0	0
0	1	1	trzy i więce	3	0	0	0	0
1	1	1	trzy i więce	3	0	0	0	0
0	1	1	dwa czynni	2	180	25,7143	0	25,7143
0	1	1	trzy i więce	3	0	0	0	0
0	0	1	trzy i więce	1	1320	188,571	68,5714	120
0	1	1	trzy i więce	3	120	17,1429	0	17,1429
1	1	1	trzy i więce	2	0	0	0	0
1	1	1	trzy i więce	1	840	120	0	120
1	0	1	trzy i więce	2	45	6,42857	6,42857	0
0	1	1	dwa czynni	3	0	0	0	0
0	1	1	trzy i więce	2	40	5,71429	0	5,71429
1	0	1	trzy i więce	2	720	102,857	0	102,857
0	1	1	dwa czynni	1	0	0	0	0
0	1	1	trzy i więce	3	0	0	0	0
0	1	1	trzy i więce	2	30	4,28571	0	4,28571
0	0	1	trzy i więce	2	90	12,8571	0	12,8571
0	0	1	jeden czyn	1	1260	180	0	180
1	0	1	trzy i więce	2	210	30	0	30
0	1	1	trzy i więce	3	330	47,1429	30	17,1429
0	0	1	jeden czyn	3	210	30	0	30
0	0	1	dwa czynni	1	0	0	0	0
0	0	1	dwa czynni	1	1380	197,143	120	77,1429
0	1	1	trzy i więce	2	120	17,1429	0	17,1429
0	1	1	trzy i więce	3	0	0	0	0
1	1	1	trzy i więce	2	840	120	60	60
1	1	1	trzy i więce	3	30	4,28571	0	4,28571
0	0	0	jeden czyn	3	420	60	0	60
0	0	1	dwa czynni	1	1260	180	60	120

0	1	1	trzy i więce	1	60	8,57143	0	8,57143
1	1	1	trzy i więce	2	120	17,1429	0	17,1429
1	1	1	trzy i więce	3	210	30	0	30
0	0	1	dwa czynni	3	0	0	0	0
1	1	1	trzy i więce	3	0	0	0	0
0	0	1	trzy i więce	1	60	8,57143	0	8,57143
0	1	0	dwa czynni	3	120	17,1429	0	17,1429
0	0	1	jeden czyn	3	0	0	0	0
1	0	1	trzy i więce	1	0	0	0	0
1	1	1	trzy i więce	3	240	34,2857	0	34,2857
0	0	1	jeden czyn	3	70	10	1,42857	8,57143
0	0	1	jeden czyn	1	330	47,1429	12,8571	34,2857
0	0	1	dwa czynni	2	270	38,5714	38,5714	0
0	1	1	trzy i więce	3	70	10	10	0
0	0	1	jeden czyn	1	590	84,2857	50	34,2857
1	0	1	trzy i więce	2	240	34,2857	0	34,2857
0	1	1	trzy i więce	3	360	51,4286	0	51,4286
0	0	0	metabolicz	2	150	21,4286	0	21,4286
0	1	1	trzy i więce	1	60	8,57143	0	8,57143
0	0	1	jeden czyn	1	60	8,57143	0	8,57143
0	1	1	trzy i więce	3	140	20	0	20
0	0	0	metabolicz	3	0	0	0	0
1	1	1	trzy i więce	3	0	0	0	0
0	0	1	dwa czynni	3	360	51,4286	0	51,4286
1	1	1	trzy i więce	1	120	17,1429	0	17,1429
1	1	1	trzy i więce	3	150	21,4286	21,4286	0
0	1	1	trzy i więce	3	1080	154,286	0	154,286
1	1	1	trzy i więce	3	0	0	0	0
0	0	1	jeden czyn	3	0	0	0	0
1	1	1	trzy i więce	2	0	0	0	0
0	1	1	dwa czynni	3	660	94,2857	60	34,2857
0	0	1	dwa czynni	2	240	34,2857	0	34,2857
0	0	1	dwa czynni	2	165	23,5714	12,8571	10,7143
0	1	1	trzy i więce	1	30	4,28571	4,28571	0
0	1	1	trzy i więce	2	0	0	0	0
0	1	1	trzy i więce	1	0	0	0	0
0	1	1	trzy i więce	3	420	60	0	60
1	1	1	trzy i więce	1	0	0	0	0
0	1	1	trzy i więce	3	135	19,2857	15	4,28571
0	1	1	trzy i więce	2	0	0	0	0
0	1	0	dwa czynni	3	60	8,57143	0	8,57143
0	0	1	trzy i więce	3	180	25,7143	0	25,7143
0	0	1	dwa czynni	3	480	68,5714	17,1429	51,4286
0	0	0	metabolicz	2	120	17,1429	0	17,1429
0	0	1	trzy i więce	2	180	25,7143	17,1429	8,57143
1	0	1	trzy i więce	1	60	8,57143	0	8,57143
0	1	1	trzy i więce	1	120	17,1429	0	17,1429
1	1	1	trzy i więce	3	420	60	0	60

0	0	1	dwa czynni	2	135	19,2857	0	19,2857
1	1	1	trzy i więce	1	255	36,4286	15	21,4286
0	0	1	dwa czynni	3	120	17,1429	0	17,1429
0	0	1	jeden czyn	2	180	25,7143	0	25,7143
0	0	1	dwa czynni	2	0	0	0	0
1	1	1	trzy i więce	1	0	0	0	0
0	0	0	jeden czyn	3	0	0	0	0
1	1	1	trzy i więce	3	360	51,4286	0	51,4286
0	0	1	dwa czynni	2	0	0	0	0
0	0	1	dwa czynni	2	0	0	0	0
0	0	0	jeden czyn	2	0	0	0	0
0	0	0	metabolicz	3	0	0	0	0
0	1	1	trzy i więce	2	960	137,143	77,1429	60
0	1	1	trzy i więce	2	75	10,7143	6,42857	4,28571
0	0	1	jeden czyn	3	0	0	0	0
0	1	1	dwa czynni	3	0	0	0	0
0	1	1	trzy i więce	3	195	27,8571	12,8571	15
0	0	1	jeden czyn	3	135	19,2857	10,7143	8,57143
1	1	0	dwa czynni	3	0	0	0	0
0	1	1	dwa czynni	3	0	0	0	0
0	0	1	dwa czynni	2	180	25,7143	0	25,7143
0	0	0	jeden czyn	2	0	0	0	0
0	0	1	jeden czyn	3	300	42,8571	12,8571	30
0	0	1	dwa czynni	3	0	0	0	0
0	1	1	trzy i więce	3	540	77,1429	60	17,1429
0	0	0	metabolicz	3	0	0	0	0
1	1	1	trzy i więce	2	120	17,1429	0	17,1429
1	1	1	trzy i więce	1	120	17,1429	0	17,1429
0	0	0	dwa czynni	2	0	0	0	0
0	0	1	jeden czyn	2	0	0	0	0
0	0	1	trzy i więce	3	0	0	0	0
1	0	1	trzy i więce	3	105	15	0	15
0	0	1	dwa czynni	2	180	25,7143	0	25,7143
0	0	0	metabolicz	3	0	0	0	0
1	0	1	trzy i więce	3	80	11,4286	0	11,4286
0	0	1	dwa czynni	2	0	0	0	0
0	1	1	trzy i więce	3	1260	180	0	180
0	0	0	jeden czyn	3	130	18,5714	18,5714	0
0	0	1	jeden czyn	3	140	20	0	20
0	0	1	dwa czynni	2	60	8,57143	8,57143	0
0	0	1	dwa czynni	3	980	140	20	120
0	0	1	jeden czyn	2	90	12,8571	0	12,8571
1	1	1	trzy i więce	3	630	90	30	60
0	0	1	dwa czynni	2	50	7,14286	7,14286	0
0	0	1	dwa czynni	1	120	17,1429	0	17,1429
0	1	1	trzy i więce	3	100	14,2857	0	14,2857
0	0	1	jeden czyn	1	180	25,7143	0	25,7143
1	1	1	trzy i więce	3	660	94,2857	25,7143	68,5714

0	0	0	jeden czyn	1	360	51,4286	0	51,4286
0	1	1	trzy i więce	3	0	0	0	0
0	0	1	jeden czyn	3	0	0	0	0
0	1	0	trzy i więce	3	60	8,57143	0	8,57143
0	1	1	trzy i więce	2	0	0	0	0
0	1	0	dwa czynni	2	600	85,7143	25,7143	60
1	0	1	trzy i więce	2	0	0	0	0
0	1	1	trzy i więce	1	60	8,57143	0	8,57143
0	1	1	trzy i więce	2	0	0	0	0
0	0	1	dwa czynni	2	0	0	0	0
1	1	1	trzy i więce	2	180	25,7143	0	25,7143
1	0	1	trzy i więce	1	90	12,8571	4,28571	8,57143
0	1	1	dwa czynni	2	420	60	0	60
0	1	1	trzy i więce	1	60	8,57143	0	8,57143
0	0	1	dwa czynni	3	0	0	0	0
1	0	1	trzy i więce	3	0	0	0	0
0	0	1	jeden czyn	1	120	17,1429	0	17,1429
0	0	1	dwa czynni	3	120	17,1429	0	17,1429
1	1	1	trzy i więce	3	0	0	0	0
0	1	1	trzy i więce	1	180	25,7143	17,1429	8,57143
0	0	1	trzy i więce	2	180	25,7143	0	25,7143
0	0	1	jeden czyn	2	60	8,57143	0	8,57143
0	0	1	jeden czyn	2	0	0	0	0
0	1	1	trzy i więce	3	720	102,857	0	102,857
1	1	1	trzy i więce	2	0	0	0	0
1	1	1	trzy i więce	2	120	17,1429	0	17,1429
0	1	1	trzy i więce	2	0	0	0	0
0	1	1	trzy i więce	1	0	0	0	0
0	1	1	trzy i więce	3	420	60	0	60
1	0	1	trzy i więce	2	0	0	0	0
1	0	1	trzy i więce	2	210	30	0	30
0	0	0	jeden czyn	3	90	12,8571	0	12,8571
1	1	1	trzy i więce	2	105	15	6,42857	8,57143
0	0	1	dwa czynni	2	180	25,7143	0	25,7143
0	1	1	trzy i więce	2	120	17,1429	0	17,1429
1	0	1	trzy i więce	2	150	21,4286	0	21,4286
0	0	0	jeden czyn	2	135	19,2857	10,7143	8,57143
0	0	0	metabolicz	2	240	34,2857	17,1429	17,1429
1	0	1	trzy i więce	2	0	0	0	0
0	0	1	dwa czynni	2	840	120	60	60
0	0	0	metabolicz	3	60	8,57143	0	8,57143
0	0	1	trzy i więce	3	240	34,2857	0	34,2857
0	1	1	trzy i więce	3	580	82,8571	14,2857	68,5714
0	1	1	trzy i więce	2	80	11,4286	0	11,4286
1	0	1	trzy i więce	1	0	0	0	0
0	1	1	trzy i więce	3	220	31,4286	8,57143	22,8571
0	0	1	dwa czynni	3	280	40	0	40
0	1	0	trzy i więce	2	60	8,57143	8,57143	0

1	1	1	trzy i więc	3	0	0	0	0
1	0	1	trzy i więc	1	180	25,7143	0	25,7143
1	0	1	trzy i więc	1	120	17,1429	17,1429	0
0	0	1	trzy i więc	3	0	0	0	0
1	1	1	trzy i więc	3	0	0	0	0
0	0	0	jeden czyn	3	30	4,28571	4,28571	0
0	0	1	jeden czyn	2	360	51,4286	25,7143	25,7143
0	0	1	trzy i więc	3	0	0	0	0
0	0	1	dwa czynni	1	210	30	0	30
0	0	1	jeden czyn	3	540	77,1429	0	77,1429
0	0	1	jeden czyn	2	210	30	0	30
0	1	1	dwa czynni	2	560	80	20	60
1	1	1	trzy i więc	3	0	0	0	0
0	1	1	trzy i więc	3	300	42,8571	0	42,8571
1	1	1	trzy i więc	3	105	15	6,42857	8,57143
0	0	1	dwa czynni	3	0	0	0	0
0	0	1	jeden czyn	3	170	24,2857	11,4286	12,8571
0	0	1	dwa czynni	2	0	0	0	0
0	0	0	metabolicz	1	0	0	0	0
0	1	1	trzy i więc	3	300	42,8571	0	42,8571
0	0	0	jeden czyn	2	140	20	0	20
1	1	1	trzy i więc	3	0	0	0	0
0	0	1	jeden czyn	3	0	0	0	0
1	0	1	trzy i więc	3	420	60	0	60
1	1	1	trzy i więc	1	240	34,2857	0	34,2857
0	0	1	dwa czynni	3	0	0	0	0
0	1	1	trzy i więc	2	210	30	0	30
1	0	1	trzy i więc	1	0	0	0	0
0	1	1	dwa czynni	2	0	0	0	0
0	1	1	trzy i więc	2	60	8,57143	0	8,57143
0	0	1	jeden czyn	2	0	0	0	0
0	0	1	trzy i więc	2	0	0	0	0
0	0	1	dwa czynni	2	210	30	0	30
0	0	1	dwa czynni	1	0	0	0	0
0	0	1	jeden czyn	2	120	17,1429	0	17,1429
0	0	1	trzy i więc	3	0	0	0	0
0	1	1	trzy i więc	1	0	0	0	0
1	1	1	trzy i więc	3	420	60	0	60
0	1	1	trzy i więc	3	120	17,1429	0	17,1429
0	1	1	trzy i więc	3	180	25,7143	0	25,7143
0	0	1	jeden czyn	1	60	8,57143	0	8,57143
0	1	1	dwa czynni	3	420	60	0	60
0	0	1	jeden czyn	1	0	0	0	0
0	0	1	trzy i więc	2	720	102,857	0	102,857
0	1	1	trzy i więc	2	0	0	0	0
0	1	1	trzy i więc	2	180	25,7143	12,8571	12,8571
0	1	1	trzy i więc	3	0	0	0	0
1	0	1	trzy i więc	3	0	0	0	0

1	1	1	trzy i więce	3	420	60	0	60
0	1	1	trzy i więce	1	0	0	0	0
0	1	0	trzy i więce	2	0	0	0	0
0	0	1	dwa czynni	2	120	17,1429	17,1429	0
1	0	1	dwa czynni	2	120	17,1429	8,57143	8,57143
0	1	1	trzy i więce	2	0	0	0	0
1	1	1	trzy i więce	1	0	0	0	0
1	0	1	trzy i więce	3	840	120	0	120
0	1	1	trzy i więce	1	0	0	0	0
1	0	1	trzy i więce	3	60	8,57143	0	8,57143
0	1	1	dwa czynni	3	420	60	0	60
0	1	0	dwa czynni	3	210	30	21,4286	8,57143
0	0	1	dwa czynni	3	150	21,4286	4,28571	17,1429
0	0	1	trzy i więce	1	540	77,1429	0	77,1429
0	0	1	dwa czynni	3	480	68,5714	4,28571	64,2857
0	1	0	trzy i więce	2	0	0	0	0
0	0	1	dwa czynni	2	60	8,57143	0	8,57143
0	0	1	dwa czynni	3	60	8,57143	0	8,57143
0	1	1	trzy i więce	1	0	0	0	0
1	1	1	trzy i więce	1	240	34,2857	0	34,2857
0	0	0	jeden czyn	3	120	17,1429	0	17,1429
0	1	1	trzy i więce	1	360	51,4286	0	51,4286
0	1	1	trzy i więce	2	180	25,7143	0	25,7143
0	0	1	dwa czynni	3	60	8,57143	0	8,57143
0	1	1	trzy i więce	3	30	4,28571	0	4,28571
0	0	1	dwa czynni	1	0	0	0	0
0	0	1	jeden czyn	3	0	0	0	0
1	1	1	trzy i więce	3	210	30	0	30
0	1	1	dwa czynni	3	120	17,1429	0	17,1429
0	0	0	jeden czyn	1	150	21,4286	0	21,4286
0	1	1	trzy i więce	2	540	77,1429	17,1429	60
0	1	1	trzy i więce	2	0	0	0	0
0	0	1	dwa czynni	2	0	0	0	0
1	1	1	trzy i więce	3	480	68,5714	0	68,5714
1	1	0	trzy i więce	1	0	0	0	0
0	1	1	dwa czynni	1	0	0	0	0
0	1	1	trzy i więce	3	120	17,1429	0	17,1429
0	1	1	trzy i więce	2	180	25,7143	0	25,7143
0	1	1	trzy i więce	3	0	0	0	0
1	0	1	dwa czynni	1	120	17,1429	0	17,1429
0	1	1	trzy i więce	3	300	42,8571	17,1429	25,7143
0	0	1	dwa czynni	2	720	102,857	17,1429	85,7143
0	0	1	dwa czynni	3	0	0	0	0
0	0	1	dwa czynni	2	0	0	0	0
1	1	1	trzy i więce	2	60	8,57143	8,57143	0
0	0	0	jeden czyn	2	0	0	0	0
0	1	1	trzy i więce	3	360	51,4286	51,4286	0
0	1	1	dwa czynni	3	840	120	0	120

0	0	1	dwa czynni	2	0	0	0	0
1	1	1	trzy i więce	3	300	42,8571	0	42,8571
1	1	1	trzy i więce	3	0	0	0	0
0	0	1	dwa czynni	3	120	17,1429	0	17,1429
1	1	1	trzy i więce	2	0	0	0	0
0	1	0	dwa czynni	2	0	0	0	0
0	1	1	trzy i więce	2	180	25,7143	0	25,7143
0	1	1	trzy i więce	2	0	0	0	0
0	0	1	dwa czynni	3	330	47,1429	30	17,1429
0	0	1	jeden czyn	2	360	51,4286	0	51,4286
0	0	1	dwa czynni	3	1260	180	0	180
0	0	1	jeden czyn	2	190	27,1429	10	17,1429
0	0	1	jeden czyn	3	120	17,1429	0	17,1429
1	0	1	trzy i więce	3	0	0	0	0
0	1	1	trzy i więce	3	300	42,8571	0	42,8571
0	1	1	dwa czynni	3	35	5	0	5
1	1	1	trzy i więce	3	0	0	0	0
0	1	1	trzy i więce	3	0	0	0	0
0	0	1	dwa czynni	2	210	30	4,28571	25,7143
0	0	1	jeden czyn	1	0	0	0	0
0	1	1	trzy i więce	2	120	17,1429	0	17,1429
0	0	1	trzy i więce	3	300	42,8571	0	42,8571
0	0	1	jeden czyn	1	780	111,429	51,4286	60
0	0	1	dwa czynni	2	0	0	0	0
1	0	1	trzy i więce	2	0	0	0	0
0	1	1	dwa czynni	3	30	4,28571	0	4,28571
0	1	0	dwa czynni	2	120	17,1429	0	17,1429
0	1	1	trzy i więce	3	0	0	0	0
0	0	1	dwa czynni	1	0	0	0	0
1	1	0	trzy i więce	1	140	20	20	0
0	0	1	jeden czyn	2	30	4,28571	0	4,28571
0	0	0	metabolicz	2	0	0	0	0
0	0	1	jeden czyn	2	60	8,57143	0	8,57143
1	1	0	trzy i więce	3	420	60	0	60
1	0	1	trzy i więce	2	510	72,8571	12,8571	60
1	0	1	dwa czynni	3	150	21,4286	0	21,4286
0	1	0	dwa czynni	3	60	8,57143	8,57143	0
0	1	1	trzy i więce	3	60	8,57143	4,28571	4,28571
0	0	1	dwa czynni	3	0	0	0	0
1	0	0	dwa czynni	3	720	102,857	0	102,857
0	1	1	trzy i więce	3	60	8,57143	0	8,57143
1	1	1	trzy i więce	3	495	70,7143	19,2857	51,4286
1	0	1	trzy i więce	3	340	48,5714	40	8,57143
0	1	1	dwa czynni	3	60	8,57143	8,57143	0
0	1	1	trzy i więce	3	0	0	0	0
0	1	1	trzy i więce	3	0	0	0	0
1	0	1	trzy i więce	3	0	0	0	0
0	0	1	jeden czyn	1	0	0	0	0

0	1	1	trzy i więce	3	240	34,2857	0	34,2857
0	0	1	dwa czynni	3	0	0	0	0
0	0	1	dwa czynni	3	0	0	0	0
0	0	1	trzy i więce	2	20	2,85714	0	2,85714
0	1	1	trzy i więce	3	95	13,5714	7,14286	6,42857
1	0	1	dwa czynni	1	0	0	0	0
0	0	1	dwa czynni	1	120	17,1429	0	17,1429
0	0	1	jeden czyn	2	80	11,4286	0	11,4286
0	0	1	jeden czyn	3	120	17,1429	0	17,1429
0	0	1	trzy i więce	3	0	0	0	0
0	1	1	trzy i więce	3	0	0	0	0
0	0	1	dwa czynni	3	270	38,5714	12,8571	25,7143
0	0	1	dwa czynni	3	105	15	0	15
0	1	1	trzy i więce	3	120	17,1429	0	17,1429
1	0	1	trzy i więce	3	0	0	0	0
0	0	0	jeden czyn	2	120	17,1429	0	17,1429
0	0	1	trzy i więce	3	90	12,8571	0	12,8571
1	1	0	trzy i więce	3	0	0	0	0
0	1	1	trzy i więce	2	1680	240	60	180
0	0	1	dwa czynni	3	0	0	0	0
0	1	1	trzy i więce	3	0	0	0	0
1	0	1	trzy i więce	1	60	8,57143	0	8,57143
0	1	0	dwa czynni	2	150	21,4286	21,4286	0
0	1	1	dwa czynni	2	0	0	0	0
0	0	0	jeden czyn	1	60	8,57143	0	8,57143
1	1	1	trzy i więce	3	60	8,57143	0	8,57143
0	0	0	metabolicz	2	660	94,2857	34,2857	60
0	0	1	trzy i więce	3	0	0	0	0
0	0	1	trzy i więce	3	840	120	0	120
0	0	1	dwa czynni	2	180	25,7143	0	25,7143
0	1	1	trzy i więce	2	180	25,7143	0	25,7143
0	0	1	jeden czyn	1	210	30	0	30
0	0	1	jeden czyn	2	300	42,8571	34,2857	8,57143
1	1	1	trzy i więce	3	45	6,42857	6,42857	0
0	1	1	trzy i więce	3	80	11,4286	0	11,4286
0	0	1	jeden czyn	2	0	0	0	0
1	0	1	trzy i więce	3	70	10	10	0
0	1	1	trzy i więce	2	300	42,8571	0	42,8571
0	0	0	metabolicz	1	0	0	0	0
0	0	1	dwa czynni	3	0	0	0	0
0	1	1	trzy i więce	2	120	17,1429	0	17,1429
1	1	1	trzy i więce	2	80	11,4286	0	11,4286
0	1	1	trzy i więce	1	150	21,4286	0	21,4286
0	1	0	dwa czynni	2	0	0	0	0
1	1	1	trzy i więce	1	0	0	0	0
1	0	1	trzy i więce	3	0	0	0	0
0	0	1	dwa czynni	2	120	17,1429	0	17,1429
0	1	1	dwa czynni	1	0	0	0	0

0	0	1	trzy i więce	2	0	0	0	0
0	0	1	dwa czynni	2	300	42,8571	17,1429	25,7143
0	1	1	dwa czynni	2	0	0	0	0
0	0	0	metabolicz	1	480	68,5714	0	68,5714
0	0	0	metabolicz	3	0	0	0	0
0	1	0	dwa czynni	2	30	4,28571	0	4,28571
0	1	1	trzy i więce	3	120	17,1429	0	17,1429
0	0	1	dwa czynni	2	100	14,2857	0	14,2857
0	0	0	jeden czyn	3	90	12,8571	0	12,8571
0	0	1	dwa czynni	1	180	25,7143	0	25,7143
0	1	1	trzy i więce	2	420	60	60	0
0	0	1	jeden czyn	2	450	64,2857	4,28571	60
0	1	1	trzy i więce	3	180	25,7143	21,4286	4,28571
0	1	1	trzy i więce	3	420	60	0	60
0	1	1	trzy i więce	3	0	0	0	0
0	1	1	dwa czynni	2	900	128,571	51,4286	77,1429
0	1	1	trzy i więce	3	210	30	0	30
0	0	1	trzy i więce	3	120	17,1429	0	17,1429
1	0	1	trzy i więce	3	0	0	0	0
0	1	1	trzy i więce	3	480	68,5714	34,2857	34,2857
0	0	1	dwa czynni	2	0	0	0	0
0	0	0	metabolicz	1	0	0	0	0
1	1	1	trzy i więce	3	180	25,7143	0	25,7143
1	0	1	trzy i więce	3	840	120	0	120
0	1	1	dwa czynni	2	0	0	0	0
0	1	1	trzy i więce	1	900	128,571	0	128,571
0	1	1	trzy i więce	3	600	85,7143	0	85,7143
0	1	1	trzy i więce	3	60	8,57143	0	8,57143
0	0	1	dwa czynni	3	100	14,2857	14,2857	0
0	0	1	dwa czynni	2	420	60	0	60
0	1	0	trzy i więce	3	120	17,1429	8,57143	8,57143
1	1	1	trzy i więce	1	0	0	0	0
1	0	0	trzy i więce	1	0	0	0	0
0	0	0	jeden czyn	3	90	12,8571	0	12,8571
0	0	1	trzy i więce	1	60	8,57143	0	8,57143
0	1	1	trzy i więce	3	120	17,1429	0	17,1429
1	1	0	trzy i więce	2	30	4,28571	4,28571	0
0	1	1	trzy i więce	2	240	34,2857	0	34,2857
1	1	1	trzy i więce	1	0	0	0	0
1	1	1	trzy i więce	2	255	36,4286	10,7143	25,7143
0	1	1	trzy i więce	3	420	60	0	60
0	1	1	dwa czynni	3	0	0	0	0
1	0	1	trzy i więce	2	30	4,28571	0	4,28571
0	0	1	trzy i więce	3	300	42,8571	0	42,8571
1	0	1	trzy i więce	3	180	25,7143	0	25,7143
0	1	1	trzy i więce	2	0	0	0	0
0	0	1	dwa czynni	3	120	17,1429	0	17,1429
0	1	0	jeden czyn	2	0	0	0	0

0	1	0	trzy i więce	3	0	0	0	0
0	1	1	trzy i więce	1	0	0	0	0
0	0	1	jeden czyn	2	480	68,5714	17,1429	51,4286
0	0	1	jeden czyn	3	75	10,7143	10,7143	0
0	0	1	jeden czyn	2	60	8,57143	0	8,57143
1	1	1	trzy i więce	2	90	12,8571	4,28571	8,57143
0	1	0	dwa czynni	3	0	0	0	0
0	1	1	trzy i więce	3	0	0	0	0
0	1	1	trzy i więce	2	210	30	0	30
0	0	1	dwa czynni	1	0	0	0	0
0	0	1	trzy i więce	3	0	0	0	0
1	1	1	trzy i więce	3	0	0	0	0
0	1	1	trzy i więce	3	120	17,1429	0	17,1429
1	1	1	trzy i więce	2	0	0	0	0
0	0	0	jeden czyn	2	0	0	0	0
1	1	1	trzy i więce	2	240	34,2857	0	34,2857
1	0	1	trzy i więce	2	300	42,8571	42,8571	0
1	1	1	trzy i więce	2	120	17,1429	0	17,1429
0	1	1	trzy i więce	2	0	0	0	0
0	1	1	trzy i więce	2	240	34,2857	0	34,2857
1	0	1	trzy i więce	2	360	51,4286	0	51,4286
1	0	0	dwa czynni	2	225	32,1429	25,7143	6,42857
1	1	1	trzy i więce	3	120	17,1429	0	17,1429
0	1	0	dwa czynni	2	0	0	0	0
0	1	1	dwa czynni	1	0	0	0	0
0	0	0	jeden czyn	2	0	0	0	0
0	0	1	trzy i więce	3	360	51,4286	0	51,4286
0	1	1	trzy i więce	2	840	120	0	120
0	0	1	jeden czyn	3	0	0	0	0
0	0	1	dwa czynni	1	0	0	0	0
0	0	1	dwa czynni	2	60	8,57143	0	8,57143
1	1	1	trzy i więce	2	0	0	0	0
1	1	1	trzy i więce	3	240	34,2857	0	34,2857
0	1	1	dwa czynni	2	465	66,4286	15	51,4286
1	0	1	trzy i więce	3	60	8,57143	8,57143	0
1	1	1	trzy i więce	1	60	8,57143	0	8,57143
0	1	1	trzy i więce	2	180	25,7143	25,7143	0
0	1	1	trzy i więce	3	40	5,71429	0	5,71429
0	1	0	dwa czynni	2	0	0	0	0
1	1	1	trzy i więce	3	60	8,57143	0	8,57143
1	0	1	dwa czynni	3	0	0	0	0
0	0	1	trzy i więce	3	60	8,57143	0	8,57143
1	1	1	trzy i więce	3	0	0	0	0
0	0	1	jeden czyn	2	40	5,71429	5,71429	0
1	0	0	trzy i więce	1	180	25,7143	0	25,7143
1	0	1	trzy i więce	3	280	40	0	40
1	0	0	jeden czyn	2	0	0	0	0
0	0	1	trzy i więce	3	0	0	0	0

0	1	1	trzy i więce	3	960	137,143	51,4286	85,7143
0	1	1	trzy i więce	2	0	0	0	0
0	1	1	trzy i więce	3	0	0	0	0
1	0	1	trzy i więce	2	120	17,1429	17,1429	0
0	1	0	dwa czynni	1	0	0	0	0
0	0	1	dwa czynni	3	0	0	0	0
0	0	1	jeden czyn	1	0	0	0	0
0	0	1	jeden czyn	1	0	0	0	0
1	1	1	trzy i więce	3	350	50	20	30
0	1	1	trzy i więce	3	120	17,1429	0	17,1429
0	0	1	trzy i więce	3	660	94,2857	77,1429	17,1429
0	1	1	trzy i więce	3	90	12,8571	12,8571	0
0	0	1	dwa czynni	1	60	8,57143	0	8,57143
0	1	1	trzy i więce	3	0	0	0	0
0	1	1	trzy i więce	3	420	60	0	60
1	1	1	trzy i więce	3	150	21,4286	4,28571	17,1429
0	1	1	trzy i więce	3	60	8,57143	0	8,57143
0	0	1	dwa czynni	2	90	12,8571	0	12,8571
1	0	1	trzy i więce	1	120	17,1429	0	17,1429
0	1	1	trzy i więce	3	0	0	0	0
0	0	1	dwa czynni	2	0	0	0	0
1	1	1	trzy i więce	3	90	12,8571	0	12,8571
0	1	0	dwa czynni	2	210	30	0	30
0	1	1	trzy i więce	3	0	0	0	0
0	0	1	dwa czynni	3	0	0	0	0
0	1	1	trzy i więce	2	0	0	0	0
0	0	1	dwa czynni	3	540	77,1429	42,8571	34,2857
0	0	1	jeden czyn	2	0	0	0	0
0	0	1	dwa czynni	3	120	17,1429	8,57143	8,57143
1	1	1	trzy i więce	3	0	0	0	0
0	0	1	dwa czynni	3	375	53,5714	45	8,57143
0	1	1	trzy i więce	3	0	0	0	0
0	1	1	dwa czynni	3	180	25,7143	0	25,7143
1	1	1	trzy i więce	2	240	34,2857	34,2857	0
0	0	1	dwa czynni	3	30	4,28571	4,28571	0
0	1	1	trzy i więce	3	0	0	0	0
1	1	1	trzy i więce	1	60	8,57143	0	8,57143
0	1	1	trzy i więce	3	120	17,1429	8,57143	8,57143
0	1	1	trzy i więce	3	60	8,57143	8,57143	0
0	1	1	trzy i więce	1	60	8,57143	0	8,57143
0	0	1	dwa czynni	2	310	44,2857	10	34,2857
0	1	1	dwa czynni	1	210	30	0	30
1	0	1	trzy i więce	3	360	51,4286	51,4286	0
1	0	1	trzy i więce	2	420	60	0	60
0	1	1	trzy i więce	2	60	8,57143	0	8,57143
0	0	1	jeden czyn	3	60	8,57143	8,57143	0
0	0	1	trzy i więce	2	480	68,5714	0	68,5714
0	1	1	trzy i więce	2	120	17,1429	0	17,1429

0	0	1	dwa czynni	2	0	0	0	0
0	1	1	trzy i więce	2	0	0	0	0
0	0	1	jeden czyn	2	420	60	60	0
0	0	1	dwa czynni	2	140	20	0	20
0	0	1	dwa czynni	2	0	0	0	0
0	0	1	jeden czyn	3	450	64,2857	64,2857	0
1	0	1	trzy i więce	3	180	25,7143	8,57143	17,1429
1	1	1	trzy i więce	1	180	25,7143	0	25,7143
1	1	1	trzy i więce	3	495	70,7143	19,2857	51,4286
0	0	1	dwa czynni	3	150	21,4286	6,42857	15
1	1	0	trzy i więce	3	0	0	0	0
1	0	1	trzy i więce	3	0	0	0	0
0	0	1	dwa czynni	2	0	0	0	0
0	1	1	trzy i więce	1	300	42,8571	0	42,8571
1	1	1	trzy i więce	3	0	0	0	0
0	1	1	trzy i więce	3	480	68,5714	0	68,5714
0	0	1	trzy i więce	2	0	0	0	0
0	0	1	jeden czyn	1	0	0	0	0
1	1	0	trzy i więce	2	60	8,57143	0	8,57143
0	0	0	jeden czyn	2	0	0	0	0
0	1	0	dwa czynni	2	0	0	0	0
0	0	1	dwa czynni	2	0	0	0	0
1	1	1	trzy i więce	3	360	51,4286	0	51,4286
1	1	1	trzy i więce	3	30	4,28571	0	4,28571
0	0	1	jeden czyn	2	240	34,2857	34,2857	0
0	1	1	dwa czynni	1	120	17,1429	0	17,1429
0	0	1	trzy i więce	2	1260	180	0	180
0	0	1	dwa czynni	1	60	8,57143	0	8,57143
0	1	0	dwa czynni	3	120	17,1429	8,57143	8,57143
1	1	1	trzy i więce	2	60	8,57143	0	8,57143
0	0	1	dwa czynni	3	0	0	0	0
1	1	1	trzy i więce	2	140	20	0	20
0	1	1	trzy i więce	2	120	17,1429	0	17,1429
0	1	1	dwa czynni	2	600	85,7143	0	85,7143
1	1	1	trzy i więce	3	0	0	0	0
0	0	0	metabolicz	1	60	8,57143	0	8,57143
0	0	1	jeden czyn	2	0	0	0	0
0	0	1	jeden czyn	3	105	15	6,42857	8,57143
0	1	1	trzy i więce	2	0	0	0	0
0	1	1	trzy i więce	1	60	8,57143	8,57143	0
0	1	1	trzy i więce	2	105	15	0	15
0	0	1	jeden czyn	1	60	8,57143	8,57143	0
0	0	1	dwa czynni	3	50	7,14286	0	7,14286
0	1	1	trzy i więce	2	0	0	0	0
0	0	1	dwa czynni	3	180	25,7143	0	25,7143
1	1	1	trzy i więce	1	0	0	0	0
0	1	1	trzy i więce	2	0	0	0	0
0	0	1	dwa czynni	3	0	0	0	0

0	0	0	metabolicz	1	240	34,2857	0	34,2857
0	0	1	dwa czynni	3	60	8,57143	0	8,57143
1	1	1	trzy i więce	2	0	0	0	0
0	1	1	trzy i więce	3	90	12,8571	0	12,8571
0	0	1	dwa czynni	3	60	8,57143	0	8,57143
0	1	1	trzy i więce	2	0	0	0	0
0	1	0	dwa czynni	2	0	0	0	0
0	1	1	trzy i więce	2	540	77,1429	42,8571	34,2857
0	0	1	jeden czyn	1	0	0	0	0
0	1	1	trzy i więce	3	120	17,1429	0	17,1429
1	1	1	trzy i więce	3	120	17,1429	0	17,1429
1	1	1	trzy i więce	2	600	85,7143	0	85,7143
0	0	0	jeden czyn	3	180	25,7143	0	25,7143
0	0	1	dwa czynni	2	180	25,7143	25,7143	0
0	0	0	jeden czyn	2	0	0	0	0
1	1	1	trzy i więce	3	30	4,28571	4,28571	0
0	1	1	trzy i więce	3	0	0	0	0
0	0	1	trzy i więce	3	210	30	0	30
0	1	0	jeden czyn	1	240	34,2857	0	34,2857
0	0	0	metabolicz	3	0	0	0	0
0	0	0	jeden czyn	3	0	0	0	0
0	1	1	dwa czynni	3	600	85,7143	0	85,7143
0	0	1	dwa czynni	2	0	0	0	0
0	1	1	trzy i więce	2	0	0	0	0
0	1	1	dwa czynni	2	360	51,4286	17,1429	34,2857
0	1	1	trzy i więce	2	60	8,57143	0	8,57143
0	1	1	trzy i więce	3	40	5,71429	0	5,71429
1	0	1	trzy i więce	2	1680	240	60	180
0	0	1	dwa czynni	1	600	85,7143	0	85,7143
0	0	1	dwa czynni	2	360	51,4286	0	51,4286
0	1	1	trzy i więce	3	510	72,8571	51,4286	21,4286
1	1	1	trzy i więce	3	0	0	0	0
1	1	1	trzy i więce	3	120	17,1429	0	17,1429
1	0	0	jeden czyn	1	0	0	0	0
0	1	1	trzy i więce	3	140	20	0	20
1	0	1	trzy i więce	3	840	120	0	120
0	0	0	metabolicz	1	0	0	0	0
0	1	1	trzy i więce	2	120	17,1429	0	17,1429
0	0	1	dwa czynni	3	420	60	60	0
1	0	1	trzy i więce	3	120	17,1429	8,57143	8,57143
0	1	1	dwa czynni	1	0	0	0	0
0	0	1	dwa czynni	3	180	25,7143	0	25,7143
0	0	1	dwa czynni	1	720	102,857	68,5714	34,2857
1	0	1	trzy i więce	3	390	55,7143	4,28571	51,4286
0	0	1	dwa czynni	2	0	0	0	0
0	1	1	trzy i więce	2	60	8,57143	0	8,57143
0	1	1	trzy i więce	2	120	17,1429	0	17,1429
0	1	1	trzy i więce	2	0	0	0	0

0	0	1	dwa czynni	2	525	75	15	60
1	0	1	trzy i więce	2	60	8,57143	8,57143	0
0	0	1	trzy i więce	1	60	8,57143	0	8,57143
0	0	1	dwa czynni	2	0	0	0	0
0	0	1	jeden czyn	3	0	0	0	0
0	1	1	trzy i więce	3	320	45,7143	20	25,7143
1	0	1	trzy i więce	3	60	8,57143	0	8,57143
0	1	1	trzy i więce	2	60	8,57143	0	8,57143
1	0	1	trzy i więce	2	30	4,28571	0	4,28571
1	1	1	trzy i więce	1	180	25,7143	0	25,7143
1	1	1	trzy i więce	3	90	12,8571	8,57143	4,28571
1	0	1	trzy i więce	3	75	10,7143	10,7143	0
0	0	1	dwa czynni	2	0	0	0	0
1	1	1	trzy i więce	2	0	0	0	0
0	0	1	jeden czyn	3	120	17,1429	0	17,1429
0	0	1	jeden czyn	2	20	2,85714	2,85714	0
0	1	1	trzy i więce	2	120	17,1429	0	17,1429
0	0	1	trzy i więce	2	150	21,4286	0	21,4286
0	1	1	trzy i więce	3	60	8,57143	0	8,57143
0	0	1	jeden czyn	3	120	17,1429	0	17,1429
0	0	0	metabolicz	3	0	0	0	0
1	0	1	trzy i więce	1	0	0	0	0
0	0	1	trzy i więce	3	70	10	5,71429	4,28571
0	1	0	dwa czynni	1	0	0	0	0
0	0	1	dwa czynni	2	120	17,1429	17,1429	0
0	0	1	jeden czyn	1	280	40	0	40
1	0	1	trzy i więce	2	0	0	0	0
0	1	1	trzy i więce	3	180	25,7143	0	25,7143
1	1	1	trzy i więce	1	360	51,4286	0	51,4286
0	0	1	dwa czynni	1	0	0	0	0
1	0	1	trzy i więce	1	120	17,1429	0	17,1429
1	0	1	trzy i więce	2	0	0	0	0
0	0	1	jeden czyn	3	0	0	0	0
0	0	1	dwa czynni	3	620	88,5714	2,85714	85,7143
0	0	1	dwa czynni	2	60	8,57143	0	8,57143
0	0	1	trzy i więce	3	0	0	0	0
0	0	1	trzy i więce	3	0	0	0	0
0	1	1	trzy i więce	2	1260	180	0	180
0	1	1	dwa czynni	1	1740	248,571	68,5714	180
0	1	0	jeden czyn	1	420	60	0	60
0	0	0	metabolicz	3	645	92,1429	15	77,1429
0	0	1	dwa czynni	2	60	8,57143	8,57143	0
0	0	1	trzy i więce	1	0	0	0	0
0	1	1	trzy i więce	3	360	51,4286	51,4286	0
0	0	1	jeden czyn	2	60	8,57143	0	8,57143
0	0	1	dwa czynni	3	30	4,28571	0	4,28571
0	0	1	trzy i więce	2	0	0	0	0
1	1	1	trzy i więce	1	0	0	0	0

1	1	1	trzy i więc	3	0	0	0	0
0	0	0	metabolicz	1	0	0	0	0
0	0	1	jeden czyn	2	0	0	0	0
0	0	1	dwa czynni	3	0	0	0	0
1	1	1	trzy i więc	3	360	51,4286	51,4286	0
0	0	1	trzy i więc	3	0	0	0	0
0	0	1	dwa czynni	2	390	55,7143	30	25,7143
1	0	1	trzy i więc	2	0	0	0	0
1	0	1	trzy i więc	3	0	0	0	0
0	0	1	dwa czynni	3	0	0	0	0
0	1	1	trzy i więc	2	210	30	15	15
1	1	0	trzy i więc	2	0	0	0	0
1	1	1	trzy i więc	3	120	17,1429	0	17,1429
0	0	0	dwa czynni	3	660	94,2857	34,2857	60
0	1	1	trzy i więc	3	120	17,1429	12,8571	4,28571
0	1	1	trzy i więc	2	0	0	0	0
1	1	1	trzy i więc	3	60	8,57143	0	8,57143
1	1	1	trzy i więc	3	240	34,2857	17,1429	17,1429
1	1	1	trzy i więc	3	240	34,2857	0	34,2857
1	0	1	trzy i więc	2	0	0	0	0
0	1	1	dwa czynni	2	120	17,1429	17,1429	0
0	1	1	trzy i więc	3	0	0	0	0
0	0	0	dwa czynni	1	120	17,1429	0	17,1429
1	1	1	trzy i więc	2	60	8,57143	0	8,57143
0	0	1	dwa czynni	1	120	17,1429	0	17,1429
0	0	1	dwa czynni	2	0	0	0	0
1	0	0	dwa czynni	3	0	0	0	0
0	1	1	trzy i więc	2	0	0	0	0
0	0	1	dwa czynni	3	0	0	0	0
1	1	1	trzy i więc	2	360	51,4286	0	51,4286
0	1	0	trzy i więc	3	210	30	0	30
0	0	0	jeden czyn	2	180	25,7143	0	25,7143
0	1	1	trzy i więc	3	900	128,571	0	128,571
0	1	1	trzy i więc	3	0	0	0	0
0	1	0	jeden czyn	1	0	0	0	0
0	1	1	dwa czynni	3	0	0	0	0
0	1	1	trzy i więc	2	120	17,1429	0	17,1429
0	1	1	trzy i więc	2	0	0	0	0
0	1	1	dwa czynni	2	630	90	77,1429	12,8571
1	1	1	trzy i więc	1	120	17,1429	0	17,1429
1	1	1	trzy i więc	2	210	30	0	30
0	0	1	dwa czynni	3	60	8,57143	0	8,57143
0	1	1	trzy i więc	3	420	60	0	60
0	0	1	dwa czynni	2	120	17,1429	8,57143	8,57143
0	1	1	trzy i więc	3	0	0	0	0
1	1	1	trzy i więc	3	0	0	0	0
1	1	1	trzy i więc	3	60	8,57143	0	8,57143
0	1	1	trzy i więc	3	360	51,4286	0	51,4286

0	0	1	trzy i więce	2	0	0	0	0
0	1	1	trzy i więce	2	60	8,57143	0	8,57143
0	1	1	trzy i więce	1	180	25,7143	0	25,7143
0	1	1	trzy i więce	3	300	42,8571	0	42,8571
0	1	1	trzy i więce	2	420	60	0	60
0	0	1	jeden czyn	1	30	4,28571	0	4,28571
0	1	0	dwa czynni	1	480	68,5714	8,57143	60
0	0	1	jeden czyn	2	0	0	0	0
0	1	1	trzy i więce	2	0	0	0	0
0	0	1	jeden czyn	3	180	25,7143	25,7143	0
0	0	1	jeden czyn	2	0	0	0	0
1	0	1	trzy i więce	2	0	0	0	0
0	0	1	dwa czynni	1	0	0	0	0
0	0	1	trzy i więce	3	0	0	0	0
1	1	1	trzy i więce	1	0	0	0	0
0	0	1	dwa czynni	2	390	55,7143	4,28571	51,4286
0	1	0	dwa czynni	3	45	6,42857	6,42857	0
0	1	1	trzy i więce	2	240	34,2857	0	34,2857
0	1	1	trzy i więce	3	120	17,1429	0	17,1429
0	1	1	trzy i więce	3	0	0	0	0
0	0	1	dwa czynni	3	0	0	0	0
1	0	1	trzy i więce	1	300	42,8571	25,7143	17,1429
0	0	1	dwa czynni	3	135	19,2857	19,2857	0
0	0	1	dwa czynni	2	0	0	0	0
0	1	1	dwa czynni	1	0	0	0	0
1	0	0	dwa czynni	2	0	0	0	0
1	1	1	trzy i więce	3	0	0	0	0
0	0	1	dwa czynni	2	0	0	0	0
0	0	1	dwa czynni	2	360	51,4286	51,4286	0
0	1	1	trzy i więce	3	0	0	0	0
1	0	1	trzy i więce	2	0	0	0	0
1	1	1	trzy i więce	1	225	32,1429	0	32,1429
0	1	1	trzy i więce	3	0	0	0	0
0	1	1	trzy i więce	2	480	68,5714	0	68,5714
0	1	1	trzy i więce	2	30	4,28571	4,28571	0
1	1	1	trzy i więce	2	270	38,5714	25,7143	12,8571
1	0	1	trzy i więce	2	300	42,8571	42,8571	0
0	1	1	dwa czynni	3	720	102,857	0	102,857
0	0	1	jeden czyn	3	60	8,57143	0	8,57143
0	1	0	dwa czynni	3	720	102,857	85,7143	17,1429
0	0	0	metabolicz	1	240	34,2857	0	34,2857
0	0	1	dwa czynni	1	0	0	0	0
0	1	1	trzy i więce	1	60	8,57143	0	8,57143
0	1	1	trzy i więce	2	60	8,57143	0	8,57143
0	1	1	trzy i więce	3	360	51,4286	51,4286	0
0	0	1	dwa czynni	2	0	0	0	0
1	1	1	trzy i więce	3	300	42,8571	34,2857	8,57143
0	1	1	dwa czynni	2	40	5,71429	0	5,71429

0	0	1	jeden czyn	3	855	122,143	105	17,1429
0	1	1	dwa czynni	1	0	0	0	0
0	1	1	trzy i więce	3	0	0	0	0
0	1	1	dwa czynni	2	630	90	0	90
0	0	1	jeden czyn	2	0	0	0	0
0	1	1	trzy i więce	2	210	30	0	30
0	0	1	dwa czynni	2	30	4,28571	4,28571	0
0	0	0	metabolicz	3	0	0	0	0
0	1	1	trzy i więce	1	180	25,7143	0	25,7143
0	1	0	dwa czynni	2	240	34,2857	0	34,2857
0	1	0	dwa czynni	2	240	34,2857	0	34,2857
0	0	1	dwa czynni	3	0	0	0	0
0	0	1	dwa czynni	2	120	17,1429	0	17,1429
0	1	0	dwa czynni	3	0	0	0	0
0	0	1	dwa czynni	3	30	4,28571	0	4,28571
0	1	1	trzy i więce	3	420	60	0	60
0	1	1	trzy i więce	3	30	4,28571	0	4,28571
1	1	1	trzy i więce	2	240	34,2857	0	34,2857
0	0	1	trzy i więce	1	240	34,2857	0	34,2857
0	0	1	dwa czynni	3	780	111,429	51,4286	60
0	1	0	dwa czynni	2	80	11,4286	0	11,4286
0	0	1	jeden czyn	3	0	0	0	0
1	1	1	trzy i więce	3	420	60	34,2857	25,7143
0	1	1	trzy i więce	2	0	0	0	0
1	0	1	trzy i więce	3	30	4,28571	0	4,28571
0	1	1	trzy i więce	3	120	17,1429	0	17,1429
0	0	1	dwa czynni	1	240	34,2857	0	34,2857
0	0	0	metabolicz	2	60	8,57143	0	8,57143
0	0	0	metabolicz	3	60	8,57143	0	8,57143
0	1	1	trzy i więce	3	0	0	0	0
0	0	1	dwa czynni	3	180	25,7143	0	25,7143
0	1	1	dwa czynni	1	120	17,1429	0	17,1429
0	0	1	trzy i więce	3	0	0	0	0
1	0	1	trzy i więce	2	195	27,8571	15	12,8571
0	1	1	trzy i więce	3	525	75	15	60
0	0	0	metabolicz	2	120	17,1429	17,1429	0
0	0	1	jeden czyn	2	0	0	0	0
1	0	1	trzy i więce	2	0	0	0	0
0	1	1	trzy i więce	3	140	20	0	20
0	0	1	dwa czynni	1	120	17,1429	0	17,1429
1	0	1	trzy i więce	3	180	25,7143	0	25,7143
0	1	1	trzy i więce	1	0	0	0	0
0	1	1	trzy i więce	1	0	0	0	0
0	0	1	dwa czynni	3	180	25,7143	0	25,7143
0	1	1	trzy i więce	3	0	0	0	0
0	1	1	dwa czynni	3	180	25,7143	0	25,7143
0	1	1	dwa czynni	3	0	0	0	0
1	1	0	trzy i więce	2	0	0	0	0

0	1	1	trzy i więce	3	0	0	0	0
1	1	1	trzy i więce	3	0	0	0	0
0	1	1	dwa czynni	1	0	0	0	0
0	1	1	trzy i więce	3	0	0	0	0
0	1	1	trzy i więce	3	0	0	0	0
0	0	0	metabolicz	2	0	0	0	0
0	0	1	jeden czyn	2	0	0	0	0
1	0	0	trzy i więce	1	60	8,57143	0	8,57143
0	1	1	trzy i więce	3	500	71,4286	20	51,4286
0	1	1	trzy i więce	3	420	60	34,2857	25,7143
1	1	0	trzy i więce	3	60	8,57143	0	8,57143
0	0	1	dwa czynni	2	30	4,28571	0	4,28571
0	0	0	jeden czyn	3	0	0	0	0
1	0	1	trzy i więce	2	285	40,7143	40,7143	0
0	1	1	trzy i więce	3	1080	154,286	0	154,286
1	0	1	trzy i więce	2	0	0	0	0
0	1	1	trzy i więce	3	385	55	15	40
0	1	1	trzy i więce	1	0	0	0	0
0	0	1	dwa czynni	2	0	0	0	0
0	0	1	trzy i więce	1	0	0	0	0
1	1	1	trzy i więce	3	360	51,4286	51,4286	0
0	1	1	trzy i więce	1	0	0	0	0
0	1	1	trzy i więce	3	240	34,2857	0	34,2857
0	1	1	trzy i więce	3	300	42,8571	0	42,8571
0	0	1	dwa czynni	2	60	8,57143	0	8,57143
0	1	1	trzy i więce	2	180	25,7143	0	25,7143
1	1	1	trzy i więce	1	360	51,4286	0	51,4286
0	1	0	dwa czynni	1	480	68,5714	0	68,5714
0	1	1	trzy i więce	1	0	0	0	0
1	1	1	trzy i więce	3	45	6,42857	6,42857	0
0	1	0	dwa czynni	2	60	8,57143	0	8,57143
0	0	1	trzy i więce	2	120	17,1429	0	17,1429
0	1	1	trzy i więce	3	600	85,7143	51,4286	34,2857
0	0	1	jeden czyn	2	360	51,4286	51,4286	0
1	0	1	trzy i więce	2	0	0	0	0
1	1	1	trzy i więce	2	120	17,1429	0	17,1429
1	1	1	trzy i więce	1	0	0	0	0
0	0	1	trzy i więce	3	0	0	0	0
0	1	1	trzy i więce	2	90	12,8571	12,8571	0
0	1	1	trzy i więce	3	0	0	0	0
0	1	0	dwa czynni	3	420	60	0	60
0	1	1	trzy i więce	3	0	0	0	0
0	1	1	trzy i więce	3	0	0	0	0
1	0	1	trzy i więce	3	0	0	0	0
0	0	1	dwa czynni	2	170	24,2857	24,2857	0
1	0	1	trzy i więce	3	0	0	0	0
0	0	1	trzy i więce	1	360	51,4286	0	51,4286
0	1	1	trzy i więce	3	160	22,8571	5,71429	17,1429

0	0	1	dwa czynni	3	290	41,4286	28,5714	12,8571
0	1	1	trzy i więce	3	0	0	0	0
1	1	1	trzy i więce	3	0	0	0	0
1	1	1	trzy i więce	1	840	120	17,1429	102,857
0	1	1	trzy i więce	2	220	31,4286	5,71429	25,7143
0	1	1	trzy i więce	2	0	0	0	0
0	1	1	dwa czynni	2	60	8,57143	8,57143	0
0	1	1	trzy i więce	2	60	8,57143	0	8,57143
0	0	1	dwa czynni	3	0	0	0	0
0	0	0	dwa czynni	3	0	0	0	0
0	1	1	dwa czynni	1	0	0	0	0
0	1	1	trzy i więce	2	0	0	0	0
0	1	1	trzy i więce	2	0	0	0	0
0	1	1	trzy i więce	3	0	0	0	0
1	1	1	trzy i więce	3	30	4,28571	0	4,28571
1	0	1	trzy i więce	1	360	51,4286	0	51,4286
1	0	1	trzy i więce	3	300	42,8571	17,1429	25,7143
0	0	1	jeden czyn	2	360	51,4286	0	51,4286
1	1	1	trzy i więce	1	0	0	0	0
0	1	1	trzy i więce	3	120	17,1429	17,1429	0
1	0	1	dwa czynni	1	1260	180	0	180
1	0	1	trzy i więce	1	135	19,2857	10,7143	8,57143
1	1	1	trzy i więce	1	0	0	0	0
1	1	1	trzy i więce	2	0	0	0	0
0	1	0	dwa czynni	2	130	18,5714	10	8,57143
1	0	1	trzy i więce	2	40	5,71429	0	5,71429
0	0	1	dwa czynni	3	0	0	0	0
0	0	1	dwa czynni	1	60	8,57143	0	8,57143
1	1	0	trzy i więce	2	0	0	0	0
0	1	0	dwa czynni	2	180	25,7143	0	25,7143
0	0	1	jeden czyn	2	0	0	0	0
0	1	0	trzy i więce	2	210	30	30	0
0	0	1	dwa czynni	1	60	8,57143	0	8,57143
0	1	1	trzy i więce	3	0	0	0	0
1	1	1	trzy i więce	1	0	0	0	0
1	1	1	trzy i więce	2	0	0	0	0
0	0	1	dwa czynni	3	45	6,42857	6,42857	0
0	1	1	trzy i więce	3	120	17,1429	0	17,1429
0	1	1	trzy i więce	3	0	0	0	0
1	0	1	trzy i więce	3	240	34,2857	0	34,2857
0	0	1	jeden czyn	2	30	4,28571	0	4,28571
0	0	1	jeden czyn	1	120	17,1429	0	17,1429
0	0	1	jeden czyn	2	120	17,1429	0	17,1429
0	1	1	trzy i więce	2	0	0	0	0
0	0	1	dwa czynni	3	540	77,1429	64,2857	12,8571
0	0	1	dwa czynni	1	420	60	0	60
0	1	1	trzy i więce	1	1260	180	0	180
0	0	1	jeden czyn	3	0	0	0	0

0	1	0	dwa czynni	3	0	0	0	0
1	0	1	trzy i więc	2	150	21,4286	0	21,4286
0	0	0	jeden czyn	2	350	50	50	0
0	0	1	trzy i więc	2	420	60	0	60
0	0	1	dwa czynni	3	180	25,7143	0	25,7143
1	0	1	trzy i więc	1	60	8,57143	0	8,57143
0	0	1	jeden czyn	1	0	0	0	0
1	0	0	trzy i więc	3	120	17,1429	0	17,1429
0	0	0	metabolicz	3	540	77,1429	51,4286	25,7143
0	0	1	dwa czynni	3	0	0	0	0
0	0	1	jeden czyn	3	840	120	0	120
0	1	0	trzy i więc	2	120	17,1429	0	17,1429
0	1	1	trzy i więc	3	360	51,4286	0	51,4286
0	1	1	trzy i więc	3	120	17,1429	0	17,1429
0	0	1	dwa czynni	3	210	30	0	30
1	1	1	trzy i więc	1	180	25,7143	25,7143	0
0	0	1	jeden czyn	3	150	21,4286	17,1429	4,28571
0	1	1	trzy i więc	3	0	0	0	0
0	0	1	jeden czyn	1	0	0	0	0
1	0	1	trzy i więc	3	240	34,2857	0	34,2857
0	0	1	dwa czynni	3	180	25,7143	0	25,7143
1	1	1	trzy i więc	3	270	38,5714	8,57143	30
0	1	1	dwa czynni	1	0	0	0	0
0	0	1	dwa czynni	3	0	0	0	0
1	1	1	trzy i więc	1	90	12,8571	0	12,8571
0	0	1	dwa czynni	3	40	5,71429	0	5,71429
0	0	1	dwa czynni	3	720	102,857	51,4286	51,4286
1	1	1	trzy i więc	1	140	20	0	20
0	0	1	dwa czynni	1	240	34,2857	25,7143	8,57143
0	0	0	metabolicz	1	60	8,57143	0	8,57143
0	0	1	jeden czyn	1	60	8,57143	0	8,57143
0	0	1	trzy i więc	1	180	25,7143	8,57143	17,1429
1	1	1	trzy i więc	2	210	30	0	30
0	0	0	jeden czyn	3	60	8,57143	8,57143	0
1	1	1	trzy i więc	3	450	64,2857	12,8571	51,4286
1	0	1	trzy i więc	3	0	0	0	0
0	0	1	dwa czynni	2	165	23,5714	6,42857	17,1429
0	0	1	dwa czynni	2	160	22,8571	5,71429	17,1429
0	1	1	dwa czynni	1	0	0	0	0
0	0	1	jeden czyn	3	120	17,1429	17,1429	0
0	0	0	metabolicz	3	720	102,857	0	102,857
1	1	1	trzy i więc	2	0	0	0	0
1	0	1	trzy i więc	2	90	12,8571	0	12,8571
0	0	1	dwa czynni	3	1400	200	20	180
0	0	0	jeden czyn	2	420	60	0	60
0	0	1	dwa czynni	1	0	0	0	0
1	1	1	trzy i więc	1	140	20	0	20
0	0	1	dwa czynni	2	210	30	0	30

0	0	1	dwa czynni	3	120	17,1429	0	17,1429
0	1	0	trzy i więce	2	60	8,57143	8,57143	0
0	0	1	jeden czyn	1	0	0	0	0
1	0	1	trzy i więce	3	240	34,2857	0	34,2857
0	1	0	dwa czynni	2	45	6,42857	6,42857	0
0	1	1	dwa czynni	1	120	17,1429	0	17,1429
0	1	1	trzy i więce	2	180	25,7143	0	25,7143
0	0	1	dwa czynni	3	60	8,57143	0	8,57143
0	1	1	trzy i więce	2	0	0	0	0
0	1	1	dwa czynni	3	120	17,1429	0	17,1429
1	1	1	trzy i więce	1	0	0	0	0
0	0	1	dwa czynni	2	120	17,1429	0	17,1429
0	0	1	dwa czynni	3	0	0	0	0
0	0	1	dwa czynni	3	60	8,57143	0	8,57143
0	1	1	trzy i więce	3	60	8,57143	0	8,57143
0	1	1	dwa czynni	1	0	0	0	0
0	1	1	trzy i więce	1	0	0	0	0
0	1	1	trzy i więce	1	120	17,1429	0	17,1429
0	1	1	trzy i więce	3	100	14,2857	0	14,2857
0	0	1	jeden czyn	2	960	137,143	77,1429	60
0	1	1	trzy i więce	3	210	30	0	30
1	0	1	trzy i więce	1	30	4,28571	0	4,28571
1	1	1	trzy i więce	3	180	25,7143	0	25,7143
0	0	1	dwa czynni	3	10	1,42857	1,42857	0
0	0	1	jeden czyn	3	180	25,7143	0	25,7143
1	1	1	trzy i więce	2	240	34,2857	0	34,2857
0	0	1	dwa czynni	3	0	0	0	0
0	0	1	dwa czynni	2	0	0	0	0
0	1	1	trzy i więce	1	0	0	0	0
0	1	1	dwa czynni	3	420	60	0	60
0	0	1	dwa czynni	3	0	0	0	0
0	0	1	dwa czynni	1	0	0	0	0
0	1	1	trzy i więce	1	0	0	0	0
0	0	1	trzy i więce	3	0	0	0	0
0	1	1	trzy i więce	3	0	0	0	0
0	0	1	trzy i więce	2	160	22,8571	8,57143	14,2857
0	1	1	trzy i więce	3	180	25,7143	25,7143	0
0	1	1	dwa czynni	3	0	0	0	0
0	1	0	jeden czyn	3	120	17,1429	0	17,1429
1	1	1	trzy i więce	3	0	0	0	0
0	1	1	trzy i więce	3	120	17,1429	17,1429	0
0	1	0	trzy i więce	1	60	8,57143	0	8,57143
0	0	1	jeden czyn	2	165	23,5714	6,42857	17,1429
1	0	1	trzy i więce	2	60	8,57143	0	8,57143
0	1	1	trzy i więce	2	120	17,1429	0	17,1429
0	1	1	trzy i więce	3	360	51,4286	0	51,4286
0	1	0	dwa czynni	1	30	4,28571	0	4,28571
0	0	1	jeden czyn	2	150	21,4286	17,1429	4,28571

0	*0*	*1*	trzy i więce	2	*0*	*0*	*0*	*0*
0	*0*	*1*	dwa czynni	2	*120*	*17,1429*	*8,57143*	*8,57143*
0	*1*	*1*	dwa czynni	2	*420*	*60*	*0*	*60*
0	*1*	*0*	jeden czyn	2	*0*	*0*	*0*	*0*
0	*1*	*0*	trzy i więce	2	*0*	*0*	*0*	*0*
0	*0*	*1*	jeden czyn	3	*0*	*0*	*0*	*0*
0	*1*	*1*	trzy i więce	1	*0*	*0*	*0*	*0*
0	*0*	*1*	jeden czyn	3	*15*	*2,14286*	*0*	*2,14286*
0	*1*	*1*	trzy i więce	1	*30*	*4,28571*	*0*	*4,28571*
1	*0*	*1*	trzy i więce	2	*180*	*25,7143*	*17,1429*	*8,57143*
0	*1*	*1*	trzy i więce	3	*300*	*42,8571*	*0*	*42,8571*
0	*0*	*0*	metabolicz	2	*0*	*0*	*0*	*0*
0	*1*	*1*	trzy i więce	1	*240*	*34,2857*	*0*	*34,2857*
0	*0*	*0*	metabolicz	1	*0*	*0*	*0*	*0*
1	*1*	*1*	trzy i więce	1	*20*	*2,85714*	*0*	*2,85714*
0	*0*	*1*	dwa czynni	3	*0*	*0*	*0*	*0*
1	*1*	*1*	trzy i więce	2	*140*	*20*	*0*	*20*
0	*1*	*1*	trzy i więce	3	*180*	*25,7143*	*17,1429*	*8,57143*
0	*0*	*1*	dwa czynni	3	*210*	*30*	*0*	*30*
0	*0*	*1*	jeden czyn	2	*0*	*0*	*0*	*0*
0	*0*	*0*	dwa czynni	2	*30*	*4,28571*	*4,28571*	*0*
0	*0*	*1*	dwa czynni	2	*0*	*0*	*0*	*0*
0	*0*	*0*	jeden czyn	3	*0*	*0*	*0*	*0*
0	*0*	*0*	dwa czynni	3	*480*	*68,5714*	*0*	*68,5714*
0	*0*	*1*	jeden czyn	3	*330*	*47,1429*	*21,4286*	*25,7143*
0	*0*	*1*	trzy i więce	3	*0*	*0*	*0*	*0*
0	*0*	*1*	dwa czynni	2	*0*	*0*	*0*	*0*
0	*1*	*1*	trzy i więce	1	*120*	*17,1429*	*0*	*17,1429*
1	*0*	*1*	trzy i więce	3	*40*	*5,71429*	*5,71429*	*0*
0	*0*	*1*	jeden czyn	3	*0*	*0*	*0*	*0*
1	*1*	*1*	trzy i więce	3	*300*	*42,8571*	*0*	*42,8571*
1	*1*	*1*	trzy i więce	1	*0*	*0*	*0*	*0*
0	*0*	*1*	dwa czynni	2	*0*	*0*	*0*	*0*
1	*0*	*1*	trzy i więce	1	*0*	*0*	*0*	*0*
1	*0*	*1*	trzy i więce	3	*120*	*17,1429*	*0*	*17,1429*
0	*0*	*1*	dwa czynni	2	*1260*	*180*	*0*	*180*
0	*0*	*1*	trzy i więce	3	*0*	*0*	*0*	*0*
0	*0*	*0*	metabolicz	2	*840*	*120*	*0*	*120*
0	*0*	*1*	jeden czyn	2	*45*	*6,42857*	*6,42857*	*0*
0	*1*	*1*	trzy i więce	3	*180*	*25,7143*	*0*	*25,7143*
0	*1*	*1*	trzy i więce	3	*30*	*4,28571*	*0*	*4,28571*
1	*1*	*1*	trzy i więce	1	*480*	*68,5714*	*0*	*68,5714*
0	*1*	*0*	dwa czynni	1	*0*	*0*	*0*	*0*
0	*0*	*1*	dwa czynni	3	*0*	*0*	*0*	*0*
0	*1*	*1*	trzy i więce	2	*120*	*17,1429*	*0*	*17,1429*
1	*0*	*1*	trzy i więce	1	*0*	*0*	*0*	*0*
0	*0*	*1*	jeden czyn	1	*0*	*0*	*0*	*0*
0	*1*	*1*	trzy i więce	3	*0*	*0*	*0*	*0*

1	*1*	*1*	trzy i więce	2	*0*	*0*	*0*	*0*
0	*0*	*0*	metabolicz	2	*60*	*8,57143*	*8,57143*	*0*
0	*1*	*1*	trzy i więce	2	*0*	*0*	*0*	*0*
0	*0*	*1*	dwa czynni	3	*90*	*12,8571*	*4,28571*	*8,57143*
0	*0*	*1*	dwa czynni	3	*0*	*0*	*0*	*0*
1	*1*	*1*	trzy i więce	1	*120*	*17,1429*	*0*	*17,1429*
1	*0*	*1*	trzy i więce	1	*0*	*0*	*0*	*0*
0	*1*	*1*	trzy i więce	3	*120*	*17,1429*	*0*	*17,1429*
0	*1*	*1*	trzy i więce	1	*210*	*30*	*30*	*0*
0	*0*	*1*	dwa czynni	3	*200*	*28,5714*	*28,5714*	*0*
0	*0*	*1*	trzy i więce	3	*0*	*0*	*0*	*0*
1	*1*	*1*	trzy i więce	3	*0*	*0*	*0*	*0*
1	*0*	*1*	trzy i więce	2	*1050*	*150*	*90*	*60*
0	*0*	*1*	dwa czynni	3	*90*	*12,8571*	*8,57143*	*4,28571*
0	*1*	*1*	dwa czynni	1	*270*	*38,5714*	*4,28571*	*34,2857*
0	*1*	*0*	trzy i więce	1	*270*	*38,5714*	*8,57143*	*30*
0	*0*	*1*	jeden czyn	1	*0*	*0*	*0*	*0*
0	*0*	*1*	jeden czyn	1	*0*	*0*	*0*	*0*
0	*0*	*1*	trzy i więce	3	*100*	*14,2857*	*0*	*14,2857*
0	*0*	*1*	trzy i więce	2	*0*	*0*	*0*	*0*
0	*1*	*1*	trzy i więce	1	*0*	*0*	*0*	*0*
1	*1*	*1*	trzy i więce	2	*60*	*8,57143*	*0*	*8,57143*
0	*0*	*1*	jeden czyn	2	*240*	*34,2857*	*0*	*34,2857*
0	*1*	*1*	trzy i więce	2	*60*	*8,57143*	*0*	*8,57143*
0	*1*	*0*	jeden czyn	3	*490*	*70*	*10*	*60*
1	*1*	*1*	trzy i więce	3	*0*	*0*	*0*	*0*
0	*1*	*1*	trzy i więce	3	*0*	*0*	*0*	*0*
0	*0*	*1*	dwa czynni	3	*340*	*48,5714*	*42,8571*	*5,71429*
0	*0*	*1*	dwa czynni	3	*240*	*34,2857*	*8,57143*	*25,7143*
0	*1*	*1*	trzy i więce	2	*210*	*30*	*0*	*30*
0	*0*	*0*	jeden czyn	3	*210*	*30*	*0*	*30*
0	*0*	*1*	dwa czynni	2	*200*	*28,5714*	*0*	*28,5714*
0	*1*	*0*	dwa czynni	2	*360*	*51,4286*	*38,5714*	*12,8571*
1	*1*	*1*	trzy i więce	2	*0*	*0*	*0*	*0*
1	*1*	*1*	trzy i więce	2	*180*	*25,7143*	*0*	*25,7143*
0	*1*	*0*	trzy i więce	3	*0*	*0*	*0*	*0*
0	*0*	*0*	metabolicz	2	*430*	*61,4286*	*51,4286*	*10*
1	*1*	*1*	trzy i więce	3	*0*	*0*	*0*	*0*
0	*0*	*1*	trzy i więce	2	*180*	*25,7143*	*0*	*25,7143*
1	*0*	*1*	trzy i więce	1	*420*	*60*	*60*	*0*
1	*1*	*1*	trzy i więce	3	*45*	*6,42857*	*0*	*6,42857*
1	*1*	*1*	trzy i więce	2	*240*	*34,2857*	*0*	*34,2857*
1	*0*	*1*	trzy i więce	3	*840*	*120*	*0*	*120*
0	*1*	*1*	dwa czynni	2	*100*	*14,2857*	*0*	*14,2857*
0	*0*	*0*	dwa czynni	1	*20*	*2,85714*	*0*	*2,85714*
0	*1*	*1*	trzy i więce	3	*0*	*0*	*0*	*0*
1	*0*	*1*	trzy i więce	2	*140*	*20*	*0*	*20*
0	*0*	*1*	dwa czynni	3	*510*	*72,8571*	*60*	*12,8571*

0	1	1	trzy i więce	3	420	60	0	60
0	1	1	trzy i więce	3	0	0	0	0
1	1	1	trzy i więce	2	0	0	0	0
0	1	0	jeden czyn	1	0	0	0	0
1	0	0	jeden czyn	3	60	8,57143	0	8,57143
0	1	1	trzy i więce	1	120	17,1429	0	17,1429
0	0	1	dwa czynni	2	1620	231,429	180	51,4286
0	1	0	dwa czynni	3	0	0	0	0
0	0	1	jeden czyn	3	0	0	0	0
0	1	0	jeden czyn	1	0	0	0	0
0	0	1	jeden czyn	2	120	17,1429	8,57143	8,57143
0	1	1	trzy i więce	2	200	28,5714	0	28,5714
0	1	1	trzy i więce	3	120	17,1429	0	17,1429
0	1	0	trzy i więce	3	0	0	0	0
0	0	1	dwa czynni	3	0	0	0	0
0	1	1	trzy i więce	3	120	17,1429	0	17,1429
0	1	1	trzy i więce	1	315	45	0	45
0	0	1	dwa czynni	1	0	0	0	0
1	1	1	trzy i więce	3	105	15	15	0
0	1	0	dwa czynni	3	690	98,5714	68,5714	30
0	1	1	dwa czynni	2	60	8,57143	0	8,57143
0	0	1	jeden czyn	2	0	0	0	0
0	1	1	trzy i więce	3	240	34,2857	25,7143	8,57143
0	1	0	dwa czynni	2	1260	180	0	180
1	0	1	trzy i więce	2	270	38,5714	12,8571	25,7143
1	0	1	trzy i więce	2	360	51,4286	25,7143	25,7143
0	0	0	dwa czynni	2	290	41,4286	7,14286	34,2857
0	1	1	trzy i więce	3	60	8,57143	0	8,57143
0	0	0	jeden czyn	3	30	4,28571	0	4,28571
0	1	1	trzy i więce	1	240	34,2857	0	34,2857
0	0	1	jeden czyn	1	240	34,2857	0	34,2857
1	0	1	trzy i więce	3	60	8,57143	0	8,57143
0	0	1	dwa czynni	2	0	0	0	0
0	0	1	dwa czynni	3	120	17,1429	0	17,1429
0	0	0	jeden czyn	3	0	0	0	0
0	1	1	trzy i więce	3	330	47,1429	4,28571	42,8571
0	0	1	jeden czyn	2	150	21,4286	0	21,4286
0	1	1	trzy i więce	1	720	102,857	102,857	0
0	1	1	trzy i więce	1	0	0	0	0
0	1	1	trzy i więce	2	840	120	0	120
0	1	1	trzy i więce	2	0	0	0	0
1	1	1	trzy i więce	3	420	60	0	60
0	0	0	dwa czynni	1	180	25,7143	0	25,7143
0	0	0	dwa czynni	3	0	0	0	0
0	0	1	jeden czyn	2	300	42,8571	42,8571	0
0	1	1	trzy i więce	3	30	4,28571	4,28571	0
0	1	0	jeden czyn	1	135	19,2857	0	19,2857
0	0	0	metabolicz	3	120	17,1429	0	17,1429

0	0	0	metabolicz	3	210	30	0	30
0	1	1	trzy i więce	1	480	68,5714	42,8571	25,7143
0	1	1	trzy i więce	2	30	4,28571	0	4,28571
0	1	1	trzy i więce	3	0	0	0	0
1	0	1	trzy i więce	2	0	0	0	0
0	0	1	dwa czynni	2	0	0	0	0
1	0	1	trzy i więce	3	60	8,57143	8,57143	0
0	1	1	trzy i więce	2	120	17,1429	8,57143	8,57143
1	1	1	trzy i więce	3	900	128,571	128,571	0
0	1	1	dwa czynni	3	240	34,2857	25,7143	8,57143
0	0	1	jeden czyn	2	120	17,1429	0	17,1429
0	0	1	trzy i więce	3	60	8,57143	0	8,57143
0	0	0	metabolicz	3	120	17,1429	17,1429	0
1	1	1	trzy i więce	2	40	5,71429	0	5,71429
0	1	1	dwa czynni	3	60	8,57143	0	8,57143
0	1	1	trzy i więce	2	0	0	0	0
0	0	1	jeden czyn	1	200	28,5714	0	28,5714
0	1	1	trzy i więce	2	60	8,57143	0	8,57143
1	1	1	trzy i więce	3	0	0	0	0
0	1	1	trzy i więce	3	0	0	0	0
0	0	1	jeden czyn	2	210	30	30	0
0	1	0	dwa czynni	1	0	0	0	0
0	0	1	jeden czyn	3	180	25,7143	0	25,7143
0	1	1	trzy i więce	3	60	8,57143	0	8,57143
0	1	1	trzy i więce	3	60	8,57143	0	8,57143
1	0	1	trzy i więce	2	0	0	0	0
0	1	1	trzy i więce	3	540	77,1429	17,1429	60
0	0	1	trzy i więce	3	0	0	0	0
0	0	1	dwa czynni	2	60	8,57143	0	8,57143
0	1	1	dwa czynni	3	0	0	0	0
0	1	1	trzy i więce	2	120	17,1429	8,57143	8,57143
0	0	1	jeden czyn	1	60	8,57143	8,57143	0
0	1	0	dwa czynni	3	720	102,857	68,5714	34,2857
1	0	1	trzy i więce	3	840	120	0	120
0	0	1	jeden czyn	3	0	0	0	0
0	0	1	dwa czynni	2	60	8,57143	8,57143	0
0	1	1	trzy i więce	3	0	0	0	0
1	0	1	trzy i więce	2	180	25,7143	0	25,7143
0	1	1	trzy i więce	3	120	17,1429	0	17,1429
0	0	1	dwa czynni	2	0	0	0	0
0	0	1	dwa czynni	2	0	0	0	0
0	1	1	trzy i więce	3	195	27,8571	12,8571	15
0	1	0	trzy i więce	1	0	0	0	0
0	1	0	dwa czynni	2	0	0	0	0
0	1	1	trzy i więce	1	40	5,71429	5,71429	0
0	0	1	jeden czyn	1	0	0	0	0
1	0	1	trzy i więce	3	120	17,1429	0	17,1429
1	0	1	trzy i więce	2	225	32,1429	32,1429	0

1	0	0	jeden czyn	2	0	0	0	0
0	0	1	trzy i więc	3	0	0	0	0
0	1	1	trzy i więc	3	0	0	0	0
0	1	1	trzy i więc	3	60	8,57143	0	8,57143
1	1	0	trzy i więc	3	0	0	0	0
1	1	1	trzy i więc	2	0	0	0	0
0	0	1	dwa czynni	3	150	21,4286	21,4286	0
0	1	1	trzy i więc	1	0	0	0	0
1	1	1	trzy i więc	2	300	42,8571	34,2857	8,57143
0	0	1	jeden czyn	2	0	0	0	0
0	0	1	trzy i więc	1	30	4,28571	0	4,28571
0	1	1	trzy i więc	3	0	0	0	0
1	1	0	trzy i więc	2	20	2,85714	0	2,85714
0	1	1	trzy i więc	3	840	120	42,8571	77,1429
0	0	1	dwa czynni	3	0	0	0	0
0	0	1	jeden czyn	3	70	10	10	0
0	0	1	trzy i więc	3	100	14,2857	14,2857	0
0	1	1	trzy i więc	2	120	17,1429	0	17,1429
0	0	0	metabolicz	1	60	8,57143	0	8,57143
0	1	1	trzy i więc	2	120	17,1429	0	17,1429
0	0	1	dwa czynni	2	0	0	0	0
1	0	1	trzy i więc	3	390	55,7143	38,5714	17,1429
0	1	0	dwa czynni	2	180	25,7143	17,1429	8,57143
0	1	1	trzy i więc	1	0	0	0	0
0	1	1	trzy i więc	3	0	0	0	0
0	1	1	trzy i więc	3	660	94,2857	34,2857	60
0	0	0	dwa czynni	1	120	17,1429	0	17,1429
0	1	0	dwa czynni	2	0	0	0	0
0	0	1	trzy i więc	1	90	12,8571	0	12,8571
0	0	1	jeden czyn	1	0	0	0	0
0	1	0	dwa czynni	2	120	17,1429	17,1429	0
0	1	1	dwa czynni	2	0	0	0	0
0	0	1	jeden czyn	3	0	0	0	0
0	1	1	trzy i więc	1	0	0	0	0
0	0	0	metabolicz	2	60	8,57143	0	8,57143
0	1	1	dwa czynni	3	0	0	0	0
0	1	1	trzy i więc	2	0	0	0	0
0	0	0	jeden czyn	3	0	0	0	0
0	0	1	dwa czynni	3	30	4,28571	4,28571	0
0	1	0	dwa czynni	1	0	0	0	0
1	0	1	trzy i więc	1	210	30	0	30
0	0	1	dwa czynni	2	60	8,57143	0	8,57143
1	1	1	trzy i więc	1	720	102,857	0	102,857
0	0	0	jeden czyn	1	15	2,14286	2,14286	0
1	1	1	trzy i więc	2	220	31,4286	5,71429	25,7143
0	0	0	metabolicz	2	0	0	0	0
0	0	0	metabolicz	1	0	0	0	0
0	0	1	jeden czyn	1	90	12,8571	4,28571	8,57143

0	1	1	dwa czynni	2	60	8,57143	0	8,57143
0	1	0	jeden czyn	1	360	51,4286	42,8571	8,57143
1	0	1	trzy i więce	3	120	17,1429	0	17,1429
0	1	1	trzy i więce	2	25	3,57143	0	3,57143
0	1	1	trzy i więce	3	105	15	8,57143	6,42857
0	0	1	trzy i więce	3	165	23,5714	12,8571	10,7143
0	1	1	trzy i więce	3	0	0	0	0
0	0	1	jeden czyn	1	840	120	0	120
0	1	0	dwa czynni	1	0	0	0	0
1	1	1	trzy i więce	3	0	0	0	0
0	0	1	dwa czynni	1	0	0	0	0
0	0	0	jeden czyn	2	0	0	0	0
0	0	1	jeden czyn	1	15	2,14286	2,14286	0
0	0	1	dwa czynni	3	225	32,1429	15	17,1429
0	0	1	dwa czynni	1	0	0	0	0
0	0	0	dwa czynni	3	0	0	0	0
0	1	1	trzy i więce	1	120	17,1429	0	17,1429
0	0	1	dwa czynni	1	0	0	0	0
1	0	1	trzy i więce	3	0	0	0	0
0	0	1	dwa czynni	3	60	8,57143	8,57143	0
0	0	0	metabolicz	2	120	17,1429	17,1429	0
0	1	1	trzy i więce	1	0	0	0	0
0	1	1	trzy i więce	1	840	120	0	120
0	1	1	dwa czynni	2	405	57,8571	23,5714	34,2857
0	1	1	trzy i więce	3	60	8,57143	0	8,57143
0	0	0	jeden czyn	3	900	128,571	8,57143	120
1	1	1	trzy i więce	3	0	0	0	0
0	0	1	dwa czynni	2	0	0	0	0
0	1	1	trzy i więce	1	0	0	0	0
1	0	1	trzy i więce	2	0	0	0	0
0	0	1	dwa czynni	2	0	0	0	0
0	1	1	trzy i więce	2	140	20	0	20
0	0	1	jeden czyn	1	210	30	0	30
0	1	1	trzy i więce	3	210	30	0	30
0	1	1	trzy i więce	3	0	0	0	0
0	1	1	trzy i więce	2	270	38,5714	8,57143	30
1	1	1	trzy i więce	3	90	12,8571	12,8571	0
0	1	1	trzy i więce	3	120	17,1429	17,1429	0
1	1	1	trzy i więce	1	20	2,85714	0	2,85714
0	0	1	dwa czynni	2	180	25,7143	25,7143	0
0	0	1	jeden czyn	2	420	60	0	60
0	1	0	jeden czyn	2	600	85,7143	34,2857	51,4286
0	0	1	jeden czyn	3	0	0	0	0
0	1	1	trzy i więce	2	0	0	0	0
0	0	0	metabolicz	2	120	17,1429	17,1429	0
0	0	0	jeden czyn	3	0	0	0	0
0	0	1	trzy i więce	1	420	60	42,8571	17,1429
0	0	1	jeden czyn	3	210	30	0	30

0	1	1	trzy i więce	2	180	25,7143	0	25,7143
0	1	1	trzy i więce	3	270	38,5714	21,4286	17,1429
0	0	1	dwa czynni	1	30	4,28571	0	4,28571
0	0	0	jeden czyn	2	70	10	10	0
0	0	1	dwa czynni	2	360	51,4286	0	51,4286
0	0	1	dwa czynni	3	0	0	0	0
0	1	1	trzy i więce	2	240	34,2857	0	34,2857
1	1	1	trzy i więce	3	420	60	0	60
0	1	1	trzy i więce	3	0	0	0	0
0	0	1	dwa czynni	3	0	0	0	0
0	1	1	trzy i więce	3	225	32,1429	15	17,1429
0	1	1	trzy i więce	3	120	17,1429	0	17,1429
0	0	1	trzy i więce	2	240	34,2857	8,57143	25,7143
1	1	1	trzy i więce	2	300	42,8571	25,7143	17,1429
1	0	1	dwa czynni	3	0	0	0	0
1	1	1	trzy i więce	1	90	12,8571	12,8571	0
1	0	0	jeden czyn	3	240	34,2857	0	34,2857
1	0	1	trzy i więce	3	0	0	0	0
0	0	0	jeden czyn	1	0	0	0	0
0	0	0	metabolicz	2	540	77,1429	77,1429	0
0	1	1	trzy i więce	3	40	5,71429	0	5,71429
1	1	1	trzy i więce	3	165	23,5714	6,42857	17,1429
0	0	1	jeden czyn	1	60	8,57143	8,57143	0
0	0	0	jeden czyn	1	120	17,1429	0	17,1429
0	0	1	dwa czynni	1	45	6,42857	0	6,42857
1	1	1	trzy i więce	3	420	60	60	0
0	0	1	dwa czynni	3	180	25,7143	0	25,7143
0	0	0	jeden czyn	2	210	30	0	30
0	0	1	dwa czynni	2	60	8,57143	8,57143	0
0	0	1	dwa czynni	1	0	0	0	0
0	0	1	jeden czyn	3	60	8,57143	0	8,57143
0	1	1	trzy i więce	2	270	38,5714	25,7143	12,8571
1	1	1	trzy i więce	3	0	0	0	0
1	0	1	trzy i więce	3	0	0	0	0
0	0	1	jeden czyn	3	240	34,2857	0	34,2857
0	0	1	dwa czynni	2	0	0	0	0
0	0	1	trzy i więce	1	90	12,8571	0	12,8571
1	1	1	trzy i więce	1	0	0	0	0
0	0	1	dwa czynni	3	0	0	0	0
0	0	0	jeden czyn	3	120	17,1429	0	17,1429
0	0	1	trzy i więce	2	0	0	0	0
0	1	1	trzy i więce	3	210	30	17,1429	12,8571
0	1	1	trzy i więce	3	0	0	0	0
1	1	1	trzy i więce	3	0	0	0	0
0	0	1	dwa czynni	2	0	0	0	0
0	0	1	dwa czynni	1	0	0	0	0
0	0	0	metabolicz	2	0	0	0	0
0	0	1	dwa czynni	2	0	0	0	0

1	1	1	trzy i więce	3	175	25	0	25
0	1	1	dwa czynni	1	0	0	0	0
0	0	1	trzy i więce	2	20	2,85714	0	2,85714
1	0	1	trzy i więce	2	120	17,1429	0	17,1429
1	1	1	trzy i więce	1	0	0	0	0
1	1	1	trzy i więce	1	90	12,8571	0	12,8571
0	0	1	dwa czynni	2	110	15,7143	10	5,71429
0	1	1	trzy i więce	3	30	4,28571	0	4,28571
0	0	1	dwa czynni	2	0	0	0	0
1	0	1	trzy i więce	2	1320	188,571	8,57143	180
0	0	1	dwa czynni	3	0	0	0	0
0	1	1	trzy i więce	2	120	17,1429	0	17,1429
0	0	1	jeden czyn	2	30	4,28571	4,28571	0
0	0	1	trzy i więce	3	420	60	0	60
0	0	0	metabolicz	2	0	0	0	0
0	0	1	dwa czynni	3	60	8,57143	0	8,57143
0	1	1	trzy i więce	2	180	25,7143	4,28571	21,4286
0	1	1	trzy i więce	2	120	17,1429	0	17,1429
0	0	0	jeden czyn	2	0	0	0	0
0	0	1	jeden czyn	3	640	91,4286	91,4286	0
0	0	0	metabolicz	2	330	47,1429	42,8571	4,28571
0	1	1	trzy i więce	1	0	0	0	0
0	0	1	trzy i więce	2	0	0	0	0
0	1	1	trzy i więce	3	1080	154,286	0	154,286
0	1	1	trzy i więce	1	180	25,7143	0	25,7143
0	0	1	jeden czyn	1	0	0	0	0
1	1	1	trzy i więce	3	540	77,1429	0	77,1429
0	0	0	metabolicz	2	15	2,14286	0	2,14286
0	1	1	trzy i więce	2	60	8,57143	0	8,57143
0	1	1	trzy i więce	2	240	34,2857	34,2857	0
0	0	0	metabolicz	2	140	20	20	0
0	0	1	trzy i więce	2	180	25,7143	17,1429	8,57143
0	1	1	trzy i więce	3	30	4,28571	0	4,28571
0	0	1	jeden czyn	2	380	54,2857	34,2857	20
0	1	1	trzy i więce	2	0	0	0	0
0	0	1	dwa czynni	2	360	51,4286	51,4286	0
0	1	1	trzy i więce	2	0	0	0	0
0	0	1	jeden czyn	1	0	0	0	0
0	0	1	trzy i więce	2	0	0	0	0
0	1	1	trzy i więce	1	630	90	0	90
0	0	0	jeden czyn	1	120	17,1429	0	17,1429
0	0	1	jeden czyn	1	0	0	0	0
0	1	1	dwa czynni	2	60	8,57143	0	8,57143
0	1	1	dwa czynni	2	30	4,28571	0	4,28571
0	1	1	trzy i więce	2	435	62,1429	45	17,1429
1	0	0	dwa czynni	3	0	0	0	0
0	1	1	dwa czynni	1	420	60	0	60
0	1	1	dwa czynni	3	0	0	0	0

0	1	1	trzy i więc	3	60	8,57143	0	8,57143
0	0	1	jeden czyn	2	420	60	0	60
0	0	1	dwa czynni	2	240	34,2857	0	34,2857
0	1	1	trzy i więc	2	0	0	0	0
0	0	0	jeden czyn	3	90	12,8571	12,8571	0
0	1	1	trzy i więc	3	30	4,28571	0	4,28571
0	1	1	trzy i więc	3	0	0	0	0
1	1	1	trzy i więc	3	120	17,1429	0	17,1429
0	1	1	trzy i więc	3	0	0	0	0
0	0	1	trzy i więc	3	0	0	0	0
0	1	1	trzy i więc	1	0	0	0	0
1	1	1	trzy i więc	2	240	34,2857	0	34,2857
0	0	1	dwa czynni	2	210	30	12,8571	17,1429
0	1	1	dwa czynni	3	180	25,7143	25,7143	0
0	0	1	dwa czynni	2	0	0	0	0
0	0	1	dwa czynni	2	240	34,2857	34,2857	0
0	0	0	metabolicz	2	405	57,8571	23,5714	34,2857
1	1	1	trzy i więc	3	180	25,7143	0	25,7143
0	1	1	trzy i więc	3	0	0	0	0
0	1	1	dwa czynni	1	0	0	0	0
0	1	0	dwa czynni	1	30	4,28571	0	4,28571
1	0	1	trzy i więc	3	0	0	0	0
0	0	0	jeden czyn	2	30	4,28571	0	4,28571
0	0	0	metabolicz	1	120	17,1429	0	17,1429
0	1	1	trzy i więc	2	60	8,57143	0	8,57143
0	1	0	dwa czynni	2	0	0	0	0
1	0	1	trzy i więc	3	180	25,7143	0	25,7143
0	1	1	trzy i więc	3	0	0	0	0
0	0	1	dwa czynni	1	0	0	0	0
0	1	1	trzy i więc	2	120	17,1429	17,1429	0
0	0	1	dwa czynni	2	0	0	0	0
0	1	1	trzy i więc	3	270	38,5714	21,4286	17,1429
0	1	1	trzy i więc	2	480	68,5714	17,1429	51,4286
0	0	0	jeden czyn	3	120	17,1429	0	17,1429
0	0	1	dwa czynni	3	80	11,4286	0	11,4286
0	0	1	jeden czyn	1	145	20,7143	10	10,7143
1	0	1	trzy i więc	3	0	0	0	0
0	0	0	metabolicz	1	0	0	0	0
0	0	0	metabolicz	3	25	3,57143	0	3,57143
0	1	1	trzy i więc	2	240	34,2857	0	34,2857
0	1	1	trzy i więc	2	60	8,57143	0	8,57143
0	1	1	trzy i więc	2	0	0	0	0
0	0	1	dwa czynni	2	60	8,57143	0	8,57143
0	0	0	metabolicz	3	0	0	0	0
1	1	1	trzy i więc	3	60	8,57143	8,57143	0
0	0	1	jeden czyn	1	0	0	0	0
0	1	1	trzy i więc	3	360	51,4286	17,1429	34,2857
0	0	1	dwa czynni	3	60	8,57143	0	8,57143

0	1	1	dwa czynni	2	120	17,1429	0	17,1429
0	1	1	trzy i więce	2	120	17,1429	0	17,1429
1	0	1	trzy i więce	3	0	0	0	0
0	0	1	jeden czyn	2	0	0	0	0
0	1	1	trzy i więce	1	120	17,1429	17,1429	0
0	0	1	trzy i więce	1	0	0	0	0
0	0	1	jeden czyn	3	60	8,57143	0	8,57143
0	0	1	dwa czynni	2	0	0	0	0
0	0	1	trzy i więce	1	70	10	10	0
0	1	1	trzy i więce	1	180	25,7143	0	25,7143
0	1	1	trzy i więce	2	0	0	0	0
0	1	1	trzy i więce	3	60	8,57143	0	8,57143
1	1	1	trzy i więce	3	0	0	0	0
0	0	1	dwa czynni	3	420	60	34,2857	25,7143
0	0	1	dwa czynni	3	60	8,57143	0	8,57143
0	0	1	dwa czynni	1	0	0	0	0
1	0	0	dwa czynni	2	180	25,7143	25,7143	0
1	1	1	trzy i więce	3	360	51,4286	51,4286	0
0	1	0	dwa czynni	1	0	0	0	0
0	0	1	dwa czynni	2	0	0	0	0
0	1	0	dwa czynni	3	0	0	0	0
0	0	1	trzy i więce	1	720	102,857	0	102,857
1	1	1	trzy i więce	3	210	30	0	30
1	1	1	trzy i więce	1	420	60	0	60
0	0	1	dwa czynni	1	0	0	0	0
0	1	1	dwa czynni	1	0	0	0	0
0	0	0	dwa czynni	3	210	30	0	30
0	0	1	dwa czynni	2	80	11,4286	0	11,4286
1	1	1	trzy i więce	3	140	20	0	20
0	1	1	trzy i więce	3	0	0	0	0
1	0	1	trzy i więce	2	480	68,5714	17,1429	51,4286
0	1	1	trzy i więce	2	60	8,57143	0	8,57143
0	1	1	dwa czynni	2	60	8,57143	0	8,57143
0	1	1	trzy i więce	2	0	0	0	0
0	1	1	trzy i więce	3	190	27,1429	1,42857	25,7143
0	0	1	jeden czyn	3	0	0	0	0
0	1	0	dwa czynni	3	330	47,1429	17,1429	30
0	0	1	jeden czyn	1	0	0	0	0
1	1	1	trzy i więce	3	225	32,1429	15	17,1429
0	0	1	jeden czyn	2	780	111,429	51,4286	60
1	1	1	trzy i więce	1	60	8,57143	0	8,57143
0	1	1	trzy i więce	3	0	0	0	0
0	1	1	trzy i więce	1	360	51,4286	0	51,4286
0	0	1	jeden czyn	2	0	0	0	0
0	1	1	dwa czynni	2	0	0	0	0
0	1	0	trzy i więce	3	1440	205,714	120	85,7143
1	0	0	jeden czyn	3	15	2,14286	0	2,14286
0	1	1	trzy i więce	2	0	0	0	0

0	0	0	jeden czyn	2	0	0	0	0
1	0	1	trzy i więc	2	60	8,57143	0	8,57143
0	0	1	dwa czynni	3	20	2,85714	0	2,85714
0	0	1	dwa czynni	2	420	60	0	60
0	1	0	jeden czyn	3	0	0	0	0
0	1	0	jeden czyn	2	0	0	0	0
0	1	1	trzy i więc	2	60	8,57143	0	8,57143
0	1	0	dwa czynni	1	0	0	0	0
0	0	1	dwa czynni	2	70	10	10	0
0	1	1	trzy i więc	3	0	0	0	0
1	0	1	trzy i więc	1	45	6,42857	6,42857	0
0	1	1	trzy i więc	3	0	0	0	0
1	0	1	trzy i więc	2	0	0	0	0
0	0	1	dwa czynni	3	0	0	0	0
0	1	1	trzy i więc	2	60	8,57143	0	8,57143
0	1	1	trzy i więc	1	300	42,8571	25,7143	17,1429
0	0	1	jeden czyn	1	280	40	0	40
1	0	1	dwa czynni	3	50	7,14286	0	7,14286
0	1	1	trzy i więc	2	0	0	0	0
0	0	1	dwa czynni	3	540	77,1429	42,8571	34,2857
0	1	1	trzy i więc	2	0	0	0	0
0	1	0	dwa czynni	3	0	0	0	0
0	0	1	dwa czynni	2	270	38,5714	30	8,57143
0	0	0	dwa czynni	2	0	0	0	0
0	1	0	dwa czynni	3	135	19,2857	0	19,2857
0	0	1	dwa czynni	2	780	111,429	25,7143	85,7143
0	0	0	jeden czyn	2	0	0	0	0
0	1	1	trzy i więc	3	385	55	3,57143	51,4286
0	1	1	trzy i więc	2	0	0	0	0
0	0	0	metabolicz	3	150	21,4286	0	21,4286
0	0	1	jeden czyn	3	120	17,1429	0	17,1429
0	0	1	dwa czynni	2	0	0	0	0
0	1	1	dwa czynni	1	0	0	0	0
0	0	1	dwa czynni	2	540	77,1429	17,1429	60
0	1	1	trzy i więc	3	0	0	0	0
1	0	0	trzy i więc	3	60	8,57143	8,57143	0
1	1	1	trzy i więc	2	40	5,71429	0	5,71429
1	0	1	dwa czynni	2	60	8,57143	0	8,57143
0	0	1	trzy i więc	2	0	0	0	0
0	0	1	trzy i więc	3	120	17,1429	0	17,1429
1	1	1	trzy i więc	1	0	0	0	0
0	1	1	trzy i więc	1	100	14,2857	0	14,2857
0	1	1	trzy i więc	3	240	34,2857	0	34,2857
0	0	1	dwa czynni	1	0	0	0	0
1	1	1	trzy i więc	2	60	8,57143	0	8,57143
0	1	0	jeden czyn	2	120	17,1429	0	17,1429
0	1	0	dwa czynni	3	0	0	0	0
1	0	0	dwa czynni	1	300	42,8571	0	42,8571

0	0	1	trzy i więce	1	420	60	0	60
0	1	0	trzy i więce	1	360	51,4286	0	51,4286
0	0	0	metabolicz	3	0	0	0	0
0	1	1	trzy i więce	2	0	0	0	0
0	0	1	jeden czyn	1	0	0	0	0
0	1	0	dwa czynni	3	0	0	0	0
1	1	1	trzy i więce	3	120	17,1429	0	17,1429
0	0	1	jeden czyn	2	385	55	15	40
0	0	1	jeden czyn	3	120	17,1429	0	17,1429
0	1	1	trzy i więce	1	60	8,57143	0	8,57143
0	0	1	jeden czyn	2	870	124,286	64,2857	60
0	1	1	trzy i więce	2	240	34,2857	34,2857	0
0	0	1	dwa czynni	1	0	0	0	0
0	0	1	trzy i więce	3	150	21,4286	8,57143	12,8571
0	0	1	jeden czyn	2	420	60	30	30
0	0	1	dwa czynni	3	0	0	0	0
0	0	1	jeden czyn	1	420	60	42,8571	17,1429
0	1	1	trzy i więce	1	0	0	0	0
0	0	0	metabolicz	1	0	0	0	0
0	0	1	dwa czynni	3	0	0	0	0
0	0	1	trzy i więce	1	0	0	0	0
0	0	1	trzy i więce	2	60	8,57143	0	8,57143
0	1	1	trzy i więce	2	1020	145,714	85,7143	60
0	0	1	dwa czynni	2	310	44,2857	44,2857	0
0	0	1	jeden czyn	1	0	0	0	0
0	0	1	trzy i więce	2	0	0	0	0
0	1	0	jeden czyn	3	210	30	30	0
1	0	1	dwa czynni	3	120	17,1429	17,1429	0
0	0	1	dwa czynni	2	240	34,2857	34,2857	0
0	0	0	metabolicz	2	300	42,8571	0	42,8571
1	1	1	trzy i więce	3	0	0	0	0
0	1	1	trzy i więce	1	360	51,4286	0	51,4286
0	0	1	dwa czynni	3	360	51,4286	34,2857	17,1429
0	0	1	dwa czynni	3	30	4,28571	0	4,28571
0	0	1	trzy i więce	3	0	0	0	0
0	1	1	trzy i więce	2	0	0	0	0
1	0	1	trzy i więce	3	0	0	0	0
0	0	0	dwa czynni	3	30	4,28571	0	4,28571
1	1	0	trzy i więce	3	0	0	0	0
1	1	1	trzy i więce	2	0	0	0	0
0	1	0	jeden czyn	1	0	0	0	0
0	0	1	jeden czyn	1	0	0	0	0
1	1	0	trzy i więce	3	360	51,4286	0	51,4286
1	1	1	trzy i więce	3	630	90	0	90
1	1	0	trzy i więce	3	120	17,1429	17,1429	0
0	0	1	dwa czynni	3	480	68,5714	25,7143	42,8571
1	1	1	trzy i więce	2	60	8,57143	8,57143	0
0	1	1	trzy i więce	3	140	20	2,85714	17,1429

0	*0*	*1*	dwa czynni	1	*240*	*34,2857*	*0*	*34,2857*
0	*1*	*0*	jeden czyn	2	*100*	*14,2857*	*8,57143*	*5,71429*
0	*0*	*0*	jeden czyn	3	*30*	*4,28571*	*0*	*4,28571*
0	*1*	*1*	dwa czynni	1	*0*	*0*	*0*	*0*
0	*0*	*0*	dwa czynni	3	*140*	*20*	*8,57143*	*11,4286*
1	*1*	*1*	trzy i więc	1	*0*	*0*	*0*	*0*
0	*1*	*0*	trzy i więc	2	*320*	*45,7143*	*20*	*25,7143*
0	*0*	*1*	dwa czynni	2	*90*	*12,8571*	*0*	*12,8571*
0	*0*	*1*	dwa czynni	1	*240*	*34,2857*	*34,2857*	*0*
0	*0*	*0*	jeden czyn	2	*0*	*0*	*0*	*0*
0	*0*	*0*	dwa czynni	3	*60*	*8,57143*	*0*	*8,57143*
1	*0*	*1*	trzy i więc	3	*600*	*85,7143*	*51,4286*	*34,2857*
0	*1*	*1*	dwa czynni	1	*0*	*0*	*0*	*0*
0	*0*	*1*	jeden czyn	3	*540*	*77,1429*	*0*	*77,1429*
0	*0*	*0*	metabolicz	1	*0*	*0*	*0*	*0*
0	*0*	*1*	jeden czyn	3	*0*	*0*	*0*	*0*
0	*1*	*1*	trzy i więc	3	*270*	*38,5714*	*30*	*8,57143*
0	*1*	*1*	trzy i więc	3	*120*	*17,1429*	*0*	*17,1429*
0	*1*	*1*	trzy i więc	1	*0*	*0*	*0*	*0*
0	*0*	*0*	metabolicz	3	*1500*	*214,286*	*111,429*	*102,857*
0	*0*	*0*	jeden czyn	2	*90*	*12,8571*	*0*	*12,8571*
0	*0*	*0*	metabolicz	2	*120*	*17,1429*	*8,57143*	*8,57143*
0	*1*	*1*	trzy i więc	2	*0*	*0*	*0*	*0*
0	*0*	*1*	jeden czyn	2	*120*	*17,1429*	*0*	*17,1429*
0	*0*	*0*	metabolicz	3	*360*	*51,4286*	*0*	*51,4286*
0	*1*	*1*	trzy i więc	2	*420*	*60*	*34,2857*	*25,7143*
0	*0*	*0*	metabolicz	1	*0*	*0*	*0*	*0*
0	*1*	*1*	trzy i więc	3	*60*	*8,57143*	*0*	*8,57143*
0	*1*	*1*	trzy i więc	1	*0*	*0*	*0*	*0*
0	*0*	*1*	dwa czynni	1	*0*	*0*	*0*	*0*
1	*0*	*1*	trzy i więc	3	*0*	*0*	*0*	*0*
0	*0*	*1*	jeden czyn	3	*60*	*8,57143*	*0*	*8,57143*
0	*1*	*1*	dwa czynni	3	*0*	*0*	*0*	*0*
1	*1*	*0*	trzy i więc	2	*60*	*8,57143*	*0*	*8,57143*
0	*0*	*1*	jeden czyn	2	*1080*	*154,286*	*0*	*154,286*
0	*0*	*1*	trzy i więc	2	*0*	*0*	*0*	*0*
0	*1*	*0*	jeden czyn	3	*0*	*0*	*0*	*0*
0	*0*	*1*	jeden czyn	3	*0*	*0*	*0*	*0*
1	*0*	*1*	trzy i więc	3	*420*	*60*	*0*	*60*
0	*0*	*1*	jeden czyn	1	*0*	*0*	*0*	*0*
1	*0*	*0*	dwa czynni	2	*10*	*1,42857*	*0*	*1,42857*
0	*0*	*1*	dwa czynni	3	*180*	*25,7143*	*0*	*25,7143*
0	*1*	*1*	trzy i więc	2	*60*	*8,57143*	*0*	*8,57143*
0	*1*	*1*	trzy i więc	2	*360*	*51,4286*	*0*	*51,4286*
0	*1*	*1*	trzy i więc	1	*0*	*0*	*0*	*0*
1	*1*	*1*	trzy i więc	3	*300*	*42,8571*	*8,57143*	*34,2857*
0	*1*	*0*	dwa czynni	2	*900*	*128,571*	*0*	*128,571*
0	*0*	*1*	dwa czynni	1	*390*	*55,7143*	*4,28571*	*51,4286*

1	1	1	trzy i więce	2	240	34,2857	0	34,2857
0	0	1	trzy i więce	1	0	0	0	0
1	1	1	trzy i więce	1	420	60	0	60
0	0	1	dwa czynni	3	210	30	0	30
0	0	1	dwa czynni	1	420	60	0	60
1	1	0	trzy i więce	3	570	81,4286	21,4286	60
0	0	1	dwa czynni	2	30	4,28571	0	4,28571
0	0	1	dwa czynni	2	0	0	0	0
0	0	1	dwa czynni	1	40	5,71429	0	5,71429
0	0	1	jeden czyn	3	0	0	0	0
0	1	0	trzy i więce	1	0	0	0	0
0	0	1	dwa czynni	3	0	0	0	0
0	0	0	jeden czyn	2	150	21,4286	0	21,4286
1	1	1	trzy i więce	2	120	17,1429	0	17,1429
1	0	1	trzy i więce	3	60	8,57143	0	8,57143
0	0	1	jeden czyn	2	45	6,42857	6,42857	0
0	0	0	metabolicz	2	100	14,2857	14,2857	0
0	0	1	dwa czynni	3	0	0	0	0
0	0	1	dwa czynni	3	60	8,57143	0	8,57143
0	0	1	dwa czynni	2	120	17,1429	17,1429	0
0	1	1	trzy i więce	3	100	14,2857	14,2857	0
0	0	1	dwa czynni	1	0	0	0	0
0	0	0	jeden czyn	2	840	120	0	120
0	0	1	dwa czynni	2	0	0	0	0
1	1	1	trzy i więce	3	60	8,57143	0	8,57143
1	1	1	trzy i więce	3	420	60	0	60
0	0	1	dwa czynni	1	30	4,28571	0	4,28571
0	0	1	jeden czyn	3	0	0	0	0
1	0	0	dwa czynni	1	0	0	0	0
0	1	1	trzy i więce	3	0	0	0	0
0	1	1	trzy i więce	1	420	60	0	60
0	1	1	trzy i więce	3	0	0	0	0
0	1	0	jeden czyn	1	1260	180	180	0
0	1	1	trzy i więce	3	50	7,14286	7,14286	0
0	0	1	jeden czyn	2	0	0	0	0
0	0	1	trzy i więce	3	360	51,4286	0	51,4286
0	1	1	trzy i więce	3	0	0	0	0
0	0	1	jeden czyn	3	60	8,57143	0	8,57143
1	1	0	trzy i więce	3	1490	212,857	32,8571	180
0	0	1	jeden czyn	1	0	0	0	0
1	1	1	trzy i więce	1	600	85,7143	0	85,7143
0	0	0	metabolicz	3	0	0	0	0
0	0	0	jeden czyn	2	480	68,5714	0	68,5714
0	1	1	trzy i więce	3	0	0	0	0
1	1	1	trzy i więce	1	180	25,7143	0	25,7143
0	0	1	dwa czynni	3	0	0	0	0
0	0	1	dwa czynni	3	0	0	0	0
0	0	1	jeden czyn	1	0	0	0	0

0	0	1	dwa czynni	1	0	0	0	0
0	1	1	trzy i więce	3	360	51,4286	0	51,4286
1	0	1	trzy i więce	3	0	0	0	0
0	0	1	dwa czynni	3	0	0	0	0
0	0	0	metabolicz	2	60	8,57143	0	8,57143
0	0	0	jeden czyn	3	40	5,71429	0	5,71429
0	0	1	dwa czynni	3	60	8,57143	8,57143	0
0	1	1	trzy i więce	2	0	0	0	0
0	0	1	trzy i więce	2	0	0	0	0
0	1	1	trzy i więce	1	0	0	0	0
1	1	1	trzy i więce	3	120	17,1429	0	17,1429
1	0	0	dwa czynni	3	240	34,2857	4,28571	30
0	0	0	metabolicz	1	240	34,2857	17,1429	17,1429
0	0	1	dwa czynni	2	65	9,28571	6,42857	2,85714
0	1	1	trzy i więce	3	0	0	0	0
0	0	1	dwa czynni	2	60	8,57143	8,57143	0
0	1	1	trzy i więce	2	0	0	0	0
0	1	1	trzy i więce	3	0	0	0	0
0	0	1	dwa czynni	2	60	8,57143	0	8,57143
0	0	0	metabolicz	1	30	4,28571	0	4,28571
0	0	1	dwa czynni	2	75	10,7143	6,42857	4,28571
0	0	0	jeden czyn	1	45	6,42857	6,42857	0
0	0	1	dwa czynni	1	0	0	0	0
1	0	1	trzy i więce	3	360	51,4286	17,1429	34,2857
0	0	1	jeden czyn	2	0	0	0	0
0	0	1	dwa czynni	2	60	8,57143	0	8,57143
1	0	1	trzy i więce	3	60	8,57143	8,57143	0
1	0	1	trzy i więce	3	120	17,1429	17,1429	0
0	0	1	trzy i więce	3	30	4,28571	0	4,28571
0	0	1	trzy i więce	2	240	34,2857	0	34,2857
0	1	1	trzy i więce	3	0	0	0	0
0	1	0	dwa czynni	3	0	0	0	0
0	0	1	dwa czynni	3	0	0	0	0
0	1	0	dwa czynni	3	420	60	0	60
1	0	1	trzy i więce	2	0	0	0	0
0	1	0	dwa czynni	2	60	8,57143	0	8,57143
0	0	1	dwa czynni	2	60	8,57143	0	8,57143
0	1	1	trzy i więce	2	120	17,1429	0	17,1429
0	0	0	jeden czyn	1	105	15	15	0
0	0	1	trzy i więce	3	240	34,2857	17,1429	17,1429
0	1	1	dwa czynni	1	0	0	0	0
0	1	1	trzy i więce	2	540	77,1429	0	77,1429
0	1	1	trzy i więce	2	350	50	0	50
1	1	1	trzy i więce	2	0	0	0	0
0	1	0	trzy i więce	2	120	17,1429	17,1429	0
1	1	0	dwa czynni	1	120	17,1429	0	17,1429
1	0	1	trzy i więce	2	840	120	120	0
0	1	1	trzy i więce	3	0	0	0	0

0	1	1	trzy i więce	2	0	0	0	0
1	0	1	trzy i więce	1	60	8,57143	0	8,57143
0	0	1	jeden czyn	1	0	0	0	0
0	0	1	dwa czynni	3	240	34,2857	34,2857	0
0	0	1	dwa czynni	3	300	42,8571	0	42,8571
0	0	1	jeden czyn	3	120	17,1429	17,1429	0
0	1	1	trzy i więce	2	0	0	0	0
0	0	1	dwa czynni	3	0	0	0	0
0	0	1	dwa czynni	2	300	42,8571	17,1429	25,7143
0	1	1	trzy i więce	3	0	0	0	0
1	1	1	trzy i więce	2	0	0	0	0
0	1	1	dwa czynni	1	0	0	0	0
0	1	1	trzy i więce	2	60	8,57143	8,57143	0
0	1	1	trzy i więce	3	120	17,1429	0	17,1429
0	1	1	trzy i więce	3	0	0	0	0
1	1	1	trzy i więce	3	0	0	0	0
0	0	1	dwa czynni	2	0	0	0	0
0	0	1	trzy i więce	3	420	60	0	60
0	1	1	trzy i więce	3	120	17,1429	17,1429	0
0	0	1	jeden czyn	3	540	77,1429	77,1429	0
0	0	1	jeden czyn	3	70	10	10	0
0	1	1	trzy i więce	3	0	0	0	0
0	1	1	trzy i więce	2	90	12,8571	0	12,8571
0	1	1	trzy i więce	3	0	0	0	0
0	0	1	dwa czynni	3	0	0	0	0
0	0	1	dwa czynni	2	0	0	0	0
0	0	1	dwa czynni	3	0	0	0	0
1	1	0	trzy i więce	1	240	34,2857	0	34,2857
0	1	0	dwa czynni	2	0	0	0	0
1	0	1	trzy i więce	3	0	0	0	0
0	0	1	dwa czynni	3	0	0	0	0
0	0	1	dwa czynni	3	540	77,1429	51,4286	25,7143
0	1	1	trzy i więce	2	0	0	0	0
0	1	1	trzy i więce	2	60	8,57143	0	8,57143
0	0	0	metabolicz	1	1260	180	0	180
0	0	1	dwa czynni	3	0	0	0	0
0	0	1	dwa czynni	3	120	17,1429	0	17,1429
1	0	1	trzy i więce	3	180	25,7143	0	25,7143
1	1	1	trzy i więce	2	0	0	0	0
0	1	1	trzy i więce	2	120	17,1429	0	17,1429
1	1	0	trzy i więce	3	60	8,57143	8,57143	0
0	1	1	trzy i więce	3	0	0	0	0
0	1	1	trzy i więce	2	0	0	0	0
0	1	1	dwa czynni	2	30	4,28571	0	4,28571
1	0	1	trzy i więce	1	0	0	0	0
0	1	1	trzy i więce	3	0	0	0	0
0	0	1	jeden czyn	3	50	7,14286	1,42857	5,71429
0	0	1	dwa czynni	3	210	30	12,8571	17,1429

0	0	1	trzy i więc	3	720	102,857	51,4286	51,4286
1	0	1	dwa czynni	2	0	0	0	0
0	1	1	trzy i więc	2	0	0	0	0
0	1	1	trzy i więc	1	60	8,57143	0	8,57143
0	0	0	jeden czyn	2	0	0	0	0
0	0	0	dwa czynni	2	40	5,71429	0	5,71429
0	1	1	trzy i więc	3	0	0	0	0
0	0	1	jeden czyn	1	0	0	0	0
0	0	1	trzy i więc	3	0	0	0	0
0	0	0	metabolicz	2	210	30	0	30
1	1	1	trzy i więc	3	300	42,8571	30	12,8571
0	1	1	trzy i więc	2	120	17,1429	0	17,1429
0	0	0	jeden czyn	2	30	4,28571	0	4,28571
0	0	0	metabolicz	2	0	0	0	0
1	1	1	trzy i więc	3	120	17,1429	0	17,1429
0	0	1	dwa czynni	2	30	4,28571	0	4,28571
0	0	1	jeden czyn	1	540	77,1429	51,4286	25,7143
0	0	0	metabolicz	2	60	8,57143	0	8,57143
0	0	0	metabolicz	3	45	6,42857	0	6,42857
1	1	1	trzy i więc	1	0	0	0	0
0	0	1	dwa czynni	1	0	0	0	0
0	1	1	trzy i więc	2	420	60	0	60
0	1	1	dwa czynni	3	420	60	8,57143	51,4286
0	0	1	dwa czynni	2	90	12,8571	0	12,8571
0	1	1	dwa czynni	2	180	25,7143	8,57143	17,1429
1	1	1	trzy i więc	3	0	0	0	0
0	0	1	dwa czynni	3	135	19,2857	0	19,2857
0	0	1	dwa czynni	3	490	70	30	40
1	1	1	trzy i więc	1	0	0	0	0
0	1	1	dwa czynni	2	0	0	0	0
0	0	1	dwa czynni	1	0	0	0	0
0	0	1	jeden czyn	2	300	42,8571	34,2857	8,57143
0	0	1	trzy i więc	2	460	65,7143	57,1429	8,57143
1	1	1	trzy i więc	3	0	0	0	0
0	0	0	metabolicz	3	350	50	0	50
0	0	1	dwa czynni	3	0	0	0	0
0	0	1	jeden czyn	3	0	0	0	0
0	0	1	jeden czyn	2	0	0	0	0
0	0	0	dwa czynni	1	0	0	0	0
0	0	0	jeden czyn	2	0	0	0	0
0	1	1	trzy i więc	3	0	0	0	0
0	1	0	jeden czyn	3	0	0	0	0
0	0	0	metabolicz	2	0	0	0	0
1	0	0	trzy i więc	3	0	0	0	0
0	0	1	dwa czynni	2	0	0	0	0
0	0	1	trzy i więc	2	60	8,57143	0	8,57143
0	0	1	jeden czyn	1	60	8,57143	0	8,57143
0	0	1	dwa czynni	3	0	0	0	0

0	0	1	jeden czyn	2	60	8,57143	0	8,57143
0	1	1	trzy i więc	2	60	8,57143	0	8,57143
0	0	1	jeden czyn	2	70	10	10	0
0	1	1	trzy i więc	1	0	0	0	0
0	1	1	trzy i więc	1	120	17,1429	0	17,1429
0	1	1	trzy i więc	1	90	12,8571	12,8571	0
0	1	0	dwa czynni	2	0	0	0	0
0	1	1	dwa czynni	3	380	54,2857	45,7143	8,57143
0	0	1	jeden czyn	3	60	8,57143	8,57143	0
0	0	1	dwa czynni	3	840	120	0	120
0	1	1	dwa czynni	1	120	17,1429	0	17,1429
0	0	1	jeden czyn	1	0	0	0	0
0	0	0	dwa czynni	2	0	0	0	0
0	0	0	dwa czynni	1	0	0	0	0
0	0	0	metabolicz	2	0	0	0	0
0	0	1	dwa czynni	3	285	40,7143	6,42857	34,2857
0	0	0	metabolicz	3	0	0	0	0
0	1	1	trzy i więc	3	120	17,1429	0	17,1429
0	1	0	jeden czyn	2	360	51,4286	0	51,4286
0	0	1	jeden czyn	2	120	17,1429	0	17,1429
0	0	0	metabolicz	1	0	0	0	0
0	1	1	trzy i więc	2	60	8,57143	0	8,57143
0	0	1	dwa czynni	2	420	60	0	60
0	0	1	jeden czyn	2	180	25,7143	0	25,7143
1	0	1	trzy i więc	1	0	0	0	0
0	1	1	dwa czynni	2	390	55,7143	25,7143	30
0	1	1	trzy i więc	2	330	47,1429	17,1429	30
1	0	1	trzy i więc	3	0	0	0	0
0	1	1	trzy i więc	2	540	77,1429	17,1429	60
1	0	1	trzy i więc	2	0	0	0	0
0	0	1	dwa czynni	3	0	0	0	0
0	0	1	jeden czyn	1	20	2,85714	0	2,85714
0	0	0	metabolicz	3	0	0	0	0
0	0	1	jeden czyn	2	240	34,2857	34,2857	0
0	0	1	dwa czynni	1	0	0	0	0
0	0	1	jeden czyn	2	60	8,57143	0	8,57143
0	1	1	trzy i więc	1	0	0	0	0
0	0	1	dwa czynni	3	270	38,5714	8,57143	30
0	0	0	jeden czyn	2	210	30	17,1429	12,8571
0	0	1	trzy i więc	2	90	12,8571	0	12,8571
1	1	1	trzy i więc	3	75	10,7143	2,14286	8,57143
0	0	1	dwa czynni	3	0	0	0	0
0	0	1	dwa czynni	3	390	55,7143	21,4286	34,2857
0	1	1	dwa czynni	3	90	12,8571	0	12,8571
1	1	1	trzy i więc	1	300	42,8571	0	42,8571
0	1	1	trzy i więc	3	90	12,8571	12,8571	0
1	0	1	dwa czynni	1	0	0	0	0
0	1	1	trzy i więc	2	0	0	0	0

0	1	1	trzy i więce	2	300	42,8571	8,57143	34,2857
1	0	1	trzy i więce	2	150	21,4286	12,8571	8,57143
1	1	0	trzy i więce	2	120	17,1429	17,1429	0
0	1	1	dwa czynni	1	0	0	0	0
0	0	1	dwa czynni	1	240	34,2857	0	34,2857
0	0	1	dwa czynni	3	360	51,4286	51,4286	0
1	1	1	trzy i więce	1	840	120	85,7143	34,2857
0	1	1	trzy i więce	3	0	0	0	0
0	1	1	dwa czynni	2	210	30	0	30
0	0	1	dwa czynni	1	0	0	0	0
0	1	1	dwa czynni	2	60	8,57143	0	8,57143
0	0	1	dwa czynni	2	0	0	0	0
0	0	1	trzy i więce	3	40	5,71429	0	5,71429
0	0	1	trzy i więce	3	660	94,2857	25,7143	68,5714
0	1	1	trzy i więce	2	60	8,57143	0	8,57143
0	1	1	trzy i więce	2	60	8,57143	0	8,57143
0	0	0	metabolicz	2	120	17,1429	8,57143	8,57143
0	0	0	dwa czynni	3	60	8,57143	0	8,57143
0	0	1	trzy i więce	3	420	60	60	0
0	1	1	trzy i więce	2	1140	162,857	8,57143	154,286
1	0	1	trzy i więce	3	30	4,28571	0	4,28571
0	1	1	trzy i więce	3	240	34,2857	0	34,2857
0	1	0	dwa czynni	2	120	17,1429	0	17,1429
0	0	0	metabolicz	1	15	2,14286	0	2,14286
0	1	1	trzy i więce	1	20	2,85714	0	2,85714
0	0	1	dwa czynni	3	0	0	0	0
1	0	1	trzy i więce	2	60	8,57143	8,57143	0
0	0	1	dwa czynni	1	180	25,7143	25,7143	0
0	1	1	trzy i więce	2	60	8,57143	0	8,57143
0	1	1	trzy i więce	2	0	0	0	0
0	0	1	jeden czyn	2	600	85,7143	0	85,7143
0	0	1	dwa czynni	3	60	8,57143	0	8,57143
0	0	0	jeden czyn	3	0	0	0	0
0	1	1	dwa czynni	2	150	21,4286	12,8571	8,57143
0	0	1	jeden czyn	2	0	0	0	0
0	1	1	trzy i więce	3	720	102,857	0	102,857
0	0	1	trzy i więce	3	0	0	0	0
0	0	1	dwa czynni	3	630	90	0	90
1	1	1	trzy i więce	2	420	60	17,1429	42,8571
0	0	1	jeden czyn	3	360	51,4286	0	51,4286
0	0	1	trzy i więce	1	0	0	0	0
0	1	1	trzy i więce	3	0	0	0	0
0	0	0	metabolicz	2	240	34,2857	0	34,2857
0	0	1	dwa czynni	3	90	12,8571	0	12,8571
0	0	1	dwa czynni	3	60	8,57143	8,57143	0
0	0	0	jeden czyn	2	0	0	0	0
0	0	0	dwa czynni	2	480	68,5714	51,4286	17,1429
0	0	1	dwa czynni	2	0	0	0	0

1	0	1	trzy i więce	1	60	8,57143	8,57143	0
1	1	1	trzy i więce	3	60	8,57143	0	8,57143
0	1	1	trzy i więce	3	120	17,1429	0	17,1429
0	1	1	trzy i więce	3	420	60	0	60
0	0	0	metabolicz	2	0	0	0	0
0	0	1	dwa czynni	2	60	8,57143	0	8,57143
0	1	1	trzy i więce	3	60	8,57143	0	8,57143
1	1	1	trzy i więce	1	120	17,1429	0	17,1429
0	0	1	jeden czyn	3	60	8,57143	0	8,57143
0	0	1	trzy i więce	3	0	0	0	0
0	0	1	dwa czynni	3	300	42,8571	25,7143	17,1429
0	1	1	trzy i więce	3	240	34,2857	17,1429	17,1429
0	1	1	trzy i więce	1	0	0	0	0
0	0	1	dwa czynni	2	180	25,7143	0	25,7143
1	0	1	trzy i więce	1	0	0	0	0
0	1	1	trzy i więce	3	0	0	0	0
0	0	0	jeden czyn	2	120	17,1429	17,1429	0
0	0	0	jeden czyn	3	180	25,7143	0	25,7143
0	1	0	dwa czynni	3	0	0	0	0
1	0	1	trzy i więce	2	0	0	0	0
1	0	0	trzy i więce	1	0	0	0	0
1	0	0	dwa czynni	1	30	4,28571	0	4,28571
0	1	0	trzy i więce	3	0	0	0	0
0	1	1	trzy i więce	2	0	0	0	0
1	1	1	trzy i więce	2	30	4,28571	0	4,28571
0	0	1	dwa czynni	3	840	120	0	120
0	0	0	jeden czyn	2	420	60	0	60
0	0	0	jeden czyn	1	120	17,1429	0	17,1429
0	0	1	jeden czyn	2	1305	186,429	6,42857	180
0	1	0	dwa czynni	3	0	0	0	0
1	0	0	dwa czynni	3	120	17,1429	0	17,1429
1	1	1	trzy i więce	2	0	0	0	0
0	0	1	dwa czynni	2	420	60	0	60
0	0	1	jeden czyn	1	60	8,57143	0	8,57143
0	0	1	trzy i więce	1	240	34,2857	17,1429	17,1429
0	0	1	jeden czyn	1	120	17,1429	0	17,1429
0	0	1	dwa czynni	1	240	34,2857	25,7143	8,57143
0	0	0	dwa czynni	1	210	30	0	30
0	1	1	dwa czynni	2	60	8,57143	0	8,57143
0	0	1	dwa czynni	3	0	0	0	0
1	0	1	trzy i więce	2	170	24,2857	21,4286	2,85714
0	1	1	trzy i więce	3	0	0	0	0
0	0	1	dwa czynni	3	0	0	0	0
0	1	0	dwa czynni	2	0	0	0	0
0	0	1	dwa czynni	3	720	102,857	0	102,857
0	0	1	dwa czynni	2	45	6,42857	6,42857	0
0	0	1	jeden czyn	3	180	25,7143	0	25,7143
0	0	1	dwa czynni	3	0	0	0	0

0	0	1	trzy i więce	2	120	17,1429	0	17,1429
0	0	1	jeden czyn	3	30	4,28571	0	4,28571
0	1	0	jeden czyn	3	105	15	0	15
0	1	1	trzy i więce	3	30	4,28571	0	4,28571
0	0	1	dwa czynni	3	420	60	17,1429	42,8571
0	0	1	dwa czynni	1	60	8,57143	0	8,57143
0	0	1	jeden czyn	2	360	51,4286	0	51,4286
0	1	1	dwa czynni	3	320	45,7143	25,7143	20
1	1	1	trzy i więce	3	420	60	0	60
1	1	1	trzy i więce	3	385	55	15	40
0	1	1	trzy i więce	2	0	0	0	0
0	0	1	jeden czyn	2	180	25,7143	0	25,7143
0	1	1	trzy i więce	3	0	0	0	0
0	0	1	dwa czynni	2	60	8,57143	8,57143	0
0	0	1	dwa czynni	3	30	4,28571	0	4,28571
0	0	1	jeden czyn	2	120	17,1429	0	17,1429
1	0	1	trzy i więce	2	0	0	0	0
0	0	1	trzy i więce	3	210	30	0	30
0	0	0	jeden czyn	1	690	98,5714	90	8,57143
0	1	1	dwa czynni	3	0	0	0	0
0	0	1	jeden czyn	1	0	0	0	0
0	0	1	dwa czynni	1	150	21,4286	4,28571	17,1429
0	1	1	trzy i więce	2	0	0	0	0
1	0	1	trzy i więce	3	30	4,28571	0	4,28571
0	0	0	jeden czyn	3	210	30	0	30
0	1	1	trzy i więce	2	120	17,1429	0	17,1429
0	0	1	dwa czynni	3	120	17,1429	17,1429	0
0	1	1	trzy i więce	3	180	25,7143	12,8571	12,8571
0	1	1	trzy i więce	2	0	0	0	0
0	0	0	metabolicz	2	180	25,7143	0	25,7143
0	0	1	jeden czyn	2	210	30	21,4286	8,57143
1	1	1	trzy i więce	2	60	8,57143	0	8,57143
1	0	1	trzy i więce	2	0	0	0	0
0	1	1	trzy i więce	2	0	0	0	0
0	0	1	dwa czynni	3	300	42,8571	25,7143	17,1429
0	0	0	metabolicz	2	0	0	0	0
1	1	1	trzy i więce	3	120	17,1429	0	17,1429
0	1	0	dwa czynni	2	90	12,8571	0	12,8571
1	0	1	trzy i więce	2	0	0	0	0
0	1	1	trzy i więce	3	0	0	0	0
0	0	1	jeden czyn	1	0	0	0	0
0	1	1	trzy i więce	2	0	0	0	0
0	0	1	dwa czynni	3	150	21,4286	1,42857	20
1	0	1	trzy i więce	2	180	25,7143	25,7143	0
0	1	1	trzy i więce	2	0	0	0	0
0	1	1	trzy i więce	1	60	8,57143	0	8,57143
0	1	1	dwa czynni	3	280	40	40	0
0	0	1	dwa czynni	3	60	8,57143	0	8,57143

0	0	1	trzy i więce	1	20	2,85714	0	2,85714
0	1	1	dwa czynni	3	105	15	15	0
0	0	1	trzy i więce	3	0	0	0	0
0	1	1	trzy i więce	3	0	0	0	0
0	0	1	jeden czyn	2	560	80	20	60
0	1	1	trzy i więce	2	90	12,8571	12,8571	0
0	0	0	jeden czyn	2	0	0	0	0
0	1	1	trzy i więce	3	120	17,1429	0	17,1429
0	0	1	jeden czyn	1	90	12,8571	12,8571	0
0	0	0	dwa czynni	2	30	4,28571	0	4,28571
0	0	1	jeden czyn	2	210	30	0	30
0	1	1	trzy i więce	1	0	0	0	0
1	0	1	trzy i więce	3	420	60	25,7143	34,2857
0	0	1	dwa czynni	1	480	68,5714	68,5714	0
0	1	1	trzy i więce	3	120	17,1429	0	17,1429
1	0	1	trzy i więce	3	75	10,7143	8,57143	2,14286
0	0	0	jeden czyn	3	60	8,57143	0	8,57143
1	0	1	trzy i więce	1	90	12,8571	0	12,8571
1	1	1	trzy i więce	3	130	18,5714	10	8,57143
0	0	0	metabolicz	2	180	25,7143	21,4286	4,28571
1	1	1	trzy i więce	2	0	0	0	0
0	0	1	dwa czynni	3	0	0	0	0
1	0	1	trzy i więce	3	90	12,8571	12,8571	0
1	0	1	trzy i więce	3	210	30	0	30
0	1	0	dwa czynni	2	45	6,42857	6,42857	0
0	0	1	jeden czyn	3	240	34,2857	17,1429	17,1429
0	0	1	jeden czyn	2	270	38,5714	0	38,5714
0	0	1	dwa czynni	3	150	21,4286	4,28571	17,1429
0	1	1	trzy i więce	2	0	0	0	0
1	0	1	trzy i więce	3	0	0	0	0
0	1	1	trzy i więce	1	1050	150	90	60
0	1	1	trzy i więce	1	0	0	0	0
0	0	1	dwa czynni	3	660	94,2857	85,7143	8,57143
0	1	1	trzy i więce	2	0	0	0	0
1	0	1	trzy i więce	2	0	0	0	0
1	0	0	jeden czyn	2	240	34,2857	34,2857	0
0	1	1	trzy i więce	1	0	0	0	0
0	1	1	dwa czynni	3	0	0	0	0
0	0	0	dwa czynni	3	510	72,8571	38,5714	34,2857
0	0	1	dwa czynni	1	0	0	0	0
0	0	0	jeden czyn	3	0	0	0	0
1	1	1	trzy i więce	1	0	0	0	0
0	1	0	dwa czynni	2	60	8,57143	8,57143	0
0	0	1	trzy i więce	2	0	0	0	0
0	0	1	trzy i więce	2	120	17,1429	17,1429	0
0	1	1	trzy i więce	3	360	51,4286	0	51,4286
0	0	1	dwa czynni	2	210	30	8,57143	21,4286
0	0	1	dwa czynni	2	320	45,7143	45,7143	0

0	1	1	trzy i więce	1	0	0	0	0
0	1	1	trzy i więce	2	180	25,7143	8,57143	17,1429
0	0	1	dwa czynni	3	120	17,1429	0	17,1429
0	0	1	jeden czyn	3	60	8,57143	0	8,57143
0	1	1	trzy i więce	1	0	0	0	0
0	1	1	trzy i więce	3	270	38,5714	0	38,5714
1	1	1	trzy i więce	3	420	60	0	60
0	1	1	trzy i więce	1	0	0	0	0
1	0	1	trzy i więce	3	0	0	0	0
0	0	1	jeden czyn	1	180	25,7143	0	25,7143
0	1	1	trzy i więce	2	120	17,1429	8,57143	8,57143
0	0	1	jeden czyn	3	460	65,7143	40	25,7143
1	0	1	trzy i więce	2	0	0	0	0
1	0	1	trzy i więce	2	0	0	0	0
0	1	1	dwa czynni	1	105	15	6,42857	8,57143
0	0	1	jeden czyn	1	20	2,85714	0	2,85714
0	0	1	trzy i więce	3	0	0	0	0
0	1	1	trzy i więce	1	230	32,8571	11,4286	21,4286
0	0	1	dwa czynni	2	105	15	6,42857	8,57143
0	0	1	dwa czynni	2	360	51,4286	0	51,4286
0	0	0	dwa czynni	1	120	17,1429	0	17,1429
1	1	1	trzy i więce	1	300	42,8571	34,2857	8,57143
1	0	1	trzy i więce	1	60	8,57143	0	8,57143
0	1	1	trzy i więce	2	105	15	0	15
0	1	1	dwa czynni	1	0	0	0	0
1	1	1	trzy i więce	3	600	85,7143	0	85,7143
0	0	1	jeden czyn	3	210	30	30	0
0	1	1	trzy i więce	2	1560	222,857	180	42,8571
1	0	1	trzy i więce	1	0	0	0	0
0	0	0	metabolicz	2	1470	210	30	180
1	0	1	trzy i więce	2	360	51,4286	17,1429	34,2857
0	1	1	trzy i więce	3	60	8,57143	0	8,57143
0	0	1	jeden czyn	2	240	34,2857	25,7143	8,57143
0	0	1	dwa czynni	2	0	0	0	0
1	0	1	trzy i więce	3	60	8,57143	0	8,57143
0	1	1	trzy i więce	1	0	0	0	0
1	1	1	trzy i więce	3	70	10	4,28571	5,71429
0	0	1	dwa czynni	2	0	0	0	0
0	0	0	metabolicz	1	570	81,4286	51,4286	30
0	1	1	trzy i więce	1	40	5,71429	0	5,71429
0	0	1	dwa czynni	2	0	0	0	0
0	1	1	dwa czynni	2	450	64,2857	64,2857	0
1	1	1	trzy i więce	2	0	0	0	0
1	0	1	trzy i więce	2	0	0	0	0
0	0	1	dwa czynni	2	240	34,2857	8,57143	25,7143
0	0	1	jeden czyn	3	690	98,5714	68,5714	30
1	0	1	trzy i więce	3	0	0	0	0
0	0	1	jeden czyn	3	120	17,1429	17,1429	0

0	0	0	metabolicz	2	300	42,8571	17,1429	25,7143
0	0	0	jeden czyn	3	0	0	0	0
0	1	1	trzy i więc	3	80	11,4286	0	11,4286
0	1	0	trzy i więc	2	600	85,7143	77,1429	8,57143
1	0	1	trzy i więc	3	30	4,28571	0	4,28571
0	0	0	jeden czyn	1	180	25,7143	25,7143	0
1	1	0	trzy i więc	1	180	25,7143	0	25,7143
0	0	1	dwa czynni	3	0	0	0	0
0	0	1	dwa czynni	2	60	8,57143	0	8,57143
0	1	1	trzy i więc	3	540	77,1429	51,4286	25,7143
0	1	1	trzy i więc	3	150	21,4286	0	21,4286
0	0	0	dwa czynni	2	120	17,1429	0	17,1429
1	1	1	trzy i więc	3	0	0	0	0
0	0	1	jeden czyn	2	255	36,4286	23,5714	12,8571
0	0	1	dwa czynni	3	0	0	0	0
0	1	1	trzy i więc	3	210	30	0	30
1	0	1	trzy i więc	1	0	0	0	0
0	1	1	trzy i więc	3	240	34,2857	0	34,2857
0	0	0	metabolicz	3	0	0	0	0
0	1	1	dwa czynni	2	120	17,1429	0	17,1429
1	1	1	trzy i więc	1	0	0	0	0
0	1	1	trzy i więc	2	90	12,8571	0	12,8571
1	0	1	trzy i więc	3	120	17,1429	0	17,1429
0	0	0	metabolicz	3	0	0	0	0
0	0	1	jeden czyn	2	60	8,57143	8,57143	0
0	0	0	jeden czyn	3	0	0	0	0
0	1	1	trzy i więc	1	60	8,57143	0	8,57143
0	1	1	dwa czynni	3	720	102,857	0	102,857
0	0	0	jeden czyn	3	180	25,7143	25,7143	0
1	1	1	trzy i więc	2	0	0	0	0
0	1	1	trzy i więc	1	120	17,1429	0	17,1429
0	1	1	trzy i więc	3	70	10	5,71429	4,28571
0	1	1	trzy i więc	1	720	102,857	0	102,857
1	1	1	trzy i więc	3	180	25,7143	0	25,7143
0	0	1	jeden czyn	1	420	60	0	60
0	0	1	dwa czynni	3	120	17,1429	0	17,1429
0	0	0	jeden czyn	1	0	0	0	0
0	0	1	trzy i więc	2	240	34,2857	0	34,2857
1	1	1	trzy i więc	3	0	0	0	0
0	0	1	dwa czynni	2	360	51,4286	0	51,4286
0	0	1	dwa czynni	2	180	25,7143	17,1429	8,57143
0	0	1	dwa czynni	2	40	5,71429	0	5,71429
1	1	1	trzy i więc	3	240	34,2857	12,8571	21,4286
0	1	1	trzy i więc	3	90	12,8571	0	12,8571
0	0	1	trzy i więc	2	240	34,2857	0	34,2857
0	1	1	trzy i więc	2	0	0	0	0
0	0	1	jeden czyn	1	0	0	0	0
1	1	1	trzy i więc	1	0	0	0	0

0	1	1	trzy i więce	3	0	0	0	0
0	1	1	dwa czynni	3	210	30	30	0
0	0	1	dwa czynni	2	180	25,7143	0	25,7143
0	0	0	dwa czynni	3	0	0	0	0
0	0	0	jeden czyn	1	120	17,1429	0	17,1429
0	0	1	jeden czyn	1	0	0	0	0
0	0	1	trzy i więce	1	0	0	0	0
1	0	1	trzy i więce	3	0	0	0	0
0	0	1	jeden czyn	2	360	51,4286	0	51,4286
0	0	1	jeden czyn	3	120	17,1429	17,1429	0
0	1	1	trzy i więce	2	0	0	0	0
0	0	1	dwa czynni	3	360	51,4286	12,8571	38,5714
1	1	1	trzy i więce	3	0	0	0	0
0	1	1	dwa czynni	1	0	0	0	0
0	0	1	jeden czyn	2	0	0	0	0
0	1	1	trzy i więce	1	0	0	0	0
0	0	0	metabolicz	2	0	0	0	0
0	1	1	trzy i więce	3	0	0	0	0
0	0	1	dwa czynni	3	150	21,4286	0	21,4286
1	1	1	trzy i więce	1	0	0	0	0
0	1	1	trzy i więce	1	140	20	0	20
1	1	1	trzy i więce	3	420	60	0	60
0	0	0	jeden czyn	3	420	60	0	60
0	1	1	trzy i więce	3	480	68,5714	0	68,5714
0	1	1	trzy i więce	2	240	34,2857	8,57143	25,7143
0	0	1	jeden czyn	3	140	20	20	0
0	0	0	jeden czyn	2	180	25,7143	25,7143	0
0	0	1	trzy i więce	1	0	0	0	0
0	0	1	jeden czyn	2	60	8,57143	0	8,57143
0	0	1	dwa czynni	2	315	45	0	45
0	0	1	dwa czynni	3	60	8,57143	0	8,57143
0	1	0	dwa czynni	2	360	51,4286	0	51,4286
1	0	1	trzy i więce	3	0	0	0	0
0	1	1	trzy i więce	3	120	17,1429	0	17,1429
0	0	1	dwa czynni	2	720	102,857	51,4286	51,4286
0	0	1	jeden czyn	1	1260	180	0	180
0	0	1	dwa czynni	3	420	60	8,57143	51,4286
0	0	1	dwa czynni	2	420	60	0	60
0	1	0	dwa czynni	2	60	8,57143	8,57143	0
0	1	1	trzy i więce	3	0	0	0	0
0	1	1	trzy i więce	3	0	0	0	0
0	0	1	dwa czynni	1	60	8,57143	0	8,57143
0	1	1	trzy i więce	3	180	25,7143	0	25,7143
0	0	1	dwa czynni	2	120	17,1429	0	17,1429
0	0	1	dwa czynni	1	0	0	0	0
0	1	1	trzy i więce	1	0	0	0	0
0	0	1	jeden czyn	1	120	17,1429	0	17,1429
0	1	1	trzy i więce	1	0	0	0	0

0	1	1	trzy i więce	2	0	0	0	0
0	0	1	dwa czynni	3	120	17,1429	17,1429	0
0	0	0	metabolicz	2	180	25,7143	0	25,7143
0	1	1	trzy i więce	3	140	20	0	20
0	1	0	jeden czyn	1	0	0	0	0
0	1	1	trzy i więce	3	300	42,8571	42,8571	0
0	0	1	trzy i więce	3	60	8,57143	0	8,57143
0	1	1	trzy i więce	2	60	8,57143	0	8,57143
0	0	0	metabolicz	2	180	25,7143	0	25,7143
0	1	1	trzy i więce	2	15	2,14286	0	2,14286
1	0	1	dwa czynni	2	105	15	6,42857	8,57143
1	1	1	trzy i więce	1	75	10,7143	10,7143	0
0	0	1	dwa czynni	1	585	83,5714	38,5714	45
0	0	1	dwa czynni	2	240	34,2857	17,1429	17,1429
0	1	1	trzy i więce	2	60	8,57143	8,57143	0
0	1	1	trzy i więce	1	30	4,28571	0	4,28571
0	1	1	trzy i więce	3	0	0	0	0
0	1	1	trzy i więce	3	120	17,1429	0	17,1429
1	1	1	trzy i więce	1	600	85,7143	51,4286	34,2857
0	1	1	trzy i więce	3	0	0	0	0
0	0	1	dwa czynni	3	630	90	0	90
1	1	0	trzy i więce	3	420	60	60	0
0	0	1	jeden czyn	3	30	4,28571	0	4,28571
0	0	0	jeden czyn	3	0	0	0	0
0	1	1	trzy i więce	3	0	0	0	0
0	1	1	dwa czynni	2	120	17,1429	0	17,1429
1	0	1	trzy i więce	3	165	23,5714	6,42857	17,1429
0	1	1	trzy i więce	3	0	0	0	0
0	0	0	metabolicz	3	0	0	0	0
0	0	1	trzy i więce	3	660	94,2857	17,1429	77,1429
0	1	1	dwa czynni	2	0	0	0	0
0	0	1	dwa czynni	3	0	0	0	0
1	1	1	trzy i więce	3	330	47,1429	25,7143	21,4286
0	0	0	dwa czynni	3	300	42,8571	0	42,8571
0	0	0	metabolicz	2	0	0	0	0
1	0	1	trzy i więce	2	60	8,57143	0	8,57143
0	1	0	dwa czynni	2	120	17,1429	0	17,1429
0	0	1	trzy i więce	1	60	8,57143	0	8,57143
1	0	1	trzy i więce	3	60	8,57143	0	8,57143
0	0	1	jeden czyn	2	105	15	0	15
0	0	1	trzy i więce	1	0	0	0	0
0	1	1	trzy i więce	3	0	0	0	0
0	1	1	trzy i więce	3	0	0	0	0
0	1	1	trzy i więce	3	1140	162,857	162,857	0
0	0	1	jeden czyn	2	200	28,5714	20	8,57143
0	0	1	jeden czyn	1	180	25,7143	0	25,7143
1	0	1	trzy i więce	2	0	0	0	0
0	0	1	trzy i więce	2	0	0	0	0

0	0	0	metabolicz	1	420	60	0	60
0	0	1	trzy i więc	3	20	2,85714	0	2,85714
1	1	1	trzy i więc	3	840	120	60	60
0	0	1	dwa czynni	2	60	8,57143	0	8,57143
1	1	1	trzy i więc	3	120	17,1429	0	17,1429
0	0	0	jeden czyn	3	30	4,28571	0	4,28571
1	0	0	dwa czynni	1	0	0	0	0
0	1	1	dwa czynni	2	150	21,4286	8,57143	12,8571
0	1	1	trzy i więc	3	0	0	0	0
0	0	1	dwa czynni	1	40	5,71429	0	5,71429
1	1	1	trzy i więc	2	60	8,57143	2,85714	5,71429
0	0	1	dwa czynni	2	0	0	0	0
0	0	0	jeden czyn	3	60	8,57143	0	8,57143
0	0	1	jeden czyn	1	0	0	0	0
0	1	1	trzy i więc	3	120	17,1429	0	17,1429
0	0	1	trzy i więc	3	360	51,4286	51,4286	0
0	1	1	trzy i więc	3	360	51,4286	0	51,4286
0	1	0	trzy i więc	3	420	60	0	60
0	0	1	dwa czynni	3	180	25,7143	25,7143	0
0	1	1	trzy i więc	2	0	0	0	0
0	1	0	jeden czyn	2	360	51,4286	40	11,4286
0	0	1	trzy i więc	3	0	0	0	0
1	0	1	trzy i więc	3	0	0	0	0
0	0	1	jeden czyn	1	360	51,4286	0	51,4286
0	1	1	trzy i więc	1	0	0	0	0
0	0	0	jeden czyn	2	0	0	0	0
0	0	1	dwa czynni	3	0	0	0	0
0	0	1	trzy i więc	1	0	0	0	0
0	0	1	trzy i więc	3	315	45	0	45
1	0	0	trzy i więc	3	420	60	20	40
0	1	1	trzy i więc	1	0	0	0	0
0	1	1	trzy i więc	2	60	8,57143	8,57143	0
0	1	0	jeden czyn	2	0	0	0	0
0	1	1	trzy i więc	3	120	17,1429	17,1429	0
0	0	1	jeden czyn	2	840	120	120	0
0	0	1	dwa czynni	2	420	60	8,57143	51,4286
1	1	1	trzy i więc	2	420	60	0	60
0	0	0	jeden czyn	3	0	0	0	0
0	0	0	jeden czyn	1	210	30	12,8571	17,1429
1	0	1	trzy i więc	2	280	40	0	40
1	0	1	trzy i więc	2	0	0	0	0
1	0	1	trzy i więc	1	180	25,7143	25,7143	0
1	1	1	trzy i więc	3	120	17,1429	17,1429	0
0	0	1	jeden czyn	1	60	8,57143	0	8,57143
0	1	1	trzy i więc	2	0	0	0	0
0	0	1	jeden czyn	1	120	17,1429	0	17,1429
0	0	1	jeden czyn	1	240	34,2857	0	34,2857
0	0	1	dwa czynni	3	0	0	0	0

0	1	1	trzy i więce	3	960	137,143	17,1429	120
0	0	1	jeden czyn	3	420	60	0	60
0	0	1	jeden czyn	3	0	0	0	0
0	0	0	metabolicz	3	180	25,7143	25,7143	0
0	0	1	dwa czynni	2	360	51,4286	25,7143	25,7143
0	1	1	trzy i więce	2	0	0	0	0
0	0	1	trzy i więce	1	60	8,57143	8,57143	0
1	0	1	trzy i więce	2	0	0	0	0
0	1	1	trzy i więce	3	420	60	60	0
0	0	1	dwa czynni	3	720	102,857	102,857	0
0	0	0	dwa czynni	2	0	0	0	0
0	0	1	jeden czyn	1	600	85,7143	51,4286	34,2857
0	0	0	jeden czyn	3	120	17,1429	0	17,1429
0	0	0	metabolicz	3	300	42,8571	42,8571	0
0	1	1	trzy i więce	3	420	60	0	60
1	0	1	trzy i więce	3	0	0	0	0
1	0	1	trzy i więce	3	0	0	0	0
0	0	1	trzy i więce	3	120	17,1429	17,1429	0
0	1	1	trzy i więce	3	0	0	0	0
0	0	0	jeden czyn	2	90	12,8571	8,57143	4,28571
0	0	1	jeden czyn	2	60	8,57143	0	8,57143
0	0	1	jeden czyn	2	0	0	0	0
0	1	1	trzy i więce	3	120	17,1429	0	17,1429
0	0	0	jeden czyn	2	0	0	0	0
0	1	1	trzy i więce	2	140	20	0	20
1	0	1	trzy i więce	3	60	8,57143	0	8,57143
1	0	1	trzy i więce	3	0	0	0	0
0	0	1	trzy i więce	1	0	0	0	0
0	0	1	jeden czyn	2	60	8,57143	0	8,57143
0	0	1	dwa czynni	2	90	12,8571	12,8571	0
0	1	1	trzy i więce	3	420	60	0	60
1	1	1	trzy i więce	2	0	0	0	0
1	0	0	trzy i więce	1	0	0	0	0
0	0	1	jeden czyn	2	0	0	0	0
0	1	0	dwa czynni	2	0	0	0	0
0	0	1	jeden czyn	2	420	60	60	0
0	0	1	dwa czynni	1	0	0	0	0
0	0	0	metabolicz	2	90	12,8571	12,8571	0
0	0	1	dwa czynni	3	0	0	0	0
0	1	0	dwa czynni	1	180	25,7143	0	25,7143
1	0	1	trzy i więce	2	0	0	0	0
0	0	1	jeden czyn	2	720	102,857	102,857	0
0	1	1	trzy i więce	3	0	0	0	0
0	0	1	dwa czynni	2	0	0	0	0
1	0	1	trzy i więce	3	175	25	0	25
0	0	1	dwa czynni	3	60	8,57143	8,57143	0
0	0	1	trzy i więce	3	0	0	0	0
0	0	1	dwa czynni	1	0	0	0	0

0	1	1	trzy i więce	3	60	8,57143	0	8,57143
0	0	1	jeden czyn	3	420	60	0	60
0	0	1	trzy i więce	1	120	17,1429	0	17,1429
0	0	1	dwa czynni	3	60	8,57143	0	8,57143
0	1	1	trzy i więce	3	0	0	0	0
0	1	1	trzy i więce	3	0	0	0	0
0	1	0	jeden czyn	2	0	0	0	0
0	0	0	jeden czyn	1	0	0	0	0
0	0	1	trzy i więce	3	120	17,1429	17,1429	0
0	1	1	dwa czynni	3	0	0	0	0
0	1	1	trzy i więce	1	120	17,1429	0	17,1429
0	1	0	trzy i więce	3	100	14,2857	14,2857	0
0	0	1	dwa czynni	2	1260	180	0	180
0	0	1	jeden czyn	3	300	42,8571	34,2857	8,57143
0	0	1	jeden czyn	2	180	25,7143	0	25,7143
0	1	1	trzy i więce	3	0	0	0	0
0	1	1	trzy i więce	1	30	4,28571	0	4,28571
0	0	1	dwa czynni	2	180	25,7143	0	25,7143
0	0	1	trzy i więce	1	220	31,4286	1,42857	30
0	1	1	trzy i więce	2	210	30	12,8571	17,1429
0	0	0	metabolicz	1	0	0	0	0
1	0	1	trzy i więce	2	420	60	0	60
0	0	0	metabolicz	2	180	25,7143	0	25,7143
0	1	1	trzy i więce	2	120	17,1429	0	17,1429
0	1	1	trzy i więce	1	0	0	0	0
0	1	1	trzy i więce	2	180	25,7143	25,7143	0
0	0	1	trzy i więce	1	0	0	0	0
0	0	1	dwa czynni	3	960	137,143	17,1429	120
1	0	1	trzy i więce	3	0	0	0	0
0	0	1	jeden czyn	2	30	4,28571	0	4,28571
0	0	0	dwa czynni	2	0	0	0	0
1	0	1	trzy i więce	3	210	30	0	30
0	0	0	metabolicz	3	0	0	0	0
1	0	1	dwa czynni	1	0	0	0	0
0	0	1	dwa czynni	3	1020	145,714	42,8571	102,857
0	0	1	dwa czynni	2	0	0	0	0
0	0	1	trzy i więce	2	240	34,2857	8,57143	25,7143
0	1	1	trzy i więce	2	60	8,57143	0	8,57143
0	1	0	dwa czynni	3	0	0	0	0
0	1	1	trzy i więce	1	0	0	0	0
0	0	0	metabolicz	2	840	120	102,857	17,1429
0	0	0	metabolicz	3	30	4,28571	4,28571	0
0	0	1	dwa czynni	2	0	0	0	0
0	0	1	dwa czynni	3	90	12,8571	0	12,8571
0	1	1	trzy i więce	3	0	0	0	0
0	0	0	metabolicz	3	0	0	0	0
1	0	1	trzy i więce	3	120	17,1429	0	17,1429
0	0	0	jeden czyn	3	180	25,7143	25,7143	0

0	0	0	metabolicz	1	180	25,7143	0	25,7143
0	1	1	trzy i więce	3	720	102,857	51,4286	51,4286
0	0	1	trzy i więce	3	0	0	0	0
0	0	1	dwa czynni	3	1260	180	60	120
0	0	0	jeden czyn	3	0	0	0	0
0	1	1	dwa czynni	3	0	0	0	0
0	0	0	metabolicz	2	165	23,5714	6,42857	17,1429
0	1	1	trzy i więce	2	600	85,7143	85,7143	0
0	0	1	dwa czynni	2	30	4,28571	4,28571	0
0	1	1	trzy i więce	2	90	12,8571	12,8571	0
0	1	1	trzy i więce	1	0	0	0	0
0	0	0	jeden czyn	2	280	40	0	40
0	1	1	dwa czynni	3	570	81,4286	51,4286	30
0	0	1	jeden czyn	2	0	0	0	0
0	1	0	dwa czynni	2	660	94,2857	34,2857	60
0	0	1	dwa czynni	3	0	0	0	0
0	1	1	trzy i więce	2	0	0	0	0
0	0	1	jeden czyn	1	0	0	0	0
0	1	0	dwa czynni	2	0	0	0	0
0	0	1	jeden czyn	3	120	17,1429	0	17,1429
0	0	1	dwa czynni	3	70	10	4,28571	5,71429
0	0	1	dwa czynni	2	0	0	0	0
1	0	0	dwa czynni	1	840	120	120	0
0	0	1	trzy i więce	2	0	0	0	0
0	0	1	trzy i więce	2	80	11,4286	0	11,4286
1	0	1	dwa czynni	3	30	4,28571	0	4,28571
0	0	0	jeden czyn	1	600	85,7143	34,2857	51,4286
0	1	1	trzy i więce	3	30	4,28571	0	4,28571
0	0	0	jeden czyn	2	0	0	0	0
0	1	0	dwa czynni	2	0	0	0	0
0	1	1	trzy i więce	2	0	0	0	0
0	0	1	dwa czynni	2	300	42,8571	0	42,8571
0	0	1	jeden czyn	2	60	8,57143	0	8,57143
0	0	1	jeden czyn	2	1080	154,286	34,2857	120
0	1	1	dwa czynni	1	0	0	0	0
0	1	1	trzy i więce	3	30	4,28571	0	4,28571
0	1	1	trzy i więce	1	60	8,57143	0	8,57143
1	1	1	trzy i więce	2	0	0	0	0
0	1	1	dwa czynni	2	300	42,8571	42,8571	0
0	0	0	metabolicz	3	150	21,4286	17,1429	4,28571
0	0	1	dwa czynni	2	0	0	0	0
0	0	0	metabolicz	2	90	12,8571	0	12,8571
0	0	1	jeden czyn	3	0	0	0	0
1	1	1	trzy i więce	3	60	8,57143	0	8,57143
1	0	0	jeden czyn	2	0	0	0	0
0	0	1	dwa czynni	1	0	0	0	0
0	0	1	dwa czynni	3	30	4,28571	0	4,28571
0	0	0	dwa czynni	1	0	0	0	0

0	1	1	trzy i więce	3	0	0	0	0
0	0	1	jeden czyn	2	300	42,8571	34,2857	8,57143
0	0	1	dwa czynni	2	180	25,7143	8,57143	17,1429
0	1	1	trzy i więce	1	200	28,5714	2,85714	25,7143
0	0	0	jeden czyn	3	60	8,57143	8,57143	0
0	1	0	trzy i więce	2	60	8,57143	8,57143	0
0	0	1	dwa czynni	1	105	15	0	15
0	0	1	dwa czynni	1	0	0	0	0
0	1	1	trzy i więce	1	0	0	0	0
1	1	1	trzy i więce	1	0	0	0	0
0	1	1	trzy i więce	3	0	0	0	0
0	0	1	jeden czyn	1	0	0	0	0
0	1	1	trzy i więce	1	0	0	0	0
0	1	1	trzy i więce	3	0	0	0	0
0	0	0	metabolicz	2	60	8,57143	0	8,57143
0	1	1	trzy i więce	1	0	0	0	0
1	0	0	jeden czyn	3	180	25,7143	8,57143	17,1429
0	0	1	dwa czynni	1	350	50	0	50
1	0	1	trzy i więce	3	0	0	0	0
0	0	1	dwa czynni	1	120	17,1429	0	17,1429
0	1	1	trzy i więce	1	210	30	0	30
1	1	0	trzy i więce	3	60	8,57143	0	8,57143
0	1	1	dwa czynni	2	0	0	0	0
0	0	1	dwa czynni	3	600	85,7143	77,1429	8,57143
0	0	0	dwa czynni	1	0	0	0	0
0	1	1	trzy i więce	2	40	5,71429	5,71429	0
0	1	1	trzy i więce	1	60	8,57143	0	8,57143
0	0	1	jeden czyn	2	660	94,2857	51,4286	42,8571
1	0	1	trzy i więce	3	60	8,57143	0	8,57143
0	0	0	metabolicz	2	495	70,7143	53,5714	17,1429
0	0	1	trzy i więce	1	160	22,8571	5,71429	17,1429
0	0	0	dwa czynni	3	90	12,8571	0	12,8571
0	0	1	dwa czynni	3	180	25,7143	0	25,7143
0	0	1	jeden czyn	3	90	12,8571	12,8571	0
0	1	0	jeden czyn	3	120	17,1429	0	17,1429
0	0	0	metabolicz	2	320	45,7143	31,4286	14,2857
0	0	1	dwa czynni	2	60	8,57143	0	8,57143
0	0	1	dwa czynni	2	0	0	0	0
0	0	1	dwa czynni	3	60	8,57143	0	8,57143
0	1	1	trzy i więce	3	0	0	0	0
0	0	1	dwa czynni	2	420	60	60	0
0	0	1	jeden czyn	3	0	0	0	0
0	0	1	jeden czyn	2	0	0	0	0
1	1	1	trzy i więce	3	1260	180	0	180
0	0	1	trzy i więce	1	120	17,1429	0	17,1429
1	1	0	trzy i więce	2	540	77,1429	0	77,1429
0	1	1	trzy i więce	2	660	94,2857	34,2857	60
0	0	0	jeden czyn	1	0	0	0	0

0	1	1	dwa czynni	3	0	0	0	0
0	0	1	dwa czynni	3	0	0	0	0
0	0	1	dwa czynni	2	125	17,8571	0	17,8571
0	1	1	trzy i więce	1	240	34,2857	0	34,2857
1	0	1	trzy i więce	3	240	34,2857	8,57143	25,7143
0	1	1	trzy i więce	2	0	0	0	0
0	1	0	dwa czynni	2	0	0	0	0
0	0	1	dwa czynni	2	240	34,2857	25,7143	8,57143
0	0	1	jeden czyn	2	120	17,1429	0	17,1429
0	0	1	trzy i więce	3	600	85,7143	85,7143	0
1	0	1	trzy i więce	2	0	0	0	0
1	0	1	trzy i więce	2	240	34,2857	34,2857	0
0	1	1	trzy i więce	3	270	38,5714	12,8571	25,7143
0	0	1	dwa czynni	1	210	30	0	30
0	0	0	dwa czynni	2	180	25,7143	12,8571	12,8571
0	0	0	metabolicz	2	60	8,57143	0	8,57143
0	0	1	dwa czynni	1	350	50	0	50
0	0	1	dwa czynni	2	0	0	0	0
0	0	0	jeden czyn	2	135	19,2857	19,2857	0
0	1	1	trzy i więce	2	0	0	0	0
0	1	1	trzy i więce	3	210	30	21,4286	8,57143
0	1	0	jeden czyn	1	120	17,1429	0	17,1429
1	0	1	trzy i więce	1	0	0	0	0
0	0	1	dwa czynni	1	0	0	0	0
1	0	0	trzy i więce	3	60	8,57143	0	8,57143
0	0	1	dwa czynni	2	470	67,1429	17,1429	50
0	0	0	dwa czynni	3	225	32,1429	17,1429	15
0	1	1	dwa czynni	2	300	42,8571	0	42,8571
1	0	0	trzy i więce	3	0	0	0	0
0	1	0	jeden czyn	3	180	25,7143	8,57143	17,1429
0	0	1	jeden czyn	3	180	25,7143	17,1429	8,57143
0	0	0	jeden czyn	3	285	40,7143	34,2857	6,42857
0	1	1	trzy i więce	3	160	22,8571	14,2857	8,57143
0	0	1	dwa czynni	2	0	0	0	0
0	0	1	dwa czynni	2	190	27,1429	10	17,1429
0	1	1	dwa czynni	3	90	12,8571	12,8571	0
0	0	0	jeden czyn	3	0	0	0	0
0	1	0	jeden czyn	1	0	0	0	0
0	1	1	trzy i więce	3	0	0	0	0
0	1	1	trzy i więce	2	0	0	0	0
0	0	0	metabolicz	1	240	34,2857	0	34,2857
1	0	1	dwa czynni	2	0	0	0	0
0	0	0	metabolicz	1	0	0	0	0
0	1	1	trzy i więce	2	120	17,1429	8,57143	8,57143
0	0	1	jeden czyn	3	120	17,1429	17,1429	0
0	0	1	jeden czyn	3	480	68,5714	68,5714	0
1	1	1	trzy i więce	3	60	8,57143	0	8,57143
0	1	1	dwa czynni	3	60	8,57143	0	8,57143

0	0	1	dwa czynni	2	120	17,1429	17,1429	0
0	1	1	dwa czynni	2	60	8,57143	8,57143	0
0	0	1	dwa czynni	3	0	0	0	0
1	1	1	trzy i więce	1	0	0	0	0
0	1	1	dwa czynni	2	160	22,8571	5,71429	17,1429
0	1	1	trzy i więce	3	30	4,28571	0	4,28571
0	0	1	dwa czynni	2	180	25,7143	25,7143	0
0	0	1	dwa czynni	2	90	12,8571	0	12,8571
0	1	1	trzy i więce	3	60	8,57143	4,28571	4,28571
0	0	0	metabolicz	3	0	0	0	0
0	0	1	jeden czyn	1	240	34,2857	0	34,2857
0	0	1	dwa czynni	2	300	42,8571	42,8571	0
0	0	0	metabolicz	1	0	0	0	0
0	1	0	trzy i więce	1	210	30	0	30
1	1	1	trzy i więce	2	60	8,57143	0	8,57143
1	0	1	trzy i więce	2	1020	145,714	25,7143	120
1	0	1	trzy i więce	1	0	0	0	0
0	0	1	dwa czynni	3	0	0	0	0
0	0	1	dwa czynni	3	80	11,4286	0	11,4286
0	0	1	dwa czynni	1	0	0	0	0
0	0	0	dwa czynni	1	60	8,57143	8,57143	0
0	1	0	jeden czyn	2	0	0	0	0
1	0	1	trzy i więce	3	0	0	0	0
0	1	0	jeden czyn	2	30	4,28571	0	4,28571
0	1	1	trzy i więce	3	0	0	0	0
0	0	1	trzy i więce	3	0	0	0	0
0	0	1	jeden czyn	2	210	30	30	0
1	0	1	trzy i więce	2	60	8,57143	0	8,57143
0	0	0	metabolicz	1	480	68,5714	68,5714	0
0	0	1	dwa czynni	3	0	0	0	0
0	0	1	jeden czyn	2	0	0	0	0
0	0	0	metabolicz	1	0	0	0	0
0	0	0	metabolicz	1	210	30	0	30
1	1	0	trzy i więce	2	180	25,7143	25,7143	0
0	0	0	jeden czyn	2	60	8,57143	8,57143	0
0	1	1	trzy i więce	3	180	25,7143	8,57143	17,1429
1	1	1	trzy i więce	1	0	0	0	0
1	0	1	dwa czynni	1	0	0	0	0
0	0	1	dwa czynni	3	0	0	0	0
0	0	1	dwa czynni	2	0	0	0	0
0	1	1	trzy i więce	1	360	51,4286	51,4286	0
0	1	1	dwa czynni	2	780	111,429	51,4286	60
0	1	1	trzy i więce	2	0	0	0	0
0	0	1	jeden czyn	2	60	8,57143	0	8,57143
0	0	1	jeden czyn	2	120	17,1429	0	17,1429
0	1	1	trzy i więce	3	285	40,7143	15	25,7143
0	0	1	trzy i więce	3	0	0	0	0
0	1	1	trzy i więce	2	840	120	120	0

0	0	1	dwa czynni	1	0	0	0	0
0	0	0	dwa czynni	3	0	0	0	0
0	0	1	jeden czyn	2	150	21,4286	0	21,4286
0	1	1	trzy i więc	1	0	0	0	0
0	0	1	jeden czyn	2	540	77,1429	42,8571	34,2857
1	0	1	trzy i więc	2	0	0	0	0
0	0	1	trzy i więc	2	0	0	0	0
1	1	0	trzy i więc	3	0	0	0	0
1	1	1	trzy i więc	2	0	0	0	0
1	0	1	trzy i więc	2	180	25,7143	25,7143	0
0	0	1	jeden czyn	3	0	0	0	0
0	0	1	jeden czyn	2	840	120	60	60
1	0	1	trzy i więc	2	0	0	0	0
0	0	1	jeden czyn	1	0	0	0	0
0	1	1	trzy i więc	3	0	0	0	0
0	1	1	trzy i więc	2	420	60	0	60
0	0	1	dwa czynni	1	0	0	0	0
0	1	1	trzy i więc	3	20	2,85714	0	2,85714
0	0	1	jeden czyn	2	180	25,7143	8,57143	17,1429
0	0	1	dwa czynni	1	60	8,57143	0	8,57143
0	1	1	trzy i więc	2	140	20	0	20
0	0	1	dwa czynni	3	0	0	0	0
0	0	0	metabolicz	2	90	12,8571	0	12,8571
0	0	1	trzy i więc	3	90	12,8571	0	12,8571
0	1	1	trzy i więc	3	0	0	0	0
0	0	1	jeden czyn	2	0	0	0	0
0	0	0	jeden czyn	1	60	8,57143	0	8,57143
0	1	1	trzy i więc	2	180	25,7143	0	25,7143
0	1	1	trzy i więc	3	50	7,14286	0	7,14286
0	1	0	dwa czynni	2	0	0	0	0
0	1	0	jeden czyn	2	60	8,57143	0	8,57143
0	0	0	metabolicz	3	0	0	0	0
1	0	1	trzy i więc	1	0	0	0	0
0	0	1	jeden czyn	2	0	0	0	0
0	1	1	trzy i więc	1	120	17,1429	0	17,1429
0	0	1	dwa czynni	1	420	60	0	60
0	0	1	trzy i więc	1	180	25,7143	8,57143	17,1429
0	1	1	trzy i więc	1	210	30	17,1429	12,8571
0	0	0	jeden czyn	1	120	17,1429	0	17,1429
0	0	1	jeden czyn	2	60	8,57143	8,57143	0
0	1	1	dwa czynni	2	60	8,57143	0	8,57143
1	0	0	dwa czynni	2	280	40	20	20
0	0	0	dwa czynni	1	180	25,7143	0	25,7143
1	1	1	trzy i więc	2	120	17,1429	0	17,1429
1	0	1	trzy i więc	3	60	8,57143	8,57143	0
0	1	1	trzy i więc	2	0	0	0	0
0	1	1	trzy i więc	3	130	18,5714	10	8,57143
0	0	1	dwa czynni	3	60	8,57143	0	8,57143

0	0	1	trzy i więce	1	0	0	0	0
0	0	1	dwa czynni	2	140	20	0	20
0	0	0	metabolicz	1	0	0	0	0
0	1	0	dwa czynni	2	0	0	0	0
0	0	1	dwa czynni	1	0	0	0	0
0	1	1	trzy i więce	2	60	8,57143	8,57143	0
1	1	1	trzy i więce	2	40	5,71429	5,71429	0
0	0	1	trzy i więce	1	0	0	0	0
0	0	1	dwa czynni	1	180	25,7143	25,7143	0
0	0	1	dwa czynni	2	0	0	0	0
0	1	1	trzy i więce	2	180	25,7143	17,1429	8,57143
0	0	1	dwa czynni	2	240	34,2857	0	34,2857
0	0	0	metabolicz	1	60	8,57143	0	8,57143
1	1	1	trzy i więce	2	0	0	0	0
0	0	1	dwa czynni	1	240	34,2857	34,2857	0
1	0	0	trzy i więce	1	0	0	0	0
0	0	1	dwa czynni	2	150	21,4286	12,8571	8,57143
0	0	0	dwa czynni	1	0	0	0	0
0	0	1	dwa czynni	1	0	0	0	0
1	0	1	trzy i więce	1	0	0	0	0
0	0	1	dwa czynni	1	0	0	0	0
0	1	1	trzy i więce	3	0	0	0	0
0	0	1	dwa czynni	2	0	0	0	0
0	0	0	jeden czyn	1	240	34,2857	4,28571	30
0	0	1	dwa czynni	2	270	38,5714	34,2857	4,28571
0	0	0	metabolicz	2	0	0	0	0
0	1	1	trzy i więce	1	300	42,8571	42,8571	0
0	0	1	trzy i więce	3	0	0	0	0
0	1	1	trzy i więce	2	315	45	0	45
0	0	1	jeden czyn	1	0	0	0	0
1	0	1	trzy i więce	2	240	34,2857	0	34,2857
0	0	1	dwa czynni	3	120	17,1429	0	17,1429
1	1	1	trzy i więce	1	120	17,1429	0	17,1429
0	0	1	jeden czyn	1	60	8,57143	0	8,57143
0	0	0	metabolicz	1	0	0	0	0
0	0	1	jeden czyn	3	0	0	0	0
0	1	1	trzy i więce	1	420	60	0	60
0	1	0	jeden czyn	1	105	15	15	0
0	0	1	dwa czynni	1	0	0	0	0
0	0	1	dwa czynni	3	600	85,7143	51,4286	34,2857
1	1	1	trzy i więce	1	105	15	15	0
0	0	0	jeden czyn	1	0	0	0	0
0	1	1	trzy i więce	2	0	0	0	0
0	1	1	trzy i więce	2	0	0	0	0
1	0	1	trzy i więce	3	0	0	0	0
0	1	0	dwa czynni	2	210	30	4,28571	25,7143
0	0	1	jeden czyn	3	45	6,42857	6,42857	0
0	1	1	trzy i więce	3	420	60	34,2857	25,7143

0	1	1	trzy i więce	2	90	12,8571	12,8571	0
0	0	1	dwa czynni	1	0	0	0	0
0	0	1	dwa czynni	1	120	17,1429	0	17,1429
0	0	1	jeden czyn	2	250	35,7143	35,7143	0
1	0	1	trzy i więce	1	240	34,2857	0	34,2857
0	0	0	metabolicz	2	180	25,7143	0	25,7143
1	1	1	trzy i więce	1	360	51,4286	47,1429	4,28571
0	0	0	metabolicz	2	120	17,1429	0	17,1429
1	0	1	trzy i więce	2	120	17,1429	0	17,1429
1	0	0	trzy i więce	3	0	0	0	0
0	0	1	dwa czynni	2	80	11,4286	0	11,4286
0	0	0	metabolicz	1	30	4,28571	0	4,28571
0	0	1	dwa czynni	2	0	0	0	0
0	0	0	jeden czyn	3	840	120	0	120
0	0	1	dwa czynni	2	0	0	0	0
0	1	1	trzy i więce	2	120	17,1429	0	17,1429
0	0	1	dwa czynni	2	60	8,57143	0	8,57143
0	0	1	dwa czynni	1	180	25,7143	0	25,7143
0	0	1	dwa czynni	3	400	57,1429	51,4286	5,71429
0	1	1	trzy i więce	3	0	0	0	0
0	1	0	jeden czyn	1	30	4,28571	0	4,28571
1	0	1	trzy i więce	1	0	0	0	0
0	0	0	jeden czyn	1	0	0	0	0
0	0	1	trzy i więce	3	0	0	0	0
0	0	1	dwa czynni	2	400	57,1429	22,8571	34,2857
0	0	0	metabolicz	3	240	34,2857	17,1429	17,1429
0	1	1	trzy i więce	2	120	17,1429	0	17,1429
0	1	1	dwa czynni	3	180	25,7143	8,57143	17,1429
0	1	1	trzy i więce	3	240	34,2857	0	34,2857
0	0	1	trzy i więce	3	0	0	0	0
0	0	0	metabolicz	3	330	47,1429	38,5714	8,57143
0	0	1	dwa czynni	3	0	0	0	0
0	0	0	dwa czynni	2	0	0	0	0
1	1	1	trzy i więce	1	240	34,2857	30	4,28571
0	0	0	jeden czyn	1	0	0	0	0
1	0	1	trzy i więce	2	0	0	0	0
0	1	1	trzy i więce	2	180	25,7143	0	25,7143
0	0	1	jeden czyn	2	0	0	0	0
0	1	1	trzy i więce	1	0	0	0	0
1	1	1	trzy i więce	3	90	12,8571	12,8571	0
0	0	0	jeden czyn	2	180	25,7143	17,1429	8,57143
0	0	0	metabolicz	3	100	14,2857	14,2857	0
0	0	1	trzy i więce	3	0	0	0	0
0	0	1	dwa czynni	3	15	2,14286	0	2,14286
0	0	0	jeden czyn	1	180	25,7143	0	25,7143
0	0	0	metabolicz	1	150	21,4286	4,28571	17,1429
0	0	1	dwa czynni	2	0	0	0	0
0	1	1	trzy i więce	3	240	34,2857	17,1429	17,1429

0	0	0	jeden czyn	3	0	0	0	0
0	0	1	jeden czyn	1	480	68,5714	0	68,5714
0	1	0	dwa czynni	3	180	25,7143	8,57143	17,1429
0	1	1	trzy i więc	3	90	12,8571	0	12,8571
0	0	1	dwa czynni	2	180	25,7143	8,57143	17,1429
1	0	0	trzy i więc	3	120	17,1429	17,1429	0
0	0	0	metabolicz	2	0	0	0	0
0	0	1	jeden czyn	2	80	11,4286	11,4286	0
0	1	0	dwa czynni	2	30	4,28571	0	4,28571
0	1	1	trzy i więc	1	10	1,42857	0	1,42857
0	1	0	dwa czynni	2	240	34,2857	17,1429	17,1429
0	0	0	metabolicz	3	30	4,28571	4,28571	0
0	0	1	jeden czyn	1	0	0	0	0
0	0	0	metabolicz	2	340	48,5714	10	38,5714
0	1	1	dwa czynni	3	280	40	0	40
0	0	1	jeden czyn	3	0	0	0	0
0	0	1	trzy i więc	2	520	74,2857	14,2857	60
0	0	0	metabolicz	2	690	98,5714	68,5714	30
0	1	1	trzy i więc	1	180	25,7143	25,7143	0
0	1	1	trzy i więc	3	90	12,8571	0	12,8571
1	0	1	trzy i więc	1	900	128,571	0	128,571
1	1	1	trzy i więc	3	0	0	0	0
0	0	1	dwa czynni	3	180	25,7143	0	25,7143
0	0	1	dwa czynni	2	90	12,8571	0	12,8571
1	0	1	trzy i więc	2	0	0	0	0
1	1	1	trzy i więc	3	0	0	0	0
0	1	1	dwa czynni	3	600	85,7143	68,5714	17,1429
0	0	1	jeden czyn	2	0	0	0	0
0	0	1	jeden czyn	3	0	0	0	0
0	1	1	trzy i więc	2	0	0	0	0
0	0	0	dwa czynni	3	240	34,2857	0	34,2857
0	0	1	jeden czyn	1	0	0	0	0
1	1	1	trzy i więc	3	0	0	0	0
0	1	1	trzy i więc	3	0	0	0	0
0	0	0	dwa czynni	2	0	0	0	0
0	0	1	dwa czynni	3	120	17,1429	0	17,1429
0	0	1	dwa czynni	2	120	17,1429	17,1429	0
0	0	1	trzy i więc	2	90	12,8571	0	12,8571
1	0	1	trzy i więc	3	360	51,4286	34,2857	17,1429
1	1	1	trzy i więc	2	0	0	0	0
1	1	1	trzy i więc	1	240	34,2857	0	34,2857
1	0	1	trzy i więc	3	240	34,2857	34,2857	0
0	0	0	metabolicz	3	420	60	0	60
1	0	0	trzy i więc	3	540	77,1429	51,4286	25,7143
0	1	1	trzy i więc	3	0	0	0	0
1	0	1	trzy i więc	2	435	62,1429	62,1429	0
0	1	1	dwa czynni	2	810	115,714	115,714	0
0	0	1	jeden czyn	3	240	34,2857	17,1429	17,1429

0	0	1	jeden czyn	2	60	8,57143	0	8,57143
0	0	1	dwa czynni	2	0	0	0	0
0	1	1	trzy i więce	1	0	0	0	0
0	0	0	jeden czyn	3	480	68,5714	8,57143	60
0	1	1	trzy i więce	3	0	0	0	0
1	0	1	trzy i więce	2	360	51,4286	51,4286	0
0	1	1	trzy i więce	2	0	0	0	0
0	0	1	dwa czynni	1	0	0	0	0
0	0	1	dwa czynni	2	0	0	0	0
0	0	1	jeden czyn	3	300	42,8571	17,1429	25,7143
0	0	1	dwa czynni	2	720	102,857	102,857	0
0	0	0	metabolicz	3	280	40	40	0
1	0	1	trzy i więce	1	165	23,5714	15	8,57143
0	0	1	jeden czyn	1	180	25,7143	0	25,7143
0	1	0	jeden czyn	2	0	0	0	0
0	0	0	dwa czynni	2	120	17,1429	17,1429	0
0	1	0	dwa czynni	2	200	28,5714	2,85714	25,7143
0	0	1	trzy i więce	2	120	17,1429	17,1429	0
0	0	1	jeden czyn	2	360	51,4286	51,4286	0
0	0	0	metabolicz	3	420	60	0	60
0	0	0	dwa czynni	2	90	12,8571	0	12,8571
0	0	1	dwa czynni	3	40	5,71429	0	5,71429
0	0	1	trzy i więce	3	360	51,4286	51,4286	0
0	0	1	dwa czynni	3	120	17,1429	0	17,1429
0	0	0	metabolicz	1	0	0	0	0
0	0	1	jeden czyn	3	180	25,7143	25,7143	0
0	0	1	trzy i więce	3	0	0	0	0
0	0	1	trzy i więce	2	0	0	0	0
0	1	1	trzy i więce	3	0	0	0	0
1	1	1	trzy i więce	1	0	0	0	0
0	0	1	dwa czynni	3	60	8,57143	8,57143	0
1	0	1	trzy i więce	2	45	6,42857	6,42857	0
0	0	0	metabolicz	2	360	51,4286	0	51,4286
1	1	1	trzy i więce	1	450	64,2857	51,4286	12,8571
0	0	0	jeden czyn	3	120	17,1429	17,1429	0
0	0	0	metabolicz	1	60	8,57143	0	8,57143
0	0	0	metabolicz	3	240	34,2857	34,2857	0
0	0	1	jeden czyn	2	540	77,1429	77,1429	0
1	0	1	dwa czynni	2	100	14,2857	10	4,28571
0	1	1	trzy i więce	3	45	6,42857	0	6,42857
0	0	1	trzy i więce	3	75	10,7143	0	10,7143
0	0	1	dwa czynni	3	0	0	0	0
0	1	1	trzy i więce	3	0	0	0	0
0	1	1	trzy i więce	1	120	17,1429	8,57143	8,57143
0	1	1	trzy i więce	2	270	38,5714	30	8,57143
0	0	1	jeden czyn	2	0	0	0	0
1	0	1	trzy i więce	3	0	0	0	0
0	0	1	jeden czyn	2	2520	360	180	180

0	0	0	dwa czynni	3	135	19,2857	17,1429	2,14286
0	1	1	trzy i więc	2	0	0	0	0
0	1	0	jeden czyn	1	0	0	0	0
0	0	1	jeden czyn	1	0	0	0	0
0	0	0	metabolicz	2	420	60	20	40
1	1	1	trzy i więc	1	420	60	0	60
0	0	0	metabolicz	2	15	2,14286	0	2,14286
0	1	1	dwa czynni	3	0	0	0	0
0	0	1	trzy i więc	1	0	0	0	0
0	0	1	dwa czynni	1	0	0	0	0
1	0	1	trzy i więc	1	0	0	0	0
0	0	0	metabolicz	3	0	0	0	0
0	1	1	trzy i więc	3	0	0	0	0
0	0	1	dwa czynni	2	0	0	0	0
0	0	1	dwa czynni	1	120	17,1429	0	17,1429
0	1	0	dwa czynni	1	75	10,7143	8,57143	2,14286
0	0	1	dwa czynni	2	210	30	0	30
0	0	1	jeden czyn	2	0	0	0	0
0	0	1	jeden czyn	2	1020	145,714	120	25,7143
0	0	1	jeden czyn	3	120	17,1429	0	17,1429
0	0	1	trzy i więc	1	0	0	0	0
0	0	1	jeden czyn	2	240	34,2857	17,1429	17,1429
1	1	1	trzy i więc	2	0	0	0	0
0	0	0	metabolicz	3	0	0	0	0
1	1	1	trzy i więc	3	120	17,1429	8,57143	8,57143
0	0	1	dwa czynni	3	120	17,1429	0	17,1429
0	0	1	dwa czynni	1	0	0	0	0
0	1	1	dwa czynni	1	120	17,1429	0	17,1429
0	0	1	dwa czynni	2	0	0	0	0
0	1	1	dwa czynni	3	360	51,4286	0	51,4286
0	1	0	trzy i więc	2	0	0	0	0
0	1	1	trzy i więc	2	90	12,8571	12,8571	0
0	0	0	metabolicz	1	40	5,71429	0	5,71429
0	0	1	trzy i więc	2	0	0	0	0
0	0	0	dwa czynni	3	0	0	0	0
0	0	1	dwa czynni	2	360	51,4286	0	51,4286
0	0	1	jeden czyn	2	60	8,57143	0	8,57143
1	1	1	trzy i więc	3	180	25,7143	8,57143	17,1429
0	1	1	trzy i więc	1	420	60	0	60
0	0	1	dwa czynni	2	120	17,1429	17,1429	0
0	1	1	trzy i więc	3	240	34,2857	0	34,2857
1	1	0	trzy i więc	3	60	8,57143	0	8,57143
0	1	1	trzy i więc	2	0	0	0	0
1	1	1	trzy i więc	2	120	17,1429	0	17,1429
0	0	1	trzy i więc	1	0	0	0	0
0	0	1	trzy i więc	1	150	21,4286	4,28571	17,1429
0	0	1	dwa czynni	3	280	40	0	40
0	1	0	dwa czynni	3	0	0	0	0

0	0	0	metabolicz	2	0	0	0	0
0	0	1	trzy i więce	3	0	0	0	0
0	0	0	metabolicz	3	120	17,1429	0	17,1429
0	0	1	trzy i więce	2	600	85,7143	85,7143	0
1	0	0	trzy i więce	1	30	4,28571	0	4,28571
0	1	0	jeden czyn	3	420	60	0	60
0	0	1	trzy i więce	2	420	60	60	0
0	0	1	dwa czynni	1	0	0	0	0
0	0	1	dwa czynni	3	180	25,7143	0	25,7143
0	0	0	metabolicz	1	1260	180	0	180
0	1	1	trzy i więce	3	60	8,57143	0	8,57143
0	0	1	jeden czyn	2	0	0	0	0
1	0	1	trzy i więce	2	0	0	0	0
0	0	0	metabolicz	2	210	30	0	30
0	1	1	dwa czynni	1	360	51,4286	0	51,4286
0	0	1	jeden czyn	1	30	4,28571	4,28571	0
1	0	1	trzy i więce	3	420	60	17,1429	42,8571
0	0	1	dwa czynni	2	105	15	0	15
0	0	0	metabolicz	3	0	0	0	0
1	0	0	trzy i więce	1	0	0	0	0
0	1	1	trzy i więce	3	840	120	0	120
1	0	1	trzy i więce	1	360	51,4286	21,4286	30
0	1	1	trzy i więce	3	0	0	0	0
0	0	0	metabolicz	2	660	94,2857	51,4286	42,8571
0	0	1	jeden czyn	1	600	85,7143	0	85,7143
0	1	1	trzy i więce	3	0	0	0	0
0	0	0	metabolicz	1	60	8,57143	0	8,57143
1	0	1	trzy i więce	2	0	0	0	0
0	0	1	jeden czyn	2	360	51,4286	0	51,4286
0	1	0	jeden czyn	2	75	10,7143	10,7143	0
0	1	1	trzy i więce	3	0	0	0	0
1	1	0	dwa czynni	3	0	0	0	0
0	1	1	trzy i więce	2	30	4,28571	0	4,28571
0	0	0	metabolicz	2	0	0	0	0
0	1	1	dwa czynni	1	0	0	0	0
0	1	1	dwa czynni	2	0	0	0	0
0	0	1	trzy i więce	1	0	0	0	0
0	1	1	trzy i więce	2	160	22,8571	22,8571	0
0	0	1	dwa czynni	3	240	34,2857	34,2857	0
0	0	0	metabolicz	3	0	0	0	0
0	1	1	trzy i więce	2	60	8,57143	0	8,57143
0	1	0	jeden czyn	1	0	0	0	0
0	0	1	dwa czynni	3	240	34,2857	30	4,28571
1	0	1	trzy i więce	2	0	0	0	0
0	0	1	jeden czyn	1	0	0	0	0
0	0	1	jeden czyn	2	180	25,7143	0	25,7143
0	0	1	dwa czynni	2	180	25,7143	17,1429	8,57143
0	0	0	jeden czyn	1	840	120	0	120

0	0	0	metabolicz	3	90	12,8571	12,8571	0
0	0	1	jeden czyn	2	70	10	10	0
1	0	1	trzy i więce	3	105	15	15	0
0	0	0	jeden czyn	3	120	17,1429	0	17,1429
0	0	1	jeden czyn	3	0	0	0	0
0	0	1	jeden czyn	3	120	17,1429	8,57143	8,57143
0	0	0	metabolicz	2	120	17,1429	17,1429	0
1	0	1	trzy i więce	2	1260	180	102,857	77,1429
0	0	1	dwa czynni	2	180	25,7143	0	25,7143
0	0	1	jeden czyn	1	840	120	120	0
0	0	1	dwa czynni	3	180	25,7143	0	25,7143
0	0	1	dwa czynni	3	130	18,5714	10	8,57143
0	1	0	dwa czynni	1	0	0	0	0
0	0	1	jeden czyn	1	300	42,8571	0	42,8571
1	1	1	trzy i więce	1	120	17,1429	17,1429	0
1	1	1	trzy i więce	1	0	0	0	0
0	0	1	jeden czyn	1	105	15	0	15
0	0	1	jeden czyn	1	60	8,57143	0	8,57143
0	0	1	trzy i więce	2	540	77,1429	17,1429	60
0	0	1	dwa czynni	2	0	0	0	0
1	0	1	trzy i więce	3	220	31,4286	8,57143	22,8571
0	0	0	metabolicz	1	370	52,8571	51,4286	1,42857
1	0	1	trzy i więce	1	120	17,1429	0	17,1429
0	1	1	dwa czynni	2	2030	290	240	50
0	0	1	dwa czynni	1	240	34,2857	17,1429	17,1429
0	0	1	jeden czyn	1	0	0	0	0
0	1	0	trzy i więce	3	0	0	0	0
0	0	1	dwa czynni	2	1440	205,714	25,7143	180
0	1	1	trzy i więce	2	210	30	0	30
0	0	1	dwa czynni	2	0	0	0	0
0	0	1	dwa czynni	1	0	0	0	0
1	1	0	trzy i więce	1	120	17,1429	0	17,1429
0	0	1	dwa czynni	2	0	0	0	0
0	1	0	trzy i więce	1	180	25,7143	0	25,7143
0	0	1	dwa czynni	2	150	21,4286	4,28571	17,1429
0	0	1	trzy i więce	3	60	8,57143	0	8,57143
0	1	0	jeden czyn	1	0	0	0	0
0	0	1	dwa czynni	3	40	5,71429	0	5,71429
0	0	1	trzy i więce	3	120	17,1429	0	17,1429
0	1	1	trzy i więce	3	60	8,57143	0	8,57143
0	0	1	dwa czynni	2	240	34,2857	34,2857	0
0	0	1	jeden czyn	2	180	25,7143	8,57143	17,1429
0	1	1	trzy i więce	3	0	0	0	0
0	0	1	trzy i więce	1	0	0	0	0
0	1	1	trzy i więce	1	150	21,4286	0	21,4286
0	1	1	trzy i więce	3	210	30	0	30
0	0	1	dwa czynni	2	150	21,4286	21,4286	0
0	1	1	trzy i więce	1	0	0	0	0

0	1	1	trzy i więce	3	0	0	0	0
0	0	0	jeden czyn	2	490	70	40	30
0	1	1	trzy i więce	1	330	47,1429	30	17,1429
0	1	1	dwa czynni	2	90	12,8571	12,8571	0
0	1	1	trzy i więce	2	0	0	0	0
1	0	1	trzy i więce	2	30	4,28571	0	4,28571
0	0	0	metabolicz	2	0	0	0	0
0	1	1	trzy i więce	3	0	0	0	0
0	0	1	dwa czynni	3	0	0	0	0
0	1	1	trzy i więce	1	0	0	0	0
0	0	0	metabolicz	2	600	85,7143	34,2857	51,4286
0	0	0	jeden czyn	1	210	30	0	30
1	1	1	trzy i więce	1	230	32,8571	20	12,8571
0	0	1	jeden czyn	2	120	17,1429	4,28571	12,8571
0	0	0	jeden czyn	1	0	0	0	0
0	1	1	trzy i więce	3	240	34,2857	34,2857	0
0	0	1	trzy i więce	2	420	60	0	60
0	0	1	dwa czynni	2	0	0	0	0
0	1	1	trzy i więce	3	60	8,57143	8,57143	0
0	0	1	dwa czynni	2	0	0	0	0
0	0	1	jeden czyn	3	60	8,57143	8,57143	0
0	1	1	dwa czynni	2	855	122,143	77,1429	45
0	0	0	metabolicz	2	320	45,7143	20	25,7143
0	0	1	dwa czynni	3	0	0	0	0
0	1	0	jeden czyn	2	0	0	0	0
0	1	1	trzy i więce	2	0	0	0	0
0	0	1	trzy i więce	3	60	8,57143	0	8,57143
1	1	1	trzy i więce	2	60	8,57143	0	8,57143
0	1	1	trzy i więce	3	0	0	0	0
0	0	1	jeden czyn	1	0	0	0	0
0	0	1	dwa czynni	2	60	8,57143	0	8,57143
0	1	0	jeden czyn	2	300	42,8571	34,2857	8,57143
0	0	1	trzy i więce	1	420	60	0	60
0	0	0	jeden czyn	2	0	0	0	0
0	0	1	jeden czyn	2	0	0	0	0
0	0	1	dwa czynni	3	60	8,57143	0	8,57143
0	0	1	jeden czyn	1	0	0	0	0
0	1	0	dwa czynni	2	40	5,71429	0	5,71429
0	1	1	dwa czynni	1	30	4,28571	0	4,28571
0	1	0	jeden czyn	1	300	42,8571	34,2857	8,57143
0	0	0	jeden czyn	2	120	17,1429	0	17,1429
0	0	1	jeden czyn	1	60	8,57143	0	8,57143
0	0	1	trzy i więce	3	90	12,8571	0	12,8571
0	1	1	dwa czynni	2	0	0	0	0
0	0	1	dwa czynni	2	20	2,85714	0	2,85714
1	0	1	trzy i więce	2	165	23,5714	15	8,57143
0	0	1	dwa czynni	3	320	45,7143	42,8571	2,85714
0	0	1	dwa czynni	2	60	8,57143	0	8,57143

0	0	1	dwa czynni	1	0	0	0	0
1	1	1	trzy i więce	2	0	0	0	0
0	1	1	trzy i więce	3	0	0	0	0
0	0	1	dwa czynni	3	570	81,4286	81,4286	0
0	1	1	dwa czynni	1	120	17,1429	0	17,1429
0	0	1	jeden czyn	1	0	0	0	0
0	0	1	dwa czynni	3	105	15	2,14286	12,8571
0	0	1	dwa czynni	2	600	85,7143	85,7143	0
0	1	1	trzy i więce	3	240	34,2857	8,57143	25,7143
0	1	1	trzy i więce	3	240	34,2857	34,2857	0
0	0	1	trzy i więce	3	50	7,14286	0	7,14286
0	0	1	dwa czynni	3	120	17,1429	17,1429	0
0	1	1	trzy i więce	1	0	0	0	0
0	0	0	jeden czyn	1	60	8,57143	0	8,57143
0	0	1	jeden czyn	3	180	25,7143	25,7143	0
0	1	1	trzy i więce	2	480	68,5714	8,57143	60
0	1	1	trzy i więce	2	60	8,57143	0	8,57143
0	0	1	dwa czynni	2	120	17,1429	0	17,1429
0	0	1	dwa czynni	2	120	17,1429	17,1429	0
1	1	1	trzy i więce	3	0	0	0	0
0	0	1	dwa czynni	1	0	0	0	0
0	1	0	dwa czynni	2	90	12,8571	0	12,8571
0	0	1	jeden czyn	2	420	60	30	30
0	0	1	jeden czyn	2	0	0	0	0
0	0	1	jeden czyn	2	120	17,1429	8,57143	8,57143
0	0	1	dwa czynni	1	0	0	0	0
0	0	0	metabolicz	3	60	8,57143	0	8,57143
0	0	1	jeden czyn	1	210	30	0	30
1	0	1	trzy i więce	2	180	25,7143	25,7143	0
0	1	1	trzy i więce	1	780	111,429	51,4286	60
0	0	0	metabolicz	2	30	4,28571	0	4,28571
0	0	1	dwa czynni	1	0	0	0	0
0	0	0	jeden czyn	3	360	51,4286	0	51,4286
0	0	1	dwa czynni	3	0	0	0	0
0	0	1	dwa czynni	2	120	17,1429	17,1429	0
0	0	1	trzy i więce	2	0	0	0	0
1	0	1	trzy i więce	2	75	10,7143	0	10,7143
1	0	1	trzy i więce	1	0	0	0	0
1	0	0	dwa czynni	3	0	0	0	0
0	0	1	dwa czynni	2	80	11,4286	0	11,4286
0	1	1	trzy i więce	3	0	0	0	0
0	0	0	jeden czyn	3	0	0	0	0
0	0	1	jeden czyn	3	30	4,28571	0	4,28571
0	0	0	metabolicz	2	0	0	0	0
0	0	0	jeden czyn	3	40	5,71429	0	5,71429
0	0	1	dwa czynni	2	0	0	0	0
0	0	1	dwa czynni	3	540	77,1429	17,1429	60
0	0	1	dwa czynni	3	0	0	0	0

0	0	1	dwa czynni	1	210	30	17,1429	12,8571
0	0	1	jeden czyn	1	0	0	0	0
0	0	1	jeden czyn	3	540	77,1429	77,1429	0
1	0	1	trzy i więce	1	120	17,1429	0	17,1429
0	1	0	dwa czynni	2	0	0	0	0
0	1	0	dwa czynni	2	60	8,57143	0	8,57143
0	1	0	jeden czyn	1	120	17,1429	17,1429	0
0	0	0	jeden czyn	2	120	17,1429	17,1429	0
0	1	1	dwa czynni	1	0	0	0	0
0	0	0	jeden czyn	2	120	17,1429	0	17,1429
0	0	1	jeden czyn	3	0	0	0	0
0	0	1	dwa czynni	2	120	17,1429	17,1429	0
0	0	1	jeden czyn	1	85	12,1429	10	2,14286
0	0	1	jeden czyn	2	60	8,57143	8,57143	0
0	0	1	dwa czynni	3	540	77,1429	0	77,1429
0	1	1	trzy i więce	3	0	0	0	0
0	0	0	metabolicz	3	180	25,7143	25,7143	0
0	0	0	jeden czyn	2	75	10,7143	0	10,7143
0	1	0	dwa czynni	3	0	0	0	0
0	1	1	trzy i więce	3	0	0	0	0
0	0	0	jeden czyn	2	0	0	0	0
0	1	1	trzy i więce	1	240	34,2857	34,2857	0
0	0	1	dwa czynni	2	60	8,57143	0	8,57143
1	1	1	trzy i więce	2	120	17,1429	0	17,1429
0	0	1	dwa czynni	2	70	10	10	0
0	0	1	dwa czynni	3	0	0	0	0
0	1	1	trzy i więce	3	90	12,8571	0	12,8571
0	1	1	trzy i więce	2	0	0	0	0
0	0	1	jeden czyn	2	0	0	0	0
1	0	0	jeden czyn	2	40	5,71429	0	5,71429
0	1	1	trzy i więce	3	0	0	0	0
0	0	0	metabolicz	1	0	0	0	0
1	0	1	trzy i więce	1	60	8,57143	0	8,57143
0	1	1	trzy i więce	3	840	120	0	120
0	0	0	metabolicz	2	720	102,857	102,857	0
1	0	1	trzy i więce	1	420	60	0	60
1	0	1	trzy i więce	1	120	17,1429	17,1429	0
0	0	0	dwa czynni	2	1140	162,857	154,286	8,57143
1	0	0	trzy i więce	3	0	0	0	0
0	0	0	metabolicz	2	60	8,57143	0	8,57143
0	1	1	trzy i więce	2	60	8,57143	8,57143	0
0	0	0	jeden czyn	3	90	12,8571	0	12,8571
0	0	1	jeden czyn	1	0	0	0	0
0	0	1	jeden czyn	2	0	0	0	0
0	1	1	trzy i więce	1	300	42,8571	17,1429	25,7143
1	0	0	trzy i więce	1	360	51,4286	8,57143	42,8571
0	0	1	dwa czynni	3	0	0	0	0
0	0	1	trzy i więce	3	0	0	0	0

1	*1*	*1*	trzy i więce	3	*120*	*17,1429*	*8,57143*	*8,57143*
0	*0*	*1*	dwa czynni	1	*0*	*0*	*0*	*0*
0	*1*	*1*	trzy i więce	3	*0*	*0*	*0*	*0*
1	*0*	*0*	dwa czynni	1	*0*	*0*	*0*	*0*
0	*0*	*0*	metabolicz	1	*420*	*60*	*60*	*0*
0	*1*	*0*	dwa czynni	2	*190*	*27,1429*	*10*	*17,1429*
0	*0*	*1*	dwa czynni	1	*60*	*8,57143*	*0*	*8,57143*
0	*0*	*1*	trzy i więce	2	*0*	*0*	*0*	*0*
0	*0*	*1*	dwa czynni	2	*120*	*17,1429*	*0*	*17,1429*
0	*0*	*1*	dwa czynni	3	*0*	*0*	*0*	*0*
0	*0*	*1*	dwa czynni	1	*60*	*8,57143*	*0*	*8,57143*
1	*0*	*1*	trzy i więce	2	*0*	*0*	*0*	*0*
0	*0*	*1*	dwa czynni	3	*180*	*25,7143*	*0*	*25,7143*
0	*0*	*0*	dwa czynni	2	*180*	*25,7143*	*0*	*25,7143*
0	*0*	*0*	dwa czynni	2	*0*	*0*	*0*	*0*
0	*0*	*1*	jeden czyn	3	*195*	*27,8571*	*19,2857*	*8,57143*
0	*1*	*1*	dwa czynni	2	*90*	*12,8571*	*12,8571*	*0*
0	*0*	*0*	metabolicz	2	*150*	*21,4286*	*21,4286*	*0*
0	*0*	*1*	jeden czyn	2	*50*	*7,14286*	*0*	*7,14286*
0	*1*	*0*	dwa czynni	3	*0*	*0*	*0*	*0*
1	*0*	*1*	trzy i więce	3	*180*	*25,7143*	*8,57143*	*17,1429*
0	*0*	*1*	jeden czyn	1	*210*	*30*	*0*	*30*
0	*0*	*1*	trzy i więce	2	*60*	*8,57143*	*0*	*8,57143*
1	*1*	*0*	dwa czynni	2	*120*	*17,1429*	*17,1429*	*0*
1	*0*	*0*	trzy i więce	3	*0*	*0*	*0*	*0*
0	*1*	*1*	trzy i więce	3	*0*	*0*	*0*	*0*
0	*0*	*1*	trzy i więce	1	*960*	*137,143*	*34,2857*	*102,857*
0	*1*	*1*	trzy i więce	1	*300*	*42,8571*	*0*	*42,8571*
0	*0*	*0*	jeden czyn	2	*0*	*0*	*0*	*0*
0	*0*	*1*	jeden czyn	2	*0*	*0*	*0*	*0*
0	*1*	*1*	trzy i więce	2	*105*	*15*	*0*	*15*
0	*0*	*0*	metabolicz	2	*1260*	*180*	*0*	*180*
0	*0*	*1*	trzy i więce	3	*0*	*0*	*0*	*0*
1	*1*	*1*	trzy i więce	3	*560*	*80*	*80*	*0*
1	*0*	*1*	trzy i więce	3	*0*	*0*	*0*	*0*
1	*1*	*1*	trzy i więce	2	*30*	*4,28571*	*0*	*4,28571*
0	*0*	*1*	dwa czynni	1	*0*	*0*	*0*	*0*
0	*0*	*0*	jeden czyn	2	*0*	*0*	*0*	*0*
0	*1*	*1*	trzy i więce	2	*140*	*20*	*0*	*20*
0	*1*	*1*	trzy i więce	2	*0*	*0*	*0*	*0*
1	*0*	*1*	trzy i więce	2	*420*	*60*	*0*	*60*
1	*0*	*1*	trzy i więce	3	*60*	*8,57143*	*0*	*8,57143*
1	*0*	*1*	trzy i więce	3	*340*	*48,5714*	*8,57143*	*40*
0	*1*	*1*	trzy i więce	3	*1260*	*180*	*0*	*180*
1	*0*	*1*	trzy i więce	2	*0*	*0*	*0*	*0*
0	*0*	*0*	jeden czyn	2	*60*	*8,57143*	*8,57143*	*0*
1	*1*	*1*	trzy i więce	3	*90*	*12,8571*	*0*	*12,8571*
0	*1*	*1*	trzy i więce	3	*0*	*0*	*0*	*0*

0	0	0	jeden czyn	2	180	25,7143	12,8571	12,8571
1	0	1	trzy i więce	2	960	137,143	85,7143	51,4286
1	1	1	trzy i więce	3	0	0	0	0
0	1	1	trzy i więce	2	180	25,7143	0	25,7143
0	0	1	trzy i więce	3	0	0	0	0
0	0	1	jeden czyn	2	0	0	0	0
1	0	1	trzy i więce	2	0	0	0	0
0	0	0	jeden czyn	2	60	8,57143	0	8,57143
0	0	1	trzy i więce	1	0	0	0	0
0	0	1	dwa czynni	3	330	47,1429	25,7143	21,4286
0	0	1	dwa czynni	2	0	0	0	0
0	0	1	jeden czyn	2	420	60	0	60
1	1	1	trzy i więce	1	0	0	0	0
1	0	1	trzy i więce	3	180	25,7143	8,57143	17,1429
1	1	1	trzy i więce	3	0	0	0	0
0	0	0	metabolicz	3	255	36,4286	19,2857	17,1429
0	0	1	dwa czynni	2	105	15	2,14286	12,8571
0	0	0	jeden czyn	2	240	34,2857	0	34,2857
0	0	1	dwa czynni	3	0	0	0	0
0	1	1	trzy i więce	3	345	49,2857	49,2857	0
0	0	0	jeden czyn	2	0	0	0	0
0	0	1	dwa czynni	2	210	30	0	30
0	1	1	trzy i więce	1	0	0	0	0
1	0	1	trzy i więce	3	420	60	0	60
0	0	1	dwa czynni	3	420	60	0	60
0	0	1	dwa czynni	2	0	0	0	0
0	0	1	trzy i więce	2	0	0	0	0
0	0	1	trzy i więce	3	60	8,57143	0	8,57143
0	0	1	dwa czynni	3	480	68,5714	17,1429	51,4286
1	1	1	trzy i więce	1	150	21,4286	0	21,4286
0	1	1	dwa czynni	3	120	17,1429	17,1429	0
0	1	0	jeden czyn	2	0	0	0	0
1	1	1	trzy i więce	2	0	0	0	0
0	0	1	dwa czynni	3	240	34,2857	0	34,2857
1	0	1	trzy i więce	3	100	14,2857	14,2857	0
1	1	1	trzy i więce	3	0	0	0	0
1	0	1	trzy i więce	2	120	17,1429	0	17,1429
0	1	1	trzy i więce	2	420	60	0	60
0	0	0	metabolicz	2	70	10	10	0
0	1	1	trzy i więce	2	300	42,8571	0	42,8571
0	0	1	dwa czynni	3	345	49,2857	32,1429	17,1429
1	0	0	dwa czynni	2	525	75	30	45
1	1	1	trzy i więce	2	600	85,7143	0	85,7143
1	1	1	trzy i więce	2	90	12,8571	0	12,8571
0	0	1	dwa czynni	2	360	51,4286	0	51,4286
0	1	1	trzy i więce	1	180	25,7143	0	25,7143
0	0	1	dwa czynni	2	0	0	0	0
0	0	1	dwa czynni	2	0	0	0	0

1	0	0	trzy i więce	2	0	0	0	0
0	0	1	dwa czynni	3	280	40	0	40
1	0	1	trzy i więce	2	0	0	0	0
0	0	1	dwa czynni	2	0	0	0	0
0	1	1	trzy i więce	2	240	34,2857	0	34,2857
1	1	1	trzy i więce	2	210	30	0	30
1	0	0	dwa czynni	2	90	12,8571	4,28571	8,57143
0	0	1	dwa czynni	2	0	0	0	0
0	0	1	dwa czynni	2	90	12,8571	0	12,8571
1	1	1	trzy i więce	2	0	0	0	0
1	0	1	trzy i więce	2	330	47,1429	25,7143	21,4286
0	0	1	trzy i więce	3	60	8,57143	8,57143	0
0	0	1	dwa czynni	3	0	0	0	0
0	0	0	metabolicz	3	180	25,7143	25,7143	0
1	1	1	trzy i więce	2	210	30	0	30
0	1	1	trzy i więce	2	180	25,7143	25,7143	0
1	1	1	trzy i więce	2	840	120	0	120
1	1	1	trzy i więce	3	120	17,1429	0	17,1429
1	1	1	trzy i więce	2	0	0	0	0
1	0	1	trzy i więce	2	0	0	0	0
0	0	1	dwa czynni	3	120	17,1429	17,1429	0
0	0	1	dwa czynni	2	120	17,1429	0	17,1429
0	1	1	trzy i więce	2	0	0	0	0
0	0	1	dwa czynni	2	0	0	0	0
0	1	1	trzy i więce	2	0	0	0	0
1	0	1	trzy i więce	3	80	11,4286	0	11,4286
0	1	1	trzy i więce	3	120	17,1429	0	17,1429
0	1	1	trzy i więce	1	0	0	0	0
0	0	1	dwa czynni	3	0	0	0	0
0	0	0	jeden czyn	2	0	0	0	0
1	1	1	trzy i więce	3	200	28,5714	0	28,5714
0	0	1	dwa czynni	2	60	8,57143	0	8,57143
0	1	1	trzy i więce	2	0	0	0	0
0	0	0	jeden czyn	2	30	4,28571	0	4,28571
0	1	1	trzy i więce	2	0	0	0	0
0	0	1	dwa czynni	2	0	0	0	0
0	0	1	dwa czynni	2	120	17,1429	0	17,1429
1	1	1	trzy i więce	2	0	0	0	0
0	0	1	trzy i więce	3	180	25,7143	0	25,7143
0	0	0	jeden czyn	2	260	37,1429	11,4286	25,7143
1	0	1	trzy i więce	2	0	0	0	0
0	0	0	jeden czyn	2	165	23,5714	6,42857	17,1429
1	0	1	trzy i więce	1	0	0	0	0
0	0	1	dwa czynni	3	0	0	0	0
1	0	0	dwa czynni	2	0	0	0	0
1	1	1	trzy i więce	2	0	0	0	0
1	1	0	trzy i więce	2	0	0	0	0
0	0	1	dwa czynni	3	120	17,1429	0	17,1429

1	0	1	trzy i więc	1	0	0	0	0
1	1	1	trzy i więc	2	0	0	0	0
0	0	1	dwa czynni	2	0	0	0	0
1	0	1	dwa czynni	2	180	25,7143	0	25,7143
0	1	1	trzy i więc	2	120	17,1429	0	17,1429
1	1	1	trzy i więc	2	660	94,2857	42,8571	51,4286
1	1	1	trzy i więc	3	135	19,2857	10,7143	8,57143
1	0	0	trzy i więc	3	155	22,1429	2,14286	20
0	0	1	dwa czynni	2	525	75	15	60
0	1	0	dwa czynni	2	0	0	0	0
0	1	1	trzy i więc	3	120	17,1429	17,1429	0
0	0	1	dwa czynni	2	120	17,1429	0	17,1429
1	1	1	trzy i więc	2	300	42,8571	0	42,8571
1	1	1	trzy i więc	2	0	0	0	0
0	0	1	jeden czyn	2	910	130	10	120
1	0	1	trzy i więc	2	0	0	0	0
1	1	1	trzy i więc	2	285	40,7143	15	25,7143
0	0	1	dwa czynni	2	0	0	0	0
0	0	0	metabolicz	2	120	17,1429	0	17,1429
1	1	1	trzy i więc	2	120	17,1429	0	17,1429
0	1	1	trzy i więc	2	75	10,7143	10,7143	0
0	1	0	dwa czynni	2	0	0	0	0
1	1	1	trzy i więc	3	180	25,7143	0	25,7143
1	1	1	trzy i więc	2	300	42,8571	0	42,8571
0	0	1	dwa czynni	2	140	20	20	0
0	1	1	trzy i więc	2	0	0	0	0
0	0	1	jeden czyn	2	120	17,1429	0	17,1429
0	1	1	trzy i więc	3	60	8,57143	0	8,57143
0	0	1	jeden czyn	3	0	0	0	0
0	0	1	trzy i więc	3	180	25,7143	0	25,7143
0	0	0	metabolicz	2	0	0	0	0
0	0	1	jeden czyn	3	315	45	19,2857	25,7143
1	1	1	trzy i więc	2	120	17,1429	0	17,1429
0	1	1	trzy i więc	1	0	0	0	0
1	0	0	trzy i więc	1	0	0	0	0
0	1	0	dwa czynni	2	150	21,4286	17,1429	4,28571
0	0	1	dwa czynni	2	160	22,8571	0	22,8571
1	1	1	trzy i więc	2	420	60	0	60
1	0	1	trzy i więc	1	0	0	0	0
0	0	0	jeden czyn	3	210	30	0	30
1	0	1	trzy i więc	3	1260	180	0	180
1	1	0	trzy i więc	3	120	17,1429	8,57143	8,57143
0	0	1	trzy i więc	2	180	25,7143	0	25,7143
0	0	1	trzy i więc	1	0	0	0	0
0	1	1	trzy i więc	3	0	0	0	0
0	0	0	jeden czyn	3	0	0	0	0
1	0	1	trzy i więc	3	0	0	0	0
0	0	1	jeden czyn	2	300	42,8571	0	42,8571

0	1	1	trzy i więce	3	270	38,5714	21,4286	17,1429
1	1	1	trzy i więce	2	0	0	0	0
0	1	1	trzy i więce	2	360	51,4286	0	51,4286
0	0	1	trzy i więce	3	210	30	30	0
0	0	1	dwa czynni	2	0	0	0	0
1	1	0	trzy i więce	1	90	12,8571	12,8571	0
0	0	1	trzy i więce	3	60	8,57143	0	8,57143
1	1	1	trzy i więce	3	240	34,2857	0	34,2857
1	0	1	trzy i więce	3	30	4,28571	0	4,28571
1	1	1	trzy i więce	2	420	60	0	60
1	0	0	trzy i więce	2	90	12,8571	2,85714	10
1	1	1	trzy i więce	3	0	0	0	0
0	1	1	trzy i więce	1	240	34,2857	0	34,2857
1	1	1	trzy i więce	3	30	4,28571	0	4,28571
1	0	1	trzy i więce	2	0	0	0	0
0	1	1	trzy i więce	2	120	17,1429	0	17,1429
1	0	1	trzy i więce	2	120	17,1429	0	17,1429
0	0	1	jeden czyn	2	180	25,7143	0	25,7143
0	0	1	jeden czyn	2	60	8,57143	0	8,57143
0	0	1	dwa czynni	2	60	8,57143	0	8,57143
0	0	1	dwa czynni	2	120	17,1429	0	17,1429
0	0	1	dwa czynni	2	0	0	0	0
0	1	0	jeden czyn	2	0	0	0	0
0	1	1	trzy i więce	2	120	17,1429	0	17,1429
1	1	1	trzy i więce	2	780	111,429	51,4286	60
0	0	1	dwa czynni	2	0	0	0	0
0	0	1	dwa czynni	2	360	51,4286	0	51,4286
1	0	1	trzy i więce	2	0	0	0	0
1	1	1	trzy i więce	1	150	21,4286	0	21,4286
1	1	1	trzy i więce	2	140	20	0	20
0	1	1	trzy i więce	2	150	21,4286	0	21,4286
0	1	1	trzy i więce	2	70	10	10	0
1	1	1	trzy i więce	3	190	27,1429	10	17,1429
1	0	1	trzy i więce	2	630	90	0	90
1	1	1	trzy i więce	1	360	51,4286	0	51,4286
0	1	1	trzy i więce	2	1860	265,714	85,7143	180
1	0	1	trzy i więce	3	0	0	0	0
0	1	0	dwa czynni	2	975	139,286	19,2857	120
0	1	1	trzy i więce	2	0	0	0	0
1	1	1	trzy i więce	3	0	0	0	0
1	0	1	trzy i więce	3	0	0	0	0
0	0	0	metabolicz	1	1260	180	0	180
1	0	1	trzy i więce	2	0	0	0	0
1	0	1	trzy i więce	3	0	0	0	0
0	0	1	jeden czyn	1	60	8,57143	0	8,57143
0	0	1	dwa czynni	2	0	0	0	0
0	1	1	trzy i więce	3	60	8,57143	0	8,57143
1	1	0	trzy i więce	3	420	60	0	60

0	1	0	trzy i więce	2	60	8,57143	0	8,57143
0	0	1	jeden czyn	2	330	47,1429	30	17,1429
0	0	1	dwa czynni	2	0	0	0	0
0	0	1	dwa czynni	2	0	0	0	0
1	1	1	trzy i więce	2	420	60	8,57143	51,4286
1	1	1	trzy i więce	2	0	0	0	0
0	0	1	jeden czyn	3	360	51,4286	0	51,4286
0	0	1	dwa czynni	3	105	15	15	0
0	0	1	dwa czynni	3	0	0	0	0
0	1	1	trzy i więce	2	0	0	0	0
0	0	1	dwa czynni	2	0	0	0	0
1	0	1	trzy i więce	3	260	37,1429	20	17,1429
0	0	1	trzy i więce	2	780	111,429	51,4286	60
0	0	1	dwa czynni	3	0	0	0	0
0	0	1	dwa czynni	2	120	17,1429	0	17,1429
1	1	1	trzy i więce	2	20	2,85714	0	2,85714
0	1	1	dwa czynni	3	420	60	0	60
0	0	1	dwa czynni	3	705	100,714	32,1429	68,5714
0	1	1	trzy i więce	2	420	60	0	60
0	0	1	dwa czynni	2	0	0	0	0
0	0	1	dwa czynni	2	210	30	0	30
0	0	1	dwa czynni	2	0	0	0	0
0	0	1	dwa czynni	2	60	8,57143	0	8,57143
1	0	1	trzy i więce	2	360	51,4286	0	51,4286
0	0	1	dwa czynni	2	60	8,57143	0	8,57143
0	0	1	dwa czynni	2	0	0	0	0
0	0	0	metabolicz	1	180	25,7143	0	25,7143
0	0	0	jeden czyn	2	60	8,57143	0	8,57143
0	0	0	jeden czyn	2	330	47,1429	38,5714	8,57143
0	0	1	dwa czynni	3	210	30	30	0
1	0	1	trzy i więce	3	480	68,5714	0	68,5714
1	0	0	trzy i więce	2	690	98,5714	8,57143	90
1	0	1	trzy i więce	2	210	30	12,8571	17,1429
0	0	1	dwa czynni	1	0	0	0	0
0	1	1	trzy i więce	3	0	0	0	0
1	1	1	trzy i więce	2	150	21,4286	8,57143	12,8571
0	0	1	jeden czyn	2	0	0	0	0
1	1	1	trzy i więce	2	90	12,8571	0	12,8571
0	0	1	dwa czynni	1	0	0	0	0
1	0	1	trzy i więce	1	20	2,85714	0	2,85714
0	1	1	dwa czynni	2	630	90	4,28571	85,7143
0	1	1	trzy i więce	2	0	0	0	0
1	0	0	trzy i więce	2	180	25,7143	0	25,7143
0	1	1	trzy i więce	3	60	8,57143	0	8,57143
0	1	1	trzy i więce	3	40	5,71429	5,71429	0
0	0	1	trzy i więce	2	15	2,14286	0	2,14286
0	0	1	dwa czynni	2	0	0	0	0
0	1	1	trzy i więce	3	0	0	0	0

0	0	1	dwa czynni	3	240	34,2857	0	34,2857
0	0	1	dwa czynni	3	0	0	0	0
0	0	1	dwa czynni	2	720	102,857	0	102,857
0	0	1	dwa czynni	2	480	68,5714	51,4286	17,1429
0	0	1	dwa czynni	2	315	45	0	45
0	0	0	jeden czyn	3	280	40	40	0
0	1	1	trzy i więc	2	75	10,7143	0	10,7143
0	0	0	jeden czyn	2	945	135	15	120
0	0	1	dwa czynni	1	120	17,1429	0	17,1429
1	0	1	trzy i więc	2	180	25,7143	25,7143	0
0	0	1	dwa czynni	3	120	17,1429	17,1429	0
0	0	0	jeden czyn	2	90	12,8571	12,8571	0
1	0	1	trzy i więc	2	30	4,28571	0	4,28571
1	0	1	trzy i więc	2	60	8,57143	0	8,57143
1	0	1	trzy i więc	1	0	0	0	0
1	1	1	trzy i więc	3	60	8,57143	0	8,57143
1	0	1	trzy i więc	3	60	8,57143	0	8,57143
1	1	1	trzy i więc	2	500	71,4286	20	51,4286
0	0	1	dwa czynni	2	0	0	0	0
0	0	1	dwa czynni	3	360	51,4286	0	51,4286
0	0	1	dwa czynni	2	90	12,8571	0	12,8571
0	0	1	dwa czynni	2	420	60	0	60
1	1	1	trzy i więc	1	720	102,857	0	102,857
0	0	0	jeden czyn	3	420	60	0	60
0	0	1	dwa czynni	3	0	0	0	0
1	0	1	trzy i więc	2	60	8,57143	0	8,57143
0	1	1	dwa czynni	3	840	120	0	120
1	0	1	trzy i więc	3	90	12,8571	0	12,8571
0	0	1	dwa czynni	3	0	0	0	0
0	0	1	jeden czyn	2	60	8,57143	8,57143	0
1	0	1	trzy i więc	2	420	60	8,57143	51,4286
1	0	1	trzy i więc	2	320	45,7143	20	25,7143
1	0	1	trzy i więc	3	0	0	0	0
0	0	1	dwa czynni	2	360	51,4286	25,7143	25,7143
0	0	1	jeden czyn	2	315	45	0	45
1	1	1	trzy i więc	2	210	30	4,28571	25,7143
0	0	1	dwa czynni	2	420	60	17,1429	42,8571
0	0	0	metabolicz	3	180	25,7143	0	25,7143
1	0	1	trzy i więc	2	390	55,7143	30	25,7143
1	0	1	trzy i więc	2	120	17,1429	0	17,1429
1	1	1	trzy i więc	3	120	17,1429	0	17,1429
0	0	1	trzy i więc	3	120	17,1429	0	17,1429
0	0	0	metabolicz	3	0	0	0	0
1	1	1	trzy i więc	2	360	51,4286	0	51,4286
1	0	1	trzy i więc	2	140	20	2,85714	17,1429
0	1	1	dwa czynni	2	0	0	0	0
0	1	1	trzy i więc	3	0	0	0	0
0	0	0	metabolicz	1	60	8,57143	0	8,57143

0	0	0	dwa czynni	2	420	60	0	60
1	1	1	trzy i więce	3	0	0	0	0
0	0	1	dwa czynni	2	0	0	0	0
0	0	0	metabolicz	2	375	53,5714	19,2857	34,2857
0	0	1	jeden czyn	2	120	17,1429	0	17,1429
0	1	1	trzy i więce	2	420	60	0	60
0	0	1	trzy i więce	2	0	0	0	0
1	0	1	dwa czynni	2	135	19,2857	19,2857	0
0	0	1	dwa czynni	2	0	0	0	0
1	0	1	trzy i więce	3	240	34,2857	0	34,2857
1	0	1	trzy i więce	2	180	25,7143	0	25,7143
1	1	0	trzy i więce	1	120	17,1429	0	17,1429
1	1	1	trzy i więce	2	440	62,8571	2,85714	60
0	0	1	trzy i więce	2	60	8,57143	8,57143	0
1	1	1	trzy i więce	2	240	34,2857	0	34,2857
1	1	1	trzy i więce	2	180	25,7143	0	25,7143
1	0	1	trzy i więce	2	480	68,5714	25,7143	42,8571
1	0	0	trzy i więce	2	420	60	0	60
0	0	1	dwa czynni	3	0	0	0	0
1	1	0	trzy i więce	2	240	34,2857	0	34,2857
0	0	0	jeden czyn	3	465	66,4286	15	51,4286
1	0	1	trzy i więce	2	360	51,4286	17,1429	34,2857
0	1	0	jeden czyn	1	180	25,7143	0	25,7143
0	1	1	trzy i więce	3	120	17,1429	0	17,1429
0	0	0	jeden czyn	2	0	0	0	0
1	0	0	dwa czynni	3	0	0	0	0
0	1	1	trzy i więce	3	120	17,1429	0	17,1429
0	0	1	jeden czyn	2	90	12,8571	12,8571	0
1	1	1	trzy i więce	1	210	30	0	30
1	0	1	trzy i więce	2	30	4,28571	0	4,28571
0	0	1	jeden czyn	3	260	37,1429	17,1429	20
1	0	0	trzy i więce	2	1305	186,429	6,42857	180
1	0	1	trzy i więce	3	420	60	0	60
0	1	1	trzy i więce	3	60	8,57143	0	8,57143
1	0	1	trzy i więce	3	0	0	0	0
1	0	1	trzy i więce	2	210	30	4,28571	25,7143
1	1	1	trzy i więce	2	0	0	0	0
0	0	1	trzy i więce	1	0	0	0	0
0	1	1	trzy i więce	1	420	60	0	60
0	0	0	jeden czyn	2	1080	154,286	0	154,286
1	0	1	trzy i więce	3	380	54,2857	28,5714	25,7143
0	0	1	trzy i więce	2	360	51,4286	0	51,4286
1	1	1	trzy i więce	3	0	0	0	0
1	0	1	trzy i więce	3	105	15	15	0
0	0	1	dwa czynni	3	420	60	8,57143	51,4286
1	1	1	trzy i więce	3	1080	154,286	34,2857	120
1	1	1	trzy i więce	3	510	72,8571	12,8571	60
1	1	1	trzy i więce	2	420	60	0	60

1	1	1	trzy i więce	3	90	12,8571	12,8571	0
1	0	1	trzy i więce	3	735	105	15	90
0	1	1	dwa czynni	2	180	25,7143	25,7143	0
0	1	1	trzy i więce	3	440	62,8571	11,4286	51,4286
1	0	1	trzy i więce	2	495	70,7143	10,7143	60
1	1	1	trzy i więce	2	0	0	0	0
0	0	1	dwa czynni	2	0	0	0	0
1	1	1	trzy i więce	2	140	20	0	20
0	1	1	trzy i więce	2	0	0	0	0
0	0	0	metabolicz	1	0	0	0	0
1	0	0	trzy i więce	3	60	8,57143	0	8,57143
1	1	1	trzy i więce	3	90	12,8571	0	12,8571
1	1	0	trzy i więce	3	120	17,1429	0	17,1429
1	1	1	trzy i więce	2	0	0	0	0
0	0	1	dwa czynni	2	105	15	0	15
0	0	1	dwa czynni	3	0	0	0	0
1	0	1	trzy i więce	2	420	60	0	60
0	0	0	jeden czyn	3	120	17,1429	17,1429	0
0	1	1	dwa czynni	2	0	0	0	0
0	0	1	jeden czyn	2	60	8,57143	8,57143	0
1	1	1	trzy i więce	2	60	8,57143	0	8,57143
1	0	1	trzy i więce	2	0	0	0	0
0	0	1	dwa czynni	2	240	34,2857	0	34,2857
0	1	1	trzy i więce	3	150	21,4286	0	21,4286
0	0	1	dwa czynni	2	0	0	0	0
0	0	1	dwa czynni	2	180	25,7143	0	25,7143
0	0	1	dwa czynni	2	840	120	0	120
0	0	1	dwa czynni	1	0	0	0	0
0	1	1	trzy i więce	3	0	0	0	0
1	1	1	trzy i więce	3	130	18,5714	10	8,57143
0	0	1	dwa czynni	2	80	11,4286	0	11,4286
1	1	1	trzy i więce	2	240	34,2857	4,28571	30
1	1	1	trzy i więce	2	0	0	0	0
1	0	0	dwa czynni	2	180	25,7143	0	25,7143
0	1	1	trzy i więce	2	840	120	0	120
1	0	1	trzy i więce	2	150	21,4286	0	21,4286
0	0	1	dwa czynni	3	420	60	0	60
0	0	1	dwa czynni	2	420	60	0	60
0	1	1	trzy i więce	2	0	0	0	0
1	1	0	trzy i więce	2	240	34,2857	0	34,2857
1	0	1	trzy i więce	3	285	40,7143	15	25,7143
0	0	1	dwa czynni	1	330	47,1429	4,28571	42,8571
0	1	0	jeden czyn	3	90	12,8571	0	12,8571
1	0	1	trzy i więce	2	180	25,7143	8,57143	17,1429
0	1	1	trzy i więce	3	0	0	0	0
0	0	0	jeden czyn	2	0	0	0	0
1	0	1	trzy i więce	2	315	45	19,2857	25,7143
0	1	1	trzy i więce	3	180	25,7143	0	25,7143

0	0	1	dwa czynni	2	480	68,5714	0	68,5714
0	0	1	dwa czynni	3	0	0	0	0
0	0	0	jeden czyn	2	120	17,1429	0	17,1429
1	1	1	trzy i więc	1	420	60	0	60
0	0	1	dwa czynni	2	120	17,1429	0	17,1429
0	1	1	dwa czynni	1	180	25,7143	0	25,7143
0	0	1	dwa czynni	2	0	0	0	0
0	1	1	trzy i więc	3	420	60	0	60
1	0	1	trzy i więc	2	360	51,4286	0	51,4286
0	0	0	metabolicz	3	240	34,2857	34,2857	0
1	1	1	trzy i więc	2	200	28,5714	0	28,5714
0	1	1	trzy i więc	3	210	30	0	30
1	1	1	trzy i więc	1	0	0	0	0
0	0	1	dwa czynni	2	45	6,42857	6,42857	0
1	1	1	trzy i więc	2	420	60	0	60
1	1	1	trzy i więc	2	30	4,28571	4,28571	0
1	0	1	trzy i więc	2	0	0	0	0
0	0	0	jeden czyn	2	0	0	0	0
1	0	1	dwa czynni	2	1260	180	0	180
0	0	0	metabolicz	2	210	30	0	30
1	0	1	dwa czynni	2	630	90	30	60
0	0	0	jeden czyn	2	420	60	0	60
0	0	1	dwa czynni	2	360	51,4286	42,8571	8,57143
0	1	1	trzy i więc	3	630	90	30	60
1	1	0	trzy i więc	3	160	22,8571	22,8571	0
0	0	1	dwa czynni	3	1020	145,714	25,7143	120
0	0	1	dwa czynni	2	0	0	0	0
0	0	1	dwa czynni	2	0	0	0	0
0	1	1	dwa czynni	3	270	38,5714	8,57143	30
0	0	0	jeden czyn	2	80	11,4286	11,4286	0
0	0	1	dwa czynni	2	180	25,7143	25,7143	0
1	1	1	trzy i więc	2	160	22,8571	0	22,8571
0	0	0	jeden czyn	3	300	42,8571	42,8571	0
1	1	1	trzy i więc	1	0	0	0	0
0	1	0	dwa czynni	3	135	19,2857	19,2857	0
0	1	1	trzy i więc	3	0	0	0	0
1	0	1	trzy i więc	2	240	34,2857	0	34,2857
1	0	1	trzy i więc	2	420	60	0	60
1	0	1	trzy i więc	2	280	40	40	0
1	1	1	trzy i więc	3	60	8,57143	0	8,57143
1	0	1	trzy i więc	2	480	68,5714	0	68,5714
0	0	1	dwa czynni	2	0	0	0	0
0	1	1	trzy i więc	1	0	0	0	0
1	0	0	trzy i więc	2	0	0	0	0
0	0	0	metabolicz	2	300	42,8571	0	42,8571
1	0	1	trzy i więc	1	60	8,57143	0	8,57143
1	1	1	trzy i więc	2	0	0	0	0
1	0	1	trzy i więc	2	480	68,5714	68,5714	0

1	0	1	trzy i więce	2	0	0	0	0
0	0	1	jeden czyn	2	420	60	0	60
0	0	0	metabolicz	1	0	0	0	0
0	0	1	jeden czyn	3	360	51,4286	0	51,4286
1	1	1	trzy i więce	2	0	0	0	0
0	0	1	trzy i więce	2	225	32,1429	0	32,1429
0	0	1	jeden czyn	3	90	12,8571	0	12,8571
0	0	1	dwa czynni	2	40	5,71429	5,71429	0
0	0	0	jeden czyn	3	0	0	0	0
0	0	1	trzy i więce	3	0	0	0	0
0	0	0	metabolicz	3	360	51,4286	0	51,4286
1	1	0	trzy i więce	3	930	132,857	12,8571	120
0	0	1	jeden czyn	3	60	8,57143	0	8,57143
0	1	1	trzy i więce	2	0	0	0	0
1	0	0	dwa czynni	2	190	27,1429	10	17,1429
0	0	0	jeden czyn	2	60	8,57143	0	8,57143
0	1	1	trzy i więce	2	140	20	0	20
0	0	1	dwa czynni	2	0	0	0	0
1	0	0	trzy i więce	3	420	60	0	60
0	0	1	dwa czynni	2	0	0	0	0
0	1	1	trzy i więce	3	310	44,2857	19,2857	25
1	0	0	trzy i więce	1	120	17,1429	0	17,1429
0	0	1	dwa czynni	3	0	0	0	0
0	0	1	dwa czynni	3	660	94,2857	34,2857	60
0	0	1	dwa czynni	3	435	62,1429	45	17,1429
0	0	1	trzy i więce	2	420	60	0	60
1	0	1	trzy i więce	2	0	0	0	0
0	1	1	trzy i więce	3	600	85,7143	17,1429	68,5714
1	1	1	trzy i więce	2	0	0	0	0
0	0	0	jeden czyn	2	0	0	0	0
0	0	0	metabolicz	2	360	51,4286	0	51,4286
0	1	1	trzy i więce	2	120	17,1429	0	17,1429
0	0	1	dwa czynni	2	540	77,1429	25,7143	51,4286
1	0	1	trzy i więce	2	120	17,1429	0	17,1429
0	0	1	dwa czynni	3	420	60	0	60
0	0	1	dwa czynni	3	310	44,2857	8,57143	35,7143
0	0	1	dwa czynni	1	600	85,7143	68,5714	17,1429
0	0	1	trzy i więce	3	0	0	0	0
1	0	1	trzy i więce	2	0	0	0	0
1	0	1	trzy i więce	2	900	128,571	0	128,571
1	1	1	trzy i więce	2	120	17,1429	0	17,1429
0	0	1	jeden czyn	2	880	125,714	5,71429	120
0	1	1	trzy i więce	3	120	17,1429	0	17,1429
1	0	1	trzy i więce	3	190	27,1429	12,1429	15
1	1	1	trzy i więce	2	1260	180	0	180
0	0	1	dwa czynni	2	120	17,1429	0	17,1429
0	1	1	trzy i więce	2	360	51,4286	0	51,4286
1	1	1	trzy i więce	2	20	2,85714	2,85714	0

1	1	1	trzy i więc	3	0	0	0	0
0	0	0	metabolicz	2	0	0	0	0
1	1	1	trzy i więc	1	0	0	0	0
0	1	1	trzy i więc	2	30	4,28571	0	4,28571
0	0	1	dwa czynni	2	600	85,7143	0	85,7143
1	0	1	trzy i więc	3	60	8,57143	8,57143	0
1	1	1	trzy i więc	2	0	0	0	0
1	1	1	trzy i więc	2	150	21,4286	17,1429	4,28571
1	0	1	trzy i więc	3	0	0	0	0
0	1	1	trzy i więc	3	560	80	20	60
1	0	0	dwa czynni	3	330	47,1429	30	17,1429
1	1	1	trzy i więc	2	1260	180	0	180
0	0	0	jeden czyn	2	570	81,4286	42,8571	38,5714
1	1	1	trzy i więc	2	140	20	0	20
0	1	1	dwa czynni	3	480	68,5714	0	68,5714
1	1	1	trzy i więc	2	0	0	0	0
1	0	1	trzy i więc	2	480	68,5714	17,1429	51,4286
1	1	1	trzy i więc	2	180	25,7143	25,7143	0
0	0	1	dwa czynni	2	0	0	0	0
1	0	1	trzy i więc	2	300	42,8571	0	42,8571
0	0	0	metabolicz	3	420	60	0	60
0	0	1	dwa czynni	2	60	8,57143	0	8,57143
0	0	1	dwa czynni	2	420	60	0	60
1	0	1	trzy i więc	1	360	51,4286	0	51,4286
1	0	1	trzy i więc	2	120	17,1429	17,1429	0
0	0	0	metabolicz	3	0	0	0	0
0	0	0	jeden czyn	2	870	124,286	4,28571	120
0	0	1	trzy i więc	2	840	120	0	120
1	1	1	trzy i więc	3	0	0	0	0
1	1	1	trzy i więc	2	0	0	0	0
0	1	1	trzy i więc	3	300	42,8571	0	42,8571
0	0	1	jeden czyn	3	0	0	0	0
0	0	1	dwa czynni	3	45	6,42857	6,42857	0
1	0	1	trzy i więc	2	40	5,71429	0	5,71429
0	1	1	trzy i więc	2	1260	180	0	180
1	1	1	trzy i więc	1	840	120	60	60
1	1	1	trzy i więc	3	720	102,857	51,4286	51,4286
1	1	1	trzy i więc	3	280	40	25,7143	14,2857
1	1	1	trzy i więc	3	120	17,1429	0	17,1429
0	0	1	trzy i więc	3	240	34,2857	0	34,2857
0	0	0	metabolicz	1	105	15	15	0
1	1	1	trzy i więc	2	90	12,8571	0	12,8571
0	0	1	dwa czynni	2	120	17,1429	0	17,1429
1	1	0	trzy i więc	3	0	0	0	0
0	0	1	dwa czynni	3	120	17,1429	0	17,1429
0	0	0	jeden czyn	1	0	0	0	0
0	0	1	dwa czynni	3	210	30	0	30
0	1	1	trzy i więc	1	240	34,2857	0	34,2857

0	1	1	trzy i więce	3	0	0	0	0
0	0	0	jeden czyn	2	145	20,7143	6,42857	14,2857
1	0	1	trzy i więce	2	315	45	15	30
0	0	1	jeden czyn	2	165	23,5714	15	8,57143
0	1	1	trzy i więce	2	150	21,4286	0	21,4286
0	0	1	dwa czynni	3	0	0	0	0
0	0	0	jeden czyn	3	300	42,8571	0	42,8571
1	0	1	trzy i więce	2	250	35,7143	10	25,7143
0	0	0	metabolicz	2	0	0	0	0
0	1	1	trzy i więce	3	0	0	0	0
1	1	1	trzy i więce	2	60	8,57143	0	8,57143
0	0	1	trzy i więce	2	0	0	0	0
1	0	1	trzy i więce	2	80	11,4286	0	11,4286
1	1	1	trzy i więce	1	105	15	0	15
1	0	1	trzy i więce	2	180	25,7143	0	25,7143
0	0	1	dwa czynni	2	225	32,1429	12,8571	19,2857
1	1	1	trzy i więce	3	0	0	0	0
0	1	1	trzy i więce	3	360	51,4286	0	51,4286
0	1	1	trzy i więce	3	1260	180	0	180
0	0	1	jeden czyn	1	0	0	0	0
0	0	1	dwa czynni	2	0	0	0	0
0	1	1	trzy i więce	1	0	0	0	0
1	1	0	trzy i więce	3	0	0	0	0
1	1	1	trzy i więce	3	60	8,57143	0	8,57143
0	0	1	dwa czynni	2	210	30	0	30
0	1	1	trzy i więce	3	420	60	0	60
1	1	1	trzy i więce	2	270	38,5714	12,8571	25,7143
0	0	0	metabolicz	2	240	34,2857	8,57143	25,7143
0	0	1	trzy i więce	2	40	5,71429	0	5,71429
1	0	1	trzy i więce	2	0	0	0	0
1	0	1	trzy i więce	1	540	77,1429	0	77,1429
0	0	1	dwa czynni	3	660	94,2857	17,1429	77,1429
1	0	1	trzy i więce	2	525	75	15	60
1	0	0	trzy i więce	2	0	0	0	0
1	0	1	trzy i więce	2	0	0	0	0
1	1	1	trzy i więce	2	930	132,857	12,8571	120
0	0	1	dwa czynni	1	120	17,1429	8,57143	8,57143
0	0	0	jeden czyn	2	0	0	0	0
0	0	1	jeden czyn	2	0	0	0	0
0	0	1	jeden czyn	3	340	48,5714	5,71429	42,8571
1	1	1	trzy i więce	3	0	0	0	0
0	1	1	trzy i więce	2	120	17,1429	0	17,1429
1	0	0	dwa czynni	3	120	17,1429	0	17,1429
1	0	1	trzy i więce	2	560	80	20	60
1	0	1	trzy i więce	3	0	0	0	0
1	0	1	trzy i więce	2	0	0	0	0
0	0	1	jeden czyn	2	180	25,7143	25,7143	0
1	1	1	trzy i więce	2	0	0	0	0

0	0	1	dwa czynni	2	420	60	0	60
0	0	1	dwa czynni	1	60	8,57143	0	8,57143
0	1	1	trzy i więc	3	630	90	30	60
0	0	1	dwa czynni	2	180	25,7143	17,1429	8,57143
1	0	1	trzy i więc	2	0	0	0	0
0	0	0	metabolicz	2	180	25,7143	0	25,7143
0	0	1	jeden czyn	1	420	60	0	60
1	1	1	trzy i więc	1	240	34,2857	0	34,2857
1	0	1	trzy i więc	2	285	40,7143	15	25,7143
0	0	1	jeden czyn	2	0	0	0	0
1	1	1	trzy i więc	2	0	0	0	0
1	0	1	trzy i więc	2	150	21,4286	17,1429	4,28571
0	0	1	dwa czynni	3	120	17,1429	0	17,1429
1	0	1	trzy i więc	2	0	0	0	0
1	1	1	trzy i więc	2	90	12,8571	12,8571	0
0	0	1	dwa czynni	2	240	34,2857	0	34,2857
0	1	0	dwa czynni	2	180	25,7143	0	25,7143
1	1	1	trzy i więc	2	90	12,8571	0	12,8571
1	1	1	trzy i więc	2	180	25,7143	8,57143	17,1429
1	0	1	trzy i więc	2	0	0	0	0
1	0	1	trzy i więc	3	450	64,2857	30	34,2857
0	0	1	dwa czynni	2	0	0	0	0
1	0	1	trzy i więc	2	30	4,28571	0	4,28571
1	1	1	trzy i więc	2	300	42,8571	42,8571	0
1	1	1	trzy i więc	3	60	8,57143	0	8,57143
0	0	1	dwa czynni	2	0	0	0	0
1	0	1	trzy i więc	2	0	0	0	0
0	0	0	jeden czyn	2	20	2,85714	0	2,85714
0	1	1	trzy i więc	3	420	60	60	0
1	0	1	trzy i więc	2	360	51,4286	0	51,4286
1	0	1	trzy i więc	1	0	0	0	0
1	0	0	trzy i więc	2	160	22,8571	8,57143	14,2857
1	1	1	trzy i więc	1	840	120	0	120
0	1	1	trzy i więc	2	0	0	0	0
0	0	0	jeden czyn	2	0	0	0	0
1	1	1	trzy i więc	2	120	17,1429	0	17,1429
1	0	1	trzy i więc	2	360	51,4286	0	51,4286
0	1	1	trzy i więc	1	210	30	0	30
1	0	1	trzy i więc	3	0	0	0	0
0	0	0	metabolicz	2	60	8,57143	0	8,57143
0	0	1	dwa czynni	2	120	17,1429	0	17,1429
1	0	1	trzy i więc	2	420	60	0	60
1	1	0	trzy i więc	2	315	45	15	30
1	0	0	trzy i więc	2	120	17,1429	0	17,1429
0	0	0	jeden czyn	2	840	120	30	90
0	0	1	dwa czynni	2	0	0	0	0
0	0	1	trzy i więc	2	60	8,57143	8,57143	0
0	1	0	dwa czynni	1	60	8,57143	0	8,57143

1	0	1	trzy i więce	2	60	8,57143	0	8,57143
0	0	0	jeden czyn	1	60	8,57143	0	8,57143
1	1	0	trzy i więce	3	360	51,4286	0	51,4286
0	0	0	jeden czyn	2	420	60	0	60
1	0	1	trzy i więce	2	10	1,42857	1,42857	0
0	0	1	trzy i więce	3	300	42,8571	0	42,8571
1	1	1	trzy i więce	3	120	17,1429	0	17,1429
0	0	1	dwa czynni	3	0	0	0	0
1	0	1	dwa czynni	2	220	31,4286	5,71429	25,7143
0	0	1	dwa czynni	1	300	42,8571	0	42,8571
0	1	1	trzy i więce	2	180	25,7143	0	25,7143
0	0	1	dwa czynni	2	0	0	0	0
0	0	1	jeden czyn	3	40	5,71429	0	5,71429
0	1	1	trzy i więce	2	180	25,7143	17,1429	8,57143
0	0	1	dwa czynni	3	0	0	0	0
0	0	1	trzy i więce	3	0	0	0	0
0	1	0	dwa czynni	2	140	20	20	0
0	1	1	trzy i więce	3	240	34,2857	0	34,2857
0	0	1	dwa czynni	3	60	8,57143	0	8,57143
1	0	1	trzy i więce	3	960	137,143	17,1429	120
0	1	1	trzy i więce	3	150	21,4286	0	21,4286
1	1	1	trzy i więce	3	480	68,5714	0	68,5714
1	0	1	trzy i więce	3	840	120	0	120
1	1	1	trzy i więce	2	240	34,2857	0	34,2857
0	0	1	dwa czynni	2	140	20	0	20
0	0	1	jeden czyn	2	60	8,57143	0	8,57143
0	0	0	jeden czyn	2	60	8,57143	0	8,57143
0	0	1	jeden czyn	3	0	0	0	0
1	1	0	trzy i więce	3	120	17,1429	0	17,1429
1	0	1	trzy i więce	2	90	12,8571	12,8571	0
0	0	0	jeden czyn	3	420	60	0	60
1	1	1	trzy i więce	1	280	40	0	40
1	1	1	trzy i więce	2	0	0	0	0
1	1	1	trzy i więce	2	0	0	0	0
1	0	1	trzy i więce	1	310	44,2857	14,2857	30
0	0	1	dwa czynni	2	60	8,57143	0	8,57143
0	0	1	dwa czynni	2	0	0	0	0
0	1	1	trzy i więce	1	240	34,2857	0	34,2857
0	1	1	trzy i więce	2	0	0	0	0
0	0	0	jeden czyn	2	0	0	0	0
1	0	1	trzy i więce	3	60	8,57143	0	8,57143
1	1	1	trzy i więce	2	0	0	0	0
1	0	1	trzy i więce	2	180	25,7143	0	25,7143
0	1	0	dwa czynni	3	0	0	0	0
0	0	1	dwa czynni	3	240	34,2857	0	34,2857
0	0	1	dwa czynni	2	60	8,57143	0	8,57143
0	1	1	trzy i więce	2	0	0	0	0
1	0	1	trzy i więce	2	0	0	0	0

0	1	1	trzy i więce	2	120	17,1429	0	17,1429
1	0	1	trzy i więce	2	300	42,8571	0	42,8571
0	0	1	trzy i więce	3	120	17,1429	0	17,1429
1	0	0	trzy i więce	2	0	0	0	0
1	0	0	dwa czynni	1	0	0	0	0
0	1	1	trzy i więce	1	0	0	0	0
0	0	0	jeden czyn	2	350	50	0	50
1	1	1	trzy i więce	1	1260	180	0	180
1	1	1	trzy i więce	1	0	0	0	0
1	0	1	trzy i więce	3	0	0	0	0
1	1	1	trzy i więce	2	175	25	0	25
0	1	1	trzy i więce	3	30	4,28571	4,28571	0
1	0	1	trzy i więce	3	120	17,1429	0	17,1429
0	0	1	dwa czynni	3	80	11,4286	2,85714	8,57143
1	0	1	trzy i więce	2	180	25,7143	0	25,7143
1	0	1	trzy i więce	3	270	38,5714	30	8,57143
1	1	1	trzy i więce	2	120	17,1429	0	17,1429
0	1	1	trzy i więce	2	120	17,1429	0	17,1429
1	0	0	dwa czynni	2	180	25,7143	0	25,7143
0	1	0	dwa czynni	2	15	2,14286	2,14286	0
1	0	0	dwa czynni	2	180	25,7143	0	25,7143
0	0	1	trzy i więce	2	540	77,1429	60	17,1429
0	0	1	jeden czyn	3	260	37,1429	20	17,1429
1	0	1	trzy i więce	3	375	53,5714	19,2857	34,2857
0	1	1	trzy i więce	2	255	36,4286	6,42857	30
1	0	1	trzy i więce	2	30	4,28571	4,28571	0
0	1	1	trzy i więce	2	465	66,4286	6,42857	60
0	0	0	jeden czyn	2	315	45	0	45
0	0	1	dwa czynni	2	0	0	0	0
0	1	1	trzy i więce	2	0	0	0	0
1	1	1	trzy i więce	2	150	21,4286	0	21,4286
1	1	1	trzy i więce	2	0	0	0	0
1	1	0	trzy i więce	2	0	0	0	0
0	1	0	dwa czynni	3	0	0	0	0
0	0	1	jeden czyn	1	180	25,7143	0	25,7143
0	1	1	trzy i więce	1	840	120	0	120
0	0	0	metabolicz	2	340	48,5714	14,2857	34,2857
1	0	1	trzy i więce	3	0	0	0	0
0	0	1	dwa czynni	3	240	34,2857	17,1429	17,1429
1	1	1	trzy i więce	1	0	0	0	0
1	0	1	trzy i więce	2	420	60	0	60
1	0	0	trzy i więce	1	420	60	0	60
1	0	1	dwa czynni	2	0	0	0	0
1	0	1	trzy i więce	2	180	25,7143	8,57143	17,1429
0	1	1	trzy i więce	3	150	21,4286	8,57143	12,8571
0	1	1	trzy i więce	3	0	0	0	0
1	1	1	trzy i więce	2	0	0	0	0
1	1	1	trzy i więce	2	0	0	0	0

0	0	0	metabolicz	1	600	85,7143	0	85,7143
1	0	1	trzy i więce	3	0	0	0	0
0	0	1	jeden czyn	2	180	25,7143	8,57143	17,1429
1	0	1	trzy i więce	2	0	0	0	0
0	0	0	jeden czyn	2	60	8,57143	0	8,57143
1	1	1	trzy i więce	2	180	25,7143	0	25,7143
0	1	1	trzy i więce	2	210	30	0	30
0	1	1	trzy i więce	3	0	0	0	0
0	0	1	jeden czyn	1	300	42,8571	0	42,8571
1	1	0	trzy i więce	3	420	60	0	60
1	0	1	trzy i więce	3	0	0	0	0
0	1	1	trzy i więce	2	120	17,1429	0	17,1429
1	1	1	trzy i więce	2	0	0	0	0
0	0	1	jeden czyn	3	120	17,1429	0	17,1429
0	0	1	dwa czynni	3	150	21,4286	0	21,4286
0	0	0	jeden czyn	2	180	25,7143	17,1429	8,57143
1	0	1	trzy i więce	2	280	40	0	40
1	0	1	trzy i więce	2	180	25,7143	25,7143	0
0	0	1	dwa czynni	1	840	120	0	120
0	0	0	jeden czyn	3	420	60	0	60
1	0	0	trzy i więce	3	0	0	0	0
1	0	0	dwa czynni	1	360	51,4286	0	51,4286
1	0	1	trzy i więce	3	120	17,1429	0	17,1429
1	0	1	trzy i więce	2	435	62,1429	10,7143	51,4286
0	0	0	metabolicz	2	0	0	0	0
0	1	1	trzy i więce	3	30	4,28571	0	4,28571
0	0	1	dwa czynni	2	120	17,1429	0	17,1429
0	1	1	trzy i więce	2	180	25,7143	25,7143	0
0	0	1	dwa czynni	3	270	38,5714	0	38,5714
1	0	1	trzy i więce	2	210	30	12,8571	17,1429
0	0	1	dwa czynni	2	0	0	0	0
0	1	0	jeden czyn	1	180	25,7143	0	25,7143
0	1	1	trzy i więce	2	60	8,57143	0	8,57143
0	1	1	trzy i więce	2	840	120	0	120
1	0	1	trzy i więce	1	0	0	0	0
0	1	1	trzy i więce	2	345	49,2857	15	34,2857
1	0	1	trzy i więce	2	255	36,4286	19,2857	17,1429
1	1	1	trzy i więce	3	0	0	0	0
0	0	1	dwa czynni	3	180	25,7143	25,7143	0
0	0	1	dwa czynni	2	60	8,57143	0	8,57143
0	0	1	dwa czynni	3	350	50	20	30
1	1	1	trzy i więce	2	0	0	0	0
0	1	1	trzy i więce	2	210	30	0	30
1	1	1	trzy i więce	2	200	28,5714	20	8,57143
0	0	1	dwa czynni	3	60	8,57143	0	8,57143
0	1	1	trzy i więce	2	180	25,7143	25,7143	0
1	1	1	trzy i więce	2	285	40,7143	32,1429	8,57143
0	1	1	trzy i więce	2	60	8,57143	0	8,57143

1	0	1	trzy i więce	2	0	0	0	0
0	0	0	jeden czyn	3	0	0	0	0
0	0	0	jeden czyn	2	420	60	30	30
0	1	1	trzy i więce	1	120	17,1429	0	17,1429
0	0	1	jeden czyn	3	255	36,4286	19,2857	17,1429
0	1	1	trzy i więce	2	120	17,1429	0	17,1429
0	0	1	dwa czynni	2	480	68,5714	68,5714	0
0	1	1	trzy i więce	1	1080	154,286	0	154,286
1	0	1	trzy i więce	2	1200	171,429	51,4286	120
1	0	1	trzy i więce	2	1020	145,714	120	25,7143
0	1	1	dwa czynni	1	180	25,7143	25,7143	0
1	1	1	trzy i więce	2	30	4,28571	0	4,28571
1	0	1	trzy i więce	2	90	12,8571	0	12,8571
0	0	1	dwa czynni	2	175	25	2,14286	22,8571
0	1	0	trzy i więce	2	0	0	0	0
0	0	1	dwa czynni	2	120	17,1429	0	17,1429
0	0	1	dwa czynni	3	150	21,4286	21,4286	0
0	0	1	jeden czyn	2	240	34,2857	8,57143	25,7143
1	1	1	trzy i więce	3	10	1,42857	1,42857	0
1	0	1	trzy i więce	2	40	5,71429	5,71429	0
0	0	1	trzy i więce	2	0	0	0	0
1	0	1	trzy i więce	3	0	0	0	0
1	1	1	trzy i więce	2	420	60	8,57143	51,4286
0	1	1	trzy i więce	3	330	47,1429	30	17,1429
0	0	1	dwa czynni	2	560	80	30	50
1	1	1	trzy i więce	2	0	0	0	0
1	0	0	dwa czynni	2	0	0	0	0
0	0	0	jeden czyn	2	0	0	0	0
1	0	0	trzy i więce	2	315	45	10,7143	34,2857
0	0	1	dwa czynni	2	60	8,57143	0	8,57143
0	1	1	trzy i więce	3	0	0	0	0
0	0	1	jeden czyn	1	0	0	0	0
0	0	1	jeden czyn	3	0	0	0	0
1	0	0	trzy i więce	1	735	105	45	60
0	1	1	trzy i więce	2	60	8,57143	8,57143	0
0	0	0	metabolicz	2	150	21,4286	0	21,4286
0	0	1	dwa czynni	3	300	42,8571	0	42,8571
1	0	1	trzy i więce	2	0	0	0	0
0	0	0	jeden czyn	1	1260	180	0	180
1	1	1	trzy i więce	2	0	0	0	0
1	1	1	trzy i więce	1	240	34,2857	0	34,2857
0	1	1	trzy i więce	3	60	8,57143	0	8,57143
0	1	1	trzy i więce	3	300	42,8571	0	42,8571
1	0	1	trzy i więce	2	0	0	0	0
1	0	1	trzy i więce	1	60	8,57143	0	8,57143
0	0	1	dwa czynni	1	1260	180	0	180
0	0	0	metabolicz	1	1260	180	0	180
0	1	0	dwa czynni	2	0	0	0	0

0	0	0	jeden czyn	2	120	17,1429	17,1429	0
0	0	0	jeden czyn	2	45	6,42857	6,42857	0
0	1	1	trzy i więce	3	120	17,1429	0	17,1429
1	1	1	trzy i więce	1	410	58,5714	7,14286	51,4286
0	0	1	dwa czynni	2	100	14,2857	0	14,2857
0	1	1	trzy i więce	3	300	42,8571	42,8571	0
0	0	0	metabolicz	1	180	25,7143	0	25,7143
1	0	0	trzy i więce	3	420	60	0	60
1	1	1	trzy i więce	3	30	4,28571	0	4,28571
0	0	1	dwa czynni	2	420	60	0	60
0	1	1	trzy i więce	3	720	102,857	0	102,857
0	0	1	dwa czynni	3	0	0	0	0
0	1	1	trzy i więce	2	0	0	0	0
1	1	1	trzy i więce	3	0	0	0	0
1	0	0	dwa czynni	1	0	0	0	0
1	0	1	trzy i więce	1	420	60	0	60
1	0	1	trzy i więce	2	150	21,4286	12,8571	8,57143
1	0	1	trzy i więce	3	140	20	20	0
0	0	0	metabolicz	2	375	53,5714	27,8571	25,7143
1	1	1	trzy i więce	3	0	0	0	0
1	0	1	trzy i więce	2	180	25,7143	8,57143	17,1429
0	0	0	jeden czyn	2	0	0	0	0
0	1	1	trzy i więce	2	0	0	0	0
1	0	0	dwa czynni	1	420	60	8,57143	51,4286
1	0	0	trzy i więce	3	1425	203,571	23,5714	180
0	0	0	metabolicz	2	90	12,8571	12,8571	0
1	0	1	trzy i więce	2	420	60	34,2857	25,7143
1	0	1	trzy i więce	3	180	25,7143	0	25,7143
1	0	1	trzy i więce	3	420	60	34,2857	25,7143
0	0	1	trzy i więce	1	0	0	0	0
0	1	1	trzy i więce	3	240	34,2857	0	34,2857
0	1	1	trzy i więce	2	60	8,57143	0	8,57143
1	1	1	trzy i więce	1	0	0	0	0
0	0	1	dwa czynni	3	30	4,28571	0	4,28571
0	0	0	metabolicz	3	60	8,57143	0	8,57143
0	0	1	dwa czynni	3	0	0	0	0
0	0	1	dwa czynni	2	700	100	40	60
1	0	1	trzy i więce	2	0	0	0	0
0	0	0	metabolicz	1	0	0	0	0
1	1	1	trzy i więce	2	240	34,2857	0	34,2857
0	0	1	dwa czynni	3	180	25,7143	8,57143	17,1429
0	1	1	trzy i więce	2	540	77,1429	8,57143	68,5714
0	0	1	dwa czynni	2	600	85,7143	0	85,7143
1	1	1	trzy i więce	2	0	0	0	0
1	1	1	trzy i więce	2	120	17,1429	0	17,1429
1	0	1	trzy i więce	2	0	0	0	0
1	0	1	trzy i więce	2	780	111,429	68,5714	42,8571
0	1	1	trzy i więce	2	0	0	0	0

0	1	1	dwa czynni	3	255	36,4286	15	21,4286
1	0	1	trzy i więce	2	240	34,2857	8,57143	25,7143
1	0	0	trzy i więce	2	0	0	0	0
0	0	1	dwa czynni	3	420	60	0	60
0	1	1	trzy i więce	2	1380	197,143	17,1429	180
0	1	1	trzy i więce	2	120	17,1429	8,57143	8,57143
0	1	1	trzy i więce	3	60	8,57143	0	8,57143
0	0	1	dwa czynni	3	850	121,429	1,42857	120
0	0	0	jeden czyn	3	1260	180	120	60
1	1	1	trzy i więce	1	0	0	0	0
1	0	1	trzy i więce	3	0	0	0	0
0	0	0	metabolicz	3	0	0	0	0
0	1	1	trzy i więce	3	360	51,4286	0	51,4286
1	0	0	dwa czynni	3	240	34,2857	34,2857	0
1	1	1	trzy i więce	1	0	0	0	0
1	0	1	trzy i więce	2	420	60	0	60
1	1	1	trzy i więce	2	60	8,57143	0	8,57143
1	0	1	trzy i więce	3	120	17,1429	0	17,1429
0	0	0	jeden czyn	2	80	11,4286	0	11,4286
0	1	1	trzy i więce	3	720	102,857	0	102,857
1	1	1	trzy i więce	3	0	0	0	0
0	1	1	trzy i więce	2	0	0	0	0
0	1	1	trzy i więce	2	0	0	0	0
0	0	1	jeden czyn	1	200	28,5714	17,1429	11,4286
0	1	1	trzy i więce	3	840	120	0	120
0	1	1	trzy i więce	3	160	22,8571	5,71429	17,1429
1	1	1	trzy i więce	2	0	0	0	0
0	0	1	dwa czynni	2	720	102,857	17,1429	85,7143
1	0	1	trzy i więce	2	120	17,1429	0	17,1429
0	0	1	dwa czynni	2	0	0	0	0
0	0	1	dwa czynni	3	60	8,57143	0	8,57143
0	1	1	dwa czynni	3	270	38,5714	38,5714	0
1	1	0	dwa czynni	3	75	10,7143	2,14286	8,57143
0	0	1	trzy i więce	3	120	17,1429	17,1429	0
0	0	1	dwa czynni	2	0	0	0	0
0	0	1	dwa czynni	3	350	50	20	30
0	0	0	jeden czyn	3	135	19,2857	6,42857	12,8571
1	0	1	trzy i więce	2	120	17,1429	0	17,1429
1	0	0	dwa czynni	3	120	17,1429	17,1429	0
1	1	1	trzy i więce	2	120	17,1429	0	17,1429
0	0	1	dwa czynni	2	0	0	0	0
0	1	1	trzy i więce	2	310	44,2857	4,28571	40
0	0	1	dwa czynni	1	420	60	0	60
0	1	1	trzy i więce	2	90	12,8571	12,8571	0
1	1	1	trzy i więce	2	120	17,1429	0	17,1429
0	0	1	dwa czynni	2	60	8,57143	0	8,57143
1	0	1	trzy i więce	2	250	35,7143	10	25,7143
0	1	1	trzy i więce	2	120	17,1429	0	17,1429

1	1	1	trzy i więc	2	0	0	0	0
0	0	1	jeden czyn	3	180	25,7143	0	25,7143
0	1	1	trzy i więc	1	210	30	0	30
0	0	0	jeden czyn	2	600	85,7143	0	85,7143
0	0	1	trzy i więc	2	40	5,71429	0	5,71429
0	0	0	jeden czyn	1	120	17,1429	0	17,1429
0	0	1	jeden czyn	2	60	8,57143	0	8,57143
0	0	1	dwa czynni	2	90	12,8571	0	12,8571
0	0	1	dwa czynni	3	0	0	0	0
0	0	1	dwa czynni	2	90	12,8571	0	12,8571
1	1	1	trzy i więc	2	360	51,4286	0	51,4286
1	1	1	trzy i więc	2	0	0	0	0
0	0	0	jeden czyn	2	60	8,57143	0	8,57143
1	0	1	trzy i więc	2	0	0	0	0
0	0	1	dwa czynni	2	120	17,1429	0	17,1429
0	0	1	jeden czyn	1	460	65,7143	40	25,7143
0	1	1	dwa czynni	3	250	35,7143	5,71429	30
0	0	1	dwa czynni	1	0	0	0	0
1	0	1	trzy i więc	3	120	17,1429	0	17,1429
1	0	1	trzy i więc	3	720	102,857	0	102,857
1	1	1	trzy i więc	3	60	8,57143	0	8,57143
1	1	1	trzy i więc	3	0	0	0	0
1	1	1	trzy i więc	2	350	50	20	30
1	1	0	trzy i więc	2	60	8,57143	0	8,57143
0	1	1	trzy i więc	2	975	139,286	19,2857	120
0	0	1	dwa czynni	2	0	0	0	0
0	0	1	dwa czynni	2	0	0	0	0
1	0	1	trzy i więc	2	120	17,1429	17,1429	0
0	1	1	trzy i więc	2	90	12,8571	0	12,8571
1	1	1	trzy i więc	2	420	60	0	60
0	0	1	dwa czynni	3	180	25,7143	0	25,7143
0	0	1	trzy i więc	3	120	17,1429	0	17,1429
0	1	1	trzy i więc	2	0	0	0	0
1	1	1	trzy i więc	2	360	51,4286	0	51,4286
0	1	1	trzy i więc	3	20	2,85714	0	2,85714
0	1	1	trzy i więc	3	420	60	0	60
1	1	1	trzy i więc	3	0	0	0	0
0	0	1	dwa czynni	2	480	68,5714	8,57143	60
0	1	1	trzy i więc	2	0	0	0	0
1	0	1	trzy i więc	3	840	120	0	120
0	1	1	trzy i więc	3	0	0	0	0
0	1	1	trzy i więc	2	240	34,2857	0	34,2857
0	0	1	trzy i więc	2	420	60	0	60
1	0	1	trzy i więc	2	735	105	15	90
0	0	1	dwa czynni	2	60	8,57143	0	8,57143
0	0	1	dwa czynni	2	120	17,1429	0	17,1429
0	0	1	dwa czynni	3	120	17,1429	17,1429	0
1	1	1	trzy i więc	1	0	0	0	0

1	0	1	trzy i więce	2	0	0	0	0
0	0	1	dwa czynni	3	200	28,5714	25,7143	2,85714
0	0	1	dwa czynni	3	0	0	0	0
0	0	1	dwa czynni	3	360	51,4286	17,1429	34,2857
1	1	1	trzy i więce	2	360	51,4286	0	51,4286
0	0	1	trzy i więce	1	420	60	0	60
0	0	1	dwa czynni	1	0	0	0	0
0	0	0	metabolicz	2	700	100	14,2857	85,7143
0	0	0	metabolicz	2	120	17,1429	8,57143	8,57143
0	0	1	dwa czynni	3	285	40,7143	6,42857	34,2857
0	0	1	trzy i więce	2	60	8,57143	0	8,57143
1	1	1	trzy i więce	2	140	20	20	0
0	0	1	dwa czynni	3	240	34,2857	34,2857	0
0	0	0	jeden czyn	2	0	0	0	0
1	0	1	trzy i więce	2	315	45	15	30
0	1	1	trzy i więce	2	0	0	0	0
1	0	1	trzy i więce	2	0	0	0	0
0	0	1	dwa czynni	2	0	0	0	0
0	0	1	dwa czynni	2	520	74,2857	5,71429	68,5714
1	0	1	trzy i więce	2	90	12,8571	0	12,8571
0	1	1	trzy i więce	2	120	17,1429	0	17,1429
0	0	0	metabolicz	2	360	51,4286	0	51,4286
0	1	1	trzy i więce	2	0	0	0	0
1	0	1	trzy i więce	2	0	0	0	0
1	0	0	dwa czynni	2	360	51,4286	0	51,4286
0	0	1	dwa czynni	2	180	25,7143	25,7143	0
0	0	0	metabolicz	2	45	6,42857	2,14286	4,28571
0	0	1	dwa czynni	2	60	8,57143	0	8,57143
1	1	1	trzy i więce	2	60	8,57143	8,57143	0
0	0	0	metabolicz	1	180	25,7143	25,7143	0
0	0	1	dwa czynni	2	0	0	0	0
0	1	0	dwa czynni	2	180	25,7143	8,57143	17,1429
0	0	0	jeden czyn	2	0	0	0	0
1	0	1	trzy i więce	1	0	0	0	0
1	0	1	trzy i więce	2	190	27,1429	21,4286	5,71429
0	0	0	jeden czyn	2	0	0	0	0
0	0	1	dwa czynni	2	180	25,7143	0	25,7143
0	0	0	jeden czyn	2	240	34,2857	0	34,2857
0	0	1	dwa czynni	3	375	53,5714	8,57143	45
0	1	1	trzy i więce	1	120	17,1429	8,57143	8,57143
0	0	1	dwa czynni	2	450	64,2857	4,28571	60
0	0	1	dwa czynni	1	210	30	12,8571	17,1429
1	1	1	trzy i więce	3	660	94,2857	34,2857	60
1	0	1	trzy i więce	2	0	0	0	0
1	0	1	trzy i więce	3	120	17,1429	0	17,1429
1	0	1	trzy i więce	2	0	0	0	0
0	0	1	dwa czynni	3	15	2,14286	2,14286	0
1	1	1	trzy i więce	2	255	36,4286	10,7143	25,7143

0	0	1	dwa czynni	1	60	8,57143	0	8,57143
0	0	1	jeden czyn	1	0	0	0	0
0	0	0	jeden czyn	2	30	4,28571	0	4,28571
1	1	1	trzy i więc	2	0	0	0	0
0	0	0	jeden czyn	2	360	51,4286	21,4286	30
0	1	1	dwa czynni	3	1260	180	0	180
1	1	1	trzy i więc	2	0	0	0	0
0	0	1	dwa czynni	2	0	0	0	0
0	0	1	jeden czyn	2	60	8,57143	8,57143	0
0	1	1	trzy i więc	3	280	40	0	40
0	0	1	dwa czynni	2	0	0	0	0
0	1	1	trzy i więc	2	60	8,57143	0	8,57143
0	0	1	dwa czynni	3	0	0	0	0
0	1	1	trzy i więc	2	0	0	0	0
1	0	1	trzy i więc	3	360	51,4286	0	51,4286
1	0	1	trzy i więc	2	840	120	0	120
1	0	1	trzy i więc	2	240	34,2857	34,2857	0
1	0	0	trzy i więc	2	60	8,57143	0	8,57143
1	1	1	trzy i więc	2	0	0	0	0
0	0	1	jeden czyn	2	420	60	0	60
0	1	1	trzy i więc	2	0	0	0	0
0	0	1	jeden czyn	2	120	17,1429	0	17,1429
1	0	1	trzy i więc	2	0	0	0	0
1	0	1	trzy i więc	1	930	132,857	12,8571	120
0	1	1	trzy i więc	2	60	8,57143	0	8,57143
0	0	1	jeden czyn	2	360	51,4286	17,1429	34,2857
0	0	0	metabolicz	2	120	17,1429	17,1429	0
0	0	1	dwa czynni	2	0	0	0	0
1	0	1	trzy i więc	2	60	8,57143	0	8,57143
0	1	1	dwa czynni	2	240	34,2857	30	4,28571
0	0	0	jeden czyn	3	610	87,1429	51,4286	35,7143
0	1	1	trzy i więc	3	630	90	30	60
1	1	1	trzy i więc	3	960	137,143	17,1429	120
0	1	1	dwa czynni	3	360	51,4286	40	11,4286
0	0	1	dwa czynni	3	720	102,857	34,2857	68,5714
0	0	0	jeden czyn	2	0	0	0	0
1	1	1	trzy i więc	2	840	120	0	120
1	0	1	trzy i więc	2	150	21,4286	12,8571	8,57143
1	1	1	trzy i więc	2	60	8,57143	8,57143	0
0	0	1	jeden czyn	3	360	51,4286	0	51,4286
0	1	1	trzy i więc	3	900	128,571	25,7143	102,857
0	0	1	jeden czyn	1	0	0	0	0
0	1	1	trzy i więc	2	60	8,57143	0	8,57143
0	0	1	jeden czyn	2	60	8,57143	0	8,57143
0	1	0	dwa czynni	1	1080	154,286	0	154,286
1	0	0	jeden czyn	1	120	17,1429	0	17,1429
0	0	1	dwa czynni	2	150	21,4286	12,8571	8,57143
0	1	1	trzy i więc	2	720	102,857	0	102,857

0	0	1	jeden czyn	2	420	60	0	60
0	0	0	dwa czynni	2	180	25,7143	0	25,7143
0	0	1	jeden czyn	2	450	64,2857	30	34,2857
1	0	1	trzy i więce	3	300	42,8571	17,1429	25,7143
1	0	0	trzy i więce	2	30	4,28571	4,28571	0
1	0	1	trzy i więce	3	40	5,71429	0	5,71429
0	0	1	dwa czynni	2	90	12,8571	0	12,8571
0	0	0	metabolicz	1	30	4,28571	0	4,28571
1	1	1	trzy i więce	1	210	30	15	15
0	1	1	trzy i więce	1	0	0	0	0
1	1	0	trzy i więce	2	300	42,8571	0	42,8571
0	1	1	trzy i więce	2	0	0	0	0
0	0	0	metabolicz	3	360	51,4286	0	51,4286
0	0	1	dwa czynni	2	180	25,7143	25,7143	0
1	0	0	dwa czynni	2	0	0	0	0
0	0	1	trzy i więce	1	310	44,2857	10	34,2857
0	0	1	trzy i więce	3	420	60	0	60
0	0	1	jeden czyn	2	120	17,1429	0	17,1429
0	0	1	dwa czynni	2	60	8,57143	6,42857	2,14286
0	0	1	dwa czynni	3	120	17,1429	0	17,1429
0	0	0	jeden czyn	2	60	8,57143	0	8,57143
0	0	1	trzy i więce	3	630	90	0	90
0	0	0	metabolicz	3	315	45	19,2857	25,7143
0	0	0	jeden czyn	2	75	10,7143	10,7143	0
0	0	1	dwa czynni	3	0	0	0	0
1	0	1	trzy i więce	3	90	12,8571	0	12,8571
0	0	0	jeden czyn	2	0	0	0	0
0	0	0	jeden czyn	3	180	25,7143	25,7143	0
1	1	1	trzy i więce	1	360	51,4286	0	51,4286
0	0	0	jeden czyn	2	120	17,1429	0	17,1429
1	0	1	trzy i więce	2	40	5,71429	0	5,71429
0	0	0	jeden czyn	2	540	77,1429	17,1429	60
0	0	1	dwa czynni	2	0	0	0	0
1	0	1	trzy i więce	2	0	0	0	0
1	0	1	trzy i więce	3	180	25,7143	0	25,7143
0	1	1	trzy i więce	2	0	0	0	0
0	0	1	dwa czynni	2	150	21,4286	0	21,4286
1	1	1	trzy i więce	2	315	45	0	45
0	0	1	dwa czynni	3	360	51,4286	0	51,4286
0	1	0	dwa czynni	2	0	0	0	0
0	1	1	trzy i więce	2	45	6,42857	6,42857	0
0	1	1	trzy i więce	2	720	102,857	0	102,857
1	1	1	trzy i więce	2	60	8,57143	0	8,57143
0	0	1	dwa czynni	2	60	8,57143	0	8,57143
0	0	0	metabolicz	2	80	11,4286	2,85714	8,57143
1	1	1	trzy i więce	2	300	42,8571	0	42,8571
1	0	1	trzy i więce	3	0	0	0	0
1	1	1	trzy i więce	3	210	30	12,8571	17,1429

0	0	0	metabolicz	2	300	42,8571	0	42,8571
1	0	0	trzy i więce	1	60	8,57143	0	8,57143
1	1	1	trzy i więce	2	240	34,2857	0	34,2857
1	0	1	trzy i więce	2	0	0	0	0
0	1	1	trzy i więce	1	420	60	0	60
1	1	1	trzy i więce	3	105	15	15	0
0	0	1	trzy i więce	3	285	40,7143	15	25,7143
0	0	1	jeden czyn	2	420	60	0	60
0	0	1	dwa czynni	3	0	0	0	0
1	0	1	trzy i więce	2	45	6,42857	6,42857	0
1	0	1	dwa czynni	2	840	120	0	120
1	0	0	dwa czynni	2	0	0	0	0
1	1	1	trzy i więce	2	0	0	0	0
0	0	0	jeden czyn	3	0	0	0	0
0	0	1	dwa czynni	3	90	12,8571	0	12,8571
0	0	1	dwa czynni	2	225	32,1429	19,2857	12,8571
0	1	0	trzy i więce	3	240	34,2857	0	34,2857
0	0	1	dwa czynni	3	360	51,4286	17,1429	34,2857
1	1	1	trzy i więce	3	300	42,8571	0	42,8571
0	0	1	dwa czynni	2	0	0	0	0
1	0	0	jeden czyn	3	0	0	0	0
1	0	1	trzy i więce	2	40	5,71429	0	5,71429
0	0	1	trzy i więce	3	80	11,4286	2,85714	8,57143
0	0	1	trzy i więce	2	0	0	0	0
1	1	1	trzy i więce	2	120	17,1429	0	17,1429
0	0	1	dwa czynni	3	60	8,57143	0	8,57143
1	0	0	trzy i więce	2	230	32,8571	20	12,8571
1	1	1	trzy i więce	3	60	8,57143	0	8,57143
0	0	0	metabolicz	3	420	60	0	60
0	1	1	trzy i więce	2	0	0	0	0
0	0	1	jeden czyn	2	600	85,7143	0	85,7143
1	1	1	trzy i więce	3	130	18,5714	10	8,57143
0	1	1	dwa czynni	2	120	17,1429	0	17,1429
0	0	1	dwa czynni	2	0	0	0	0
0	0	0	jeden czyn	2	840	120	0	120
0	0	1	jeden czyn	3	540	77,1429	17,1429	60
0	1	0	dwa czynni	3	0	0	0	0
1	1	1	trzy i więce	2	15	2,14286	0	2,14286
0	0	0	jeden czyn	2	465	66,4286	6,42857	60
1	1	1	trzy i więce	2	0	0	0	0
1	0	1	trzy i więce	2	840	120	0	120
1	0	1	dwa czynni	3	240	34,2857	17,1429	17,1429
1	0	1	trzy i więce	3	420	60	0	60
0	0	1	dwa czynni	3	0	0	0	0
0	0	1	dwa czynni	2	540	77,1429	17,1429	60
0	1	1	trzy i więce	2	210	30	30	0
1	1	1	trzy i więce	3	510	72,8571	12,8571	60
1	1	1	trzy i więce	2	120	17,1429	0	17,1429

1	0	1	trzy i więce	2	900	128,571	0	128,571
0	0	0	jeden czyn	2	90	12,8571	0	12,8571
0	0	1	jeden czyn	1	1110	158,571	128,571	30
0	1	1	trzy i więce	1	60	8,57143	0	8,57143
0	0	1	dwa czynni	2	0	0	0	0
1	0	1	trzy i więce	2	120	17,1429	0	17,1429
1	1	0	trzy i więce	1	210	30	0	30
0	0	1	jeden czyn	2	120	17,1429	0	17,1429
1	0	1	trzy i więce	3	120	17,1429	0	17,1429
1	0	1	trzy i więce	2	0	0	0	0
1	1	1	trzy i więce	1	60	8,57143	8,57143	0
0	1	1	trzy i więce	3	0	0	0	0
0	0	1	dwa czynni	3	600	85,7143	25,7143	60
0	0	0	jeden czyn	2	0	0	0	0
1	0	1	trzy i więce	1	0	0	0	0
0	0	1	jeden czyn	1	120	17,1429	0	17,1429
1	0	1	trzy i więce	1	90	12,8571	0	12,8571
1	0	1	trzy i więce	2	0	0	0	0
0	0	1	dwa czynni	2	0	0	0	0
0	0	1	dwa czynni	3	240	34,2857	0	34,2857
0	0	1	dwa czynni	3	210	30	0	30
0	1	1	trzy i więce	2	225	32,1429	12,8571	19,2857
0	0	1	dwa czynni	2	120	17,1429	17,1429	0
0	1	1	trzy i więce	2	660	94,2857	34,2857	60
1	0	0	trzy i więce	2	120	17,1429	0	17,1429
1	0	0	trzy i więce	1	140	20	0	20
0	0	1	jeden czyn	2	240	34,2857	17,1429	17,1429
1	1	1	trzy i więce	2	75	10,7143	0	10,7143
0	0	1	dwa czynni	2	240	34,2857	0	34,2857
1	0	1	trzy i więce	2	0	0	0	0
0	0	1	trzy i więce	2	300	42,8571	0	42,8571
1	0	1	trzy i więce	3	240	34,2857	0	34,2857
0	1	1	dwa czynni	3	20	2,85714	0	2,85714
0	1	1	trzy i więce	1	0	0	0	0
0	0	1	dwa czynni	2	0	0	0	0
0	0	0	jeden czyn	2	60	8,57143	0	8,57143
1	0	1	trzy i więce	3	900	128,571	0	128,571
0	0	0	jeden czyn	2	0	0	0	0
1	1	1	trzy i więce	1	390	55,7143	38,5714	17,1429
1	0	0	trzy i więce	2	300	42,8571	0	42,8571
1	0	1	trzy i więce	2	360	51,4286	0	51,4286
0	0	1	dwa czynni	2	0	0	0	0
1	0	1	trzy i więce	3	420	60	0	60
0	0	0	jeden czyn	1	0	0	0	0
1	0	1	trzy i więce	2	270	38,5714	8,57143	30
0	1	1	trzy i więce	2	240	34,2857	0	34,2857
0	0	0	metabolicz	3	180	25,7143	0	25,7143
1	1	1	trzy i więce	1	0	0	0	0

0	1	1	trzy i więce	2	310	44,2857	38,5714	5,71429
1	0	1	trzy i więce	1	840	120	0	120
0	0	0	metabolicz	2	840	120	0	120
0	0	1	dwa czynni	2	420	60	0	60
0	1	0	jeden czyn	2	1260	180	0	180
1	0	1	trzy i więce	2	600	85,7143	25,7143	60
0	0	1	jeden czyn	1	840	120	0	120
1	1	1	trzy i więce	3	0	0	0	0
0	0	1	dwa czynni	2	0	0	0	0
1	1	1	trzy i więce	3	60	8,57143	0	8,57143
1	0	1	trzy i więce	2	0	0	0	0
0	0	1	dwa czynni	2	90	12,8571	0	12,8571
0	0	1	dwa czynni	2	100	14,2857	0	14,2857
0	0	1	dwa czynni	2	840	120	120	0
0	1	1	trzy i więce	2	105	15	15	0
1	0	1	trzy i więce	3	280	40	0	40
0	0	1	dwa czynni	2	420	60	0	60
1	0	1	trzy i więce	1	165	23,5714	6,42857	17,1429
0	1	1	trzy i więce	2	200	28,5714	0	28,5714
0	0	1	trzy i więce	2	0	0	0	0
0	0	1	dwa czynni	2	0	0	0	0
1	0	1	trzy i więce	1	0	0	0	0
0	0	1	dwa czynni	3	130	18,5714	14,2857	4,28571
0	0	1	dwa czynni	3	300	42,8571	30	12,8571
0	1	1	trzy i więce	1	0	0	0	0
0	1	0	dwa czynni	1	720	102,857	42,8571	60
0	1	1	trzy i więce	2	900	128,571	8,57143	120
0	0	1	jeden czyn	2	180	25,7143	0	25,7143
1	0	1	trzy i więce	2	420	60	8,57143	51,4286
1	1	1	trzy i więce	2	0	0	0	0
1	0	0	trzy i więce	2	420	60	0	60
0	1	1	trzy i więce	3	240	34,2857	0	34,2857
1	1	1	trzy i więce	3	0	0	0	0
0	0	1	dwa czynni	2	210	30	0	30
1	0	1	trzy i więce	2	0	0	0	0
1	1	1	trzy i więce	3	0	0	0	0
0	0	1	jeden czyn	3	300	42,8571	0	42,8571
1	0	1	trzy i więce	2	0	0	0	0
1	1	1	trzy i więce	2	285	40,7143	15	25,7143
0	1	0	jeden czyn	3	320	45,7143	20	25,7143
1	0	1	trzy i więce	2	390	55,7143	25,7143	30
1	0	1	trzy i więce	2	135	19,2857	2,14286	17,1429
0	0	1	jeden czyn	2	240	34,2857	0	34,2857
1	1	1	trzy i więce	3	0	0	0	0
0	1	1	trzy i więce	2	360	51,4286	0	51,4286
0	1	0	jeden czyn	2	0	0	0	0
1	0	0	dwa czynni	2	60	8,57143	0	8,57143
1	0	1	trzy i więce	2	120	17,1429	0	17,1429

0	0	0	jeden czyn	2	140	20	0	20
0	0	0	jeden czyn	2	50	7,14286	0	7,14286
0	0	1	dwa czynni	1	120	17,1429	17,1429	0
0	0	1	dwa czynni	3	300	42,8571	34,2857	8,57143
1	0	1	trzy i więc	2	120	17,1429	0	17,1429
0	0	1	trzy i więc	2	0	0	0	0
1	1	1	trzy i więc	2	0	0	0	0
1	0	1	trzy i więc	3	0	0	0	0
0	1	0	dwa czynni	1	90	12,8571	0	12,8571
0	0	0	jeden czyn	2	0	0	0	0
0	0	1	dwa czynni	3	200	28,5714	0	28,5714
0	1	1	trzy i więc	2	110	15,7143	4,28571	11,4286
0	1	0	trzy i więc	2	360	51,4286	51,4286	0
0	0	0	jeden czyn	2	240	34,2857	0	34,2857
0	0	0	jeden czyn	1	630	90	77,1429	12,8571
0	0	1	dwa czynni	2	840	120	0	120
0	0	1	trzy i więc	2	0	0	0	0
0	1	1	trzy i więc	1	0	0	0	0
0	1	1	trzy i więc	3	270	38,5714	12,8571	25,7143
0	0	1	dwa czynni	2	120	17,1429	0	17,1429
1	0	1	trzy i więc	2	45	6,42857	6,42857	0
0	1	1	trzy i więc	1	600	85,7143	0	85,7143
0	0	1	jeden czyn	3	240	34,2857	0	34,2857
0	1	1	trzy i więc	3	540	77,1429	0	77,1429
0	0	1	trzy i więc	2	0	0	0	0
0	1	1	trzy i więc	3	630	90	0	90
0	0	1	jeden czyn	2	0	0	0	0
0	1	1	trzy i więc	2	120	17,1429	0	17,1429
0	0	1	dwa czynni	3	0	0	0	0
0	0	0	jeden czyn	1	0	0	0	0
1	1	1	trzy i więc	2	0	0	0	0
0	1	1	trzy i więc	2	180	25,7143	0	25,7143
0	0	1	dwa czynni	2	30	4,28571	0	4,28571
1	1	1	trzy i więc	3	60	8,57143	0	8,57143
1	1	0	trzy i więc	2	780	111,429	25,7143	85,7143
1	1	1	trzy i więc	3	180	25,7143	25,7143	0
0	0	0	metabolicz	3	180	25,7143	0	25,7143
0	0	1	jeden czyn	3	0	0	0	0
0	0	0	jeden czyn	2	60	8,57143	0	8,57143
0	0	1	jeden czyn	1	0	0	0	0
0	0	0	jeden czyn	2	0	0	0	0
0	1	0	dwa czynni	3	840	120	0	120
1	1	1	trzy i więc	2	1080	154,286	0	154,286
1	0	0	trzy i więc	1	510	72,8571	12,8571	60
0	0	0	jeden czyn	2	330	47,1429	12,8571	34,2857
0	0	1	jeden czyn	1	0	0	0	0
0	0	1	jeden czyn	3	240	34,2857	0	34,2857
1	0	1	trzy i więc	2	60	8,57143	0	8,57143

0	1	1	trzy i więce	2	0	0	0	0
0	1	1	trzy i więce	1	0	0	0	0
0	0	0	jeden czyn	2	0	0	0	0
0	0	1	dwa czynni	3	960	137,143	17,1429	120
0	0	1	dwa czynni	3	120	17,1429	0	17,1429
1	1	1	trzy i więce	3	420	60	0	60
0	0	1	dwa czynni	3	275	39,2857	19,2857	20
0	0	1	dwa czynni	2	300	42,8571	0	42,8571
1	1	1	trzy i więce	3	120	17,1429	0	17,1429
1	0	0	trzy i więce	2	210	30	0	30
0	0	1	jeden czyn	2	0	0	0	0
1	1	1	trzy i więce	2	0	0	0	0
1	0	1	dwa czynni	2	1470	210	30	180
0	0	1	jeden czyn	3	45	6,42857	6,42857	0
0	0	0	metabolicz	2	60	8,57143	0	8,57143
1	1	1	trzy i więce	3	680	97,1429	80	17,1429
0	0	1	dwa czynni	2	0	0	0	0
1	1	1	trzy i więce	2	240	34,2857	0	34,2857
0	0	1	jeden czyn	3	0	0	0	0
0	0	1	jeden czyn	2	540	77,1429	0	77,1429
0	0	1	dwa czynni	3	0	0	0	0
0	0	1	trzy i więce	3	120	17,1429	0	17,1429
0	0	1	trzy i więce	2	420	60	0	60
1	0	0	dwa czynni	1	0	0	0	0
1	1	1	trzy i więce	1	0	0	0	0
1	0	0	dwa czynni	2	90	12,8571	12,8571	0
0	0	1	dwa czynni	2	0	0	0	0
0	0	1	trzy i więce	3	0	0	0	0
1	1	1	trzy i więce	3	60	8,57143	8,57143	0
1	0	1	trzy i więce	2	0	0	0	0
0	0	0	jeden czyn	2	525	75	15	60
1	0	1	trzy i więce	3	875	125	5	120
1	0	0	trzy i więce	2	120	17,1429	0	17,1429
0	0	1	dwa czynni	2	0	0	0	0
1	0	1	trzy i więce	2	60	8,57143	0	8,57143
0	0	1	jeden czyn	2	180	25,7143	0	25,7143
0	0	1	jeden czyn	2	90	12,8571	0	12,8571
0	1	1	dwa czynni	2	225	32,1429	15	17,1429
1	0	0	dwa czynni	2	780	111,429	42,8571	68,5714
0	0	1	jeden czyn	3	420	60	0	60
0	0	0	jeden czyn	2	420	60	0	60
0	1	1	trzy i więce	2	0	0	0	0
1	0	1	trzy i więce	2	60	8,57143	0	8,57143
0	1	1	trzy i więce	1	180	25,7143	0	25,7143
1	0	1	trzy i więce	2	1800	257,143	77,1429	180
0	0	1	trzy i więce	3	240	34,2857	17,1429	17,1429
1	0	1	trzy i więce	3	160	22,8571	17,1429	5,71429
1	1	1	trzy i więce	2	510	72,8571	12,8571	60

1	1	1	trzy i więce	2	280	40	25,7143	14,2857
1	1	1	trzy i więce	2	0	0	0	0
0	0	0	jeden czyn	2	420	60	0	60
1	1	1	trzy i więce	2	0	0	0	0
1	1	1	trzy i więce	2	0	0	0	0
1	1	1	trzy i więce	1	0	0	0	0
1	1	1	trzy i więce	2	60	8,57143	0	8,57143
0	1	0	dwa czynni	3	40	5,71429	5,71429	0
0	1	1	trzy i więce	2	120	17,1429	0	17,1429
0	0	1	trzy i więce	2	180	25,7143	0	25,7143
1	1	0	trzy i więce	2	150	21,4286	21,4286	0
1	0	1	trzy i więce	2	270	38,5714	0	38,5714
0	0	1	dwa czynni	3	40	5,71429	5,71429	0
0	0	0	jeden czyn	2	90	12,8571	4,28571	8,57143
1	0	1	trzy i więce	3	1400	200	20	180
0	0	1	jeden czyn	2	1155	165	45	120
0	0	1	jeden czyn	2	585	83,5714	6,42857	77,1429
0	1	1	trzy i więce	2	0	0	0	0
0	1	1	trzy i więce	2	600	85,7143	0	85,7143
0	0	0	jeden czyn	2	60	8,57143	0	8,57143
1	0	1	trzy i więce	3	210	30	0	30
0	0	0	jeden czyn	3	60	8,57143	8,57143	0
0	0	1	jeden czyn	2	600	85,7143	0	85,7143
0	0	1	trzy i więce	2	0	0	0	0
1	1	1	trzy i więce	3	120	17,1429	8,57143	8,57143
0	0	0	jeden czyn	2	390	55,7143	30	25,7143
1	1	1	trzy i więce	3	180	25,7143	17,1429	8,57143
0	0	0	metabolicz	2	360	51,4286	34,2857	17,1429
0	0	0	jeden czyn	3	390	55,7143	12,8571	42,8571
0	0	1	dwa czynni	2	540	77,1429	25,7143	51,4286
0	0	1	dwa czynni	2	420	60	0	60
0	0	0	jeden czyn	3	90	12,8571	12,8571	0
0	0	0	jeden czyn	3	420	60	0	60
1	1	1	trzy i więce	3	0	0	0	0
0	0	1	dwa czynni	2	180	25,7143	0	25,7143
0	1	1	trzy i więce	1	180	25,7143	0	25,7143
0	0	0	metabolicz	2	195	27,8571	15	12,8571
1	0	1	trzy i więce	3	0	0	0	0
1	0	1	trzy i więce	3	50	7,14286	7,14286	0
1	0	1	trzy i więce	3	60	8,57143	0	8,57143
1	0	1	trzy i więce	2	240	34,2857	34,2857	0
0	0	1	dwa czynni	2	60	8,57143	0	8,57143
1	1	1	trzy i więce	2	0	0	0	0
0	1	1	trzy i więce	3	360	51,4286	0	51,4286
0	1	1	trzy i więce	3	0	0	0	0
0	0	0	metabolicz	2	750	107,143	21,4286	85,7143
0	0	1	trzy i więce	1	0	0	0	0
0	0	1	dwa czynni	3	240	34,2857	17,1429	17,1429

0	0	0	jeden czyn	2	60	8,57143	0	8,57143
0	0	1	dwa czynni	3	0	0	0	0
0	1	1	trzy i więc	3	0	0	0	0
0	0	1	dwa czynni	3	240	34,2857	0	34,2857
1	0	1	trzy i więc	2	480	68,5714	0	68,5714
0	0	1	dwa czynni	2	75	10,7143	6,42857	4,28571
0	0	1	trzy i więc	1	240	34,2857	0	34,2857
0	0	0	metabolicz	3	360	51,4286	0	51,4286
1	1	1	trzy i więc	3	0	0	0	0
0	0	1	dwa czynni	3	490	70	10	60
0	0	1	jeden czyn	2	1050	150	30	120
0	0	1	dwa czynni	3	60	8,57143	0	8,57143
0	1	1	dwa czynni	1	840	120	0	120
1	0	1	trzy i więc	3	0	0	0	0
0	1	1	trzy i więc	1	420	60	0	60
0	0	1	dwa czynni	3	270	38,5714	21,4286	17,1429
1	0	1	trzy i więc	2	1260	180	0	180
0	0	1	dwa czynni	2	0	0	0	0
0	1	1	trzy i więc	2	200	28,5714	0	28,5714
1	0	1	trzy i więc	2	120	17,1429	0	17,1429
1	0	1	trzy i więc	2	45	6,42857	6,42857	0
0	0	1	dwa czynni	2	0	0	0	0
0	0	0	jeden czyn	3	60	8,57143	0	8,57143
0	0	1	trzy i więc	3	840	120	0	120
0	0	1	dwa czynni	1	540	77,1429	0	77,1429
0	1	1	trzy i więc	2	180	25,7143	0	25,7143
1	1	0	trzy i więc	2	0	0	0	0
1	0	0	trzy i więc	1	60	8,57143	0	8,57143
0	0	1	jeden czyn	3	0	0	0	0
1	1	1	trzy i więc	2	30	4,28571	0	4,28571
0	0	0	jeden czyn	1	135	19,2857	0	19,2857
0	0	1	dwa czynni	1	180	25,7143	25,7143	0
0	1	1	trzy i więc	3	0	0	0	0
0	0	0	metabolicz	2	0	0	0	0
0	0	1	dwa czynni	3	120	17,1429	17,1429	0
1	0	0	dwa czynni	2	900	128,571	0	128,571
0	1	1	trzy i więc	2	0	0	0	0
1	1	1	trzy i więc	3	0	0	0	0
0	1	0	dwa czynni	3	465	66,4286	15	51,4286
0	0	1	dwa czynni	2	0	0	0	0
1	1	1	trzy i więc	2	0	0	0	0
1	1	1	trzy i więc	2	40	5,71429	0	5,71429
0	0	1	dwa czynni	2	240	34,2857	0	34,2857
0	0	1	dwa czynni	2	540	77,1429	17,1429	60
0	0	1	dwa czynni	2	0	0	0	0
0	1	1	trzy i więc	3	160	22,8571	14,2857	8,57143
0	0	1	dwa czynni	2	0	0	0	0
1	0	1	trzy i więc	1	100	14,2857	14,2857	0

1	0	0	dwa czynni	2	0	0	0	0
0	1	1	trzy i więc	3	0	0	0	0
0	1	1	trzy i więc	2	60	8,57143	8,57143	0
1	0	1	trzy i więc	3	30	4,28571	0	4,28571
0	0	0	metabolicz	3	780	111,429	8,57143	102,857
0	1	0	dwa czynni	1	0	0	0	0
0	1	0	jeden czyn	2	120	17,1429	17,1429	0
0	1	0	jeden czyn	2	325	46,4286	10,7143	35,7143
0	1	0	dwa czynni	2	0	0	0	0
1	0	1	trzy i więc	2	180	25,7143	0	25,7143
1	0	1	dwa czynni	2	0	0	0	0
0	0	0	metabolicz	2	90	12,8571	0	12,8571
1	0	1	dwa czynni	2	420	60	0	60
0	0	0	metabolicz	2	0	0	0	0
1	0	1	trzy i więc	1	0	0	0	0
1	0	1	dwa czynni	3	60	8,57143	8,57143	0
0	1	0	dwa czynni	2	120	17,1429	17,1429	0
0	0	1	jeden czyn	2	120	17,1429	0	17,1429
1	0	1	trzy i więc	2	240	34,2857	0	34,2857
1	0	1	trzy i więc	2	180	25,7143	0	25,7143
0	0	0	jeden czyn	1	60	8,57143	0	8,57143
1	0	0	trzy i więc	2	180	25,7143	0	25,7143
1	0	1	trzy i więc	2	300	42,8571	0	42,8571
0	1	1	trzy i więc	2	0	0	0	0
0	0	1	dwa czynni	1	420	60	0	60
1	1	1	trzy i więc	1	0	0	0	0
1	0	1	trzy i więc	2	0	0	0	0
1	0	1	trzy i więc	3	0	0	0	0
0	0	1	trzy i więc	2	300	42,8571	0	42,8571
0	0	1	jeden czyn	3	210	30	8,57143	21,4286
0	0	1	dwa czynni	3	420	60	0	60
1	0	1	trzy i więc	2	0	0	0	0
0	0	1	dwa czynni	3	1065	152,143	32,1429	120
1	0	1	trzy i więc	2	140	20	0	20
0	0	1	dwa czynni	1	0	0	0	0
1	1	1	trzy i więc	3	40	5,71429	0	5,71429
1	0	1	trzy i więc	2	0	0	0	0
0	0	1	dwa czynni	2	60	8,57143	0	8,57143
0	0	1	jeden czyn	2	0	0	0	0
0	0	0	jeden czyn	1	60	8,57143	0	8,57143
1	0	1	trzy i więc	2	90	12,8571	0	12,8571
1	1	1	trzy i więc	2	540	77,1429	0	77,1429
0	0	1	dwa czynni	2	210	30	0	30
0	0	0	jeden czyn	2	0	0	0	0
0	1	0	dwa czynni	3	0	0	0	0
0	0	1	jeden czyn	2	0	0	0	0
0	0	1	dwa czynni	2	0	0	0	0
1	1	1	trzy i więc	2	420	60	0	60

0	1	0	dwa czynni	1	60	8,57143	0	8,57143
1	0	0	trzy i więce	3	0	0	0	0
0	1	0	dwa czynni	2	0	0	0	0
0	0	1	dwa czynni	2	0	0	0	0
0	0	1	trzy i więce	3	45	6,42857	0	6,42857
0	0	1	dwa czynni	2	60	8,57143	0	8,57143
1	0	1	trzy i więce	1	0	0	0	0
0	0	1	jeden czyn	2	1080	154,286	34,2857	120
0	0	0	jeden czyn	1	360	51,4286	0	51,4286
1	0	1	trzy i więce	1	210	30	0	30
0	0	1	jeden czyn	2	150	21,4286	0	21,4286
1	1	1	trzy i więce	3	90	12,8571	0	12,8571
0	0	1	dwa czynni	2	420	60	0	60
1	0	1	trzy i więce	3	0	0	0	0
1	0	1	trzy i więce	3	420	60	0	60
0	0	1	dwa czynni	2	0	0	0	0
0	0	0	metabolicz	2	30	4,28571	0	4,28571
0	0	1	jeden czyn	2	30	4,28571	0	4,28571
0	0	1	dwa czynni	2	0	0	0	0
0	0	1	dwa czynni	2	200	28,5714	0	28,5714
0	0	1	dwa czynni	2	280	40	5,71429	34,2857
1	0	1	trzy i więce	2	270	38,5714	12,8571	25,7143
0	0	0	metabolicz	3	60	8,57143	0	8,57143
1	0	1	trzy i więce	2	420	60	0	60
0	0	1	dwa czynni	1	840	120	0	120
0	0	0	jeden czyn	3	720	102,857	51,4286	51,4286
1	0	1	trzy i więce	2	420	60	0	60
1	1	1	trzy i więce	3	195	27,8571	10,7143	17,1429
0	1	1	trzy i więce	3	360	51,4286	0	51,4286
0	0	1	dwa czynni	1	0	0	0	0
1	1	1	trzy i więce	3	0	0	0	0
0	1	1	dwa czynni	1	0	0	0	0
0	0	1	jeden czyn	3	60	8,57143	0	8,57143
1	0	1	trzy i więce	1	420	60	0	60
0	0	1	dwa czynni	2	240	34,2857	8,57143	25,7143
0	0	1	dwa czynni	3	960	137,143	17,1429	120
1	0	1	trzy i więce	2	120	17,1429	0	17,1429
1	0	1	trzy i więce	3	0	0	0	0
0	0	0	metabolicz	2	315	45	19,2857	25,7143
0	0	1	dwa czynni	2	30	4,28571	0	4,28571
1	0	0	trzy i więce	2	0	0	0	0
0	1	1	trzy i więce	2	30	4,28571	0	4,28571
0	0	1	dwa czynni	2	210	30	0	30
0	1	0	trzy i więce	3	120	17,1429	0	17,1429
0	1	1	trzy i więce	2	75	10,7143	0	10,7143
1	1	1	trzy i więce	3	720	102,857	0	102,857
0	1	1	trzy i więce	1	0	0	0	0
0	0	1	trzy i więce	2	120	17,1429	0	17,1429

1	0	1	trzy i więce	2	0	0	0	0
0	0	1	dwa czynni	3	60	8,57143	8,57143	0
1	0	1	trzy i więce	2	840	120	0	120
0	0	0	jeden czyn	3	300	42,8571	0	42,8571
1	0	1	trzy i więce	2	120	17,1429	0	17,1429
1	0	1	trzy i więce	2	0	0	0	0
0	0	1	jeden czyn	2	90	12,8571	12,8571	0
0	0	1	dwa czynni	2	90	12,8571	12,8571	0
0	1	1	trzy i więce	3	0	0	0	0
0	1	1	trzy i więce	2	0	0	0	0
1	1	1	trzy i więce	2	200	28,5714	0	28,5714
0	1	0	jeden czyn	3	45	6,42857	6,42857	0
1	1	0	trzy i więce	3	0	0	0	0
0	0	0	jeden czyn	3	240	34,2857	0	34,2857
0	0	1	dwa czynni	3	0	0	0	0
0	1	1	trzy i więce	3	270	38,5714	4,28571	34,2857
0	0	1	dwa czynni	2	120	17,1429	0	17,1429
0	0	0	jeden czyn	2	105	15	6,42857	8,57143
0	0	0	metabolicz	1	0	0	0	0
0	1	1	dwa czynni	3	510	72,8571	12,8571	60
0	0	1	trzy i więce	2	0	0	0	0
1	0	0	trzy i więce	3	720	102,857	0	102,857
0	0	0	jeden czyn	2	210	30	12,8571	17,1429
1	0	1	trzy i więce	2	20	2,85714	2,85714	0
0	0	1	jeden czyn	2	60	8,57143	0	8,57143
0	0	0	jeden czyn	2	540	77,1429	0	77,1429
0	0	0	metabolicz	1	60	8,57143	0	8,57143
1	0	1	trzy i więce	2	180	25,7143	0	25,7143
0	1	1	trzy i więce	2	0	0	0	0
1	0	1	trzy i więce	3	0	0	0	0
1	0	1	trzy i więce	3	0	0	0	0
0	0	1	jeden czyn	3	300	42,8571	17,1429	25,7143
0	0	1	dwa czynni	2	120	17,1429	17,1429	0
0	0	1	trzy i więce	2	140	20	0	20
0	0	1	trzy i więce	3	0	0	0	0
0	0	1	trzy i więce	1	0	0	0	0
1	0	1	trzy i więce	2	120	17,1429	0	17,1429
0	0	1	trzy i więce	3	1260	180	0	180
0	1	1	trzy i więce	3	180	25,7143	0	25,7143
1	1	1	trzy i więce	1	0	0	0	0
0	0	1	dwa czynni	3	210	30	0	30
0	1	1	trzy i więce	2	0	0	0	0
1	0	1	trzy i więce	2	90	12,8571	0	12,8571
0	0	1	dwa czynni	3	140	20	0	20
1	1	1	trzy i więce	2	120	17,1429	0	17,1429
0	0	0	metabolicz	2	300	42,8571	25,7143	17,1429
1	0	1	trzy i więce	2	420	60	0	60
0	0	1	trzy i więce	2	180	25,7143	0	25,7143

0	0	0	jeden czyn	3	600	85,7143	0	85,7143
0	0	0	jeden czyn	2	480	68,5714	0	68,5714
0	1	1	trzy i więc	2	10	1,42857	1,42857	0
1	0	0	dwa czynni	1	420	60	0	60
0	1	0	dwa czynni	2	15	2,14286	2,14286	0
0	1	1	trzy i więc	2	60	8,57143	0	8,57143
0	0	1	jeden czyn	2	0	0	0	0
1	0	0	trzy i więc	2	0	0	0	0
1	0	1	trzy i więc	2	120	17,1429	0	17,1429
1	0	1	trzy i więc	2	60	8,57143	0	8,57143
1	0	1	trzy i więc	3	240	34,2857	0	34,2857
0	1	1	trzy i więc	2	180	25,7143	0	25,7143
0	0	1	jeden czyn	1	1260	180	0	180
0	0	1	dwa czynni	3	0	0	0	0
1	1	1	trzy i więc	3	0	0	0	0
0	1	1	trzy i więc	3	0	0	0	0
0	0	1	dwa czynni	2	480	68,5714	68,5714	0
0	0	1	jeden czyn	3	1080	154,286	51,4286	102,857
1	0	1	trzy i więc	2	420	60	0	60
0	0	1	jeden czyn	2	0	0	0	0
1	0	1	trzy i więc	2	0	0	0	0
1	0	1	trzy i więc	1	0	0	0	0
0	1	1	dwa czynni	1	0	0	0	0
0	0	1	dwa czynni	2	0	0	0	0
1	0	1	trzy i więc	2	690	98,5714	47,1429	51,4286
0	0	0	jeden czyn	2	240	34,2857	34,2857	0
0	0	0	metabolicz	1	420	60	0	60
0	0	1	trzy i więc	3	0	0	0	0
0	0	1	dwa czynni	1	0	0	0	0
1	1	1	trzy i więc	3	180	25,7143	0	25,7143
1	1	1	trzy i więc	2	310	44,2857	10	34,2857
0	1	1	trzy i więc	2	120	17,1429	17,1429	0
1	0	1	trzy i więc	3	70	10	0	10
0	0	1	dwa czynni	1	0	0	0	0
0	0	1	dwa czynni	2	240	34,2857	0	34,2857
0	0	0	dwa czynni	2	0	0	0	0
0	0	0	metabolicz	1	315	45	19,2857	25,7143
0	0	1	jeden czyn	2	0	0	0	0
1	0	1	trzy i więc	2	0	0	0	0
0	1	1	trzy i więc	1	360	51,4286	0	51,4286
0	0	1	jeden czyn	2	240	34,2857	0	34,2857
1	0	1	trzy i więc	3	120	17,1429	0	17,1429
1	0	1	trzy i więc	1	0	0	0	0
0	0	1	jeden czyn	1	0	0	0	0
1	1	1	trzy i więc	1	0	0	0	0
0	0	1	dwa czynni	2	30	4,28571	4,28571	0
1	1	1	trzy i więc	2	570	81,4286	30	51,4286
0	0	1	dwa czynni	1	225	32,1429	32,1429	0

0	0	1	dwa czynni	1	60	8,57143	0	8,57143
0	0	0	metabolicz	2	0	0	0	0
1	0	1	trzy i więce	2	60	8,57143	8,57143	0
0	0	1	jeden czyn	3	180	25,7143	0	25,7143
0	0	1	dwa czynni	2	280	40	10	30
0	0	1	dwa czynni	2	220	31,4286	5,71429	25,7143
1	0	1	trzy i więce	1	0	0	0	0
1	0	0	trzy i więce	2	0	0	0	0
0	0	0	metabolicz	3	150	21,4286	0	21,4286
1	1	1	trzy i więce	3	0	0	0	0
1	1	1	trzy i więce	3	60	8,57143	0	8,57143
0	1	0	dwa czynni	2	0	0	0	0
0	1	0	jeden czyn	2	0	0	0	0
0	1	1	dwa czynni	2	0	0	0	0
0	1	1	trzy i więce	1	120	17,1429	17,1429	0
1	0	1	trzy i więce	3	180	25,7143	0	25,7143
1	0	1	trzy i więce	2	0	0	0	0
1	1	1	trzy i więce	3	0	0	0	0
0	1	1	trzy i więce	2	270	38,5714	38,5714	0
0	0	1	dwa czynni	2	420	60	0	60
0	0	1	dwa czynni	2	0	0	0	0
0	1	0	dwa czynni	1	180	25,7143	0	25,7143
0	0	0	metabolicz	2	0	0	0	0
1	1	1	trzy i więce	3	0	0	0	0
0	1	1	trzy i więce	1	0	0	0	0
0	0	0	jeden czyn	2	0	0	0	0
0	0	1	jeden czyn	1	600	85,7143	0	85,7143
1	1	1	trzy i więce	3	60	8,57143	0	8,57143
0	0	0	jeden czyn	1	0	0	0	0
1	1	1	trzy i więce	2	400	57,1429	22,8571	34,2857
1	1	1	trzy i więce	2	630	90	0	90
0	0	1	jeden czyn	3	0	0	0	0
0	0	1	dwa czynni	2	60	8,57143	0	8,57143
0	0	1	dwa czynni	2	135	19,2857	0	19,2857
0	0	1	dwa czynni	3	360	51,4286	0	51,4286
0	1	1	trzy i więce	1	60	8,57143	0	8,57143
1	1	1	trzy i więce	1	0	0	0	0
1	1	1	trzy i więce	1	105	15	15	0
1	1	1	trzy i więce	2	180	25,7143	0	25,7143
0	0	1	dwa czynni	2	105	15	15	0
0	1	1	trzy i więce	1	0	0	0	0
0	0	0	jeden czyn	2	0	0	0	0
0	0	0	metabolicz	2	1260	180	0	180
0	0	1	trzy i więce	3	45	6,42857	6,42857	0
0	0	1	dwa czynni	3	540	77,1429	25,7143	51,4286
0	1	1	trzy i więce	2	0	0	0	0
0	0	1	dwa czynni	2	0	0	0	0
1	0	1	trzy i więce	2	120	17,1429	0	17,1429

1	0	1	trzy i więc	2	60	8,57143	0	8,57143
1	0	1	trzy i więc	3	180	25,7143	0	25,7143
1	0	1	trzy i więc	3	180	25,7143	8,57143	17,1429
0	1	1	trzy i więc	2	60	8,57143	0	8,57143
0	0	1	jeden czyn	2	240	34,2857	0	34,2857
0	0	0	dwa czynni	3	0	0	0	0
0	1	1	trzy i więc	2	0	0	0	0
0	0	1	dwa czynni	1	360	51,4286	0	51,4286
0	0	1	dwa czynni	2	45	6,42857	6,42857	0
1	1	1	trzy i więc	2	140	20	2,85714	17,1429
1	1	1	trzy i więc	1	0	0	0	0
1	1	1	trzy i więc	2	240	34,2857	0	34,2857
1	1	1	trzy i więc	3	420	60	0	60
1	1	1	trzy i więc	2	60	8,57143	0	8,57143
0	0	1	dwa czynni	1	180	25,7143	0	25,7143
0	0	1	jeden czyn	2	720	102,857	0	102,857
0	0	0	metabolicz	3	60	8,57143	0	8,57143
0	1	1	trzy i więc	2	135	19,2857	2,14286	17,1429
0	0	1	dwa czynni	3	180	25,7143	0	25,7143
1	0	1	trzy i więc	3	360	51,4286	0	51,4286
0	0	1	trzy i więc	3	350	50	0	50
0	1	1	trzy i więc	2	140	20	0	20
0	0	0	jeden czyn	2	340	48,5714	14,2857	34,2857
0	0	1	dwa czynni	3	210	30	0	30
0	0	1	jeden czyn	3	705	100,714	15	85,7143
0	0	1	trzy i więc	2	540	77,1429	77,1429	0
1	1	1	trzy i więc	2	175	25	25	0
0	0	0	jeden czyn	3	90	12,8571	0	12,8571
0	1	1	dwa czynni	2	120	17,1429	0	17,1429
0	0	0	jeden czyn	3	60	8,57143	0	8,57143
1	1	1	trzy i więc	2	420	60	0	60
0	0	0	jeden czyn	3	180	25,7143	0	25,7143
0	1	1	trzy i więc	3	585	83,5714	6,42857	77,1429
0	1	1	trzy i więc	1	300	42,8571	0	42,8571
0	0	1	dwa czynni	1	0	0	0	0
1	0	0	dwa czynni	1	180	25,7143	0	25,7143
0	0	1	dwa czynni	2	70	10	10	0
0	0	1	dwa czynni	3	0	0	0	0
0	1	1	trzy i więc	2	180	25,7143	0	25,7143
0	0	1	jeden czyn	1	360	51,4286	0	51,4286
0	0	1	dwa czynni	1	840	120	0	120
1	1	1	trzy i więc	2	0	0	0	0
0	0	0	metabolicz	2	0	0	0	0
0	0	1	dwa czynni	2	0	0	0	0
1	1	1	trzy i więc	3	60	8,57143	0	8,57143
1	1	1	trzy i więc	2	165	23,5714	15	8,57143
0	1	1	trzy i więc	3	120	17,1429	17,1429	0
0	1	1	trzy i więc	2	150	21,4286	0	21,4286

1	1	1	trzy i więce	2	360	51,4286	0	51,4286
1	1	1	trzy i więce	1	0	0	0	0
0	1	0	dwa czynni	1	0	0	0	0
1	0	1	trzy i więce	2	0	0	0	0
1	1	1	trzy i więce	1	0	0	0	0
1	0	1	trzy i więce	2	360	51,4286	51,4286	0
0	0	1	dwa czynni	2	60	8,57143	0	8,57143
1	0	0	dwa czynni	2	180	25,7143	0	25,7143
0	0	1	dwa czynni	3	340	48,5714	40	8,57143
0	0	0	metabolicz	2	45	6,42857	6,42857	0
0	0	1	jeden czyn	3	450	64,2857	4,28571	60
1	1	1	trzy i więce	3	120	17,1429	0	17,1429
0	0	0	jeden czyn	2	120	17,1429	0	17,1429
1	1	1	trzy i więce	2	0	0	0	0
1	0	1	trzy i więce	3	540	77,1429	42,8571	34,2857
0	1	1	trzy i więce	2	240	34,2857	0	34,2857
0	1	0	trzy i więce	2	0	0	0	0
1	1	1	trzy i więce	2	0	0	0	0
1	1	1	trzy i więce	2	220	31,4286	5,71429	25,7143
0	1	1	trzy i więce	1	0	0	0	0
0	0	0	metabolicz	3	480	68,5714	0	68,5714
1	1	1	trzy i więce	3	0	0	0	0
1	0	0	trzy i więce	2	180	25,7143	25,7143	0
0	0	1	dwa czynni	1	360	51,4286	0	51,4286
0	0	1	dwa czynni	3	70	10	1,42857	8,57143
0	1	1	trzy i więce	1	180	25,7143	0	25,7143
0	0	1	trzy i więce	3	0	0	0	0
0	0	1	dwa czynni	1	0	0	0	0
1	1	0	trzy i więce	1	420	60	0	60
0	0	1	dwa czynni	3	840	120	0	120
0	0	1	jeden czyn	2	0	0	0	0
0	1	0	trzy i więce	1	120	17,1429	0	17,1429
0	0	0	jeden czyn	2	90	12,8571	12,8571	0
1	0	1	trzy i więce	2	210	30	0	30
1	1	1	trzy i więce	3	0	0	0	0
0	0	1	jeden czyn	1	300	42,8571	0	42,8571
0	1	0	jeden czyn	1	0	0	0	0
0	1	1	trzy i więce	2	360	51,4286	0	51,4286
0	1	1	trzy i więce	2	280	40	0	40
1	0	1	trzy i więce	3	60	8,57143	0	8,57143
0	0	1	dwa czynni	2	60	8,57143	0	8,57143
1	0	1	trzy i więce	2	100	14,2857	10	4,28571
0	1	1	trzy i więce	2	60	8,57143	0	8,57143
1	0	1	trzy i więce	1	120	17,1429	0	17,1429
1	1	1	trzy i więce	2	420	60	0	60
0	0	1	dwa czynni	1	60	8,57143	0	8,57143
0	0	1	dwa czynni	3	450	64,2857	30	34,2857
1	0	1	trzy i więce	1	60	8,57143	0	8,57143

0	1	1	trzy i więce	2	0	0	0	0
0	0	1	dwa czynni	2	0	0	0	0
1	0	1	trzy i więce	2	0	0	0	0
0	0	1	trzy i więce	2	540	77,1429	25,7143	51,4286
0	0	1	dwa czynni	3	60	8,57143	0	8,57143
0	0	1	dwa czynni	2	60	8,57143	0	8,57143
1	1	1	trzy i więce	2	450	64,2857	12,8571	51,4286
0	0	1	dwa czynni	2	0	0	0	0
0	1	1	trzy i więce	1	420	60	30	30
0	0	1	jeden czyn	2	0	0	0	0
1	1	1	trzy i więce	2	0	0	0	0
0	0	1	dwa czynni	2	630	90	30	60
0	0	0	jeden czyn	3	0	0	0	0
1	1	1	trzy i więce	2	420	60	8,57143	51,4286
1	0	1	trzy i więce	3	0	0	0	0
0	0	1	dwa czynni	3	0	0	0	0
0	0	1	dwa czynni	2	120	17,1429	0	17,1429
0	0	1	trzy i więce	2	30	4,28571	0	4,28571
0	1	1	trzy i więce	3	210	30	0	30
0	0	1	dwa czynni	2	0	0	0	0
1	0	1	trzy i więce	3	0	0	0	0
0	0	1	dwa czynni	2	630	90	30	60
0	0	1	dwa czynni	2	180	25,7143	8,57143	17,1429
0	1	1	trzy i więce	1	135	19,2857	0	19,2857
0	0	0	metabolicz	3	720	102,857	0	102,857
1	0	1	trzy i więce	1	450	64,2857	12,8571	51,4286
0	0	1	dwa czynni	2	120	17,1429	17,1429	0
1	0	1	trzy i więce	2	0	0	0	0
0	0	0	jeden czyn	3	120	17,1429	17,1429	0
1	0	0	dwa czynni	3	600	85,7143	0	85,7143
0	0	1	dwa czynni	2	180	25,7143	0	25,7143
0	0	0	jeden czyn	3	0	0	0	0
1	1	1	trzy i więce	2	150	21,4286	0	21,4286
1	1	1	trzy i więce	3	600	85,7143	8,57143	77,1429
0	0	1	dwa czynni	1	0	0	0	0
0	0	1	dwa czynni	1	180	25,7143	0	25,7143
1	0	1	trzy i więce	1	60	8,57143	0	8,57143
0	0	1	dwa czynni	2	285	40,7143	6,42857	34,2857
1	1	1	trzy i więce	2	0	0	0	0
0	0	0	jeden czyn	2	180	25,7143	0	25,7143
0	1	1	trzy i więce	2	50	7,14286	7,14286	0
0	0	1	dwa czynni	1	40	5,71429	0	5,71429
0	0	1	dwa czynni	2	225	32,1429	6,42857	25,7143
0	1	1	trzy i więce	1	120	17,1429	0	17,1429
0	0	0	jeden czyn	2	0	0	0	0
0	0	0	metabolicz	3	0	0	0	0
0	0	1	dwa czynni	2	60	8,57143	0	8,57143
0	0	1	trzy i więce	3	80	11,4286	7,14286	4,28571

1	0	1	trzy i więce	3	240	34,2857	0	34,2857
1	1	1	trzy i więce	2	0	0	0	0
0	0	1	jeden czyn	2	350	50	10	40
0	1	1	trzy i więce	1	360	51,4286	0	51,4286
1	0	1	trzy i więce	2	0	0	0	0
1	0	1	trzy i więce	2	240	34,2857	0	34,2857
1	0	1	trzy i więce	3	0	0	0	0
0	0	0	metabolicz	2	30	4,28571	0	4,28571
0	1	1	trzy i więce	3	0	0	0	0
1	1	1	trzy i więce	2	60	8,57143	8,57143	0
0	1	1	trzy i więce	2	0	0	0	0
1	0	0	dwa czynni	3	300	42,8571	34,2857	8,57143
0	0	1	dwa czynni	3	195	27,8571	10,7143	17,1429
1	0	1	trzy i więce	3	0	0	0	0
0	1	1	trzy i więce	2	420	60	30	30
0	0	0	jeden czyn	2	105	15	0	15
0	0	1	dwa czynni	3	420	60	34,2857	25,7143
1	1	1	trzy i więce	3	120	17,1429	0	17,1429
1	1	1	trzy i więce	3	240	34,2857	0	34,2857
1	1	1	trzy i więce	2	210	30	0	30
0	0	1	trzy i więce	1	0	0	0	0
0	0	0	metabolicz	3	60	8,57143	0	8,57143
0	1	1	trzy i więce	2	0	0	0	0
1	1	1	trzy i więce	1	420	60	0	60
0	0	0	metabolicz	1	0	0	0	0
1	0	1	trzy i więce	1	240	34,2857	0	34,2857
1	1	1	trzy i więce	1	40	5,71429	5,71429	0
0	0	1	dwa czynni	3	0	0	0	0
0	0	0	dwa czynni	3	0	0	0	0
1	0	1	trzy i więce	2	1270	181,429	1,42857	180
0	0	1	jeden czyn	3	40	5,71429	0	5,71429
1	0	0	dwa czynni	1	0	0	0	0
0	0	1	dwa czynni	1	0	0	0	0
0	1	1	trzy i więce	2	240	34,2857	0	34,2857
0	0	1	dwa czynni	2	420	60	0	60
1	0	1	trzy i więce	3	615	87,8571	19,2857	68,5714
0	0	1	dwa czynni	2	0	0	0	0
1	0	1	trzy i więce	2	0	0	0	0
0	0	1	dwa czynni	3	60	8,57143	0	8,57143
1	0	1	trzy i więce	2	0	0	0	0
0	0	0	metabolicz	1	45	6,42857	0	6,42857
1	1	1	trzy i więce	2	60	8,57143	0	8,57143
0	0	1	trzy i więce	2	0	0	0	0
1	0	1	trzy i więce	3	720	102,857	0	102,857
1	1	1	trzy i więce	2	120	17,1429	0	17,1429
1	1	1	trzy i więce	3	0	0	0	0
0	0	0	jeden czyn	3	720	102,857	0	102,857
0	1	1	trzy i więce	3	0	0	0	0

0	0	1	dwa czynni	3	420	60	0	60
0	0	0	metabolicz	2	120	17,1429	0	17,1429
0	0	1	jeden czyn	3	0	0	0	0
0	1	1	trzy i więc	2	180	25,7143	25,7143	0
1	1	1	trzy i więc	3	120	17,1429	8,57143	8,57143
0	1	1	trzy i więc	3	60	8,57143	0	8,57143
0	1	1	dwa czynni	2	180	25,7143	0	25,7143
0	0	0	metabolicz	1	1020	145,714	25,7143	120
1	1	1	trzy i więc	2	120	17,1429	0	17,1429
1	1	0	trzy i więc	1	120	17,1429	0	17,1429
1	0	0	trzy i więc	1	60	8,57143	0	8,57143
0	1	1	trzy i więc	3	80	11,4286	0	11,4286
0	0	1	jeden czyn	3	300	42,8571	42,8571	0
1	0	0	dwa czynni	1	0	0	0	0
0	0	1	jeden czyn	2	80	11,4286	0	11,4286
0	0	0	metabolicz	2	0	0	0	0
1	0	1	trzy i więc	2	1080	154,286	0	154,286
0	0	1	jeden czyn	2	1050	150	30	120
0	0	1	dwa czynni	2	240	34,2857	0	34,2857
1	1	1	trzy i więc	2	210	30	0	30
0	0	1	dwa czynni	2	840	120	0	120
0	0	0	jeden czyn	3	0	0	0	0
0	0	0	jeden czyn	2	0	0	0	0
1	1	1	trzy i więc	1	0	0	0	0
0	0	1	dwa czynni	2	120	17,1429	0	17,1429
0	0	1	trzy i więc	1	150	21,4286	12,8571	8,57143
0	1	1	trzy i więc	1	120	17,1429	0	17,1429
0	0	0	metabolicz	3	360	51,4286	0	51,4286
0	0	1	jeden czyn	3	420	60	0	60
0	1	0	jeden czyn	2	480	68,5714	8,57143	60
0	0	1	jeden czyn	3	480	68,5714	0	68,5714
1	0	1	trzy i więc	2	0	0	0	0
1	0	0	trzy i więc	3	180	25,7143	25,7143	0
1	0	1	trzy i więc	3	45	6,42857	6,42857	0
0	1	1	trzy i więc	3	0	0	0	0
0	0	0	metabolicz	2	315	45	15	30
0	0	1	jeden czyn	3	360	51,4286	0	51,4286
0	1	1	trzy i więc	3	360	51,4286	8,57143	42,8571
0	1	1	trzy i więc	2	210	30	0	30
0	0	0	jeden czyn	2	420	60	0	60
0	0	1	dwa czynni	3	840	120	0	120
0	1	1	trzy i więc	2	60	8,57143	0	8,57143
1	0	1	trzy i więc	2	225	32,1429	15	17,1429
0	0	0	metabolicz	3	315	45	19,2857	25,7143
1	1	0	trzy i więc	2	0	0	0	0
0	0	1	dwa czynni	2	0	0	0	0
0	1	0	dwa czynni	3	0	0	0	0
0	0	0	jeden czyn	2	120	17,1429	0	17,1429

0	0	1	dwa czynni	3	345	49,2857	23,5714	25,7143
0	0	1	dwa czynni	3	60	8,57143	0	8,57143
0	1	1	trzy i więc	2	140	20	20	0
1	1	1	trzy i więc	2	0	0	0	0
0	0	1	dwa czynni	3	1860	265,714	85,7143	180
0	0	1	trzy i więc	2	360	51,4286	51,4286	0
1	1	1	trzy i więc	3	90	12,8571	0	12,8571
1	1	1	trzy i więc	2	180	25,7143	0	25,7143
0	0	1	dwa czynni	2	840	120	0	120
1	0	1	trzy i więc	2	260	37,1429	11,4286	25,7143
0	0	1	dwa czynni	2	330	47,1429	17,1429	30
0	0	0	jeden czyn	1	870	124,286	4,28571	120
0	0	0	jeden czyn	3	0	0	0	0
0	0	1	dwa czynni	3	240	34,2857	8,57143	25,7143
0	0	1	dwa czynni	2	0	0	0	0
1	1	1	trzy i więc	2	0	0	0	0
0	0	1	trzy i więc	2	0	0	0	0
0	1	0	dwa czynni	2	450	64,2857	38,5714	25,7143
1	0	1	trzy i więc	3	0	0	0	0
0	0	0	metabolicz	2	30	4,28571	4,28571	0
0	0	1	dwa czynni	2	0	0	0	0
0	0	1	dwa czynni	3	0	0	0	0
0	0	1	dwa czynni	2	240	34,2857	0	34,2857
0	0	1	dwa czynni	2	105	15	0	15
0	1	1	trzy i więc	3	120	17,1429	0	17,1429
0	0	0	metabolicz	2	45	6,42857	6,42857	0
0	0	0	jeden czyn	2	30	4,28571	0	4,28571
0	0	0	jeden czyn	3	300	42,8571	42,8571	0
0	0	1	trzy i więc	3	45	6,42857	6,42857	0
1	1	1	trzy i więc	2	120	17,1429	17,1429	0
1	0	1	trzy i więc	2	140	20	8,57143	11,4286
1	0	0	dwa czynni	1	120	17,1429	0	17,1429
0	0	0	jeden czyn	2	0	0	0	0
0	0	1	jeden czyn	1	180	25,7143	0	25,7143
0	0	1	dwa czynni	3	60	8,57143	0	8,57143
0	0	0	jeden czyn	3	0	0	0	0
0	1	1	trzy i więc	3	0	0	0	0
1	1	1	trzy i więc	2	120	17,1429	0	17,1429
1	0	0	dwa czynni	3	0	0	0	0
1	0	1	trzy i więc	1	165	23,5714	6,42857	17,1429
0	0	0	jeden czyn	3	720	102,857	102,857	0
0	1	1	trzy i więc	3	0	0	0	0
0	1	1	dwa czynni	1	660	94,2857	42,8571	51,4286
1	1	1	trzy i więc	2	100	14,2857	0	14,2857
0	0	1	dwa czynni	3	180	25,7143	0	25,7143
0	0	1	jeden czyn	2	0	0	0	0
0	0	0	jeden czyn	1	0	0	0	0
0	0	1	dwa czynni	1	210	30	0	30

0	0	1	jeden czyn	3	45	6,42857	6,42857	0
0	0	1	dwa czynni	3	600	85,7143	8,57143	77,1429
0	0	1	dwa czynni	3	0	0	0	0
0	1	1	trzy i więce	2	80	11,4286	0	11,4286
0	0	1	dwa czynni	2	1170	167,143	77,1429	90
0	1	1	trzy i więce	2	0	0	0	0
0	0	1	dwa czynni	1	840	120	0	120
0	0	0	jeden czyn	3	450	64,2857	51,4286	12,8571
1	1	0	trzy i więce	1	0	0	0	0
1	0	1	trzy i więce	2	420	60	0	60
0	1	1	dwa czynni	2	0	0	0	0
1	0	1	trzy i więce	3	240	34,2857	0	34,2857
0	0	1	jeden czyn	2	300	42,8571	17,1429	25,7143
0	0	1	dwa czynni	3	120	17,1429	0	17,1429
1	0	1	trzy i więce	2	120	17,1429	8,57143	8,57143
0	0	1	dwa czynni	2	900	128,571	102,857	25,7143
0	0	0	jeden czyn	1	240	34,2857	0	34,2857
0	0	1	jeden czyn	2	0	0	0	0
0	0	1	jeden czyn	2	300	42,8571	30	12,8571
0	1	1	trzy i więce	3	240	34,2857	0	34,2857
1	0	1	trzy i więce	2	0	0	0	0
0	1	1	trzy i więce	2	0	0	0	0
1	0	1	trzy i więce	3	720	102,857	0	102,857
0	1	1	trzy i więce	3	0	0	0	0
0	0	0	jeden czyn	2	225	32,1429	15	17,1429
0	1	0	dwa czynni	3	630	90	30	60
0	0	1	dwa czynni	2	1020	145,714	128,571	17,1429
1	0	1	trzy i więce	2	180	25,7143	0	25,7143
1	0	1	trzy i więce	2	540	77,1429	0	77,1429
1	1	1	trzy i więce	2	420	60	60	0
0	0	1	dwa czynni	2	0	0	0	0
0	0	1	dwa czynni	1	70	10	10	0
1	0	1	trzy i więce	2	120	17,1429	17,1429	0
0	0	1	trzy i więce	1	435	62,1429	45	17,1429
1	1	0	trzy i więce	1	0	0	0	0
0	0	0	jeden czyn	3	180	25,7143	0	25,7143
0	0	1	dwa czynni	1	150	21,4286	0	21,4286
0	1	1	trzy i więce	2	0	0	0	0
0	1	0	dwa czynni	2	210	30	0	30
1	0	0	trzy i więce	2	0	0	0	0
1	0	1	trzy i więce	2	0	0	0	0
1	0	1	trzy i więce	3	490	70	10	60
1	1	1	trzy i więce	2	0	0	0	0
0	0	1	dwa czynni	2	150	21,4286	0	21,4286
1	0	0	dwa czynni	1	300	42,8571	0	42,8571
0	0	0	jeden czyn	3	60	8,57143	0	8,57143
1	0	0	trzy i więce	3	0	0	0	0
0	0	0	jeden czyn	2	480	68,5714	0	68,5714

1	0	0	trzy i więce	2	30	4,28571	4,28571	0
0	1	1	trzy i więce	3	120	17,1429	0	17,1429
0	1	1	trzy i więce	1	0	0	0	0
1	0	1	trzy i więce	3	60	8,57143	0	8,57143
0	0	1	dwa czynni	2	0	0	0	0
0	1	1	trzy i więce	2	0	0	0	0
0	0	1	trzy i więce	2	0	0	0	0
0	0	1	dwa czynni	1	0	0	0	0
0	0	1	dwa czynni	1	300	42,8571	12,8571	30
1	0	1	trzy i więce	1	120	17,1429	0	17,1429
0	0	1	dwa czynni	2	0	0	0	0
0	1	1	trzy i więce	2	0	0	0	0
0	0	0	jeden czyn	3	330	47,1429	38,5714	8,57143
0	0	0	metabolicz	3	360	51,4286	17,1429	34,2857
0	0	1	dwa czynni	2	0	0	0	0
0	0	1	jeden czyn	3	960	137,143	17,1429	120
0	1	1	trzy i więce	3	60	8,57143	0	8,57143
0	1	1	trzy i więce	3	210	30	0	30
0	0	1	dwa czynni	2	75	10,7143	10,7143	0
0	1	1	trzy i więce	3	0	0	0	0
1	1	1	trzy i więce	1	0	0	0	0
1	1	1	trzy i więce	3	900	128,571	8,57143	120
0	0	0	metabolicz	2	720	102,857	0	102,857
0	0	1	jeden czyn	3	180	25,7143	0	25,7143
1	1	1	trzy i więce	3	120	17,1429	0	17,1429
0	0	1	dwa czynni	3	120	17,1429	0	17,1429
0	0	1	dwa czynni	2	60	8,57143	0	8,57143
1	0	0	trzy i więce	3	40	5,71429	0	5,71429
0	0	1	dwa czynni	3	360	51,4286	34,2857	17,1429
0	0	1	dwa czynni	2	0	0	0	0
0	0	1	trzy i więce	3	0	0	0	0
1	0	1	trzy i więce	2	60	8,57143	0	8,57143
1	0	1	trzy i więce	2	15	2,14286	2,14286	0
0	0	1	dwa czynni	2	0	0	0	0
0	0	1	dwa czynni	2	120	17,1429	0	17,1429
0	0	0	jeden czyn	3	420	60	0	60
1	1	1	trzy i więce	3	0	0	0	0
0	0	1	dwa czynni	2	120	17,1429	0	17,1429
0	0	1	jeden czyn	1	0	0	0	0
1	0	0	dwa czynni	1	960	137,143	137,143	0
0	0	0	jeden czyn	2	60	8,57143	0	8,57143
1	0	1	trzy i więce	3	0	0	0	0
1	0	0	dwa czynni	1	285	40,7143	6,42857	34,2857
1	0	1	trzy i więce	2	0	0	0	0
1	0	1	trzy i więce	2	120	17,1429	0	17,1429
1	0	1	trzy i więce	2	180	25,7143	0	25,7143
0	1	1	trzy i więce	3	0	0	0	0
0	0	0	jeden czyn	1	0	0	0	0

1	0	0	trzy i więce	2	120	17,1429	0	17,1429
0	0	1	trzy i więce	3	0	0	0	0
0	0	1	dwa czynni	3	120	17,1429	0	17,1429
0	0	1	dwa czynni	2	840	120	0	120
0	1	1	trzy i więce	3	405	57,8571	6,42857	51,4286
1	0	0	dwa czynni	1	60	8,57143	0	8,57143
1	1	1	trzy i więce	1	255	36,4286	19,2857	17,1429
1	0	1	trzy i więce	1	60	8,57143	0	8,57143
0	0	1	dwa czynni	2	60	8,57143	0	8,57143
0	0	1	dwa czynni	2	360	51,4286	0	51,4286
1	0	1	dwa czynni	3	330	47,1429	34,2857	12,8571
0	0	0	metabolicz	1	0	0	0	0
0	0	1	dwa czynni	2	0	0	0	0
0	0	0	jeden czyn	3	165	23,5714	15	8,57143
0	0	1	jeden czyn	1	300	42,8571	25,7143	17,1429
0	0	1	dwa czynni	2	210	30	0	30
0	0	1	jeden czyn	2	1050	150	30	120
0	0	1	trzy i więce	2	0	0	0	0
0	0	0	jeden czyn	1	120	17,1429	17,1429	0
0	1	1	trzy i więce	3	240	34,2857	0	34,2857
0	0	0	metabolicz	3	60	8,57143	8,57143	0
1	0	0	dwa czynni	3	0	0	0	0
0	1	1	trzy i więce	1	280	40	0	40
0	0	1	dwa czynni	3	0	0	0	0
1	0	1	trzy i więce	2	90	12,8571	12,8571	0
0	0	1	dwa czynni	3	0	0	0	0
1	1	1	trzy i więce	3	210	30	0	30
1	0	0	trzy i więce	2	360	51,4286	0	51,4286
0	0	1	dwa czynni	2	0	0	0	0
1	0	1	trzy i więce	1	120	17,1429	0	17,1429
0	0	1	trzy i więce	1	0	0	0	0
1	1	1	trzy i więce	2	300	42,8571	0	42,8571
0	0	0	metabolicz	3	180	25,7143	25,7143	0
0	1	1	trzy i więce	3	45	6,42857	6,42857	0
1	1	1	trzy i więce	2	0	0	0	0
0	0	1	dwa czynni	2	240	34,2857	0	34,2857
0	0	1	jeden czyn	1	90	12,8571	0	12,8571
1	0	1	trzy i więce	2	120	17,1429	17,1429	0
0	0	1	dwa czynni	2	60	8,57143	0	8,57143
0	1	0	trzy i więce	3	540	77,1429	25,7143	51,4286
0	0	0	jeden czyn	3	810	115,714	30	85,7143
0	0	1	dwa czynni	2	120	17,1429	0	17,1429
1	1	1	trzy i więce	2	0	0	0	0
0	0	0	metabolicz	2	60	8,57143	0	8,57143
1	0	1	trzy i więce	2	240	34,2857	0	34,2857
0	0	1	dwa czynni	2	0	0	0	0
0	0	0	metabolicz	3	240	34,2857	0	34,2857
0	0	1	dwa czynni	3	0	0	0	0

1	0	1	trzy i więc	2	30	4,28571	0	4,28571
0	0	0	metabolicz	2	300	42,8571	25,7143	17,1429
0	0	1	dwa czynni	3	525	75	15	60
0	1	1	trzy i więc	2	420	60	30	30
0	0	1	dwa czynni	2	60	8,57143	0	8,57143
1	0	1	trzy i więc	2	0	0	0	0
0	1	1	trzy i więc	3	120	17,1429	0	17,1429
1	0	0	jeden czyn	2	120	17,1429	0	17,1429
0	0	0	jeden czyn	3	600	85,7143	17,1429	68,5714
0	1	0	dwa czynni	2	0	0	0	0
0	1	1	trzy i więc	1	0	0	0	0
1	1	1	trzy i więc	3	70	10	10	0
0	0	1	trzy i więc	3	0	0	0	0
1	1	1	trzy i więc	1	360	51,4286	0	51,4286
1	1	1	trzy i więc	1	105	15	15	0
0	0	0	metabolicz	2	360	51,4286	0	51,4286
1	0	1	trzy i więc	3	600	85,7143	0	85,7143
0	0	0	jeden czyn	2	150	21,4286	0	21,4286
0	0	0	jeden czyn	1	630	90	77,1429	12,8571
0	1	0	jeden czyn	1	1260	180	0	180
0	0	1	dwa czynni	3	0	0	0	0
0	0	1	jeden czyn	2	60	8,57143	8,57143	0
1	1	1	trzy i więc	2	360	51,4286	0	51,4286
0	0	0	metabolicz	2	15	2,14286	2,14286	0
1	0	1	trzy i więc	2	720	102,857	0	102,857
0	0	1	trzy i więc	3	120	17,1429	0	17,1429
0	0	1	dwa czynni	2	0	0	0	0
0	0	0	jeden czyn	3	0	0	0	0
0	0	1	dwa czynni	2	0	0	0	0
0	0	1	jeden czyn	1	0	0	0	0
0	0	1	jeden czyn	2	0	0	0	0
0	0	1	dwa czynni	2	150	21,4286	12,8571	8,57143
1	0	0	dwa czynni	2	0	0	0	0
0	0	1	dwa czynni	2	720	102,857	0	102,857
0	1	1	trzy i więc	1	60	8,57143	8,57143	0
0	0	1	trzy i więc	3	0	0	0	0
1	0	1	dwa czynni	2	840	120	0	120
0	0	0	jeden czyn	2	0	0	0	0
1	1	1	trzy i więc	2	480	68,5714	68,5714	0
0	0	1	jeden czyn	3	0	0	0	0
1	0	1	trzy i więc	2	0	0	0	0
0	0	0	metabolicz	2	0	0	0	0
0	1	1	trzy i więc	2	0	0	0	0
1	1	1	trzy i więc	2	0	0	0	0
1	1	1	trzy i więc	3	480	68,5714	0	68,5714
0	0	1	trzy i więc	2	420	60	0	60
1	0	1	trzy i więc	2	55	7,85714	0	7,85714
1	1	1	trzy i więc	3	0	0	0	0

0	0	1	dwa czynni	2	360	51,4286	0	51,4286
0	0	1	dwa czynni	2	150	21,4286	0	21,4286
0	0	1	dwa czynni	3	0	0	0	0
0	0	0	jeden czyn	3	420	60	0	60
0	0	0	jeden czyn	2	150	21,4286	21,4286	0
0	1	1	trzy i więc	2	135	19,2857	15	4,28571
1	0	1	trzy i więc	3	465	66,4286	15	51,4286
0	1	1	dwa czynni	1	540	77,1429	0	77,1429
0	1	1	trzy i więc	1	180	25,7143	4,28571	21,4286
1	1	1	trzy i więc	2	280	40	10	30
0	0	1	dwa czynni	3	90	12,8571	0	12,8571
1	1	1	trzy i więc	2	15	2,14286	0	2,14286
1	0	1	trzy i więc	3	180	25,7143	0	25,7143
0	0	0	jeden czyn	2	0	0	0	0
1	0	1	trzy i więc	2	0	0	0	0
0	1	0	dwa czynni	3	60	8,57143	8,57143	0
1	1	1	trzy i więc	2	240	34,2857	0	34,2857
0	0	1	jeden czyn	1	30	4,28571	4,28571	0
0	0	1	dwa czynni	3	360	51,4286	0	51,4286
0	0	1	dwa czynni	3	0	0	0	0
0	0	0	jeden czyn	2	210	30	0	30
0	0	1	trzy i więc	3	135	19,2857	0	19,2857
0	0	1	dwa czynni	3	540	77,1429	0	77,1429
0	1	1	trzy i więc	3	30	4,28571	0	4,28571
0	0	1	dwa czynni	1	0	0	0	0
1	0	0	trzy i więc	1	135	19,2857	19,2857	0
0	0	0	dwa czynni	3	120	17,1429	0	17,1429
0	0	0	jeden czyn	2	0	0	0	0
0	1	1	trzy i więc	2	930	132,857	12,8571	120
0	0	1	jeden czyn	3	0	0	0	0
0	1	1	trzy i więc	2	0	0	0	0
0	1	1	trzy i więc	3	240	34,2857	0	34,2857
0	1	1	trzy i więc	2	420	60	0	60
0	0	0	metabolicz	1	360	51,4286	42,8571	8,57143
0	0	1	jeden czyn	2	420	60	0	60
1	0	0	dwa czynni	1	465	66,4286	6,42857	60
0	0	0	metabolicz	3	0	0	0	0
0	0	0	metabolicz	2	0	0	0	0
0	1	1	trzy i więc	3	630	90	64,2857	25,7143
1	0	1	trzy i więc	3	240	34,2857	0	34,2857
1	1	0	trzy i więc	3	660	94,2857	42,8571	51,4286
1	1	0	trzy i więc	3	120	17,1429	17,1429	0
0	0	0	jeden czyn	3	120	17,1429	0	17,1429
0	0	0	jeden czyn	2	0	0	0	0
0	1	1	trzy i więc	2	0	0	0	0
1	1	1	trzy i więc	2	0	0	0	0
1	1	1	trzy i więc	2	0	0	0	0
0	0	0	metabolicz	3	0	0	0	0

0	0	0	jeden czyn	2	600	85,7143	25,7143	60
1	1	1	trzy i więc	3	0	0	0	0
0	1	1	trzy i więc	2	180	25,7143	0	25,7143
0	0	1	dwa czynni	2	0	0	0	0
0	0	1	dwa czynni	3	0	0	0	0
0	1	1	trzy i więc	2	0	0	0	0
0	0	1	jeden czyn	3	0	0	0	0
0	0	1	jeden czyn	2	120	17,1429	0	17,1429
1	0	1	trzy i więc	2	0	0	0	0
1	0	0	dwa czynni	2	105	15	0	15
0	0	1	dwa czynni	1	420	60	0	60
0	1	1	trzy i więc	2	0	0	0	0
0	0	1	jeden czyn	2	250	35,7143	0	35,7143
0	0	1	jeden czyn	2	0	0	0	0
1	1	1	trzy i więc	3	60	8,57143	0	8,57143
1	0	1	trzy i więc	1	0	0	0	0
0	0	0	jeden czyn	2	0	0	0	0
1	1	0	trzy i więc	2	330	47,1429	30	17,1429
0	0	0	jeden czyn	3	465	66,4286	6,42857	60
0	0	0	dwa czynni	2	0	0	0	0
0	0	1	dwa czynni	2	0	0	0	0
1	0	1	trzy i więc	2	315	45	2,14286	42,8571
1	0	0	dwa czynni	2	660	94,2857	34,2857	60
0	0	1	jeden czyn	1	0	0	0	0
0	1	1	trzy i więc	1	420	60	0	60
0	1	1	trzy i więc	1	0	0	0	0
0	1	1	trzy i więc	2	45	6,42857	6,42857	0
0	0	1	dwa czynni	3	210	30	0	30
1	1	1	trzy i więc	2	0	0	0	0
1	0	1	trzy i więc	3	60	8,57143	0	8,57143
1	1	0	trzy i więc	2	420	60	0	60
0	0	1	jeden czyn	2	240	34,2857	0	34,2857
1	0	0	trzy i więc	2	180	25,7143	0	25,7143
1	0	1	trzy i więc	1	540	77,1429	0	77,1429
0	0	0	jeden czyn	3	120	17,1429	0	17,1429
1	0	1	trzy i więc	2	120	17,1429	0	17,1429
0	1	1	trzy i więc	2	0	0	0	0
0	0	1	dwa czynni	1	120	17,1429	0	17,1429
0	0	0	jeden czyn	2	0	0	0	0
0	0	1	jeden czyn	3	0	0	0	0
0	0	0	dwa czynni	3	80	11,4286	11,4286	0
1	1	1	trzy i więc	1	420	60	0	60
0	0	0	jeden czyn	2	0	0	0	0
0	0	1	trzy i więc	3	0	0	0	0
0	0	1	trzy i więc	3	120	17,1429	0	17,1429
0	0	1	jeden czyn	2	260	37,1429	2,85714	34,2857
1	0	1	trzy i więc	3	325	46,4286	14,2857	32,1429
0	1	1	trzy i więc	3	60	8,57143	0	8,57143

0	0	1	dwa czynni	2	0	0	0	0
1	0	1	trzy i więce	3	420	60	0	60
0	1	1	trzy i więce	3	420	60	42,8571	17,1429
0	1	0	dwa czynni	3	585	83,5714	23,5714	60
0	0	0	jeden czyn	2	120	17,1429	0	17,1429
0	0	0	jeden czyn	2	50	7,14286	7,14286	0
0	0	0	jeden czyn	2	20	2,85714	0	2,85714
1	0	1	trzy i więce	2	10	1,42857	1,42857	0
1	1	1	trzy i więce	3	360	51,4286	0	51,4286
1	0	1	trzy i więce	1	60	8,57143	8,57143	0
0	0	1	jeden czyn	1	120	17,1429	0	17,1429
0	0	1	jeden czyn	3	0	0	0	0
1	0	1	trzy i więce	2	0	0	0	0
0	1	1	trzy i więce	1	0	0	0	0
1	1	1	trzy i więce	2	30	4,28571	0	4,28571
0	0	1	dwa czynni	2	0	0	0	0
0	0	1	dwa czynni	2	60	8,57143	0	8,57143
0	0	1	dwa czynni	3	720	102,857	0	102,857
0	1	1	trzy i więce	2	0	0	0	0
0	0	1	jeden czyn	3	0	0	0	0
0	0	1	dwa czynni	3	420	60	0	60
0	0	1	dwa czynni	2	30	4,28571	0	4,28571
0	1	1	trzy i więce	2	80	11,4286	0	11,4286
0	0	1	trzy i więce	1	240	34,2857	0	34,2857
1	0	0	dwa czynni	2	0	0	0	0
0	0	1	dwa czynni	3	0	0	0	0
0	0	1	dwa czynni	3	660	94,2857	17,1429	77,1429
0	0	1	dwa czynni	2	180	25,7143	0	25,7143
0	0	1	jeden czyn	2	130	18,5714	10	8,57143
1	0	0	dwa czynni	3	90	12,8571	12,8571	0
0	0	1	dwa czynni	1	0	0	0	0
0	1	1	dwa czynni	3	90	12,8571	0	12,8571
0	0	1	dwa czynni	2	0	0	0	0
0	1	1	trzy i więce	3	0	0	0	0
0	0	0	metabolicz	3	300	42,8571	0	42,8571
1	0	0	dwa czynni	2	120	17,1429	0	17,1429
0	0	0	dwa czynni	2	45	6,42857	6,42857	0
0	0	1	jeden czyn	1	90	12,8571	0	12,8571
0	0	1	trzy i więce	1	120	17,1429	0	17,1429
1	1	1	trzy i więce	3	270	38,5714	38,5714	0
1	0	1	trzy i więce	2	45	6,42857	0	6,42857
0	0	1	trzy i więce	2	0	0	0	0
0	0	0	metabolicz	2	120	17,1429	0	17,1429
0	1	1	trzy i więce	1	180	25,7143	0	25,7143
1	0	1	trzy i więce	1	105	15	0	15
0	0	1	dwa czynni	2	360	51,4286	0	51,4286
0	0	1	jeden czyn	3	0	0	0	0
0	0	0	jeden czyn	2	180	25,7143	25,7143	0

0	0	1	dwa czynni	2	130	18,5714	1,42857	17,1429
1	1	1	trzy i więce	1	10	1,42857	0	1,42857
0	0	1	dwa czynni	2	180	25,7143	17,1429	8,57143
0	1	1	trzy i więce	3	0	0	0	0
1	1	1	trzy i więce	2	420	60	0	60
0	0	0	jeden czyn	2	0	0	0	0
0	0	1	dwa czynni	2	160	22,8571	5,71429	17,1429
1	0	1	trzy i więce	2	200	28,5714	0	28,5714
1	1	1	trzy i więce	1	295	42,1429	6,42857	35,7143
0	0	0	metabolicz	2	0	0	0	0
0	1	1	trzy i więce	3	60	8,57143	0	8,57143
1	1	1	trzy i więce	2	0	0	0	0
0	0	1	trzy i więce	3	560	80	20	60
1	1	1	trzy i więce	2	450	64,2857	30	34,2857
0	0	1	dwa czynni	3	180	25,7143	0	25,7143
0	1	0	dwa czynni	3	750	107,143	64,2857	42,8571
0	0	1	jeden czyn	3	0	0	0	0
0	1	1	trzy i więce	3	40	5,71429	1,42857	4,28571
0	1	0	trzy i więce	2	0	0	0	0
0	1	1	trzy i więce	3	300	42,8571	17,1429	25,7143
0	1	1	trzy i więce	1	240	34,2857	0	34,2857
0	0	0	dwa czynni	2	255	36,4286	6,42857	30
0	1	1	trzy i więce	1	255	36,4286	15	21,4286
0	0	0	dwa czynni	2	0	0	0	0
0	1	1	trzy i więce	2	0	0	0	0
0	0	1	trzy i więce	3	420	60	0	60
0	1	1	trzy i więce	2	60	8,57143	0	8,57143
0	1	1	trzy i więce	2	510	72,8571	30	42,8571
0	0	1	dwa czynni	3	0	0	0	0
0	1	1	trzy i więce	2	360	51,4286	11,4286	40
1	0	1	trzy i więce	2	60	8,57143	0	8,57143
0	1	1	trzy i więce	3	45	6,42857	6,42857	0
1	0	1	trzy i więce	2	420	60	0	60
0	1	1	trzy i więce	3	180	25,7143	25,7143	0
0	0	1	dwa czynni	2	30	4,28571	0	4,28571
1	1	1	trzy i więce	2	0	0	0	0
0	0	0	jeden czyn	1	180	25,7143	0	25,7143
1	1	1	trzy i więce	3	0	0	0	0
1	0	1	trzy i więce	3	150	21,4286	4,28571	17,1429
0	0	0	metabolicz	2	315	45	45	0
0	1	1	trzy i więce	2	0	0	0	0
0	0	1	dwa czynni	3	420	60	0	60
1	0	0	trzy i więce	1	450	64,2857	30	34,2857
0	0	1	dwa czynni	2	15	2,14286	2,14286	0
0	0	0	metabolicz	2	0	0	0	0
1	1	1	trzy i więce	2	420	60	0	60
0	0	1	jeden czyn	3	0	0	0	0
0	0	1	jeden czyn	1	420	60	0	60

0	0	1	dwa czynni	3	60	8,57143	8,57143	0
0	0	0	metabolicz	2	0	0	0	0
0	0	1	dwa czynni	2	120	17,1429	17,1429	0
0	0	1	dwa czynni	1	0	0	0	0
0	0	0	jeden czyn	3	120	17,1429	0	17,1429
0	0	1	trzy i więc	3	840	120	120	0
0	1	1	trzy i więc	1	210	30	0	30
0	0	1	jeden czyn	2	0	0	0	0
1	0	1	trzy i więc	2	690	98,5714	68,5714	30
1	1	1	trzy i więc	2	120	17,1429	0	17,1429
0	0	1	dwa czynni	2	360	51,4286	0	51,4286
0	0	1	dwa czynni	2	0	0	0	0
0	0	1	dwa czynni	3	360	51,4286	34,2857	17,1429
1	0	1	trzy i więc	2	0	0	0	0
1	0	1	trzy i więc	3	0	0	0	0
1	0	1	trzy i więc	3	0	0	0	0
0	0	1	dwa czynni	3	480	68,5714	34,2857	34,2857
0	0	1	trzy i więc	2	0	0	0	0
0	0	1	jeden czyn	2	0	0	0	0
0	0	1	jeden czyn	1	180	25,7143	0	25,7143
0	1	1	trzy i więc	2	180	25,7143	0	25,7143
0	1	1	trzy i więc	3	210	30	0	30
0	0	1	jeden czyn	3	630	90	12,8571	77,1429
0	0	0	jeden czyn	2	60	8,57143	0	8,57143
0	0	0	jeden czyn	1	0	0	0	0
1	0	0	trzy i więc	2	60	8,57143	0	8,57143
1	1	1	trzy i więc	2	120	17,1429	0	17,1429
1	1	1	trzy i więc	2	120	17,1429	0	17,1429
1	0	1	trzy i więc	3	0	0	0	0
0	0	1	dwa czynni	3	0	0	0	0
0	1	1	trzy i więc	2	0	0	0	0
0	0	0	jeden czyn	1	120	17,1429	0	17,1429
1	0	1	trzy i więc	2	120	17,1429	0	17,1429
0	1	1	trzy i więc	2	0	0	0	0
0	0	0	jeden czyn	2	0	0	0	0
1	0	0	dwa czynni	2	0	0	0	0
0	1	1	trzy i więc	1	910	130	10	120
1	1	1	trzy i więc	2	0	0	0	0
1	0	1	trzy i więc	3	140	20	11,4286	8,57143
0	0	1	trzy i więc	3	0	0	0	0
0	0	0	metabolicz	2	150	21,4286	17,1429	4,28571
1	0	1	trzy i więc	3	0	0	0	0
1	0	0	dwa czynni	2	0	0	0	0
1	0	1	trzy i więc	1	360	51,4286	0	51,4286
0	0	0	metabolicz	2	0	0	0	0
0	0	1	dwa czynni	3	0	0	0	0
0	0	0	metabolicz	3	0	0	0	0
1	0	1	trzy i więc	3	600	85,7143	51,4286	34,2857

0	0	1	jeden czyn	2	120	17,1429	0	17,1429
0	0	1	dwa czynni	3	140	20	20	0
1	1	1	trzy i więce	2	0	0	0	0
1	1	1	trzy i więce	2	90	12,8571	0	12,8571
1	1	1	trzy i więce	2	510	72,8571	12,8571	60
0	0	1	trzy i więce	3	240	34,2857	0	34,2857
1	1	1	trzy i więce	2	60	8,57143	8,57143	0
1	1	1	trzy i więce	3	120	17,1429	0	17,1429
0	0	0	metabolicz	3	0	0	0	0
0	1	0	dwa czynni	2	210	30	30	0
0	0	0	jeden czyn	2	210	30	12,8571	17,1429
0	0	0	metabolicz	3	10	1,42857	1,42857	0
0	0	1	dwa czynni	3	120	17,1429	0	17,1429
0	0	0	jeden czyn	1	420	60	0	60
1	0	1	trzy i więce	1	210	30	0	30
0	1	1	trzy i więce	3	60	8,57143	0	8,57143
0	1	1	trzy i więce	2	0	0	0	0
0	1	1	trzy i więce	1	0	0	0	0
0	0	0	jeden czyn	3	780	111,429	25,7143	85,7143
0	0	0	jeden czyn	2	200	28,5714	0	28,5714
1	0	1	trzy i więce	3	230	32,8571	20	12,8571
0	0	1	dwa czynni	2	165	23,5714	6,42857	17,1429
1	0	0	trzy i więce	2	180	25,7143	8,57143	17,1429
0	0	1	dwa czynni	3	10	1,42857	1,42857	0
0	1	1	trzy i więce	1	120	17,1429	0	17,1429
0	0	1	dwa czynni	2	60	8,57143	0	8,57143
0	0	1	dwa czynni	2	360	51,4286	34,2857	17,1429
0	0	1	jeden czyn	2	150	21,4286	0	21,4286
1	0	1	trzy i więce	2	70	10	10	0
1	1	1	trzy i więce	3	0	0	0	0
0	1	1	trzy i więce	2	0	0	0	0
1	0	1	trzy i więce	2	180	25,7143	0	25,7143
0	0	0	jeden czyn	2	840	120	0	120
0	0	0	metabolicz	3	480	68,5714	34,2857	34,2857
0	0	0	metabolicz	2	0	0	0	0
0	0	1	jeden czyn	2	0	0	0	0
0	1	1	trzy i więce	2	40	5,71429	0	5,71429
0	0	1	dwa czynni	2	420	60	34,2857	25,7143
0	0	1	jeden czyn	3	0	0	0	0
1	0	1	trzy i więce	1	0	0	0	0
0	1	1	trzy i więce	1	0	0	0	0
0	1	1	trzy i więce	2	0	0	0	0
0	0	0	dwa czynni	1	60	8,57143	8,57143	0
0	0	1	dwa czynni	2	0	0	0	0
0	1	0	dwa czynni	3	300	42,8571	34,2857	8,57143
0	1	1	trzy i więce	3	420	60	17,1429	42,8571
1	0	1	trzy i więce	3	0	0	0	0
0	0	0	metabolicz	3	90	12,8571	0	12,8571

0	1	1	trzy i więce	1	240	34,2857	0	34,2857
0	0	1	jeden czyn	2	420	60	8,57143	51,4286
0	1	1	trzy i więce	2	120	17,1429	0	17,1429
1	1	1	trzy i więce	2	50	7,14286	7,14286	0
0	0	1	dwa czynni	1	240	34,2857	0	34,2857
0	0	0	dwa czynni	1	0	0	0	0
0	0	0	jeden czyn	2	60	8,57143	0	8,57143
1	0	0	trzy i więce	3	365	52,1429	20	32,1429
1	0	0	trzy i więce	1	0	0	0	0
0	1	1	trzy i więce	3	0	0	0	0
0	0	1	dwa czynni	3	60	8,57143	0	8,57143
0	0	0	jeden czyn	2	0	0	0	0
0	1	1	trzy i więce	2	225	32,1429	32,1429	0
1	1	1	trzy i więce	1	60	8,57143	0	8,57143
0	0	1	jeden czyn	1	0	0	0	0
1	1	1	trzy i więce	1	140	20	0	20
0	1	1	trzy i więce	2	180	25,7143	25,7143	0
1	1	1	trzy i więce	2	30	4,28571	0	4,28571
0	0	1	dwa czynni	2	0	0	0	0
1	1	1	trzy i więce	3	120	17,1429	0	17,1429
0	0	1	dwa czynni	2	120	17,1429	0	17,1429
0	0	0	dwa czynni	1	20	2,85714	0	2,85714
0	0	1	jeden czyn	1	0	0	0	0
0	0	0	jeden czyn	1	45	6,42857	6,42857	0
1	1	1	trzy i więce	3	210	30	30	0
0	1	1	trzy i więce	2	120	17,1429	8,57143	8,57143
0	0	1	dwa czynni	1	60	8,57143	0	8,57143
0	1	1	trzy i więce	3	840	120	0	120
0	0	1	jeden czyn	3	180	25,7143	0	25,7143
1	0	0	trzy i więce	3	0	0	0	0
0	0	1	trzy i więce	1	0	0	0	0
0	0	1	jeden czyn	1	0	0	0	0
1	0	0	trzy i więce	3	0	0	0	0
0	0	0	jeden czyn	2	270	38,5714	30	8,57143
0	1	0	trzy i więce	2	360	51,4286	0	51,4286
0	1	1	dwa czynni	2	210	30	0	30
0	0	1	dwa czynni	1	0	0	0	0
1	0	1	trzy i więce	2	20	2,85714	2,85714	0
1	0	1	trzy i więce	2	0	0	0	0
1	0	1	trzy i więce	3	120	17,1429	0	17,1429
0	0	0	jeden czyn	3	120	17,1429	0	17,1429
0	1	1	trzy i więce	2	60	8,57143	0	8,57143
1	1	1	trzy i więce	3	150	21,4286	0	21,4286
0	1	1	trzy i więce	2	180	25,7143	25,7143	0
0	1	1	trzy i więce	1	140	20	0	20
1	1	0	trzy i więce	1	240	34,2857	34,2857	0
1	0	1	trzy i więce	3	0	0	0	0
1	1	1	trzy i więce	3	90	12,8571	0	12,8571

1	0	1	trzy i więce	2	150	21,4286	0	21,4286
0	0	1	dwa czynni	3	0	0	0	0
0	0	1	dwa czynni	2	420	60	34,2857	25,7143
0	0	0	jeden czyn	3	180	25,7143	0	25,7143
0	0	0	dwa czynni	3	0	0	0	0
0	0	1	dwa czynni	2	345	49,2857	19,2857	30
0	0	0	jeden czyn	2	420	60	0	60
1	0	1	trzy i więce	2	210	30	30	0
0	0	1	dwa czynni	2	210	30	0	30
0	0	1	jeden czyn	1	120	17,1429	0	17,1429
0	0	0	metabolicz	2	120	17,1429	0	17,1429
1	1	0	trzy i więce	1	90	12,8571	12,8571	0
1	0	1	trzy i więce	1	945	135	15	120
1	1	1	trzy i więce	2	140	20	20	0
0	1	1	trzy i więce	2	0	0	0	0
0	0	0	metabolicz	1	0	0	0	0
1	0	1	trzy i więce	3	60	8,57143	0	8,57143
0	1	0	dwa czynni	2	120	17,1429	0	17,1429
0	1	1	dwa czynni	1	0	0	0	0
1	1	0	trzy i więce	2	0	0	0	0
0	1	1	trzy i więce	2	210	30	0	30
1	1	1	trzy i więce	1	240	34,2857	0	34,2857
1	0	1	trzy i więce	1	330	47,1429	12,8571	34,2857
0	0	1	dwa czynni	3	0	0	0	0
0	0	1	dwa czynni	2	0	0	0	0
0	1	1	dwa czynni	2	0	0	0	0
0	0	0	metabolicz	2	125	17,8571	0	17,8571
0	1	1	trzy i więce	3	60	8,57143	0	8,57143
1	0	1	trzy i więce	2	210	30	0	30
0	0	1	dwa czynni	2	0	0	0	0
0	0	0	metabolicz	2	0	0	0	0
1	0	1	trzy i więce	2	0	0	0	0
0	0	0	metabolicz	3	0	0	0	0
0	0	1	jeden czyn	2	390	55,7143	21,4286	34,2857
0	0	1	dwa czynni	1	0	0	0	0
0	0	1	dwa czynni	1	140	20	0	20
1	0	1	trzy i więce	2	0	0	0	0
0	0	0	metabolicz	3	0	0	0	0
0	1	1	trzy i więce	3	0	0	0	0
0	1	0	dwa czynni	2	0	0	0	0
1	0	0	dwa czynni	1	420	60	0	60
0	0	0	jeden czyn	2	840	120	0	120
0	1	0	dwa czynni	2	40	5,71429	5,71429	0
1	1	1	trzy i więce	1	0	0	0	0
1	0	1	trzy i więce	2	0	0	0	0
1	0	1	trzy i więce	2	90	12,8571	4,28571	8,57143
0	1	1	trzy i więce	3	0	0	0	0
0	0	1	dwa czynni	2	180	25,7143	0	25,7143

0	0	0	dwa czynni	3	360	51,4286	0	51,4286
1	1	1	trzy i więc	1	120	17,1429	0	17,1429
0	0	1	dwa czynni	2	60	8,57143	0	8,57143
0	0	0	metabolicz	1	840	120	0	120
1	0	1	trzy i więc	1	0	0	0	0
1	1	1	trzy i więc	2	0	0	0	0
0	0	1	dwa czynni	1	120	17,1429	0	17,1429
1	0	1	trzy i więc	3	0	0	0	0
0	0	1	dwa czynni	3	210	30	0	30
0	0	1	dwa czynni	2	60	8,57143	8,57143	0
1	0	1	trzy i więc	2	930	132,857	12,8571	120
0	0	1	dwa czynni	2	0	0	0	0
0	1	1	trzy i więc	2	70	10	1,42857	8,57143
0	0	1	jeden czyn	3	840	120	0	120
1	1	0	trzy i więc	3	315	45	0	45
1	0	1	trzy i więc	3	150	21,4286	12,8571	8,57143
0	0	1	dwa czynni	1	0	0	0	0
0	0	1	trzy i więc	2	180	25,7143	0	25,7143
0	0	1	trzy i więc	3	60	8,57143	0	8,57143
0	0	1	dwa czynni	3	510	72,8571	12,8571	60
0	1	0	dwa czynni	3	10	1,42857	1,42857	0
1	0	1	trzy i więc	3	0	0	0	0
0	0	1	dwa czynni	2	20	2,85714	2,85714	0
0	1	1	trzy i więc	2	120	17,1429	8,57143	8,57143
0	1	0	dwa czynni	3	60	8,57143	8,57143	0
0	0	1	dwa czynni	3	450	64,2857	12,8571	51,4286
0	0	1	jeden czyn	2	120	17,1429	0	17,1429
1	0	1	trzy i więc	2	0	0	0	0
1	1	1	trzy i więc	2	0	0	0	0
0	0	1	dwa czynni	2	0	0	0	0
0	0	1	jeden czyn	2	180	25,7143	0	25,7143
0	1	0	jeden czyn	3	0	0	0	0
0	0	1	dwa czynni	3	120	17,1429	0	17,1429
0	0	0	metabolicz	2	0	0	0	0
0	0	1	dwa czynni	2	780	111,429	51,4286	60
1	0	1	dwa czynni	2	0	0	0	0
0	0	0	jeden czyn	2	180	25,7143	8,57143	17,1429
1	1	1	trzy i więc	2	240	34,2857	0	34,2857
0	1	1	trzy i więc	1	140	20	0	20
0	0	0	jeden czyn	2	540	77,1429	25,7143	51,4286
0	1	1	trzy i więc	2	15	2,14286	0	2,14286
1	1	1	trzy i więc	2	120	17,1429	0	17,1429
1	0	0	dwa czynni	2	300	42,8571	17,1429	25,7143
1	0	1	trzy i więc	1	60	8,57143	8,57143	0
0	1	1	trzy i więc	1	0	0	0	0
1	1	1	trzy i więc	2	30	4,28571	0	4,28571
0	0	1	trzy i więc	2	80	11,4286	0	11,4286
0	1	1	trzy i więc	3	40	5,71429	0	5,71429

0	0	1	trzy i więce	2	0	0	0	0
0	0	1	trzy i więce	2	360	51,4286	0	51,4286
0	0	1	dwa czynni	2	240	34,2857	8,57143	25,7143
0	0	0	jeden czyn	3	0	0	0	0
0	1	0	dwa czynni	2	90	12,8571	0	12,8571
0	0	0	metabolicz	1	420	60	60	0
0	1	1	trzy i więce	2	840	120	0	120
0	0	1	dwa czynni	3	105	15	6,42857	8,57143
0	0	0	metabolicz	1	840	120	85,7143	34,2857
1	0	1	trzy i więce	2	120	17,1429	0	17,1429
0	0	1	dwa czynni	2	0	0	0	0
0	1	1	trzy i więce	1	60	8,57143	0	8,57143
0	0	1	dwa czynni	2	0	0	0	0
0	0	1	jeden czyn	2	0	0	0	0
1	1	1	trzy i więce	2	0	0	0	0
0	0	1	dwa czynni	3	0	0	0	0
0	0	0	metabolicz	2	330	47,1429	30	17,1429
0	1	1	trzy i więce	2	120	17,1429	17,1429	0
0	1	1	trzy i więce	1	420	60	0	60
0	1	1	dwa czynni	2	0	0	0	0
1	1	0	dwa czynni	3	210	30	30	0
0	1	1	trzy i więce	1	420	60	0	60
1	1	1	trzy i więce	3	540	77,1429	77,1429	0
0	0	1	jeden czyn	1	0	0	0	0
0	0	0	metabolicz	1	240	34,2857	17,1429	17,1429
0	1	1	trzy i więce	3	450	64,2857	12,8571	51,4286
0	0	1	dwa czynni	3	780	111,429	85,7143	25,7143
1	0	0	dwa czynni	2	0	0	0	0
0	0	1	jeden czyn	1	165	23,5714	23,5714	0
0	1	1	trzy i więce	2	0	0	0	0
0	0	1	jeden czyn	3	300	42,8571	0	42,8571
0	0	1	jeden czyn	1	0	0	0	0
0	0	1	dwa czynni	3	90	12,8571	12,8571	0
0	0	1	dwa czynni	2	60	8,57143	0	8,57143
1	0	1	trzy i więce	2	0	0	0	0
0	0	1	dwa czynni	2	0	0	0	0
0	0	1	trzy i więce	1	120	17,1429	0	17,1429
0	0	1	trzy i więce	1	420	60	0	60
1	0	0	trzy i więce	2	0	0	0	0
0	0	1	dwa czynni	3	720	102,857	102,857	0
0	0	0	metabolicz	2	120	17,1429	0	17,1429
1	1	0	trzy i więce	3	0	0	0	0
0	1	1	trzy i więce	1	0	0	0	0
0	0	1	dwa czynni	2	360	51,4286	0	51,4286
1	0	1	trzy i więce	2	20	2,85714	2,85714	0
0	1	1	trzy i więce	2	0	0	0	0
1	0	1	trzy i więce	2	0	0	0	0
0	0	1	dwa czynni	3	420	60	0	60

0	0	0	jeden czyn	3	60	8,57143	0	8,57143
0	0	0	jeden czyn	3	120	17,1429	0	17,1429
0	0	1	jeden czyn	2	600	85,7143	0	85,7143
0	0	1	trzy i więc	2	420	60	0	60
0	0	1	dwa czynni	2	120	17,1429	0	17,1429
0	1	1	trzy i więc	3	285	40,7143	6,42857	34,2857
0	0	1	dwa czynni	3	350	50	37,1429	12,8571
0	0	1	dwa czynni	1	180	25,7143	8,57143	17,1429
0	0	1	dwa czynni	2	60	8,57143	0	8,57143
0	0	0	jeden czyn	3	0	0	0	0
1	0	1	trzy i więc	2	650	92,8571	7,14286	85,7143
0	1	1	trzy i więc	1	60	8,57143	0	8,57143
1	0	0	dwa czynni	3	75	10,7143	10,7143	0
0	0	1	trzy i więc	3	90	12,8571	0	12,8571
1	0	1	trzy i więc	2	480	68,5714	0	68,5714
1	0	1	trzy i więc	2	0	0	0	0
0	1	0	dwa czynni	2	0	0	0	0
1	1	1	trzy i więc	2	0	0	0	0
0	1	1	trzy i więc	2	0	0	0	0
1	0	0	dwa czynni	1	0	0	0	0
1	1	1	trzy i więc	1	0	0	0	0
0	0	0	metabolicz	1	240	34,2857	25,7143	8,57143
0	0	0	jeden czyn	1	15	2,14286	2,14286	0
1	0	1	trzy i więc	2	100	14,2857	0	14,2857
0	1	1	trzy i więc	2	0	0	0	0
0	0	1	jeden czyn	2	420	60	25,7143	34,2857
0	0	0	metabolicz	3	210	30	0	30
0	0	1	dwa czynni	2	60	8,57143	0	8,57143
1	0	0	trzy i więc	2	240	34,2857	0	34,2857
0	1	1	trzy i więc	2	600	85,7143	51,4286	34,2857
0	0	1	jeden czyn	3	300	42,8571	0	42,8571
1	0	1	trzy i więc	1	60	8,57143	8,57143	0
1	1	1	trzy i więc	2	0	0	0	0
1	1	0	trzy i więc	1	136	19,4286	0	19,4286
0	1	1	trzy i więc	1	0	0	0	0
0	0	1	trzy i więc	3	60	8,57143	0	8,57143
1	0	1	trzy i więc	2	0	0	0	0
0	1	1	dwa czynni	3	65	9,28571	9,28571	0
1	0	0	jeden czyn	2	0	0	0	0
1	0	0	trzy i więc	2	230	32,8571	20	12,8571
1	0	1	trzy i więc	3	0	0	0	0
0	0	0	jeden czyn	1	30	4,28571	0	4,28571
0	1	1	trzy i więc	2	360	51,4286	0	51,4286
0	0	1	jeden czyn	3	240	34,2857	8,57143	25,7143
0	0	1	jeden czyn	3	750	107,143	4,28571	102,857
0	0	0	metabolicz	2	120	17,1429	17,1429	0
0	1	1	trzy i więc	2	465	66,4286	15	51,4286
0	0	1	trzy i więc	2	180	25,7143	0	25,7143

0	*0*	*0*	jeden czyn	3	*500*	*71,4286*	*20*	*51,4286*
1	*0*	*1*	trzy i więc	3	*1110*	*158,571*	*38,5714*	*120*
0	*0*	*0*	metabolicz	2	*120*	*17,1429*	*17,1429*	*0*
0	*1*	*1*	trzy i więc	3	*420*	*60*	*25,7143*	*34,2857*
0	*0*	*1*	dwa czynni	2	*150*	*21,4286*	*21,4286*	*0*
0	*0*	*1*	dwa czynni	2	*180*	*25,7143*	*8,57143*	*17,1429*
0	*0*	*1*	dwa czynni	2	*0*	*0*	*0*	*0*
1	*0*	*1*	trzy i więc	2	*0*	*0*	*0*	*0*
0	*0*	*1*	dwa czynni	3	*240*	*34,2857*	*0*	*34,2857*
0	*0*	*1*	dwa czynni	2	*0*	*0*	*0*	*0*
0	*1*	*1*	trzy i więc	1	*20*	*2,85714*	*0*	*2,85714*
0	*0*	*1*	trzy i więc	3	*0*	*0*	*0*	*0*
1	*1*	*1*	trzy i więc	2	*420*	*60*	*0*	*60*
1	*1*	*1*	trzy i więc	2	*210*	*30*	*30*	*0*
0	*0*	*1*	dwa czynni	3	*210*	*30*	*0*	*30*
0	*0*	*0*	jeden czyn	2	*510*	*72,8571*	*12,8571*	*60*
1	*0*	*1*	trzy i więc	1	*180*	*25,7143*	*0*	*25,7143*
0	*0*	*1*	dwa czynni	3	*270*	*38,5714*	*38,5714*	*0*
0	*0*	*1*	dwa czynni	2	*560*	*80*	*20*	*60*
1	*0*	*1*	trzy i więc	2	*300*	*42,8571*	*0*	*42,8571*
1	*1*	*1*	trzy i więc	2	*0*	*0*	*0*	*0*
0	*0*	*1*	dwa czynni	3	*0*	*0*	*0*	*0*
0	*0*	*0*	jeden czyn	3	*1380*	*197,143*	*17,1429*	*180*
1	*0*	*0*	trzy i więc	1	*240*	*34,2857*	*0*	*34,2857*
0	*0*	*1*	jeden czyn	3	*250*	*35,7143*	*10*	*25,7143*
0	*0*	*0*	metabolicz	2	*420*	*60*	*0*	*60*
0	*0*	*1*	dwa czynni	2	*240*	*34,2857*	*0*	*34,2857*
0	*0*	*0*	jeden czyn	1	*600*	*85,7143*	*0*	*85,7143*
0	*0*	*0*	jeden czyn	3	*165*	*23,5714*	*17,1429*	*6,42857*
0	*0*	*1*	jeden czyn	3	*0*	*0*	*0*	*0*
0	*0*	*1*	trzy i więc	2	*0*	*0*	*0*	*0*
1	*1*	*1*	trzy i więc	2	*270*	*38,5714*	*12,8571*	*25,7143*
1	*0*	*0*	dwa czynni	3	*240*	*34,2857*	*0*	*34,2857*
0	*0*	*1*	trzy i więc	3	*0*	*0*	*0*	*0*
0	*1*	*1*	trzy i więc	3	*120*	*17,1429*	*0*	*17,1429*
0	*0*	*1*	jeden czyn	3	*300*	*42,8571*	*0*	*42,8571*
1	*0*	*1*	trzy i więc	1	*120*	*17,1429*	*0*	*17,1429*
0	*0*	*0*	jeden czyn	1	*0*	*0*	*0*	*0*
0	*1*	*1*	dwa czynni	2	*840*	*120*	*0*	*120*
1	*0*	*1*	trzy i więc	1	*0*	*0*	*0*	*0*
1	*0*	*1*	trzy i więc	2	*900*	*128,571*	*0*	*128,571*
0	*0*	*0*	jeden czyn	2	*40*	*5,71429*	*0*	*5,71429*
0	*0*	*1*	dwa czynni	2	*90*	*12,8571*	*12,8571*	*0*
0	*0*	*1*	dwa czynni	3	*0*	*0*	*0*	*0*
0	*0*	*0*	jeden czyn	3	*0*	*0*	*0*	*0*
0	*0*	*1*	dwa czynni	3	*45*	*6,42857*	*0*	*6,42857*
0	*1*	*0*	dwa czynni	1	*0*	*0*	*0*	*0*
0	*0*	*1*	jeden czyn	3	*180*	*25,7143*	*0*	*25,7143*

0	1	1	trzy i więce	3	0	0	0	0
1	0	1	trzy i więce	2	420	60	0	60
0	0	1	dwa czynni	2	0	0	0	0
0	0	0	metabolicz	1	0	0	0	0
0	1	1	trzy i więce	2	840	120	0	120
0	1	1	trzy i więce	2	0	0	0	0
0	1	1	trzy i więce	3	0	0	0	0
1	0	0	trzy i więce	2	210	30	17,1429	12,8571
0	0	0	jeden czyn	3	0	0	0	0
0	1	1	trzy i więce	1	160	22,8571	0	22,8571
0	0	1	jeden czyn	1	420	60	0	60
1	0	0	dwa czynni	3	180	25,7143	17,1429	8,57143
0	0	1	dwa czynni	1	420	60	0	60
0	0	1	dwa czynni	3	0	0	0	0
1	0	1	trzy i więce	2	420	60	0	60
0	0	1	jeden czyn	2	0	0	0	0
1	1	0	trzy i więce	2	300	42,8571	42,8571	0
0	0	1	jeden czyn	2	60	8,57143	8,57143	0
0	0	1	dwa czynni	2	30	4,28571	0	4,28571
0	0	1	dwa czynni	2	165	23,5714	6,42857	17,1429
0	0	1	dwa czynni	1	240	34,2857	0	34,2857
0	0	0	jeden czyn	3	420	60	42,8571	17,1429
0	0	1	dwa czynni	3	345	49,2857	15	34,2857
1	1	1	trzy i więce	2	840	120	0	120
1	1	1	trzy i więce	3	0	0	0	0
1	0	1	trzy i więce	2	120	17,1429	0	17,1429
0	0	0	metabolicz	1	0	0	0	0
0	1	1	trzy i więce	1	60	8,57143	0	8,57143
0	1	1	trzy i więce	3	360	51,4286	0	51,4286
1	0	1	trzy i więce	3	0	0	0	0
0	0	0	jeden czyn	1	120	17,1429	0	17,1429
0	0	0	jeden czyn	3	45	6,42857	6,42857	0
1	1	1	trzy i więce	2	0	0	0	0
0	0	1	dwa czynni	3	360	51,4286	0	51,4286
1	1	1	trzy i więce	2	0	0	0	0
0	0	1	trzy i więce	3	150	21,4286	4,28571	17,1429
1	0	0	dwa czynni	2	60	8,57143	0	8,57143
0	0	0	jeden czyn	1	0	0	0	0
0	0	0	metabolicz	3	200	28,5714	28,5714	0
1	0	1	trzy i więce	3	0	0	0	0
1	0	0	trzy i więce	3	90	12,8571	12,8571	0
0	0	1	dwa czynni	2	0	0	0	0
1	0	1	dwa czynni	1	40	5,71429	0	5,71429
0	1	1	trzy i więce	1	0	0	0	0
0	0	1	jeden czyn	3	360	51,4286	0	51,4286
0	0	1	dwa czynni	2	0	0	0	0
0	0	0	metabolicz	2	105	15	0	15
1	1	1	trzy i więce	3	840	120	0	120

0	1	1	trzy i więce	2	150	21,4286	0	21,4286
0	0	1	jeden czyn	3	120	17,1429	0	17,1429
0	0	1	jeden czyn	2	105	15	15	0
1	0	0	dwa czynni	3	0	0	0	0
0	0	1	jeden czyn	1	105	15	15	0
1	0	0	trzy i więce	3	0	0	0	0
1	0	1	trzy i więce	1	0	0	0	0
0	0	1	dwa czynni	2	0	0	0	0
0	0	0	metabolicz	1	420	60	0	60
0	1	1	trzy i więce	3	90	12,8571	0	12,8571
0	0	1	dwa czynni	3	0	0	0	0
0	0	1	dwa czynni	3	160	22,8571	5,71429	17,1429
0	0	1	dwa czynni	1	840	120	0	120
0	0	1	dwa czynni	2	0	0	0	0
0	0	1	trzy i więce	2	120	17,1429	0	17,1429
0	0	1	trzy i więce	3	100	14,2857	0	14,2857
1	0	1	trzy i więce	3	420	60	0	60
0	0	0	jeden czyn	1	0	0	0	0
0	0	0	dwa czynni	2	0	0	0	0
1	0	0	trzy i więce	1	0	0	0	0
1	0	1	trzy i więce	2	420	60	0	60
0	0	0	metabolicz	1	660	94,2857	25,7143	68,5714
0	0	1	dwa czynni	2	240	34,2857	0	34,2857
0	0	1	dwa czynni	2	360	51,4286	0	51,4286
0	0	0	metabolicz	3	195	27,8571	15	12,8571
0	0	1	dwa czynni	3	90	12,8571	12,8571	0
0	1	1	trzy i więce	1	120	17,1429	0	17,1429
0	0	0	jeden czyn	3	0	0	0	0
1	0	0	trzy i więce	1	0	0	0	0
0	0	1	dwa czynni	2	90	12,8571	0	12,8571
1	0	1	trzy i więce	2	840	120	0	120
1	0	1	dwa czynni	1	0	0	0	0
0	1	1	trzy i więce	1	840	120	0	120
0	0	1	dwa czynni	2	120	17,1429	0	17,1429
0	0	1	jeden czyn	2	590	84,2857	34,2857	50
1	0	1	trzy i więce	2	0	0	0	0
0	0	1	dwa czynni	3	420	60	0	60
0	1	1	trzy i więce	2	0	0	0	0
0	0	1	dwa czynni	3	120	17,1429	0	17,1429
0	1	1	trzy i więce	2	60	8,57143	0	8,57143
0	0	1	dwa czynni	3	600	85,7143	0	85,7143
0	0	0	metabolicz	3	100	14,2857	14,2857	0
0	0	1	dwa czynni	3	420	60	0	60
0	0	1	dwa czynni	2	0	0	0	0
0	0	1	dwa czynni	2	195	27,8571	2,14286	25,7143
0	0	0	dwa czynni	3	0	0	0	0
1	1	1	trzy i więce	1	200	28,5714	0	28,5714
0	0	1	trzy i więce	1	60	8,57143	0	8,57143

0	0	1	dwa czynni	3	255	36,4286	19,2857	17,1429
0	1	1	trzy i więce	1	0	0	0	0
0	1	1	trzy i więce	1	0	0	0	0
0	0	1	jeden czyn	1	1080	154,286	51,4286	102,857
1	1	1	trzy i więce	2	30	4,28571	0	4,28571
1	1	1	trzy i więce	1	0	0	0	0
0	0	1	dwa czynni	2	0	0	0	0
0	0	0	metabolicz	2	0	0	0	0
0	0	1	jeden czyn	3	525	75	15	60
0	0	1	trzy i więce	1	0	0	0	0
0	0	1	dwa czynni	2	0	0	0	0
0	0	0	jeden czyn	3	120	17,1429	17,1429	0
0	1	0	jeden czyn	1	960	137,143	17,1429	120
1	1	1	trzy i więce	2	420	60	25,7143	34,2857
1	0	0	trzy i więce	3	60	8,57143	0	8,57143
0	0	1	dwa czynni	3	600	85,7143	85,7143	0
1	0	1	trzy i więce	1	0	0	0	0
0	0	0	jeden czyn	2	120	17,1429	0	17,1429
0	0	1	trzy i więce	2	420	60	0	60
1	1	0	trzy i więce	3	360	51,4286	0	51,4286
0	0	1	trzy i więce	2	0	0	0	0
0	0	0	metabolicz	2	210	30	0	30
1	0	1	trzy i więce	1	0	0	0	0
0	0	1	dwa czynni	2	0	0	0	0
1	0	0	dwa czynni	2	75	10,7143	0	10,7143
0	0	0	metabolicz	3	60	8,57143	0	8,57143
0	0	0	jeden czyn	2	135	19,2857	19,2857	0
0	0	0	metabolicz	2	190	27,1429	10	17,1429
1	0	1	trzy i więce	2	30	4,28571	0	4,28571
0	0	1	dwa czynni	2	0	0	0	0
0	1	1	trzy i więce	2	75	10,7143	0	10,7143
1	0	1	trzy i więce	3	0	0	0	0
0	0	0	metabolicz	2	0	0	0	0
0	0	0	metabolicz	2	240	34,2857	0	34,2857
1	0	1	trzy i więce	3	0	0	0	0
0	1	1	trzy i więce	2	240	34,2857	0	34,2857
0	0	1	dwa czynni	2	180	25,7143	0	25,7143
0	1	1	trzy i więce	2	0	0	0	0
1	0	1	trzy i więce	2	0	0	0	0
0	0	1	jeden czyn	3	90	12,8571	0	12,8571
1	0	1	trzy i więce	1	1260	180	0	180
1	0	0	dwa czynni	1	45	6,42857	0	6,42857
1	1	1	trzy i więce	3	60	8,57143	8,57143	0
1	0	1	trzy i więce	3	315	45	45	0
0	0	1	dwa czynni	2	360	51,4286	0	51,4286
1	0	0	jeden czyn	3	240	34,2857	17,1429	17,1429
0	0	1	dwa czynni	2	0	0	0	0
0	0	0	metabolicz	2	420	60	0	60

0	*0*	*0*	metabolicz	1	*0*	*0*	*0*	*0*
0	*0*	*0*	metabolicz	2	*0*	*0*	*0*	*0*
0	*0*	*1*	jeden czyn	2	*60*	*8,57143*	*0*	*8,57143*
0	*0*	*0*	dwa czynni	1	*840*	*120*	*0*	*120*
0	*0*	*1*	dwa czynni	3	*180*	*25,7143*	*0*	*25,7143*
1	*0*	*1*	trzy i więce	1	*180*	*25,7143*	*0*	*25,7143*
1	*0*	*0*	jeden czyn	1	*0*	*0*	*0*	*0*
1	*0*	*0*	dwa czynni	2	*360*	*51,4286*	*0*	*51,4286*
0	*0*	*1*	dwa czynni	2	*180*	*25,7143*	*0*	*25,7143*
0	*0*	*1*	dwa czynni	3	*60*	*8,57143*	*0*	*8,57143*
0	*0*	*1*	trzy i więce	2	*490*	*70*	*20*	*50*
1	*1*	*1*	trzy i więce	2	*180*	*25,7143*	*12,8571*	*12,8571*
0	*0*	*0*	jeden czyn	2	*180*	*25,7143*	*25,7143*	*0*
0	*0*	*1*	jeden czyn	3	*180*	*25,7143*	*0*	*25,7143*
1	*1*	*1*	trzy i więce	3	*195*	*27,8571*	*27,8571*	*0*
0	*0*	*1*	trzy i więce	2	*60*	*8,57143*	*0*	*8,57143*
0	*0*	*0*	jeden czyn	1	*0*	*0*	*0*	*0*
1	*1*	*0*	trzy i więce	1	*60*	*8,57143*	*8,57143*	*0*
0	*1*	*1*	trzy i więce	2	*0*	*0*	*0*	*0*
0	*0*	*1*	dwa czynni	1	*0*	*0*	*0*	*0*
0	*0*	*0*	metabolicz	2	*60*	*8,57143*	*0*	*8,57143*
0	*1*	*1*	trzy i więce	3	*0*	*0*	*0*	*0*
0	*0*	*1*	jeden czyn	1	*405*	*57,8571*	*6,42857*	*51,4286*
0	*0*	*0*	metabolicz	2	*300*	*42,8571*	*8,57143*	*34,2857*
1	*1*	*1*	trzy i więce	2	*0*	*0*	*0*	*0*
1	*0*	*0*	trzy i więce	2	*450*	*64,2857*	*60*	*4,28571*
1	*1*	*0*	trzy i więce	2	*0*	*0*	*0*	*0*
1	*1*	*1*	trzy i więce	2	*75*	*10,7143*	*0*	*10,7143*
0	*1*	*1*	trzy i więce	3	*210*	*30*	*0*	*30*
0	*1*	*1*	trzy i więce	2	*60*	*8,57143*	*0*	*8,57143*
0	*1*	*0*	jeden czyn	2	*0*	*0*	*0*	*0*
1	*0*	*1*	trzy i więce	2	*0*	*0*	*0*	*0*
0	*0*	*0*	metabolicz	1	*0*	*0*	*0*	*0*
0	*0*	*1*	dwa czynni	3	*60*	*8,57143*	*0*	*8,57143*
1	*0*	*0*	dwa czynni	2	*0*	*0*	*0*	*0*
0	*0*	*1*	dwa czynni	3	*0*	*0*	*0*	*0*
0	*1*	*1*	dwa czynni	1	*450*	*64,2857*	*60*	*4,28571*
0	*0*	*0*	jeden czyn	2	*0*	*0*	*0*	*0*
0	*0*	*0*	dwa czynni	3	*60*	*8,57143*	*0*	*8,57143*
0	*0*	*1*	dwa czynni	2	*240*	*34,2857*	*17,1429*	*17,1429*
0	*0*	*1*	dwa czynni	2	*90*	*12,8571*	*8,57143*	*4,28571*
0	*1*	*1*	trzy i więce	2	*360*	*51,4286*	*0*	*51,4286*
0	*0*	*0*	jeden czyn	3	*0*	*0*	*0*	*0*
0	*0*	*0*	dwa czynni	2	*0*	*0*	*0*	*0*
1	*0*	*1*	trzy i więce	3	*60*	*8,57143*	*0*	*8,57143*
1	*0*	*1*	trzy i więce	2	*0*	*0*	*0*	*0*
0	*1*	*1*	trzy i więce	3	*780*	*111,429*	*60*	*51,4286*
0	*0*	*0*	dwa czynni	2	*120*	*17,1429*	*0*	*17,1429*

0	0	1	dwa czynni	2	0	0	0	0
0	0	0	jeden czyn	2	0	0	0	0
0	0	1	dwa czynni	3	0	0	0	0
0	0	0	metabolicz	2	240	34,2857	0	34,2857
1	1	1	trzy i więc	3	0	0	0	0
0	1	1	trzy i więc	2	0	0	0	0
0	0	1	jeden czyn	2	120	17,1429	0	17,1429
0	1	1	trzy i więc	1	0	0	0	0
0	0	0	jeden czyn	2	0	0	0	0
1	1	1	trzy i więc	3	495	70,7143	10,7143	60
0	0	1	jeden czyn	3	0	0	0	0
0	0	1	dwa czynni	3	0	0	0	0
0	0	0	metabolicz	3	60	8,57143	0	8,57143
0	0	0	jeden czyn	1	360	51,4286	34,2857	17,1429
1	0	1	trzy i więc	2	60	8,57143	8,57143	0
0	0	1	dwa czynni	2	0	0	0	0
1	0	0	trzy i więc	1	700	100	40	60
1	0	0	trzy i więc	2	140	20	20	0
0	0	1	jeden czyn	2	180	25,7143	25,7143	0
1	1	1	trzy i więc	3	180	25,7143	0	25,7143
0	0	1	jeden czyn	2	120	17,1429	0	17,1429
0	0	1	dwa czynni	3	210	30	30	0
0	0	1	dwa czynni	2	1080	154,286	0	154,286
0	0	0	jeden czyn	2	180	25,7143	0	25,7143
0	1	1	trzy i więc	2	0	0	0	0
0	0	0	metabolicz	1	0	0	0	0
1	0	1	trzy i więc	2	420	60	0	60
0	1	1	trzy i więc	1	780	111,429	8,57143	102,857
0	0	1	dwa czynni	3	120	17,1429	0	17,1429
1	0	1	trzy i więc	2	60	8,57143	0	8,57143
1	0	1	trzy i więc	3	885	126,429	6,42857	120
1	1	0	trzy i więc	1	390	55,7143	30	25,7143
0	0	1	dwa czynni	1	0	0	0	0
0	0	0	jeden czyn	2	0	0	0	0
1	0	0	trzy i więc	3	190	27,1429	10	17,1429
0	0	1	dwa czynni	2	420	60	0	60
0	0	1	dwa czynni	3	420	60	0	60
0	1	1	trzy i więc	2	60	8,57143	0	8,57143
0	0	0	jeden czyn	2	690	98,5714	64,2857	34,2857
0	0	0	jeden czyn	1	0	0	0	0
0	0	0	jeden czyn	2	90	12,8571	0	12,8571
0	1	1	trzy i więc	2	150	21,4286	0	21,4286
0	0	0	metabolicz	1	0	0	0	0
0	1	1	trzy i więc	3	300	42,8571	30	12,8571
0	0	1	jeden czyn	3	60	8,57143	0	8,57143
0	0	1	dwa czynni	2	0	0	0	0
0	0	0	metabolicz	2	120	17,1429	17,1429	0
0	0	1	jeden czyn	3	540	77,1429	0	77,1429

0	0	0	jeden czyn	2	0	0	0	0
1	0	1	trzy i więc	3	210	30	0	30
1	1	1	trzy i więc	1	0	0	0	0
1	0	0	dwa czynni	2	0	0	0	0
0	0	0	dwa czynni	2	930	132,857	12,8571	120
1	0	0	dwa czynni	2	225	32,1429	15	17,1429
0	1	1	trzy i więc	3	40	5,71429	0	5,71429
1	0	0	trzy i więc	2	50	7,14286	0	7,14286
0	0	1	jeden czyn	1	0	0	0	0
1	1	0	trzy i więc	2	0	0	0	0
0	0	0	jeden czyn	2	80	11,4286	0	11,4286
0	1	0	dwa czynni	2	120	17,1429	0	17,1429
0	0	1	dwa czynni	2	0	0	0	0
1	1	1	trzy i więc	1	540	77,1429	0	77,1429
1	1	1	trzy i więc	3	420	60	0	60
0	0	1	dwa czynni	3	420	60	0	60
0	0	1	jeden czyn	2	45	6,42857	0	6,42857
0	0	1	dwa czynni	2	140	20	20	0
0	1	1	trzy i więc	3	120	17,1429	17,1429	0
0	1	0	trzy i więc	3	0	0	0	0
1	1	1	trzy i więc	2	0	0	0	0
0	0	1	dwa czynni	2	0	0	0	0
0	0	1	dwa czynni	2	1800	257,143	77,1429	180
0	0	0	metabolicz	2	60	8,57143	0	8,57143
0	1	1	trzy i więc	2	300	42,8571	8,57143	34,2857
0	0	1	dwa czynni	3	0	0	0	0
0	0	1	dwa czynni	2	45	6,42857	0	6,42857
0	0	0	metabolicz	3	480	68,5714	0	68,5714
0	0	0	metabolicz	3	0	0	0	0
1	1	1	trzy i więc	3	0	0	0	0
0	0	1	jeden czyn	1	0	0	0	0
0	0	0	metabolicz	3	60	8,57143	8,57143	0
0	1	0	jeden czyn	2	60	8,57143	0	8,57143
0	0	1	dwa czynni	2	0	0	0	0
1	0	1	trzy i więc	1	360	51,4286	0	51,4286
0	1	1	trzy i więc	1	0	0	0	0
0	1	0	dwa czynni	2	105	15	0	15
1	1	1	trzy i więc	3	20	2,85714	0	2,85714
1	0	1	trzy i więc	2	90	12,8571	12,8571	0
0	0	0	dwa czynni	2	210	30	0	30
1	0	1	trzy i więc	3	60	8,57143	0	8,57143
1	0	1	trzy i więc	3	60	8,57143	8,57143	0
1	1	1	trzy i więc	2	45	6,42857	0	6,42857
0	1	1	trzy i więc	1	840	120	0	120
1	0	0	trzy i więc	1	0	0	0	0
0	0	1	jeden czyn	1	0	0	0	0
0	0	0	metabolicz	1	75	10,7143	6,42857	4,28571
0	0	1	trzy i więc	3	0	0	0	0

0	0	0	jeden czyn	2	0	0	0	0
0	0	1	jeden czyn	3	30	4,28571	4,28571	0
0	1	1	trzy i więc	1	0	0	0	0
0	0	1	dwa czynni	2	120	17,1429	0	17,1429
0	0	1	dwa czynni	3	240	34,2857	8,57143	25,7143
1	0	1	trzy i więc	2	0	0	0	0
0	0	0	metabolicz	2	0	0	0	0
0	0	1	trzy i więc	1	120	17,1429	17,1429	0
0	0	1	dwa czynni	2	105	15	15	0
0	1	0	dwa czynni	2	120	17,1429	0	17,1429
0	0	1	jeden czyn	2	360	51,4286	0	51,4286
0	1	0	trzy i więc	1	0	0	0	0
0	0	0	jeden czyn	1	0	0	0	0
0	0	0	jeden czyn	3	30	4,28571	0	4,28571
0	0	0	jeden czyn	2	0	0	0	0
0	0	1	dwa czynni	3	0	0	0	0
0	0	1	dwa czynni	1	120	17,1429	0	17,1429
0	0	1	trzy i więc	3	0	0	0	0
1	0	1	trzy i więc	3	180	25,7143	0	25,7143
1	0	1	trzy i więc	3	120	17,1429	17,1429	0
0	0	0	jeden czyn	3	0	0	0	0
0	0	1	jeden czyn	3	810	115,714	12,8571	102,857
1	1	0	trzy i więc	2	480	68,5714	34,2857	34,2857
1	0	1	trzy i więc	2	105	15	0	15
0	1	1	trzy i więc	2	285	40,7143	15	25,7143
1	0	1	trzy i więc	2	90	12,8571	0	12,8571
0	0	0	metabolicz	1	60	8,57143	0	8,57143
0	1	1	trzy i więc	3	0	0	0	0
1	0	1	trzy i więc	2	0	0	0	0
0	0	1	dwa czynni	3	380	54,2857	20	34,2857
1	0	0	dwa czynni	2	90	12,8571	0	12,8571
0	0	1	jeden czyn	3	210	30	30	0
1	1	1	trzy i więc	1	90	12,8571	12,8571	0
0	0	1	jeden czyn	1	0	0	0	0
0	0	1	jeden czyn	1	60	8,57143	8,57143	0
0	1	1	dwa czynni	1	840	120	0	120
0	0	1	dwa czynni	2	150	21,4286	0	21,4286
0	0	1	trzy i więc	2	405	57,8571	6,42857	51,4286
0	1	1	trzy i więc	2	75	10,7143	6,42857	4,28571
1	1	1	trzy i więc	2	0	0	0	0
1	0	1	trzy i więc	2	420	60	42,8571	17,1429
1	0	1	trzy i więc	1	0	0	0	0
0	1	1	trzy i więc	2	0	0	0	0
0	1	1	trzy i więc	2	330	47,1429	34,2857	12,8571
0	1	1	trzy i więc	3	120	17,1429	17,1429	0
0	0	1	dwa czynni	3	0	0	0	0
0	0	1	jeden czyn	1	435	62,1429	45	17,1429
0	1	1	trzy i więc	2	225	32,1429	15	17,1429

1	1	1	trzy i więce	3	0	0	0	0
0	1	1	trzy i więce	3	0	0	0	0
1	0	0	dwa czynni	2	120	17,1429	17,1429	0
0	1	1	trzy i więce	1	180	25,7143	0	25,7143
0	0	1	dwa czynni	2	180	25,7143	25,7143	0
0	1	1	trzy i więce	2	105	15	0	15
0	0	1	dwa czynni	2	195	27,8571	12,8571	15
1	0	1	trzy i więce	1	120	17,1429	0	17,1429
1	0	1	trzy i więce	2	0	0	0	0
0	0	1	jeden czyn	3	0	0	0	0
1	0	1	trzy i więce	2	80	11,4286	8,57143	2,85714
1	1	1	trzy i więce	2	330	47,1429	7,14286	40
0	1	1	trzy i więce	2	0	0	0	0
0	0	1	dwa czynni	3	130	18,5714	10	8,57143
0	0	1	jeden czyn	1	420	60	0	60
0	1	0	jeden czyn	1	90	12,8571	0	12,8571
0	1	1	trzy i więce	2	0	0	0	0
0	0	1	jeden czyn	2	210	30	12,8571	17,1429
0	0	1	dwa czynni	1	120	17,1429	0	17,1429
0	0	1	dwa czynni	2	180	25,7143	0	25,7143
0	0	0	jeden czyn	3	0	0	0	0
0	0	1	dwa czynni	2	0	0	0	0
1	0	1	trzy i więce	2	0	0	0	0
0	0	1	dwa czynni	2	0	0	0	0
1	0	1	trzy i więce	2	0	0	0	0
0	0	1	dwa czynni	2	0	0	0	0
0	0	1	dwa czynni	2	240	34,2857	0	34,2857
1	0	0	trzy i więce	1	0	0	0	0
0	0	1	dwa czynni	3	150	21,4286	0	21,4286
1	0	1	trzy i więce	2	0	0	0	0
0	0	1	dwa czynni	2	180	25,7143	0	25,7143
1	1	1	trzy i więce	2	0	0	0	0
0	0	1	jeden czyn	3	360	51,4286	17,1429	34,2857
0	0	1	jeden czyn	2	120	17,1429	17,1429	0
0	0	1	dwa czynni	3	0	0	0	0
0	0	0	jeden czyn	2	70	10	10	0
0	1	1	trzy i więce	3	20	2,85714	0	2,85714
0	0	1	jeden czyn	2	0	0	0	0
0	0	0	jeden czyn	3	240	34,2857	0	34,2857
1	1	1	trzy i więce	2	0	0	0	0
1	0	0	dwa czynni	1	0	0	0	0
0	0	1	trzy i więce	3	420	60	0	60
0	0	0	metabolicz	2	120	17,1429	0	17,1429
0	1	1	trzy i więce	3	0	0	0	0
0	0	1	dwa czynni	1	240	34,2857	0	34,2857
0	0	1	dwa czynni	2	0	0	0	0
0	0	0	dwa czynni	2	0	0	0	0
1	0	0	trzy i więce	3	30	4,28571	0	4,28571

0	0	0	metabolicz	3	120	17,1429	17,1429	0
1	0	1	trzy i więce	1	0	0	0	0
0	0	1	dwa czynni	2	300	42,8571	42,8571	0
0	1	1	trzy i więce	3	210	30	30	0
1	0	1	trzy i więce	2	30	4,28571	0	4,28571
0	0	1	dwa czynni	2	0	0	0	0
0	1	1	trzy i więce	3	120	17,1429	0	17,1429
0	1	1	trzy i więce	2	0	0	0	0
0	0	1	trzy i więce	1	630	90	0	90
0	0	1	jeden czyn	2	180	25,7143	0	25,7143
1	1	1	trzy i więce	3	60	8,57143	0	8,57143
0	0	1	jeden czyn	1	240	34,2857	0	34,2857
0	1	0	jeden czyn	1	0	0	0	0
0	1	1	dwa czynni	1	200	28,5714	20	8,57143
0	0	1	dwa czynni	2	0	0	0	0
1	0	1	trzy i więce	3	0	0	0	0
0	0	1	dwa czynni	3	180	25,7143	0	25,7143
1	1	1	trzy i więce	2	0	0	0	0
0	0	0	metabolicz	2	30	4,28571	0	4,28571
0	1	1	trzy i więce	2	120	17,1429	0	17,1429
0	0	1	dwa czynni	2	0	0	0	0
0	0	0	metabolicz	2	420	60	0	60
1	0	1	trzy i więce	1	0	0	0	0
0	0	1	dwa czynni	2	300	42,8571	0	42,8571
0	0	1	jeden czyn	2	210	30	4,28571	25,7143
0	1	1	trzy i więce	1	210	30	0	30
0	0	1	trzy i więce	2	60	8,57143	0	8,57143
1	0	1	trzy i więce	3	60	8,57143	0	8,57143
0	0	0	jeden czyn	2	0	0	0	0
0	0	1	dwa czynni	1	240	34,2857	0	34,2857
0	0	1	dwa czynni	2	60	8,57143	0	8,57143
1	0	1	trzy i więce	2	180	25,7143	25,7143	0
0	0	0	metabolicz	1	120	17,1429	17,1429	0
1	1	1	trzy i więce	2	0	0	0	0
1	1	1	trzy i więce	1	120	17,1429	0	17,1429
1	0	1	trzy i więce	3	80	11,4286	0	11,4286
0	0	0	jeden czyn	2	0	0	0	0
1	1	1	trzy i więce	1	0	0	0	0
1	1	1	trzy i więce	2	120	17,1429	0	17,1429
0	0	0	jeden czyn	2	60	8,57143	0	8,57143
0	0	1	dwa czynni	2	120	17,1429	0	17,1429
0	0	0	jeden czyn	1	60	8,57143	8,57143	0
1	0	1	trzy i więce	3	420	60	0	60
0	1	0	dwa czynni	2	1260	180	0	180
0	0	1	dwa czynni	2	0	0	0	0
0	0	0	metabolicz	2	360	51,4286	25,7143	25,7143
0	1	1	trzy i więce	2	0	0	0	0
0	0	0	jeden czyn	3	840	120	0	120

0	1	1	dwa czynni	2	210	30	0	30
0	0	1	dwa czynni	2	420	60	0	60
0	1	1	trzy i więce	3	70	10	10	0
0	1	1	trzy i więce	3	70	10	0	10
0	1	0	dwa czynni	3	465	66,4286	15	51,4286
0	0	1	trzy i więce	1	0	0	0	0
0	1	1	trzy i więce	2	180	25,7143	0	25,7143
0	0	1	dwa czynni	2	90	12,8571	12,8571	0
0	0	0	jeden czyn	1	0	0	0	0
1	1	1	trzy i więce	2	0	0	0	0
1	0	1	trzy i więce	3	300	42,8571	0	42,8571
1	0	0	trzy i więce	2	0	0	0	0
1	1	1	trzy i więce	3	0	0	0	0
0	0	0	jeden czyn	2	0	0	0	0
1	0	1	trzy i więce	2	180	25,7143	0	25,7143
1	0	1	trzy i więce	2	0	0	0	0
0	0	1	jeden czyn	3	120	17,1429	5,71429	11,4286
0	1	1	trzy i więce	2	0	0	0	0
0	0	0	jeden czyn	2	600	85,7143	0	85,7143
1	0	1	trzy i więce	1	0	0	0	0
0	0	1	jeden czyn	1	120	17,1429	0	17,1429
1	0	1	trzy i więce	1	0	0	0	0
0	0	1	jeden czyn	3	80	11,4286	11,4286	0
1	0	0	jeden czyn	2	0	0	0	0
0	0	1	dwa czynni	3	840	120	0	120
0	0	1	dwa czynni	2	465	66,4286	15	51,4286
0	0	1	dwa czynni	2	120	17,1429	0	17,1429
0	1	0	dwa czynni	1	0	0	0	0
0	0	1	dwa czynni	2	0	0	0	0
0	0	1	dwa czynni	2	1260	180	0	180
0	0	0	jeden czyn	2	360	51,4286	0	51,4286
0	0	0	dwa czynni	3	0	0	0	0
1	1	1	trzy i więce	2	210	30	30	0
1	0	1	trzy i więce	1	420	60	0	60
0	0	1	dwa czynni	3	420	60	0	60
1	0	0	dwa czynni	1	0	0	0	0
0	0	0	metabolicz	3	135	19,2857	4,28571	15
0	1	1	trzy i więce	2	0	0	0	0
0	0	1	dwa czynni	2	0	0	0	0
1	0	1	trzy i więce	3	180	25,7143	25,7143	0
1	1	1	trzy i więce	2	0	0	0	0
0	0	1	dwa czynni	3	0	0	0	0
0	0	1	dwa czynni	3	240	34,2857	0	34,2857
1	1	1	trzy i więce	1	0	0	0	0
0	0	0	jeden czyn	2	1860	265,714	85,7143	180
1	1	1	trzy i więce	1	30	4,28571	4,28571	0
0	1	0	trzy i więce	3	255	36,4286	19,2857	17,1429
0	0	1	jeden czyn	3	0	0	0	0

0	0	1	trzy i więc	2	180	25,7143	0	25,7143
1	0	0	dwa czynni	3	120	17,1429	17,1429	0
0	0	0	jeden czyn	1	180	25,7143	0	25,7143
1	0	1	dwa czynni	2	360	51,4286	17,1429	34,2857
1	0	1	trzy i więc	1	210	30	0	30
0	0	1	jeden czyn	3	240	34,2857	25,7143	8,57143
0	0	1	dwa czynni	3	180	25,7143	0	25,7143
0	1	0	dwa czynni	1	140	20	0	20
1	1	1	trzy i więc	2	0	0	0	0
0	0	0	metabolicz	2	60	8,57143	0	8,57143
0	0	1	jeden czyn	1	120	17,1429	0	17,1429
0	1	1	trzy i więc	1	0	0	0	0
0	0	0	jeden czyn	2	30	4,28571	0	4,28571
0	0	1	trzy i więc	1	60	8,57143	0	8,57143
0	0	1	dwa czynni	2	240	34,2857	0	34,2857
0	1	1	trzy i więc	2	0	0	0	0
0	1	1	dwa czynni	3	255	36,4286	10,7143	25,7143
0	0	0	metabolicz	2	0	0	0	0
1	0	1	trzy i więc	2	0	0	0	0
1	0	1	trzy i więc	2	0	0	0	0
0	0	1	dwa czynni	3	0	0	0	0
0	0	1	trzy i więc	1	0	0	0	0
1	1	1	trzy i więc	3	540	77,1429	0	77,1429
0	0	1	dwa czynni	2	840	120	0	120
0	0	1	jeden czyn	3	390	55,7143	51,4286	4,28571
0	0	1	dwa czynni	2	30	4,28571	0	4,28571
0	0	1	dwa czynni	3	0	0	0	0
1	0	0	trzy i więc	2	105	15	6,42857	8,57143
0	0	1	trzy i więc	2	60	8,57143	0	8,57143
0	0	1	dwa czynni	1	180	25,7143	0	25,7143
0	0	1	jeden czyn	1	80	11,4286	0	11,4286
0	1	1	trzy i więc	2	0	0	0	0
0	0	1	jeden czyn	2	360	51,4286	0	51,4286
0	1	1	trzy i więc	2	0	0	0	0
1	0	0	trzy i więc	1	0	0	0	0
0	0	1	trzy i więc	2	0	0	0	0
0	1	1	trzy i więc	2	90	12,8571	0	12,8571
0	0	1	dwa czynni	2	90	12,8571	0	12,8571
1	0	1	trzy i więc	2	210	30	0	30
0	0	1	dwa czynni	3	0	0	0	0
1	0	1	trzy i więc	3	0	0	0	0
0	0	0	jeden czyn	3	0	0	0	0
1	0	1	trzy i więc	2	300	42,8571	0	42,8571
1	1	1	trzy i więc	2	450	64,2857	12,8571	51,4286
0	0	1	jeden czyn	2	30	4,28571	0	4,28571
1	1	1	trzy i więc	3	0	0	0	0
1	0	1	trzy i więc	3	390	55,7143	4,28571	51,4286
0	0	1	dwa czynni	3	120	17,1429	8,57143	8,57143

0	1	1	trzy i więce	1	450	64,2857	21,4286	42,8571
0	0	1	dwa czynni	3	360	51,4286	0	51,4286
0	1	1	trzy i więce	2	0	0	0	0
0	1	1	trzy i więce	3	495	70,7143	10,7143	60
0	1	1	trzy i więce	2	0	0	0	0
0	0	0	metabolicz	2	165	23,5714	6,42857	17,1429
0	0	1	dwa czynni	2	360	51,4286	0	51,4286
1	1	1	trzy i więce	1	0	0	0	0
0	1	1	trzy i więce	3	250	35,7143	10	25,7143
0	0	0	metabolicz	2	0	0	0	0
0	0	1	dwa czynni	1	60	8,57143	0	8,57143
0	0	1	jeden czyn	2	0	0	0	0
0	1	1	trzy i więce	3	120	17,1429	0	17,1429
1	0	1	trzy i więce	2	180	25,7143	0	25,7143
0	0	1	dwa czynni	2	0	0	0	0
1	0	0	dwa czynni	2	840	120	0	120
0	1	1	trzy i więce	2	60	8,57143	0	8,57143
0	0	0	metabolicz	1	420	60	0	60
0	0	1	trzy i więce	3	0	0	0	0
1	1	1	trzy i więce	1	0	0	0	0
0	0	1	trzy i więce	1	0	0	0	0
1	0	1	trzy i więce	3	0	0	0	0
1	1	1	trzy i więce	3	490	70	10	60
0	1	1	trzy i więce	2	165	23,5714	6,42857	17,1429
0	0	0	jeden czyn	3	300	42,8571	34,2857	8,57143
0	0	1	dwa czynni	2	0	0	0	0
1	1	1	trzy i więce	2	420	60	0	60
0	0	1	dwa czynni	3	0	0	0	0
0	0	1	dwa czynni	3	120	17,1429	0	17,1429
1	1	1	trzy i więce	1	0	0	0	0
0	0	1	jeden czyn	3	0	0	0	0
1	0	0	trzy i więce	2	0	0	0	0
1	0	1	trzy i więce	3	180	25,7143	0	25,7143
1	1	1	trzy i więce	2	90	12,8571	0	12,8571
0	0	0	metabolicz	2	60	8,57143	0	8,57143
1	0	0	trzy i więce	3	360	51,4286	51,4286	0
0	1	1	trzy i więce	2	0	0	0	0
0	0	0	jeden czyn	2	420	60	0	60
0	1	1	dwa czynni	2	60	8,57143	0	8,57143
1	0	0	trzy i więce	1	0	0	0	0
0	0	1	dwa czynni	3	0	0	0	0
0	1	1	trzy i więce	3	20	2,85714	0	2,85714
0	0	1	dwa czynni	3	210	30	0	30
0	0	1	dwa czynni	3	420	60	0	60
0	0	0	jeden czyn	1	420	60	0	60
0	0	1	jeden czyn	3	180	25,7143	25,7143	0
1	1	0	trzy i więce	3	240	34,2857	0	34,2857
0	0	1	jeden czyn	3	0	0	0	0

0	1	0	jeden czyn	2	150	21,4286	21,4286	0
0	0	0	jeden czyn	2	420	60	0	60
0	0	0	metabolicz	1	420	60	0	60
1	0	1	trzy i więc	2	0	0	0	0
0	1	1	dwa czynni	1	0	0	0	0
0	0	0	jeden czyn	1	0	0	0	0
0	0	0	metabolicz	2	240	34,2857	0	34,2857
0	0	1	dwa czynni	3	0	0	0	0
0	0	1	dwa czynni	3	0	0	0	0
0	0	1	jeden czyn	2	420	60	0	60
0	0	1	dwa czynni	3	0	0	0	0
0	0	1	dwa czynni	2	240	34,2857	0	34,2857
1	0	1	trzy i więc	1	315	45	10,7143	34,2857
0	0	1	dwa czynni	2	180	25,7143	0	25,7143
0	0	1	dwa czynni	2	60	8,57143	0	8,57143
0	0	1	dwa czynni	3	0	0	0	0
0	0	1	jeden czyn	3	80	11,4286	11,4286	0
0	0	0	metabolicz	1	50	7,14286	7,14286	0
1	0	1	trzy i więc	3	120	17,1429	17,1429	0
0	0	0	jeden czyn	1	210	30	0	30
0	0	0	jeden czyn	2	100	14,2857	0	14,2857
1	0	1	trzy i więc	2	0	0	0	0
1	0	1	trzy i więc	2	840	120	0	120
0	0	1	trzy i więc	1	0	0	0	0
0	0	0	dwa czynni	3	0	0	0	0
1	1	1	trzy i więc	1	120	17,1429	0	17,1429
1	0	1	trzy i więc	3	0	0	0	0
1	0	0	trzy i więc	3	0	0	0	0
1	1	1	trzy i więc	2	0	0	0	0
0	0	1	trzy i więc	3	735	105	45	60
1	0	1	trzy i więc	2	0	0	0	0
0	0	1	dwa czynni	2	0	0	0	0
0	0	0	metabolicz	1	0	0	0	0
0	0	1	dwa czynni	3	0	0	0	0
0	0	0	jeden czyn	2	0	0	0	0
1	0	1	trzy i więc	2	0	0	0	0
0	0	1	jeden czyn	2	0	0	0	0
0	0	1	dwa czynni	2	180	25,7143	17,1429	8,57143
1	1	1	trzy i więc	3	230	32,8571	2,85714	30
0	0	0	jeden czyn	2	60	8,57143	0	8,57143
0	1	1	trzy i więc	3	600	85,7143	0	85,7143
1	0	0	dwa czynni	2	125	17,8571	15	2,85714
1	0	1	trzy i więc	2	180	25,7143	0	25,7143
0	1	1	trzy i więc	2	75	10,7143	2,14286	8,57143
1	0	1	trzy i więc	1	120	17,1429	0	17,1429
0	0	1	dwa czynni	3	120	17,1429	0	17,1429
1	1	0	trzy i więc	3	90	12,8571	0	12,8571
0	0	1	dwa czynni	2	630	90	0	90

0	0	0	metabolicz	1	60	8,57143	0	8,57143
0	1	1	trzy i więce	2	0	0	0	0
0	0	0	jeden czyn	2	900	128,571	0	128,571
0	0	1	dwa czynni	1	0	0	0	0
0	0	0	metabolicz	2	90	12,8571	0	12,8571
0	0	1	dwa czynni	2	0	0	0	0
0	0	1	jeden czyn	3	210	30	21,4286	8,57143
1	1	1	trzy i więce	2	0	0	0	0
1	0	1	trzy i więce	2	75	10,7143	10,7143	0
0	0	1	dwa czynni	2	270	38,5714	25,7143	12,8571
0	1	1	trzy i więce	2	0	0	0	0
0	0	1	dwa czynni	2	120	17,1429	0	17,1429
0	1	1	trzy i więce	2	15	2,14286	0	2,14286
0	0	0	jeden czyn	3	240	34,2857	0	34,2857
1	1	1	trzy i więce	2	510	72,8571	30	42,8571
0	0	1	dwa czynni	1	180	25,7143	0	25,7143
0	0	1	dwa czynni	2	100	14,2857	0	14,2857
0	0	1	dwa czynni	2	210	30	0	30
0	0	0	jeden czyn	2	600	85,7143	85,7143	0
1	1	0	trzy i więce	2	0	0	0	0
0	0	1	dwa czynni	1	0	0	0	0
0	0	1	dwa czynni	2	270	38,5714	17,1429	21,4286
0	1	1	trzy i więce	2	0	0	0	0
0	1	1	dwa czynni	3	120	17,1429	0	17,1429
0	1	0	dwa czynni	3	0	0	0	0
1	1	1	trzy i więce	2	0	0	0	0
0	0	0	jeden czyn	2	540	77,1429	8,57143	68,5714
1	0	1	trzy i więce	2	0	0	0	0
1	0	1	trzy i więce	1	720	102,857	102,857	0
0	1	1	trzy i więce	3	120	17,1429	0	17,1429
0	1	0	trzy i więce	2	0	0	0	0
0	0	1	jeden czyn	1	420	60	0	60
1	0	1	trzy i więce	2	60	8,57143	8,57143	0
0	0	1	trzy i więce	1	480	68,5714	0	68,5714
0	0	1	dwa czynni	3	30	4,28571	0	4,28571
0	0	1	dwa czynni	2	0	0	0	0
0	0	1	trzy i więce	3	0	0	0	0
1	1	1	trzy i więce	2	0	0	0	0
0	0	0	jeden czyn	2	300	42,8571	42,8571	0
1	1	1	trzy i więce	2	0	0	0	0
1	0	0	dwa czynni	2	30	4,28571	0	4,28571
0	0	0	jeden czyn	3	150	21,4286	0	21,4286
0	0	0	metabolicz	1	420	60	0	60
0	0	0	metabolicz	3	60	8,57143	0	8,57143
0	0	1	trzy i więce	3	0	0	0	0
0	0	0	jeden czyn	1	60	8,57143	0	8,57143
0	1	0	dwa czynni	3	90	12,8571	12,8571	0
0	0	0	metabolicz	2	180	25,7143	0	25,7143

0	0	1	jeden czyn	1	0	0	0	0
0	0	1	jeden czyn	2	840	120	0	120
1	1	1	trzy i więce	3	360	51,4286	8,57143	42,8571
0	0	1	dwa czynni	2	420	60	0	60
1	1	1	trzy i więce	3	180	25,7143	0	25,7143
0	1	1	trzy i więce	1	165	23,5714	19,2857	4,28571
0	0	0	jeden czyn	3	0	0	0	0
1	0	1	trzy i więce	2	420	60	60	0
1	0	1	trzy i więce	2	0	0	0	0
0	1	1	trzy i więce	1	60	8,57143	0	8,57143
1	0	1	trzy i więce	1	240	34,2857	0	34,2857
0	0	1	trzy i więce	1	15	2,14286	2,14286	0
0	0	1	jeden czyn	2	360	51,4286	25,7143	25,7143
0	0	1	dwa czynni	3	120	17,1429	17,1429	0
0	1	1	trzy i więce	1	0	0	0	0
0	1	0	jeden czyn	1	0	0	0	0
0	0	1	jeden czyn	2	120	17,1429	0	17,1429
0	0	1	dwa czynni	3	0	0	0	0
1	1	1	trzy i więce	2	270	38,5714	12,8571	25,7143
0	0	1	dwa czynni	2	300	42,8571	17,1429	25,7143
0	0	1	dwa czynni	2	0	0	0	0
1	0	1	trzy i więce	2	180	25,7143	0	25,7143
0	1	1	trzy i więce	1	60	8,57143	0	8,57143
0	0	1	dwa czynni	2	240	34,2857	17,1429	17,1429
0	0	1	dwa czynni	1	390	55,7143	30	25,7143
0	0	1	dwa czynni	2	0	0	0	0
0	0	0	jeden czyn	2	140	20	20	0
0	0	0	jeden czyn	1	0	0	0	0
1	0	0	dwa czynni	2	0	0	0	0
0	0	1	jeden czyn	2	0	0	0	0
0	0	1	jeden czyn	2	360	51,4286	0	51,4286
0	0	0	metabolicz	1	240	34,2857	0	34,2857
0	0	0	jeden czyn	2	180	25,7143	0	25,7143
0	0	0	jeden czyn	3	240	34,2857	0	34,2857
1	0	0	dwa czynni	2	240	34,2857	34,2857	0
0	1	0	dwa czynni	2	180	25,7143	0	25,7143
0	0	0	metabolicz	2	120	17,1429	0	17,1429
0	1	0	dwa czynni	3	0	0	0	0
0	0	0	metabolicz	2	180	25,7143	0	25,7143
0	1	0	jeden czyn	3	0	0	0	0
0	0	1	dwa czynni	3	335	47,8571	2,85714	45
1	0	0	dwa czynni	3	0	0	0	0
0	0	0	jeden czyn	3	0	0	0	0
0	0	0	jeden czyn	1	180	25,7143	0	25,7143
0	1	1	trzy i więce	1	345	49,2857	6,42857	42,8571
0	1	0	jeden czyn	1	210	30	0	30
0	0	1	dwa czynni	1	0	0	0	0
0	0	1	dwa czynni	2	120	17,1429	0	17,1429

0	1	1	trzy i więce	2	0	0	0	0
1	0	1	trzy i więce	1	225	32,1429	0	32,1429
0	1	1	trzy i więce	2	60	8,57143	8,57143	0
1	1	1	trzy i więce	2	0	0	0	0
0	0	1	dwa czynni	3	240	34,2857	0	34,2857
0	0	1	dwa czynni	2	25	3,57143	0	3,57143
0	1	1	trzy i więce	2	0	0	0	0
0	0	1	dwa czynni	3	0	0	0	0
0	1	1	trzy i więce	2	300	42,8571	34,2857	8,57143
1	0	0	dwa czynni	2	240	34,2857	17,1429	17,1429
0	0	1	jeden czyn	2	180	25,7143	0	25,7143
0	1	1	trzy i więce	1	120	17,1429	17,1429	0
1	0	0	trzy i więce	3	240	34,2857	0	34,2857
0	0	1	dwa czynni	2	450	64,2857	30	34,2857
0	0	1	dwa czynni	3	240	34,2857	0	34,2857
0	1	0	dwa czynni	2	885	126,429	6,42857	120
0	0	1	dwa czynni	2	0	0	0	0
1	0	0	trzy i więce	1	70	10	10	0
0	0	1	jeden czyn	2	0	0	0	0
0	0	1	dwa czynni	1	0	0	0	0
0	0	0	jeden czyn	3	420	60	0	60
0	1	0	dwa czynni	1	120	17,1429	0	17,1429
0	0	1	dwa czynni	1	60	8,57143	0	8,57143
0	0	1	dwa czynni	3	600	85,7143	51,4286	34,2857
0	0	0	jeden czyn	3	420	60	0	60
0	0	1	dwa czynni	3	0	0	0	0
0	0	1	dwa czynni	2	210	30	30	0
1	0	0	trzy i więce	2	500	71,4286	11,4286	60
1	1	1	trzy i więce	3	450	64,2857	34,2857	30
0	0	0	jeden czyn	3	0	0	0	0
0	0	1	dwa czynni	3	90	12,8571	0	12,8571
1	0	1	trzy i więce	2	0	0	0	0
0	0	0	jeden czyn	3	330	47,1429	17,1429	30
1	0	1	trzy i więce	2	0	0	0	0
0	0	1	jeden czyn	1	0	0	0	0
0	0	1	dwa czynni	2	180	25,7143	0	25,7143
0	0	1	jeden czyn	3	780	111,429	51,4286	60
0	0	1	dwa czynni	2	90	12,8571	12,8571	0
0	1	0	dwa czynni	1	10	1,42857	0	1,42857
1	1	1	trzy i więce	2	900	128,571	8,57143	120
0	0	0	metabolicz	3	420	60	25,7143	34,2857
0	1	0	trzy i więce	2	0	0	0	0
1	1	1	trzy i więce	2	0	0	0	0
1	0	1	trzy i więce	2	225	32,1429	15	17,1429
1	0	0	jeden czyn	3	240	34,2857	0	34,2857
1	1	1	trzy i więce	2	70	10	10	0
0	0	1	dwa czynni	3	0	0	0	0
0	0	1	jeden czyn	3	240	34,2857	0	34,2857

0	0	1	dwa czynni	2	0	0	0	0
0	0	0	jeden czyn	2	735	105	45	60
0	0	1	dwa czynni	1	0	0	0	0
1	1	1	trzy i więc	1	300	42,8571	0	42,8571
0	0	1	jeden czyn	3	0	0	0	0
1	1	1	trzy i więc	2	0	0	0	0
0	1	1	dwa czynni	1	140	20	0	20
0	0	0	jeden czyn	1	60	8,57143	0	8,57143
1	1	1	trzy i więc	2	0	0	0	0
1	0	1	trzy i więc	1	0	0	0	0
0	0	1	dwa czynni	2	30	4,28571	0	4,28571
1	1	1	trzy i więc	2	0	0	0	0
0	0	0	jeden czyn	1	0	0	0	0
0	0	0	metabolicz	2	100	14,2857	0	14,2857
0	0	0	jeden czyn	1	0	0	0	0
1	0	1	trzy i więc	3	0	0	0	0
0	0	1	dwa czynni	2	0	0	0	0
1	0	0	dwa czynni	2	210	30	0	30
1	0	0	dwa czynni	3	0	0	0	0
1	0	1	trzy i więc	2	60	8,57143	0	8,57143
0	0	1	dwa czynni	3	0	0	0	0
0	0	1	dwa czynni	2	210	30	0	30
0	0	0	metabolicz	3	0	0	0	0
0	0	1	dwa czynni	3	280	40	0	40
0	0	1	dwa czynni	3	0	0	0	0
1	1	1	trzy i więc	3	100	14,2857	14,2857	0
0	1	1	trzy i więc	2	0	0	0	0
0	0	1	dwa czynni	3	0	0	0	0
1	0	1	trzy i więc	1	180	25,7143	0	25,7143
0	1	1	trzy i więc	1	0	0	0	0
1	1	1	trzy i więc	3	295	42,1429	42,1429	0
0	0	0	jeden czyn	2	0	0	0	0
1	1	1	trzy i więc	2	0	0	0	0
0	1	1	trzy i więc	2	120	17,1429	8,57143	8,57143
0	1	1	trzy i więc	2	60	8,57143	0	8,57143
0	0	0	jeden czyn	2	360	51,4286	0	51,4286
0	0	1	trzy i więc	3	0	0	0	0
0	0	0	jeden czyn	2	120	17,1429	0	17,1429
1	0	0	dwa czynni	3	360	51,4286	0	51,4286
0	1	1	trzy i więc	1	60	8,57143	0	8,57143
0	0	1	dwa czynni	2	360	51,4286	51,4286	0
0	0	1	jeden czyn	1	15	2,14286	0	2,14286
0	0	0	metabolicz	2	240	34,2857	0	34,2857
0	0	1	jeden czyn	3	120	17,1429	0	17,1429
0	0	0	jeden czyn	1	0	0	0	0
0	0	0	jeden czyn	1	120	17,1429	0	17,1429
0	1	0	dwa czynni	3	150	21,4286	21,4286	0
1	0	1	trzy i więc	3	900	128,571	8,57143	120

1	0	1	trzy i więce	2	45	6,42857	0	6,42857
1	0	0	trzy i więce	3	90	12,8571	0	12,8571
0	1	1	dwa czynni	3	0	0	0	0
1	1	1	trzy i więce	2	210	30	17,1429	12,8571
0	0	1	dwa czynni	3	60	8,57143	8,57143	0
0	0	0	jeden czyn	1	180	25,7143	21,4286	4,28571
1	0	1	trzy i więce	2	120	17,1429	0	17,1429
1	0	0	jeden czyn	3	300	42,8571	0	42,8571
0	0	1	dwa czynni	2	120	17,1429	0	17,1429
0	1	1	trzy i więce	2	30	4,28571	0	4,28571
0	0	0	jeden czyn	3	0	0	0	0
0	0	0	jeden czyn	2	255	36,4286	6,42857	30
1	0	1	trzy i więce	3	60	8,57143	0	8,57143
0	0	0	dwa czynni	2	0	0	0	0
1	1	1	trzy i więce	2	180	25,7143	0	25,7143
0	0	1	jeden czyn	2	0	0	0	0
0	0	1	dwa czynni	1	165	23,5714	6,42857	17,1429
0	0	0	jeden czyn	2	120	17,1429	0	17,1429
0	0	1	dwa czynni	2	200	28,5714	0	28,5714
0	1	1	trzy i więce	2	60	8,57143	0	8,57143
0	0	1	jeden czyn	3	420	60	0	60
0	0	0	metabolicz	3	0	0	0	0
0	0	0	metabolicz	2	150	21,4286	12,8571	8,57143
0	0	1	jeden czyn	1	840	120	0	120
1	0	1	trzy i więce	2	420	60	0	60
1	0	0	trzy i więce	2	0	0	0	0
0	0	0	jeden czyn	2	180	25,7143	0	25,7143
0	0	1	jeden czyn	1	120	17,1429	0	17,1429
0	0	1	dwa czynni	3	30	4,28571	0	4,28571
0	1	1	trzy i więce	2	60	8,57143	8,57143	0
0	0	0	jeden czyn	1	0	0	0	0
0	0	0	jeden czyn	2	0	0	0	0
0	1	1	trzy i więce	3	0	0	0	0
1	0	1	trzy i więce	2	450	64,2857	30	34,2857
1	1	0	trzy i więce	3	240	34,2857	0	34,2857
0	0	1	dwa czynni	2	0	0	0	0
0	0	1	jeden czyn	1	0	0	0	0
0	0	1	jeden czyn	2	0	0	0	0
0	1	0	dwa czynni	1	0	0	0	0
0	0	1	dwa czynni	2	120	17,1429	0	17,1429
0	0	0	jeden czyn	3	0	0	0	0
1	0	1	dwa czynni	3	120	17,1429	8,57143	8,57143
0	0	1	jeden czyn	3	180	25,7143	0	25,7143
1	0	1	trzy i więce	1	0	0	0	0
0	0	0	jeden czyn	2	0	0	0	0
1	0	1	trzy i więce	3	60	8,57143	0	8,57143
0	0	1	jeden czyn	1	105	15	15	0
1	0	1	trzy i więce	2	300	42,8571	25,7143	17,1429

0	0	0	metabolicz	1	0	0	0	0
1	1	0	trzy i więce	3	120	17,1429	0	17,1429
0	0	1	jeden czyn	3	270	38,5714	12,8571	25,7143
1	1	1	trzy i więce	1	0	0	0	0
1	1	1	trzy i więce	1	420	60	0	60
0	0	0	metabolicz	2	100	14,2857	5,71429	8,57143
1	0	1	trzy i więce	2	0	0	0	0
0	0	0	jeden czyn	2	180	25,7143	0	25,7143
0	0	0	jeden czyn	2	900	128,571	68,5714	60
1	0	1	trzy i więce	1	0	0	0	0
0	0	1	trzy i więce	1	60	8,57143	0	8,57143
0	0	1	trzy i więce	2	0	0	0	0
0	1	1	trzy i więce	3	600	85,7143	51,4286	34,2857
0	0	1	dwa czynni	3	0	0	0	0
0	1	1	trzy i więce	3	120	17,1429	17,1429	0
0	0	1	dwa czynni	2	120	17,1429	0	17,1429
0	1	1	trzy i więce	1	210	30	0	30
0	1	0	dwa czynni	3	0	0	0	0
0	1	0	dwa czynni	2	120	17,1429	0	17,1429
0	0	1	jeden czyn	2	270	38,5714	30	8,57143
1	0	0	dwa czynni	2	0	0	0	0
0	1	1	trzy i więce	3	0	0	0	0
1	1	1	trzy i więce	3	0	0	0	0
0	0	1	dwa czynni	2	0	0	0	0
1	0	1	trzy i więce	1	140	20	0	20
1	0	1	trzy i więce	2	120	17,1429	0	17,1429
0	0	1	jeden czyn	1	0	0	0	0
0	0	1	dwa czynni	2	60	8,57143	0	8,57143
0	0	1	trzy i więce	2	660	94,2857	8,57143	85,7143
0	0	0	metabolicz	3	0	0	0	0
0	0	1	jeden czyn	3	0	0	0	0
0	0	1	jeden czyn	3	465	66,4286	6,42857	60
1	0	1	trzy i więce	3	0	0	0	0
0	0	1	dwa czynni	2	240	34,2857	0	34,2857
0	0	1	dwa czynni	2	60	8,57143	0	8,57143
1	0	0	trzy i więce	2	210	30	30	0
1	0	1	trzy i więce	2	0	0	0	0
0	0	0	metabolicz	3	0	0	0	0
1	0	1	trzy i więce	3	45	6,42857	6,42857	0
0	0	1	trzy i więce	1	0	0	0	0
0	0	1	dwa czynni	1	840	120	0	120
1	0	1	trzy i więce	3	420	60	0	60
0	0	0	jeden czyn	3	150	21,4286	4,28571	17,1429
0	1	1	trzy i więce	2	0	0	0	0
0	1	1	trzy i więce	2	300	42,8571	0	42,8571
0	0	1	jeden czyn	1	0	0	0	0
1	0	0	trzy i więce	2	480	68,5714	34,2857	34,2857
0	0	1	dwa czynni	3	420	60	0	60

0	0	1	jeden czyn	2	60	8,57143	0	8,57143
1	1	1	trzy i więce	1	0	0	0	0
0	0	0	metabolicz	1	0	0	0	0
1	0	0	dwa czynni	2	120	17,1429	0	17,1429
1	0	1	trzy i więce	3	350	50	0	50
0	1	1	trzy i więce	2	0	0	0	0
0	1	1	trzy i więce	2	240	34,2857	0	34,2857
0	1	1	trzy i więce	3	420	60	0	60
0	0	1	jeden czyn	2	510	72,8571	21,4286	51,4286
0	1	1	trzy i więce	2	0	0	0	0
1	1	1	trzy i więce	3	0	0	0	0
0	0	1	dwa czynni	3	0	0	0	0
0	0	0	dwa czynni	1	30	4,28571	0	4,28571
0	0	1	dwa czynni	2	60	8,57143	0	8,57143
0	0	1	dwa czynni	1	60	8,57143	0	8,57143
1	0	1	trzy i więce	2	210	30	0	30
0	0	0	metabolicz	2	0	0	0	0
1	1	1	trzy i więce	1	0	0	0	0
0	0	1	dwa czynni	2	0	0	0	0
0	0	1	dwa czynni	3	0	0	0	0
0	0	0	jeden czyn	3	0	0	0	0
0	0	1	dwa czynni	3	0	0	0	0
0	1	1	trzy i więce	3	195	27,8571	10,7143	17,1429
0	0	1	dwa czynni	2	0	0	0	0
1	1	1	trzy i więce	2	0	0	0	0
0	0	1	dwa czynni	2	540	77,1429	77,1429	0
0	0	1	dwa czynni	3	210	30	0	30
0	1	1	dwa czynni	2	80	11,4286	11,4286	0
1	0	1	dwa czynni	1	0	0	0	0
1	0	1	trzy i więce	1	0	0	0	0
0	0	1	dwa czynni	3	0	0	0	0
0	0	0	jeden czyn	2	0	0	0	0
0	0	0	metabolicz	2	180	25,7143	17,1429	8,57143
0	1	1	trzy i więce	2	0	0	0	0
0	1	1	trzy i więce	2	240	34,2857	0	34,2857
0	0	0	metabolicz	2	0	0	0	0
1	1	1	trzy i więce	2	0	0	0	0
0	1	0	jeden czyn	3	1680	240	60	180
1	0	1	trzy i więce	3	210	30	4,28571	25,7143
1	0	1	trzy i więce	3	0	0	0	0
1	0	1	trzy i więce	2	180	25,7143	0	25,7143
1	0	1	trzy i więce	3	30	4,28571	4,28571	0
1	0	0	dwa czynni	1	0	0	0	0
0	1	1	trzy i więce	3	120	17,1429	0	17,1429
0	0	1	dwa czynni	2	840	120	0	120
0	0	1	jeden czyn	1	0	0	0	0
0	1	0	jeden czyn	2	0	0	0	0
0	1	1	trzy i więce	2	420	60	51,4286	8,57143

0	0	1	jeden czyn	1	240	34,2857	0	34,2857
1	0	1	trzy i więc	2	150	21,4286	0	21,4286
1	0	0	dwa czynni	3	0	0	0	0
0	0	1	jeden czyn	3	90	12,8571	4,28571	8,57143
0	0	0	dwa czynni	2	720	102,857	0	102,857
0	1	1	trzy i więc	2	120	17,1429	0	17,1429
1	0	1	trzy i więc	2	218	31,1429	14	17,1429
0	0	1	dwa czynni	2	0	0	0	0
0	0	1	dwa czynni	3	120	17,1429	0	17,1429
0	0	1	dwa czynni	2	120	17,1429	0	17,1429
1	1	1	trzy i więc	2	0	0	0	0
0	1	0	jeden czyn	2	240	34,2857	0	34,2857
1	0	1	trzy i więc	3	0	0	0	0
0	0	1	dwa czynni	3	0	0	0	0
0	0	1	dwa czynni	1	0	0	0	0
1	0	0	dwa czynni	1	90	12,8571	12,8571	0
1	1	1	trzy i więc	1	120	17,1429	0	17,1429
0	0	0	metabolicz	2	0	0	0	0
1	0	1	trzy i więc	1	15	2,14286	2,14286	0
0	0	1	dwa czynni	2	480	68,5714	34,2857	34,2857
0	0	1	dwa czynni	2	550	78,5714	18,5714	60
0	0	0	metabolicz	2	60	8,57143	0	8,57143
0	0	0	metabolicz	2	140	20	20	0
0	0	1	dwa czynni	3	0	0	0	0
0	0	1	dwa czynni	2	300	42,8571	0	42,8571
1	0	1	trzy i więc	2	630	90	0	90
0	0	0	jeden czyn	2	0	0	0	0
0	1	1	trzy i więc	2	90	12,8571	0	12,8571
0	0	0	metabolicz	1	0	0	0	0
0	1	1	trzy i więc	2	120	17,1429	0	17,1429
0	1	0	dwa czynni	2	450	64,2857	12,8571	51,4286
0	1	1	trzy i więc	2	0	0	0	0
0	0	1	trzy i więc	1	0	0	0	0
1	0	0	dwa czynni	1	60	8,57143	0	8,57143
0	0	0	metabolicz	2	300	42,8571	0	42,8571
0	0	1	jeden czyn	2	225	32,1429	15	17,1429
0	0	1	dwa czynni	3	60	8,57143	0	8,57143
1	0	1	trzy i więc	2	105	15	6,42857	8,57143
1	1	1	trzy i więc	2	0	0	0	0
1	0	0	dwa czynni	3	420	60	8,57143	51,4286
1	0	0	dwa czynni	1	0	0	0	0
0	1	1	trzy i więc	2	280	40	0	40
0	0	1	dwa czynni	1	150	21,4286	0	21,4286
1	0	1	trzy i więc	2	120	17,1429	8,57143	8,57143
0	0	1	dwa czynni	2	90	12,8571	0	12,8571
1	1	1	trzy i więc	2	0	0	0	0
1	0	1	trzy i więc	3	210	30	0	30
1	0	1	trzy i więc	2	60	8,57143	0	8,57143

0	0	1	dwa czynni	2	210	30	8,57143	21,4286
0	0	0	jeden czyn	3	45	6,42857	2,14286	4,28571
0	0	1	dwa czynni	2	0	0	0	0
1	0	0	dwa czynni	2	180	25,7143	17,1429	8,57143
1	0	1	trzy i więce	2	140	20	20	0
0	0	0	metabolicz	2	150	21,4286	17,1429	4,28571
0	0	1	dwa czynni	2	480	68,5714	25,7143	42,8571
0	0	0	metabolicz	2	0	0	0	0
0	1	1	trzy i więce	3	0	0	0	0
0	0	1	dwa czynni	3	420	60	0	60
1	0	1	trzy i więce	2	120	17,1429	8,57143	8,57143
0	0	1	dwa czynni	1	185	26,4286	15	11,4286
1	0	1	trzy i więce	1	105	15	6,42857	8,57143
0	0	0	jeden czyn	1	120	17,1429	0	17,1429
0	0	1	dwa czynni	2	60	8,57143	0	8,57143
0	0	0	jeden czyn	2	0	0	0	0
0	1	1	trzy i więce	1	0	0	0	0
0	0	1	dwa czynni	2	660	94,2857	34,2857	60
0	0	0	jeden czyn	2	0	0	0	0
0	1	1	trzy i więce	1	0	0	0	0
0	0	0	metabolicz	2	300	42,8571	17,1429	25,7143
0	0	0	jeden czyn	1	90	12,8571	0	12,8571
0	0	0	metabolicz	3	360	51,4286	0	51,4286
0	0	0	jeden czyn	3	30	4,28571	0	4,28571
0	0	1	dwa czynni	3	200	28,5714	2,85714	25,7143
0	0	1	dwa czynni	3	0	0	0	0
1	1	0	trzy i więce	1	120	17,1429	0	17,1429
0	1	0	trzy i więce	1	0	0	0	0
0	1	1	trzy i więce	2	60	8,57143	0	8,57143
0	0	1	jeden czyn	2	200	28,5714	11,4286	17,1429
0	1	1	trzy i więce	2	0	0	0	0
1	0	1	trzy i więce	3	420	60	0	60
0	1	1	trzy i więce	2	120	17,1429	0	17,1429
0	0	1	dwa czynni	1	0	0	0	0
0	1	0	trzy i więce	1	120	17,1429	0	17,1429
0	0	0	jeden czyn	2	0	0	0	0
0	0	0	jeden czyn	2	220	31,4286	10	21,4286
0	0	1	jeden czyn	2	0	0	0	0
0	0	1	dwa czynni	2	465	66,4286	15	51,4286
0	0	0	jeden czyn	2	40	5,71429	5,71429	0
1	0	1	trzy i więce	2	0	0	0	0
0	0	1	dwa czynni	1	0	0	0	0
0	0	1	dwa czynni	3	360	51,4286	0	51,4286
0	1	1	trzy i więce	3	60	8,57143	0	8,57143
0	0	0	jeden czyn	3	0	0	0	0
0	0	0	metabolicz	3	0	0	0	0
0	0	0	jeden czyn	2	70	10	4,28571	5,71429
0	0	1	dwa czynni	3	60	8,57143	0	8,57143

1	0	1	trzy i więce	2	0	0	0	0
0	0	0	metabolicz	2	30	4,28571	0	4,28571
0	0	0	metabolicz	1	420	60	0	60
0	1	1	trzy i więce	2	180	25,7143	0	25,7143
0	0	1	dwa czynni	2	0	0	0	0
1	0	1	trzy i więce	2	840	120	0	120
1	1	1	trzy i więce	2	60	8,57143	4,28571	4,28571
1	0	1	trzy i więce	3	360	51,4286	0	51,4286
0	0	0	jeden czyn	3	120	17,1429	17,1429	0
1	0	0	dwa czynni	2	315	45	0	45
0	0	1	dwa czynni	1	240	34,2857	17,1429	17,1429
0	1	1	trzy i więce	2	0	0	0	0
0	0	0	jeden czyn	2	120	17,1429	0	17,1429
0	0	0	metabolicz	2	45	6,42857	6,42857	0
1	1	1	trzy i więce	3	0	0	0	0
1	1	1	trzy i więce	3	210	30	0	30
0	0	1	jeden czyn	3	480	68,5714	0	68,5714
0	0	1	dwa czynni	2	60	8,57143	0	8,57143
1	0	1	trzy i więce	2	240	34,2857	0	34,2857
0	0	1	dwa czynni	3	140	20	0	20
0	0	1	dwa czynni	2	840	120	0	120
0	0	1	dwa czynni	1	0	0	0	0
0	0	1	dwa czynni	1	0	0	0	0
0	0	0	jeden czyn	3	840	120	0	120
1	0	0	trzy i więce	2	60	8,57143	0	8,57143
1	0	1	trzy i więce	2	0	0	0	0
1	0	1	trzy i więce	3	45	6,42857	6,42857	0
0	0	0	jeden czyn	3	1680	240	180	60
0	0	1	dwa czynni	2	120	17,1429	0	17,1429
0	0	1	jeden czyn	2	420	60	0	60
1	0	1	trzy i więce	1	0	0	0	0
0	0	0	jeden czyn	2	120	17,1429	17,1429	0
0	1	0	trzy i więce	2	60	8,57143	0	8,57143
1	1	1	trzy i więce	3	0	0	0	0
0	1	1	dwa czynni	3	240	34,2857	0	34,2857
0	0	1	dwa czynni	2	60	8,57143	0	8,57143
0	0	1	dwa czynni	2	80	11,4286	8,57143	2,85714
0	0	1	dwa czynni	2	120	17,1429	8,57143	8,57143
0	0	1	dwa czynni	2	210	30	0	30
0	0	0	jeden czyn	2	420	60	0	60
1	0	0	dwa czynni	2	0	0	0	0
0	1	1	dwa czynni	2	120	17,1429	8,57143	8,57143
0	0	1	dwa czynni	3	360	51,4286	0	51,4286
0	1	0	dwa czynni	3	120	17,1429	0	17,1429
0	0	1	dwa czynni	3	120	17,1429	0	17,1429
0	0	1	trzy i więce	1	210	30	0	30
0	0	1	dwa czynni	2	180	25,7143	17,1429	8,57143
1	0	0	trzy i więce	3	2280	325,714	205,714	120

1	0	1	trzy i więce	2	60	8,57143	0	8,57143
0	1	1	trzy i więce	1	0	0	0	0
1	0	0	trzy i więce	1	60	8,57143	0	8,57143
1	1	1	trzy i więce	2	600	85,7143	0	85,7143
0	0	0	jeden czyn	2	120	17,1429	0	17,1429
1	0	1	trzy i więce	3	300	42,8571	0	42,8571
0	0	0	jeden czyn	2	270	38,5714	21,4286	17,1429
0	1	1	trzy i więce	3	300	42,8571	17,1429	25,7143
0	1	0	jeden czyn	2	780	111,429	51,4286	60
0	0	1	dwa czynni	2	0	0	0	0
0	0	1	dwa czynni	2	400	57,1429	14,2857	42,8571
0	0	0	jeden czyn	2	105	15	0	15
0	0	1	trzy i więce	1	60	8,57143	0	8,57143
0	0	1	dwa czynni	2	0	0	0	0
0	0	1	jeden czyn	2	540	77,1429	0	77,1429
0	0	1	dwa czynni	2	120	17,1429	0	17,1429
1	0	1	trzy i więce	3	720	102,857	0	102,857
0	0	1	dwa czynni	2	0	0	0	0
0	0	1	jeden czyn	3	180	25,7143	8,57143	17,1429
1	0	0	dwa czynni	2	420	60	60	0
1	0	0	trzy i więce	2	135	19,2857	0	19,2857
0	0	0	metabolicz	2	180	25,7143	17,1429	8,57143
0	0	1	jeden czyn	2	360	51,4286	17,1429	34,2857
0	0	1	dwa czynni	3	0	0	0	0
1	0	1	trzy i więce	1	150	21,4286	0	21,4286
0	0	0	metabolicz	3	600	85,7143	55,7143	30
0	0	1	dwa czynni	1	0	0	0	0
0	1	0	jeden czyn	2	0	0	0	0
0	0	0	jeden czyn	3	210	30	30	0
1	1	1	trzy i więce	2	0	0	0	0
0	0	1	jeden czyn	3	240	34,2857	0	34,2857
0	0	1	dwa czynni	2	0	0	0	0
1	0	0	dwa czynni	1	300	42,8571	25,7143	17,1429
1	0	1	trzy i więce	3	840	120	0	120
0	0	1	trzy i więce	2	240	34,2857	0	34,2857
0	0	0	jeden czyn	2	180	25,7143	0	25,7143
0	0	0	jeden czyn	3	90	12,8571	0	12,8571
0	0	1	jeden czyn	2	0	0	0	0
0	1	1	trzy i więce	3	0	0	0	0
0	1	0	dwa czynni	2	0	0	0	0
0	0	0	dwa czynni	2	0	0	0	0
0	0	0	jeden czyn	2	0	0	0	0
0	0	0	jeden czyn	3	0	0	0	0
1	0	1	dwa czynni	2	180	25,7143	8,57143	17,1429
0	0	1	dwa czynni	2	60	8,57143	0	8,57143
1	0	0	dwa czynni	1	420	60	0	60
0	0	1	dwa czynni	2	30	4,28571	0	4,28571
1	1	1	trzy i więce	3	1050	150	90	60

0	0	1	dwa czynni	2	0	0	0	0
0	0	0	metabolicz	2	360	51,4286	17,1429	34,2857
0	1	1	trzy i więc	3	90	12,8571	0	12,8571
1	0	1	trzy i więc	1	180	25,7143	25,7143	0
0	0	1	dwa czynni	1	0	0	0	0
1	0	1	trzy i więc	3	15	2,14286	2,14286	0
0	0	1	dwa czynni	3	300	42,8571	0	42,8571
1	0	1	dwa czynni	2	300	42,8571	8,57143	34,2857
0	0	1	dwa czynni	2	100	14,2857	8,57143	5,71429
1	0	1	trzy i więc	1	120	17,1429	0	17,1429
0	0	0	dwa czynni	1	945	135	15	120
0	1	1	trzy i więc	3	0	0	0	0
0	1	1	trzy i więc	3	360	51,4286	25,7143	25,7143
0	0	0	metabolicz	3	120	17,1429	0	17,1429
1	0	1	trzy i więc	2	180	25,7143	0	25,7143
0	0	1	trzy i więc	2	0	0	0	0
0	1	1	trzy i więc	1	0	0	0	0
0	1	1	trzy i więc	2	20	2,85714	2,85714	0
1	0	1	trzy i więc	3	315	45	0	45
0	0	0	jeden czyn	3	365	52,1429	35	17,1429
0	0	0	jeden czyn	2	120	17,1429	0	17,1429
1	0	0	jeden czyn	2	210	30	8,57143	21,4286
0	1	1	trzy i więc	2	0	0	0	0
0	0	1	jeden czyn	3	180	25,7143	25,7143	0
0	1	1	trzy i więc	3	0	0	0	0
0	1	1	dwa czynni	3	360	51,4286	0	51,4286
0	0	0	jeden czyn	3	360	51,4286	0	51,4286
1	1	1	trzy i więc	3	0	0	0	0
0	0	0	metabolicz	2	440	62,8571	20	42,8571
1	0	1	trzy i więc	2	0	0	0	0
0	0	1	trzy i więc	1	0	0	0	0
1	1	1	trzy i więc	2	280	40	25,7143	14,2857
0	0	1	jeden czyn	2	200	28,5714	11,4286	17,1429
0	0	0	jeden czyn	2	0	0	0	0
0	1	1	trzy i więc	1	780	111,429	102,857	8,57143
0	0	0	jeden czyn	1	0	0	0	0
0	0	0	jeden czyn	3	0	0	0	0
0	0	0	metabolicz	1	150	21,4286	21,4286	0
1	0	1	trzy i więc	3	350	50	0	50
0	0	0	jeden czyn	2	40	5,71429	0	5,71429
1	0	1	trzy i więc	3	240	34,2857	17,1429	17,1429
0	0	1	dwa czynni	2	0	0	0	0
0	0	0	metabolicz	1	0	0	0	0
0	0	1	trzy i więc	3	120	17,1429	0	17,1429
0	1	1	trzy i więc	2	0	0	0	0
0	0	0	jeden czyn	3	120	17,1429	0	17,1429
0	0	1	dwa czynni	3	0	0	0	0
0	0	0	metabolicz	2	240	34,2857	0	34,2857

1	0	1	trzy i więce	3	360	51,4286	0	51,4286
0	0	1	dwa czynni	1	0	0	0	0
1	1	1	trzy i więce	1	300	42,8571	0	42,8571
0	0	1	jeden czyn	3	420	60	0	60
0	1	0	dwa czynni	3	50	7,14286	7,14286	0
0	0	0	jeden czyn	1	0	0	0	0
1	0	1	trzy i więce	2	15	2,14286	2,14286	0
0	1	1	trzy i więce	3	180	25,7143	17,1429	8,57143
0	0	1	jeden czyn	2	60	8,57143	0	8,57143
0	0	1	jeden czyn	1	120	17,1429	0	17,1429
0	1	0	dwa czynni	3	840	120	0	120
0	0	1	dwa czynni	2	0	0	0	0
0	0	0	metabolicz	3	60	8,57143	0	8,57143
0	0	1	dwa czynni	1	0	0	0	0
1	1	1	trzy i więce	2	0	0	0	0
1	0	1	trzy i więce	3	180	25,7143	25,7143	0
0	0	1	dwa czynni	2	360	51,4286	51,4286	0
0	0	0	jeden czyn	2	90	12,8571	4,28571	8,57143
0	0	0	jeden czyn	1	480	68,5714	51,4286	17,1429
0	0	1	jeden czyn	2	180	25,7143	0	25,7143
0	0	1	dwa czynni	2	0	0	0	0
0	0	0	jeden czyn	2	540	77,1429	60	17,1429
0	0	0	jeden czyn	2	420	60	25,7143	34,2857
0	0	1	dwa czynni	1	840	120	30	90
0	0	1	jeden czyn	1	0	0	0	0
0	0	1	dwa czynni	3	180	25,7143	0	25,7143
1	0	1	trzy i więce	2	255	36,4286	15	21,4286
1	0	0	dwa czynni	2	60	8,57143	0	8,57143
0	0	1	jeden czyn	2	0	0	0	0
0	0	1	dwa czynni	2	120	17,1429	0	17,1429
0	0	1	dwa czynni	2	300	42,8571	0	42,8571
0	0	1	trzy i więce	2	150	21,4286	4,28571	17,1429
0	0	1	dwa czynni	3	240	34,2857	25,7143	8,57143
0	1	1	trzy i więce	1	10	1,42857	1,42857	0
0	0	0	metabolicz	2	20	2,85714	0	2,85714
0	0	1	dwa czynni	2	0	0	0	0
0	0	0	metabolicz	3	0	0	0	0
1	0	0	dwa czynni	3	0	0	0	0
0	0	0	metabolicz	2	0	0	0	0
1	0	1	trzy i więce	1	480	68,5714	34,2857	34,2857
0	0	0	jeden czyn	2	0	0	0	0
1	1	0	trzy i więce	3	300	42,8571	0	42,8571
0	0	1	dwa czynni	3	0	0	0	0
0	1	1	trzy i więce	2	80	11,4286	11,4286	0
0	0	1	dwa czynni	2	0	0	0	0
0	0	0	jeden czyn	3	0	0	0	0
0	0	1	trzy i więce	2	900	128,571	102,857	25,7143
1	0	1	dwa czynni	2	85	12,1429	5,71429	6,42857

1	0	0	trzy i więce	2	120	17,1429	0	17,1429
1	1	0	trzy i więce	1	210	30	0	30
0	0	1	dwa czynni	3	70	10	0	10
0	0	1	dwa czynni	1	160	22,8571	0	22,8571
0	0	1	dwa czynni	2	300	42,8571	25,7143	17,1429
0	0	1	dwa czynni	2	420	60	0	60
0	0	0	metabolicz	3	60	8,57143	0	8,57143
0	0	1	trzy i więce	3	70	10	10	0
0	0	0	metabolicz	2	600	85,7143	0	85,7143
0	0	0	jeden czyn	1	840	120	0	120
1	1	1	trzy i więce	2	0	0	0	0
0	0	1	dwa czynni	2	0	0	0	0
0	0	1	jeden czyn	1	0	0	0	0
0	0	0	metabolicz	3	210	30	0	30
1	1	1	trzy i więce	1	60	8,57143	0	8,57143
1	1	1	trzy i więce	3	180	25,7143	0	25,7143
0	0	0	metabolicz	2	0	0	0	0
0	0	1	dwa czynni	3	420	60	0	60
1	1	1	trzy i więce	3	0	0	0	0
0	0	0	jeden czyn	3	160	22,8571	17,1429	5,71429
0	0	1	dwa czynni	2	420	60	0	60
0	0	1	jeden czyn	1	0	0	0	0
1	0	1	trzy i więce	3	0	0	0	0
1	0	1	trzy i więce	3	360	51,4286	0	51,4286
0	0	0	jeden czyn	3	0	0	0	0
0	0	1	jeden czyn	2	360	51,4286	0	51,4286
0	0	1	dwa czynni	1	50	7,14286	7,14286	0
0	0	1	jeden czyn	2	0	0	0	0
1	1	1	trzy i więce	2	150	21,4286	0	21,4286
1	0	0	dwa czynni	2	450	64,2857	0	64,2857
0	1	1	trzy i więce	2	15	2,14286	2,14286	0
0	0	1	trzy i więce	3	0	0	0	0
1	0	1	trzy i więce	2	30	4,28571	0	4,28571
0	0	0	jeden czyn	3	210	30	30	0
1	1	1	trzy i więce	1	0	0	0	0
1	0	1	trzy i więce	1	60	8,57143	0	8,57143
0	0	1	jeden czyn	2	0	0	0	0
0	0	1	dwa czynni	2	300	42,8571	0	42,8571
0	0	0	jeden czyn	2	240	34,2857	0	34,2857
0	1	1	trzy i więce	3	0	0	0	0
1	1	1	trzy i więce	1	240	34,2857	0	34,2857
0	0	1	dwa czynni	3	120	17,1429	0	17,1429
1	0	1	trzy i więce	3	0	0	0	0
0	1	0	dwa czynni	3	120	17,1429	17,1429	0
0	0	1	jeden czyn	2	360	51,4286	0	51,4286
0	1	0	jeden czyn	3	540	77,1429	25,7143	51,4286
0	0	0	jeden czyn	1	360	51,4286	51,4286	0
1	0	1	trzy i więce	2	0	0	0	0

0	0	1	dwa czynni	2	0	0	0	0
1	0	1	trzy i więc	2	360	51,4286	0	51,4286
0	0	1	dwa czynni	2	240	34,2857	0	34,2857
1	1	1	trzy i więc	2	0	0	0	0
0	0	0	jeden czyn	2	0	0	0	0
0	0	1	dwa czynni	1	0	0	0	0
0	1	1	trzy i więc	2	0	0	0	0
0	1	0	trzy i więc	3	120	17,1429	17,1429	0
1	0	0	trzy i więc	2	180	25,7143	0	25,7143
1	0	1	trzy i więc	2	420	60	0	60
0	0	1	jeden czyn	2	0	0	0	0
0	0	0	metabolicz	1	360	51,4286	0	51,4286
0	0	1	dwa czynni	2	0	0	0	0
1	1	1	trzy i więc	3	105	15	0	15
0	0	1	jeden czyn	2	210	30	27,1429	2,85714
1	0	1	trzy i więc	3	1080	154,286	154,286	0
0	0	1	jeden czyn	3	150	21,4286	0	21,4286
0	0	1	dwa czynni	2	0	0	0	0
0	0	1	jeden czyn	2	0	0	0	0
0	1	1	trzy i więc	1	0	0	0	0
0	0	0	jeden czyn	2	180	25,7143	0	25,7143
0	0	1	dwa czynni	2	90	12,8571	0	12,8571
0	0	1	jeden czyn	1	0	0	0	0
0	0	1	dwa czynni	2	120	17,1429	0	17,1429
0	1	1	trzy i więc	3	0	0	0	0
0	0	1	dwa czynni	3	30	4,28571	0	4,28571
1	1	0	trzy i więc	2	420	60	0	60
0	0	1	dwa czynni	1	0	0	0	0
1	0	1	dwa czynni	2	30	4,28571	0	4,28571
0	0	0	metabolicz	2	0	0	0	0
1	0	1	trzy i więc	2	0	0	0	0
0	1	1	trzy i więc	3	180	25,7143	0	25,7143
0	0	0	metabolicz	1	0	0	0	0
0	1	1	trzy i więc	2	0	0	0	0
0	0	1	dwa czynni	3	360	51,4286	0	51,4286
0	0	1	jeden czyn	1	60	8,57143	0	8,57143
0	0	1	dwa czynni	1	240	34,2857	0	34,2857
0	0	0	metabolicz	2	420	60	0	60
0	0	0	metabolicz	2	150	21,4286	0	21,4286
0	0	1	dwa czynni	1	0	0	0	0
1	1	0	trzy i więc	3	0	0	0	0
1	1	1	trzy i więc	2	0	0	0	0
0	1	1	trzy i więc	2	20	2,85714	2,85714	0
0	0	0	metabolicz	3	0	0	0	0
0	0	1	jeden czyn	2	0	0	0	0
1	0	1	trzy i więc	3	170	24,2857	7,14286	17,1429
0	1	1	trzy i więc	3	60	8,57143	0	8,57143
0	0	1	dwa czynni	2	2640	377,143	197,143	180

0	0	1	dwa czynni	3	600	85,7143	0	85,7143
0	0	1	dwa czynni	3	0	0	0	0
0	0	1	dwa czynni	2	240	34,2857	0	34,2857
0	0	1	jeden czyn	2	0	0	0	0
1	1	1	trzy i więc	2	0	0	0	0
1	1	1	trzy i więc	3	60	8,57143	0	8,57143
0	0	0	metabolicz	3	120	17,1429	17,1429	0
0	0	0	dwa czynni	2	420	60	42,8571	17,1429
0	1	1	trzy i więc	2	90	12,8571	0	12,8571
0	1	1	trzy i więc	2	0	0	0	0
0	0	1	jeden czyn	2	300	42,8571	25,7143	17,1429
0	0	0	jeden czyn	3	0	0	0	0
1	0	1	dwa czynni	1	720	102,857	102,857	0
0	0	0	dwa czynni	2	20	2,85714	0	2,85714
0	0	1	dwa czynni	3	120	17,1429	0	17,1429
1	1	1	trzy i więc	2	420	60	0	60
0	0	1	dwa czynni	3	420	60	0	60
1	0	1	trzy i więc	2	0	0	0	0
0	0	1	dwa czynni	1	0	0	0	0
1	0	0	trzy i więc	3	10	1,42857	1,42857	0
1	0	1	trzy i więc	3	240	34,2857	34,2857	0
1	0	0	dwa czynni	3	60	8,57143	0	8,57143
0	0	0	metabolicz	2	210	30	0	30
0	1	1	trzy i więc	2	0	0	0	0
0	1	1	trzy i więc	1	0	0	0	0
0	0	0	metabolicz	1	90	12,8571	0	12,8571
1	1	1	trzy i więc	1	50	7,14286	7,14286	0
1	0	1	trzy i więc	1	40	5,71429	5,71429	0
0	1	1	trzy i więc	2	0	0	0	0
0	0	1	dwa czynni	3	20	2,85714	2,85714	0
0	1	1	trzy i więc	3	0	0	0	0
0	0	1	dwa czynni	2	600	85,7143	0	85,7143
0	0	0	metabolicz	3	0	0	0	0
0	0	0	metabolicz	3	360	51,4286	0	51,4286
0	1	1	trzy i więc	3	160	22,8571	17,1429	5,71429
0	0	1	jeden czyn	2	180	25,7143	0	25,7143
1	1	1	trzy i więc	1	90	12,8571	0	12,8571
0	0	1	dwa czynni	2	240	34,2857	17,1429	17,1429
0	0	1	trzy i więc	3	75	10,7143	2,14286	8,57143
1	1	0	trzy i więc	1	80	11,4286	0	11,4286
0	0	1	dwa czynni	3	0	0	0	0
0	0	1	dwa czynni	3	840	120	0	120
1	0	0	dwa czynni	3	0	0	0	0
0	1	1	trzy i więc	3	0	0	0	0
0	0	1	jeden czyn	3	600	85,7143	68,5714	17,1429
0	0	0	jeden czyn	2	15	2,14286	0	2,14286
0	1	1	trzy i więc	2	0	0	0	0
0	1	1	trzy i więc	1	180	25,7143	0	25,7143

0	1	1	trzy i więc	3	135	19,2857	0	19,2857
0	0	1	dwa czynni	2	120	17,1429	0	17,1429
0	0	0	jeden czyn	2	0	0	0	0
0	0	1	trzy i więc	2	0	0	0	0
0	1	1	trzy i więc	2	280	40	10	30
0	0	0	metabolicz	2	120	17,1429	0	17,1429
1	0	0	trzy i więc	2	120	17,1429	0	17,1429
0	0	0	jeden czyn	2	280	40	5,71429	34,2857
1	0	1	dwa czynni	2	120	17,1429	0	17,1429
1	0	1	trzy i więc	3	70	10	10	0
0	1	1	trzy i więc	1	300	42,8571	0	42,8571
0	0	1	dwa czynni	3	420	60	0	60
0	1	1	trzy i więc	2	0	0	0	0
0	0	1	dwa czynni	2	0	0	0	0
1	0	1	trzy i więc	1	60	8,57143	0	8,57143
0	0	0	jeden czyn	3	0	0	0	0
0	1	1	trzy i więc	2	90	12,8571	0	12,8571
0	0	1	trzy i więc	1	0	0	0	0
0	1	1	trzy i więc	2	225	32,1429	6,42857	25,7143
0	1	1	trzy i więc	2	0	0	0	0
1	1	1	trzy i więc	2	180	25,7143	0	25,7143
0	1	0	dwa czynni	2	0	0	0	0
0	0	0	metabolicz	2	1440	205,714	85,7143	120
0	0	0	jeden czyn	2	240	34,2857	17,1429	17,1429
0	1	0	dwa czynni	2	420	60	0	60
0	0	0	metabolicz	2	120	17,1429	0	17,1429
1	0	0	dwa czynni	3	0	0	0	0
0	0	1	dwa czynni	3	1440	205,714	25,7143	180
0	1	1	trzy i więc	2	120	17,1429	0	17,1429
0	0	1	dwa czynni	2	0	0	0	0
0	0	0	metabolicz	1	0	0	0	0
0	0	0	metabolicz	1	350	50	0	50
0	0	1	trzy i więc	1	120	17,1429	0	17,1429
0	0	1	dwa czynni	1	225	32,1429	0	32,1429
0	1	0	dwa czynni	2	180	25,7143	0	25,7143
1	0	0	trzy i więc	3	0	0	0	0
0	0	0	dwa czynni	3	420	60	0	60
0	0	0	jeden czyn	1	150	21,4286	0	21,4286
0	0	0	jeden czyn	3	0	0	0	0
1	1	1	trzy i więc	1	30	4,28571	2,14286	2,14286
0	0	0	jeden czyn	1	60	8,57143	0	8,57143
0	0	1	dwa czynni	3	0	0	0	0
1	1	1	trzy i więc	2	40	5,71429	0	5,71429
0	0	0	metabolicz	2	70	10	10	0
0	0	1	jeden czyn	2	180	25,7143	8,57143	17,1429
1	0	1	trzy i więc	2	0	0	0	0
0	0	1	trzy i więc	1	0	0	0	0
0	0	1	dwa czynni	3	0	0	0	0

0	0	1	dwa czynni	1	90	12,8571	0	12,8571
1	1	1	trzy i więce	3	420	60	25,7143	34,2857
0	0	0	jeden czyn	2	0	0	0	0
0	1	1	trzy i więce	2	315	45	19,2857	25,7143
1	0	1	trzy i więce	2	255	36,4286	25	11,4286
1	0	1	trzy i więce	3	420	60	0	60
0	0	0	metabolicz	1	300	42,8571	8,57143	34,2857
0	0	1	jeden czyn	3	105	15	0	15
0	0	1	dwa czynni	2	240	34,2857	0	34,2857
0	0	0	jeden czyn	1	210	30	0	30
1	1	1	trzy i więce	2	180	25,7143	0	25,7143
0	1	1	trzy i więce	2	0	0	0	0
0	0	1	jeden czyn	1	0	0	0	0
0	0	1	dwa czynni	3	0	0	0	0
0	1	1	dwa czynni	2	60	8,57143	0	8,57143
1	1	1	trzy i więce	2	420	60	34,2857	25,7143
0	0	1	jeden czyn	1	0	0	0	0
1	0	0	dwa czynni	3	120	17,1429	0	17,1429
0	0	1	dwa czynni	3	180	25,7143	0	25,7143
0	0	0	dwa czynni	2	210	30	0	30
0	1	0	jeden czyn	2	670	95,7143	44,2857	51,4286
0	1	1	trzy i więce	2	60	8,57143	0	8,57143
1	1	1	trzy i więce	2	0	0	0	0
0	0	1	dwa czynni	2	280	40	40	0
0	0	0	jeden czyn	2	0	0	0	0
0	0	1	dwa czynni	3	0	0	0	0
1	0	0	jeden czyn	2	0	0	0	0
0	1	1	dwa czynni	3	0	0	0	0
1	0	1	trzy i więce	2	150	21,4286	0	21,4286
0	1	1	trzy i więce	2	180	25,7143	0	25,7143
0	0	1	dwa czynni	2	60	8,57143	0	8,57143
0	1	0	dwa czynni	1	225	32,1429	32,1429	0
0	0	1	jeden czyn	3	0	0	0	0
0	1	1	trzy i więce	3	0	0	0	0
0	0	1	dwa czynni	1	120	17,1429	0	17,1429
0	1	1	trzy i więce	3	0	0	0	0
1	0	1	trzy i więce	1	0	0	0	0
1	0	1	trzy i więce	1	105	15	15	0
0	0	1	dwa czynni	2	0	0	0	0
0	0	0	jeden czyn	1	0	0	0	0
0	0	1	dwa czynni	2	0	0	0	0
0	0	1	trzy i więce	2	60	8,57143	0	8,57143
0	0	1	dwa czynni	2	40	5,71429	0	5,71429
0	0	1	trzy i więce	2	480	68,5714	51,4286	17,1429
0	0	1	dwa czynni	2	240	34,2857	0	34,2857
0	0	1	dwa czynni	2	90	12,8571	0	12,8571
1	1	1	trzy i więce	3	35	5	2,85714	2,14286
1	0	0	trzy i więce	2	0	0	0	0

1	0	1	trzy i więce	3	540	77,1429	25,7143	51,4286
1	0	1	trzy i więce	2	150	21,4286	0	21,4286
1	1	1	trzy i więce	3	190	27,1429	14,2857	12,8571
0	1	1	trzy i więce	1	30	4,28571	0	4,28571
0	0	1	dwa czynni	2	0	0	0	0
0	1	0	dwa czynni	2	360	51,4286	0	51,4286
1	0	0	dwa czynni	1	1260	180	0	180
0	1	1	trzy i więce	1	300	42,8571	0	42,8571
1	0	1	trzy i więce	2	0	0	0	0
0	1	1	trzy i więce	1	300	42,8571	0	42,8571
0	1	1	trzy i więce	2	0	0	0	0
0	1	1	trzy i więce	3	240	34,2857	17,1429	17,1429
0	0	0	metabolicz	3	60	8,57143	0	8,57143
0	1	1	trzy i więce	2	40	5,71429	0	5,71429
1	0	1	trzy i więce	2	290	41,4286	21,4286	20
0	0	1	dwa czynni	1	40	5,71429	0	5,71429
0	1	0	dwa czynni	2	30	4,28571	4,28571	0
1	1	0	trzy i więce	1	0	0	0	0
0	0	1	dwa czynni	2	0	0	0	0
0	0	0	jeden czyn	2	180	25,7143	0	25,7143
0	0	1	trzy i więce	1	120	17,1429	0	17,1429
1	0	0	trzy i więce	1	140	20	20	0
1	0	1	trzy i więce	2	405	57,8571	6,42857	51,4286
0	0	1	jeden czyn	3	0	0	0	0
1	0	1	trzy i więce	1	0	0	0	0
0	1	0	dwa czynni	2	60	8,57143	8,57143	0
0	0	1	dwa czynni	2	0	0	0	0
1	0	0	jeden czyn	1	600	85,7143	25,7143	60
0	1	1	trzy i więce	1	0	0	0	0
1	0	1	trzy i więce	1	0	0	0	0
0	0	1	jeden czyn	1	420	60	0	60
1	0	1	trzy i więce	2	120	17,1429	0	17,1429
0	0	1	dwa czynni	2	0	0	0	0
0	0	0	metabolicz	2	0	0	0	0
0	0	0	jeden czyn	1	30	4,28571	4,28571	0
1	0	0	jeden czyn	3	120	17,1429	0	17,1429
0	0	1	dwa czynni	1	0	0	0	0
0	0	0	metabolicz	2	15	2,14286	0	2,14286
0	0	0	jeden czyn	3	160	22,8571	22,8571	0
0	0	0	jeden czyn	3	0	0	0	0
1	0	1	trzy i więce	2	1020	145,714	25,7143	120
0	0	0	metabolicz	2	0	0	0	0
0	0	0	dwa czynni	2	20	2,85714	0	2,85714
0	0	1	jeden czyn	3	1050	150	30	120
0	0	1	dwa czynni	1	0	0	0	0
0	1	1	trzy i więce	2	30	4,28571	4,28571	0
1	0	1	trzy i więce	3	180	25,7143	17,1429	8,57143
0	0	1	dwa czynni	2	60	8,57143	0	8,57143

1	1	1	trzy i więce	2	0	0	0	0
0	0	1	jeden czyn	1	0	0	0	0
0	1	1	trzy i więce	3	140	20	11,4286	8,57143
0	0	1	jeden czyn	2	30	4,28571	4,28571	0
1	0	1	trzy i więce	3	0	0	0	0
0	0	0	dwa czynni	3	0	0	0	0
0	1	1	trzy i więce	1	140	20	0	20
0	0	1	dwa czynni	2	280	40	0	40
1	0	1	trzy i więce	3	0	0	0	0
0	0	1	jeden czyn	3	0	0	0	0
1	0	1	trzy i więce	3	0	0	0	0
0	0	0	metabolicz	2	90	12,8571	0	12,8571
0	0	0	jeden czyn	1	330	47,1429	21,4286	25,7143
0	0	1	dwa czynni	1	420	60	0	60
1	1	1	trzy i więce	3	140	20	0	20
0	0	1	dwa czynni	3	120	17,1429	0	17,1429
1	0	0	jeden czyn	3	120	17,1429	0	17,1429
0	1	0	jeden czyn	1	60	8,57143	8,57143	0
0	1	1	trzy i więce	1	0	0	0	0
0	0	0	jeden czyn	2	60	8,57143	0	8,57143
0	0	0	jeden czyn	3	360	51,4286	0	51,4286
0	0	1	jeden czyn	2	360	51,4286	0	51,4286
0	0	0	metabolicz	1	60	8,57143	0	8,57143
0	1	0	dwa czynni	3	0	0	0	0
0	0	1	dwa czynni	2	0	0	0	0
0	1	1	trzy i więce	2	0	0	0	0
0	1	1	trzy i więce	3	60	8,57143	8,57143	0
1	0	1	trzy i więce	2	0	0	0	0
0	1	1	trzy i więce	1	0	0	0	0
0	1	1	trzy i więce	2	0	0	0	0
1	0	1	trzy i więce	3	210	30	0	30
0	0	0	jeden czyn	3	180	25,7143	12,8571	12,8571
0	0	1	dwa czynni	2	840	120	60	60
1	0	1	trzy i więce	3	60	8,57143	0	8,57143
0	0	0	jeden czyn	2	135	19,2857	10,7143	8,57143
0	0	1	jeden czyn	3	465	66,4286	15	51,4286
0	0	1	jeden czyn	3	180	25,7143	0	25,7143
0	0	1	dwa czynni	2	150	21,4286	0	21,4286
1	0	1	trzy i więce	3	120	17,1429	8,57143	8,57143
1	0	1	dwa czynni	2	90	12,8571	0	12,8571
0	1	1	trzy i więce	2	150	21,4286	0	21,4286
1	0	0	trzy i więce	1	90	12,8571	12,8571	0
1	1	1	trzy i więce	2	480	68,5714	8,57143	60
0	1	1	trzy i więce	2	420	60	0	60
1	1	1	trzy i więce	2	0	0	0	0
0	1	0	dwa czynni	2	60	8,57143	0	8,57143
0	1	1	trzy i więce	3	0	0	0	0
0	0	1	trzy i więce	2	0	0	0	0

1	1	1	trzy i więce	2	0	0	0	0
0	0	1	trzy i więce	3	165	23,5714	6,42857	17,1429
0	0	1	dwa czynni	3	420	60	0	60
0	0	1	jeden czyn	1	220	31,4286	14,2857	17,1429
0	0	1	dwa czynni	3	0	0	0	0
0	0	1	jeden czyn	1	550	78,5714	10	68,5714
0	0	1	dwa czynni	2	90	12,8571	12,8571	0
0	1	1	trzy i więce	3	0	0	0	0
1	1	1	trzy i więce	1	270	38,5714	0	38,5714
0	0	0	metabolicz	1	240	34,2857	34,2857	0
1	1	1	trzy i więce	2	300	42,8571	0	42,8571
1	1	1	trzy i więce	1	0	0	0	0
0	0	0	metabolicz	3	0	0	0	0
0	0	1	dwa czynni	3	45	6,42857	6,42857	0
0	0	1	dwa czynni	3	60	8,57143	0	8,57143
0	0	1	dwa czynni	1	330	47,1429	30	17,1429
0	0	0	jeden czyn	1	0	0	0	0
1	1	1	trzy i więce	2	0	0	0	0
0	0	1	dwa czynni	3	120	17,1429	0	17,1429
0	0	1	trzy i więce	2	0	0	0	0
0	0	1	jeden czyn	1	120	17,1429	0	17,1429
0	0	1	dwa czynni	2	80	11,4286	11,4286	0
0	1	1	dwa czynni	2	360	51,4286	0	51,4286
0	0	0	jeden czyn	3	120	17,1429	0	17,1429
0	0	0	metabolicz	3	540	77,1429	17,1429	60
0	1	0	jeden czyn	1	150	21,4286	12,8571	8,57143
0	0	1	trzy i więce	1	60	8,57143	0	8,57143
0	0	1	dwa czynni	2	0	0	0	0
0	0	0	metabolicz	1	180	25,7143	8,57143	17,1429
0	0	1	dwa czynni	1	165	23,5714	15	8,57143
1	0	1	trzy i więce	2	60	8,57143	0	8,57143
0	0	0	metabolicz	3	120	17,1429	17,1429	0
0	0	1	dwa czynni	2	420	60	0	60
1	1	1	trzy i więce	2	195	27,8571	10,7143	17,1429
0	0	1	dwa czynni	3	0	0	0	0
0	1	0	trzy i więce	1	0	0	0	0
1	0	1	trzy i więce	2	0	0	0	0
0	0	0	jeden czyn	2	0	0	0	0
0	0	0	metabolicz	2	60	8,57143	0	8,57143
0	0	1	dwa czynni	2	90	12,8571	0	12,8571
0	0	1	dwa czynni	2	20	2,85714	0	2,85714
0	0	1	jeden czyn	2	375	53,5714	19,2857	34,2857
0	0	0	dwa czynni	3	180	25,7143	0	25,7143
0	0	0	metabolicz	3	0	0	0	0
0	1	1	dwa czynni	3	75	10,7143	10,7143	0
1	0	0	dwa czynni	3	30	4,28571	0	4,28571
0	0	1	dwa czynni	3	60	8,57143	0	8,57143
0	0	1	trzy i więce	1	0	0	0	0

0	0	1	dwa czynni	1	240	34,2857	34,2857	0
0	0	0	metabolicz	1	0	0	0	0
0	1	0	jeden czyn	3	250	35,7143	0	35,7143
0	0	1	jeden czyn	2	0	0	0	0
0	0	0	jeden czyn	2	0	0	0	0
0	0	0	metabolicz	1	90	12,8571	12,8571	0
0	0	1	dwa czynni	2	0	0	0	0
0	0	0	jeden czyn	2	0	0	0	0
1	1	1	trzy i więce	1	660	94,2857	17,1429	77,1429
0	0	1	trzy i więce	1	60	8,57143	0	8,57143
1	1	1	trzy i więce	1	0	0	0	0
0	0	1	dwa czynni	2	0	0	0	0
0	1	1	trzy i więce	2	0	0	0	0
1	1	1	trzy i więce	2	0	0	0	0
0	0	0	jeden czyn	3	0	0	0	0
0	0	1	dwa czynni	3	480	68,5714	0	68,5714
0	0	1	jeden czyn	1	0	0	0	0
0	0	1	dwa czynni	3	370	52,8571	40	12,8571
1	0	1	trzy i więce	2	60	8,57143	0	8,57143
1	1	1	trzy i więce	3	30	4,28571	0	4,28571
0	0	1	dwa czynni	2	1560	222,857	102,857	120
0	0	1	dwa czynni	2	30	4,28571	0	4,28571
1	0	1	trzy i więce	2	0	0	0	0
0	0	1	dwa czynni	3	0	0	0	0
0	0	0	jeden czyn	2	0	0	0	0
0	0	0	metabolicz	2	330	47,1429	17,1429	30
0	0	0	jeden czyn	3	420	60	0	60
1	1	1	trzy i więce	3	0	0	0	0
0	0	0	jeden czyn	2	440	62,8571	2,85714	60
0	1	1	trzy i więce	1	0	0	0	0
1	0	1	trzy i więce	2	840	120	0	120
0	0	1	jeden czyn	2	210	30	0	30
0	0	0	metabolicz	2	900	128,571	0	128,571
1	0	0	dwa czynni	2	0	0	0	0
0	0	0	jeden czyn	2	0	0	0	0
0	0	1	dwa czynni	2	240	34,2857	17,1429	17,1429
0	1	1	trzy i więce	3	150	21,4286	0	21,4286
0	1	1	trzy i więce	2	0	0	0	0
0	0	1	dwa czynni	2	30	4,28571	4,28571	0
1	0	1	trzy i więce	3	90	12,8571	0	12,8571
0	0	1	dwa czynni	2	30	4,28571	0	4,28571
0	1	1	trzy i więce	2	0	0	0	0
1	0	1	trzy i więce	1	60	8,57143	0	8,57143
0	0	1	jeden czyn	3	105	15	0	15
0	0	0	metabolicz	3	90	12,8571	12,8571	0
1	1	1	trzy i więce	2	0	0	0	0
0	0	1	dwa czynni	3	0	0	0	0
0	0	1	dwa czynni	3	0	0	0	0

0	0	0	jeden czyn	3	0	0	0	0
0	0	1	dwa czynni	2	690	98,5714	85,7143	12,8571
0	0	0	metabolicz	2	360	51,4286	0	51,4286
1	1	0	dwa czynni	1	60	8,57143	0	8,57143
0	0	1	jeden czyn	2	240	34,2857	0	34,2857
0	0	1	jeden czyn	3	720	102,857	42,8571	60
1	1	1	trzy i więc	3	0	0	0	0
0	1	1	trzy i więc	1	0	0	0	0
0	0	1	dwa czynni	3	210	30	0	30
0	0	0	metabolicz	1	0	0	0	0
0	0	1	dwa czynni	2	0	0	0	0
0	0	1	trzy i więc	2	90	12,8571	12,8571	0
0	0	1	jeden czyn	3	60	8,57143	0	8,57143
1	1	1	trzy i więc	3	40	5,71429	5,71429	0
0	0	1	dwa czynni	3	60	8,57143	0	8,57143
0	0	0	jeden czyn	1	0	0	0	0
0	0	1	dwa czynni	1	390	55,7143	34,2857	21,4286
0	0	1	dwa czynni	2	0	0	0	0
1	0	0	trzy i więc	2	0	0	0	0
1	0	1	trzy i więc	2	80	11,4286	2,85714	8,57143
0	0	0	metabolicz	3	0	0	0	0
0	0	1	dwa czynni	2	0	0	0	0
0	0	1	dwa czynni	3	0	0	0	0
1	0	1	trzy i więc	2	120	17,1429	0	17,1429
0	0	1	dwa czynni	3	210	30	30	0
1	1	1	trzy i więc	2	0	0	0	0
0	0	0	metabolicz	2	360	51,4286	51,4286	0
1	1	0	trzy i więc	2	0	0	0	0
0	1	0	dwa czynni	3	120	17,1429	0	17,1429
0	0	1	dwa czynni	2	0	0	0	0
0	0	1	dwa czynni	2	0	0	0	0
1	1	1	trzy i więc	2	120	17,1429	17,1429	0
0	0	0	jeden czyn	3	0	0	0	0
1	0	0	dwa czynni	3	120	17,1429	0	17,1429
1	0	1	trzy i więc	1	0	0	0	0
0	0	0	jeden czyn	2	60	8,57143	0	8,57143
0	0	1	dwa czynni	3	165	23,5714	15	8,57143
0	0	1	dwa czynni	2	0	0	0	0
0	0	1	dwa czynni	2	840	120	0	120
1	0	0	jeden czyn	1	0	0	0	0
0	0	1	trzy i więc	2	240	34,2857	0	34,2857
0	0	1	trzy i więc	1	30	4,28571	0	4,28571
0	0	1	trzy i więc	2	0	0	0	0
0	0	1	jeden czyn	3	45	6,42857	0	6,42857
0	0	1	dwa czynni	2	120	17,1429	0	17,1429
0	0	0	metabolicz	1	10	1,42857	1,42857	0
1	0	1	trzy i więc	1	180	25,7143	0	25,7143
0	0	1	dwa czynni	3	60	8,57143	0	8,57143

1	1	1	trzy i więce	3	0	0	0	0
0	0	1	dwa czynni	3	0	0	0	0
1	1	1	trzy i więce	2	120	17,1429	0	17,1429
0	0	1	dwa czynni	2	60	8,57143	0	8,57143
0	0	0	jeden czyn	2	120	17,1429	0	17,1429
0	1	1	trzy i więce	3	490	70	10	60
1	1	0	trzy i więce	1	0	0	0	0
0	1	0	dwa czynni	1	135	19,2857	10,7143	8,57143
0	1	1	trzy i więce	2	0	0	0	0
1	1	1	trzy i więce	2	120	17,1429	0	17,1429
0	1	1	trzy i więce	2	0	0	0	0
0	1	0	dwa czynni	1	0	0	0	0
1	0	1	trzy i więce	2	120	17,1429	0	17,1429
1	1	1	trzy i więce	3	40	5,71429	0	5,71429
0	0	1	dwa czynni	2	240	34,2857	0	34,2857
0	0	1	dwa czynni	2	450	64,2857	30	34,2857
0	0	1	trzy i więce	3	0	0	0	0
0	0	1	jeden czyn	2	30	4,28571	4,28571	0
1	1	0	trzy i więce	3	280	40	0	40
0	0	1	jeden czyn	3	360	51,4286	0	51,4286
1	1	0	trzy i więce	2	0	0	0	0
0	0	1	dwa czynni	1	0	0	0	0
0	0	1	jeden czyn	2	240	34,2857	17,1429	17,1429
0	0	0	jeden czyn	2	0	0	0	0
0	0	0	jeden czyn	3	1050	150	30	120
0	0	1	dwa czynni	2	0	0	0	0
0	0	0	metabolicz	2	240	34,2857	0	34,2857
0	0	0	dwa czynni	3	90	12,8571	4,28571	8,57143
0	0	1	jeden czyn	3	180	25,7143	8,57143	17,1429
0	0	1	jeden czyn	2	0	0	0	0
0	1	1	trzy i więce	1	0	0	0	0
0	0	1	dwa czynni	2	350	50	0	50
0	0	1	dwa czynni	3	240	34,2857	0	34,2857
0	1	0	dwa czynni	1	0	0	0	0
0	0	0	jeden czyn	2	570	81,4286	21,4286	60
0	0	1	dwa czynni	2	0	0	0	0
1	0	1	trzy i więce	3	420	60	0	60
0	0	1	dwa czynni	1	60	8,57143	0	8,57143
0	0	1	dwa czynni	2	0	0	0	0
0	0	1	jeden czyn	3	300	42,8571	25,7143	17,1429
1	0	1	trzy i więce	1	150	21,4286	12,8571	8,57143
0	0	1	dwa czynni	2	30	4,28571	0	4,28571
0	1	1	trzy i więce	1	600	85,7143	60	25,7143
1	0	1	trzy i więce	3	180	25,7143	8,57143	17,1429
0	1	1	trzy i więce	1	0	0	0	0
0	1	1	trzy i więce	1	0	0	0	0
1	0	0	dwa czynni	3	0	0	0	0
0	0	0	metabolicz	2	0	0	0	0

0	0	1	dwa czynni	2	120	17,1429	0	17,1429
0	0	1	dwa czynni	2	120	17,1429	0	17,1429
0	0	1	dwa czynni	3	120	17,1429	0	17,1429
0	0	1	trzy i więce	3	150	21,4286	0	21,4286
0	0	1	dwa czynni	2	40	5,71429	0	5,71429
0	1	1	trzy i więce	1	0	0	0	0
0	0	1	dwa czynni	2	600	85,7143	8,57143	77,1429
0	1	0	jeden czyn	2	0	0	0	0
0	0	1	trzy i więce	1	0	0	0	0
0	0	1	dwa czynni	1	120	17,1429	0	17,1429
0	0	0	jeden czyn	3	90	12,8571	0	12,8571
0	1	1	trzy i więce	2	0	0	0	0
0	0	1	dwa czynni	2	0	0	0	0
1	0	1	trzy i więce	3	120	17,1429	8,57143	8,57143
0	0	1	trzy i więce	3	0	0	0	0
0	0	0	metabolicz	2	0	0	0	0
0	1	0	dwa czynni	1	60	8,57143	0	8,57143
0	0	0	metabolicz	3	60	8,57143	0	8,57143
0	0	1	dwa czynni	2	0	0	0	0
0	0	0	dwa czynni	3	280	40	10	30
0	0	1	trzy i więce	2	240	34,2857	17,1429	17,1429
0	0	1	jeden czyn	2	300	42,8571	8,57143	34,2857
0	0	1	dwa czynni	1	0	0	0	0
0	0	1	jeden czyn	1	0	0	0	0
1	1	1	trzy i więce	2	0	0	0	0
0	0	1	dwa czynni	2	140	20	0	20
1	0	1	trzy i więce	2	0	0	0	0
1	0	0	trzy i więce	2	0	0	0	0
0	0	0	jeden czyn	3	0	0	0	0
0	1	0	dwa czynni	2	105	15	15	0
1	1	0	trzy i więce	2	420	60	21,4286	38,5714
0	0	0	metabolicz	3	270	38,5714	8,57143	30
0	0	0	metabolicz	1	300	42,8571	25,7143	17,1429
0	0	1	dwa czynni	3	100	14,2857	0	14,2857
0	0	1	jeden czyn	2	30	4,28571	0	4,28571
0	0	1	dwa czynni	2	210	30	0	30
0	0	1	dwa czynni	1	240	34,2857	34,2857	0
0	1	1	trzy i więce	1	540	77,1429	60	17,1429
0	0	1	dwa czynni	3	600	85,7143	0	85,7143
0	0	1	dwa czynni	2	1260	180	0	180
0	0	1	dwa czynni	3	90	12,8571	0	12,8571
0	0	1	dwa czynni	3	0	0	0	0
1	1	1	trzy i więce	3	0	0	0	0
0	1	1	trzy i więce	2	0	0	0	0
0	0	1	jeden czyn	1	0	0	0	0
0	0	1	dwa czynni	3	0	0	0	0
1	0	1	trzy i więce	2	0	0	0	0
0	0	1	dwa czynni	3	0	0	0	0

0	0	0	jeden czyn	2	330	47,1429	17,1429	30
0	0	1	dwa czynni	2	0	0	0	0
0	0	1	dwa czynni	3	0	0	0	0
0	0	0	jeden czyn	2	50	7,14286	7,14286	0
0	0	0	metabolicz	1	0	0	0	0
0	0	0	jeden czyn	1	220	31,4286	1,42857	30
0	0	1	trzy i więc	2	720	102,857	0	102,857
0	0	1	trzy i więc	3	60	8,57143	8,57143	0
0	0	0	metabolicz	1	0	0	0	0
1	0	0	dwa czynni	2	240	34,2857	8,57143	25,7143
0	1	1	trzy i więc	1	60	8,57143	0	8,57143
1	0	1	trzy i więc	2	840	120	0	120
1	1	0	trzy i więc	1	0	0	0	0
0	0	1	dwa czynni	2	0	0	0	0
1	0	0	dwa czynni	2	60	8,57143	0	8,57143
0	0	1	trzy i więc	2	0	0	0	0
0	1	1	trzy i więc	2	30	4,28571	0	4,28571
0	0	1	dwa czynni	2	0	0	0	0
0	0	0	jeden czyn	2	120	17,1429	0	17,1429
0	1	1	dwa czynni	2	120	17,1429	17,1429	0
1	0	1	trzy i więc	1	150	21,4286	0	21,4286
0	0	1	dwa czynni	2	0	0	0	0
0	0	1	jeden czyn	2	210	30	17,1429	12,8571
0	0	1	dwa czynni	3	0	0	0	0
1	0	1	trzy i więc	3	0	0	0	0
0	0	1	dwa czynni	2	360	51,4286	34,2857	17,1429
0	0	1	dwa czynni	3	60	8,57143	8,57143	0
0	1	1	trzy i więc	3	420	60	8,57143	51,4286
0	0	1	dwa czynni	1	60	8,57143	0	8,57143
0	1	1	trzy i więc	3	0	0	0	0
0	0	1	dwa czynni	2	0	0	0	0
0	0	0	metabolicz	2	60	8,57143	8,57143	0
0	0	0	jeden czyn	1	180	25,7143	0	25,7143
0	0	0	jeden czyn	1	0	0	0	0
1	0	1	trzy i więc	2	210	30	0	30
0	1	0	dwa czynni	2	0	0	0	0
0	0	0	metabolicz	3	120	17,1429	0	17,1429
0	0	0	metabolicz	2	100	14,2857	0	14,2857
0	1	1	trzy i więc	2	50	7,14286	7,14286	0
0	1	1	trzy i więc	2	45	6,42857	6,42857	0
0	0	1	jeden czyn	3	0	0	0	0
1	0	0	trzy i więc	3	100	14,2857	0	14,2857
0	0	1	dwa czynni	1	0	0	0	0
0	0	1	dwa czynni	1	180	25,7143	0	25,7143
0	0	1	jeden czyn	1	0	0	0	0
0	0	0	jeden czyn	2	240	34,2857	8,57143	25,7143
1	1	1	trzy i więc	2	0	0	0	0
0	0	1	jeden czyn	1	0	0	0	0

1	0	0	trzy i więce	2	60	8,57143	0	8,57143
0	0	1	jeden czyn	3	360	51,4286	0	51,4286
0	0	1	dwa czynni	1	90	12,8571	0	12,8571
0	0	1	dwa czynni	2	0	0	0	0
0	0	1	dwa czynni	3	360	51,4286	51,4286	0
1	0	1	trzy i więce	2	0	0	0	0
0	1	1	trzy i więce	3	0	0	0	0
0	0	0	metabolicz	2	0	0	0	0
0	0	0	jeden czyn	2	75	10,7143	2,14286	8,57143
0	0	0	metabolicz	3	600	85,7143	51,4286	34,2857
1	1	1	trzy i więce	2	60	8,57143	0	8,57143
0	1	1	trzy i więce	3	420	60	0	60
0	0	1	jeden czyn	3	140	20	20	0
0	1	0	dwa czynni	3	240	34,2857	0	34,2857
1	1	1	trzy i więce	3	105	15	15	0
0	0	0	metabolicz	2	180	25,7143	25,7143	0
0	0	1	dwa czynni	2	0	0	0	0
0	0	1	trzy i więce	1	840	120	0	120
1	0	1	trzy i więce	1	40	5,71429	0	5,71429
1	0	0	dwa czynni	3	480	68,5714	8,57143	60
0	0	0	jeden czyn	2	420	60	0	60
0	0	0	metabolicz	2	45	6,42857	0	6,42857
0	1	1	trzy i więce	3	0	0	0	0
0	1	1	trzy i więce	3	0	0	0	0
0	0	1	dwa czynni	2	180	25,7143	0	25,7143
1	1	0	trzy i więce	3	0	0	0	0
0	1	0	dwa czynni	3	0	0	0	0
0	0	0	dwa czynni	2	120	17,1429	0	17,1429
0	1	1	trzy i więce	2	60	8,57143	8,57143	0
0	0	0	jeden czyn	2	0	0	0	0
1	1	0	trzy i więce	1	180	25,7143	0	25,7143
1	0	0	trzy i więce	2	180	25,7143	0	25,7143
0	0	1	jeden czyn	3	420	60	0	60
0	0	1	jeden czyn	3	240	34,2857	0	34,2857
0	0	0	jeden czyn	2	0	0	0	0
1	0	1	dwa czynni	2	45	6,42857	6,42857	0
1	0	1	trzy i więce	2	60	8,57143	0	8,57143
0	0	1	dwa czynni	3	270	38,5714	0	38,5714
0	0	1	dwa czynni	2	180	25,7143	0	25,7143
0	0	1	dwa czynni	2	0	0	0	0
0	0	1	dwa czynni	3	0	0	0	0
0	0	1	dwa czynni	2	420	60	25,7143	34,2857
0	1	1	trzy i więce	3	180	25,7143	0	25,7143
0	0	1	dwa czynni	3	45	6,42857	6,42857	0
0	1	1	trzy i więce	2	40	5,71429	0	5,71429
1	0	1	trzy i więce	2	60	8,57143	8,57143	0
0	1	1	trzy i więce	1	0	0	0	0
0	0	1	trzy i więce	2	240	34,2857	0	34,2857

0	0	1	dwa czynni	3	0	0	0	0
0	0	0	metabolicz	2	0	0	0	0
0	1	1	trzy i więce	2	120	17,1429	0	17,1429
0	1	0	dwa czynni	2	360	51,4286	51,4286	0
0	0	0	jeden czyn	2	60	8,57143	0	8,57143
0	0	0	jeden czyn	2	0	0	0	0
0	0	0	jeden czyn	1	0	0	0	0
0	0	1	jeden czyn	3	195	27,8571	17,1429	10,7143
0	0	1	jeden czyn	2	0	0	0	0
0	0	1	dwa czynni	2	80	11,4286	0	11,4286
1	0	0	trzy i więce	3	120	17,1429	0	17,1429
0	1	1	trzy i więce	3	100	14,2857	0	14,2857
0	1	0	dwa czynni	2	180	25,7143	8,57143	17,1429
0	0	1	dwa czynni	1	0	0	0	0
0	1	0	jeden czyn	1	0	0	0	0
0	0	0	jeden czyn	2	0	0	0	0
1	1	1	trzy i więce	2	105	15	15	0
0	0	1	dwa czynni	2	165	23,5714	15	8,57143
0	1	1	trzy i więce	2	0	0	0	0
0	0	1	dwa czynni	2	120	17,1429	0	17,1429
0	0	1	dwa czynni	3	120	17,1429	0	17,1429
0	0	1	jeden czyn	3	0	0	0	0
0	0	1	trzy i więce	3	40	5,71429	0	5,71429
0	0	0	jeden czyn	1	0	0	0	0
1	0	0	dwa czynni	3	120	17,1429	17,1429	0
0	0	1	jeden czyn	2	0	0	0	0
0	0	1	dwa czynni	2	480	68,5714	8,57143	60
0	0	1	jeden czyn	2	120	17,1429	0	17,1429
0	0	1	jeden czyn	3	0	0	0	0
0	1	1	dwa czynni	1	0	0	0	0
1	1	1	trzy i więce	2	0	0	0	0
0	0	0	metabolicz	2	0	0	0	0
0	0	1	trzy i więce	1	240	34,2857	17,1429	17,1429
1	1	0	trzy i więce	2	0	0	0	0
1	1	1	trzy i więce	2	180	25,7143	0	25,7143
1	0	0	trzy i więce	2	120	17,1429	0	17,1429
0	1	1	trzy i więce	1	180	25,7143	0	25,7143
1	0	0	trzy i więce	2	40	5,71429	5,71429	0
0	1	1	trzy i więce	3	0	0	0	0
1	0	0	dwa czynni	3	0	0	0	0
0	0	0	jeden czyn	2	90	12,8571	0	12,8571
0	0	1	jeden czyn	2	420	60	0	60
0	0	1	dwa czynni	2	90	12,8571	4,28571	8,57143
0	0	1	trzy i więce	1	0	0	0	0
1	0	1	trzy i więce	3	0	0	0	0
0	0	0	jeden czyn	2	300	42,8571	0	42,8571
1	1	0	trzy i więce	2	60	8,57143	8,57143	0
1	0	0	trzy i więce	3	420	60	17,1429	42,8571

1	*0*	*0*	trzy i więc	3	*105*	*15*	*0*	*15*
0	*0*	*0*	metabolicz	2	*55*	*7,85714*	*5*	*2,85714*
0	*0*	*1*	trzy i więc	1	*0*	*0*	*0*	*0*
0	*1*	*0*	dwa czynni	3	*210*	*30*	*17,1429*	*12,8571*
0	*0*	*1*	dwa czynni	2	*70*	*10*	*0*	*10*
0	*0*	*1*	jeden czyn	1	*0*	*0*	*0*	*0*
0	*0*	*1*	dwa czynni	2	*0*	*0*	*0*	*0*
0	*0*	*0*	metabolicz	1	*60*	*8,57143*	*0*	*8,57143*
0	*0*	*0*	jeden czyn	2	*240*	*34,2857*	*8,57143*	*25,7143*
0	*1*	*1*	dwa czynni	2	*240*	*34,2857*	*17,1429*	*17,1429*
0	*0*	*1*	dwa czynni	3	*0*	*0*	*0*	*0*
0	*1*	*1*	trzy i więc	2	*0*	*0*	*0*	*0*
0	*0*	*1*	dwa czynni	2	*120*	*17,1429*	*0*	*17,1429*
1	*0*	*1*	dwa czynni	2	*0*	*0*	*0*	*0*
0	*0*	*1*	dwa czynni	2	*150*	*21,4286*	*0*	*21,4286*
1	*0*	*1*	trzy i więc	1	*500*	*71,4286*	*20*	*51,4286*
1	*0*	*1*	trzy i więc	1	*360*	*51,4286*	*0*	*51,4286*
0	*0*	*1*	dwa czynni	3	*0*	*0*	*0*	*0*
0	*0*	*1*	dwa czynni	2	*0*	*0*	*0*	*0*
0	*0*	*1*	dwa czynni	2	*0*	*0*	*0*	*0*
1	*0*	*0*	dwa czynni	1	*120*	*17,1429*	*0*	*17,1429*
0	*0*	*1*	dwa czynni	2	*0*	*0*	*0*	*0*
0	*0*	*1*	dwa czynni	2	*420*	*60*	*0*	*60*
0	*1*	*1*	trzy i więc	3	*0*	*0*	*0*	*0*
0	*1*	*0*	dwa czynni	1	*0*	*0*	*0*	*0*
1	*0*	*1*	trzy i więc	3	*240*	*34,2857*	*34,2857*	*0*
0	*0*	*1*	dwa czynni	2	*180*	*25,7143*	*0*	*25,7143*
0	*0*	*1*	dwa czynni	3	*1260*	*180*	*120*	*60*
0	*0*	*0*	jeden czyn	1	*120*	*17,1429*	*0*	*17,1429*
1	*0*	*0*	dwa czynni	3	*0*	*0*	*0*	*0*
1	*0*	*1*	dwa czynni	3	*0*	*0*	*0*	*0*
0	*1*	*1*	trzy i więc	2	*0*	*0*	*0*	*0*
0	*0*	*1*	jeden czyn	3	*120*	*17,1429*	*0*	*17,1429*
0	*0*	*1*	dwa czynni	1	*0*	*0*	*0*	*0*
0	*0*	*0*	jeden czyn	1	*0*	*0*	*0*	*0*
0	*0*	*1*	dwa czynni	1	*280*	*40*	*0*	*40*
0	*0*	*1*	trzy i więc	2	*0*	*0*	*0*	*0*
0	*0*	*1*	dwa czynni	1	*0*	*0*	*0*	*0*
0	*0*	*0*	metabolicz	2	*15*	*2,14286*	*0*	*2,14286*
0	*0*	*0*	jeden czyn	2	*105*	*15*	*6,42857*	*8,57143*
0	*0*	*1*	dwa czynni	1	*0*	*0*	*0*	*0*
0	*0*	*1*	dwa czynni	2	*80*	*11,4286*	*0*	*11,4286*
1	*0*	*1*	trzy i więc	2	*190*	*27,1429*	*14,2857*	*12,8571*
0	*1*	*1*	trzy i więc	2	*90*	*12,8571*	*0*	*12,8571*
0	*0*	*0*	metabolicz	2	*160*	*22,8571*	*14,2857*	*8,57143*
0	*0*	*1*	dwa czynni	2	*160*	*22,8571*	*10*	*12,8571*
1	*1*	*1*	trzy i więc	2	*0*	*0*	*0*	*0*
0	*1*	*1*	trzy i więc	3	*700*	*100*	*60*	*40*

0	0	1	dwa czynni	1	105	15	0	15
0	0	0	jeden czyn	3	0	0	0	0
1	0	0	trzy i więce	2	105	15	0	15
0	0	1	jeden czyn	2	1020	145,714	120	25,7143
1	0	1	trzy i więce	3	120	17,1429	17,1429	0
0	0	1	jeden czyn	3	900	128,571	68,5714	60
0	0	1	jeden czyn	2	100	14,2857	5,71429	8,57143
0	0	1	jeden czyn	2	30	4,28571	4,28571	0
0	0	1	dwa czynni	3	630	90	0	90
0	0	1	dwa czynni	3	0	0	0	0
1	0	1	trzy i więce	1	0	0	0	0
0	0	0	jeden czyn	2	840	120	0	120
0	0	1	dwa czynni	3	180	25,7143	0	25,7143
1	0	0	dwa czynni	3	0	0	0	0
0	0	0	jeden czyn	2	0	0	0	0
0	0	1	dwa czynni	1	240	34,2857	0	34,2857
0	0	1	dwa czynni	2	0	0	0	0
0	0	1	dwa czynni	2	60	8,57143	0	8,57143
1	0	1	trzy i więce	2	0	0	0	0
0	1	1	trzy i więce	3	0	0	0	0
1	0	1	trzy i więce	2	255	36,4286	15	21,4286
0	0	1	jeden czyn	2	240	34,2857	8,57143	25,7143
0	0	0	jeden czyn	3	0	0	0	0
0	1	1	dwa czynni	1	0	0	0	0
0	0	0	metabolicz	3	555	79,2857	19,2857	60
0	0	1	dwa czynni	3	160	22,8571	8,57143	14,2857
0	1	1	trzy i więce	2	180	25,7143	12,8571	12,8571
0	0	1	jeden czyn	2	240	34,2857	8,57143	25,7143
0	1	0	jeden czyn	3	60	8,57143	0	8,57143
0	0	1	dwa czynni	2	0	0	0	0
0	0	0	metabolicz	2	225	32,1429	15	17,1429
0	0	1	dwa czynni	1	630	90	0	90
0	0	1	dwa czynni	3	0	0	0	0
0	0	1	jeden czyn	2	540	77,1429	0	77,1429
0	0	1	dwa czynni	3	40	5,71429	0	5,71429
0	0	0	jeden czyn	2	0	0	0	0
0	1	1	trzy i więce	2	0	0	0	0
0	0	0	metabolicz	2	0	0	0	0
0	0	1	dwa czynni	2	0	0	0	0
1	1	1	trzy i więce	2	0	0	0	0
1	0	1	trzy i więce	2	120	17,1429	17,1429	0
1	1	1	trzy i więce	3	90	12,8571	0	12,8571
1	1	1	trzy i więce	1	180	25,7143	0	25,7143
0	0	1	jeden czyn	3	120	17,1429	0	17,1429
0	0	1	dwa czynni	3	105	15	15	0
0	0	0	jeden czyn	3	420	60	0	60
0	1	0	jeden czyn	3	360	51,4286	0	51,4286
0	0	1	dwa czynni	3	0	0	0	0

0	0	1	dwa czynni	2	0	0	0	0
1	1	1	trzy i więc	2	420	60	0	60
0	0	0	metabolicz	2	450	64,2857	34,2857	30
0	0	0	jeden czyn	1	600	85,7143	51,4286	34,2857
0	1	1	trzy i więc	2	120	17,1429	0	17,1429
0	0	0	jeden czyn	3	240	34,2857	0	34,2857
0	0	1	dwa czynni	2	460	65,7143	14,2857	51,4286
0	0	1	dwa czynni	3	300	42,8571	12,8571	30
0	1	1	trzy i więc	2	120	17,1429	0	17,1429
0	0	1	trzy i więc	3	300	42,8571	0	42,8571
0	0	0	jeden czyn	3	30	4,28571	4,28571	0
0	0	1	dwa czynni	2	0	0	0	0
0	1	0	dwa czynni	2	0	0	0	0
1	0	0	trzy i więc	3	370	52,8571	12,8571	40
0	1	1	trzy i więc	3	0	0	0	0
0	1	1	trzy i więc	3	120	17,1429	0	17,1429
0	0	1	dwa czynni	1	180	25,7143	0	25,7143
1	0	1	trzy i więc	1	0	0	0	0
0	0	1	dwa czynni	2	30	4,28571	0	4,28571
1	1	1	trzy i więc	3	40	5,71429	0	5,71429
0	0	1	dwa czynni	2	0	0	0	0
0	0	1	dwa czynni	1	120	17,1429	0	17,1429
0	1	0	jeden czyn	1	180	25,7143	25,7143	0
1	0	1	trzy i więc	2	210	30	17,1429	12,8571
0	0	1	dwa czynni	2	60	8,57143	0	8,57143
0	1	0	trzy i więc	2	90	12,8571	0	12,8571
0	0	1	dwa czynni	2	270	38,5714	12,8571	25,7143
1	0	0	jeden czyn	2	120	17,1429	0	17,1429
0	0	1	dwa czynni	3	120	17,1429	17,1429	0
0	0	0	metabolicz	3	390	55,7143	38,5714	17,1429
0	0	1	jeden czyn	1	0	0	0	0
0	0	1	dwa czynni	3	0	0	0	0
0	0	1	jeden czyn	2	260	37,1429	25,7143	11,4286
1	0	1	trzy i więc	2	120	17,1429	0	17,1429
0	0	1	dwa czynni	2	60	8,57143	0	8,57143
0	1	1	trzy i więc	2	0	0	0	0
0	0	1	dwa czynni	2	0	0	0	0
0	0	0	jeden czyn	3	0	0	0	0
1	0	1	trzy i więc	2	180	25,7143	8,57143	17,1429
1	1	1	trzy i więc	1	0	0	0	0
0	0	0	jeden czyn	3	0	0	0	0
0	1	1	trzy i więc	3	570	81,4286	51,4286	30
0	0	0	metabolicz	2	0	0	0	0
0	0	0	metabolicz	2	240	34,2857	0	34,2857
1	1	1	trzy i więc	1	0	0	0	0
0	1	1	trzy i więc	2	210	30	4,28571	25,7143
0	0	0	jeden czyn	1	0	0	0	0
0	0	1	dwa czynni	3	360	51,4286	0	51,4286

0	0	0	metabolicz	3	0	0	0	0
1	0	1	trzy i więc	2	330	47,1429	30	17,1429
1	0	0	jeden czyn	2	0	0	0	0
0	1	1	trzy i więc	2	30	4,28571	0	4,28571
0	0	1	dwa czynni	2	150	21,4286	17,1429	4,28571
0	0	1	dwa czynni	1	300	42,8571	42,8571	0
0	0	1	jeden czyn	3	330	47,1429	17,1429	30
0	1	0	dwa czynni	1	380	54,2857	54,2857	0
0	0	0	jeden czyn	3	120	17,1429	0	17,1429
0	0	0	metabolicz	2	0	0	0	0
0	0	0	jeden czyn	1	0	0	0	0
1	0	1	trzy i więc	3	0	0	0	0
1	0	1	trzy i więc	2	135	19,2857	0	19,2857
0	0	1	jeden czyn	2	315	45	45	0
0	0	1	trzy i więc	2	0	0	0	0
0	0	1	trzy i więc	2	300	42,8571	12,8571	30
1	0	1	trzy i więc	2	180	25,7143	8,57143	17,1429
0	0	0	jeden czyn	3	0	0	0	0
0	1	1	trzy i więc	2	120	17,1429	0	17,1429
0	0	0	jeden czyn	2	120	17,1429	0	17,1429
0	0	0	dwa czynni	3	0	0	0	0
1	1	1	trzy i więc	2	420	60	0	60
1	0	0	trzy i więc	1	270	38,5714	38,5714	0
0	0	1	trzy i więc	3	0	0	0	0
1	0	1	trzy i więc	1	120	17,1429	0	17,1429
0	0	1	dwa czynni	2	0	0	0	0
1	0	1	trzy i więc	2	0	0	0	0
0	0	0	metabolicz	2	60	8,57143	0	8,57143
0	0	0	metabolicz	2	120	17,1429	0	17,1429
0	0	1	dwa czynni	3	50	7,14286	7,14286	0
1	0	1	trzy i więc	2	180	25,7143	0	25,7143
0	0	1	dwa czynni	3	180	25,7143	25,7143	0
1	1	1	trzy i więc	1	0	0	0	0
0	0	0	metabolicz	1	360	51,4286	0	51,4286
1	0	1	dwa czynni	2	0	0	0	0
0	0	1	dwa czynni	2	165	23,5714	6,42857	17,1429
0	1	0	dwa czynni	2	225	32,1429	15	17,1429
0	1	1	trzy i więc	3	60	8,57143	8,57143	0
0	0	0	jeden czyn	3	540	77,1429	51,4286	25,7143
0	0	0	jeden czyn	2	0	0	0	0
1	1	1	trzy i więc	2	360	51,4286	8,57143	42,8571
0	0	1	dwa czynni	2	60	8,57143	0	8,57143
0	0	0	dwa czynni	3	35	5	0	5
1	1	1	trzy i więc	1	0	0	0	0
0	0	0	jeden czyn	2	15	2,14286	2,14286	0
0	0	1	jeden czyn	2	180	25,7143	0	25,7143
0	0	0	metabolicz	3	60	8,57143	8,57143	0
0	0	1	jeden czyn	2	360	51,4286	34,2857	17,1429

1	0	1	trzy i więce	3	0	0	0	0
0	0	0	metabolicz	3	1080	154,286	0	154,286
1	1	1	trzy i więce	2	0	0	0	0
0	1	1	trzy i więce	2	100	14,2857	14,2857	0
0	0	0	metabolicz	1	60	8,57143	0	8,57143
0	0	0	jeden czyn	3	510	72,8571	12,8571	60
0	0	1	dwa czynni	3	0	0	0	0
1	0	1	trzy i więce	2	60	8,57143	0	8,57143
0	1	0	dwa czynni	2	0	0	0	0
0	0	1	jeden czyn	1	0	0	0	0
0	0	1	dwa czynni	3	135	19,2857	19,2857	0
0	0	1	trzy i więce	1	180	25,7143	0	25,7143
0	0	1	dwa czynni	2	0	0	0	0
0	1	1	trzy i więce	1	130	18,5714	1,42857	17,1429
0	0	1	jeden czyn	2	540	77,1429	68,5714	8,57143
1	0	1	trzy i więce	2	0	0	0	0
0	1	1	trzy i więce	1	0	0	0	0
0	1	1	trzy i więce	2	120	17,1429	0	17,1429
0	0	0	metabolicz	2	360	51,4286	0	51,4286
1	0	0	dwa czynni	2	200	28,5714	0	28,5714
0	0	1	dwa czynni	2	20	2,85714	0	2,85714
0	0	0	metabolicz	3	180	25,7143	8,57143	17,1429
0	0	0	jeden czyn	3	240	34,2857	25,7143	8,57143
0	0	1	dwa czynni	3	30	4,28571	4,28571	0
0	0	1	dwa czynni	2	60	8,57143	0	8,57143
0	0	1	dwa czynni	1	75	10,7143	0	10,7143
0	0	0	jeden czyn	1	240	34,2857	17,1429	17,1429
0	0	1	jeden czyn	3	0	0	0	0
0	0	1	jeden czyn	2	60	8,57143	8,57143	0
0	0	1	dwa czynni	2	0	0	0	0
0	0	1	dwa czynni	2	0	0	0	0
1	0	1	trzy i więce	1	420	60	0	60
1	0	1	trzy i więce	2	210	30	0	30
0	0	0	jeden czyn	2	270	38,5714	12,8571	25,7143
0	0	1	jeden czyn	1	60	8,57143	0	8,57143
1	0	1	trzy i więce	3	60	8,57143	0	8,57143
0	0	0	jeden czyn	3	600	85,7143	25,7143	60
0	0	1	jeden czyn	3	0	0	0	0
0	0	1	dwa czynni	2	70	10	10	0
0	0	1	dwa czynni	3	120	17,1429	0	17,1429
1	0	1	trzy i więce	1	60	8,57143	8,57143	0
0	1	1	trzy i więce	2	360	51,4286	0	51,4286
1	0	0	trzy i więce	3	60	8,57143	8,57143	0
0	0	1	trzy i więce	2	180	25,7143	0	25,7143
0	0	0	jeden czyn	2	120	17,1429	17,1429	0
0	0	0	metabolicz	2	0	0	0	0
0	1	1	trzy i więce	2	0	0	0	0
0	0	1	dwa czynni	1	0	0	0	0

0	0	0	metabolicz	3	60	8,57143	8,57143	0
1	0	1	trzy i więce	3	0	0	0	0
1	0	1	trzy i więce	1	1680	240	154,286	85,7143
1	1	1	trzy i więce	3	160	22,8571	0	22,8571
0	0	0	metabolicz	2	210	30	12,8571	17,1429
0	0	0	dwa czynni	1	420	60	0	60
0	0	1	dwa czynni	2	600	85,7143	60	25,7143
0	0	1	dwa czynni	2	0	0	0	0
0	0	0	metabolicz	3	120	17,1429	0	17,1429
0	0	0	jeden czyn	2	0	0	0	0
0	1	1	trzy i więce	3	0	0	0	0
0	0	0	jeden czyn	2	0	0	0	0
0	0	1	dwa czynni	2	120	17,1429	0	17,1429
0	0	0	metabolicz	3	1080	154,286	68,5714	85,7143
0	0	1	dwa czynni	2	0	0	0	0
1	1	1	trzy i więce	2	300	42,8571	8,57143	34,2857
0	1	1	trzy i więce	2	0	0	0	0
0	1	1	trzy i więce	3	0	0	0	0
0	0	1	jeden czyn	2	280	40	0	40
0	1	1	trzy i więce	1	120	17,1429	0	17,1429
1	0	1	trzy i więce	1	120	17,1429	0	17,1429
1	1	1	trzy i więce	2	60	8,57143	0	8,57143
0	1	0	dwa czynni	2	0	0	0	0
1	0	0	trzy i więce	1	45	6,42857	0	6,42857
1	1	1	trzy i więce	2	0	0	0	0
0	1	0	dwa czynni	2	390	55,7143	21,4286	34,2857
0	0	1	dwa czynni	3	0	0	0	0
0	1	1	trzy i więce	3	0	0	0	0
0	1	0	jeden czyn	2	0	0	0	0
0	0	1	dwa czynni	2	810	115,714	64,2857	51,4286
0	1	0	trzy i więce	1	0	0	0	0
0	0	0	jeden czyn	2	0	0	0	0
0	0	0	metabolicz	2	180	25,7143	0	25,7143
0	0	0	dwa czynni	2	0	0	0	0
0	0	1	trzy i więce	1	320	45,7143	17,1429	28,5714
0	0	0	jeden czyn	2	0	0	0	0
0	0	1	dwa czynni	3	0	0	0	0
0	0	0	jeden czyn	3	120	17,1429	0	17,1429
1	1	1	trzy i więce	3	210	30	0	30
0	0	0	metabolicz	2	240	34,2857	17,1429	17,1429
0	0	1	dwa czynni	1	420	60	0	60
1	1	1	trzy i więce	1	0	0	0	0
0	0	0	jeden czyn	2	135	19,2857	10,7143	8,57143
0	1	1	trzy i więce	2	210	30	12,8571	17,1429
0	0	0	jeden czyn	2	120	17,1429	0	17,1429
0	0	0	jeden czyn	2	60	8,57143	0	8,57143
0	0	1	dwa czynni	2	0	0	0	0
1	0	0	jeden czyn	3	120	17,1429	0	17,1429

0	0	0	jeden czyn	2	0	0	0	0
1	0	1	trzy i więce	1	45	6,42857	0	6,42857
0	0	1	dwa czynni	3	0	0	0	0
0	0	1	dwa czynni	3	0	0	0	0
1	1	1	trzy i więce	3	0	0	0	0
0	1	1	trzy i więce	2	0	0	0	0
1	0	0	trzy i więce	3	540	77,1429	17,1429	60
0	0	0	dwa czynni	2	0	0	0	0
0	0	1	dwa czynni	3	0	0	0	0
0	1	1	dwa czynni	1	0	0	0	0
0	0	1	dwa czynni	3	240	34,2857	8,57143	25,7143
0	0	1	dwa czynni	3	0	0	0	0
0	1	0	dwa czynni	2	0	0	0	0
0	0	0	jeden czyn	2	60	8,57143	0	8,57143
0	0	1	dwa czynni	3	0	0	0	0
0	0	0	jeden czyn	1	90	12,8571	0	12,8571
1	1	1	trzy i więce	2	420	60	0	60
0	0	0	jeden czyn	2	0	0	0	0
0	0	0	jeden czyn	3	300	42,8571	42,8571	0
0	0	1	dwa czynni	2	240	34,2857	0	34,2857
0	0	1	jeden czyn	1	240	34,2857	25,7143	8,57143
0	1	1	trzy i więce	2	0	0	0	0
0	0	1	dwa czynni	2	0	0	0	0
0	0	1	jeden czyn	2	360	51,4286	51,4286	0
1	1	1	trzy i więce	1	210	30	0	30
0	0	0	metabolicz	3	60	8,57143	6,42857	2,14286
1	0	0	dwa czynni	1	0	0	0	0
0	1	1	trzy i więce	3	0	0	0	0
0	0	1	dwa czynni	3	210	30	0	30
0	0	1	dwa czynni	2	60	8,57143	0	8,57143
0	0	1	dwa czynni	2	0	0	0	0
0	0	1	dwa czynni	3	255	36,4286	19,2857	17,1429
1	0	0	trzy i więce	2	60	8,57143	8,57143	0
0	1	1	dwa czynni	3	0	0	0	0
0	0	1	dwa czynni	3	810	115,714	12,8571	102,857
1	0	1	trzy i więce	2	520	74,2857	34,2857	40
0	0	1	trzy i więce	2	0	0	0	0
0	0	1	jeden czyn	2	310	44,2857	10	34,2857
0	0	0	jeden czyn	3	0	0	0	0
0	0	0	jeden czyn	3	180	25,7143	25,7143	0
0	0	0	jeden czyn	1	240	34,2857	8,57143	25,7143
0	0	0	metabolicz	2	120	17,1429	0	17,1429
0	0	0	metabolicz	2	0	0	0	0
0	0	0	metabolicz	2	210	30	0	30
0	0	0	jeden czyn	1	0	0	0	0
0	1	1	trzy i więce	3	0	0	0	0
0	0	0	jeden czyn	2	0	0	0	0
0	0	1	jeden czyn	3	90	12,8571	12,8571	0

0	0	0	metabolicz	2	360	51,4286	0	51,4286
0	1	0	dwa czynni	2	0	0	0	0
1	1	0	trzy i więc	3	0	0	0	0
1	1	1	trzy i więc	3	0	0	0	0
1	0	1	trzy i więc	3	60	8,57143	0	8,57143
0	0	0	metabolicz	1	180	25,7143	0	25,7143
0	0	0	metabolicz	1	0	0	0	0
1	0	1	trzy i więc	1	120	17,1429	0	17,1429
1	0	1	trzy i więc	3	0	0	0	0
0	0	1	jeden czyn	1	180	25,7143	0	25,7143
0	0	1	dwa czynni	3	160	22,8571	22,8571	0
1	0	1	trzy i więc	1	0	0	0	0
0	0	0	metabolicz	1	140	20	0	20
0	0	1	jeden czyn	2	0	0	0	0
1	1	1	trzy i więc	2	180	25,7143	17,1429	8,57143
0	1	1	trzy i więc	2	0	0	0	0
0	1	1	trzy i więc	2	300	42,8571	25,7143	17,1429
0	0	1	jeden czyn	1	0	0	0	0
1	1	1	trzy i więc	2	30	4,28571	0	4,28571
1	0	1	trzy i więc	2	540	77,1429	0	77,1429
1	0	1	trzy i więc	3	240	34,2857	0	34,2857
0	0	1	dwa czynni	2	420	60	0	60
0	0	0	metabolicz	1	0	0	0	0
0	0	1	jeden czyn	3	240	34,2857	0	34,2857
0	0	1	dwa czynni	3	0	0	0	0
1	1	1	trzy i więc	3	180	25,7143	25,7143	0
1	0	1	dwa czynni	2	0	0	0	0
0	0	0	metabolicz	3	0	0	0	0
0	0	1	dwa czynni	2	0	0	0	0
1	0	1	trzy i więc	2	0	0	0	0
0	0	1	dwa czynni	2	420	60	0	60
0	0	0	metabolicz	2	0	0	0	0
0	0	0	metabolicz	3	120	17,1429	17,1429	0
0	1	1	dwa czynni	2	0	0	0	0
0	0	1	jeden czyn	2	80	11,4286	0	11,4286
0	1	1	trzy i więc	3	270	38,5714	12,8571	25,7143
0	0	1	trzy i więc	2	120	17,1429	0	17,1429
0	0	1	jeden czyn	2	0	0	0	0
0	0	1	jeden czyn	2	0	0	0	0
0	0	0	metabolicz	2	60	8,57143	0	8,57143
0	1	1	trzy i więc	3	225	32,1429	6,42857	25,7143
0	1	1	trzy i więc	1	1260	180	0	180
1	0	1	trzy i więc	2	0	0	0	0
0	1	1	trzy i więc	3	40	5,71429	0	5,71429
0	0	0	jeden czyn	3	0	0	0	0
0	0	1	dwa czynni	3	180	25,7143	0	25,7143
1	1	0	trzy i więc	2	840	120	0	120
0	0	0	jeden czyn	2	240	34,2857	0	34,2857

0	0	1	jeden czyn	2	105	15	6,42857	8,57143
1	0	0	trzy i więc	2	360	51,4286	51,4286	0
0	0	0	metabolicz	2	420	60	0	60
0	0	0	metabolicz	2	1500	214,286	34,2857	180
1	0	1	trzy i więc	1	120	17,1429	0	17,1429
0	0	1	jeden czyn	2	90	12,8571	12,8571	0
1	0	1	trzy i więc	1	0	0	0	0
0	0	1	dwa czynni	1	150	21,4286	0	21,4286
0	0	0	jeden czyn	3	0	0	0	0
0	0	1	dwa czynni	2	120	17,1429	8,57143	8,57143
1	0	1	trzy i więc	1	0	0	0	0
0	0	0	jeden czyn	1	0	0	0	0
0	0	1	dwa czynni	2	0	0	0	0
1	1	1	trzy i więc	2	60	8,57143	8,57143	0
0	0	1	jeden czyn	3	0	0	0	0
0	0	1	dwa czynni	2	120	17,1429	0	17,1429
1	1	1	trzy i więc	1	0	0	0	0
0	0	1	dwa czynni	1	120	17,1429	8,57143	8,57143
0	0	0	metabolicz	2	105	15	15	0
0	0	0	jeden czyn	3	0	0	0	0
1	1	1	trzy i więc	2	0	0	0	0
0	0	1	jeden czyn	1	180	25,7143	0	25,7143
0	0	1	jeden czyn	1	0	0	0	0
1	0	1	trzy i więc	2	90	12,8571	8,57143	4,28571
0	0	1	jeden czyn	2	45	6,42857	6,42857	0
1	0	0	trzy i więc	1	60	8,57143	0	8,57143
0	0	1	jeden czyn	2	90	12,8571	0	12,8571
0	0	0	metabolicz	2	0	0	0	0
0	0	1	trzy i więc	2	0	0	0	0
0	1	1	trzy i więc	2	0	0	0	0
0	1	1	trzy i więc	2	420	60	0	60
0	0	1	dwa czynni	2	0	0	0	0
1	1	1	trzy i więc	3	0	0	0	0
0	0	1	jeden czyn	3	240	34,2857	8,57143	25,7143
0	0	1	dwa czynni	1	210	30	0	30
0	0	0	jeden czyn	2	120	17,1429	0	17,1429
0	0	1	dwa czynni	2	0	0	0	0
1	0	1	trzy i więc	1	105	15	6,42857	8,57143
0	0	1	dwa czynni	1	300	42,8571	0	42,8571
0	0	1	trzy i więc	2	0	0	0	0
0	0	1	dwa czynni	2	0	0	0	0
0	0	1	jeden czyn	1	0	0	0	0
1	0	1	trzy i więc	2	0	0	0	0
1	0	1	trzy i więc	2	240	34,2857	17,1429	17,1429
0	0	1	dwa czynni	2	60	8,57143	0	8,57143
0	0	0	jeden czyn	3	0	0	0	0
0	0	0	jeden czyn	1	540	77,1429	25,7143	51,4286
0	1	1	trzy i więc	1	0	0	0	0

0	0	0	metabolicz	3	0	0	0	0
1	0	1	trzy i więce	3	0	0	0	0
1	0	1	trzy i więce	1	480	68,5714	51,4286	17,1429
1	0	1	trzy i więce	2	0	0	0	0
0	0	1	dwa czynni	1	60	8,57143	0	8,57143
0	0	1	jeden czyn	3	130	18,5714	8,57143	10
0	0	0	metabolicz	3	450	64,2857	47,1429	17,1429
0	0	0	jeden czyn	3	60	8,57143	0	8,57143
1	0	0	dwa czynni	3	300	42,8571	42,8571	0
0	0	1	dwa czynni	3	150	21,4286	0	21,4286
0	0	1	dwa czynni	1	60	8,57143	0	8,57143
0	0	1	jeden czyn	3	120	17,1429	0	17,1429
0	0	0	metabolicz	1	840	120	0	120
0	1	1	trzy i więce	2	0	0	0	0
0	0	1	jeden czyn	2	60	8,57143	0	8,57143
1	1	1	trzy i więce	3	0	0	0	0
1	0	0	trzy i więce	1	120	17,1429	0	17,1429
1	0	1	trzy i więce	1	420	60	0	60
0	0	1	dwa czynni	1	120	17,1429	0	17,1429
0	1	1	trzy i więce	3	0	0	0	0
1	1	1	trzy i więce	1	190	27,1429	5,71429	21,4286
0	1	1	trzy i więce	2	900	128,571	0	128,571
0	0	0	jeden czyn	2	60	8,57143	0	8,57143
0	1	1	trzy i więce	1	840	120	0	120
0	1	1	trzy i więce	2	180	25,7143	0	25,7143
0	1	1	trzy i więce	3	120	17,1429	0	17,1429
1	0	0	dwa czynni	3	0	0	0	0
0	0	1	dwa czynni	3	0	0	0	0
0	0	0	metabolicz	1	0	0	0	0
1	0	1	trzy i więce	1	420	60	0	60
0	0	1	trzy i więce	2	0	0	0	0
0	1	1	trzy i więce	3	240	34,2857	0	34,2857
0	0	1	dwa czynni	2	120	17,1429	0	17,1429
0	0	1	dwa czynni	2	105	15	6,42857	8,57143
1	0	1	trzy i więce	2	1080	154,286	0	154,286
0	0	1	dwa czynni	3	80	11,4286	0	11,4286
0	1	1	trzy i więce	2	120	17,1429	0	17,1429
0	0	1	jeden czyn	2	0	0	0	0
1	1	1	trzy i więce	3	0	0	0	0
0	0	0	jeden czyn	2	120	17,1429	0	17,1429
0	0	1	dwa czynni	2	120	17,1429	0	17,1429
0	0	0	jeden czyn	1	0	0	0	0
1	0	1	dwa czynni	3	90	12,8571	12,8571	0
0	0	1	dwa czynni	3	0	0	0	0
0	1	0	jeden czyn	1	150	21,4286	0	21,4286
0	1	1	trzy i więce	2	420	60	0	60
0	0	0	metabolicz	3	0	0	0	0
0	0	1	trzy i więce	2	120	17,1429	17,1429	0

1	1	1	trzy i więce	2	90	12,8571	4,28571	8,57143
0	1	1	trzy i więce	2	60	8,57143	0	8,57143
0	1	1	trzy i więce	2	60	8,57143	0	8,57143
1	0	1	trzy i więce	2	0	0	0	0
0	1	1	trzy i więce	2	200	28,5714	0	28,5714
0	0	1	dwa czynni	3	240	34,2857	0	34,2857
0	1	1	trzy i więce	1	0	0	0	0
0	0	0	jeden czyn	3	45	6,42857	6,42857	0
0	0	0	jeden czyn	2	600	85,7143	51,4286	34,2857
0	0	1	jeden czyn	3	630	90	0	90
1	0	1	trzy i więce	2	60	8,57143	0	8,57143
0	1	1	trzy i więce	1	0	0	0	0
0	1	1	trzy i więce	3	0	0	0	0
0	0	1	jeden czyn	1	0	0	0	0
0	0	1	dwa czynni	3	0	0	0	0
0	0	0	metabolicz	3	120	17,1429	17,1429	0
0	0	1	dwa czynni	2	60	8,57143	0	8,57143
0	0	0	metabolicz	2	360	51,4286	0	51,4286
1	0	1	trzy i więce	2	0	0	0	0
0	0	0	metabolicz	2	360	51,4286	0	51,4286
0	1	0	dwa czynni	1	60	8,57143	8,57143	0
1	0	1	dwa czynni	3	210	30	0	30
1	0	1	trzy i więce	1	0	0	0	0
0	0	1	dwa czynni	1	0	0	0	0
0	0	1	dwa czynni	2	30	4,28571	0	4,28571
0	0	1	dwa czynni	2	0	0	0	0
0	0	1	dwa czynni	2	20	2,85714	0	2,85714
0	0	1	jeden czyn	3	180	25,7143	0	25,7143
0	0	0	metabolicz	2	120	17,1429	0	17,1429
0	0	1	dwa czynni	1	210	30	0	30
0	0	0	metabolicz	2	90	12,8571	4,28571	8,57143
1	0	1	trzy i więce	2	120	17,1429	17,1429	0
1	0	0	trzy i więce	3	180	25,7143	0	25,7143
0	1	1	trzy i więce	2	0	0	0	0
0	0	0	dwa czynni	2	0	0	0	0
0	0	1	jeden czyn	2	120	17,1429	0	17,1429
1	0	1	trzy i więce	1	60	8,57143	0	8,57143
1	0	0	dwa czynni	2	60	8,57143	0	8,57143
0	0	0	metabolicz	2	0	0	0	0
1	0	0	jeden czyn	1	360	51,4286	51,4286	0
0	0	1	dwa czynni	2	560	80	20	60
0	1	0	dwa czynni	3	0	0	0	0
1	0	1	trzy i więce	2	0	0	0	0
0	0	1	dwa czynni	3	15	2,14286	0	2,14286
1	1	1	trzy i więce	2	120	17,1429	0	17,1429
0	0	0	metabolicz	3	60	8,57143	0	8,57143
0	0	1	jeden czyn	2	0	0	0	0
0	0	0	metabolicz	1	105	15	15	0

1	0	1	trzy i więc	1	315	45	0	45
0	0	0	jeden czyn	2	30	4,28571	0	4,28571
0	0	0	jeden czyn	3	240	34,2857	0	34,2857
0	1	0	dwa czynni	1	0	0	0	0
0	0	1	dwa czynni	2	630	90	60	30
0	0	0	jeden czyn	2	30	4,28571	0	4,28571
0	0	1	dwa czynni	2	240	34,2857	0	34,2857
1	0	0	dwa czynni	1	60	8,57143	0	8,57143
0	0	0	metabolicz	1	390	55,7143	51,4286	4,28571
0	0	0	metabolicz	2	60	8,57143	0	8,57143
0	1	1	dwa czynni	1	0	0	0	0
1	1	1	trzy i więc	3	0	0	0	0
1	0	0	dwa czynni	2	240	34,2857	0	34,2857
0	0	1	dwa czynni	2	45	6,42857	6,42857	0
0	0	1	dwa czynni	2	0	0	0	0
0	0	0	jeden czyn	3	180	25,7143	25,7143	0
1	0	0	dwa czynni	2	360	51,4286	34,2857	17,1429
0	0	1	dwa czynni	3	0	0	0	0
0	0	0	jeden czyn	1	60	8,57143	0	8,57143
1	0	0	jeden czyn	2	320	45,7143	20	25,7143
1	1	1	trzy i więc	2	0	0	0	0
0	1	1	trzy i więc	2	120	17,1429	0	17,1429
0	0	1	jeden czyn	2	60	8,57143	8,57143	0
0	0	0	metabolicz	2	0	0	0	0
0	0	0	metabolicz	2	120	17,1429	0	17,1429
1	1	1	trzy i więc	3	0	0	0	0
0	0	1	dwa czynni	1	0	0	0	0
0	0	1	jeden czyn	1	120	17,1429	0	17,1429
0	1	0	dwa czynni	2	60	8,57143	0	8,57143
0	0	1	jeden czyn	3	180	25,7143	0	25,7143
0	0	0	jeden czyn	3	120	17,1429	0	17,1429
0	0	1	dwa czynni	2	0	0	0	0
1	0	1	trzy i więc	2	360	51,4286	0	51,4286
0	0	1	dwa czynni	2	130	18,5714	10	8,57143
0	0	1	jeden czyn	3	0	0	0	0
0	0	1	dwa czynni	2	80	11,4286	11,4286	0
1	1	1	trzy i więc	3	210	30	0	30
0	0	1	jeden czyn	3	80	11,4286	0	11,4286
1	0	1	trzy i więc	1	90	12,8571	0	12,8571
0	0	0	jeden czyn	2	0	0	0	0
0	0	1	dwa czynni	3	255	36,4286	6,42857	30
0	1	1	trzy i więc	3	0	0	0	0
0	0	1	dwa czynni	2	120	17,1429	0	17,1429
0	1	1	trzy i więc	3	0	0	0	0
0	0	0	jeden czyn	3	240	34,2857	0	34,2857
0	1	0	jeden czyn	2	60	8,57143	0	8,57143
1	0	1	trzy i więc	2	0	0	0	0
0	0	1	dwa czynni	3	240	34,2857	0	34,2857

1	0	0	trzy i więce	3	0	0	0	0
0	0	1	dwa czynni	2	0	0	0	0
0	1	1	dwa czynni	3	90	12,8571	12,8571	0
0	1	1	trzy i więce	2	0	0	0	0
0	0	0	metabolicz	2	120	17,1429	0	17,1429
0	0	0	jeden czyn	1	40	5,71429	0	5,71429
0	0	1	jeden czyn	2	60	8,57143	0	8,57143
0	0	1	dwa czynni	1	0	0	0	0
0	0	0	metabolicz	1	0	0	0	0
0	0	1	dwa czynni	3	0	0	0	0
0	0	0	metabolicz	1	420	60	0	60
0	0	0	jeden czyn	2	180	25,7143	0	25,7143
0	0	0	jeden czyn	2	0	0	0	0
0	0	1	dwa czynni	2	60	8,57143	0	8,57143
0	0	0	jeden czyn	1	60	8,57143	0	8,57143
0	0	0	jeden czyn	2	60	8,57143	0	8,57143
0	0	0	jeden czyn	3	40	5,71429	0	5,71429
0	0	1	dwa czynni	3	285	40,7143	30	10,7143
0	0	0	jeden czyn	3	0	0	0	0
0	1	0	jeden czyn	1	0	0	0	0
0	0	1	dwa czynni	1	240	34,2857	17,1429	17,1429
0	0	1	jeden czyn	2	540	77,1429	0	77,1429
0	0	0	metabolicz	1	240	34,2857	0	34,2857
0	1	1	dwa czynni	2	210	30	30	0
0	0	1	dwa czynni	3	120	17,1429	0	17,1429
0	0	0	jeden czyn	2	0	0	0	0
1	0	1	trzy i więce	2	0	0	0	0
0	1	1	trzy i więce	2	900	128,571	0	128,571
0	0	1	dwa czynni	3	180	25,7143	0	25,7143
0	0	1	dwa czynni	2	300	42,8571	8,57143	34,2857
0	1	1	trzy i więce	2	60	8,57143	0	8,57143
0	0	1	trzy i więce	1	390	55,7143	30	25,7143
0	0	1	dwa czynni	2	860	122,857	2,85714	120
0	1	0	jeden czyn	1	255	36,4286	36,4286	0
0	0	1	dwa czynni	2	360	51,4286	0	51,4286
0	0	0	metabolicz	1	0	0	0	0
0	0	0	metabolicz	1	30	4,28571	0	4,28571
1	1	1	trzy i więce	3	0	0	0	0
0	0	0	metabolicz	3	0	0	0	0
0	0	1	dwa czynni	1	0	0	0	0
1	0	1	trzy i więce	2	840	120	60	60
1	0	1	trzy i więce	3	240	34,2857	0	34,2857
1	0	1	trzy i więce	2	120	17,1429	0	17,1429
1	0	1	trzy i więce	1	120	17,1429	0	17,1429
0	0	0	metabolicz	2	840	120	0	120
0	0	1	dwa czynni	1	0	0	0	0
0	0	1	jeden czyn	2	180	25,7143	8,57143	17,1429
0	0	1	dwa czynni	2	180	25,7143	0	25,7143

0	0	0	metabolicz	1	0	0	0	0
0	0	0	dwa czynni	3	120	17,1429	0	17,1429
0	0	0	metabolicz	2	0	0	0	0
0	0	1	dwa czynni	2	0	0	0	0
1	0	1	dwa czynni	2	420	60	0	60
0	1	1	dwa czynni	2	360	51,4286	38,5714	12,8571
0	0	1	jeden czyn	3	0	0	0	0
0	0	0	jeden czyn	1	60	8,57143	0	8,57143
1	1	1	trzy i więc	1	0	0	0	0
0	0	0	metabolicz	2	180	25,7143	25,7143	0
0	0	1	trzy i więc	2	20	2,85714	2,85714	0
0	0	1	dwa czynni	3	45	6,42857	0	6,42857
1	1	0	trzy i więc	2	0	0	0	0
0	0	0	metabolicz	2	120	17,1429	17,1429	0
0	0	0	metabolicz	2	70	10	10	0
0	0	0	jeden czyn	3	0	0	0	0
0	1	0	dwa czynni	3	0	0	0	0
0	0	0	jeden czyn	1	420	60	0	60
0	0	1	trzy i więc	1	240	34,2857	0	34,2857
0	0	1	dwa czynni	2	180	25,7143	25,7143	0
1	0	0	trzy i więc	3	0	0	0	0
1	0	0	dwa czynni	1	0	0	0	0
1	0	0	dwa czynni	1	240	34,2857	0	34,2857
0	0	1	dwa czynni	2	0	0	0	0
1	0	1	trzy i więc	2	0	0	0	0
1	0	0	dwa czynni	1	0	0	0	0
0	1	0	trzy i więc	3	420	60	0	60
0	0	0	metabolicz	3	350	50	0	50
0	0	0	metabolicz	3	180	25,7143	0	25,7143
1	0	0	trzy i więc	2	120	17,1429	0	17,1429
0	1	1	trzy i więc	3	360	51,4286	0	51,4286
0	0	1	dwa czynni	1	60	8,57143	0	8,57143
0	1	1	trzy i więc	3	30	4,28571	4,28571	0
0	0	1	jeden czyn	1	60	8,57143	0	8,57143
0	0	1	dwa czynni	2	375	53,5714	8,57143	45
0	0	1	jeden czyn	2	780	111,429	51,4286	60
0	0	0	jeden czyn	1	0	0	0	0
0	0	1	jeden czyn	1	240	34,2857	0	34,2857
0	0	0	metabolicz	1	0	0	0	0
0	0	0	jeden czyn	3	240	34,2857	0	34,2857
0	0	0	metabolicz	3	120	17,1429	17,1429	0
0	0	0	dwa czynni	1	0	0	0	0
1	0	1	trzy i więc	2	0	0	0	0
1	0	0	trzy i więc	2	180	25,7143	0	25,7143
0	0	1	jeden czyn	3	0	0	0	0
1	0	1	trzy i więc	3	180	25,7143	0	25,7143
1	0	0	jeden czyn	3	0	0	0	0
0	0	0	metabolicz	3	240	34,2857	8,57143	25,7143

0	1	1	trzy i więce	2	0	0	0	0
0	0	0	dwa czynni	2	0	0	0	0
0	0	1	dwa czynni	2	420	60	0	60
0	0	1	dwa czynni	2	120	17,1429	17,1429	0
1	0	1	trzy i więce	2	300	42,8571	0	42,8571
0	0	1	jeden czyn	3	540	77,1429	77,1429	0
0	0	1	jeden czyn	3	420	60	0	60
0	0	0	metabolicz	1	0	0	0	0
0	1	1	trzy i więce	2	0	0	0	0
0	0	1	trzy i więce	1	60	8,57143	0	8,57143
1	0	1	trzy i więce	2	0	0	0	0
1	1	1	trzy i więce	1	0	0	0	0
0	0	0	metabolicz	1	0	0	0	0
0	0	1	dwa czynni	2	240	34,2857	17,1429	17,1429
0	0	0	metabolicz	1	0	0	0	0
0	0	0	metabolicz	2	480	68,5714	17,1429	51,4286
0	0	1	jeden czyn	3	180	25,7143	0	25,7143
0	0	1	dwa czynni	3	0	0	0	0
0	0	1	dwa czynni	3	0	0	0	0
0	0	0	jeden czyn	3	0	0	0	0
0	0	0	dwa czynni	2	60	8,57143	0	8,57143
0	0	1	dwa czynni	2	420	60	0	60
0	0	0	metabolicz	1	660	94,2857	8,57143	85,7143
0	0	0	metabolicz	2	150	21,4286	4,28571	17,1429
0	0	1	trzy i więce	2	420	60	0	60
0	1	1	trzy i więce	3	0	0	0	0
0	0	1	dwa czynni	1	120	17,1429	0	17,1429
1	1	1	trzy i więce	1	180	25,7143	0	25,7143
0	0	1	dwa czynni	2	150	21,4286	21,4286	0
0	0	0	metabolicz	3	240	34,2857	34,2857	0
0	0	0	metabolicz	2	120	17,1429	0	17,1429
0	0	0	jeden czyn	2	120	17,1429	0	17,1429
0	0	1	dwa czynni	1	0	0	0	0
0	0	1	jeden czyn	1	0	0	0	0
0	1	1	trzy i więce	2	60	8,57143	0	8,57143
1	1	0	trzy i więce	2	0	0	0	0
0	0	1	dwa czynni	3	0	0	0	0
1	0	1	trzy i więce	2	45	6,42857	6,42857	0
1	0	1	trzy i więce	2	60	8,57143	0	8,57143
0	1	1	trzy i więce	2	0	0	0	0
1	0	0	dwa czynni	3	300	42,8571	42,8571	0
0	0	1	dwa czynni	2	0	0	0	0
0	1	1	trzy i więce	3	150	21,4286	0	21,4286
0	0	0	metabolicz	2	240	34,2857	25,7143	8,57143
0	0	0	jeden czyn	3	0	0	0	0
0	0	1	dwa czynni	1	60	8,57143	0	8,57143
0	0	1	dwa czynni	3	100	14,2857	5,71429	8,57143
0	1	1	dwa czynni	2	120	17,1429	0	17,1429

0	0	1	jeden czyn	2	0	0	0	0
0	0	1	jeden czyn	1	300	42,8571	0	42,8571
0	1	1	dwa czynni	1	360	51,4286	0	51,4286
1	0	0	dwa czynni	2	120	17,1429	0	17,1429
1	0	1	trzy i więc	2	60	8,57143	8,57143	0
0	0	1	jeden czyn	3	60	8,57143	8,57143	0
0	0	1	dwa czynni	2	0	0	0	0
1	1	1	trzy i więc	1	0	0	0	0
0	0	0	jeden czyn	3	165	23,5714	6,42857	17,1429
1	0	1	trzy i więc	3	210	30	8,57143	21,4286
0	0	1	dwa czynni	3	0	0	0	0
1	0	0	trzy i więc	1	60	8,57143	0	8,57143
0	0	0	jeden czyn	2	60	8,57143	0	8,57143
0	0	0	jeden czyn	3	300	42,8571	25,7143	17,1429
0	0	1	dwa czynni	3	285	40,7143	15	25,7143
0	1	1	trzy i więc	1	0	0	0	0
1	0	1	trzy i więc	2	0	0	0	0
0	0	1	jeden czyn	2	120	17,1429	0	17,1429
0	0	1	dwa czynni	2	0	0	0	0
0	0	1	trzy i więc	2	0	0	0	0
0	0	1	dwa czynni	1	0	0	0	0
0	0	0	jeden czyn	2	0	0	0	0
0	0	1	dwa czynni	3	0	0	0	0
0	0	0	metabolicz	2	810	115,714	12,8571	102,857
0	0	1	dwa czynni	2	360	51,4286	0	51,4286
0	1	1	trzy i więc	2	105	15	15	0
0	0	0	jeden czyn	3	430	61,4286	1,42857	60
1	0	0	trzy i więc	2	60	8,57143	0	8,57143
0	0	0	jeden czyn	1	120	17,1429	0	17,1429
0	0	1	jeden czyn	2	120	17,1429	0	17,1429
0	0	1	dwa czynni	2	0	0	0	0
0	0	1	jeden czyn	3	330	47,1429	17,1429	30
1	0	1	trzy i więc	3	0	0	0	0
0	0	1	dwa czynni	2	0	0	0	0
0	0	1	dwa czynni	2	0	0	0	0
0	0	0	metabolicz	1	60	8,57143	0	8,57143
0	1	1	trzy i więc	1	60	8,57143	0	8,57143
0	0	0	metabolicz	2	300	42,8571	42,8571	0
0	0	1	trzy i więc	2	120	17,1429	0	17,1429
0	0	1	dwa czynni	2	0	0	0	0
0	0	1	dwa czynni	2	210	30	0	30
0	0	1	trzy i więc	1	100	14,2857	0	14,2857
0	0	1	jeden czyn	3	310	44,2857	30	14,2857
0	0	0	jeden czyn	2	120	17,1429	0	17,1429
0	0	1	dwa czynni	2	60	8,57143	0	8,57143
0	0	1	jeden czyn	1	60	8,57143	0	8,57143
0	0	0	jeden czyn	2	420	60	0	60
0	0	1	dwa czynni	3	340	48,5714	40	8,57143

1	1	0	trzy i więce	3	0	0	0	0
0	0	0	jeden czyn	2	0	0	0	0
0	0	0	metabolicz	2	330	47,1429	17,1429	30
0	0	0	jeden czyn	2	210	30	12,8571	17,1429
0	1	1	trzy i więce	1	100	14,2857	0	14,2857
0	0	1	dwa czynni	1	240	34,2857	0	34,2857
0	0	1	dwa czynni	1	0	0	0	0
0	0	0	metabolicz	2	480	68,5714	0	68,5714
0	0	0	jeden czyn	3	135	19,2857	6,42857	12,8571
0	0	0	jeden czyn	3	120	17,1429	0	17,1429
0	0	0	jeden czyn	2	240	34,2857	0	34,2857
0	0	1	dwa czynni	3	300	42,8571	0	42,8571
0	1	1	trzy i więce	2	0	0	0	0
0	1	0	dwa czynni	2	0	0	0	0
0	0	1	jeden czyn	1	110	15,7143	7,14286	8,57143
0	0	1	dwa czynni	2	180	25,7143	25,7143	0
1	0	1	trzy i więce	2	210	30	30	0
0	1	1	trzy i więce	3	45	6,42857	0	6,42857
1	0	1	trzy i więce	2	10	1,42857	0	1,42857
0	0	0	dwa czynni	3	390	55,7143	4,28571	51,4286
0	0	1	dwa czynni	1	0	0	0	0
0	0	0	jeden czyn	1	300	42,8571	12,8571	30
0	0	0	jeden czyn	3	60	8,57143	0	8,57143
0	0	0	jeden czyn	3	120	17,1429	17,1429	0
0	0	0	metabolicz	2	360	51,4286	0	51,4286
0	0	1	dwa czynni	2	0	0	0	0
0	0	1	dwa czynni	2	0	0	0	0
0	0	1	jeden czyn	1	150	21,4286	4,28571	17,1429
0	0	1	dwa czynni	2	0	0	0	0
0	0	1	dwa czynni	1	60	8,57143	0	8,57143
1	0	1	trzy i więce	2	0	0	0	0
0	0	0	jeden czyn	3	60	8,57143	0	8,57143
0	1	0	jeden czyn	3	60	8,57143	0	8,57143
0	0	0	jeden czyn	2	30	4,28571	0	4,28571
1	1	1	trzy i więce	2	0	0	0	0
0	0	0	metabolicz	2	60	8,57143	0	8,57143
0	1	0	dwa czynni	3	0	0	0	0
0	0	1	dwa czynni	2	0	0	0	0
0	0	1	dwa czynni	1	60	8,57143	8,57143	0
0	0	1	dwa czynni	2	0	0	0	0
0	0	1	dwa czynni	3	60	8,57143	0	8,57143
0	0	1	dwa czynni	2	0	0	0	0
0	0	0	metabolicz	3	120	17,1429	0	17,1429
1	0	1	trzy i więce	1	60	8,57143	0	8,57143
0	1	1	trzy i więce	2	0	0	0	0
0	0	0	metabolicz	2	240	34,2857	34,2857	0
0	0	1	jeden czyn	2	1020	145,714	60	85,7143
1	0	0	trzy i więce	3	0	0	0	0

0	1	1	trzy i więce	3	85	12,1429	3,57143	8,57143
0	0	1	trzy i więce	1	120	17,1429	0	17,1429
1	0	1	trzy i więce	2	0	0	0	0
0	0	0	jeden czyn	3	0	0	0	0
0	0	1	jeden czyn	1	280	40	10	30
1	1	1	trzy i więce	2	0	0	0	0
1	0	1	trzy i więce	1	0	0	0	0
0	0	1	dwa czynni	3	0	0	0	0
1	0	0	dwa czynni	3	45	6,42857	6,42857	0
0	0	0	metabolicz	2	0	0	0	0
0	0	1	dwa czynni	2	0	0	0	0
0	0	1	jeden czyn	1	420	60	34,2857	25,7143
1	0	0	dwa czynni	1	60	8,57143	0	8,57143
1	0	1	trzy i więce	3	840	120	0	120
1	0	1	trzy i więce	1	0	0	0	0
0	0	0	jeden czyn	2	0	0	0	0
0	1	1	trzy i więce	1	0	0	0	0
0	0	1	dwa czynni	2	60	8,57143	0	8,57143
0	0	1	jeden czyn	2	120	17,1429	0	17,1429
0	0	1	jeden czyn	2	240	34,2857	0	34,2857
0	0	0	jeden czyn	3	270	38,5714	38,5714	0
0	0	1	dwa czynni	2	120	17,1429	0	17,1429
0	0	1	dwa czynni	3	45	6,42857	6,42857	0
0	0	0	jeden czyn	2	0	0	0	0
0	0	0	metabolicz	1	0	0	0	0
0	0	0	metabolicz	2	180	25,7143	25,7143	0
0	1	0	jeden czyn	2	1080	154,286	102,857	51,4286
0	1	1	trzy i więce	2	120	17,1429	17,1429	0
0	0	0	metabolicz	2	105	15	15	0
0	0	0	jeden czyn	2	0	0	0	0
1	0	0	trzy i więce	3	120	17,1429	0	17,1429
0	0	0	dwa czynni	2	0	0	0	0
0	0	1	dwa czynni	2	0	0	0	0
1	0	1	trzy i więce	2	0	0	0	0
0	0	1	jeden czyn	1	60	8,57143	0	8,57143
0	0	1	dwa czynni	3	315	45	0	45
1	0	0	trzy i więce	1	0	0	0	0
0	0	0	metabolicz	1	1260	180	0	180
0	0	1	jeden czyn	2	0	0	0	0
1	0	0	dwa czynni	1	0	0	0	0
0	0	1	jeden czyn	2	420	60	25,7143	34,2857
0	0	0	jeden czyn	3	60	8,57143	0	8,57143
0	0	1	dwa czynni	3	0	0	0	0
0	0	0	metabolicz	3	45	6,42857	6,42857	0
0	0	1	trzy i więce	2	105	15	6,42857	8,57143
1	1	1	trzy i więce	2	0	0	0	0
1	0	1	trzy i więce	3	30	4,28571	0	4,28571
0	1	1	trzy i więce	3	120	17,1429	0	17,1429

1	1	1	trzy i więce	2	360	51,4286	0	51,4286
0	1	0	dwa czynni	1	0	0	0	0
0	0	0	metabolicz	2	600	85,7143	25,7143	60
0	0	1	dwa czynni	2	120	17,1429	0	17,1429
0	0	1	dwa czynni	2	150	21,4286	17,1429	4,28571
0	1	1	trzy i więce	2	150	21,4286	0	21,4286
0	0	0	jeden czyn	3	0	0	0	0
0	0	1	dwa czynni	3	150	21,4286	21,4286	0
1	0	0	dwa czynni	2	60	8,57143	0	8,57143
0	0	0	metabolicz	3	315	45	42,8571	2,14286
0	0	1	dwa czynni	1	60	8,57143	0	8,57143
1	0	1	trzy i więce	2	120	17,1429	8,57143	8,57143
1	1	1	trzy i więce	1	480	68,5714	0	68,5714
0	0	1	dwa czynni	3	0	0	0	0
1	0	1	trzy i więce	3	60	8,57143	0	8,57143
0	0	1	dwa czynni	1	0	0	0	0
0	0	1	jeden czyn	3	0	0	0	0
0	0	0	jeden czyn	3	60	8,57143	0	8,57143
0	1	1	trzy i więce	1	360	51,4286	0	51,4286
0	1	1	trzy i więce	2	0	0	0	0
0	1	0	dwa czynni	1	180	25,7143	25,7143	0
0	0	1	dwa czynni	3	120	17,1429	0	17,1429
0	0	1	jeden czyn	1	0	0	0	0
0	0	1	dwa czynni	3	45	6,42857	6,42857	0
0	0	1	dwa czynni	2	200	28,5714	8,57143	20
0	1	1	trzy i więce	3	0	0	0	0
0	1	0	dwa czynni	2	280	40	0	40
0	0	1	dwa czynni	2	420	60	0	60
0	0	0	jeden czyn	2	0	0	0	0
0	0	1	dwa czynni	2	0	0	0	0
1	0	1	trzy i więce	2	0	0	0	0
0	0	1	trzy i więce	2	360	51,4286	17,1429	34,2857
1	0	1	trzy i więce	2	300	42,8571	25,7143	17,1429
1	0	1	trzy i więce	2	420	60	0	60
1	0	1	trzy i więce	2	840	120	0	120
0	0	1	dwa czynni	3	30	4,28571	0	4,28571
0	0	1	dwa czynni	2	180	25,7143	0	25,7143
0	0	1	jeden czyn	1	0	0	0	0
0	0	0	jeden czyn	1	10	1,42857	0	1,42857
0	0	0	jeden czyn	2	80	11,4286	2,85714	8,57143
0	0	1	dwa czynni	3	480	68,5714	0	68,5714
0	0	1	dwa czynni	2	180	25,7143	0	25,7143
0	1	1	trzy i więce	2	70	10	10	0
0	0	0	jeden czyn	2	75	10,7143	10,7143	0
0	0	0	metabolicz	2	0	0	0	0
0	1	1	trzy i więce	2	20	2,85714	2,85714	0
1	0	1	trzy i więce	2	0	0	0	0
1	0	0	trzy i więce	2	920	131,429	11,4286	120

0	0	1	dwa czynni	2	40	5,71429	0	5,71429
0	0	1	dwa czynni	2	0	0	0	0
0	0	1	jeden czyn	3	330	47,1429	15	32,1429
1	1	1	trzy i więce	1	90	12,8571	0	12,8571
0	0	1	dwa czynni	2	240	34,2857	17,1429	17,1429
1	1	1	trzy i więce	1	120	17,1429	0	17,1429
0	0	1	dwa czynni	2	60	8,57143	0	8,57143
0	0	1	jeden czyn	2	300	42,8571	17,1429	25,7143
1	0	1	trzy i więce	3	0	0	0	0
0	1	1	trzy i więce	2	0	0	0	0
0	0	1	jeden czyn	1	0	0	0	0
1	0	1	trzy i więce	2	0	0	0	0
0	0	1	dwa czynni	2	0	0	0	0
0	1	1	dwa czynni	1	0	0	0	0
0	0	1	dwa czynni	1	240	34,2857	0	34,2857
0	0	1	dwa czynni	2	210	30	0	30
0	0	1	trzy i więce	1	900	128,571	128,571	0
1	0	1	trzy i więce	3	0	0	0	0
0	0	1	dwa czynni	3	240	34,2857	0	34,2857
0	0	0	jeden czyn	3	210	30	0	30
1	1	1	trzy i więce	2	420	60	0	60
1	1	1	trzy i więce	3	120	17,1429	0	17,1429
1	0	0	dwa czynni	1	0	0	0	0
0	0	1	dwa czynni	2	60	8,57143	0	8,57143
0	1	0	jeden czyn	2	240	34,2857	25,7143	8,57143
1	0	1	trzy i więce	2	140	20	11,4286	8,57143
0	0	0	jeden czyn	1	0	0	0	0
0	0	1	dwa czynni	3	90	12,8571	8,57143	4,28571
1	1	1	trzy i więce	1	100	14,2857	0	14,2857
1	0	0	jeden czyn	2	660	94,2857	25,7143	68,5714
0	0	1	jeden czyn	2	50	7,14286	0	7,14286
0	0	1	dwa czynni	3	840	120	0	120
0	0	1	dwa czynni	3	120	17,1429	0	17,1429
0	0	1	dwa czynni	2	150	21,4286	0	21,4286
0	0	0	jeden czyn	3	60	8,57143	0	8,57143
0	0	1	jeden czyn	2	0	0	0	0
0	1	1	trzy i więce	2	60	8,57143	0	8,57143
0	0	1	dwa czynni	3	540	77,1429	25,7143	51,4286
1	0	1	trzy i więce	2	0	0	0	0
1	0	0	dwa czynni	2	0	0	0	0
1	0	1	trzy i więce	3	0	0	0	0
0	1	0	dwa czynni	1	210	30	21,4286	8,57143
0	0	1	dwa czynni	2	360	51,4286	51,4286	0
0	0	1	dwa czynni	2	0	0	0	0
0	1	0	jeden czyn	1	840	120	0	120
0	0	1	jeden czyn	2	120	17,1429	0	17,1429
1	0	1	trzy i więce	1	30	4,28571	0	4,28571
0	0	1	dwa czynni	1	0	0	0	0

0	0	1	dwa czynni	2	70	10	10	0
0	0	1	dwa czynni	2	45	6,42857	6,42857	0
0	1	1	trzy i więce	2	0	0	0	0
1	0	1	trzy i więce	2	240	34,2857	0	34,2857
0	0	0	jeden czyn	3	0	0	0	0
0	0	0	jeden czyn	3	0	0	0	0
0	0	0	jeden czyn	1	60	8,57143	0	8,57143
0	0	1	jeden czyn	2	180	25,7143	8,57143	17,1429
0	0	1	jeden czyn	2	200	28,5714	8,57143	20
0	0	1	dwa czynni	3	180	25,7143	0	25,7143
1	0	0	jeden czyn	1	30	4,28571	0	4,28571
0	0	1	trzy i więce	1	0	0	0	0
1	1	1	trzy i więce	2	0	0	0	0
0	0	0	jeden czyn	1	60	8,57143	0	8,57143
0	0	0	metabolicz	2	660	94,2857	60	34,2857
0	0	0	metabolicz	2	180	25,7143	17,1429	8,57143
0	0	1	jeden czyn	2	0	0	0	0
0	0	1	jeden czyn	2	60	8,57143	0	8,57143
0	0	1	dwa czynni	2	150	21,4286	8,57143	12,8571
1	0	1	trzy i więce	1	0	0	0	0
0	1	1	trzy i więce	1	0	0	0	0
0	0	0	metabolicz	2	0	0	0	0
1	0	1	trzy i więce	2	60	8,57143	0	8,57143
0	0	0	metabolicz	1	120	17,1429	0	17,1429
0	0	1	jeden czyn	3	60	8,57143	0	8,57143
0	0	0	metabolicz	3	240	34,2857	8,57143	25,7143
0	1	1	trzy i więce	2	240	34,2857	34,2857	0
0	1	1	trzy i więce	2	120	17,1429	0	17,1429
0	0	0	jeden czyn	2	140	20	0	20
0	0	1	dwa czynni	2	240	34,2857	0	34,2857
1	0	1	trzy i więce	1	0	0	0	0
0	0	0	metabolicz	3	100	14,2857	0	14,2857
0	0	1	dwa czynni	2	0	0	0	0
0	0	0	metabolicz	2	90	12,8571	0	12,8571
0	0	1	jeden czyn	2	30	4,28571	0	4,28571
1	0	1	trzy i więce	2	0	0	0	0
0	0	1	dwa czynni	2	60	8,57143	0	8,57143
0	1	0	dwa czynni	2	0	0	0	0
0	1	1	trzy i więce	3	0	0	0	0
1	1	0	trzy i więce	3	140	20	20	0
0	0	1	dwa czynni	3	0	0	0	0
0	0	1	dwa czynni	3	0	0	0	0
0	1	1	dwa czynni	2	90	12,8571	4,28571	8,57143
0	0	1	jeden czyn	2	0	0	0	0
0	0	1	dwa czynni	2	15	2,14286	0	2,14286
0	0	0	jeden czyn	2	160	22,8571	10	12,8571
0	1	1	trzy i więce	3	0	0	0	0
0	0	1	dwa czynni	2	840	120	0	120

1	0	0	dwa czynni	3	0	0	0	0
0	0	1	trzy i więce	2	108	15,4286	6,85714	8,57143
0	0	0	metabolicz	3	180	25,7143	25,7143	0
0	0	0	metabolicz	2	0	0	0	0
1	0	1	dwa czynni	2	0	0	0	0
1	1	1	trzy i więce	2	60	8,57143	0	8,57143
0	0	0	metabolicz	3	350	50	0	50
1	0	1	trzy i więce	1	0	0	0	0
0	1	0	dwa czynni	1	720	102,857	102,857	0
0	0	0	jeden czyn	2	0	0	0	0
0	0	1	dwa czynni	2	0	0	0	0
1	0	1	trzy i więce	3	0	0	0	0
0	0	0	jeden czyn	3	120	17,1429	8,57143	8,57143
0	0	0	jeden czyn	2	135	19,2857	10,7143	8,57143
0	1	1	trzy i więce	2	60	8,57143	0	8,57143
0	0	1	jeden czyn	1	70	10	10	0
1	0	1	trzy i więce	2	45	6,42857	6,42857	0
0	0	1	jeden czyn	1	0	0	0	0
0	0	1	jeden czyn	2	0	0	0	0
0	0	1	jeden czyn	2	255	36,4286	6,42857	30
0	0	0	metabolicz	2	420	60	0	60
0	0	0	jeden czyn	2	0	0	0	0
0	0	1	dwa czynni	1	180	25,7143	25,7143	0
1	0	1	trzy i więce	1	120	17,1429	0	17,1429
0	1	0	dwa czynni	1	120	17,1429	0	17,1429
1	0	1	trzy i więce	3	150	21,4286	12,8571	8,57143
1	0	0	trzy i więce	3	410	58,5714	20	38,5714
0	0	1	jeden czyn	2	0	0	0	0
0	0	1	dwa czynni	3	0	0	0	0
1	0	1	trzy i więce	2	300	42,8571	0	42,8571
0	0	0	metabolicz	2	135	19,2857	6,42857	12,8571
0	1	1	trzy i więce	2	600	85,7143	85,7143	0
0	0	0	metabolicz	1	60	8,57143	0	8,57143
1	0	1	trzy i więce	1	0	0	0	0
1	0	1	dwa czynni	3	105	15	15	0
1	0	0	dwa czynni	1	420	60	8,57143	51,4286
0	0	1	dwa czynni	3	0	0	0	0
1	1	1	trzy i więce	2	30	4,28571	0	4,28571
0	0	1	dwa czynni	2	165	23,5714	15	8,57143
0	0	1	dwa czynni	2	120	17,1429	8,57143	8,57143
0	0	0	jeden czyn	2	60	8,57143	0	8,57143
0	0	0	metabolicz	1	300	42,8571	25,7143	17,1429
0	0	1	dwa czynni	2	0	0	0	0
0	0	0	metabolicz	3	90	12,8571	0	12,8571
0	0	0	dwa czynni	3	150	21,4286	12,8571	8,57143
0	1	1	trzy i więce	2	420	60	0	60
0	0	1	dwa czynni	3	315	45	15	30
0	0	1	trzy i więce	1	90	12,8571	0	12,8571

0	*0*	*0*	metabolicz	1	*120*	*17,1429*	*17,1429*	*0*
0	*0*	*1*	dwa czynni	3	*180*	*25,7143*	*0*	*25,7143*
0	*1*	*1*	trzy i więce	2	*120*	*17,1429*	*17,1429*	*0*
0	*0*	*1*	trzy i więce	2	*0*	*0*	*0*	*0*
0	*0*	*1*	dwa czynni	2	*360*	*51,4286*	*0*	*51,4286*
0	*0*	*1*	dwa czynni	2	*60*	*8,57143*	*0*	*8,57143*
0	*0*	*0*	jeden czyn	3	*60*	*8,57143*	*8,57143*	*0*
0	*1*	*1*	trzy i więce	2	*120*	*17,1429*	*17,1429*	*0*
0	*0*	*1*	dwa czynni	2	*360*	*51,4286*	*0*	*51,4286*
0	*0*	*1*	jeden czyn	1	*420*	*60*	*0*	*60*
0	*1*	*0*	jeden czyn	2	*70*	*10*	*0*	*10*
0	*0*	*0*	metabolicz	1	*840*	*120*	*0*	*120*
0	*0*	*1*	jeden czyn	1	*0*	*0*	*0*	*0*
0	*0*	*0*	jeden czyn	1	*120*	*17,1429*	*0*	*17,1429*
0	*0*	*0*	jeden czyn	2	*40*	*5,71429*	*0*	*5,71429*
0	*0*	*1*	jeden czyn	2	*390*	*55,7143*	*38,5714*	*17,1429*
0	*0*	*1*	dwa czynni	2	*0*	*0*	*0*	*0*
1	*1*	*1*	trzy i więce	2	*630*	*90*	*30*	*60*
1	*0*	*0*	dwa czynni	2	*0*	*0*	*0*	*0*
0	*1*	*0*	trzy i więce	2	*420*	*60*	*0*	*60*
1	*0*	*0*	dwa czynni	2	*360*	*51,4286*	*0*	*51,4286*
0	*0*	*1*	jeden czyn	2	*120*	*17,1429*	*0*	*17,1429*
0	*0*	*0*	metabolicz	3	*0*	*0*	*0*	*0*
0	*1*	*1*	trzy i więce	3	*300*	*42,8571*	*0*	*42,8571*
1	*0*	*0*	dwa czynni	3	*0*	*0*	*0*	*0*
0	*0*	*0*	dwa czynni	1	*360*	*51,4286*	*0*	*51,4286*
0	*0*	*1*	dwa czynni	3	*180*	*25,7143*	*0*	*25,7143*
0	*1*	*1*	trzy i więce	2	*60*	*8,57143*	*0*	*8,57143*
0	*0*	*1*	dwa czynni	2	*0*	*0*	*0*	*0*
0	*1*	*0*	dwa czynni	2	*60*	*8,57143*	*0*	*8,57143*
0	*1*	*0*	dwa czynni	2	*180*	*25,7143*	*21,4286*	*4,28571*
0	*0*	*0*	dwa czynni	2	*420*	*60*	*0*	*60*
0	*0*	*1*	dwa czynni	3	*120*	*17,1429*	*17,1429*	*0*
0	*1*	*1*	trzy i więce	1	*0*	*0*	*0*	*0*
0	*0*	*1*	dwa czynni	2	*150*	*21,4286*	*0*	*21,4286*
0	*0*	*0*	jeden czyn	2	*0*	*0*	*0*	*0*
0	*0*	*1*	trzy i więce	1	*450*	*64,2857*	*42,8571*	*21,4286*
0	*1*	*1*	trzy i więce	3	*20*	*2,85714*	*2,85714*	*0*
0	*0*	*1*	jeden czyn	1	*60*	*8,57143*	*0*	*8,57143*
0	*1*	*1*	trzy i więce	2	*0*	*0*	*0*	*0*
0	*0*	*1*	dwa czynni	2	*60*	*8,57143*	*0*	*8,57143*
0	*0*	*1*	dwa czynni	2	*30*	*4,28571*	*0*	*4,28571*
0	*1*	*1*	dwa czynni	3	*170*	*24,2857*	*12,8571*	*11,4286*
0	*0*	*1*	jeden czyn	3	*0*	*0*	*0*	*0*
0	*1*	*1*	trzy i więce	3	*360*	*51,4286*	*0*	*51,4286*
0	*0*	*1*	trzy i więce	2	*840*	*120*	*0*	*120*
0	*0*	*0*	metabolicz	2	*0*	*0*	*0*	*0*
0	*0*	*1*	jeden czyn	2	*180*	*25,7143*	*0*	*25,7143*

0	0	0	metabolicz	2	480	68,5714	34,2857	34,2857
0	0	0	metabolicz	1	0	0	0	0
0	0	1	jeden czyn	2	260	37,1429	17,1429	20
0	0	0	jeden czyn	2	0	0	0	0
0	0	0	jeden czyn	2	0	0	0	0
0	0	1	dwa czynni	1	400	57,1429	17,1429	40
1	0	1	dwa czynni	3	120	17,1429	8,57143	8,57143
0	0	0	jeden czyn	3	420	60	0	60
0	0	1	dwa czynni	3	0	0	0	0
0	0	0	metabolicz	3	420	60	0	60
0	0	1	dwa czynni	2	30	4,28571	4,28571	0
1	1	0	trzy i więc	1	0	0	0	0
1	0	1	trzy i więc	2	160	22,8571	0	22,8571
0	0	1	dwa czynni	3	0	0	0	0
0	0	0	jeden czyn	1	210	30	0	30
1	0	1	trzy i więc	2	60	8,57143	0	8,57143
0	0	1	dwa czynni	3	0	0	0	0
0	1	1	trzy i więc	2	120	17,1429	0	17,1429
0	0	0	metabolicz	3	120	17,1429	0	17,1429
1	1	1	trzy i więc	1	150	21,4286	0	21,4286
1	0	1	trzy i więc	1	0	0	0	0
0	0	0	dwa czynni	1	300	42,8571	8,57143	34,2857
0	1	1	trzy i więc	2	120	17,1429	0	17,1429
0	0	1	dwa czynni	2	210	30	0	30
1	1	0	trzy i więc	2	0	0	0	0
1	0	0	dwa czynni	3	60	8,57143	8,57143	0
0	0	1	dwa czynni	3	0	0	0	0
1	0	1	trzy i więc	2	0	0	0	0
0	1	1	trzy i więc	1	0	0	0	0
0	0	0	dwa czynni	2	0	0	0	0
1	0	0	dwa czynni	1	0	0	0	0
0	0	0	jeden czyn	2	30	4,28571	0	4,28571
0	0	0	jeden czyn	2	60	8,57143	0	8,57143
0	0	0	metabolicz	1	240	34,2857	17,1429	17,1429
0	1	1	trzy i więc	2	0	0	0	0
0	0	0	metabolicz	1	210	30	25,7143	4,28571
0	1	1	trzy i więc	2	0	0	0	0
0	1	1	trzy i więc	1	60	8,57143	0	8,57143
1	0	1	trzy i więc	2	0	0	0	0
0	0	0	dwa czynni	2	90	12,8571	0	12,8571
1	0	1	trzy i więc	2	0	0	0	0
0	0	1	jeden czyn	2	0	0	0	0
0	0	0	jeden czyn	2	0	0	0	0
0	0	0	jeden czyn	2	0	0	0	0
1	0	1	trzy i więc	2	420	60	0	60
0	0	0	jeden czyn	2	225	32,1429	17,1429	15
0	0	0	jeden czyn	2	300	42,8571	20	22,8571
1	0	1	trzy i więc	1	840	120	0	120

0	0	1	dwa czynni	1	420	60	0	60
0	1	1	trzy i więce	2	105	15	4,28571	10,7143
0	0	1	dwa czynni	2	0	0	0	0
0	0	0	metabolicz	2	0	0	0	0
1	1	1	trzy i więce	1	1080	154,286	120	34,2857
1	0	1	trzy i więce	2	160	22,8571	14,2857	8,57143
0	0	1	dwa czynni	2	1260	180	0	180
0	1	1	trzy i więce	3	0	0	0	0
1	0	1	trzy i więce	2	0	0	0	0
0	0	0	jeden czyn	3	240	34,2857	0	34,2857
0	0	1	dwa czynni	3	60	8,57143	0	8,57143
1	0	1	trzy i więce	2	0	0	0	0
0	1	0	dwa czynni	3	120	17,1429	0	17,1429
1	0	1	trzy i więce	1	420	60	0	60
0	0	0	jeden czyn	3	0	0	0	0
1	0	0	dwa czynni	2	135	19,2857	10,7143	8,57143
0	0	0	metabolicz	1	0	0	0	0
0	0	1	dwa czynni	2	240	34,2857	17,1429	17,1429
0	0	1	dwa czynni	1	140	20	0	20
0	0	0	jeden czyn	3	120	17,1429	0	17,1429
0	0	0	metabolicz	3	0	0	0	0
1	0	1	trzy i więce	3	0	0	0	0
0	0	0	metabolicz	1	120	17,1429	0	17,1429
0	0	0	jeden czyn	2	0	0	0	0
0	0	1	jeden czyn	2	0	0	0	0
1	1	1	trzy i więce	1	0	0	0	0
0	0	0	metabolicz	3	180	25,7143	17,1429	8,57143
0	0	1	jeden czyn	1	0	0	0	0
0	0	1	dwa czynni	2	180	25,7143	0	25,7143
0	1	1	trzy i więce	1	0	0	0	0
1	0	0	jeden czyn	1	0	0	0	0
0	1	0	jeden czyn	2	120	17,1429	0	17,1429
0	0	1	dwa czynni	2	0	0	0	0
0	0	0	jeden czyn	2	0	0	0	0
0	0	1	dwa czynni	1	0	0	0	0
1	1	1	trzy i więce	1	160	22,8571	0	22,8571
0	0	0	jeden czyn	1	0	0	0	0
0	0	1	jeden czyn	2	40	5,71429	0	5,71429
0	1	0	dwa czynni	3	480	68,5714	17,1429	51,4286
1	0	1	trzy i więce	3	0	0	0	0
0	0	0	metabolicz	2	670	95,7143	5,71429	90
0	0	0	jeden czyn	2	0	0	0	0
0	0	0	metabolicz	1	60	8,57143	4,28571	4,28571
0	0	0	jeden czyn	2	0	0	0	0
1	1	1	trzy i więce	3	0	0	0	0
0	0	1	dwa czynni	2	60	8,57143	0	8,57143
0	0	0	dwa czynni	2	0	0	0	0
0	0	0	metabolicz	1	420	60	0	60

0	0	1	jeden czyn	3	0	0	0	0
0	0	1	dwa czynni	3	180	25,7143	0	25,7143
0	0	1	dwa czynni	2	120	17,1429	0	17,1429
0	0	1	dwa czynni	3	240	34,2857	8,57143	25,7143
0	0	1	dwa czynni	2	90	12,8571	8,57143	4,28571
0	1	0	dwa czynni	3	180	25,7143	25,7143	0
0	0	1	dwa czynni	2	280	40	0	40
0	0	0	dwa czynni	2	390	55,7143	30	25,7143
0	0	1	dwa czynni	3	60	8,57143	0	8,57143
0	0	0	jeden czyn	3	0	0	0	0
0	0	1	dwa czynni	2	360	51,4286	51,4286	0
0	0	0	metabolicz	2	45	6,42857	6,42857	0
1	0	1	trzy i więc	2	1200	171,429	128,571	42,8571
0	0	1	dwa czynni	3	0	0	0	0
0	0	1	trzy i więc	2	120	17,1429	0	17,1429
0	0	1	jeden czyn	2	240	34,2857	0	34,2857
0	0	1	dwa czynni	3	0	0	0	0
0	0	1	dwa czynni	3	330	47,1429	17,1429	30
0	0	1	dwa czynni	1	660	94,2857	34,2857	60
0	0	1	trzy i więc	2	240	34,2857	0	34,2857
0	0	0	jeden czyn	2	120	17,1429	0	17,1429
1	0	0	trzy i więc	3	180	25,7143	0	25,7143
0	0	1	dwa czynni	3	105	15	15	0
1	0	1	trzy i więc	3	0	0	0	0
0	0	1	trzy i więc	3	0	0	0	0
1	0	1	trzy i więc	3	0	0	0	0
1	0	0	trzy i więc	1	350	50	0	50
0	0	1	jeden czyn	1	0	0	0	0
0	0	1	dwa czynni	2	180	25,7143	25,7143	0
0	0	0	jeden czyn	2	20	2,85714	0	2,85714
0	0	1	dwa czynni	1	225	32,1429	15	17,1429
0	0	0	metabolicz	2	0	0	0	0
1	0	0	dwa czynni	2	60	8,57143	0	8,57143
0	0	0	jeden czyn	2	360	51,4286	0	51,4286
0	1	0	dwa czynni	2	60	8,57143	0	8,57143
0	0	1	dwa czynni	2	30	4,28571	0	4,28571
0	1	0	dwa czynni	3	100	14,2857	0	14,2857
0	0	0	metabolicz	3	0	0	0	0
1	0	1	trzy i więc	2	120	17,1429	17,1429	0
0	0	1	dwa czynni	3	420	60	60	0
0	0	0	jeden czyn	1	180	25,7143	0	25,7143
0	0	1	dwa czynni	2	30	4,28571	4,28571	0
0	1	0	trzy i więc	2	60	8,57143	0	8,57143
0	0	1	dwa czynni	2	720	102,857	34,2857	68,5714
0	0	1	trzy i więc	1	140	20	20	0
0	0	0	metabolicz	2	0	0	0	0
0	0	1	dwa czynni	1	525	75	15	60
0	1	1	trzy i więc	1	120	17,1429	0	17,1429

0	0	0	jeden czyn	1	120	17,1429	0	17,1429
0	0	0	jeden czyn	1	0	0	0	0
0	0	0	metabolicz	2	45	6,42857	0	6,42857
1	0	0	dwa czynni	1	0	0	0	0
1	0	1	trzy i więc	3	120	17,1429	0	17,1429
0	0	1	dwa czynni	1	0	0	0	0
1	1	1	trzy i więc	1	180	25,7143	0	25,7143
0	0	0	jeden czyn	1	0	0	0	0
0	0	0	metabolicz	3	540	77,1429	34,2857	42,8571
0	0	0	jeden czyn	2	210	30	4,28571	25,7143
0	0	1	dwa czynni	2	100	14,2857	14,2857	0
1	0	0	trzy i więc	1	0	0	0	0
0	0	1	jeden czyn	2	0	0	0	0
1	0	1	trzy i więc	2	0	0	0	0
0	0	0	metabolicz	2	360	51,4286	25,7143	25,7143
0	0	1	jeden czyn	3	840	120	0	120
0	0	1	dwa czynni	2	0	0	0	0
0	0	1	dwa czynni	1	120	17,1429	0	17,1429
0	0	0	jeden czyn	3	0	0	0	0
0	0	0	metabolicz	3	180	25,7143	0	25,7143
0	0	1	dwa czynni	3	210	30	0	30
0	0	1	dwa czynni	2	0	0	0	0
0	0	1	jeden czyn	2	300	42,8571	17,1429	25,7143
0	0	1	dwa czynni	2	0	0	0	0
0	0	0	metabolicz	3	180	25,7143	25,7143	0
0	0	1	dwa czynni	2	0	0	0	0
0	0	1	dwa czynni	2	0	0	0	0
0	1	1	trzy i więc	1	120	17,1429	17,1429	0
0	1	0	dwa czynni	2	0	0	0	0
0	0	0	jeden czyn	1	0	0	0	0
0	0	1	dwa czynni	1	180	25,7143	8,57143	17,1429
1	1	1	trzy i więc	2	0	0	0	0
1	0	1	trzy i więc	2	240	34,2857	0	34,2857
0	0	1	dwa czynni	2	120	17,1429	0	17,1429
0	0	0	metabolicz	2	120	17,1429	17,1429	0
0	0	0	metabolicz	2	0	0	0	0
0	1	1	trzy i więc	2	0	0	0	0
0	1	1	trzy i więc	2	0	0	0	0
0	0	0	dwa czynni	2	300	42,8571	0	42,8571
0	0	1	jeden czyn	3	780	111,429	51,4286	60
1	0	0	trzy i więc	3	180	25,7143	0	25,7143
0	0	1	dwa czynni	2	0	0	0	0
0	0	0	metabolicz	2	30	4,28571	0	4,28571
0	0	0	metabolicz	3	0	0	0	0
0	0	0	metabolicz	1	0	0	0	0
0	0	0	jeden czyn	1	0	0	0	0
1	1	1	trzy i więc	3	0	0	0	0
1	0	1	trzy i więc	1	840	120	0	120

0	0	1	dwa czynni	2	0	0	0	0
1	1	1	trzy i więce	2	0	0	0	0
1	0	0	dwa czynni	1	120	17,1429	0	17,1429
0	0	0	jeden czyn	1	60	8,57143	8,57143	0
0	0	1	dwa czynni	3	90	12,8571	0	12,8571
0	0	1	dwa czynni	2	150	21,4286	0	21,4286
1	1	1	trzy i więce	2	180	25,7143	25,7143	0
1	1	1	trzy i więce	1	420	60	0	60
1	0	1	trzy i więce	2	0	0	0	0
0	0	0	jeden czyn	3	0	0	0	0
0	0	0	metabolicz	2	300	42,8571	0	42,8571
0	1	1	trzy i więce	2	150	21,4286	0	21,4286
0	1	1	trzy i więce	2	120	17,1429	8,57143	8,57143
1	0	0	trzy i więce	2	0	0	0	0
0	0	1	trzy i więce	1	0	0	0	0
0	0	0	metabolicz	2	0	0	0	0
0	0	1	jeden czyn	2	0	0	0	0
0	1	1	trzy i więce	3	0	0	0	0
0	0	0	jeden czyn	2	60	8,57143	0	8,57143
0	1	0	trzy i więce	1	0	0	0	0
1	0	0	trzy i więce	2	200	28,5714	0	28,5714
0	0	1	dwa czynni	2	390	55,7143	51,4286	4,28571
0	0	1	jeden czyn	3	180	25,7143	0	25,7143
0	0	0	metabolicz	2	0	0	0	0
1	0	0	trzy i więce	1	0	0	0	0
0	1	1	trzy i więce	2	120	17,1429	0	17,1429
0	1	1	trzy i więce	2	900	128,571	8,57143	120
0	0	1	trzy i więce	2	0	0	0	0
0	0	0	metabolicz	1	0	0	0	0
0	0	0	metabolicz	2	210	30	0	30
0	0	0	metabolicz	3	60	8,57143	0	8,57143
0	0	0	jeden czyn	2	90	12,8571	0	12,8571
0	0	0	dwa czynni	2	80	11,4286	0	11,4286
0	0	1	dwa czynni	1	120	17,1429	0	17,1429
0	0	0	jeden czyn	2	120	17,1429	17,1429	0
0	0	0	jeden czyn	3	0	0	0	0
0	0	1	dwa czynni	2	360	51,4286	34,2857	17,1429
0	0	0	metabolicz	1	240	34,2857	0	34,2857
1	0	1	trzy i więce	2	30	4,28571	0	4,28571
0	1	1	trzy i więce	3	100	14,2857	0	14,2857
0	0	0	dwa czynni	2	0	0	0	0
0	0	0	metabolicz	1	0	0	0	0
0	0	0	metabolicz	1	180	25,7143	17,1429	8,57143
0	0	1	dwa czynni	3	0	0	0	0
0	0	0	jeden czyn	3	210	30	0	30
0	0	1	jeden czyn	2	120	17,1429	0	17,1429
0	0	0	metabolicz	2	240	34,2857	0	34,2857
0	0	1	dwa czynni	3	0	0	0	0

0	1	1	dwa czynni	2	375	53,5714	45	8,57143
0	1	0	dwa czynni	1	0	0	0	0
0	0	0	metabolicz	2	465	66,4286	6,42857	60
1	0	1	dwa czynni	1	200	28,5714	11,4286	17,1429
0	0	0	metabolicz	1	1260	180	0	180
0	0	0	metabolicz	3	180	25,7143	17,1429	8,57143
1	0	1	trzy i więc	2	150	21,4286	4,28571	17,1429
0	1	1	trzy i więc	1	0	0	0	0
0	0	0	metabolicz	3	420	60	0	60
0	0	1	dwa czynni	3	90	12,8571	0	12,8571
0	0	0	jeden czyn	2	180	25,7143	0	25,7143
0	0	0	jeden czyn	2	400	57,1429	22,8571	34,2857
1	0	0	dwa czynni	2	240	34,2857	0	34,2857
0	0	1	dwa czynni	3	0	0	0	0
1	0	1	trzy i więc	1	120	17,1429	0	17,1429
0	0	1	dwa czynni	2	0	0	0	0
0	0	1	dwa czynni	2	40	5,71429	0	5,71429
0	0	1	dwa czynni	2	0	0	0	0
0	1	0	dwa czynni	2	210	30	30	0
0	0	0	dwa czynni	2	60	8,57143	8,57143	0
0	0	1	jeden czyn	3	0	0	0	0
0	0	0	jeden czyn	3	0	0	0	0
1	0	1	trzy i więc	2	60	8,57143	0	8,57143
1	0	1	trzy i więc	2	180	25,7143	8,57143	17,1429
0	0	1	jeden czyn	2	195	27,8571	2,14286	25,7143
0	0	1	dwa czynni	2	180	25,7143	0	25,7143
0	1	1	trzy i więc	3	0	0	0	0
0	0	1	dwa czynni	2	140	20	0	20
0	0	0	metabolicz	2	120	17,1429	0	17,1429
0	0	0	metabolicz	3	90	12,8571	0	12,8571
0	0	0	metabolicz	2	120	17,1429	17,1429	0
0	0	0	jeden czyn	2	0	0	0	0
1	0	1	trzy i więc	1	360	51,4286	0	51,4286
0	0	1	trzy i więc	3	180	25,7143	8,57143	17,1429
0	0	0	metabolicz	2	0	0	0	0
1	0	1	trzy i więc	1	0	0	0	0
0	0	0	jeden czyn	2	150	21,4286	0	21,4286
0	0	0	metabolicz	3	1140	162,857	128,571	34,2857
0	0	1	dwa czynni	2	280	40	0	40
0	1	1	trzy i więc	1	330	47,1429	17,1429	30
0	0	1	dwa czynni	2	40	5,71429	5,71429	0
1	0	0	dwa czynni	2	0	0	0	0
0	0	1	jeden czyn	2	0	0	0	0
0	0	0	metabolicz	2	280	40	22,8571	17,1429
0	0	0	jeden czyn	2	60	8,57143	0	8,57143
0	0	1	trzy i więc	2	0	0	0	0
0	0	1	dwa czynni	2	160	22,8571	5,71429	17,1429
0	0	0	jeden czyn	2	90	12,8571	0	12,8571

0	0	1	dwa czynni	1	120	17,1429	0	17,1429
0	0	0	metabolicz	2	0	0	0	0
1	1	1	trzy i więce	2	210	30	30	0
0	0	1	jeden czyn	3	480	68,5714	8,57143	60
0	0	0	jeden czyn	2	0	0	0	0
0	0	0	jeden czyn	2	840	120	0	120
0	0	1	dwa czynni	1	0	0	0	0
0	0	1	jeden czyn	2	300	42,8571	0	42,8571
0	0	0	metabolicz	2	535	76,4286	25	51,4286
0	0	0	jeden czyn	3	0	0	0	0
0	0	0	jeden czyn	2	60	8,57143	0	8,57143
0	0	0	dwa czynni	2	0	0	0	0
0	1	1	trzy i więce	2	0	0	0	0
0	0	0	jeden czyn	3	300	42,8571	0	42,8571
0	0	1	jeden czyn	2	30	4,28571	4,28571	0
0	0	1	dwa czynni	2	180	25,7143	0	25,7143
1	0	0	dwa czynni	3	0	0	0	0
0	0	0	jeden czyn	1	0	0	0	0
1	1	1	trzy i więce	3	0	0	0	0
1	0	1	trzy i więce	2	195	27,8571	13,5714	14,2857
0	0	1	dwa czynni	2	180	25,7143	0	25,7143
1	0	0	trzy i więce	1	40	5,71429	0	5,71429
0	0	0	metabolicz	3	0	0	0	0
0	0	1	dwa czynni	3	30	4,28571	0	4,28571
0	0	0	jeden czyn	2	0	0	0	0
0	0	1	jeden czyn	3	240	34,2857	17,1429	17,1429
0	0	0	jeden czyn	2	280	40	10	30
0	0	0	metabolicz	1	60	8,57143	8,57143	0
0	0	1	dwa czynni	2	120	17,1429	17,1429	0
0	0	1	jeden czyn	3	90	12,8571	4,28571	8,57143
0	0	0	metabolicz	2	60	8,57143	0	8,57143
0	1	0	jeden czyn	1	180	25,7143	17,1429	8,57143
0	0	1	dwa czynni	3	180	25,7143	0	25,7143
0	1	1	trzy i więce	1	0	0	0	0
0	1	0	trzy i więce	2	240	34,2857	0	34,2857
0	0	0	metabolicz	2	0	0	0	0
0	0	0	jeden czyn	3	0	0	0	0
1	1	1	trzy i więce	2	60	8,57143	0	8,57143
0	0	1	jeden czyn	2	0	0	0	0
0	0	1	jeden czyn	3	240	34,2857	8,57143	25,7143
1	1	1	trzy i więce	3	420	60	60	0
1	0	1	trzy i więce	2	0	0	0	0
0	1	0	jeden czyn	2	240	34,2857	34,2857	0
0	0	0	jeden czyn	2	140	20	7,14286	12,8571
1	0	1	trzy i więce	1	0	0	0	0
0	0	0	metabolicz	1	360	51,4286	0	51,4286
0	0	1	jeden czyn	1	0	0	0	0
0	0	0	metabolicz	2	60	8,57143	0	8,57143

1	0	1	trzy i więc	1	0	0	0	0
0	0	0	jeden czyn	3	105	15	0	15
0	0	1	dwa czynni	3	360	51,4286	0	51,4286
0	0	1	trzy i więc	1	0	0	0	0
0	0	0	metabolicz	3	60	8,57143	0	8,57143
1	1	0	trzy i więc	3	0	0	0	0
0	0	1	dwa czynni	2	360	51,4286	34,2857	17,1429
0	0	1	trzy i więc	3	0	0	0	0
0	0	1	dwa czynni	2	360	51,4286	0	51,4286
0	0	0	jeden czyn	2	120	17,1429	17,1429	0
0	0	0	metabolicz	2	45	6,42857	6,42857	0
0	0	1	dwa czynni	1	240	34,2857	0	34,2857
0	0	1	trzy i więc	1	0	0	0	0
0	0	0	metabolicz	2	840	120	0	120
0	0	0	metabolicz	2	0	0	0	0
0	0	1	dwa czynni	2	600	85,7143	34,2857	51,4286
0	1	0	dwa czynni	3	60	8,57143	0	8,57143
0	0	1	dwa czynni	2	0	0	0	0
1	1	1	trzy i więc	2	0	0	0	0
0	0	0	metabolicz	2	60	8,57143	0	8,57143
0	1	1	trzy i więc	2	0	0	0	0
0	0	1	trzy i więc	2	240	34,2857	0	34,2857
0	0	0	jeden czyn	2	90	12,8571	0	12,8571
1	0	0	dwa czynni	3	0	0	0	0
0	1	1	dwa czynni	3	0	0	0	0
1	1	1	trzy i więc	3	0	0	0	0
0	0	1	dwa czynni	2	360	51,4286	0	51,4286
0	0	0	jeden czyn	2	0	0	0	0
0	0	0	jeden czyn	2	450	64,2857	51,4286	12,8571
0	0	1	jeden czyn	2	160	22,8571	17,1429	5,71429
1	0	1	dwa czynni	2	0	0	0	0
0	0	1	dwa czynni	2	0	0	0	0
0	0	0	jeden czyn	2	150	21,4286	12,8571	8,57143
0	0	0	metabolicz	3	840	120	0	120
0	0	0	jeden czyn	2	300	42,8571	0	42,8571
0	0	1	jeden czyn	2	540	77,1429	17,1429	60
0	0	0	jeden czyn	2	0	0	0	0
0	1	1	trzy i więc	2	120	17,1429	0	17,1429
0	0	1	dwa czynni	2	180	25,7143	8,57143	17,1429
0	0	1	trzy i więc	2	0	0	0	0
1	0	1	trzy i więc	2	180	25,7143	0	25,7143
0	0	1	dwa czynni	2	240	34,2857	0	34,2857
0	0	1	dwa czynni	3	420	60	0	60
0	0	1	dwa czynni	3	180	25,7143	17,1429	8,57143
0	1	1	trzy i więc	1	0	0	0	0
0	0	1	dwa czynni	2	120	17,1429	0	17,1429
0	0	0	metabolicz	3	0	0	0	0
0	0	1	jeden czyn	3	0	0	0	0

0	0	1	trzy i więc	2	0	0	0	0
0	0	1	dwa czynni	2	0	0	0	0
0	0	1	jeden czyn	2	0	0	0	0
0	0	0	jeden czyn	2	0	0	0	0
0	1	1	trzy i więc	2	0	0	0	0
1	0	1	trzy i więc	2	150	21,4286	0	21,4286
0	1	1	trzy i więc	3	660	94,2857	25,7143	68,5714
0	0	0	jeden czyn	2	0	0	0	0
0	0	0	metabolicz	2	540	77,1429	0	77,1429
0	0	1	dwa czynni	1	600	85,7143	60	25,7143
0	0	1	dwa czynni	2	150	21,4286	21,4286	0
1	0	1	trzy i więc	2	0	0	0	0
0	1	0	dwa czynni	3	420	60	51,4286	8,57143
0	0	1	dwa czynni	1	0	0	0	0
0	1	0	dwa czynni	1	225	32,1429	15	17,1429
0	0	1	dwa czynni	2	0	0	0	0
0	0	1	dwa czynni	2	0	0	0	0
0	0	1	trzy i więc	3	420	60	0	60
1	0	1	trzy i więc	1	180	25,7143	8,57143	17,1429
0	0	0	jeden czyn	2	30	4,28571	0	4,28571
0	0	1	jeden czyn	3	360	51,4286	17,1429	34,2857
0	0	0	metabolicz	2	30	4,28571	4,28571	0
1	0	1	trzy i więc	1	60	8,57143	0	8,57143
0	0	1	dwa czynni	2	225	32,1429	15	17,1429
0	0	0	jeden czyn	2	0	0	0	0
0	0	0	jeden czyn	2	0	0	0	0
0	0	1	dwa czynni	2	120	17,1429	0	17,1429
0	0	1	dwa czynni	2	60	8,57143	0	8,57143
1	1	1	trzy i więc	2	0	0	0	0
0	0	0	jeden czyn	3	0	0	0	0
0	1	0	trzy i więc	2	0	0	0	0
0	0	0	metabolicz	3	840	120	120	0
1	0	1	trzy i więc	3	840	120	0	120
0	0	1	dwa czynni	2	30	4,28571	0	4,28571
0	1	1	trzy i więc	3	30	4,28571	4,28571	0
0	0	0	jeden czyn	3	120	17,1429	0	17,1429
0	0	0	jeden czyn	2	240	34,2857	34,2857	0
0	0	1	dwa czynni	3	0	0	0	0
0	0	0	metabolicz	1	0	0	0	0
0	0	0	metabolicz	3	0	0	0	0
0	0	1	jeden czyn	2	30	4,28571	0	4,28571
0	0	1	trzy i więc	1	0	0	0	0
0	0	1	dwa czynni	2	105	15	15	0
0	0	1	jeden czyn	2	60	8,57143	0	8,57143
0	0	1	dwa czynni	2	120	17,1429	0	17,1429
0	0	1	jeden czyn	2	0	0	0	0
0	0	1	jeden czyn	2	120	17,1429	0	17,1429
0	0	1	dwa czynni	3	525	75	60	15

0	0	0	jeden czyn	3	60	8,57143	0	8,57143
1	1	1	trzy i więce	1	420	60	0	60
1	0	1	trzy i więce	2	0	0	0	0
0	0	0	jeden czyn	2	0	0	0	0
0	1	1	trzy i więce	2	120	17,1429	0	17,1429
0	0	1	dwa czynni	2	0	0	0	0
1	0	1	trzy i więce	2	210	30	30	0
0	0	1	dwa czynni	2	0	0	0	0
1	1	1	trzy i więce	2	210	30	0	30
1	0	1	trzy i więce	2	0	0	0	0
0	0	1	dwa czynni	3	60	8,57143	0	8,57143
0	0	1	dwa czynni	3	80	11,4286	2,85714	8,57143
0	0	1	dwa czynni	3	0	0	0	0
0	0	1	dwa czynni	2	120	17,1429	0	17,1429
1	1	1	trzy i więce	2	60	8,57143	0	8,57143
1	1	0	trzy i więce	3	0	0	0	0
0	0	1	dwa czynni	3	0	0	0	0
1	1	1	trzy i więce	2	0	0	0	0
0	0	1	dwa czynni	3	420	60	0	60
1	0	0	jeden czyn	2	0	0	0	0
1	0	0	dwa czynni	2	60	8,57143	0	8,57143
0	1	1	trzy i więce	1	0	0	0	0
0	0	0	jeden czyn	3	0	0	0	0
0	1	1	trzy i więce	1	60	8,57143	8,57143	0
0	0	0	dwa czynni	1	210	30	0	30
0	0	0	metabolicz	3	0	0	0	0
0	0	1	dwa czynni	2	0	0	0	0
0	0	1	dwa czynni	2	0	0	0	0
1	0	0	jeden czyn	2	180	25,7143	25,7143	0
0	0	0	metabolicz	2	120	17,1429	17,1429	0
0	0	0	metabolicz	3	45	6,42857	6,42857	0
0	0	1	dwa czynni	2	60	8,57143	0	8,57143
1	1	1	trzy i więce	2	0	0	0	0
0	0	0	metabolicz	2	390	55,7143	25,7143	30
0	0	1	dwa czynni	2	60	8,57143	0	8,57143
0	0	0	jeden czyn	3	180	25,7143	0	25,7143
0	0	0	jeden czyn	3	0	0	0	0
1	1	1	trzy i więce	2	0	0	0	0
0	0	1	jeden czyn	2	480	68,5714	60	8,57143
0	0	0	jeden czyn	3	450	64,2857	21,4286	42,8571
1	0	1	trzy i więce	2	0	0	0	0
1	0	0	dwa czynni	1	0	0	0	0
0	0	0	jeden czyn	2	0	0	0	0
1	1	1	trzy i więce	1	200	28,5714	0	28,5714
0	0	1	jeden czyn	2	60	8,57143	8,57143	0
1	1	1	trzy i więce	2	0	0	0	0
0	0	1	dwa czynni	2	0	0	0	0
0	1	1	trzy i więce	2	120	17,1429	0	17,1429

1	0	1	trzy i więce	2	0	0	0	0
0	0	1	dwa czynni	3	0	0	0	0
1	0	0	dwa czynni	1	0	0	0	0
1	0	1	trzy i więce	3	1080	154,286	0	154,286
1	1	1	trzy i więce	3	30	4,28571	4,28571	0
0	0	0	jeden czyn	2	0	0	0	0
0	0	1	dwa czynni	1	0	0	0	0
0	0	1	dwa czynni	2	210	30	12,8571	17,1429
0	1	0	dwa czynni	2	0	0	0	0
0	0	0	jeden czyn	2	40	5,71429	0	5,71429
0	0	1	jeden czyn	3	60	8,57143	0	8,57143
0	0	1	dwa czynni	2	120	17,1429	0	17,1429
0	0	0	metabolicz	2	0	0	0	0
0	0	0	metabolicz	1	240	34,2857	34,2857	0
0	0	1	dwa czynni	2	0	0	0	0
0	0	0	jeden czyn	3	0	0	0	0
0	0	0	metabolicz	1	420	60	0	60
0	0	1	dwa czynni	1	0	0	0	0
0	0	1	dwa czynni	2	0	0	0	0
0	0	1	dwa czynni	3	60	8,57143	0	8,57143
0	1	1	trzy i więce	2	90	12,8571	0	12,8571
0	0	1	jeden czyn	3	720	102,857	85,7143	17,1429
1	1	1	trzy i więce	2	525	75	30	45
0	0	1	dwa czynni	3	420	60	34,2857	25,7143
0	0	0	metabolicz	3	300	42,8571	17,1429	25,7143
0	1	1	trzy i więce	2	0	0	0	0
0	0	0	jeden czyn	3	280	40	0	40
0	0	1	dwa czynni	3	900	128,571	8,57143	120
0	0	0	jeden czyn	2	0	0	0	0
1	1	0	trzy i więce	1	0	0	0	0
0	0	0	metabolicz	2	270	38,5714	12,8571	25,7143
0	0	1	dwa czynni	2	30	4,28571	4,28571	0
0	0	0	metabolicz	2	0	0	0	0
1	0	1	trzy i więce	2	0	0	0	0
0	0	0	metabolicz	3	840	120	120	0
0	0	0	jeden czyn	2	360	51,4286	0	51,4286
1	0	1	trzy i więce	1	60	8,57143	0	8,57143
1	0	1	trzy i więce	2	240	34,2857	34,2857	0
0	0	0	jeden czyn	1	0	0	0	0
0	0	1	jeden czyn	2	60	8,57143	0	8,57143
0	0	1	jeden czyn	1	0	0	0	0
0	0	1	jeden czyn	3	0	0	0	0
0	0	1	jeden czyn	2	280	40	10	30
0	0	0	metabolicz	2	180	25,7143	0	25,7143
0	1	1	trzy i więce	3	0	0	0	0
1	0	0	jeden czyn	2	120	17,1429	0	17,1429
0	0	0	metabolicz	2	75	10,7143	0	10,7143
0	1	1	trzy i więce	2	385	55	15,7143	39,2857

0	0	1	dwa czynni	2	60	8,57143	0	8,57143
0	0	1	jeden czyn	2	120	17,1429	0	17,1429
0	0	0	jeden czyn	2	300	42,8571	0	42,8571
0	1	1	trzy i więce	2	90	12,8571	0	12,8571
0	0	0	jeden czyn	2	0	0	0	0
0	1	1	trzy i więce	3	240	34,2857	0	34,2857
0	1	0	jeden czyn	3	300	42,8571	0	42,8571
1	1	1	trzy i więce	1	0	0	0	0
0	0	1	dwa czynni	2	100	14,2857	0	14,2857
0	0	0	jeden czyn	2	60	8,57143	8,57143	0
0	0	0	metabolicz	1	120	17,1429	17,1429	0
0	0	1	dwa czynni	2	0	0	0	0
1	1	1	trzy i więce	3	0	0	0	0
0	0	0	jeden czyn	3	330	47,1429	4,28571	42,8571
0	1	0	dwa czynni	2	110	15,7143	2,85714	12,8571
0	1	1	trzy i więce	2	180	25,7143	25,7143	0
0	0	1	dwa czynni	2	120	17,1429	17,1429	0
1	0	1	trzy i więce	2	0	0	0	0
0	0	0	metabolicz	3	0	0	0	0
0	1	1	trzy i więce	3	30	4,28571	0	4,28571
0	0	1	dwa czynni	3	150	21,4286	0	21,4286
0	0	1	dwa czynni	2	180	25,7143	0	25,7143
0	0	0	metabolicz	2	90	12,8571	0	12,8571
0	0	0	jeden czyn	2	180	25,7143	0	25,7143
0	0	0	metabolicz	2	0	0	0	0
0	0	0	dwa czynni	2	30	4,28571	4,28571	0
1	0	1	trzy i więce	1	120	17,1429	0	17,1429
0	0	1	dwa czynni	1	60	8,57143	0	8,57143
1	0	1	trzy i więce	1	60	8,57143	0	8,57143
0	0	0	metabolicz	2	40	5,71429	0	5,71429
0	1	1	trzy i więce	2	210	30	12,8571	17,1429
0	0	1	trzy i więce	3	0	0	0	0
0	0	0	metabolicz	2	630	90	0	90
0	0	1	jeden czyn	2	240	34,2857	0	34,2857
0	0	0	jeden czyn	2	110	15,7143	7,14286	8,57143
0	0	1	jeden czyn	1	0	0	0	0
0	0	0	jeden czyn	2	0	0	0	0
0	0	0	metabolicz	1	0	0	0	0
0	0	1	dwa czynni	2	0	0	0	0
0	0	0	jeden czyn	1	540	77,1429	25,7143	51,4286
0	0	0	metabolicz	2	140	20	20	0
0	1	1	trzy i więce	1	60	8,57143	8,57143	0
0	0	0	jeden czyn	1	60	8,57143	0	8,57143
1	0	1	trzy i więce	2	0	0	0	0
0	0	0	jeden czyn	2	40	5,71429	0	5,71429
0	0	0	metabolicz	2	245	35	0	35
0	1	1	trzy i więce	2	0	0	0	0
0	0	1	dwa czynni	3	0	0	0	0

0	0	0	metabolicz	2	0	0	0	0
0	0	0	jeden czyn	2	90	12,8571	0	12,8571
0	0	0	metabolicz	2	0	0	0	0
0	0	1	dwa czynni	3	360	51,4286	0	51,4286
0	0	0	jeden czyn	2	225	32,1429	15	17,1429
0	0	0	metabolicz	1	120	17,1429	0	17,1429
0	0	1	jeden czyn	1	0	0	0	0
1	0	0	dwa czynni	1	0	0	0	0
0	0	0	metabolicz	3	70	10	0	10
0	0	1	trzy i więc	3	60	8,57143	8,57143	0
0	0	0	metabolicz	1	210	30	0	30
0	0	1	jeden czyn	2	120	17,1429	17,1429	0
0	0	1	jeden czyn	2	300	42,8571	0	42,8571
0	0	1	dwa czynni	2	0	0	0	0
1	0	1	trzy i więc	2	110	15,7143	11,4286	4,28571
1	0	0	dwa czynni	1	50	7,14286	7,14286	0
0	0	0	metabolicz	1	0	0	0	0
0	0	0	jeden czyn	2	60	8,57143	8,57143	0
1	0	1	trzy i więc	1	0	0	0	0
0	0	0	jeden czyn	2	60	8,57143	0	8,57143
0	0	0	metabolicz	2	60	8,57143	0	8,57143
0	0	0	metabolicz	3	90	12,8571	4,28571	8,57143
1	0	1	trzy i więc	3	120	17,1429	0	17,1429
0	0	1	jeden czyn	2	0	0	0	0
0	0	1	dwa czynni	3	60	8,57143	0	8,57143
0	1	1	dwa czynni	2	255	36,4286	19,2857	17,1429
0	0	0	jeden czyn	2	180	25,7143	0	25,7143
0	1	0	dwa czynni	1	60	8,57143	0	8,57143
0	0	1	jeden czyn	3	60	8,57143	8,57143	0
1	0	1	trzy i więc	3	300	42,8571	0	42,8571
0	0	1	jeden czyn	2	120	17,1429	0	17,1429
0	0	1	jeden czyn	2	0	0	0	0
0	1	1	trzy i więc	3	0	0	0	0
0	0	1	dwa czynni	1	210	30	30	0
1	0	0	dwa czynni	2	0	0	0	0
0	0	0	metabolicz	2	30	4,28571	0	4,28571
0	0	0	metabolicz	3	345	49,2857	49,2857	0
0	0	0	metabolicz	2	180	25,7143	0	25,7143
0	0	0	metabolicz	2	45	6,42857	6,42857	0
0	0	1	jeden czyn	2	60	8,57143	0	8,57143
0	0	0	jeden czyn	1	0	0	0	0
1	1	1	trzy i więc	2	0	0	0	0
0	0	0	metabolicz	1	120	17,1429	0	17,1429
0	0	0	metabolicz	2	0	0	0	0
0	0	0	jeden czyn	3	360	51,4286	51,4286	0
0	0	1	dwa czynni	1	165	23,5714	10,7143	12,8571
0	0	0	jeden czyn	2	0	0	0	0
0	0	0	metabolicz	2	300	42,8571	8,57143	34,2857

0	0	1	dwa czynni	1	120	17,1429	0	17,1429
1	0	0	dwa czynni	3	180	25,7143	8,57143	17,1429
0	0	0	metabolicz	2	0	0	0	0
1	0	1	trzy i więce	2	0	0	0	0
1	0	1	trzy i więce	2	0	0	0	0
0	0	1	jeden czyn	2	90	12,8571	12,8571	0
1	0	1	trzy i więce	2	60	8,57143	0	8,57143
0	0	0	metabolicz	2	210	30	0	30
0	0	1	dwa czynni	3	270	38,5714	8,57143	30
0	0	1	jeden czyn	3	180	25,7143	0	25,7143
1	1	1	trzy i więce	2	0	0	0	0
0	0	0	jeden czyn	2	60	8,57143	0	8,57143
0	0	1	dwa czynni	1	360	51,4286	0	51,4286
0	1	1	dwa czynni	1	0	0	0	0
0	0	0	jeden czyn	2	360	51,4286	47,1429	4,28571
1	0	0	dwa czynni	2	240	34,2857	0	34,2857
0	0	1	jeden czyn	1	560	80	20	60
0	0	1	jeden czyn	1	0	0	0	0
1	0	0	jeden czyn	2	60	8,57143	0	8,57143
0	0	0	metabolicz	2	210	30	30	0
1	1	1	trzy i więce	3	220	31,4286	14,2857	17,1429
0	0	1	dwa czynni	2	30	4,28571	0	4,28571
0	1	0	trzy i więce	1	0	0	0	0
1	0	1	trzy i więce	1	210	30	0	30
0	0	0	metabolicz	2	180	25,7143	0	25,7143
0	0	1	trzy i więce	3	0	0	0	0
0	0	1	dwa czynni	3	0	0	0	0
0	0	0	jeden czyn	3	0	0	0	0
0	0	0	jeden czyn	2	0	0	0	0
0	0	1	dwa czynni	2	570	81,4286	21,4286	60
0	0	1	jeden czyn	2	0	0	0	0
0	0	0	metabolicz	1	0	0	0	0
0	0	1	dwa czynni	2	120	17,1429	0	17,1429
0	0	0	metabolicz	2	180	25,7143	0	25,7143
1	0	0	trzy i więce	2	0	0	0	0
0	1	1	dwa czynni	2	40	5,71429	5,71429	0
0	0	0	jeden czyn	2	60	8,57143	0	8,57143
0	0	1	dwa czynni	2	30	4,28571	0	4,28571
0	1	1	dwa czynni	2	0	0	0	0
0	0	0	metabolicz	3	20	2,85714	2,85714	0
0	0	0	jeden czyn	1	0	0	0	0
0	0	0	metabolicz	2	60	8,57143	0	8,57143
0	0	1	dwa czynni	3	720	102,857	0	102,857
1	1	1	trzy i więce	2	0	0	0	0
0	0	0	jeden czyn	2	240	34,2857	0	34,2857
1	0	0	jeden czyn	1	0	0	0	0
0	0	0	jeden czyn	2	180	25,7143	0	25,7143
0	0	0	jeden czyn	3	180	25,7143	8,57143	17,1429

0	0	1	dwa czynni	2	180	25,7143	25,7143	0
0	0	0	metabolicz	2	0	0	0	0
0	0	0	metabolicz	2	60	8,57143	8,57143	0
0	0	1	dwa czynni	2	210	30	12,8571	17,1429
0	0	0	metabolicz	3	0	0	0	0
0	1	1	dwa czynni	1	405	57,8571	6,42857	51,4286
0	0	0	metabolicz	2	60	8,57143	0	8,57143
0	0	1	jeden czyn	3	330	47,1429	12,8571	34,2857
0	0	0	metabolicz	2	120	17,1429	17,1429	0
0	0	1	dwa czynni	1	240	34,2857	34,2857	0
0	0	0	metabolicz	2	600	85,7143	17,1429	68,5714
0	1	1	trzy i więc	2	0	0	0	0
1	0	1	trzy i więc	2	0	0	0	0
0	0	1	jeden czyn	2	180	25,7143	17,1429	8,57143
0	0	0	jeden czyn	1	90	12,8571	12,8571	0
0	0	1	dwa czynni	3	120	17,1429	8,57143	8,57143
0	1	0	dwa czynni	2	0	0	0	0
0	0	1	jeden czyn	2	90	12,8571	0	12,8571
0	0	1	jeden czyn	3	180	25,7143	0	25,7143
0	0	0	metabolicz	2	0	0	0	0
1	0	1	trzy i więc	2	0	0	0	0
0	0	1	trzy i więc	2	0	0	0	0
1	1	1	trzy i więc	2	840	120	120	0
1	0	0	jeden czyn	2	0	0	0	0
1	0	0	dwa czynni	2	330	47,1429	30	17,1429
0	1	1	trzy i więc	3	0	0	0	0
0	0	1	jeden czyn	2	0	0	0	0
0	1	1	trzy i więc	2	120	17,1429	0	17,1429
0	0	1	dwa czynni	1	0	0	0	0
1	1	1	trzy i więc	1	0	0	0	0
1	0	1	trzy i więc	2	0	0	0	0
0	0	1	dwa czynni	3	120	17,1429	17,1429	0
0	0	0	metabolicz	2	240	34,2857	0	34,2857
0	0	1	dwa czynni	2	90	12,8571	12,8571	0
0	0	1	jeden czyn	1	0	0	0	0
0	0	0	metabolicz	2	180	25,7143	0	25,7143
1	1	1	trzy i więc	3	0	0	0	0
1	0	0	dwa czynni	3	0	0	0	0
0	0	0	jeden czyn	1	0	0	0	0
0	1	1	trzy i więc	2	150	21,4286	0	21,4286
0	0	1	dwa czynni	2	0	0	0	0
0	0	0	jeden czyn	3	0	0	0	0
1	0	1	trzy i więc	2	480	68,5714	8,57143	60
1	0	1	trzy i więc	1	0	0	0	0
0	0	1	jeden czyn	2	90	12,8571	0	12,8571
0	0	0	metabolicz	2	75	10,7143	6,42857	4,28571
0	0	1	dwa czynni	2	60	8,57143	0	8,57143
0	0	1	jeden czyn	2	60	8,57143	0	8,57143

0	0	1	dwa czynni	2	840	120	0	120
0	0	1	dwa czynni	2	120	17,1429	0	17,1429
0	0	1	dwa czynni	2	120	17,1429	0	17,1429
0	0	1	dwa czynni	2	0	0	0	0
0	0	0	metabolicz	1	0	0	0	0
0	0	0	jeden czyn	2	0	0	0	0
0	1	0	dwa czynni	2	90	12,8571	12,8571	0
0	0	0	metabolicz	3	0	0	0	0
0	0	1	jeden czyn	2	420	60	0	60
0	0	0	jeden czyn	2	0	0	0	0
0	0	0	metabolicz	3	0	0	0	0
0	0	0	jeden czyn	2	60	8,57143	0	8,57143
1	0	0	dwa czynni	2	0	0	0	0
0	1	1	dwa czynni	2	105	15	6,42857	8,57143
1	0	1	trzy i więc	1	0	0	0	0
0	0	0	jeden czyn	2	0	0	0	0
0	0	0	jeden czyn	2	120	17,1429	0	17,1429
0	0	0	jeden czyn	3	240	34,2857	0	34,2857
0	0	1	dwa czynni	2	390	55,7143	30	25,7143
0	0	0	jeden czyn	3	30	4,28571	0	4,28571
0	0	0	jeden czyn	1	0	0	0	0
0	0	0	metabolicz	3	480	68,5714	34,2857	34,2857
0	0	1	dwa czynni	2	480	68,5714	38,5714	30
0	0	1	jeden czyn	3	420	60	0	60
0	0	1	jeden czyn	2	0	0	0	0
0	0	0	jeden czyn	1	420	60	0	60
0	0	1	jeden czyn	2	480	68,5714	0	68,5714
0	0	1	jeden czyn	1	180	25,7143	17,1429	8,57143
0	0	0	jeden czyn	2	30	4,28571	0	4,28571
1	1	1	trzy i więc	2	0	0	0	0
0	0	1	jeden czyn	2	0	0	0	0
1	0	1	trzy i więc	1	0	0	0	0
0	0	1	jeden czyn	2	30	4,28571	0	4,28571
0	0	0	jeden czyn	2	60	8,57143	0	8,57143
0	1	0	jeden czyn	2	210	30	8,57143	21,4286
0	0	1	dwa czynni	2	210	30	0	30
0	0	1	dwa czynni	2	0	0	0	0
0	0	0	dwa czynni	3	0	0	0	0
0	0	1	dwa czynni	2	0	0	0	0
0	0	0	metabolicz	2	0	0	0	0
0	0	0	metabolicz	2	0	0	0	0
1	1	1	trzy i więc	3	360	51,4286	0	51,4286
0	0	1	dwa czynni	1	0	0	0	0
0	1	0	dwa czynni	3	0	0	0	0
0	0	1	jeden czyn	2	840	120	0	120
0	0	0	metabolicz	3	0	0	0	0
0	0	1	jeden czyn	2	0	0	0	0
0	0	1	jeden czyn	2	105	15	15	0

1	1	0	trzy i więce	2	15	2,14286	0	2,14286
0	0	0	metabolicz	2	0	0	0	0
0	0	1	dwa czynni	2	0	0	0	0
1	0	1	trzy i więce	1	30	4,28571	0	4,28571
0	0	1	dwa czynni	2	60	8,57143	0	8,57143
0	0	1	dwa czynni	2	540	77,1429	17,1429	60
0	0	1	jeden czyn	3	0	0	0	0
0	0	0	metabolicz	2	120	17,1429	0	17,1429
0	0	0	metabolicz	2	0	0	0	0
0	1	0	trzy i więce	2	90	12,8571	0	12,8571
1	1	1	trzy i więce	2	90	12,8571	0	12,8571
1	0	1	trzy i więce	2	0	0	0	0
0	0	0	jeden czyn	1	210	30	0	30
0	1	1	trzy i więce	2	0	0	0	0
0	0	1	jeden czyn	2	0	0	0	0
0	0	0	jeden czyn	1	80	11,4286	0	11,4286
1	1	0	trzy i więce	3	180	25,7143	8,57143	17,1429
0	1	0	jeden czyn	1	0	0	0	0
0	0	1	dwa czynni	3	120	17,1429	0	17,1429
1	0	1	trzy i więce	1	0	0	0	0
0	1	0	jeden czyn	2	0	0	0	0
0	0	1	dwa czynni	1	120	17,1429	0	17,1429
0	1	0	trzy i więce	2	240	34,2857	34,2857	0
0	0	1	dwa czynni	2	60	8,57143	8,57143	0
0	0	1	dwa czynni	3	0	0	0	0
0	0	0	metabolicz	1	0	0	0	0
0	1	1	trzy i więce	1	90	12,8571	12,8571	0
0	0	0	jeden czyn	2	0	0	0	0
0	0	1	dwa czynni	2	0	0	0	0
0	1	0	trzy i więce	2	120	17,1429	0	17,1429
1	1	0	trzy i więce	2	90	12,8571	0	12,8571
0	0	1	dwa czynni	2	330	47,1429	12,8571	34,2857
0	1	1	trzy i więce	2	420	60	0	60
1	0	1	trzy i więce	1	840	120	0	120
0	0	0	metabolicz	1	120	17,1429	17,1429	0
0	0	0	metabolicz	2	80	11,4286	11,4286	0
0	0	0	dwa czynni	3	45	6,42857	6,42857	0
0	0	1	dwa czynni	3	0	0	0	0
0	0	0	jeden czyn	2	840	120	0	120
0	0	0	metabolicz	2	420	60	51,4286	8,57143
0	0	1	jeden czyn	2	0	0	0	0
0	0	0	metabolicz	3	120	17,1429	17,1429	0
0	0	1	dwa czynni	3	0	0	0	0
0	0	0	jeden czyn	2	0	0	0	0
0	0	0	jeden czyn	3	60	8,57143	0	8,57143
1	0	1	trzy i więce	2	0	0	0	0
0	0	0	metabolicz	1	420	60	0	60
0	0	1	jeden czyn	2	180	25,7143	0	25,7143

0	0	1	dwa czynni	3	240	34,2857	34,2857	0
0	1	0	dwa czynni	1	0	0	0	0
0	0	1	trzy i więce	3	1020	145,714	85,7143	60
0	0	0	jeden czyn	3	0	0	0	0
0	0	1	jeden czyn	2	0	0	0	0
0	0	1	jeden czyn	2	180	25,7143	0	25,7143
1	0	0	dwa czynni	1	60	8,57143	0	8,57143
1	0	1	trzy i więce	1	0	0	0	0
1	0	0	jeden czyn	1	120	17,1429	0	17,1429
0	1	1	dwa czynni	2	0	0	0	0
0	0	0	jeden czyn	2	0	0	0	0
0	0	0	jeden czyn	2	30	4,28571	0	4,28571
0	0	1	dwa czynni	1	0	0	0	0
0	0	1	dwa czynni	2	0	0	0	0
1	0	1	trzy i więce	2	0	0	0	0
0	0	0	metabolicz	3	0	0	0	0
0	0	1	dwa czynni	3	270	38,5714	21,4286	17,1429
0	0	1	dwa czynni	2	360	51,4286	51,4286	0
1	0	1	trzy i więce	1	0	0	0	0
0	0	0	jeden czyn	2	60	8,57143	8,57143	0
1	1	1	trzy i więce	2	0	0	0	0
0	0	0	metabolicz	3	20	2,85714	0	2,85714
1	0	1	trzy i więce	2	120	17,1429	0	17,1429
1	0	0	dwa czynni	1	150	21,4286	6,42857	15
0	0	0	metabolicz	2	60	8,57143	0	8,57143
0	0	1	dwa czynni	2	720	102,857	0	102,857
0	0	0	metabolicz	3	360	51,4286	0	51,4286
1	0	1	trzy i więce	2	60	8,57143	0	8,57143
0	1	0	trzy i więce	1	420	60	0	60
0	0	0	metabolicz	3	0	0	0	0
0	0	1	dwa czynni	2	0	0	0	0
0	0	0	metabolicz	2	300	42,8571	12,8571	30
0	0	1	jeden czyn	3	0	0	0	0
0	0	0	jeden czyn	2	0	0	0	0
0	0	1	dwa czynni	2	120	17,1429	0	17,1429
0	1	0	trzy i więce	1	420	60	0	60
0	0	1	dwa czynni	2	120	17,1429	0	17,1429
0	1	1	trzy i więce	2	360	51,4286	0	51,4286
0	0	0	metabolicz	3	60	8,57143	0	8,57143
1	0	1	trzy i więce	1	0	0	0	0
1	0	0	trzy i więce	3	30	4,28571	4,28571	0
0	1	1	trzy i więce	1	0	0	0	0
0	1	0	dwa czynni	2	480	68,5714	34,2857	34,2857
1	0	1	trzy i więce	2	0	0	0	0
0	0	1	jeden czyn	1	0	0	0	0
1	0	0	trzy i więce	2	0	0	0	0
0	1	1	trzy i więce	3	0	0	0	0
0	1	1	dwa czynni	1	0	0	0	0

1	0	1	trzy i więce	2	0	0	0	0
1	0	0	jeden czyn	1	0	0	0	0
0	0	0	metabolicz	3	120	17,1429	17,1429	0
0	0	0	jeden czyn	2	0	0	0	0
0	0	1	jeden czyn	2	285	40,7143	34,2857	6,42857
0	0	0	metabolicz	2	570	81,4286	60	21,4286
0	1	1	trzy i więce	2	225	32,1429	15	17,1429
0	1	0	dwa czynni	2	60	8,57143	8,57143	0
0	0	1	jeden czyn	1	0	0	0	0
0	0	1	jeden czyn	2	320	45,7143	20	25,7143
1	0	1	trzy i więce	1	0	0	0	0
0	0	1	trzy i więce	2	480	68,5714	8,57143	60
0	0	1	dwa czynni	1	130	18,5714	12,8571	5,71429
0	1	0	trzy i więce	2	60	8,57143	0	8,57143
0	0	0	jeden czyn	2	0	0	0	0
0	1	0	trzy i więce	2	0	0	0	0
0	0	1	dwa czynni	2	0	0	0	0
0	0	0	jeden czyn	3	0	0	0	0
0	0	1	jeden czyn	2	60	8,57143	0	8,57143
0	0	0	jeden czyn	3	0	0	0	0
0	0	0	metabolicz	2	60	8,57143	0	8,57143
1	0	1	trzy i więce	2	150	21,4286	0	21,4286
0	0	0	metabolicz	3	600	85,7143	60	25,7143
0	0	0	metabolicz	2	60	8,57143	8,57143	0
1	1	1	trzy i więce	2	90	12,8571	6,42857	6,42857
0	0	1	jeden czyn	2	0	0	0	0
0	0	1	jeden czyn	2	480	68,5714	51,4286	17,1429
0	0	0	jeden czyn	1	180	25,7143	0	25,7143
0	0	1	dwa czynni	3	840	120	0	120
0	0	0	metabolicz	2	120	17,1429	17,1429	0
0	0	0	jeden czyn	2	50	7,14286	7,14286	0
0	1	1	trzy i więce	2	60	8,57143	0	8,57143
0	0	1	jeden czyn	1	405	57,8571	23,5714	34,2857
0	0	1	jeden czyn	1	420	60	0	60
0	0	0	metabolicz	3	120	17,1429	0	17,1429
1	0	1	trzy i więce	2	240	34,2857	17,1429	17,1429
0	0	0	metabolicz	2	0	0	0	0
1	0	0	dwa czynni	3	60	8,57143	0	8,57143
1	0	1	trzy i więce	1	0	0	0	0
1	0	1	trzy i więce	2	120	17,1429	17,1429	0
0	0	1	jeden czyn	1	90	12,8571	12,8571	0
0	1	1	trzy i więce	2	240	34,2857	17,1429	17,1429
1	0	0	dwa czynni	3	120	17,1429	0	17,1429
0	0	0	metabolicz	1	0	0	0	0
0	0	1	dwa czynni	3	840	120	0	120
0	0	0	metabolicz	3	315	45	0	45
0	0	1	jeden czyn	3	0	0	0	0
0	1	1	trzy i więce	1	0	0	0	0

1	1	0	trzy i więce	1	0	0	0	0
0	0	0	jeden czyn	1	490	70	10	60
0	0	0	jeden czyn	3	0	0	0	0
0	0	0	metabolicz	2	60	8,57143	0	8,57143
1	0	0	dwa czynni	2	180	25,7143	4,28571	21,4286
0	0	0	jeden czyn	2	0	0	0	0
0	0	0	jeden czyn	2	0	0	0	0
1	0	0	trzy i więce	2	0	0	0	0
0	0	0	jeden czyn	3	30	4,28571	0	4,28571
0	0	1	dwa czynni	2	180	25,7143	0	25,7143
0	0	0	jeden czyn	2	0	0	0	0
0	1	0	dwa czynni	2	60	8,57143	0	8,57143
0	0	0	metabolicz	3	240	34,2857	30	4,28571
0	0	1	dwa czynni	1	0	0	0	0
0	0	1	dwa czynni	2	560	80	11,4286	68,5714
0	0	1	dwa czynni	3	80	11,4286	0	11,4286
1	1	0	trzy i więce	3	180	25,7143	0	25,7143
0	0	1	trzy i więce	2	120	17,1429	17,1429	0
0	1	0	trzy i więce	2	60	8,57143	0	8,57143
0	0	1	dwa czynni	2	630	90	12,8571	77,1429
0	0	0	jeden czyn	2	30	4,28571	4,28571	0
0	0	1	dwa czynni	2	0	0	0	0
0	0	1	dwa czynni	1	45	6,42857	6,42857	0
1	1	0	trzy i więce	3	1020	145,714	77,1429	68,5714
0	0	1	trzy i więce	2	840	120	120	0
0	1	0	jeden czyn	2	240	34,2857	0	34,2857
1	1	1	trzy i więce	3	0	0	0	0
0	0	0	jeden czyn	2	0	0	0	0
1	1	0	trzy i więce	2	30	4,28571	4,28571	0
1	1	1	trzy i więce	2	0	0	0	0
1	0	0	dwa czynni	2	60	8,57143	0	8,57143
0	0	1	jeden czyn	2	0	0	0	0
0	0	0	metabolicz	2	60	8,57143	0	8,57143
0	0	1	dwa czynni	1	0	0	0	0
0	0	1	trzy i więce	3	155	22,1429	10,7143	11,4286
1	1	1	trzy i więce	2	0	0	0	0
0	0	1	dwa czynni	1	0	0	0	0
0	0	0	metabolicz	2	720	102,857	25,7143	77,1429
0	0	0	metabolicz	2	90	12,8571	12,8571	0
0	0	1	jeden czyn	3	60	8,57143	0	8,57143
1	1	0	trzy i więce	2	0	0	0	0
0	0	1	dwa czynni	1	525	75	6,42857	68,5714
0	0	0	metabolicz	2	0	0	0	0
0	0	0	jeden czyn	2	120	17,1429	0	17,1429
0	0	0	metabolicz	2	420	60	8,57143	51,4286
0	0	1	jeden czyn	2	0	0	0	0
0	0	0	jeden czyn	3	0	0	0	0
0	0	0	dwa czynni	1	70	10	0	10

0	0	0	metabolicz	2	420	60	0	60
0	1	1	dwa czynni	1	735	105	45	60
0	0	1	dwa czynni	3	0	0	0	0
0	0	0	jeden czyn	3	0	0	0	0
1	0	0	dwa czynni	2	460	65,7143	25,7143	40
0	0	1	jeden czyn	2	300	42,8571	17,1429	25,7143
0	1	1	trzy i więc	2	430	61,4286	10	51,4286
0	1	1	dwa czynni	1	105	15	6,42857	8,57143
0	0	1	dwa czynni	2	0	0	0	0
0	0	1	dwa czynni	2	180	25,7143	25,7143	0
0	1	0	dwa czynni	2	15	2,14286	2,14286	0
0	0	0	jeden czyn	2	0	0	0	0
0	0	0	metabolicz	1	105	15	15	0
0	0	1	jeden czyn	3	105	15	15	0
0	0	0	jeden czyn	2	0	0	0	0
0	0	0	jeden czyn	3	0	0	0	0
1	0	1	trzy i więc	2	0	0	0	0
0	1	0	jeden czyn	2	70	10	0	10
0	0	1	jeden czyn	1	0	0	0	0
0	0	0	metabolicz	1	0	0	0	0
0	0	1	dwa czynni	1	420	60	0	60
0	0	1	jeden czyn	2	60	8,57143	0	8,57143
0	0	1	dwa czynni	1	120	17,1429	0	17,1429
1	0	1	trzy i więc	2	40	5,71429	0	5,71429
0	0	0	metabolicz	3	0	0	0	0
1	1	1	trzy i więc	3	180	25,7143	0	25,7143
0	0	1	dwa czynni	2	160	22,8571	14,2857	8,57143
0	0	1	jeden czyn	3	1020	145,714	25,7143	120
0	1	0	dwa czynni	2	60	8,57143	0	8,57143
0	0	1	dwa czynni	2	120	17,1429	0	17,1429
0	0	0	metabolicz	3	120	17,1429	17,1429	0
0	0	0	jeden czyn	3	420	60	0	60
1	0	1	trzy i więc	2	90	12,8571	0	12,8571
0	0	1	dwa czynni	2	0	0	0	0
1	1	0	trzy i więc	2	0	0	0	0
1	1	1	trzy i więc	2	240	34,2857	0	34,2857
0	0	0	metabolicz	2	780	111,429	25,7143	85,7143
0	0	1	trzy i więc	2	270	38,5714	34,2857	4,28571
0	0	1	jeden czyn	2	80	11,4286	0	11,4286
0	0	1	dwa czynni	2	0	0	0	0
0	1	1	trzy i więc	2	210	30	0	30
0	0	0	metabolicz	1	30	4,28571	0	4,28571
0	0	1	dwa czynni	2	105	15	15	0
0	0	1	jeden czyn	2	80	11,4286	0	11,4286
0	0	0	metabolicz	2	930	132,857	12,8571	120
0	1	1	trzy i więc	3	360	51,4286	0	51,4286
0	0	1	dwa czynni	2	0	0	0	0
0	0	1	jeden czyn	2	120	17,1429	0	17,1429

1	0	1	trzy i więce	3	720	102,857	42,8571	60
0	1	1	trzy i więce	2	120	17,1429	0	17,1429
0	0	0	metabolicz	1	1080	154,286	154,286	0
0	0	0	jeden czyn	1	210	30	30	0
0	0	1	dwa czynni	2	420	60	34,2857	25,7143
0	0	0	metabolicz	3	720	102,857	68,5714	34,2857
0	0	1	jeden czyn	3	150	21,4286	0	21,4286
0	0	1	dwa czynni	2	60	8,57143	0	8,57143
1	0	0	dwa czynni	1	0	0	0	0
0	0	1	jeden czyn	2	150	21,4286	21,4286	0
0	0	1	trzy i więce	2	0	0	0	0
1	0	0	dwa czynni	1	60	8,57143	0	8,57143
0	1	1	dwa czynni	2	1320	188,571	120	68,5714
0	0	1	dwa czynni	3	480	68,5714	17,1429	51,4286
1	0	1	trzy i więce	2	0	0	0	0
0	1	0	jeden czyn	2	0	0	0	0
0	0	1	trzy i więce	2	0	0	0	0
0	0	1	dwa czynni	3	120	17,1429	0	17,1429
1	1	1	trzy i więce	1	120	17,1429	0	17,1429
0	0	1	dwa czynni	2	240	34,2857	25,7143	8,57143
1	0	0	dwa czynni	2	300	42,8571	0	42,8571
1	0	1	trzy i więce	2	90	12,8571	6,42857	6,42857
0	0	1	dwa czynni	3	240	34,2857	0	34,2857
0	0	0	metabolicz	3	0	0	0	0
0	0	1	trzy i więce	2	200	28,5714	0	28,5714
0	0	0	metabolicz	1	0	0	0	0
1	0	0	dwa czynni	1	0	0	0	0
1	0	1	trzy i więce	3	0	0	0	0
0	0	0	metabolicz	3	0	0	0	0
0	0	1	trzy i więce	1	30	4,28571	0	4,28571
0	0	0	jeden czyn	3	0	0	0	0
0	0	1	dwa czynni	3	0	0	0	0
0	0	1	dwa czynni	2	0	0	0	0
0	0	0	jeden czyn	3	270	38,5714	12,8571	25,7143
0	0	1	jeden czyn	1	0	0	0	0
1	0	0	dwa czynni	2	0	0	0	0
1	0	1	trzy i więce	3	0	0	0	0
0	0	0	jeden czyn	2	60	8,57143	0	8,57143
0	0	0	jeden czyn	2	420	60	0	60
0	0	0	metabolicz	1	180	25,7143	0	25,7143
0	0	0	jeden czyn	1	0	0	0	0
0	0	1	dwa czynni	2	60	8,57143	0	8,57143
0	0	0	jeden czyn	3	855	122,143	77,1429	45
1	0	1	trzy i więce	3	145	20,7143	17,1429	3,57143
0	0	1	jeden czyn	3	30	4,28571	4,28571	0
1	0	1	trzy i więce	2	360	51,4286	51,4286	0
0	0	0	jeden czyn	1	0	0	0	0
0	0	1	dwa czynni	2	120	17,1429	0	17,1429

1	0	0	dwa czynni	2	0	0	0	0
0	1	1	trzy i więc	1	165	23,5714	6,42857	17,1429
0	0	0	jeden czyn	3	0	0	0	0
0	0	1	dwa czynni	2	0	0	0	0
1	1	1	trzy i więc	3	15	2,14286	0	2,14286
0	0	0	dwa czynni	1	25	3,57143	0	3,57143
0	1	1	trzy i więc	3	120	17,1429	0	17,1429
0	0	1	dwa czynni	3	0	0	0	0
0	0	0	metabolicz	2	0	0	0	0
0	0	0	jeden czyn	1	60	8,57143	0	8,57143
0	0	0	jeden czyn	2	0	0	0	0
0	1	1	dwa czynni	2	0	0	0	0
1	1	1	trzy i więc	2	0	0	0	0
0	0	1	jeden czyn	2	20	2,85714	0	2,85714
1	1	1	trzy i więc	3	420	60	0	60
0	0	0	metabolicz	2	60	8,57143	0	8,57143
0	0	1	jeden czyn	2	300	42,8571	42,8571	0
0	1	1	trzy i więc	1	180	25,7143	25,7143	0
1	0	0	jeden czyn	1	1080	154,286	128,571	25,7143
0	1	1	trzy i więc	3	0	0	0	0
0	0	1	jeden czyn	2	120	17,1429	0	17,1429
0	0	1	jeden czyn	2	140	20	0	20
0	0	1	jeden czyn	2	0	0	0	0
0	0	1	dwa czynni	1	0	0	0	0
0	0	1	jeden czyn	2	0	0	0	0
0	0	0	metabolicz	1	45	6,42857	6,42857	0
0	0	1	dwa czynni	1	300	42,8571	17,1429	25,7143
0	1	1	trzy i więc	2	0	0	0	0
0	0	0	metabolicz	2	300	42,8571	17,1429	25,7143
0	0	1	dwa czynni	2	1260	180	0	180
1	0	1	trzy i więc	2	120	17,1429	0	17,1429
0	0	0	metabolicz	1	280	40	14,2857	25,7143
0	0	0	metabolicz	2	240	34,2857	8,57143	25,7143
0	1	1	trzy i więc	1	0	0	0	0
1	0	0	dwa czynni	1	0	0	0	0
0	0	0	jeden czyn	2	0	0	0	0
1	0	0	dwa czynni	1	0	0	0	0
1	0	1	trzy i więc	2	195	27,8571	10,7143	17,1429
0	0	1	dwa czynni	1	210	30	0	30
0	0	0	jeden czyn	1	60	8,57143	0	8,57143
1	0	1	trzy i więc	2	300	42,8571	30	12,8571
0	0	0	metabolicz	2	60	8,57143	0	8,57143
0	0	0	metabolicz	3	90	12,8571	0	12,8571
0	0	1	jeden czyn	1	0	0	0	0
0	0	1	jeden czyn	2	180	25,7143	0	25,7143
0	0	0	metabolicz	2	0	0	0	0
0	0	0	jeden czyn	2	0	0	0	0
1	1	1	trzy i więc	3	840	120	0	120

0	0	0	metabolicz	2	185	26,4286	20	6,42857
0	0	1	trzy i więce	2	0	0	0	0
0	0	0	metabolicz	2	300	42,8571	42,8571	0
0	0	0	metabolicz	2	0	0	0	0
0	0	0	metabolicz	3	0	0	0	0
0	1	0	dwa czynni	1	60	8,57143	0	8,57143
0	0	1	dwa czynni	1	0	0	0	0
0	1	0	dwa czynni	2	0	0	0	0
0	0	0	metabolicz	1	120	17,1429	0	17,1429
0	0	1	jeden czyn	1	510	72,8571	38,5714	34,2857
0	1	0	jeden czyn	3	570	81,4286	51,4286	30
0	0	0	jeden czyn	2	900	128,571	0	128,571
0	0	1	dwa czynni	2	280	40	0	40
1	0	0	dwa czynni	1	0	0	0	0
0	0	1	dwa czynni	2	120	17,1429	0	17,1429
0	0	0	jeden czyn	2	60	8,57143	0	8,57143
0	0	1	jeden czyn	2	60	8,57143	0	8,57143
0	0	0	metabolicz	2	0	0	0	0
1	0	1	trzy i więce	1	0	0	0	0
0	0	0	metabolicz	2	45	6,42857	6,42857	0
0	1	1	dwa czynni	3	90	12,8571	0	12,8571
0	0	0	metabolicz	2	0	0	0	0
0	0	1	trzy i więce	1	0	0	0	0
0	0	0	jeden czyn	2	120	17,1429	8,57143	8,57143
0	0	0	metabolicz	1	80	11,4286	8,57143	2,85714
0	0	0	jeden czyn	2	0	0	0	0
0	0	0	metabolicz	2	140	20	2,85714	17,1429
0	0	1	jeden czyn	2	0	0	0	0
0	0	1	jeden czyn	1	0	0	0	0
1	0	0	dwa czynni	2	120	17,1429	17,1429	0
1	0	0	dwa czynni	1	120	17,1429	0	17,1429
0	1	0	dwa czynni	1	0	0	0	0
0	0	1	dwa czynni	2	120	17,1429	0	17,1429
0	0	1	jeden czyn	2	60	8,57143	0	8,57143
0	0	0	dwa czynni	2	0	0	0	0
0	0	0	jeden czyn	2	0	0	0	0
0	0	0	jeden czyn	2	180	25,7143	8,57143	17,1429
0	0	0	jeden czyn	2	0	0	0	0
0	0	1	jeden czyn	2	150	21,4286	17,1429	4,28571
0	0	1	jeden czyn	2	0	0	0	0
0	1	1	trzy i więce	2	0	0	0	0
1	0	1	trzy i więce	2	0	0	0	0
1	0	1	trzy i więce	2	180	25,7143	0	25,7143
0	0	1	dwa czynni	2	240	34,2857	0	34,2857
0	0	1	dwa czynni	3	0	0	0	0
0	0	1	dwa czynni	3	60	8,57143	0	8,57143
0	0	0	metabolicz	3	600	85,7143	34,2857	51,4286
0	0	1	dwa czynni	3	0	0	0	0

0	0	0	metabolicz	3	0	0	0	0
0	0	0	jeden czyn	3	420	60	0	60
0	0	1	jeden czyn	2	180	25,7143	17,1429	8,57143
0	0	0	metabolicz	2	30	4,28571	0	4,28571
0	0	1	dwa czynni	1	0	0	0	0
0	0	1	jeden czyn	2	0	0	0	0
0	0	0	jeden czyn	3	0	0	0	0
0	0	0	metabolicz	3	180	25,7143	17,1429	8,57143
0	0	0	metabolicz	3	0	0	0	0
0	0	0	metabolicz	2	320	45,7143	45,7143	0
0	0	0	metabolicz	3	300	42,8571	0	42,8571
0	0	0	metabolicz	2	0	0	0	0
1	0	1	dwa czynni	2	0	0	0	0
1	0	0	dwa czynni	2	120	17,1429	17,1429	0
0	0	0	jeden czyn	3	0	0	0	0
0	0	0	metabolicz	2	105	15	15	0
0	0	1	dwa czynni	2	30	4,28571	0	4,28571
0	1	1	dwa czynni	2	0	0	0	0
0	0	1	jeden czyn	3	240	34,2857	34,2857	0
0	0	0	metabolicz	1	345	49,2857	6,42857	42,8571
0	0	1	dwa czynni	2	0	0	0	0
0	0	0	metabolicz	3	0	0	0	0
0	0	1	jeden czyn	2	60	8,57143	8,57143	0
0	0	1	dwa czynni	3	620	88,5714	2,85714	85,7143
0	0	0	metabolicz	2	0	0	0	0
0	1	1	trzy i więce	3	45	6,42857	6,42857	0
0	0	1	dwa czynni	2	60	8,57143	0	8,57143
0	0	1	dwa czynni	2	0	0	0	0
0	0	0	metabolicz	3	120	17,1429	0	17,1429
0	0	1	trzy i więce	3	0	0	0	0
0	1	1	trzy i więce	2	60	8,57143	0	8,57143
0	0	1	jeden czyn	2	60	8,57143	0	8,57143
1	1	1	trzy i więce	1	220	31,4286	8,57143	22,8571
0	1	1	trzy i więce	3	40	5,71429	5,71429	0
1	1	1	trzy i więce	2	0	0	0	0
0	0	0	metabolicz	3	0	0	0	0
0	0	0	jeden czyn	2	0	0	0	0
0	0	1	dwa czynni	2	40	5,71429	5,71429	0
0	0	1	dwa czynni	2	0	0	0	0
1	1	1	trzy i więce	1	0	0	0	0
0	0	0	metabolicz	1	180	25,7143	25,7143	0
0	0	0	metabolicz	2	0	0	0	0
0	0	1	dwa czynni	3	45	6,42857	6,42857	0
0	1	0	jeden czyn	2	180	25,7143	0	25,7143
1	0	0	jeden czyn	2	80	11,4286	0	11,4286
0	0	1	jeden czyn	3	450	64,2857	34,2857	30
0	0	0	jeden czyn	2	60	8,57143	0	8,57143
0	0	1	dwa czynni	2	630	90	85,7143	4,28571

0	0	0	metabolicz	2	360	51,4286	0	51,4286
0	0	0	metabolicz	3	0	0	0	0
0	0	1	jeden czyn	2	420	60	0	60
0	0	1	jeden czyn	2	250	35,7143	5,71429	30
0	0	1	jeden czyn	2	60	8,57143	0	8,57143
0	0	1	dwa czynni	2	90	12,8571	12,8571	0
0	0	1	dwa czynni	2	0	0	0	0
0	0	0	jeden czyn	3	10	1,42857	0	1,42857
0	0	0	metabolicz	2	120	17,1429	0	17,1429
0	0	1	jeden czyn	2	80	11,4286	0	11,4286
1	0	1	trzy i więc	2	210	30	0	30
0	1	0	dwa czynni	2	30	4,28571	0	4,28571
0	0	0	jeden czyn	2	0	0	0	0
1	0	1	trzy i więc	1	45	6,42857	0	6,42857
0	0	0	metabolicz	2	60	8,57143	8,57143	0
0	0	0	metabolicz	2	210	30	0	30
1	0	0	jeden czyn	2	180	25,7143	17,1429	8,57143
0	0	1	dwa czynni	1	420	60	0	60
0	1	1	dwa czynni	2	0	0	0	0
0	1	1	trzy i więc	2	180	25,7143	0	25,7143
0	0	1	jeden czyn	2	60	8,57143	0	8,57143
0	0	1	dwa czynni	2	0	0	0	0
0	0	1	dwa czynni	2	315	45	15	30
0	1	0	dwa czynni	2	105	15	6,42857	8,57143
0	0	0	metabolicz	2	0	0	0	0
0	0	1	jeden czyn	3	30	4,28571	4,28571	0
0	0	0	jeden czyn	2	540	77,1429	68,5714	8,57143
0	1	0	jeden czyn	2	210	30	30	0
0	0	1	trzy i więc	1	240	34,2857	0	34,2857
0	0	0	metabolicz	2	0	0	0	0
0	1	1	trzy i więc	2	120	17,1429	0	17,1429
0	0	0	metabolicz	2	360	51,4286	0	51,4286
0	0	1	dwa czynni	2	0	0	0	0
0	0	0	jeden czyn	3	300	42,8571	34,2857	8,57143
1	0	1	trzy i więc	1	0	0	0	0
0	0	0	jeden czyn	2	540	77,1429	0	77,1429
0	0	1	jeden czyn	2	120	17,1429	0	17,1429
0	0	1	dwa czynni	2	225	32,1429	15	17,1429
0	0	0	jeden czyn	2	0	0	0	0
0	0	0	metabolicz	2	120	17,1429	0	17,1429
0	0	1	dwa czynni	3	360	51,4286	51,4286	0
0	0	1	dwa czynni	2	630	90	12,8571	77,1429
0	0	0	jeden czyn	2	0	0	0	0
0	0	0	dwa czynni	2	0	0	0	0
0	0	0	metabolicz	1	60	8,57143	8,57143	0
0	0	0	metabolicz	3	90	12,8571	12,8571	0
0	0	0	metabolicz	3	210	30	0	30
0	0	0	metabolicz	1	0	0	0	0

1	0	1	trzy i więce	1	60	8,57143	0	8,57143
0	0	1	dwa czynni	2	660	94,2857	25,7143	68,5714
0	0	1	dwa czynni	3	200	28,5714	0	28,5714
0	0	0	metabolicz	1	630	90	30	60
1	0	0	trzy i więce	2	450	64,2857	42,8571	21,4286
0	0	1	dwa czynni	3	60	8,57143	0	8,57143
0	1	1	dwa czynni	2	420	60	0	60
0	0	0	metabolicz	1	840	120	0	120
0	0	0	jeden czyn	2	0	0	0	0
0	0	0	jeden czyn	3	480	68,5714	68,5714	0
0	0	0	metabolicz	1	0	0	0	0
1	0	0	trzy i więce	2	180	25,7143	0	25,7143
0	0	0	dwa czynni	2	85	12,1429	6,42857	5,71429
1	0	1	trzy i więce	3	60	8,57143	0	8,57143
0	0	0	jeden czyn	2	0	0	0	0
0	0	1	jeden czyn	1	140	20	0	20
0	1	0	jeden czyn	2	810	115,714	64,2857	51,4286
1	1	1	trzy i więce	2	600	85,7143	51,4286	34,2857
0	0	1	dwa czynni	3	120	17,1429	17,1429	0
0	0	1	jeden czyn	3	250	35,7143	12,8571	22,8571
0	0	0	jeden czyn	2	60	8,57143	0	8,57143
0	0	0	jeden czyn	3	180	25,7143	25,7143	0
0	0	1	jeden czyn	2	0	0	0	0
0	0	1	dwa czynni	2	0	0	0	0
0	0	0	jeden czyn	3	0	0	0	0
0	0	1	jeden czyn	2	0	0	0	0
0	1	1	trzy i więce	2	60	8,57143	8,57143	0
0	0	0	metabolicz	2	120	17,1429	17,1429	0
0	0	0	metabolicz	2	0	0	0	0
0	0	1	jeden czyn	2	180	25,7143	25,7143	0
0	0	1	jeden czyn	3	840	120	120	0
0	0	0	jeden czyn	2	105	15	15	0
0	0	1	jeden czyn	2	480	68,5714	25,7143	42,8571
0	0	1	dwa czynni	2	60	8,57143	0	8,57143
0	0	0	metabolicz	1	120	17,1429	0	17,1429
1	1	1	trzy i więce	2	0	0	0	0
0	0	0	dwa czynni	3	630	90	12,8571	77,1429
0	0	1	jeden czyn	2	540	77,1429	42,8571	34,2857
0	0	0	metabolicz	2	0	0	0	0
0	0	0	metabolicz	2	540	77,1429	17,1429	60
0	0	1	jeden czyn	2	420	60	0	60
0	0	0	jeden czyn	2	390	55,7143	12,8571	42,8571
0	0	1	jeden czyn	3	120	17,1429	0	17,1429
0	0	0	jeden czyn	2	0	0	0	0
0	0	1	trzy i więce	1	300	42,8571	0	42,8571
0	0	0	jeden czyn	2	0	0	0	0
0	0	0	jeden czyn	2	0	0	0	0
0	0	0	jeden czyn	1	0	0	0	0

0	0	0	jeden czyn	3	120	17,1429	0	17,1429
0	0	1	trzy i więce	3	0	0	0	0
0	0	1	jeden czyn	2	60	8,57143	0	8,57143
0	0	1	jeden czyn	1	120	17,1429	0	17,1429
0	0	1	dwa czynni	2	120	17,1429	17,1429	0
0	0	1	jeden czyn	2	0	0	0	0
0	0	1	jeden czyn	2	180	25,7143	17,1429	8,57143
1	0	0	dwa czynni	2	0	0	0	0
0	0	0	jeden czyn	2	0	0	0	0
0	0	1	dwa czynni	3	0	0	0	0
1	0	1	trzy i więce	3	120	17,1429	17,1429	0
1	1	1	trzy i więce	3	120	17,1429	0	17,1429
0	0	1	dwa czynni	1	150	21,4286	0	21,4286
0	0	0	jeden czyn	3	0	0	0	0
0	0	0	jeden czyn	3	840	120	102,857	17,1429
1	0	1	trzy i więce	2	0	0	0	0
0	0	0	jeden czyn	2	70	10	10	0
0	0	0	jeden czyn	1	30	4,28571	0	4,28571
0	0	1	dwa czynni	2	165	23,5714	6,42857	17,1429
0	0	1	dwa czynni	2	0	0	0	0
1	0	1	trzy i więce	2	0	0	0	0
0	0	0	metabolicz	2	30	4,28571	4,28571	0
0	0	0	metabolicz	2	0	0	0	0
0	0	1	jeden czyn	2	0	0	0	0
0	0	1	jeden czyn	2	0	0	0	0
0	0	1	dwa czynni	3	0	0	0	0
0	0	0	metabolicz	2	0	0	0	0
0	0	1	jeden czyn	2	60	8,57143	0	8,57143
0	1	1	trzy i więce	1	90	12,8571	0	12,8571
0	0	1	trzy i więce	2	600	85,7143	68,5714	17,1429
0	0	0	jeden czyn	2	120	17,1429	0	17,1429
0	0	1	dwa czynni	2	120	17,1429	0	17,1429
1	1	1	trzy i więce	1	1080	154,286	154,286	0
0	0	0	metabolicz	2	0	0	0	0
0	0	0	jeden czyn	1	30	4,28571	0	4,28571
0	0	0	metabolicz	3	0	0	0	0
0	1	0	dwa czynni	2	60	8,57143	0	8,57143
0	0	0	metabolicz	2	0	0	0	0
0	0	1	jeden czyn	1	40	5,71429	0	5,71429
0	0	0	jeden czyn	2	0	0	0	0
0	0	1	dwa czynni	2	840	120	0	120
0	0	1	dwa czynni	1	120	17,1429	0	17,1429
1	0	1	trzy i więce	2	420	60	0	60
0	0	0	metabolicz	3	480	68,5714	51,4286	17,1429
1	0	1	trzy i więce	2	0	0	0	0
0	0	0	metabolicz	1	0	0	0	0
0	0	1	jeden czyn	2	360	51,4286	25,7143	25,7143
0	0	1	dwa czynni	2	420	60	0	60

1	0	1	trzy i więce	2	120	17,1429	17,1429	0
0	0	1	dwa czynni	1	360	51,4286	0	51,4286
1	0	0	jeden czyn	3	240	34,2857	8,57143	25,7143
0	0	1	dwa czynni	3	160	22,8571	0	22,8571
0	0	0	jeden czyn	2	0	0	0	0
0	0	1	dwa czynni	1	0	0	0	0
0	0	0	jeden czyn	2	120	17,1429	0	17,1429
1	0	1	dwa czynni	2	150	21,4286	21,4286	0
0	0	1	trzy i więce	1	0	0	0	0
0	0	0	metabolicz	1	45	6,42857	6,42857	0
0	0	0	jeden czyn	1	0	0	0	0
1	0	1	trzy i więce	3	0	0	0	0
1	0	1	trzy i więce	2	10	1,42857	1,42857	0
0	0	0	metabolicz	1	800	114,286	11,4286	102,857
0	1	1	trzy i więce	3	0	0	0	0
0	0	1	jeden czyn	2	0	0	0	0
0	0	1	jeden czyn	3	0	0	0	0
1	0	1	dwa czynni	1	420	60	0	60
0	0	0	metabolicz	2	45	6,42857	0	6,42857
0	1	0	dwa czynni	3	120	17,1429	0	17,1429
1	0	0	jeden czyn	1	0	0	0	0
0	0	0	metabolicz	3	0	0	0	0
0	0	1	dwa czynni	3	240	34,2857	0	34,2857
0	0	0	metabolicz	2	60	8,57143	8,57143	0
0	0	0	metabolicz	2	240	34,2857	0	34,2857
0	0	0	metabolicz	2	60	8,57143	0	8,57143
1	0	1	trzy i więce	2	0	0	0	0
1	1	1	trzy i więce	2	90	12,8571	12,8571	0
1	0	1	trzy i więce	2	0	0	0	0
0	0	0	metabolicz	2	270	38,5714	38,5714	0
0	0	0	metabolicz	2	0	0	0	0
0	0	0	jeden czyn	2	0	0	0	0
0	0	0	jeden czyn	2	0	0	0	0
1	1	1	trzy i więce	1	0	0	0	0
1	0	0	dwa czynni	2	280	40	5,71429	34,2857
0	0	0	metabolicz	1	180	25,7143	0	25,7143
0	0	1	trzy i więce	1	0	0	0	0
0	0	1	jeden czyn	2	120	17,1429	17,1429	0
0	0	1	trzy i więce	2	0	0	0	0
1	0	0	dwa czynni	2	0	0	0	0
1	1	1	trzy i więce	2	840	120	0	120
0	0	1	dwa czynni	3	0	0	0	0
0	0	0	jeden czyn	2	0	0	0	0
0	1	1	trzy i więce	3	60	8,57143	8,57143	0
1	0	0	dwa czynni	1	360	51,4286	25,7143	25,7143
0	0	0	metabolicz	2	360	51,4286	0	51,4286
0	0	1	jeden czyn	1	180	25,7143	25,7143	0
0	0	0	metabolicz	1	120	17,1429	0	17,1429

0	0	1	jeden czyn	3	0	0	0	0
0	0	1	jeden czyn	2	195	27,8571	10,7143	17,1429
0	0	0	metabolicz	1	90	12,8571	12,8571	0
0	0	0	metabolicz	2	120	17,1429	11,4286	5,71429
0	0	0	jeden czyn	2	60	8,57143	0	8,57143
0	0	0	jeden czyn	2	105	15	0	15
0	0	0	metabolicz	2	360	51,4286	0	51,4286
0	0	0	jeden czyn	2	60	8,57143	0	8,57143
0	0	0	jeden czyn	2	60	8,57143	0	8,57143
0	0	1	jeden czyn	3	0	0	0	0
1	0	0	trzy i więc	2	0	0	0	0
1	1	1	trzy i więc	2	90	12,8571	12,8571	0
0	0	1	dwa czynni	1	60	8,57143	0	8,57143
0	0	0	metabolicz	2	0	0	0	0
1	1	0	trzy i więc	1	150	21,4286	4,28571	17,1429
0	0	0	jeden czyn	2	360	51,4286	0	51,4286
0	0	0	dwa czynni	2	360	51,4286	25,7143	25,7143
0	0	0	jeden czyn	3	45	6,42857	6,42857	0
0	0	0	metabolicz	1	180	25,7143	8,57143	17,1429
0	0	1	jeden czyn	2	120	17,1429	17,1429	0
0	1	0	jeden czyn	1	0	0	0	0
0	0	0	metabolicz	3	240	34,2857	17,1429	17,1429
0	0	1	dwa czynni	2	0	0	0	0
0	0	1	dwa czynni	2	510	72,8571	42,8571	30
0	0	1	dwa czynni	2	120	17,1429	17,1429	0
0	0	0	jeden czyn	3	280	40	0	40
1	1	1	trzy i więc	2	120	17,1429	0	17,1429
0	0	1	trzy i więc	3	0	0	0	0
1	0	1	dwa czynni	1	0	0	0	0
0	0	0	metabolicz	2	30	4,28571	0	4,28571
0	0	1	dwa czynni	3	180	25,7143	0	25,7143
0	1	1	trzy i więc	2	60	8,57143	4,28571	4,28571
0	0	1	trzy i więc	1	0	0	0	0
0	1	1	trzy i więc	3	60	8,57143	8,57143	0
1	0	0	dwa czynni	1	0	0	0	0
0	0	1	dwa czynni	2	210	30	0	30
0	0	1	jeden czyn	2	20	2,85714	0	2,85714
0	0	0	jeden czyn	2	0	0	0	0
1	0	0	jeden czyn	1	0	0	0	0
0	0	0	jeden czyn	2	0	0	0	0
1	0	0	dwa czynni	1	0	0	0	0
0	0	1	dwa czynni	2	0	0	0	0
0	0	0	jeden czyn	2	30	4,28571	4,28571	0
0	0	0	metabolicz	1	60	8,57143	0	8,57143
0	0	1	jeden czyn	2	0	0	0	0
1	0	1	trzy i więc	1	0	0	0	0
0	0	1	dwa czynni	3	360	51,4286	17,1429	34,2857
0	0	1	dwa czynni	2	220	31,4286	22,8571	8,57143

0	0	1	dwa czynni	1	0	0	0	0
1	1	1	trzy i więc	2	90	12,8571	0	12,8571
0	0	0	jeden czyn	2	0	0	0	0
0	0	1	dwa czynni	1	0	0	0	0
0	0	0	metabolicz	2	0	0	0	0
0	0	1	jeden czyn	1	0	0	0	0
0	0	0	jeden czyn	2	60	8,57143	0	8,57143
0	1	0	dwa czynni	2	120	17,1429	8,57143	8,57143
0	0	1	jeden czyn	2	0	0	0	0
0	0	1	dwa czynni	2	210	30	0	30
0	1	0	dwa czynni	3	60	8,57143	0	8,57143
1	0	0	jeden czyn	2	0	0	0	0
0	1	1	trzy i więc	2	100	14,2857	5,71429	8,57143
0	0	1	dwa czynni	1	60	8,57143	0	8,57143
0	0	0	metabolicz	1	180	25,7143	0	25,7143
0	0	1	dwa czynni	2	0	0	0	0
1	0	0	dwa czynni	2	0	0	0	0
0	0	0	metabolicz	2	60	8,57143	8,57143	0
0	0	0	metabolicz	3	0	0	0	0
0	0	1	dwa czynni	3	70	10	4,28571	5,71429
0	0	1	jeden czyn	2	120	17,1429	0	17,1429
0	0	1	dwa czynni	2	150	21,4286	0	21,4286
0	0	0	jeden czyn	1	300	42,8571	0	42,8571
0	0	1	dwa czynni	2	180	25,7143	17,1429	8,57143
0	0	0	metabolicz	2	105	15	0	15
0	0	1	jeden czyn	3	140	20	0	20
0	1	0	dwa czynni	1	60	8,57143	0	8,57143
0	0	1	dwa czynni	2	20	2,85714	0	2,85714
0	0	1	jeden czyn	2	0	0	0	0
0	0	0	jeden czyn	2	420	60	0	60
0	0	0	metabolicz	2	0	0	0	0
0	0	0	jeden czyn	2	30	4,28571	0	4,28571
0	1	0	dwa czynni	2	0	0	0	0
0	1	1	trzy i więc	2	120	17,1429	17,1429	0
0	0	1	dwa czynni	1	120	17,1429	0	17,1429
0	0	0	jeden czyn	3	60	8,57143	8,57143	0
0	0	0	metabolicz	3	420	60	60	0
0	0	1	jeden czyn	1	0	0	0	0
0	0	0	jeden czyn	2	90	12,8571	0	12,8571
0	0	0	jeden czyn	3	45	6,42857	6,42857	0
0	1	0	jeden czyn	1	0	0	0	0
0	0	1	dwa czynni	2	180	25,7143	17,1429	8,57143
1	0	1	trzy i więc	1	240	34,2857	0	34,2857
0	0	1	dwa czynni	2	0	0	0	0
0	1	1	trzy i więc	1	60	8,57143	0	8,57143
0	0	1	dwa czynni	2	0	0	0	0
0	0	1	jeden czyn	2	120	17,1429	0	17,1429
0	1	0	dwa czynni	2	0	0	0	0

1	0	0	jeden czyn	3	60	8,57143	0	8,57143
0	0	0	metabolicz	3	120	17,1429	0	17,1429
0	1	1	trzy i więc	2	600	85,7143	17,1429	68,5714
1	0	1	trzy i więc	2	260	37,1429	28,5714	8,57143
0	0	0	jeden czyn	3	0	0	0	0
1	0	1	trzy i więc	2	60	8,57143	8,57143	0
0	0	1	dwa czynni	2	210	30	0	30
0	0	1	dwa czynni	1	300	42,8571	42,8571	0
1	1	1	trzy i więc	2	60	8,57143	8,57143	0
0	0	1	dwa czynni	2	0	0	0	0
0	0	0	jeden czyn	1	180	25,7143	0	25,7143
1	0	1	trzy i więc	2	420	60	0	60
0	1	1	dwa czynni	3	60	8,57143	0	8,57143
0	0	1	jeden czyn	1	90	12,8571	4,28571	8,57143
0	0	0	jeden czyn	2	480	68,5714	51,4286	17,1429
0	0	0	jeden czyn	2	0	0	0	0
0	1	1	trzy i więc	3	90	12,8571	4,28571	8,57143
0	0	0	jeden czyn	2	0	0	0	0
0	0	0	metabolicz	3	0	0	0	0
1	0	0	dwa czynni	2	0	0	0	0
0	0	0	jeden czyn	2	60	8,57143	8,57143	0
0	1	1	trzy i więc	2	180	25,7143	0	25,7143
0	1	0	dwa czynni	2	210	30	30	0
1	1	1	trzy i więc	1	0	0	0	0
0	0	0	metabolicz	2	0	0	0	0
1	1	1	trzy i więc	1	315	45	45	0
0	0	0	metabolicz	2	60	8,57143	0	8,57143
0	0	0	jeden czyn	2	90	12,8571	12,8571	0
0	0	0	metabolicz	2	0	0	0	0
0	0	1	jeden czyn	2	90	12,8571	0	12,8571
0	0	0	jeden czyn	2	135	19,2857	0	19,2857
1	0	1	trzy i więc	2	225	32,1429	20,7143	11,4286
1	0	1	trzy i więc	2	0	0	0	0
0	0	1	jeden czyn	2	0	0	0	0
1	0	1	trzy i więc	2	0	0	0	0
0	0	1	jeden czyn	1	0	0	0	0
0	1	1	trzy i więc	3	390	55,7143	30	25,7143
0	0	0	metabolicz	2	120	17,1429	0	17,1429
0	0	0	metabolicz	2	0	0	0	0
0	0	0	metabolicz	1	60	8,57143	0	8,57143
0	0	0	metabolicz	2	0	0	0	0
0	0	0	jeden czyn	3	0	0	0	0
0	0	0	metabolicz	2	150	21,4286	21,4286	0
0	0	1	jeden czyn	2	0	0	0	0
0	0	1	dwa czynni	2	300	42,8571	17,1429	25,7143
0	0	1	jeden czyn	2	0	0	0	0
1	0	0	dwa czynni	2	0	0	0	0
0	0	1	dwa czynni	2	60	8,57143	0	8,57143

0	0	1	jeden czyn	2	420	60	0	60
0	1	0	dwa czynni	3	0	0	0	0
0	0	0	metabolicz	2	0	0	0	0
1	0	0	jeden czyn	2	120	17,1429	0	17,1429
0	0	0	metabolicz	1	180	25,7143	0	25,7143
0	0	1	jeden czyn	2	90	12,8571	12,8571	0
0	0	1	jeden czyn	1	0	0	0	0
0	0	0	metabolicz	1	0	0	0	0
0	0	0	metabolicz	3	300	42,8571	0	42,8571
0	0	0	metabolicz	2	0	0	0	0
0	0	0	metabolicz	2	570	81,4286	51,4286	30
1	0	1	trzy i więc	1	0	0	0	0
1	0	1	dwa czynni	1	0	0	0	0
0	0	1	dwa czynni	2	240	34,2857	17,1429	17,1429
1	0	1	trzy i więc	2	240	34,2857	8,57143	25,7143
1	1	0	dwa czynni	1	240	34,2857	0	34,2857
0	0	0	metabolicz	2	50	7,14286	7,14286	0
0	0	1	jeden czyn	2	90	12,8571	12,8571	0
0	0	0	dwa czynni	2	0	0	0	0
0	0	0	metabolicz	1	0	0	0	0
1	0	0	dwa czynni	1	60	8,57143	0	8,57143
0	0	0	metabolicz	1	0	0	0	0
0	0	1	dwa czynni	2	120	17,1429	17,1429	0
0	0	0	metabolicz	2	670	95,7143	60	35,7143
0	0	0	jeden czyn	1	840	120	0	120
0	0	1	dwa czynni	3	240	34,2857	0	34,2857
0	0	1	dwa czynni	3	450	64,2857	30	34,2857
0	0	0	metabolicz	2	660	94,2857	34,2857	60
0	0	1	jeden czyn	2	385	55	15	40
0	0	0	jeden czyn	2	180	25,7143	0	25,7143
0	1	0	jeden czyn	3	270	38,5714	8,57143	30
0	0	0	jeden czyn	2	0	0	0	0
0	1	1	dwa czynni	1	60	8,57143	0	8,57143
0	0	1	trzy i więc	3	60	8,57143	0	8,57143
0	0	0	metabolicz	2	180	25,7143	8,57143	17,1429
0	0	1	dwa czynni	1	0	0	0	0
1	0	0	dwa czynni	1	45	6,42857	0	6,42857
0	0	1	dwa czynni	2	0	0	0	0
0	0	0	metabolicz	1	0	0	0	0
0	0	1	jeden czyn	2	120	17,1429	8,57143	8,57143
0	0	0	metabolicz	1	0	0	0	0
0	0	0	metabolicz	2	240	34,2857	34,2857	0
0	0	0	jeden czyn	1	0	0	0	0
0	1	1	trzy i więc	2	0	0	0	0
0	1	1	trzy i więc	2	0	0	0	0
0	0	1	dwa czynni	1	80	11,4286	11,4286	0
0	0	0	metabolicz	2	225	32,1429	32,1429	0
0	0	1	jeden czyn	2	60	8,57143	0	8,57143

0	*0*	*1*	jeden czyn	1	*180*	*25,7143*	*0*	*25,7143*
0	*0*	*0*	metabolicz	3	*0*	*0*	*0*	*0*
0	*0*	*1*	dwa czynni	3	*300*	*42,8571*	*42,8571*	*0*
1	*0*	*1*	trzy i więce	3	*600*	*85,7143*	*42,8571*	*42,8571*
0	*0*	*0*	jeden czyn	2	*0*	*0*	*0*	*0*
0	*0*	*1*	jeden czyn	1	*105*	*15*	*0*	*15*

var 14	var 15	var 16	var 17	var 18	var 19	var 20	var 21	var 22
0	0	0	37,1429	0	17,1429	0	0	3
0	0	0	162,857	17,1429	0	1	0	2
0	0	0	25,7143	8,57143	21,4286	0	0	1
0	0	0	60	115,714	120	1	1	1
0	0	0	20	25,7143	8,57143	0	0	3
0	0	0	0	102,857	0	0	1	2
0	0	0	1,42857	8,57143	0	0	0	1
0	0	0	25,7143	42,8571	17,1429	0	1	1
0	0	0	25,7143	0	0	0	0	1
0	0	0	31,4286	14,2857	0	0	0	2
0	0	0	0	12,8571	0	0	0	2
0	0	0	0	97,1429	102,857	0	1	2
0	0	0	47,1429	0	0	0	0	2
0	0	0	0	12,8571	0	0	0	2
0	0	0	14,2857	68,5714	20	0	1	2
0	0	0	25,7143	34,2857	34,2857	0	1	2
0	1	1	171,429	90	0	1	1	2
0	0	0	0	169,286	0	0	1	1
1	0	1	34,2857	102,857	85,7143	0	1	2
0	0	0	60	30	42,8571	1	1	2
0	0	0	0	17,1429	0	0	0	1
0	0	0	21,4286	17,1429	0	0	0	1
0	0	0	21,4286	0	0	0	0	2
0	1	1	214,286	0	0	1	0	2
0	0	0	102,857	0	0	1	0	1
0	0	0	68,5714	0	0	1	0	1
0	0	1	8,57143	60	0	0	1	2
0	0	0	17,1429	52,8571	0	0	0	2
0	0	0	42,8571	51,4286	77,1429	0	1	1
0	0	0	14,2857	74,2857	154,286	0	1	1
0	1	1	180	0	0	1	0	2
0	1	1	180	47,1429	0	1	0	1
0	0	0	34,2857	34,2857	0	0	0	3
0	0	0	0	0	0	0	0	2
0	0	0	85,7143	42,8571	88,5714	1	1	2
0	0	1	65,7143	21,4286	0	1	0	2
0	0	0	132,857	51,4286	25,7143	1	1	2
0	0	0	64,2857	12,8571	0	1	0	3
0	0	0	102,857	8,57143	0	1	0	3
0	0	1	0	112,857	0	0	1	2
0	0	0	4,28571	0	0	0	0	1
0	0	0	42,8571	145,714	0	0	1	2
0	0	0	34,2857	154,286	0	0	1	2
0	0	0	42,8571	30	17,1429	0	0	2
0	0	0	40	0	154,286	0	1	1
0	0	0	38,5714	87,8571	25,7143	0	1	2
0	0	0	15	0	17,1429	0	0	2

0	0	0	8,57143	79,2857	0	0	1	1
0	0	0	180	60	0	1	1	1
0	0	1	25,7143	70,7143	0	0	1	1
0	1	1	214,286	85,7143	0	1	1	2
0	0	1	8,57143	90	0	0	1	2
0	0	0	55,7143	12,8571	0	0	0	1
0	0	0	28,5714	138,571	0	0	1	2
0	0	0	0	154,286	0	0	1	3
0	0	0	151,429	132,857	0	1	1	2
0	0	0	51,4286	8,57143	0	0	0	2
0	0	0	57,1429	20	0	0	0	1
0	0	0	8,57143	42,8571	0	0	0	2
0	0	0	0	128,571	154,286	0	1	2
0	0	0	25,7143	41,4286	0	0	0	2
0	0	0	51,4286	171,429	0	0	1	2
0	0	0	8,57143	25,7143	102,857	0	1	2
0	0	0	180	17,1429	0	1	0	2
0	0	0	42,8571	120	0	0	1	2
0	0	0	0	154,286	51,4286	0	1	2
0	0	0	8,57143	68,5714	0	0	1	1
0	0	0	38,5714	8,57143	0	0	0	2
0	0	1	72,8571	68,5714	0	1	1	2
0	0	0	8,57143	68,5714	34,2857	0	1	3
0	1	1	150	71,4286	0	1	1	1
0	0	0	55,7143	25,7143	0	0	0	2
0	0	0	51,4286	0	0	0	0	1
0	0	0	57,1429	240	0	0	1	2
0	0	0	28,2857	4,28571	0	0	0	2
0	1	1	67,1429	34,2857	0	1	0	2
0	0	0	12,8571	5,71429	0	0	0	2
0	0	0	17,1429	17,1429	0	0	0	2
0	0	0	8,57143	0	0	0	0	2
0	0	0	5,71429	0	0	0	0	2
0	0	0	81,4286	53,5714	0	1	0	2
0	0	0	15,7143	20	4,28571	0	0	1
0	1	1	60	8,57143	0	1	0	2
0	0	0	21,4286	34,2857	0	0	0	2
0	0	0	42,8571	85,7143	0	0	1	2
0	0	0	0	128,571	85,7143	0	1	3
0	1	1	102,857	30	8,57143	1	0	2
0	1	1	85,7143	0	0	1	0	2
0	0	0	2,85714	2,85714	0	0	0	2
0	0	0	0	60	0	0	1	2
0	0	0	28,5714	4,28571	0	0	0	2
0	0	0	120	0	0	1	0	2
0	0	0	0	51,4286	0	0	0	3
0	0	0	31,4286	42,8571	21,4286	0	1	2
0	0	0	94,2857	0	60	1	1	2

0	0	0	17,1429	0	42,8571	0	0	2
0	0	0	21,4286	60	0	0	1	1
0	1	1	130,714	12,8571	0	1	0	2
0	0	0	63,4286	44,2857	0	1	0	2
0	0	0	60	34,2857	0	1	0	2
0	0	0	21,4286	55,7143	0	0	0	1
0	0	0	34,2857	34,2857	0	0	0	2
0	0	0	38,5714	154,286	0	0	1	2
0	1	1	211,429	34,2857	0	1	0	2
0	0	0	0	8,57143	0	0	0	2
0	1	1	60	60	0	1	1	3
0	0	0	0	39,2857	0	0	0	3
0	0	0	34,2857	51,4286	0	0	0	1
0	0	0	8,57143	17,1429	0	0	0	2
0	0	0	10,7143	34,2857	0	0	0	2
0	0	0	0	21,4286	0	0	0	2
0	1	1	102,857	0	0	1	0	1
0	1	1	117,143	34,2857	51,4286	1	1	2
0	0	0	20	55,7143	0	0	0	2
0	0	0	64,2857	21,4286	0	1	0	2
0	0	0	42,8571	14,2857	25,7143	0	0	2
0	0	0	8,57143	0	0	0	0	2
0	0	1	77,1429	60	0	1	1	2
0	0	0	28,5714	0	0	0	0	2
0	0	0	21,4286	0	0	0	0	2
0	0	0	21,4286	60	0	0	1	1
0	0	0	17,8571	34,2857	0	0	0	2
0	0	0	34,2857	34,2857	0	0	0	2
0	0	0	81,4286	0	0	1	0	2
0	0	0	42,8571	17,1429	0	0	0	1
0	0	0	0	169,286	0	0	1	2
0	0	0	51,4286	42,8571	0	0	0	1
0	0	0	42,8571	0	60	0	1	2
0	0	0	35,7143	21,4286	0	0	0	2
0	0	0	21,4286	17,1429	0	0	0	2
0	0	0	14,2857	8,57143	0	0	0	2
0	0	0	28,5714	18,5714	0	0	0	1
0	0	0	34,2857	17,1429	0	0	0	1
0	0	0	17,1429	27,8571	17,1429	0	0	1
0	0	0	71,4286	0	0	1	0	2
0	0	0	17,1429	51,4286	34,2857	0	1	1
0	0	0	0	0	8,57143	0	0	2
0	0	0	34,2857	34,2857	0	0	0	1
1	0	1	0	174,286	77,1429	0	1	2
0	0	0	34,2857	40,7143	0	0	0	2
0	0	0	0	0	5,71429	0	0	2
0	0	0	128,571	145,714	0	1	1	2
0	0	0	30	5,71429	0	0	0	2

0	0	0	95,7143	34,2857	8,57143	1	0	2
0	0	0	0	188,571	102,857	0	1	2
0	0	0	34,2857	25,7143	0	0	0	2
0	0	0	34,2857	60	17,1429	0	1	1
0	0	0	8,57143	0	0	0	0	2
0	1	1	141,429	0	0	1	0	2
0	0	0	111,429	308,571	0	1	1	1
0	0	0	25,7143	110	77,1429	0	1	2
0	0	0	0	8,57143	0	0	0	2
0	0	0	85,7143	64,2857	0	1	1	2
0	0	0	0	0	0	0	0	2
0	0	0	17,1429	0	0	0	0	2
0	0	0	10	81,4286	0	0	1	2
0	0	0	8,57143	0	0	0	0	2
0	0	0	73,5714	0	0	1	0	1
0	0	0	8,57143	17,1429	8,57143	0	0	1
0	0	0	4,28571	0	0	0	0	2
0	1	1	60	60	0	1	1	3
0	0	0	75	53,5714	8,57143	1	1	2
0	1	1	102,857	60	60	1	1	2
0	0	0	0	167,143	0	0	1	2
0	0	0	64,2857	42,8571	0	1	0	3
0	0	0	17,1429	0	0	0	0	3
0	0	0	0	208,571	128,571	0	1	2
0	0	1	25,7143	180	180	0	1	2
0	0	0	0	8,57143	0	0	0	2
0	0	0	47,1429	0	0	0	0	3
0	0	0	77,1429	11,4286	0	1	0	1
0	0	0	2,85714	2,85714	0	0	0	1
0	0	0	21,4286	145,714	12,8571	0	1	3
0	0	0	15	28,5714	0	0	0	2
0	0	0	14,2857	0	0	0	0	2
0	0	1	171,429	25,7143	8,57143	1	0	3
0	0	0	57,1429	28,5714	17,1429	0	0	2
0	0	0	27,1429	85,7143	0	0	1	2
0	0	0	25,7143	42,8571	0	0	0	1
0	0	0	42,8571	8,57143	0	0	0	2
0	0	0	11,4286	68,5714	0	0	1	1
0	0	0	0	34,2857	0	0	0	2
0	0	0	15	11,4286	25,7143	0	0	2
0	0	0	40,7143	17,1429	0	0	0	2
0	0	0	0	51,4286	51,4286	0	1	2
0	0	0	77,1429	0	0	1	0	2
0	0	0	42,8571	0	34,2857	0	0	2
0	0	0	51,4286	138,571	0	0	1	2
0	0	0	45	0	0	0	0	3
0	0	0	12,8571	8,57143	0	0	0	2
0	0	0	137,143	154,286	0	1	1	1

0	*1*	*1*	*74,2857*	*8,57143*	*0*	*1*	*0*	*2*
0	*0*	*0*	*8,57143*	*0*	*0*	*0*	*0*	*2*
0	*0*	*0*	*21,4286*	*0*	*0*	*0*	*0*	*2*
0	*0*	*0*	*42,8571*	*171,429*	*68,5714*	*0*	*1*	*2*
0	*0*	*0*	*2,14286*	*85,7143*	*25,7143*	*0*	*1*	*1*
0	*0*	*0*	*20*	*180*	*0*	*0*	*1*	*2*
0	*1*	*1*	*75,7143*	*8,57143*	*0*	*1*	*0*	*2*
0	*0*	*0*	*60*	*190*	*154,286*	*1*	*1*	*2*
0	*1*	*1*	*111,429*	*102,857*	*0*	*1*	*1*	*2*
0	*0*	*0*	*12,8571*	*17,1429*	*0*	*0*	*0*	*2*
0	*1*	*1*	*368,571*	*17,1429*	*0*	*1*	*0*	*2*
0	*0*	*0*	*85,7143*	*51,4286*	*0*	*1*	*0*	*2*
0	*0*	*0*	*8,57143*	*2,14286*	*0*	*0*	*0*	*3*
0	*0*	*0*	*60*	*30*	*0*	*1*	*0*	*2*
0	*1*	*1*	*180*	*0*	*17,1429*	*1*	*0*	*2*
0	*0*	*0*	*21,4286*	*25,7143*	*0*	*0*	*0*	*3*
0	*0*	*0*	*120*	*8,57143*	*0*	*1*	*0*	*2*
0	*0*	*0*	*94,2857*	*128,571*	*77,1429*	*1*	*1*	*2*
0	*0*	*0*	*5,71429*	*0*	*0*	*0*	*0*	*2*
0	*0*	*0*	*0*	*5,71429*	*51,4286*	*0*	*0*	*2*
0	*0*	*1*	*102,857*	*60*	*0*	*1*	*1*	*2*
0	*0*	*0*	*42,8571*	*51,4286*	*0*	*0*	*0*	*2*
0	*0*	*0*	*17,1429*	*25,7143*	*68,5714*	*0*	*1*	*2*
0	*0*	*0*	*11,4286*	*17,1429*	*0*	*0*	*0*	*2*
0	*0*	*0*	*37,1429*	*51,4286*	*0*	*0*	*0*	*2*
0	*0*	*0*	*28,5714*	*0*	*0*	*0*	*0*	*1*
0	*0*	*0*	*19,2857*	*77,1429*	*0*	*0*	*1*	*2*
0	*1*	*1*	*180*	*102,857*	*0*	*1*	*1*	*1*
0	*0*	*0*	*30*	*171,429*	*102,857*	*0*	*1*	*2*
0	*0*	*0*	*36,4286*	*4,28571*	*0*	*0*	*0*	*1*
0	*0*	*0*	*0*	*77,1429*	*25,7143*	*0*	*1*	*3*
0	*0*	*0*	*0*	*70,7143*	*51,4286*	*0*	*1*	*1*
0	*0*	*0*	*25,7143*	*60*	*0*	*0*	*1*	*2*
0	*0*	*0*	*20*	*5,71429*	*68,5714*	*0*	*1*	*2*
0	*0*	*0*	*51,4286*	*102,857*	*0*	*0*	*1*	*2*
0	*0*	*0*	*51,4286*	*0*	*0*	*0*	*0*	*2*
1	*0*	*1*	*21,4286*	*25,7143*	*64,2857*	*0*	*1*	*1*
0	*0*	*0*	*32,1429*	*117,857*	*0*	*0*	*1*	*3*
0	*0*	*0*	*0*	*85,7143*	*34,2857*	*0*	*1*	*2*
0	*0*	*0*	*25,7143*	*77,1429*	*0*	*0*	*1*	*2*
0	*0*	*0*	*77,1429*	*68,5714*	*0*	*1*	*1*	*2*
0	*0*	*0*	*42,8571*	*17,1429*	*0*	*0*	*0*	*2*
0	*0*	*0*	*0*	*0*	*0*	*0*	*0*	*2*
0	*0*	*0*	*8,57143*	*40*	*0*	*0*	*0*	*3*
0	*0*	*0*	*64,2857*	*34,2857*	*0*	*1*	*0*	*1*
0	*0*	*0*	*188,571*	*34,2857*	*0*	*1*	*0*	*2*
0	*0*	*0*	*45*	*17,1429*	*42,8571*	*0*	*1*	*2*
0	*0*	*0*	*53,5714*	*0*	*4,28571*	*0*	*0*	*1*

0	0	0	0	0	0	0	0	2
0	0	0	60	0	0	1	0	3
0	0	0	51,4286	34,2857	0	0	0	1
0	0	0	150	47,1429	0	1	0	2
0	0	0	34,2857	51,4286	0	0	0	1
0	1	1	128,571	0	0	1	0	1
0	0	0	0	65,7143	0	0	1	2
0	0	0	4,28571	17,1429	0	0	0	2
0	0	0	17,1429	19,2857	0	0	0	2
0	0	1	282,857	214,286	25,7143	1	1	1
0	0	0	17,8571	68,5714	0	0	1	2
0	1	1	80	68,5714	0	1	1	2
0	0	0	48,5714	34,2857	0	0	0	2
0	0	0	30	0	0	0	0	1
0	0	1	51,4286	77,1429	0	0	1	1
0	0	0	222,857	17,1429	0	1	0	1
0	0	0	30	128,571	17,1429	0	1	2
0	0	0	30	0	154,286	0	1	2
0	0	0	0	0	102,857	0	1	2
0	1	1	107,143	8,57143	34,2857	1	0	1
0	1	1	60	60	0	1	1	3
0	0	0	17,1429	51,4286	0	0	0	2
0	0	0	0	102,857	0	0	1	2
0	0	0	85,7143	17,1429	0	1	0	1
0	0	0	65,7143	77,1429	0	1	1	2
0	0	0	0	42,8571	0	0	0	2
0	1	1	110	4,28571	60	1	1	3
0	0	0	128,571	17,1429	0	1	0	2
0	0	0	0	154,286	154,286	0	1	2
0	0	0	47,1429	42,8571	25,7143	0	1	2
0	1	1	162,857	102,857	51,4286	1	1	2
0	0	0	32,1429	154,286	5,71429	0	1	3
0	0	0	8,57143	111,429	77,1429	0	1	2
0	1	1	120	12,8571	0	1	0	2
0	0	0	51,4286	30	0	0	0	2
0	0	0	168,571	72,8571	0	1	1	2
0	0	0	7,14286	25,7143	0	0	0	2
0	0	0	42,8571	15	0	0	0	2
0	0	0	171,429	120	102,857	1	1	1
0	0	0	60	60	0	1	1	3
0	0	0	21,4286	120	0	0	1	2
0	0	0	102,857	68,5714	0	1	1	2
0	0	0	102,857	8,57143	0	1	0	3
0	0	0	72,8571	8,57143	0	1	0	2
0	0	0	20	30	0	0	0	3
0	0	0	24,2857	17,1429	0	0	0	2
1	1	1	145,714	300	0	1	1	1
0	0	1	248,571	25,7143	25,7143	1	0	2

0	0	0	51,4286	51,4286	0	0	0	2
0	1	1	120	0	0	1	0	1
1	0	1	171,429	98,5714	90	1	1	2
0	0	1	188,571	64,2857	0	1	1	1
0	0	0	45,7143	109,286	0	0	1	1
0	0	0	42,8571	15,7143	0	0	0	1
0	0	0	21,4286	0	0	0	0	2
0	0	0	25,7143	184,286	0	0	1	2
0	0	0	34,2857	51,4286	17,1429	0	1	2
0	0	0	80	0	15	1	0	2
0	0	0	0	4,28571	0	0	0	2
0	0	0	20	51,4286	85,7143	0	1	2
0	0	0	0	154,286	85,7143	0	1	2
0	0	0	8,57143	25,7143	8,57143	0	0	2
0	0	0	21,4286	68,5714	85,7143	0	1	2
0	0	0	60	197,143	154,286	1	1	2
0	0	0	72,8571	128,571	0	1	1	2
0	0	0	14,2857	55,7143	0	0	0	2
0	0	0	21,4286	90	45,7143	0	1	2
0	0	0	35,7143	257,143	0	0	1	3
0	0	0	0	154,286	102,857	0	1	3
0	0	0	42,8571	0	0	0	0	1
0	0	0	37,1429	8,57143	0	0	0	2
0	0	0	20	21,4286	77,1429	0	1	2
0	0	0	0	167,143	0	0	1	3
0	0	0	25,7143	64,2857	0	0	1	2
0	0	0	66,4286	0	0	1	0	2
0	0	0	60	157,143	34,2857	1	1	2
0	0	0	17,1429	45,7143	0	0	0	1
0	0	0	7,14286	7,14286	0	0	0	2
0	0	0	48,5714	0	0	0	0	2
0	1	1	137,143	4,28571	25,7143	1	0	1
0	0	0	0	0	0	0	0	1
0	0	0	30	7,85714	25,7143	0	0	2
0	0	0	30	4,28571	15,7143	0	0	1
0	0	0	145,714	68,5714	0	1	1	2
0	0	0	8,57143	205,714	77,1429	0	1	2
0	0	0	94,2857	25,7143	34,2857	1	1	2
0	0	0	38,5714	55,7143	8,57143	0	1	2
0	0	1	81,4286	93,5714	0	1	1	2
0	1	1	122,143	30	0	1	0	1
0	0	0	45	205,714	51,4286	0	1	2
0	1	1	80	20	77,1429	1	1	2
0	0	0	0	0	0	0	0	1
0	0	0	21,4286	40	0	0	0	2
0	0	0	40	34,2857	0	0	0	1
0	0	0	4,28571	192,857	0	0	1	2
0	0	0	90	17,1429	0	1	0	1

0	0	0	8,57143	34,2857	0	0	0	2
0	0	0	5,71429	0	94,2857	0	1	2
0	0	0	21,4286	162,857	0	0	1	1
0	0	0	30	115,714	0	0	1	1
0	0	0	25	0	0	0	0	2
0	0	0	22,1429	0	0	0	0	2
0	0	0	8,57143	8,57143	0	0	0	3
0	0	0	68,5714	51,4286	0	1	0	2
0	0	0	12,8571	0	25,7143	0	0	2
0	0	0	0	25,7143	0	0	0	2
0	0	0	50	4,28571	0	0	0	2
0	0	0	169,286	34,2857	17,1429	1	0	2
0	0	1	34,2857	30	0	0	0	2
0	0	0	5,71429	0	0	0	0	2
0	0	0	12,8571	154,286	0	0	1	1
0	0	0	12,8571	55,7143	0	0	0	1
1	0	1	11,4286	94,2857	154,286	0	1	2
0	1	1	111,429	4,28571	0	1	0	2
0	0	0	38,5714	0	0	0	0	2
0	1	1	60	51,4286	0	1	0	2
0	0	0	27,8571	0	0	0	0	2
0	0	0	0	38,5714	0	0	0	2
0	0	0	0	0	10	0	0	2
0	0	0	4,28571	0	51,4286	0	0	2
0	0	0	0	141,429	0	0	1	2
0	0	0	8,57143	60	0	0	1	2
0	1	1	120	180	0	1	1	2
0	0	0	154,286	34,2857	17,1429	1	0	1
0	0	0	27,1429	34,2857	0	0	0	2
0	0	0	34,2857	0	6,42857	0	0	2
0	0	0	34,2857	8,57143	0	0	0	2
0	0	0	60	8,57143	0	1	0	1
0	1	1	120	0	25,7143	1	0	2
0	1	1	90	17,1429	0	1	0	1
0	1	1	189,143	102,857	0	1	1	1
0	0	0	21,4286	17,1429	0	0	0	2
0	0	0	145,714	17,1429	15	1	0	2
0	0	0	0	0	8,57143	0	0	1
0	0	0	17,1429	0	0	0	0	2
0	0	0	30	30	0	0	0	2
0	0	0	45,7143	21,4286	0	0	0	2
0	0	0	30	47,1429	128,571	0	1	2
0	0	0	17,1429	0	0	0	0	2
0	0	0	6,42857	105,714	0	0	1	2
0	0	1	65,7143	0	17,1429	1	0	1
1	1	1	240	268,571	334,286	1	1	1
0	0	0	37,1429	8,57143	51,4286	0	1	2
0	0	0	22,8571	17,1429	0	0	0	2

0	*0*	*0*	*17,1429*	*5,71429*	*0*	*0*	*0*	*2*
0	*0*	*0*	*25,7143*	*25,7143*	*0*	*0*	*0*	*1*
0	*0*	*0*	*68,5714*	*182,143*	*180*	*1*	*1*	*1*
0	*0*	*0*	*60*	*44,2857*	*17,1429*	*1*	*1*	*2*
0	*0*	*0*	*0*	*78,5714*	*0*	*0*	*1*	*2*
0	*0*	*0*	*37,1429*	*51,4286*	*0*	*0*	*0*	*2*
0	*0*	*1*	*70*	*8,57143*	*51,4286*	*1*	*1*	*2*
0	*0*	*0*	*72,8571*	*5,71429*	*0*	*1*	*0*	*2*
0	*0*	*0*	*30*	*100,714*	*0*	*0*	*1*	*2*
0	*1*	*1*	*70*	*12,8571*	*51,4286*	*1*	*1*	*2*
0	*1*	*1*	*110*	*0*	*0*	*1*	*0*	*2*
0	*0*	*1*	*42,8571*	*94,2857*	*0*	*0*	*1*	*3*
0	*1*	*1*	*162,857*	*0*	*0*	*1*	*0*	*2*
0	*0*	*0*	*78,5714*	*38,5714*	*0*	*1*	*0*	*2*
0	*0*	*0*	*0*	*61,4286*	*0*	*0*	*1*	*3*
0	*1*	*1*	*187,143*	*240*	*0*	*1*	*1*	*1*
0	*0*	*0*	*12,8571*	*17,1429*	*0*	*0*	*0*	*2*
0	*0*	*0*	*8,57143*	*300*	*0*	*0*	*1*	*1*
0	*0*	*0*	*38,5714*	*0*	*0*	*0*	*0*	*2*
0	*0*	*0*	*27,1429*	*54,2857*	*0*	*0*	*0*	*3*
0	*0*	*0*	*10*	*55,7143*	*0*	*0*	*0*	*2*
0	*0*	*0*	*51,4286*	*34,2857*	*77,1429*	*0*	*1*	*2*
0	*1*	*1*	*120*	*194,286*	*5,71429*	*1*	*1*	*1*
0	*0*	*0*	*51,4286*	*51,4286*	*0*	*0*	*0*	*2*
0	*0*	*0*	*20*	*0*	*11,4286*	*0*	*0*	*2*
0	*1*	*1*	*120*	*17,1429*	*0*	*1*	*0*	*1*
0	*0*	*0*	*30*	*11*	*0*	*0*	*0*	*3*
0	*0*	*0*	*90*	*36,4286*	*0*	*1*	*0*	*2*
0	*1*	*1*	*77,1429*	*2,85714*	*25,7143*	*1*	*0*	*2*
0	*0*	*0*	*68,5714*	*25,7143*	*0*	*1*	*0*	*2*
0	*0*	*0*	*5,71429*	*51,4286*	*128,571*	*0*	*1*	*2*
0	*1*	*1*	*205,714*	*306,429*	*135*	*1*	*1*	*2*
0	*0*	*0*	*14,2857*	*30*	*0*	*0*	*0*	*2*
0	*0*	*0*	*8,57143*	*74,2857*	*0*	*0*	*1*	*2*
0	*0*	*0*	*1,42857*	*205,714*	*0*	*0*	*1*	*2*
0	*1*	*1*	*222,857*	*34,2857*	*0*	*1*	*0*	*2*
0	*0*	*0*	*42,8571*	*51,4286*	*51,4286*	*0*	*1*	*2*
0	*0*	*1*	*66,4286*	*25,7143*	*45*	*1*	*1*	*1*
0	*0*	*0*	*12,8571*	*51,4286*	*0*	*0*	*0*	*2*
0	*1*	*1*	*141,429*	*12,8571*	*0*	*1*	*0*	*1*
0	*0*	*1*	*34,2857*	*165*	*0*	*0*	*1*	*3*
1	*1*	*1*	*102,857*	*47,1429*	*120*	*1*	*1*	*1*
0	*0*	*0*	*51,4286*	*128,571*	*0*	*0*	*1*	*2*
0	*0*	*0*	*51,4286*	*0*	*0*	*0*	*0*	*2*
0	*0*	*0*	*0*	*34,2857*	*0*	*0*	*0*	*1*
0	*0*	*0*	*96,4286*	*0*	*0*	*1*	*0*	*2*
0	*0*	*1*	*34,2857*	*64,2857*	*0*	*0*	*1*	*2*
0	*0*	*0*	*60*	*137,143*	*0*	*1*	*1*	*1*

0	0	0	64,2857	12,8571	0	1	0	2
0	0	0	51,4286	0	0	0	0	2
0	0	0	55,7143	47,1429	0	0	0	2
0	0	0	34,2857	17,1429	0	0	0	2
0	1	1	124,286	55,7143	0	1	0	2
0	1	1	60	77,1429	25,7143	1	1	1
0	0	0	0	55,7143	0	0	0	2
0	0	0	152,857	85,7143	8,57143	1	1	1
0	0	0	140	77,1429	0	1	1	1
0	0	0	17,1429	0	0	0	0	3
0	0	0	11,4286	130,714	0	0	1	2
0	0	0	51,4286	85,7143	0	0	1	2
0	0	0	21,4286	0	0	0	0	2
0	0	0	50	68,5714	0	0	1	2
0	0	0	47,1429	2,85714	0	0	0	1
0	0	0	0	77,1429	180	0	1	2
0	0	0	4,28571	8,57143	0	0	0	2
0	0	0	90	8,57143	0	1	0	3
0	1	1	111,429	24,2857	0	1	0	2
0	0	0	85,7143	85,7143	0	1	1	2
0	0	0	64,2857	24,2857	0	1	0	1
0	0	0	143,571	81,4286	17,1429	1	1	2
0	0	0	6,42857	34,2857	34,2857	0	1	2
0	1	1	120	111,429	120	1	1	2
0	0	0	42,8571	34,2857	0	0	0	1
0	0	0	28,5714	17,1429	0	0	0	2
0	0	0	28,5714	35,7143	0	0	0	1
0	0	0	10,7143	68,5714	15	0	1	1
0	0	0	68,5714	24,2857	0	1	0	2
0	0	0	30	94,2857	38,5714	0	1	1
0	0	0	30	34,2857	0	0	0	2
0	0	0	55,7143	25,7143	0	0	0	2
0	0	0	28,5714	0	0	0	0	3
0	0	0	162,857	218,571	51,4286	1	1	2
0	0	0	171,429	118,571	0	1	1	2
0	0	0	2,85714	64,2857	0	0	1	2
0	0	0	235,714	137,143	25,7143	1	1	2
0	0	0	188,571	162,857	85,7143	1	1	2
0	0	0	20	0	17,1429	0	0	2
0	0	0	0	0	17,1429	0	0	2
0	0	0	27,1429	55,7143	0	0	0	1
0	1	1	65,7143	1,42857	0	1	0	1
0	0	0	10	64,2857	0	0	1	3
0	0	0	45	4,28571	0	0	0	2
0	0	0	17,1429	0	0	0	0	2
0	0	0	42,8571	34,2857	0	0	0	1
0	0	0	24,2857	7,14286	0	0	0	2
0	0	0	7,14286	0	0	0	0	1

0	0	0	0	51,4286	0	0	0	2
0	0	0	28,5714	0	0	0	0	1
0	0	0	52,8571	0	0	0	0	2
0	1	1	70	180	180	1	1	2
0	0	0	51,4286	8,57143	6,42857	0	0	2
0	0	0	45	17,1429	0	0	0	2
0	0	0	11,4286	8,57143	0	0	0	2
0	0	0	28,5714	0	0	0	0	2
0	0	0	17,1429	20	0	0	0	2
0	0	0	130,714	30	25,7143	1	0	1
0	0	0	51,4286	72,8571	0	0	1	3
0	0	0	30	25,7143	34,2857	0	1	2
0	0	0	17,1429	8,57143	0	0	0	1
0	0	0	72,8571	25,7143	0	1	0	2
0	0	0	71,4286	162,857	0	1	1	2
0	0	0	197,143	57,8571	0	1	0	2
0	0	0	60	85,7143	51,4286	1	1	2
0	0	0	49,2857	20	0	0	0	2
0	1	1	120	154,286	0	1	1	3
0	0	0	12,8571	0	0	0	0	2
0	0	0	0	15	0	0	0	2
0	0	0	0	124,286	77,1429	0	1	3
0	0	0	38,5714	128,571	51,4286	0	1	2
0	0	0	21,4286	0	51,4286	0	0	2
1	1	1	120	285,714	120	1	1	2
0	0	0	231,429	197,143	51,4286	1	1	2
0	0	0	71,4286	34,2857	34,2857	1	1	2
0	1	1	145,714	18,5714	0	1	0	1
0	0	0	51,4286	17,1429	6,42857	0	0	2
0	0	0	34,2857	34,2857	0	0	0	2
0	0	0	45,7143	188,571	68,5714	0	1	2
0	1	1	145,714	51,4286	0	1	0	2
0	0	0	68,5714	168,571	0	1	1	2
0	0	0	18,5714	12,8571	0	0	0	3
0	0	0	18,5714	17,1429	17,1429	0	0	2
0	0	0	25,7143	17,1429	68,5714	0	1	2
0	1	1	360	34,2857	0	1	0	1
0	0	0	51,4286	21,4286	0	0	0	2
0	0	0	25,7143	68,5714	0	0	1	3
0	0	0	218,571	82,8571	17,1429	1	1	2
0	0	0	62,1429	12,8571	0	1	0	2
1	1	1	154,286	120	0	1	1	3
0	0	0	197,143	17,1429	6,42857	1	0	2
0	0	0	0	0	0	0	0	2
0	1	1	60	12,8571	88,5714	1	1	2
0	0	0	4,28571	17,1429	0	0	0	2
0	1	1	70	34,2857	0	1	0	1
0	1	1	268,571	122,143	0	1	1	2

0	*0*	*0*	*22,8571*	*111,429*	*0*	*0*	*1*	*2*
0	*0*	*0*	*27,8571*	*4,28571*	*0*	*0*	*0*	*2*
0	*0*	*0*	*178,571*	*0*	*0*	*1*	*0*	*1*
0	*0*	*0*	*30*	*4,28571*	*0*	*0*	*0*	*1*
0	*0*	*0*	*80*	*8,57143*	*0*	*1*	*0*	*1*
0	*0*	*0*	*34,2857*	*17,1429*	*0*	*0*	*0*	*3*
0	*0*	*0*	*25,7143*	*17,1429*	*0*	*0*	*0*	*2*
0	*0*	*0*	*175,714*	*145,714*	*0*	*1*	*1*	*2*
0	*0*	*0*	*30*	*17,1429*	*8,57143*	*0*	*0*	*2*
0	*0*	*0*	*54,2857*	*10*	*0*	*0*	*0*	*2*
0	*0*	*0*	*8,57143*	*10*	*0*	*0*	*0*	*2*
0	*0*	*0*	*34,2857*	*72,8571*	*0*	*0*	*1*	*2*
0	*0*	*0*	*0*	*25,7143*	*38,5714*	*0*	*1*	*2*
0	*0*	*0*	*128,571*	*18,5714*	*0*	*1*	*0*	*2*
0	*0*	*1*	*47,1429*	*77,8571*	*21,4286*	*0*	*1*	*2*
0	*0*	*0*	*77,1429*	*25,7143*	*0*	*1*	*0*	*1*
0	*0*	*0*	*71,4286*	*17,1429*	*0*	*1*	*0*	*2*
0	*0*	*0*	*81,4286*	*8,57143*	*0*	*1*	*0*	*2*
0	*0*	*0*	*51,4286*	*42,8571*	*0*	*0*	*0*	*2*
0	*0*	*0*	*60*	*182,857*	*0*	*1*	*1*	*2*
0	*0*	*0*	*148,571*	*55,7143*	*25,7143*	*1*	*1*	*1*
0	*0*	*0*	*0*	*540*	*197,143*	*0*	*1*	*2*
0	*0*	*0*	*0*	*51,4286*	*0*	*0*	*0*	*2*
0	*0*	*0*	*51,4286*	*25,7143*	*0*	*0*	*0*	*3*
0	*0*	*0*	*28,5714*	*0*	*0*	*0*	*0*	*2*
0	*0*	*0*	*51,4286*	*77,1429*	*0*	*0*	*1*	*2*
0	*1*	*1*	*274,286*	*0*	*17,1429*	*1*	*0*	*2*
0	*0*	*0*	*25,7143*	*4,28571*	*0*	*0*	*0*	*2*
0	*0*	*0*	*85,7143*	*4,28571*	*51,4286*	*1*	*0*	*2*
0	*0*	*0*	*5,71429*	*0*	*0*	*0*	*0*	*3*
0	*0*	*1*	*51,4286*	*60*	*68,5714*	*0*	*1*	*2*
0	*0*	*0*	*68,5714*	*34,2857*	*0*	*1*	*0*	*2*
0	*0*	*0*	*10,7143*	*0*	*12,8571*	*0*	*0*	*1*
0	*0*	*0*	*34,2857*	*4,28571*	*154,286*	*0*	*1*	*3*
0	*0*	*0*	*30*	*25,7143*	*120*	*0*	*1*	*1*
0	*0*	*0*	*0*	*8,57143*	*0*	*0*	*0*	*2*
0	*1*	*1*	*317,143*	*8,57143*	*0*	*1*	*0*	*1*
0	*0*	*0*	*17,1429*	*15*	*15*	*0*	*0*	*3*
0	*0*	*0*	*124,286*	*75*	*0*	*1*	*1*	*1*
0	*0*	*0*	*96,4286*	*0*	*0*	*1*	*0*	*2*
0	*0*	*0*	*34,2857*	*0*	*0*	*0*	*0*	*2*
0	*0*	*0*	*25,7143*	*205,714*	*102,857*	*0*	*1*	*1*
0	*0*	*1*	*51,4286*	*17,1429*	*0*	*0*	*0*	*3*
0	*0*	*0*	*42,8571*	*25,7143*	*0*	*0*	*0*	*2*
0	*0*	*0*	*12,8571*	*8,57143*	*8,57143*	*0*	*0*	*2*
0	*0*	*0*	*111,429*	*0*	*0*	*1*	*0*	*2*
0	*0*	*0*	*38,5714*	*124,286*	*77,1429*	*0*	*1*	*2*
0	*1*	*1*	*74,2857*	*8,57143*	*0*	*1*	*0*	*2*

0	0	0	19,2857	4,28571	0	0	0	2
0	0	0	81,4286	36,4286	0	1	0	1
0	0	0	45,7143	51,4286	0	0	0	2
0	0	0	25,7143	14,2857	0	0	0	2
0	0	0	47,1429	68,5714	0	0	1	2
0	0	0	34,2857	154,286	154,286	0	1	2
0	0	0	0	38,5714	0	0	0	2
0	0	0	65,7143	102,857	154,286	1	1	2
0	0	0	30	0	0	0	0	1
0	0	0	12,8571	157,143	0	0	1	2
0	0	0	42,8571	167,143	0	0	1	2
0	0	0	270	25,7143	0	1	0	2
1	1	1	120	25,7143	51,4286	1	1	2
0	0	0	4,28571	23,5714	0	0	0	2
0	0	0	8,57143	0	154,286	0	1	2
0	0	0	158,571	128,571	0	1	1	2
0	0	0	47,8571	53,5714	4,28571	0	0	1
0	0	0	41,4286	10	6,42857	0	0	2
0	0	0	30	188,571	77,1429	0	1	1
0	0	0	6,42857	12,8571	0	0	0	2
0	0	0	68,5714	85,7143	51,4286	1	1	2
0	0	0	28,5714	51,4286	0	0	0	2
0	0	0	150	150	12,8571	1	1	2
0	0	0	94,2857	134,286	128,571	1	1	2
0	0	1	57,1429	77,1429	30	0	1	2
0	0	0	0	2,85714	120	0	1	1
0	0	0	81,4286	145,714	0	1	1	3
0	0	0	25,7143	20	0	0	0	2
0	0	0	0	40	0	0	0	2
0	0	0	30	0	0	0	0	2
0	0	0	0	158,571	0	0	1	2
0	0	0	32,1429	17,1429	17,1429	0	0	2
0	0	0	55,7143	8,57143	0	0	0	1
0	0	0	10,7143	6,42857	0	0	0	3
0	0	0	11,4286	8,57143	0	0	0	2
0	0	0	91,4286	0	0	1	0	2
0	1	1	308,571	27,8571	0	1	0	1
0	0	0	0	22,8571	12,8571	0	0	1
0	0	0	52,1429	51,4286	0	0	0	1
0	0	0	60	11,4286	51,4286	1	1	2
0	1	1	132,857	71,4286	0	1	1	2
0	0	0	85,7143	12,8571	0	1	0	2
0	1	1	71,4286	30	0	1	0	3
0	0	0	0	32,8571	0	0	0	3
0	0	0	17,1429	139,286	0	0	1	2
0	0	0	20,7143	0	0	0	0	1
0	0	0	60	231,429	25,7143	1	1	1
0	1	1	68,5714	0	25,7143	1	0	2

0	0	0	51,4286	154,286	0	0	1	2
0	0	0	0	180	0	0	1	2
0	0	0	51,4286	77,1429	25,7143	0	1	2
0	0	0	58,5714	17,1429	0	0	0	1
0	0	0	0	8,57143	0	0	0	2
0	1	1	60	25,7143	0	1	0	2
0	0	0	4,28571	25,7143	0	0	0	1
0	0	0	162,857	102,857	25,7143	1	1	2
0	0	0	0	17,1429	51,4286	0	1	1
0	0	0	60	64,2857	0	1	1	1
0	0	0	25,7143	0	0	0	0	2
0	0	0	8,57143	21,4286	17,1429	0	0	2
0	1	1	90	42,8571	60	1	1	2
0	0	0	38,5714	0	0	0	0	2
0	0	0	85,7143	111,429	51,4286	1	1	1
0	0	0	0	180	0	0	1	1
0	0	0	42,8571	20	0	0	0	2
0	0	0	45,7143	34,2857	0	0	0	2
0	0	0	21,4286	102,857	0	0	1	1
0	0	0	144,286	17,1429	0	1	0	2
0	0	0	34,2857	0	25,7143	0	0	2
0	0	0	111,429	17,1429	0	1	0	2
0	0	0	12,8571	0	0	0	0	2
0	1	1	102,857	6,42857	0	1	0	2
0	0	0	0	0	0	0	0	1
0	0	0	55,7143	0	0	0	0	1
0	0	0	0	8,57143	0	0	0	2
0	0	0	135,714	55,7143	0	1	0	2
0	1	1	60	23,5714	47,1429	1	1	3
0	0	0	11,4286	8,57143	0	0	0	1
0	0	0	30	0	0	0	0	2
0	0	0	25,7143	8,57143	25,7143	0	0	3
0	0	0	22,8571	6,42857	0	0	0	2
0	0	0	25,7143	0	0	0	0	2
0	0	0	52,8571	0	0	0	0	1
0	0	0	51,4286	102,857	0	0	1	1
0	0	0	137,143	36,4286	0	1	0	2
0	0	0	57,8571	30	47,1429	0	1	3
0	0	0	51,4286	25,7143	0	0	0	2
0	1	1	180	197,143	0	1	1	2
0	0	0	27,8571	25,7143	0	0	0	3
0	0	0	77,1429	0	0	1	0	1
0	1	1	188,571	74,2857	0	1	1	2
0	0	0	20	0	0	0	0	2
0	0	0	0	22,8571	0	0	0	1
0	0	0	40	8,57143	0	0	0	2
0	0	0	100	0	0	1	0	1
0	0	0	6,42857	60	154,286	0	1	2

0	*0*	*0*	*51,4286*	*0*	*5,71429*	*0*	*0*	*1*
0	*0*	*0*	*30*	*0*	*0*	*0*	*0*	*3*
0	*0*	*0*	*248,571*	*111,429*	*51,4286*	*1*	*1*	*2*
0	*0*	*0*	*0*	*68,5714*	*102,857*	*0*	*1*	*2*
0	*0*	*0*	*17,1429*	*0*	*34,2857*	*0*	*0*	*2*
0	*0*	*0*	*5,71429*	*90*	*0*	*0*	*1*	*1*
0	*0*	*0*	*250,714*	*77,1429*	*0*	*1*	*1*	*2*
0	*0*	*0*	*85,7143*	*114,286*	*25,7143*	*1*	*1*	*2*
0	*0*	*0*	*75*	*38,5714*	*51,4286*	*1*	*1*	*2*
0	*1*	*1*	*77,1429*	*77,1429*	*0*	*1*	*1*	*2*
0	*0*	*0*	*42,8571*	*8,57143*	*17,1429*	*0*	*0*	*2*
0	*1*	*1*	*98,5714*	*71,4286*	*0*	*1*	*1*	*2*
0	*0*	*0*	*0*	*102,857*	*0*	*0*	*1*	*2*
0	*0*	*0*	*60*	*42,8571*	*8,57143*	*1*	*0*	*2*
0	*0*	*0*	*57,8571*	*32,1429*	*0*	*0*	*0*	*1*
0	*0*	*0*	*0*	*30*	*0*	*0*	*0*	*3*
0	*0*	*0*	*34,2857*	*20*	*34,2857*	*0*	*0*	*1*
0	*0*	*0*	*8,57143*	*17,1429*	*0*	*0*	*0*	*2*
0	*0*	*0*	*2,14286*	*0*	*51,4286*	*0*	*0*	*2*
0	*0*	*0*	*54,2857*	*34,2857*	*0*	*0*	*0*	*2*
0	*0*	*0*	*148,571*	*282,857*	*128,571*	*1*	*1*	*2*
0	*0*	*0*	*21,4286*	*34,2857*	*0*	*0*	*0*	*2*
0	*0*	*0*	*21,4286*	*265,714*	*0*	*0*	*1*	*2*
0	*1*	*1*	*120*	*0*	*0*	*1*	*0*	*1*
0	*0*	*0*	*34,2857*	*77,1429*	*34,2857*	*0*	*1*	*2*
0	*0*	*0*	*21,4286*	*42,8571*	*0*	*0*	*0*	*2*
0	*0*	*0*	*51,4286*	*59,2857*	*8,57143*	*0*	*1*	*2*
0	*0*	*0*	*150*	*17,1429*	*0*	*1*	*0*	*1*
0	*0*	*0*	*0*	*154,286*	*0*	*0*	*1*	*2*
0	*0*	*0*	*17,1429*	*0*	*0*	*0*	*0*	*2*
0	*0*	*0*	*60*	*265,714*	*25,7143*	*1*	*1*	*2*
0	*0*	*0*	*150*	*97,1429*	*25,7143*	*1*	*1*	*2*
0	*0*	*0*	*64,2857*	*0*	*0*	*1*	*0*	*2*
0	*0*	*0*	*10,7143*	*8,57143*	*0*	*0*	*0*	*2*
0	*0*	*0*	*156,429*	*158,571*	*0*	*1*	*1*	*2*
0	*0*	*0*	*0*	*21,4286*	*0*	*0*	*0*	*2*
0	*0*	*0*	*90*	*68,5714*	*0*	*1*	*1*	*2*
0	*1*	*1*	*100*	*120*	*8,57143*	*1*	*1*	*2*
0	*0*	*0*	*25,7143*	*8,57143*	*154,286*	*0*	*1*	*2*
0	*0*	*0*	*25,7143*	*32,1429*	*8,57143*	*0*	*0*	*2*
0	*0*	*0*	*8,57143*	*128,571*	*0*	*0*	*1*	*2*
0	*1*	*1*	*120*	*184,286*	*154,286*	*1*	*1*	*2*
0	*0*	*0*	*20*	*240*	*180*	*0*	*1*	*2*
0	*1*	*1*	*124,286*	*77,1429*	*51,4286*	*1*	*1*	*2*
0	*0*	*0*	*21,4286*	*17,1429*	*0*	*0*	*0*	*1*
0	*0*	*0*	*51,4286*	*68,5714*	*55,7143*	*0*	*1*	*2*
0	*0*	*0*	*60*	*102,857*	*0*	*1*	*1*	*2*
0	*0*	*0*	*60*	*68,5714*	*0*	*1*	*1*	*2*

0	*1*	*1*	*240*	*25,7143*	*0*	*1*	*0*	*1*
0	*0*	*0*	*0*	*25,7143*	*0*	*0*	*0*	*1*
0	*0*	*0*	*162,857*	*2,14286*	*0*	*1*	*0*	*2*
0	*0*	*0*	*17,1429*	*21,4286*	*17,1429*	*0*	*0*	*2*
0	*0*	*0*	*42,8571*	*30*	*154,286*	*0*	*1*	*2*
0	*0*	*0*	*0*	*7,14286*	*0*	*0*	*0*	*2*
0	*0*	*0*	*60*	*180*	*0*	*1*	*1*	*1*
0	*1*	*1*	*180*	*120*	*0*	*1*	*1*	*3*
0	*0*	*0*	*148,571*	*25,7143*	*0*	*1*	*0*	*2*
0	*0*	*0*	*34,2857*	*51,4286*	*0*	*0*	*0*	*1*
0	*1*	*1*	*110*	*8,57143*	*0*	*1*	*0*	*1*
0	*0*	*0*	*8,57143*	*150*	*51,4286*	*0*	*1*	*2*
0	*0*	*0*	*17,1429*	*23,5714*	*0*	*0*	*0*	*2*
0	*1*	*1*	*137,143*	*17,1429*	*0*	*1*	*0*	*2*
0	*1*	*1*	*67,1429*	*64,2857*	*0*	*1*	*1*	*2*
0	*0*	*0*	*0*	*34,2857*	*34,2857*	*0*	*1*	*2*
0	*0*	*0*	*28,5714*	*14,2857*	*8,57143*	*0*	*0*	*1*
0	*0*	*0*	*8,57143*	*25,7143*	*8,57143*	*0*	*0*	*2*
0	*0*	*0*	*0*	*0*	*0*	*0*	*0*	*2*
0	*0*	*0*	*140*	*141,429*	*17,1429*	*1*	*1*	*2*
0	*0*	*0*	*54,2857*	*128,571*	*0*	*0*	*1*	*2*
0	*0*	*0*	*51,4286*	*0*	*68,5714*	*0*	*1*	*3*
0	*0*	*0*	*47,1429*	*14,2857*	*0*	*0*	*0*	*2*
0	*0*	*0*	*34,2857*	*105,714*	*17,1429*	*0*	*1*	*1*
0	*0*	*0*	*4,28571*	*0*	*4,28571*	*0*	*0*	*2*
0	*0*	*0*	*0*	*90*	*0*	*0*	*1*	*2*
0	*0*	*0*	*8,57143*	*17,1429*	*0*	*0*	*0*	*2*
0	*0*	*0*	*60*	*8,57143*	*0*	*1*	*0*	*1*
0	*0*	*0*	*111,429*	*102,857*	*0*	*1*	*1*	*2*
0	*0*	*0*	*41,4286*	*8,57143*	*0*	*0*	*0*	*1*
0	*1*	*1*	*102,857*	*34,2857*	*180*	*1*	*1*	*2*
0	*0*	*0*	*21,4286*	*17,1429*	*0*	*0*	*0*	*2*
0	*0*	*0*	*60*	*49,2857*	*85,7143*	*1*	*1*	*2*
0	*1*	*1*	*68,5714*	*0*	*0*	*1*	*0*	*2*
0	*0*	*0*	*0*	*0*	*0*	*0*	*0*	*1*
0	*0*	*0*	*180*	*248,571*	*154,286*	*1*	*1*	*2*
0	*0*	*0*	*17,1429*	*34,2857*	*0*	*0*	*0*	*2*
0	*0*	*0*	*42,8571*	*17,1429*	*4,28571*	*0*	*0*	*1*
0	*0*	*0*	*0*	*262,857*	*0*	*0*	*1*	*1*
0	*0*	*0*	*28,5714*	*0*	*0*	*0*	*0*	*2*
0	*0*	*0*	*77,1429*	*214,286*	*0*	*1*	*1*	*2*
0	*1*	*1*	*85,7143*	*205,714*	*0*	*1*	*1*	*2*
0	*0*	*0*	*68,5714*	*222,857*	*60*	*1*	*1*	*2*
0	*0*	*0*	*25,7143*	*85,7143*	*0*	*0*	*1*	*2*
0	*0*	*0*	*0*	*21,4286*	*0*	*0*	*0*	*2*
0	*0*	*0*	*128,571*	*128,571*	*34,2857*	*1*	*1*	*2*
0	*0*	*0*	*0*	*85,7143*	*0*	*0*	*1*	*2*
0	*1*	*1*	*140*	*8,57143*	*0*	*1*	*0*	*2*

0	0	0	85,7143	120	0	1	1	3
0	0	0	72,8571	0	0	1	0	2
0	0	0	25,7143	51,4286	0	0	0	3
0	0	0	24,2857	25,7143	0	0	0	2
0	0	0	35,7143	157,714	0	0	1	2
0	0	0	14,2857	85,7143	0	0	1	2
0	0	0	25,7143	5,71429	0	0	0	2
0	0	0	0	192,857	0	0	1	2
0	0	0	37,1429	38,5714	60	0	1	3
0	0	0	171,429	17,1429	25,7143	1	0	2
0	1	1	360	51,4286	154,286	1	1	2
0	0	0	34,2857	10	102,857	0	1	2
0	0	0	42,8571	17,1429	0	0	0	2
0	0	0	0	0	34,2857	0	0	2
0	0	0	54,2857	11,4286	0	0	0	3
0	0	0	5	128,571	0	0	1	3
0	0	0	38,5714	34,2857	0	0	0	1
0	0	0	135	88,5714	8,57143	1	1	2
0	0	0	34,2857	8,57143	4,28571	0	0	2
0	0	0	0	214,286	0	0	1	2
0	0	0	34,2857	91,4286	0	0	1	2
0	0	0	64,2857	14,2857	0	1	0	2
0	1	1	77,1429	205,714	25,7143	1	1	1
0	0	0	60	47,1429	0	1	0	2
0	0	0	142,857	34,2857	0	1	0	2
0	0	0	7,14286	17,1429	0	0	0	2
0	0	0	137,143	42,8571	42,8571	1	1	2
0	0	0	0	17,1429	0	0	0	3
0	0	0	21,4286	8,57143	0	0	0	2
0	0	0	11,4286	28,5714	0	0	0	1
0	0	0	47,1429	34,2857	0	0	0	2
0	0	0	154,286	257,143	0	1	1	2
0	0	0	64,2857	180	0	1	1	1
0	1	1	210	8,57143	8,57143	1	0	2
0	1	1	68,5714	90	51,4286	1	1	1
0	0	0	51,4286	0	0	0	0	3
0	0	0	145,714	24,2857	0	1	0	2
0	0	0	10	4,28571	0	0	0	2
0	0	0	34,2857	17,1429	0	0	0	2
0	1	1	222,857	0	0	1	0	2
0	0	0	111,429	0	51,4286	1	0	2
0	0	1	55,7143	36,4286	0	0	0	2
0	0	0	155	254,286	0	1	1	1
0	0	0	51,4286	42,8571	25,7143	0	1	2
0	0	0	0	36,4286	25,7143	0	1	1
0	0	0	128,571	180	0	1	1	2
0	0	0	64,2857	51,4286	12,8571	1	1	1
0	0	0	42,8571	68,5714	0	0	1	1

0	0	0	77,1429	167,143	51,4286	1	1	2
0	0	0	42,8571	58,5714	2,85714	0	1	2
0	0	0	14,2857	28,5714	0	0	0	1
0	0	0	28,5714	0	0	0	0	1
0	0	0	42,1429	22,1429	2,85714	0	0	1
0	0	0	64,2857	34,2857	0	1	0	2
0	0	0	107,143	25,7143	0	1	0	2
0	0	0	12,8571	4,28571	0	0	0	2
0	0	0	17,1429	8,57143	0	0	0	2
0	0	0	0	30	0	0	0	1
0	0	0	2,85714	137,143	0	0	1	2
0	0	0	25,7143	58,5714	0	0	0	2
0	0	0	30	0	0	0	0	1
0	0	0	25,7143	17,1429	0	0	0	2
0	0	0	4,28571	128,571	0	0	1	2
0	0	0	150	115,714	0	1	1	2
0	0	0	42,8571	51,4286	0	0	0	2
0	0	0	0	120	0	0	1	2
0	1	1	185,714	60	0	1	1	2
0	0	0	0	17,1429	0	0	0	2
0	0	0	48,5714	128,571	0	0	1	2
0	0	0	40,7143	85,7143	0	0	1	2
0	0	0	128,571	107,143	25,7143	1	1	3
0	0	0	120	17,1429	0	1	0	2
0	0	0	17,1429	17,1429	0	0	0	2
0	0	0	25,7143	0	0	0	0	1
0	1	1	158,571	77,1429	34,2857	1	1	2
0	0	0	60	30	0	1	0	1
0	1	1	120	0	0	1	0	2
0	0	0	154,286	10,7143	0	1	0	2
0	0	0	54,2857	154,286	0	0	1	2
0	0	0	132,857	81,4286	0	1	1	2
0	0	0	105	42,8571	0	1	0	1
0	0	0	0	27,8571	0	0	0	1
0	0	0	11,4286	180	0	0	1	3
0	0	0	0	68,5714	34,2857	0	1	2
0	0	0	107,143	10	0	1	0	2
0	0	0	171,429	51,4286	51,4286	1	1	1
0	0	0	154,286	0	51,4286	1	0	1
0	0	0	8,57143	4,28571	0	0	0	1
0	0	0	137,143	8,57143	0	1	0	2
0	0	0	242,857	160	0	1	1	2
0	0	0	35,7143	0	0	0	0	1
0	0	0	128,571	137,143	0	1	1	1
0	0	0	0	102,857	154,286	0	1	1
0	0	0	21,4286	0	8,57143	0	0	2
0	0	0	47,1429	0	0	0	0	2
0	0	0	0	0	0	0	0	2

0	0	0	0	17,1429	0	0	0	2
0	0	0	120	34,2857	8,57143	1	0	2
0	0	0	11,4286	17,1429	34,2857	0	0	2
0	1	1	68,5714	34,2857	0	1	0	2
0	0	0	5,71429	4,28571	8,57143	0	0	2
0	0	0	30	210	25,7143	0	1	1
0	0	0	17,1429	0	0	0	0	2
0	0	0	142,857	0	0	1	0	1
0	0	0	38,5714	51,4286	128,571	0	1	1
0	0	0	122,857	34,2857	0	1	0	2
0	0	1	10	60	60	0	1	3
0	1	1	124,286	29,2857	0	1	0	2
0	0	0	4,28571	124,286	0	0	1	2
0	1	1	60	102,857	0	1	1	2
0	0	0	30	2,85714	8,57143	0	0	2
0	1	1	120	60	0	1	1	1
0	0	0	40,7143	62,8571	0	0	1	3
0	0	0	17,1429	0	34,2857	0	0	3
0	0	0	171,429	85,7143	0	1	1	2
0	0	1	45,7143	85,7143	0	0	1	2
0	0	0	0	0	0	0	0	1
0	0	0	138,571	142,857	4,28571	1	1	2
0	0	0	47,1429	80	0	0	1	2
0	1	1	240	8,57143	0	1	0	2
0	0	0	30	17,1429	0	0	0	1
0	1	1	257,143	180	0	1	1	2
0	1	1	137,143	19,2857	0	1	0	2
0	0	0	12,8571	52,8571	25,7143	0	1	1
0	0	0	85,7143	27,1429	0	1	0	1
0	1	1	75	120	0	1	1	1
0	0	0	51,4286	8,57143	0	0	0	2
0	0	0	0	60	0	0	1	2
0	0	0	60	72,8571	25,7143	1	1	2
0	0	0	25,7143	77,1429	0	0	1	2
0	0	0	137,143	25,7143	0	1	0	1
0	0	0	24,5714	3,42857	0	0	0	1
0	0	0	2,85714	19,2857	34,2857	0	0	2
0	0	0	34,2857	0	0	0	0	2
0	0	0	120	72,8571	0	1	1	2
0	0	0	42,8571	19,2857	0	0	0	2
0	1	1	60	12,8571	0	1	0	2
0	0	0	12,8571	38,5714	0	0	0	2
0	0	0	132,857	32,8571	0	1	0	2
0	0	0	85,7143	17,1429	0	1	0	3
0	0	0	25,7143	17,1429	0	0	0	1
0	0	0	0	231,429	0	0	1	2
0	0	0	102,857	17,1429	0	1	0	1
0	0	0	168,571	0	0	1	0	1

0	0	0	42,8571	8,57143	0	0	0	2
0	0	0	0	0	0	0	0	2
0	0	1	72,8571	22,8571	25,7143	1	0	2
0	0	0	0	42,1429	0	0	0	2
0	0	0	94,2857	231,429	8,57143	1	1	2
0	0	0	23,5714	55,7143	0	0	0	1
0	0	0	0	51,4286	0	0	0	2
0	0	0	0	28,5714	25,7143	0	0	2
0	0	0	42,8571	34,2857	0	0	0	2
0	0	0	17,1429	0	51,4286	0	0	2
0	0	0	30	34,2857	0	0	0	1
0	0	0	7,14286	12,8571	0	0	0	2
0	0	0	17,1429	51,4286	0	0	0	2
0	0	0	128,571	0	77,1429	1	1	3
0	0	0	46,4286	60	0	0	1	2
0	0	0	51,4286	34,2857	0	0	0	2
0	0	0	51,4286	180	0	0	1	2
0	0	0	25,7143	0	0	0	0	2
0	0	0	0	82,8571	0	0	1	2
0	0	0	162,857	120	120	1	1	1
0	0	0	87,1429	17,1429	0	1	0	2
0	0	0	35	85,7143	8,57143	0	1	1
0	0	0	38,5714	25,7143	0	0	0	2
0	0	0	51,4286	25,7143	0	0	0	2
0	0	0	15	14,2857	0	0	0	2
0	0	0	180	135	17,1429	1	1	2
0	0	0	77,1429	60	0	1	1	1
0	1	1	240	137,143	51,4286	1	1	1
0	0	0	60	171,429	0	1	1	2
0	0	0	35	8,57143	25,7143	0	0	2
0	0	0	33,5714	231,429	0	0	1	2
0	0	0	0	17,1429	0	0	0	2
0	0	0	47,1429	42,8571	0	0	0	2
0	0	1	57,1429	117,857	128,571	0	1	1
0	0	0	84,2857	85,7143	8,57143	1	1	1
0	0	0	37,1429	12,8571	0	0	0	2
0	0	0	17,1429	85,7143	0	0	1	2
0	0	0	40	38,5714	0	0	0	2
0	0	0	10,7143	12,8571	128,571	0	1	3
0	0	0	34,2857	0	0	0	0	2
0	0	0	0	0	0	0	0	2
0	0	0	8,57143	4,28571	0	0	0	2
0	0	0	14,2857	0	0	0	0	2
0	0	0	17,1429	57,1429	0	0	0	2
0	0	0	111,429	128,571	128,571	1	1	1
0	0	0	170,286	102,857	0	1	1	2
0	0	0	0	25,7143	0	0	0	2
0	0	0	0	0	15,7143	0	0	2

0	1	1	115,714	64,2857	0	1	1	2
0	0	0	164,286	68,5714	4,28571	1	1	2
0	0	0	42,8571	17,1429	0	0	0	2
0	0	0	194,286	171,429	60	1	1	2
0	0	0	138,571	162,857	25,7143	1	1	2
0	0	0	51,4286	11,4286	17,1429	0	0	3
0	0	0	21,4286	27,8571	0	0	0	3
0	0	0	248,571	128,571	205,714	1	1	2
0	0	0	60	37,1429	0	1	0	2
0	0	0	47,1429	120	51,4286	0	1	3
1	0	1	40	0	77,1429	0	1	2
0	0	0	0	0	38,5714	0	0	3
0	0	0	20	17,1429	0	0	0	3
0	0	0	21,4286	8,57143	0	0	0	2
0	1	1	90	0	0	1	0	2
0	0	0	175,714	197,143	0	1	1	2
0	0	0	17,1429	0	0	0	0	2
0	0	0	34,2857	4,28571	0	0	0	2
0	0	0	102,857	0	0	1	0	2
0	0	0	64,2857	128,571	0	1	1	2
0	0	0	34,2857	51,4286	6,42857	0	0	1
0	0	0	15	17,1429	0	0	0	2
0	0	0	55,7143	47,1429	102,857	0	1	1
0	0	0	12,8571	34,2857	0	0	0	2
0	0	0	141,429	154,286	68,5714	1	1	3
0	0	0	128,571	105	0	1	1	1
0	0	1	60	102,857	0	1	1	2
0	0	0	0	0	77,1429	0	1	2
0	0	0	30	60	0	0	1	3
0	0	0	4,28571	12,8571	0	0	0	2
0	0	0	197,143	242,143	51,4286	1	1	2
0	0	0	128,571	34,2857	102,857	1	1	1
0	0	0	25,7143	111,429	77,1429	0	1	2
0	0	0	0	51,4286	111,429	0	1	1
0	0	0	128,571	38,5714	0	1	0	1
0	0	0	7,14286	30	0	0	0	1
0	0	0	102,857	8,57143	0	1	0	2
0	0	0	17,1429	42,8571	0	0	0	2
0	0	0	0	8,57143	0	0	0	1
0	0	0	68,5714	0	0	1	0	2
0	0	0	41,4286	18,5714	0	0	0	1
0	0	0	60	32,1429	0	1	0	2
0	0	0	0	17,1429	60	0	1	1
0	1	1	240	120	42,8571	1	1	2
0	0	0	8,57143	42,8571	0	0	0	2
0	0	0	104,286	68,5714	17,1429	1	1	2
0	1	1	205,714	154,286	25,7143	1	1	2
0	0	0	165,714	51,4286	0	1	0	2

0	0	0	90	124,286	0	1	1	2
0	0	0	42,8571	85,7143	42,8571	0	1	2
0	0	1	68,5714	90	0	1	1	1
0	0	0	118,571	111,429	0	1	1	2
0	0	0	25,7143	4,28571	0	0	0	2
1	0	1	20	64,2857	0	0	1	2
0	0	0	25,7143	8,57143	0	0	0	2
0	0	0	78,5714	12,8571	17,1429	1	0	2
0	0	1	85,7143	45	0	1	0	2
0	0	0	25	34,2857	0	0	0	2
0	0	0	1,42857	25,7143	0	0	0	2
0	0	0	15	17,1429	0	0	0	1
0	0	0	11,4286	128,571	25,7143	0	1	2
0	0	0	94,2857	8,57143	0	1	0	3
0	0	0	4,28571	8,57143	0	0	0	2
0	1	1	82,8571	4,28571	0	1	0	2
0	0	0	187,143	17,1429	0	1	0	1
0	0	0	102,857	0	0	1	0	2
0	0	0	20	102,857	34,2857	0	1	3
0	0	0	0	0	0	0	0	2
0	0	0	50	17,1429	0	0	0	2
0	0	0	120	42,8571	0	1	0	1
0	0	0	54,2857	150	0	0	1	2
0	0	0	14,2857	8,57143	0	0	0	2
0	0	0	94,2857	85,7143	17,1429	1	1	1
0	0	0	31,4286	8,57143	0	0	0	3
0	1	1	282,857	192,857	77,1429	1	1	2
0	0	0	124,286	205,714	25,7143	1	1	2
0	0	0	137,143	51,4286	0	1	0	1
0	0	0	137,143	0	0	1	0	2
0	0	0	51,4286	77,1429	0	0	1	2
0	0	0	20	60	120	0	1	2
0	0	0	55,7143	120	17,1429	0	1	2
0	1	1	128,571	0	0	1	0	2
0	0	0	0	0	8,57143	0	0	2
0	0	0	12,8571	28,5714	0	0	0	3
0	0	0	15	17,1429	0	0	0	2
0	0	0	34,2857	92,1429	0	0	1	2
0	0	0	102,857	0	0	1	0	2
0	0	0	17,1429	0	8,57143	0	0	2
0	0	0	27,8571	34,2857	0	0	0	2
0	0	0	14,2857	8,57143	8,57143	0	0	2
0	0	0	24,2857	11,4286	0	0	0	2
0	0	0	138,571	128,571	0	1	1	2
0	0	0	25,7143	0	0	0	0	2
0	0	0	64,2857	42,8571	0	1	0	1
0	0	0	154,286	17,1429	0	1	0	2
0	0	0	42,8571	21,4286	8,57143	0	0	2

0	*0*	*0*	*34,2857*	*25,7143*	*77,1429*	*0*	*1*	*2*
0	*0*	*0*	*147,143*	*10*	*0*	*1*	*0*	*1*
0	*0*	*0*	*40*	*17,1429*	*0*	*0*	*0*	*1*
0	*0*	*0*	*55,7143*	*0*	*0*	*0*	*0*	*2*
0	*0*	*0*	*8,57143*	*18,5714*	*17,1429*	*0*	*0*	*2*
0	*0*	*0*	*0*	*60*	*17,1429*	*0*	*1*	*1*
0	*0*	*0*	*0*	*128,571*	*77,1429*	*0*	*1*	*2*
0	*0*	*1*	*77,1429*	*25,7143*	*34,2857*	*1*	*1*	*2*
0	*0*	*0*	*85,7143*	*154,286*	*85,7143*	*1*	*1*	*2*
0	*0*	*0*	*34,2857*	*85,7143*	*8,57143*	*0*	*1*	*2*
0	*0*	*0*	*38,5714*	*180*	*0*	*0*	*1*	*2*
0	*1*	*1*	*137,143*	*55,7143*	*51,4286*	*1*	*1*	*2*
0	*0*	*0*	*25,7143*	*235,714*	*154,286*	*0*	*1*	*2*
0	*0*	*0*	*14,2857*	*42,8571*	*0*	*0*	*0*	*2*
0	*0*	*0*	*0*	*25,7143*	*17,1429*	*0*	*0*	*2*
0	*0*	*0*	*0*	*4,28571*	*0*	*0*	*0*	*2*
0	*0*	*0*	*210*	*22,8571*	*0*	*1*	*0*	*2*
0	*0*	*0*	*190,714*	*175,714*	*0*	*1*	*1*	*2*
0	*0*	*0*	*34,2857*	*51,4286*	*0*	*0*	*0*	*2*
0	*0*	*0*	*17,8571*	*85,7143*	*0*	*0*	*1*	*2*
0	*0*	*0*	*0*	*8,57143*	*0*	*0*	*0*	*2*
0	*1*	*1*	*121,429*	*151,429*	*8,57143*	*1*	*1*	*2*
0	*0*	*0*	*94,2857*	*180*	*0*	*1*	*1*	*2*
0	*0*	*0*	*72,8571*	*25,7143*	*0*	*1*	*0*	*2*
0	*0*	*0*	*94,2857*	*77,1429*	*60*	*1*	*1*	*2*
0	*0*	*0*	*8,57143*	*25,7143*	*51,4286*	*0*	*1*	*3*
0	*0*	*0*	*5,71429*	*120*	*0*	*0*	*1*	*2*
0	*1*	*1*	*210*	*25,7143*	*60*	*1*	*1*	*2*
0	*1*	*1*	*120*	*85,7143*	*0*	*1*	*1*	*1*
0	*0*	*0*	*180*	*231,429*	*0*	*1*	*1*	*2*
0	*0*	*1*	*42,8571*	*51,4286*	*0*	*0*	*0*	*2*
0	*0*	*0*	*0*	*77,1429*	*25,7143*	*0*	*1*	*1*
0	*0*	*0*	*77,1429*	*0*	*0*	*1*	*0*	*2*
0	*0*	*0*	*15*	*8,57143*	*128,571*	*0*	*1*	*2*
0	*0*	*0*	*20*	*0*	*0*	*0*	*0*	*1*
0	*1*	*1*	*137,143*	*0*	*0*	*1*	*0*	*2*
0	*0*	*0*	*68,5714*	*102,857*	*8,57143*	*1*	*1*	*2*
0	*0*	*0*	*51,4286*	*51,4286*	*0*	*0*	*0*	*1*
0	*0*	*1*	*0*	*124,286*	*0*	*0*	*1*	*2*
0	*0*	*0*	*48,5714*	*28,5714*	*8,57143*	*0*	*0*	*1*
0	*0*	*0*	*0*	*120*	*0*	*0*	*1*	*2*
0	*0*	*0*	*25,7143*	*17,1429*	*0*	*0*	*0*	*2*
1	*0*	*1*	*102,857*	*214,286*	*0*	*1*	*1*	*3*
0	*0*	*0*	*190,714*	*21,4286*	*0*	*1*	*0*	*1*
0	*0*	*0*	*42,8571*	*137,143*	*128,571*	*0*	*1*	*2*
0	*0*	*0*	*22,8571*	*25,7143*	*0*	*0*	*0*	*2*
0	*0*	*0*	*60*	*42,8571*	*0*	*1*	*0*	*2*
0	*0*	*0*	*25*	*94,2857*	*68,5714*	*0*	*1*	*2*

0	1	1	97,1429	23,5714	17,1429	1	0	1
0	0	0	8,57143	23,5714	0	0	0	2
0	0	0	68,5714	12,8571	0	1	0	2
0	0	0	0	162,857	25,7143	0	1	1
0	0	0	0	0	0	0	0	2
0	0	0	47,1429	28,5714	0	0	0	1
0	0	0	60	81,4286	0	1	1	2
0	0	0	151,429	210	8,57143	1	1	2
0	0	0	4,28571	5,71429	0	0	0	2
0	0	0	60	171,429	25,7143	1	1	1
0	0	0	34,2857	60	0	0	1	2
0	0	0	45	87,8571	0	0	1	1
0	0	0	30	122,857	0	0	1	2
0	0	0	131,429	17,1429	25,7143	1	0	2
0	0	0	145,714	94,2857	0	1	1	2
0	0	0	154,286	62,8571	0	1	1	2
0	0	0	145,714	128,571	0	1	1	2
0	0	0	188,571	158,571	17,1429	1	1	2
0	0	0	94,2857	137,143	51,4286	1	1	1
0	0	0	17,1429	180	102,857	0	1	2
0	0	0	0	8,57143	0	0	0	1
0	0	0	42,8571	0	0	0	0	1
0	0	0	168,857	12,8571	0	1	0	2
0	0	0	0	57,1429	0	0	0	2
0	0	0	180	420	102,857	1	1	2
0	0	0	57,1429	2,14286	0	0	0	2
0	0	0	188,571	342,857	17,1429	1	1	2
0	0	0	25,7143	8,57143	0	0	0	1
0	0	0	60	8,57143	0	1	0	2
0	0	0	21,4286	102,857	25,7143	0	1	1
0	0	0	23,5714	25,7143	0	0	0	2
0	0	0	20	42,8571	0	0	0	1
0	0	0	0	90	0	0	1	2
0	1	1	85,7143	2,85714	0	1	0	2
0	0	0	214,286	291,429	68,5714	1	1	2
0	0	0	0	160	17,1429	0	1	2
0	0	0	2,85714	0	0	0	0	2
0	1	1	360	265,714	180	1	1	2
1	1	1	205,714	120	51,4286	1	1	2
0	1	1	150	8,57143	0	1	0	2
0	1	1	102,143	17,1429	15	1	0	2
0	0	0	85,7143	145,714	17,1429	1	1	2
0	0	0	10,7143	17,1429	0	0	0	2
0	0	0	102,857	77,1429	0	1	1	1
0	0	0	42,8571	51,4286	25,7143	0	1	2
0	0	0	175,714	162,857	25,7143	1	1	2
0	0	0	0	0	0	0	0	2
0	0	0	115,714	17,1429	0	1	0	2

0	0	0	8,57143	4,28571	0	0	0	2
0	0	0	113,571	21,4286	0	1	0	1
0	0	0	22,8571	0	0	0	0	2
0	0	0	0	102,857	0	0	1	2
0	0	0	30	17,1429	51,4286	0	1	1
0	0	0	34,2857	0	0	0	0	2
0	0	0	25,7143	65,7143	30	0	1	2
0	0	0	22,8571	8,57143	0	0	0	2
0	0	0	128,571	0	0	1	0	2
0	0	0	107,143	158,571	25,7143	1	1	1
0	0	0	109,286	128,571	40,7143	1	1	2
0	0	0	0	0	0	0	0	3
0	0	0	197,143	0	0	1	0	1
0	1	1	110	85,7143	0	1	1	2
0	0	0	94,2857	23,5714	12,8571	1	0	2
0	0	0	222,857	60	0	1	1	1
0	0	0	111,429	180	0	1	1	2
0	0	0	57,1429	68,5714	17,1429	0	1	2
0	0	0	62,8571	0	0	1	0	2
0	0	0	115,714	5,71429	0	1	0	2
0	0	0	30	30	17,1429	0	0	3
0	0	0	5,71429	55,7143	0	0	0	2
0	0	0	42,8571	40	0	0	0	3
0	0	0	47,1429	0	8,57143	0	0	1
0	0	0	60	60	0	1	1	2
0	0	0	95,7143	0	128,571	1	1	2
0	0	0	68,5714	188,571	120	1	1	3
0	0	0	17,1429	25,7143	0	0	0	2
0	0	0	60	30	0	1	0	1
0	0	0	51,4286	17,1429	0	0	0	2
0	0	0	40	10,7143	0	0	0	2
0	0	0	85,7143	4,28571	17,1429	1	0	1
0	1	1	128,571	42,8571	0	1	0	1
0	0	0	214,286	24,2857	17,1429	1	0	2
0	0	0	30	128,571	0	0	1	1
0	0	0	0	282,857	3,14286	0	1	2
0	0	0	21,4286	5,71429	0	0	0	2
0	0	0	21,4286	34,2857	0	0	0	2
1	0	1	42,8571	51,4286	128,571	0	1	2
0	0	0	67,1429	25,7143	0	1	0	2
0	0	0	218,571	114,286	0	1	1	1
0	0	0	30	2,14286	0	0	0	1
0	1	1	60	51,4286	0	1	0	2
0	0	0	8,57143	8,57143	0	0	0	2
0	0	0	60	8,57143	0	1	0	1
0	0	0	34,2857	0	0	0	0	1
0	0	0	8,57143	0	0	0	0	2
0	0	0	154,286	205,714	0	1	1	2

0	0	0	30	0	120	0	1	2
0	0	0	25,7143	107,143	0	0	1	2
0	0	0	51,4286	85,7143	42,8571	0	1	2
0	0	0	77,1429	8,57143	0	1	0	2
0	1	1	60	0	51,4286	1	0	3
0	0	0	34,2857	17,1429	0	0	0	2
0	1	1	62,8571	17,1429	51,4286	1	1	1
0	0	0	162,857	34,2857	0	1	0	1
0	0	0	44,2857	8,57143	0	0	0	2
0	0	0	15	51,4286	25,7143	0	1	2
0	0	0	171,429	145,714	0	1	1	2
0	0	0	25,7143	77,1429	0	0	1	2
0	0	0	6,42857	21,4286	0	0	0	1
0	0	0	134,286	42,8571	0	1	0	2
0	0	0	128,571	342,857	0	1	1	2
0	0	0	167,143	72,8571	51,4286	1	1	2
0	0	0	34,2857	23,5714	0	0	0	2
0	0	0	77,1429	8,57143	0	1	0	2
0	0	0	68,5714	17,1429	25,7143	1	0	2
0	0	0	132,857	128,571	8,57143	1	1	2
0	0	0	0	120	0	0	1	1
0	0	0	160	34,2857	25,7143	1	1	2
0	0	0	94,2857	105	23,5714	1	1	2
0	0	0	141,429	94,2857	0	1	1	2
0	0	0	158,571	145,714	0	1	1	2
0	0	0	128,571	148,571	0	1	1	2
0	0	0	21,4286	0	0	0	0	2
0	0	0	0	131,429	0	0	1	2
0	0	0	30	102,857	34,2857	0	1	2
0	0	0	51,4286	124,286	8,57143	0	1	2
0	0	0	0	20	0	0	0	2
0	0	0	32,1429	25,7143	0	0	0	1
0	0	0	107,143	34,2857	0	1	0	1
0	1	1	81,4286	25,7143	0	1	0	1
0	0	0	0	0	4,28571	0	0	2
0	0	0	141,429	85,7143	68,5714	1	1	2
0	0	0	45,7143	60	0	0	1	2
0	1	1	188,571	145,714	0	1	1	2
0	0	0	38,5714	107,143	0	0	1	1
1	0	1	36,4286	224,286	120	0	1	2
0	0	0	201,429	188,571	0	1	1	2
0	0	0	0	17,1429	0	0	0	1
0	0	0	15,7143	4,28571	4,28571	0	0	2
0	0	0	188,571	51,4286	12,8571	1	1	2
0	0	0	0	51,4286	0	0	0	2
0	0	0	85,7143	0	4,28571	1	0	2
0	0	0	42,8571	58,5714	0	0	0	2
0	0	0	65,7143	5,71429	0	1	0	1

1	0	1	45,7143	80,7143	60	0	1	2
0	0	0	158,571	188,571	25,7143	1	1	1
0	0	0	162,857	162,857	0	1	1	3
0	1	1	150	102,857	0	1	1	2
0	0	0	0	274,286	0	0	1	2
0	0	0	50,7143	0	0	0	0	1
0	0	0	60	10	0	1	0	2
0	0	0	21,4286	30	4,28571	0	0	2
0	0	0	171,429	8,57143	0	1	0	2
0	0	0	94,2857	30	0	1	0	2
0	0	0	137,143	342,857	128,571	1	1	1
0	0	0	42,8571	85,7143	85,7143	0	1	2
0	0	0	62,1429	8,57143	0	1	0	2
0	0	0	34,2857	34,2857	0	0	0	3
0	0	0	17,1429	6,42857	0	0	0	2
0	1	1	145,714	85,7143	128,571	1	1	1
0	0	0	140	171,429	25,7143	1	1	1
0	0	0	55,7143	111,429	0	0	1	3
0	0	0	68,5714	8,57143	0	1	0	2
0	1	1	142,857	68,5714	0	1	1	2
0	0	0	25,7143	128,571	0	0	1	2
0	0	0	11,4286	27,8571	4,28571	0	0	1
0	0	1	171,429	180	0	1	1	2
0	0	0	107,143	55,7143	0	1	0	2
0	0	0	4,28571	51,4286	51,4286	0	1	1
0	0	0	145,714	34,2857	25,7143	1	1	1
0	0	0	222,857	214,286	0	1	1	2
0	0	0	17,1429	102,857	0	0	1	2
0	0	0	8,57143	0	0	0	0	2
0	0	0	157,143	8,57143	0	1	0	2
0	0	0	214,286	202,857	0	1	1	2
0	0	0	40	0	0	0	0	2
0	0	0	0	231,429	51,4286	0	1	2
0	0	0	143,571	105	25,7143	1	1	2
0	1	1	71,4286	23,5714	0	1	0	2
0	0	0	64,2857	0	17,1429	1	0	1
0	0	0	0	201,429	8,57143	0	1	1
0	0	0	2,85714	4,28571	0	0	0	3
0	0	0	37,1429	25,7143	0	0	0	2
0	0	0	38,5714	0	0	0	0	2
0	0	0	111,429	68,5714	68,5714	1	1	2
0	0	0	42,8571	128,571	0	0	1	2
0	0	0	120	42,8571	0	1	0	2
0	0	0	98,5714	0	0	1	0	2
0	0	0	0	120	94,2857	0	1	1
0	0	0	25,7143	137,143	0	0	1	2
0	0	0	154,286	0	8,57143	1	0	2
0	0	0	180	60	42,8571	1	1	3

0	0	0	0	110	145,714	0	1	1
0	0	0	42,8571	128,571	0	0	1	2
0	0	0	32,1429	83,5714	25,7143	0	1	3
0	0	0	34,2857	102,857	0	0	1	1
0	0	0	60	20	4,28571	1	0	2
0	0	0	128,571	68,5714	0	1	1	2
0	0	0	0	128,571	17,1429	0	1	1
0	0	0	25,7143	17,1429	0	0	0	2
0	0	1	122,857	28,5714	0	1	0	2
0	0	1	145,714	25,7143	17,1429	1	0	2
0	0	0	8,57143	8,57143	0	0	0	2
0	0	0	30	132,857	32,8571	0	1	2
0	0	0	27,1429	150	0	0	1	1
0	0	0	128,571	6,42857	34,2857	1	0	1
0	1	1	240	308,571	102,857	1	1	2
0	0	0	137,143	145,714	34,2857	1	1	1
0	0	0	155,714	117,857	0	1	1	2
0	0	0	128,571	102,857	0	1	1	1
0	0	0	180	120	145,714	1	1	2
0	0	0	175,714	197,143	0	1	1	2
0	0	0	200	51,4286	0	1	0	2
0	0	0	30	0	0	0	0	3
0	0	0	48,5714	17,1429	0	0	0	2
0	0	0	205,714	77,1429	42,8571	1	1	1
0	0	0	8,57143	162,857	17,1429	0	1	2
0	0	0	25,7143	25,7143	0	0	0	1
0	0	0	102,857	0	0	1	0	1
0	1	1	94,2857	25,7143	77,1429	1	1	3
0	0	0	20	171,429	25,7143	0	1	2
0	0	0	98,5714	83,5714	8,57143	1	1	1
0	0	0	94,2857	131,429	25,7143	1	1	2
0	0	0	60	85,7143	8,57143	1	1	2
0	0	1	41,4286	111,429	0	0	1	2
0	0	0	25,7143	60	0	0	1	2
0	0	0	60	42,8571	85,7143	1	1	2
0	0	0	45,7143	17,1429	0	0	0	1
0	0	0	2,85714	2,85714	0	0	0	1
0	0	0	60	120	0	1	1	2
0	0	0	40	201,429	128,571	0	1	2
0	0	0	27,1429	4,28571	0	0	0	2
0	1	1	102,857	142,857	8,57143	1	1	2
0	0	0	12,8571	85,7143	0	0	1	2
0	0	0	128,571	128,571	0	1	1	2
0	0	0	5,71429	4,28571	0	0	0	2
0	0	0	0	125,714	6,42857	0	1	1
0	0	0	1,42857	12,8571	0	0	0	2
0	0	0	57,1429	31,4286	0	0	0	2
0	0	0	148,571	284,286	62,8571	1	1	1

0	0	0	12,8571	45,7143	0	0	0	2
0	0	0	8,57143	17,1429	0	0	0	2
0	0	0	8,57143	0	0	0	0	3
0	1	1	261,429	34,2857	42,8571	1	1	3
0	0	0	34,2857	17,1429	0	0	0	2
0	0	0	21,4286	25,7143	42,8571	0	1	2
0	0	0	0	85,7143	0	0	1	2
0	0	0	111,429	0	51,4286	1	0	2
0	0	0	14,2857	17,1429	8,57143	0	0	2
0	0	0	8,57143	20	0	0	0	2
0	0	0	25,7143	92,1429	25,7143	0	1	2
0	0	0	0	160,714	0	0	1	1
0	0	0	85,7143	0	0	1	0	2
0	0	0	2,85714	120	0	0	1	2
0	0	0	53,5714	0	0	0	0	1
0	0	0	115,714	38,5714	0	1	0	2
0	0	0	85,7143	68,5714	0	1	1	2
0	0	0	68,5714	102,857	0	1	1	1
0	0	0	0	0	0	0	0	2
0	0	0	60	2,14286	30	1	0	2
0	1	1	180	0	0	1	0	2
0	0	0	8,57143	165	34,2857	0	1	1
0	0	0	0	60	0	0	1	1
0	0	0	5,71429	318,571	180	0	1	2
0	0	0	41,4286	138,571	0	0	1	2
0	0	0	22,8571	24,2857	0	0	0	2
0	0	0	128,571	25,7143	0	1	0	1
0	0	0	8,57143	0	2,85714	0	0	2
0	0	0	51,4286	51,4286	17,1429	0	1	2
0	0	0	42,8571	17,1429	0	0	0	1
0	0	0	1,42857	51,4286	0	0	0	3
0	0	0	8,57143	168,571	0	0	1	2
0	0	0	157,143	24,2857	2,85714	1	0	2
0	0	0	4,28571	0	0	0	0	2
0	0	0	8,57143	25,7143	0	0	0	2
0	0	0	214,286	31,4286	25,7143	1	0	2
0	0	0	37,1429	0	32,1429	0	0	3
0	0	0	31,4286	94,2857	0	0	1	1
0	0	0	7,14286	0	0	0	0	1
0	0	0	34,2857	58,5714	128,571	0	1	3
0	0	0	4,28571	8,57143	0	0	0	2
0	0	0	77,1429	155,714	154,286	1	1	2
0	0	0	145,714	0	4,28571	1	0	2
0	0	0	28,5714	0	0	0	0	2
1	0	1	12,8571	34,2857	64,2857	0	1	1
0	1	1	248,571	0	25,7143	1	0	2
0	1	1	360	60	0	1	1	1
0	0	0	42,8571	137,143	0	0	1	2

0	0	0	0	150	180	0	1	1
0	0	0	42,8571	10	0	0	0	2
0	0	0	42,8571	150	28,5714	0	1	1
0	1	1	68,5714	4,28571	0	1	0	2
0	0	0	162,857	1,42857	11,4286	1	0	1
0	0	0	137,143	180	25,7143	1	1	2
0	0	0	25,7143	12,8571	0	0	0	1
0	0	0	85,7143	25,7143	0	1	0	2
0	0	1	42,8571	317,143	42,8571	0	1	1
0	0	0	94,2857	132,857	0	1	1	2
0	1	1	180	240	0	1	1	2
0	0	0	38,5714	231,429	51,4286	0	1	2
0	0	0	190	4,28571	34,2857	1	0	2
0	0	0	154,286	0	0	1	0	1
0	0	0	150	25,7143	0	1	0	1
0	0	0	0	128,571	0	0	1	2
0	0	0	188,571	17,1429	17,1429	1	0	2
0	0	0	8,57143	8,57143	0	0	0	2
0	0	0	248,571	77,1429	0	1	1	2
0	0	0	34,2857	90	0	0	1	2
0	0	0	25,7143	80	120	0	1	2
0	0	0	30	8,57143	0	0	0	2
0	0	0	128,571	25,7143	0	1	0	2
0	0	0	85,7143	0	25,7143	1	0	1
0	0	0	171,429	102,857	0	1	1	2
0	0	0	5,71429	14,2857	0	0	0	1
0	0	1	62,8571	90	3,42857	1	1	2
0	0	0	180,714	18,8571	0	1	0	1
0	0	0	17,1429	68,5714	0	0	1	1
0	0	0	115,714	21,4286	0	1	0	2
0	0	0	8,57143	91,4286	25,7143	0	1	3
0	0	0	147,143	25,7143	0	1	0	1
0	0	0	111,429	0	0	1	0	2
0	0	0	0	155,714	8,57143	0	1	1
0	0	1	270	141,429	17,1429	1	1	2
0	0	0	0	12,8571	0	0	0	1
0	0	0	32,1429	15	0	0	0	2
0	0	0	60	0	5,71429	1	0	1
0	0	0	32,1429	11,4286	0	0	0	2
0	0	0	12,8571	51,4286	0	0	0	1
0	1	1	205,714	0	0	1	0	2
0	0	0	2,14286	0	0	0	0	2
0	0	0	12,8571	51,4286	42,8571	0	1	3
0	1	1	360	4,28571	20	1	0	1
0	1	1	60	72,8571	102,857	1	1	1
0	0	0	8,57143	137,143	0	0	1	3
0	0	0	47,1429	128,571	0	0	1	1
0	0	0	98,5714	17,1429	68,5714	1	1	1

0	0	0	34,2857	145,714	0	0	1	2
0	0	0	28,5714	25,7143	0	0	0	2
0	0	0	154,286	214,286	85,7143	1	1	1
0	0	0	100,714	12,8571	0	1	0	2
0	0	0	51,4286	177,857	0	0	1	2
0	0	0	111,429	205,714	0	1	1	2
0	0	0	48,5714	25,7143	12,8571	0	0	2
0	0	0	15	128,571	0	0	1	2
0	0	0	42,8571	128,571	0	0	1	2
0	0	0	42,8571	68,5714	0	0	1	2
0	0	0	0	51,4286	0	0	0	2
0	0	0	201,429	360	180	1	1	2
0	0	0	85,7143	85,7143	0	1	1	2
0	0	0	42,8571	94,2857	25,7143	0	1	1
0	0	0	8,57143	0	0	0	0	2
0	0	0	154,286	154,286	25,7143	1	1	2
0	0	0	11,4286	0	0	0	0	2
0	0	0	17,1429	0	0	0	0	2
0	0	0	100	11,4286	0	1	0	2
1	1	1	162,857	145,714	128,571	1	1	2
0	0	0	70	4,28571	0	1	0	1
0	0	0	4,28571	0	0	0	0	1
0	0	0	125,714	30	0	1	0	1
0	0	0	7,14286	22,8571	0	0	0	1
0	0	0	68,5714	51,4286	25,7143	1	1	2
0	0	0	300	34,2857	31,4286	1	1	2
0	0	0	205,714	68,5714	25,7143	1	1	2
0	0	0	6,42857	74,2857	42,8571	0	1	2
0	0	0	0	60	77,1429	0	1	2
0	1	1	60	60	0	1	1	1
0	0	0	0	0	0	0	0	2
0	0	0	180	162,857	102,857	1	1	1
0	0	0	177,143	8,57143	0	1	0	2
0	0	0	20	20	0	0	0	3
0	0	0	8,57143	0	0	0	0	3
0	0	0	177,143	60	0	1	1	2
0	0	0	0	25,7143	0	0	0	1
0	0	0	2,85714	145,714	0	0	1	1
0	0	0	42,8571	0	0	0	0	1
0	0	0	128,571	145,714	0	1	1	2
0	0	0	0	17,1429	0	0	0	2
0	0	0	18,5714	4,28571	0	0	0	2
0	0	0	42,8571	23,5714	0	0	0	1
0	0	0	141,429	64,2857	0	1	1	1
0	0	0	24,2857	0	0	0	0	2
0	0	0	68,5714	154,286	0	1	1	1
0	0	0	12,8571	182,857	0	0	1	1
0	0	0	107,143	8,57143	17,1429	1	0	1

0	0	0	30	42,8571	0	0	0	2
0	0	0	141,429	120	8,57143	1	1	2
0	1	1	120	210	180	1	1	2
0	0	0	231,429	0	42,8571	1	0	1
0	0	0	240	180	145,714	1	1	1
0	0	0	0	0	3,14286	0	0	2
0	0	0	248,571	102,857	0	1	1	2
0	0	0	2,14286	25,7143	0	0	0	2
0	0	0	17,1429	5,71429	0	0	0	2
0	0	0	42,8571	8,57143	25,7143	0	0	2
0	0	0	77,1429	154,286	17,1429	1	1	2
0	0	0	132,857	17,1429	0	1	0	1
0	0	0	58,5714	50	0	0	0	2
0	0	0	128,571	0	25,7143	1	0	1
0	0	0	27,1429	22,8571	0	0	0	1
0	0	0	81,4286	120	0	1	1	1
0	0	0	20	34,2857	0	0	0	2
0	0	0	8,57143	94,2857	25,7143	0	1	2
0	0	0	60	51,4286	0	1	0	2
0	0	0	102,857	214,286	0	1	1	2
0	0	0	158,571	12,8571	0	1	0	2
0	0	0	21,4286	8,57143	154,286	0	1	2
0	0	0	19,2857	0	17,1429	0	0	1
0	1	1	120	0	0	1	0	2
0	0	0	36,4286	27,1429	8,57143	0	0	2
0	0	0	0	90	102,857	0	1	2
0	0	0	51,4286	154,286	68,5714	0	1	2
0	0	0	68,5714	8,57143	0	1	0	2
0	0	0	51,4286	21,4286	5,71429	0	0	2
0	0	0	137,143	17,1429	0	1	0	1
0	0	0	274,286	162,857	0	1	1	2
0	0	0	48,5714	197,143	0	0	1	1
0	0	0	128,571	214,286	0	1	1	2
0	0	0	0	0	0	0	0	2
0	0	0	77,1429	17,1429	0	1	0	2
0	1	1	180	0	8,57143	1	0	2
0	0	0	128,571	282,857	0	1	1	2
0	1	1	128,571	25,7143	0	1	0	2
0	0	0	8,57143	25,7143	6,42857	0	0	1
0	0	0	25,7143	51,4286	25,7143	0	1	3
0	0	0	30	10	0	0	0	1
0	1	1	70	38,5714	17,1429	1	0	1
0	0	0	158,571	169,286	17,1429	1	1	2
0	0	0	77,1429	0	0	1	0	2
0	0	0	77,1429	17,1429	0	1	0	2
0	0	0	94,2857	12,8571	0	1	0	2
0	0	0	128,571	145,714	0	1	1	1
0	0	0	85,7143	0	17,1429	1	0	2

0	0	0	25,7143	0	0	0	0	2
0	0	0	5,71429	25,7143	0	0	0	2
0	0	0	111,429	4,28571	0	1	0	2
0	0	0	14,2857	62,8571	21,4286	0	1	2
0	0	0	72,8571	102,857	180	1	1	2
0	0	0	197,143	137,143	8,57143	1	1	2
0	0	0	44,2857	25,7143	0	0	0	1
0	0	0	31,4286	128,571	0	0	1	1
0	0	0	81,4286	28,5714	30	1	0	2
0	0	0	10,7143	182,857	0	0	1	1
0	0	0	13,5714	17,1429	0	0	0	2
0	0	0	17,1429	94,2857	0	0	1	2
1	1	1	231,429	24,2857	77,1429	1	1	1
0	0	0	4,28571	42,8571	0	0	0	1
0	0	0	57,1429	55,7143	0	0	0	2
0	0	0	30	0	8,57143	0	0	2
0	0	0	140	25,7143	0	1	0	2
0	0	0	139,286	22,8571	0	1	0	2
0	0	0	74,2857	205,714	42,8571	1	1	2
0	0	0	5,71429	0	0	0	0	2
0	0	0	42,8571	42,8571	0	0	0	2
0	0	0	14,2857	162,857	0	0	1	2
0	0	0	34,2857	51,4286	51,4286	0	1	2
0	0	0	188,571	154,286	0	1	1	2
0	1	1	111,429	35,7143	0	1	0	1
0	0	0	0	8,57143	0	0	0	2
0	0	0	0	150	120	0	1	2
0	0	0	27,1429	102,857	51,4286	0	1	2
0	0	0	162,857	8,57143	128,571	1	1	2
0	0	0	40	111,429	0	0	1	2
0	0	0	30	28,5714	0	0	0	2
0	0	0	28,5714	34,2857	128,571	0	1	2
0	0	0	150	64,2857	0	1	1	2
0	0	0	128,571	38,5714	0	1	0	2
0	0	0	102,857	257,143	128,571	1	1	1
0	0	0	0	154,286	154,286	0	1	2
0	0	1	98,5714	51,4286	0	1	0	1
0	0	0	6,42857	0	8,57143	0	0	2
0	0	0	90	128,571	0	1	1	2
0	0	1	32,1429	65,7143	0	0	1	2
0	0	0	66,4286	154,286	0	1	1	1
0	0	0	162,857	132,857	17,1429	1	1	2
0	1	1	137,143	145,714	77,1429	1	1	2
0	0	0	40	145,714	0	0	1	1
0	0	0	24,2857	17,1429	0	0	0	2
0	0	0	5,71429	154,286	25,7143	0	1	2
0	0	0	40	8,57143	0	0	0	1
0	0	1	190	291,429	0	1	1	2

0	*1*	*1*	*60*	*184,286*	*0*	*1*	*1*	*1*
0	*0*	*0*	*0*	*30*	*0*	*0*	*0*	*2*
0	*0*	*0*	*21,4286*	*77,1429*	*0*	*0*	*1*	*3*
0	*0*	*0*	*85,7143*	*0*	*8,57143*	*1*	*0*	*2*
0	*0*	*0*	*111,429*	*167,143*	*0*	*1*	*1*	*2*
0	*0*	*0*	*197,143*	*42,8571*	*42,8571*	*1*	*1*	*2*
1	*0*	*1*	*317,143*	*0*	*257,143*	*1*	*1*	*2*
0	*0*	*0*	*128,571*	*180*	*0*	*1*	*1*	*2*
0	*0*	*0*	*0*	*0*	*0*	*0*	*0*	*2*
0	*0*	*0*	*4,28571*	*72,8571*	*0*	*0*	*1*	*2*
0	*0*	*0*	*68,5714*	*214,286*	*0*	*1*	*1*	*1*
0	*0*	*0*	*28,5714*	*158,571*	*0*	*0*	*1*	*1*
0	*0*	*0*	*171,429*	*34,2857*	*0*	*1*	*0*	*2*
0	*0*	*0*	*154,286*	*175,714*	*8,57143*	*1*	*1*	*2*
0	*0*	*0*	*142,857*	*0*	*0*	*1*	*0*	*2*
0	*0*	*0*	*35,7143*	*8,57143*	*0*	*0*	*0*	*2*
0	*0*	*0*	*45*	*17,1429*	*0*	*0*	*0*	*2*
0	*0*	*0*	*5,71429*	*85,7143*	*0*	*0*	*1*	*2*
0	*0*	*0*	*231,429*	*17,8571*	*0*	*1*	*0*	*1*
1	*0*	*1*	*184,286*	*252,857*	*0*	*1*	*1*	*2*
0	*0*	*0*	*28,5714*	*8,57143*	*0*	*0*	*0*	*1*
0	*0*	*0*	*128,571*	*25,7143*	*0*	*1*	*0*	*1*
0	*0*	*0*	*47,1429*	*34,2857*	*25,7143*	*0*	*1*	*2*
0	*1*	*1*	*197,143*	*12,8571*	*51,4286*	*1*	*1*	*2*
0	*0*	*0*	*108,571*	*12,8571*	*0*	*1*	*0*	*2*
0	*0*	*0*	*75,7143*	*8,57143*	*25,7143*	*1*	*0*	*2*
0	*0*	*0*	*162,857*	*0*	*7,14286*	*1*	*0*	*1*
0	*0*	*0*	*20*	*8,57143*	*51,4286*	*0*	*1*	*1*
0	*0*	*0*	*17,1429*	*205,714*	*0*	*0*	*1*	*3*
0	*0*	*0*	*94,2857*	*4,28571*	*0*	*1*	*0*	*2*
0	*0*	*0*	*162,857*	*94,2857*	*25,7143*	*1*	*1*	*2*
0	*0*	*0*	*120*	*8,57143*	*0*	*1*	*0*	*2*
0	*0*	*0*	*2,85714*	*8,57143*	*0*	*0*	*0*	*2*
0	*0*	*0*	*205,714*	*68,5714*	*25,7143*	*1*	*1*	*2*
0	*0*	*0*	*188,571*	*137,143*	*0*	*1*	*1*	*1*
0	*0*	*0*	*145,714*	*158,571*	*0*	*1*	*1*	*2*
0	*0*	*0*	*46,4286*	*137,143*	*25,7143*	*0*	*1*	*2*
1	*0*	*1*	*8,57143*	*17,1429*	*102,857*	*0*	*1*	*2*
0	*0*	*0*	*51,4286*	*0*	*17,1429*	*0*	*0*	*1*
0	*1*	*1*	*235,714*	*0*	*12,8571*	*1*	*0*	*1*
0	*0*	*0*	*0*	*0*	*0*	*0*	*0*	*2*
0	*1*	*1*	*74,2857*	*68,5714*	*51,4286*	*1*	*1*	*2*
0	*0*	*0*	*31,4286*	*128,571*	*0*	*0*	*1*	*2*
0	*0*	*0*	*8,57143*	*38,5714*	*8,57143*	*0*	*0*	*2*
0	*0*	*0*	*0*	*188,571*	*0*	*0*	*1*	*2*
0	*0*	*0*	*60*	*115,714*	*102,857*	*1*	*1*	*2*
0	*0*	*0*	*32,1429*	*8,57143*	*0*	*0*	*0*	*2*
0	*0*	*0*	*96,4286*	*38,5714*	*0*	*1*	*0*	*2*

0	0	0	30	34,2857	0	0	0	2
0	0	1	25,7143	25,7143	17,1429	0	0	2
0	0	0	21,4286	25,7143	0	0	0	3
0	0	0	85,7143	102,857	0	1	1	2
0	0	0	27,1429	60	25,7143	0	1	2
0	0	0	0	85,7143	0	0	1	2
0	0	0	0	8,57143	0	0	0	2
0	0	0	8,57143	60	0	0	1	2
1	0	1	85,7143	141,429	0	1	1	2
0	0	0	115,714	171,429	0	1	1	2
0	0	0	120	0	0	1	0	2
0	0	0	154,286	180	0	1	1	3
0	0	0	0	160	94,2857	0	1	2
0	0	0	12,1429	17,1429	0	0	0	1
0	0	0	22,8571	6,42857	0	0	0	2
0	0	0	0	128,571	17,1429	0	1	2
0	0	0	31,4286	14,2857	0	0	0	2
0	0	0	25,7143	34,2857	0	0	0	2
0	0	0	150	7,14286	8,57143	1	0	3
0	0	0	248,571	231,429	0	1	1	2
0	0	0	171,429	25,7143	30	1	0	2
0	0	0	180	102,857	25,7143	1	1	2
0	0	0	55,7143	60	0	0	1	2
0	0	0	197,143	34,2857	111,429	1	1	1
0	0	0	137,143	14,2857	180	1	1	2
0	0	0	42,8571	128,571	17,1429	0	1	2
0	1	1	60	94,2857	8,57143	1	1	2
0	0	0	12,8571	25,7143	0	0	0	1
0	0	0	30	120	25,7143	0	1	2
0	0	0	21,4286	154,286	0	0	1	2
0	0	0	17,1429	77,1429	0	0	1	1
0	0	0	17,1429	25,7143	0	0	0	1
1	0	1	44,2857	85,7143	0	0	1	2
0	1	1	291,429	222,857	25,7143	1	1	1
0	0	0	11,4286	184,286	0	0	1	2
0	0	0	0	17,1429	8,57143	0	0	2
0	0	0	51,4286	12,8571	0	0	0	2
0	0	0	154,286	197,143	0	1	1	2
0	0	0	171,429	128,571	0	1	1	2
0	0	0	0	51,4286	0	0	0	2
0	0	0	0	0	51,4286	0	0	1
0	0	0	57,8571	127,143	102,857	0	1	2
0	0	0	154,286	128,571	0	1	1	1
0	0	0	214,286	51,4286	17,1429	1	1	1
0	0	0	2,85714	31,4286	30	0	1	2
0	0	0	7,14286	0	0	0	0	2
0	0	0	60	0	85,7143	1	1	2
0	0	0	0	83,5714	0	0	1	2

0	*0*	*0*	*0*	*34,2857*	*0*	*0*	*0*	*2*
0	*0*	*0*	*274,286*	*248,571*	*17,1429*	*1*	*1*	*1*
0	*0*	*0*	*8,57143*	*34,2857*	*0*	*0*	*0*	*1*
0	*0*	*0*	*51,4286*	*4,28571*	*34,2857*	*0*	*0*	*1*
0	*0*	*0*	*0*	*188,571*	*154,286*	*0*	*1*	*2*
0	*0*	*0*	*158,571*	*0*	*0*	*1*	*0*	*2*
0	*0*	*0*	*102,857*	*210*	*60*	*1*	*1*	*3*
0	*0*	*0*	*4,28571*	*17,1429*	*0*	*0*	*0*	*2*
0	*0*	*0*	*15,7143*	*0*	*38,5714*	*0*	*0*	*2*
0	*0*	*0*	*7,14286*	*181,429*	*0*	*0*	*1*	*2*
0	*0*	*0*	*132,857*	*77,1429*	*0*	*1*	*1*	*2*
0	*0*	*0*	*180*	*131,429*	*0*	*1*	*1*	*1*
0	*0*	*0*	*32,8571*	*137,143*	*0*	*0*	*1*	*2*
0	*1*	*1*	*216,429*	*85,7143*	*68,5714*	*1*	*1*	*1*
0	*0*	*0*	*25,7143*	*102,857*	*8,57143*	*0*	*1*	*2*
0	*0*	*0*	*110*	*14,2857*	*0*	*1*	*0*	*2*
0	*0*	*0*	*145,714*	*185,714*	*5,71429*	*1*	*1*	*2*
0	*0*	*0*	*145,714*	*8,57143*	*0*	*1*	*0*	*2*
0	*0*	*0*	*38,5714*	*4,28571*	*0*	*0*	*0*	*2*
0	*0*	*0*	*77,1429*	*180*	*0*	*1*	*1*	*2*
0	*0*	*0*	*0*	*0*	*0*	*0*	*0*	*2*
0	*0*	*0*	*124,286*	*38,5714*	*0*	*1*	*0*	*1*
0	*0*	*0*	*34,2857*	*8,57143*	*17,1429*	*0*	*0*	*2*
0	*0*	*0*	*145,714*	*240*	*0*	*1*	*1*	*2*
0	*0*	*0*	*42,8571*	*0*	*0*	*0*	*0*	*1*
0	*1*	*1*	*60*	*102,857*	*94,2857*	*1*	*1*	*2*
0	*0*	*0*	*27,8571*	*31,4286*	*0*	*0*	*0*	*2*
0	*0*	*0*	*102,857*	*8,57143*	*0*	*1*	*0*	*2*
0	*0*	*0*	*72,8571*	*0*	*0*	*1*	*0*	*3*
0	*0*	*0*	*158,571*	*145,714*	*17,1429*	*1*	*1*	*2*
0	*0*	*0*	*21,4286*	*102,857*	*0*	*0*	*1*	*2*
0	*0*	*0*	*0*	*8,57143*	*34,2857*	*0*	*0*	*2*
0	*0*	*0*	*48,5714*	*21,4286*	*0*	*0*	*0*	*1*
0	*0*	*0*	*85,7143*	*30*	*25,7143*	*1*	*0*	*2*
0	*0*	*0*	*8,57143*	*30*	*180*	*0*	*1*	*2*
0	*0*	*0*	*180*	*11,4286*	*77,1429*	*1*	*1*	*2*
0	*0*	*0*	*30*	*30*	*17,1429*	*0*	*0*	*2*
0	*0*	*0*	*0*	*41,4286*	*0*	*0*	*0*	*2*
0	*0*	*0*	*173,571*	*137,143*	*115,714*	*1*	*1*	*2*
0	*0*	*0*	*42,8571*	*128,571*	*51,4286*	*0*	*1*	*2*
0	*0*	*0*	*30*	*0*	*0*	*0*	*0*	*2*
0	*0*	*0*	*167,143*	*188,571*	*17,1429*	*1*	*1*	*2*
0	*1*	*1*	*281,429*	*214,286*	*25,7143*	*1*	*1*	*1*
0	*0*	*0*	*0*	*15*	*0*	*0*	*0*	*2*
0	*0*	*0*	*58,5714*	*40*	*0*	*0*	*0*	*2*
0	*0*	*0*	*0*	*128,571*	*0*	*0*	*1*	*2*
0	*0*	*0*	*137,143*	*8,57143*	*0*	*1*	*0*	*1*
0	*0*	*0*	*21,4286*	*18,5714*	*0*	*0*	*0*	*1*

0	0	0	137,143	25,7143	0	1	0	1
0	0	0	47,1429	85,7143	51,4286	0	1	2
0	0	0	23,5714	25,7143	0	0	0	2
0	0	0	12,1429	8,57143	0	0	0	1
0	0	0	6,42857	42,8571	25,7143	0	1	2
0	0	0	42,8571	21,4286	0	0	0	1
0	0	0	85,7143	162,857	8,57143	1	1	2
0	1	1	141,429	51,4286	0	1	0	1
0	0	0	21,4286	0	0	0	0	1
0	0	0	28,5714	17,1429	0	0	0	2
0	0	0	0	0	128,571	0	1	2
0	0	0	0	0	0	0	0	3
0	0	0	90	216,429	0	1	1	2
0	0	0	171,429	15	30	1	0	3
0	0	0	35,7143	334,286	25,7143	0	1	2
0	0	0	0	0	0	0	0	2
0	0	0	188,571	0	0	1	0	2
0	0	0	68,5714	28,5714	0	1	0	2
0	0	0	16,4286	34,2857	0	0	0	2
0	0	0	0	60	51,4286	0	1	2
0	0	0	0	0	17,1429	0	0	2
0	0	0	162,857	0	0	1	0	1
0	1	1	171,429	120	0	1	1	1
0	0	0	60	15	17,1429	1	0	1
0	0	0	171,429	77,1429	0	1	1	1
0	1	1	137,143	77,1429	0	1	1	2
0	0	0	128,571	51,4286	8,57143	1	1	3
0	0	0	60	214,286	154,286	1	1	2
0	0	0	0	17,1429	0	0	0	2
0	0	0	0	8,57143	4,28571	0	0	2
0	0	0	191	180	25,7143	1	1	3
0	0	0	71,4286	34,2857	0	1	0	2
0	0	0	218,571	85,7143	0	1	1	2
0	0	0	218,571	120	0	1	1	2
0	0	0	94,2857	141,429	0	1	1	2
0	0	0	58,5714	51,4286	51,4286	0	1	1
0	0	0	0	0	12,8571	0	0	1
0	0	0	128,571	205,714	0	1	1	2
0	0	0	2,85714	0	0	0	0	2
0	0	0	128,571	222,857	0	1	1	2
0	1	1	60	42,8571	0	1	0	2
0	0	1	214,286	188,571	0	1	1	2
0	0	0	17,1429	0	4,28571	0	0	2
0	0	0	0	137,143	0	0	1	2
0	0	0	128,571	130,714	0	1	1	2
0	0	0	0	154,286	0	0	1	2
0	0	1	68,5714	188,571	17,1429	1	1	2
0	0	0	115,714	25,7143	34,2857	1	1	2

0	0	0	32,1429	30	60	0	1	2
0	0	0	38,5714	30	17,1429	0	0	2
0	0	0	4,28571	112,857	154,286	0	1	1
0	0	0	20	61,4286	102,857	0	1	2
0	0	0	65,7143	0	0	1	0	1
0	0	0	4,28571	188,571	8,57143	0	1	2
0	0	0	65,7143	51,4286	0	1	0	2
0	1	1	68,5714	0	0	1	0	1
0	0	0	21,4286	135	0	0	1	2
0	0	0	97,1429	128,571	8,57143	1	1	2
0	0	0	37,1429	32,1429	0	0	0	2
0	0	0	77,1429	8,57143	0	1	0	2
0	0	0	68,5714	42,8571	51,4286	1	1	1
0	0	0	57,8571	145,714	0	0	1	2
0	0	0	134,286	132,857	0	1	1	2
0	0	0	102,857	23,5714	6,42857	1	0	1
0	0	0	168,571	60	77,1429	1	1	2
0	0	0	60	51,4286	51,4286	1	1	2
0	0	0	28,5714	128,571	0	0	1	1
1	0	1	30	94,2857	0	0	1	2
0	0	0	5,71429	0	0	0	0	2
0	0	0	51,4286	6,42857	0	0	0	2
0	0	0	128,571	85,7143	17,1429	1	1	2
0	0	0	137,143	66,4286	0	1	1	1
0	0	0	106,429	17,1429	0	1	0	2
0	0	1	308,571	317,143	0	1	1	2
0	0	0	38,5714	8,57143	0	0	0	2
0	0	0	30	25,7143	0	0	0	1
0	0	0	0	0	8,57143	0	0	2
0	0	0	0	8,57143	0	0	0	2
0	0	0	175,714	222,857	0	1	1	2
0	0	0	90	0	77,1429	1	1	2
0	0	0	34,2857	17,1429	0	0	0	2
0	0	0	77,1429	2,14286	0	1	0	1
0	0	0	34,2857	8,57143	0	0	0	2
0	0	0	0	157,143	0	0	1	1
0	0	0	22,8571	77,1429	0	0	1	3
0	0	0	128,571	17,1429	8,57143	1	0	1
0	0	0	32,1429	42,8571	0	0	0	1
0	0	0	28,5714	68,5714	0	0	1	2
0	0	0	6,85714	0	0	0	0	1
0	0	0	17,1429	8,57143	17,1429	0	0	2
0	0	0	42,8571	137,143	0	0	1	1
0	0	0	10	17,1429	0	0	0	2
0	0	0	23,5714	68,5714	0	0	1	1
0	0	0	205,714	270	180	1	1	2
0	0	0	0	4,28571	0	0	0	2
0	0	0	25,7143	154,286	25,7143	0	1	1

0	0	0	46,4286	17,1429	4,28571	0	0	1
0	0	0	128,571	138,571	17,1429	1	1	2
0	0	0	2,85714	60	0	0	1	1
0	0	0	188,571	231,429	25,7143	1	1	1
0	0	0	132,857	85,7143	0	1	1	1
0	0	0	214,286	145,714	60	1	1	2
0	0	0	61,4286	164,286	0	1	1	3
0	0	0	4,28571	62,1429	0	0	1	1
0	0	0	87,1429	90	60	1	1	2
0	1	1	265,714	42,8571	8,57143	1	0	1
0	0	0	85,7143	68,5714	51,4286	1	1	2
0	0	0	137,143	128,571	25,7143	1	1	1
0	0	0	0	4,28571	8,57143	0	0	2
0	1	1	150	102,857	68,5714	1	1	2
0	0	0	0	111,429	0	0	1	1
0	0	0	10,7143	17,1429	0	0	0	1
0	0	0	21,4286	94,2857	25,7143	0	1	1
0	0	0	124,286	0	0	1	0	2
0	0	0	0	102,857	128,571	0	1	2
1	0	1	4,28571	5,71429	85,7143	0	1	2
0	0	0	22,8571	30	30	0	1	2
0	0	0	0	21,4286	0	0	0	1
0	0	0	0	0	94,2857	0	1	2
0	1	1	297,143	308,571	0	1	1	2
0	0	0	231,429	308,571	102,857	1	1	2
0	0	0	5,71429	34,2857	0	0	0	2
0	1	1	128,571	0	17,1429	1	0	2
0	0	0	6,42857	37,1429	34,2857	0	1	2
0	0	0	38,5714	17,1429	0	0	0	3
0	0	0	42,8571	141,429	42,8571	0	1	2
0	0	0	128,571	105,714	0	1	1	2
0	0	0	14,2857	31,4286	8,57143	0	0	2
0	0	0	8,57143	0	0	0	0	1
0	0	0	37,1429	72,8571	0	0	1	2
0	0	0	14,2857	25,7143	25,7143	0	0	2
0	0	0	5,71429	102,857	0	0	1	1
0	0	0	27,1429	128,571	14,2857	0	1	1
0	0	0	48,5714	180	17,1429	0	1	2
0	0	0	0	12,8571	25,7143	0	0	2
0	1	1	107,143	25,7143	25,7143	1	0	1
0	0	0	77,1429	51,4286	25,7143	1	1	1
0	0	0	0	0	34,2857	0	0	2
0	0	0	120	162,857	25,7143	1	1	2
0	0	0	97,1429	57,8571	68,5714	1	1	1
0	0	1	120	70,7143	0	1	1	1
0	0	0	21,4286	34,2857	51,4286	0	1	2
0	1	1	60	21,4286	8,57143	1	0	2
0	0	0	30	2,14286	25,7143	0	0	2

0	0	0	8,57143	0	0	0	0	2
0	1	1	180	120	17,1429	1	1	2
0	0	0	180	227,143	25,7143	1	1	2
0	0	0	42,8571	188,571	42,8571	0	1	1
0	0	0	8,57143	38,5714	0	0	0	1
0	0	0	4,28571	0	0	0	0	2
0	0	0	178,571	0	25,7143	1	0	1
0	0	0	21,4286	17,1429	0	0	0	1
0	0	0	40	0	17,1429	0	0	2
0	0	0	11,4286	85,7143	0	0	1	2
0	0	0	25,7143	0	0	0	0	1
0	0	0	111,429	154,286	25,7143	1	1	2
0	0	0	120	12,8571	17,1429	1	0	2
0	0	0	0	25,7143	42,8571	0	1	1
0	0	0	128,571	42,8571	0	1	0	2
0	0	0	72,8571	120	51,4286	1	1	2
0	0	0	48,5714	23,5714	17,1429	0	0	2
0	0	0	70,7143	60	0	1	1	2
0	0	0	8,57143	0	0	0	0	2
0	0	0	7,14286	17,1429	25,7143	0	0	2
0	0	0	34,2857	128,571	0	0	1	2
0	0	0	128,571	8,57143	0	1	0	2
0	0	0	30	0	17,1429	0	0	2
0	0	0	17,1429	68,5714	0	0	1	2
0	0	0	77,1429	20	25,7143	1	0	2
0	0	0	257,143	360	0	1	1	2
0	0	0	37,1429	111,429	0	0	1	2
0	0	0	51,4286	0	17,1429	0	0	2
0	0	0	42,8571	0	68,5714	0	1	2
0	0	0	17,1429	39,2857	0	0	0	2
0	0	0	128,571	102,857	0	1	1	2
0	0	0	64,2857	8,57143	47,1429	1	0	2
0	0	1	222,857	34,2857	0	1	0	2
0	0	0	60	145,714	0	1	1	2
0	0	0	40	21,4286	0	0	0	2
0	0	0	20,7143	55	85,7143	0	1	1
0	0	0	0	155	25,7143	0	1	2
0	0	0	40	17,1429	0	0	0	1
0	0	0	13,5714	158,571	0	0	1	1
0	0	0	120	128,571	60	1	1	2
0	0	0	68,5714	25,7143	0	1	0	2
0	0	0	0	23,5714	0	0	0	2
0	0	0	100	231,429	0	1	1	2
0	0	0	4,28571	14,2857	4,28571	0	0	1
0	0	0	21,4286	162,857	8,57143	0	1	1
0	0	0	17,1429	0	0	0	0	1
0	0	0	205,714	64,2857	0	1	1	2
0	0	0	51,4286	51,4286	51,4286	0	1	2

0	0	0	197,143	252,857	17,1429	1	1	1
0	0	0	77,1429	8,57143	0	1	0	1
0	0	0	115,714	128,571	0	1	1	2
0	0	0	85,7143	162,857	25,7143	1	1	2
0	0	0	2,85714	17,1429	17,1429	0	0	2
0	0	0	2,14286	98,5714	0	0	1	2
0	0	0	23,5714	34,2857	0	0	0	2
0	0	0	8,57143	0	0	0	0	2
0	0	0	115,714	118,571	51,4286	1	1	1
0	0	0	308,571	257,143	17,1429	1	1	1
0	0	0	134,286	21,4286	0	1	0	2
0	0	0	128,571	128,571	8,57143	1	1	2
0	0	0	0	8,57143	0	0	0	2
0	0	1	92,1429	51,4286	0	1	0	2
0	0	0	111,429	35,7143	0	1	0	1
0	0	0	137,143	102,857	0	1	1	2
0	0	0	60	6,42857	25,7143	1	0	1
0	0	0	42,8571	57,1429	0	0	0	1
0	0	0	0	85,7143	0	0	1	2
0	0	0	4,28571	55,7143	0	0	0	1
0	0	0	6,42857	120	0	0	1	2
0	1	1	137,143	282,857	0	1	1	2
0	0	0	184,286	222,857	25,7143	1	1	1
0	1	1	145,714	0	0	1	0	1
0	0	0	0	47,1429	0	0	0	2
0	0	0	67,1429	25,7143	0	1	0	2
0	0	0	55,7143	0	0	0	0	2
0	0	0	55,7143	0	0	0	0	1
0	0	0	20	205,714	154,286	0	1	2
0	0	0	40	0	0	0	0	2
0	0	1	51,4286	17,1429	0	0	0	3
0	0	0	94,2857	180	25,7143	1	1	2
0	0	0	25,7143	20	0	0	0	2
0	0	0	11,4286	0	0	0	0	1
0	0	0	25,7143	0	10	0	0	1
0	0	0	142,857	51,4286	0	1	0	2
0	0	0	98,5714	197,143	34,2857	1	1	1
0	0	0	30	197,143	25,7143	0	1	2
0	0	0	17,1429	169,286	0	0	1	2
0	1	1	77,1429	51,4286	0	1	0	2
0	0	0	64,2857	117,143	0	1	1	2
0	0	0	42,8571	102,857	8,57143	0	1	1
0	0	0	137,143	60	0	1	1	2
0	0	0	0	8,57143	60	0	1	2
0	0	0	12,8571	17,1429	0	0	0	1
1	1	1	95,7143	57,1429	171,429	1	1	3
0	0	0	83,5714	4,28571	0	1	0	1
0	0	0	8,57143	57,1429	0	0	0	1

0	0	0	8,57143	8,57143	0	0	0	1
0	0	0	150	45,7143	0	1	0	2
0	0	0	45,7143	4,28571	0	0	0	2
0	1	1	180	132,857	102,857	1	1	2
0	0	0	265,714	60	0	1	1	1
0	0	0	128,571	102,857	128,571	1	1	2
0	0	0	180	102,857	0	1	1	1
0	0	0	88,5714	16,4286	0	1	0	1
0	0	0	2,85714	112,857	25,7143	0	1	2
0	0	0	68,5714	17,1429	0	1	0	2
0	0	0	0	100,714	0	0	1	2
0	0	0	2,14286	17,1429	0	0	0	3
0	0	0	0	162,857	120	0	1	1
0	0	0	120	240	34,2857	1	1	2
0	0	0	188,571	60	0	1	1	2
0	0	0	34,2857	214,286	0	0	1	2
0	0	0	48,5714	53,5714	0	0	0	2
0	0	0	50	85,7143	54,2857	0	1	2
0	0	0	130	175,714	0	1	1	2
0	0	1	34,2857	128,571	0	0	1	2
0	0	0	14,2857	42,8571	10,7143	0	0	2
0	0	0	139,286	7,14286	25,7143	1	0	2
0	0	0	109,286	30	42,8571	1	1	2
0	0	0	0	34,2857	0	0	0	2
0	0	0	37,1429	10,7143	0	0	0	2
0	1	1	107,143	42,8571	0	1	0	1
0	0	0	0	171,429	0	0	1	2
0	0	0	57,1429	92,1429	51,4286	0	1	2
0	0	0	60	107,143	0	1	1	3
0	0	0	167,143	85,7143	25,7143	1	1	2
0	0	0	31,4286	25,7143	21,4286	0	0	2
0	0	0	15	0	8,57143	0	0	2
0	0	0	154,286	102,857	25,7143	1	1	2
0	1	1	120	308,571	180	1	1	1
0	0	0	2,14286	42,8571	0	0	0	2
0	0	0	140	50	8,57143	1	0	1
0	0	0	65,7143	17,1429	0	1	0	2
0	0	0	15,7143	0	34,2857	0	0	1
0	0	0	0	0	0	0	0	2
0	0	0	167,143	14,2857	4,28571	1	0	1
0	0	0	102,857	60	0	1	1	2
0	0	0	14,2857	6,42857	0	0	0	1
0	0	0	100,714	0	0	1	0	1
0	0	0	85,7143	102,857	68,5714	1	1	2
0	0	0	145,714	0	17,1429	1	0	2
0	0	0	45,7143	23,5714	0	0	0	2
0	0	0	0	128,571	0	0	1	2
0	0	0	102,857	17,1429	0	1	0	1

0	1	1	214,286	25,7143	0	1	0	2
0	0	0	180	145,714	102,857	1	1	2
0	0	0	34,2857	0	0	0	0	2
0	0	0	10,7143	50	0	0	0	2
0	0	0	90	128,571	0	1	1	1
0	0	0	110	10,7143	0	1	0	2
0	0	0	34,2857	17,1429	17,1429	0	0	2
0	0	0	314,286	186,429	25,7143	1	1	1
0	0	0	47,1429	5,71429	8,57143	0	0	2
0	0	0	58,5714	51,4286	0	0	0	1
1	1	1	65,7143	90	4,28571	1	1	3
0	0	0	21,4286	29,2857	34,2857	0	1	1
0	0	0	20	77,1429	0	0	1	2
0	0	0	161,429	175,714	77,1429	1	1	2
0	0	1	51,4286	64,2857	34,2857	0	1	2
0	0	0	128,571	105,714	51,4286	1	1	2
0	0	1	17,1429	94,2857	0	0	1	1
0	0	0	32,1429	102,857	17,1429	0	1	2
0	0	0	20	188,571	180	0	1	2
0	0	0	0	120	25,7143	0	1	1
0	0	0	128,571	212,857	0	1	1	2
0	0	0	188,571	188,571	25,7143	1	1	2
1	1	1	180	42,8571	102,857	1	1	2
0	0	0	14,2857	18,5714	34,2857	0	0	2
0	0	0	0	34,2857	0	0	0	2
0	0	0	0	0	4,28571	0	0	2
0	0	0	17,1429	25,7143	30	0	0	2
0	0	0	10,7143	32,1429	0	0	0	2
0	0	0	0	117,143	45,7143	0	1	2
0	0	0	42,8571	12,8571	0	0	0	2
0	0	0	11,4286	8,57143	0	0	0	2
0	0	0	54,2857	77,1429	0	0	1	2
0	0	0	17,1429	25,7143	34,2857	0	1	2
0	0	0	192,857	308,571	0	1	1	1
0	0	0	128,571	0	25,7143	1	0	2
0	0	0	44,2857	30	120	0	1	2
0	0	0	14,2857	68,5714	0	0	1	2
0	0	0	141,429	7,14286	0	1	0	2
0	0	0	0	10	0	0	0	1
0	0	0	42,1429	12,8571	0	0	0	2
0	0	0	12,8571	25,7143	0	0	0	1
0	0	0	137,143	141,429	0	1	1	1
0	0	0	51,4286	55,7143	102,857	0	1	2
0	1	1	110	90	0	1	1	1
0	0	0	17,1429	77,1429	34,2857	0	1	2
0	0	1	42,8571	51,4286	0	0	0	2
0	0	0	30	8,57143	0	0	0	3
0	0	0	171,429	8,57143	2,85714	1	0	3

0	0	0	50	72,8571	25,7143	0	1	2
0	0	0	5,71429	25,7143	0	0	0	2
0	0	0	168,571	51,4286	21,4286	1	1	1
0	0	0	10	0	0	0	0	2
0	0	0	195,714	17,1429	128,571	1	1	1
0	0	0	141,429	34,2857	0	1	0	2
0	0	0	68,5714	122,857	25,7143	1	1	2
0	0	0	111,429	137,143	0	1	1	1
0	0	0	0	51,4286	0	0	0	1
0	0	0	128,571	154,286	25,7143	1	1	2
0	0	0	148,571	77,1429	0	1	1	1
0	0	1	154,286	128,571	0	1	1	3
0	0	0	188,571	128,571	0	1	1	2
0	1	1	94,2857	17,1429	0	1	0	1
0	0	0	34,2857	0	0	0	0	2
0	0	0	17,1429	25,7143	0	0	0	2
0	0	0	58,5714	98,5714	34,2857	0	1	1
0	0	0	17,1429	205,714	102,857	0	1	1
0	0	0	7,14286	28,5714	0	0	0	2
1	1	1	120	265,714	162,857	1	1	2
0	0	0	27,1429	0	0	0	0	1
0	0	0	21,4286	8,57143	0	0	0	2
0	0	0	0	0	60	0	1	2
0	0	0	77,1429	42,8571	25,7143	1	1	3
0	0	0	257,143	42,8571	0	1	0	2
0	0	1	184,286	77,1429	34,2857	1	1	2
0	0	0	0	214,286	51,4286	0	1	2
0	0	0	177,143	154,286	0	1	1	2
0	0	0	8,57143	85,7143	0	0	1	2
0	0	0	51,4286	0	0	0	0	1
0	0	0	248,571	270	180	1	1	2
0	0	0	115,714	17,1429	0	1	0	2
0	0	0	171,429	0	0	1	0	2
0	0	0	30	28,5714	0	0	0	2
0	1	1	154,286	102,857	0	1	1	2
0	0	0	42,8571	128,571	25,7143	0	1	2
0	0	0	0	72,8571	34,2857	0	1	2
0	0	0	85,7143	121,429	42,8571	1	1	2
0	1	1	72,8571	10	4,28571	1	0	2
0	0	0	138,571	115,714	90	1	1	1
0	0	0	1,42857	0	0	0	0	2
0	0	0	162,857	154,286	25,7143	1	1	2
0	0	0	8,57143	51,4286	0	0	0	2
0	0	0	72,8571	210	102,857	1	1	1
0	0	0	0	0	0	0	0	2
0	0	0	124,286	42,8571	0	1	0	2
0	1	1	402,857	0	0	1	0	2
0	0	0	182,857	107,143	77,1429	1	1	1

0	0	0	100,714	0	0	1	0	2
0	0	0	51,4286	0	0	0	0	1
0	1	1	68,5714	12,8571	0	1	0	2
0	0	0	158,571	21,4286	60	1	1	2
0	1	1	60	0	8,57143	1	0	2
0	1	1	60	21,4286	0	1	0	1
0	0	0	4,28571	51,4286	0	0	0	2
0	0	0	0	64,2857	0	0	1	2
0	0	0	5,71429	0	68,5714	0	1	2
0	0	0	0	53,5714	0	0	0	2
0	0	0	162,857	308,571	42,8571	1	1	2
0	0	0	54,2857	36,4286	102,857	0	1	1
0	0	0	21,4286	154,286	0	0	1	2
0	0	0	30	51,4286	0	0	0	2
0	0	0	8,57143	231,429	51,4286	0	1	1
0	0	0	0	0	6,42857	0	0	2
0	0	0	0	31,4286	0	0	0	1
0	0	0	51,4286	154,286	0	0	1	1
0	0	0	72,8571	17,1429	25,7143	1	0	2
0	0	0	107,143	60	8,57143	1	1	2
0	0	0	0	14,2857	17,1429	0	0	2
0	0	0	17,8571	17,1429	0	0	0	2
0	1	1	120	51,4286	51,4286	1	1	2
0	0	0	20	0	34,2857	0	0	2
0	0	0	8,57143	2,85714	0	0	0	3
0	1	1	68,5714	0	0	1	0	2
0	0	0	55,7143	5,71429	0	0	0	3
0	0	0	21,4286	0	42,8571	0	0	2
0	0	0	300	8,57143	17,1429	1	0	2
0	0	0	0	51,4286	0	0	0	1
0	1	1	85,7143	0	0	1	0	1
0	0	0	15,7143	8,57143	0	0	0	1
1	0	1	0	60	180	0	1	1
0	0	0	14,2857	140	0	0	1	2
0	0	0	0	38,5714	0	0	0	2
0	0	0	167,143	102,857	0	1	1	2
0	0	0	42,8571	4,28571	0	0	0	2
0	0	0	51,4286	8,57143	51,4286	0	1	1
0	1	1	405	17,1429	15,7143	1	0	2
0	0	0	111,429	0	0	1	0	2
0	1	1	107,143	11,4286	0	1	0	2
0	0	0	5,71429	17,1429	8,57143	0	0	2
0	1	1	207,857	231,429	25,7143	1	1	2
0	0	0	60	128,571	17,1429	1	1	2
0	0	0	40,7143	8,57143	0	0	0	1
0	0	0	128,571	0	0	1	0	2
0	0	0	180	154,286	17,1429	1	1	2
0	0	0	60	145,714	0	1	1	2

0	0	0	120	30	0	1	0	1
0	0	0	180	25,7143	8,57143	1	0	1
0	0	0	42,8571	25,7143	0	0	0	2
0	0	0	50	257,143	25,7143	0	1	2
0	0	0	158,571	137,143	17,1429	1	1	3
0	0	0	23,5714	32,8571	0	0	0	1
0	0	0	0	0	8,57143	0	0	1
0	0	0	0	4,28571	0	0	0	1
0	0	0	17,1429	120	77,1429	0	1	2
0	0	0	0	0	0	0	0	2
0	0	0	155,714	77,1429	0	1	1	1
0	0	0	32,1429	47,1429	0	0	0	2
0	0	0	68,5714	25,7143	0	1	0	1
0	0	0	8,57143	82,8571	47,1429	0	1	2
0	0	0	0	0	0	0	0	2
0	0	0	151,429	94,2857	0	1	1	2
0	0	0	92,8571	97,1429	0	1	1	2
0	0	0	5,71429	77,1429	0	0	1	2
0	0	0	8,57143	0	0	0	0	2
0	0	0	140	154,286	0	1	1	1
0	0	0	81,4286	143,571	34,2857	1	1	1
0	0	0	17,1429	62,1429	0	0	1	1
0	0	0	10	134,286	0	0	1	2
0	0	0	52,8571	51,4286	137,143	0	1	1
0	0	0	0	171,429	25,7143	0	1	2
0	0	0	188,571	188,571	94,2857	1	1	2
0	0	0	0	68,5714	0	0	1	2
0	0	0	32,1429	45	25,7143	0	1	2
0	0	0	8,57143	10,7143	0	0	0	2
0	0	0	282,857	101,429	0	1	1	2
0	0	0	87,8571	132,857	0	1	1	2
0	0	0	0	34,2857	77,1429	0	1	1
0	0	0	0	0	0	0	0	2
0	1	1	60	0	0	1	0	2
0	0	0	128,571	4,28571	0	1	0	2
0	0	0	137,143	197,143	77,1429	1	1	2
0	0	0	48,5714	17,1429	0	0	0	2
0	0	0	17,1429	85,7143	25,7143	0	1	2
0	0	0	55,7143	15	0	0	0	1
0	0	0	25,7143	81,4286	0	0	1	2
0	0	0	0	308,571	0	0	1	2
0	1	1	77,1429	162,857	0	1	1	2
0	0	0	64,2857	128,571	51,4286	1	1	2
0	0	0	154,286	0	25,7143	1	0	2
0	0	0	8,57143	34,2857	0	0	0	2
0	0	0	162,857	102,857	25,7143	1	1	2
1	0	1	51,4286	8,57143	145,714	0	1	2
0	0	0	0	77,1429	25,7143	0	1	1

0	0	0	20	14,2857	12,8571	0	0	2
0	0	0	187,143	25,7143	0	1	0	2
0	0	0	0	188,571	0	0	1	2
0	0	0	0	68,5714	0	0	1	2
0	0	0	117,143	17,1429	0	1	0	2
0	0	0	0	51,4286	0	0	0	2
0	0	0	162,857	60	51,4286	1	1	2
0	0	0	34,2857	314,286	17,1429	0	1	2
0	0	0	68,5714	0	17,1429	1	0	1
0	0	0	30	12,8571	0	0	0	2
0	0	0	0	0	25,7143	0	0	2
0	0	0	0	0	0	0	0	1
0	0	0	0	0	8,57143	0	0	2
0	0	0	81,4286	282,857	0	1	1	2
0	0	0	171,429	137,143	25,7143	1	1	2
0	0	0	145,714	68,5714	0	1	1	2
0	0	0	8,57143	11,4286	0	0	0	2
0	1	1	102,857	0	0	1	0	1
0	0	0	0	17,1429	0	0	0	3
1	0	1	14,2857	87,8571	0	0	1	2
0	0	0	180	18,5714	25,7143	1	0	2
0	0	0	5,71429	42,8571	0	0	0	2
0	0	0	147,143	85,7143	0	1	1	2
0	0	0	131,429	5,71429	0	1	0	1
0	0	0	154,286	231,429	0	1	1	2
0	0	0	132,857	128,571	128,571	1	1	3
0	0	0	148,571	42,8571	8,57143	1	0	2
0	0	0	72,8571	47,1429	0	1	0	1
0	0	0	145,714	128,571	25,7143	1	1	2
0	0	0	85,7143	217,143	25,7143	1	1	2
0	0	0	20	171,429	0	0	1	2
0	0	1	77,1429	171,429	51,4286	1	1	2
0	0	0	17,1429	18,5714	4,28571	0	0	2
0	0	0	96,4286	4,28571	0	1	0	2
0	1	1	197,857	21,4286	0	1	0	2
0	0	0	4,28571	102,857	8,57143	0	1	2
0	0	0	77,1429	68,5714	25,7143	1	1	2
0	0	0	25,7143	57,1429	0	0	0	2
0	0	0	8,57143	77,1429	0	0	1	1
0	0	0	28,5714	34,2857	51,4286	0	1	2
0	0	0	154,286	162,857	0	1	1	2
0	0	0	10,7143	55,7143	0	0	0	2
0	0	0	102,857	25,7143	0	1	0	1
0	0	0	8,57143	42,8571	5,71429	0	0	1
0	0	0	102,857	0	122,857	1	1	2
0	0	0	8,57143	42,8571	0	0	0	2
0	0	0	164,286	250	8,57143	1	1	2
0	0	0	188,571	141,429	25,7143	1	1	2

0	0	1	214,286	8,57143	51,4286	1	1	2
0	0	0	60	0	0	1	0	2
0	0	0	17,1429	51,4286	17,1429	0	1	2
0	0	0	15,7143	8,57143	8,57143	0	0	2
0	0	0	128,571	25,7143	0	1	0	2
0	0	0	11,4286	4,28571	0	0	0	2
0	0	0	50,7143	0	0	0	0	1
0	0	0	90	4,28571	0	1	0	2
0	0	0	0	0	0	0	0	2
0	0	0	30	85,7143	51,4286	0	1	2
0	0	0	182,143	197,143	0	1	1	2
0	0	0	188,571	214,286	17,1429	1	1	2
0	0	0	141,429	68,5714	0	1	1	2
0	0	0	85,7143	65,7143	0	1	1	2
0	0	0	17,1429	160	4,28571	0	1	1
0	0	0	7,14286	17,1429	0	0	0	2
0	0	1	205,714	205,714	25,7143	1	1	2
0	0	0	180	295,714	0	1	1	2
0	0	0	36,4286	154,286	25,7143	0	1	2
0	0	0	100,714	180	0	1	1	2
0	0	0	20	4,28571	17,1429	0	0	1
0	1	1	122,857	60	8,57143	1	1	3
0	0	1	102,857	17,1429	0	1	0	2
0	0	0	150	214,286	94,2857	1	1	2
0	0	0	42,1429	34,2857	0	0	0	2
0	0	0	0	0	0	0	0	2
0	0	0	19,2857	180	25,7143	0	1	2
0	0	1	108,571	34,2857	0	1	0	1
0	0	0	10,7143	68,5714	0	0	1	2
0	0	0	128,571	205,714	0	1	1	2
0	0	0	165,714	162,857	34,2857	1	1	2
0	0	0	50	42,8571	34,2857	0	1	2
0	0	1	36,4286	10	68,5714	0	1	1
0	0	0	14,2857	8,57143	0	0	0	1
0	0	0	64,2857	25,7143	0	1	0	1
0	0	0	42,8571	42,8571	0	0	0	2
0	0	0	128,571	225,714	51,4286	1	1	2
0	0	0	158,571	102,857	0	1	1	2
0	0	0	0	51,4286	25,7143	0	1	3
0	0	0	180	90	51,4286	1	1	2
0	0	0	102,857	17,1429	0	1	0	1
0	0	0	141,429	162,857	34,2857	1	1	2
0	0	0	154,286	274,286	0	1	1	2
0	0	0	109,286	85,7143	17,1429	1	1	1
0	0	0	17,1429	180	25,7143	0	1	2
0	0	0	188,571	250	0	1	1	2
0	0	0	15,7143	34,2857	0	0	0	2
0	0	0	175,714	185,714	51,4286	1	1	2

0	0	0	38,5714	25,7143	51,4286	0	1	2
0	0	0	8,57143	154,286	17,1429	0	1	2
0	0	0	12,8571	92,1429	0	0	1	2
0	0	0	21,4286	42,8571	12,8571	0	0	2
0	0	0	30	77,1429	0	0	1	2
0	0	0	128,571	128,571	12,8571	1	1	3
0	0	0	167,143	274,286	25,7143	1	1	2
0	0	0	51,4286	21,4286	45,7143	0	1	2
0	0	0	0	68,5714	4,28571	0	1	2
0	1	1	240	0	0	1	0	1
0	0	0	37,1429	8,57143	51,4286	0	1	2
0	0	0	139,286	77,1429	0	1	1	2
0	0	0	17,1429	98,5714	0	0	1	2
0	0	0	25,7143	11,4286	0	0	0	2
0	0	0	58,5714	180	0	0	1	2
0	0	0	188,571	26,4286	0	1	0	2
0	0	0	34,2857	17,1429	0	0	0	2
0	0	0	150	64,2857	0	1	1	2
0	0	0	111,429	205,714	51,4286	1	1	2
0	0	0	25,7143	5,71429	0	0	0	2
0	0	0	167,143	60	25,7143	1	1	2
0	0	0	20	55,7143	51,4286	0	1	2
0	1	1	274,286	154,286	8,57143	1	1	2
0	0	0	42,8571	0	21,4286	0	0	2
0	0	0	231,429	171,429	0	1	1	1
0	0	0	70,7143	37,1429	17,1429	1	0	2
0	0	0	30	0	111,429	0	1	2
0	0	0	0	180	8,57143	0	1	2
0	1	1	184,286	162,857	0	1	1	1
0	0	0	25,7143	0	0	0	0	2
0	0	0	180	222,857	25,7143	1	1	2
0	0	0	2,85714	21,4286	0	0	0	2
0	0	0	137,143	31,4286	8,57143	1	0	2
0	0	0	124,286	51,4286	34,2857	1	1	2
0	0	0	105,714	31,4286	25,7143	1	0	1
0	0	0	167,143	218,571	5,71429	1	1	2
0	0	0	190,714	42,8571	0	1	0	3
0	0	0	42,8571	8,57143	8,57143	0	0	2
0	0	0	34,2857	111,429	0	0	1	1
0	0	0	18,5714	0	0	0	0	2
0	0	0	98,5714	10,7143	0	1	0	1
0	0	0	77,1429	17,1429	0	1	0	1
0	0	0	70,7143	51,4286	30	1	1	2
0	0	0	128,571	171,429	28,5714	1	1	2
0	0	0	72,8571	22,8571	0	1	0	2
0	0	0	135,714	150	0	1	1	1
0	0	0	42,8571	17,1429	0	0	0	2
0	0	0	42,8571	145,714	42,8571	0	1	2

0	0	0	191,429	17,1429	0	1	0	2
0	0	0	81,4286	17,1429	12,8571	1	0	1
0	0	0	0	60	0	0	1	1
0	0	0	0	0	0	0	0	2
0	0	0	34,2857	51,4286	25,7143	0	1	1
0	0	0	0	4,28571	51,4286	0	0	1
1	0	1	228,571	231,429	34,2857	1	1	2
0	0	0	51,4286	51,4286	0	0	0	1
0	0	0	35,7143	0	0	0	0	2
0	0	0	30	17,1429	0	0	0	1
0	0	0	8,57143	51,4286	0	0	0	1
0	0	0	128,571	85,7143	0	1	1	1
0	0	0	5,71429	8,57143	0	0	0	2
0	1	1	111,429	80	0	1	1	1
0	0	0	60	171,429	34,2857	1	1	2
0	0	0	30	28,5714	0	0	0	1
0	0	0	67,1429	22,8571	0	1	0	2
0	0	0	30	70	17,1429	0	1	2
0	0	1	0	62,8571	0	0	1	2
0	1	1	165	11,4286	0	1	0	2
0	0	0	21,4286	47,1429	0	0	0	2
0	0	0	175,714	68,5714	0	1	1	2
0	0	0	17,1429	8,57143	0	0	0	2
0	0	0	140,714	282,857	0	1	1	2
0	0	0	17,8571	0	0	0	0	2
0	0	0	0	154,286	0	0	1	1
0	0	0	0	171,429	0	0	1	2
0	0	0	0	141,429	25,7143	0	1	2
0	0	0	8,57143	231,429	25,7143	0	1	2
0	0	0	0	120	0	0	1	2
0	1	1	102,857	85,7143	0	1	1	1
0	0	0	17,1429	81,4286	0	0	1	1
0	0	0	0	85,7143	0	0	1	2
0	0	0	162,857	167,143	0	1	1	2
0	0	0	0	51,4286	0	0	0	2
0	1	1	102,857	0	8,57143	1	0	2
0	0	0	42,8571	77,1429	0	0	1	2
0	1	1	312,857	107,143	0	1	1	2
0	0	1	114,286	17,1429	22,8571	1	0	2
0	0	0	154,286	7,14286	0	1	0	2
0	0	0	45	48,5714	11,4286	0	1	1
0	0	0	0	25,7143	25,7143	0	0	2
0	0	0	85,7143	120	0	1	1	1
0	0	0	12,8571	0	0	0	0	2
0	0	0	60	128,571	77,1429	1	1	2
0	0	0	28,5714	68,5714	0	0	1	2
0	0	1	145,714	137,143	17,1429	1	1	2
0	0	0	180	132,857	0	1	1	2

0	0	0	4,28571	77,1429	51,4286	0	1	2
0	0	0	8,57143	0	0	0	0	2
0	0	0	42,8571	0	0	0	0	2
0	1	1	300	51,4286	0	1	0	1
0	0	0	187,143	51,4286	51,4286	1	1	2
0	0	0	162,857	128,571	0	1	1	1
0	0	0	175,714	257,143	17,1429	1	1	2
0	0	0	47,1429	128,571	0	0	1	1
0	0	0	214,286	308,571	85,7143	1	1	2
0	0	0	128,571	94,2857	120	1	1	2
0	0	0	120	4,28571	25,7143	1	0	2
0	0	0	21,4286	42,8571	17,1429	0	1	1
0	0	0	17,1429	0	0	0	0	2
0	0	0	145,714	377,143	102,857	1	1	2
0	0	0	25,7143	128,571	17,1429	0	1	2
0	0	0	0	85,7143	0	0	1	1
0	0	0	0	17,1429	0	0	0	2
0	0	0	154,286	154,286	180	1	1	2
0	0	0	0	192,857	51,4286	0	1	2
0	0	0	34,2857	0	0	0	0	2
0	0	0	128,571	257,143	180	1	1	2
0	0	0	184,286	10	25,7143	1	0	1
0	0	0	35,7143	5,71429	0	0	0	1
0	0	0	68,5714	38,5714	102,857	1	1	1
0	0	0	132,857	201,429	0	1	1	2
0	1	1	300	0	0	1	0	2
0	1	1	102,857	228,571	0	1	1	2
0	0	0	145,714	147,143	0	1	1	2
0	1	1	360	34,2857	6,42857	1	0	2
0	0	0	42,8571	60	17,1429	0	1	2
0	0	0	17,1429	72,8571	0	0	1	2
0	0	0	5,71429	42,8571	0	0	0	2
0	1	1	60	154,286	0	1	1	2
0	0	0	51,4286	0	0	0	0	2
0	0	0	28,5714	57,1429	0	0	0	1
0	0	0	17,1429	34,2857	0	0	0	2
0	0	0	38,5714	17,1429	25,7143	0	0	2
0	0	0	158,571	168,571	0	1	1	2
0	0	0	47,1429	57,8571	0	0	0	2
0	0	0	131,429	162,857	0	1	1	1
0	0	0	2,85714	67,8571	0	0	1	2
0	0	0	102,857	132,857	8,57143	1	1	2
0	0	0	160	257,143	154,286	1	1	2
0	0	0	85,7143	124,286	51,4286	1	1	2
0	1	1	124,286	25,7143	0	1	0	3
0	0	0	57,1429	49,2857	0	0	0	2
0	0	0	156,429	68,5714	25,7143	1	1	2
0	0	0	128,571	77,1429	25,7143	1	1	3

0	0	0	30	0	102,857	0	1	2
0	0	0	10,7143	2,85714	0	0	0	2
0	0	0	203,571	17,1429	51,4286	1	1	1
0	0	0	4,28571	0	0	0	0	2
0	0	1	42,8571	17,1429	0	0	0	2
0	0	0	122,857	128,571	0	1	1	2
0	0	0	51,4286	0	0	0	0	2
0	0	0	55,7143	17,1429	25,7143	0	0	2
0	1	1	188,571	60	25,7143	1	1	1
0	0	0	40	0	15	0	0	1
0	0	0	11,4286	102,857	0	0	1	2
0	0	0	62,8571	36,4286	0	1	0	2
0	0	0	90	0	0	1	0	2
0	0	0	0	10,7143	8,57143	0	0	2
0	0	0	4,28571	154,286	0	0	1	3
0	0	0	17,1429	42,8571	60	0	1	2
0	0	0	154,286	85,7143	8,57143	1	1	2
0	0	0	102,857	8,57143	0	1	0	2
1	0	1	71,4286	201,429	90	1	1	2
0	0	0	120	12,8571	0	1	0	1
0	0	0	32,1429	68,5714	0	0	1	2
0	0	0	261,429	85,7143	4,28571	1	1	2
0	0	0	0	0	17,1429	0	0	1
0	0	0	55,7143	17,1429	0	0	0	2
0	0	0	158,571	30	0	1	0	1
0	0	0	42,8571	102,857	0	0	1	2
0	0	0	90	51,4286	0	1	0	2
0	0	0	27,1429	0	55,7143	0	0	1
0	0	0	0	25,7143	0	0	0	2
0	0	0	25,7143	0	0	0	0	1
0	0	0	21,4286	12,8571	17,1429	0	0	2
0	0	0	51,4286	85,7143	0	0	1	2
0	0	0	1,42857	34,2857	0	0	0	1
0	0	0	157,143	128,571	0	1	1	2
0	0	0	35	82,8571	0	0	1	2
0	0	0	0	154,286	102,857	0	1	2
0	0	0	42,8571	21,4286	0	0	0	1
0	0	0	12,8571	0	0	0	0	1
0	0	0	91,4286	0	0	1	0	1
0	0	0	0	168,571	17,1429	0	1	2
0	0	0	0	180	0	0	1	3
0	0	0	49,2857	11,4286	0	0	0	1
0	0	0	31,4286	18,5714	0	0	0	2
0	0	0	14,2857	0	38,5714	0	0	3
0	0	0	128,571	17,1429	111,429	1	1	2
0	0	0	20	85,7143	0	0	1	1
0	0	0	0	48,5714	0	0	0	2
0	0	0	8,57143	0	0	0	0	2

0	0	0	10	0	0	0	0	2
0	0	0	60	81,4286	15	1	1	2
0	0	0	60	27,1429	0	1	0	1
0	0	0	0	165	0	0	1	1
0	1	1	218,571	71,4286	0	1	1	3
0	0	0	158,571	147,143	0	1	1	2
0	0	0	248,571	214,286	68,5714	1	1	2
0	0	0	145,714	51,4286	25,7143	1	1	2
0	0	0	154,286	30	0	1	0	1
0	0	0	134,286	102,857	0	1	1	2
0	0	0	218,571	77,1429	0	1	1	2
0	0	0	128,571	188,571	0	1	1	1
0	0	1	158,571	60	25,7143	1	1	2
1	0	1	0	197,143	128,571	0	1	2
0	0	0	77,1429	60	0	1	1	1
0	0	0	2,14286	10,7143	8,57143	0	0	1
0	0	0	34,2857	25,7143	0	0	0	2
0	0	0	124,286	0	0	1	0	2
0	0	0	8,57143	30	2,85714	0	0	2
0	0	0	12,8571	21,4286	0	0	0	2
0	0	0	64,2857	11,4286	0	1	0	2
0	0	0	60	47,1429	0	1	0	1
0	0	0	8,57143	12,8571	0	0	0	2
0	0	0	137,143	25,7143	0	1	0	1
0	0	0	111,429	6,42857	0	1	0	2
0	0	0	27,8571	25,7143	8,57143	0	0	2
0	0	0	88,5714	8,57143	0	1	0	1
0	0	0	25,7143	38,5714	0	0	0	3
0	0	0	102,857	0	0	1	0	2
0	0	0	154,286	162,857	68,5714	1	1	2
1	1	1	120	90	60	1	1	2
0	0	0	17,1429	0	0	0	0	2
1	0	1	162,857	68,5714	51,4286	1	1	2
0	0	0	128,571	8,57143	25,7143	1	0	2
0	0	0	42,8571	162,857	0	0	1	2
0	0	0	120	25,7143	34,2857	1	1	1
0	0	0	8,57143	0	77,1429	0	1	1
0	0	0	0	132,857	180	0	1	2
0	0	1	111,429	184,286	25,7143	1	1	2
0	0	0	68,5714	171,429	25,7143	1	1	1
0	0	0	11,4286	51,4286	0	0	0	2
0	0	0	85,7143	205,714	0	1	1	1
0	0	0	5,71429	17,1429	0	0	0	3
0	0	0	214,286	154,286	17,1429	1	1	1
0	0	0	0	34,2857	0	0	0	1
0	0	0	68,5714	102,857	0	1	1	2
0	0	0	21,4286	0	8,57143	0	0	1
0	0	0	25,7143	37,1429	17,1429	0	0	2

0	0	0	34,2857	8,57143	0	0	0	1
0	0	0	17,1429	0	45	0	0	2
0	0	0	154,286	102,857	51,4286	1	1	2
0	0	0	45,7143	25,7143	25,7143	0	0	3
0	0	0	180	34,2857	51,4286	1	1	1
0	0	0	38,5714	67,1429	25,7143	0	1	2
0	1	1	368,571	34,2857	0	1	0	2
0	0	0	81,4286	25,7143	25,7143	1	0	1
0	0	0	128,571	0	8,57143	1	0	2
0	0	0	25,7143	68,5714	68,5714	0	1	2
0	0	0	51,4286	12,8571	17,1429	0	0	1
0	0	1	195	154,286	65,7143	1	1	1
0	0	0	175,714	257,143	94,2857	1	1	2
0	0	0	151,429	120	17,1429	1	1	2
0	0	0	60	32,1429	0	1	0	2
0	0	0	45,7143	94,2857	0	0	1	3
0	0	0	4,28571	83,5714	0	0	1	1
0	0	0	57,1429	0	11,4286	0	0	1
0	0	0	137,143	137,857	34,2857	1	1	2
0	0	0	210	51,4286	17,1429	1	1	1
0	0	0	17,1429	0	0	0	0	1
0	0	0	162,857	19,2857	34,2857	1	0	3
0	0	0	8,57143	201,429	0	0	1	2
0	0	0	135	188,571	18,5714	1	1	2
0	0	0	111,429	8,57143	2,85714	1	0	3
0	1	1	105,714	15	0	1	0	3
0	0	0	141,429	100,714	66,4286	1	1	1
1	0	1	71,4286	85,7143	180	1	1	1
0	0	0	0	28,5714	0	0	0	1
0	1	1	360	30	0	1	0	1
0	0	0	49,2857	64,2857	0	0	1	2
0	0	0	8,57143	0	8,57143	0	0	2
0	0	0	45	85,7143	34,2857	0	1	2
0	0	0	25,7143	23,5714	0	0	0	2
0	0	0	18,5714	36,4286	0	0	0	1
0	0	0	4,28571	0	0	0	0	2
0	0	0	5,71429	34,2857	4,28571	0	0	2
0	0	0	0	17,1429	0	0	0	2
0	0	1	150	128,571	0	1	1	2
0	0	0	5,71429	25,7143	0	0	0	2
0	0	0	12,8571	4,28571	0	0	0	1
1	0	1	55,7143	4,28571	64,2857	0	1	2
0	0	0	29,2857	147,857	8,57143	0	1	3
0	0	0	0	231,429	0	0	1	2
0	0	0	38,5714	25,7143	0	0	0	2
1	0	1	38,5714	68,5714	34,2857	0	1	2
0	0	0	0	17,1429	85,7143	0	1	2
0	0	0	11,4286	17,1429	0	0	0	2

0	0	0	51,4286	34,2857	0	0	0	2
0	0	0	0	51,4286	0	0	0	1
0	0	0	37,1429	51,4286	0	0	0	1
1	0	1	38,5714	128,571	0	0	1	2
0	0	0	31,4286	0	0	0	0	2
0	0	0	141,429	155,714	0	1	1	1
0	0	0	175,714	0	0	1	0	2
0	0	0	85,7143	25,7143	0	1	0	2
0	0	0	137,143	128,571	25,7143	1	1	2
0	0	1	77,1429	77,1429	34,2857	1	1	2
0	0	0	64,2857	0	0	1	0	3
0	0	0	17,1429	28,5714	34,2857	0	1	2
0	0	0	91,4286	94,2857	0	1	1	3
0	0	0	98,5714	127,143	17,1429	1	1	1
0	0	0	128,571	0	51,4286	1	0	2
0	0	0	60	10,7143	0	1	0	2
0	0	0	0	34,2857	0	0	0	2
0	0	0	171,429	137,143	0	1	1	1
0	0	0	0	68,5714	0	0	1	2
0	0	0	90	12,8571	25,7143	1	0	2
0	0	0	0	32,8571	0	0	0	2
0	0	0	90	88,5714	17,1429	1	1	2
0	0	0	28,5714	25,7143	0	0	0	1
0	0	0	188,571	158,571	0	1	1	2
0	0	0	60	8,57143	0	1	0	2
0	0	0	0	257,143	0	0	1	2
0	0	0	14,2857	145,714	0	0	1	2
0	1	1	107,143	111,429	0	1	1	1
0	0	0	10,7143	34,2857	0	0	0	2
0	0	0	42,8571	0	0	0	0	1
0	0	0	77,1429	77,1429	0	1	1	2
0	0	0	10	35,7143	0	0	0	2
0	1	1	102,857	180	0	1	1	1
0	0	0	47,1429	34,2857	34,2857	0	1	2
0	1	1	120	2,14286	0	1	0	2
0	0	0	17,1429	8,57143	8,57143	0	0	1
0	0	0	0	0	102,857	0	1	1
0	0	0	182,857	102,857	25,7143	1	1	1
0	0	0	25,7143	51,4286	8,57143	0	1	1
0	0	0	180	180	0	1	1	2
0	0	0	8,57143	214,286	102,857	0	1	2
0	0	0	45,7143	120	0	0	1	2
0	0	0	151,429	0	38,5714	1	0	2
0	0	0	12,8571	0	25,7143	0	0	2
0	0	0	55,7143	137,143	0	0	1	2
0	0	0	120	248,571	34,2857	1	1	1
0	0	0	154,286	107,143	0	1	1	1
0	0	0	34,2857	34,2857	37,7143	0	1	2

0	0	0	0	128,571	77,1429	0	1	2
0	0	0	64,2857	167,143	0	1	1	2
0	0	0	282,857	77,1429	0	1	1	1
0	0	0	42,8571	25,7143	85,7143	0	1	2
0	0	0	17,1429	145,714	0	0	1	1
0	0	0	128,571	0	0	1	0	2
0	0	0	102,857	34,2857	25,7143	1	1	2
0	0	0	14,2857	8,57143	0	0	0	2
0	0	0	60	77,1429	0	1	1	2
0	0	0	120	162,857	17,1429	1	1	2
0	0	0	128,571	42,8571	68,5714	1	1	2
0	0	0	120	42,8571	8,57143	1	0	2
0	0	0	257,143	257,143	85,7143	1	1	2
0	0	0	0	17,1429	0	0	0	2
0	0	0	7,14286	8,57143	0	0	0	2
0	0	0	0	154,286	0	0	1	2
0	0	0	171,429	154,286	0	1	1	2
0	0	0	5,71429	12,8571	0	0	0	2
0	0	0	66,4286	6,42857	0	1	0	2
0	0	0	8,57143	8,57143	0	0	0	2
0	0	0	40	17,1429	0	0	0	2
0	1	1	180	85,7143	60	1	1	2
0	1	1	195,714	90	42,8571	1	1	2
0	1	1	212,143	0	0	1	0	1
0	0	0	25,7143	77,1429	25,7143	0	1	2
0	0	0	7,85714	20	0	0	0	2
0	0	0	102,857	31,4286	8,57143	1	0	1
0	0	0	14,2857	0	77,1429	0	1	2
0	0	0	8,57143	0	25,7143	0	0	2
0	0	0	87,8571	188,571	25,7143	1	1	2
0	0	0	81,4286	34,2857	0	1	0	2
0	0	0	62,8571	0	0	1	0	2
0	0	0	12,8571	17,1429	25,7143	0	0	1
0	0	0	154,286	128,571	0	1	1	2
0	0	1	102,857	107,143	0	1	1	2
0	1	1	488,571	38,5714	0	1	0	2
0	0	1	65,7143	34,2857	34,2857	1	1	2
0	1	1	60	52,8571	8,57143	1	1	3
0	0	0	180	248,571	205,714	1	1	1
0	0	0	0	0	0	0	0	1
0	0	0	85,7143	51,4286	0	1	0	2
0	0	0	11,4286	11,4286	0	0	0	1
0	0	0	34,2857	17,1429	0	0	0	3
0	0	0	17,1429	145,714	0	0	1	2
0	0	0	102,857	25,7143	0	1	0	1
0	0	0	4,28571	10	0	0	0	1
0	0	0	57,1429	114,286	85,7143	0	1	2
0	0	0	30	0	0	0	0	1

0	0	0	85,7143	100	8,57143	1	1	2
0	0	0	128,571	81,4286	42,8571	1	1	1
0	0	0	137,143	277,143	0	1	1	2
0	0	0	34,2857	17,1429	0	0	0	2
0	0	0	5,71429	0	0	0	0	2
0	0	0	0	94,2857	17,1429	0	1	2
0	0	0	8,57143	60	0	0	1	2
0	0	0	145,714	248,571	17,1429	1	1	1
0	0	0	65,7143	11,4286	34,2857	1	0	3
0	0	0	5	0	0	0	0	2
0	0	0	94,2857	0	6,42857	1	0	2
0	0	0	134,286	96,4286	25,7143	1	1	2
0	0	1	45	64,2857	25,7143	0	1	3
0	0	0	85,7143	94,2857	17,1429	1	1	2
0	0	0	0	0	12,8571	0	0	2
0	0	0	4,28571	128,571	0	0	1	2
0	0	0	24,2857	132,857	0	0	1	3
0	0	0	42,8571	51,4286	25,7143	0	1	2
0	0	1	141,429	10,7143	51,4286	1	1	2
0	0	0	17,1429	162,857	0	0	1	2
0	1	1	278,571	128,571	0	1	1	2
0	0	1	60	137,143	0	1	1	1
0	0	0	30	38,5714	0	0	0	2
0	0	0	8,57143	0	77,1429	0	1	1
0	0	0	51,4286	51,4286	4,28571	0	0	2
0	0	0	17,1429	85,7143	0	0	1	2
0	0	0	45,7143	4,28571	6,42857	0	0	2
0	0	0	0	0	0	0	0	2
0	0	0	5,71429	98,5714	0	0	1	1
0	1	1	98,5714	60	0	1	1	2
0	0	0	60	51,4286	0	1	0	2
0	0	0	102,857	111,429	0	1	1	2
0	0	0	40	42,8571	0	0	0	2
0	0	0	128,571	282,857	0	1	1	2
0	0	0	0	257,143	0	0	1	1
0	0	0	137,143	128,571	17,1429	1	1	2
0	0	0	77,1429	64,2857	0	1	1	2
0	0	0	8,57143	154,286	8,57143	0	1	2
0	0	0	169,286	145,714	25,7143	1	1	2
0	0	0	115,714	77,1429	25,7143	1	1	2
0	0	0	12,8571	0	0	0	0	2
0	0	0	21,4286	77,1429	0	0	1	2
0	0	0	0	0	0	0	0	1
1	0	1	10	34,2857	128,571	0	1	2
0	0	0	167,143	41,4286	0	1	0	2
0	0	0	25,7143	8,57143	0	0	0	1
0	0	0	0	0	3,57143	0	0	2
0	0	0	0	17,1429	0	0	0	2

0	1	1	199,286	51,4286	0	1	0	2
0	0	0	120,714	8,57143	0	1	0	2
0	1	1	60	0	60	1	1	2
0	0	0	111,429	291,429	4,28571	1	1	2
0	0	0	131,429	148,571	0	1	1	2
0	0	0	77,1429	17,1429	0	1	0	2
0	0	0	128,571	154,286	51,4286	1	1	2
0	0	0	45	14,2857	0	0	0	2
0	0	0	4,28571	0	0	0	0	2
0	0	0	5,71429	34,2857	0	0	0	2
0	0	0	8,57143	42,8571	54,2857	0	1	2
0	0	0	30	51,4286	0	0	0	2
0	0	0	122,857	68,5714	51,4286	1	1	2
0	0	0	300	17,1429	0	1	0	1
0	0	0	17,1429	0	0	0	0	2
0	0	0	0	17,1429	51,4286	0	1	2
0	0	0	51,4286	88,5714	0	0	1	2
0	1	1	68,5714	0	0	1	0	2
0	0	0	0	132,857	8,57143	0	1	2
0	0	0	17,1429	0	17,1429	0	0	1
0	0	0	15,7143	42,1429	0	0	0	1
0	0	0	10,7143	42,8571	0	0	0	2
0	0	0	240	94,2857	25,7143	1	1	2
0	0	0	98,5714	98,5714	0	1	1	2
0	0	0	0	0	0	0	0	1
0	0	0	17,1429	5,71429	0	0	0	1
0	0	0	200	20	25,7143	1	0	1
0	0	0	145,714	128,571	0	1	1	2
0	0	0	182,143	17,1429	0	1	0	1
0	0	1	54,2857	32,8571	0	0	0	2
0	0	0	42,8571	17,1429	0	0	0	1
0	0	0	0	8,57143	0	0	0	2
0	0	0	0	0	0	0	0	1
0	0	0	164,286	128,571	154,286	1	1	2
1	0	1	17,1429	120	0	0	1	1
0	0	1	51,4286	132,857	0	0	1	1
0	1	1	60	231,429	180	1	1	2
0	0	0	210	0	17,1429	1	0	1
0	0	0	47,1429	21,4286	0	0	0	2
0	0	0	112,857	0	0	1	0	2
0	0	0	4,28571	2,85714	128,571	0	1	1
0	0	0	17,1429	11,4286	25,7143	0	0	1
0	0	0	30	98,5714	0	0	1	2
0	0	0	34,2857	0	0	0	0	2
0	0	0	0	25,7143	0	0	0	2
0	0	0	154,286	17,1429	0	1	0	1
0	0	0	77,1429	0	0	1	0	2
0	0	0	0	60	0	0	1	1

0	1	1	120	171,429	51,4286	1	1	2
0	1	1	102,857	300	2,85714	1	1	1
0	0	0	28,5714	8,57143	0	0	0	2
0	0	0	101,429	8,57143	34,2857	1	0	2
0	0	0	60	70	34,2857	1	1	1
0	0	0	128,571	437,143	0	1	1	2
0	0	0	25,7143	154,286	0	0	1	1
0	0	0	40	0	0	0	0	1
0	0	1	8,57143	60	0	0	1	2
1	0	1	0	17,1429	180	0	1	2
0	0	0	0	162,857	17,1429	0	1	2
0	0	1	111,429	128,571	51,4286	1	1	2
0	0	0	37,1429	60	8,57143	0	1	2
0	0	0	0	25,7143	197,143	0	1	2
0	1	1	220,714	154,286	0	1	1	1
0	0	0	18,5714	51,4286	60	0	1	2
0	0	0	42,8571	385,714	0	0	1	2
0	0	0	128,571	137,143	0	1	1	2
0	0	0	0	154,286	0	0	1	2
0	0	0	132,857	68,5714	154,286	1	1	1
0	0	0	94,2857	130,714	0	1	1	2
0	0	0	14,2857	68,5714	0	0	1	2
0	0	0	188,571	145,714	51,4286	1	1	2
0	0	0	188,571	162,857	180	1	1	1
0	0	0	24,2857	5,71429	51,4286	0	0	2
0	0	0	214,286	42,8571	51,4286	1	1	2
0	0	0	35,7143	0	77,1429	0	1	2
0	0	0	0	15	51,4286	0	1	1
0	0	0	51,4286	21,4286	0	0	0	1
0	0	0	145,714	35,7143	0	1	0	1
0	1	1	60	137,143	0	1	1	2
0	0	0	0	14,2857	0	0	0	2
0	0	0	0	171,429	0	0	1	2
0	0	0	60	14,2857	0	1	0	1
0	0	0	128,571	85,7143	0	1	1	2
0	0	1	0	25,7143	51,4286	0	1	2
0	0	0	20	94,2857	85,7143	0	1	2
0	0	0	137,143	21,4286	8,57143	1	0	2
0	0	0	0	158,571	102,857	0	1	2
0	0	0	128,571	188,571	25,7143	1	1	2
0	0	0	42,8571	154,286	180	0	1	2
1	0	1	12,8571	47,1429	105,714	0	1	2
0	0	0	0	17,1429	0	0	0	2
0	0	0	6,42857	60	0	0	1	1
0	0	0	33,5714	0	0	0	0	1
0	0	0	0	25,7143	0	0	0	1
0	0	0	128,571	128,571	0	1	1	2
0	0	0	2,14286	51,4286	0	0	0	2

0	0	0	8,57143	64,2857	0	0	1	2
0	1	1	60	17,1429	0	1	0	2
0	0	0	60	0	0	1	0	1
0	0	0	154,286	30	0	1	0	2
0	0	0	102,857	214,286	0	1	1	2
0	0	0	2,14286	10,7143	8,57143	0	0	2
0	0	0	25,7143	0	0	0	0	1
0	0	0	154,286	77,1429	0	1	1	2
0	0	0	17,1429	42,8571	0	0	0	2
0	0	0	5,71429	60	34,2857	0	1	2
0	0	0	137,143	154,286	102,857	1	1	2
0	0	0	62,8571	8,57143	14,2857	1	0	1
0	1	1	201,429	34,2857	0	1	0	2
0	0	0	12,8571	42,8571	0	0	0	2
0	0	0	68,5714	21,4286	0	1	0	2
0	0	0	139,286	214,286	0	1	1	1
0	0	0	12,8571	0	0	0	0	2
0	0	0	25,7143	102,857	6,42857	0	1	1
0	0	0	30	10	0	0	0	2
0	0	0	57,1429	25,7143	0	0	0	2
0	0	0	8,57143	102,857	60	0	1	1
0	1	1	248,571	145,714	0	1	1	2
0	0	0	128,571	197,143	51,4286	1	1	2
0	0	0	17,1429	0	0	0	0	2
0	0	0	128,571	7,14286	0	1	0	2
0	0	0	12,8571	17,1429	8,57143	0	0	2
0	0	0	0	17,1429	0	0	0	2
0	1	1	150	25,7143	0	1	0	2
0	0	0	2,85714	0	0	0	0	2
0	0	0	47,1429	68,5714	8,57143	0	1	2
0	0	0	167,143	60	8,57143	1	1	1
0	0	0	30	17,1429	0	0	0	1
0	0	0	0	25,7143	0	0	0	2
0	0	0	214,286	182,857	0	1	1	1
0	1	1	102,857	78,5714	17,1429	1	1	2
0	0	0	158,571	180	0	1	1	2
0	0	0	154,286	51,4286	77,1429	1	1	3
0	0	0	8,57143	162,857	0	0	1	2
0	0	0	0	30	0	0	0	1
0	0	0	0	42,8571	25,7143	0	1	2
1	0	1	60,7143	117,143	0	1	1	1
0	0	0	14,2857	10,7143	0	0	0	2
0	0	0	85,7143	21,4286	0	1	0	2
0	0	0	17,1429	0	0	0	0	2
0	0	0	21,4286	102,857	0	0	1	2
0	0	0	115,714	17,1429	51,4286	1	1	2
0	0	0	31,4286	120	0	0	1	2
0	0	0	30	32,1429	0	0	0	2

0	0	0	240	257,143	25,7143	1	1	1
0	0	1	77,1429	72,8571	0	1	1	1
0	0	0	128,571	17,1429	60	1	1	1
0	1	1	120	191,429	0	1	1	1
0	0	0	42,8571	51,4286	0	0	0	2
0	0	0	85,7143	0	51,4286	1	0	1
0	0	0	90	23,5714	0	1	0	2
1	0	1	77,1429	94,2857	0	1	1	2
0	0	0	14,2857	158,571	0	0	1	2
0	0	0	60	4,28571	12,8571	1	0	2
0	0	0	14,2857	68,5714	0	0	1	2
0	0	0	168,571	8,57143	0	1	0	1
0	0	1	42,8571	107,143	0	0	1	2
0	0	0	257,143	131,429	0	1	1	2
0	1	1	150	60	34,2857	1	1	1
0	0	0	20	162,857	154,286	0	1	2
0	0	0	42,8571	171,429	0	0	1	1
0	0	0	42,8571	42,8571	17,1429	0	1	1
0	0	0	0	177,143	128,571	0	1	1
0	0	0	68,5714	17,1429	0	1	0	3
0	0	0	57,1429	95,7143	8,57143	0	1	2
0	0	0	0	0	205,714	0	1	2
1	0	1	0	222,857	0	0	1	1
0	0	0	138,571	25,7143	51,4286	1	1	1
0	0	0	47,1429	0	0	0	0	1
0	0	0	137,143	94,2857	0	1	1	2
0	0	1	54,2857	51,4286	34,2857	0	1	2
0	0	0	90	34,2857	0	1	0	2
0	0	0	71,4286	128,571	0	1	1	2
0	0	0	0	128,571	30	0	1	2
0	0	0	51,4286	308,571	57,1429	0	1	2
0	0	0	257,143	74,2857	0	1	1	1
0	0	0	25,7143	10	0	0	0	1
0	1	1	134,286	0	34,2857	1	0	2
0	0	0	175,714	411,429	0	1	1	2
0	0	0	171,429	154,286	51,4286	1	1	2
0	0	0	8,57143	0	0	0	0	2
0	0	0	0	8,57143	0	0	0	2
0	0	0	115,714	51,4286	0	1	0	2
0	0	0	37,1429	77,1429	8,57143	0	1	2
0	0	0	14,2857	51,4286	0	0	0	1
0	0	0	47,1429	51,4286	154,286	0	1	2
0	0	0	0	128,571	25,7143	0	1	1
0	0	0	51,4286	0	0	0	0	2
0	0	0	111,429	42,8571	25,7143	1	1	1
0	0	0	47,1429	66,4286	0	0	1	2
0	0	0	15	51,4286	0	0	0	2
0	0	0	0	102,857	0	0	1	2

0	0	0	0	102,857	0	0	1	2
0	0	0	115,714	137,143	0	1	1	2
0	0	0	47,1429	75	0	0	1	2
0	0	0	28,5714	191,429	94,2857	0	1	2
0	0	0	82,8571	5,71429	8,57143	1	0	1
0	0	0	60	51,4286	0	1	0	2
0	0	0	27,8571	94,2857	34,2857	0	1	1
0	0	0	7,14286	171,429	0	0	1	2
0	0	0	132,857	80	17,1429	1	1	2
0	0	0	282,857	231,429	0	1	1	1
0	0	0	128,571	128,571	0	1	1	1
0	0	0	25,7143	68,5714	0	0	1	2
0	0	0	14,2857	17,1429	77,1429	0	1	2
0	0	0	0	0	0	0	0	1
0	0	0	8,57143	167,143	0	0	1	2
0	0	0	139,286	98,5714	0	1	1	2
0	0	0	25,7143	17,1429	0	0	0	1
0	0	0	178,571	0	0	1	0	2
0	0	0	0	0	0	0	0	2
0	0	0	17,1429	25,7143	0	0	0	2
0	0	0	158,571	351,429	180	1	1	2
0	0	0	60	34,2857	0	1	0	2
0	0	0	5,71429	51,4286	51,4286	0	1	2
1	0	1	94,2857	94,2857	0	1	1	2
0	0	0	0	51,4286	0	0	0	3
0	0	0	0	91,4286	0	0	1	2
0	0	0	17,1429	107,143	0	0	1	2
0	0	1	53,5714	263,571	77,1429	0	1	2
0	0	0	30	8,57143	0	0	0	1
0	0	1	17,1429	15	38,5714	0	0	2
0	0	0	25,7143	52,8571	60	0	1	2
0	0	0	24,2857	0	0	0	0	2
0	0	0	180	42,8571	0	1	0	2
0	0	0	8,57143	30	0	0	0	2
0	0	0	68,5714	25,7143	51,4286	1	1	2
0	0	0	14,2857	25,7143	65,7143	0	1	2
0	0	0	17,1429	17,1429	0	0	0	2
0	0	0	40,7143	34,2857	0	0	0	2
0	0	0	141,429	12,8571	3,14286	1	0	2
0	0	0	175,714	257,143	128,571	1	1	1
0	0	1	145,714	85,7143	128,571	1	1	2
0	0	0	51,4286	188,571	0	0	1	2
0	0	0	17,1429	85,7143	0	0	1	1
0	1	1	180	0	0	1	0	2
0	0	0	38,5714	21,4286	0	0	0	2
0	1	1	77,1429	94,2857	12,8571	1	1	2
0	1	1	70	60	8,57143	1	1	2
0	0	0	0	150	0	0	1	2

0	0	0	68,5714	0	17,1429	1	0	2
0	0	0	0	0	8,57143	0	0	1
0	0	0	40,7143	20	34,2857	0	0	2
0	0	0	150	88,5714	77,1429	1	1	2
0	0	0	32,1429	17,1429	0	0	0	1
0	0	0	19,2857	0	8,57143	0	0	2
0	0	0	34,2857	246,429	11,4286	0	1	2
0	0	0	17,1429	34,2857	25,7143	0	1	2
0	0	0	74,2857	102,857	0	1	1	1
1	0	1	94,2857	34,2857	51,4286	1	1	2
0	0	0	134,286	111,429	42,8571	1	1	2
0	0	0	30	8,57143	38,5714	0	0	2
0	0	0	67,1429	55,7143	12,8571	1	1	1
0	0	0	78,5714	34,2857	0	1	0	2
0	0	0	202,857	4,28571	12,8571	1	0	2
0	0	0	137,143	30	2,85714	1	0	2
0	0	0	50	0	0	0	0	2
0	0	0	57,1429	42,8571	17,1429	0	1	2
0	0	0	114,286	33,5714	0	1	0	1
0	0	0	25,7143	51,4286	0	0	0	1
0	0	0	34,2857	17,1429	21,4286	0	0	1
0	0	0	17,1429	30	0	0	0	2
0	0	0	2,85714	0	8,57143	0	0	2
0	0	0	128,571	197,143	205,714	1	1	2
0	0	0	58,5714	34,2857	0	0	0	2
0	0	1	67,1429	25,7143	17,1429	1	0	2
0	0	0	15	210	137,143	0	1	2
0	0	0	68,5714	0	8,57143	1	0	2
0	0	0	0	0	0	0	0	1
0	0	0	154,286	231,429	0	1	1	2
0	0	0	8,57143	75	0	0	1	2
0	0	0	6,42857	25,7143	17,1429	0	0	2
0	0	0	142,857	82,8571	0	1	1	2
0	0	0	140	85,7143	0	1	1	2
0	0	0	265,714	258,571	137,143	1	1	1
0	0	0	32,8571	19,2857	0	0	0	2
0	0	0	12,8571	0	0	0	0	1
0	0	0	51,4286	265,714	25,7143	0	1	2
0	0	0	17,1429	171,429	25,7143	0	1	2
0	0	0	21,4286	17,1429	0	0	0	1
0	0	0	51,4286	77,1429	0	0	1	2
0	0	0	102,857	42,8571	0	1	0	2
0	0	0	10	85,7143	51,4286	0	1	2
0	0	0	18,5714	180	17,1429	0	1	2
0	0	0	0	34,2857	17,1429	0	0	1
1	0	1	60	122,857	0	1	1	2
0	0	0	20	128,571	25,7143	0	1	1
0	0	0	77,1429	21,4286	0	1	0	2

0	0	0	17,1429	34,2857	17,1429	0	0	3
0	0	0	0	12,1429	8,57143	0	0	2
0	0	0	128,571	180	0	1	1	2
0	0	0	154,286	154,286	0	1	1	2
0	0	0	145,714	74,2857	0	1	1	1
0	0	0	137,143	113,571	0	1	1	1
0	0	0	17,1429	85,7143	51,4286	0	1	2
0	0	0	104,286	27,8571	0	1	0	1
0	0	0	25,7143	17,1429	4,28571	0	0	1
0	0	0	11,4286	34,2857	128,571	0	1	2
0	0	0	154,286	411,429	180	1	1	2
0	0	0	24,2857	60	94,2857	0	1	2
0	0	0	10,7143	0	0	0	0	2
0	0	0	30	180	180	0	1	2
0	0	0	14,2857	0	0	0	0	1
0	1	1	240	132,857	38,5714	1	1	2
0	0	0	53,5714	50	0	0	0	2
0	0	0	0	154,286	68,5714	0	1	1
0	0	0	43,5714	0	0	0	0	1
0	0	0	0	128,571	0	0	1	1
0	0	0	0	111,429	0	0	1	2
0	0	0	6,42857	42,8571	34,2857	0	1	2
0	0	0	0	7,14286	94,2857	0	1	2
0	0	0	192,857	34,2857	25,7143	1	1	2
0	0	0	8,57143	0	0	0	0	2
0	0	0	8,57143	17,1429	0	0	0	1
0	0	0	12,8571	110	0	0	1	2
0	0	0	51,4286	197,143	17,1429	0	1	2
1	0	1	21,4286	25,7143	171,429	0	1	2
0	0	0	0	0	4,28571	0	0	1
0	0	0	42,8571	75,7143	0	0	1	1
0	0	0	2,85714	0	0	0	0	2
0	0	0	50	0	0	0	0	2
0	0	0	42,8571	79,2857	6,42857	0	1	2
0	0	0	8,57143	68,5714	25,7143	0	1	2
0	0	0	25,7143	0	8,57143	0	0	2
0	0	0	141,429	49,2857	0	1	0	1
0	0	0	51,4286	51,4286	17,1429	0	1	2
0	0	0	0	154,286	0	0	1	2
0	0	0	137,143	154,286	0	1	1	2
0	0	0	111,429	35,7143	51,4286	1	1	1
0	1	1	218,571	77,1429	25,7143	1	1	2
0	0	0	128,571	194,286	154,286	1	1	2
0	0	0	8,57143	34,2857	17,1429	0	0	1
0	0	0	180	0	2,85714	1	0	2
0	0	0	25,7143	184,286	15	0	1	2
0	0	0	17,1429	180	0	0	1	2
1	0	1	205,714	25,7143	171,429	1	1	1

0	0	0	162,857	34,2857	17,1429	1	0	2
0	0	0	85,7143	51,4286	68,5714	1	1	2
0	0	0	210	128,571	0	1	1	2
0	0	0	8,57143	0	0	0	0	1
0	0	1	171,429	98,5714	25,7143	1	1	1
0	0	0	77,1429	17,1429	0	1	0	1
0	0	0	120,714	4,28571	0	1	0	2
0	0	0	0	0	12,8571	0	0	2
0	0	0	0	128,571	0	0	1	2
0	0	0	0	5,71429	154,286	0	1	2
0	0	0	35,7143	137,143	17,1429	0	1	2
0	1	1	180	51,4286	60	1	1	1
0	0	0	42,8571	102,857	0	0	1	1
0	0	0	128,571	162,857	120	1	1	2
0	0	0	34,2857	128,571	0	0	1	3
0	1	1	218,571	60	0	1	1	1
0	0	0	180	128,571	25,7143	1	1	1
0	0	0	2,85714	0	25,7143	0	0	2
0	0	0	48,5714	51,4286	17,1429	0	1	2
0	0	0	8,57143	51,4286	0	0	0	1
0	0	0	20	0	0	0	0	3
0	0	0	0	128,571	5,71429	0	1	2
0	0	0	158,571	180	0	1	1	2
0	0	0	197,143	60	0	1	1	2
0	0	0	0	0	0	0	0	1
0	0	0	7,14286	8,57143	0	0	0	2
0	0	0	8,57143	0	154,286	0	1	2
0	0	0	68,5714	257,143	0	1	1	1
0	0	0	24,2857	6,42857	10,7143	0	0	3
0	0	0	0	102,857	25,7143	0	1	2
0	0	0	8,57143	21,4286	0	0	0	2
0	0	0	0	17,1429	0	0	0	2
0	0	0	0	154,286	0	0	1	2
0	0	0	102,857	128,571	17,1429	1	1	2
0	0	0	36,4286	8,57143	25,7143	0	0	2
0	1	1	180	51,4286	0	1	0	2
0	0	0	25,7143	34,2857	0	0	0	2
0	0	0	17,1429	14,2857	37,1429	0	0	2
0	0	0	102,857	128,571	0	1	1	2
0	0	0	0	162,857	0	0	1	2
0	0	0	14,2857	0	5,71429	0	0	2
0	0	0	80	17,1429	20	1	0	2
0	0	0	55,7143	11,4286	0	0	0	3
0	0	0	145,714	0	0	1	0	1
0	0	0	150	8,57143	45,7143	1	0	2
0	0	0	158,571	8,57143	25,7143	1	0	2
0	0	0	98,5714	67,1429	0	1	1	1
0	0	0	188,571	25,7143	0	1	0	2

0	0	0	150	257,143	0	1	1	2
0	0	0	40	0	0	0	0	2
0	0	0	51,4286	0	0	0	0	1
0	0	0	214,286	402,857	68,5714	1	1	2
0	0	0	162,857	205,714	0	1	1	2
0	0	0	5,71429	25,7143	0	0	0	2
0	0	0	88,5714	40	0	1	0	2
0	0	0	42,8571	154,286	5,71429	0	1	1
0	0	0	0	42,8571	0	0	0	2
0	0	0	25,7143	34,2857	0	0	0	2
0	0	0	21,4286	20	0	0	0	1
0	0	0	192,857	68,5714	12,8571	1	1	2
0	0	0	100	30	5,71429	1	0	1
0	0	0	17,1429	102,857	154,286	0	1	2
0	0	0	17,1429	77,1429	0	0	1	2
0	0	0	34,2857	85,7143	0	0	1	2
0	0	0	265,714	132,857	0	1	1	1
0	0	0	157,143	154,286	0	1	1	1
0	0	0	21,4286	12,8571	0	0	0	2
0	0	0	2,85714	42,8571	0	0	0	2
0	0	0	42,8571	72,8571	0	0	1	1
0	0	0	8,57143	0	0	0	0	1
0	0	0	60	23,5714	0	1	0	1
0	0	0	50	8,57143	8,57143	0	0	2
0	0	0	175,714	210	25,7143	1	1	1
0	0	0	4,28571	17,1429	0	0	0	2
0	0	0	0	34,2857	25,7143	0	1	2
0	0	0	0	0	0	0	0	2
0	0	0	105	77,1429	0	1	1	1
0	0	0	128,571	197,143	154,286	1	1	1
0	0	0	57,1429	25,7143	0	0	0	2
0	0	0	17,1429	0	0	0	0	2
0	0	0	51,4286	17,1429	0	0	0	2
0	0	0	8,57143	50	0	0	0	2
0	0	0	4,28571	132,857	17,1429	0	1	2
0	0	0	11,4286	131,429	25,7143	0	1	2
0	1	1	90	34,2857	0	1	0	2
0	0	0	22,8571	113,571	25,7143	0	1	2
0	0	0	5,71429	171,429	0	0	1	2
0	0	1	34,2857	85,7143	0	0	1	2
0	0	0	1,42857	23,5714	0	0	0	2
0	0	0	154,286	162,857	42,8571	1	1	1
0	0	0	0	102,857	25,7143	0	1	1
0	0	0	10,7143	11,4286	0	0	0	2
0	0	0	0	51,4286	0	0	0	2
0	0	0	197,143	60	8,57143	1	1	2
0	0	0	17,1429	51,4286	6,42857	0	0	2
0	0	1	94,2857	37,1429	34,2857	1	1	2

0	0	0	60	102,857	0	1	1	1
0	0	0	28,5714	274,286	0	0	1	2
0	0	0	84,2857	145,714	0	1	1	2
0	0	0	128,571	120	52,8571	1	1	2
0	0	0	34,2857	197,143	0	0	1	2
0	0	0	25,7143	111,429	0	0	1	2
0	0	0	4,28571	30	34,2857	0	1	2
0	0	0	17,1429	167,143	0	0	1	1
0	0	0	17,1429	51,4286	0	0	0	2
0	0	0	128,571	231,429	51,4286	1	1	2
0	0	0	11,4286	77,1429	0	0	1	2
0	0	0	55,7143	137,143	77,1429	0	1	2
0	0	0	10	0	0	0	0	2
0	1	1	140	85,7143	0	1	1	2
0	0	0	180	15	14,2857	1	0	2
0	0	0	17,1429	51,4286	0	0	0	2
0	0	0	25,7143	34,2857	0	0	0	2
0	0	0	47,1429	0	0	0	0	1
0	0	0	5,71429	17,1429	34,2857	0	0	1
0	0	0	25,7143	8,57143	0	0	0	2
0	0	0	127,143	154,286	60	1	1	2
0	0	0	222,857	68,5714	25,7143	1	1	1
0	0	0	0	128,571	0	0	1	2
0	0	0	0	8,57143	25,7143	0	0	2
0	0	0	34,2857	22,8571	0	0	0	1
0	0	0	49,2857	25,7143	0	0	0	2
0	0	0	17,1429	0	0	0	0	2
0	0	0	27,8571	21,4286	0	0	0	1
0	0	0	85,7143	42,8571	0	1	0	2
0	0	0	42,8571	0	25,7143	0	0	2
0	0	0	94,2857	12,8571	30	1	0	1
0	0	0	50	54,2857	28,5714	0	1	2
0	0	0	105,714	1,42857	0	1	0	1
0	0	0	167,143	77,1429	30	1	1	1
0	0	0	10	128,571	25,7143	0	1	2
0	0	0	12,8571	102,857	0	0	1	2
0	0	0	28,5714	231,429	8,57143	0	1	1
0	0	0	85,7143	377,143	102,857	1	1	2
0	0	0	158,571	111,429	77,1429	1	1	3
0	0	0	120	47,1429	0	1	0	2
0	0	0	28,5714	75	32,8571	0	1	2
0	0	0	5,71429	12,8571	18,5714	0	0	2
0	0	0	0	17,1429	0	0	0	2
0	0	0	6,42857	8,57143	0	0	0	2
0	0	0	68,5714	0	51,4286	1	0	2
0	0	0	17,1429	175,714	25,7143	0	1	1
0	0	0	128,571	197,143	0	1	1	2
0	0	0	192,857	51,4286	0	1	0	2

0	0	0	141,429	154,286	17,1429	1	1	2
0	1	1	88,5714	0	0	1	0	1
0	0	0	60	68,5714	0	1	1	1
0	0	0	19,2857	0	0	0	0	2
0	0	0	42,8571	25,7143	25,7143	0	0	2
0	0	0	7,14286	27,8571	0	0	0	3
0	0	0	20	25,7143	0	0	0	1
0	0	0	12,8571	28,5714	0	0	0	2
0	0	0	4,28571	0	0	0	0	2
0	0	0	1,42857	0	0	0	0	1
0	0	0	77,1429	8,57143	17,1429	1	0	2
0	0	0	0	31,4286	4,28571	0	0	2
0	0	0	60	60	60	1	1	2
0	0	0	184,286	172,857	0	1	1	1
0	0	0	44,2857	25,7143	0	0	0	2
0	0	0	45	30	0	0	0	2
0	1	1	102,857	477,143	0	1	1	2
1	0	1	107,143	214,286	60	1	1	1
0	0	0	145,714	162,857	25,7143	1	1	2
0	0	0	12,8571	0	34,2857	0	0	2
0	1	1	411,429	231,429	0	1	1	2
0	0	0	7,14286	128,571	25,7143	0	1	2
0	0	0	128,571	17,1429	0	1	0	2
0	0	0	192,857	17,1429	0	1	0	2
0	0	0	120	58,5714	77,1429	1	1	2
0	0	0	128,571	31,4286	0	1	0	2
1	0	1	102,857	154,286	68,5714	1	1	2
0	0	0	34,2857	42,8571	0	0	0	2
0	0	0	190	0	25,7143	1	0	1
0	0	0	8,57143	28,5714	0	0	0	3
0	0	0	51,4286	85,7143	128,571	0	1	3
0	0	0	128,571	128,571	0	1	1	2
0	0	0	137,143	42,8571	0	1	0	1
0	0	0	0	0	0	0	0	2
0	0	0	85,7143	2,14286	0	1	0	2
0	0	0	42,8571	8,57143	0	0	0	2
0	0	0	0	0	17,1429	0	0	2
0	0	0	171,429	10	25,7143	1	0	2
0	0	0	205,714	154,286	25,7143	1	1	2
0	0	0	0	162,857	0	0	1	1
0	0	0	85,7143	34,2857	0	1	0	2
0	0	0	120	85,7143	0	1	1	2
0	1	1	60	0	0	1	0	2
0	0	1	45	54,2857	28,5714	0	1	1
0	0	0	0	17,1429	0	0	0	1
1	0	1	10,7143	45	17,1429	0	1	2
1	0	1	135	145,714	120	1	1	1
0	0	0	105	85,7143	0	1	1	2

0	0	0	8,57143	162,857	0	0	1	3
0	0	0	85,7143	128,571	0	1	1	2
0	0	0	102,857	60	0	1	1	2
0	1	1	195,714	34,2857	0	1	0	2
0	0	0	42,8571	128,571	17,1429	0	1	2
0	0	0	10	55,7143	0	0	0	2
0	0	0	154,286	145,714	25,7143	1	1	1
0	0	0	201,429	145,714	0	1	1	1
0	0	0	128,571	85,7143	0	1	1	2
0	0	0	51,4286	98,5714	0	0	1	2
1	0	1	64,2857	162,857	102,857	1	1	2
0	0	0	60	74,2857	0	1	1	3
0	0	0	8,57143	38,5714	36,4286	0	1	2
0	0	0	25,7143	180	17,1429	0	1	2
0	0	0	8,57143	102,857	25,7143	0	1	2
0	0	0	0	85,7143	25,7143	0	1	2
0	0	0	68,5714	24,2857	40	1	1	2
0	0	0	6,42857	42,8571	17,1429	0	1	2
0	0	0	5,71429	94,2857	25,7143	0	1	2
0	1	1	217,143	128,571	0	1	1	1
0	0	0	141,429	180	51,4286	1	1	2
0	0	0	142,857	32,1429	25,7143	1	0	2
0	0	0	60	62,1429	0	1	1	1
0	0	0	24,2857	51,4286	0	0	0	2
0	0	0	35,7143	34,2857	0	0	0	1
0	0	0	4,28571	8,57143	25,7143	0	0	2
0	0	0	42,8571	162,857	0	0	1	2
0	0	0	19,2857	0	0	0	0	2
0	0	0	0	0	0	0	0	2
0	0	0	131,429	160	17,1429	1	1	3
0	0	0	8,57143	25,7143	0	0	0	2
0	0	0	57,8571	57,8571	0	0	0	2
0	0	0	80	197,143	8,57143	1	1	1
0	0	1	38,5714	8,57143	77,1429	0	1	1
0	0	0	0	34,2857	17,1429	0	0	2
0	0	0	136,429	197,143	77,1429	1	1	1
0	0	0	8,57143	52,8571	0	0	0	1
1	0	1	0	0	77,1429	0	1	3
0	0	0	30	27,1429	25,7143	0	0	2
0	0	0	40,7143	17,1429	7,14286	0	0	2
0	0	0	25,7143	51,4286	0	0	0	2
0	0	0	42,8571	137,143	60	0	1	2
0	0	0	85,7143	272,857	154,286	1	1	2
0	0	0	60	42,8571	0	1	0	2
0	0	0	81,4286	47,1429	0	1	0	2
0	0	0	85,7143	257,143	25,7143	1	1	2
0	0	0	12,8571	137,143	128,571	0	1	2
1	1	1	308,571	600	120	1	1	2

0	0	0	2,14286	8,57143	25,7143	0	0	2
0	0	0	128,571	8,57143	5,71429	1	0	2
0	0	0	113,571	4,28571	157,143	1	1	1
0	0	0	139,286	0	0	1	0	1
0	0	1	40	8,57143	20	0	0	2
0	1	1	111,429	141,429	25,7143	1	1	2
0	0	0	2,14286	0	0	0	0	2
0	0	0	5,71429	2,14286	0	0	0	2
0	0	0	22,8571	0	0	0	0	1
0	0	0	21,4286	34,2857	0	0	0	2
0	0	0	150	180	0	1	1	1
0	0	0	222,857	111,429	25,7143	1	1	2
0	0	0	4,28571	94,2857	0	0	1	1
0	0	0	154,286	154,286	25,7143	1	1	3
0	0	0	57,8571	77,1429	0	0	1	2
0	0	0	336,429	51,4286	68,5714	1	1	2
0	0	0	72,8571	42,8571	0	1	0	2
0	0	0	11,4286	0	0	0	0	2
1	0	1	154,286	142,143	25,7143	1	1	2
0	0	0	182,857	111,429	0	1	1	2
0	0	0	30	34,2857	25,7143	0	1	2
0	0	0	145,714	17,1429	17,1429	1	0	2
0	0	0	0	42,8571	128,571	0	1	2
0	0	0	134,286	128,571	25,7143	1	1	1
0	0	0	60	98,5714	85,7143	1	1	2
0	0	0	137,143	85,7143	0	1	1	2
0	0	0	102,857	68,5714	0	1	1	2
0	0	0	47,1429	0	0	0	0	2
0	0	0	60	0	8,57143	1	0	2
0	0	0	271,429	126,429	0	1	1	1
0	0	0	0	28,5714	25,7143	0	0	2
0	0	0	4,28571	25,7143	0	0	0	1
0	0	0	12,8571	128,571	77,1429	0	1	1
0	0	0	21,4286	85,7143	0	0	1	2
0	0	0	137,143	128,571	94,2857	1	1	2
0	0	0	64,2857	51,4286	0	1	0	2
0	0	0	8,57143	8,57143	0	0	0	2
0	0	0	60	222,857	51,4286	1	1	2
0	1	1	90	0	0	1	0	2
0	0	0	0	0	17,1429	0	0	2
0	0	0	77,1429	42,8571	0	1	0	2
0	0	0	51,4286	0	0	0	0	2
0	0	0	0	51,4286	0	0	0	2
0	0	0	205,714	17,1429	0	1	0	2
0	0	0	12,1429	17,1429	0	0	0	2
0	0	0	17,1429	38,5714	0	0	0	1
0	0	0	65,7143	8,57143	0	1	0	2
0	0	0	85,7143	102,857	0	1	1	2

0	0	0	0	171,429	0	0	1	1
0	0	0	8,57143	0	0	0	0	2
0	0	0	37,1429	60	0	0	1	2
1	0	1	128,571	111,429	85,7143	1	1	2
0	0	0	7,14286	0	0	0	0	2
0	1	1	128,571	34,2857	0	1	0	2
0	0	1	85,7143	137,143	0	1	1	2
0	0	0	21,4286	4,28571	0	0	0	2
0	0	0	282,857	282,857	85,7143	1	1	2
0	1	1	360	128,571	25,7143	1	1	2
0	0	0	154,286	257,143	0	1	1	2
0	0	0	7,14286	0	0	0	0	2
0	0	0	64,2857	85,7143	8,57143	1	1	2
0	0	0	45	31,4286	0	0	0	2
0	0	0	205,714	197,143	0	1	1	2
0	0	0	25,7143	4,28571	51,4286	0	0	2
0	0	1	61,4286	51,4286	0	1	0	3
0	0	0	15	8,57143	0	0	0	2
0	0	0	85,7143	128,571	0	1	1	2
0	0	0	0	0	0	0	0	3
0	1	1	165	25,7143	0	1	0	2
0	0	0	210	171,429	12,8571	1	1	2
0	0	0	92,1429	0	0	1	0	1
0	0	1	162,857	105	0	1	1	1
0	1	1	125,714	57,1429	0	1	0	1
0	0	0	180	145,714	25,7143	1	1	2
0	0	0	222,857	8,57143	0	1	0	2
0	0	0	60	0	77,1429	1	1	2
0	0	0	137,143	21,4286	0	1	0	2
0	0	0	17,1429	27,8571	0	0	0	2
0	0	0	35,7143	0	0	0	0	2
0	0	0	128,571	257,143	0	1	1	2
0	0	0	4,28571	0	0	0	0	2
0	0	0	0	0	180	0	1	2
0	0	0	50	8,57143	4,28571	0	0	2
0	0	0	17,8571	34,2857	0	0	0	2
0	0	0	4,28571	5,71429	0	0	0	2
0	0	0	21,4286	82,8571	42,8571	0	1	1
0	0	0	17,1429	17,1429	34,2857	0	0	2
0	0	0	128,571	257,143	0	1	1	2
0	0	0	25,7143	115,714	0	0	1	2
0	0	0	17,1429	68,5714	0	0	1	2
0	0	0	8,57143	17,1429	47,1429	0	1	2
0	0	0	0	8,57143	0	0	0	1
0	0	0	85,7143	137,143	0	1	1	1
0	0	0	68,5714	17,1429	0	1	0	2
0	0	0	31,4286	45	0	0	0	2
0	1	1	480	205,714	120	1	1	2

0	*0*	*0*	*17,1429*	*17,1429*	*12,8571*	*0*	*0*	*2*
0	*0*	*0*	*2,85714*	*168,571*	*0*	*0*	*1*	*2*
0	*0*	*0*	*79,2857*	*143,571*	*0*	*1*	*1*	*2*
0	*0*	*0*	*120*	*25,7143*	*60*	*1*	*1*	*2*
0	*0*	*0*	*128,571*	*0*	*0*	*1*	*0*	*2*
0	*0*	*0*	*22,8571*	*60*	*0*	*0*	*1*	*2*
0	*0*	*0*	*0*	*34,2857*	*0*	*0*	*0*	*2*
1	*1*	*1*	*145,714*	*68,5714*	*51,4286*	*1*	*1*	*1*
0	*0*	*0*	*68,5714*	*128,571*	*0*	*1*	*1*	*2*
1	*0*	*1*	*60*	*120*	*137,143*	*1*	*1*	*2*
0	*0*	*0*	*188,571*	*25,7143*	*0*	*1*	*0*	*1*
0	*0*	*0*	*11,4286*	*27,1429*	*0*	*0*	*0*	*1*
0	*0*	*0*	*1,42857*	*8,57143*	*0*	*0*	*0*	*1*
0	*0*	*0*	*60*	*25,7143*	*0*	*1*	*0*	*1*
0	*0*	*0*	*135*	*85,7143*	*8,57143*	*1*	*1*	*2*
0	*0*	*0*	*0*	*17,1429*	*0*	*0*	*0*	*2*
0	*0*	*0*	*29,2857*	*11,4286*	*0*	*0*	*0*	*1*
0	*0*	*0*	*214,286*	*0*	*0*	*1*	*0*	*2*
0	*1*	*1*	*68,5714*	*85,7143*	*17,1429*	*1*	*1*	*2*
0	*0*	*0*	*0*	*111,429*	*25,7143*	*0*	*1*	*1*
0	*0*	*0*	*40*	*8,57143*	*0*	*0*	*0*	*2*
0	*0*	*0*	*50,7143*	*214,286*	*0*	*0*	*1*	*1*
0	*0*	*0*	*51,4286*	*0*	*0*	*0*	*0*	*2*
1	*0*	*1*	*50*	*368,571*	*205,714*	*0*	*1*	*1*
0	*0*	*0*	*17,1429*	*94,2857*	*0*	*0*	*1*	*2*
0	*0*	*0*	*131,429*	*17,1429*	*0*	*1*	*0*	*2*
0	*0*	*0*	*4,28571*	*0*	*0*	*0*	*0*	*3*
0	*1*	*1*	*190*	*30*	*25,7143*	*1*	*0*	*1*
0	*0*	*0*	*130*	*0*	*0*	*1*	*0*	*2*
0	*0*	*0*	*77,1429*	*34,2857*	*0*	*1*	*0*	*2*
0	*0*	*0*	*51,4286*	*25,7143*	*0*	*0*	*0*	*1*
0	*0*	*0*	*40*	*171,429*	*25,7143*	*0*	*1*	*2*
0	*0*	*0*	*17,1429*	*25,7143*	*0*	*0*	*0*	*1*
0	*0*	*0*	*154,286*	*128,571*	*145,714*	*1*	*1*	*2*
0	*0*	*0*	*67,1429*	*21,4286*	*0*	*1*	*0*	*1*
0	*0*	*0*	*17,1429*	*94,2857*	*17,1429*	*0*	*1*	*2*
0	*0*	*0*	*9,42857*	*36*	*17,1429*	*0*	*0*	*2*
0	*0*	*0*	*5,71429*	*0*	*0*	*0*	*0*	*2*
0	*0*	*0*	*37,1429*	*81,4286*	*8,57143*	*0*	*1*	*2*
0	*0*	*0*	*25,7143*	*15*	*0*	*0*	*0*	*3*
0	*0*	*0*	*185,714*	*102,857*	*0*	*1*	*1*	*2*
0	*0*	*0*	*38,5714*	*94,2857*	*0*	*0*	*1*	*1*
0	*0*	*0*	*42,8571*	*51,4286*	*37,1429*	*0*	*1*	*1*
0	*0*	*0*	*34,2857*	*34,2857*	*1,42857*	*0*	*0*	*2*
0	*0*	*0*	*81,4286*	*38,5714*	*0*	*1*	*0*	*1*
0	*0*	*0*	*30*	*0*	*0*	*0*	*0*	*2*
0	*0*	*0*	*8,57143*	*14,2857*	*20*	*0*	*0*	*2*
0	*0*	*0*	*128,571*	*171,429*	*0*	*1*	*1*	*1*

0	0	0	0	65,7143	0	0	1	2
0	0	1	40	40	0	0	0	2
0	0	0	42,8571	81,4286	0	0	1	1
0	0	0	21,4286	17,1429	12,8571	0	0	2
0	0	0	203,571	55,7143	25,7143	1	1	1
0	0	0	184,286	197,143	25,7143	1	1	1
0	0	0	6,42857	8,57143	8,57143	0	0	1
0	0	0	0	0	0	0	0	1
0	0	0	0	171,429	34,2857	0	1	2
0	0	0	8,57143	21,4286	17,1429	0	0	2
0	0	1	51,4286	174,286	0	0	1	2
0	0	0	30	51,4286	2,85714	0	0	1
0	0	0	37,8571	28,5714	0	0	0	2
0	0	0	17,1429	31,4286	21,4286	0	0	2
0	0	0	141,429	128,571	25,7143	1	1	2
0	0	0	120	64,2857	0	1	1	3
0	1	1	80	8,57143	0	1	0	2
0	0	0	145,714	60	17,1429	1	1	2
0	0	0	0	60	17,1429	0	1	2
0	0	0	30	171,429	25,7143	0	1	2
0	0	0	0	231,429	0	0	1	2
1	0	1	47,1429	94,2857	0	0	1	2
0	0	0	55,7143	20	0	0	0	1
0	0	0	6,42857	34,2857	0	0	0	1
0	0	0	0	180	0	0	1	1
0	0	0	248,571	134,286	31,4286	1	1	2
0	0	0	51,4286	128,571	0	0	1	2
0	0	0	17,1429	0	51,4286	0	0	2
0	0	0	42,8571	34,2857	0	0	0	2
0	0	0	0	0	30	0	0	1
0	0	0	21,4286	51,4286	0	0	0	2
0	0	0	11,4286	42,8571	11,4286	0	0	2
0	1	1	248,571	82,8571	0	1	1	2
0	0	0	0	0	8,57143	0	0	2
0	0	0	60	0	25,7143	1	0	1
0	0	0	12,8571	137,143	8,57143	0	1	2
0	0	0	51,4286	60	0	0	1	2
0	0	0	140,714	128,571	60	1	1	2
0	0	0	4,28571	11,4286	0	0	0	1
0	0	0	25,7143	17,1429	34,2857	0	0	1
0	0	0	77,1429	132,857	0	1	1	2
0	0	0	162,857	167,143	0	1	1	2
0	0	0	30	2,85714	0	0	0	2
0	0	0	120	85,7143	51,4286	1	1	1
0	0	0	62,8571	180	51,4286	1	1	2
0	0	0	8,57143	27,8571	0	0	0	2
0	0	0	62,8571	81,4286	0	1	1	2
0	0	0	8,57143	154,286	0	0	1	1

0	0	0	14,2857	8,57143	0	0	0	2
0	0	0	0	0	17,1429	0	0	2
0	0	0	188,571	0	0	1	0	2
1	0	1	120	120	51,4286	1	1	2
0	0	0	25,7143	37,1429	0	0	0	2
0	0	0	12,8571	25,7143	0	0	0	2
0	0	0	27,1429	36,4286	8,57143	0	0	2
1	0	1	11,4286	8,57143	85,7143	0	1	2
0	0	0	82,8571	60	0	1	1	2
0	0	0	12,8571	128,571	0	0	1	2
0	0	0	22,1429	57,1429	0	0	0	2
0	0	0	0	34,2857	0	0	0	1
0	0	0	96,4286	8,57143	0	1	0	1
0	0	0	20	171,429	77,1429	0	1	2
0	0	0	85,7143	90	180	1	1	1
0	1	1	188,571	137,143	42,8571	1	1	2
0	0	0	51,4286	72,8571	25,7143	0	1	2
0	0	0	51,4286	42,8571	0	0	0	2
0	0	0	102,857	141,429	51,4286	1	1	2
0	0	0	128,571	215,714	25,7143	1	1	2
0	0	0	128,571	5,71429	60	1	1	2
0	0	0	25,7143	0	3,42857	0	0	2
0	0	1	60	38,5714	0	1	0	2
0	0	0	137,143	128,571	42,8571	1	1	2
0	0	0	102,857	34,2857	0	1	0	2
0	0	0	0	0	17,1429	0	0	2
0	0	0	197,143	282,857	154,286	1	1	2
0	0	0	162,857	60	25,7143	1	1	2
0	0	0	11,4286	77,1429	0	0	1	2
0	1	1	81,4286	171,429	51,4286	1	1	2
0	0	0	5,71429	8,57143	0	0	0	2
0	0	0	88,5714	5,71429	0	1	0	2
0	0	0	66,4286	60	0	1	1	2
0	0	0	80	158,571	17,1429	1	1	2
0	0	0	0	34,2857	8,57143	0	0	2
0	0	0	134,286	180	0	1	1	1
0	0	0	79,2857	0	0	1	0	2
0	0	0	0	8,57143	0	0	0	1
0	0	0	51,4286	188,571	94,2857	0	1	2
0	0	0	11,4286	128,571	0	0	1	2
0	0	0	0	0	0	0	0	2
0	0	0	48,5714	0	0	0	0	2
0	0	0	38,5714	51,4286	0	0	0	1
0	0	0	85,7143	214,286	0	1	1	1
0	0	0	93,5714	51,4286	8,57143	1	1	2
0	0	0	128,571	154,286	8,57143	1	1	3
0	1	1	248,571	51,4286	0	1	0	2
0	0	0	51,4286	2,85714	0	0	0	1

0	0	0	12,8571	81,4286	8,57143	0	1	2
0	0	0	244,286	154,286	25,7143	1	1	1
1	0	1	5,71429	154,286	94,2857	0	1	2
0	0	0	80	17,1429	2,85714	1	0	2
0	0	0	32	0	0	0	0	2
0	0	0	15,7143	0	25,7143	0	0	2
0	0	0	111,429	205,714	51,4286	1	1	1
0	0	0	53,5714	51,4286	0	0	0	2
0	0	0	132,857	137,143	25,7143	1	1	1
0	0	0	77,1429	137,143	0	1	1	2
0	0	0	97,1429	51,4286	0	1	0	2
0	0	0	102,857	257,143	94,2857	1	1	2
0	0	0	2,14286	16,4286	0	0	0	2
0	0	0	30	0	8,57143	0	0	1
0	1	1	177,143	4,28571	0	1	0	1
0	0	0	7,14286	0	0	0	0	2
0	0	0	308,571	308,571	145,714	1	1	2
0	0	0	85	128,571	0	1	1	1
0	0	0	60	17,1429	25,7143	1	0	1
0	0	0	102,857	222,857	25,7143	1	1	2
0	0	0	0	162,857	0	0	1	1
0	0	0	222,857	120	17,1429	1	1	2
0	0	0	11,4286	0	85,7143	0	1	2
0	0	0	34,2857	4,28571	0	0	0	2
0	0	0	5,71429	10	0	0	0	2
0	0	0	0	205,714	0	0	1	1
0	0	0	30	141,429	0	0	1	3
0	0	0	108,571	154,286	25,7143	1	1	2
0	0	0	30	68,5714	0	0	1	2
0	0	0	16,4286	12,8571	0	0	0	2
0	0	0	107,143	180	0	1	1	1
0	0	0	128,571	110	25,7143	1	1	2
0	0	0	21,4286	8,57143	0	0	0	2
0	1	1	312,857	274,286	0	1	1	2
1	0	1	4,28571	111,429	0	0	1	2
0	1	1	60	25,7143	0	1	0	1
0	0	0	0	34,2857	0	0	0	1
1	0	1	50	94,2857	77,1429	0	1	2
0	0	0	60	94,2857	68,5714	1	1	2
0	0	0	154,286	158,571	17,1429	1	1	2
0	0	0	128,571	137,143	0	1	1	2
0	0	0	12,8571	8,57143	0	0	0	2
0	0	0	128,571	81,4286	0	1	1	2
0	0	0	0	38,5714	0	0	0	2
0	0	0	47,1429	145,714	17,1429	0	1	2
0	0	0	71,4286	77,1429	8,57143	1	1	2
0	0	0	145,714	197,143	51,4286	1	1	1
0	0	0	17,1429	47,1429	0	0	0	1

0	0	0	40	163,571	0	0	1	1
0	0	0	21,4286	25,7143	0	0	0	2
0	0	0	154,286	300	0	1	1	2
0	0	0	85,7143	137,143	0	1	1	1
0	0	1	138,571	17,1429	60	1	1	1
0	0	0	34,2857	14,2857	0	0	0	2
0	0	0	137,143	137,143	0	1	1	1
0	0	0	0	154,286	0	0	1	2
0	0	0	145,714	158,571	68,5714	1	1	2
0	0	0	25,7143	102,857	51,4286	0	1	2
0	0	0	51,4286	150	102,857	0	1	2
0	0	0	47,1429	8,57143	0	0	0	2
0	0	0	25,7143	17,1429	154,286	0	1	2
0	0	0	128,571	11,1429	0	1	0	2
0	0	0	12,8571	0	0	0	0	1
0	0	0	154,286	36,4286	0	1	0	1
0	0	0	7,14286	4,28571	12,8571	0	0	2
0	0	0	8,57143	4,28571	17,1429	0	0	2
0	0	0	52,8571	4,28571	0	0	0	2
0	0	0	7,14286	0	17,1429	0	0	2
0	0	0	34,2857	25,7143	0	0	0	2
0	0	0	85,7143	85,7143	0	1	1	1
0	0	0	162,857	120	25,7143	1	1	2
0	0	0	137,143	231,429	0	1	1	1
0	0	0	111,429	8,57143	0	1	0	2
0	0	0	77,1429	0	154,286	1	1	1
0	1	1	222,857	77,1429	0	1	1	2
0	0	0	50	28,5714	25,7143	0	0	2
0	0	0	282,857	21,4286	0	1	0	2
0	0	0	8,57143	25,7143	0	0	0	2
0	0	0	126,429	17,1429	0	1	0	2
0	1	1	240	62,1429	51,4286	1	1	2
0	0	0	115,714	94,2857	0	1	1	1
1	0	1	42,8571	40	40	0	1	3
0	0	0	42,8571	102,857	0	0	1	1
0	0	0	55,7143	17,1429	0	0	0	2
0	0	0	12,8571	51,4286	0	0	0	1
0	0	0	34,2857	42,8571	0	0	0	1
0	0	0	62,8571	0	0	1	0	2
0	0	0	34,2857	30	0	0	0	1
0	1	1	60	11,4286	0	1	0	3
0	0	0	21,4286	25,7143	0	0	0	3
0	0	0	125,714	94,2857	0	1	1	2
0	1	1	188,571	8,57143	0	1	0	2
0	0	0	14,2857	21,4286	0	0	0	1
0	0	0	17,1429	60	8,57143	0	1	2
0	0	0	17,1429	180	0	0	1	2
0	0	0	17,1429	42,8571	0	0	0	1

0	0	0	27,8571	21,4286	0	0	0	2
1	0	1	71,4286	145,714	0	1	1	2
0	0	0	30	60	0	0	1	2
0	0	0	55,7143	17,1429	0	0	0	1
0	0	0	8,57143	102,857	0	0	1	2
0	0	0	30	90	12,8571	0	1	3
0	0	0	0	25,7143	68,5714	0	1	1
0	0	0	15,7143	25,7143	0	0	0	1
0	0	0	7,14286	0	0	0	0	2
0	0	0	81,4286	37,1429	25,7143	1	1	2
0	0	0	11,4286	51,4286	0	0	0	2
0	1	1	60	34,2857	0	1	0	2
0	0	0	0	0	0	0	0	2
0	0	0	28,5714	51,4286	0	0	0	1
0	0	0	5,71429	51,4286	0	0	0	3
0	0	0	145,714	49,2857	0	1	0	2
0	0	0	25,7143	23,5714	0	0	0	2
0	0	0	89,2857	171,429	17,1429	1	1	1
0	0	0	17,1429	68,5714	0	0	1	1
0	0	0	8,57143	77,1429	6,42857	0	1	3
0	0	0	60	85,7143	0	1	1	2
0	0	0	81,4286	25,7143	0	1	0	1
0	0	0	5,71429	51,4286	0	0	0	2
0	1	1	72,8571	17,1429	0	1	0	1
0	1	1	85,7143	0	0	1	0	2
0	0	0	0	25,7143	0	0	0	2
0	0	0	6,42857	55,7143	0	0	0	2
0	0	0	21,4286	0	0	0	0	1
0	0	1	85,7143	51,4286	0	1	0	1
0	0	0	21,4286	0	17,1429	0	0	2
0	0	0	5,71429	0	17,1429	0	0	2
0	0	0	120	228,571	180	1	1	2
0	0	0	51,4286	25,7143	0	0	0	1
0	0	0	120	68,5714	0	1	1	2
0	0	0	0	31,4286	0	0	0	2
0	0	0	0	85,7143	0	0	1	1
0	0	0	34,2857	25,7143	0	0	0	2
0	1	1	74,2857	40	0	1	0	2
0	0	0	60	78,5714	0	1	1	2
0	0	0	85,7143	85,7143	0	1	1	1
0	0	0	34,2857	25,7143	32,1429	0	0	1
0	0	1	75	98,5714	0	1	1	2
0	1	1	145,714	8,57143	0	1	0	1
0	0	0	21,4286	34,2857	0	0	0	2
0	0	0	102,857	154,286	0	1	1	1
0	0	0	42,8571	0	0	0	0	2
0	0	0	17,1429	77,1429	0	0	1	3
0	0	0	0	180	34,2857	0	1	2

0	0	0	0	51,4286	2,85714	0	0	1
0	0	0	54,2857	0	8,57143	0	0	2
0	0	0	14,2857	42,8571	0	0	0	1
0	0	0	60	120	0	1	1	1
0	0	0	54,2857	17,1429	0	0	0	2
0	0	0	90	51,4286	0	1	0	2
0	0	0	15	38,5714	0	0	0	2
0	0	0	0	77,1429	0	0	1	2
0	0	0	17,1429	137,143	0	0	1	2
0	0	0	60	30	0	1	0	2
0	0	0	141,429	231,429	25,7143	1	1	1
0	0	0	0	34,2857	8,57143	0	0	2
0	0	0	0	90	0	0	1	1
0	0	0	120	38,5714	0	1	0	2
0	0	0	55,7143	25,7143	0	0	0	2
0	0	0	21,4286	197,143	0	0	1	2
0	1	1	120	28,5714	0	1	0	2
0	0	0	45,7143	42,8571	0	0	0	2
0	0	0	8,57143	25,7143	0	0	0	2
0	0	0	30	205,714	0	0	1	3
0	0	0	0	94,2857	0	0	1	2
0	0	0	47,1429	20	0	0	0	2
0	0	0	162,857	188,571	0	1	1	2
0	0	0	8,57143	60	0	0	1	2
0	0	0	8,57143	34,2857	0	0	0	1
0	0	0	31,4286	30	0	0	0	3
0	0	0	167,143	34,2857	0	1	0	2
0	0	0	0	34,2857	25,7143	0	1	2
0	0	0	2,85714	17,1429	0	0	0	1
0	0	0	34,2857	34,2857	0	0	0	2
0	0	0	54,2857	17,1429	0	0	0	2
0	0	0	51,4286	47,1429	0	0	0	1
0	0	0	34,2857	188,571	0	0	1	2
0	0	0	17,1429	137,143	0	0	1	2
0	0	0	10	8,57143	0	0	0	1
0	0	0	0	45	0	0	0	3
0	0	0	57,1429	5,71429	0	0	0	2
0	0	0	17,1429	34,2857	0	0	0	1
0	0	0	68,5714	25,7143	0	1	0	2
0	0	0	47,1429	37,1429	0	0	0	2
0	0	0	20	30	0	0	0	2
0	0	0	68,5714	49,2857	0	1	0	2
0	0	0	30	25,7143	0	0	0	2
0	0	0	21,4286	17,1429	0	0	0	2
0	0	0	2,14286	17,1429	0	0	0	2
0	0	0	30	51,4286	0	0	0	1
0	0	0	5,71429	97,1429	0	0	1	2
0	0	0	17,1429	25,7143	0	0	0	2

0	0	0	14,2857	51,4286	0	0	0	2
0	0	0	8,57143	60	128,571	0	1	1
0	0	0	35,7143	95,7143	0	0	1	2
0	0	0	25,7143	62,8571	0	0	1	2
0	0	0	54,2857	8,57143	2,85714	0	0	2
0	0	1	72,8571	6,42857	42,8571	1	0	2
0	0	0	21,4286	10,7143	10,7143	0	0	2
0	0	0	20	6,42857	0	0	0	2
0	1	1	120	75	0	1	1	2
0	0	0	0	77,1429	0	0	1	1
0	0	0	148,571	60	0	1	1	2
0	0	0	24,2857	25,7143	0	0	0	2
0	0	0	47,1429	14,2857	0	0	0	2
0	0	0	25,7143	17,1429	0	0	0	3
0	1	1	300	18,5714	0	1	0	2
0	0	0	20	77,1429	0	0	1	2
0	0	0	55,7143	36,4286	0	0	0	2
0	0	0	5,71429	17,1429	17,1429	0	0	3
0	0	0	23,5714	68,5714	0	0	1	2
0	0	0	51,4286	42,8571	0	0	0	1
0	0	0	28,5714	36,4286	0	0	0	2
0	0	0	0	180	0	0	1	1
0	0	0	25,7143	17,1429	0	0	0	2
0	0	0	94,2857	0	0	1	0	1
0	0	0	21,4286	28,5714	0	0	0	1
0	0	0	171,429	25,7143	0	1	0	1
0	0	0	38,5714	85,7143	0	0	1	2
0	0	0	60	51,4286	0	1	0	2
0	0	0	30	282,857	0	0	1	2
0	0	0	51,4286	0	0	0	0	3
0	0	0	0	17,1429	0	0	0	1
0	0	0	145,714	49,2857	0	1	0	1
0	0	0	87,8571	30	0	1	0	1
0	0	0	7,14286	8,57143	0	0	0	1
0	0	0	12,8571	128,571	0	0	1	2
0	0	0	4,28571	60	17,1429	0	1	1
0	0	0	37,8571	154,286	0	0	1	2
0	1	1	68,5714	34,2857	0	1	0	2
0	0	0	11,4286	77,1429	0	0	1	1
0	0	0	150	51,4286	0	1	0	3
0	1	1	308,571	120	0	1	1	3
0	0	0	21,4286	38,5714	0	0	0	1
0	0	0	77,1429	51,4286	0	1	0	2
0	0	0	21,4286	42,8571	0	0	0	2
0	0	0	10,7143	85,7143	0	0	1	2
0	0	0	30	34,2857	0	0	0	2
0	0	0	7,14286	94,2857	0	0	1	2
0	0	0	42,8571	0	0	0	0	2

0	0	0	47,1429	25,7143	12,8571	0	0	2
0	0	0	128,571	214,286	34,2857	1	1	2
0	0	0	65,7143	120	0	1	1	2
0	0	0	0	38,5714	0	0	0	2
0	0	0	34,2857	102,857	0	0	1	2
0	0	0	0	30	0	0	0	1
0	0	0	30	0	0	0	0	1
0	0	0	45,7143	12,8571	0	0	0	1
0	0	0	8,57143	17,1429	0	0	0	1
0	1	1	90	162,857	120	1	1	2
0	0	0	10	105,714	0	0	1	2
0	0	0	21,4286	42,8571	0	0	0	2
0	0	0	51,4286	51,4286	0	0	0	2
0	0	0	11,4286	17,1429	0	0	0	1
0	0	0	6,42857	74,2857	0	0	1	2
0	0	0	38,5714	25,7143	0	0	0	2
0	0	0	42,8571	34,2857	25,7143	0	1	1
0	0	0	60	42,8571	0	1	0	2
0	0	0	167,143	30	0	1	0	2
0	0	0	14,2857	25,7143	0	0	0	1
0	0	0	38,5714	11,4286	0	0	0	2
0	0	0	8,57143	80	0	0	1	2
0	0	0	40	25,7143	17,1429	0	0	1
0	0	0	25,7143	137,143	0	0	1	2
0	1	1	150	248,571	0	1	1	1
0	0	0	30	42,8571	0	0	0	3
0	0	0	71,4286	34,2857	0	1	0	2
0	0	0	21,4286	85,7143	0	0	1	2
0	0	0	24,2857	17,1429	0	0	0	1
0	0	0	20	4,28571	0	0	0	3
0	0	0	64,2857	42,8571	0	1	0	2
0	0	0	0	115,714	0	0	1	2
0	0	0	31,4286	74,2857	0	0	1	1
0	1	1	130	60	0	1	1	2
0	0	0	68,5714	34,2857	0	1	0	1
1	1	1	240	137,143	0	1	1	1
0	0	0	25,7143	25,7143	0	0	0	1
0	1	1	134,286	70,7143	0	1	1	3
0	0	0	5,71429	31,4286	0	0	0	2
0	0	0	25,7143	0	0	0	0	2
0	0	0	85,7143	128,571	180	1	1	2
0	1	1	201,429	42,8571	0	1	0	2
0	0	0	2,85714	128,571	0	0	1	3
0	0	0	35,7143	120	0	0	1	2
0	0	0	22,8571	47,1429	21,4286	0	1	2
0	0	0	0	42,8571	34,2857	0	1	2
0	0	0	8,57143	17,1429	0	0	0	3
0	1	1	81,4286	180	0	1	1	2

0	0	0	22,8571	85,7143	0	0	1	3
0	0	0	37,1429	64,2857	0	0	1	2
0	0	0	0	60	0	0	1	2
0	0	0	21,4286	107,143	0	0	1	2
0	0	1	85,7143	60	0	1	1	1
0	0	0	17,8571	70,7143	0	0	1	2
0	0	0	83,5714	34,2857	0	1	0	1
0	0	0	20	55	0	0	0	1
0	0	0	14,2857	145,714	0	0	1	1
0	0	0	0	91,4286	0	0	1	1
0	0	0	40	77,1429	0	0	1	1
0	0	0	47,1429	62,8571	0	0	1	1
0	1	1	120	85,7143	25,7143	1	1	3
0	0	0	2,85714	51,4286	0	0	0	2
0	0	0	45,7143	25,7143	0	0	0	1
0	0	0	32,8571	21,4286	0	0	0	2
0	1	1	227,143	184,286	17,1429	1	1	1
0	1	1	81,4286	37,1429	32,1429	1	1	2
0	1	1	60	17,1429	0	1	0	2
0	0	0	17,1429	34,2857	0	0	0	1
0	0	0	30	17,1429	0	0	0	2
0	0	0	0	0	0	0	0	2
0	0	0	37,1429	8,57143	0	0	0	2
0	0	0	171,429	180	0	1	1	2
0	0	0	68,5714	25,7143	0	1	0	1
0	0	0	17,1429	162,857	0	0	1	1
0	0	0	42,8571	25,7143	0	0	0	2
0	0	0	51,4286	51,4286	0	0	0	2
0	0	0	165,714	192,857	8,57143	1	1	2
0	0	0	51,4286	90	0	0	1	1
0	1	1	210	34,2857	0	1	0	1
0	1	1	150	154,286	0	1	1	2
0	0	0	38,5714	64,2857	0	0	1	1
0	0	0	22,8571	4,28571	0	0	0	3
0	0	0	34,2857	0	0	0	0	1
0	0	0	38,5714	60	0	0	1	1
0	0	0	14,2857	64,2857	42,8571	0	1	2
0	0	0	25,7143	30	0	0	0	1
0	0	0	20	120	0	0	1	2
0	0	0	28,5714	111,429	0	0	1	2
0	1	1	128,571	248,571	0	1	1	1
0	0	0	94,2857	51,4286	0	1	0	2
0	0	0	55,7143	8,57143	85,7143	0	1	3
0	0	0	25,7143	120	0	0	1	2
0	0	0	5,71429	40	0	0	0	2
0	0	0	2,14286	34,2857	0	0	0	2
0	0	0	51,4286	51,4286	0	0	0	2
0	0	0	60	17,1429	0	1	0	2

0	*0*	*0*	*64,2857*	*25,7143*	*42,8571*	*1*	*1*	*3*
0	*0*	*0*	*3,57143*	*60*	*0*	*0*	*1*	*2*
0	*1*	*1*	*117,857*	*60*	*0*	*1*	*1*	*1*
0	*0*	*1*	*47,1429*	*77,1429*	*51,4286*	*0*	*1*	*3*
0	*0*	*0*	*45*	*34,2857*	*0*	*0*	*0*	*2*
0	*0*	*0*	*8,57143*	*134,286*	*0*	*0*	*1*	*2*
0	*0*	*0*	*130,714*	*14,2857*	*0*	*1*	*0*	*1*
0	*1*	*1*	*126,429*	*49,2857*	*0*	*1*	*0*	*2*
0	*0*	*0*	*34,2857*	*265,714*	*0*	*0*	*1*	*2*
0	*0*	*0*	*2,85714*	*60*	*0*	*0*	*1*	*2*
0	*0*	*0*	*8,57143*	*0*	*17,1429*	*0*	*0*	*1*
0	*0*	*0*	*17,1429*	*38,5714*	*12,8571*	*0*	*0*	*2*
0	*0*	*0*	*21,4286*	*21,4286*	*0*	*0*	*0*	*1*
0	*0*	*0*	*8,57143*	*102,857*	*0*	*0*	*1*	*1*
0	*0*	*0*	*35,7143*	*17,1429*	*0*	*0*	*0*	*1*
0	*0*	*0*	*23,5714*	*17,1429*	*0*	*0*	*0*	*1*
0	*0*	*0*	*38,5714*	*180*	*0*	*0*	*1*	*2*
0	*0*	*1*	*71,4286*	*37,1429*	*0*	*1*	*0*	*3*
0	*0*	*0*	*60*	*20*	*120*	*1*	*1*	*2*
0	*0*	*0*	*57,1429*	*17,1429*	*0*	*0*	*0*	*3*
0	*0*	*0*	*12,8571*	*42,8571*	*25,7143*	*0*	*1*	*2*
0	*1*	*1*	*60*	*171,429*	*0*	*1*	*1*	*1*
0	*1*	*1*	*111,429*	*42,8571*	*0*	*1*	*0*	*1*
0	*1*	*1*	*68,5714*	*17,1429*	*0*	*1*	*0*	*2*
0	*0*	*0*	*30*	*17,1429*	*51,4286*	*0*	*1*	*1*
0	*0*	*0*	*25,7143*	*51,4286*	*0*	*0*	*0*	*2*
0	*1*	*1*	*120*	*0*	*0*	*1*	*0*	*2*
0	*0*	*0*	*72,8571*	*51,4286*	*0*	*1*	*0*	*1*
0	*0*	*0*	*0*	*42,8571*	*0*	*0*	*0*	*2*
0	*0*	*0*	*42,8571*	*158,571*	*0*	*0*	*1*	*2*
0	*0*	*1*	*111,429*	*291,429*	*0*	*1*	*1*	*2*
0	*0*	*0*	*128,571*	*174,286*	*0*	*1*	*1*	*1*
0	*0*	*0*	*120*	*0*	*0*	*1*	*0*	*1*
0	*0*	*0*	*68,5714*	*47,1429*	*0*	*1*	*0*	*2*
0	*0*	*0*	*53,5714*	*34,2857*	*0*	*0*	*0*	*2*
0	*0*	*0*	*55,7143*	*30*	*0*	*0*	*0*	*2*
0	*0*	*1*	*85,7143*	*17,1429*	*0*	*1*	*0*	*2*
0	*0*	*0*	*35,7143*	*128,571*	*154,286*	*0*	*1*	*2*
0	*0*	*0*	*28,5714*	*64,2857*	*0*	*0*	*1*	*2*
0	*0*	*0*	*137,143*	*17,1429*	*0*	*1*	*0*	*3*
0	*0*	*0*	*31,4286*	*38,5714*	*0*	*0*	*0*	*2*
0	*0*	*0*	*25,7143*	*51,4286*	*0*	*0*	*0*	*3*
0	*0*	*0*	*51,4286*	*51,4286*	*0*	*0*	*0*	*2*
0	*0*	*0*	*51,4286*	*42,8571*	*0*	*0*	*0*	*1*
0	*0*	*0*	*17,1429*	*32,8571*	*0*	*0*	*0*	*2*
0	*0*	*0*	*40*	*17,1429*	*0*	*0*	*0*	*2*
0	*0*	*0*	*8,57143*	*60*	*34,2857*	*0*	*1*	*2*
0	*0*	*0*	*19,2857*	*17,1429*	*0*	*0*	*0*	*2*

0	1	1	60	8,57143	0	1	0	2
0	0	0	154,286	32,1429	4,28571	1	0	1
0	0	0	60	300	0	1	1	3
0	0	0	62,8571	4,28571	19,2857	1	0	1
0	0	0	34,2857	0	0	0	0	2
0	1	1	60	25,7143	0	1	0	2
0	0	0	0	42,8571	0	0	0	3
0	0	0	77,1429	27,8571	0	1	0	2
0	0	0	30	77,1429	34,2857	0	1	1
0	0	0	38,5714	34,2857	0	0	0	1
0	0	0	111,429	90	0	1	1	2
0	0	0	85,7143	60	0	1	1	2
0	1	1	81,4286	24,2857	0	1	0	1
0	0	0	4,28571	17,1429	8,57143	0	0	2
0	0	0	34,2857	102,857	0	0	1	2
0	0	0	145,714	60	0	1	1	2
0	0	1	111,429	42,8571	25,7143	1	1	1
0	1	1	60	0	0	1	0	2
0	0	0	0	60	0	0	1	2
0	0	0	77,1429	25,7143	0	1	0	2
0	0	1	171,429	34,2857	15	1	0	2
0	0	0	34,2857	51,4286	0	0	0	2
0	0	0	36,4286	12,8571	0	0	0	2
0	0	0	35	8,57143	0	0	0	2
0	0	0	21,4286	17,1429	0	0	0	2
0	0	0	21,4286	0	0	0	0	2
0	0	0	28,5714	42,8571	0	0	0	2
0	0	0	10	57,8571	0	0	0	3
0	0	0	64,2857	51,4286	0	1	0	3
0	0	0	55,7143	64,2857	0	0	1	2
0	0	0	20	51,4286	0	0	0	2
0	1	1	191,429	23,5714	0	1	0	1
0	1	1	120	17,1429	0	1	0	3
0	0	0	25,7143	25,7143	0	0	0	2
0	0	0	15	8,57143	0	0	0	2
0	0	0	51,4286	201,429	0	0	1	1
0	0	0	12,8571	51,4286	0	0	0	2
0	0	0	34,2857	42,8571	0	0	0	3
0	1	1	145,714	1,42857	0	1	0	2
0	1	1	205,714	102,857	0	1	1	2
0	0	0	45,7143	37,1429	8,57143	0	0	1
0	0	0	102,857	68,5714	0	1	1	2
0	0	0	0	145,714	0	0	1	2
0	0	0	0	135	0	0	1	1
0	0	1	81,4286	98,5714	0	1	1	2
0	1	1	240	51,4286	0	1	0	2
0	1	1	85,7143	30	0	1	0	2
0	1	1	110	120	0	1	1	1

0	0	0	30	180	12,8571	0	1	1
0	1	1	94,2857	147,857	0	1	1	2
0	0	0	34,2857	98,5714	0	0	1	2
0	0	1	57,8571	15,7143	0	0	0	2
0	1	1	80	62,1429	0	1	1	2
0	0	0	60	38,5714	0	1	0	1
0	0	0	11,4286	64,2857	0	0	1	3
0	0	0	20	0	0	0	0	2
0	0	0	0	128,571	0	0	1	2
0	0	0	102,857	90	0	1	1	1
0	0	0	17,1429	94,2857	0	0	1	1
0	0	0	12,8571	40,7143	0	0	0	2
0	0	0	51,4286	17,1429	0	0	0	2
0	0	0	17,1429	25,7143	0	0	0	1
0	0	0	75	17,1429	0	1	0	1
0	0	0	158,571	334,286	0	1	1	1
0	1	1	68,5714	0	17,1429	1	0	2
0	0	0	34,2857	77,1429	17,1429	0	1	2
0	0	0	157,143	17,1429	0	1	0	2
0	0	0	40	8,57143	0	0	0	2
0	0	0	17,1429	51,4286	0	0	0	1
0	0	0	34,2857	51,4286	0	0	0	2
0	0	0	85,7143	60	0	1	1	2
0	0	0	35,7143	21,4286	0	0	0	2
0	0	0	0	120	0	0	1	2
0	0	0	57,8571	17,1429	0	0	0	3
0	1	1	150	120	0	1	1	1
0	0	0	51,4286	42,8571	0	0	0	1
0	0	0	0	34,2857	0	0	0	2
0	0	0	68,5714	10	0	1	0	2
0	0	0	28,5714	94,2857	0	0	1	2
0	0	0	40	41,4286	4,28571	0	0	1
0	0	0	51,4286	17,1429	0	0	0	2
0	0	0	42,8571	42,8571	0	0	0	2
0	1	1	180	34,2857	0	1	0	1
0	0	0	56,4286	45	0	0	0	2
0	1	1	60	68,5714	0	1	1	2
0	1	1	98,5714	21,4286	0	1	0	2
0	0	0	38,5714	180	0	0	1	3
0	0	0	34,2857	28,5714	0	0	0	1
0	0	0	45,7143	23,5714	0	0	0	2
0	0	0	94,2857	0	4,28571	1	0	2
0	0	0	184,286	17,1429	0	1	0	1
0	0	0	34,2857	42,8571	0	0	0	2
0	0	0	4,28571	30	0	0	0	2
0	0	0	5,71429	145,714	0	0	1	2
0	0	0	80,7143	25,7143	45	1	1	1
0	0	0	55,7143	42,8571	0	0	0	2

0	1	1	102,857	154,286	0	1	1	1
0	0	0	40	64,2857	0	0	1	1
0	0	0	38,5714	0	0	0	0	1
0	1	1	77,1429	25,7143	0	1	0	2
0	0	0	27,8571	57,1429	8,57143	0	1	1
0	0	0	42,8571	77,1429	0	0	1	1
0	0	0	68,5714	0	0	1	0	2
0	1	1	75	21,4286	0	1	0	1
0	0	0	77,1429	137,143	0	1	1	2
0	0	0	0	94,2857	0	0	1	2
0	0	0	48,5714	8,57143	0	0	0	1
0	0	0	45	102,857	0	0	1	1
0	0	0	34,2857	0	0	0	0	1
0	0	0	8,57143	126,429	0	0	1	2
0	1	1	75	68,5714	120	1	1	2
0	0	0	60	60	0	1	1	1
0	0	0	17,1429	42,8571	8,57143	0	0	2
0	0	0	19,2857	171,429	0	0	1	2
0	1	1	195	4,28571	0	1	0	3
0	0	0	115,714	42,8571	0	1	0	1
0	1	1	180	42,8571	64,2857	1	1	2
0	1	1	80	25,7143	0	1	0	2
0	0	0	60	85,7143	0	1	1	2
0	1	1	60	107,143	0	1	1	3
0	0	0	11,4286	65,7143	0	0	1	1
0	1	1	165	23,5714	17,1429	1	0	2
0	0	0	12,8571	25,7143	0	0	0	2
0	0	0	2,85714	180	0	0	1	2
0	0	0	49,2857	11,4286	0	0	0	1
0	0	0	21,4286	51,4286	11,4286	0	1	1
0	0	0	17,1429	77,1429	0	0	1	2
0	0	0	65,7143	21,4286	0	1	0	1
0	0	0	14,2857	77,1429	0	0	1	2
0	0	0	42,8571	51,4286	0	0	0	2
0	0	0	30	165	0	0	1	3
0	0	0	22,8571	60	0	0	1	3
0	0	0	64,2857	214,286	0	1	1	2
0	1	1	75	94,2857	0	1	1	2
0	0	0	2,85714	0	40	0	0	1
0	0	0	8,57143	42,8571	0	0	0	1
0	1	1	98,5714	47,1429	0	1	0	2
0	0	0	30	197,143	0	0	1	2
0	0	0	68,5714	0	0	1	0	1
0	0	0	8,57143	142,857	51,4286	0	1	1
0	0	0	102,857	77,1429	0	1	1	2
0	0	0	68,5714	34,2857	0	1	0	1
0	0	0	10	34,2857	0	0	0	2
1	0	1	171,429	0	85,7143	1	1	2

0	0	0	102,857	205,714	128,571	1	1	2
0	1	1	111,429	25,7143	0	1	0	2
0	0	0	8,57143	0	0	0	0	1
0	0	0	54,2857	17,1429	8,57143	0	0	1
0	0	0	162,857	102,857	0	1	1	1
0	0	0	49,2857	0	0	0	0	3
0	0	0	25,7143	154,286	0	0	1	2
0	0	0	12,8571	31,4286	8,57143	0	0	1
0	0	0	15	8,57143	0	0	0	2
0	0	0	0	102,857	0	0	1	3
0	0	0	94,2857	145,714	0	1	1	3
0	1	1	120	38,5714	0	1	0	3
0	0	0	23,5714	85,7143	0	0	1	3
0	0	0	85,7143	42,8571	0	1	0	1
0	0	0	25,7143	35,7143	2,14286	0	0	1
0	0	0	8,57143	0	0	0	0	1
0	0	0	80	0	0	1	0	2
0	0	0	28,5714	77,1429	0	0	1	2
0	1	1	60	77,1429	17,1429	1	1	2
0	0	0	17,1429	17,1429	0	0	0	2
0	0	0	55	47,8571	0	0	0	1
0	0	0	42,8571	51,4286	0	0	0	2
0	0	0	25,7143	68,5714	0	0	1	2
0	1	1	102,857	60	0	1	1	2
0	0	1	47,1429	32,1429	30	0	1	2
0	1	1	68,5714	0	0	1	0	2
0	0	0	17,1429	25,7143	0	0	0	2
0	1	1	85,7143	205,714	0	1	1	3
0	0	0	30	8,57143	0	0	0	2
0	0	0	11,4286	17,1429	0	0	0	2
0	0	0	62,1429	17,1429	0	1	0	1
0	0	0	22,8571	34,2857	0	0	0	1
0	0	1	205,714	19,2857	19,2857	1	0	2
0	0	0	120	34,2857	0	1	0	2
0	1	1	120	145,714	77,1429	1	1	1
0	0	0	57,1429	51,4286	0	0	0	1
1	0	1	60	120	0	1	1	2
0	0	0	4,28571	4,28571	0	0	0	3
0	0	0	38,5714	22,8571	0	0	0	2
0	1	1	131,429	25,7143	0	1	0	2
0	0	0	37,1429	17,1429	0	0	0	2
0	1	1	140	120	5,71429	1	1	2
0	0	0	27,1429	17,1429	0	0	0	2
0	0	0	75	31,4286	6,42857	1	0	1
0	1	1	205,714	51,4286	0	1	0	1
0	0	0	17,1429	0	0	0	0	2
0	0	0	68,5714	180	0	1	1	2
0	0	0	0	31,4286	0	0	0	2

0	0	0	30	51,4286	0	0	0	2
0	0	0	30	17,1429	0	0	0	2
0	0	0	17,1429	42,8571	0	0	0	2
0	0	0	55,7143	102,857	0	0	1	3
0	1	1	102,857	8,57143	0	1	0	2
0	0	0	35,7143	60	0	0	1	2
0	0	0	28,5714	0	0	0	0	2
0	0	0	11,4286	42,8571	0	0	0	2
0	0	0	0	25,7143	0	0	0	2
0	1	1	70	127,143	0	1	1	2
0	0	0	32,1429	64,2857	0	0	1	1
0	1	1	180	0	0	1	0	1
0	0	1	38,5714	65,7143	51,4286	0	1	2
0	0	0	20	60	85,7143	0	1	2
0	1	1	88,5714	51,4286	0	1	0	2
0	0	0	0	34,2857	0	0	0	2
0	0	1	51,4286	17,1429	0	0	0	1
0	0	0	17,1429	120	0	0	1	2
0	0	0	14,2857	34,2857	0	0	0	2
0	0	0	55,7143	0	0	0	0	2
0	1	1	85,7143	25,7143	0	1	0	3
0	0	0	68,5714	68,5714	0	1	1	2
0	1	1	75	51,4286	0	1	0	2
0	0	0	72,8571	25,7143	0	1	0	2
0	0	0	0	17,1429	17,1429	0	0	1
0	0	0	25,7143	25,7143	25,7143	0	0	1
0	1	1	128,571	17,1429	17,1429	1	0	2
0	1	1	210	180	0	1	1	2
0	0	0	28,5714	180	0	0	1	2
0	0	0	14,2857	25,7143	0	0	0	3
0	0	0	85,7143	145,714	0	1	1	1
0	0	0	60	214,286	102,857	1	1	2
0	0	0	21,4286	92,1429	0	0	1	3
0	0	0	12,1429	100	0	0	1	1
0	1	1	360	17,1429	51,4286	1	1	3
0	1	1	80	51,4286	51,4286	1	1	2
0	0	1	205,714	154,286	0	1	1	3
0	0	0	42,8571	22,8571	25,7143	0	0	3
0	0	0	30	88,5714	0	0	1	2
0	0	0	45,7143	25,7143	0	0	0	1
0	0	0	20	40,7143	0	0	0	1
0	0	0	30	107,143	0	0	1	2
0	0	0	17,1429	15	0	0	0	2
0	0	0	34,2857	60	0	0	1	3
0	0	0	32,1429	98,5714	0	0	1	2
0	0	0	180	257,143	128,571	1	1	2
0	0	0	47,1429	5,71429	0	0	0	2
0	0	0	51,4286	0	0	0	0	3

0	***0***	***0***	***0***	***34,2857***	***0***	***0***	***0***	***1***
0	***0***	***0***	***22,8571***	***25,7143***	***6,42857***	***0***	***0***	***2***
0	***0***	***0***	***90***	***30***	***0***	***1***	***0***	***1***
0	***0***	***0***	***38,5714***	***79,2857***	***0***	***0***	***1***	***1***
0	***0***	***0***	***28,5714***	***21,4286***	***0***	***0***	***0***	***2***
0	***0***	***0***	***22,8571***	***25,7143***	***0***	***0***	***0***	***1***
0	***0***	***0***	***51,4286***	***60***	***0***	***0***	***1***	***2***
0	***0***	***0***	***55,7143***	***87,1429***	***0***	***0***	***1***	***1***
0	***0***	***0***	***0***	***222,857***	***120***	***0***	***1***	***2***
0	***0***	***0***	***120***	***47,1429***	***0***	***1***	***0***	***2***
0	***0***	***0***	***8,57143***	***60***	***0***	***0***	***1***	***1***
0	***0***	***0***	***14,2857***	***60***	***0***	***0***	***1***	***2***
0	***0***	***0***	***11,4286***	***90***	***0***	***0***	***1***	***3***
0	***0***	***0***	***135***	***2,14286***	***0***	***1***	***0***	***2***
0	***0***	***0***	***38,5714***	***34,2857***	***0***	***0***	***0***	***2***
0	***0***	***0***	***36,4286***	***72,8571***	***0***	***0***	***1***	***3***
0	***0***	***0***	***11,4286***	***102,857***	***0***	***0***	***1***	***2***
0	***0***	***0***	***85,7143***	***51,4286***	***0***	***1***	***0***	***3***
0	***1***	***1***	***334,286***	***0***	***11,4286***	***1***	***0***	***1***
0	***0***	***0***	***17,1429***	***34,2857***	***0***	***0***	***0***	***2***
0	***0***	***0***	***17,1429***	***0***	***0***	***0***	***0***	***2***
0	***0***	***0***	***8,57143***	***25,7143***	***0***	***0***	***0***	***2***
0	***0***	***0***	***120***	***77,1429***	***0***	***1***	***1***	***2***
0	***0***	***0***	***28,5714***	***25,7143***	***0***	***0***	***0***	***1***
0	***0***	***0***	***90***	***25,7143***	***0***	***1***	***0***	***1***
0	***1***	***1***	***90***	***34,2857***	***0***	***1***	***0***	***2***
0	***0***	***0***	***25,7143***	***82,8571***	***0***	***0***	***1***	***2***
0	***0***	***0***	***40,7143***	***34,2857***	***8,57143***	***0***	***0***	***2***
0	***0***	***0***	***22,8571***	***8,57143***	***0***	***0***	***0***	***2***
0	***0***	***0***	***30***	***17,1429***	***0***	***0***	***0***	***1***
0	***1***	***1***	***137,143***	***180***	***0***	***1***	***1***	***2***
0	***1***	***1***	***107,143***	***42,8571***	***0***	***1***	***0***	***1***
0	***1***	***1***	***120***	***156,429***	***0***	***1***	***1***	***2***
0	***0***	***0***	***25,7143***	***51,4286***	***0***	***0***	***0***	***2***
0	***0***	***0***	***0***	***90***	***4,28571***	***0***	***1***	***1***
0	***1***	***1***	***140***	***51,4286***	***27,1429***	***1***	***1***	***2***
0	***0***	***0***	***77,1429***	***68,5714***	***0***	***1***	***1***	***2***
0	***0***	***0***	***0***	***60***	***85,7143***	***0***	***1***	***3***
0	***0***	***0***	***102,857***	***171,429***	***0***	***1***	***1***	***2***
0	***0***	***0***	***51,4286***	***22,8571***	***0***	***0***	***0***	***2***
0	***0***	***0***	***8,57143***	***0***	***0***	***0***	***0***	***1***
0	***0***	***0***	***25,7143***	***42,8571***	***0***	***0***	***0***	***2***
0	***0***	***0***	***17,1429***	***0***	***0***	***0***	***0***	***2***
0	***1***	***1***	***60***	***122,857***	***154,286***	***1***	***1***	***3***
0	***0***	***0***	***25***	***180***	***0***	***0***	***1***	***2***
0	***0***	***0***	***20***	***90***	***102,857***	***0***	***1***	***1***
0	***0***	***0***	***0***	***77,1429***	***0***	***0***	***1***	***2***
0	***0***	***0***	***30***	***120***	***0***	***0***	***1***	***2***

0	1	1	111,429	154,286	0	1	1	2
0	0	0	60	14,2857	0	1	0	2
0	1	1	120	47,1429	0	1	0	1
0	0	0	21,4286	60	0	0	1	1
0	0	0	60	8,57143	0	1	0	2
0	0	0	48,5714	42,8571	0	0	0	1
0	1	1	62,8571	30	0	1	0	2
0	0	0	154,286	34,2857	0	1	0	1
0	0	0	48,5714	15	0	0	0	2
0	0	0	77,1429	200	0	1	1	1
0	0	0	5,71429	0	0	0	0	1
0	0	0	4,28571	178,571	0	0	1	2
0	0	0	60	34,2857	0	1	0	2
0	0	0	12,8571	51,4286	0	0	0	1
0	0	0	25,7143	34,2857	12,8571	0	0	2
0	0	0	205,714	154,286	0	1	1	1
0	0	0	77,1429	102,857	0	1	1	1
0	0	0	34,2857	19,2857	0	0	0	2
0	0	0	120	111,429	34,2857	1	1	2
0	0	0	60	111,429	60	1	1	2
0	0	1	60	98,5714	0	1	1	1
0	0	0	7,14286	112,857	0	0	1	1
0	0	0	4,28571	102,857	0	0	1	2
0	0	0	114,286	214,286	0	1	1	2
0	0	0	12,8571	21,4286	0	0	0	3
0	0	0	12,8571	51,4286	0	0	0	1
0	0	0	60	120	0	1	1	2
0	0	0	66,4286	83,5714	0	1	1	2
0	0	1	25,7143	162,857	0	0	1	1
0	0	0	102,857	25,7143	25,7143	1	0	2
0	0	0	0	25,7143	0	0	0	2
0	0	0	134,286	68,5714	0	1	1	2
0	1	1	210	115,714	0	1	1	2
0	0	0	0	124,286	0	0	1	2
0	0	0	120	30	0	1	0	1
0	0	0	51,4286	42,8571	0	0	0	1
0	0	0	102,857	25,7143	0	1	0	2
0	0	0	38,5714	85,7143	0	0	1	2
0	0	0	28,5714	42,8571	0	0	0	2
0	0	0	85,7143	81,4286	0	1	1	2
0	0	0	145,714	128,571	42,8571	1	1	2
0	1	1	180	34,2857	0	1	0	1
0	0	0	60	75	0	1	1	2
0	0	0	120	51,4286	0	1	0	3
0	1	1	210	120	0	1	1	2
0	0	0	17,1429	17,1429	0	0	0	1
0	0	0	60	51,4286	3,57143	1	0	1
0	0	0	30	111,429	0	0	1	2

0	0	0	42,8571	114,286	0	0	1	2
0	0	0	28,5714	25,7143	0	0	0	3
0	0	0	85,7143	85,7143	0	1	1	2
0	1	1	60	94,2857	25,7143	1	1	2
0	0	0	8,57143	52,8571	0	0	0	2
0	0	0	42,8571	111,429	0	0	1	2
0	0	0	47,1429	17,1429	0	0	0	3
0	0	0	17,1429	17,1429	0	0	0	1
0	0	0	51,4286	22,8571	0	0	0	2
0	0	0	42,8571	60	0	0	1	1
0	0	0	28,5714	77,1429	0	0	1	3
0	0	0	60	405	0	1	1	1
0	0	0	40	42,8571	0	0	0	1
0	0	0	21,4286	32,1429	0	0	0	3
0	0	0	128,571	154,286	0	1	1	2
0	0	0	0	68,5714	0	0	1	2
0	0	0	45	45,7143	0	0	0	1
0	0	0	64,2857	17,1429	0	1	0	2
0	0	0	25,7143	154,286	0	0	1	3
0	1	1	240	137,143	0	1	1	1
0	0	0	81,4286	34,2857	0	1	0	2
0	1	1	171,429	111,429	0	1	1	2
0	1	1	180	120	0	1	1	1
0	0	0	34,2857	8,57143	0	0	0	3
0	0	0	24,2857	34,2857	0	0	0	2
0	0	0	30	0	0	0	0	2
0	0	0	150	141,429	11,4286	1	1	1
0	0	0	120	171,429	0	1	1	2
0	0	0	32,1429	25,7143	0	0	0	3
0	0	0	120	132,857	0	1	1	2
0	1	1	60	60	25,7143	1	1	2
0	0	0	70	14,2857	0	1	0	1
0	0	0	0	21,4286	0	0	0	1
0	0	0	15	12,8571	0	0	0	2
0	0	0	30	85,7143	48,5714	0	1	1
0	0	0	8,57143	72,8571	0	0	1	2
0	0	0	0	122,857	12,8571	0	1	3
0	0	0	40	111,429	0	0	1	2
0	0	0	21,4286	175,714	0	0	1	2
0	0	0	180	240	0	1	1	2
0	0	0	26,4286	34,2857	0	0	0	2
0	0	0	12,8571	42,8571	0	0	0	2
0	0	0	31,4286	34,2857	77,1429	0	1	2
0	0	0	5,71429	34,2857	0	0	0	2
0	0	0	60	25,7143	0	1	0	3
0	0	0	25,7143	42,8571	0	0	0	2
0	0	0	17,1429	128,571	4,28571	0	1	1
0	0	0	6,42857	77,1429	4,28571	0	1	3

0	0	0	19,2857	51,4286	0	0	0	2
0	0	0	162,857	0	0	1	0	2
0	0	0	17,1429	72,8571	0	0	1	1
0	0	0	90	17,1429	0	1	0	2
0	0	0	8,57143	51,4286	0	0	0	1
0	0	0	2,85714	0	0	0	0	2
0	0	0	62,8571	21,4286	0	1	0	2
0	1	1	208,571	34,2857	0	1	0	2
0	0	0	0	25,7143	0	0	0	2
0	0	0	0	34,2857	0	0	0	2
0	0	0	25	20	10	0	0	1
0	0	0	14,2857	22,8571	4,28571	0	0	1
0	0	0	38,5714	25,7143	0	0	0	2
0	0	0	34,2857	37,1429	0	0	0	1
0	0	0	42,8571	51,4286	0	0	0	2
0	0	0	40,7143	32,1429	0	0	0	2
0	0	0	37,1429	12,8571	0	0	0	2
0	0	0	120	102,857	0	1	1	1
0	0	0	34,2857	171,429	17,1429	0	1	2
0	0	0	10,7143	79,2857	0	0	1	2
0	0	0	40,7143	85,7143	0	0	1	2
0	0	1	37,1429	21,4286	51,4286	0	1	2
0	0	0	22,8571	114,286	0	0	1	2
0	0	0	77,1429	70,7143	0	1	1	2
0	0	0	60	23,5714	0	1	0	2
0	0	0	25,7143	192,857	0	0	1	2
0	1	1	162,857	40,7143	0	1	0	1
0	0	0	45	0	128,571	0	1	1
0	0	0	30	5,71429	0	0	0	2
0	0	0	12,8571	42,8571	0	0	0	1
0	0	0	38,5714	51,4286	0	0	0	2
0	0	0	0	42,8571	0	0	0	2
0	0	0	51,4286	68,5714	0	0	1	1
0	0	0	0	17,1429	0	0	0	2
0	0	0	57,8571	77,1429	0	0	1	1
0	1	1	120	102,857	0	1	1	2
0	0	0	77,1429	57,1429	0	1	0	1
0	0	0	2,85714	122,143	0	0	1	2
0	0	0	17,1429	60	0	0	1	2
0	0	0	0	34,2857	0	0	0	2
0	1	1	80	60	0	1	1	2
0	1	1	120	102,857	0	1	1	1
0	0	0	14,2857	94,2857	0	0	1	2
0	0	0	37,1429	34,2857	0	0	0	2
0	0	0	12,8571	0	8,57143	0	0	2
0	0	0	0	145,714	180	0	1	2
0	0	0	42,8571	137,143	180	0	1	3
0	0	0	120	154,286	0	1	1	1

0	1	1	100	128,571	0	1	1	2
0	0	0	30	21,4286	0	0	0	2
0	0	0	24,2857	8,57143	0	0	0	1
0	0	0	8,57143	205,714	0	0	1	2
0	0	0	51,4286	77,1429	0	0	1	2
0	0	0	48,5714	34,2857	0	0	0	2
0	0	0	30	8,57143	0	0	0	1
0	0	0	51,4286	85,7143	0	0	1	2
0	0	0	42,8571	67,1429	0	0	1	2
0	1	1	80	30	0	1	0	2
0	0	0	32,8571	17,1429	0	0	0	2
0	0	0	38,5714	188,571	0	0	1	2
0	0	0	21,4286	51,4286	0	0	0	2
0	0	0	42,8571	51,4286	0	0	0	2
0	0	0	150	42,8571	0	1	0	2
0	0	0	22,8571	17,1429	17,1429	0	0	1
0	0	0	74,2857	42,8571	0	1	0	2
0	0	0	51,4286	107,143	4,28571	0	1	1
0	1	1	180	34,2857	0	1	0	2
0	1	1	180	137,143	0	1	1	2
0	0	0	14,2857	42,8571	0	0	0	2
0	0	0	60	17,1429	0	1	0	2
0	0	0	30	25,7143	0	0	0	2
0	0	1	57,1429	190,714	0	0	1	2
0	0	0	14,2857	54,2857	128,571	0	1	2
0	0	0	10	102,857	0	0	1	2
0	0	0	17,1429	17,1429	2,85714	0	0	2
0	0	0	7,14286	51,4286	0	0	0	2
0	0	0	128,571	128,571	0	1	1	2
0	0	0	31,4286	30	0	0	0	2
0	0	0	68,5714	51,4286	8,57143	1	1	1
0	0	0	48,5714	25,7143	0	0	0	1
0	0	0	25,7143	17,1429	0	0	0	3
0	1	1	180	214,286	0	1	1	2
0	0	0	11,4286	12,8571	0	0	0	2
0	0	0	34,2857	45	0	0	0	1
0	0	0	51,4286	105	0	0	1	2
0	0	0	50	25,7143	0	0	0	1
0	0	0	180	128,571	17,1429	1	1	2
0	0	0	25,7143	60	0	0	1	2
0	0	0	60	28,5714	0	1	0	2
0	0	0	0	180	0	0	1	1
0	0	0	90	180	60	1	1	2
0	0	0	11,4286	157,143	0	0	1	2
0	0	0	60	25,7143	0	1	0	2
0	0	0	0	111,429	25,7143	0	1	1
0	0	0	38,5714	35,7143	32,1429	0	1	2
0	0	0	47,1429	21,4286	0	0	0	2

0	0	0	0	25,7143	0	0	0	1
0	0	0	0	68,5714	0	0	1	1
0	0	1	30	72,8571	0	0	1	2
0	0	0	21,4286	34,2857	0	0	0	1
0	0	0	47,1429	40,7143	12,8571	0	0	1
0	0	0	25,7143	25,7143	0	0	0	3
1	0	1	14,2857	240	0	0	1	2
0	1	1	182,857	0	0	1	0	1
0	1	1	137,143	60	25,7143	1	1	1
1	0	1	38,5714	141,429	0	0	1	2
0	0	0	25,7143	111,429	0	0	1	2
0	0	0	55,7143	68,5714	0	0	1	2
0	0	0	30	111,429	0	0	1	1
0	0	0	52,8571	36,4286	0	0	0	1
0	0	0	60	308,571	128,571	1	1	2
0	0	0	77,1429	0	0	1	0	2
0	0	0	5,71429	64,2857	0	0	1	1
0	0	0	25,7143	25,7143	0	0	0	2
0	0	0	21,4286	27,1429	0	0	0	1
0	0	0	20	35,7143	0	0	0	1
0	0	0	162,857	60	0	1	1	1
0	0	0	120	60	0	1	1	2
0	0	1	72,8571	94,2857	0	1	1	2
0	0	0	47,1429	38,5714	0	0	0	2
0	0	1	101,429	235,714	0	1	1	1
0	0	0	14,2857	77,1429	0	0	1	2
0	0	0	60	77,1429	0	1	1	1
0	0	0	0	34,2857	0	0	0	1
0	0	0	57,1429	19,2857	0	0	0	2
0	0	0	34,2857	0	0	0	0	1
0	0	0	0	128,571	0	0	1	2
0	0	0	8,57143	25,7143	0	0	0	1
0	0	0	0	51,4286	25,7143	0	1	2
0	1	1	120	45	0	1	0	3
0	0	0	20	34,2857	0	0	0	2
0	0	0	35,7143	62,1429	0	0	1	2
0	0	0	51,4286	102,857	0	0	1	3
0	0	0	6,42857	94,2857	180	0	1	1
0	1	1	197,143	34,2857	0	1	0	2
0	0	0	0	22,8571	0	0	0	3
0	0	0	42,8571	8,57143	0	0	0	2
0	0	0	34,2857	120	51,4286	0	1	2
0	0	0	82,8571	66,4286	0	1	1	1
0	0	0	0	128,571	0	0	1	2
0	0	0	18,5714	182,143	0	0	1	1
0	1	1	360	51,4286	0	1	0	1
0	1	1	300	17,1429	0	1	0	2
0	0	0	17,1429	8,57143	0	0	0	2

0	0	0	8,57143	77,1429	94,2857	0	1	2
0	0	0	21,4286	23,5714	0	0	0	2
0	0	0	42,8571	102,857	0	0	1	2
0	0	0	61,4286	24,2857	0	1	0	1
0	0	0	25,7143	47,1429	51,4286	0	1	1
0	0	0	60	68,5714	0	1	1	1
0	0	0	85,7143	180	0	1	1	2
0	1	1	60	60	0	1	1	2
0	0	0	25,7143	30	0	0	0	2
0	1	1	60	51,4286	0	1	0	2
0	1	1	137,143	171,429	0	1	1	2
0	0	0	12,8571	4,28571	0	0	0	2
0	0	0	0	25,7143	0	0	0	2
0	0	0	0	25,7143	0	0	0	2
0	0	0	45,7143	180	0	0	1	2
0	1	1	180	42,8571	0	1	0	1
0	0	0	38,5714	30	0	0	0	3
0	0	0	1,42857	54,2857	0	0	0	1
0	0	0	51,4286	49,2857	12,8571	0	1	2
0	0	0	42,8571	120	0	0	1	2
0	0	0	51,4286	94,2857	8,57143	0	1	2
0	0	0	4,28571	72,8571	0	0	1	1
0	0	0	11,4286	31,4286	0	0	0	2
0	0	1	94,2857	128,571	8,57143	1	1	2
0	1	1	257,143	17,1429	6,42857	1	0	2
0	0	0	60	252,857	0	1	1	3
0	0	1	38,5714	171,429	34,2857	0	1	2
0	0	0	94,2857	42,8571	0	1	0	1
0	0	1	45,7143	47,1429	0	0	0	1
0	0	0	85,7143	171,429	128,571	1	1	2
0	0	0	49,2857	34,2857	17,1429	0	0	2
0	0	0	68,5714	351,429	0	1	1	2
0	0	0	60	42,8571	0	1	0	1
0	0	0	12,8571	34,2857	0	0	0	2
0	0	0	23,5714	34,2857	0	0	0	2
0	0	0	128,571	137,143	0	1	1	2
0	1	1	65,7143	74,2857	0	1	1	2
0	0	0	46,4286	42,8571	0	0	0	2
0	0	0	5,71429	8,57143	0	0	0	1
0	0	0	34,2857	51,4286	0	0	0	2
0	0	0	25,7143	8,57143	0	0	0	2
0	1	1	128,571	102,857	8,57143	1	1	3
0	1	1	205,714	94,2857	0	1	1	2
0	0	0	17,1429	42,8571	0	0	0	3
0	0	0	30	25,7143	0	0	0	2
0	0	0	0	8,57143	11,4286	0	0	2
1	0	1	162,857	128,571	17,1429	1	1	2
0	0	0	4,28571	30	0	0	0	2

0	0	0	28,5714	19,2857	0	0	0	1
0	0	0	25,7143	34,2857	8,57143	0	0	1
0	0	0	4,28571	17,1429	0	0	0	2
0	1	1	145,714	51,4286	0	1	0	2
0	1	1	360	68,5714	0	1	1	2
0	0	0	17,1429	4,28571	4,28571	0	0	2
0	0	0	8,57143	111,429	102,857	0	1	1
0	1	1	145,714	52,8571	0	1	0	2
1	1	1	180	368,571	0	1	1	2
0	0	0	6,42857	60	0	0	1	2
0	0	0	12,8571	188,571	0	0	1	1
0	0	0	4,28571	5,71429	0	0	0	2
0	0	0	51,4286	51,4286	0	0	0	1
0	0	0	20	60	34,2857	0	1	2
0	0	0	17,1429	68,5714	0	0	1	3
0	1	1	77,1429	47,1429	0	1	0	2
0	0	0	30	8,57143	0	0	0	2
0	0	0	77,1429	17,1429	0	1	0	2
0	0	0	11,4286	51,4286	0	0	0	2
0	1	1	231,429	85,7143	0	1	1	1
0	0	0	2,85714	17,1429	0	0	0	2
0	0	0	42,8571	120	0	0	1	2
0	0	0	34,2857	34,2857	0	0	0	2
0	0	0	12,8571	68,5714	0	0	1	2
0	1	1	274,286	154,286	0	1	1	2
0	0	0	77,1429	40	0	1	0	1
0	0	0	0	145,714	7,14286	0	1	2
0	1	1	98,5714	38,5714	0	1	0	2
0	0	0	102,857	38,5714	0	1	0	1
0	0	0	0	240	34,2857	0	1	2
0	0	0	21,4286	42,8571	0	0	0	3
0	0	0	25,7143	141,429	0	0	1	2
0	0	0	38,5714	130,714	0	0	1	2
0	0	0	77,1429	34,2857	0	1	0	1
0	0	0	7,14286	68,5714	0	0	1	2
0	0	0	90	71,4286	0	1	1	3
0	0	0	42,8571	17,1429	6,42857	0	0	1
0	0	0	68,5714	12,8571	25,7143	1	0	3
0	0	0	17,1429	77,1429	0	0	1	2
0	0	0	47,1429	120	0	0	1	1
0	0	0	0	0	0	0	0	2
0	0	0	55	77,1429	6,42857	0	1	1
0	1	1	70	25,7143	0	1	0	1
0	0	0	15	64,2857	0	0	1	2
0	0	0	28,5714	34,2857	0	0	0	3
0	0	0	8,57143	90	0	0	1	2
0	0	0	64,2857	87,1429	17,1429	1	1	2
0	0	0	60	34,2857	0	1	0	2

0	0	0	17,8571	21,4286	0	0	0	1
0	0	0	32,8571	120	0	0	1	2
0	0	0	90	51,4286	0	1	0	2
0	1	1	100	42,8571	0	1	0	1
0	0	0	5,71429	54,2857	0	0	0	3
0	0	0	30	77,1429	0	0	1	2
0	0	0	30	34,2857	0	0	0	2
0	0	0	12,8571	137,143	68,5714	0	1	3
0	0	0	0	287,143	51,4286	0	1	2
0	0	0	27,1429	34,2857	0	0	0	2
0	0	0	66,4286	34,2857	0	1	0	2
0	0	0	21,4286	42,8571	0	0	0	1
0	0	0	17,1429	85,7143	0	0	1	2
0	0	0	10	96,4286	0	0	1	3
0	0	0	23,5714	25,7143	0	0	0	2
0	0	1	25,7143	8,57143	40	0	0	1
0	0	0	115,714	48,5714	0	1	0	2
0	0	0	120	25,7143	0	1	0	1
0	0	0	67,1429	60	0	1	1	2
0	1	1	142,857	34,2857	0	1	0	2
0	0	0	68,5714	0	0	1	0	1
0	0	0	17,1429	12,8571	0	0	0	2
0	0	0	30	97,1429	0	0	1	3
0	0	0	8,57143	0	0	0	0	1
0	1	1	140	45	0	1	0	2
0	0	0	50	28,5714	0	0	0	2
0	0	0	2,14286	197,143	0	0	1	3
0	0	0	21,4286	42,8571	0	0	0	2
0	0	0	30	8,57143	0	0	0	2
0	1	1	60	120	0	1	1	2
0	0	0	25,7143	47,1429	0	0	0	2
0	0	0	42,8571	17,1429	0	0	0	1
0	0	0	30	102,857	0	0	1	2
0	0	0	64,2857	34,2857	0	1	0	1
0	0	0	38,5714	4,28571	0	0	0	2
0	1	1	60	120	0	1	1	2
0	0	0	0	49,2857	21,4286	0	1	2
0	1	1	120	8,57143	8,57143	1	0	2
0	0	0	14,2857	12,8571	0	0	0	2
0	1	1	125,714	137,143	0	1	1	3
0	0	0	15	17,1429	0	0	0	2
0	0	0	62,8571	154,286	0	1	1	2
0	1	1	68,5714	17,1429	0	1	0	2
0	1	1	270	75	0	1	1	1
0	0	0	21,4286	0	0	0	0	1
0	0	0	17,1429	42,8571	25,7143	0	1	2
0	0	0	8,57143	17,1429	17,1429	0	0	2
0	0	0	25,7143	40	0	0	0	1

0	0	0	5,71429	8,57143	0	0	0	2
0	0	0	15,7143	51,4286	0	0	0	2
0	0	0	0	0	0	0	0	2
0	0	0	68,5714	34,2857	0	1	0	2
0	0	0	62,8571	158,571	0	1	1	2
0	1	1	180	17,1429	0	1	0	2
0	0	0	30	25,7143	0	0	0	1
0	1	1	85,7143	78,5714	6,42857	1	1	2
0	0	0	20	42,8571	0	0	0	2
0	0	0	42,8571	66,4286	0	0	1	2
0	0	0	19,2857	51,4286	0	0	0	2
0	0	0	17,1429	45,7143	0	0	0	2
0	0	0	0	167,143	0	0	1	1
0	0	0	14,2857	51,4286	0	0	0	2
0	0	0	47,1429	66,4286	0	0	1	2
0	0	0	7,14286	51,4286	0	0	0	2
0	0	0	68,5714	21,4286	0	1	0	2
0	0	0	12,8571	120	0	0	1	1
0	1	1	128,571	40	51,4286	1	1	1
0	0	0	102,857	141,429	0	1	1	1
0	0	0	34,2857	85,7143	0	0	1	1
0	0	0	154,286	205,714	154,286	1	1	2
0	0	0	35,7143	131,429	0	0	1	1
0	0	0	10,7143	51,4286	0	0	0	2
0	0	0	102,857	17,1429	0	1	0	2
0	0	0	0	94,2857	0	0	1	2
0	0	0	38,5714	70,7143	0	0	1	2
0	0	0	17,1429	64,2857	0	0	1	1
0	0	0	0	85,7143	0	0	1	1
0	0	0	0	60	0	0	1	2
0	0	0	0	85,7143	0	0	1	2
0	0	0	77,1429	77,1429	0	1	1	2
0	0	0	0	94,2857	0	0	1	1
0	0	0	0	257,143	0	0	1	1
0	0	0	5,71429	55,7143	0	0	0	2
0	0	0	25,7143	111,429	0	0	1	1
0	0	0	34,2857	81,4286	0	0	1	3
0	0	0	141,429	8,57143	0	1	0	2
0	0	0	96,4286	45	0	1	0	3
0	0	0	51,4286	34,2857	0	0	0	2
0	1	1	162,857	47,1429	8,57143	1	0	3
0	0	0	31,4286	102,857	0	0	1	2
0	1	1	80	60	34,2857	1	1	2
0	0	0	11,4286	25,7143	0	0	0	1
0	0	0	24,2857	63,5714	0	0	1	3
0	0	0	60	25,7143	0	1	0	1
0	0	0	8,57143	87,8571	0	0	1	3
0	0	0	34,2857	23,5714	0	0	0	2

0	0	0	25,7143	34,2857	0	0	0	2
0	0	0	34,2857	162,857	0	0	1	2
0	0	0	21,4286	21,4286	0	0	0	1
0	0	0	17,1429	240	0	0	1	2
0	0	0	107,143	38,5714	0	1	0	2
0	1	1	194,286	42,8571	25,7143	1	1	2
0	0	0	12,8571	8,57143	0	0	0	2
0	0	0	12,8571	34,2857	0	0	0	2
0	0	0	12,8571	162,857	0	0	1	2
0	0	0	44,2857	120	0	0	1	2
0	0	0	14,2857	42,8571	0	0	0	2
0	0	0	58,5714	17,1429	0	0	0	2
0	0	0	145,714	77,1429	0	1	1	1
0	0	0	84,2857	102,857	0	1	1	2
0	0	0	171,429	17,1429	0	1	0	2
0	1	1	154,286	17,1429	0	1	0	2
0	0	0	42,8571	154,286	34,2857	0	1	2
0	0	0	34,2857	8,57143	0	0	0	1
0	0	0	21,4286	68,5714	0	0	1	2
0	1	1	105	60	0	1	1	3
0	0	0	47,1429	81,4286	34,2857	0	1	3
0	0	0	45,7143	34,2857	0	0	0	1
0	0	0	14,2857	34,2857	0	0	0	2
0	1	1	300	51,4286	12,8571	1	1	2
0	0	0	60	34,2857	0	1	0	1
0	0	0	68,5714	51,4286	0	1	0	2
0	0	0	60	51,4286	0	1	0	2
0	0	0	42,8571	60	0	0	1	3
0	0	0	8,57143	17,1429	14,2857	0	0	2
0	0	0	8,57143	102,857	0	0	1	1
0	0	1	57,1429	102,857	32,1429	0	1	1
0	1	1	180	197,143	0	1	1	2
0	1	1	180	137,143	0	1	1	1
0	0	0	37,1429	74,2857	0	0	1	1
0	1	1	85,7143	276,429	8,57143	1	1	1
0	0	0	20	42,8571	0	0	0	2
0	1	1	145,714	51,4286	0	1	0	1
0	0	0	94,2857	81,4286	0	1	1	2
0	0	0	22,8571	111,429	0	0	1	2
0	0	0	101,429	17,1429	0	1	0	2
0	1	1	145,714	77,1429	0	1	1	2
0	0	0	34,2857	94,2857	0	0	1	2
0	0	0	8,57143	154,286	0	0	1	2
0	0	0	25,7143	0	0	0	0	2
0	1	1	154,286	30,7143	0	1	0	2
0	0	0	42,8571	120	0	0	1	2
0	0	0	8,57143	15,7143	0	0	0	1
0	1	1	111,429	102,857	51,4286	1	1	1

0	1	1	120	5,71429	0	1	0	2
0	0	0	34,2857	34,2857	0	0	0	3
0	0	1	85,7143	55,7143	0	1	0	1
0	0	0	74,2857	70	51,4286	1	1	2
0	0	0	11,4286	21,4286	0	0	0	2
0	0	0	5,71429	17,1429	0	0	0	2
0	0	0	30	137,143	0	0	1	2
0	0	0	7,14286	25,7143	0	0	0	2
0	0	0	22,1429	62,1429	0	0	1	2
0	0	0	0	235,714	68,5714	0	1	2
0	0	0	85,7143	85,7143	0	1	1	2
0	0	0	20	20	17,1429	0	0	1
0	0	0	57,1429	20	0	0	0	2
0	0	0	218,571	77,1429	0	1	1	2
0	0	0	50	51,4286	0	0	0	2
0	0	0	54,2857	35,7143	0	0	0	3
0	1	1	90	25,7143	0	1	0	2
0	0	0	47,1429	334,286	0	0	1	1
0	0	0	62,1429	75	0	1	1	2
0	0	0	30	34,2857	0	0	0	1
0	0	0	111,429	25,7143	0	1	0	2
0	1	1	150	60	0	1	1	2
0	0	0	141,429	57,8571	12,8571	1	1	2
0	0	0	14,2857	199,286	0	0	1	1
0	0	0	60	42,8571	0	1	0	2
0	0	0	12,8571	0	0	0	0	2
0	0	0	21,4286	34,2857	0	0	0	2
0	0	0	14,2857	21,4286	51,4286	0	1	2
0	0	0	91,4286	51,4286	0	1	0	2
0	0	0	47,1429	68,5714	128,571	0	1	2
0	0	0	12,1429	17,1429	0	0	0	1
0	1	1	102,857	64,2857	0	1	1	3
0	0	0	42,8571	17,1429	0	0	0	1
0	0	0	60	51,4286	0	1	0	2
0	0	0	38,5714	85,7143	0	0	1	3
0	0	0	2,85714	315	0	0	1	1
0	0	0	81,4286	17,1429	0	1	0	2
0	0	0	75	0	0	1	0	2
0	0	0	94,2857	171,429	0	1	1	3
0	0	0	15	88,5714	0	0	1	2
0	0	0	0	23,5714	0	0	0	1
0	1	1	132,857	34,2857	0	1	0	2
0	0	0	25,7143	0	0	0	0	1
0	0	0	38,5714	7,14286	0	0	0	1
0	0	0	17,1429	28,5714	0	0	0	2
0	0	0	94,2857	94,2857	0	1	1	2
0	0	0	0	100,714	25,7143	0	1	2
0	0	0	45,7143	55,7143	0	0	0	2

0	0	0	85,7143	34,2857	0	1	0	2
0	0	0	38,5714	8,57143	0	0	0	2
0	0	0	51,4286	25,7143	0	0	0	1
0	0	0	30	85,7143	0	0	1	2
0	1	1	162,857	51,4286	0	1	0	2
0	0	0	12,8571	57,8571	0	0	0	2
0	0	0	85,7143	75	0	1	1	2
0	1	1	111,429	17,1429	0	1	0	2
0	0	0	8,57143	60	0	0	1	1
0	0	0	12,8571	75	0	0	1	2
0	1	1	120	60	0	1	1	2
0	0	0	0	162,857	0	0	1	2
0	0	0	8,57143	111,429	0	0	1	2
0	0	0	42,8571	25,7143	0	0	0	3
0	0	0	42,8571	12,8571	0	0	0	1
0	0	0	20	34,2857	19,2857	0	0	2
0	0	0	34,2857	17,1429	0	0	0	2
0	0	0	154,286	34,2857	0	1	0	3
0	0	0	64,2857	0	0	1	0	2
0	0	0	2,85714	85,7143	0	0	1	2
0	0	0	0	5,71429	2,14286	0	0	2
0	0	0	14,2857	34,2857	0	0	0	2
0	0	0	38,5714	182,857	0	0	1	2
0	0	0	0	42,8571	154,286	0	1	2
0	0	0	17,1429	17,1429	0	0	0	2
0	0	0	60	60	0	1	1	2
0	0	0	12,8571	62,8571	0	0	1	2
0	0	0	28,5714	25,7143	0	0	0	2
0	1	1	60	34,2857	0	1	0	1
0	0	0	42,8571	42,8571	0	0	0	2
0	1	1	240	12,8571	85,7143	1	1	1
0	0	0	14,2857	52,8571	0	0	0	1
0	0	0	137,143	55,7143	0	1	0	2
0	0	0	21,4286	0	0	0	0	2
0	1	1	150	68,5714	0	1	1	1
0	1	1	90	47,1429	0	1	0	2
0	0	0	25,7143	60	0	0	1	2
0	0	0	8,57143	31,7143	0	0	0	2
0	1	1	120	246,429	120	1	1	3
0	0	0	0	120	180	0	1	2
0	1	1	171,429	60	0	1	1	2
0	0	0	30	137,143	77,1429	0	1	2
0	1	1	65,7143	90	0	1	1	2
0	0	0	2,85714	85,7143	85,7143	0	1	1
0	1	1	85,7143	57,1429	11,4286	1	1	1
0	0	0	21,4286	51,4286	0	0	0	2
0	1	1	180	85,7143	12,8571	1	1	1
0	0	0	38,5714	25,7143	0	0	0	2

0	*1*	*1*	*188,571*	*191,429*	*85,7143*	*1*	*1*	*2*
0	*0*	*0*	*32,8571*	*180*	*0*	*0*	*1*	*1*
1	*0*	*1*	*175,714*	*14,2857*	*128,571*	*1*	*1*	*3*
0	*0*	*0*	*12,8571*	*300*	*0*	*0*	*1*	*2*
0	*0*	*0*	*12,8571*	*8,57143*	*0*	*0*	*0*	*2*
0	*0*	*0*	*27,1429*	*42,8571*	*0*	*0*	*0*	*2*
0	*0*	*0*	*38,5714*	*25,7143*	*0*	*0*	*0*	*2*
0	*0*	*0*	*17,1429*	*120*	*0*	*0*	*1*	*2*
0	*0*	*0*	*17,1429*	*42,8571*	*0*	*0*	*0*	*2*
0	*0*	*0*	*120*	*72,8571*	*0*	*1*	*1*	*2*
0	*0*	*0*	*60*	*94,2857*	*0*	*1*	*1*	*2*
0	*0*	*0*	*30*	*42,8571*	*0*	*0*	*0*	*2*
0	*1*	*1*	*75*	*128,571*	*25,7143*	*1*	*1*	*2*
0	*0*	*0*	*128,571*	*34,2857*	*8,57143*	*1*	*0*	*2*
0	*0*	*0*	*30*	*17,1429*	*0*	*0*	*0*	*1*
0	*0*	*0*	*37,1429*	*30*	*0*	*0*	*0*	*2*
0	*0*	*0*	*30*	*132,857*	*0*	*0*	*1*	*2*
0	*0*	*0*	*10,7143*	*231,429*	*0*	*0*	*1*	*1*
0	*0*	*0*	*14,2857*	*60*	*0*	*0*	*1*	*3*
0	*0*	*0*	*55,7143*	*41,4286*	*25,7143*	*0*	*1*	*1*
0	*0*	*0*	*70*	*25,7143*	*0*	*1*	*0*	*2*
0	*0*	*0*	*53,5714*	*55,7143*	*0*	*0*	*0*	*2*
0	*0*	*0*	*0*	*377,143*	*180*	*0*	*1*	*1*
0	*1*	*1*	*67,1429*	*42,8571*	*0*	*1*	*0*	*2*
0	*0*	*0*	*25,7143*	*205,714*	*0*	*0*	*1*	*3*
0	*0*	*0*	*28,5714*	*0*	*0*	*0*	*0*	*2*
0	*0*	*0*	*68,5714*	*85,7143*	*120*	*1*	*1*	*1*
0	*0*	*0*	*25*	*0*	*0*	*0*	*0*	*2*
0	*0*	*0*	*34,2857*	*40*	*0*	*0*	*0*	*2*
0	*0*	*0*	*102,857*	*68,5714*	*0*	*1*	*1*	*1*
0	*0*	*0*	*42,8571*	*8,57143*	*0*	*0*	*0*	*3*
0	*0*	*0*	*60*	*0*	*0*	*1*	*0*	*2*
0	*0*	*0*	*11,4286*	*6,42857*	*0*	*0*	*0*	*1*
0	*0*	*0*	*15*	*8,57143*	*0*	*0*	*0*	*2*
0	*0*	*0*	*8,57143*	*42,8571*	*0*	*0*	*0*	*2*
0	*0*	*0*	*137,143*	*188,571*	*180*	*1*	*1*	*2*
0	*1*	*1*	*128,571*	*0*	*0*	*1*	*0*	*2*
0	*0*	*0*	*20*	*17,1429*	*25,7143*	*0*	*0*	*1*
0	*0*	*0*	*32,1429*	*47,1429*	*0*	*0*	*0*	*3*
0	*0*	*0*	*94,2857*	*137,143*	*0*	*1*	*1*	*2*
0	*0*	*0*	*51,4286*	*154,286*	*0*	*0*	*1*	*2*
0	*0*	*0*	*34,2857*	*145,714*	*0*	*0*	*1*	*2*
0	*1*	*1*	*77,1429*	*25,7143*	*0*	*1*	*0*	*2*
0	*0*	*0*	*12,8571*	*51,4286*	*0*	*0*	*0*	*2*
0	*0*	*0*	*34,2857*	*30*	*77,1429*	*0*	*1*	*2*
0	*0*	*0*	*54,2857*	*25,7143*	*0*	*0*	*0*	*2*
0	*0*	*0*	*65,7143*	*17,1429*	*0*	*1*	*0*	*3*
0	*0*	*0*	*17,1429*	*17,1429*	*0*	*0*	*0*	*2*

0	0	0	25,7143	60	38,5714	0	1	2
0	1	1	131,429	77,1429	0	1	1	2
0	1	1	120	25,7143	0	1	0	2
0	1	1	145,714	102,857	0	1	1	1
0	1	1	240	120	0	1	1	1
0	1	1	77,1429	85,7143	0	1	1	2
0	1	1	162,857	34,2857	0	1	0	2
0	0	0	0	257,143	102,857	0	1	1
0	0	0	30	25,7143	0	0	0	2
0	0	0	8,57143	25,7143	0	0	0	2
0	0	0	28,5714	108,571	180	0	1	2
0	0	0	51,4286	102,857	0	0	1	2
0	0	0	14,2857	145,714	85,7143	0	1	1
1	0	1	0	270	0	0	1	1
0	0	0	11,4286	75	0	0	1	2
0	0	0	48,5714	94,2857	0	0	1	1
0	1	1	60	93,5714	0	1	1	3
0	0	0	22,8571	40,7143	0	0	0	1
0	0	0	80	64,2857	0	1	1	2
0	0	0	25,7143	25,7143	0	0	0	2
0	0	0	20	0	0	0	0	1
0	0	0	68,5714	77,1429	0	1	1	2
0	0	0	47,1429	65,7143	0	0	1	2
0	0	0	72,8571	55,7143	0	1	0	2
0	0	0	0	25,7143	0	0	0	2
0	1	1	111,429	154,286	17,1429	1	1	2
0	1	1	240	145,714	51,4286	1	1	2
0	0	0	55,7143	0	0	0	0	3
0	0	1	111,429	60	0	1	1	2
0	0	0	0	34,2857	0	0	0	2
0	1	1	105	17,1429	0	1	0	2
0	0	0	68,5714	131,429	8,57143	1	1	1
0	0	0	0	65,7143	0	0	1	1
0	0	0	64,2857	60	0	1	1	3
0	0	0	25,7143	60	0	0	1	2
0	0	0	2,85714	34,2857	0	0	0	2
0	0	0	68,5714	102,857	0	1	1	2
0	0	0	60	83,5714	0	1	1	2
0	0	0	88,5714	55	0	1	0	1
0	0	0	40	45,7143	0	0	0	2
0	0	0	45	27,8571	25,7143	0	0	1
0	0	0	23,5714	27,8571	0	0	0	2
0	0	0	85,7143	120	0	1	1	1
0	0	0	82,1429	17,1429	0	1	0	1
0	0	0	58,5714	21,4286	0	0	0	2
0	0	0	51,4286	60	0	0	1	2
0	0	0	42,8571	8,57143	0	0	0	1
0	0	0	60	94,2857	0	1	1	1

0	0	0	54,2857	111,429	0	0	1	2
0	0	0	50	0	28,5714	0	0	1
0	0	0	51,4286	34,2857	0	0	0	2
0	0	0	38,5714	60	34,2857	0	1	3
0	0	0	157,143	34,2857	0	1	0	2
0	0	0	5,71429	25,7143	0	0	0	2
0	0	0	10	77,1429	51,4286	0	1	1
0	0	0	40	51,4286	0	0	0	2
0	0	0	81,4286	42,8571	0	1	0	2
0	0	0	0	260	0	0	1	1
0	0	0	28,5714	120	0	0	1	1
0	0	0	28,5714	85,7143	0	0	1	1
0	0	0	0	188,571	0	0	1	2
0	0	0	42,8571	77,1429	0	0	1	1
1	0	1	12,8571	111,429	0	0	1	1
0	1	1	154,286	102,857	0	1	1	2
0	0	0	120	180	0	1	1	1
0	0	0	17,1429	68,5714	0	0	1	1
0	0	0	25,7143	38,5714	0	0	0	2
0	0	0	37,1429	205,714	0	0	1	1
0	0	0	20	34,2857	6,42857	0	0	1
0	1	1	107,143	60	60	1	1	3
0	0	0	51,4286	34,2857	0	0	0	2
0	1	1	137,143	94,2857	0	1	1	2
0	0	0	35,7143	12,8571	0	0	0	2
0	1	1	107,143	34,2857	0	1	0	1
0	0	0	51,4286	50	0	0	0	3
0	0	0	25,7143	25,7143	0	0	0	2
0	0	0	0	325,714	0	0	1	2
0	0	0	11,4286	25,7143	0	0	0	2
0	0	0	0	20	0	0	0	3
0	0	0	47,1429	60	0	0	1	2
0	0	0	10,7143	0	25,7143	0	0	2
0	0	0	128,571	30	0	1	0	1
0	1	1	231,429	102,857	0	1	1	2
0	0	0	10	81,4286	0	0	1	3
0	0	0	42,8571	25,7143	0	0	0	2
0	0	0	28,5714	102,857	0	0	1	2
0	0	0	68,5714	42,8571	0	1	0	2
0	0	0	162,857	368,571	0	1	1	2
0	0	0	0	51,4286	0	0	0	1
0	1	1	128,571	25,7143	0	1	0	2
0	1	1	205,714	180	0	1	1	2
0	1	1	214,286	115,714	0	1	1	2
0	0	0	105,714	30	0	1	0	2
0	0	0	60	120	0	1	1	1
0	0	0	68,5714	51,4286	0	1	0	1
0	0	0	8,57143	64,2857	0	0	1	2

0	*0*	*0*	*8,57143*	*4,28571*	*0*	*0*	*0*	*2*
0	*0*	*0*	*12,8571*	*17,1429*	*0*	*0*	*0*	*2*
0	*0*	*0*	*0*	*0*	*0*	*0*	*0*	*1*
0	*1*	*1*	*120*	*207,143*	*0*	*1*	*1*	*1*
0	*0*	*0*	*68,5714*	*62,8571*	*0*	*1*	*1*	*2*
0	*1*	*1*	*90*	*68,5714*	*25,7143*	*1*	*1*	*2*
0	*0*	*0*	*28,5714*	*19,2857*	*12,8571*	*0*	*0*	*2*
0	*0*	*0*	*57,1429*	*111,429*	*0*	*0*	*1*	*2*
0	*0*	*0*	*28,5714*	*34,2857*	*0*	*0*	*0*	*2*
0	*0*	*0*	*55,7143*	*17,1429*	*0*	*0*	*0*	*1*
0	*0*	*0*	*11,4286*	*128,571*	*0*	*0*	*1*	*2*
0	*0*	*0*	*0*	*19,2857*	*0*	*0*	*0*	*1*
0	*1*	*1*	*240*	*94,2857*	*0*	*1*	*1*	*1*
0	*0*	*0*	*25,7143*	*77,1429*	*6,42857*	*0*	*1*	*2*
0	*0*	*0*	*30*	*51,4286*	*0*	*0*	*0*	*2*
1	*0*	*1*	*57,1429*	*120*	*0*	*0*	*1*	*2*
0	*0*	*0*	*120*	*55,7143*	*0*	*1*	*0*	*1*
0	*0*	*0*	*45,7143*	*25,7143*	*0*	*0*	*0*	*3*
0	*0*	*0*	*21,4286*	*68,5714*	*0*	*0*	*1*	*1*
0	*1*	*1*	*231,429*	*25,7143*	*0*	*1*	*0*	*2*
0	*0*	*0*	*0*	*81,4286*	*0*	*0*	*1*	*2*
0	*0*	*0*	*34,2857*	*17,1429*	*0*	*0*	*0*	*2*
0	*1*	*1*	*60*	*145,714*	*0*	*1*	*1*	*2*
0	*0*	*0*	*34,2857*	*0*	*34,2857*	*0*	*0*	*2*
0	*0*	*0*	*2,85714*	*42,8571*	*0*	*0*	*0*	*2*
0	*0*	*0*	*77,1429*	*90*	*12,8571*	*1*	*1*	*3*
0	*0*	*0*	*11,4286*	*34,2857*	*0*	*0*	*0*	*2*
0	*0*	*0*	*14,2857*	*51,4286*	*0*	*0*	*0*	*2*
0	*0*	*0*	*15*	*25,7143*	*0*	*0*	*0*	*3*
0	*0*	*0*	*8,57143*	*51,4286*	*0*	*0*	*0*	*2*
0	*1*	*1*	*85,7143*	*92,1429*	*0*	*1*	*1*	*1*
0	*1*	*1*	*137,143*	*56,4286*	*0*	*1*	*0*	*2*
0	*0*	*0*	*34,2857*	*68,5714*	*0*	*0*	*1*	*2*
0	*0*	*0*	*28,5714*	*28,5714*	*0*	*0*	*0*	*2*
0	*0*	*0*	*22,8571*	*68,5714*	*0*	*0*	*1*	*2*
0	*0*	*0*	*128,571*	*145,714*	*0*	*1*	*1*	*2*
0	*0*	*0*	*187,143*	*244,286*	*0*	*1*	*1*	*2*
0	*0*	*0*	*25,7143*	*49,2857*	*0*	*0*	*0*	*2*
0	*1*	*1*	*120*	*111,429*	*0*	*1*	*1*	*2*
0	*1*	*1*	*317,143*	*0*	*0*	*1*	*0*	*2*
0	*1*	*1*	*65,7143*	*8,57143*	*0*	*1*	*0*	*3*
0	*0*	*0*	*21,4286*	*4,28571*	*0*	*0*	*0*	*2*
0	*0*	*0*	*19,2857*	*42,8571*	*0*	*0*	*0*	*2*
0	*0*	*0*	*85,7143*	*102,857*	*102,857*	*1*	*1*	*2*
1	*1*	*1*	*191,429*	*231,429*	*0*	*1*	*1*	*2*
0	*0*	*0*	*32,1429*	*137,143*	*0*	*0*	*1*	*2*
0	*0*	*0*	*15,7143*	*71,4286*	*0*	*0*	*1*	*2*
0	*1*	*1*	*77,1429*	*32,8571*	*0*	*1*	*0*	*3*

0	0	0	14,2857	90	0	0	1	2
0	0	0	60	188,571	180	1	1	2
0	1	1	102,857	51,4286	0	1	0	2
0	0	0	17,1429	34,2857	0	0	0	2
0	0	0	0	0	0	0	0	2
0	0	0	0	137,143	0	0	1	2
0	0	0	25,7143	42,8571	0	0	0	2
0	0	0	5,71429	22,8571	0	0	0	3
0	0	0	23,5714	51,4286	0	0	0	2
0	0	0	47,1429	205,714	0	0	1	2
0	0	0	0	71,4286	0	0	1	2
0	0	0	128,571	90	8,57143	1	1	2
0	0	0	42,8571	82,8571	17,1429	0	1	2
0	0	0	30	201,429	0	0	1	2
0	1	1	231,429	105,714	0	1	1	1
0	1	1	165	17,1429	70,7143	1	1	2
0	1	1	127,143	17,1429	6,42857	1	0	2
0	0	0	28,5714	111,429	0	0	1	2
0	1	1	102,857	25,7143	0	1	0	2
0	0	0	111,429	102,857	0	1	1	2
0	0	0	37,1429	42,8571	0	0	0	2
0	0	0	64,2857	30	0	1	0	2
0	1	1	98,5714	128,571	0	1	1	1
0	0	0	12,8571	42,8571	0	0	0	3
0	0	0	35,7143	17,1429	0	0	0	2
0	0	0	25,7143	111,429	0	0	1	1
0	0	0	42,8571	42,8571	0	0	0	2
0	0	0	77,1429	102,857	42,8571	1	1	2
0	0	0	85,7143	17,1429	12,8571	1	0	2
0	0	1	72,8571	55,7143	0	1	0	2
0	1	1	60	30	0	1	0	1
0	0	0	10,7143	35,7143	12,8571	0	0	2
0	1	1	60	17,1429	34,2857	1	0	2
0	0	0	21,4286	85,7143	0	0	1	2
0	0	0	42,8571	111,429	0	0	1	3
0	0	0	25,7143	42,8571	0	0	0	2
0	0	0	30	2,85714	15	0	0	1
0	0	0	7,14286	25,7143	0	0	0	1
0	0	0	30	37,1429	0	0	0	1
0	0	0	8,57143	25,7143	0	0	0	2
0	0	0	7,14286	244,286	34,2857	0	1	2
0	0	0	25,7143	0	0	0	0	2
0	0	0	4,28571	19,2857	2,14286	0	0	2
0	0	0	205,714	0	0	1	0	2
0	0	0	11,4286	25,7143	0	0	0	1
0	1	1	85,7143	21,4286	0	1	0	2
0	0	0	40	17,1429	0	0	0	2
0	0	0	17,1429	317,143	0	0	1	2

0	0	0	128,571	107,143	0	1	1	2
0	0	0	171,429	120	0	1	1	1
0	0	0	40	90	0	0	1	3
0	0	0	60	42,8571	0	1	0	2
0	1	1	85,7143	51,4286	0	1	0	2
0	0	0	124,286	57,8571	0	1	0	2
0	0	0	55,7143	42,8571	0	0	0	1
0	0	0	60	51,4286	0	1	0	2
0	0	0	7,14286	17,1429	0	0	0	2
0	1	1	81,4286	61,4286	0	1	1	2
0	1	1	137,143	42,8571	0	1	0	2
0	0	0	51,4286	107,143	0	0	1	2
0	1	1	154,286	17,1429	0	1	0	2
0	0	0	20	77,1429	17,1429	0	1	3
0	1	1	68,5714	42,8571	0	1	0	2
0	0	0	62,1429	8,57143	21,4286	1	0	2
0	1	1	360	0	0	1	0	2
0	0	0	0	64,2857	17,1429	0	1	2
0	0	0	71,4286	0	0	1	0	3
0	0	0	62,1429	17,1429	0	1	0	2
0	0	0	17,1429	66,4286	0	0	1	1
0	0	0	10	8,57143	0	0	0	3
0	0	0	68,5714	25,7143	0	1	0	1
0	1	1	120	77,1429	0	1	1	3
0	1	1	77,1429	51,4286	0	1	0	2
0	0	0	25,7143	91,4286	0	0	1	2
0	0	0	17,1429	17,1429	0	0	0	3
0	0	0	8,57143	10,7143	77,1429	0	1	1
0	0	0	5,71429	317,143	60	0	1	3
0	0	0	23,5714	51,4286	30	0	1	1
0	0	0	36,4286	8,57143	0	0	0	2
0	0	0	0	42,8571	0	0	0	1
0	0	0	8,57143	154,286	0	0	1	1
0	0	0	17,1429	34,2857	0	0	0	2
0	0	0	0	102,857	0	0	1	2
0	1	1	128,571	0	0	1	0	2
0	0	0	17,1429	90	0	0	1	2
0	0	0	0	51,4286	0	0	0	2
0	0	1	205,714	40,7143	0	1	0	1
0	0	0	2,85714	51,4286	0	0	0	3
0	0	0	51,4286	120	0	0	1	1
0	0	0	11,4286	0	0	0	0	2
0	0	0	54,2857	17,1429	0	0	0	1
0	1	1	81,4286	168,571	0	1	1	1
0	0	0	0	240	0	0	1	3
0	0	0	11,4286	44,2857	0	0	0	3
0	0	0	51,4286	68,5714	0	0	1	2
0	0	0	12,8571	100	0	0	1	2

0	*0*	*0*	*0*	*145,714*	*30*	*0*	*1*	*2*
0	*0*	*0*	*0*	*0*	*0*	*0*	*0*	*2*
0	*0*	*0*	*0*	*162,857*	*0*	*0*	*1*	*2*
0	*0*	*0*	*32,8571*	*8,57143*	*0*	*0*	*0*	*2*
0	*1*	*1*	*102,857*	*115,714*	*21,4286*	*1*	*1*	*2*
0	*0*	*0*	*21,4286*	*71,4286*	*0*	*0*	*1*	*2*
0	*0*	*0*	*85,7143*	*94,2857*	*8,57143*	*1*	*1*	*2*
0	*0*	*0*	*207,143*	*39,2857*	*0*	*1*	*0*	*2*
0	*0*	*0*	*10,7143*	*124,286*	*0*	*0*	*1*	*1*
0	*0*	*0*	*51,4286*	*68,5714*	*0*	*0*	*1*	*2*
0	*0*	*0*	*60*	*51,4286*	*0*	*1*	*0*	*1*
0	*0*	*0*	*32,8571*	*17,1429*	*0*	*0*	*0*	*2*
0	*1*	*1*	*120*	*51,4286*	*0*	*1*	*0*	*1*
0	*0*	*0*	*20*	*55,7143*	*25,7143*	*0*	*1*	*1*
0	*0*	*0*	*2,85714*	*0*	*0*	*0*	*0*	*1*
0	*0*	*0*	*0*	*17,1429*	*8,57143*	*0*	*0*	*2*
0	*0*	*0*	*21,4286*	*68,5714*	*0*	*0*	*1*	*1*
0	*0*	*0*	*27,1429*	*51,4286*	*0*	*0*	*0*	*1*
0	*0*	*0*	*51,4286*	*64,2857*	*0*	*0*	*1*	*1*
0	*0*	*0*	*47,1429*	*51,4286*	*0*	*0*	*0*	*2*
0	*0*	*0*	*42,8571*	*51,4286*	*0*	*0*	*0*	*2*
0	*0*	*0*	*68,5714*	*0*	*0*	*1*	*0*	*2*
0	*0*	*0*	*94,2857*	*40*	*0*	*1*	*0*	*1*
0	*0*	*0*	*38,5714*	*51,4286*	*0*	*0*	*0*	*2*
0	*1*	*1*	*85,7143*	*60*	*0*	*1*	*1*	*1*
0	*0*	*0*	*8,57143*	*25,7143*	*0*	*0*	*0*	*1*
0	*0*	*0*	*51,4286*	*34,2857*	*0*	*0*	*0*	*2*
0	*0*	*0*	*61,4286*	*51,4286*	*0*	*1*	*0*	*2*
0	*0*	*0*	*50*	*25,7143*	*0*	*0*	*0*	*3*
0	*0*	*0*	*42,8571*	*60*	*0*	*0*	*1*	*2*
0	*1*	*1*	*80*	*30*	*0*	*1*	*0*	*2*
0	*0*	*0*	*7,14286*	*8,57143*	*0*	*0*	*0*	*2*
0	*1*	*1*	*300*	*199,286*	*12,8571*	*1*	*1*	*2*
0	*0*	*0*	*50*	*120*	*0*	*0*	*1*	*2*
0	*0*	*0*	*0*	*51,4286*	*0*	*0*	*0*	*1*
0	*0*	*0*	*14,2857*	*17,1429*	*0*	*0*	*0*	*2*
0	*0*	*0*	*30*	*141,429*	*8,57143*	*0*	*1*	*2*
0	*0*	*0*	*141,429*	*145,714*	*8,57143*	*1*	*1*	*2*
0	*0*	*0*	*8,57143*	*180*	*0*	*0*	*1*	*2*
0	*0*	*0*	*101,429*	*8,57143*	*0*	*1*	*0*	*2*
0	*0*	*0*	*150*	*171,429*	*0*	*1*	*1*	*2*
0	*1*	*1*	*197,143*	*222,857*	*77,1429*	*1*	*1*	*2*
0	*0*	*0*	*38,5714*	*42,8571*	*0*	*0*	*0*	*1*
0	*0*	*0*	*17,1429*	*68,5714*	*0*	*0*	*1*	*1*
0	*0*	*0*	*21,4286*	*68,5714*	*0*	*0*	*1*	*2*
0	*0*	*0*	*10,7143*	*128,571*	*0*	*0*	*1*	*2*
0	*0*	*0*	*0*	*67,1429*	*0*	*0*	*1*	*2*
0	*1*	*1*	*240*	*0*	*0*	*1*	*0*	*2*

0	0	0	28,5714	51,4286	0	0	0	1
0	0	0	7,14286	12,8571	0	0	0	1
0	0	0	30	51,4286	0	0	0	1
0	0	0	10,7143	128,571	0	0	1	2
0	0	0	32,1429	8,57143	0	0	0	2
0	0	0	37,1429	25,7143	0	0	0	2
0	0	0	57,8571	111,429	0	0	1	2
0	1	1	210	25,7143	17,1429	1	0	2
0	0	0	51,4286	25,7143	0	0	0	2
0	0	0	30	217,143	0	0	1	3
0	0	0	115,714	107,143	0	1	1	2
0	0	0	23,5714	14,2857	0	0	0	2
0	1	1	60	102,857	0	1	1	2
0	0	0	0	51,4286	34,2857	0	1	2
0	1	1	64,2857	51,4286	0	1	0	3
0	0	0	0	30	0	0	0	3
0	0	0	25,7143	22,8571	0	0	0	2
0	0	0	120	17,1429	0	1	0	2
0	0	0	60	308,571	128,571	1	1	2
0	0	0	28,5714	139,286	0	0	1	2
0	0	0	54,2857	25,7143	14,2857	0	0	2
0	0	0	47,1429	47,1429	0	0	0	2
0	0	0	53,5714	180	0	0	1	2
0	1	1	77,1429	19,2857	0	1	0	2
0	1	1	145,714	34,2857	0	1	0	2
0	0	1	184,286	27,8571	51,4286	1	1	2
0	1	1	60	102,857	0	1	1	2
0	0	0	68,5714	36,4286	0	1	0	1
0	0	0	51,4286	17,1429	0	0	0	1
0	0	0	30	22,8571	0	0	0	2
0	0	0	145,714	102,857	0	1	1	2
0	0	0	21,4286	85,7143	0	0	1	2
0	0	0	68,5714	17,1429	0	1	0	2
0	1	1	90	120	0	1	1	1
0	0	0	42,8571	8,57143	0	0	0	1
0	1	1	160	128,571	0	1	1	3
0	0	0	34,2857	60	0	0	1	1
0	0	0	21,4286	17,1429	0	0	0	2
0	0	0	30	39,2857	0	0	0	2
0	0	0	34,2857	25,7143	0	0	0	2
0	0	0	0	180	128,571	0	1	2
0	0	0	4,28571	77,1429	0	0	1	1
0	0	0	30	30	0	0	0	1
0	0	0	68,5714	0	0	1	0	2
0	0	0	32,1429	22,8571	0	0	0	2
0	1	1	162,857	60	0	1	1	3
0	0	0	7,14286	25,7143	0	0	0	1
0	0	0	77,1429	102,857	0	1	1	2

0	0	0	20	57,1429	0	0	0	2
0	0	0	51,4286	51,4286	0	0	0	2
0	1	1	120	90	34,2857	1	1	2
0	0	0	75	42,8571	0	1	0	2
0	0	0	51,4286	85,7143	102,857	0	1	2
0	0	0	21,4286	366,429	0	0	1	2
0	0	0	128,571	34,2857	0	1	0	2
0	0	0	17,1429	42,8571	12,8571	0	0	2
0	0	0	7,14286	102,857	0	0	1	2
0	0	0	14,2857	17,1429	0	0	0	3
0	0	0	71,4286	25,7143	0	1	0	1
0	0	0	30	83,5714	0	0	1	1
0	0	0	180	25,7143	0	1	0	3
0	0	0	94,2857	25,7143	0	1	0	1
0	0	0	17,1429	51,4286	0	0	0	2
0	0	0	68,5714	72,8571	0	1	1	1
0	0	0	68,5714	68,5714	0	1	1	2
0	0	0	25,7143	92,1429	102,857	0	1	2
0	0	0	5,71429	68,5714	0	0	1	1
0	1	1	120	30	0	1	0	1
0	0	0	25,7143	42,8571	0	0	0	2
0	1	1	102,857	0	0	1	0	2
0	0	0	34,2857	177,857	38,5714	0	1	2
0	0	0	0	12,8571	28,5714	0	0	2
0	0	0	14,2857	51,4286	0	0	0	2
0	1	1	77,1429	85,7143	0	1	1	2
0	0	0	30	77,1429	0	0	1	2
0	0	0	42,8571	51,4286	0	0	0	1
0	0	0	0	21,4286	0	0	0	1
0	0	0	60	30	0	1	0	1
0	0	0	120	0	0	1	0	1
0	0	0	55,7143	55,7143	17,1429	0	1	2
0	0	0	7,14286	25,7143	0	0	0	1
0	0	0	50	1,42857	0	0	0	2
0	0	0	64,2857	12,8571	0	1	0	1
0	0	0	17,1429	120	0	0	1	3
0	0	0	22,1429	45,7143	12,8571	0	0	2
0	1	1	240	25,7143	0	1	0	2
0	0	0	32,1429	25,7143	0	0	0	2
0	0	0	85,7143	21,4286	0	1	0	1
0	0	0	50	90	0	0	1	2
0	0	0	17,1429	111,429	0	0	1	2
0	0	0	60	30	0	1	0	3
0	0	0	20	34,2857	51,4286	0	1	2
0	0	0	34,2857	25,7143	0	0	0	2
0	0	0	60	30	12,8571	1	0	2
0	1	1	60	37,1429	25,7143	1	1	2
0	0	0	45,7143	8,57143	0	0	0	2

0	1	1	96,4286	34,2857	0	1	0	1
0	1	1	128,571	180	0	1	1	3
0	0	0	15	27,1429	0	0	0	2
0	1	1	120	137,143	0	1	1	1
0	0	0	17,1429	19,2857	0	0	0	3
0	0	0	51,4286	34,2857	0	0	0	1
0	0	0	10	25,7143	0	0	0	1
0	0	0	1,42857	60	0	0	1	2
0	0	0	102,857	85,7143	0	1	1	2
0	0	0	38,5714	154,286	0	0	1	2
0	0	0	34,2857	0	0	0	0	1
0	0	0	55,7143	42,8571	0	0	0	2
0	1	1	360	51,4286	0	1	0	2
0	0	0	12,8571	72,8571	0	0	1	2
0	0	0	0	188,571	0	0	1	3
0	0	0	28,5714	51,4286	0	0	0	2
1	0	1	4,28571	145,714	17,1429	0	1	2
0	1	1	162,857	210	0	1	1	2
0	1	1	70	8,57143	0	1	0	2
0	0	0	4,28571	25,7143	0	0	0	2
0	0	0	0	124,286	0	0	1	2
0	0	0	31,4286	154,286	51,4286	0	1	1
0	0	0	32,1429	60	0	0	1	3
0	0	0	154,286	137,143	25,7143	1	1	3
0	0	1	57,1429	30	34,2857	0	1	2
0	0	0	0	162,857	0	0	1	2
0	1	1	61,4286	15,7143	0	1	0	1
0	0	0	0	21,4286	0	0	0	2
0	0	0	0	25,7143	0	0	0	2
0	0	0	27,1429	0	0	0	0	2
0	0	0	34,2857	174,286	0	0	1	2
0	0	0	4,28571	68,5714	0	0	1	2
0	0	0	138,571	0	0	1	0	2
0	0	0	42,8571	137,143	17,1429	0	1	2
0	0	0	51,4286	25,7143	0	0	0	1
0	0	0	85,7143	6,42857	0	1	0	1
0	0	0	55,7143	45	0	0	0	2
0	0	0	17,1429	94,2857	0	0	1	1
0	0	0	0	34,2857	17,1429	0	0	2
0	0	0	102,857	42,8571	0	1	0	2
0	0	0	120	21,4286	0	1	0	2
0	0	0	24,2857	42,8571	0	0	0	2
0	0	0	0	175,714	0	0	1	2
0	0	0	17,1429	128,571	0	0	1	2
0	0	0	30	34,2857	102,857	0	1	1
0	0	0	5,71429	55,7143	0	0	0	1
0	0	1	102,857	210	0	1	1	1
0	0	0	30	0	32,1429	0	0	2

0	0	0	30	274,286	0	0	1	2
0	0	0	0	0	0	0	0	2
0	0	0	4,28571	72,8571	0	0	1	2
0	0	0	51,4286	111,429	0	0	1	2
0	0	0	120	117,143	0	1	1	2
0	0	0	128,571	22,8571	0	1	0	2
0	0	0	17,1429	145,714	0	0	1	3
0	0	0	34,2857	34,2857	0	0	0	1
0	0	0	21,4286	4,28571	0	0	0	2
0	0	0	30	51,4286	0	0	0	1
0	0	0	8,57143	0	0	0	0	1
0	0	0	14,2857	12,8571	0	0	0	1
0	0	0	102,857	188,571	0	1	1	2
0	0	0	10,7143	25,7143	0	0	0	2
0	0	0	0	51,4286	0	0	0	1
0	0	0	30	94,2857	51,4286	0	1	2
0	0	0	8,57143	34,2857	30	0	1	2
0	0	0	2,85714	8,57143	0	0	0	2
0	0	0	128,571	32,1429	32,1429	1	1	1
0	1	1	85,7143	14,2857	0	1	0	2
0	0	0	30	38,5714	0	0	0	1
0	0	0	32,8571	42,8571	0	0	0	3
0	0	0	55,7143	57,1429	0	0	0	2
0	0	0	0	51,4286	0	0	0	1
0	0	0	47,1429	12,8571	0	0	0	3
0	0	0	12,8571	205,714	0	0	1	2
0	1	1	128,571	68,5714	0	1	1	1
0	0	0	8,57143	37,1429	0	0	0	2
0	0	0	10,7143	32,1429	0	0	0	2
0	0	0	34,2857	10	17,1429	0	0	1
0	1	1	150	44,2857	0	1	0	1
0	0	0	21,4286	25,7143	0	0	0	2
0	0	0	51,4286	42,8571	0	0	0	2
0	0	0	37,1429	145,714	0	0	1	2
0	0	0	154,286	60	0	1	1	2
0	0	0	68,5714	60	0	1	1	1
0	0	0	8,57143	42,8571	0	0	0	1
0	0	0	120	195	0	1	1	2
0	0	0	68,5714	42,8571	0	1	0	2
0	0	0	0	40,7143	0	0	0	2
0	0	0	12,8571	17,1429	0	0	0	3
0	0	0	10,7143	25,7143	85,7143	0	1	2
0	1	1	191,429	0	0	1	0	2
0	0	0	8,57143	45	0	0	0	2
0	0	1	77,1429	85,7143	0	1	1	1
0	0	0	21,4286	77,1429	0	0	1	2
0	0	0	42,8571	68,5714	0	0	1	3
0	0	0	21,4286	8,57143	0	0	0	3

0	0	0	15,7143	102,857	25,7143	0	1	2
0	0	0	65,7143	17,1429	0	1	0	2
0	0	0	17,1429	25,7143	0	0	0	2
0	0	0	38,5714	85,7143	0	0	1	2
0	0	0	34,2857	30	0	0	0	1
0	0	0	51,4286	25,7143	0	0	0	2
0	0	0	37,1429	31,4286	0	0	0	2
0	0	0	77,1429	231,429	0	1	1	2
0	0	0	17,1429	17,1429	6,42857	0	0	3
0	0	0	31,4286	80	0	0	1	2
0	0	0	7,14286	51,4286	0	0	0	1
0	0	0	55,7143	45	0	0	0	2
0	1	1	111,429	68,5714	0	1	1	2
0	0	0	12,8571	42,8571	51,4286	0	1	2
0	0	0	77,1429	8,57143	0	1	0	1
0	1	1	142,857	60	0	1	1	2
0	0	0	8,57143	94,2857	0	0	1	2
0	0	0	38,5714	53,5714	0	0	0	2
0	0	0	34,2857	21,4286	0	0	0	1
0	0	0	94,2857	94,2857	17,1429	1	1	2
0	0	0	84,2857	17,1429	0	1	0	2
0	0	0	20	120	51,4286	0	1	1
0	0	0	85,7143	125,714	77,1429	1	1	2
0	0	0	51,4286	14,2857	0	0	0	2
0	1	1	145,714	45	0	1	0	2
1	0	1	25,7143	145,714	0	0	1	2
0	0	0	6,42857	50,7143	0	0	0	2
0	0	0	12,8571	152,857	0	0	1	3
0	0	0	42,8571	35,7143	0	0	0	1
0	0	0	42,8571	51,4286	0	0	0	2
0	1	1	180	120	0	1	1	2
0	0	0	25,7143	0	0	0	0	1
0	1	1	128,571	53,5714	0	1	0	2
0	0	0	128,571	42,8571	28,5714	1	1	2
0	0	0	15	17,1429	0	0	0	2
0	0	0	36,4286	51,4286	0	0	0	2
0	0	0	30	147,143	0	0	1	2
0	0	0	8,57143	4,28571	0	0	0	2
0	0	0	25,7143	197,143	0	0	1	2
0	0	0	111,429	60	0	1	1	2
0	1	1	240	102,857	0	1	1	2
0	0	0	248,571	231,429	25,7143	1	1	2
0	0	0	145,714	51,4286	0	1	0	2
0	0	0	34,2857	51,4286	0	0	0	2
0	0	0	28,5714	120	30	0	1	2
0	0	0	38,5714	49,2857	2,14286	0	0	1
0	0	0	20	12,8571	17,1429	0	0	2
0	0	0	23,5714	21,4286	0	0	0	2

0	0	0	60	17,1429	0	1	0	1
0	0	0	0	60	42,8571	0	1	2
0	0	0	10,7143	42,8571	0	0	0	3
0	0	0	15	25,7143	0	0	0	1
0	0	0	60	205,714	0	1	1	2
0	0	0	60	4,28571	51,4286	1	0	2
0	0	0	21,4286	180	0	0	1	2
0	0	0	45,7143	8,57143	4,28571	0	0	2
0	0	0	17,1429	64,2857	0	0	1	2
0	0	0	17,1429	49,2857	0	0	0	3
0	1	1	80	21,4286	0	1	0	2
0	0	0	34,2857	0	0	0	0	3
0	0	0	25,7143	30	0	0	0	1
0	0	0	0	68,5714	0	0	1	2
0	0	1	171,429	197,143	17,1429	1	1	2
0	0	0	94,2857	94,2857	0	1	1	2
0	0	0	0	77,1429	0	0	1	2
0	0	0	10	17,1429	0	0	0	2
0	0	0	85,7143	108,571	0	1	1	1
0	0	0	35,7143	25,7143	0	0	0	1
0	1	1	120	0	0	1	0	2
0	0	0	51,4286	47,1429	0	0	0	3
0	0	0	60	17,1429	25,7143	1	0	3
0	0	0	102,857	71,4286	17,1429	1	1	1
0	0	0	17,1429	52,8571	0	0	0	2
0	0	0	47,1429	128,571	0	0	1	1
0	0	0	0	68,5714	85,7143	0	1	2
0	0	0	85,7143	34,2857	0	1	0	2
0	1	1	120	68,5714	0	1	1	1
0	1	1	180	51,4286	0	1	0	1
0	0	0	1,42857	197,143	0	0	1	1
0	0	0	38,5714	34,2857	0	0	0	3
0	0	0	20	30	0	0	0	2
0	0	0	30	77,1429	0	0	1	1
0	0	0	0	8,57143	0	0	0	1
0	0	0	42,8571	128,571	0	0	1	2
0	0	0	91,4286	102,857	0	1	1	1
0	0	0	83,5714	102,857	0	1	1	2
0	0	0	80	42,8571	0	1	0	2
0	0	0	21,4286	34,2857	0	0	0	2
0	0	0	60	60	0	1	1	2
0	0	0	38,5714	8,57143	14,2857	0	0	2
0	0	0	17,1429	25,7143	0	0	0	3
0	0	0	17,1429	8,57143	0	0	0	1
0	1	1	60	10	0	1	0	1
0	0	0	60	51,4286	0	1	0	2
0	0	1	85,7143	38,5714	0	1	0	1
0	0	0	38,5714	25,7143	0	0	0	2

0	0	0	12,8571	85,7143	17,1429	0	1	2
0	0	0	30	60	0	0	1	3
0	0	0	34,2857	85,7143	0	0	1	2
0	0	1	231,429	42,8571	0	1	0	2
0	0	0	8,57143	37,1429	0	0	0	2
0	0	0	34,2857	68,5714	0	0	1	2
0	0	1	77,1429	38,5714	0	1	0	2
0	0	0	28,5714	30	0	0	0	1
0	0	1	90	287,143	0	1	1	2
0	0	0	30	30	0	0	0	2
0	0	0	0	0	0	0	0	1
0	1	1	90	47,1429	0	1	0	1
0	0	0	8,57143	0	0	0	0	2
0	0	1	94,2857	8,57143	0	1	0	2
0	0	0	5,71429	25,7143	0	0	0	2
0	0	0	120	111,429	0	1	1	3
0	0	0	34,2857	42,8571	0	0	0	2
0	0	0	184,286	17,1429	17,1429	1	0	2
0	0	0	40	34,2857	0	0	0	2
0	0	0	60	51,4286	0	1	0	2
0	0	0	7,14286	17,1429	0	0	0	2
0	1	1	180	167,143	0	1	1	1
0	0	0	47,1429	51,4286	0	0	0	2
0	0	0	45	137,143	0	0	1	1
0	1	1	122,857	25,7143	0	1	0	2
0	0	1	171,429	192,857	0	1	1	3
0	0	0	8,57143	34,2857	0	0	0	2
0	0	0	17,1429	25,7143	0	0	0	2
0	0	0	2,85714	25,7143	0	0	0	2
0	1	1	92,8571	21,4286	0	1	0	3
0	0	0	31,4286	51,4286	0	0	0	1
0	0	0	42,8571	42,8571	0	0	0	1
0	0	0	50	55,7143	0	0	0	1
0	1	1	197,143	128,571	8,57143	1	1	2
0	0	0	0	42,8571	25,7143	0	1	2
0	0	0	55,7143	77,1429	0	0	1	1
0	0	0	20	25,7143	0	0	0	1
0	0	0	68,5714	17,1429	6,42857	1	0	2
0	0	0	0	240	0	0	1	1
0	0	0	47,1429	77,1429	0	0	1	2
0	0	0	8,57143	41,4286	0	0	0	2
0	0	0	35,7143	0	0	0	0	2
0	0	0	111,429	135	0	1	1	2
0	0	0	22,8571	85,7143	0	0	1	2
0	0	0	8,57143	8,57143	0	0	0	2
0	0	0	20	25,7143	0	0	0	2
0	0	0	8,57143	25,7143	0	0	0	3
0	0	0	10	142,143	154,286	0	1	1

0	0	0	94,2857	60	0	1	1	1
0	0	0	12,8571	60	0	0	1	2
0	0	0	70	61,4286	0	1	1	2
0	0	0	65,7143	68,5714	0	1	1	2
0	0	0	20	260	180	0	1	2
0	0	0	37,1429	12,8571	0	0	0	2
0	0	0	42,8571	0	0	0	0	2
0	0	0	8,57143	77,1429	0	0	1	1
0	0	0	2,85714	274,286	77,1429	0	1	1
0	0	0	77,1429	80	8,57143	1	1	2
0	0	0	62,1429	60	0	1	1	2
0	0	0	68,5714	60	34,2857	1	1	2
0	0	0	17,1429	53,5714	0	0	0	1
0	0	0	128,571	42,8571	85,7143	1	1	2
0	0	1	218,571	252,857	60	1	1	2
0	0	0	33,5714	17,1429	17,1429	0	0	1
0	0	1	60	85,7143	0	1	1	2
0	0	0	77,1429	180	0	1	1	1
0	0	0	44,2857	60	0	0	1	1
0	0	0	60	51,4286	0	1	0	3
0	0	0	21,4286	60	0	0	1	2
0	0	0	27,8571	2,85714	0	0	0	2
0	0	0	12,8571	60	0	0	1	1
0	1	1	120	25,7143	0	1	0	2
0	0	0	67,1429	25,7143	0	1	0	2
0	0	0	34,2857	51,4286	0	0	0	1
0	0	0	20	57,1429	51,4286	0	1	2
0	0	0	25,7143	205,714	0	0	1	2
0	0	0	0	257,143	0	0	1	1
0	1	1	195	52,8571	0	1	0	2
0	0	0	48,5714	25,7143	0	0	0	1
0	0	0	21,4286	4,28571	4,28571	0	0	1
0	0	0	142,857	0	0	1	0	1
0	0	0	42,8571	51,4286	0	0	0	1
0	1	1	90	17,1429	0	1	0	2
0	1	1	98,5714	59,2857	0	1	0	1
0	0	0	30	17,1429	0	0	0	1
0	0	0	35,7143	171,429	0	0	1	1
0	0	0	51,4286	51,4286	0	0	0	2
0	0	0	0	141,429	0	0	1	3
0	0	0	27,8571	51,4286	0	0	0	2
0	0	0	8,57143	30	0	0	0	3
0	0	0	128,571	231,429	85,7143	1	1	2
0	1	1	105,714	102,857	0	1	1	2
0	0	0	34,2857	68,5714	0	0	1	3
0	0	0	5,71429	64,2857	40	0	1	2
0	1	1	205,714	25,7143	0	1	0	2
0	0	0	154,286	0	0	1	0	3

0	1	1	94,2857	51,4286	0	1	0	2
0	0	0	47,1429	8,57143	0	0	0	2
0	0	0	21,4286	42,8571	25,7143	0	1	2
0	0	0	25,7143	77,1429	0	0	1	2
0	0	0	42,8571	25,7143	0	0	0	3
0	0	0	17,1429	51,4286	25,7143	0	1	2
0	0	0	38,5714	77,1429	102,857	0	1	2
0	1	1	180	60	0	1	1	2
0	0	0	21,4286	34,2857	5,71429	0	0	2
0	0	0	47,1429	0	0	0	0	1
0	0	0	137,143	188,571	34,2857	1	1	2
0	0	0	11,4286	102,857	0	0	1	1
0	0	0	28,5714	25,7143	42,8571	0	1	2
0	0	0	51,4286	68,5714	0	0	1	2
0	0	0	24,2857	35,7143	0	0	0	1
0	0	0	0	4,28571	0	0	0	2
0	1	1	205,714	17,1429	0	1	0	2
0	1	1	240	81,4286	0	1	1	2
0	0	0	42,8571	34,2857	17,1429	0	0	2
0	0	0	70	42,8571	0	1	0	1
0	1	1	300	34,2857	0	1	0	2
0	0	0	102,857	102,857	0	1	1	2
0	0	0	14,2857	17,1429	0	0	0	3
0	0	0	8,57143	17,1429	0	0	0	2
0	0	0	25,7143	8,57143	0	0	0	2
0	0	0	23,5714	90	8,57143	0	1	1
0	0	0	51,4286	34,2857	0	0	0	1
0	0	0	122,143	90	0	1	1	2
0	1	1	60	132,857	0	1	1	2
0	1	1	94,2857	34,2857	0	1	0	1
0	1	1	108,571	72,8571	0	1	1	1
0	0	0	0	25,7143	0	0	0	1
0	0	0	17,1429	68,5714	0	0	1	2
0	0	0	0	53,5714	17,1429	0	1	2
0	0	0	21,4286	21,4286	0	0	0	2
0	0	0	38,5714	25,7143	15	0	0	2
0	0	0	64,2857	35,7143	0	1	0	2
0	0	0	94,2857	37,1429	0	1	0	1
0	0	0	70	77,1429	0	1	1	2
0	1	1	66,4286	34,2857	0	1	0	3
0	1	1	180	17,1429	0	1	0	2
0	0	0	68,5714	42,8571	0	1	0	2
0	0	0	51,4286	34,2857	15	0	0	1
0	0	0	77,1429	32,1429	0	1	0	1
0	0	0	8,57143	25,7143	0	0	0	2
0	0	0	0	145,714	0	0	1	1
0	0	0	0	85,7143	0	0	1	2
0	0	0	51,4286	25,7143	0	0	0	1

0	0	0	51,4286	40,7143	17,1429	0	0	2
0	0	0	8,57143	8,57143	0	0	0	1
0	0	0	12,8571	45,7143	0	0	0	3
0	0	0	14,2857	77,1429	0	0	1	1
1	1	1	240	42,8571	145,714	1	1	2
0	0	0	7,14286	38,5714	51,4286	0	1	2
0	0	0	30	30	0	0	0	2
0	0	0	55,7143	124,286	0	0	1	2
0	1	1	137,143	51,4286	0	1	0	2
0	0	0	51,4286	51,4286	28,5714	0	1	1
0	0	0	72,8571	51,4286	17,1429	1	1	2
0	1	1	300	51,4286	4,28571	1	0	2
0	0	0	17,1429	34,2857	0	0	0	2
0	0	0	31,4286	34,2857	0	0	0	2
0	0	0	0	137,143	0	0	1	2
0	0	0	28,5714	17,1429	0	0	0	2
0	0	0	2,85714	124,286	0	0	1	3
0	0	1	85,7143	180	38,5714	1	1	1
0	0	0	60	25,7143	0	1	0	1
0	0	0	12,8571	55,7143	0	0	0	2
0	0	0	10,7143	85,7143	0	0	1	2
0	0	0	20	20	2,85714	0	0	2
0	0	0	34,2857	17,1429	8,57143	0	0	1
0	0	0	30	34,2857	0	0	0	2
0	0	0	27,1429	180	0	0	1	1
0	0	0	51,4286	212,143	0	0	1	2
0	0	0	18,5714	154,286	25,7143	0	1	2
0	0	0	23,5714	137,143	27,8571	0	1	1
0	0	0	8,57143	66,4286	0	0	1	1
0	0	0	0	94,2857	0	0	1	2
0	0	0	62,8571	77,1429	0	1	1	2
0	0	0	17,1429	47,1429	0	0	0	1
0	0	0	0	111,429	0	0	1	1
0	0	0	60	51,4286	0	1	0	1
0	0	0	30	102,857	0	0	1	1
0	0	0	64,2857	5,71429	0	1	0	2
0	0	0	42,8571	42,8571	0	0	0	1
0	0	0	42,8571	111,429	0	0	1	2
0	0	0	0	90	0	0	1	1
0	0	0	31,4286	109,286	0	0	1	2
1	0	1	8,57143	25,7143	102,857	0	1	2
0	0	0	38,5714	120	0	0	1	1
0	0	1	102,857	137,143	60	1	1	2
0	0	0	28,5714	42,8571	32,1429	0	1	3
0	0	0	68,5714	12,8571	0	1	0	1
0	0	0	60	330	120	1	1	1
0	0	0	12,8571	34,2857	0	0	0	2
0	0	0	47,1429	51,4286	0	0	0	3

0	0	0	68,5714	55,7143	15	1	1	2
0	1	1	77,1429	188,571	0	1	1	2
0	0	0	58,5714	25,7143	0	0	0	2
0	0	0	80	11,4286	0	1	0	1
1	1	1	141,429	94,2857	0	1	1	2
0	0	0	34,2857	188,571	0	0	1	2
0	1	1	240	4,28571	0	1	0	2
0	0	1	115,714	128,571	85,7143	1	1	3
0	0	0	0	42,8571	0	0	0	2
0	1	1	77,1429	42,8571	0	1	0	1
0	0	0	60	68,5714	0	1	1	1
0	0	0	55,7143	51,4286	0	0	0	2
0	0	0	34,2857	34,2857	0	0	0	2
0	0	0	162,857	68,5714	0	1	1	2
0	0	0	17,1429	8,57143	0	0	0	2
1	0	1	85,7143	608,571	34,2857	1	1	1
0	0	0	40,7143	51,4286	0	0	0	2
0	0	0	20	260	180	0	1	2
0	0	0	57,8571	64,2857	30	0	1	3
0	0	0	214,286	120	0	1	1	2
0	0	0	0	0	0	0	0	2
0	0	0	0	25,7143	0	0	0	1
0	1	1	142,857	51,4286	0	1	0	2
0	0	0	0	55,7143	85,7143	0	1	2
0	0	0	137,143	15	34,2857	1	0	2
0	1	1	60	197,143	0	1	1	3
1	0	1	145,714	291,429	42,8571	1	1	2
0	0	0	51,4286	34,2857	0	0	0	2
0	1	1	107,143	25,7143	0	1	0	2
0	0	1	28,5714	77,1429	0	0	1	3
0	0	0	42,8571	34,2857	0	0	0	2
0	0	0	8,57143	160	68,5714	0	1	1
0	0	0	17,1429	102,857	17,1429	0	1	1
0	0	1	68,5714	122,143	68,5714	1	1	2
0	0	0	17,1429	25,7143	0	0	0	2
0	0	0	55,7143	60	0	0	1	2
0	0	0	35,7143	171,429	0	0	1	2
0	0	0	14,2857	42,8571	0	0	0	3
0	0	0	30	117,143	17,1429	0	1	2
0	0	0	30	17,1429	0	0	0	1
0	0	0	17,1429	231,429	0	0	1	3
0	1	1	90	52,8571	0	1	0	2
0	0	0	5,71429	34,2857	0	0	0	1
0	0	0	30	25,7143	0	0	0	2
0	0	0	85,7143	42,8571	0	1	0	2
0	0	0	31,4286	70	0	0	1	1
0	0	0	8,57143	0	0	0	0	3
0	1	1	82,8571	197,143	0	1	1	1

0	0	0	0	30	0	0	0	2
0	0	0	85,7143	25,7143	0	1	0	1
0	0	0	38,5714	32,1429	0	0	0	2
0	0	0	40,7143	42,8571	0	0	0	2
0	0	0	42,8571	42,8571	0	0	0	2
0	0	0	60	180	0	1	1	2
0	0	0	25,7143	34,2857	0	0	0	1
0	0	0	50	42,8571	0	0	0	2
0	0	0	47,1429	30	0	0	0	1
0	0	0	51,4286	120	0	0	1	2
0	0	0	51,4286	8,57143	0	0	0	1
0	0	0	7,14286	25,7143	0	0	0	1
0	0	0	51,4286	25,7143	38,5714	0	1	1
0	0	0	60	25,7143	17,1429	1	0	2
0	0	0	17,1429	42,8571	0	0	0	3
0	1	1	240	145,714	0	1	1	2
0	0	0	14,2857	0	0	0	0	2
0	0	0	30	71,4286	51,4286	0	1	2
0	0	0	8,57143	10,7143	0	0	0	2
0	0	0	0	128,571	0	0	1	1
0	0	0	6,42857	145,714	0	0	1	2
0	1	1	130	60	8,57143	1	1	2
0	1	1	147,857	85,7143	0	1	1	2
0	0	0	70,7143	128,571	0	1	1	2
0	0	0	17,1429	21,4286	25,7143	0	0	3
0	0	0	27,8571	25,7143	0	0	0	1
0	0	0	17,1429	102,857	0	0	1	1
0	0	0	5,71429	114,286	0	0	1	1
0	0	0	145,714	145,714	0	1	1	2
0	0	0	5,71429	94,2857	0	0	1	2
0	0	0	42,8571	188,571	25,7143	0	1	1
0	0	0	12,8571	111,429	0	0	1	2
0	0	0	51,4286	49,2857	0	0	0	3
0	0	0	10,7143	19,2857	4,28571	0	0	2
0	0	0	25,7143	102,857	0	0	1	2
0	1	1	90	120	0	1	1	2
0	0	0	0	120	0	0	1	2
0	0	0	102,857	0	0	1	0	2
0	0	0	60	102,857	0	1	1	2
1	0	1	0	248,571	145,714	0	1	1
0	0	0	137,143	205,714	0	1	1	1
0	0	0	0	85,7143	0	0	1	1
0	0	0	64,2857	15	0	1	0	2
0	0	0	17,1429	47,1429	0	0	0	2
0	0	0	37,1429	20	0	0	0	2
0	0	0	145,714	25,7143	0	1	0	1
0	0	0	5,71429	17,1429	0	0	0	1
0	0	0	102,857	40	2,85714	1	0	1

0	0	0	17,1429	68,5714	0	0	1	1
0	0	0	30	85,7143	0	0	1	2
0	0	0	42,8571	17,1429	0	0	0	2
0	1	1	127,143	25,7143	0	1	0	1
0	0	0	102,857	8,57143	0	1	0	2
0	0	0	8,57143	2,85714	0	0	0	2
0	0	0	21,4286	36,4286	4,28571	0	0	2
0	0	0	28,5714	162,857	68,5714	0	1	2
0	0	0	188,571	192,857	0	1	1	2
0	0	0	85,7143	68,5714	6,42857	1	1	2
0	0	0	38,5714	98,5714	0	0	1	2
0	0	0	25,7143	85,7143	0	0	1	1
0	0	0	5,71429	60	0	0	1	2
0	0	0	8,57143	40,7143	0	0	0	2
0	0	0	38,5714	28,5714	17,1429	0	0	2
0	0	0	90	68,5714	51,4286	1	1	1
0	1	1	140	55,7143	0	1	0	1
0	0	0	0	42,8571	0	0	0	2
0	0	0	25,7143	120	60	0	1	2
0	0	0	64,2857	8,57143	0	1	0	2
0	0	0	0	17,1429	0	0	0	2
0	0	0	30	12,8571	0	0	0	1
0	0	0	100	19,2857	0	1	0	2
0	0	0	92,8571	171,429	0	1	1	1
0	0	0	0	137,143	12,8571	0	1	2
0	0	0	25,7143	8,57143	0	0	0	2
0	0	0	100	35,7143	34,2857	1	1	3
0	0	0	62,8571	6,42857	0	1	0	2
0	0	0	0	102,857	0	0	1	2
0	0	0	265,714	162,857	0	1	1	2
0	0	0	23,5714	180	0	0	1	2
0	0	0	42,8571	28,5714	0	0	0	1
0	0	0	42,8571	42,8571	0	0	0	1
0	0	0	14,2857	57,8571	0	0	0	2
0	0	0	14,2857	25,7143	0	0	0	2
0	0	0	44,2857	0	0	0	0	1
0	0	0	34,2857	85,7143	0	0	1	1
0	0	0	11,4286	77,1429	17,1429	0	1	2
0	0	0	37,1429	55,7143	0	0	0	2
0	0	1	102,857	60	0	1	1	1
0	1	1	85,7143	132,857	0	1	1	2
0	0	0	27,8571	85,7143	0	0	1	2
0	0	0	25,7143	7,14286	8,57143	0	0	3
0	0	0	42,8571	17,1429	0	0	0	2
0	0	0	60	34,2857	0	1	0	1
0	0	0	17,1429	25,7143	0	0	0	2
0	0	0	98,5714	45	0	1	0	1
0	0	0	64,2857	17,1429	0	1	0	1

0	0	0	57,1429	8,57143	0	0	0	2
0	0	0	77,1429	210	12,8571	1	1	1
0	1	1	240	66,4286	0	1	1	2
0	0	1	107,143	94,2857	0	1	1	2
0	0	0	8,57143	17,1429	0	0	0	2
0	0	0	17,1429	150	0	0	1	2
0	0	0	51,4286	34,2857	0	0	0	2
0	0	0	17,1429	34,2857	0	0	0	1
0	1	1	111,429	171,429	0	1	1	2
0	0	0	128,571	397,143	42,8571	1	1	2
0	0	0	28,5714	17,1429	0	0	0	1
0	0	0	162,857	232,857	0	1	1	2
0	0	0	214,286	58,5714	25,7143	1	1	2
0	0	0	111,429	180	0	1	1	2
0	0	0	25,7143	117,857	0	0	1	2
0	0	0	98,5714	34,2857	0	1	0	2
0	1	1	100,714	30	0	1	0	2
0	0	0	98,5714	77,1429	17,1429	1	1	3
1	0	1	23,5714	180	81,4286	0	1	2
0	1	1	360	25,7143	0	1	0	3
0	0	0	0	38,5714	0	0	0	2
0	0	0	60	188,571	102,857	1	1	2
0	0	0	51,4286	64,2857	25,7143	0	1	2
0	0	0	94,2857	27,8571	0	1	0	2
0	1	1	145,714	17,1429	0	1	0	2
0	0	0	42,8571	12,8571	0	0	0	3
0	0	0	15	222,857	0	0	1	1
0	0	0	17,1429	17,1429	0	0	0	2
0	0	0	20	128,571	8,57143	0	1	2
0	0	0	90	147,143	0	1	1	1
0	0	0	21,4286	0	0	0	0	1
0	0	0	21,4286	167,143	0	0	1	2
0	0	0	34,2857	68,5714	0	0	1	3
0	1	1	154,286	34,2857	0	1	0	1
0	0	0	0	34,2857	0	0	0	1
0	0	0	0	102,857	0	0	1	1
0	1	1	130	120	0	1	1	1
0	0	0	0	137,143	0	0	1	2
1	0	1	85,7143	102,857	128,571	1	1	2
0	0	0	8,57143	42,8571	0	0	0	2
0	0	0	42,8571	60	0	0	1	2
0	0	0	30	120	0	0	1	2
0	0	0	42,8571	51,4286	17,1429	0	1	1
0	0	0	51,4286	19,2857	0	0	0	2
0	1	1	102,857	0	0	1	0	3
0	1	1	60	25,7143	0	1	0	1
0	0	0	16,4286	154,286	8,57143	0	1	2
0	0	0	51,4286	137,143	0	0	1	2

0	0	0	71,4286	120	0	1	1	2
0	0	0	42,8571	68,5714	0	0	1	2
0	0	0	6,42857	50	0	0	0	2
0	1	1	65,7143	51,4286	17,1429	1	1	2
0	0	0	102,857	72,8571	0	1	1	2
0	0	0	12,8571	69,2857	25,7143	0	1	2
0	0	1	111,429	75	0	1	1	1
0	1	1	141,429	10,7143	0	1	0	3
0	0	0	72,8571	30	0	1	0	1
0	0	0	90	84,2857	0	1	1	2
0	0	0	72,8571	120	0	1	1	1
0	0	0	17,1429	8,57143	22,8571	0	0	2
0	0	0	34,2857	32,1429	0	0	0	3
0	0	0	25,7143	25,7143	0	0	0	2
0	0	0	102,857	111,429	8,57143	1	1	2
0	0	0	30	94,2857	0	0	1	1
0	0	0	34,2857	51,4286	0	0	0	2
0	0	0	14,2857	38,5714	0	0	0	2
0	0	0	81,4286	102,857	0	1	1	1
0	0	0	85,7143	85,7143	0	1	1	1
0	0	0	90	47,1429	0	1	0	1
0	0	0	237,857	265,714	0	1	1	1
0	1	1	77,1429	0	0	1	0	3
0	0	0	12,8571	60	0	0	1	2
0	0	0	5,71429	42,8571	15	0	0	3
0	0	0	60	105	102,857	1	1	3
0	0	0	31,4286	30	0	0	0	2
0	0	0	20	85,7143	154,286	0	1	2
0	1	1	240	132,857	0	1	1	2
0	0	0	22,8571	60	0	0	1	2
0	0	0	21,4286	85,7143	0	0	1	2
0	0	0	51,4286	68,5714	0	0	1	2
0	1	1	68,5714	60	0	1	1	1
0	0	0	53,5714	137,143	0	0	1	1
0	1	1	162,857	0	0	1	0	1
0	1	1	60	40,7143	25,7143	1	1	2
0	0	0	171,429	30	8,57143	1	0	1
0	0	0	0	140	0	0	1	3
1	0	1	58,5714	38,5714	60	0	1	2
0	0	0	54,2857	17,1429	0	0	0	3
0	0	1	51,4286	197,143	51,4286	0	1	2
0	0	0	25,7143	51,4286	0	0	0	2
0	0	0	24,2857	77,1429	0	0	1	2
0	0	0	0	0	0	0	0	2
0	0	0	154,286	154,286	17,1429	1	1	1
0	0	0	7,14286	154,286	0	0	1	2
0	0	0	15	222,857	17,1429	0	1	2
0	0	0	0	21,4286	0	0	0	2

0	1	1	60	94,2857	17,1429	1	1	1
0	0	0	14,2857	4,28571	0	0	0	2
0	0	0	40	42,8571	0	0	0	2
0	0	0	8,57143	34,2857	0	0	0	2
0	0	0	4,28571	68,5714	17,1429	0	1	2
0	0	0	197,143	214,286	0	1	1	2
0	0	0	12,8571	14,2857	0	0	0	3
0	0	0	51,4286	0	0	0	0	2
0	0	0	128,571	8,57143	1,42857	1	0	2
0	0	0	50,7143	8,57143	0	0	0	2
0	1	1	74,2857	42,8571	0	1	0	2
0	0	0	8,57143	51,4286	0	0	0	1
0	0	0	40	17,1429	0	0	0	2
0	0	0	42,8571	25,7143	0	0	0	2
0	0	0	17,1429	184,286	0	0	1	2
0	0	0	20	32,8571	12,8571	0	0	2
0	0	0	17,1429	25,7143	0	0	0	2
0	0	0	34,2857	90	0	0	1	2
0	1	1	186,429	40,7143	0	1	0	2
0	0	0	4,28571	21,4286	0	0	0	2
0	0	0	51,4286	102,857	0	0	1	2
0	0	0	101,429	36,4286	0	1	0	2
0	1	1	214,286	188,571	17,1429	1	1	2
0	0	0	0	85,7143	0	0	1	1
0	1	1	60	60	180	1	1	1
0	0	0	0	231,429	0	0	1	1
0	0	0	10	66,4286	0	0	1	2
0	0	0	30	17,1429	5,71429	0	0	1
0	0	0	0	5,71429	0	0	0	2
0	0	0	18,5714	111,429	0	0	1	2
0	1	1	120	34,2857	0	1	0	2
0	0	0	75	25,7143	0	1	0	1
0	0	0	214,286	102,857	0	1	1	2
0	1	1	102,857	17,1429	0	1	0	2
0	0	0	37,1429	25,7143	0	0	0	1
0	0	0	68,5714	154,286	0	1	1	2
0	0	0	21,4286	128,571	51,4286	0	1	2
0	0	0	102,857	68,5714	0	1	1	1
0	0	0	14,2857	30	0	0	0	3
0	0	0	188,571	171,429	0	1	1	1
0	0	0	0	80	0	0	1	1
0	1	1	67,1429	17,1429	0	1	0	2
0	0	0	17,1429	34,2857	0	0	0	2
0	0	0	11,4286	85,7143	0	0	1	2
0	0	0	25,7143	102,857	0	0	1	2
0	0	0	64,2857	28,5714	0	1	0	2
0	0	0	65	74,2857	0	1	1	1
0	0	0	38,5714	8,57143	0	0	0	2

0	0	0	0	25,7143	0	0	0	2
0	1	1	111,429	51,4286	0	1	0	2
0	0	1	37,1429	77,1429	0	0	1	3
0	1	1	105	32,1429	8,57143	1	0	3
0	0	0	68,5714	94,2857	0	1	1	2
0	0	0	51,4286	138,571	0	0	1	3
0	0	0	11,4286	51,4286	0	0	0	2
0	0	0	25,7143	27,1429	17,1429	0	0	3
0	0	0	94,2857	42,8571	0	1	0	2
0	0	0	34,2857	51,4286	8,57143	0	1	2
0	0	0	42,8571	0	0	0	0	2
0	0	0	2,14286	128,571	0	0	1	2
0	0	0	0	7,14286	0	0	0	2
0	0	0	8,57143	231,429	0	0	1	2
0	0	0	64,2857	81,4286	4,28571	1	1	3
0	0	0	20	25,7143	0	0	0	2
0	0	0	21,4286	0	0	0	0	2
0	1	1	162,857	17,1429	0	1	0	2
0	0	0	0	222,857	0	0	1	2
0	0	0	57,1429	25,7143	0	0	0	2
0	1	1	81,4286	72,8571	17,1429	1	1	2
0	0	0	4,28571	51,4286	0	0	0	1
0	0	0	42,8571	25,7143	51,4286	0	1	3
0	0	0	47,1429	51,4286	0	0	0	2
0	0	0	7,14286	180	0	0	1	2
0	0	0	0	240	0	0	1	2
0	1	1	98,5714	102,857	0	1	1	1
0	0	0	274,286	205,714	0	1	1	1
0	0	0	25,7143	27,1429	0	0	0	2
0	0	0	25,7143	55,7143	0	0	0	3
0	0	0	6,42857	34,2857	0	0	0	1
0	0	0	132,857	120	0	1	1	2
0	0	0	17,1429	14,2857	4,28571	0	0	2
0	0	0	14,2857	85,7143	0	0	1	2
0	0	0	85,7143	38,5714	0	1	0	2
0	0	0	30	34,2857	0	0	0	2
0	0	0	30	32,1429	0	0	0	1
0	0	0	132,857	38,5714	0	1	0	2
0	0	0	31,4286	72,8571	0	0	1	2
0	0	0	34,2857	15	32,1429	0	0	3
0	0	0	15	51,4286	0	0	0	2
0	0	0	0	94,2857	25,7143	0	1	2
0	0	0	25,7143	77,1429	0	0	1	1
0	0	0	47,8571	132,857	42,8571	0	1	3
0	0	0	27,8571	77,1429	0	0	1	2
0	0	0	51,4286	102,857	0	0	1	1
0	0	0	17,1429	154,286	0	0	1	2
0	0	0	21,4286	42,8571	25,7143	0	1	2

0	0	0	27,1429	78,5714	0	0	1	2
0	0	0	8,57143	122,857	0	0	1	1
0	0	0	22,8571	64,2857	0	0	1	2
0	0	0	0	205,714	0	0	1	2
0	1	1	67,1429	120	0	1	1	2
0	0	0	0	17,1429	0	0	0	2
0	0	0	34,2857	31,4286	0	0	0	2
0	0	0	58,5714	8,57143	0	0	0	3
0	0	0	35,7143	6,42857	0	0	0	1
0	0	0	60	34,2857	0	1	0	2
0	0	0	37,1429	145,714	25,7143	0	1	1
0	0	0	17,1429	25,7143	0	0	0	1
0	1	1	60	60	20	1	1	3
0	0	1	54,2857	47,1429	0	0	0	1
0	0	0	40	55,7143	0	0	0	2
1	0	1	85,7143	66,4286	32,1429	1	1	3
0	0	0	12,8571	17,1429	0	0	0	1
0	0	0	12,8571	78,5714	0	0	1	2
0	0	0	20	120	154,286	0	1	2
0	0	0	55,7143	34,2857	0	0	0	2
0	0	0	85,7143	42,8571	0	1	0	2
0	0	0	92,1429	23,5714	0	1	0	1
0	0	0	21,4286	150	0	0	1	1
0	0	0	14,2857	51,4286	0	0	0	3
0	0	0	25,7143	42,8571	0	0	0	1
0	1	1	60	222,857	0	1	1	2
0	0	0	34,2857	8,57143	0	0	0	2
0	0	1	62,8571	55,7143	0	1	0	2
0	0	0	0	42,8571	0	0	0	2
0	0	0	40	71,4286	0	0	1	2
0	0	0	162,857	158,571	8,57143	1	1	3
0	0	0	5,71429	17,1429	6,42857	0	0	1
0	1	1	111,429	51,4286	0	1	0	2
0	0	0	21,4286	128,571	0	0	1	2
0	0	0	90	154,286	0	1	1	3
0	0	0	0	17,1429	0	0	0	3
0	0	0	51,4286	51,4286	0	0	0	1
0	0	0	60	90	8,57143	1	1	2
0	0	0	17,1429	77,1429	0	0	1	2
0	0	0	60	17,1429	45	1	1	2
0	0	0	6,85714	34,2857	0	0	0	1
0	1	1	74,2857	25,7143	0	1	0	2
0	0	1	94,2857	60	30	1	1	2
0	0	0	25	62,1429	0	0	1	2
0	0	0	0	32,1429	0	0	0	2
0	1	1	80	257,143	0	1	1	2
0	0	0	34,2857	111,429	0	0	1	2
0	1	1	110	0	0	1	0	1

0	*0*	*0*	*0*	*42,8571*	*34,2857*	*0*	*1*	*2*
0	*0*	*0*	*42,8571*	*34,2857*	*42,8571*	*0*	*1*	*1*
0	*0*	*0*	*120*	*60*	*0*	*1*	*1*	*2*
0	*0*	*0*	*2,85714*	*94,2857*	*34,2857*	*0*	*1*	*2*
0	*0*	*0*	*42,8571*	*205,714*	*0*	*0*	*1*	*2*
1	*0*	*1*	*12,8571*	*81,4286*	*120*	*0*	*1*	*3*
0	*0*	*0*	*169,286*	*25,7143*	*25,7143*	*1*	*0*	*2*
0	*0*	*0*	*4,28571*	*192,857*	*0*	*0*	*1*	*1*
1	*0*	*1*	*60*	*135*	*85,7143*	*1*	*1*	*1*
0	*0*	*0*	*30*	*107,143*	*0*	*0*	*1*	*2*
0	*0*	*0*	*96,4286*	*25,7143*	*0*	*1*	*0*	*2*
0	*0*	*0*	*60*	*107,143*	*0*	*1*	*1*	*2*
0	*0*	*0*	*47,1429*	*34,2857*	*17,1429*	*0*	*0*	*2*
0	*0*	*0*	*0*	*32,1429*	*0*	*0*	*0*	*1*
0	*0*	*0*	*17,8571*	*25,7143*	*0*	*0*	*0*	*2*
0	*0*	*0*	*60*	*25,7143*	*0*	*1*	*0*	*2*
0	*0*	*1*	*42,8571*	*90*	*0*	*0*	*1*	*1*
0	*0*	*0*	*30*	*120*	*0*	*0*	*1*	*2*
0	*0*	*0*	*25,7143*	*51,4286*	*0*	*0*	*0*	*1*
0	*0*	*0*	*55,7143*	*154,286*	*0*	*0*	*1*	*1*
0	*0*	*0*	*45,7143*	*8,57143*	*0*	*0*	*0*	*3*
0	*0*	*0*	*81,4286*	*77,1429*	*0*	*1*	*1*	*2*
0	*1*	*1*	*77,1429*	*0*	*12,8571*	*1*	*0*	*2*
0	*0*	*0*	*8,57143*	*51,4286*	*0*	*0*	*0*	*1*
0	*0*	*0*	*21,4286*	*51,4286*	*0*	*0*	*0*	*3*
0	*0*	*0*	*94,2857*	*111,429*	*0*	*1*	*1*	*2*
0	*0*	*0*	*17,1429*	*102,857*	*0*	*0*	*1*	*2*
0	*0*	*0*	*17,1429*	*42,8571*	*0*	*0*	*0*	*2*
0	*0*	*0*	*0*	*51,4286*	*0*	*0*	*0*	*2*
0	*0*	*0*	*42,8571*	*51,4286*	*0*	*0*	*0*	*2*
0	*0*	*0*	*42,8571*	*42,8571*	*0*	*0*	*0*	*2*
0	*0*	*0*	*60*	*51,4286*	*0*	*1*	*0*	*2*
0	*0*	*0*	*60*	*102,857*	*0*	*1*	*1*	*3*
0	*0*	*0*	*41,4286*	*68,5714*	*0*	*0*	*1*	*1*
0	*0*	*0*	*154,286*	*375*	*154,286*	*1*	*1*	*2*
0	*0*	*0*	*158,571*	*171,429*	*34,2857*	*1*	*1*	*2*
0	*1*	*1*	*128,571*	*121,429*	*0*	*1*	*1*	*2*
0	*0*	*0*	*0*	*154,286*	*25,7143*	*0*	*1*	*1*
0	*0*	*0*	*150*	*11,4286*	*0*	*1*	*0*	*1*
0	*0*	*0*	*142,857*	*94,2857*	*0*	*1*	*1*	*2*
0	*0*	*0*	*17,1429*	*25,7143*	*17,1429*	*0*	*0*	*1*
0	*0*	*0*	*47,1429*	*20*	*0*	*0*	*0*	*2*
0	*0*	*0*	*5,71429*	*51,4286*	*0*	*0*	*0*	*2*
0	*0*	*0*	*72,8571*	*77,1429*	*0*	*1*	*1*	*2*
0	*0*	*0*	*21,4286*	*0*	*0*	*0*	*0*	*1*
0	*0*	*0*	*11,4286*	*34,2857*	*0*	*0*	*0*	*2*
0	*0*	*0*	*0*	*145,714*	*0*	*0*	*1*	*2*
0	*0*	*1*	*64,2857*	*68,5714*	*0*	*1*	*1*	*1*

0	0	0	137,143	150	25,7143	1	1	2
0	0	0	25,7143	28,5714	0	0	0	1
0	0	0	7,14286	162,857	0	0	1	2
0	0	0	34,2857	60	0	0	1	2
0	1	1	134,286	252,857	0	1	1	2
0	0	0	67,1429	17,1429	0	1	0	2
0	0	0	0	124,286	0	0	1	2
0	0	0	17,1429	85,7143	0	0	1	3
0	0	0	0	53,5714	0	0	0	2
0	0	0	4,28571	132,857	8,57143	0	1	2
0	0	0	34,2857	64,2857	0	0	1	2
0	0	0	115,714	207,143	25,7143	1	1	2
0	0	0	21,4286	192,857	0	0	1	2
0	1	1	60	180	0	1	1	2
0	0	0	60	25,7143	0	1	0	1
0	0	0	8,57143	2,85714	0	0	0	2
0	0	0	60	154,286	17,1429	1	1	2
0	0	0	0	25,7143	0	0	0	2
0	1	1	205,714	30	17,1429	1	0	2
0	0	0	45,7143	8,57143	0	0	0	2
0	0	0	47,1429	54,2857	0	0	0	1
0	0	0	17,1429	27,8571	0	0	0	3
0	0	0	40	85,7143	0	0	1	2
0	0	0	0	87,1429	0	0	1	1
0	0	0	17,1429	34,2857	0	0	0	1
0	0	0	51,4286	180	0	0	1	2
0	0	0	102,857	222,857	0	1	1	2
0	0	0	47,1429	34,2857	0	0	0	2
0	0	0	10,7143	46,4286	0	0	0	2
0	0	0	0	81,4286	0	0	1	1
0	0	0	0	42,8571	0	0	0	2
0	0	0	51,4286	128,571	0	0	1	2
0	1	1	128,571	211,429	120	1	1	1
0	0	1	145,714	102,857	0	1	1	1
0	0	0	40,7143	77,1429	0	0	1	1
0	0	0	7,14286	257,143	0	0	1	2
0	0	0	155,714	64,2857	0	1	1	1
0	0	1	77,1429	68,5714	0	1	1	2
0	0	0	14,2857	0	0	0	0	2
0	0	0	0	50	0	0	0	1
0	0	0	25,7143	51,4286	0	0	0	2
0	0	0	20	21,4286	0	0	0	1
0	0	0	102,857	85,7143	51,4286	1	1	2
0	0	0	47,1429	102,857	0	0	1	2
0	0	0	38,5714	47,1429	42,8571	0	1	2
0	0	1	42,8571	0	17,1429	0	0	2
0	0	0	0	68,5714	0	0	1	2
0	0	0	72,8571	68,5714	0	1	1	2

0	*0*	*0*	*68,5714*	*51,4286*	*0*	*1*	*0*	*2*
0	*0*	*1*	*77,1429*	*25,7143*	*8,57143*	*1*	*0*	*2*
0	*0*	*0*	*30*	*60*	*0*	*0*	*1*	*2*
0	*0*	*0*	*0*	*45,7143*	*0*	*0*	*0*	*1*
0	*0*	*0*	*184,286*	*231,429*	*0*	*1*	*1*	*2*
0	*0*	*0*	*21,4286*	*102,857*	*0*	*0*	*1*	*2*
0	*0*	*0*	*8,57143*	*0*	*0*	*0*	*0*	*2*
0	*0*	*0*	*32,1429*	*54,2857*	*0*	*0*	*0*	*2*
0	*0*	*0*	*15*	*17,1429*	*0*	*0*	*0*	*2*
0	*0*	*0*	*17,1429*	*30*	*0*	*0*	*0*	*2*
0	*0*	*0*	*49,2857*	*30*	*17,1429*	*0*	*0*	*2*
0	*0*	*0*	*150*	*42,8571*	*0*	*1*	*0*	*1*
0	*0*	*0*	*34,2857*	*75*	*0*	*0*	*1*	*2*
0	*0*	*0*	*60*	*25,7143*	*0*	*1*	*0*	*2*
0	*0*	*0*	*6,42857*	*160*	*0*	*0*	*1*	*2*
0	*0*	*0*	*25,7143*	*85,7143*	*0*	*0*	*1*	*3*
0	*0*	*0*	*2,85714*	*38,5714*	*30*	*0*	*1*	*2*
0	*0*	*0*	*4,28571*	*64,2857*	*0*	*0*	*1*	*2*
0	*0*	*0*	*0*	*192,857*	*42,8571*	*0*	*1*	*1*
0	*0*	*0*	*97,1429*	*68,5714*	*0*	*1*	*1*	*2*
0	*0*	*0*	*145,714*	*34,2857*	*0*	*1*	*0*	*2*
0	*0*	*0*	*61,4286*	*128,571*	*0*	*1*	*1*	*2*
0	*0*	*0*	*167,143*	*282,857*	*0*	*1*	*1*	*2*
0	*0*	*0*	*28,5714*	*77,1429*	*6,42857*	*0*	*1*	*2*
0	*0*	*0*	*5,71429*	*30*	*0*	*0*	*0*	*1*
0	*0*	*0*	*60*	*111,429*	*0*	*1*	*1*	*2*
0	*0*	*0*	*137,143*	*180*	*17,1429*	*1*	*1*	*2*
0	*1*	*1*	*134,286*	*25,7143*	*0*	*1*	*0*	*2*
0	*0*	*0*	*60,7143*	*25,7143*	*0*	*1*	*0*	*2*
0	*0*	*0*	*0*	*41,4286*	*0*	*0*	*0*	*2*
0	*0*	*0*	*8,57143*	*42,8571*	*0*	*0*	*0*	*1*
0	*0*	*0*	*0*	*25,7143*	*51,4286*	*0*	*1*	*2*
0	*0*	*0*	*20*	*145,714*	*0*	*0*	*1*	*2*
0	*0*	*0*	*34,2857*	*55,7143*	*0*	*0*	*0*	*3*
0	*0*	*0*	*101,429*	*21,4286*	*0*	*1*	*0*	*1*
0	*0*	*0*	*42,8571*	*197,143*	*0*	*0*	*1*	*2*
0	*0*	*0*	*51,4286*	*51,4286*	*0*	*0*	*0*	*2*
0	*0*	*0*	*60*	*117,143*	*0*	*1*	*1*	*2*
0	*0*	*0*	*0*	*244,286*	*0*	*0*	*1*	*2*
0	*0*	*0*	*30*	*25,7143*	*0*	*0*	*0*	*2*
0	*0*	*0*	*17,1429*	*42,8571*	*0*	*0*	*0*	*2*
0	*0*	*0*	*30*	*34,2857*	*0*	*0*	*0*	*2*
0	*0*	*0*	*150*	*120*	*0*	*1*	*1*	*1*
0	*0*	*0*	*31,4286*	*188,571*	*17,1429*	*0*	*1*	*2*
0	*0*	*0*	*92,8571*	*5,71429*	*0*	*1*	*0*	*2*
0	*0*	*0*	*0*	*60*	*0*	*0*	*1*	*2*
0	*0*	*0*	*0*	*17,1429*	*0*	*0*	*0*	*2*
0	*0*	*0*	*57,1429*	*51,4286*	*0*	*0*	*0*	*2*

0	0	0	98,5714	154,286	0	1	1	2
0	0	0	11,4286	51,4286	0	0	0	1
0	0	1	60	74,2857	0	1	1	2
0	0	0	68,5714	17,1429	0	1	0	1
0	0	0	42,8571	274,286	0	0	1	2
0	0	0	47,1429	45	0	0	0	2
0	1	1	70	8,57143	0	1	0	2
0	0	0	7,14286	158,571	0	0	1	2
0	0	0	51,4286	17,1429	0	0	0	2
0	0	0	25,7143	68,5714	0	0	1	2
0	0	0	25,7143	17,1429	0	0	0	2
0	0	0	7,14286	227,143	0	0	1	2
0	1	1	300	75	0	1	1	2
0	0	0	5,71429	140	0	0	1	2
0	0	0	21,4286	32,1429	0	0	0	2
0	0	0	51,4286	98,5714	0	0	1	2
0	0	0	8,57143	8,57143	0	0	0	3
0	0	0	77,1429	17,1429	0	1	0	2
0	0	0	0	102,857	0	0	1	1
0	0	0	14,2857	17,1429	0	0	0	2
0	0	0	120	304,286	0	1	1	2
0	0	0	51,4286	51,4286	0	0	0	2
0	0	0	115,714	42,8571	0	1	0	2
0	0	0	0	60	0	0	1	2
0	0	0	28,5714	128,571	34,2857	0	1	2
0	0	0	100	141,429	0	1	1	2
0	0	0	17,8571	137,143	0	0	1	2
0	0	0	37,1429	131,429	0	0	1	1
0	0	0	75	68,5714	128,571	1	1	1
0	0	0	0	120	8,57143	0	1	1
0	0	0	4,28571	17,1429	0	0	0	2
0	0	0	17,1429	51,4286	0	0	0	2
0	0	0	17,1429	154,286	0	0	1	1
0	0	0	55,7143	38,5714	0	0	0	2
0	0	0	42,8571	145,714	51,4286	0	1	2
0	0	0	32,8571	102,857	0	0	1	2
0	0	0	11,4286	55,7143	0	0	0	2
0	0	0	171,429	42,8571	0	1	0	1
0	0	0	140	180	0	1	1	2
0	0	0	85,7143	128,571	0	1	1	2
0	1	1	211,429	42,8571	0	1	0	1
0	1	1	300	120	0	1	1	2
0	0	0	0	95	0	0	1	3
0	0	0	17,1429	25,7143	0	0	0	1
0	0	0	139,286	171,429	0	1	1	1
0	0	0	51,4286	47,1429	0	0	0	2
0	0	0	21,4286	128,571	0	0	1	2
0	0	0	77,1429	60	0	1	1	1

0	0	0	141,429	90	0	1	1	2
0	0	0	47,1429	25,7143	0	0	0	1
0	0	0	51,4286	42,8571	0	0	0	2
0	1	1	192,857	60	0	1	1	2
0	0	0	8,57143	51,4286	0	0	0	2
0	0	0	68,5714	25,7143	4,28571	1	0	2
0	0	0	34,2857	102,857	0	0	1	2
0	0	0	28,5714	0	0	0	0	1
0	0	0	150	0	0	1	0	1
0	0	0	8,57143	42,8571	0	0	0	3
0	1	1	132,857	30	0	1	0	1
0	0	0	8,57143	32,1429	0	0	0	2
0	0	0	19,2857	17,1429	2,85714	0	0	2
0	1	1	147,857	25,7143	0	1	0	1
0	0	0	47,8571	12,8571	0	0	0	1
0	0	0	68,5714	192,857	0	1	1	1
0	0	0	42,8571	162,857	0	0	1	2
0	0	0	77,1429	120	102,857	1	1	2
0	0	0	120	60	0	1	1	2
0	1	1	75	38,5714	0	1	0	3
0	0	0	17,1429	52,8571	0	0	0	2
0	0	0	35,7143	17,1429	0	0	0	2
0	0	0	25,7143	140	0	0	1	2
0	0	0	23,5714	37,1429	0	0	0	2
0	0	0	5,71429	68,5714	0	0	1	1
0	0	1	51,4286	15,7143	12,8571	0	0	2
0	0	0	47,1429	120	0	0	1	1
0	0	0	22,8571	94,2857	0	0	1	2
0	0	0	21,4286	51,4286	0	0	0	2
0	0	0	4,28571	36,4286	0	0	0	2
0	0	0	28,5714	51,4286	0	0	0	2
0	0	0	30	30	0	0	0	1
0	0	0	51,4286	4,28571	0	0	0	1
0	0	0	20	68,5714	0	0	1	3
0	1	1	68,5714	128,571	0	1	1	2
0	0	0	4,28571	51,4286	0	0	0	2
0	0	0	60	68,5714	0	1	1	2
0	0	0	162,857	21,4286	0	1	0	2
0	0	0	80	85,7143	0	1	1	2
0	0	1	102,857	77,1429	0	1	1	2
0	0	0	5	57,1429	0	0	0	3
0	0	0	38,5714	90	17,1429	0	1	1
0	0	0	145,714	137,143	0	1	1	1
0	0	0	51,4286	34,2857	0	0	0	2
0	0	0	8,57143	17,1429	0	0	0	3
0	0	0	14,2857	20	0	0	0	2
0	0	0	32,8571	185,714	0	0	1	1
0	0	0	31,4286	51,4286	0	0	0	2

0	0	0	0	107,143	17,1429	0	1	2
0	0	0	132,857	17,1429	0	1	0	2
0	0	0	65,7143	137,143	0	1	1	2
0	0	0	14,2857	51,4286	0	0	0	2
0	0	0	55,7143	4,28571	0	0	0	2
0	0	1	0	137,143	0	0	1	1
0	1	1	134,286	0	0	1	0	2
0	0	0	60	57,8571	0	1	0	2
1	0	1	64,2857	120	0	1	1	2
0	0	0	37,1429	85,7143	51,4286	0	1	3
0	0	0	4,28571	111,429	25,7143	0	1	2
0	0	0	25,7143	175,714	0	0	1	2
0	0	0	0	35,7143	0	0	0	1
0	0	0	120	300	180	1	1	2
0	0	0	6,42857	0	4,28571	0	0	1
0	0	0	0	126,429	0	0	1	3
0	0	0	68,5714	41,4286	30	1	1	1
0	0	0	51,4286	68,5714	0	0	1	2
0	1	1	80	154,286	25,7143	1	1	3
0	0	0	0	25,7143	0	0	0	1
0	0	0	51,4286	38,5714	0	0	0	2
0	1	1	60	85,7143	0	1	1	2
1	0	1	128,571	0	77,1429	1	1	3
0	0	0	51,4286	51,4286	0	0	0	2
0	0	0	61,4286	120	21,4286	1	1	2
0	0	1	102,857	47,1429	0	1	0	2
1	0	1	37,1429	0	85,7143	0	1	2
0	0	0	102,857	45,7143	0	1	0	1
0	0	0	52,8571	55,7143	6,42857	0	1	1
0	0	0	0	4,28571	0	0	0	1
0	0	0	42,8571	0	0	0	0	1
0	0	0	102,857	60	0	1	1	1
0	0	0	34,2857	137,143	12,8571	0	1	1
0	0	0	17,1429	8,57143	0	0	0	2
0	0	0	0	46,4286	0	0	0	1
0	0	0	30	34,2857	0	0	0	3
0	0	0	27,1429	60	0	0	1	2
0	1	1	60	22,1429	11,4286	1	0	2
0	0	0	15	25,7143	0	0	0	2
1	0	1	34,2857	145,714	0	0	1	2
0	0	0	25,7143	25,7143	0	0	0	2
0	0	0	85,7143	128,571	60	1	1	2
0	0	0	30	8,57143	0	0	0	2
0	0	0	64,2857	85,7143	0	1	1	2
0	0	0	0	54,2857	0	0	0	2
0	0	0	10,7143	51,4286	0	0	0	2
0	0	0	32,1429	17,1429	0	0	0	2
0	1	1	68,5714	17,1429	0	1	0	2

0	0	0	18,5714	77,1429	8,57143	0	1	2
0	0	0	25,7143	34,2857	0	0	0	1
0	1	1	102,857	11,4286	0	1	0	1
0	1	1	85,7143	30	0	1	0	1
0	0	0	28,5714	257,143	0	0	1	2
0	0	0	85,7143	32,1429	0	1	0	1
0	0	0	14,2857	34,2857	2,85714	0	0	1
0	0	0	137,143	21,4286	0	1	0	2
0	0	0	44,2857	188,571	0	0	1	2
0	0	0	60	52,8571	0	1	0	2
0	1	1	102,857	61,4286	25,7143	1	1	2
0	0	0	17,1429	17,1429	0	0	0	2
0	0	0	17,1429	45	0	0	0	3
0	0	0	34,2857	128,571	25,7143	0	1	3
0	1	1	128,571	22,8571	0	1	0	1
0	0	0	8,57143	40	0	0	0	2
0	0	0	85,7143	214,286	0	1	1	2
0	0	0	0	0	0	0	0	2
0	0	0	0	0	0	0	0	1
0	0	0	14,2857	55,7143	0	0	0	2
0	0	0	21,4286	68,5714	0	0	1	2
0	0	0	17,1429	51,4286	0	0	0	1
0	0	0	12,8571	36,4286	0	0	0	3
0	0	0	28,5714	0	0	0	0	2
0	0	0	60	81,4286	85,7143	1	1	2
0	0	1	41,4286	70,7143	47,1429	0	1	2
0	0	0	64,2857	5,71429	0	1	0	2
0	0	0	17,1429	34,2857	0	0	0	1
0	0	0	34,2857	222,857	0	0	1	2
0	0	1	34,2857	51,4286	0	0	0	2
0	0	0	64,2857	17,1429	0	1	0	2
0	0	0	168,571	248,571	25,7143	1	1	1
0	0	0	0	51,4286	8,57143	0	1	2
0	0	0	19,4286	85,7143	0	0	1	2
0	0	0	1,42857	210	8,57143	0	1	2
0	0	0	40	25,7143	0	0	0	1
0	0	0	0	42,8571	34,2857	0	1	2
0	0	0	15	54,2857	6,42857	0	1	2
0	0	0	0	0	0	0	0	2
0	0	0	42,8571	71,4286	0	0	1	2
0	0	0	30	231,429	0	0	1	1
0	0	0	21,4286	22,8571	0	0	0	1
0	0	0	111,429	17,1429	0	1	0	2
0	0	0	81,4286	25,7143	0	1	0	2
0	1	1	131,429	30	0	1	0	2
0	0	0	45,7143	128,571	0	0	1	2
0	0	1	68,5714	23,5714	0	1	0	2
0	0	0	77,1429	205,714	0	1	1	3

0	0	1	71,4286	37,1429	0	1	0	1
0	1	1	120	120	90	1	1	2
0	0	0	42,8571	34,2857	0	0	0	2
0	0	1	147,857	167,143	0	1	1	2
0	0	0	0	79,2857	0	0	1	2
0	0	0	208,571	214,286	8,57143	1	1	3
0	0	0	85,7143	34,2857	0	1	0	2
0	0	0	7,14286	85,7143	0	0	1	3
0	0	0	47,1429	0	0	0	0	2
0	0	0	0	17,1429	0	0	0	1
0	0	0	39,2857	25,7143	0	0	0	1
0	0	0	30	197,143	0	0	1	2
0	1	1	90	17,1429	0	1	0	2
0	0	0	60	184,286	0	1	1	2
0	0	0	38,5714	4,28571	0	0	0	1
0	1	1	77,1429	17,1429	12,8571	1	0	1
0	0	0	68,5714	28,5714	0	1	0	1
0	0	0	8,57143	81,4286	0	0	1	2
0	1	1	60	80	0	1	1	1
0	0	0	78,5714	47,1429	0	1	0	3
0	0	0	20	25,7143	0	0	0	1
0	0	0	2,85714	120	0	0	1	1
0	1	1	360	25,7143	17,1429	1	0	3
0	0	0	74,2857	50	0	1	0	2
0	0	0	35,7143	35,7143	0	0	0	1
0	1	1	85,7143	34,2857	0	1	0	1
0	0	0	57,1429	25,7143	0	0	0	2
0	1	1	96,4286	85,7143	0	1	1	2
0	0	0	246,429	42,8571	0	1	0	1
0	0	0	192,857	271,429	0	1	1	2
0	0	0	25,7143	25,7143	0	0	0	2
0	0	0	28,5714	60	17,1429	0	1	2
0	0	0	34,2857	171,429	0	0	1	1
0	0	0	21,4286	49,2857	0	0	0	1
0	0	0	145,714	64,2857	0	1	1	1
0	0	0	62,8571	4,28571	0	1	0	2
0	0	0	31,4286	0	0	0	0	2
0	0	0	28,5714	0	0	0	0	2
0	1	1	300	60	0	1	1	2
0	0	0	0	8,57143	0	0	0	1
0	1	1	128,571	34,2857	0	1	0	1
0	0	0	5,71429	0	0	0	0	2
0	0	0	7,14286	38,5714	0	0	0	2
0	0	0	34,2857	34,2857	34,2857	0	1	2
0	0	0	17,1429	137,143	0	0	1	2
0	0	0	15	12,8571	0	0	0	1
0	0	0	0	271,429	60	0	1	2
0	0	0	51,4286	42,8571	0	0	0	2

0	*0*	*0*	*0*	*8,57143*	*0*	*0*	*0*	*2*
0	*1*	*1*	*94,2857*	*60*	*0*	*1*	*1*	*2*
0	*0*	*0*	*175,714*	*222,857*	*17,1429*	*1*	*1*	*2*
0	*0*	*0*	*12,8571*	*205,714*	*0*	*0*	*1*	*2*
0	*1*	*1*	*135*	*12,8571*	*0*	*1*	*0*	*2*
0	*0*	*0*	*85,7143*	*85,7143*	*0*	*1*	*1*	*2*
0	*0*	*0*	*30*	*68,5714*	*17,1429*	*0*	*1*	*2*
0	*0*	*0*	*45*	*68,5714*	*17,1429*	*0*	*1*	*2*
0	*0*	*0*	*17,1429*	*42,8571*	*0*	*0*	*0*	*2*
0	*0*	*0*	*32,8571*	*60*	*51,4286*	*0*	*1*	*1*
0	*1*	*1*	*60*	*25,7143*	*0*	*1*	*0*	*2*
0	*0*	*0*	*8,57143*	*17,1429*	*0*	*0*	*0*	*2*
0	*1*	*1*	*71,1429*	*12,8571*	*0*	*1*	*0*	*1*
0	*0*	*0*	*30*	*20*	*0*	*0*	*0*	*2*
0	*1*	*1*	*68,5714*	*17,1429*	*0*	*1*	*0*	*2*
0	*0*	*0*	*20*	*188,571*	*0*	*0*	*1*	*1*
0	*0*	*0*	*2,85714*	*17,1429*	*60*	*0*	*1*	*2*
0	*0*	*0*	*70,7143*	*8,57143*	*0*	*1*	*0*	*2*
0	*0*	*0*	*132,857*	*208,571*	*0*	*1*	*1*	*1*
0	*0*	*0*	*34,2857*	*32,1429*	*4,28571*	*0*	*0*	*2*
0	*0*	*0*	*48,5714*	*197,143*	*0*	*0*	*1*	*1*
0	*0*	*1*	*31,4286*	*94,2857*	*0*	*0*	*1*	*2*
0	*0*	*0*	*64,2857*	*32,1429*	*0*	*1*	*0*	*2*
0	*1*	*1*	*150*	*21,4286*	*0*	*1*	*0*	*2*
0	*0*	*0*	*60*	*34,2857*	*0*	*1*	*0*	*2*
0	*0*	*0*	*27,1429*	*68,5714*	*0*	*0*	*1*	*2*
0	*0*	*0*	*30*	*120*	*34,2857*	*0*	*1*	*1*
0	*0*	*0*	*17,1429*	*102,857*	*34,2857*	*0*	*1*	*2*
0	*0*	*0*	*102,857*	*51,4286*	*0*	*1*	*0*	*2*
0	*0*	*0*	*35,7143*	*68,5714*	*0*	*0*	*1*	*2*
0	*0*	*0*	*145,714*	*77,1429*	*0*	*1*	*1*	*2*
0	*0*	*0*	*2,85714*	*49,2857*	*0*	*0*	*0*	*2*
0	*0*	*0*	*17,1429*	*51,4286*	*0*	*0*	*0*	*2*
0	*0*	*0*	*80*	*42,8571*	*0*	*1*	*0*	*2*
0	*0*	*0*	*0*	*201,429*	*0*	*0*	*1*	*2*
0	*0*	*0*	*17,1429*	*38,5714*	*0*	*0*	*0*	*2*
0	*0*	*0*	*58,5714*	*85,7143*	*0*	*0*	*1*	*2*
0	*0*	*0*	*128,571*	*8,57143*	*0*	*1*	*0*	*2*
0	*0*	*0*	*17,1429*	*102,857*	*28,5714*	*0*	*1*	*2*
0	*0*	*0*	*0*	*0*	*0*	*0*	*0*	*2*
0	*0*	*0*	*30*	*72,8571*	*0*	*0*	*1*	*1*
0	*0*	*0*	*0*	*34,2857*	*0*	*0*	*0*	*3*
0	*0*	*0*	*185,714*	*28,5714*	*0*	*1*	*0*	*2*
0	*0*	*0*	*42,8571*	*0*	*0*	*0*	*0*	*3*
0	*0*	*0*	*120*	*25,7143*	*8,57143*	*1*	*0*	*1*
0	*0*	*0*	*14,2857*	*42,8571*	*0*	*0*	*0*	*2*
0	*0*	*0*	*45*	*12,8571*	*0*	*0*	*0*	*2*
0	*1*	*1*	*180*	*60*	*0*	*1*	*1*	*1*

0	0	0	38,5714	122,857	0	0	1	2
0	0	0	47,1429	205,714	0	0	1	2
0	0	0	20,7143	150	0	0	1	2
0	0	0	62,1429	42,8571	0	1	0	2
0	0	0	308,571	40,7143	0	1	0	1
0	0	0	117,143	171,429	0	1	1	1
0	0	0	0	155,714	0	0	1	2
0	0	0	134,286	25,7143	0	1	0	2
0	1	1	120	42,8571	0	1	0	2
0	0	0	15,7143	5,71429	0	0	0	2
0	0	0	121,429	55,7143	0	1	0	2
0	0	0	34,2857	22,8571	51,4286	0	1	2
0	1	1	120	38,5714	34,2857	1	1	2
0	0	0	0	175,714	0	0	1	1
0	0	0	34,2857	51,4286	0	0	0	2
0	0	0	35	25,7143	0	0	0	1
0	1	1	77,1429	38,5714	0	1	0	1
0	0	0	139,286	15,7143	0	1	0	1
0	0	0	158,571	115,714	34,2857	1	1	1
0	0	0	38,5714	17,1429	0	0	0	2
0	1	1	128,571	77,1429	102,857	1	1	2
0	1	1	90	51,4286	8,57143	1	1	2
0	0	0	85,7143	0	0	1	0	1
0	0	0	90,7143	0	0	1	0	1
0	0	0	42,8571	23,5714	0	0	0	2
0	0	0	17,1429	38,5714	0	0	0	2
0	0	0	195,714	128,571	25,7143	1	1	1
0	0	0	5,71429	27,8571	25,7143	0	0	1
0	0	0	0	8,57143	0	0	0	1
0	0	0	77,1429	42,8571	0	1	0	2
0	1	1	131,429	17,1429	0	1	0	2
0	0	0	60	25,7143	0	1	0	3
0	1	1	127,143	5,71429	0	1	0	1
0	0	0	38,5714	102,857	0	0	1	2
0	0	1	58,5714	22,8571	34,2857	0	0	2
0	0	0	8,57143	102,857	0	0	1	1
0	1	1	60	154,286	0	1	1	2
0	0	0	107,143	137,143	0	1	1	2
0	0	0	28,5714	8,57143	0	0	0	2
0	0	0	12,8571	55,7143	0	0	0	1
0	1	1	145,714	34,2857	0	1	0	2
0	0	0	7,14286	35,7143	0	0	0	1
0	1	1	162,857	180	0	1	1	2
0	0	0	185,714	180	25,7143	1	1	2
0	0	0	31,4286	49,2857	14,2857	0	1	2
0	0	0	0	154,286	120	0	1	2
0	0	0	28,5714	85,7143	0	0	1	2
0	0	0	42,8571	77,1429	0	0	1	2

0	0	0	171,429	53,5714	0	1	0	2
0	0	0	0	79,2857	0	0	1	2
0	0	0	34,2857	162,857	0	0	1	2
0	1	1	102,857	205,714	0	1	1	2
0	0	0	15	77,1429	0	0	1	2
0	0	0	60	308,571	0	1	1	2
0	0	0	0	97,1429	0	0	1	2
0	0	0	188,571	214,286	85,7143	1	1	2
0	1	1	102,857	40,7143	128,571	1	1	3
0	0	0	12,8571	132,857	0	0	1	2
0	0	0	14,2857	85,7143	0	0	1	2
0	0	0	21,4286	60	0	0	1	2
0	1	1	240	120	17,1429	1	1	1
0	0	1	202,857	180	0	1	1	2
0	0	0	184,286	141,429	0	1	1	2
1	0	1	128,571	98,5714	51,4286	1	1	2
0	0	0	60	51,4286	0	1	0	1
0	0	0	34,2857	51,4286	0	0	0	1
0	1	1	60	8,57143	0	1	0	2
0	0	0	102,857	205,714	0	1	1	2
0	0	0	5,71429	17,1429	0	0	0	2
0	0	0	51,4286	60	0	0	1	2
0	0	0	0	85,7143	0	0	1	2
0	0	0	17,1429	77,1429	0	0	1	1
0	0	0	56,4286	8,57143	0	0	0	1
0	0	0	8,57143	34,2857	0	0	0	2
0	0	0	15	4,28571	15	0	0	2
0	0	0	21,4286	87,1429	0	0	1	1
0	0	0	4,28571	265,714	0	0	1	2
0	0	0	8,57143	51,4286	0	0	0	2
0	0	0	10,7143	0	0	0	0	2
0	0	0	188,571	248,571	0	1	1	3
0	0	0	0	17,1429	4,28571	0	0	1
0	0	0	55,7143	51,4286	0	0	0	2
0	0	0	8,57143	85,7143	0	0	1	1
0	0	0	137,143	34,2857	0	1	0	1
0	0	0	25,7143	42,8571	0	0	0	2
0	0	0	135,714	25,7143	0	1	0	2
0	0	0	44,2857	8,57143	8,57143	0	0	1
0	0	0	38,5714	42,8571	0	0	0	2
0	1	1	240	51,4286	0	1	0	2
0	0	0	8,57143	77,1429	0	0	1	2
0	0	0	14,2857	30	0	0	0	2
0	0	0	4,28571	50	45	0	1	2
0	0	0	171,429	68,5714	0	1	1	2
0	0	0	77,1429	34,2857	0	1	0	2
0	0	0	21,4286	4,28571	0	0	0	2
0	1	1	145,714	25,7143	0	1	0	3

0	0	0	0	68,5714	12,8571	0	1	1
0	0	0	8,57143	128,571	0	0	1	3
0	0	0	22,8571	20	0	0	0	1
0	1	1	140	85,7143	25,7143	1	1	3
0	0	0	25,7143	42,8571	0	0	0	1
0	0	0	85,7143	180	0	1	1	1
0	0	0	100	64,2857	0	1	1	2
0	0	0	162,857	42,8571	0	1	0	1
0	0	0	51,4286	34,2857	0	0	0	2
0	0	0	158,571	77,1429	0	1	1	2
0	0	1	50	20	0	0	0	2
0	0	0	27,8571	47,1429	0	0	0	1
0	0	0	4,28571	68,5714	0	0	1	1
0	0	0	214,286	51,4286	0	1	0	1
0	0	0	25,7143	53,5714	8,57143	0	1	1
0	0	0	17,1429	25,7143	0	0	0	1
0	0	0	128,571	54,2857	8,57143	1	1	1
0	0	0	12,8571	171,429	8,57143	0	1	2
0	0	0	135,714	154,286	8,57143	1	1	2
0	0	0	98,5714	85,7143	0	1	1	2
0	0	0	137,143	205,714	0	1	1	1
0	0	0	15	154,286	0	0	1	2
0	0	0	94,2857	135	0	1	1	2
0	0	0	51,4286	8,57143	0	0	0	1
0	0	0	34,2857	162,857	0	0	1	2
0	0	1	4,28571	85,7143	0	0	1	2
0	0	0	0	17,1429	0	0	0	1
0	0	0	25,7143	17,1429	0	0	0	2
0	0	0	42,8571	17,1429	0	0	0	2
0	0	0	61,4286	100,714	0	1	1	2
0	0	0	17,1429	77,1429	0	0	1	3
0	0	0	0	37,1429	120	0	1	2
0	0	0	0	142,857	0	0	1	2
0	0	0	111,429	181,714	0	1	1	2
0	0	0	0	8,57143	0	0	0	1
0	0	0	60	180	0	1	1	1
0	0	1	57,1429	42,8571	51,4286	0	1	3
0	0	0	8,57143	17,1429	0	0	0	2
0	0	0	25,7143	12,8571	0	0	0	2
0	0	0	42,8571	60	25,7143	0	1	2
0	0	0	4,28571	34,2857	8,57143	0	0	1
0	0	0	205,714	111,429	0	1	1	2
0	0	0	128,571	158,571	25,7143	1	1	2
0	0	0	120	102,857	0	1	1	2
0	0	0	8,57143	47,1429	0	0	0	2
0	0	0	10,7143	77,1429	0	0	1	2
0	0	1	222,857	188,571	17,1429	1	1	2
0	0	0	102,857	38,5714	0	1	0	2

0	0	0	34,2857	77,1429	0	0	1	2
0	0	0	25,7143	30	0	0	0	2
0	0	0	17,1429	42,8571	0	0	0	2
0	0	0	85,7143	120	0	1	1	2
0	0	0	0	77,1429	34,2857	0	1	2
0	0	0	51,4286	48,5714	0	0	0	1
0	0	0	37,1429	12,8571	4,28571	0	0	2
0	0	0	8,57143	68,5714	0	0	1	2
0	0	0	51,4286	11,4286	0	0	0	2
0	1	1	120	222,857	0	1	1	2
0	0	0	0	257,143	0	0	1	1
0	0	0	8,57143	268,571	0	0	1	1
0	0	0	34,2857	32,8571	0	0	0	2
0	0	0	137,143	98,5714	60	1	1	1
0	0	0	92,8571	111,429	0	1	1	2
0	0	0	19,2857	102,857	0	0	1	2
0	1	1	60	228,571	0	1	1	2
0	0	0	20	25,7143	28,5714	0	0	2
0	0	0	60	95,7143	25,7143	1	1	2
0	0	0	45,7143	25,7143	0	0	0	2
0	0	0	38,5714	17,1429	0	0	0	1
0	0	0	0	150	0	0	1	2
0	1	1	205,714	102,857	0	1	1	2
0	0	0	37,1429	25,7143	0	0	0	2
0	0	0	0	188,571	0	0	1	2
0	0	0	0	360	0	0	1	2
0	1	1	60	77,1429	34,2857	1	1	2
0	1	1	110	68,5714	51,4286	1	1	2
0	0	0	24,2857	4,28571	0	0	0	2
0	0	0	47,1429	92,8571	17,1429	0	1	2
0	1	1	240	57,8571	0	1	0	2
0	0	0	32,8571	30	0	0	0	2
0	0	0	14,2857	22,8571	0	0	0	1
0	0	0	128,571	25,7143	0	1	0	1
0	0	0	28,5714	62,1429	0	0	1	3
0	1	1	90	87,1429	25,7143	1	1	2
0	1	1	94,2857	60	0	1	1	1
0	0	0	37,1429	47,1429	0	0	0	2
1	0	1	192,857	90	64,2857	1	1	2
0	0	0	8,57143	34,2857	0	0	0	1
0	0	0	72,8571	34,2857	0	1	0	2
0	0	0	21,4286	42,8571	0	0	0	1
0	0	0	28,5714	102,857	0	0	1	2
0	0	0	42,8571	38,5714	0	0	0	1
0	0	0	8,57143	42,8571	0	0	0	2
0	0	0	22,8571	34,2857	0	0	0	2
0	0	0	14,2857	34,2857	17,1429	0	0	1
0	1	1	77,1429	25,7143	0	1	0	2

0	0	0	14,2857	60	0	0	1	3
0	0	0	47,1429	51,4286	0	0	0	2
0	0	0	8,57143	8,57143	0	0	0	2
0	0	0	14,2857	85,7143	0	0	1	2
0	1	1	154,286	55,7143	0	1	0	1
0	0	0	57,1429	57,8571	0	0	0	1
0	0	0	109,286	51,4286	0	1	0	2
0	0	0	30	64,2857	12,8571	0	1	2
0	0	0	30	17,1429	0	0	0	2
0	0	0	50	77,1429	0	0	1	2
0	0	0	11,4286	20	0	0	0	2
0	0	0	107,143	70,7143	0	1	1	2
0	0	0	22,8571	180	0	0	1	2
0	1	1	154,286	180	34,2857	1	1	1
0	1	1	75	51,4286	0	1	0	2
0	1	1	90	4,28571	0	1	0	2
0	0	0	57,8571	22,8571	0	0	0	2
0	0	0	30	71,4286	0	0	1	2
0	0	0	32,1429	42,8571	0	0	0	1
0	0	0	38,5714	44,2857	0	0	0	1
0	0	0	0	208,571	180	0	1	2
0	0	0	8,57143	94,2857	0	0	1	1
1	1	1	205,714	148,571	0	1	1	1
0	0	0	47,8571	0	2,85714	0	0	2
0	0	0	197,143	128,571	77,1429	1	1	3
0	0	0	0	128,571	0	0	1	2
0	0	0	19,2857	85,7143	0	0	1	2
0	1	1	197,143	94,2857	8,57143	1	1	2
0	0	0	248,571	132,857	0	1	1	1
0	0	0	68,5714	77,1429	0	1	1	2
0	0	0	0	182,857	25,7143	0	1	1
0	0	0	120	17,1429	8,57143	1	0	3
0	0	0	25,7143	102,857	0	0	1	1
0	0	0	30	19,2857	0	0	0	2
0	0	0	61,4286	25,7143	0	1	0	2
0	0	0	0	265,714	0	0	1	1
0	0	0	40	154,286	0	0	1	3
0	0	0	2,85714	240	0	0	1	1
0	0	0	10	38,5714	0	0	0	2
0	0	0	40	51,4286	0	0	0	2
0	0	0	180	167,143	0	1	1	2
0	0	0	17,1429	42,8571	0	0	0	2
0	0	0	6,42857	60	0	0	1	2
0	1	1	231,429	60	0	1	1	2
0	0	0	128,571	25,7143	0	1	0	1
0	0	0	12,8571	17,1429	0	0	0	2
0	0	0	47,1429	109,286	0	0	1	2
0	0	0	0	80	0	0	1	2

0	0	0	128,571	171,429	8,57143	1	1	2
0	0	0	7,14286	188,571	0	0	1	1
0	0	0	32,1429	0	0	0	0	1
0	0	0	25,7143	51,4286	0	0	0	2
0	0	0	25,7143	120	0	0	1	1
0	0	0	50	34,2857	0	0	0	2
0	0	0	42,8571	4,28571	0	0	0	2
0	0	0	128,571	197,143	0	1	1	2
0	0	0	171,429	105	0	1	1	2
0	0	0	55,7143	42,8571	17,1429	0	1	1
0	0	0	69,2857	60	0	1	1	2
0	0	0	60	150	0	1	1	2
0	0	0	0	145,714	102,857	0	1	2
0	0	0	152,857	50	4,28571	1	0	1
0	0	0	34,2857	8,57143	0	0	0	3
0	0	0	0	231,429	0	0	1	2
0	0	0	120	25,7143	0	1	0	2
0	0	0	0	51,4286	0	0	0	2
0	0	0	82,8571	154,286	25,7143	1	1	2
0	0	0	14,2857	51,4286	17,1429	0	1	2
0	0	0	0	51,4286	0	0	0	2
0	1	1	154,286	79,2857	0	1	1	1
0	0	1	154,286	124,286	0	1	1	2
0	0	0	15	12,8571	2,85714	0	0	2
0	0	0	51,4286	69,2857	0	0	1	3
0	0	0	35,7143	68,5714	0	0	1	3
0	0	0	158,571	222,857	0	1	1	1
0	0	0	148,571	51,4286	0	1	0	1
0	0	0	8,57143	8,57143	0	0	0	3
0	0	0	60	45,7143	0	1	0	2
0	0	0	81,4286	34,2857	0	1	0	1
0	0	0	68,5714	68,5714	30	1	1	3
0	0	0	157,143	192,857	0	1	1	2
0	0	0	42,8571	17,1429	0	0	0	1
0	0	0	14,2857	77,1429	0	0	1	2
0	1	1	240	208,571	0	1	1	1
0	0	0	21,4286	154,286	0	0	1	2
0	0	0	60	17,8571	0	1	0	2
0	0	0	4,28571	6,42857	0	0	0	2
0	0	0	15	214,286	85,7143	0	1	1
0	0	1	30	85,7143	0	0	1	2
0	0	0	40	180	25,7143	0	1	2
0	0	0	0	180	0	0	1	2
0	0	0	12,8571	98,5714	0	0	1	2
0	0	0	12,8571	51,4286	17,1429	0	1	2
0	0	0	30	85,7143	0	0	1	1
0	0	1	351,429	255	30	1	1	2
0	0	0	17,1429	60	23,5714	0	1	2

0	0	0	0	17,1429	0	0	0	3
0	0	0	0	102,857	0	0	1	2
0	0	0	32,1429	51,4286	0	0	0	2
0	0	0	42,8571	77,1429	0	0	1	2
0	0	0	17,1429	77,1429	0	0	1	3
0	0	0	35	34,2857	0	0	0	2
0	0	0	15	85,7143	12,8571	0	1	2
0	0	0	77,1429	34,2857	0	1	0	2
0	0	0	0	102,857	0	0	1	2
0	0	0	34,2857	34,2857	0	0	0	1
0	0	0	13,5714	25,7143	0	0	0	2
0	0	0	82,8571	170	0	1	1	2
0	0	0	51,4286	60	0	0	1	2
0	0	0	25	155,714	10	0	1	2
0	1	1	60	45	0	1	0	1
0	0	0	68,5714	25,7143	0	1	0	2
0	0	0	7,14286	21,4286	0	0	0	2
0	0	0	17,1429	0	12,8571	0	0	2
0	0	0	102,857	248,571	180	1	1	2
0	0	0	188,571	34,2857	25,7143	1	1	1
0	0	0	6,42857	70,7143	0	0	1	2
0	0	0	14,2857	51,4286	0	0	0	1
0	0	0	51,4286	120	0	0	1	2
0	0	0	128,571	32,1429	0	1	0	1
0	0	0	30	25,7143	0	0	0	1
0	0	0	31,4286	78,5714	0	0	1	2
0	0	0	77,1429	51,4286	0	1	0	1
0	0	0	0	37,1429	0	0	0	1
0	0	0	124,286	197,143	0	1	1	2
0	0	0	60	72,8571	8,57143	1	1	1
0	0	0	42,8571	25,7143	0	0	0	2
0	0	0	120	280,714	68,5714	1	1	2
0	0	0	34,2857	51,4286	51,4286	0	1	2
0	0	0	0	80	17,1429	0	1	2
0	0	0	35,7143	102,857	0	0	1	2
0	0	0	55,7143	35,7143	0	0	0	2
0	0	0	15,7143	68,5714	0	0	1	1
0	0	0	0	64,2857	0	0	1	2
0	0	0	127,143	51,4286	0	1	0	2
0	0	0	60	25,7143	0	1	0	2
0	0	0	194,286	282,857	0	1	1	2
0	1	1	162,857	68,5714	0	1	1	1
0	0	0	25,7143	154,286	0	0	1	3
0	0	0	4,28571	0	0	0	0	2
0	0	0	94,2857	68,5714	0	1	1	2
0	0	0	137,143	154,286	0	1	1	2
0	0	0	17,1429	22,8571	0	0	0	2
0	0	0	18,5714	25,7143	0	0	0	2

0	0	0	34,2857	102,857	0	0	1	1
0	0	0	40	8,57143	0	0	0	2
0	0	0	0	100	51,4286	0	1	1
0	0	0	8,57143	171,429	0	0	1	2
0	0	0	4,28571	0	0	0	0	2
0	0	0	32,1429	64,2857	0	0	1	2
0	0	0	25,7143	34,2857	0	0	0	2
0	0	0	0	102,857	0	0	1	1
0	1	1	398,571	137,143	25,7143	1	1	1
0	0	0	54,2857	0	0	0	0	2
0	0	0	17,1429	51,4286	0	0	0	2
0	0	0	94,2857	111,429	17,1429	1	1	2
0	0	0	51,4286	47,1429	0	0	0	1
0	0	0	25,7143	80	25,7143	0	1	2
0	0	0	0	25,7143	0	0	0	2
0	0	0	21,4286	0	0	0	0	1
0	0	0	205,714	0	0	1	0	1
0	0	0	0	137,143	0	0	1	1
0	0	0	14,2857	34,2857	0	0	0	2
0	0	0	38,5714	94,2857	0	0	1	2
0	0	0	72,8571	25,7143	0	1	0	2
0	1	1	100	17,1429	0	1	0	2
0	0	0	17,1429	107,143	21,4286	0	1	2
0	0	0	102,857	25,7143	0	1	0	1
0	0	0	25,7143	37,1429	0	0	0	2
0	0	0	38,5714	25,7143	0	0	0	2
0	0	0	8,57143	0	0	0	0	2
0	0	0	197,143	173,571	25,7143	1	1	1
0	0	0	128,571	94,2857	0	1	1	2
0	0	0	40,7143	55,7143	0	0	0	1
0	0	0	8,57143	0	0	0	0	2
0	0	0	0	68,5714	0	0	1	2
0	0	0	51,4286	17,1429	0	0	0	2
0	0	0	188,571	215,714	77,1429	1	1	2
0	0	0	37,1429	51,4286	0	0	0	3
0	0	0	41,4286	51,4286	0	0	0	1
0	0	0	60	143,571	42,8571	1	1	2
0	0	0	38,5714	42,8571	0	0	0	1
0	0	0	171,429	34,2857	0	1	0	2
0	0	0	180	111,429	0	1	1	2
0	0	0	107,143	180	0	1	1	3
0	0	0	2,85714	90	77,1429	0	1	1
0	1	1	180	34,2857	0	1	0	1
0	1	1	187,143	8,57143	0	1	0	2
0	0	0	0	171,429	0	0	1	1
0	0	0	85,7143	55,7143	0	1	0	1
0	0	0	128,571	180	0	1	1	2
0	1	1	120	111,429	42,8571	1	1	1

0	0	0	90	60	0	1	1	2
0	1	1	77,1429	257,143	0	1	1	2
0	0	0	85,7143	40	0	1	0	2
0	0	0	20,7143	145,714	0	0	1	1
0	0	1	66,4286	36,4286	0	1	0	2
0	0	0	25,7143	45,7143	40	0	1	2
0	0	0	130	42,8571	0	1	0	1
0	0	0	150	150	0	1	1	2
0	0	0	60	34,2857	0	1	0	1
0	0	0	10,7143	17,1429	0	0	0	2
0	0	0	42,8571	120	0	0	1	2
0	0	0	60	317,143	85,7143	1	1	2
0	0	0	10	77,1429	0	0	1	2
0	0	0	72,8571	68,5714	0	1	1	2
0	0	0	85,7143	120	0	1	1	1
0	0	0	4,28571	79,2857	0	0	1	2
0	0	0	41,4286	22,8571	0	0	0	2
0	0	0	0	30	0	0	0	2
0	1	1	188,571	40,7143	0	1	0	2
0	0	0	0	25,7143	0	0	0	1
0	0	0	150	47,1429	17,1429	1	1	2
0	0	0	85,7143	128,571	0	1	1	2
0	0	0	132,857	131,429	0	1	1	2
0	0	0	14,2857	51,4286	0	0	0	2
0	1	1	162,857	60	0	1	1	1
0	0	1	57,8571	27,8571	0	0	0	2
0	0	0	25,7143	45,7143	12,8571	0	0	1
0	0	0	148,571	51,4286	8,57143	1	1	1
0	0	0	25,7143	38,5714	0	0	0	2
0	1	1	195	42,8571	0	1	0	2
0	0	0	90	120	0	1	1	2
0	0	0	128,571	326,429	0	1	1	2
0	0	0	111,429	150	0	1	1	2
0	1	1	85,7143	68,5714	0	1	1	2
0	1	1	85,7143	42,8571	0	1	0	2
0	0	0	4,28571	51,4286	0	0	0	2
0	0	0	25	21,4286	0	0	0	2
0	0	0	0	25,7143	0	0	0	2
0	0	0	0	28,5714	0	0	0	2
0	0	0	8,57143	42,8571	0	0	0	1
0	0	0	38,5714	34,2857	0	0	0	2
0	0	0	48,5714	0	0	0	0	2
0	0	0	34,2857	17,1429	0	0	0	2
0	0	0	25,7143	180	0	0	1	1
1	1	1	300	85,7143	60	1	1	2
0	0	0	0	4,28571	17,1429	0	0	1
0	0	0	148,571	32,1429	12,8571	1	0	2
0	0	0	0	102,857	0	0	1	2

0	0	0	34,2857	51,4286	0	0	0	1
0	0	0	20	34,2857	0	0	0	1
0	0	0	68,5714	34,2857	0	1	0	2
0	0	0	120	34,2857	17,1429	1	0	2
0	0	0	30	85,7143	34,2857	0	1	2
0	0	0	37,1429	54,2857	0	0	0	2
0	0	0	111,429	25,7143	34,2857	1	1	2
0	0	0	62,8571	71,4286	0	1	1	2
0	0	0	8,57143	51,4286	0	0	0	2
0	0	0	42,8571	17,1429	0	0	0	1
0	0	0	17,1429	0	0	0	0	2
0	0	0	0	0	25,7143	0	0	2
0	0	0	15	68,5714	0	0	1	2
0	0	0	150	51,4286	0	1	0	1
0	0	0	74,2857	12,8571	34,2857	1	0	2
0	0	0	7,14286	145,714	0	0	1	1
0	0	0	154,286	130,714	0	1	1	2
0	0	0	188,571	85,7143	0	1	1	1
0	0	0	0	51,4286	0	0	0	1
0	0	0	128,571	45	0	1	0	3
0	0	0	0	20	0	0	0	2
0	0	0	0	51,4286	0	0	0	2
0	1	1	180	34,2857	0	1	0	2
0	1	1	300	102,857	0	1	1	2
0	0	0	85,7143	18,5714	51,4286	1	1	2
0	0	0	18,5714	68,5714	0	0	1	2
0	0	0	28,5714	180	25,7143	0	1	3
0	0	0	137,143	69,2857	21,4286	1	1	1
0	0	0	8,57143	34,2857	0	0	0	2
0	0	0	55,7143	55,7143	0	0	0	2
0	0	0	47,1429	162,857	0	0	1	2
0	0	0	128,571	120	0	1	1	1
0	0	0	216,429	2,85714	25,7143	1	0	2
0	0	0	142,857	128,571	0	1	1	1
0	0	0	231,429	342,857	128,571	1	1	2
0	0	0	55,7143	120	0	0	1	2
0	0	0	12,8571	85,7143	0	0	1	1
0	0	0	17,1429	25,7143	0	0	0	2
0	0	0	107,143	180	0	1	1	2
0	0	0	180	261,429	0	1	1	1
0	0	0	25,7143	30	25,7143	0	0	3
0	0	0	145,714	191,429	0	1	1	2
0	0	0	51,4286	42,8571	0	0	0	2
0	0	1	51,4286	32,1429	6,42857	0	0	2
0	0	0	55,7143	11,4286	0	0	0	2
0	0	0	68,5714	8,57143	0	1	0	2
0	0	0	85,7143	21,4286	0	1	0	2
0	0	0	94,2857	205,714	8,57143	1	1	2

0	0	1	120	252,857	0	1	1	2
0	0	0	181,429	40	0	1	0	2
0	0	0	40	102,857	0	0	1	2
0	1	1	102,857	45	0	1	0	2
0	0	0	0	0	0	0	0	2
0	0	0	68,5714	100,714	0	1	1	2
0	0	0	171,429	120	0	1	1	3
0	0	0	0	120	51,4286	0	1	2
0	0	0	42,8571	27,1429	0	0	0	2
0	0	0	25,7143	38,5714	17,1429	0	0	2
0	0	0	51,4286	25,7143	0	0	0	1
0	0	0	128,571	25,7143	0	1	0	3
0	0	0	45,7143	68,5714	0	0	1	1
0	0	0	54,2857	42,8571	0	0	0	2
0	0	0	0	12,8571	0	0	0	1
0	1	1	240	17,1429	0	1	0	2
0	0	0	44,2857	162,857	0	0	1	1
0	1	1	210	164,286	0	1	1	2
0	0	0	7,14286	38,5714	12,8571	0	0	2
0	0	0	14,2857	34,2857	0	0	0	2
0	0	0	137,143	76,4286	8,57143	1	1	2
0	0	0	42,8571	12,8571	0	0	0	2
0	1	1	68,5714	121,429	0	1	1	2
0	0	0	57,1429	32,1429	0	0	0	1
0	0	0	68,5714	60	17,1429	1	1	2
0	0	0	51,4286	19,2857	0	0	0	2
0	1	1	150	17,1429	0	1	0	2
0	0	0	148,571	231,429	0	1	1	2
0	0	0	171,429	137,143	0	1	1	2
0	0	0	0	12,8571	0	0	0	2
0	0	0	180	180	8,57143	1	1	2
0	0	0	25,7143	51,4286	0	0	0	2
0	0	0	45,7143	17,1429	0	0	0	2
0	0	0	127,143	30	0	1	0	2
0	0	0	8,57143	60	4,28571	0	1	2
0	0	0	25,7143	137,143	0	0	1	2
0	0	0	4,28571	285,714	0	0	1	2
0	1	1	74,2857	25,7143	0	1	0	2
0	0	0	188,571	285	0	1	1	2
0	0	0	32,1429	85,7143	0	0	1	2
0	0	0	180	265,714	0	1	1	2
0	0	0	2,85714	0	0	0	0	2
0	0	0	75	17,1429	0	1	0	1
0	1	1	75	17,1429	0	1	0	2
0	1	1	74,2857	102,857	0	1	1	2
0	0	0	5,71429	145,714	0	0	1	1
0	0	0	42,8571	27,8571	0	0	0	2
0	0	0	14,2857	17,1429	0	0	0	1

0	0	0	51,4286	72,8571	0	0	1	2
0	1	1	60	240	0	1	1	1
0	1	1	115,714	25,7143	0	1	0	2
0	0	0	0	34,2857	0	0	0	2
0	0	0	2,85714	257,143	0	0	1	1
0	0	0	10,7143	34,2857	0	0	0	2
0	0	0	34,2857	171,429	0	0	1	1
0	0	0	24,2857	17,1429	0	0	0	1
0	0	0	77,1429	17,1429	17,1429	1	0	2
0	1	1	120	17,1429	0	1	0	3
0	0	0	0	85,7143	0	0	1	1
0	0	0	34,2857	25,7143	0	0	0	2
0	0	0	171,429	62,1429	0	1	1	1
0	0	0	47,1429	85,7143	0	0	1	3
0	0	0	21,4286	77,1429	0	0	1	3
0	0	0	10,7143	25,7143	0	0	0	2
0	0	0	0	11,4286	0	0	0	1
0	0	0	2,85714	193,571	0	0	1	1
0	0	0	25,7143	94,2857	34,2857	0	1	2
0	0	0	47,1429	115,714	0	0	1	1
0	0	0	14,2857	115,714	0	0	1	3
0	0	0	145,714	17,1429	0	1	0	3
0	1	1	128,571	60	85,7143	1	1	1
0	0	0	42,8571	77,1429	0	0	1	2
0	0	0	128,571	21,4286	0	1	0	2
0	0	0	17,1429	12,8571	0	0	0	2
0	0	0	17,1429	51,4286	0	0	0	2
0	0	0	17,1429	128,571	0	0	1	2
0	0	0	51,4286	257,143	17,1429	0	1	2
0	1	1	180	96,4286	0	1	1	2
0	0	0	8,57143	120	0	0	1	2
0	0	0	171,429	360	25,7143	1	1	2
0	0	0	17,1429	64,2857	0	0	1	2
0	0	0	188,571	257,143	0	1	1	1
0	0	0	117,857	62,1429	0	1	1	2
0	0	0	51,4286	54,2857	128,571	0	1	2
0	0	0	128,571	102,857	0	1	1	1
0	0	0	25,7143	202,857	0	0	1	2
0	0	0	150	105,714	0	1	1	2
0	0	0	94,2857	157,143	0	1	1	2
0	1	1	94,2857	102,857	0	1	1	2
0	0	0	131,429	57,8571	0	1	0	2
0	0	0	145,714	85,7143	0	1	1	2
0	0	0	68,5714	53,5714	0	1	0	2
0	0	0	47,1429	25,7143	0	0	0	2
0	0	0	184,286	137,143	25,7143	1	1	2
0	0	0	107,143	40,7143	0	1	0	3
0	1	1	150	51,4286	0	1	0	2

0	0	0	23,5714	17,1429	0	0	0	2
0	0	0	91,4286	94,2857	0	1	1	3
0	1	1	128,571	77,1429	0	1	1	1
0	0	0	0	61,4286	25,7143	0	1	3
0	0	0	30	34,2857	0	0	0	1
0	0	0	85,7143	60	0	1	1	1
0	0	0	51,4286	124,286	0	0	1	2
0	0	0	68,5714	34,2857	0	1	0	1
0	0	0	51,4286	105	17,1429	0	1	2
0	0	0	55,7143	184,286	25,7143	0	1	1
0	0	0	0	2,85714	0	0	0	3
0	0	0	42,8571	17,1429	0	0	0	2
0	0	0	72,8571	244,286	154,286	1	1	1
0	0	0	85,7143	85,7143	0	1	1	2
0	0	1	222,857	40	47,1429	1	1	2
0	0	0	77,1429	60	0	1	1	2
0	0	0	14,2857	17,1429	0	0	0	1
0	0	0	58,5714	85,7143	0	0	1	2
1	0	1	42,8571	188,571	51,4286	0	1	2
0	0	0	17,1429	154,286	0	0	1	1
0	0	0	10	145,714	0	0	1	2
0	0	0	132,857	64,2857	0	1	1	2
0	0	0	5,71429	17,1429	0	0	0	1
0	0	0	265,714	60	51,4286	1	1	2
0	0	0	20	25,7143	0	0	0	1
0	0	0	2,85714	111,429	0	0	1	2
0	1	1	94,2857	111,429	0	1	1	2
0	0	0	8,57143	17,1429	0	0	0	1
1	0	1	128,571	360	0	1	1	2
0	0	0	111,429	34,2857	0	1	0	2
0	0	0	21,4286	42,8571	0	0	0	1
0	1	1	202,857	141,429	0	1	1	2
0	0	0	25,7143	0	8,57143	0	0	2
0	1	1	98,5714	51,4286	0	1	0	2
0	0	0	52,8571	17,1429	0	0	0	2
0	0	0	34,2857	51,4286	0	0	0	2
0	0	0	22,8571	34,2857	0	0	0	3
0	0	0	4,28571	109,286	0	0	1	1
0	0	0	0	85	0	0	1	2
0	0	0	15	111,429	0	0	1	2
0	0	0	4,28571	105,714	0	0	1	2
0	0	0	75	14,2857	0	1	0	2
0	1	1	100	17,1429	0	1	0	1
0	0	0	28,5714	21,4286	0	0	0	2
0	0	0	14,2857	42,8571	0	0	0	2
0	0	0	128,571	171,429	0	1	1	2
0	0	0	115,714	214,286	0	1	1	2
0	0	0	25,7143	34,2857	0	0	0	2

0	0	0	17,8571	42,8571	0	0	0	2
0	1	1	140	180	0	1	1	1
0	0	0	42,8571	77,1429	0	0	1	2
0	1	1	274,286	128,571	85,7143	1	1	1
0	0	0	25,7143	175,714	0	0	1	3
0	0	0	25,7143	87,8571	0	0	1	2
0	0	0	24,2857	8,57143	0	0	0	2
0	0	1	0	60	0	0	1	3
0	0	0	4,28571	85,7143	0	0	1	2
0	0	0	38,5714	42,8571	0	0	0	2
0	0	0	34,2857	231,429	0	0	1	2
0	0	0	45	27,8571	0	0	0	2
0	0	0	65,7143	60	0	1	1	1
0	0	0	30	30	17,1429	0	0	2
0	0	0	34,2857	154,286	0	0	1	3
0	0	0	50,7143	25,7143	0	0	0	1
0	0	0	49,2857	42,8571	0	0	0	2
0	0	0	137,143	231,429	25,7143	1	1	2
0	0	0	68,5714	141,429	0	1	1	2
0	0	0	40	42,8571	0	0	0	2
0	0	0	42,8571	51,4286	0	0	0	2
0	0	0	185,714	128,571	0	1	1	2
0	0	0	21,4286	25,7143	0	0	0	1
0	0	0	70,7143	24,2857	0	1	0	2
0	0	0	214,286	205,714	4,28571	1	1	1
0	0	0	85,7143	42,8571	0	1	0	1
0	0	0	17,1429	82,8571	0	0	1	2
0	0	0	10,7143	51,4286	0	0	0	2
0	0	0	10,7143	115,714	0	0	1	1
0	0	0	20	231,429	0	0	1	1
0	0	0	111,429	17,1429	0	1	0	1
0	0	0	34,2857	214,286	17,1429	0	1	3
0	0	0	205,714	34,2857	25,7143	1	1	2
0	0	0	171,429	238,571	0	1	1	1
0	0	0	11,4286	85,7143	0	0	1	1
0	0	0	25,7143	110	85,7143	0	1	2
0	0	0	27,8571	60	51,4286	0	1	3
0	0	0	128,571	141,429	0	1	1	3
0	0	0	42,8571	12,8571	0	0	0	2
0	0	0	0	77,1429	0	0	1	2
0	0	0	57,8571	2,85714	0	0	0	1
0	0	0	15	0	25,7143	0	0	2
0	0	0	30	60	0	0	1	1
0	0	0	154,286	308,571	0	1	1	1
0	0	0	107,143	49,2857	0	1	0	2
0	0	0	30	180	0	0	1	1
0	0	0	0	5,71429	0	0	0	1
0	0	0	168,571	180	0	1	1	2

0	0	0	81,4286	34,2857	0	1	0	2
0	0	0	92,1429	120	8,57143	1	1	2
0	0	0	0	25,7143	0	0	0	2
0	0	0	188,571	205,714	0	1	1	1
0	0	0	47,1429	85,7143	0	0	1	2
0	0	0	12,1429	17,1429	0	0	0	1
0	0	0	154,286	274,286	0	1	1	2
0	0	0	0	154,286	0	0	1	2
0	0	0	17,1429	85,7143	0	0	1	2
0	0	0	22,8571	42,8571	0	0	0	2
0	0	0	128,571	51,4286	0	1	0	3
0	0	0	0	111,429	51,4286	0	1	2
0	0	0	54,2857	25,7143	0	0	0	3
0	0	1	77,1429	150	0	1	1	2
0	0	0	94,2857	180	51,4286	1	1	2
0	1	1	274,286	15	0	1	0	2
0	0	0	17,8571	17,1429	0	0	0	2
0	0	0	6,42857	44,2857	0	0	0	1
0	0	0	102,857	107,143	0	1	1	2
0	0	0	91,4286	98,5714	0	1	1	2
0	1	1	65,7143	90	0	1	1	2
0	0	0	42,8571	120	0	0	1	3
0	0	0	38,5714	60	0	0	1	2
0	0	1	34,2857	77,1429	25,7143	0	1	1
0	1	1	77,1429	25,7143	51,4286	1	1	2
0	0	0	15	8,57143	0	0	0	1
0	0	0	12,8571	42,8571	0	0	0	3
0	1	1	120	122,857	17,1429	1	1	2
0	0	1	41,4286	19,2857	17,1429	0	0	2
0	0	0	32,8571	81,4286	0	0	1	1
0	0	0	30	51,4286	0	0	0	1
0	0	0	51,4286	25,7143	0	0	0	2
0	0	0	37,1429	55,7143	17,1429	0	1	1
0	0	0	42,8571	85,7143	0	0	1	1
0	0	0	85,7143	25,7143	0	1	0	2
0	0	0	171,429	60	0	1	1	2
0	1	1	180	111,429	0	1	1	2
0	0	0	0	185,714	12,8571	0	1	1
0	0	0	11,4286	34,2857	0	0	0	2
0	1	1	205,714	47,1429	0	1	0	2
0	0	1	77,1429	20	25,7143	1	0	2
0	0	0	137,143	137,143	0	1	1	1
0	0	0	22,8571	30	0	0	0	2
0	0	0	45,7143	32,1429	0	0	0	1
0	0	0	59,2857	0	0	0	0	1
0	0	0	148,571	10	0	1	0	1
0	0	0	21,4286	51,4286	0	0	0	2
0	0	0	115,714	205,714	0	1	1	2

0	0	0	120	197,143	0	1	1	2
0	1	1	240	214,286	36,4286	1	1	2
0	0	0	1,42857	4,28571	0	0	0	1
0	0	0	94,2857	42,8571	0	1	0	1
0	0	0	100,714	51,4286	8,57143	1	1	2
0	0	0	0	34,2857	51,4286	0	1	2
0	0	0	24,2857	17,1429	0	0	0	1
0	0	0	154,286	25,7143	0	1	0	2
0	0	0	17,1429	8,57143	0	0	0	3
0	0	0	0	98,5714	0	0	1	1
0	0	0	34,2857	182,143	0	0	1	2
0	0	0	20	148,571	180	0	1	2
0	0	0	60	154,286	0	1	1	2
0	0	0	14,2857	47,1429	0	0	0	2
0	0	0	124,286	85,7143	0	1	1	1
0	0	0	51,4286	171,429	0	0	1	2
0	0	0	30	180	0	0	1	1
0	0	0	30	17,1429	0	0	0	1
0	0	0	0	25,7143	0	0	0	2
0	0	0	17,1429	121,429	0	0	1	2
0	0	0	180	222,857	0	1	1	1
0	0	0	81,4286	120	0	1	1	2
0	0	0	12,8571	128,571	0	0	1	1
0	0	0	160	30	0	1	0	1
0	0	0	17,1429	42,8571	0	0	0	2
0	0	0	47,1429	134,286	0	0	1	1
0	0	0	21,4286	25,7143	0	0	0	1
0	0	0	98,5714	17,1429	0	1	0	2
0	0	0	25,7143	85,7143	128,571	0	1	2
0	0	0	12,8571	0	0	0	0	3
0	0	0	21,4286	100	19,2857	0	1	2
0	0	0	0	188,571	0	0	1	2
0	0	0	25,7143	60	8,57143	0	1	1
0	0	0	11,4286	17,1429	0	0	0	2
0	0	0	8,57143	0	51,4286	0	0	2
0	0	0	201,429	248,571	4,28571	1	1	2
0	0	0	2,85714	214,286	0	0	1	2
0	0	0	60	102,857	0	1	1	1
0	0	0	77,1429	25,7143	0	1	0	2
0	0	0	34,2857	25,7143	0	0	0	2
0	0	0	10,7143	171,429	51,4286	0	1	1
0	0	0	7,85714	15	0	0	0	1
0	0	0	47,1429	145,714	0	0	1	2
0	0	0	17,1429	72,8571	0	0	1	2
0	0	0	34,2857	51,4286	0	0	0	2
0	0	0	102,857	77,1429	0	1	1	2
0	0	0	141,429	177,143	150	1	1	2
0	1	1	325,714	102,857	60	1	1	1

0	0	0	19,2857	55,7143	0	0	0	1
0	0	0	64,2857	87,1429	14,2857	1	1	1
0	0	0	154,286	257,143	0	1	1	2
0	0	0	12,8571	15	17,1429	0	0	2
0	0	0	0	30	0	0	0	1
0	0	0	77,1429	57,8571	6,42857	1	1	2
0	0	0	68,5714	85,7143	77,1429	1	1	2
0	0	0	42,8571	11,4286	0	0	0	2
0	0	0	87,1429	72,8571	4,28571	1	1	2
0	0	0	4,28571	68,5714	0	0	1	1
0	0	0	98,5714	102,857	0	1	1	2
0	0	0	37,1429	19,2857	0	0	0	2
0	0	0	165,714	197,143	8,57143	1	1	1
0	0	0	0	225,714	0	0	1	2
0	0	0	45,7143	21,4286	0	0	0	2
0	0	0	257,143	282,857	0	1	1	2
0	0	0	34,2857	45	0	0	0	1
0	0	0	160	102,857	8,57143	1	1	2
0	0	0	57,1429	60	0	0	1	3
0	0	0	28,5714	60	0	0	1	1
0	1	1	102,857	0	0	1	0	2
0	0	0	0	8,57143	0	0	0	2
0	0	0	17,1429	132,857	0	0	1	2
0	1	1	216,429	17,1429	0	1	0	3
0	1	1	67,1429	94,2857	128,571	1	1	2
0	0	0	7,14286	120	0	0	1	2
0	0	0	40	85,7143	0	0	1	2
0	0	0	42,8571	120	0	0	1	2
0	0	0	6,42857	17,1429	25,7143	0	0	2
0	0	0	0	34,2857	0	0	0	2
0	0	0	127,143	144,286	25,7143	1	1	3
0	0	0	58,5714	80	0	0	1	2
0	0	0	21,4286	25,7143	0	0	0	2
0	0	1	278,571	171,429	30	1	1	2
0	0	0	94,2857	77,1429	0	1	1	3
0	0	0	0	85,7143	0	0	1	2
0	0	0	25	68,5714	0	0	1	1
0	0	0	10,7143	34,2857	0	0	0	1
0	0	0	0	111,429	0	0	1	2
0	0	0	34,2857	51,4286	0	0	0	1
0	0	0	6,42857	42,8571	0	0	0	2
0	0	0	8,57143	38,5714	8,57143	0	0	1
0	0	0	45,7143	17,1429	0	0	0	1
0	0	0	90	102,857	0	1	1	2
0	0	0	0	154,286	0	0	1	2
0	0	0	34,2857	42,8571	0	0	0	2
0	0	0	132,857	135	0	1	1	1
0	0	0	34,2857	49,2857	8,57143	0	0	3

0	0	0	274,286	334,286	0	1	1	2
0	0	0	17,1429	25,7143	0	0	0	1
0	0	0	65	0	12,8571	1	0	1
0	0	0	42,8571	51,4286	0	0	0	2
0	1	1	120	34,2857	0	1	0	2
0	0	0	34,2857	31,4286	0	0	0	2
0	0	0	14,2857	222,857	34,2857	0	1	2
0	0	0	85,7143	120	0	1	1	2
1	1	1	120	137,143	137,143	1	1	2
0	0	0	0	5,71429	0	0	0	2
0	0	0	154,286	25,7143	0	1	0	2
0	0	0	154,286	51,4286	0	1	0	2
0	0	1	64,2857	102,857	0	1	1	2
0	0	0	20	180	102,857	0	1	2
0	0	0	0	42,8571	0	0	0	2
0	0	0	167,143	188,571	0	1	1	2
0	0	0	30	51,4286	0	0	0	2
0	0	0	2,85714	17,1429	0	0	0	2
0	0	0	38,5714	30	0	0	0	2
0	0	0	102,857	77,1429	47,1429	1	1	2
0	0	0	128,571	42,8571	0	1	0	1
0	0	0	8,57143	210	77,1429	0	1	2
0	0	0	135,714	171,429	0	1	1	2
0	0	0	14,2857	180	0	0	1	1
0	0	0	20	17,1429	0	0	0	2
0	0	0	17,1429	34,2857	0	0	0	2
0	0	0	0	0	0	0	0	2
0	0	0	30	31,4286	0	0	0	1
0	1	1	240	222,857	25,7143	1	1	1
0	0	0	51,4286	94,2857	0	0	1	1
0	0	0	32,1429	171,429	0	0	1	1
0	1	1	90	195	0	1	1	1
0	0	0	180	34,2857	0	1	0	2
0	0	0	48,5714	34,2857	0	0	0	2
0	0	0	197,143	17,1429	0	1	0	1
0	0	0	21,4286	154,286	30	0	1	2
0	0	0	22,8571	77,1429	0	0	1	2
0	0	0	200	120	0	1	1	1
0	0	0	4,28571	36,4286	0	0	0	2
0	0	0	128,571	53,5714	0	1	0	1
0	1	1	300	68,5714	0	1	1	2
0	1	1	60	30	0	1	0	2
0	0	0	51,4286	21,4286	0	0	0	2
0	0	0	94,2857	180	0	1	1	3
0	0	0	148,571	120	0	1	1	1
0	0	0	137,143	111,429	0	1	1	2
0	0	1	64,2857	60	0	1	1	1
0	1	1	180	30	0	1	0	1

0	0	0	222,857	180	34,2857	1	1	2
0	0	0	102,857	94,2857	0	1	1	2
0	0	0	154,286	259,286	0	1	1	1
0	0	0	47,1429	25	0	0	0	1
0	0	0	90	42,8571	0	1	0	2
0	0	0	150	162,857	51,4286	1	1	2
0	0	0	40	60	0	0	1	2
0	1	1	85,7143	205,714	0	1	1	3
0	0	1	240	25,7143	21,4286	1	0	1
0	0	0	28,5714	188,571	42,8571	0	1	2
0	0	0	0	60	0	0	1	2
0	0	0	0	17,1429	17,1429	0	0	2
0	0	0	4,28571	85,7143	0	0	1	2
0	0	0	188,571	51,4286	0	1	0	1
0	0	0	155	171,429	0	1	1	2
0	0	0	278,571	175,714	0	1	1	1
0	0	0	141,429	60	0	1	1	1
0	0	0	20	214,286	0	0	1	2
0	0	0	81,4286	34,2857	0	1	0	1
0	0	0	21,4286	106,429	0	0	1	1
0	0	0	0	81,4286	0	0	1	2
0	0	0	0	165	0	0	1	3
0	0	0	17,1429	143,571	0	0	1	2
0	0	0	64,2857	0	0	1	0	2
0	0	0	34,2857	34,2857	0	0	0	1
1	0	1	8,57143	91,4286	0	0	1	2
0	0	0	150	312,857	0	1	1	2
0	0	0	21,4286	45,7143	0	0	0	2
0	0	0	0	25,7143	0	0	0	3
0	0	0	34,2857	102,857	68,5714	0	1	2
0	0	0	42,8571	51,4286	0	0	0	2
0	0	0	54,2857	42,8571	0	0	0	2
0	0	0	165,714	120	17,1429	1	1	1
0	0	0	14,2857	25,7143	0	0	0	1
0	0	0	92,1429	30	0	1	0	1
0	0	0	28,5714	120	0	0	1	2
0	0	0	94,2857	231,429	128,571	1	1	2
0	1	1	282,857	68,5714	0	1	1	2
0	0	0	38,5714	21,4286	0	0	0	3
0	0	0	51,4286	162,857	0	0	1	2
0	0	0	31,4286	77,1429	8,57143	0	1	1
0	0	0	0	120	30	0	1	2
0	0	0	14,2857	207,143	51,4286	0	1	2
0	0	0	31,4286	12,8571	0	0	0	1
0	1	1	120	25,7143	0	1	0	2
0	0	0	102,857	85,7143	0	1	1	2
0	0	0	115,714	25,7143	0	1	0	1
0	0	1	38,5714	68,5714	51,4286	0	1	2

0	0	0	41,4286	161,429	0	0	1	2
0	0	0	61,4286	154,286	0	1	1	1
0	0	0	55,7143	8,57143	0	0	0	2
0	0	0	171,429	47,1429	0	1	0	1
0	1	1	114,286	68,5714	0	1	1	1
0	0	0	34,2857	25,7143	0	0	0	1
0	0	0	24,2857	74	0	0	1	2
0	0	0	154,286	257,143	154,286	1	1	2
0	0	0	84,2857	8,57143	0	1	0	1
0	0	0	180	25,7143	0	1	0	1
0	0	0	180	274,286	0	1	1	1
0	0	0	55,7143	102,857	0	0	1	2
0	0	0	20	25,7143	0	0	0	2
0	0	0	0	154,286	51,4286	0	1	2
0	0	0	158,571	171,429	17,1429	1	1	1
0	0	0	102,857	64,2857	0	1	1	3
0	0	0	162,857	154,286	0	1	1	2
0	0	0	2,85714	77,1429	0	0	1	2
0	0	0	137,143	70,7143	0	1	1	1
0	0	1	111,429	137,143	0	1	1	1
0	1	1	110	35,7143	8,57143	1	0	2
0	0	0	8,57143	17,1429	0	0	0	2
0	0	0	0	45,7143	0	0	0	3
0	0	0	102,857	85,7143	0	1	1	2
0	0	0	68,5714	200	0	1	1	2
0	1	1	120	25,7143	0	1	0	1
0	0	0	21,4286	128,571	0	0	1	2
0	0	0	12,8571	51,4286	0	0	0	2
0	0	0	14,2857	291,429	180	0	1	2
0	0	0	45,7143	17,1429	0	0	0	2
0	0	1	150	41,4286	0	1	0	2
0	0	0	7,14286	102,857	0	0	1	2
0	0	0	84,2857	25,7143	25,7143	1	0	1
0	0	0	158,571	71,4286	128,571	1	1	2
0	0	0	128,571	0	0	1	0	2
0	0	0	68,5714	83,5714	0	1	1	2
0	0	0	158,571	51,4286	0	1	0	2
0	0	0	81,4286	61,4286	0	1	1	1
0	0	0	41,4286	94,2857	0	0	1	1
0	0	1	120	31,4286	0	1	0	2
0	0	0	32,8571	31,8571	0	0	0	2
0	0	0	154,286	72,8571	0	1	1	2
0	0	0	78,5714	94,2857	0	1	1	2
0	0	0	72,8571	60	0	1	1	1
0	0	0	98,5714	25,7143	0	1	0	2
0	0	0	21,4286	38,5714	0	0	0	2
0	0	0	38,5714	17,1429	0	0	0	2
0	0	0	137,143	24,2857	17,1429	1	0	2

0	0	0	42,8571	115,714	0	0	1	3
0	0	0	26,4286	49,2857	0	0	0	2
0	0	0	34,2857	231,429	17,1429	0	1	2
0	0	0	40	145,714	0	0	1	1
0	0	0	5,71429	37,1429	0	0	0	3
0	0	0	61,4286	21,4286	17,1429	1	0	2
0	0	1	57,1429	87,8571	0	0	1	2
0	0	0	27,1429	120	0	0	1	2
0	0	0	12,8571	102,857	0	0	1	3
0	1	1	154,286	171,429	60	1	1	2
0	0	0	8,57143	42,8571	0	0	0	2
0	0	0	97,1429	27,8571	0	1	0	2
0	0	0	60	6,42857	0	1	0	2
0	0	0	27,8571	17,1429	0	0	0	2
0	0	0	25,7143	77,1429	0	0	1	2
0	0	0	1,42857	77,1429	0	0	1	2
0	0	0	0	94,2857	0	0	1	2
0	1	1	134,286	102,857	0	1	1	2
0	0	0	85,7143	77,1429	77,1429	1	1	2
0	0	0	200	111,429	0	1	1	2
0	0	0	81,4286	94,2857	0	1	1	2
0	0	0	27,1429	34,2857	0	0	0	2
0	0	0	72,8571	42,8571	0	1	0	2
0	0	0	175,714	218,571	55,7143	1	1	1
0	0	0	160	165,714	0	1	1	2
0	0	0	10,7143	42,8571	0	0	0	1
0	0	0	78,5714	25,7143	25,7143	1	0	2
0	0	0	77,1429	38,5714	0	1	0	2
0	0	0	23,5714	25,7143	0	0	0	2
0	0	0	56,4286	20	0	0	0	1
0	0	0	42,8571	34,2857	0	0	0	1
0	1	1	188,571	178,571	0	1	1	2
0	0	0	42,8571	128,571	34,2857	0	1	3
0	0	0	0	0	0	0	0	2
0	0	0	167,143	120	0	1	1	2
0	0	0	34,2857	34,2857	0	0	0	2
0	0	0	50	87,1429	0	0	1	2
0	0	0	51,4286	5,71429	0	0	0	1
0	0	1	85,7143	57,8571	0	1	0	3
0	0	0	8,57143	65,7143	0	0	1	2
0	0	0	21,4286	85,7143	0	0	1	2
0	0	0	131,429	154,286	0	1	1	2
0	0	0	51,4286	25,7143	8,57143	0	0	2
0	0	0	171,429	171,429	0	1	1	1
0	0	0	0	120	0	0	1	1
0	0	0	30	51,4286	0	0	0	2
0	0	0	125,714	212,857	0	1	1	1
0	0	0	8,57143	42,8571	0	0	0	2

0	0	0	5,71429	195,714	25,7143	0	1	3
0	0	0	18,5714	38,5714	0	0	0	2
0	1	1	90	42,8571	0	1	0	2
0	0	0	25,7143	180	0	0	1	2
0	0	0	12,8571	94,2857	0	0	1	2
0	1	1	120	57,8571	17,1429	1	1	2
0	0	0	34,2857	55,7143	0	0	0	2
0	0	0	71,4286	120	0	1	1	2
0	0	0	10,7143	128,571	17,1429	0	1	2
0	0	0	45	147,857	0	0	1	1
0	0	0	55,7143	77,1429	0	0	1	2
0	0	0	22,8571	0	0	0	0	2
0	0	0	52,8571	85,7143	0	0	1	2
0	0	0	0	40,7143	6,42857	0	0	2
0	0	0	8,57143	17,1429	0	0	0	2
0	0	0	51,4286	34,2857	0	0	0	2
0	1	1	211,429	60	51,4286	1	1	2
0	0	0	11,4286	0	0	0	0	2
0	0	0	34,2857	25,7143	0	0	0	1
0	0	0	30,7143	85,7143	0	0	1	2
0	1	1	180	51,4286	0	1	0	3
0	0	0	51,4286	102,857	17,1429	0	1	2
0	0	0	128,571	180	0	1	1	1
0	1	1	282,857	0	0	1	0	1
0	0	0	51,4286	42,8571	0	0	0	2
0	0	0	17,1429	30	0	0	0	1
0	0	0	68,5714	126,429	0	1	1	1
1	1	1	94,2857	154,286	60	1	1	1
0	0	0	34,2857	25,7143	0	0	0	2
0	1	1	162,857	25,7143	0	1	0	2
0	0	0	17,1429	8,57143	0	0	0	1
0	0	0	22,8571	25,7143	17,1429	0	0	2
0	0	0	48,5714	30	0	0	0	2
0	0	0	41,4286	25,7143	0	0	0	1
0	0	0	61,4286	17,1429	0	1	0	1
0	0	0	42,8571	17,1429	0	0	0	2
0	0	0	2,85714	197,143	8,57143	0	1	2
0	0	0	8,57143	25,7143	0	0	0	1
0	0	0	35,7143	17,1429	0	0	0	1
0	1	1	111,429	17,1429	0	1	0	2
0	0	0	14,2857	17,1429	0	0	0	2
0	0	0	42,8571	55,7143	0	0	0	2
0	0	0	137,143	25,7143	0	1	0	2
0	0	0	185,714	245,714	0	1	1	1
0	0	0	35	8,57143	0	0	0	1
0	0	0	38,5714	184,286	0	0	1	2
0	0	0	8,57143	30	17,1429	0	0	1
1	1	1	420	522,857	120	1	1	1

0	0	0	22,8571	55,7143	0	0	0	2
0	0	0	30	30	0	0	0	1
0	0	0	15,7143	25,7143	0	0	0	2
0	1	1	95,7143	25,7143	0	1	0	2
0	0	0	70,7143	25,7143	0	1	0	1
0	0	0	92,8571	51,4286	12,8571	1	1	2
0	0	0	28,5714	115,714	0	0	1	2
0	0	0	64,2857	51,4286	17,1429	1	1	2
0	1	1	120	94,2857	51,4286	1	1	2
0	0	0	188,571	308,571	0	1	1	3
0	0	0	42,8571	68,5714	14,2857	0	1	2
0	0	0	15	0	0	0	0	2
0	0	0	60	205,714	0	1	1	2
0	0	0	135,714	25,7143	0	1	0	2
0	1	1	205,714	164,286	0	1	1	2
0	0	0	188,571	231,429	0	1	1	1
0	1	1	105	25,7143	0	1	0	1
0	0	0	0	137,143	120	0	1	2
0	0	0	171,429	42,8571	17,1429	1	1	2
0	0	1	42,8571	85,7143	0	0	1	2
0	0	0	147,857	197,143	8,57143	1	1	2
0	0	0	23,5714	60	17,1429	0	1	2
0	0	0	265,714	145,714	0	1	1	2
0	0	0	42,8571	17,1429	0	0	0	2
0	0	0	31,4286	25,7143	0	0	0	1
0	0	1	51,4286	30	25,7143	0	0	2
0	0	0	128,571	51,4286	25,7143	1	1	2
0	0	0	137,143	180	0	1	1	1
0	0	0	25,7143	72,8571	0	0	1	1
0	0	0	188,571	381,429	90	1	1	2
0	0	0	60	0	25,7143	1	0	1
0	0	0	0	17,1429	17,1429	0	0	1
0	0	0	27,1429	85,7143	0	0	1	1
0	1	1	171,429	34,2857	0	1	0	1
0	0	0	34,2857	128,571	0	0	1	2
0	0	0	32,8571	34,2857	0	0	0	1
0	0	0	42,8571	25,7143	0	0	0	3
0	0	0	39,2857	25,7143	0	0	0	2
0	0	0	42,8571	42,8571	0	0	0	2
0	0	0	0	137,143	0	0	1	1
0	0	0	34,2857	51,4286	12,8571	0	1	2
0	0	0	0	67,1429	0	0	1	2
0	0	0	24,2857	42,8571	0	0	0	2
0	0	0	22,8571	8,57143	0	0	0	3
0	0	0	17,1429	25,7143	0	0	0	2
0	1	1	102,857	0	0	1	0	2
0	0	0	77,1429	67,1429	0	1	1	2
1	1	1	60	141,429	0	1	1	2

0	0	0	30	60	0	0	1	2
0	0	0	154,286	34,2857	0	1	0	2
0	0	0	24,2857	128,571	0	0	1	2
0	0	0	186,857	111,429	25,7143	1	1	1
0	0	0	85,7143	51,4286	0	1	0	1
0	0	0	102,857	53,5714	0	1	0	2
0	0	0	60	0	0	1	0	2
0	0	0	49,2857	17,1429	0	0	0	2
0	0	0	100	60	0	1	1	2
0	0	0	30	143,571	0	0	1	2
0	1	1	222,857	150	0	1	1	1
0	0	0	12,8571	25,7143	0	0	0	2
0	0	0	68,5714	251,429	0	1	1	2
0	0	0	42,8571	34,2857	17,1429	0	0	2
0	0	0	85,7143	240	0	1	1	2
0	0	0	42,8571	128,571	0	0	1	2
0	0	0	57,1429	34,2857	0	0	0	2
0	0	0	37,1429	51,4286	2,85714	0	0	1
0	0	0	130,714	17,1429	0	1	0	2
0	0	0	74,2857	49,2857	20	1	1	1
0	0	0	154,286	51,4286	1,42857	1	0	1
0	0	0	28,5714	21,4286	0	0	0	1
0	0	0	42,8571	85,7143	0	0	1	2
0	0	0	36,4286	22,8571	25,7143	0	0	1
0	0	0	160	160	8,57143	1	1	2
0	0	0	51,4286	120	0	0	1	1
0	0	0	72,8571	34,2857	0	1	0	2
0	0	0	148,571	25,7143	51,4286	1	1	1
0	0	1	42,8571	45,7143	0	0	0	1
0	0	0	0	257,143	128,571	0	1	2
0	0	0	81,4286	111,429	0	1	1	2
0	0	0	27,1429	85,7143	12,8571	0	1	1
0	0	0	17,1429	107,143	0	0	1	2
0	0	0	2,14286	19,2857	0	0	0	2
1	0	1	12,8571	60	102,857	0	1	2
0	0	0	17,1429	252,857	0	0	1	2
0	0	0	10,7143	6,42857	0	0	0	2
0	0	0	2,85714	174,286	10	0	1	2
0	0	0	101,429	25,7143	0	1	0	2
0	0	0	31,4286	42,8571	0	0	0	2
0	0	0	70,7143	111,429	0	1	1	2
0	0	0	42,8571	42,8571	0	0	0	2
0	0	0	0	51,4286	0	0	0	2
0	0	0	32,1429	25,7143	0	0	0	1
0	0	0	0	180	0	0	1	2
0	0	0	17,1429	8,57143	0	0	0	1
0	0	0	42,8571	17,1429	0	0	0	2
0	0	0	141,429	0	0	1	0	2

0	0	0	141,429	85,7143	0	1	1	2
0	0	0	17,1429	25,7143	0	0	0	2
0	0	0	64,2857	64,2857	0	1	1	1
0	1	1	180	42,8571	0	1	0	1
0	0	0	34,2857	32,8571	0	0	0	1
0	0	0	120	120	0	1	1	1
0	0	0	74,2857	27,8571	25,7143	1	0	2
0	0	0	38,5714	25,7143	17,1429	0	0	1
0	0	0	26,4286	34,2857	0	0	0	2
0	0	0	67,1429	214,286	17,1429	1	1	1
0	1	1	274,286	85,7143	0	1	1	2
0	0	0	8,57143	17,1429	0	0	0	2
0	0	0	30	51,4286	0	0	0	2
0	0	0	0	47,1429	17,1429	0	1	2
0	0	0	4,28571	154,286	0	0	1	1
0	0	0	85,7143	17,1429	25,7143	1	0	2
0	0	0	10	60	17,1429	0	1	1
0	0	0	8,57143	34,2857	0	0	0	1
0	0	1	77,1429	94,2857	0	1	1	2
0	0	0	111,429	25,7143	0	1	0	2
0	0	0	0	188,571	0	0	1	2
0	0	1	47,1429	144,286	55,7143	0	1	2
0	0	1	34,2857	8,57143	17,1429	0	0	2
0	1	1	150	158,571	0	1	1	1
0	0	0	92,8571	51,4286	0	1	0	2
0	0	0	55,7143	154,286	0	0	1	3
0	0	0	72,8571	92,1429	0	1	1	2
0	0	0	38,5714	31,4286	0	0	0	1
0	0	0	205,714	120	0	1	1	2
0	0	0	17,1429	77,1429	102,857	0	1	2
0	0	0	52,8571	81,4286	0	0	1	1
0	0	0	181,429	37,1429	0	1	0	2
0	0	0	25,7143	51,4286	0	0	0	1
0	0	0	0	172,857	0	0	1	3
0	0	0	22,1429	17,1429	0	0	0	2
0	0	0	0	40,7143	0	0	0	1
0	0	0	21,4286	85,7143	0	0	1	2
0	0	0	51,4286	205,714	17,1429	0	1	2
0	0	0	25,7143	42,8571	102,857	0	1	2
0	0	1	192,857	120	0	1	1	2
0	0	0	10	42,8571	0	0	0	2
0	0	0	55,7143	51,4286	0	0	0	1
0	0	0	0	17,1429	0	0	0	1
0	0	0	21,4286	62,8571	0	0	1	2
0	0	0	0	188,571	0	0	1	2
0	0	0	22,8571	128,571	0	0	1	2
1	0	1	227,143	325,714	0	1	1	2
0	0	0	6,42857	8,57143	0	0	0	2

0	0	0	194,286	274,286	51,4286	1	1	2
0	0	0	248,571	282,857	0	1	1	2
0	0	0	112,857	154,286	0	1	1	2
0	0	0	44,2857	25,7143	0	0	0	3
0	0	0	102,857	85,7143	0	1	1	2
0	1	1	71,4286	17,1429	0	1	0	1
0	0	0	48,5714	171,429	0	0	1	1
0	0	0	30	18,5714	0	0	0	2
0	1	1	85,7143	231,429	51,4286	1	1	2
0	1	1	300	68,5714	68,5714	1	1	2
0	0	0	139,286	67,1429	0	1	1	2
0	0	0	17,1429	17,1429	0	0	0	1
0	0	0	51,4286	51,4286	0	0	0	3
0	0	0	45	77,1429	17,1429	0	1	2
0	0	0	25,7143	12,8571	0	0	0	1
0	0	0	34,2857	42,8571	0	0	0	2
0	0	0	0	25,7143	0	0	0	1
0	1	1	120	17,1429	0	1	0	2
0	0	0	4,28571	0	0	0	0	2
0	0	0	7,14286	34,2857	0	0	0	2
0	1	1	79,2857	42,8571	0	1	0	2
0	0	0	17,1429	34,2857	0	0	0	2
0	0	0	128,571	158,571	0	1	1	3
0	0	0	81,4286	51,4286	0	1	0	2
0	0	0	11,4286	8,57143	0	0	0	2
0	0	0	132,857	51,4286	0	1	0	2
0	0	0	20	12,8571	0	0	0	3
0	0	0	4,28571	188,571	102,857	0	1	2
0	0	0	64,2857	180	0	1	1	2
0	1	1	142,857	25,7143	0	1	0	1
0	0	0	12,8571	62,1429	0	0	1	2
0	0	0	60	14,2857	0	1	0	1
0	0	0	192,857	51,4286	0	1	0	1
0	0	0	138,571	244,286	81,4286	1	1	2
0	0	0	0	160	0	0	1	3
0	0	0	26,2857	28,5714	0	0	0	2
0	0	0	168,571	214,286	0	1	1	2
0	0	0	197,143	8,57143	0	1	0	1
0	0	0	214,286	34,2857	8,57143	1	0	1
0	0	0	8,57143	30	25,7143	0	0	2
0	0	0	68,5714	42,8571	0	1	0	1
0	0	0	28,5714	222,857	0	0	1	2
0	0	0	128,571	150	0	1	1	2
0	0	0	111,429	137,143	17,1429	1	1	1
0	0	0	94,2857	34,2857	0	1	0	1
0	0	1	51,4286	128,571	0	0	1	1
0	0	0	0	51,4286	51,4286	0	1	2
0	0	0	42,8571	21,4286	0	0	0	1

0	0	0	60	210	77,1429	1	1	1
0	0	0	68,5714	23,5714	0	1	0	2
0	0	0	37,1429	34,2857	0	0	0	2
0	0	0	15	8,57143	0	0	0	2
0	0	0	25,7143	85,7143	0	0	1	2
0	0	0	128,571	120	0	1	1	2
0	0	0	20	17,8571	0	0	0	2
0	0	0	25	0	17,1429	0	0	2
0	0	0	39,2857	17,1429	0	0	0	1
0	1	1	120	51,4286	0	1	0	2
0	0	0	42,8571	282,857	17,1429	0	1	2
0	0	0	137,143	47,1429	8,57143	1	0	2
0	0	0	154,286	58,5714	0	1	0	2
0	0	0	225	192,857	0	1	1	1
0	0	0	20	18,5714	25,7143	0	0	2
1	0	1	42,8571	188,571	308,571	0	1	2
0	0	0	83,5714	51,4286	0	1	0	2
0	0	0	0	25,7143	0	0	0	2
0	0	0	162,857	360	102,857	1	1	2
0	0	0	7,14286	34,2857	0	0	0	2
0	0	0	171,429	334,286	0	1	1	3
0	0	0	52,8571	51,4286	8,57143	0	1	1
0	0	0	25,7143	102,857	0	0	1	1
0	0	0	120	25,7143	0	1	0	2
0	0	0	34,2857	25,7143	0	0	0	2
0	0	0	90	51,4286	0	1	0	2
0	1	1	75	77,1429	0	1	1	2
0	0	0	0	5,71429	0	0	0	2
0	0	0	12,8571	68,5714	0	0	1	2
0	0	0	30	25,7143	0	0	0	1
0	0	0	128,571	205,714	0	1	1	2
0	0	0	45,7143	25,7143	0	0	0	2
0	0	0	5,71429	77,1429	180	0	1	1
0	0	0	17,1429	60	0	0	1	2
0	0	0	111,429	17,1429	0	1	0	1
0	0	0	68,5714	142,143	0	1	1	1
0	0	0	85,7143	17,1429	0	1	0	2
0	1	1	148,571	94,2857	0	1	1	2
0	0	0	47,1429	141,429	25,7143	0	1	3
0	0	0	135,714	16,4286	0	1	0	2
0	0	0	17,1429	17,1429	0	0	0	2
0	0	0	10	8,57143	0	0	0	1
0	0	0	42,8571	54,2857	0	0	0	1
0	0	0	308,571	188,571	0	1	1	2
0	0	0	2,85714	111,429	85,7143	0	1	2
0	0	0	38,5714	50	0	0	0	2
0	0	0	128,571	42,8571	0	1	0	2
1	1	1	360	300	77,1429	1	1	1

0	*1*	*1*	*97,1429*	*120*	*0*	*1*	*1*	*3*
0	*0*	*0*	*42,8571*	*42,8571*	*0*	*0*	*0*	*2*
0	*0*	*0*	*55,7143*	*64,2857*	*0*	*0*	*1*	*1*
0	*0*	*0*	*20*	*17,1429*	*0*	*0*	*0*	*1*
0	*0*	*0*	*120*	*25,7143*	*0*	*1*	*0*	*3*
0	*0*	*0*	*162,857*	*205,714*	*0*	*1*	*1*	*1*
0	*0*	*0*	*25,7143*	*51,4286*	*17,1429*	*0*	*1*	*3*
0	*0*	*1*	*117,143*	*60*	*25,7143*	*1*	*1*	*2*
0	*0*	*0*	*82,8571*	*428,571*	*205,714*	*1*	*1*	*1*
0	*0*	*0*	*22,8571*	*72,8571*	*0*	*0*	*1*	*2*
0	*0*	*0*	*38,5714*	*17,1429*	*25,7143*	*0*	*0*	*2*
0	*0*	*0*	*5,71429*	*8,57143*	*0*	*0*	*0*	*2*
1	*0*	*1*	*1,42857*	*15*	*102,857*	*0*	*1*	*1*
0	*0*	*0*	*20*	*50*	*0*	*0*	*0*	*2*
0	*0*	*0*	*120*	*60*	*0*	*1*	*1*	*2*
0	*1*	*1*	*91,4286*	*25,7143*	*25,7143*	*1*	*0*	*2*
0	*1*	*1*	*60*	*25,7143*	*17,1429*	*1*	*0*	*3*
0	*0*	*0*	*4,28571*	*8,57143*	*0*	*0*	*0*	*1*
0	*0*	*0*	*2,14286*	*25,7143*	*0*	*0*	*0*	*1*
0	*0*	*0*	*17,1429*	*18,5714*	*0*	*0*	*0*	*2*
0	*0*	*0*	*30*	*137,143*	*30*	*0*	*1*	*2*
0	*0*	*0*	*30*	*25,7143*	*0*	*0*	*0*	*2*
0	*0*	*0*	*81,4286*	*162,857*	*0*	*1*	*1*	*2*
0	*0*	*0*	*184,286*	*77,1429*	*8,57143*	*1*	*1*	*2*
0	*0*	*0*	*57,8571*	*120*	*0*	*0*	*1*	*1*
0	*0*	*0*	*30*	*40*	*0*	*0*	*0*	*2*
0	*0*	*0*	*4,28571*	*7,14286*	*0*	*0*	*0*	*2*
0	*0*	*0*	*145,714*	*82,8571*	*0*	*1*	*1*	*2*
0	*0*	*0*	*4,28571*	*51,4286*	*0*	*0*	*0*	*2*
0	*0*	*0*	*5,71429*	*58,5714*	*0*	*0*	*0*	*2*
0	*0*	*0*	*0*	*85,7143*	*0*	*0*	*1*	*2*
0	*1*	*1*	*128,571*	*25,7143*	*0*	*1*	*0*	*3*
0	*0*	*0*	*35,7143*	*25,7143*	*0*	*0*	*0*	*2*
0	*0*	*0*	*194,286*	*257,143*	*0*	*1*	*1*	*3*
0	*0*	*0*	*5,71429*	*48,5714*	*0*	*0*	*0*	*2*
0	*0*	*0*	*54,2857*	*102,857*	*0*	*0*	*1*	*2*
0	*0*	*0*	*34,2857*	*64,2857*	*0*	*0*	*1*	*2*
0	*0*	*0*	*90*	*137,143*	*17,1429*	*1*	*1*	*1*
0	*0*	*0*	*21,4286*	*53,5714*	*0*	*0*	*0*	*1*
0	*0*	*0*	*18,5714*	*30*	*0*	*0*	*0*	*1*
0	*0*	*0*	*0*	*50*	*128,571*	*0*	*1*	*2*
0	*1*	*1*	*145,714*	*25,7143*	*0*	*1*	*0*	*3*
0	*0*	*0*	*20,7143*	*188,571*	*0*	*0*	*1*	*2*
0	*0*	*0*	*150*	*102,857*	*0*	*1*	*1*	*1*
1	*0*	*1*	*23,5714*	*17,1429*	*51,4286*	*0*	*1*	*2*
0	*0*	*0*	*27,8571*	*34,2857*	*0*	*0*	*0*	*1*
0	*0*	*0*	*0*	*8,57143*	*0*	*0*	*0*	*2*
0	*0*	*0*	*34,2857*	*0*	*0*	*0*	*0*	*2*

0	0	0	40,7143	180	0	0	1	3
0	0	0	27,8571	19,2857	0	0	0	2
0	0	0	150	34,2857	0	1	0	2
0	0	0	120	105,714	154,286	1	1	2
0	0	0	60	67,1429	0	1	1	2
0	0	0	55,7143	94,2857	0	0	1	2
0	0	0	42,8571	30	0	0	0	2
0	0	0	64,2857	31,4286	0	1	0	1
0	0	0	32,1429	17,1429	0	0	0	2
0	0	0	60	27,1429	0	1	0	1
0	0	0	102,857	102,857	0	1	1	1
0	1	1	81,4286	102,857	0	1	1	2
0	0	0	31,4286	132,857	0	0	1	1
0	0	0	7,14286	34,2857	0	0	0	1
0	0	0	80	88,5714	0	1	1	1
0	0	0	124,286	128,571	0	1	1	1
0	0	0	72,8571	25,7143	0	1	0	2
0	0	0	0	51,4286	0	0	0	2
0	0	0	282,857	23,5714	0	1	0	2
0	0	0	0	222,857	0	0	1	2
0	0	0	85	102,857	8,57143	1	1	2
0	0	0	85,7143	188,571	0	1	1	2
1	1	1	180	8,57143	85,7143	1	1	2
0	0	0	22,8571	25,7143	0	0	0	2
0	1	1	60	51,4286	0	1	0	2
0	0	0	180	120	0	1	1	2
0	0	0	15	34,2857	0	0	0	2
0	1	1	195	25,7143	0	1	0	2
0	0	0	120	21,4286	0	1	0	1
0	0	0	10,7143	128,571	0	0	1	1
0	0	0	0	188,571	85,7143	0	1	2
0	0	0	52,8571	25,7143	0	0	0	1
0	0	0	325,714	25,7143	0	1	0	2
0	0	0	220,714	42,8571	0	1	0	2
0	0	0	45,7143	34,2857	0	0	0	1
0	0	0	4,28571	45	0	0	0	2
0	1	1	205,714	102,857	17,1429	1	1	1
0	0	0	31,4286	0	0	0	0	2
0	0	0	162,857	162,857	0	1	1	1
0	0	0	92,1429	122,143	0	1	1	2
0	0	0	20	68,5714	25,7143	0	1	1
0	0	0	90	51,4286	0	1	0	1
0	0	0	5,71429	5,71429	0	0	0	1
0	0	0	0	232,857	0	0	1	2
0	0	0	77,1429	34,2857	0	1	0	2
0	0	0	2,14286	25,7143	0	0	0	1
0	0	0	0	248,571	8,57143	0	1	2
0	0	0	173,571	77,1429	0	1	1	1

0	0	0	148,571	68,5714	68,5714	1	1	3
0	0	1	48,5714	32,1429	12,8571	0	0	2
0	0	0	128,571	188,571	0	1	1	2
0	0	0	55,7143	199,286	0	0	1	3
0	0	0	30,7143	42,1429	0	0	0	3
0	1	1	180	120	0	1	1	2
0	0	0	45,7143	128,571	17,8571	0	1	2
0	0	0	35	36,4286	8,57143	0	0	3
0	0	0	197,143	171,429	0	1	1	2
0	0	0	188,571	21,4286	0	1	0	2
0	0	0	124,286	25,7143	0	1	0	2
0	0	0	167,143	12,8571	0	1	0	2
0	0	0	130,714	0	0	1	0	2
0	0	0	32,1429	117,143	0	0	1	2
0	0	0	8,57143	51,4286	154,286	0	1	1
0	0	1	115,714	214,286	0	1	1	2
0	0	0	180	64,2857	0	1	1	2
0	0	0	158,571	85,7143	0	1	1	1
0	0	0	111,429	85,7143	0	1	1	2
0	0	0	34,2857	241,429	60	0	1	2
0	0	1	92,8571	35,7143	34,2857	1	1	2
0	0	0	8,57143	25,7143	0	0	0	2
0	0	0	162,857	30	0	1	0	2
0	0	0	210	248,571	43,7143	1	1	2
0	0	0	7,14286	87,8571	0	0	1	1
0	0	0	100	248,571	0	1	1	2
0	0	0	14,2857	25,7143	0	0	0	1
0	0	0	0	60	0	0	1	2
0	0	0	21,4286	128,571	0	0	1	1
0	0	0	85,7143	102,857	0	1	1	2
0	0	0	162,857	77,1429	0	1	1	1
0	0	0	0	233,571	0	0	1	2
0	0	0	195	107,143	8,57143	1	1	2
0	0	0	0	5,71429	0	0	0	2
0	0	0	34,2857	0	0	0	0	2
0	0	0	0	21,4286	0	0	0	2
0	0	0	139,286	184,286	0	1	1	2
0	0	0	8,57143	49,2857	0	0	0	2
0	0	0	131,429	171,429	0	1	1	1
0	0	0	0	188,571	0	0	1	2
0	0	0	42,8571	300	17,1429	0	1	1
0	0	0	171,429	111,429	0	1	1	2
0	0	0	91,4286	185,714	0	1	1	1
0	0	1	110	60	51,4286	1	1	1
0	0	0	34,2857	0	0	0	0	2
0	0	0	12,8571	42,8571	0	0	0	2
0	0	0	3,57143	8,57143	0	0	0	2
0	0	0	51,4286	50	0	0	0	2

0	0	1	81,4286	17,1429	17,1429	1	0	2
0	0	0	38,5714	0	0	0	0	2
0	0	0	20	82,8571	0	0	1	2
0	0	0	10	32,8571	0	0	0	2
0	0	0	60,7143	25,7143	0	1	0	1
0	0	0	85,7143	162,857	0	1	1	2
0	1	1	180	60	0	1	1	2
0	0	0	171,429	32,1429	0	1	0	2
0	0	0	11,4286	25,7143	0	0	0	2
0	0	0	51,4286	30	0	0	0	1
0	0	0	132,857	34,2857	25,7143	1	1	1
0	0	0	25,7143	42,8571	0	0	0	1
0	0	0	68,5714	51,4286	0	1	0	2
0	0	0	185,714	94,2857	8,57143	1	1	2
0	0	0	32,8571	60	12,8571	0	1	2
0	0	0	35,7143	25,7143	0	0	0	2
0	0	0	25,7143	51,4286	21,4286	0	1	1
0	0	0	12,1429	42,8571	0	0	0	1
0	0	0	7,14286	34,2857	0	0	0	2
0	0	0	82,8571	17,1429	0	1	0	1
0	0	0	120	17,1429	0	1	0	1
0	0	0	360	200	0	1	1	2
0	0	0	81,4286	25,7143	6,42857	1	0	2
0	0	0	188,571	85,7143	0	1	1	2
0	0	0	25,7143	60	0	0	1	2
0	0	0	1,42857	34,2857	0	0	0	2
0	0	0	17,1429	51,4286	0	0	0	3
0	1	1	80,5714	77,1429	51,4286	1	1	2
0	0	0	130	308,571	25,7143	1	1	2
0	0	0	107,143	240	0	1	1	2
0	1	1	270	171,429	8,57143	1	1	2
0	0	0	42,8571	51,4286	0	0	0	2
0	0	0	60	51,4286	0	1	0	1
0	0	0	21,4286	162,857	0	0	1	3
0	0	0	12,8571	21,4286	0	0	0	2
0	0	0	145,714	188,571	0	1	1	2
0	0	0	85,7143	25,7143	0	1	0	3
0	0	0	12,1429	154,286	0	0	1	2
0	0	0	71,4286	40	0	1	0	1
0	0	0	0	154,286	0	0	1	2
0	1	1	205,714	94,2857	0	1	1	3
0	0	0	6,42857	42,8571	0	0	0	2
0	0	0	88,5714	42,8571	0	1	0	2
0	1	1	261,429	227,143	0	1	1	2
0	0	0	102,857	68,5714	25,7143	1	1	2
0	0	0	154,286	137,143	180	1	1	3
0	0	0	8,57143	42,8571	8,57143	0	0	2
0	0	0	22,8571	17,1429	17,1429	0	0	2

0	*0*	*0*	*128,571*	*248,571*	*0*	*1*	*1*	*2*
0	*0*	*0*	*7,14286*	*154,286*	*17,1429*	*0*	*1*	*2*
0	*0*	*0*	*68,5714*	*67,1429*	*0*	*1*	*1*	*2*
0	*0*	*0*	*30*	*227,143*	*0*	*0*	*1*	*3*
0	*0*	*0*	*4,28571*	*34,2857*	*0*	*0*	*0*	*2*
0	*0*	*0*	*10,7143*	*70,7143*	*0*	*0*	*1*	*2*
0	*0*	*0*	*174,286*	*25,7143*	*0*	*1*	*0*	*2*
0	*0*	*0*	*57,1429*	*12,8571*	*0*	*0*	*0*	*2*
0	*0*	*0*	*20*	*68,5714*	*8,57143*	*0*	*1*	*2*
0	*0*	*0*	*160*	*51,4286*	*0*	*1*	*0*	*2*
0	*0*	*0*	*40,7143*	*42,8571*	*0*	*0*	*0*	*2*
0	*0*	*0*	*55,7143*	*17,1429*	*0*	*0*	*0*	*2*
0	*0*	*0*	*82,8571*	*201,429*	*0*	*1*	*1*	*2*
0	*1*	*1*	*60*	*51,4286*	*0*	*1*	*0*	*1*
0	*0*	*0*	*155,714*	*25,7143*	*12,8571*	*1*	*0*	*3*
0	*0*	*0*	*25,7143*	*34,2857*	*0*	*0*	*0*	*1*
0	*0*	*0*	*34,2857*	*17,1429*	*0*	*0*	*0*	*1*
0	*0*	*0*	*28,5714*	*34,2857*	*8,57143*	*0*	*0*	*1*
0	*0*	*0*	*102,857*	*180*	*2,85714*	*1*	*1*	*2*
0	*0*	*0*	*30*	*25,7143*	*0*	*0*	*0*	*1*
0	*0*	*0*	*58,5714*	*42,8571*	*0*	*0*	*0*	*2*
0	*0*	*0*	*202,857*	*128,571*	*0*	*1*	*1*	*2*
0	*0*	*0*	*31,4286*	*34,2857*	*0*	*0*	*0*	*2*
0	*0*	*0*	*0*	*42,8571*	*0*	*0*	*0*	*1*
0	*0*	*0*	*171,429*	*85,7143*	*25,7143*	*1*	*1*	*1*
0	*0*	*0*	*0*	*102,857*	*0*	*0*	*1*	*2*
0	*0*	*0*	*85,7143*	*8,57143*	*68,5714*	*1*	*1*	*2*
0	*0*	*0*	*12*	*190*	*17,1429*	*0*	*1*	*2*
0	*0*	*0*	*102,857*	*154,286*	*0*	*1*	*1*	*2*
0	*0*	*0*	*139,286*	*85,7143*	*0*	*1*	*1*	*3*
0	*0*	*0*	*45*	*8,57143*	*0*	*0*	*0*	*2*
0	*0*	*0*	*20*	*47,1429*	*0*	*0*	*0*	*1*
0	*1*	*1*	*214,286*	*111,429*	*0*	*1*	*1*	*2*
0	*0*	*0*	*45*	*25,7143*	*0*	*0*	*0*	*2*
0	*0*	*0*	*25,7143*	*35*	*0*	*0*	*0*	*2*
0	*0*	*1*	*111,429*	*75*	*0*	*1*	*1*	*1*
0	*0*	*0*	*124,286*	*25,7143*	*0*	*1*	*0*	*2*
0	*0*	*0*	*36,4286*	*30*	*0*	*0*	*0*	*1*
0	*0*	*0*	*25,7143*	*25,7143*	*8,57143*	*0*	*0*	*1*
0	*0*	*0*	*132,857*	*34,2857*	*0*	*1*	*0*	*2*
0	*0*	*0*	*98,5714*	*94,2857*	*0*	*1*	*1*	*2*
0	*0*	*0*	*180*	*141,429*	*77,1429*	*1*	*1*	*2*
0	*1*	*1*	*205,714*	*180*	*8,57143*	*1*	*1*	*1*
0	*1*	*1*	*60*	*25,7143*	*0*	*1*	*0*	*3*
0	*0*	*0*	*128,571*	*128,571*	*128,571*	*1*	*1*	*2*
0	*0*	*0*	*8,57143*	*22,8571*	*0*	*0*	*0*	*3*
0	*0*	*0*	*8,57143*	*102,857*	*0*	*0*	*1*	*2*
0	*0*	*0*	*12,8571*	*308,571*	*34,2857*	*0*	*1*	*2*

0	0	0	34,2857	205,714	0	0	1	2
0	0	0	25,7143	15	0	0	0	1
0	1	1	248,571	60	0	1	1	2
0	0	0	42,8571	142,857	0	0	1	2
0	0	0	35,7143	64,2857	0	0	1	2
0	1	1	171,429	190	0	1	1	1
0	0	0	124,286	104,286	180	1	1	2
0	0	0	30	25,7143	0	0	0	2
0	0	0	158,571	120	0	1	1	1
0	0	0	0	120	34,2857	0	1	2
0	0	0	57,8571	17,1429	0	0	0	1
0	0	0	30	8,57143	0	0	0	2
0	0	0	8,57143	25,7143	0	0	0	1
0	0	0	8,57143	83,5714	0	0	1	2
0	0	0	60	94,2857	0	1	1	1
0	0	0	128,571	235,714	0	1	1	1
0	0	0	12,8571	4,28571	0	0	0	2
0	0	0	22,8571	0	28,5714	0	0	2
0	0	0	17,1429	27,8571	1,42857	0	0	2
0	0	0	17,1429	34,2857	0	0	0	2
0	0	0	34,2857	100	0	0	1	1
0	0	0	72,8571	122,857	0	1	1	2
0	0	0	197,143	17,1429	0	1	0	2
0	0	0	42,1429	34,2857	0	0	0	2
0	1	1	120	90	8,57143	1	1	3
0	0	0	68,5714	42,8571	0	1	0	2
0	0	0	20	0	0	0	0	2
0	0	0	5,71429	17,1429	0	0	0	2
0	0	0	31,4286	34,2857	0	0	0	2
0	0	0	197,143	85,7143	23,5714	1	1	2
0	0	0	40,7143	167,143	77,1429	0	1	2
0	0	0	180	42,8571	0	1	0	2
0	1	1	210	57,1429	0	1	0	2
0	0	0	188,571	192,857	0	1	1	3
0	0	0	17,1429	25,7143	0	0	0	1
0	0	0	175,714	231,429	17,1429	1	1	1
0	0	0	128,571	154,286	102,857	1	1	2
0	0	0	42,8571	1,42857	0	0	0	2
0	0	0	12,8571	25,7143	0	0	0	2
0	0	0	141,429	20,7143	0	1	0	3
0	0	0	2,85714	34,2857	0	0	0	1
0	0	0	94,2857	90	92,1429	1	1	2
0	0	0	42,8571	25,7143	0	0	0	2
0	0	0	171,429	154,286	0	1	1	2
0	0	0	135,714	267,857	0	1	1	2
0	0	0	12,8571	12,8571	0	0	0	2
0	0	0	51,4286	68,5714	0	0	1	2
0	0	0	8,57143	25,7143	0	0	0	2

0	0	0	51,4286	34,2857	34,2857	0	1	1
0	0	0	51,4286	94,2857	0	0	1	2
0	0	0	52,8571	34,2857	0	0	0	2
0	0	0	15	0	0	0	0	1
0	0	0	111,429	180	0	1	1	3
0	0	0	42,8571	64,2857	0	0	1	2
0	0	0	15,7143	68,5714	0	0	1	2
0	0	0	17,1429	35,7143	0	0	0	2
0	1	1	77,1429	51,4286	0	1	0	1
0	0	0	12,8571	34,2857	0	0	0	2
0	0	0	143,571	30	17,1429	1	0	2
0	0	0	10,7143	17,1429	0	0	0	3
0	0	0	4,28571	17,1429	0	0	0	3
0	0	0	34,2857	17,1429	0	0	0	2
0	0	0	148,571	231,429	77,1429	1	1	2
0	1	1	188,571	17,1429	17,1429	1	0	3
0	0	0	0	17,1429	0	0	0	2
0	0	0	30	48,5714	0	0	0	2
0	0	0	33,5714	34,2857	0	0	0	2
0	0	0	64,2857	77,1429	0	1	1	2
1	1	1	184,286	291,429	17,1429	1	1	2
0	0	0	24,2857	94,2857	17,1429	0	1	2
0	0	0	17,1429	68,5714	0	0	1	2
0	0	0	85,7143	154,286	0	1	1	2
0	0	0	6,42857	0	0	0	0	1
0	0	0	248,571	94,2857	12,8571	1	1	2
0	1	1	120	51,4286	0	1	0	2
0	0	0	57,1429	42,8571	0	0	0	1
0	1	1	107,143	37,1429	51,4286	1	1	3
0	0	0	10,7143	17,1429	0	0	0	1
0	1	1	180	180	0	1	1	2
0	0	0	38,5714	0	0	0	0	1
0	1	1	128,571	25,7143	0	1	0	2
0	0	0	85,7143	222,857	0	1	1	2
0	0	0	10,7143	120	0	0	1	2
0	0	0	167,143	188,571	0	1	1	2
0	0	0	38,5714	47,1429	0	0	0	1
0	0	0	167,143	334,286	8,57143	1	1	1
0	0	0	35,7143	90	0	0	1	1
0	0	0	12,8571	180	0	0	1	2
0	0	0	85,7143	120	8,57143	1	1	3
0	0	0	16,4286	25,7143	0	0	0	2
0	0	0	137,143	120	8,57143	1	1	2
0	0	0	35	85,7143	0	0	1	3
0	0	0	142,857	175,714	0	1	1	2
0	0	0	8,57143	85,7143	0	0	1	1
0	0	0	5,71429	17,1429	0	0	0	2
0	0	0	4,28571	107,143	0	0	1	1

0	0	0	28,5714	25	0	0	0	1
1	0	1	20	154,286	0	0	1	2
0	0	0	68,5714	154,286	0	1	1	2
0	0	0	147,857	125,714	0	1	1	2
0	0	0	51,4286	154,286	0	0	1	2
0	1	1	60	145,714	0	1	1	2
0	0	0	185,714	94,2857	34,2857	1	1	2
0	0	0	19,2857	0	0	0	0	1
0	0	0	58,5714	12,8571	0	0	0	2
0	0	0	0	19,2857	0	0	0	2
0	0	0	0	128,571	42,8571	0	1	2
0	0	0	42,8571	231,429	17,1429	0	1	3
0	0	0	32,1429	25,7143	0	0	0	2
0	0	0	51,4286	41,4286	17,1429	0	0	2
0	0	0	60	38,5714	17,1429	1	0	2
0	0	0	0	145,714	0	0	1	1
0	0	0	21,4286	94,2857	0	0	1	3
0	0	0	8,57143	214,286	0	0	1	2
0	0	0	60	17,1429	0	1	0	2
0	0	0	18,5714	60	8,57143	0	1	1
0	0	0	42,8571	154,286	0	0	1	2
0	0	0	60	267,143	137,143	1	1	2
0	0	0	21,4286	42,8571	0	0	0	2
0	0	0	45,7143	42,8571	17,1429	0	1	2
0	0	0	0	90	8,57143	0	1	2
0	0	0	0	131,429	34,2857	0	1	2
0	0	0	17,1429	94,2857	0	0	1	2
0	0	0	4,28571	76,4286	0	0	1	2
0	0	0	47,1429	21,4286	0	0	0	1
0	0	0	10,7143	25,7143	0	0	0	2
0	0	0	6,42857	124,286	0	0	1	2
0	0	0	10	53,5714	8,57143	0	1	2
0	0	0	52,8571	188,571	0	0	1	1
0	0	0	31,4286	17,1429	0	0	0	2
0	0	0	87,8571	17,1429	25,7143	1	0	2
0	0	0	8,57143	8,57143	0	0	0	2
0	0	0	162,857	49,2857	0	1	0	1
0	0	0	30	60	0	0	1	2
0	1	1	150	60	0	1	1	2
0	0	0	102,857	171,429	0	1	1	1
0	0	0	102,857	8,57143	0	1	0	2
0	0	0	143,571	248,571	25,7143	1	1	1
0	0	0	8,57143	90	25,7143	0	1	2
0	0	0	25,7143	27,8571	0	0	0	1
0	0	0	90	132,857	0	1	1	2
0	0	0	72,8571	61,4286	4,28571	1	1	2
0	0	0	25,7143	30	0	0	0	2
0	0	0	30	68,5714	0	0	1	3

0	0	0	42,8571	40,7143	0	0	0	3
0	0	0	0	0	0	0	0	1
0	0	0	81,4286	205,714	25,7143	1	1	2
0	0	0	21,4286	154,286	0	0	1	1
0	0	0	167,143	305,714	0	1	1	2
0	1	1	137,143	22,8571	0	1	0	3
0	0	0	12,8571	25,7143	0	0	0	1
0	0	0	8,57143	138,571	0	0	1	3
0	0	0	111,429	270	0	1	1	2
0	0	0	120	102,857	0	1	1	2
0	0	0	8,57143	85,7143	0	0	1	2
0	0	0	12,8571	157,143	0	0	1	2
0	0	0	49,2857	60	0	0	1	2
0	0	0	112,857	30	4,28571	1	0	2
0	0	0	180	120	0	1	1	2
0	0	1	45	47,1429	17,1429	0	1	2
0	0	0	145,714	180	0	1	1	2
0	0	0	21,4286	25,7143	0	0	0	2
0	0	0	70	8,57143	0	1	0	2
0	0	0	171,429	34,2857	0	1	0	2
0	0	0	6,42857	180	0	0	1	3
0	0	0	85,7143	21,4286	0	1	0	2
0	0	0	180	197,143	0	1	1	3
0	0	0	10,7143	17,1429	0	0	0	1
0	1	1	160	81,4286	0	1	1	1
0	0	0	17,1429	51,4286	0	0	0	2
0	0	0	68,5714	34,2857	0	1	0	2
0	0	0	26,4286	158,571	17,1429	0	1	2
0	0	0	197,143	128,571	0	1	1	1
0	0	0	0	171,429	0	0	1	2
0	0	0	11,4286	154,286	0	0	1	2
0	0	0	212,857	51,4286	0	1	0	2
0	0	0	60	17,1429	0	1	0	2
0	0	0	40	50	0	0	0	1
0	1	1	74,2857	81,4286	0	1	1	2
0	0	0	60	191,429	0	1	1	2
0	1	1	60	34,2857	0	1	0	3
0	0	0	47,8571	102,857	111,429	0	1	2
0	0	0	34,2857	42,8571	51,4286	0	1	2
0	0	0	77,1429	51,4286	42,8571	1	1	2
0	0	0	17,1429	17,1429	12,8571	0	0	1
0	0	0	24,2857	77,1429	0	0	1	2
0	0	1	49,2857	102,857	34,2857	0	1	1
0	0	0	188,571	145,714	0	1	1	3
0	0	0	2,85714	34,2857	0	0	0	2
0	0	0	42,8571	68,5714	0	0	1	2
0	0	0	139,286	154,286	25,7143	1	1	1
0	0	0	7,14286	17,1429	0	0	0	2

0	0	0	17,1429	21,4286	0	0	0	2
0	0	0	145,714	21,4286	0	1	0	2
0	0	0	35,7143	51,4286	0	0	0	1
0	0	0	28,5714	192,857	0	0	1	2
0	0	0	27,1429	51,4286	0	0	0	2
0	0	0	53,5714	25,7143	0	0	0	2
0	1	1	94,2857	77,1429	8,57143	1	1	1
0	0	0	109,286	300	0	1	1	2
0	0	0	180	64,2857	0	1	1	2
0	0	0	174,286	68,5714	0	1	1	2
0	0	0	12,8571	0	0	0	0	1
0	0	0	150	42,8571	0	1	0	1
0	0	0	7,14286	34,2857	0	0	0	2
0	0	0	101,429	214,286	51,4286	1	1	2
0	0	0	45	8,57143	0	0	0	2
0	0	0	42,8571	51,4286	25,7143	0	1	2
0	0	0	114,286	42,8571	0	1	0	2
0	0	0	47,1429	94,2857	0	0	1	2
0	0	0	51,4286	210	12,8571	0	1	2
0	0	0	98,5714	35,7143	8,57143	1	0	2
0	0	0	162,857	60	0	1	1	2
0	0	0	102,857	77,1429	42,8571	1	1	2
0	0	0	14,2857	85,7143	0	0	1	2
0	0	0	51,4286	51,4286	0	0	0	2
0	0	0	107,143	120	17,1429	1	1	2
0	0	0	40	100	0	0	1	2
0	0	0	42,8571	222,857	0	0	1	2
0	0	0	154,286	51,4286	77,1429	1	1	2
0	0	0	2,85714	188,571	0	0	1	3
0	0	0	17,1429	42,8571	15	0	0	2
0	0	1	237,857	197,143	98,5714	1	1	1
0	0	0	120	141,429	0	1	1	2
0	0	0	231,429	205,714	0	1	1	1
0	0	0	14,2857	85,7143	0	0	1	2
0	0	0	24,2857	51,4286	0	0	0	2
0	0	0	51,4286	0	34,2857	0	0	2
0	0	0	102,857	171,429	17,1429	1	1	1
0	0	1	90	60	0	1	1	2
0	1	1	205,714	145,714	0	1	1	2
0	1	1	210	85,7143	0	1	1	1
0	0	0	12,8571	0	0	0	0	2
0	0	0	25,7143	171,429	0	0	1	2
0	0	0	0	5,71429	0	0	0	2
0	0	0	2,85714	25,7143	0	0	0	2
0	0	0	114,286	60	0	1	1	1
0	0	0	17,1429	68,5714	0	0	1	2
0	0	0	4,28571	257,143	0	0	1	2
0	0	0	180	321,429	0	1	1	1

0	0	0	55	61,4286	0	0	1	2
0	0	0	5,71429	180	0	0	1	2
0	0	0	8,57143	17,1429	0	0	0	2
0	0	0	141,429	58,5714	0	1	0	1
0	0	0	0	21,4286	0	0	0	2
0	0	0	158,571	241,429	0	1	1	2
0	1	1	175,714	60	0	1	1	1
0	0	0	78,5714	25,7143	0	1	0	2
0	0	0	34,2857	205,714	34,2857	0	1	2
0	0	0	154,286	51,4286	0	1	0	2
0	0	0	8,57143	0	0	0	0	1
0	1	1	300	120	85,7143	1	1	2
0	0	0	171,429	60	0	1	1	2
0	0	0	128,571	205,714	0	1	1	1
0	0	0	51,4286	42,8571	0	0	0	1
0	0	0	12,8571	25,7143	0	0	0	2
0	0	0	4,28571	17,1429	0	0	0	2
0	0	0	0	128,571	0	0	1	2
0	0	0	80	85,7143	0	1	1	2
0	0	0	10,7143	17,1429	8,57143	0	0	1
0	0	0	65,7143	102,857	0	1	1	1
0	0	0	2,85714	17,1429	0	0	0	1
0	0	0	42,8571	34,2857	0	0	0	1
0	0	0	0	51,4286	0	0	0	2
0	0	0	11,4286	7,14286	0	0	0	2
0	0	0	222,857	188,571	34,2857	1	1	2
0	0	0	25,7143	25,7143	0	0	0	1
0	0	1	201,429	60	0	1	1	2
0	0	0	8,57143	128,571	30	0	1	1
0	0	0	137,143	120	25,7143	1	1	2
0	0	0	4,28571	109,286	0	0	1	2
0	0	0	17,1429	70	0	0	1	2
0	0	0	162,857	248,571	0	1	1	1
0	0	0	124,286	80	0	1	1	2
0	0	0	30	21,4286	0	0	0	1
0	0	0	188,571	145,714	0	1	1	2
0	0	0	85,7143	17,1429	0	1	0	2
0	0	0	14,2857	51,4286	0	0	0	1
0	0	0	158,571	57,1429	0	1	0	2
0	0	0	164,286	280,714	0	1	1	1
0	0	0	7,14286	42,8571	0	0	0	2
0	0	0	153,571	60	0	1	1	2
0	0	0	8,57143	102,857	0	0	1	2
0	0	0	154,286	51,4286	0	1	0	2
0	0	0	17,1429	0	0	0	0	2
0	0	0	36,4286	42,8571	0	0	0	2
0	0	0	57,1429	0	0	0	0	2
0	0	0	154,286	214,286	25,7143	1	1	2

0	0	0	282,857	34,2857	0	1	0	2
0	0	0	68,5714	34,2857	0	1	0	1
0	0	0	72,8571	8,57143	0	1	0	1
0	0	0	102,857	211,429	0	1	1	3
0	0	0	14,2857	214,286	0	0	1	2
0	0	0	21,4286	34,2857	25,7143	0	1	1
0	0	0	34,2857	128,571	0	0	1	2
0	0	0	0	231,429	51,4286	0	1	2
0	0	0	87,1429	123,571	0	1	1	2
0	0	1	85,7143	128,571	0	1	1	2
0	0	0	8,57143	0	0	0	0	1
0	1	1	124,286	85,7143	0	1	1	1
0	0	0	128,571	148,571	25,7143	1	1	1
0	0	0	162,857	102,857	0	1	1	2
0	0	0	112,857	75	0	1	1	1
0	0	0	107,143	115,714	0	1	1	2
0	0	0	8,57143	12,8571	0	0	0	2
0	1	1	180	51,4286	0	1	0	2
0	0	0	50,7143	60	0	0	1	2
0	1	1	207,857	17,1429	0	1	0	3
0	1	1	274,286	120	0	1	1	1
0	0	0	12,8571	51,4286	51,4286	0	1	2
0	0	0	42,8571	77,1429	0	0	1	2
0	0	0	34,2857	34,2857	0	0	0	2
0	0	0	55,7143	25,7143	0	0	0	2
0	0	0	21,4286	60	0	0	1	2
0	0	0	0	98,5714	0	0	1	1
0	0	0	22,8571	51,4286	0	0	0	2
0	0	0	154,286	60	0	1	1	2
0	0	0	25	51,4286	0	0	0	1
0	0	0	32,8571	128,571	0	0	1	2
0	0	0	32,8571	184,286	17,1429	0	1	2
0	1	1	162,857	154,286	0	1	1	1
0	0	0	182,857	77,1429	0	1	1	2
0	0	0	85,7143	308,571	102,857	1	1	2
0	0	0	158,571	133,571	0	1	1	2
0	0	0	22,8571	141,429	0	0	1	2
0	0	0	64,2857	38,5714	0	1	0	2
0	0	0	25,7143	240	102,857	0	1	1
0	0	0	162,857	142,857	0	1	1	3
0	0	0	51,4286	25,7143	0	0	0	1
0	0	1	214,286	34,2857	42,8571	1	1	2
0	0	0	55,7143	60	0	0	1	3
0	0	0	60	120	109,286	1	1	2
0	0	0	34,2857	17,1429	0	0	0	2
0	0	0	120	85,7143	34,2857	1	1	2
0	0	0	10	42,8571	0	0	0	2
0	0	0	51,4286	17,1429	0	0	0	2

0	0	0	188,571	394,286	0	1	1	2
0	0	0	17,1429	47,1429	0	0	0	2
0	0	0	39,2857	24,2857	0	0	0	2
0	0	0	72,8571	214,286	17,1429	1	1	2
0	0	0	94,2857	60	0	1	1	2
0	0	0	0	77,1429	0	0	1	1
0	0	0	22,8571	197,143	8,57143	0	1	2
0	0	0	27,8571	25,7143	17,1429	0	0	1
0	0	0	11,4286	8,57143	0	0	0	2
0	0	0	22,1429	0	0	0	0	2
0	0	0	17,1429	51,4286	0	0	0	2
0	0	0	35,7143	17,1429	0	0	0	3
0	0	0	47,1429	25,7143	0	0	0	2
0	0	0	140	8,57143	0	1	0	1
0	0	0	51,4286	51,4286	0	0	0	2
0	0	0	137,143	78,5714	0	1	1	2
0	0	0	42,8571	169,286	0	0	1	2
0	0	0	14,2857	15	0	0	0	2
0	0	0	12,8571	34,2857	0	0	0	1
0	0	0	38,5714	85,7143	0	0	1	2
0	0	0	42,8571	102,857	0	0	1	2
0	0	0	0	17,1429	0	0	0	2
0	0	0	148,571	171,429	25,7143	1	1	2
0	0	0	8,57143	25,7143	0	0	0	2
0	0	0	124,286	60	8,57143	1	1	2
0	0	0	60	94,2857	0	1	1	1
0	1	1	68,5714	77,1429	0	1	1	2
0	0	0	57,1429	120	2,85714	0	1	2
0	0	0	158,571	180	0	1	1	2
0	0	0	91,4286	102,857	0	1	1	1
0	0	0	10,7143	158,571	0	0	1	2
0	0	0	64,2857	17,1429	0	1	0	2
0	0	0	81,4286	102,857	8,57143	1	1	2
0	0	0	161,429	124,286	34,2857	1	1	2
0	0	0	45,7143	132,857	0	0	1	1
0	0	0	17,1429	42,8571	0	0	0	3
0	0	0	55,7143	102,857	0	0	1	1
0	0	0	42,8571	57,1429	0	0	0	2
0	0	0	11,4286	85,7143	0	0	1	2
0	0	0	14,2857	34,2857	0	0	0	3
0	0	0	55,7143	17,1429	0	0	0	3
0	1	1	180	77,1429	0	1	1	2
0	0	0	157,143	261,429	25,7143	1	1	2
0	0	0	20	17,8571	0	0	0	1
0	0	0	120	25,7143	0	1	0	1
0	0	0	85,7143	42,8571	0	1	0	2
0	0	0	110	222,857	128,571	1	1	2
0	0	1	85,7143	102,857	42,8571	1	1	1

0	0	0	15	0	0	0	0	2
0	0	0	7,14286	158,571	19,2857	0	1	2
0	0	0	0	0	0	0	0	2
0	0	0	85,7143	94,2857	0	1	1	1
0	0	0	10	17,1429	42,8571	0	1	2
0	0	0	231,429	128,571	0	1	1	1
0	0	0	27,8571	60	0	0	1	2
0	0	0	158,571	51,4286	0	1	0	2
0	0	0	38,5714	25,7143	8,57143	0	0	1
0	0	0	175,714	222,857	171,429	1	1	2
0	0	0	102,857	334,286	0	1	1	2
0	0	0	222,857	51,4286	0	1	0	2
0	0	0	174,286	0	0	1	0	2
0	0	0	131,429	257,143	0	1	1	2
0	0	0	21,4286	0	0	0	0	2
0	0	1	210	105,714	8,57143	1	1	2
0	0	0	111,429	60	0	1	1	2
0	0	0	0	274,286	0	0	1	2
0	0	0	17,1429	34,2857	0	0	0	2
0	0	0	77,1429	17,1429	0	1	0	2
0	0	0	17,1429	34,2857	25,7143	0	1	2
0	0	0	0	102,857	0	0	1	2
0	1	1	70	115,714	51,4286	1	1	2
0	0	0	50	8,57143	0	0	0	3
0	0	0	53,5714	40	0	0	0	1
0	0	0	7,85714	158,571	0	0	1	1
0	0	0	111,429	40	0	1	0	2
1	1	1	342,857	171,429	34,2857	1	1	1
0	0	0	88,5714	25,7143	0	1	0	2
0	0	0	11,4286	85,7143	0	0	1	1
0	0	0	128,571	128,571	0	1	1	1
0	0	0	60	17,1429	0	1	0	2
0	0	0	150	102,857	0	1	1	1
0	0	0	0	162,857	0	0	1	2
0	0	0	0	0	0	0	0	2
0	0	0	100	51,4286	25,7143	1	1	2
0	0	0	14,2857	25,7143	0	0	0	2
0	0	0	120	137,143	85,7143	1	1	2
0	0	0	2,14286	25,7143	0	0	0	2
0	0	0	64,2857	49,2857	0	1	0	1
0	0	0	134,286	131,429	0	1	1	2
0	0	0	161,429	300	8,57143	1	1	2
0	0	0	53,5714	78,5714	0	0	1	2
0	0	0	64,2857	162,857	102,857	1	1	1
0	0	0	128,571	55,7143	7,14286	1	1	2
0	0	0	77,1429	181,429	0	1	1	3
0	0	0	0	102,857	25,7143	0	1	2
0	0	1	74,2857	60	0	1	1	1

0	0	0	15	4,28571	0	0	0	1
0	0	0	240	205,714	0	1	1	2
0	0	0	97,8571	34,2857	0	1	0	2
1	0	1	40	85,7143	111,429	0	1	2
0	0	0	10,7143	77,1429	4,28571	0	1	2
1	1	1	90	17,1429	51,4286	1	1	1
0	0	0	18,5714	42,8571	5,71429	0	0	2
0	0	0	0	30	0	0	0	1
0	1	1	141,429	308,571	0	1	1	2
0	0	0	128,571	291,429	68,5714	1	1	2
0	0	0	97,1429	94,2857	0	1	1	1
0	1	1	150	47,1429	0	1	0	2
0	0	0	197,143	68,5714	0	1	1	2
0	0	0	8,57143	188,571	0	0	1	2
0	0	0	21,4286	100	25,7143	0	1	2
0	0	0	49,2857	120	0	0	1	1
0	0	0	158,571	257,143	0	1	1	1
0	0	0	22,8571	214,286	102,857	0	1	2
0	0	0	167,143	205,714	0	1	1	2
0	0	0	167,143	25,7143	0	1	0	1
0	0	0	87,8571	66,4286	0	1	1	2
0	0	0	25,7143	85,7143	0	0	1	2
0	0	0	77,1429	25,7143	0	1	0	1
0	0	0	0	51,4286	0	0	0	1
0	1	1	74,2857	15	12,8571	1	0	2
0	0	0	64,2857	17,1429	0	1	0	2
0	0	0	30,7143	51,4286	12,8571	0	1	1
0	0	0	154,286	42,8571	0	1	0	3
0	0	0	60	51,4286	0	1	0	2
0	0	0	0	180	0	0	1	1
0	0	0	188,571	0	15	1	0	2
0	1	1	218,571	51,4286	0	1	0	2
0	0	0	8,57143	77,1429	0	0	1	2
0	1	1	77,1429	34,2857	0	1	0	1
0	0	0	14,2857	24,2857	4,28571	0	0	2
0	0	0	51,4286	38,5714	0	0	0	1
0	0	0	0	104,286	128,571	0	1	2
0	0	0	150	120	0	1	1	2
0	0	0	7,14286	42,8571	0	0	0	2
0	0	0	10,7143	17,1429	0	0	0	2
0	0	0	10	38,5714	25,7143	0	1	1
0	0	0	38,5714	188,571	0	0	1	2
0	0	0	68,5714	17,1429	0	1	0	1
0	0	0	38,5714	34,2857	0	0	0	1
0	0	0	21,4286	152,143	0	0	1	1
0	1	1	214,286	214,286	25,7143	1	1	2
0	0	0	85,7143	34,2857	0	1	0	2
0	0	0	30	25,7143	0	0	0	2

0	0	0	17,1429	17,1429	0	0	0	2
0	1	1	240	17,1429	0	1	0	1
0	0	1	192,857	90	34,2857	1	1	1
0	0	1	42,8571	51,4286	77,1429	0	1	2
0	0	0	51,4286	4,28571	0	0	0	3
0	0	0	77,1429	120	42,8571	1	1	2
0	0	1	51,4286	151,429	51,4286	0	1	2
0	0	0	44,2857	175,714	0	0	1	2
0	0	0	17,1429	30	0	0	0	1
0	0	0	85,7143	25,7143	0	1	0	2
0	0	0	32,1429	4,28571	17,1429	0	0	2
0	0	0	25,7143	68,5714	8,57143	0	1	1
0	0	0	17,1429	95,7143	0	0	1	2
0	0	0	60	175,714	0	1	1	2
0	0	0	0	120	0	0	1	2
0	0	0	31,4286	42,8571	0	0	0	2
0	0	0	96,4286	25,7143	0	1	0	2
0	0	0	85,7143	25,7143	0	1	0	3
0	0	0	25,7143	17,1429	0	0	0	2
0	0	0	35,7143	60	0	0	1	1
0	0	0	21,4286	42,8571	0	0	0	2
0	0	0	77,1429	150	0	1	1	1
0	0	0	0	74,2857	0	0	1	2
0	0	0	12,8571	68,5714	0	0	1	2
0	0	0	97,1429	17,1429	0	1	0	2
0	0	0	38,5714	32,8571	0	0	0	2
0	0	0	47,1429	64,2857	0	0	1	2
0	0	0	60	107,143	0	1	1	2
0	0	0	21,4286	158,571	17,1429	0	1	2
0	0	0	37,1429	25,7143	38,5714	0	1	2
0	0	0	248,571	132,857	154,286	1	1	2
0	0	0	134,286	85,7143	0	1	1	2
0	0	0	20	51,4286	0	0	0	2
0	0	0	17,1429	51,4286	0	0	0	2
0	0	0	8,57143	0	0	0	0	1
0	0	0	12,8571	77,1429	0	0	1	2
0	0	0	27,8571	17,1429	0	0	0	3
0	0	0	137,143	111,429	0	1	1	1
0	0	0	77,1429	111,429	0	1	1	2
0	0	0	10	8,57143	0	0	0	1
0	0	0	70	60	0	1	1	1
0	0	1	55	42,8571	54,2857	0	1	2
0	0	0	0	145,714	0	0	1	2
0	0	0	102,857	8,57143	0	1	0	2
0	0	0	21,4286	17,1429	0	0	0	2
0	0	0	184,286	154,286	30	1	1	3
0	0	0	77,1429	120	0	1	1	2
0	0	0	105,714	60	17,1429	1	1	2

0	0	0	0	197,143	128,571	0	1	1
0	0	0	37,1429	38,5714	0	0	0	2
0	0	0	2,85714	102,857	0	0	1	2
0	0	0	4,28571	25,7143	0	0	0	1
0	0	0	192,857	205,714	0	1	1	2
0	0	0	150	128,571	0	1	1	2
0	0	0	40	34,2857	8,57143	0	0	2
0	0	0	17,1429	311,429	25,7143	0	1	2
0	0	0	231,429	94,2857	0	1	1	1
0	0	0	77,1429	51,4286	0	1	0	1
0	0	0	17,1429	102,857	0	0	1	2
0	0	0	60	180	0	1	1	3
0	0	0	62,1429	51,4286	0	1	0	2
0	0	0	87,8571	173,571	0	1	1	2
0	0	0	102,857	102,857	0	1	1	2
0	0	0	30	81,4286	51,4286	0	1	2
0	0	0	158,571	38,5714	0	1	0	1
0	0	0	8,57143	25,7143	0	0	0	2
0	0	0	24,2857	51,4286	0	0	0	2
0	0	0	154,286	145,714	0	1	1	2
0	0	0	15	51,4286	0	0	0	2
0	1	1	62,8571	40	0	1	0	3
0	0	0	81,4286	120	8,57143	1	1	2
0	0	0	25,7143	60	0	0	1	2
0	0	0	175,714	231,429	0	1	1	2
0	0	0	25,7143	188,571	51,4286	0	1	2
0	0	0	150	291,429	0	1	1	1
0	0	0	26,4286	200	8,57143	0	1	2
0	0	0	34,2857	25,7143	0	0	0	1
0	0	0	131,429	67,1429	0	1	1	1
0	0	0	90	17,1429	0	1	0	2
0	0	0	20	77,1429	0	0	1	2
0	0	0	7,14286	25,7143	0	0	0	1
0	0	0	80	32,1429	0	1	0	2
0	0	0	17,1429	214,286	0	0	1	2
0	0	0	24,2857	38,5714	0	0	0	3
0	0	0	17,1429	69,2857	0	0	1	2
0	0	0	82,8571	42,8571	0	1	0	2
0	0	1	67,1429	25,7143	34,2857	1	1	2
0	0	0	51,4286	102,857	0	0	1	2
0	0	0	68,5714	34,2857	0	1	0	2
0	0	0	38,5714	94,2857	0	0	1	1
0	0	0	5	85,7143	0	0	1	2
0	0	0	4,28571	17,1429	2,85714	0	0	2
0	0	0	139,286	96,4286	0	1	1	2
0	0	0	200	205,714	25,7143	1	1	2
0	0	0	308,571	317,143	0	1	1	2
0	0	0	17,1429	0	34,2857	0	0	1

0	0	0	171,429	68,5714	0	1	1	2
0	1	1	248,571	42,8571	0	1	0	2
0	0	0	17,1429	51,4286	0	0	0	2
0	0	0	12,8571	40	0	0	0	2
0	0	0	42,8571	154,286	85,7143	0	1	1
0	1	1	180	72,8571	0	1	1	3
0	0	0	0	248,571	0	0	1	2
0	0	0	8,57143	60	0	0	1	2
0	0	0	2,14286	30	0	0	0	1
0	0	0	21,4286	0	0	0	0	2
0	0	0	0	68,5714	19,2857	0	1	2
0	0	0	55,7143	25,7143	0	0	0	1
0	0	0	34,2857	51,4286	0	0	0	2
0	0	0	81,4286	5,71429	10	1	0	2
1	0	1	143,571	42,8571	68,5714	1	1	2
0	0	0	15,7143	0	0	0	0	3
0	0	0	34,2857	25,7143	0	0	0	1
0	0	0	156,429	222,857	42,8571	1	1	1
0	0	0	141,429	205,714	0	1	1	3
0	0	0	92,8571	128,571	0	1	1	2
0	0	0	25	17,1429	0	0	0	1
0	0	0	45,7143	21,4286	8,57143	0	0	2
0	0	0	22,8571	34,2857	17,1429	0	0	1
0	0	0	4,28571	124,286	0	0	1	2
0	0	0	111,429	248,571	0	1	1	2
0	0	0	19,2857	34,2857	0	0	0	3
0	0	0	17,1429	68,5714	17,1429	0	1	2
0	0	0	14,2857	85,7143	0	0	1	2
0	0	0	20	28,5714	0	0	0	2
0	0	0	0	8,57143	0	0	0	2
0	0	0	34,2857	360	0	0	1	2
0	1	1	60	15	120	1	1	2
0	0	0	158,571	17,1429	17,1429	1	0	2
0	0	0	40,7143	107,143	0	0	1	2
0	0	0	25,7143	28,5714	0	0	0	2
0	0	0	128,571	60	0	1	1	2
0	1	1	120	25,7143	17,1429	1	0	3
0	0	0	17,1429	17,1429	25,7143	0	0	2
0	0	0	139,286	147,143	0	1	1	2
0	0	0	30	17,1429	0	0	0	2
0	0	0	5,71429	17,1429	0	0	0	2
0	0	0	231,429	111,429	77,1429	1	1	2
0	0	0	0	60	0	0	1	2
0	0	0	171,429	25,7143	0	1	0	1
0	0	0	42,8571	68,5714	0	0	1	2
0	0	0	11,4286	51,4286	0	0	0	1
0	0	0	34,2857	21,4286	0	0	0	2
0	0	0	8,57143	42,8571	0	0	0	1

0	0	0	10,7143	0	8,57143	0	0	2
0	0	0	34,2857	0	0	0	0	1
1	1	1	94,2857	171,429	0	1	1	1
0	0	0	42,8571	25,7143	0	0	0	1
0	0	0	54,2857	47,1429	0	0	0	2
0	1	1	120	120	34,2857	1	1	2
0	0	1	25,7143	120	0	0	1	2
0	0	0	8,57143	25,7143	0	0	0	3
0	0	0	60	34,2857	0	1	0	2
0	0	0	102,857	0	0	1	0	2
0	0	0	42,8571	60	25,7143	0	1	2
0	0	0	94,2857	192,857	17,1429	1	1	2
0	0	0	77,1429	34,2857	0	1	0	2
1	1	1	205,714	122,143	42,8571	1	1	1
0	0	0	218,571	162,857	0	1	1	2
0	0	0	54,2857	42,8571	0	0	0	1
0	0	0	57,1429	17,1429	0	0	0	2
0	0	0	10	60	0	0	1	2
0	0	0	52,8571	68,5714	0	0	1	3
0	0	0	38,5714	102,857	68,5714	0	1	2
0	0	0	17,1429	20	0	0	0	2
0	0	0	68,5714	182,857	25,7143	1	1	3
0	0	0	7,14286	14,2857	8,57143	0	0	2
0	0	0	8,57143	30	0	0	0	1
0	0	0	145,714	197,143	0	1	1	2
0	0	0	175,714	47,1429	0	1	0	2
0	0	0	20	8,57143	0	0	0	2
0	0	0	5,71429	257,143	51,4286	0	1	1
0	0	0	8,57143	0	0	0	0	1
1	0	1	51,4286	132,857	85,7143	0	1	1
0	0	0	145,714	282,857	0	1	1	2
0	0	0	150	120	25,7143	1	1	2
0	0	0	47,1429	25,7143	0	0	0	2
0	0	0	60	94,2857	154,286	1	1	2
0	0	0	42,8571	0	17,1429	0	0	2
0	0	0	0	68,5714	0	0	1	2
0	0	0	150	231,429	17,1429	1	1	1
0	0	0	180	120	0	1	1	2
0	0	0	30	120	0	0	1	2
0	0	0	17,1429	51,4286	0	0	0	2
0	1	1	77,1429	11,4286	0	1	0	1
0	0	0	231,429	231,429	0	1	1	2
0	0	0	19,2857	75	17,1429	0	1	1
0	0	0	31,4286	64,2857	0	0	1	2
0	0	0	57,1429	42,8571	0	0	0	1
0	0	0	30	0	0	0	0	2
0	0	0	145,714	154,286	77,1429	1	1	2
0	0	0	57,8571	51,4286	0	0	0	1

0	*0*	*0*	*188,571*	*351,429*	*25,7143*	*1*	*1*	*2*
0	*0*	*0*	*10,7143*	*60*	*102,857*	*0*	*1*	*2*
0	*0*	*0*	*0*	*77,1429*	*0*	*0*	*1*	*2*
0	*0*	*0*	*160*	*81,4286*	*17,1429*	*1*	*1*	*1*
0	*0*	*0*	*25,7143*	*68,5714*	*0*	*0*	*1*	*3*
0	*0*	*0*	*0*	*120*	*0*	*0*	*1*	*2*
0	*1*	*1*	*195,714*	*265,714*	*0*	*1*	*1*	*2*
0	*0*	*0*	*12,8571*	*25,7143*	*0*	*0*	*0*	*2*
0	*0*	*0*	*35,7143*	*85,7143*	*0*	*0*	*1*	*2*
0	*0*	*0*	*35,7143*	*68,5714*	*0*	*0*	*1*	*1*
0	*0*	*0*	*48,5714*	*25,7143*	*0*	*0*	*0*	*1*
0	*0*	*0*	*17,8571*	*208,571*	*0*	*0*	*1*	*2*
0	*0*	*0*	*12,8571*	*154,286*	*0*	*0*	*1*	*2*
0	*0*	*0*	*21,4286*	*72,8571*	*0*	*0*	*1*	*3*
0	*0*	*0*	*42,8571*	*17,1429*	*0*	*0*	*0*	*2*
0	*0*	*0*	*34,2857*	*12,8571*	*0*	*0*	*0*	*2*
0	*1*	*1*	*250*	*120*	*25,7143*	*1*	*1*	*3*
0	*0*	*0*	*88,5714*	*0*	*0*	*1*	*0*	*1*
0	*0*	*0*	*0*	*0*	*94,2857*	*0*	*1*	*2*
0	*0*	*0*	*37,1429*	*51,4286*	*0*	*0*	*0*	*2*
0	*0*	*0*	*17,1429*	*25,7143*	*0*	*0*	*0*	*2*
0	*0*	*0*	*8,57143*	*17,1429*	*0*	*0*	*0*	*2*
0	*0*	*0*	*21,4286*	*40*	*0*	*0*	*0*	*2*
0	*0*	*0*	*28,5714*	*154,286*	*0*	*0*	*1*	*2*
0	*0*	*0*	*41,4286*	*25,7143*	*0*	*0*	*0*	*2*
0	*0*	*0*	*57,8571*	*90*	*6,42857*	*0*	*1*	*2*
0	*0*	*0*	*97,1429*	*51,4286*	*0*	*1*	*0*	*2*
0	*0*	*0*	*85,7143*	*34,2857*	*0*	*1*	*0*	*2*
0	*0*	*0*	*55,7143*	*28,5714*	*0*	*0*	*0*	*2*
0	*0*	*0*	*115,714*	*47,1429*	*0*	*1*	*0*	*2*
0	*0*	*0*	*157,143*	*42,8571*	*0*	*1*	*0*	*1*
0	*0*	*0*	*34,2857*	*105*	*0*	*0*	*1*	*2*
0	*0*	*0*	*0*	*51,4286*	*0*	*0*	*0*	*3*
0	*0*	*0*	*21,4286*	*51,4286*	*0*	*0*	*0*	*2*
0	*1*	*1*	*111,429*	*64,2857*	*0*	*1*	*1*	*2*
0	*0*	*1*	*48,5714*	*85,7143*	*0*	*0*	*1*	*2*
0	*0*	*0*	*0*	*231,429*	*0*	*0*	*1*	*2*
0	*0*	*0*	*41,4286*	*44,2857*	*0*	*0*	*0*	*2*
0	*0*	*0*	*197,143*	*215,714*	*180*	*1*	*1*	*2*
0	*0*	*0*	*188,571*	*137,143*	*17,1429*	*1*	*1*	*1*
0	*0*	*0*	*77,1429*	*120*	*0*	*1*	*1*	*2*
0	*0*	*0*	*188,571*	*42,8571*	*0*	*1*	*0*	*2*
0	*0*	*0*	*128,571*	*368,571*	*120*	*1*	*1*	*2*
0	*0*	*0*	*38,5714*	*0*	*0*	*0*	*0*	*2*
0	*0*	*0*	*17,1429*	*17,1429*	*0*	*0*	*0*	*2*
0	*0*	*0*	*60*	*42,8571*	*0*	*1*	*0*	*1*
0	*0*	*0*	*137,143*	*222,857*	*25,7143*	*1*	*1*	*1*
0	*0*	*0*	*0*	*25,7143*	*0*	*0*	*0*	*2*

0	0	0	111,429	137,143	0	1	1	1
0	0	0	142,857	28,5714	0	1	0	2
0	0	0	30	94,2857	0	0	1	1
0	0	0	85,7143	17,1429	0	1	0	1
0	0	0	22,8571	102,857	0	0	1	2
0	0	0	58,5714	42,8571	0	0	0	2
0	0	0	19,2857	17,1429	0	0	0	1
0	0	0	47,1429	145,714	0	0	1	2
0	0	0	8,57143	25,7143	0	0	0	2
0	0	0	25,7143	51,4286	0	0	0	2
0	0	0	128,571	45,7143	8,57143	1	0	1
0	0	0	85,7143	102,857	0	1	1	3
0	0	0	20	91,4286	0	0	1	2
0	0	0	35,7143	17,1429	0	0	0	1
0	0	0	15,7143	197,143	0	0	1	3
0	0	0	21,4286	34,2857	0	0	0	2
0	0	0	30	51,4286	0	0	0	2
0	0	0	27,1429	8,57143	0	0	0	2
0	0	0	64,2857	17,1429	0	1	0	1
0	1	1	97,1429	60	0	1	1	2
0	0	0	62,8571	85,7143	0	1	1	2
0	1	1	90	120	60	1	1	3
0	0	0	17,1429	51,4286	0	0	0	2
0	0	0	42,8571	60	0	0	1	1
0	0	0	154,286	205,714	0	1	1	2
0	0	0	8,57143	42,8571	8,57143	0	0	1
0	0	0	137,143	42,8571	0	1	0	1
0	0	0	60	17,1429	0	1	0	2
0	0	0	0	40,7143	0	0	0	2
0	0	0	128,571	277,143	85,7143	1	1	1
0	1	1	127,143	51,4286	0	1	0	2
0	0	0	20	124,286	51,4286	0	1	2
0	0	0	158,571	308,571	42,8571	1	1	2
0	0	0	7,14286	42,8571	0	0	0	2
0	0	0	32,8571	25,7143	0	0	0	2
0	0	0	32,8571	47,1429	0	0	0	2
0	0	0	77,1429	34,2857	0	1	0	2
0	0	0	17,1429	34,2857	0	0	0	2
0	0	0	30	8,57143	0	0	0	2
0	0	0	8,57143	188,571	85,7143	0	1	1
0	0	0	45,7143	21,4286	6,42857	0	0	2
0	1	1	197,143	51,4286	0	1	0	1
0	0	0	57,1429	85,7143	0	0	1	1
0	0	0	18,5714	17,1429	0	0	0	1
0	0	0	140	174,286	171,429	1	1	1
0	0	0	128,571	51,4286	0	1	0	2
0	1	1	154,286	34,2857	0	1	0	1
0	0	0	34,2857	94,2857	0	0	1	2

0	0	0	105,714	66,4286	0	1	1	2
0	0	0	21,4286	77,1429	0	0	1	2
0	1	1	98,5714	17,1429	0	1	0	2
0	1	1	240	17,1429	34,2857	1	0	1
0	0	0	27,8571	42,8571	0	0	0	2
0	0	0	7,14286	60	0	0	1	2
0	0	0	0	60	0	0	1	2
0	0	0	124,286	77,1429	0	1	1	3
0	0	0	0	0	0	0	0	2
0	0	0	34,2857	34,2857	0	0	0	1
0	0	0	128,571	308,571	25,7143	1	1	1
0	0	0	160,714	25,7143	25,7143	1	0	1
0	0	0	35,7143	8,57143	0	0	0	1
0	0	0	17,1429	17,1429	0	0	0	1
0	0	0	42,8571	257,143	0	0	1	2
0	0	0	32,1429	40	0	0	0	2
0	0	0	180	98,5714	0	1	1	1
0	0	0	8,57143	34,2857	8,57143	0	0	1
0	0	0	0	30	6,42857	0	0	2
0	0	0	2,85714	17,1429	0	0	0	1
0	0	0	150	242,857	0	1	1	2
0	0	0	214,286	102,857	0	1	1	2
0	0	0	51,4286	0	0	0	0	1
0	0	0	64,2857	128,571	8,57143	1	1	2
0	0	0	90	66,4286	0	1	1	1
0	0	0	8,57143	77,1429	25,7143	0	1	2
0	0	0	12,8571	120	0	0	1	1
0	0	0	0	17,1429	0	0	0	2
0	0	0	30,7143	25,7143	0	0	0	2
0	0	0	171,429	68,5714	0	1	1	2
0	1	1	110	34,2857	0	1	0	2
0	0	0	47,1429	120	0	0	1	2
0	0	0	10	37,1429	0	0	0	2
0	0	0	145,714	85,7143	0	1	1	2
0	0	0	240	222,857	0	1	1	1
0	0	0	17,1429	77,1429	0	0	1	1
0	0	0	157,143	62,8571	2,85714	1	1	3
0	0	0	158,571	57,8571	0	1	0	2
0	0	0	64,2857	42,8571	0	1	0	2
0	0	0	21,4286	77,1429	0	0	1	2
0	0	0	0	11,4286	0	0	0	1
0	0	0	142,857	102,857	0	1	1	1
0	0	0	0	51,4286	0	0	0	1
0	0	0	27,8571	49,2857	0	0	0	2
0	0	0	8,57143	34,2857	0	0	0	2
0	0	0	145,714	71,4286	0	1	1	2
0	0	1	175,714	102,857	0	1	1	2
0	0	0	117,857	28,5714	0	1	0	3

0	0	0	21,4286	8,57143	0	0	0	2
0	0	0	21,4286	42,8571	0	0	0	2
0	0	1	156,429	180	0	1	1	2
0	0	0	30	185,714	0	0	1	3
0	0	0	111,429	34,2857	0	1	0	2
0	0	0	32,1429	17,8571	8,57143	0	0	2
0	0	1	197,143	167,143	17,1429	1	1	2
0	0	0	188,571	60	0	1	1	2
0	0	0	28,5714	128,571	0	0	1	2
0	0	0	35,7143	85,7143	0	0	1	2
0	0	0	17,1429	94,2857	0	0	1	2
0	0	0	300	51,4286	0	1	0	1
0	1	1	120	257,143	60	1	1	2
0	0	0	4,28571	145,714	0	0	1	2
0	0	0	8,57143	42,8571	0	0	0	2
0	0	0	102,857	60	0	1	1	2
0	0	0	185,714	111,429	0	1	1	2
0	1	1	197,143	85,7143	0	1	1	2
0	0	0	85,7143	47,1429	29,1429	1	1	2
0	0	0	124,286	81,4286	12,8571	1	1	1
0	0	0	210	28,5714	8,57143	1	0	3
0	1	1	145,714	51,4286	0	1	0	2
0	0	0	8,57143	64,2857	0	0	1	2
0	1	1	181,429	188,571	60	1	1	2
0	0	0	154,286	180	25,7143	1	1	2
0	0	0	24,2857	68,5714	0	0	1	2
0	0	0	0	240	0	0	1	2
0	0	0	42,8571	60	0	0	1	2
0	0	0	51,4286	22,8571	0	0	0	1
0	1	1	60	25,7143	0	1	0	1
0	0	0	60	128,571	0	1	1	2
0	0	0	55,7143	51,4286	0	0	0	2
0	0	0	31,4286	0	0	0	0	2
0	0	0	102,857	117,857	17,1429	1	1	2
0	1	1	334,286	120	0	1	1	3
0	0	0	37,1429	8,57143	0	0	0	2
0	0	0	222,857	282,857	0	1	1	1
0	0	0	64,2857	94,2857	0	1	1	2
0	0	0	265,714	120	0	1	1	1
0	0	0	52,8571	25,7143	0	0	0	1
0	0	0	34,2857	221,429	0	0	1	3
0	0	0	8,57143	68,5714	0	0	1	2
0	0	0	248,571	105	0	1	1	2
0	0	0	173,571	308,571	0	1	1	2
0	0	0	21,4286	51,4286	0	0	0	1
0	1	1	120,714	25,7143	0	1	0	2
0	0	0	17,1429	0	0	0	0	1
0	0	0	0	308,571	0	0	1	2

0	*0*	*0*	*51,4286*	*102,857*	*0*	*0*	*1*	*2*
0	*0*	*0*	*42,8571*	*154,286*	*0*	*0*	*1*	*2*
0	*0*	*0*	*317,143*	*162,857*	*0*	*1*	*1*	*2*
0	*0*	*0*	*40,7143*	*68,5714*	*0*	*0*	*1*	*2*
0	*0*	*0*	*50*	*154,286*	*0*	*0*	*1*	*2*
0	*0*	*0*	*100*	*128,571*	*25,7143*	*1*	*1*	*1*
0	*0*	*0*	*6,42857*	*192,857*	*0*	*0*	*1*	*2*
0	*0*	*0*	*28,5714*	*40,7143*	*0*	*0*	*0*	*1*
0	*0*	*1*	*57,1429*	*52,1429*	*34,2857*	*0*	*1*	*2*
0	*1*	*1*	*150*	*25,7143*	*0*	*1*	*0*	*2*
0	*0*	*0*	*17,1429*	*60*	*0*	*0*	*1*	*2*
0	*0*	*0*	*128,571*	*164,286*	*17,1429*	*1*	*1*	*2*
0	*0*	*0*	*41,4286*	*77,1429*	*0*	*0*	*1*	*3*
0	*0*	*0*	*14,2857*	*21,4286*	*0*	*0*	*0*	*1*
0	*0*	*0*	*2,85714*	*17,1429*	*0*	*0*	*0*	*2*
0	*0*	*0*	*102,857*	*34,2857*	*0*	*1*	*0*	*2*
0	*0*	*0*	*20,5714*	*180*	*0*	*0*	*1*	*2*
0	*0*	*0*	*60*	*102,857*	*0*	*1*	*1*	*2*
0	*0*	*0*	*0*	*17,1429*	*0*	*0*	*0*	*1*
0	*0*	*0*	*51,4286*	*0*	*0*	*0*	*0*	*2*
0	*0*	*0*	*51,4286*	*94,2857*	*0*	*0*	*1*	*2*
0	*0*	*0*	*42,8571*	*34,2857*	*0*	*0*	*0*	*2*
0	*0*	*0*	*60*	*37,7143*	*0*	*1*	*0*	*1*
0	*0*	*0*	*20*	*27,8571*	*0*	*0*	*0*	*1*
0	*0*	*0*	*64,2857*	*25,7143*	*0*	*1*	*0*	*2*
0	*0*	*0*	*128,571*	*17,1429*	*0*	*1*	*0*	*1*
0	*0*	*0*	*17,1429*	*80*	*0*	*0*	*1*	*2*
0	*0*	*0*	*60*	*17,1429*	*0*	*1*	*0*	*2*
0	*0*	*0*	*42,8571*	*51,4286*	*0*	*0*	*0*	*1*
0	*0*	*0*	*30*	*8,57143*	*0*	*0*	*0*	*1*
0	*0*	*0*	*8,57143*	*98,5714*	*0*	*0*	*1*	*2*
0	*0*	*0*	*42,8571*	*94,2857*	*0*	*0*	*1*	*1*
0	*0*	*0*	*77,1429*	*85,7143*	*0*	*1*	*1*	*3*
0	*0*	*0*	*32,1429*	*34,2857*	*0*	*0*	*0*	*2*
0	*0*	*0*	*17,1429*	*85,7143*	*0*	*0*	*1*	*1*
0	*0*	*0*	*31,4286*	*51,4286*	*0*	*0*	*0*	*2*
0	*0*	*0*	*158,571*	*188,571*	*8,57143*	*1*	*1*	*2*
0	*0*	*0*	*128,571*	*17,1429*	*0*	*1*	*0*	*2*
0	*0*	*0*	*30*	*85,7143*	*0*	*0*	*1*	*3*
0	*0*	*0*	*60*	*248,571*	*0*	*1*	*1*	*2*
0	*1*	*1*	*120*	*217,143*	*0*	*1*	*1*	*3*
0	*0*	*0*	*60*	*25,7143*	*0*	*1*	*0*	*1*
0	*0*	*0*	*32,1429*	*25,7143*	*0*	*0*	*0*	*2*
0	*0*	*0*	*10,7143*	*95,7143*	*0*	*0*	*1*	*2*
0	*0*	*0*	*42,8571*	*8,57143*	*0*	*0*	*0*	*2*
0	*0*	*0*	*21,4286*	*68,5714*	*0*	*0*	*1*	*2*
0	*0*	*0*	*5,71429*	*77,1429*	*0*	*0*	*1*	*2*
0	*0*	*0*	*7,14286*	*32,1429*	*0*	*0*	*0*	*2*

0	0	0	86,4286	180	0	1	1	2
0	0	0	111,429	68,5714	5,71429	1	1	2
0	0	0	42,8571	174,286	0	0	1	2
0	0	0	128,571	162,857	0	1	1	1
0	0	1	30	25,7143	60	0	1	2
0	0	0	4,28571	51,4286	0	0	0	1
0	0	0	77,1429	17,1429	0	1	0	2
0	0	0	25,7143	77,1429	0	0	1	3
0	0	0	4,28571	95,7143	0	0	1	2
0	0	0	8,57143	167,143	0	0	1	2
0	0	0	25,7143	77,1429	0	0	1	2
0	0	0	30	81,4286	5,71429	0	1	2
0	0	0	74,2857	8,57143	0	1	0	1
0	0	0	0	32,1429	0	0	0	1
0	0	0	171,429	8,57143	0	1	0	1
0	0	0	17,1429	205,714	0	0	1	1
0	0	0	120	25,7143	34,2857	1	1	1
0	0	0	222,857	85,7143	0	1	1	2
0	0	0	151,429	90	0	1	1	1
0	0	0	30	37,1429	0	0	0	1
0	0	0	40	60	0	0	1	2
0	0	0	231,429	214,286	25,7143	1	1	2
0	0	0	51,4286	25,7143	0	0	0	1
0	0	0	21,4286	25,7143	0	0	0	1
0	0	0	24,2857	17,1429	0	0	0	1
0	0	0	148,571	252,857	60	1	1	2
0	0	0	55,7143	51,4286	0	0	0	2
0	0	0	54	25,7143	0	0	0	1
0	0	0	11,4286	77,1429	4,28571	0	1	1
0	0	0	40,7143	274,286	77,1429	0	1	1
0	0	0	62,8571	0	0	1	0	2
0	0	0	30	120	102,857	0	1	2
0	0	0	81,4286	30	25,7143	1	0	2
0	0	0	30	197,143	17,1429	0	1	3
0	0	0	34,2857	51,4286	0	0	0	1
0	0	0	67,1429	120	60	1	1	2
0	0	0	160	25,7143	0	1	0	2
0	0	0	29,2857	32,1429	0	0	0	2
0	0	0	161,429	120	0	1	1	2
0	0	0	120	118,571	12,8571	1	1	2
0	0	0	51,4286	8,57143	6,42857	0	0	2
0	0	0	128,571	128,571	0	1	1	2
0	0	0	52,8571	25,7143	0	0	0	2
0	0	0	8,57143	25,7143	0	0	0	1
0	0	0	72,8571	111,429	0	1	1	3
0	0	0	22,8571	17,1429	0	0	0	1
0	0	0	21,4286	25,7143	0	0	0	2
0	0	0	42,8571	51,4286	0	0	0	2

0	*0*	*0*	*17,1429*	*180*	*42,8571*	*0*	*1*	*2*
0	*0*	*0*	*128,571*	*42,8571*	*0*	*1*	*0*	*1*
0	*0*	*0*	*21,4286*	*30*	*0*	*0*	*0*	*2*
0	*0*	*0*	*4,28571*	*25,7143*	*0*	*0*	*0*	*2*
0	*0*	*0*	*25,7143*	*12,8571*	*0*	*0*	*0*	*2*
0	*0*	*0*	*35,7143*	*15,7143*	*0*	*0*	*0*	*2*
0	*0*	*0*	*177,143*	*222,857*	*0*	*1*	*1*	*1*
0	*0*	*0*	*164,286*	*57,1429*	*0*	*1*	*0*	*2*
0	*0*	*0*	*17,1429*	*77,1429*	*0*	*0*	*1*	*1*
0	*0*	*0*	*85,7143*	*8,57143*	*0*	*1*	*0*	*2*
0	*1*	*1*	*70,7143*	*17,1429*	*0*	*1*	*0*	*2*
0	*0*	*0*	*128,571*	*94,2857*	*0*	*1*	*1*	*1*
0	*0*	*0*	*150*	*128,571*	*0*	*1*	*1*	*2*
0	*0*	*0*	*145,714*	*85,7143*	*0*	*1*	*1*	*1*
0	*0*	*0*	*60*	*154,286*	*0*	*1*	*1*	*2*
0	*0*	*0*	*107,143*	*145,714*	*34,2857*	*1*	*1*	*1*
0	*0*	*0*	*245,714*	*188,571*	*42,8571*	*1*	*1*	*2*
0	*0*	*0*	*96,4286*	*49,2857*	*30*	*1*	*1*	*1*
0	*0*	*0*	*0*	*51,4286*	*0*	*0*	*0*	*3*
0	*0*	*0*	*140*	*128,571*	*0*	*1*	*1*	*2*
0	*0*	*0*	*192,857*	*222,857*	*0*	*1*	*1*	*2*
0	*1*	*1*	*85,7143*	*45,7143*	*0*	*1*	*0*	*2*
0	*0*	*0*	*48,5714*	*0*	*0*	*0*	*0*	*2*
0	*0*	*0*	*21,4286*	*44,2857*	*0*	*0*	*0*	*1*
0	*0*	*0*	*60*	*42,8571*	*0*	*1*	*0*	*2*
0	*0*	*0*	*4,28571*	*42,8571*	*8,57143*	*0*	*0*	*2*
0	*0*	*0*	*85,7143*	*28,5714*	*0*	*1*	*0*	*2*
0	*1*	*1*	*142,857*	*154,286*	*0*	*1*	*1*	*2*
0	*0*	*0*	*55,7143*	*19,2857*	*0*	*0*	*0*	*2*
0	*0*	*0*	*167,143*	*222,857*	*8,57143*	*1*	*1*	*2*
0	*0*	*0*	*8,57143*	*8,57143*	*0*	*0*	*0*	*1*
0	*0*	*0*	*85,7143*	*197,143*	*30*	*1*	*1*	*1*
0	*1*	*1*	*120*	*70*	*0*	*1*	*1*	*2*
0	*0*	*0*	*0*	*107,143*	*6,42857*	*0*	*1*	*1*
0	*0*	*0*	*51,4286*	*8,57143*	*0*	*0*	*0*	*2*
0	*0*	*0*	*21,4286*	*81,4286*	*0*	*0*	*1*	*2*
0	*0*	*0*	*77,1429*	*60*	*0*	*1*	*1*	*2*
0	*0*	*0*	*30*	*25,7143*	*0*	*0*	*0*	*2*
0	*0*	*0*	*25,7143*	*77,1429*	*0*	*0*	*1*	*2*
0	*0*	*0*	*38,5714*	*180*	*0*	*0*	*1*	*2*
0	*1*	*1*	*231,429*	*175,714*	*25,7143*	*1*	*1*	*2*
0	*0*	*0*	*47,1429*	*30*	*17,1429*	*0*	*0*	*2*
0	*0*	*0*	*49,2857*	*102,857*	*0*	*0*	*1*	*2*
0	*0*	*0*	*114,286*	*128,571*	*0*	*1*	*1*	*2*
0	*1*	*1*	*300*	*60*	*0*	*1*	*1*	*2*
0	*0*	*0*	*0*	*145,714*	*0*	*0*	*1*	*1*
0	*0*	*0*	*80*	*8,57143*	*8,57143*	*1*	*0*	*2*
0	*0*	*0*	*25,7143*	*30*	*0*	*0*	*0*	*2*

0	0	0	64,2857	34,2857	0	1	0	2
0	0	0	162,857	102,857	0	1	1	1
0	0	0	218,571	274,286	25,7143	1	1	2
0	0	0	0	51,4286	0	0	0	3
0	1	1	115,714	85,7143	0	1	1	2
0	0	0	57,1429	21,4286	38,5714	0	1	2
0	0	0	140	0	0	1	0	2
0	0	0	154,286	128,571	5,71429	1	1	2
0	0	0	30	77,1429	0	0	1	2
0	0	0	0	128,571	51,4286	0	1	2
0	0	0	8,57143	39,2857	28,5714	0	1	2
0	0	0	117,857	120	0	1	1	2
0	0	0	180	128,571	0	1	1	2
0	0	0	0	154,286	0	0	1	1
0	0	0	19,2857	27,1429	0	0	0	2
0	0	0	85,7143	94,2857	0	1	1	2
0	0	0	150	231,429	0	1	1	2
0	1	1	85,7143	51,4286	0	1	0	1
0	0	0	120	154,286	0	1	1	3
0	0	0	35,7143	68,5714	38,5714	0	1	2
0	0	0	148,571	34,2857	0	1	0	2
0	0	0	0	98,5714	0	0	1	2
0	0	0	154,286	282,857	0	1	1	2
0	0	0	180	132,857	0	1	1	1
0	0	0	21,4286	51,4286	0	0	0	2
0	0	0	128,571	394,286	128,571	1	1	2
0	1	1	70	68,5714	0	1	1	3
0	0	0	144,286	97,1429	17,1429	1	1	2
0	0	0	160,714	107,143	17,1429	1	1	2
0	0	0	31,4286	154,286	0	0	1	2
0	0	0	94,2857	17,1429	0	1	0	2
0	0	0	24,2857	34,2857	25,7143	0	1	2
0	0	0	158,571	98,5714	0	1	1	2
0	0	0	17,1429	17,1429	0	0	0	1
0	0	0	45	40	0	0	0	2
0	1	1	180	68,5714	0	1	1	2
0	0	0	9,42857	96	0	0	1	2
0	0	0	162,857	25,7143	0	1	0	2
0	0	0	0	38,5714	0	0	0	1
0	0	0	38,5714	34,2857	0	0	0	2
0	0	0	14,2857	42,8571	0	0	0	2
0	0	0	95,7143	231,429	51,4286	1	1	2
0	0	0	60	98,5714	42,8571	1	1	2
0	0	0	25,7143	25,7143	0	0	0	1
0	0	0	48,5714	51,4286	0	0	0	2
0	0	0	55,7143	120	0	0	1	1
0	0	0	25,7143	51,4286	0	0	0	2
0	0	0	30	34,2857	0	0	0	2

0	0	0	7,14286	42,8571	0	0	0	2
0	0	0	45	248,571	0	0	1	2
0	1	1	90	30	0	1	0	2
0	0	0	17,1429	77,1429	25,7143	0	1	1
0	0	0	214,286	102,857	0	1	1	3
1	0	1	120	197,143	0	1	1	2
0	1	1	175,714	137,143	0	1	1	2
0	0	0	85,7143	156,429	0	1	1	1
0	0	0	137,143	240	8,57143	1	1	1
0	0	0	150	85,7143	34,2857	1	1	3
0	0	0	17,1429	17,1429	0	0	0	2
0	0	0	7,14286	8,57143	0	0	0	2
0	0	0	128,571	248,571	0	1	1	2
0	0	0	17,1429	42,8571	0	0	0	2
0	0	0	180	180	0	1	1	1
0	0	1	108,571	34,2857	0	1	0	1
0	0	0	180	17,1429	0	1	0	2
0	0	0	0	17,1429	0	0	0	2
0	0	0	142,857	171,429	0	1	1	2
0	0	0	188,571	192,857	85,7143	1	1	2
0	0	0	66,4286	77,1429	0	1	1	2
0	1	1	208,571	24,2857	0	1	0	1
0	1	1	189,286	111,429	0	1	1	1
0	0	0	47,1429	21,4286	0	0	0	1
0	1	1	77,1429	85,7143	0	1	1	2
0	0	0	128,571	188,571	0	1	1	2
0	0	0	47,1429	48,5714	0	0	0	2
0	0	0	34,2857	102,857	0	0	1	2
0	0	0	30,7143	21,4286	0	0	0	2
0	0	0	25,7143	25,7143	34,2857	0	1	2
0	0	0	60	25,7143	0	1	0	2
0	0	0	88,5714	17,1429	0	1	0	3
0	0	0	10	94,2857	0	0	1	2
0	0	0	128,571	154,286	25,7143	1	1	3
0	0	0	8,57143	85,7143	8,57143	0	1	1
0	0	0	128,571	277,143	85,7143	1	1	2
0	0	0	2,85714	102,857	0	0	1	2
0	0	0	4,28571	39,2857	6,42857	0	0	1
0	0	0	19,2857	17,1429	0	0	0	1
0	0	0	17,8571	34,2857	0	0	0	2
0	0	0	45	42,8571	42,8571	0	1	1
0	0	0	21,4286	25,7143	0	0	0	2
0	0	0	64,2857	42,8571	0	1	0	2
0	0	0	137,143	60	25,7143	1	1	2
0	0	0	137,857	17,1429	0	1	0	2
0	0	0	115,714	192,857	0	1	1	1
0	0	0	12,8571	31,4286	0	0	0	2
0	0	0	37,1429	60	0	0	1	3

0	0	0	20,7143	25,7143	0	0	0	2
0	0	0	77,1429	85,7143	0	1	1	2
0	0	0	171,429	34,2857	0	1	0	2
0	0	0	47,1429	5,71429	0	0	0	2
0	0	0	0	240	0	0	1	3
0	0	0	308,571	265,714	0	1	1	2
0	0	0	12,8571	25,7143	0	0	0	2
0	0	0	60	51,4286	0	1	0	1
0	0	0	21,4286	113,571	0	0	1	2
0	0	0	21,4286	8,57143	0	0	0	2
0	0	0	60	90	0	1	1	3
0	0	0	21,4286	77,1429	0	0	1	2
0	0	0	37,1429	252,857	8,57143	0	1	2
0	0	0	124,286	128,571	0	1	1	2
0	0	0	32,8571	15	0	0	0	2
0	0	0	0	17,1429	0	0	0	2
0	0	0	158,571	231,429	0	1	1	3
0	0	0	57,1429	205,714	0	0	1	2
0	0	0	128,571	68,5714	0	1	1	2
0	0	0	85,7143	194,286	0	1	1	1
0	0	0	128,571	154,286	0	1	1	2
0	0	0	8,57143	60	0	0	1	3
0	0	0	21,4286	154,286	0	0	1	2
0	1	1	360	107,143	0	1	1	2
0	0	0	201,429	102,857	0	1	1	1
0	0	0	128,571	409,286	0	1	1	2
0	1	1	60	130	180	1	1	1
0	0	0	19,2857	120	0	0	1	2
0	0	0	34,2857	214,286	0	0	1	1
0	0	0	39,2857	51,4286	0	0	0	2
0	0	0	60	171,429	0	1	1	2
0	0	0	71,4286	248,571	0	1	1	2
0	0	0	128,571	64,2857	0	1	1	1
0	0	0	0	60	0	0	1	1
0	0	0	21,4286	25,7143	0	0	0	1
0	0	0	51,4286	102,857	0	0	1	2
0	0	0	25,7143	25,7143	0	0	0	1
0	0	0	120	60	8,57143	1	1	2
0	0	0	137,143	205,714	17,1429	1	1	2
0	0	0	55,7143	111,429	0	0	1	2
0	0	0	72,8571	68,5714	0	1	1	2
0	0	0	35,7143	6,42857	0	0	0	2
0	0	0	21,4286	68,5714	0	0	1	1
0	0	0	42,8571	51,4286	0	0	0	2
0	0	0	96,4286	17,1429	0	1	0	2
0	0	0	81,4286	34,2857	0	1	0	1
0	1	1	270	64,2857	25,7143	1	1	1
0	0	0	47,1429	74,2857	0	0	1	1

0	0	0	154,286	145,714	25,7143	1	1	2
0	0	0	205,714	171,429	25,7143	1	1	1
0	0	0	38,5714	47,1429	0	0	0	2
0	0	0	154,286	21,4286	0	1	0	2
0	0	0	114,286	120	5,71429	1	1	1
0	0	0	94,2857	34,2857	0	1	0	2
0	0	0	0	90	0	0	1	1
0	1	1	190	77,1429	0	1	1	1
0	0	0	107,143	92,1429	0	1	1	2
0	0	0	197,143	25,7143	0	1	0	3
0	0	0	34,2857	154,286	0	0	1	2
0	0	0	47,1429	261,429	0	0	1	2
0	0	0	142,857	137,143	0	1	1	1
0	0	0	0	94,2857	0	0	1	2
0	0	0	80	84,2857	0	1	1	1
0	0	0	42,8571	81,4286	0	0	1	1
0	0	0	0	72,8571	0	0	1	2
0	0	0	49,2857	21,4286	0	0	0	3
0	0	0	1,42857	0	0	0	0	1
0	0	0	62,8571	55,7143	0	1	0	1
0	0	0	8,57143	44,2857	2,14286	0	0	2
0	0	0	338,571	167,143	30	1	1	2
0	0	0	8,57143	137,143	102,857	0	1	3
0	0	0	14,2857	34,2857	17,1429	0	0	2
0	0	0	102,857	98,5714	0	1	1	2
0	0	0	0	102,857	0	0	1	2
0	0	0	128,571	268,571	180	1	1	2
0	0	0	62,8571	158,571	5,71429	1	1	2
0	0	0	0	34,2857	0	0	0	3
0	0	0	25,7143	34,2857	0	0	0	3
0	0	0	5,71429	128,571	0	0	1	2
0	0	0	57,1429	17,1429	0	0	0	2
0	0	0	140	42,8571	0	1	0	2
0	0	0	55,7143	70,7143	0	0	1	1
0	0	0	12,8571	17,1429	0	0	0	2
0	0	0	64,2857	17,1429	0	1	0	1
0	0	0	0	128,571	0	0	1	2
0	0	0	81,4286	257,143	8,57143	1	1	2
0	0	0	25,7143	214,286	6,42857	0	1	2
0	0	0	10	180	0	0	1	2
0	0	0	77,1429	0	0	1	0	2
0	0	0	60	51,4286	0	1	0	1
0	0	0	248,571	42,8571	0	1	0	2
0	0	0	191,429	334,286	0	1	1	1
0	0	0	0	25,7143	0	0	0	2
0	0	0	11,4286	94,2857	0	0	1	2
0	1	1	291,429	188,571	0	1	1	1
0	0	0	157,143	197,143	25,7143	1	1	1

0	0	0	21,4286	80,7143	0	0	1	1
0	0	0	52,8571	25,7143	17,1429	0	0	1
0	0	0	162,857	180	0	1	1	2
0	0	0	20	248,571	0	0	1	2
0	0	0	42,8571	55,7143	8,57143	0	1	3
0	0	0	137,143	108,571	34,2857	1	1	2
0	0	0	141,429	111,429	0	1	1	2
0	0	0	0	34,2857	0	0	0	1
0	0	0	42,8571	15	0	0	0	2
0	0	0	0	5,71429	0	0	0	2
0	0	0	184,286	68,5714	0	1	1	1
0	0	1	42,8571	171,429	0	0	1	2
0	0	0	29,2857	51,4286	0	0	0	2
0	1	1	120	51,4286	0	1	0	2
0	0	0	157,857	205,714	0	1	1	2
0	0	0	21,4286	77,1429	0	0	1	2
0	0	0	7,14286	28,5714	0	0	0	2
0	0	0	27,8571	17,1429	0	0	0	2
0	0	0	51,4286	115,714	0	0	1	2
0	0	0	47,1429	34,2857	0	0	0	2
0	0	0	12,8571	128,571	25,7143	0	1	2
0	0	0	27,8571	120	0	0	1	1
0	0	0	39,2857	51,4286	6,42857	0	0	2
0	0	0	0	0	0	0	0	2
0	0	0	2,14286	25,7143	4,28571	0	0	2
0	0	0	0	90	0	0	1	2
1	0	1	145,714	222,857	128,571	1	1	2
0	0	0	121,429	317,143	0	1	1	2
0	0	0	5,71429	171,429	0	0	1	2
0	0	0	5,71429	34,2857	0	0	0	2
0	0	0	102,857	180	0	1	1	2
0	0	0	42,8571	85,7143	128,571	0	1	2
0	0	0	154,286	257,143	0	1	1	2
0	0	0	0	42,8571	34,2857	0	1	2
0	0	0	8,57143	47,1429	0	0	0	2
0	0	0	65	120	0	1	1	1
0	0	0	41,4286	28,5714	71,4286	0	1	1
0	1	1	330	34,2857	1,42857	1	0	1
0	0	0	12,8571	8,57143	0	0	0	2
0	0	0	188,571	38,5714	0	1	0	2
0	0	1	205,714	145,714	0	1	1	2
0	0	0	8,57143	188,571	0	0	1	2
0	0	0	102,857	214,286	0	1	1	2
0	0	0	42,8571	36,4286	8,57143	0	0	1
0	0	0	21,4286	49,2857	0	0	0	2
0	0	0	138,571	311,429	25,7143	1	1	1
0	0	0	4,28571	150	8,57143	0	1	2
0	0	0	137,143	34,2857	0	1	0	1

0	0	0	184,286	25,7143	0	1	0	2
0	0	0	17,1429	120	0	0	1	2
0	1	1	188,571	274,286	77,1429	1	1	2
0	0	0	21,4286	25,7143	0	0	0	2
0	0	0	192,857	175,714	0	1	1	2
0	0	0	150	68,5714	0	1	1	2
0	0	0	11,4286	162,857	0	0	1	2
0	0	0	17,8571	17,1429	21,4286	0	0	2
0	0	0	8,57143	120	0	0	1	2
0	0	0	9,28571	77,1429	0	0	1	2
0	0	0	128,571	34,2857	0	1	0	1
0	0	0	128,571	60	0	1	1	2
0	1	1	175,714	77,1429	0	1	1	2
0	0	0	128,571	282,857	102,857	1	1	2
0	0	0	32,8571	94,2857	0	0	1	2
0	0	0	7,14286	34,2857	0	0	0	2
0	0	0	140	120	0	1	1	2
0	0	0	115,714	102,857	0	1	1	2
0	0	0	195	85,7143	0	1	1	2
0	0	0	188,571	351,429	0	1	1	1
0	0	0	137,143	47,1429	0	1	0	2
0	0	0	38,5714	0	0	0	0	2
0	0	0	13,7143	85,7143	25,7143	0	1	1
0	0	0	14,2857	6,42857	0	0	0	2
0	0	0	148,571	188,571	0	1	1	2
0	0	0	51,4286	130	12,8571	0	1	1
0	0	0	91,4286	25,7143	0	1	0	2
0	1	1	85	34,2857	0	1	0	1
0	0	0	10,7143	80	0	0	1	1
0	0	0	17,1429	42,8571	0	0	0	1
0	0	0	128,571	162,857	17,1429	1	1	2
0	0	0	91,4286	11,4286	17,1429	1	0	2
0	0	0	22,8571	111,429	0	0	1	2
0	1	1	120	0	0	1	0	1
0	1	1	308,571	248,571	0	1	1	2
0	0	0	175,714	188,571	17,1429	1	1	3
0	0	0	42,8571	145,714	0	0	1	1
0	0	0	15	51,4286	12,8571	0	1	2
0	0	0	44,2857	4,28571	0	0	0	2
0	0	0	14,2857	2,85714	0	0	0	2
0	1	1	137,143	0	0	1	0	2
0	0	0	94,2857	102,857	68,5714	1	1	2
0	0	0	51,4286	61,4286	0	0	1	2
0	0	0	0	62,1429	0	0	1	1
0	0	0	141,571	340,714	17,1429	1	1	1
0	0	0	171,429	157,143	0	1	1	2
0	0	0	57,1429	51,4286	51,4286	0	1	1
0	1	1	368,571	21,4286	7,14286	1	0	2

0	0	0	40	34,2857	0	0	0	2
0	0	0	15	246,429	0	0	1	3
0	0	0	64,2857	15	0	1	0	2
0	0	0	21,4286	188,571	0	0	1	2
0	0	0	47,1429	68,5714	17,1429	0	1	2
0	0	0	68,5714	158,571	0	1	1	2
0	0	0	137,143	42,8571	0	1	0	1
0	0	0	111,429	74,2857	17,1429	1	1	1
0	0	0	14,2857	57,1429	0	0	0	1
0	0	0	154,286	94,2857	0	1	1	1
0	0	0	128,571	192,857	120	1	1	2
0	0	0	2,85714	124,286	0	0	1	2
0	0	0	0	137,143	0	0	1	1
0	0	0	65,7143	84,2857	0	1	1	2
0	0	0	173,571	77,1429	0	1	1	2
0	0	0	210	77,1429	0	1	1	1
1	0	1	128,571	377,143	0	1	1	2
0	0	0	32,1429	51,4286	0	0	0	2
0	0	0	38,5714	145,714	0	0	1	2
0	0	0	60	60	0	1	1	2
0	1	1	80	25,7143	0	1	0	2
0	0	0	60	77,1429	51,4286	1	1	3
0	0	0	8,57143	4,28571	0	0	0	3
0	0	0	8,57143	25,7143	0	0	0	1
0	0	0	12,8571	42,8571	0	0	0	2
0	0	0	44,2857	25,7143	11,4286	0	0	2
0	0	0	137,143	180	0	1	1	1
0	0	0	77,1429	25,7143	0	1	0	2
0	0	0	14,2857	62,8571	0	0	1	3
0	1	1	317,143	231,429	0	1	1	1
0	0	0	7,14286	41,4286	0	0	0	3
0	1	1	150	257,143	0	1	1	1
0	0	0	274,286	180	0	1	1	2
0	0	0	31,4286	25,7143	0	0	0	2
0	0	0	42,2857	94,2857	0	0	1	3
0	0	0	0	42,8571	0	0	0	2
0	0	0	108,571	214,286	0	1	1	2
0	0	1	188,571	191,429	0	1	1	2
0	0	0	25,7143	12,8571	0	0	0	2
0	0	0	128,571	231,429	0	1	1	2
0	0	0	7,14286	34,2857	0	0	0	2
0	0	0	44,2857	107,143	0	0	1	2
0	0	0	25,7143	77,1429	0	0	1	2
0	0	0	0	188,571	0	0	1	2
0	1	1	257,143	162,857	0	1	1	1
0	0	0	124,286	0	0	1	0	2
0	0	0	184,286	8,57143	0	1	0	2
0	0	0	17,1429	40	25,7143	0	1	2

0	0	0	2,85714	10	0	0	0	2
0	0	0	128,571	66,4286	0	1	1	2
0	0	0	128,571	154,286	0	1	1	2
0	0	0	240	34,2857	0	1	0	1
0	0	0	130	34,2857	0	1	0	3
0	0	0	0	98,5714	0	0	1	2
0	0	0	34,2857	25,7143	0	0	0	2
0	0	0	17,1429	143,571	0	0	1	2
0	0	0	131,429	128,571	0	1	1	2
0	0	0	42,8571	17,1429	25,7143	0	0	2
0	0	0	158,571	51,4286	0	1	0	2
0	0	0	64,2857	128,571	0	1	1	2
0	0	0	180	274,286	68,5714	1	1	1
0	0	0	81,4286	51,4286	0	1	0	2
0	0	1	95,7143	188,571	25,7143	1	1	2
0	0	0	58,5714	4,28571	17,1429	0	0	2
0	0	0	102,857	34,2857	0	1	0	1
0	0	0	197,143	325,714	25,7143	1	1	2
0	0	0	60	50	17,1429	1	1	2
0	0	0	5,71429	25,7143	0	0	0	2
0	0	0	195	200	0	1	1	2
0	0	0	55,7143	115,714	51,4286	0	1	1
0	0	0	65,7143	137,143	0	1	1	3
0	0	0	105	120	0	1	1	2
0	0	0	28,5714	17,1429	0	0	0	2
0	0	0	231,429	137,143	111,429	1	1	2
0	0	0	21,4286	94,2857	34,2857	0	1	2
0	0	0	42,8571	68,5714	0	0	1	2
0	0	0	32,8571	120	51,4286	0	1	2
0	0	0	60	51,4286	0	1	0	1
0	0	0	85,7143	205,714	0	1	1	1
0	0	0	14,2857	154,286	51,4286	0	1	2
0	0	0	171,429	154,286	0	1	1	1
0	0	0	201,429	100,714	0	1	1	1
0	0	0	72,8571	77,1429	0	1	1	3
0	0	0	0	8,57143	0	0	0	2
0	0	0	42,8571	17,1429	0	0	0	2
0	0	0	8,57143	114,286	0	0	1	1
0	0	0	32,8571	120	0	0	1	2
0	0	0	28,5714	105,714	0	0	1	2
0	0	0	57,1429	42,8571	0	0	0	2
0	0	0	22,8571	102,857	0	0	1	1
0	0	0	60	21,4286	0	1	0	2
0	0	0	17,1429	17,1429	0	0	0	2
0	0	0	16,4286	42,8571	0	0	0	2
0	0	0	171,429	61,4286	0	1	1	2
0	0	0	0	60	0	0	1	3
0	1	1	257,143	77,1429	0	1	1	2

0	0	0	180	300	0	1	1	2
0	0	0	42,8571	34,2857	6,85714	0	0	1
0	0	0	21,4286	85,7143	0	0	1	1
0	0	0	5,71429	68,5714	0	0	1	2
0	0	0	145,714	222,857	77,1429	1	1	2
0	0	0	25,7143	25,7143	0	0	0	3
0	0	0	71,4286	8,57143	0	1	0	2
0	0	0	4,28571	0	0	0	0	2
1	0	1	84,2857	25,7143	102,857	1	1	2
0	0	0	0	102,857	0	0	1	3
0	0	0	180	231,429	128,571	1	1	2
0	0	0	0	17,1429	0	0	0	2
0	0	0	13,2857	116,143	0	0	1	2
0	0	0	72,8571	22,1429	0	1	0	1
0	0	0	28,5714	12,8571	0	0	0	1
0	0	0	334,286	130	0	1	1	1
0	0	0	0	83,5714	0	0	1	2
0	0	0	188,571	248,571	25,7143	1	1	1
0	0	0	102,857	180	180	1	1	2
0	0	0	55,7143	124,286	6,42857	0	1	2
0	1	1	60	25,7143	0	1	0	1
0	0	0	8,57143	51,4286	0	0	0	2
0	0	0	60	145,714	0	1	1	2
0	0	0	24,2857	12,8571	0	0	0	2
0	0	0	47,1429	120	0	0	1	3
0	0	0	37,1429	38,5714	0	0	0	2
0	0	0	72,8571	48,5714	0	1	0	2
0	0	0	0	111,429	0	0	1	1
0	0	0	77,1429	25,7143	0	1	0	2
0	0	0	145,714	171,429	0	1	1	2
0	0	0	12,8571	0	6,42857	0	0	2
1	0	1	0	85,7143	34,2857	0	1	1
0	0	0	85,7143	231,429	17,1429	1	1	2
0	0	0	42,8571	188,571	0	0	1	2
0	0	0	132,857	23,5714	0	1	0	1
0	0	1	77,1429	42,8571	0	1	0	2
0	0	0	12,8571	25,7143	0	0	0	2
0	0	0	35,7143	51,4286	0	0	0	2
0	0	0	81,4286	53,5714	0	1	0	2
0	0	0	77,1429	72,8571	17,1429	1	1	2
0	0	0	115,714	180	0	1	1	2
0	0	0	60	51,4286	0	1	0	1
0	0	0	22,8571	25,7143	0	0	0	2
0	0	0	97,1429	120	0	1	1	2
0	0	0	94,2857	64,2857	0	1	1	1
0	1	1	60	150	34,2857	1	1	1
0	0	0	150	32,1429	0	1	0	2
0	0	0	21,4286	25,7143	0	0	0	3

0	0	0	0	70,7143	102,857	0	1	2
0	0	0	214,286	154,286	0	1	1	2
0	0	0	60	137,143	8,57143	1	1	2
0	0	0	14,2857	25,7143	0	0	0	2
0	0	0	87,1429	35,7143	0	1	0	2
0	0	0	8,57143	17,1429	0	0	0	2
0	0	0	180	42,8571	8,57143	1	0	2
0	0	0	68,5714	68,5714	0	1	1	2
0	0	0	174,286	90	0	1	1	1
0	1	1	162,857	30	0	1	0	2
0	0	0	34,2857	17,1429	0	0	0	1
0	1	1	368,571	188,571	0	1	1	1
0	0	0	8,57143	19,2857	0	0	0	2
0	0	0	51,4286	17,1429	0	0	0	2
0	0	0	5,71429	145,714	8,57143	0	1	2
0	0	0	102,857	0	38,5714	1	0	2
0	0	0	282,857	180	0	1	1	2
0	1	1	120	68,5714	0	1	1	3
0	0	0	11,4286	60	0	0	1	2
0	1	1	60	0	25,7143	1	0	2
0	0	0	111,429	60	0	1	1	2
0	0	0	88,5714	102,857	0	1	1	2
0	0	0	0	180	0	0	1	2
0	0	0	192,857	175,714	0	1	1	2
0	0	0	98,5714	188,571	0	1	1	1
0	0	0	102,857	0	0	1	0	2
0	0	0	47,1429	27,8571	0	0	0	2
0	0	0	17,1429	0	0	0	0	2
0	0	0	14,2857	68,5714	0	0	1	3
0	0	0	31,4286	25,7143	0	0	0	2
0	0	0	32,8571	72,8571	0	0	1	2
0	1	1	124,286	51,4286	0	1	0	2
0	0	0	0	68,5714	0	0	1	1
0	0	0	60	25,7143	0	1	0	2
0	0	0	261,429	60	0	1	1	2
0	0	0	85,7143	128,571	0	1	1	2
0	0	1	32,1429	152,857	197,143	0	1	2
0	0	0	2,85714	157,143	0	0	1	2
0	0	0	42,8571	34,2857	0	0	0	2
0	0	0	180	6,42857	0	1	0	1
0	0	0	60	68,5714	2,85714	1	1	2
0	0	0	18,5714	54,2857	0	0	0	2
0	0	0	37,1429	81,4286	0	0	1	2
0	0	0	28,5714	102,857	0	0	1	2
0	0	0	94,2857	77,1429	17,1429	1	1	1
0	1	1	130	42,8571	0	1	0	2
0	0	0	10	51,4286	0	0	0	2
0	0	0	51,4286	34,2857	0	0	0	1

0	0	1	85,7143	68,5714	102,857	1	1	2
0	0	0	51,4286	162,857	0	0	1	1
0	0	0	27,1429	51,4286	0	0	0	2
0	0	0	120	60	0	1	1	2
0	0	0	0	51,4286	0	0	0	2
0	0	0	75,7143	34,2857	8,57143	1	0	2
0	0	0	25,7143	68,5714	0	0	1	2
0	1	1	68,5714	17,1429	0	1	0	2
0	0	0	120	200	85,7143	1	1	2
0	1	1	180	141,429	77,1429	1	1	2
0	0	0	154,286	75	17,1429	1	1	2
0	0	0	17,1429	25,7143	25,7143	0	0	2
0	0	0	22,8571	8,57143	0	0	0	2
0	0	0	158,571	115,714	0	1	1	2
0	0	0	52,8571	120	0	0	1	3
0	0	0	8,57143	42,8571	0	0	0	1
0	0	0	34,2857	17,1429	25,7143	0	0	2
0	0	0	68,5714	71,4286	19,2857	1	1	2
0	0	0	31,4286	60	5,71429	0	1	2
0	0	0	35,7143	42,8571	17,1429	0	1	2
0	0	0	0	0	0	0	0	2
0	0	0	54,2857	34,2857	0	0	0	1
0	0	0	162,857	180	0	1	1	2
0	0	0	167,143	72,8571	0	1	1	2
0	0	0	25,7143	85,7143	17,1429	0	1	2
0	0	0	8,57143	81,4286	17,1429	0	1	2
0	0	0	21,4286	257,143	0	0	1	2
0	0	0	12,8571	68,5714	0	0	1	2
0	0	0	34,2857	17,1429	0	0	0	2
0	0	0	44,2857	0	0	0	0	1
0	0	0	0	60	0	0	1	1
0	0	0	38,5714	17,1429	4,28571	0	0	2
0	0	0	60	120	0	1	1	2
0	0	0	70,7143	17,1429	0	1	0	2
0	0	0	0	51,4286	0	0	0	1
0	0	0	38,5714	47,1429	25,7143	0	1	1
0	0	0	81,4286	68,5714	0	1	1	1
0	0	0	145,714	68,5714	25,7143	1	1	1
0	0	0	0	8,57143	0	0	0	2
0	0	0	15	64,2857	51,4286	0	1	2
0	0	0	12,8571	102,857	77,1429	0	1	2
0	0	0	141,429	94,2857	0	1	1	2
0	0	0	38,5714	0	0	0	0	2
0	0	0	4,28571	84,2857	0	0	1	2
0	1	1	60	188,571	0	1	1	2
0	0	0	15	90	8,57143	0	1	2
0	0	0	72,8571	34,2857	0	1	0	1
0	1	1	128,571	81,4286	0	1	1	2

0	1	1	81,4286	51,4286	0	1	0	1
0	0	0	154,286	199,286	0	1	1	2
0	0	0	28,5714	60	0	0	1	2
0	0	0	35,7143	154,286	0	0	1	1
1	0	1	201,429	274,286	0	1	1	2
0	0	0	68,5714	151,429	0	1	1	2
0	1	1	205,714	0	0	1	0	1
0	0	0	42,8571	60	0	0	1	2
0	0	0	17,1429	154,286	0	0	1	2
0	0	0	38,5714	17,1429	0	0	0	2
0	0	0	128,571	68,5714	34,2857	1	1	2
0	0	0	4,28571	51,4286	0	0	0	1
0	0	0	34,2857	34,2857	0	0	0	2
0	1	1	60	25,7143	0	1	0	1
0	0	0	0	150	25,7143	0	1	3
0	0	0	137,143	30	6,42857	1	0	1
0	0	0	194,286	171,429	47,1429	1	1	2
0	0	0	131,429	180	0	1	1	2
0	0	0	60	79,2857	17,1429	1	1	1
0	0	0	27,8571	8,57143	34,2857	0	0	2
0	0	0	14,2857	257,143	0	0	1	2
0	0	0	120	257,143	205,714	1	1	2
0	0	0	34,2857	38,5714	0	0	0	2
0	0	0	0	70	0	0	1	1
0	0	0	154,286	154,286	0	1	1	2
0	0	0	120	257,143	154,286	1	1	1
0	0	0	17,1429	42,8571	34,2857	0	1	2
0	0	0	0	68,5714	0	0	1	2
0	0	0	81,4286	51,4286	0	1	0	1
0	0	0	142,857	194,286	77,1429	1	1	2
0	0	0	10	34,2857	0	0	0	1
0	0	0	45,7143	25,7143	0	0	0	2
0	0	0	138,571	128,571	1,42857	1	1	2
0	0	0	85,7143	42,8571	0	1	0	2
0	0	0	10,7143	60	0	0	1	3
0	0	0	40	25,7143	0	0	0	1
0	0	0	11,4286	91,4286	0	0	1	1
0	0	0	5,71429	20	0	0	0	3
0	0	1	68,5714	42,8571	0	1	0	2
0	0	0	66,4286	60	0	1	1	2
0	1	1	338,571	177,143	0	1	1	1
0	0	0	145,714	102,857	8,57143	1	1	2
0	0	0	97,1429	12,8571	0	1	0	1
0	0	0	197,143	205,714	0	1	1	2
0	0	0	0	171,429	180	0	1	3
0	0	0	124,286	77,1429	0	1	1	1
0	0	0	32,1429	17,1429	51,4286	0	1	2
0	1	1	162,857	134,286	0	1	1	2

0	*0*	*0*	*42,8571*	*42,8571*	*0*	*0*	*0*	*2*
0	*0*	*0*	*68,5714*	*102,857*	*0*	*1*	*1*	*2*
0	*0*	*0*	*47,1429*	*70,7143*	*51,4286*	*0*	*1*	*2*
0	*0*	*0*	*231,429*	*77,1429*	*0*	*1*	*1*	*1*
0	*0*	*0*	*21,4286*	*34,2857*	*0*	*0*	*0*	*1*
0	*0*	*0*	*11,4286*	*42,8571*	*0*	*0*	*0*	*1*
0	*0*	*0*	*80*	*14,2857*	*0*	*1*	*0*	*2*
0	*0*	*0*	*118,571*	*175,714*	*0*	*1*	*1*	*1*
0	*0*	*0*	*68,5714*	*214,286*	*0*	*1*	*1*	*2*
0	*0*	*0*	*4,28571*	*17,1429*	*0*	*0*	*0*	*1*
0	*0*	*0*	*248,571*	*85,7143*	*51,4286*	*1*	*1*	*2*
0	*0*	*0*	*42,8571*	*55*	*0*	*0*	*0*	*2*
1	*0*	*1*	*64,2857*	*111,429*	*42,8571*	*1*	*1*	*2*
0	*0*	*0*	*32,8571*	*317,143*	*154,286*	*0*	*1*	*2*
0	*0*	*0*	*64,2857*	*51,4286*	*0*	*1*	*0*	*2*
0	*0*	*0*	*55,7143*	*42,8571*	*0*	*0*	*0*	*3*
0	*0*	*0*	*102,857*	*268,571*	*0*	*1*	*1*	*2*
0	*0*	*0*	*55,7143*	*102,857*	*0*	*0*	*1*	*2*
0	*1*	*1*	*188,571*	*94,2857*	*0*	*1*	*1*	*2*
0	*0*	*0*	*205,714*	*70,7143*	*25,7143*	*1*	*1*	*2*
0	*0*	*0*	*162,857*	*205,714*	*0*	*1*	*1*	*3*
0	*0*	*0*	*300*	*180*	*25,7143*	*1*	*1*	*2*
0	*0*	*0*	*0*	*32,1429*	*0*	*0*	*0*	*2*
0	*0*	*0*	*20*	*25,7143*	*0*	*0*	*0*	*1*
0	*0*	*0*	*0*	*60*	*0*	*0*	*1*	*2*
0	*0*	*0*	*132,857*	*81,4286*	*0*	*1*	*1*	*2*
0	*0*	*0*	*67,1429*	*94,2857*	*25,7143*	*1*	*1*	*1*
0	*0*	*0*	*21,4286*	*25,7143*	*0*	*0*	*0*	*1*
0	*0*	*0*	*154,286*	*120*	*51,4286*	*1*	*1*	*2*
0	*0*	*0*	*62,8571*	*60*	*0*	*1*	*1*	*2*
0	*0*	*0*	*217,143*	*165*	*60*	*1*	*1*	*2*
0	*0*	*0*	*188,571*	*200*	*0*	*1*	*1*	*2*
0	*0*	*0*	*34,2857*	*34,2857*	*0*	*0*	*0*	*2*
0	*0*	*0*	*274,286*	*60*	*0*	*1*	*1*	*2*
0	*0*	*0*	*17,1429*	*21,4286*	*0*	*0*	*0*	*2*
0	*0*	*0*	*55,7143*	*51,4286*	*0*	*0*	*0*	*1*
0	*0*	*0*	*74,2857*	*10,7143*	*0*	*1*	*0*	*2*
0	*0*	*0*	*0*	*90*	*0*	*0*	*1*	*2*
0	*0*	*0*	*0*	*51,4286*	*17,1429*	*0*	*1*	*2*
0	*0*	*1*	*197,143*	*231,429*	*0*	*1*	*1*	*2*
0	*0*	*0*	*150*	*50*	*0*	*1*	*0*	*2*
0	*0*	*0*	*0*	*110*	*0*	*0*	*1*	*2*
0	*0*	*0*	*137,143*	*171,429*	*0*	*1*	*1*	*2*
0	*1*	*1*	*68,5714*	*167,143*	*34,2857*	*1*	*1*	*2*
0	*0*	*0*	*142,857*	*81,4286*	*0*	*1*	*1*	*2*
0	*0*	*0*	*14,2857*	*102,857*	*0*	*0*	*1*	*1*
0	*1*	*1*	*60*	*126,429*	*77,1429*	*1*	*1*	*2*
0	*0*	*0*	*137,143*	*85,7143*	*0*	*1*	*1*	*3*

0	*0*	*0*	*17,1429*	*25,7143*	*0*	*0*	*0*	*2*
0	*0*	*0*	*51,4286*	*205,714*	*0*	*0*	*1*	*2*
0	*0*	*0*	*23,5714*	*38,5714*	*0*	*0*	*0*	*2*
0	*0*	*0*	*60*	*154,286*	*0*	*1*	*1*	*2*
0	*0*	*0*	*20*	*102,857*	*0*	*0*	*1*	*2*
0	*0*	*0*	*51,4286*	*85,7143*	*25,7143*	*0*	*1*	*2*
0	*0*	*0*	*45,7143*	*8,57143*	*0*	*0*	*0*	*3*
0	*0*	*0*	*28,5714*	*158,571*	*0*	*0*	*1*	*2*
0	*0*	*1*	*60*	*27,8571*	*17,1429*	*1*	*0*	*2*
0	*0*	*0*	*154,286*	*25,7143*	*0*	*1*	*0*	*2*
0	*0*	*0*	*21,4286*	*35,7143*	*0*	*0*	*0*	*2*
0	*0*	*0*	*28,5714*	*77,1429*	*0*	*0*	*1*	*2*
0	*0*	*0*	*7,14286*	*42,8571*	*0*	*0*	*0*	*2*
0	*0*	*0*	*128,571*	*68,5714*	*51,4286*	*1*	*1*	*2*
0	*0*	*0*	*51,4286*	*128,571*	*0*	*0*	*1*	*2*
0	*1*	*1*	*129,286*	*17,1429*	*25,7143*	*1*	*0*	*2*
0	*0*	*0*	*17,1429*	*74,2857*	*0*	*0*	*1*	*2*
0	*0*	*0*	*20*	*37,8571*	*0*	*0*	*0*	*2*
0	*0*	*0*	*0*	*115,714*	*0*	*0*	*1*	*2*
0	*0*	*0*	*51,4286*	*0*	*0*	*0*	*0*	*2*
0	*0*	*0*	*64,2857*	*51,4286*	*0*	*1*	*0*	*2*
0	*0*	*0*	*15*	*25,7143*	*0*	*0*	*0*	*1*
0	*0*	*0*	*85,7143*	*137,143*	*0*	*1*	*1*	*1*
0	*0*	*0*	*162,857*	*34,2857*	*0*	*1*	*0*	*3*
0	*0*	*0*	*0*	*51,4286*	*0*	*0*	*0*	*1*
0	*0*	*0*	*135,714*	*128,571*	*0*	*1*	*1*	*2*
0	*0*	*0*	*98,5714*	*171,429*	*34,2857*	*1*	*1*	*1*
0	*0*	*0*	*0*	*98,5714*	*0*	*0*	*1*	*1*
0	*0*	*0*	*0*	*248,571*	*0*	*0*	*1*	*2*
0	*0*	*0*	*128,571*	*248,571*	*0*	*1*	*1*	*2*
0	*0*	*0*	*17,1429*	*34,2857*	*0*	*0*	*0*	*2*
0	*0*	*0*	*128,571*	*8,57143*	*0*	*1*	*0*	*1*
0	*0*	*0*	*94,2857*	*34,2857*	*0*	*1*	*0*	*1*
0	*0*	*0*	*21,4286*	*55,7143*	*0*	*0*	*0*	*2*
0	*0*	*0*	*248,571*	*162,857*	*0*	*1*	*1*	*2*
0	*0*	*0*	*251,429*	*154,286*	*77,1429*	*1*	*1*	*2*
0	*0*	*0*	*2,85714*	*13,5714*	*0*	*0*	*0*	*1*
0	*0*	*0*	*40*	*25,7143*	*0*	*0*	*0*	*2*
0	*0*	*0*	*248,571*	*17,1429*	*0*	*1*	*0*	*2*
0	*1*	*1*	*90*	*77,1429*	*0*	*1*	*1*	*2*
0	*0*	*0*	*25,7143*	*51,4286*	*0*	*0*	*0*	*2*
0	*0*	*0*	*40,7143*	*68,5714*	*0*	*0*	*1*	*2*
0	*0*	*0*	*18,5714*	*15,7143*	*17,1429*	*0*	*0*	*2*
0	*0*	*0*	*171,429*	*151,429*	*12,8571*	*1*	*1*	*2*
0	*0*	*0*	*2,85714*	*68,5714*	*0*	*0*	*1*	*1*
0	*0*	*0*	*28,5714*	*34,2857*	*0*	*0*	*0*	*2*
0	*0*	*0*	*5,71429*	*38,5714*	*0*	*0*	*0*	*3*
0	*1*	*1*	*222,857*	*94,2857*	*0*	*1*	*1*	*2*

0	0	0	180	231,429	154,286	1	1	2
0	0	0	142,857	214,286	0	1	1	2
0	0	0	68,5714	162,857	17,1429	1	1	1
0	0	0	30	47,1429	8,57143	0	0	2
0	0	0	107,143	25,7143	0	1	0	2
0	0	0	50	120	0	0	1	2
0	0	0	42,8571	111,429	0	0	1	2
0	1	1	198,571	180	0	1	1	2
0	0	0	8,57143	102,857	0	0	1	3
0	0	0	21,4286	25,7143	34,2857	0	1	2
0	0	0	42,8571	188,571	0	0	1	2
0	0	0	42,8571	68,5714	0	0	1	1
0	0	0	60	94,2857	0	1	1	1
0	0	0	0	317,143	60	0	1	2
0	0	0	85,7143	42,8571	0	1	0	2
0	0	0	14,2857	28,5714	0	0	0	2
0	0	0	92,8571	68,5714	25,7143	1	1	2
0	0	0	28,5714	30	0	0	0	2
0	0	0	10,7143	0	0	0	0	2
0	0	0	150	259,286	17,1429	1	1	2
0	0	0	42,8571	12,8571	0	0	0	2
0	0	0	42,8571	105	0	0	1	2
0	0	0	53,5714	25,7143	0	0	0	2
0	0	0	0	34,2857	0	0	0	1
0	0	0	100	20	15	1	0	2
0	0	0	162,857	34,2857	25,7143	1	1	1
0	1	1	360	8,57143	0	1	0	3
0	0	0	0	98,5714	0	0	1	3
0	0	0	102,857	141,429	0	1	1	2
0	0	0	47,1429	94,2857	0	0	1	2
0	0	0	81,4286	111,429	0	1	1	2
0	0	0	110	43,5714	0	1	0	2
0	0	0	17,8571	54,2857	0	0	0	2
0	0	0	17,1429	25,7143	0	0	0	3
0	0	0	139,286	120	0	1	1	2
0	0	0	11,4286	21,4286	0	0	0	3
0	0	0	162,857	105,714	0	1	1	3
0	0	0	64,2857	8,57143	0	1	0	3
0	0	0	165,714	12,8571	0	1	0	2
0	0	0	205	342,857	25,7143	1	1	2
0	0	0	171,429	137,143	0	1	1	2
0	0	0	5,71429	42,8571	0	0	0	2
0	0	0	162,857	12,8571	17,1429	1	0	2
0	0	0	154,286	231,429	0	1	1	2
0	0	0	40,7143	25,7143	0	0	0	2
0	0	0	38,5714	51,4286	0	0	0	2
0	0	0	64,2857	120	0	1	1	1
0	0	0	7,14286	51,4286	0	0	0	1

0	0	0	90	128,571	45	1	1	1
0	0	0	157,143	171,429	0	1	1	2
0	1	1	90	23,5714	0	1	0	2
0	0	0	120	131,429	0	1	1	2
0	1	1	180	11,4286	0	1	0	2
0	0	0	158,571	25,7143	8,57143	1	0	2
0	0	0	17,1429	90	0	0	1	2
0	0	0	0	85,7143	17,1429	0	1	2
0	1	1	132,857	145,714	0	1	1	2
0	0	0	55,7143	27,8571	5,71429	0	0	1
0	0	0	34,2857	25,7143	0	0	0	2
0	0	0	67,1429	142,857	0	1	1	2
0	0	0	55,7143	95,7143	0	0	1	2
0	0	0	0	128,571	0	0	1	1
0	0	0	137,143	47,1429	0	1	0	2
0	0	0	128,571	21,4286	0	1	0	2
0	0	0	25,7143	51,4286	0	0	0	1
0	0	0	205,714	94,2857	0	1	1	1
0	0	0	0	94,2857	0	0	1	2
0	0	0	28,5714	60	0	0	1	2
0	0	0	41,4286	30	0	0	0	2
0	0	0	167,143	180	25,7143	1	1	1
0	0	0	25,7143	55,7143	51,4286	0	1	2
0	0	0	60	214,286	0	1	1	3
0	0	0	42,8571	147,857	0	0	1	2
0	0	0	180	42,8571	0	1	0	2
0	0	0	90	137,143	0	1	1	2
0	0	0	80	42,8571	0	1	0	2
0	0	0	31,4286	11,4286	0	0	0	3
0	0	0	72,8571	171,429	0	1	1	2
0	0	0	85,7143	68,5714	0	1	1	2
0	0	0	25,7143	38,5714	0	0	0	3
0	0	0	68,5714	0	0	1	0	2
0	0	0	70,7143	25,7143	8,57143	1	0	2
0	0	0	5,71429	111,429	25,7143	0	1	2
0	0	0	0	25,7143	0	0	0	1
0	0	0	160,714	68,5714	0	1	1	2
1	0	1	77,1429	128,571	0	1	1	2
0	0	0	194,286	24,2857	0	1	0	2
0	0	0	158,571	180	42,8571	1	1	1
0	0	0	142,857	228,571	0	1	1	2
0	0	0	12,8571	17,1429	8,57143	0	0	2
0	0	0	25,7143	51,4286	0	0	0	2
0	0	0	45,7143	71,4286	11,4286	0	1	2
0	0	0	18,5714	65,7143	0	0	1	2
0	0	0	180	145,714	0	1	1	2
0	0	0	52,8571	22,8571	0	0	0	1
0	0	0	12,8571	188,571	0	0	1	3

0	0	0	17,1429	177,143	25,7143	0	1	1
0	0	0	21,4286	45	102,857	0	1	2
0	0	0	0	111,429	60	0	1	1
0	1	1	67,1429	60	0	1	1	2
0	0	0	85,7143	8,57143	0	1	0	2
0	1	1	180	182,857	30	1	1	2
0	0	0	0	20	0	0	0	3
0	0	0	78,5714	60	0	1	1	2
0	0	1	81,4286	42,1429	0	1	0	2
0	0	0	162,857	188,571	25,7143	1	1	2
0	0	0	158,571	165	21,4286	1	1	3
0	0	0	85,7143	42,8571	0	1	0	1
0	0	0	2,85714	17,1429	0	0	0	2
0	0	0	57,1429	42,8571	0	0	0	2
0	0	0	17,1429	32,8571	0	0	0	2
0	0	0	81,4286	57,1429	0	1	0	2
0	0	0	40	42,8571	0	0	0	2
0	0	0	17,1429	0	0	0	0	2
0	0	0	0	8,57143	0	0	0	2
0	0	0	14,2857	5	8,57143	0	0	2
0	0	0	54,2857	8,57143	0	0	0	3
0	0	0	14,2857	0	0	0	0	3
0	0	0	25,7143	21,4286	0	0	0	1
0	0	0	172,857	85,7143	25,7143	1	1	1
0	0	0	4,28571	0	0	0	0	2
0	0	0	21,4286	77,1429	0	0	1	2
0	0	0	50	10	0	0	0	2
0	0	0	0	34,2857	0	0	0	2
0	0	0	132,857	120	0	1	1	1
0	0	0	53,5714	158,571	0	0	1	2
0	0	0	107,143	82,8571	17,1429	1	1	2
0	0	0	128,571	51,4286	0	1	0	2
0	0	0	54,2857	60	0	0	1	2
0	0	0	17,1429	90	0	0	1	2
0	0	0	128,571	34,2857	0	1	0	1
0	0	0	10	34,2857	0	0	0	1
0	0	0	150	38,5714	0	1	0	1
0	0	0	17,1429	0	0	0	0	1
0	0	0	231,429	151,429	0	1	1	2
0	0	0	27,1429	34,2857	8,57143	0	0	1
0	0	1	8,57143	85,7143	0	0	1	2
0	0	0	128,571	107,143	25,7143	1	1	2
0	0	0	0	25,7143	34,2857	0	1	2
0	0	0	47,1429	58,5714	0	0	0	1
0	0	0	168,571	77,1429	0	1	1	2
0	0	0	51,4286	120	0	0	1	2
0	0	0	171,429	171,429	0	1	1	1
0	0	0	157,143	51,4286	0	1	0	1

0	0	0	0	42,8571	0	0	0	1
0	0	0	15	14,2857	0	0	0	2
0	0	0	68,5714	60	0	1	1	2
0	0	0	0	4,28571	0	0	0	2
0	0	0	30	17,1429	0	0	0	2
0	0	0	0	85,7143	0	0	1	3
0	0	0	175,714	42,8571	17,1429	1	1	2
0	0	0	81,4286	200	0	1	1	1
0	0	0	222,857	25,7143	0	1	0	2
0	0	0	0	47,1429	0	0	0	2
0	0	0	17,1429	83,5714	0	0	1	2
0	0	0	205,714	46,8571	0	1	0	2
0	0	0	0	0	0	0	0	2
0	1	1	140	68,5714	0	1	1	2
0	0	0	113,571	154,286	0	1	1	2
0	0	1	73,5714	120	34,2857	1	1	1
0	0	0	10	85,7143	0	0	1	2
0	0	0	21,4286	34,2857	0	0	0	2
0	0	0	60	282,857	0	1	1	1
0	0	0	8,57143	0	0	0	0	2
0	0	0	58,5714	68,5714	12,8571	0	1	3
0	0	0	34,2857	17,1429	0	0	0	2
0	0	0	17,1429	128,571	12,8571	0	1	2
0	0	0	15	34,2857	0	0	0	2
0	0	0	42,8571	25,7143	0	0	0	2
0	0	0	0	37,1429	0	0	0	2
0	0	0	51,4286	68,5714	0	0	1	1
0	0	0	158,571	158,571	0	1	1	2
0	0	1	18,5714	137,143	0	0	1	2
0	0	0	105,714	90	17,1429	1	1	3
0	0	0	145,714	85,7143	0	1	1	1
0	0	0	115,714	188,571	0	1	1	2
0	0	0	51,4286	17,1429	12,8571	0	0	2
0	1	1	180	68,5714	0	1	1	1
0	0	0	57,1429	128,571	17,1429	0	1	2
0	1	1	67,1429	368,571	17,1429	1	1	3
0	0	0	128,571	74,2857	0	1	1	2
0	0	0	145,714	48,5714	51,4286	1	1	2
0	0	0	137,143	42,8571	0	1	0	2
0	0	0	0	51,4286	0	0	0	1
0	0	0	128,571	85,7143	34,2857	1	1	2
0	0	0	147,857	51,4286	0	1	0	2
0	1	1	214,286	25,7143	0	1	0	2
0	0	0	94,2857	68,5714	0	1	1	2
0	0	0	0	205,714	0	0	1	2
0	0	0	77,1429	97,1429	17,1429	1	1	2
0	0	0	0	308,571	154,286	0	1	2
0	0	0	21,4286	68,5714	0	0	1	3

0	0	0	7,14286	17,1429	0	0	0	2
0	0	0	21,4286	94,2857	0	0	1	2
0	0	0	8,57143	94,2857	0	0	1	2
0	0	0	0	0	0	0	0	2
0	0	0	205,714	257,143	51,4286	1	1	2
0	0	0	164,286	171,429	77,1429	1	1	1
0	1	1	222,857	154,286	0	1	1	2
0	0	0	11,4286	154,286	0	0	1	2
0	1	1	114,286	34,2857	0	1	0	2
0	0	1	30	310,714	17,1429	0	1	2
0	0	0	0	218,571	25,7143	0	1	2
0	0	0	0	60	51,4286	0	1	2
0	0	1	25,7143	47,1429	34,2857	0	1	2
0	0	0	47,1429	70	0	0	1	2
0	0	0	160	23,5714	0	1	0	1
0	0	0	25,7143	6,42857	0	0	0	2
0	0	0	28,5714	68,5714	17,1429	0	1	2
0	1	1	120	154,286	0	1	1	2
0	0	0	77,1429	60	8,57143	1	1	2
0	0	0	214,286	351,429	180	1	1	2
0	0	0	60	120	17,1429	1	1	2
0	0	0	25,7143	77,1429	0	0	1	1
0	0	0	148,571	180	0	1	1	1
0	0	0	77,1429	49,2857	0	1	0	2
0	0	0	42,8571	180	0	0	1	1
0	0	0	12,8571	85,7143	77,1429	0	1	1
0	0	0	34,2857	0	0	0	0	2
0	0	0	30	17,1429	0	0	0	2
0	0	0	0	111,429	0	0	1	2
0	0	0	180	188,571	128,571	1	1	2
0	0	0	85,7143	68,5714	5,71429	1	1	1
1	0	1	12,8571	25,7143	120	0	1	2
0	1	1	240	60	0	1	1	2
0	0	0	30	231,429	0	0	1	1
0	0	0	0	38,5714	51,4286	0	1	2
0	0	0	145,714	145,714	38,5714	1	1	2
0	0	0	0	105	0	0	1	2
0	0	0	42,8571	34,2857	0	0	0	2
0	0	0	0	68,5714	0	0	1	1
0	0	0	61,4286	171,429	25,7143	1	1	1
0	0	0	55,7143	115,714	0	0	1	2
0	0	0	0	0	11,4286	0	0	2
0	0	0	60	120	143,571	1	1	2
0	0	0	188,571	137,143	34,2857	1	1	2
0	0	0	174,286	145,714	0	1	1	1
0	0	0	34,2857	34,2857	0	0	0	2
0	0	0	47,1429	25,7143	0	0	0	2
0	0	1	16,4286	95,7143	0	0	1	1

0	0	0	8,57143	51,4286	0	0	0	2
0	1	1	173,571	145,714	0	1	1	1
0	0	0	192,857	145,714	0	1	1	2
0	0	0	135,714	17,1429	34,2857	1	0	2
0	0	0	38,5714	25,7143	0	0	0	2
0	0	0	10,7143	102,857	0	0	1	1
0	0	0	21,4286	64,2857	5,71429	0	1	2
0	0	0	185,714	282,857	25,7143	1	1	2
0	0	0	47,1429	0	0	0	0	2
0	0	0	5,71429	60	0	0	1	3
0	0	0	8,57143	25,7143	0	0	0	2
0	0	0	19,2857	62,8571	0	0	1	2
0	0	0	0	137,143	0	0	1	2
0	0	0	167,143	17,1429	0	1	0	2
0	0	0	162,857	62,1429	0	1	1	2
0	0	0	51,4286	85,7143	25,7143	0	1	2
0	0	0	30	60	0	0	1	1
0	0	0	175,714	484,286	25,7143	1	1	2
0	1	1	95,7143	115,714	0	1	1	1
0	0	0	158,571	188,571	8,57143	1	1	2
0	0	0	17,1429	70,7143	0	0	1	2
0	0	0	40	85,7143	0	0	1	1
0	0	0	51,4286	60	0	0	1	2
0	0	0	14,2857	17,1429	0	0	0	2
0	0	0	50	32,8571	0	0	0	2
0	0	0	137,143	137,143	0	1	1	2
0	0	0	10	25,7143	0	0	0	2
0	0	0	17,1429	55	0	0	0	1
0	0	0	60	68,5714	0	1	1	2
0	0	0	30	180	17,1429	0	1	2
0	0	0	174,286	186,429	0	1	1	2
0	0	0	68,5714	291,429	0	1	1	1
0	0	0	2,85714	98,5714	0	0	1	3
0	0	0	98,5714	162,857	68,5714	1	1	1
0	0	0	137,143	252,857	0	1	1	3
0	0	0	122,857	85,7143	17,1429	1	1	2
0	0	0	0	87,1429	0	0	1	2
0	0	0	30,7143	180	0	0	1	2
0	0	1	68,5714	85,7143	0	1	1	2
0	0	1	137,143	81,4286	0	1	1	1
0	0	0	48,5714	111,429	42,8571	0	1	2
0	0	0	17,1429	25,7143	0	0	0	1
0	0	0	40	21,4286	0	0	0	1
0	0	0	38,5714	102,857	34,2857	0	1	2
0	0	0	10,7143	8,57143	0	0	0	2
0	0	0	0	145,714	85,7143	0	1	1
0	0	0	4,28571	205,714	0	0	1	3
0	0	0	17,1429	17,1429	0	0	0	2

0	0	0	30	17,1429	0	0	0	2
0	0	0	15	14,2857	25,7143	0	0	2
0	0	0	30	17,1429	0	0	0	2
0	1	1	167,143	102,857	0	1	1	2
0	0	0	19,2857	30	0	0	0	2
0	0	0	21,4286	8,57143	0	0	0	2
0	0	0	54,2857	51,4286	0	0	0	2
0	0	0	70,7143	51,4286	12,8571	1	1	1
0	0	0	0	197,143	8,57143	0	1	2
0	0	0	151,429	74,2857	0	1	1	2
0	0	0	21,4286	66,4286	0	0	1	1
0	0	0	34,2857	25,7143	0	0	0	2
0	0	0	137,143	102,857	0	1	1	2
0	0	0	62,8571	25,7143	17,1429	1	0	1
0	0	0	17,1429	17,1429	5,71429	0	0	2
0	0	0	154,286	240	0	1	1	3
0	1	1	180	180	0	1	1	2
0	0	0	21,4286	85,7143	0	0	1	2
0	0	0	0	214,286	25,7143	0	1	2
0	0	0	61,4286	0	0	1	0	2
0	0	0	150	98,5714	0	1	1	3
1	0	1	48,5714	90	85,7143	0	1	2
0	0	1	60	72,8571	0	1	1	2
0	0	1	54,2857	25,7143	34,2857	0	1	2
0	0	0	72,8571	154,286	0	1	1	2
0	0	0	175,714	231,429	0	1	1	2
0	0	0	60	25,7143	0	1	0	3
0	1	1	135	128,571	60	1	1	1
0	0	0	0	120	0	0	1	2
0	0	0	60,7143	60	14,2857	1	1	2
0	0	0	30	35,7143	17,1429	0	0	2
0	0	0	0	12,8571	4,28571	0	0	2
0	0	0	142,857	192,857	0	1	1	2
0	0	0	0	19,2857	14,2857	0	0	2
1	0	1	7,14286	325,714	0	0	1	3
0	0	0	154,286	17,1429	0	1	0	2
0	0	0	115,714	124,286	0	1	1	2
0	0	0	7,14286	57,1429	17,1429	0	1	2
0	0	0	145,714	274,286	0	1	1	2
0	0	0	94,2857	68,5714	0	1	1	2
0	0	0	25,7143	60	0	0	1	3
0	0	0	0	34,2857	0	0	0	2
0	0	0	338,571	147,143	0	1	1	2
0	0	0	162,857	85,7143	0	1	1	2
0	0	0	1,42857	30	0	0	0	2
0	0	0	137,143	111,429	0	1	1	2
0	0	0	10,7143	0	0	0	0	1
0	0	0	39,2857	0	18,8571	0	0	2

0	0	0	128,571	188,571	0	1	1	2
0	0	0	152,857	111,429	25,7143	1	1	1
0	0	0	51,4286	84,2857	0	0	1	3
0	0	0	122,857	197,143	0	1	1	2
0	0	0	14,2857	71,4286	180	0	1	2
0	0	0	63,5714	102,857	0	1	1	2
0	0	0	72,8571	60	0	1	1	2
0	0	0	0	91,4286	0	0	1	2
0	0	0	121,429	42,8571	0	1	0	1
0	0	0	0	154,286	0	0	1	2
0	0	0	0	34,2857	8,57143	0	0	2
0	0	0	77,1429	248,571	0	1	1	2
0	0	0	35,7143	85,7143	0	0	1	2
0	0	0	54,2857	4,28571	0	0	0	2
0	0	0	41,4286	28,5714	0	0	0	2
0	0	0	2,85714	128,571	102,857	0	1	2
0	0	0	51,4286	128,571	17,1429	0	1	2
0	0	0	0	134,286	180	0	1	2
0	0	0	39,2857	25,7143	0	0	0	1
0	0	0	25,7143	45	51,4286	0	1	2
0	0	0	50	42,8571	0	0	0	2
0	0	0	42,8571	25,7143	128,571	0	1	3
0	0	0	72,8571	162,857	25,7143	1	1	2
0	0	0	38,5714	111,429	0	0	1	2
0	0	0	128,571	317,143	34,2857	1	1	1
0	0	0	20	12,8571	0	0	0	1
0	0	0	17,1429	42,8571	8,57143	0	0	3
0	0	0	64,2857	17,1429	0	1	0	2
0	0	0	197,143	308,571	25,7143	1	1	2
0	0	0	22,8571	22,8571	0	0	0	2
0	0	0	102,857	34,2857	0	1	0	3
0	0	0	10,7143	145,714	0	0	1	2
0	1	1	171,429	180	0	1	1	1
0	0	0	77,1429	77,1429	0	1	1	2
0	0	0	94,2857	171,429	7,14286	1	1	2
0	0	0	105	120	8,57143	1	1	1
0	0	0	0	171,429	0	0	1	1
0	0	0	45	34,2857	0	0	0	2
0	0	0	0	15,7143	0	0	0	2
0	0	1	190,714	102,857	0	1	1	2
0	0	0	0	62,8571	0	0	1	2
0	0	0	25,7143	60	0	0	1	2
0	0	0	111,429	15,4286	0	1	0	2
0	0	0	51,4286	185,714	42,8571	0	1	1
0	0	0	31,4286	62,1429	0	0	1	2
0	0	0	74,2857	0	0	1	0	2
0	0	0	50	0	0	0	0	2
0	0	0	42,8571	42,8571	0	0	0	3

0	0	0	0	0	8,57143	0	0	2
0	0	0	187,143	257,143	8,57143	1	1	1
0	0	0	102,857	180	0	1	1	2
0	0	0	51,4286	111,429	0	0	1	1
0	0	0	53,5714	32,1429	8,57143	0	0	2
0	0	0	124,286	51,4286	0	1	0	1
0	0	0	12,8571	42,8571	0	0	0	2
0	0	0	171,429	51,4286	0	1	0	2
0	0	0	200	68,5714	0	1	1	2
0	0	0	158,571	162,857	8,57143	1	1	3
0	0	0	62,1429	180	0	1	1	2
0	0	0	173,571	77,1429	0	1	1	2
0	0	0	102,857	0	0	1	0	2
0	0	0	32,1429	0	0	0	0	2
0	0	0	14,2857	62,8571	0	0	1	2
0	0	0	14,2857	41,4286	0	0	0	2
0	0	0	10,7143	85,7143	0	0	1	2
0	0	0	0	95,7143	0	0	1	2
0	0	0	0	31,4286	12,8571	0	0	3
0	0	0	8,57143	205,714	0	0	1	3
0	0	0	122,857	228,571	0	1	1	2
0	0	0	150	192,857	0	1	1	1
0	0	0	102,857	17,1429	0	1	0	3
0	0	0	141,429	42,8571	0	1	0	2
0	0	0	175,714	227,143	0	1	1	2
0	0	0	26,4286	122,143	34,2857	0	1	2
0	0	0	192,857	228,571	0	1	1	2
0	0	0	140	282,857	0	1	1	2
0	0	0	128,571	8,57143	0	1	0	2
0	0	0	85,7143	160	5,71429	1	1	2
0	0	0	24,2857	51,4286	0	0	0	2
0	0	0	20	68,5714	0	0	1	1
0	0	0	10,7143	85,7143	0	0	1	2
0	0	0	154,286	201,429	180	1	1	2
0	0	0	128,571	188,571	0	1	1	2
0	0	0	4,28571	5,71429	0	0	0	2
0	0	0	17,1429	96,4286	37,1429	0	1	2
0	0	0	154,286	162,857	0	1	1	2
0	0	0	17,1429	5,71429	17,1429	0	0	2
0	0	0	68,5714	85,7143	0	1	1	1
0	0	0	0	60	0	0	1	2
0	0	0	30	30	0	0	0	3
0	0	0	167,143	51,4286	0	1	0	3
0	0	0	51,4286	51,4286	0	0	0	2
0	0	0	6,42857	141,429	0	0	1	2
0	0	0	115,714	53,5714	0	1	0	2
0	0	0	0	291,429	0	0	1	2
0	0	0	34,2857	30	4,28571	0	0	2

0	0	0	17,1429	17,1429	0	0	0	2
0	0	0	111,429	42,8571	0	1	0	2
0	0	0	71,4286	25,7143	0	1	0	2
0	0	0	22,8571	0	0	0	0	2
0	0	0	0	11,4286	0	0	0	2
0	0	0	94,2857	8,57143	12,8571	1	0	2
0	0	0	45,7143	0	0	0	0	3
0	0	0	175,714	60	0	1	1	2
0	0	0	62,1429	34,2857	94,2857	1	1	2
0	0	0	31,4286	257,143	0	0	1	1
0	0	0	171,429	270	0	1	1	3
0	0	0	162,857	102,857	154,286	1	1	1
0	0	0	51,4286	42,8571	0	0	0	2
0	0	0	21,4286	12,8571	0	0	0	1
0	0	0	132,857	141,429	34,2857	1	1	2
0	0	0	94,2857	77,1429	0	1	1	2
0	1	1	68,5714	65	0	1	1	3
0	0	0	0	96,4286	0	0	1	2
0	0	0	151,429	248,571	25,7143	1	1	2
0	0	0	35,7143	38,5714	0	0	0	3
0	0	0	21,4286	94,2857	8,57143	0	1	2
0	0	0	4,28571	50	0	0	0	2
0	0	0	128,571	154,286	0	1	1	2
0	0	0	32,1429	64,2857	34,2857	0	1	2
0	0	0	47,1429	60	0	0	1	2
0	0	0	0	231,429	128,571	0	1	2
0	0	0	0	150	0	0	1	2
0	0	0	0	25,7143	0	0	0	2
0	0	0	150	158,571	0	1	1	2
0	1	1	60	73,7143	0	1	1	3
0	0	0	8,57143	34,2857	0	0	0	2
0	0	0	128,571	25	0	1	0	2
0	0	0	54,2857	94,2857	0	0	1	2
0	0	0	54,2857	122,143	0	0	1	3
0	0	0	20	42,8571	0	0	0	2
0	0	0	12,8571	40	0	0	0	2
0	0	0	62,1429	214,286	25,7143	1	1	2
0	0	0	18,5714	8,57143	0	0	0	2
0	0	0	14,2857	120	0	0	1	2
0	0	0	14,2857	54,2857	0	0	0	2
0	0	0	0	120	0	0	1	2
0	0	0	8,57143	0	0	0	0	2
0	1	1	199,286	17,1429	0	1	0	2
0	0	0	20	51,4286	0	0	0	2
0	0	0	45,4286	115,714	4,28571	0	1	2
0	0	0	47,8571	66,4286	10,7143	0	1	2
0	0	0	51,4286	25,7143	0	0	0	2
0	0	0	17,1429	72,8571	0	0	1	3

0	0	0	0	248,571	8,57143	0	1	2
0	0	0	128,571	171,429	128,571	1	1	1
0	0	0	5,71429	25,7143	0	0	0	2
0	0	0	68,5714	38,5714	0	1	0	2
0	0	0	30	17,1429	0	0	0	2
0	0	0	68,5714	75	0	1	1	1
0	0	0	60	51,4286	0	1	0	1
0	0	0	45	38,5714	0	0	0	2
0	0	0	17,1429	51,4286	0	0	0	2
0	0	0	282,857	167,143	21,4286	1	1	1
0	1	1	79,2857	111,429	17,1429	1	1	2
0	0	0	0	21,4286	0	0	0	3
0	0	0	51,4286	120	8,57143	0	1	2
0	0	0	15,7143	42,8571	0	0	0	2
0	0	0	0	51,4286	12,8571	0	1	2
0	0	0	151,429	160	0	1	1	2
0	0	0	0	257,143	0	0	1	2
0	0	0	12,8571	205,714	25,7143	0	1	2
0	0	0	77,1429	6,42857	0	1	0	2
0	0	0	120	72,8571	0	1	1	2
0	0	0	128,571	47,1429	0	1	0	1
0	0	0	6,42857	110,714	59,2857	0	1	2
1	0	1	15	180	0	0	1	2
0	0	0	205,714	351,429	0	1	1	1
0	0	0	17,1429	37,1429	30	0	1	2
0	0	0	128,571	33,5714	0	1	0	2
0	0	0	85,7143	51,4286	0	1	0	2
0	0	0	38,5714	34,2857	0	0	0	1
0	0	0	122,857	64,2857	4,28571	1	1	1
0	0	0	85,7143	25,7143	0	1	0	2
0	0	0	150	222,857	25,7143	1	1	2
0	0	0	135,714	192,857	0	1	1	2
0	0	0	49,2857	51,4286	0	0	0	2
0	0	0	40	27,1429	0	0	0	1
0	0	0	15	0	0	0	0	1
0	0	0	65,7143	205,714	0	1	1	2
0	0	0	51,4286	34,2857	0	0	0	3
0	0	0	132,857	25,7143	0	1	0	2
0	0	0	5,71429	0	0	0	0	3
0	0	0	141,429	75	8,57143	1	1	2
0	0	0	141,429	36,4286	6,42857	1	0	2
0	0	0	192,857	180	17,1429	1	1	2
0	1	1	77,1429	11,4286	0	1	0	3
0	0	0	0	85,7143	85,7143	0	1	2
0	0	0	34,2857	102,857	17,1429	0	1	2
0	0	0	55,7143	25,7143	6,42857	0	0	2
0	0	0	137,143	2,85714	0	1	0	3
0	0	0	25,7143	77,1429	0	0	1	2

0	1	1	135	60	0	1	1	3
0	0	0	145,714	42,8571	0	1	0	1
0	0	0	17,1429	4,28571	0	0	0	1
0	0	0	0	257,143	0	0	1	2
0	0	0	96,4286	17,1429	0	1	0	2
0	0	0	25,7143	17,1429	102,857	0	1	2
0	0	0	0	87,1429	0	0	1	2
0	0	0	4,28571	25,7143	0	0	0	2
0	1	1	248,571	248,571	0	1	1	2
0	0	0	0	6,42857	8,57143	0	0	2
0	0	0	188,571	34,2857	0	1	0	2
0	0	0	21,4286	85,7143	0	0	1	2
0	0	0	128,571	77,1429	0	1	1	2
0	0	0	25,7143	6,42857	0	0	0	2
0	0	0	6,42857	17,1429	0	0	0	1
0	0	0	1,42857	17,1429	0	0	0	2
0	0	0	37,1429	225	0	0	1	2
0	0	0	49,2857	114,286	0	0	1	2
0	0	0	25,7143	64,2857	0	0	1	3
0	0	0	6,42857	20,5714	4,28571	0	0	3
0	0	0	14,2857	42,8571	0	0	0	2
0	0	1	205,714	47,1429	0	1	0	1
0	0	1	141,429	85,7143	12,8571	1	1	2
0	1	1	162,857	60	0	1	1	1
0	0	0	20	235,714	102,857	0	1	2
0	1	1	137,143	180	25,7143	1	1	1
0	1	1	91,4286	85,7143	0	1	1	2
0	0	0	45	171,429	0	0	1	2
0	0	0	50	334,286	154,286	0	1	2
0	0	0	25,7143	34,2857	0	0	0	2
0	0	0	25,7143	65,7143	0	0	1	2
0	0	0	0	42,8571	0	0	0	1
0	0	0	9,28571	0	0	0	0	2
0	0	0	34,2857	68,5714	68,5714	0	1	1
0	0	0	150	25,7143	0	1	0	2
0	0	0	50,7143	17,1429	0	0	0	1
0	0	0	4,28571	111,429	0	0	1	2
0	0	0	105,714	42,8571	111,429	1	1	2
0	0	0	25,7143	160	0	0	1	2
0	0	0	38,5714	231,429	0	0	1	2
0	0	0	25,7143	205,714	0	0	1	2
0	0	0	125,714	17,1429	0	1	0	1
0	0	0	96,4286	17,1429	0	1	0	1
0	0	0	0	120	0	0	1	2
0	1	1	120	98,5714	0	1	1	1
0	0	0	5,71429	188,571	0	0	1	2
0	0	0	14,2857	42,8571	0	0	0	2
0	0	0	105,714	66,4286	0	1	1	2

0	0	0	2,14286	0	0	0	0	2
0	0	0	115,714	145,714	0	1	1	2
0	0	0	30,7143	31,4286	2,85714	0	0	1
0	0	0	12,8571	60	0	0	1	1
0	0	0	137,143	0	0	1	0	1
0	1	1	240	248,571	0	1	1	2
0	0	0	38,5714	154,286	8,57143	0	1	1
0	0	0	17,1429	120	0	0	1	2
0	0	0	20	41,4286	0	0	0	2
0	0	0	12,8571	38,5714	0	0	0	3
0	0	0	17,1429	30	0	0	0	1
0	0	0	334,286	102,857	111,429	1	1	3
0	0	0	45	51,4286	0	0	0	2
0	0	0	77,1429	85,7143	0	1	1	2
0	0	0	4,28571	197,143	0	0	1	1
0	0	0	25,7143	51,4286	0	0	0	2
0	0	0	34,2857	34,2857	0	0	0	2
0	0	0	0	51,4286	0	0	0	2
0	0	0	32,1429	42,8571	0	0	0	2
0	0	0	12,8571	42,8571	0	0	0	1
0	0	0	34,2857	30	0	0	0	1
0	0	0	18,5714	162,857	0	0	1	2
0	0	0	0	60	0	0	1	2
0	0	0	0	94,2857	8,57143	0	1	2
0	0	0	42,8571	43,5714	0	0	0	2
0	0	0	138,571	308,571	34,2857	1	1	2
0	0	0	128,571	167,143	8,57143	1	1	1
0	0	0	135	51,4286	0	1	0	2
0	0	0	128,571	34,2857	0	1	0	2
0	0	0	80	42,8571	0	1	0	1
0	0	0	98,5714	51,4286	25,7143	1	1	2
0	0	0	188,571	347,143	25,7143	1	1	1
0	1	1	214,286	60	17,1429	1	1	3
0	1	1	290	111,429	0	1	1	1
0	0	0	0	42,8571	17,1429	0	1	2
0	0	0	14,2857	91,4286	0	0	1	2
0	0	0	157,143	180,714	0	1	1	2
0	0	0	8,57143	25,7143	0	0	0	2
0	1	1	145,714	128,571	34,2857	1	1	2
0	0	1	30,7143	128,571	2,85714	0	1	2
0	0	0	128,571	94,2857	0	1	1	2
0	0	0	128,571	82,8571	0	1	1	2
0	0	0	20,7143	34,2857	5,71429	0	0	1
0	0	0	60	282,857	0	1	1	3
0	0	0	57,1429	8,57143	0	0	0	2
0	0	0	21,4286	192,857	0	0	1	1
0	1	1	67,1429	11,4286	12,8571	1	0	2
0	0	0	60	141,429	0	1	1	3

0	*0*	*0*	*17,1429*	*25,7143*	*34,2857*	*0*	*1*	*2*
0	*0*	*0*	*135*	*175,714*	*17,1429*	*1*	*1*	*2*
1	*1*	*1*	*180*	*68,5714*	*51,4286*	*1*	*1*	*2*
0	*0*	*0*	*26,4286*	*17,1429*	*0*	*0*	*0*	*2*
0	*0*	*0*	*60*	*40*	*0*	*1*	*0*	*2*
0	*0*	*0*	*25,7143*	*260*	*0*	*0*	*1*	*2*
0	*0*	*0*	*18,5714*	*140*	*0*	*0*	*1*	*2*
0	*0*	*0*	*0*	*157,143*	*34,2857*	*0*	*1*	*2*
0	*0*	*0*	*19,2857*	*51,4286*	*0*	*0*	*0*	*2*
0	*0*	*0*	*64,2857*	*145,714*	*17,1429*	*1*	*1*	*1*
0	*0*	*0*	*0*	*274,286*	*25,7143*	*0*	*1*	*3*
0	*0*	*0*	*4,28571*	*85,7143*	*0*	*0*	*1*	*2*
0	*0*	*0*	*30*	*141,429*	*0*	*0*	*1*	*2*
0	*0*	*0*	*30*	*50*	*0*	*0*	*0*	*2*
0	*0*	*0*	*42,8571*	*25,7143*	*0*	*0*	*0*	*2*
0	*0*	*0*	*57,8571*	*51,4286*	*0*	*0*	*0*	*1*
0	*0*	*0*	*77,1429*	*21,4286*	*17,1429*	*1*	*0*	*2*
0	*0*	*0*	*28,5714*	*42,8571*	*34,2857*	*0*	*1*	*2*
0	*0*	*0*	*128,571*	*40,7143*	*0*	*1*	*0*	*2*
0	*0*	*0*	*40,7143*	*25,7143*	*0*	*0*	*0*	*2*
0	*0*	*0*	*188,571*	*25,7143*	*0*	*1*	*0*	*1*
0	*0*	*0*	*20*	*317,143*	*0*	*0*	*1*	*2*
0	*0*	*0*	*25,7143*	*120*	*60*	*0*	*1*	*2*
0	*0*	*0*	*23,5714*	*40,7143*	*0*	*0*	*0*	*2*
0	*0*	*0*	*8,57143*	*0*	*0*	*0*	*0*	*2*
0	*1*	*1*	*162,857*	*60*	*0*	*1*	*1*	*2*
0	*0*	*0*	*84,2857*	*34,2857*	*0*	*1*	*0*	*2*
0	*0*	*0*	*8,57143*	*160,714*	*0*	*0*	*1*	*2*
0	*1*	*1*	*197,143*	*60*	*0*	*1*	*1*	*2*
0	*0*	*0*	*60*	*77,1429*	*0*	*1*	*1*	*3*
0	*0*	*0*	*128,571*	*94,2857*	*0*	*1*	*1*	*2*
0	*0*	*0*	*85*	*22,8571*	*12,8571*	*1*	*0*	*2*
0	*0*	*0*	*7,14286*	*17,1429*	*0*	*0*	*0*	*2*
0	*0*	*0*	*12,8571*	*51,4286*	*0*	*0*	*0*	*2*
0	*0*	*0*	*145,714*	*137,143*	*0*	*1*	*1*	*2*
0	*1*	*1*	*120*	*60*	*34,2857*	*1*	*1*	*1*
0	*0*	*0*	*137,143*	*68,5714*	*0*	*1*	*1*	*2*
0	*0*	*0*	*111,429*	*51,4286*	*17,1429*	*1*	*1*	*3*
0	*0*	*0*	*8,57143*	*51,4286*	*0*	*0*	*0*	*2*
0	*0*	*0*	*17,1429*	*8,57143*	*0*	*0*	*0*	*2*
0	*0*	*0*	*0*	*268,571*	*0*	*0*	*1*	*3*
0	*0*	*0*	*60*	*42,8571*	*8,57143*	*1*	*0*	*1*
0	*0*	*1*	*192,857*	*205,714*	*25,7143*	*1*	*1*	*2*
0	*0*	*0*	*51,4286*	*218,571*	*25,7143*	*0*	*1*	*1*
0	*0*	*0*	*6,28571*	*34,2857*	*17,1429*	*0*	*0*	*2*
0	*0*	*0*	*94,2857*	*302,857*	*60*	*1*	*1*	*2*
0	*0*	*0*	*132*	*225*	*0*	*1*	*1*	*2*
0	*0*	*0*	*148,571*	*85,7143*	*0*	*1*	*1*	*2*

0	0	0	90	71,4286	0	1	1	2
0	0	0	42,8571	128,571	0	0	1	3
0	0	0	0	17,1429	17,1429	0	0	3
0	0	0	128,571	94,2857	0	1	1	2
0	0	0	19,2857	51,4286	0	0	0	2
0	0	1	150	145,714	0	1	1	3
0	0	0	42,8571	53,5714	42,8571	0	1	2
0	0	0	51,4286	162,857	0	0	1	2
0	0	0	225	50,7143	0	1	0	1
0	0	0	167,143	45,7143	0	1	0	2
0	0	0	0	154,286	0	0	1	2
0	1	1	60	42,8571	0	1	0	2
0	0	0	48,5714	64,2857	0	0	1	2
0	0	0	188,571	222,857	0	1	1	2
0	0	0	145,714	68,5714	25,7143	1	1	3
0	0	0	11,4286	77,1429	0	0	1	2
0	0	0	128,571	320	85,7143	1	1	2
0	0	0	7,14286	120	25,7143	0	1	1
0	0	0	22,8571	51,4286	0	0	0	1
0	0	0	0	205,714	17,1429	0	1	2
0	0	0	60	60	0	1	1	2
0	0	0	32,8571	17,1429	0	0	0	1
0	0	1	197,143	231,429	0	1	1	2
0	0	0	171,429	128,571	0	1	1	2
0	0	0	6,42857	10,7143	4,28571	0	0	1
0	0	0	30	30	0	0	0	2
0	0	1	34,2857	111,429	25,7143	0	1	2
0	0	0	231,429	135	25,7143	1	1	2
0	1	1	131,429	17,1429	0	1	0	1
0	0	0	0	34,2857	17,1429	0	0	2
0	0	0	34,2857	58,5714	0	0	0	3
0	0	0	108,571	158,571	0	1	1	2
0	0	0	54,2857	34,2857	15	0	0	2
0	1	1	60	60	0	1	1	1
0	0	0	142,857	162,857	0	1	1	2
0	0	0	34,2857	51,4286	0	0	0	2
0	0	0	205,714	205,714	0	1	1	2
0	0	0	188,571	180	0	1	1	2
0	0	0	0	300	0	0	1	1
0	0	0	42,8571	0	17,1429	0	0	2
0	0	0	180	338,571	0	1	1	2
0	0	0	102,857	77,1429	0	1	1	2
0	0	0	111,429	17,1429	0	1	0	2
0	0	0	157,143	77,1429	0	1	1	2
0	1	1	180	51,4286	0	1	0	2
0	0	0	104,286	17,1429	0	1	0	2
0	0	0	30,7143	34,2857	0	0	0	2
0	0	0	34,2857	154,286	0	0	1	3

0	0	0	14,2857	25,7143	11,4286	0	0	2
0	1	1	229,286	35,7143	0	1	0	1
0	0	0	6,42857	51,4286	0	0	0	1
0	0	0	12,8571	4,28571	0	0	0	2
0	0	0	98,5714	4,28571	0	1	0	2
0	0	0	145,714	180	0	1	1	1
0	0	0	0	218,571	0	0	1	2
0	0	0	21,4286	51,4286	0	0	0	2
0	0	0	17,1429	85,7143	0	0	1	2
0	0	0	55,7143	25,7143	0	0	0	1
0	0	0	0	68,5714	0	0	1	2
0	0	0	68,5714	10	0	1	0	2
0	0	0	51,4286	66,4286	32,1429	0	1	1
0	0	0	17,1429	51,4286	0	0	0	1
0	1	1	197,143	208,571	17,1429	1	1	2
0	0	0	31,4286	25,7143	0	0	0	2
0	0	0	145,714	282,857	0	1	1	1
0	0	0	17,1429	102,857	154,286	0	1	3
0	0	0	11,4286	120	0	0	1	2
0	1	1	177,143	38,5714	0	1	0	2
0	0	0	21,4286	202,857	0	0	1	2
0	0	0	140	291,429	44,2857	1	1	2
0	0	0	0	62,1429	0	0	1	1
1	1	1	178,571	222,857	0	1	1	2
1	0	1	7,14286	145,714	0	0	1	3
0	0	0	120	137,143	0	1	1	2
0	0	0	138,571	31,4286	0	1	0	2
0	0	0	145,714	85,7143	0	1	1	3
0	0	0	0	21,4286	12,8571	0	0	2
0	0	0	274,286	214,286	0	1	1	1
0	0	0	171,429	94,2857	0	1	1	1
0	0	0	128,571	77,1429	0	1	1	2
0	0	0	25,7143	180	0	0	1	2
0	0	0	135,714	30	0	1	0	2
0	0	0	178,571	225	0	1	1	2
0	0	0	85,7143	37,1429	25,7143	1	1	2
0	0	0	2,85714	60	0	0	1	1
0	1	1	342,857	282,857	25,7143	1	1	1
0	0	0	138,571	167,143	0	1	1	3
0	0	0	148,571	248,571	51,4286	1	1	3
0	0	0	14,2857	31,4286	0	0	0	2
0	1	1	77,1429	23,5714	0	1	0	1
0	0	0	12,8571	34,2857	0	0	0	2
0	0	0	42,8571	71,4286	0	0	1	2
0	0	1	190	261,429	0	1	1	2
0	0	0	0	167,143	34,2857	0	1	1
0	0	0	2,85714	79,2857	25,7143	0	1	2
0	0	0	24,2857	120	60	0	1	2

0	1	1	205,714	60	51,4286	1	1	2
0	1	1	300	214,286	70,7143	1	1	1
0	0	0	27,1429	51,4286	0	0	0	1
0	0	0	128,571	128,571	8,57143	1	1	2
0	0	1	40	145,714	0	0	1	1
0	0	0	120	94,2857	17,1429	1	1	2
0	0	1	111,429	27,1429	0	1	0	2
0	0	0	49,2857	23,5714	0	0	0	2
0	0	0	122,857	244,286	17,1429	1	1	2
0	0	0	180	115,714	3,42857	1	1	2
0	0	0	7,14286	139,286	0	0	1	3
0	0	0	128,571	161,429	0	1	1	1
0	0	0	180	186,429	8,57143	1	1	2
0	0	0	7,14286	40,7143	0	0	0	2
0	0	0	128,571	260	102,857	1	1	2
0	0	0	64,2857	51,4286	0	1	0	3
0	0	0	17,1429	25,7143	0	0	0	2
0	0	0	30	20	0	0	0	2
0	0	0	8,57143	25,7143	0	0	0	2
0	0	0	30	17,1429	0	0	0	1
0	1	1	60	300	0	1	1	2
0	0	0	58,5714	214,286	0	0	1	2
0	0	0	157,143	50,7143	0	1	0	1
0	0	0	16,4286	51,4286	0	0	0	2
0	0	0	8,57143	51,4286	0	0	0	2
0	0	0	25,7143	0	0	0	0	1
0	0	0	44,2857	14,2857	0	0	0	2
0	1	1	140	55,7143	17,1429	1	1	2
0	0	0	120	51,4286	0	1	0	2
0	0	0	51,4286	51,4286	0	0	0	1
0	0	0	35,7143	51,4286	0	0	0	1
0	1	1	77,1429	25,7143	0	1	0	2
0	0	0	42,8571	51,4286	0	0	0	2
0	0	0	137,143	60	0	1	1	1
0	0	0	128,571	188,571	8,57143	1	1	3
0	0	0	34,2857	10,7143	0	0	0	2
0	1	1	214,286	25,7143	0	1	0	1
0	0	0	38,5714	191,429	12,8571	0	1	2
0	0	0	41,4286	60	0	0	1	1
0	0	0	14,2857	240	180	0	1	2
0	0	0	55,7143	34,2857	0	0	0	1
0	0	0	18,5714	128,571	0	0	1	2
0	0	0	72,8571	49,2857	17,1429	1	1	2
0	0	0	22,8571	17,1429	0	0	0	2
0	1	1	180	21,4286	0	1	0	2
0	0	0	62,1429	180	0	1	1	2
0	0	0	180	72,8571	37,1429	1	1	2
0	0	0	28,5714	8,57143	0	0	0	3

0	1	1	60	94,2857	0	1	1	1
0	0	0	45	51,4286	25,7143	0	1	2
1	0	1	57,1429	128,571	77,1429	0	1	2
0	0	0	15,7143	47,1429	0	0	0	2
0	0	1	31,4286	77,1429	17,1429	0	1	2
1	0	1	282,857	188,571	0	1	1	2
0	0	0	90	25,7143	0	1	0	2
0	0	0	180	334,286	102,857	1	1	2
0	0	0	5,71429	51,4286	0	0	0	2
0	0	0	38,5714	64,2857	0	0	1	2
0	0	0	0	102,857	0	0	1	3
0	0	0	40,7143	154,286	0	0	1	2
1	1	1	82,8571	173,571	0	1	1	2
0	0	1	270	197,143	17,1429	1	1	2
0	0	0	14,2857	25,7143	0	0	0	2
0	0	0	12,8571	154,286	0	0	1	2
0	0	0	25,7143	51,4286	0	0	0	2
0	0	0	42,8571	60	0	0	1	2
0	0	0	171,429	17,1429	0	1	0	1
0	0	0	17,1429	25,7143	25,7143	0	0	2
0	0	0	77,1429	0	0	1	0	2
0	0	0	186,429	66,4286	0	1	1	1
0	0	0	222,857	180	0	1	1	2
0	0	0	0	34,2857	68,5714	0	1	2
0	0	0	37,1429	17,1429	17,1429	0	0	2
0	0	0	128,571	122,857	17,1429	1	1	2
0	0	0	0	42,8571	25,7143	0	1	2
0	0	0	7,14286	17,1429	0	0	0	1
0	0	0	145,714	171,429	0	1	1	2
0	0	0	154,286	85,7143	0	1	1	2
0	0	0	4,28571	21,4286	0	0	0	3
0	0	0	51,4286	25,7143	0	0	0	2
0	0	0	77,1429	120	0	1	1	3
0	0	0	231,429	295,714	0	1	1	2
0	0	0	17,1429	77,1429	0	0	1	2
0	0	0	0	0	0	0	0	2
0	0	0	10	135	0	0	1	2
0	0	0	57,8571	34,2857	0	0	0	2
0	1	1	60	96,4286	34,2857	1	1	2
0	0	0	38,5714	85,7143	0	0	1	1
0	0	0	8,57143	128,571	0	0	1	1
0	0	0	171,429	180	0	1	1	2
1	0	1	183,571	35	102,857	1	1	2
0	0	0	3,57143	34,2857	0	0	0	1
0	0	0	137,143	338,571	0	1	1	2
0	0	0	12,8571	42,8571	25,7143	0	1	1
0	0	0	8,57143	68,5714	0	0	1	3
0	0	0	60	265,714	0	1	1	2

0	0	0	20	15	0	0	0	1
0	0	0	17,1429	68,5714	6,42857	0	1	2
0	0	0	60	282,857	0	1	1	2
0	0	0	19,2857	70	17,1429	0	1	2
0	0	0	2,14286	82,8571	0	0	1	2
0	0	0	3,57143	11,4286	8,57143	0	0	2
0	0	0	42,8571	51,4286	0	0	0	2
0	0	0	25,7143	17,1429	0	0	0	2
0	0	0	0	265,714	51,4286	0	1	2
0	0	0	68,5714	180	0	1	1	2
0	0	0	142,857	162,857	0	1	1	2
0	0	0	205,714	38,5714	0	1	0	2
0	0	0	0	128,571	0	0	1	3
0	0	0	54,2857	77,1429	0	0	1	2
0	1	1	195,714	120	25,7143	1	1	2
0	0	0	51,4286	167,143	25,7143	0	1	2
0	0	0	21,4286	77,1429	0	0	1	1
0	0	0	0	25,7143	25,7143	0	0	2
1	0	1	25,7143	42,8571	85,7143	0	1	2
0	0	0	154,286	222,857	0	1	1	2
0	0	0	145,714	265,714	0	1	1	2
0	0	0	68,5714	137,143	17,1429	1	1	2
0	0	0	20	25,7143	0	0	0	1
0	0	0	11,4286	77,1429	0	0	1	1
0	0	0	64,2857	28,5714	0	1	0	2
0	0	0	100	49,2857	0	1	0	2
0	0	0	51,4286	257,143	0	0	1	3
0	0	0	14,2857	120	0	0	1	2
0	0	0	90	60	0	1	1	2
0	1	1	180	240	25,7143	1	1	2
0	0	0	95,7143	120	25,7143	1	1	2
0	0	0	38,5714	76,4286	0	0	1	2
0	0	0	66,4286	68,5714	0	1	1	2
0	0	0	142,857	85,7143	41,4286	1	1	2
0	0	0	0	68,5714	0	0	1	2
0	0	0	8,57143	51,4286	0	0	0	2
0	0	0	188,571	277,143	205,714	1	1	2
0	0	0	60	100	10,7143	1	1	1
0	0	0	188,571	175,714	25,7143	1	1	2
0	0	0	15	42,8571	4,28571	0	0	2
0	0	0	12,8571	60	8,57143	0	1	1
0	0	0	20	51,4286	0	0	0	2
0	0	0	21,4286	25,7143	0	0	0	1
0	0	0	49,2857	145,714	0	0	1	2
0	0	0	214,286	120	60	1	1	2
0	0	0	1,42857	64,2857	0	0	1	2
0	0	0	17,1429	171,429	8,57143	0	1	2
0	1	1	240	60	0	1	1	2

0	0	0	92,1429	110	17,1429	1	1	2
0	0	0	128,571	8,57143	0	1	0	2
0	0	0	145,714	60	34,2857	1	1	2
0	0	0	21,4286	180	0	0	1	2
0	0	0	14,2857	51,4286	0	0	0	1
0	0	0	68,5714	77,1429	0	1	1	2
0	0	0	150	19,2857	25,7143	1	0	3
0	0	0	21,4286	34,2857	0	0	0	2
0	0	0	27,8571	235,714	0	0	1	2
0	0	1	107,143	60	38,5714	1	1	2
0	0	1	184,286	162,857	8,57143	1	1	2
0	1	1	148,571	51,4286	0	1	0	2
0	0	0	50,7143	0	0	0	0	2
0	0	0	113,571	42,8571	0	1	0	2
0	0	0	40	60	0	0	1	2
0	0	0	15,7143	30	0	0	0	2
0	0	0	8,57143	120	0	0	1	1
0	0	0	95,7143	312,857	0	1	1	2
0	0	0	30	8,57143	0	0	0	3
0	0	0	8,57143	27,8571	0	0	0	2
0	0	0	167,143	154,286	0	1	1	2
0	0	0	2,85714	102,857	0	0	1	2
0	0	0	171,429	162,857	25,7143	1	1	2
0	0	0	42,8571	42,8571	17,1429	0	1	2
0	0	0	165,714	34,2857	0	1	0	1
0	0	0	154,286	171,429	77,1429	1	1	2
0	0	0	28,5714	20	0	0	0	1
0	0	0	20	308,571	0	0	1	2
0	0	0	51,4286	25,7143	0	0	0	2
0	0	0	14,2857	25,7143	17,1429	0	0	2
0	0	0	87,4286	351,429	25,7143	1	1	1
0	0	0	30	25,7143	0	0	0	3
0	0	0	81,4286	42,8571	0	1	0	2
0	0	0	25,7143	51,4286	0	0	0	2
0	0	0	128,571	68,5714	0	1	1	2
0	0	0	150	171,429	0	1	1	1
0	0	0	98,5714	60	0	1	1	2
0	0	0	14,2857	98,5714	0	0	1	1
0	0	0	47,1429	222,857	0	0	1	2
0	0	0	30	120	0	0	1	3
0	0	0	0	42,8571	42,8571	0	1	2
0	0	0	171,429	145,714	0	1	1	1
0	0	0	25,7143	77,1429	0	0	1	2
0	0	0	34,2857	21,4286	0	0	0	2
0	0	0	171,429	180	0	1	1	3
0	0	0	8,57143	102,857	0	0	1	2
0	0	1	77,1429	51,4286	0	1	0	2
0	0	0	171,429	180	8,57143	1	1	2

0	0	0	128,571	377,143	0	1	1	2
0	1	1	87,4286	77,1429	0	1	1	2
0	0	0	30	111,429	4,28571	0	1	2
0	0	0	64,2857	37,1429	0	1	0	1
0	0	0	0	214,286	0	0	1	2
0	0	0	0	8,57143	0	0	0	2
0	0	0	42,8571	25,7143	0	0	0	2
0	0	0	197,143	94,2857	0	1	1	1
0	0	0	0	308,571	32,1429	0	1	2
0	0	0	187,143	60	17,1429	1	1	2
0	0	0	188,571	394,286	0	1	1	2
0	0	0	32,1429	0	0	0	0	2
0	0	0	2,85714	40,7143	0	0	0	2
0	0	0	60	120	17,1429	1	1	2
0	0	0	7,14286	25,7143	0	0	0	1
0	0	0	150	68,5714	15	1	1	1
0	0	0	18,5714	145,714	0	0	1	1
0	0	0	8,57143	0	0	0	0	1
0	0	0	0	42,8571	34,2857	0	1	2
0	0	0	80	49,2857	8,57143	1	0	1
0	0	0	65,7143	74,2857	0	1	1	2
0	0	0	248,571	180	0	1	1	2
0	0	0	17,1429	17,1429	8,57143	0	0	1
0	1	1	186,429	51,4286	2,85714	1	0	2
0	0	0	107,143	85,7143	0	1	1	2
0	0	0	102,857	135	0	1	1	2
0	0	0	162,857	188,571	0	1	1	2
0	0	0	12,8571	41,4286	0	0	0	2
0	0	0	24,2857	4,28571	0	0	0	2
0	0	0	34,2857	25,7143	0	0	0	2
0	0	0	8,57143	60	25,7143	0	1	2
0	0	0	8,57143	60	0	0	1	2
0	0	0	65,7143	34,2857	0	1	0	1
0	0	0	42,8571	48,5714	0	0	0	2
0	0	0	0	25,7143	0	0	0	2
0	0	0	0	8,57143	0	0	0	1
0	0	0	120	197,143	0	1	1	2
0	0	0	0	117,143	0	0	1	2
0	0	0	0	8,57143	0	0	0	2
0	0	0	14,2857	25,7143	0	0	0	1
0	0	0	81,4286	25,7143	25,7143	1	0	2
0	0	0	128,571	228,571	42,8571	1	1	2
0	0	0	145,714	32,1429	0	1	0	2
0	0	0	72,8571	111,429	0	1	1	2
0	0	0	17,8571	25,7143	0	0	0	1
0	0	1	138,571	53,5714	17,1429	1	1	1
0	0	0	36,4286	21,4286	51,4286	0	1	2
1	0	1	34,2857	101,429	0	0	1	1

0	0	0	171,429	34,2857	0	1	0	2
0	0	0	34,2857	25,7143	0	0	0	2
0	1	1	127,143	51,4286	0	1	0	1
0	0	0	188,571	125,714	0	1	1	2
0	0	0	17,1429	14,2857	0	0	0	2
0	0	0	14,2857	120	12,8571	0	1	2
0	0	0	55,7143	128,571	0	0	1	3
0	0	0	87,1429	24,2857	0	1	0	2
0	0	0	22,8571	180	0	0	1	2
0	0	0	55,7143	55,7143	12,8571	0	1	2
0	0	0	30	85,7143	0	0	1	2
0	0	0	4,28571	60	0	0	1	2
0	0	0	180	154,286	0	1	1	3
0	0	0	6,42857	128,571	0	0	1	2
0	0	0	22,8571	60	8,57143	0	1	1
0	0	0	53,5714	23,5714	0	0	0	2
0	0	0	8,57143	51,4286	8,57143	0	1	2
0	1	1	91,7143	60	0	1	1	2
0	0	0	7,14286	51,4286	0	0	0	2
0	0	0	214,286	231,429	25,7143	1	1	2
0	0	0	60	0	0	1	0	2
0	0	0	128,571	128,571	0	1	1	2
0	0	0	51,4286	23,5714	0	0	0	1
0	0	0	27,8571	39,2857	0	0	0	2
0	0	0	0	102,857	0	0	1	2
0	0	0	14,2857	51,4286	0	0	0	2
1	0	1	24,2857	31,4286	42,8571	0	1	2
0	0	0	60	248,571	42,8571	1	1	2
0	0	0	155,714	42,8571	2,85714	1	0	2
0	0	0	17,1429	34,2857	0	0	0	1
0	0	0	31,4286	100	0	0	1	2
0	0	0	171,429	171,429	8,57143	1	1	2
0	0	0	8,57143	85,7143	0	0	1	2
0	0	0	111,429	8,57143	34,2857	1	0	2
0	0	0	25,7143	120	0	0	1	2
0	1	1	210	154,286	25,7143	1	1	2
0	0	0	61,4286	51,4286	0	1	0	2
0	0	0	77,1429	66,4286	0	1	1	3
0	0	0	21,4286	72,8571	0	0	1	2
0	0	0	150	8,57143	8,57143	1	0	2
0	0	0	25,7143	51,4286	51,4286	0	1	2
0	1	1	257,143	312,857	81,4286	1	1	3
0	0	0	60	110	0	1	1	2
0	0	0	14,2857	231,429	8,57143	0	1	2
0	0	0	154,286	188,571	25,7143	1	1	2
0	0	0	17,1429	98,5714	0	0	1	2
0	0	0	107,143	154,286	0	1	1	1
0	0	0	0	214,286	17,1429	0	1	2

0	0	0	158,571	210	8,57143	1	1	2
0	1	1	68,5714	60	34,2857	1	1	2
0	0	0	84,2857	77,1429	0	1	1	2
0	1	1	131,429	197,143	30	1	1	2
0	0	1	47,1429	17,1429	42,8571	0	1	2
0	0	0	274,286	111,429	0	1	1	2
0	1	1	78,5714	0	0	1	0	2
0	1	1	255,714	197,143	0	1	1	1
0	0	0	8,57143	0	0	0	0	2
1	0	1	0	34,2857	68,5714	0	1	2
0	0	0	21,4286	92	0	0	1	2
0	0	0	87,8571	17,1429	0	1	0	2
0	0	0	31,4286	6,42857	0	0	0	2
0	0	0	11,4286	0	8,57143	0	0	2
0	0	0	145,714	248,571	0	1	1	2
0	0	0	204,286	0	0	1	0	2
1	0	1	282,857	81,4286	0	1	1	2
0	0	1	197,143	77,1429	0	1	1	2
0	0	0	34,2857	102,857	17,1429	0	1	2
0	0	0	40	64,2857	0	0	1	2
0	0	0	184,286	330	17,1429	1	1	1
0	0	0	137,143	77,1429	0	1	1	1
0	0	0	0	25,7143	0	0	0	2
0	0	0	10	4,28571	0	0	0	3
0	0	0	171,429	278,571	0	1	1	2
0	0	0	135,714	68,5714	0	1	1	2
0	0	0	25,7143	12,8571	8,57143	0	0	1
0	0	0	0	8,57143	17,1429	0	0	2
0	0	0	42,8571	180	0	0	1	1
0	0	0	128,571	17,1429	25,7143	1	0	2
1	0	1	218,571	222,857	120	1	1	1
0	0	0	53,5714	65	0	0	1	2
0	0	1	50	51,4286	0	0	0	3
0	0	0	8,57143	257,143	25,7143	0	1	2
0	0	0	51,4286	22,8571	0	0	0	2
0	0	0	42,8571	85,7143	0	0	1	1
0	1	1	238,571	107,143	8,57143	1	1	1
0	0	1	48,5714	137,143	25,7143	0	1	1
0	0	0	142,857	17,1429	0	1	0	2
0	1	1	248,571	34,2857	0	1	0	1
0	1	1	111,429	34,2857	0	1	0	2
0	0	0	60	192,857	0	1	1	2
0	0	0	248,571	0	0	1	0	2
0	0	0	25,7143	117,143	0	0	1	2
0	0	0	60	111,429	0	1	1	2
0	0	0	0	17,1429	0	0	0	2
0	0	0	0	34,2857	0	0	0	3
0	0	0	188,571	8,57143	0	1	0	2

0	0	0	30	25,7143	0	0	0	2
0	0	0	128,571	154,286	0	1	1	2
0	0	0	158,571	51,4286	0	1	0	2
0	0	0	25,7143	120	0	0	1	1
0	0	0	0	69,2857	0	0	1	2
0	0	0	0	4,28571	0	0	0	2
0	0	0	137,143	34,2857	17,1429	1	0	3
0	0	0	128,571	257,143	154,286	1	1	2
0	0	0	0	51,4286	0	0	0	2
0	0	0	77,1429	154,286	0	1	1	1
0	0	0	18,5714	97,1429	0	0	1	2
0	0	0	17,1429	0	0	0	0	2
0	0	0	71,4286	137,143	0	1	1	2
0	0	0	150	108,571	0	1	1	2
1	0	1	34,2857	210	25,7143	0	1	2
0	0	0	134,286	122,857	54,2857	1	1	2
0	0	0	60	190	0	1	1	2
0	0	0	98,5714	60	0	1	1	1
0	0	0	145,714	162,857	6,42857	1	1	2
0	0	0	148,571	368,571	25,7143	1	1	1
0	0	0	4,28571	42,8571	0	0	0	1
0	0	0	28,5714	107,143	0	0	1	2
0	0	0	42,8571	2,85714	0	0	0	2
0	0	0	248,571	120	0	1	1	1
0	0	0	0	274,286	8,57143	0	1	2
0	0	0	25,7143	248,571	0	0	1	2
0	0	0	139,286	174,286	8,57143	1	1	1
0	0	0	11,4286	17,1429	0	0	0	2
0	0	0	34,2857	25,7143	0	0	0	2
1	0	1	17,1429	274,286	102,857	0	1	2
0	0	0	68,5714	0	17,1429	1	0	2
0	0	0	145,714	51,4286	25,7143	1	1	1
1	0	1	0	115,714	51,4286	0	1	1
0	0	0	25,7143	51,4286	0	0	0	2
0	0	0	47,1429	111,429	0	0	1	2
0	0	0	137,143	55,7143	0	1	0	3
0	0	0	14,2857	42,8571	0	0	0	2
0	0	0	17,1429	214,286	0	0	1	1
0	0	0	147,143	265,714	25,7143	1	1	2
0	0	0	60	8,57143	0	1	0	2
0	1	1	120	8,57143	0	1	0	2
0	0	0	47,1429	25,7143	0	0	0	2
0	1	1	120	300	0	1	1	2
0	0	1	27,1429	85,7143	0	0	1	1
0	0	0	137,143	180	8,57143	1	1	1
0	0	0	30	102,857	0	0	1	1
0	0	0	55,7143	60	0	0	1	2
0	1	1	131,429	72,8571	0	1	1	2

0	0	0	2,14286	94,2857	17,1429	0	1	2
0	0	0	145,714	77,1429	17,1429	1	1	2
0	0	0	240	51,4286	0	1	0	2
0	0	0	28,5714	8,57143	0	0	0	2
0	0	0	85,7143	0	0	1	0	2
0	0	0	111,429	102,857	0	1	1	2
0	0	0	160,714	120	0	1	1	3
0	0	0	4,28571	37,1429	12,8571	0	0	2
0	0	0	128,571	0	0	1	0	2
0	0	0	0	23,5714	0	0	0	2
0	0	0	12,8571	85,7143	105,714	0	1	2
0	0	0	7,14286	51,4286	0	0	0	1
0	0	0	57,1429	18,5714	0	0	0	2
0	1	1	154,286	45,7143	0	1	0	2
0	0	0	137,143	174,286	25,7143	1	1	2
0	0	0	42,8571	102,857	0	0	1	2
0	0	0	197,143	197,143	0	1	1	2
0	1	1	171,429	34,2857	0	1	0	2
0	0	0	49,2857	12,8571	0	0	0	2
0	0	0	38,5714	180	0	0	1	1
0	0	0	60	51,4286	0	1	0	2
0	0	0	20	360	120	0	1	2
0	0	0	50	171,429	0	0	1	2
0	0	0	17,1429	111,429	0	0	1	2
0	0	0	54,2857	25,7143	0	0	0	1
0	0	0	8,57143	154,286	0	0	1	2
0	0	0	180	300	0	1	1	2
0	0	0	14,2857	192,857	0	0	1	2
0	0	0	5,71429	34,2857	0	0	0	2
0	0	0	218,571	218,571	0	1	1	2
0	0	0	244,286	60	0	1	1	2
0	0	0	0	330	0	0	1	2
0	0	0	51,4286	34,2857	0	0	0	2
0	0	0	0	17,1429	25,7143	0	0	2
0	0	0	77,1429	75,7143	0	1	1	2
0	0	0	55,7143	34,2857	0	0	0	2
0	0	0	37,1429	102,857	0	0	1	1
0	0	0	51,4286	94,2857	0	0	1	1
0	0	0	85,7143	171,429	0	1	1	1
0	0	0	57,1429	162,857	0	0	1	2
0	1	1	120	25,7143	0	1	0	3
0	0	0	66,4286	30	0	1	0	2
0	0	0	128,571	351,429	0	1	1	2
0	0	0	0	25,7143	8,57143	0	0	2
0	0	0	47,1429	30	17,1429	0	0	2
0	0	0	111,429	51,4286	0	1	0	2
0	0	0	248,571	222,857	68,5714	1	1	2
0	0	0	64,2857	47,1429	0	1	0	3

0	0	0	164,286	162,857	8,57143	1	1	1
0	0	0	145,714	36,4286	0	1	0	2
0	0	0	0	124,286	0	0	1	3
0	0	0	5,71429	37,1429	0	0	0	1
0	0	0	111,429	68,5714	0	1	1	2
0	0	0	100,714	132,857	25,7143	1	1	1
0	0	0	77,1429	51,4286	0	1	0	1
0	0	0	17,1429	60	64,2857	0	1	2
0	0	0	83,5714	67,1429	0	1	1	2
0	0	0	68,5714	40	0	1	0	1
0	0	0	12,8571	154,286	0	0	1	1
0	0	0	102,857	60	12,8571	1	1	3
0	0	0	162,857	128,571	0	1	1	2
0	0	0	42,8571	38,5714	0	0	0	3
0	0	0	25,7143	4,28571	0	0	0	2
0	0	0	257,143	51,4286	0	1	0	2
0	0	0	102,857	120	25,7143	1	1	1
0	0	0	0	10,7143	0	0	0	2
0	0	0	145,714	180	8,57143	1	1	2
0	0	0	111,429	240	0	1	1	2
0	0	0	128,571	197,143	34,2857	1	1	2
0	0	0	44,2857	60	8,57143	0	1	2
0	0	0	257,143	51,4286	17,1429	1	1	2
0	0	1	30	141,429	42,8571	0	1	2
0	0	0	128,571	145,714	0	1	1	1
0	0	0	65,7143	77,1429	0	1	1	1
0	0	0	90	102,857	0	1	1	1
0	0	0	174,286	154,286	0	1	1	3
0	0	0	0	34,2857	0	0	0	2
0	0	0	4,28571	17,1429	0	0	0	2
0	0	0	51,4286	51,4286	0	0	0	2
0	0	0	52,8571	177,857	21,4286	0	1	2
0	0	0	128,571	188,571	0	1	1	2
0	0	0	134,286	42,8571	0	1	0	2
0	0	0	21,4286	25,7143	0	0	0	2
0	0	0	132,857	17,1429	0	1	0	2
0	0	0	2,85714	0	0	0	0	2
0	0	0	45,7143	102,857	0	0	1	2
0	0	0	150	180	0	1	1	2
0	0	0	34,2857	51,4286	0	0	0	2
0	0	0	0	68,5714	154,286	0	1	1
0	0	0	15	94,2857	0	0	1	2
0	0	0	10	21,4286	0	0	0	1
0	0	0	47,1429	94,2857	25,7143	0	1	2
0	0	0	94,2857	42,8571	0	1	0	2
0	0	0	12,8571	171,429	34,2857	0	1	1
0	0	0	34,2857	51,4286	17,1429	0	1	2
0	0	0	41,4286	177,143	0	0	1	2

0	0	0	94,2857	180	25,7143	1	1	2
0	0	0	38,5714	85,7143	2,85714	0	1	2
0	0	0	134,286	128,571	0	1	1	2
0	0	0	128,571	282,857	154,286	1	1	2
0	0	0	25,7143	34,2857	0	0	0	2
0	0	0	154,286	248,571	25,7143	1	1	2
0	0	0	150	205,714	0	1	1	2
0	0	0	137,143	111,429	0	1	1	2
0	0	0	30	120	0	0	1	2
0	0	0	42,8571	38,5714	0	0	0	2
0	0	0	158,571	184,286	0	1	1	2
0	0	0	138,571	120	0	1	1	3
0	0	0	83,5714	27,1429	0	1	0	2
0	0	0	34,2857	257,143	0	0	1	2
0	0	0	201,429	197,143	0	1	1	1
0	0	0	0	107,143	0	0	1	2
0	0	0	11,4286	154,286	0	0	1	2
0	0	0	128,571	111,429	0	1	1	2
0	0	0	2,85714	265,714	102,857	0	1	2
0	0	0	5,71429	60	0	0	1	2
0	0	0	62,1429	25,7143	0	1	0	1
0	0	0	35,7143	42,8571	0	0	0	2
0	0	0	42,8571	34,2857	0	0	0	2
0	0	0	8,57143	30	85,7143	0	1	2
0	0	0	63,5714	120	0	1	1	2
0	0	0	50	8,57143	4,28571	0	0	2
0	0	0	15,7143	42,8571	77,1429	0	1	1
0	0	0	12,8571	115,714	0	0	1	2
0	0	0	55,7143	34,2857	0	0	0	2
0	1	1	251,429	68,5714	17,1429	1	1	1
0	0	0	142,857	111,429	0	1	1	2
0	0	0	40,7143	98,5714	0	0	1	2
0	0	0	0	51,4286	0	0	0	2
0	0	0	42,8571	51,4286	0	0	0	1
0	0	0	180	274,286	0	1	1	3
0	0	0	34,2857	128,571	8,57143	0	1	2
0	0	1	0	68,5714	0	0	1	3
0	0	0	0	94,2857	0	0	1	2
0	0	0	12,8571	68,5714	51,4286	0	1	2
0	0	0	17,1429	32,1429	0	0	0	2
0	0	0	91,4286	60	0	1	1	3
0	0	0	28,5714	42,8571	17,1429	0	1	2
0	0	0	180	175,714	25,7143	1	1	2
0	0	0	17,1429	102,857	51,4286	0	1	2
0	0	0	115,714	38,5714	0	1	0	2
0	0	0	168,571	171,429	34,2857	1	1	2
0	0	0	141,429	77,1429	0	1	1	3
0	0	0	164,286	240	0	1	1	2

0	0	0	15,7143	30	0	0	0	2
0	0	0	37,1429	25,7143	0	0	0	2
0	1	1	120	47,1429	0	1	0	2
0	0	0	144,286	60	28,5714	1	1	2
0	0	0	28,5714	60	0	0	1	2
0	0	0	8,57143	197,143	0	0	1	2
0	0	0	40	222,857	102,857	0	1	2
0	0	0	88,5714	68,5714	42,8571	1	1	1
0	0	0	81,4286	132,857	0	1	1	3
0	0	0	282,857	231,429	0	1	1	2
0	0	0	25,7143	60	0	0	1	2
0	1	1	248,571	257,143	0	1	1	2
0	0	0	143,571	25,7143	0	1	0	2
0	0	0	175,714	210	0	1	1	2
0	0	1	197,143	85,7143	17,1429	1	1	2
0	0	0	128,571	107,143	0	1	1	2
0	0	0	184,286	158,571	0	1	1	2
0	0	0	30	25,7143	0	0	0	2
0	0	0	188,571	120	4,28571	1	1	2
0	0	0	25,7143	25,7143	0	0	0	2
0	0	0	68,5714	111,429	17,1429	1	1	1
0	0	0	145,714	8,57143	0	1	0	2
0	0	0	0	227,143	0	0	1	1
0	0	0	0	102,857	0	0	1	2
0	0	0	8,57143	221,429	0	0	1	1
0	0	0	171,429	25,7143	45	1	1	2
0	0	0	8,57143	34,2857	0	0	0	2
0	0	0	248,571	42,8571	0	1	0	2
0	0	0	157,143	231,429	0	1	1	1
0	0	0	12,8571	51,4286	8,57143	0	1	2
0	0	0	19,2857	85,7143	85,7143	0	1	3
0	0	0	28,5714	32,1429	5,71429	0	0	1
0	0	0	135,714	248,571	25,7143	1	1	2
0	0	0	17,1429	42,8571	0	0	0	1
0	0	0	30	51,4286	0	0	0	2
0	0	0	85,7143	120	0	1	1	1
0	0	0	334,286	120	30	1	1	2
0	0	0	68,5714	188,571	0	1	1	2
0	0	0	40	85,7143	0	0	1	3
0	0	0	14,2857	25,7143	5,71429	0	0	2
0	0	0	128,571	248,571	0	1	1	2
0	0	0	34,2857	25,7143	0	0	0	2
0	0	0	42,8571	107,143	0	0	1	2
0	0	0	248,571	17,1429	0	1	0	3
0	0	0	52,8571	60	8,57143	0	1	2
0	0	0	12,8571	0	0	0	0	2
0	0	0	10,7143	51,4286	0	0	0	1
0	0	0	42,8571	17,1429	0	0	0	1

0	1	1	60	180	0	1	1	2
0	0	0	42,8571	77,1429	8,57143	0	1	2
0	0	0	14,2857	34,2857	0	0	0	2
0	0	0	77,1429	64,2857	0	1	1	1
0	0	0	25,7143	25,7143	0	0	0	1
0	0	0	7,14286	58,5714	0	0	0	1
0	0	0	21,4286	85,7143	0	0	1	2
0	0	0	107,143	17,1429	0	1	0	2
0	0	0	222,857	51,4286	0	1	0	2
0	0	0	0	137,143	0	0	1	2
0	0	1	30	51,4286	0	0	0	2
0	0	0	2,85714	60	0	0	1	2
0	0	0	32,1429	145,714	0	0	1	3
0	0	0	111,429	17,1429	17,1429	1	0	2
0	0	0	81,4286	145,714	0	1	1	2
0	0	0	57,1429	128,571	0	0	1	1
0	0	0	27,8571	62,8571	0	0	1	1
0	0	0	20	64,2857	0	0	1	2
0	0	0	184,286	188,571	0	1	1	3
0	0	0	6,42857	17,1429	0	0	0	2
0	0	0	8,57143	0	0	0	0	1
0	0	0	128,571	168,571	17,1429	1	1	2
0	0	0	17,1429	37,1429	17,1429	0	0	2
0	0	1	61,4286	48,5714	51,4286	1	1	2
0	1	1	245,714	4,28571	0	1	0	2
0	0	0	154,286	128,571	0	1	1	2
0	0	1	282,857	162,857	17,1429	1	1	2
0	1	1	265,714	102,857	17,1429	1	1	2
0	0	0	50	52,1429	0	0	0	2
0	0	0	192,857	171,429	0	1	1	2
0	0	0	30	68,5714	0	0	1	3
0	0	0	4,28571	94,2857	0	0	1	2
0	0	0	25,7143	222,857	0	0	1	2
0	0	0	162,857	77,1429	0	1	1	2
0	0	0	42,8571	34,2857	0	0	0	3
0	0	0	139,286	94,2857	0	1	1	2
0	0	0	66,4286	154,286	0	1	1	2
0	0	0	11,4286	111,429	0	0	1	2
0	0	0	0	257,143	0	0	1	1
0	0	0	45,7143	120	20	0	1	2
0	0	0	34,2857	261,429	25,7143	0	1	2
0	0	0	7,14286	51,4286	0	0	0	2
0	0	0	154,286	8,57143	0	1	0	2
0	0	0	78,5714	42,8571	0	1	0	1
0	0	0	42,8571	205,714	17,1429	0	1	1
0	0	0	25,7143	131,429	154,286	0	1	2
0	0	0	141,429	19,2857	12,8571	1	0	2
0	0	0	142,857	34,2857	0	1	0	2

0	*0*	*0*	*107,143*	*154,286*	*0*	*1*	*1*	*1*
0	*0*	*0*	*214,286*	*218,571*	*0*	*1*	*1*	*2*
0	*0*	*0*	*8,57143*	*60*	*0*	*0*	*1*	*2*
0	*0*	*1*	*102,857*	*111,429*	*0*	*1*	*1*	*2*
0	*0*	*0*	*138,571*	*154,286*	*0*	*1*	*1*	*2*
0	*0*	*0*	*100,714*	*25,7143*	*0*	*1*	*0*	*1*

var 23	var 24	var 25	var 26	var 27	var 28	var 29	var 30	var 31
6,146	3,288	6,853	0,428	1,066	428,571	0,066	14,2464	5,289
2,043	1,484	2,484	0,428	0	454,286	1	55,944	0,143
7,971	5,641	4,473	5	4,5	522,857	0,143	21,6468	6,789
10,861	5,163	0,198	0,786	0	240	0	30,66	0,143
4,491	1,721	10,132	0,066	1	317,143	4,5	30,002	5,289
8,102	3,156	4,222	0,428	0,066	240	1	30,66	1,578
5,272	7,921	6,218	0	0	325,714	1	11,592	5,289
3,623	5,696	4,132	0,786	4,643	137,143	0	16,5648	5,289
3,623	1,284	1,341	0,189	1	497,143	1	27,972	0,572
5,563	0,932	1,209	0,428	1	145,714	0,789	12,096	0,855
2,406	1,141	4,198	0,066	4,5	565,714	1	132,916	0,143
7,878	2,51	1,789	0,428	0	325,714	4,5	0	0,143
5,003	1,842	1,13	1	0	300	4,5	15,5232	5,289
4,269	3,433	8,198	0,066	4,5	480	1	58,31	6,143
3,623	1,416	0,286	0,066	0	334,286	4,5	58,282	6,143
6,803	5,432	6,286	0,428	1	214,286	0	0	4,643
8,297	0,275	1,132	0,428	1	317,143	0	22,414	4,643
6,864	2,785	7,073	0,066	0	274,286	4,5	373,24	0,789
4,491	1,196	8,141	0,428	4,5	180	0,789	17,6652	0,143
11,092	1,35	1,264	0,066	0	222,857	0	2,1504	0,789
2,928	1,143	0,066	0,428	0	240	0	0	0,066
5,299	2,787	0,143	0,786	0	360	0	0	0,789
3,5	1,987	3,143	0,123	0	317,143	1	18,102	0,143
8,22	3,578	7,578	0,428	0	162,857	1	38,7016	0,066
4,363	1,075	1,352	0,123	1,066	214,286	0,143	23,31	0,286
6,946	2,407	3,71	2,5	0	360	0,143	116,62	1,789
5,506	2,939	2,864	2,5	0	300	1	39,5892	6,789
5,221	1,427	0,352	0,123	1	360	4,5	14,784	4,643
6,084	3,945	3,578	0,428	0,495	214,286	4,5	18,396	5,289
5,141	1,919	1,132	0	1,143	197,143	0	0	1,143
4,92	3,071	6,218	0,428	0	360	0	0	0,789
6,434	0,77	14,51	1,572	1	394,286	0	280	4,5
3,368	1,721	3,275	0,551	1,066	214,286	1	26,4936	1,218
3,427	1,864	0	0,123	0	248,571	0	279,86	4,643
4,574	1,644	1,286	0,428	0	180	1	28,7196	0,132
8,343	5,641	3,787	1	0	240	1	7,4592	0,789
9,927	1,908	5,132	0	1	137,143	0,066	1197,553	1,789
3,906	0,921	2,075	0,246	1	145,714	4,5	6,1824	0,932
4,418	0,143	0,789	0,123	4,5	137,143	0	0	5,289
7,85	1,284	7,209	0	0	60	1	150,01	1,789
3,365	2,576	0,143	0,123	0,066	282,857	4,5	57,3272	4,643
7,001	2,708	5,13	0,123	0	180	4,5	23,324	1,143
6,713	3,005	6,132	0,494	0,066	197,143	4,5	46,62	0,789
6,146	1,273	3,207	0,428	1	257,143	1	55,1502	1,066
7,34	2,139	5,352	0,786	0	317,143	1	46,2392	5,289
10,231	1,075	1,998	0	0,789	180	1	0	1,789
5,424	5,347	1,644	1	6	360	1	258,4596	6,066

6,212	3,365	3,842	0,428	4,566	420	0,066	37,4052	4,643
4,86	1,13	2,921	0,428	0	300	0	16,926	4,643
4,693	0,066	0,275	0	0	420	0	42,322	0,143
1,9	1,721	3,007	0	0	240	0	10,0926	0,789
5,647	1,93	3,644	0	0,429	120	4,5	144,606	1,143
5,517	3,578	1,998	0,123	0	411,429	0	0	0,143
6,157	0,484	4	0,428	0,789	31,4286	0,789	24,2424	0
3,272	2,205	10,216	0,123	4,5	394,286	1	21,714	0,789
5,372	2,139	3,627	0,123	0	240	1	0	5,289
5,728	6,43	6,789	1	0,789	214,286	1	93,296	5,289
5,199	1,493	4,275	1	0	300	1	8,064	0,143
3,574	1,721	0,209	0,786	0,066	342,857	0	8,6688	1,789
6,436	1,141	3,218	0,066	4,5	300	0,789	39,634	5,289
4,204	1,853	3,075	0,786	1	180	1	47,9696	0,066
1,603	1,075	3,264	0,066	0	274,286	4,5	22,5204	6,066
9,426	1,996	2,218	0	4,5	274,286	4,5	14,5656	4,643
6,218	2,073	1,141	0	0,789	300	0	13,9944	0,143
4,41	6,353	3,576	0,428	0	222,857	0	89,992	5,289
3,415	2,007	4,073	0,066	0	368,571	0	48,398	6,143
3,849	1,075	5,075	0,123	0,789	85,7143	4,5	16,2204	1,578
5,849	1,998	2,075	0,123	1	240	1	74,8832	0,286
6,069	2,642	1,066	2	0,143	120	0	157,9844	0,789
5,364	2,207	1,71	0,428	4,5	240	0	13,0704	0,143
3,643	2,71	3,341	0	0	180	4,5	3,066	4,643
4,28	2,642	0,561	0,428	4,5	377,143	1	150,01	0,143
4,074	1,787	5,209	0,123	0	77,1429	4,5	0	1,143
4,781	2,631	3,284	0,428	0	317,143	1	57,9782	4,643
5,658	2,785	6,361	0,786	0	274,286	4,5	3,4944	5,289
3,148	2,007	3,932	0,786	0,429	240	1	19,1142	5,5
6,928	7,065	0,275	0,428	0,143	531,429	1	16,2204	0,143
7,554	2,787	2,066	0,428	0	300	0,789	35,322	4,643
3,194	0,789	0,275	0,123	1	171,429	0	19,5216	4,566
6,572	2,35	5,073	0,123	0,932	308,571	0,789	48,958	5,289
3,181	1,996	5,341	0,428	0	428,571	1	50,2628	6,789
8,206	3,431	2,429	0,428	0	394,286	1	1799,84	5,289
5,004	2,787	0,132	0,786	4,5	257,143	0	304,682	4,643
3,137	1,644	0,209	0,066	0,789	317,143	4,5	0	0,143
5,628	2,13	4,864	0,428	0	257,143	4,5	120,4924	4,643
6,287	2,996	5,998	0,428	4,5	265,714	0	270,2392	5,289
3,194	2,209	4,341	0,123	0	291,429	1	263,242	6,143
4,77	1,207	2,132	0,189	0,066	420	1	180,012	0,932
10,569	5,707	1,495	1	0	274,286	1	36,204	6,143
4,935	2,139	6,132	0,428	0,572	308,571	0,143	5,376	1,143
3,348	5,138	3,075	1	0	257,143	0	21,462	6,143
13,926	3,15	0,066	0,428	0,789	600	0	3,066	4,566
2,109	1,35	3,132	0,066	4,5	600	0,789	314,748	6,429
9,723	1,715	3,286	0,428	0,066	394,286	0,789	0	1,789
2,359	3,945	1,132	0	4,5	300	1	294,2464	4,643

8,268	0,855	0	0,428	1	334,286	0,066	32,634	0,143
3,555	1,996	6,776	0,066	0	420	0	8,064	6,143
8,66	2,57	1,561	0	0	180	1	13,44	1,789
7,95	1,57	3,576	0,428	4,566	180	0,789	38,066	0,286
5,067	1,493	2,653	0,856	4,5	180	4,5	37,2176	0,066
6,482	1,427	2,209	0,123	1	342,857	0,066	13,44	6,143
3,269	1,864	1,361	0,428	0	385,714	4,5	0	1,578
14,005	2,139	0,066	0	0,789	137,143	0	91,322	0,143
5,575	0,264	0,209	0,786	1	137,143	0	81,9672	6,066
4,915	1,919	0,341	0,428	0	180	4,5	2,3394	0,789
2,972	2,653	5,218	0,428	4,5	197,143	1	0,8064	5,289
2,26	1,196	16,864	0,428	4,643	360	4,5	170,66	4,643
4,643	2,007	0,209	0,786	0	102,857	0	79,4472	1,789
3,898	6,144	6,715	0,786	0	180	1	62,5016	0,143
5,418	1,721	2,998	0,123	1	120	1	19,5132	0,143
2,983	5,553	7,919	0,123	0	394,286	0	12,726	4,643
3,915	1,93	0,198	0	1	120	1	46,62	0,143
8,137	5,498	1,064	0,428	0	300	0,066	15,9222	5,289
5,003	0,858	3,218	0,856	0	137,143	1	227,8514	6,066
2,898	2,064	7,787	1,572	6	402,857	0	41,7816	0,932
3,063	1,721	0,209	0,428	0,066	282,857	4,5	19,9122	1,218
5,403	7,221	1,143	2,5	0	600	1	36,1872	4,643
7,244	2,853	1,789	0,428	1	162,857	0	4,662	1,789
2,843	1,93	2,932	0,428	0,429	300	1	43,442	1,218
8,077	3,132	0,198	1,123	0	480	4,5	17,2564	0,066
2,12	1,776	0,132	0,066	0	240	0,789	0	0,143
6,724	2,71	5,644	0,428	6	360	4,5	15,001	0,789
4,355	6,43	5,864	1	0	274,286	0	74,592	4,643
3,772	1,053	12,132	0	4,5	240	0,789	90,664	0,789
8,129	1,776	3,341	0,428	1	480	1	10,2144	0,143
7,209	3,288	7,776	0,428	4,5	205,714	1	114,7468	0,789
6,218	2,576	6,119	0,428	0,143	240	0,789	18,6592	5,289
4,849	7,221	21,075	1	0	445,714	4,5	55,944	4,5
8,257	6,144	1,064	1	0	325,714	0	28,9632	0,209
3,645	1,075	1,721	0,428	0,789	197,143	4,5	45,7212	1,578
6,944	1,196	1,064	0,428	0,066	265,714	4,5	245,6412	0,789
5,418	0,484	1,209	0,123	0	531,429	0,143	104,7326	0,066
4,99	6,21	11,216	0,786	4,643	197,143	0,789	27,972	0,789
4,358	1,273	0,198	0,428	0,143	325,714	0,789	75,7792	0,143
4,286	2,007	0,209	0,428	0,855	180	0	0	0,789
5,368	1,853	2,418	0,428	0	342,857	1	93,296	0,932
9,523	1,855	1,286	0,428	1	0	4,5	39,6788	1,578
3,478	2,576	0,198	0,066	0	420	0,066	43,442	0,066
5,843	0,638	0	0,786	0	325,714	1	0	0,066
4,718	2,436	2,066	1	0	222,857	1	50,1844	0,066
6,438	1,908	0,33	0,428	0	240	0	37,296	5,289
2,285	1,864	2,286	1	1	325,714	1	150,01	2
2,502	2,433	8,209	0,428	0	325,714	0	42,924	1,578

9,866	3,93	8,576	0,786	0,789	291,429	0	279,888	1,143
5,652	1,352	3,286	0,246	0,143	300	1	37,1616	0,143
8,134	5,498	0,341	0,428	0,789	300	0,789	73,7884	0,789
3,917	1,644	0,932	0,066	0	265,714	0	17,2536	0,286
5,355	1,853	5,638	0,428	4,5	325,714	0	0	6,789
9,149	1,787	2,132	0,428	0	68,5714	1	11,7264	1,143
6,149	2,576	2,987	0,428	0	180	1	191,674	4,643
3,15	1,93	0,572	0,428	4,5	257,143	1	134,596	4,643
6,943	1,218	1,341	0,123	4,5	102,857	1	2,7972	4,643
2,966	1,275	1,066	0,428	4,5	240	4,5	145,376	4,643
4,262	2,275	1,361	0,123	1	334,286	1	53,3792	1,143
4,849	1,842	0,132	0,428	0,066	420	0,789	64,9712	0,066
2,623	2,785	2,638	0,428	0,066	180	1	40,754	1,578
6,796	5,641	7,064	0,786	1,066	445,714	0	189,4144	4,643
4,561	1,864	4,71	0,428	4,5	394,286	4,5	30,7524	1,578
2,249	1,262	8,007	0,428	1,143	222,857	0,143	30,002	5,289
6,524	1,218	2,789	0,786	0	137,143	0	0	0,789
7,871	3,93	11,93	0,428	0	231,429	4,5	79,4192	5,289
5,63	0,209	1,921	0,428	0	120	0	18,648	4,643
5,985	2,132	4,789	0,428	1	51,4286	0	18,0012	1,143
2,101	1,647	5,218	0,066	0	514,286	1	75,467	6,143
8,274	1,932	0,066	0,246	0	360	1	20,8992	0,143
5,355	1,13	11,576	0,786	0	360	1	35,3304	0,066
6,51	2,653	3,864	0,428	0,066	240	0	5,376	1,789
4,506	2,576	12,352	0,428	0,066	77,1429	0	116,62	5,289
3,335	0,998	7,855	0,428	4,566	257,143	1	101,5504	1,578
13,002	2,719	2,932	0,852	4,5	162,857	1	29,9964	0,572
3,558	1,196	0,352	0,428	4,5	162,857	0	36,792	0,789
2,788	2,499	2,932	0,428	0,789	437,143	1	57,9852	5,289
5,435	2,433	4,218	0,428	0	257,143	0	16,7832	4,643
6,117	2,776	0,066	2,5	0,143	120	0	56,882	1,789
7,003	2,919	2,578	1	1	0	0,429	0	1,143
12,713	0,352	4,064	0,786	1,066	120	1	30,002	0,066
4,564	2,719	1,644	0,428	0,286	300	0,429	86,3254	0,858
5,138	2,436	5,561	0,123	0	385,714	4,5	30,9232	0,932
2,76	2,653	8,286	1,214	0,066	325,714	4,5	56,882	0,143
6,155	1,218	0,495	0,123	0	300	0	9,324	0
4,988	2,132	1,209	0,123	4,5	180	1	26,88	4,643
5,861	1,207	1,075	0,428	1	162,857	0,066	3,4944	5,289
3,206	2,862	8,076	1	4,643	300	4,5	0	1,578
3,995	2,073	1,284	1,28	0,066	240	0,143	25,6536	0
2,992	1,842	4,576	0,428	0,066	240	0,143	11,662	5,289
3,06	1,262	12,198	0,428	0	317,143	1	145,95	5,289
4,927	3,071	13,493	0,428	0,066	428,571	4,5	164,6232	0,143
7,15	1,853	1,218	0,123	1	240	4,5	61,0792	0,143
4,137	1,209	3,286	0,856	0	102,857	1	34,5296	0,143
4,487	2,062	8,207	0,909	1	325,714	4,5	42,3864	1,789
6,005	8,51	2,286	0,786	0,143	120	0,789	0	1,143

2,7	1,13	0,198	0,123	0	514,286	1	21,714	0,066
6,095	6,43	5,921	0	1	445,714	1	140	6,143
4,983	0,704	22,13	1,214	4,5	411,429	0	30,002	4,643
6,876	1,284	1,361	0,428	0,066	334,286	4,5	30,66	1,789
2,994	1,196	0,407	0,123	1,789	120	4,5	78,652	0,143
7,297	2,576	3,864	0	4,5	154,286	1	231,9464	0,143
4,058	0,77	1,864	0,428	0,789	420	1	6,048	5,289
11,351	2,642	2,275	0,786	0	120	1	35,378	0,066
12,661	1,427	0,143	0	1	300	0	7,56	6,143
5,425	1,484	7,064	0,428	0,855	162,857	1	0	0,143
9,073	2,275	3,066	0	1	120	0	18,648	1,789
7,853	3,998	3,075	0,428	0	317,143	0,789	13,104	1,789
5,639	2,076	4,998	0,428	1,066	385,714	0	177,9344	0,429
7,071	2,15	13,864	0,786	0	394,286	1	1,344	4,643
2,614	1,864	3,789	0,428	4,5	240	0	51,282	5,289
6,4	6,852	1,715	2,5	0	317,143	1	22,414	6,143
3,544	6,21	0,286	0,428	0,855	411,429	1	54,3032	1,143
5,355	1,93	6,495	0,428	1	197,143	1	109,4492	0,143
4,355	2,493	13,207	0,786	0	377,143	1	60,662	0,066
4,3	1,504	2,429	0,428	0	197,143	1	37,296	1,143
3,186	2,141	1,064	0,428	4,5	240	1	53,613	0,143
5,287	2,205	4,218	0,786	0	154,286	4,5	47,586	6,143
8,991	3,365	9,361	0,786	0,066	360	0	238,3416	4,643
4,355	1,196	5,075	0,066	0,066	437,143	1	117,0988	0,066
6,502	1,998	0,286	0,551	0	240	1	4,662	0,932
1,779	1,493	5,143	0	0,066	300	4,5	46,13	0,066
2,939	1,001	4,852	0,428	0	197,143	4,5	114,52	4,643
5,515	0,341	1,932	1	0,143	60	1	9,324	0,143
5,207	0,484	0,198	0,428	0	120	1	263,242	0,789
4,561	2,062	0,715	1	1,066	300	1	0	0,932
5,292	2,436	1,209	0,428	0	240	0,143	42,266	0,143
3,549	1,713	3,141	0,786	4,5	248,571	0,066	37,8028	0,789
6,141	2,216	5,653	0,428	0	111,429	1	20,8992	4,643
6,504	2,433	0,561	0,551	1	300	1	38,9872	0,789
4,003	7,01	5,066	0,428	1	257,143	4,5	46,62	1,789
6,227	2,642	4,341	0,428	0,066	137,143	1	111,2972	0,932
4,643	3,156	5,433	0,428	0,066	385,714	1	31,7156	0,789
4,645	1,493	7,864	0	0,066	120	0	39,634	1,143
2,901	0,561	4,998	0,428	1,143	394,286	1	0	4,643
5,432	1,996	4,71	0,428	4,5	240	1	11,662	4,643
3,625	2,007	1,721	1,214	0	300	1	205,2512	1,578
4,487	2,576	4,721	0,428	4,5	265,714	1	43,442	0,789
3,915	1,71	7,721	0,428	1	300	1	503,9552	0,789
4,781	1,064	1,141	0,856	0	240	6	20,4876	0,932
4,291	2,93	4,209	0,428	4,643	531,429	4,5	0	6,429
6,572	3,367	0,143	0,428	1	317,143	0,789	36,6492	0
15,006	1,644	2,578	0,066	0	342,857	1	21,7	0,932
5,06	3,855	0,132	2	0,143	291,429	0,789	16,7832	0,143

3,064	0,998	12,066	0,428	0	514,286	0	0	4,566
8,219	6,709	7,57	0,428	0	137,143	4,5	0	5,289
7,227	2,51	5,15	0,428	0,855	300	1	454,0144	1,578
4,775	2,785	2,007	0,428	0	565,714	0,066	300,02	5,289
3,629	6,419	6,987	0,786	4,566	300	0	5,754	6
4,117	2,273	13,132	0,123	1	102,857	4,5	33,726	4,643
3,412	2,207	3,638	0,066	0,066	394,286	1	25,102	1,578
2,824	6,21	4,787	0,786	0,572	360	0	43,442	4,643
3,854	6,144	7,209	0,786	0	317,143	0,066	27,972	5,289
9,708	1,919	1,218	0,428	0,789	94,2857	1	42,322	0,143
1,342	5,918	0,987	0,428	4,566	360	0	93,296	0,789
6,503	2,642	1,286	0,428	0,789	240	0	2,688	1,789
6,563	1,493	0,198	0,123	0,789	180	4,5	27,972	4,643
4,27	7,21	0,932	0	0	300	0	0	5,289
4,157	0,352	0,275	0,786	0	180	0	0	0,143
9,217	1,13	0,921	0,123	0	385,714	4,5	37,296	6,143
6,713	1,218	2,932	0,428	0	214,286	1	6,7452	0,143
6,869	2,35	4,998	0,198	4,643	171,429	1	44,646	4,643
11,813	3,945	2,075	0,428	0	240	0	69,972	1,578
6,862	1,207	1,987	0	1	240	0	48,398	1,789
9,229	1,284	2,77	0	0,789	360	4,5	3,066	5,289
3,184	2,196	0,921	0,123	6	300	1	79,058	0,572
4,986	1,064	0,132	0,428	0	154,286	1	29,19	6,789
3,06	0,987	0,352	2,5	4,5	600	1	8,3916	0,789
7,667	2,341	0,264	0,428	4,5	240	1	46,648	1,789
8,472	3,365	0,789	0,428	1	137,143	0	81,606	0,143
5,128	4,698	4,921	0,551	0	600	4,5	0,756	0,143
3,926	2,15	5,007	0	0	514,286	1	0	4,643
11,703	2,719	4,284	0,428	4,5	188,571	4,5	108,388	6,789
7,284	2,785	7,987	0,428	0,855	197,143	1	51,2512	4,566
8,363	0,418	2,484	0,428	0	325,714	4,5	102,8342	0,932
1,812	2,293	8,495	0,428	4,5	351,429	4,5	150,01	6,789
4,709	0,561	2,921	0,856	1	120	0,789	123,9784	0,143
4,05	1,647	1,644	0	0,429	222,857	0,789	17,2536	0,143
7,442	1,864	9,284	0,428	0,066	240	1	22,5666	1,143
5,423	3,354	6,638	0,428	0	120	1	15,358	1,143
2,549	2,416	0,132	0,786	0,143	300	1	18,648	0,572
4,204	2,644	1,207	0	0	308,571	0,789	110,8772	4,566
0,957	0,638	3,064	0,132	5,5	325,714	6	26,88	5,289
4,265	1,275	2,132	0,123	0	240	0	24,528	6,789
6,66	4,365	1,721	0,786	4,5	124,286	0,143	420,2212	1,789
9,709	6,287	5,721	0,786	1	240	1	168,0112	0,143
7,149	2,425	6,352	0,428	0	257,143	0	13,986	1,789
4,784	0,847	1,495	0,428	0,789	685,714	0,143	38,542	5,289
7,934	1,996	1,075	0,428	0	257,143	0,143	157,4132	0,066
3,28	1,427	0,407	0	1	137,143	4,5	47,8324	0
10,499	0	0,789	0	0,789	30	1	9,324	0,789
6,138	2,719	1,053	0,428	4,5	197,143	0	300,02	0,789

3,966	1,856	2,264	0,246	0	180	0,143	58,7804	6,066
7,194	4,995	8,698	1	1,066	120	1	85,3132	5,289
6,715	1,996	1,141	0,856	0,429	205,714	1	194,2556	0,066
1,526	1,493	1,787	0,428	4,5	197,143	0	21,0336	0,789
3,188	1,286	2,932	0,428	0	120	0,429	4,032	1,066
10,285	1,921	8,075	1	1	120	4,5	46,3232	1,066
7,637	1,064	7,644	0,428	0,789	300	4,5	30,66	4,643
5,992	2,776	0,418	0,428	0	240	1	0	4,566
5,078	1,273	5,996	0,428	0,572	197,143	0,789	146,888	1,143
7,93	1,427	2,789	0,786	0,429	360	4,5	0	1,143
4,348	0,275	1,209	0,123	1	120	0	12,264	0,143
5,212	1,919	4,787	0,123	1	171,429	4,5	13,986	4,643
5,357	2,787	2,361	0,123	0	300	4,5	24,528	4,643
3,995	2,631	3,71	1	0	137,143	1	70,4732	1,789
5,707	2,282	0,987	0,428	0	240	0	56,882	4,643
7,015	3,431	1,998	0,428	4,5	120	0	48,398	5,289
1,603	3,585	1,71	0,123	0	231,429	0	19,992	0,143
4,3	1,864	4,075	0,428	0	394,286	1	95,704	1,143
14,431	1,853	1,855	0,428	0	240	1	0	0,789
5,498	3,354	1,341	0,428	0,066	300	0,066	36,19	0,932
7,573	3,075	4,932	0,428	1	180	4,5	172,4996	5,289
5,632	1,196	0,55	0	0	120	0,143	98,7644	0,932
8,2	1,35	2,207	0,428	4,5	240	0	7,5264	1,578
11,471	1,996	7,075	0,428	4,5	300	0	20,79	6,143
3,562	1,996	0,932	0,123	0,143	180	0	416,0184	0,143
3,274	0,704	4,275	0	1	222,857	1	43,442	4,643
5,511	2,708	6,198	0	0	480	0,066	14,2464	0,143
5,083	3,585	3,644	0,428	1	188,571	1	23,058	0,572
4,336	0,341	0,198	0,428	1,066	591,429	6	43,1872	0,855
4,401	1,339	0,341	0,856	4,5	274,286	0,066	36,3132	0,855
3,06	2,719	0,495	0,123	0,429	565,714	0	37,296	1,578
5,427	0,704	6,495	1	0	162,857	0,789	480,032	0,143
4,498	2,073	4,352	0,428	0,143	360	0	71,421	6,789
6,095	2,796	4,286	0,428	0,066	162,857	4,5	170,66	4,643
4,065	0,352	1,787	0,428	0	582,857	4,5	17,2536	4,643
14,005	1,427	18,852	0,428	0	480	0,789	60,004	6,789
9,072	2,576	11,433	0,428	0,143	120	1	9,324	5,289
6,953	3,365	2,361	0,428	0	257,143	1	27,972	0,143
7,212	2,427	16,01	0,786	4,5	222,857	4,5	38,542	5,289
4,359	4,764	4,143	0,428	4,5	180	0	0	0,789
6,434	3,565	5,075	0,428	4,5	360	1	102,256	4,643
5,055	0,418	6,841	0,428	0,858	55,7143	4,5	23,31	0
2,065	1,207	3,998	0,428	0,429	137,143	0	24,528	5,289
3,194	2,722	0,921	0	0	197,143	1	360,024	0,143
4,784	3,288	5,35	0,428	1	257,143	0	10,794	4,643
9,361	7,639	9,504	0	1,429	197,143	1	33,768	6,789
5,28	2,51	2,341	0,428	4,5	120	1	67,298	1,789
4,704	3,365	4,132	0,786	0,143	300	1	30,7692	0,143

4,566	1,845	0,998	0,428	1	300	4,5	30,002	0,143
6,208	1,636	6,064	0,428	0	282,857	1	38,9872	5,289
3,706	2,216	0,341	0,066	4,5	360	0	0	6,143
6,935	1,141	7,855	1,123	4,5	77,1429	1	5,376	1,789
2,35	1,776	4	0,428	0,066	480	1	163,268	1,143
3,709	2,062	4,132	0	0,132	300	1	33,726	5,289
2,359	1,196	5,35	0,123	0,789	317,143	1	30,66	5,289
5,574	1,141	0,341	0,123	0,143	257,143	0	60,004	1,143
5,858	1,853	2,721	1	4,5	120	0,789	78,6632	1,578
9,088	1,864	3	0,428	0,143	231,429	1	460,012	1,578
9,057	2,51	3,921	1	0	282,857	0	36,792	0,789
4,924	1,218	0,418	0	0,429	188,571	4,5	139,944	0,286
3,48	2,062	9,209	0	5,289	360	4,5	17,1696	4,643
8,437	1,647	1,998	0,428	0	120	4,5	104,594	5,289
4,649	1,143	0	0,066	4,5	240	4,5	0	0,789
3,492	4,011	3,51	0	1,429	180	0,429	54,0204	5,289
3,966	5,281	0,275	0,066	4,5	394,286	1	25,0992	5,289
6,66	5,784	1,064	0	1	282,857	1	5,376	0,209
6,295	1,996	8,367	0,786	0	162,857	4,5	35,378	0,143
4,484	2,719	1,987	0,132	0	420	0	61,9892	6,789
7,73	1,13	0,429	0,123	1,066	437,143	1	93,9764	1,789
4,841	2,919	8,578	0,786	1	197,143	1	68,0876	5,289
8,037	1,427	5,421	0,428	4,5	171,429	0,066	10,038	0,143
2,891	1,196	3,396	0,428	0,066	685,714	1	3,066	0,932
3,973	1,427	1,218	0,856	0	111,429	0	61,32	6,143
7,774	2,862	6,064	0,786	0	300	4,5	365,848	4,643
5,997	2,139	4,15	0	4,5	214,286	0	0	1,143
5,719	2,433	1,075	0,428	4,566	154,286	0,789	311,2452	0,932
5,718	1,93	7,207	0,856	1,066	180	1	16,926	4,643
1,605	1,141	0,352	0,066	0	171,429	1	0	0,066
3,928	1,275	1,427	0,428	1	68,5714	0	18,1272	4,566
3,759	2,064	2,864	1	6	540	4,5	80,0044	1,066
5,7	2,576	0,066	0,428	0	300	1	48,0032	4,643
3,904	1,13	3,067	0,123	4,566	394,286	4,5	23,8644	6,143
5,585	0,932	0	0,428	0	111,429	0	0	0,789
5,853	1,71	1,075	0,428	4,5	385,714	1	18,102	1
2,911	1,35	5,578	0,123	1	180	4,5	178,6652	5,289
4,052	3,433	1,284	0,066	4,643	360	4,5	0	4,5
3,225	1,075	1	0,551	4,5	368,571	4,5	84,3052	1,578
2,9	2,862	6,787	0,428	4,5	171,429	4,5	0	5,289
3,412	1,71	0,275	0,428	1	180	1	66,794	1,143
4,918	2,142	4,484	0,786	0	214,286	1	48,3112	5,289
10,232	6,364	8,432	0,428	0	385,714	4,5	0	4,643
4,555	6,496	6,493	0,551	0,789	197,143	4,5	69,9384	6,789
4,623	3,148	4,341	0	0	291,429	4,5	448,5516	0,855
5,83	2,139	8,073	0,428	5,289	50	0,143	39,634	4,643
5,794	3,576	2,855	0	4,566	214,286	1	256,62	4,643
5,001	2,284	3,998	0	0,429	282,857	0	8,8704	5,289

2,843	0,998	3,141	0,428	4,5	360	0	32,004	5,289
3,546	0,987	0,066	1	0	522,857	0	0	0,066
6,427	3,717	3,427	0,428	1,789	197,143	4,5	187,544	1,218
5,854	2,062	2,13	0,428	0,066	300	4,5	282,688	0,932
2,812	1,721	5,064	0,123	0	497,143	0,789	42,322	0,143
5,509	9,275	3,919	0,551	0,789	180	1	30,8028	0,789
7,864	2,15	10,289	0,428	0,143	411,429	4,5	2,7972	0,066
4,052	6,353	2,719	1	0	360	1	59,5336	4,643
1,702	2,367	9,264	0,551	0,143	102,857	4,5	50,9922	5,289
4,932	2,141	4,429	0,428	0	180	1	13,44	6,143
3,719	1,787	5,066	0,123	0	162,857	4,5	22,764	0,789
6,994	7,142	1,789	0,428	0,066	240	0	0	5,289
9,149	1,141	3,064	0	0	137,143	1	24,388	4,643
3,955	0,352	0,638	2,5	0,789	394,286	1	25,872	4,566
6,345	1,644	0,429	0,066	4,5	197,143	0	0	4,643
2,906	0,132	2,998	0,123	0	300	1	0	5,289
4,649	1,93	1,578	0	0	360	0,143	4,8384	4,643
3,997	1,644	0,286	0,428	0	214,286	1	0	1,143
5,229	1,35	0,066	0,428	0	300	4,5	10,752	0,789
5,372	3,156	6,789	0,428	0,789	394,286	0,789	60,662	0,932
4,289	2,216	10,264	0,428	4,5	240	0	5,376	1,789
7,599	3,945	3,007	0,428	0	240	1	23,31	5,289
13,298	0,638	1,578	0,428	1	248,571	1	150,01	1,143
5,772	2,075	0,143	0,428	4,5	180	0	31,9872	0,143
7,135	1,71	3,945	0	1	248,571	1	24,668	0,143
5,638	2,216	1,075	0,428	0	360	0	13,9944	5,289
7,44	0,998	1,361	1	4,5	180	0	84	0,066
6,363	0,209	4,141	0,428	4,5	205,714	0	0,8064	5,289
5,355	1,985	3,284	0,786	0,789	180	0,066	176,624	5,289
4,149	2,282	3,561	0	4,5	240	4,5	47,3928	1,789
3,926	2,499	3,13	0	0,066	77,1429	4,5	312,5808	0,286
6,223	1,864	2,644	0,428	1	265,714	0	47,6644	5,289
4,654	6,221	6,209	0,428	0	257,143	4,5	23,31	4,643
2,491	1,721	4,367	0,856	4,5	240	0,143	45,1332	5,289
1,955	0,704	11,79	0,066	4,5	394,286	6	0	0,789
4,32	4,852	2,064	0,551	0	162,857	0,066	4,8384	6,143
2,983	2,062	0	0	0,789	120	0	55,944	0,932
5,779	3,787	4,709	2	4,5	377,143	0	141,2992	0,143
5,442	0,209	9,789	0	0	360	0	194,6742	5,289
15,503	0,132	0,921	0,428	1	180	0	31,6652	0,143
14,051	3,299	3,499	0,428	6	180	0	91,8316	4,643
7,914	0,473	3,998	0,123	0	231,429	4,5	18,6592	4,566
4,701	2,073	2,855	0	0,143	197,143	4,5	36,19	0,143
3,56	2,642	7,007	0	0	360	1	136,612	5,289
6,161	1,787	0,998	0,786	4,5	360	6	67,4436	4,566
3,423	0,638	4,143	0,428	4,5	480	1	61,32	0,789
4,129	5,707	1,721	0,428	0	222,857	1	154,6132	0,789
6,434	1,93	1,141	0	1,789	214,286	1	170,66	5,289

8,932	*2,653*	*1,998*	*0,428*	*0*	*300*	*1*	47,964	*0,143*
6,576	*1,284*	*1,578*	*1*	*4,5*	*197,143*	*1*	330,008	*0,143*
7,95	*1,13*	*4,561*	*0,856*	*0*	*154,286*	*0*	5,376	*0,143*
1,559	*1,93*	*6,132*	*0,066*	*0*	*565,714*	*4,5*	0	*4,566*
3,766	*3,642*	*5,35*	*0,786*	*1,066*	*394,286*	*0*	64,4924	*1,578*
7,15	*2,576*	*1,721*	*0,786*	*1*	*205,714*	*0*	0	*4,643*
1,592	*0,341*	*0,275*	*0,123*	*0*	*265,714*	*6*	68,1044	*0,066*
3,049	*0,998*	*6,921*	*0,428*	*0*	*291,429*	*1*	10,038	*1,578*
2,392	*1,853*	*8,352*	*0,428*	*4,5*	*180*	*4,5*	79,058	*0,143*
3,918	*2,427*	*8,064*	*0,428*	*4,5*	*180*	*1*	159,3004	*0,143*
4,407	*0,561*	*2,207*	*0,428*	*0*	*240*	*0,143*	97,8796	*6,789*
8,741	*2,719*	*6,367*	*0,428*	*4,5*	*240*	*4,5*	46,62	*5,289*
6,15	*0,275*	*2,209*	*0,123*	*1*	*171,429*	*0*	61,32	*6,066*
2,128	*2,007*	*7,075*	*0,428*	*0*	*137,143*	*4,5*	60,662	*5,289*
3,458	*0,627*	*5,57*	*0,551*	*4,5*	*394,286*	*1*	2,1504	*4,643*
7,095	*2,433*	*1,864*	*0,428*	*0*	*282,857*	*1*	58,282	*5,289*
4,715	*3,717*	*11,776*	*0,123*	*0,143*	*111,429*	*4,5*	280	*1,143*
5,282	*2,576*	*5,075*	*0,428*	*1*	*180*	*0*	39,326	*6,143*
11,834	*1,284*	*1,921*	*1,132*	*4,566*	*342,857*	*0*	330,8284	*0,066*
5,289	*2,064*	*2,075*	*0,123*	*0*	*300*	*4,5*	38,6316	*6,143*
3,412	*1,864*	*0,33*	*0,428*	*1*	*240*	*1*	17,472	*1,143*
7,717	*2,708*	*3,064*	*0,123*	*1,066*	*90*	*0,789*	49,5026	*5,289*
5,064	*1,493*	*3,789*	*0,123*	*0,429*	*514,286*	*1*	298,8972	*5,289*
5,078	*2,51*	*2,721*	*0,428*	*0*	*325,714*	*0,789*	12,264	*0,143*
3,695	*0,998*	*0,418*	*0,428*	*0*	*282,857*	*0,429*	1,8396	*5,289*
4,432	*1,427*	*5,218*	*0,123*	*0*	*240*	*0*	121,282	*0,143*
7,358	*0,998*	*1,721*	*2,5*	*0,789*	*480*	*0*	3,066	*4,566*
2,603	*1,495*	*5,987*	*0,428*	*0,429*	*180*	*1*	270,004	*1,789*
2,988	*2,216*	*5,429*	*0,123*	*0,855*	*240*	*1*	0	*6,143*
7,875	*1,853*	*1,429*	*0,428*	*5,289*	*411,429*	*0*	36,9964	*5,289*
4,858	*0,932*	*2,075*	*0,066*	*1*	*240*	*0,066*	2,7972	*4,643*
3,621	*0,836*	*0,77*	*0,428*	*0,932*	*394,286*	*0*	15,9894	*0,572*
5,416	*0,473*	*2,275*	*0*	*0*	*240*	*0*	4,6284	*0,066*
7,228	*1,21*	*1,341*	*0,551*	*0,789*	*240*	*1*	27,9804	*0,932*
2,249	*1,787*	*5,858*	*0,428*	*0,855*	*514,286*	*4,5*	13,0704	*1,578*
3,109	*2,998*	*7,284*	*1*	*0,066*	*265,714*	*4,5*	13,44	*1,066*
7,758	*2,576*	*1,484*	*0,786*	*0*	*77,1429*	*6*	60,6704	*0,066*
8,456	*1,864*	*2,075*	*0,428*	*0,429*	*214,286*	*0*	12,264	*5,289*
7,093	*1,056*	*7,143*	*0*	*0,143*	*300*	*0*	5,376	*4,643*
5,214	*2,504*	*3,789*	*1*	*4,643*	*300*	*1*	26,1212	*4,643*
4,203	*2,579*	*1,209*	*0,856*	*0*	*137,143*	*1*	51,1952	*0,209*
3,549	*5,138*	*0,418*	*0,786*	*0*	*360*	*0,066*	193,424	*1,143*
5,717	*3,207*	*3,638*	*0,428*	*1*	*240*	*1*	38,822	*1,789*
6,865	*2,653*	*2,921*	*0,428*	*4,5*	*240*	*1*	100,758	*1,143*
2,755	*2,642*	*15,341*	*0,066*	*4,5*	*574,286*	*4,5*	29,8116	*0,789*
5,375	*1,79*	*1,209*	*0,428*	*1*	*137,143*	*1*	23,058	*1,578*
3,137	*1,141*	*0,429*	*0,123*	*0*	*300*	*1*	54,5916	*5,289*
4,346	*1,93*	*1,286*	*0,428*	*4,566*	*240*	*0,066*	42,2604	*1,143*

10,377	5,575	2,789	0,428	1	154,286	0	0	1,789
1,141	2,653	8,789	1	0	351,429	0,429	212,282	6,789
7,794	3,508	4,367	0	1	120	1	72,1924	0,143
4,768	0,561	0,418	1	4,643	30	0	113,7276	0,855
4,64	0,418	2,075	0,428	0	205,714	1	42,1176	6,143
8,428	0,352	0,209	0,246	0	411,429	4,5	242,2224	0
1,669	1,776	5,644	0,856	0,789	282,857	4,5	50,3832	0,932
5,992	2,216	4,361	0,428	4,5	360	0,066	93,24	5,289
5,278	3,222	10,932	0,428	0	317,143	0	12,726	1,578
13,717	0,352	1,429	0,428	0	360	1	18,102	0,143
5,282	1,627	0,495	0,428	0,066	437,143	4,5	1440,04	4,643
5,5	2,776	5,787	0,428	0	420	0	330,008	1,143
4,146	2,862	2,286	0,786	0,429	162,857	0	117,978	1,218
4,5	2,425	8,499	0,551	1	480	0,789	72,5312	4,643
5,574	1,273	1,644	0,428	0	60	4,5	244,9216	5,289
4,623	0,921	0,132	0,123	0	248,571	0	30,7272	0,132
8,496	3,499	4,484	0,428	1,143	565,714	0,429	95,928	0,789
3,711	1,853	2,075	0,428	4,566	445,714	1	10,752	1,578
3,846	0,55	21,855	0	0,066	120	0,143	12,264	5,289
5,84	7,493	10,076	0	0	360	4,5	38,542	6,143
9,771	3,431	1,721	1	0,429	394,286	6	106,12	0,789
3,568	3,367	0,066	0,786	1	197,143	0	26,88	0,066
6,41	0,495	3,143	0,428	0	137,143	0	30,66	4,643
9,077	1,853	3,209	0,786	4,5	257,143	0	46,62	0,789
5,592	1,644	2,352	0,428	0	274,286	0	26,88	1,218
12,099	2,862	3,504	1,284	4,5	668,571	0	73,4902	0,932
7,084	1,209	0,704	0,428	1	222,857	0,066	15,6324	0,855
8,176	3,284	7,198	0,428	5,289	377,143	4,5	111,944	4,643
2,615	2,216	2,853	0,786	0	462,857	0,143	71,666	5,289
3,994	1,853	1,495	0,428	4,5	325,714	1	11,13	4,566
4,775	2,708	0,495	0,123	0	120	0,066	32,9882	1,578
6,073	1,787	6,13	0,428	1	154,286	1	46,3232	4,643
13,073	1,79	10,275	0,428	1,143	548,571	4,5	27,216	4,643
4,355	1,495	7,218	1,214	0,429	162,857	4,5	153,1516	5,289
1,485	2,939	0,921	0,066	0,066	334,286	0	96,362	0,143
4,928	1,853	2,209	0,428	0,789	197,143	1	87,976	0,932
5,229	1,787	4	0	1	162,857	0	11,662	0
4,509	2,719	3,484	0,856	1	402,857	0,143	35,4872	1,789
3,436	0,495	4,495	0	1	394,286	4,5	2,688	0,143
7,809	1,996	3,721	0,428	0	120	1	32,7992	0,143
6,312	0,429	5,066	0,856	4,5	248,571	0,789	123,298	0,932
3,269	1,13	0	0,428	0,143	180	6	55,188	0,932
5,44	2,642	1,561	0	0,789	180	0,789	32,7992	1,789
3,717	5,709	5,209	0,123	0	137,143	4,5	67,753	6,143
3,496	2,007	3,721	0,856	4,5	274,286	1	76,132	1,218
5,629	3,288	10,341	0,551	0	514,286	0	64,666	0,932
5,229	1,075	0,789	0,246	0	240	1	10,8612	0
6,363	2,653	3,075	0	4,5	394,286	4,5	154,1064	5,289

4,434	0,132	0,198	0,123	4,5	180	4,5	91,322	0,789
5,428	1,787	3,064	0,856	0	240	0,143	9,1392	0,143
6,364	2,216	3,284	0,428	4,5	368,571	0	99,974	1,578
2,853	1,361	4,789	0,428	1	334,286	0	193,424	0,789
10,131	1,495	2,132	0,786	0,132	77,1429	1	143,9872	6,789
4,154	1,647	7,578	0,428	4,5	257,143	1	0	1,218
2,979	0,987	4,561	0	0	240	4,5	0	4,566
4,181	0,341	1,418	0,428	0	77,1429	1	40,754	4,643
7,359	4,8	23,721	1	4,5	222,857	1	188,0984	5,289
4,354	0,987	1,066	0,428	0	180	1	53,0012	0,789
4,289	1,361	3,855	0,428	0	154,286	1	0	0,855
14,804	0,473	1,075	0,428	0	162,857	1	120,6184	0,572
2,188	1,141	4,932	0	1	240	0	31,7436	0,066
5,149	2,007	4,416	0,786	0	265,714	1	32,3512	0,143
12,428	2,862	18,073	0,428	0	120	6	0	4,929
10,55	0,987	2,855	0,428	1	0	0	3,066	4,566
9,929	1,776	1,924	0,428	0	197,143	0	48,7172	1,578
2,851	0,789	1,275	0,428	0,789	265,714	1	24,738	0,932
5,124	2,576	4,064	0,428	0	197,143	0	46,62	0,286
9,78	1,93	10,561	0,428	1,143	171,429	4,5	54,53	1,143
3,346	1,57	1,855	0	0	282,857	4,5	48,818	0,789
6,366	2,924	2,075	0,428	0,789	214,286	1	90,664	5,289
5,563	2,644	1,644	0,428	0	377,143	0	390,012	1,143
3,357	2,796	2,132	0,428	0,789	291,429	0	79,296	0,429
8,225	3,15	1,053	0,428	4,5	342,857	4,5	90,664	0,789
4,758	1,427	0,987	1,428	0	265,714	1	30,66	0,143
7,586	7,076	4,561	0,428	1,578	137,143	4,5	191,7846	1,578
4,928	0,921	0,998	0,246	0	154,286	0,066	84,7952	0,143
3,48	1,427	2,352	0,123	0	111,429	0	57,974	4,643
4,517	0	4	0,428	1	360	0,429	0	4,5
12,505	3,998	2,352	1	4,566	120	4,5	16,7832	5,289
6,862	1,864	0,855	0,428	0	240	0	2,7972	1,143
3,852	2,075	0,987	0,428	4,5	394,286	0	124,9612	0,789
6,998	1,561	0,341	0,428	0	222,857	4,5	130,536	0,572
4,362	2,51	2,067	0,428	1	120	0	261,296	5,289
3,915	6,144	5,644	0,428	0	540	1	76,622	1,789
3,774	2,075	7,075	0	4,5	162,857	1	180,0932	7
3,117	1,275	9,921	0,428	6,066	180	0,789	209,972	6,789
9,642	3,585	10,286	0,428	4,5	411,429	6	5,376	4,643
5,341	2,341	5,132	0,428	4,643	265,714	6	23,7216	6,143
6,638	2,062	1,71	0,428	0,789	360	1	30,31	1,143
15,366	1,647	8,141	0,428	0	180	1	306,88	5,289
2,414	0,998	0,352	0,551	0	154,286	1	34,076	0
6,572	0,858	4,561	0,786	0	257,143	0,143	54,53	4,643
4,491	2,51	5,578	0,123	1	30	1	8,3916	0,932
9,63	1,853	6,644	0,123	0,789	342,857	0	35,322	0,286
11,006	1,998	0,066	0,428	0,066	274,286	1	10,752	0,932
3,981	1,262	2,853	0,123	1,066	360	1	43,1284	0,066

8,838	2,216	3,855	0,428	0	282,857	1	4,662	0,932
8,381	2,719	1,341	1	0	120	0,789	21,4452	1,789
6,872	7,296	6,429	0,428	0	342,857	4,5	42,266	1,218
6,801	0,855	3,789	0,428	0	180	1	144,0096	0,143
9,576	2,13	1,218	0,428	1	197,143	4,5	45,738	1,789
3,986	5,281	4,367	0,428	4,566	240	1	35,322	5,289
3,412	1,996	13,864	0,066	4,566	420	4,5	12,096	6,143
6,858	4,918	2,367	0,428	0	214,286	0	0	0,789
6,572	3,433	1,998	0,428	1	394,286	0,429	48,4512	0,066
4,632	3,299	3,209	0,123	0	180	0,789	68,1044	0,143
2,491	1,075	5,207	0	4,566	300	0	56,882	0,143
3,84	4,852	1,341	0,428	4,5	248,571	0,789	54,53	5,289
9,646	2,286	1,789	0,246	0,143	102,857	1	18,6592	1,143
2,832	2,073	5,141	0,428	0,143	180	1	33,726	1,143
2,625	0,418	0,132	0	0,066	300	0	56,2296	1,066
8,848	4,852	0,352	0,123	0	342,857	1	13,9944	6,143
6,909	2,062	2,407	0,123	0,789	480	1	340,004	0,066
5,432	1,064	3,064	0,856	0,066	565,714	4,5	22,2096	0,066
3,832	1,93	10,198	0	0	214,286	1	9,5172	0,143
3,115	5,421	0	0,189	0	360	4,5	23,7832	0,932
4,355	2,853	8,932	0,786	4,566	257,143	1	91,322	5,289
3,709	1,284	5,218	0,428	1,066	205,714	0,789	9,7902	1,143
2,197	0,921	2,075	0,123	0,429	62,8571	1	93,296	0,066
4,697	1,141	6,921	0,428	0,066	257,143	1	35,322	6,143
8,191	1,721	0,198	0,428	4,5	180	0	20,5912	1,143
9,229	2,139	8,71	0,428	4,5	120	4,5	15,442	0,143
4,695	2,565	2,264	0,123	0	205,714	1	75,9752	0,209
4,08	1,93	4,352	0,428	0	240	1	7,4592	0,932
2,689	1,721	3,787	0,066	0	454,286	0,789	330,022	6,789
3,918	2,787	3,499	0,189	0,429	411,429	0	35,9156	0,789
4,586	2,862	6,484	0,428	0	240	1	2,688	0,932
3,645	1,57	4,787	0	0,066	180	0	106,624	5,289
7,01	6,487	4,418	0,312	4,5	162,857	0	5,334	1,143
2,348	2,216	14,932	0,786	0	488,571	4,5	94,6232	0,789
3,129	0,341	2,998	0,428	0	214,286	1	30,8644	1,218
5,276	6,419	2,352	0,428	1	565,714	0	0	0,789
8,087	1,93	0,132	0,428	0	334,286	0	26,88	0,495
4,561	0,473	5,196	0,123	4,643	522,857	6	72,3044	4,643
4,006	1,207	4,855	0,428	0	377,143	0	13,44	4,643
7,147	2,433	0,352	0,428	1	411,429	1	120,008	0,286
13,414	1,919	4,207	0,428	0	180	1	91,322	1,218
6,926	2,427	5,786	0,856	1	351,429	1	10,752	0,143
5,722	1,209	0,789	0,428	0	342,857	4,5	4,8384	0,143
4,77	1,493	5,559	0,066	4,5	480	1	104,902	0,789
6,021	2,856	8,921	0,428	4,5	77,1429	4,5	74,592	4,643
4,649	5,564	6,855	0,428	1	420	1	12,096	0,143
5,137	2,132	4,275	0,123	0,789	60	1	91,8316	5,5
7,916	0,572	1,998	0,428	1	257,143	4,5	10,9704	0,932

2,623	2,132	6,429	0,123	0,066	274,286	1	35,322	6,143
3,403	0,855	6,066	0,494	6	317,143	0	202,9328	0,066
8,374	1,853	4,932	0,428	0	137,143	1	13,818	4,643
6,658	2,076	3,15	0,786	0,429	300	4,5	55,944	5,289
4,271	1,427	9,853	1	0	548,571	1	5,376	0,789
9,455	2,064	3,998	0,428	0	265,714	1	3,6792	0,789
3,214	1,427	0,198	0,428	0,066	428,571	0	5,376	0,143
5,706	1,495	3,561	0,551	1,066	77,1429	1	61,4684	4,643
4,267	2,073	0,198	0,066	0	531,429	0,066	54,53	0,143
8,354	2,719	4,367	0,856	0,789	60	0	23,5032	4,643
3,071	1,853	3	0,428	1	137,143	1	52,752	1,143
4,421	1,71	8,421	1,214	0,143	154,286	0,789	19,0554	0,143
13,514	1,932	4,218	0,123	1,143	205,714	1	61,8436	1,789
3,197	1,787	8,847	0,123	0,429	282,857	0,066	32,7432	0,143
6,636	1,644	5,853	0,428	1,066	300	4,5	24,7884	1,143
3,403	2,205	0,341	0,856	0	180	1	63,4704	5,289
6,212	1,493	1,429	0	4,566	360	4,5	131,4194	1,143
4,495	1,93	0,209	0,856	4,5	188,571	1	53,3232	0,066
4,832	0,561	0,987	0,856	0	265,714	4,5	63,5152	4,643
4,849	1,713	2,429	0,123	1	248,571	1	560,028	0,066
3,547	1,559	0,264	0,428	0	180	1	49,3192	1,578
9,712	3,719	3,559	1	0,143	257,143	0,066	224,602	0,066
4,638	3,433	7,075	0,786	0,143	445,714	0,143	123,242	4,643
1,485	1,218	8,064	0,123	4,5	334,286	1	96,306	0,143
3,406	1,427	0,704	0,786	0	300	4,5	62,3924	4,643
5,133	1,853	1,141	0,428	0,066	308,571	0	65,3072	0,789
4,08	4,011	6,932	0,428	1	231,429	4,5	7,35	5,289
4,919	2,862	3,075	0	0	137,143	4,5	24,528	1,218
5,35	0,33	1,275	0,428	0	497,143	0	12,726	4,643
3,045	3,855	1,644	0	4,566	420	1	0	1,789
3,214	1,864	2,578	0,428	0,143	531,429	1	66,2564	1,218
5,629	2,996	8,719	0,428	0	282,857	0,789	0	4,643
6,43	1,625	1,341	0	0	317,143	0,143	29,6632	0,066
5,429	1,352	10,998	0,123	1	411,429	1	22,414	6,143
4,549	1,57	0,561	0,123	0	428,571	0	18,396	0,789
4,986	1,361	1,209	1,214	1	60	1	33,5804	0,066
6,658	1,853	3,075	0,066	0	300	0	4,662	0,572
19,139	0,495	6,221	1	1	411,429	1	140,9604	1,066
6,726	1,787	7,864	0,786	4,5	300	0	0	5,289
3,489	0,132	1,132	0,428	0,066	274,286	0	60,004	5,289
0,968	0,198	1,209	0,428	1	480	4,5	10,752	0
5,727	1,921	4,998	1	0	300	1	228,0012	1,789
3,098	0,33	0,341	1	0	420	1	0,2268	1,578
2,26	1,787	2,407	0,123	0,143	197,143	0	25,872	5,289
5,432	7,142	1,207	0,786	0	480	0,066	605,5084	0,143
5,067	1,067	3,209	1,214	0	180	0,789	138,0092	0,286
4,124	2,293	3,284	0,428	4,5	282,857	4,5	62,9412	4,643
12,806	1,787	1,286	0,428	0	214,286	1	108,388	0,143

5,698	1,93	4,864	0,428	0	445,714	1	93,7104	0,789
2,054	1,35	4,143	0,428	0	180	4,5	0	1,218
8,2	6,004	17,575	0,428	4,5	162,857	6	0	0,143
11,596	2,721	5,15	0,428	1	274,286	1	76,622	1,789
8,357	3,796	8,209	0,428	0	300	0,066	46,62	5,289
5,284	1,908	12,064	0,428	4,5	154,286	4,5	47,082	4,566
6,069	2,348	4,429	0	4,566	248,571	1	48,0032	0,209
4,849	1,787	6,209	0,428	0	180	1	25,9084	5,289
4,926	3,216	3,721	0,123	0,789	180	0	152,264	1,143
4,432	1,13	7,275	0,428	4,5	394,286	1	40,754	0,855
6,335	2,853	2,998	0,909	1,143	154,286	1	83,6192	4,5
4,854	1,429	5,141	0,428	1	428,571	0	2,688	5,289
2,549	2,51	8,13	0,066	0	454,286	4,5	93,296	6,143
7,869	3,299	7,504	0,786	4,5	214,286	4,5	273,224	5,289
5,849	2,71	8,572	0,428	0	300	1	43,7444	6,143
4,495	1,787	0,33	0,123	0	480	1	69,93	0,572
4,832	1,273	7,998	0,189	4,566	394,286	1	151,4366	4,566
6,137	3,007	0,847	1	4,5	360	0,789	43,428	4,643
6,64	2,93	13,709	0,428	4,5	240	4,5	37,1364	6,066
2,491	2,216	8,429	0,428	0,789	180	1	100,744	0,932
11,658	2,862	3,653	0,428	4,5	137,143	4,5	36,6492	0,789
6,59	1,721	5,572	0,428	1	137,143	0	13,986	1,143
7,06	2,853	7,075	0	0,789	214,286	0	30,478	5,289
5,433	1	4,132	0	0	300	4,5	480,032	0,789
4,86	1,218	3,066	0,428	0,143	34,2857	0	0	0
4,493	1,864	1,209	0,123	0	180	0	2,688	6,143
5,583	1,864	2,352	0,428	0,143	171,429	4,5	68,068	5,289
4,06	1,141	0,429	0,123	4,5	222,857	0,789	16,6152	4,643
2,417	1,57	12,418	0,123	0	300	4,5	6,132	0,789
6,287	1,71	2,433	0	4,5	325,714	1	76,2832	0,789
5,575	2,862	2,932	0,428	0	240	1	129,948	4,643
4,643	1,064	2,921	0,123	1	197,143	0	0	6,143
3,054	1,787	2,341	0	0	360	0,066	61,9612	5,289
5,572	1,853	0,209	0,123	1	180	0,066	66,794	4,643
6,3	2,141	1,132	0,428	0	282,857	0	81,634	6,143
4,792	2,565	6,064	0,066	4,5	420	4,5	58,282	6,789
4,146	2,853	3,921	0	0,143	257,143	1	186,62	6,066
10,205	1,352	0,209	0,428	0	377,143	0	233,24	0,143
5,5	2,842	1,218	0,428	0	334,286	1	32,4744	0,932
11,017	1,495	4,361	0,786	0,066	497,143	1	164,528	5,289
6,655	3,156	8,066	0,786	4,566	240	4,5	120,652	0,789
5,225	2,776	17,286	0,428	4,5	240	4,5	0	4,643
6,144	1,57	2,715	0,428	0,066	240	FAŁSZ	61,32	0,932
4,645	2,141	2,064	0,123	0	137,143	1	4,662	1,143
4,553	3,288	10,139	0,786	4,5	505,714	1	89,4152	1,789
5,375	2,642	4,638	0,786	0	342,857	1	72,282	4,643
18,008	4,51	7,787	0,428	5,289	180	1	256,62	0,789
2,714	1,787	5,932	0,428	4,5	120	1	48,0032	5,289

6,238	1,721	0,789	0	0	120	1	39,634	0,143
4,36	1,484	3,987	0,123	4,566	428,571	6	46,62	0,066
8,765	2,853	6,77	1,428	0,066	308,571	4,5	62,5016	5,289
4,344	2,644	3,286	1	0	651,429	0	29,5302	4,643
5,858	1,35	1,853	1,123	1	248,571	0	54,5524	0,789
3,632	2,205	2,066	1,214	0,495	617,143	1	42,322	5,289
7,508	2,499	0,429	0,123	4,5	94,2857	4,5	10,2816	0,143
3,689	2,842	5,284	1	4,5	360	1	22,5204	0,789
1,482	1,998	0,429	0,428	0	480	4,5	37,296	0,143
7,449	2,796	2,932	0,786	1	248,571	6	57,3272	0,932
2,414	1,207	3,721	0,428	0	240	1	37,296	0,429
3,489	1,561	7,141	0,428	0	154,286	1	12,39	0,789
16,148	7,355	3,644	0,428	1	214,286	1	123,242	5,289
7,521	3,721	4,222	0,428	0	300	0	2,1504	4,643
5,724	3,071	10,642	0,123	4,566	540	4,5	136,325	0,789
8,365	1,352	1,275	0,066	1	102,857	1	140	0,143
2,329	1,35	4,064	0	1	154,286	0,789	103,432	5,289
5,217	1,578	0,987	0,786	0	428,571	0,066	186,0852	0
6,716	1,933	9,073	1,214	1	180	6	26,9892	5,289
11,147	2,499	5,132	0,856	0	274,286	1	386,596	0,789
8,859	3,156	3,504	0,428	0	300	1	0	1,143
6,576	5,567	7,787	0,786	5,289	137,143	1	136,612	5,289
4,061	5,643	9,075	0,786	1	265,714	0,789	73,038	6,143
4,575	1,93	6,284	0,428	1,066	240	0,789	37,3072	4,643
4,854	2,785	7,13	0,428	4,566	274,286	1	354,592	5,289
12,654	2,073	10,436	0,551	4,5	300	4,5	48,398	4,643
6,944	3,499	17,075	0,428	0	274,286	0	78,652	5,289
6,66	2,007	2,721	0,123	0,789	60	0	17,1696	0,789
9,408	2,642	1,429	0,428	4,566	462,857	1	29,4112	0,066
4,432	3,719	12,198	1	0,066	257,143	4,5	35,378	4,643
16,084	1,855	4,855	0,786	4,5	180	1	69,7116	1
4,858	5,707	8,71	0,786	0	565,714	4,5	42,2016	4,643
2,691	1,207	1,198	0,123	0	120	4,5	31,038	4,643
7,554	2,493	6,856	0	4,5	240	1	18,8776	5,289
3,008	2,007	9,721	0,123	0,143	540	4,5	10,752	5,289
9,387	5,498	2,286	0,428	4,5	197,143	0	480,032	5,289
6,078	4,154	4,367	0,428	0,789	420	4,5	100,5256	4,5
8,354	1,996	5,721	1	4,566	240	1	2,7972	0,286
2,986	1,57	3,275	0,066	0,066	351,429	0,789	91,322	0,143
6,229	2,919	3,787	0	4,5	300	1	51,282	5,289
5,555	0,704	6,352	0,428	4,5	480	1	48,65	4,643
8,593	2,064	0,407	0,428	1	411,429	0,066	5,4684	0,143
5,583	2,367	1,647	0	0	240	0	26,88	6,789
7,673	3,794	1,789	0,856	0,429	240	4,5	61,32	1,429
9,294	1,721	1,921	0	0	154,286	0	19,6224	4,643
9,721	5,575	5,064	0,428	0	300	0,143	49,1848	4,566
4,706	2,853	0,789	0,428	1	197,143	1	66,794	1,218
6,574	2,359	2,341	0,428	0	317,143	4,5	10,752	5,289

3,555	0,561	3,209	0,428	0	240	0,789	18,648	1,143
3,137	0,921	1,275	0,428	0	582,857	1	36,792	6,143
9,501	5,204	14,433	0,786	0	420	1	33,726	0,789
4,649	2,216	3,484	0,123	0,789	300	0	136,612	4,643
7,392	2,721	3,713	0,786	1	214,286	0	48,818	0,143
5,414	1,908	8,432	0,786	0	342,857	4,5	38,2284	1,789
2,546	1,787	9,075	0,428	0	240	1	37,1154	0,932
9,431	1,418	4,841	0,428	1	291,429	1	181,615	6
5,641	1,864	0,429	0,123	0	257,143	1	20,7144	0,855
4,423	0,55	2,053	0,856	0,143	60	1	97,7172	0,855
12,088	0,855	2,132	0,428	4,5	240	1	560	0
2,928	1,996	4,572	0,428	4,5	274,286	1	42,322	0,286
6,35	3,431	3,864	0,428	0	180	0	37,3072	5,289
2,645	1,853	8,998	0	4,643	514,286	0	0	5,289
18,228	1,484	2,998	0,428	0,932	325,714	0	66,794	4,643
4,577	1,561	6,209	0,428	4,5	291,429	1	60,662	4,643
6,573	3,367	0,341	0,428	1,066	325,714	4,5	30,66	0
4,427	1,559	3,352	0,428	1	120	1	90,9832	0,572
4,997	0,407	2,721	0,428	0	257,143	0	108,822	6,143
4,72	2,15	2,864	0,428	0,143	317,143	1	53,984	1,143
3,258	2,367	3,484	0,428	4,5	197,143	1	9,2484	4,643
4,919	0,998	1,198	0	4,5	300	0	56,882	4,643
8,807	2,207	5,143	0,428	0	222,857	4,5	55,944	5,289
9,349	1,919	9,218	0	0,143	145,714	4,5	43,442	1,218
9,365	1,996	3,075	0,123	0	394,286	1	148,9292	0,789
4,26	1,273	1,93	0,856	0,789	120	1	171,4664	0,789
7,164	3,585	4,436	1	0,789	154,286	1	33,5552	5,5
7,164	5,784	0,921	0,123	4,5	565,714	0,789	326,592	0,789
5,135	2,864	6,141	1,572	0	420	1	420	0,143
5,344	0,352	1,132	0,551	0	514,286	1	40,9724	0,932
4,269	1,143	2,132	0,123	1	27,1429	0,066	32,69	1,143
4,795	0,572	0	0,123	0,143	300	0	0	0,143
8,415	7,147	4,132	1	0	334,286	4,5	11,662	0,429
5,135	1,067	1,209	0,786	0,855	428,571	0	104,902	0,495
9,279	1,218	1,341	0,428	0	325,714	0	12,726	4,643
5,983	6,287	7,721	0,428	0	317,143	0	51,4472	1,789
4,717	1,985	5,141	0,123	0	214,286	1	100,744	4,643
4,291	4,154	4,207	0	0,066	600	4,5	108,388	1,143
6,695	1,495	3,141	0,786	0	480	4,5	480,032	0
5,511	1,361	3,484	0,786	0,143	197,143	0,066	30,2652	0,932
7,146	2,862	3,921	0,428	4,566	377,143	0	109,3232	0,572
4,049	1,776	4,566	0,428	0,789	394,286	4,5	82,6672	0
3,269	1,644	1,075	0,551	0,429	240	0	24,3362	0,143
7,15	0,275	0,132	0,189	1	145,714	4,5	97,9314	0,066
7,861	3,209	4,284	1	0	137,143	1	0	6,143
3,395	5,861	10,933	0	0,429	214,286	1	36,764	0,143
4,195	6,21	8,919	0,428	4,5	394,286	1	50,4896	6,789
4,66	3,945	6,141	0,428	0,143	240	4,5	570,8304	5,289

6,137	1,493	3,207	0,428	1,066	214,286	1	127,288	1,143
10,494	2,642	9,365	0,428	4,566	248,571	4,5	109,4492	0,932
8,206	0,855	3,275	0,494	0	351,429	0,789	18,102	1,143
3,926	1,416	1,141	0,428	4,5	428,571	0,143	10,752	1,578
5,135	0,55	5,207	0,428	0,855	531,429	1	48,398	0,789
5,728	1,998	4,367	0,428	1	265,714	4,5	0	1,143
4,216	1,35	5,787	0,428	0	180	1	85,946	6,143
5,394	2,064	4,209	0,123	0	394,286	0	10,038	1,143
4,689	1,418	1,207	0,428	4,5	342,857	1	389,6312	5,289
3,203	2,141	0,55	0	6	274,286	0,066	48,5492	5,289
4,503	1,493	0,209	0	0	197,143	4,5	17,472	0,143
4,561	1,787	1,141	0,428	4,5	77,1429	0	0	0,789
5,306	1,864	7,209	1	1	102,857	0	58,282	4,643
4,935	2,361	3,721	0,786	0	317,143	0	131,67	4,643
6,667	3,508	5,141	0	0,143	240	4,5	28,14	1,143
8,426	1,13	9,053	0,428	0,429	171,429	0	14,6244	0,789
9,503	5,784	5,572	0,551	4,5	197,143	1	215,5944	0,066
4,841	2,13	2,141	0,975	0,932	180	0,143	76,2832	0,789
3,703	2,928	2,216	0,786	0	197,143	0,143	39,4016	1,143
1,595	1,207	1,429	0,123	0	480	1	186,62	6,143
4,621	1,57	4,921	0,428	0	497,143	0,789	54,586	5,289
6,939	2,433	3,407	0,428	0	437,143	4,5	90,006	1,143
10,063	2,642	9,643	0,786	1	137,143	4,5	47,6644	0,855
13,933	3,578	5,575	1,856	0	180	4,5	233,24	0
7,007	3,721	3,495	0,428	0,066	300	0	54,53	1,789
3,335	1,559	3,644	0	0,429	514,286	0	151,312	6,143
5,999	2,708	1,578	0,428	0	214,286	1	39,634	5,289
4,36	2,708	4,141	1	0	60	0,143	27,7872	1,143
5,344	6,639	10,293	0,428	1,066	180	1	36,078	4,643
5,12	1,998	1,286	0,123	1	137,143	0	8,1732	4,643
4,05	3,499	0,132	0,428	0,789	197,143	0	116,62	4,643
9,664	1,57	2,578	0,428	4,5	497,143	4,5	33,1072	4,566
3,852	1,218	1,561	0,066	1,066	274,286	0,143	68,5874	0,143
8,313	1,921	6,209	1,214	0,789	291,429	4,5	18,3456	0,143
3,917	3,225	6,864	0,123	0	377,143	1	260,008	4,643
7,596	1,71	3,143	0,123	0	548,571	4,5	151,312	4,566
5,515	2,919	1,075	0,428	4,643	325,714	0,789	58,2792	1,143
3,634	1,721	0,855	0,123	4,5	120	0	5,376	0,209
5,427	0,352	3,855	0,428	0,789	51,4286	1	37,296	1,143
3,785	0,407	3,264	0,786	4,566	180	4,5	40,4152	0,143
4,558	0,198	0,932	1	0,066	240	0	73,2984	0,066
6,795	1,196	0,264	0,428	0	120	1	44,6642	0,143
2,7	4,852	2,209	0,123	0	317,143	1	5,376	0,143
4,939	3,156	6,156	0	0	154,286	0,789	38,066	4,643
8,086	2,653	1,578	0,428	0	120	1	79,94	5,289
3,497	1,987	4,209	0,123	0,066	257,143	0,143	6,72	6,143
2,463	6,078	8,987	0,066	0,143	360	0	70,056	0,789
9,154	2,007	2	0	0	240	4,5	18,396	0

7,862	2,007	4,066	0,066	1	471,429	4,5	38,136	0,932
11,636	1,207	1,352	0,246	0	180	0,429	35,5964	6,143
4,917	1,644	0,275	0,123	0	180	4,5	60,662	6,143
3,071	2,653	4,286	0,123	0,429	197,143	6	16,4346	0,572
3,711	1,933	3,842	0,786	0	197,143	1	89,992	5,289
7,087	3,365	1,71	0,551	0,789	137,143	0,789	0	0,143
4,856	1,93	5,143	0,428	4,5	120	4,5	0	4,643
5,583	2,293	5,51	0,428	4,5	11,4286	1	270,018	1,789
6,003	3,576	1,495	0,856	0,143	154,286	4,5	38,465	0,143
6,366	2,425	10,282	0	0,932	377,143	4,5	0	5,289
9,419	2,367	2	0,428	0	282,857	1	30,002	1,143
5,21	2,862	9,218	0,428	0	342,857	0,789	16,7832	1,143
4,649	1,638	13,432	2,5	0	180	4,5	13,44	4,643
5,801	2,576	4,007	0,428	0	360	0	140	5,289
6,148	1,207	0,275	0	0	248,571	4,5	6,9888	0
9,483	1,93	2,572	0	0,789	462,857	0	16,9932	0,066
7,018	1,864	0,418	0	0,066	154,286	0	0	4,643
8,72	0,987	2,407	0,428	0	240	1	3,78	0,789
6,581	3,932	10,83	0,428	1	162,857	0	300,02	6,066
3,994	0,932	0,286	0,123	4,5	197,143	0	10,752	0,143
5,124	1,647	10,653	0,786	0,429	531,429	1	18,648	5,289
2,862	3,222	8,789	0,428	0,066	77,1429	1	480,032	1,578
5,212	1,93	3,064	0,551	0,066	120	0	0	4,643
5,139	1,35	2,504	0	0,932	240	0	17,8444	0,789
7,726	2,073	2,284	0,123	0,429	240	0,789	0,2268	5,289
5,361	1,998	4,352	0,123	4,5	274,286	1	4,662	0,143
4,774	1,787	0,132	0,123	0	120	0,143	38,066	0,143
4,935	1,067	6,341	0,428	0	222,857	0,143	41,958	5,289
3,436	0,352	0,066	0,428	1	34,2857	1	23,4276	0,143
2,909	0,341	2,275	0,123	0,143	300	0	0,756	1,143
4,495	1,644	7,075	0,428	0	454,286	4,5	60,662	0,066
3,931	1,418	4,721	0,428	0	300	0	5,376	5,289
4,707	2,436	6,284	1	4,5	222,857	4,5	32,69	1,143
5,137	1,13	6,987	0,066	0,066	137,143	4,5	30,002	5,289
3,511	1,79	0,789	0,428	0	248,571	0	40,754	0,066
2,842	2,007	0,209	0,428	1	240	0	25,102	4,566
3,073	2,282	2,007	0,066	4,5	291,429	4,5	46,62	5,289
2,282	6,353	1,207	0,123	0	334,286	0	13,818	6,143
3,651	2,71	7,053	0,132	6	377,143	0,066	431,69	6,143
1,262	3,299	5,141	0,428	0	180	0,789	197,9852	1,578
6,647	2,499	2,565	0,123	0,789	600	0	57,974	0,143
2,252	2,218	0,998	1,123	0	334,286	0	120,7612	1,143
10,145	1,427	1,196	0	0	231,429	1	270,018	5,289
6,72	0,429	1,855	0,123	0	240	1	3,9312	6,789
8,163	1,218	10,007	0	0	180	1	30,002	5,289
5,638	1,056	0,132	0,066	4,5	274,286	0	9,408	0,789
5,432	2,776	3,932	0,246	4,5	411,429	0	61,32	0,789
5,718	4,222	7,416	0	0	248,571	4,5	58,282	1,789

4,621	5,784	4,576	1,572	0,789	300	0,429	106,12	0,932
9,437	1,919	2,987	0,428	0	351,429	4,5	49,5824	0,286
9,569	0,998	0,429	1	0	325,714	1	239,666	0,789
4,574	6,21	1,853	0,428	0,143	445,714	0	11,13	6,143
4,77	1,207	1,075	0,428	0	214,286	0,143	43,1284	5,289
6,201	1,57	0,998	0,856	1	437,143	1	99,6352	0,932
6,284	1,484	3,284	0,123	4,5	385,714	1	75,936	0,789
5,792	2,13	7,33	0,428	4,5	154,286	1	330,022	5,5
5,651	6,21	2	1	0,143	300	1	544,698	6,143
3,926	7,076	7,787	0,123	0,789	317,143	4,5	20,076	0,143
6,078	4,918	2,51	0,123	0,143	265,714	1	24,4902	4,643
3,772	1,064	1,407	0,066	0	548,571	1	104,902	0,143
6,126	2,713	1,352	2,5	0,143	300	4,5	0	1,218
9,921	1,919	2,495	0,428	0	231,429	0,066	139,944	0,932
3,851	0,998	1,209	0,066	1	120	1	64,666	0,143
5,427	1,273	6,921	1	6	120	0	0	5,289
9,033	2,499	2,644	0,428	4,5	394,286	0	19,0204	1,143
5,647	1,578	0,275	0,428	0	540	0,789	18,648	0,789
3,192	1,919	5,341	0,428	0	257,143	1	10,2144	6,789
3,689	4,44	6,141	0,123	1	154,286	1	48,65	1,143
19,502	2,576	1,066	1	4,5	300	4,5	180,012	4,643
29,15	1,787	9,218	0,856	6	120	0,789	0	0,143
4,711	1,275	1,429	0,428	1	68,5714	4,5	23,3016	6,143
1,474	1,493	8,352	0,123	4,929	600	4,5	54,53	6,143
8,306	3,508	4,51	0	0	120	1	11,662	4,643
5,071	3,565	5,719	0,123	4,5	137,143	1	88,592	4,643
4,014	2,644	2,007	0,428	5,289	240	4,5	330,022	4,5
11,046	2,418	0,932	0,428	1,066	154,286	0,143	22,1662	0,789
5,08	2,075	3,418	0,786	6	180	0,789	17,8332	5,289
4,856	3,928	3,341	0	0,066	445,714	1	380,016	1,143
6,507	2,15	2,143	0,786	0	177,143	1	0	6,789
5,935	1,504	0,209	1	1	291,429	0	31,7436	4,643
4,076	1,644	0,209	0,123	1	180	1	30,002	6,143
9,089	1,119	2,987	0	0,066	137,143	0,789	7,4592	1,789
5,57	3,365	3,55	1,428	0	557,143	0,066	120,008	0,495
3,34	1,284	3,932	0,786	0	60	0	41,72	0,932
1,911	1,218	10,429	0,246	0,789	300	4,5	5,376	0,932
8,159	1,853	0,418	0,428	0	137,143	0	0	0,143
10,729	3,365	2	0	0	137,143	1	73,444	0,143
4,649	1,504	0,143	1,786	0	488,571	4,5	100,6152	4,5
5,51	7,01	8	0,786	0	402,857	1	60,62	6,789
2,392	1,79	9,273	0,123	4,5	445,714	0,143	51,282	0,789
2,205	0,998	6	0,428	0	420	1	32,634	4,929
3,983	1,93	1,561	0,123	0	325,714	1	18,7656	4,566
8,417	2,711	9,987	0,428	0,066	205,714	0,143	59,4258	0,789
6,071	2,284	4,787	0,428	0	94,2857	1	26,88	4,643
4,708	2,721	6,644	0,786	0	180	0	10,752	5,289
2,562	1,93	2,209	0,066	0,066	137,143	1	111,496	4,566

2,681	5,927	8,933	0	0,429	240	0,789	69,3504	4,643
2,979	2,207	11,207	0,123	4,5	120	0,789	51,7552	0,143
5,278	2,062	9,075	0	4,643	402,857	1	45,2816	5,289
4,443	3,719	5,075	0,428	0,143	240	1	0	4,643
8,02	3,576	2,789	0	0	94,2857	1	38,066	5,289
12,008	4,442	9,499	0,786	0	171,429	4,5	126,784	0,143
2,46	2,433	9,132	0,428	0,789	137,143	6	121,324	1,143
3,416	1,207	19,275	0	0	257,143	4,5	48,398	0,789
5,412	1,35	0,286	1	0,066	300	1	7,4592	0,143
5,559	2,499	1,275	0,428	0,066	317,143	0	151,326	1,143
5,431	2,719	0,209	0,428	4,5	257,143	1	43,1872	5,289
3,698	2,13	9,71	0,786	4,566	300	0,789	35,322	4,643
4,088	1,284	4,075	0,428	0	197,143	1	78,82	1,578
5,138	2,062	4,787	0,066	1	531,429	1	99,9404	5,289
3,207	2,143	1,286	0,428	0	231,429	0,789	148,064	1,143
3,7	2,064	0,198	0,123	4,5	471,429	0	0	0,143
5,803	3,005	6,066	0,909	6	514,286	6	0	6,789
3,7	0,396	4,787	0,856	1	651,429	0,143	59,3124	1,143
11,375	1,93	3,209	0,786	0	102,857	0	46,62	0,429
3,403	1,93	1,998	0	1	274,286	0,066	116,872	0,143
6,755	1,35	4,709	0,428	0	111,429	0	108,528	0,066
4,638	1,207	3,484	0,246	0	360	1	16,2204	4,566
7,297	2,352	8,572	0,428	0	120	1	51,296	4,643
6,164	6,43	1,644	0,428	0,855	420	1	206,612	5,289
2,864	3,064	2,143	0,551	1,066	137,143	1	38,1192	1,789
4,992	2,073	1,932	0,428	1	214,286	1	80,7884	1,789
3,909	0,693	0,33	2,5	0,789	180	0,066	43,9194	1,578
4,137	1,13	10,51	0,428	4,5	351,429	0	103,432	0,789
5,298	0,704	0,932	0,428	0	300	1	29,8116	0,789
2,414	2,273	0,209	0,551	4,5	317,143	4,5	35,322	4,566
3,783	1,185	2,644	0,428	4,5	180	4,5	10,752	1,143
6,361	1,064	6,014	0,428	0,429	282,857	0,789	26,663	1,218
7,724	2,796	2,286	0,428	0,143	265,714	4,5	112,6244	4,643
19,273	3,721	5,13	0,428	5,289	402,857	1	87,4384	1,789
6,625	1,504	0,209	0,123	0	257,143	4,5	16,3212	0,143
4,775	3,365	4,728	0,428	0,066	291,429	0	22,764	1,578
3,997	0,987	0,132	0,123	4,5	548,571	1	20,79	6,143
6,37	2,425	6,842	1	0	274,286	4,5	273,224	4,643
3,346	6,353	4,156	0,428	0,143	437,143	4,5	300,02	0,143
6,142	0,638	9,222	0,428	0,066	462,857	4,5	14,4592	1,143
3,983	0,847	3,987	0,123	0	291,429	4,5	6,5772	1,143
5,645	0,913	4,209	1	0,789	154,286	4,5	4,662	4,643
5,773	6,573	6,359	0,909	0,429	325,714	0,143	0	0,143
3,339	2,51	16,721	0,428	0,789	137,143	4,5	7,707	5,289
4,995	6,498	1,864	0,428	0,789	514,286	1	724,598	0,789
5,79	1,864	2,141	0,674	0,066	197,143	1	54,1576	0,143
3,197	6,507	0,998	0,786	1	197,143	0	127,288	5,289
5,638	2,282	2,853	0,551	0	197,143	1	13,608	1,578

3,137	5,927	4,341	0,786	1,066	240	0	10,752	5,289
3,636	1,721	1,209	0,066	0	188,571	0	48,65	6,143
16,715	1,143	3,141	0	0	120	0	10,2564	0,789
5,223	1,141	0,418	0,551	4,5	257,143	0,143	63,4592	1,578
4,481	1,996	1,13	0	0,429	197,143	0	50,9502	0,143
4,486	1,787	0,998	0	0	317,143	0,066	87,7212	4,643
3,712	1,932	7,787	0,428	1	300	1	60,662	0,143
4,293	2,642	16,855	0,786	0	197,143	6	60,004	0,789
7,794	1,056	3,352	1,551	0	162,857	4,5	70,756	4,643
4,907	1,996	10,352	1	6,143	428,571	1	186,62	6,789
2,997	1,853	7,864	0,786	0,495	360	0,143	60,004	5,289
4,906	0,99	0,921	0	4,5	240	0	8,2824	0,143
5,858	1,93	9,284	0,428	0	317,143	4,5	0	6,143
3,643	1,996	3,361	0,786	0	300	4,5	208,397	0,143
6,306	1,286	0,143	0,123	1	240	0	0	0,286
4,139	0,352	1,066	0,123	1	180	0	4,662	4,566
6,114	1,787	7,721	0	4,5	377,143	0	76,622	5,289
4,357	5,421	10,275	0,428	0,143	394,286	1	69,972	4,643
6,562	2,273	10,144	0,786	0	154,286	4,5	66,794	0,286
4,491	0,352	2	0,066	0,789	300	4,5	140	5,289
4,129	1,561	2,921	0,786	0	480	1	0	0,789
2,142	0,638	6,286	0,123	0,789	214,286	4,5	45,661	0,789
3,67	2,282	5,561	1,572	0,066	265,714	1	75,2836	0,209
7,003	6,43	2,429	0,428	0	205,714	1	288,0192	1,789
5,05	1,207	3,987	0,246	0	197,143	0	69,972	5,289
5,774	2,073	4,781	0	0	420	4,5	140	4,643
6,432	2,785	3,284	0,428	0,066	154,286	0	31,542	1,143
4,915	1,218	1,13	0,428	0	257,143	4,5	60,62	4,643
5,489	2,785	1,064	0	0	394,286	1	46,13	1,578
8,429	1,141	2,132	0,428	0	402,857	1	13,986	0,932
4,184	2,207	5,418	0,428	0,429	231,429	1	239,988	4,566
11,807	2,722	0,429	0,428	0	94,2857	1	30,66	1,143
4,297	1,776	6,473	0,856	1	282,857	0,143	0	4,643
5,482	1,207	1,143	0,428	1	240	4,5	16,926	0,932
8,462	2,367	3,367	0,428	0	360	1	0	1,578
1,273	0,275	10,776	0,428	1	342,857	1	51,4472	1,218
9,798	0	0,275	1	4,5	98,5714	0,789	5,5944	0,789
3,48	1,988	1,559	1,572	0	394,286	4,5	45,528	0,066
3,717	5,707	3,638	0	1	171,429	0	39,634	1,218
5,009	2,631	15,776	0	5,289	240	4,5	30,66	6,789
4,129	2,853	6,073	2,5	0,789	265,714	4,5	342,4512	1,789
3,06	1,273	4,853	0,428	0	368,571	4,5	423,9004	0,932
4,157	1,57	1,429	0,786	0	19,2857	0,429	0	0,429
6,857	1,853	3,638	0,428	0	231,429	0	72,5312	6,789
3,425	3,222	4,998	0,428	1	205,714	1	48,4652	4,643
4,779	2,719	2,341	2,5	0,066	385,714	1	92,5036	1,066
5,058	1,284	1,209	0,428	0,789	77,1429	0,789	64,666	6,143
2,282	4,841	7,71	0,428	1	231,429	0	13,44	6,143

11,58	1,787	6,352	0,428	4,5	154,286	1	0	0,572
5,275	2,216	4,286	0,428	0	214,286	0,789	146,622	0,143
4,84	0,627	2,132	0,428	0	180	1	48,398	5,5
6,009	1,284	0,998	0,123	4,5	368,571	1	0	1,578
6,937	3,719	4,007	0,856	4,5	257,143	0	183,288	6,789
2,931	2,075	2,075	0,428	0	300	1	11,662	4,643
3,504	3,299	3,075	0,428	0	231,429	4,5	180,012	1,789
5,214	1,493	0,066	0,428	1	445,714	0,066	105,3136	0
5,067	1,57	4,644	0,428	4,566	137,143	1	49,686	5,289
8,882	1,075	1,627	0,428	0	334,286	0,143	107,9932	0,066
16,44	6,498	2,998	0,123	0	55,7143	1	13,44	0,789
5,632	0,627	3,484	0,123	0	180	4,5	7,4592	1,578
7,931	2,075	4,429	0,428	0	120	4,5	96,6644	4,643
4,572	1,647	1,218	0,428	0	497,143	1	35,378	1,218
3,838	1,572	6,987	0,066	0	565,714	1	76,622	0,143
4,412	5,564	0,132	0,428	4,5	514,286	1	73,7912	1,578
16,213	0,352	1,066	0,066	0	120	1	142,7972	4,566
4,124	3,132	5,352	0,428	0	240	1	328,8264	0,143
3,291	2,075	9,132	0,123	1,066	102,857	1	46,62	1,789
4,924	2,576	1,352	0,123	1	180	0,789	38,066	0,143
3,849	0,638	11,141	0,066	1	394,286	0,066	0	1,143
13,582	1,996	13,198	0,428	4,5	300	0,143	4,8384	6,789
5,713	6,144	3,578	0	4,5	240	0,143	20,8992	6,789
4,92	2,141	1,987	0	0,066	248,571	0	337,0192	1,789
6,794	1,361	3,578	0,428	1	300	0,143	57,9782	5,289
5,419	2,271	1,341	0,786	0	282,857	0	99,9502	5,289
5,287	2,785	10,565	0	1	445,714	1	98,3192	1,789
4,497	1,853	0,066	1	0,789	240	0	0,8064	1,143
6,656	1,636	5,51	1	0	360	4,5	0	6,143
6,067	2,565	1,919	0,428	0	205,714	0	65,2792	1,066
5,065	1,273	2,075	0,123	1	68,5714	1	90,006	0,066
4,994	2,359	0,418	0,428	0	300	4,5	780,038	0,066
7,266	1,418	1,13	0	0,789	197,143	1	69,4456	0,932
6,286	2,653	7,352	0,428	0	171,429	4,5	57,9026	6,789
4,338	3,717	1,864	0,428	0,789	600	1	44,7552	1,789
6,894	0,275	3,484	0,066	0,143	265,714	0	0	4,643
8,934	2,273	5,93	0,786	0	77,1429	1	108,108	4,643
3,777	2,273	3,855	0,786	0,066	360	1	0	4,643
6,515	2,499	2,721	0	0	180	1	35,322	6,066
4,05	2,653	11,141	1	0	600	0	66,6512	6,143
4,069	2,719	11,075	0,428	4,566	411,429	4,5	27,5352	5,289
4,72	2,218	2,789	0,786	1	257,143	1	136,612	5,289
4,212	7,296	3,218	0,428	0	172,857	1	39,858	0,572
8,242	2,51	5,643	0,428	4,5	222,857	1	22,764	0,789
6,505	1,427	8,561	0,123	0,789	214,286	0	40,0932	1,143
4,706	1,141	0,932	0,428	0	420	0	153,244	4,643
4,269	1,998	1,855	0,123	0	480	0,143	13,44	4,643
8,209	7,505	3,209	0,428	1,066	222,857	0,789	216,9132	0,286

5,995	2,273	3,141	1,428	1	308,571	0	198,6712	6,789
13,214	0,693	1,075	0,428	0,789	240	0	168,0112	0,932
5,421	1,636	5,71	0,428	1	257,143	0,789	106,624	0,932
8,492	3,156	11,427	0,428	0,429	154,286	4,5	306,88	0,495
3,579	0,921	1,209	1	1	68,5714	0,789	38,9312	0,286
9,94	2,205	2,286	0	0	300	4,5	389,8566	1,143
3,327	5,498	1,35	0,786	0	257,143	1	58,282	0,789
5,735	1,35	6,284	0,428	1,066	282,857	0,143	50,204	1,143
2,326	1,075	5,075	0,123	4,5	197,143	0,143	12,1212	0,932
5,144	0,847	0,418	0,428	0,789	154,286	4,5	63,4592	4,643
7,686	0,264	0,627	1	0	274,286	1	38,9872	0,066
8,66	2,796	4,429	0,428	0	377,143	0	1,6128	1,789
19,262	1,427	3,721	0,066	1	180	1	10,2144	6,143
4,935	3,073	0,341	0	4,5	368,571	0	16,7244	0,143
2,898	1,075	6,644	0,856	0,789	240	0	19,3704	0,932
6,432	1,196	3,075	0,786	1	94,2857	1	69,9552	0,789
5,06	6,287	4,932	0,786	1,789	240	1	270,004	1,143
4,575	1,919	4,284	0,428	0	197,143	4,5	46,13	1,143
9,705	1,625	6,421	0	0,789	214,286	1	60,662	1,789
12,308	1,776	2,075	0,123	0	308,571	0,789	25,3344	0,789
10,439	7,287	6,932	0	4,5	300	0	42,266	0,789
2,194	0,275	2,066	0,428	0	154,286	0,789	240,016	5,289
3,117	1,273	2,209	0,123	0	240	1	69,944	4,643
4,214	3,082	5,286	1	0,789	102,857	4,5	2269,38	4,643
3,94	2,482	5,141	0	0,066	180	0	233,24	5,289
5,562	0,407	0,561	0	0	55,7143	1	90,664	1,789
6,201	2,576	6,578	0,428	1	188,571	1	86,7132	0,143
7,717	3,642	8,653	0,123	1	120	4,5	65,2904	1,789
5,429	2,721	9,644	0,428	0	377,143	4,5	18,3792	6,789
8,415	2,499	22,367	0,786	6,789	180	6	121,324	0,143
5,501	2,35	11,141	0,428	0,789	420	4,5	28,7	5,289
4,574	0,418	13,284	0	0,066	120	0	5,376	4,5
4,917	2,996	5,932	0,428	4,566	514,286	1	221,5892	0,789
4,063	5,641	8,998	0,066	4,5	368,571	4,5	14,973	0,143
9,85	2,642	5,924	0,428	0,855	197,143	4,5	67,298	1,143
7,112	3,499	0,484	0,123	0,429	197,143	0,143	177,0188	1,143
3,623	1,064	6,132	0,428	0	420	0	0	4,643
8,236	1,427	8,709	0,428	1	428,571	0	38,542	1,789
6,937	1,636	7,284	0,428	1,066	180	4,5	0	1,789
8,156	2,713	3,51	0,123	4,5	265,714	1	194,6	5,289
5,627	1,93	2,066	0,428	0	162,857	4,5	49,2044	4,566
6,713	1,207	8,073	0,123	0	265,714	0	8,442	0,066
5,141	3,222	2,932	0	0,143	317,143	1	10,038	0,932
5,5	2,71	7	0,428	4,5	360	0	111,2412	0,789
1,669	0,484	5,418	0,123	1	180	0,143	11,8146	1,143
10,617	1,996	2,209	0,123	1	180	4,5	149,7342	0,143
5,298	1,853	1,075	0,428	1	257,143	1	0	1,143
6,366	0,998	0,429	0,979	0	445,714	0	45,6876	0

4,39	2,416	7,71	0,786	1	445,714	1	61,278	0,143
2,983	1,93	2,064	0,428	4,5	137,143	4,5	84,532	1,578
3,566	1,919	6,932	0	5,289	445,714	1	42,322	4,643
6,953	1,644	1,143	0,066	0	480	0	104,958	5,289
5,063	2,216	0,921	1,786	0,789	162,857	1	204,0136	0,066
6,587	1,57	0,209	0	4,5	300	0	420	0,789
4,647	1,853	1,495	0,428	0	180	4,5	269,976	0,429
1,856	1,776	4	0,066	0,066	240	6	0	0,789
15,077	1,71	0,495	0,428	0	197,143	0,143	29,456	0,143
5,203	1,067	6,418	0,428	0	111,429	1	27,972	1,143
2,243	0,484	2,921	0,123	0	94,2857	1	95,4772	4,643
2,702	0,572	0	0,428	0	360	0,066	0	1,143
6,726	3,431	3,132	0,428	0,789	471,429	0	256,62	0,789
7,937	1,407	4,429	0,066	1	394,286	1	48,0032	0,855
5,069	1,339	3,35	0,246	0,066	240	0,789	206,1332	1,066
4,713	1,855	5,5	0	4,5	300	1	13,818	0,143
9,289	1,273	3,787	1,786	0,789	308,571	1	11,9952	0,143
18,999	1,93	9,209	2,5	1	240	1	18,396	0,143
4,148	0,932	0,209	0,123	0,932	0	0	116,872	5,289
5,154	1,787	3,198	0,428	0	240	4,5	218,3636	4,566
4,574	0,998	3,433	0	4,5	240	4,5	0	4,643
4,698	1,196	5,852	0	1	317,143	1	86,884	1,789
1,603	1,053	4,13	0,856	0	282,857	6	133,448	5,289
9,209	4,984	6,787	0,428	1	248,571	4,5	20,6052	6,143
6,969	2,141	7,222	0,786	0,572	137,143	4,5	38,64	0,066
4,5	2,862	4,572	0	4,5	462,857	4,5	89,992	5,289
11,306	3,222	7,064	1	0,789	300	1	26,88	0,789
4,722	7,153	1,218	1	0,789	197,143	0	240,016	6,789
4,557	1,776	0,352	0,123	1	205,714	1	88,7572	4,643
3,423	3,148	1,053	0,066	0,429	480	1	35,322	4,643
7,212	1,776	0,932	0,428	0	180	1	26,88	0,066
7,221	2,073	7,51	0,428	4,566	188,571	0,789	68,6364	1,218
4,999	3,932	2,064	0,428	1	282,857	1	6,1824	0,932
6,642	2,264	0,132	0,123	0	180	0	93,296	1,789
4,653	2,864	11,299	0,428	0	222,857	4,5	200,8104	5,289
7,17	2,367	1	0,428	0	120	0,066	127,288	4,5
11,584	1,996	0,418	0,786	0,066	480	0	42,322	0,066
4,847	2,216	3,429	0,428	1	360	0,789	176,624	0,789
2,84	1,273	6,275	0,428	0	240	0	56,882	1,789
2,175	1,284	7,205	0	0	454,286	1	19,194	0,789
9,905	2,141	7,789	0,123	0	240	0	280	6,143
4,052	1,407	3,209	0	1,143	411,429	0	14,112	1,578
5,364	3,222	7,504	0,246	4,5	651,429	4,5	6,7452	0,932
5,26	7,722	11,433	1	0,789	600	0,789	29,4112	6,789
4,717	3,222	1,141	0,428	4,5	317,143	0	8,442	0,143
3,651	2,796	2,075	0,428	0	231,429	1	31,08	5,289
3,871	2,51	6,433	0,428	1,066	274,286	0,789	120,6184	1,578
4,843	1,919	13,207	1	6	222,857	4,5	41,6976	4,643

5,638	2,13	16,367	0	0	120	0,789	193,27	5,289
6,583	3,576	2,141	0,786	0,855	197,143	4,5	52,0772	0,143
3,778	6,287	1,075	0	0	257,143	4,5	76,622	5,289
5,062	1,493	4,578	1	1	180	0,143	35,378	5,289
4,003	1,196	3,429	0,428	4,5	137,143	1	60,662	0,572
7,803	0,998	3,721	0,428	1	107,143	4,5	0	1,789
5,497	2,073	1,429	0,551	4,566	394,286	6	153,3	5,289
7,387	4,734	2,721	0,123	4,5	257,143	0	5,6952	5,289
4,636	2,007	0,275	0,123	0,789	77,1429	0	25,102	1,578
5,366	1,853	2,495	1,214	1	257,143	1	7,2156	0,789
5,493	1,842	1,13	0,428	1	154,286	1	48,958	4,643
3,841	1,141	4,207	0,132	0	351,429	0	13,44	6,143
7,739	2,216	4,284	0,428	0	454,286	6	870,016	0,932
3,348	1,721	1,998	0,123	1	402,857	4,5	70,756	6,143
6,298	1,71	3,367	0,428	0,429	300	1	67,298	1,789
7,719	2,073	1,284	0,856	0	308,571	4,5	38,1752	0,143
2,065	0,715	3,141	0,123	0	334,286	0	304,682	0,143
3,48	1,416	0,341	1	0,789	325,714	0	0	5,289
4,357	2,282	4,855	0,786	0	240	1	76,622	6,789
5,278	1,932	3,35	0	0	171,429	0	72,982	0,143
2,271	2,367	7,064	0	0	317,143	1	33,166	6,789
16,136	5,49	2,064	0,786	0,495	77,1429	0,143	33,8352	1,143
6,572	1,853	1,721	0,123	0	265,714	1	92,0612	0,132
3,77	0,781	2,275	0,123	4,5	240	1	35,322	6,143
3,062	1,218	7,998	0,123	0,855	171,429	4,5	25,102	0,789
5,863	2,776	5,156	2,5	4,566	180	1	136,64	5,289
4,357	5,061	0,066	0,066	0	445,714	0,789	24,2592	0
2,909	1,853	3,427	0,123	4,5	394,286	1	330,008	0,789
3,995	1,93	3,218	0	0,789	94,2857	0,066	161,1092	0,789
2,84	6,061	21,786	0,123	0	77,1429	0	93,296	6,143
6,227	1,064	8,987	0,066	4,5	360	1	30,31	0,789
4,766	2,073	0,341	0,428	0	360	0	0	0,143
2,634	5,707	8,433	0,428	0	265,714	4,5	128,282	0,143
4,346	1,721	1,209	0,123	4,5	102,857	1	35,378	4,643
7,266	1,996	4,064	1,572	0,066	222,857	0,789	8,9292	0,143
12,126	2,645	1,644	0,428	0	180	0,143	53,984	0,429
4,146	2,499	2,495	0,066	1	257,143	1	66,6512	0,143
4,127	1,713	0,495	0,551	4,5	240	0	0	6,143
3,109	2,209	4,647	0,551	0	214,286	4,5	0	4,643
4,115	2,216	2,007	0,428	4,643	120	0	228,0152	5,289
6,152	2,216	6,495	0,428	0,143	120	1	48,398	1,143
3,695	1,987	5,064	0,428	0,429	180	1	91,5488	5,289
3,423	1,339	4,141	0	1	480	1	10,2816	0,789
10,074	3,576	5,842	0,786	1,218	120	0	193,5136	0,789
5,495	1,416	4,841	1	0	411,429	4,5	0	4,5
5,654	2,361	8,653	0,786	0,143	282,857	0,789	10,794	4,643
4,052	1,702	3,35	0,428	0,209	257,143	0,789	15,5694	0,932
8,587	2,073	3,653	0,856	4,566	154,286	1	171,3012	1,789

6,434	1,644	2,218	0,428	0	240	1	85,344	0,143
9,34	3,156	9,855	1	0	282,857	4,5	450,66	4,5
5,062	1,855	0,066	0,123	0	180	1	76,65	0,932
4,425	1,361	5,864	0,123	0	102,857	0,143	43,442	0,789
7,742	2,642	4,076	0,786	0	162,857	1	128,8924	1,578
4,915	3,299	0,066	0,856	0,789	180	0	72,1224	0,143
5,66	1,93	6,998	0,494	0	342,857	4,5	120,008	6,143
2,934	1,361	1,352	0,428	6	51,4286	1	82,4642	0,789
6,654	3,508	4,007	0,428	0,143	111,429	4,5	58,31	0,789
6,658	1,13	0,932	0,428	0	68,5714	1	53,9952	0,066
7,077	2,71	6,289	0,428	4,5	137,143	1	13,7088	0,789
5,065	0,704	2,275	0,123	1	342,857	1	127,288	6,789
4,726	2,282	3,209	0,428	4,566	420	1	67,298	5,289
4,583	2,15	9,13	0	0,789	317,143	4,5	0	6,789
4,838	0,924	0,352	0,428	0,143	600	1	73,5056	0,143
5,918	5,138	9,721	0,428	0,855	274,286	1	246,4322	0,143
6,93	1,075	0,429	0,123	1	197,143	1	75,9752	0,209
7,653	0,932	0,275	0,123	0	300	0,429	104,902	0,143
6,284	2,141	9,442	0,428	0,789	171,429	4,5	94,6232	6,789
4,489	4,929	6,721	0,123	0	582,857	4,5	16,2204	4,643
1,757	0,572	8,209	0,123	0,066	360	0	93,296	6,143
3,837	3,007	4,209	0	4,643	480	0	244,902	0,143
12,797	1,864	0	0,856	0	214,286	1	26,88	0,789
5,135	2,209	0,998	0,428	0,143	137,143	1	8,442	0,143
5,291	2,284	2,427	0,428	1	137,143	1	0	4,643
5,361	2,565	0,561	0,856	0	137,143	4,5	46,648	0,789
7,782	3,299	8,924	0,123	0,789	222,857	4,5	32,382	6,143
4,99	1,987	0,998	0,551	0	368,571	1	43,1032	0,143
3,546	0,418	0,286	0,428	0,789	257,143	4,5	0	4,643
9,428	2,064	0,855	0,428	1	180	0	12,3228	0,143
5,405	0,932	0,209	0,786	0	180	4,5	21,714	6,143
4,858	1,702	4,721	0,786	0	360	1	60,606	5,289
5,649	1,273	14,209	0,428	1	120	4,5	76,622	4,643
5,851	1,341	7,853	0,551	4,5	162,857	0,066	97,902	0,572
4,146	2,007	2,352	0,428	4,5	514,286	1	24,7884	1,578
5,994	0	1,143	0,428	1	420	0	84	0,143
11,215	2,141	3,286	0,856	1	214,286	1	15,0024	0,789
7,227	2,15	3,427	0,428	0	600	1	10,752	4,929
3,973	1,57	5,284	1,786	0,789	574,286	4,5	43,9404	0,932
3,052	2,576	17,776	2,5	4,5	480	6	55,4624	5,289
1,625	2,653	2,064	0,428	0,066	197,143	1	49,4564	0,572
3,493	6,43	1,064	1	4,643	274,286	4,5	65,268	6,143
6,741	1,427	0,286	0,428	0	240	1	611,702	0,789
9,076	5,641	11,007	0,428	0	411,429	4,5	0	0,789
6,161	2,142	4,51	0,428	1	128,571	0	93,24	4,643
4,786	2,427	4,862	0,246	4,5	197,143	1	34,7368	6,789
5,498	2,361	0,921	0,428	0	342,857	4,5	190,6072	4,643
2,271	1,787	0,932	0,428	0,789	197,143	0,789	40,0932	0,932

5,258	4,995	0,921	0,428	0,789	291,429	6	46,249	0,789
4,803	3,073	6,286	0,786	0,572	300	4,5	159,7694	5,289
1,122	2,139	4,218	0	0,066	94,2857	0	298,396	1,789
3,06	1,13	5,998	0,428	1	445,714	1	51,2232	4,643
3,546	2,776	4,209	0,428	1	308,571	0	229,04	0,143
10,572	1,144	1,998	0,428	0	137,143	4,5	51,7104	0,932
1,856	2,648	0,638	0,123	0	291,429	0	323,232	6
3,491	1,053	3,856	0,428	6	411,429	6	45,2256	4,643
7,155	1,218	2,495	0,428	0	565,714	0,143	55,7368	0,789
15,563	3,365	0,275	0,551	0	351,429	0	153,3	0,143
1,386	2,139	2,207	0,123	0,789	180	4,5	100,7804	1,578
3,052	0,627	4,429	0,428	0,789	171,429	1	158,396	0,143
4,926	0,407	2,075	0,428	0,789	240	1	201,2892	6,143
2,917	2,642	4,578	1	0	83,5714	1	182,6244	1,143
3,294	1,141	1,218	0,428	0,789	240	1	338,4136	0,789
4,577	1,218	9,644	0,428	1,789	214,286	0,789	30,1224	1,143
4,983	2,425	3,787	0,428	1,066	411,429	1	68,1044	1,578
1,339	2,073	5,996	0,428	0,855	154,286	1	78,4	1,578
5,449	1,779	2,715	0,123	0	240	0	46,62	4,643
4,575	2,719	7,064	0,428	0,143	257,143	1	106,9712	4,643
6,227	1,853	5,275	0,428	0	428,571	4,5	109,3232	1,143
1,603	2,987	11,71	0,428	4,5	205,714	6	41,958	0,855
2,134	2,93	7,293	1	0,143	334,286	0,066	121,324	0,789
13	6,573	0	0	1	60	0	457,296	0
4,058	1,067	0,495	0	0	214,286	4,5	43,6604	5,289
5,069	2,073	6,075	1,214	0	471,429	1	197,2684	0,143
2,565	2,565	1,275	0,123	0,429	437,143	0,066	440,02	0,932
4,489	1,262	9,721	0,428	0	154,286	1	61,0862	5,289
3,428	2,996	5,721	0	1,066	197,143	0,789	39,858	0,143
8,76	0,275	2,132	2,5	1	300	0,789	14,0812	0,143
8,206	1,998	13,71	0	6	282,857	0	4,3008	6,429
6,867	2,576	2,075	0,428	4,5	300	1	218,6156	0,932
3,783	0,921	0,987	0,428	0	214,286	4,5	139,944	0,789
6,762	1,067	4,632	0,066	0	274,286	0	38,2928	0
10,161	2,576	2,721	0,428	0	120	0	104,902	5,289
3,786	6,999	1,855	1,572	0	317,143	0	3869,656	5,289
7,991	5,993	4,275	2,5	0	342,857	1	44,5452	4,643
3,841	3,508	4,561	0,066	4,5	394,286	4,5	104,902	5,289
7,417	1,787	8,721	1	0	60	0	76,622	6,066
2,769	2,427	6,008	0,786	0	368,571	0,143	39,984	0,066
3,983	1,787	1,075	0,551	0	171,429	0	28,14	1,578
5,705	1,93	4,644	0,428	0	240	1	67,6592	5,289
1,493	1,578	10,066	0,428	1	137,143	0	24,528	0,789
3,176	1,13	3,433	0,428	0,429	342,857	0,066	48,6808	4,643
5,427	2,996	3,998	1,246	0,789	102,857	0	88,592	1,789
3,761	1,842	0,132	0,856	0,066	394,286	0,789	37,296	0,932
5,566	2,862	2,789	0	0,066	317,143	1	153,3	0,789
9,769	2,064	1,361	0,428	0	120	1	94,6232	0,143

3,107	1,13	5,987	0,066	1	334,286	1	65,982	4,643
3,852	6,933	6,499	0	1	360	1	363,8964	4,643
10,627	5,852	0,209	0,428	0,066	574,286	6	72,982	0,143
8,484	1,13	0,066	0	1	205,714	1	128,2792	0,143
12,688	2,499	2,341	0,428	4,566	368,571	4,5	21,462	4,643
5,058	1,71	2,998	0,123	1	145,714	0	7,56	4,643
3,827	1,493	1,853	0,786	0,066	282,857	4,5	21,0336	6,789
5,355	1,985	4,075	0,786	1	428,571	1	236,9332	5,289
13,586	2,359	1	0,428	0,429	274,286	0	0	0,143
3,973	1,57	6,359	0	0,429	462,857	0,143	64,386	6,789
1,823	1,196	5,644	0,428	0	240	0,789	193,998	4,643
3,038	1,776	7,842	0,123	4,5	300	0,789	37,2484	6,789
4,774	2,644	1,998	1	4,5	257,143	4,5	17,794	5,289
3,614	1,21	1,207	0,428	1	265,714	1	1059,968	1,143
2,645	0,418	9,352	0,551	4,643	300	0	78,82	1,143
7,223	2,787	7,218	0,428	0	402,857	4,5	35,588	5,289
5,851	0,143	1,143	0,123	0	222,857	4,5	2,856	0,066
7,445	2,139	1,864	0,674	1,066	137,143	1	9,408	1,789
1,089	2,865	11,864	0	0,066	360	0	26,88	5,289
7,991	1,996	4,264	0,428	4,566	120	0	102,564	4,643
4,561	0,77	4,418	0,428	0,789	240	0	25,6494	1,578
5,801	1,919	6,578	1,214	4,5	325,714	4,5	49,4172	4,643
3,423	2,51	1,075	0,856	4,5	428,571	4,5	35,0672	0,789
6,587	1,504	3,576	0,428	0	154,286	1	142,331	4,643
6,493	1,71	0,143	0,428	0	257,143	0	13,5016	0,789
4,223	1,218	3,075	0,428	0,789	77,1429	0	0	0,789
6,001	1,932	0,198	1,214	1	137,143	4,5	32,06	4,643
1,32	2,15	13,853	0,066	4,5	282,857	4,5	32,4996	0,932
6,289	1,053	0,341	0,123	4,5	171,429	1	10,794	0,066
3,854	2,205	7,442	0,786	0	325,714	0,789	2,3268	0,572
3,381	0,932	0,789	0,123	1	102,857	1	11,6592	4,643
3,489	1,284	4,429	0,428	0	120	0	33,852	1,578
3,889	2,433	5,053	0,123	0,429	180	4,5	35,378	6,143
12,765	8,207	10,075	0	0,143	300	1	29,022	6,789
4,424	2,919	3,218	0,786	0	565,714	4,5	43,442	5,289
7,927	1,789	2,143	0,428	0	480	1	237,902	5,289
4,924	0,932	2,855	0,786	0	120	4,5	46,62	0
2,062	3,508	3,638	0,428	4,5	180	1	26,3676	0,932
5,638	2,207	4,053	0,428	1	385,714	4,5	83,762	6,143
3,988	2,207	5,77	0,428	4,5	300	0	0	1,143
6,504	1,996	6,352	0,428	1	360	1	45,0632	1,143
3,548	1,416	5,132	0,428	6	394,286	4,5	56,882	0,143
5,014	1,286	5,143	0,428	4,5	171,429	0	0	5,289
7,598	1,864	0,275	0,123	1	85,7143	1	84,532	0,143
4,759	1,721	3,427	4,5	0	394,286	1	186,0292	0,143
10,639	1,495	8	0,786	0	214,286	1	89,992	0,789
15,288	3,066	1,932	0,856	4,5	154,286	0	0	0,286
6,087	1,93	0,638	0,428	0,495	240	0	41,958	0,143

7,696	1,286	8,407	0,123	0,143	154,286	1	14,2464	4,643
8,505	2,15	4,367	0,428	0	171,429	4,5	60,004	5,289
6,357	2,207	1,789	0,428	1	41,4286	0,789	0	1,789
4,412	0,998	1,998	0,123	4,5	308,571	0,789	46,2392	4,566
4,423	2,141	0,209	0,428	1	102,857	0	94,1192	0,143
4,41	2,576	5,284	0,428	1,143	197,143	1	4,662	1,143
5,506	1,075	0,998	0,428	0	240	1	60,7824	0,143
4,715	1,996	2,218	0,428	0	360	1	96,0064	0,143
5,146	1,627	4,51	0,428	1	137,143	0	150,01	1,578
4,995	2,644	1,996	0,123	1	120	4,5	55,7984	0,143
5,353	1,853	2,789	0	1	77,1429	1	150,752	1,143
5,713	1,427	5,141	0,123	0	300	1	58,282	6,143
6,441	1,842	1,996	1	0	94,2857	0,789	329,056	0,495
4,869	1,561	7,353	0,786	0	240	0	217,28	1,578
5,22	1,864	3,578	0,428	1,066	214,286	0	0	1,789
3,621	0,715	2,352	0,123	0	394,286	0,789	32,004	0,143
4,269	1,57	3,119	0,428	1	240	1	0,756	1,143
9,123	1,639	4,764	0,428	0,066	240	1	222,3116	0,143
13,919	2,565	5,209	0,428	4,5	137,143	1	7,56	0,143
3,981	1,064	3,721	0,428	0	222,857	4,5	61,4432	0,286
5,361	1,35	2,143	0,123	0,143	154,286	1	5,376	0,572
4,783	6,276	6,275	0,428	0	240	4,5	0	0,789
8,875	3,565	12,141	0	0,143	0	4,5	42,266	0,143
5,57	2,853	3,429	0,786	1,066	154,286	0,789	60,004	0,143
4,192	2,708	1,141	0,428	0,066	231,429	0,066	360,024	4,643
4,003	1,93	0,132	0,428	0	257,143	0	8,064	4,643
8,134	3,071	7,51	0,428	4,5	205,714	1	30,002	4,643
2,34	6,639	6,007	0	0,789	334,286	6	54,53	4,643
5,869	3,073	3,218	0,428	6	248,571	0	46,648	6,789
5,267	2,062	6,998	0,428	0,789	274,286	4,5	44,1056	5,289
4,425	6,221	6,921	0,428	1	334,286	0	123,298	0,143
5,357	2,576	7,284	0,428	4,5	325,714	1	30,66	4,643
3,337	2,51	2,064	2,5	4,566	497,143	1	31,9872	0,066
2,26	1,713	5,141	0	1	145,714	1	70,0672	5,289
7,248	0,132	0	0,123	0	180	0	61,32	0,066
3,555	2,499	3,198	0,123	0	428,571	0,143	186,9112	0,143
5,416	1,141	2,209	0	4,5	282,857	1	280,1232	1
5,082	1,998	0,429	0,428	0	274,286	4,5	0	1,789
1,702	2,499	0,066	0	0,789	420	4,5	84,644	6,143
5,992	1,787	0,429	0,428	1	282,857	1	30,058	1,143
5,794	2,007	2,218	0,786	0	240	1	0	0,143
5,431	2,996	0,407	0	1	120	1	520,016	1,143
7,136	1,644	2,855	0	0	300	1	330,022	0,932
8,363	0	0,855	0	0	162,857	0	0	0,143
6,139	1,721	2,495	0,428	0	248,571	0,066	49,4564	4,643
5,012	1,71	3,141	0,428	0	274,286	4,5	0	4,643
6,369	10,011	12,209	0,786	0,143	111,429	4,5	33,726	1,789
8,271	0,561	5,427	0,123	1,066	437,143	0	23,9148	6,789

7,154	0,495	2,932	0,428	0,429	145,714	0	78,0052	1,578
9,36	2,565	2,51	0,123	0,066	240	1	12,334	1,143
3,214	1,919	16,064	0,123	0	214,286	4,5	73,682	6,789
4,623	1,93	3,264	0,551	1,066	291,429	0	68,1044	1,578
4,85	1,787	5,35	0,428	0	197,143	0	30,31	0,143
4,992	2,139	13,218	0,428	0	257,143	4,5	59,01	5,289
4,935	1,218	6,715	0,428	0,789	240	1	16,884	0,572
4,992	2,719	1,264	0,428	1,066	214,286	1	19,572	5,289
1,966	1,064	0,198	0,123	0	300	0	62,636	0,143
4,349	2,139	5,141	0,428	1	240	0	39,634	1,143
2,568	1,998	1,341	0,551	0	428,571	4,5	48,454	1,143
5,634	2,499	3,787	1	0	154,286	1	8,442	6,143
4,135	1,209	8,209	0,123	0	274,286	0	9,324	1,143
5,285	1,71	4,073	0,123	0,855	257,143	0,143	76,622	4,643
4,773	2,71	10,787	1,572	0,066	360	4,5	61,4432	0,143
5,209	2,51	2,998	0,428	4,5	94,2857	1	176,792	4,643
4,354	3,51	5,436	0	0,143	437,143	1	60,662	5,289
5,867	5,861	0,275	0,428	0,066	514,286	4,5	60,8748	0,143
7,146	2,493	5,141	0	0,789	291,429	1	45,2816	1,789
10,925	0,066	1,209	2,5	0	377,143	1	52,1304	1,066
4,069	1,284	5,504	0,428	4,5	300	1	97,3	1,578
8,984	0,209	1	0,428	0	68,5714	6	0	0
7,438	2,282	4,284	0,428	0	360	0,789	78,4	4,643
3,898	1,185	6,499	0,786	0,789	420	1	25,102	1,143
7,164	1,93	2,578	0,428	4,5	428,571	1	95,9616	0,143
11,44	0,998	0,998	0,132	1	205,714	0,066	46,648	0,143
3,261	0,77	0,495	0,786	1	360	0,066	20,0004	6,066
6,658	3,576	3,427	0,066	4,5	205,714	0,789	18,648	0,789
4,071	0,704	6,721	0	0,143	94,2857	1	0	1,143
7,449	2,361	1,352	1	0	291,429	1	199,3264	0,789
3,917	2,998	12,275	0,786	0	325,714	1	40,754	4,643
4,788	1,493	3,578	0,428	4,5	120	4,5	0	0,143
3,401	2,719	7,064	0,428	0,143	240	4,5	293,244	0,066
5,559	3,585	7,789	0,856	0	300	1	10,038	6,789
4,984	3,354	4,858	0,786	0,066	480	4,5	46,62	4,643
1,262	0,495	2	0,428	0	214,286	4,5	61,32	4,566
3,384	0,638	0,921	0,198	0	360	1	114,2316	0
5,006	1,713	3,578	1,572	1	342,857	0,429	15,414	0,789
2,766	2,576	6,284	0,428	0,143	600	4,5	38,9872	1,578
10,385	4,8	3,361	0	0	240	4,5	140	1,789
9,387	2,576	0,264	0	0,143	360	1	320,012	0,143
3,131	2,205	11,427	0,786	0,066	274,286	4,5	16,128	0,143
8,943	1,93	1,853	0,856	1	342,857	0	43,442	0,143
6,126	2,576	1,996	0,428	0	351,429	1	76,65	5,289
3,217	2,939	9,207	0,123	1	94,2857	4,5	0	6,143
3,337	0,429	0,066	0	0	55,7143	1	180,012	0,286
4,76	2,053	1,064	0	0	111,429	4,5	2,688	1,789
7,016	2,205	2,578	0,428	4,566	445,714	4,5	51,3016	0,789

3,467	2,282	0,352	0,123	4,5	420	4,5	79,4192	0,789
11,07	2,075	4,341	0,428	0	411,429	1	288,0192	0,789
7,295	3,288	2,143	1,428	1,066	60	4,5	74,4632	4,643
3,482	1,35	3,352	0,123	0	197,143	0,143	0	6,789
5,645	3,561	4,852	0,428	6	180	0	126,9212	0,143
7,42	1,636	10,789	0,428	4,5	214,286	0	0	6,789
6,205	1,341	3,132	0,428	0	180	4,5	100,6992	4,643
3,557	2,073	9,218	0,428	1,066	334,286	0	32,0992	5,289
5,221	6,43	2,998	3,286	0	360	1	90,5632	5,289
4,131	5,575	0,352	0,786	1	360	4,5	179,984	0,789
3,423	1,273	3,921	0,123	0,789	162,857	1	323,344	5,289
3,632	1,273	0,198	0,551	0	154,286	1	8,2824	1,578
4,777	1,427	7,284	0,123	0,789	282,857	1	9,408	6,789
3,64	4,918	2,275	0,123	0,066	214,286	1	8,064	0,143
6,792	6,573	1,132	0,428	0	265,714	4,5	80,0128	0,855
4,014	1,504	1,721	0,428	0	360	0	31,2732	1,578
4,852	1,57	3,143	0,428	0,143	420	0	0	5,289
6,049	1,919	0,275	0,132	0	317,143	0,066	29,323	1,578
5,498	2,708	2,143	0,428	0,789	411,429	1	90,8124	0,789
5,581	2,785	0,198	0,428	4,5	197,143	1	278,4236	5,289
8,503	1,79	2,209	0,428	1	308,571	1	67,298	5,289
2,615	2,073	4,787	0,786	1,066	377,143	1	40,754	0,143
6,144	1,064	10,429	0,066	6	360	4,5	11,6592	5,289
5,202	2,721	0,143	0	4,5	360	0,066	131,67	1,789
5,57	2,785	1,133	0,428	0,132	402,857	0	69,5912	1,789
5,292	2,219	8,418	0,856	0,855	257,143	4,5	390,012	4,643
5,507	0,921	2,429	0,066	0	85,7143	1	6,93	0,932
6,304	2,796	5,141	0,428	0	154,286	0	48,398	0,932
2,243	1,856	2,932	0,123	4,5	222,857	0	234,0156	0,143
3,983	1,644	0,132	0	0	180	1	76,132	0,143
8,423	3,073	8,427	0,786	4,643	240	1	623,364	4,643
5,437	3,141	6,93	0,428	0	540	4,5	102,564	5,289
2,912	1,352	1,35	0	0	257,143	0,429	362,8212	0,143
7,304	1,064	0,932	0,428	1	411,429	1	99,6352	4,643
4,537	1,853	1,787	1	0	360	1	124,8212	1,578
5,788	2,132	4,495	0	0	85,7143	4,5	6,132	1,578
4,262	2,864	4,284	0,428	4,5	162,857	4,5	76,93	0,789
3,577	1,561	2,275	0,428	0,132	274,286	0	10,9788	1,143
7,703	1,93	4,064	0,123	0,143	454,286	0	20,1474	0,066
4,845	2,998	0,066	0,428	0	180	1	78,652	1,143
4,915	1,141	5,284	0,428	1	180	4,5	30,66	0,932
5,702	1,209	4,484	0,428	0	180	4,5	139,888	0,143
6,515	3,288	11,418	0,428	0,143	257,143	4,5	85,946	1,578
4,415	2,007	8,51	1	0	360	1	12,9696	0,143
6,289	3,222	9,853	1,123	0	377,143	1	18,5164	0,789
8,066	2,205	6,921	0	0,143	480	0,789	24,4776	0,143
6,067	2,853	6,275	0,428	0	154,286	4,5	0	6,143
4,709	1,198	2,352	0,123	0	265,714	1	14,784	6,143

5,929	6,221	11,141	1	4,5	300	6	198,142	4,643
10,334	0,484	1,218	1,786	4,5	180	0,143	21,7812	0,143
2,337	1,57	0,209	0,428	0	265,714	0	69,944	0,789
5,804	2,796	2,141	0,123	0	351,429	0	62,664	0,143
7,654	2,576	2,075	0,428	4,5	360	1	54,53	4,643
4,043	6,276	0,924	0,786	0	197,143	4,5	51,296	0,143
3,5	1,284	17,007	0,123	4,566	94,2857	4,5	151,312	0,932
2,26	0,836	5,275	0,123	4,5	214,286	1	131,9612	6,066
5,076	3,073	0,341	0,123	1	377,143	4,5	41,958	0,066
4,21	2,141	2,066	0,551	0,066	79,2857	1	90,006	1,789
2,689	1,35	7,416	0,786	4,643	317,143	1	31,542	0,066
4,847	2,216	3,776	0,428	0	300	4,5	30,66	6,066
15,792	1,71	1,209	0	2	197,143	4,5	110,6532	0,789
5,941	1,919	0,998	0,428	0	531,429	4,5	4,9056	0
6,438	1,93	2,638	0,428	0,143	197,143	1	6,5016	1,143
8,849	2,631	9,698	0,428	0,789	360	4,5	57,3272	1,578
4,693	1,561	3,209	0,123	1	120	4,5	0	0,209
5,067	2,282	5,275	0,786	4,643	360	1	104,594	5,289
5,214	3,644	13,141	0,428	0,066	240	4,5	91,322	0,143
3,764	1,627	3,704	0,123	0	394,286	0,789	0	0,143
3,535	3,499	3,998	0,066	0	420	0,143	40,74	0,789
3,041	0,77	0,987	0,066	4,5	308,571	1	26,7148	0,143
2,766	1,919	7,864	0,428	0	368,571	4,5	126,0952	4,643
5,627	2,499	1,341	0,428	0,066	214,286	1	49,4172	1,066
5,553	2,007	1,144	0,856	0	300	1	11,662	0,143
2,979	0,998	2,644	0,123	0	102,857	1	74,8216	0,855
3,838	3,222	11,284	0,123	4,5	428,571	4,5	63,728	6,789
4,623	1,57	2,776	0,428	1	162,857	4,5	27,9888	0,932
5,712	1,416	1,064	0,123	1	197,143	0,143	21,462	5,289
3,796	2,073	4,719	1	0	120	1	4,9056	5,289
3,132	1,353	6,15	0,786	0,066	368,571	1	64,666	4,643
6,667	1,361	2,286	0,123	0	600	1	0	0,932
5,138	0,132	1,921	1	0,789	222,857	1	61,4684	0
5,138	1,352	6,222	0,428	0	231,429	1	23,058	5,289
6,361	6,342	5,13	0,494	0	171,429	1	150,4944	6,789
8,661	1,864	0,998	0,428	4,5	111,429	1	72,5312	0,143
5,427	1,416	1,132	0,786	4,5	120	1	6,9972	1,578
4,636	2,073	3,653	0,428	1	205,714	0,066	281,9992	4,566
3,043	2,064	1,787	0,123	1	240	1	66,794	6,143
2,218	0,286	2,209	0,123	1	240	0	76,622	6,143
3,937	1,93	0,143	0,123	4,5	231,429	1	61,32	4,643
4,48	1,57	6,93	0,428	0	214,286	1	23,926	1,143
7,786	2,576	10,559	0,428	0	180	1	40,3816	0,209
4,847	5,553	1,71	0,786	0	214,286	1	79,9946	6,143
9,34	3,642	7,35	0,428	1	377,143	4,5	35,322	5,289
4,827	1,196	2,141	0	0,066	137,143	1	489,86	1,066
6,579	0,627	4,484	0,428	0,789	462,857	1	3,78	0,143
11,925	0,987	2,284	1,428	0	360	6	60,004	1,789

4,362	2,928	10,361	0,428	1,218	162,857	0	76,622	4,643
2,548	1,776	2,429	0,428	0	205,714	0	57,5568	4,643
3,755	1,207	2,71	1	0,789	214,286	1	37,4752	4,643
4,008	1,064	3,932	0	1	68,5714	4,5	38,066	6,143
6,135	5,707	5,495	0,428	0	274,286	1	49,7644	0,143
5,132	0,704	6,275	0,123	1	351,429	4,5	27,72	0,572
3,629	2,073	6,198	0,066	0	120	0	78,4896	0,143
5,146	1,787	7,284	0,428	1	137,143	1	89,992	1,789
2,77	1,132	0,066	0,123	0	137,143	1	36,764	0,066
3,911	1,141	6,275	0	0	300	1	14,2464	1,578
5,298	3,222	2,721	0,428	1,578	197,143	0	17,2564	0
4,772	2,565	1,855	0	0,789	197,143	0,789	36,792	0,143
4,832	1,064	0,352	1,572	0	428,571	1	26,5272	5,289
7,007	1,218	2,361	0,428	0	102,857	1	33,0022	0,789
3,143	0,932	3,066	0,123	4,5	462,857	0	153,44	5,289
4,703	6,716	1,856	0,786	0,429	265,714	0,429	37,8504	0,286
8,442	2,921	7,789	1	0	291,429	1	13,9944	1,066
3,478	1,647	4,209	0	4,5	531,429	1	61,348	0,143
10,901	4,698	0,275	1,428	0,789	171,429	1	17,1192	0,066
13,216	0,132	1,143	0,123	1,066	205,714	4,5	0	0,066
6,449	2,864	3,638	0	0	240	4,5	25,1496	1,143
5,08	1,13	6,209	0,066	4,5	308,571	4,5	17,4972	4,643
5,135	0,484	1,218	0,123	0	205,714	0,789	33,5664	1,789
6,936	0,789	0	0,428	1	51,4286	1	15,9432	0,143
4,047	0,264	4,275	0,428	0,066	394,286	4,5	9,408	0,066
4,764	0,484	5,572	0,123	1	282,857	0	19,6224	0,143
5,939	1,064	4,51	0,428	1	231,429	1	0	1,789
5,058	6,933	3,222	0,428	0	325,714	1	117,9752	0,932
2,986	2,282	5,207	0,428	0,429	180	4,5	55,7032	5,289
4,763	3,156	5,855	0	1	120	1	183,8144	1,578
5,366	2,062	8,787	0,428	0	240	4,5	24,4188	0,789
5,858	1,119	1,504	0,428	0	377,143	0,789	76,65	1,143
5,994	1,996	6,575	1	1	154,286	1	0	4,643
3,357	1,93	7,141	0,428	0,789	154,286	0	233,24	0,789
10,303	1,787	1,218	0,428	0	480	0,789	31,7016	5,289
6,726	1,35	4,638	0,189	1	180	1	34,7872	0,143
3,337	1,207	5,053	0	1	154,286	4,5	25,7656	4,643
1,988	1,721	1,855	0,786	0,789	497,143	6	74,592	1,578
0,209	6,29	10,209	1	4,5	214,286	6	360,024	5,289
4,569	2,064	1,721	0,786	6	222,857	0,143	245,4844	4,929
2,548	2,499	0,198	0,428	6,143	282,857	1	46,62	5,289
7,873	3,431	8,719	0,428	0,789	180	0	17,388	1,789
6,216	3,148	7,93	0,428	0	240	0,143	87,437	0,789
4,924	1,284	2,13	0,123	0,429	334,286	1	74,592	6,789
2,098	2,064	0,352	0,066	0	257,143	0	97,902	0,143
7,163	1,864	6,51	0,428	0	274,286	1	79,058	5,289
3,786	1,361	5,218	0,428	0,789	300	4,5	33,4404	0,932
5,194	2,853	8,852	1	1,789	214,286	4,5	26,1072	1,789

4,294	1,988	4,427	0,786	0	257,143	4,5	183,288	6,143
3,357	1,504	4,075	1	0	30	0	0	1,143
1,823	0,495	4,864	0,428	0	325,714	1	116,872	1,143
6,647	2,642	6,433	0,786	0	300	1	76,622	5,289
4,069	2,062	1,561	0,428	4,5	300	1	123,4492	4,643
6,306	6,507	5,853	1,214	0	317,143	1	0	5,289
2,329	6,933	7,578	0,786	0	385,714	1	24,528	0,143
8,857	1,864	3,932	0,428	0,789	368,571	1	0,378	0,932
6,636	1,853	0,987	2,5	1	180	1	157,3264	0,066
7,869	2,708	3,787	0,428	0,066	274,286	1	79,4192	1,789
5,715	3,082	9,787	0,428	4,643	514,286	4,5	60,3232	4,643
6,867	1,064	6,921	0,428	0	214,286	4,5	66,794	0,143
3,852	1,985	2,066	0,123	4,5	94,2857	4,5	29,5204	0,143
4,652	1,273	0,998	0,428	0	171,429	1	22,6632	0,789
7,141	0,987	8,132	0,856	4,5	180	4,5	14,2464	0,789
4,423	3,651	6,275	0,066	1	360	0,066	60,606	4,643
2,642	1,141	6,209	0,428	0	437,143	4,5	22,764	4,643
6,221	1,93	6,209	0,428	0	180	4,5	30,002	1,143
6,905	1,996	3,156	0,123	0	437,143	4,5	12,726	4,643
5,21	0,998	0,275	0,123	0	197,143	0	16,3212	4,566
5,28	1,55	2,998	0	0,066	205,714	4,5	93,9764	5,289
5,001	2,719	22,007	0,428	4,566	62,1429	4,5	53,6102	1,789
4,431	5,498	12,709	0,428	4,5	240	0	0	0,066
13,763	2,776	0,998	0,428	0	240	4,5	124,1044	5,289
10,085	1,207	13,721	0,428	1	42,8571	0,789	60,004	0,932
8,573	2,721	9,786	0,786	1	240	0	60,662	4,643
4,216	2,064	0,341	0,428	1	197,143	4,5	2,688	0,932
3,984	1,625	1,853	0,123	4,5	480	0	13,5324	6,789
4,482	2,064	4,495	0,428	0	180	4,5	188,7732	6,143
4,503	2,139	2,704	0	0	214,286	0,143	20,433	0,789
9,857	2,273	8,842	1,214	4,5	360	0,066	59,136	5,289
4,869	2,576	0,143	0,123	0	480	0	117,4264	5,289
6,794	2,062	2,143	0	0,789	377,143	4,5	0	0,143
4,861	1,985	3,924	0,428	0,789	68,5714	0	65,982	0,932
3,192	1,787	9,71	0,123	1	394,286	4,5	35,322	6,143
3,632	1,218	3,418	0,066	0	334,286	0	76,622	4,643
5,212	3,579	1,864	0,786	0,143	351,429	4,5	84,4312	5,289
5,509	1,504	1,341	0,856	0	240	0,429	13,7088	4,566
6,862	2,499	5,053	0,428	0	240	0	76,622	0,932
3,917	4,775	0,132	0,428	0	274,286	0	13,818	4,643
11,432	7,142	12,653	0,551	4,5	274,286	4,5	66,7884	6,789
8,046	2,484	1,198	0,786	0	265,714	4,5	60,5612	4,643
8,229	2,787	1,998	0,123	0	188,571	4,5	39,3372	4,643
7,218	2,144	6,144	0,428	6	30	0	0	0,066
3,566	3,365	4,35	0,786	0,209	94,2857	4,5	200,004	0,572
6,85	7,639	15,352	0	1,789	720	4,5	30,66	1,143
5,727	1,864	6,495	0,369	0	411,429	0	59,2144	0,143
14,429	6,221	0,066	0,786	0	360	0	0	0,789

7,152	0,998	1,075	0,786	0	368,571	0	0	0,789
8,231	0,693	5,638	0,428	0	197,143	0,789	49,9884	6,143
3,491	1,721	1,209	0,123	0	180	1	41,958	4,643
5,705	1,922	0,198	0,428	4,566	205,714	1	123,1216	1,143
12,376	1,787	1,572	0,428	0	171,429	4,5	37,296	4,643
4,704	1,787	5,858	0,428	0	171,429	0	124,5454	0,789
10,863	3,939	4,429	0,246	1	411,429	1	146,622	4,643
4,484	1,922	4,852	0	0	420	0	359,968	4,643
11,716	2,93	2,075	0,428	2	531,429	1	20,5128	0,932
4,641	2,282	5,073	0,551	0,789	428,571	1	12,096	0,789
5,293	2,207	5,578	0	1,066	128,571	0	26,88	0,789
5,503	1,427	2,789	0,428	1	325,714	0	330,022	5,289
2,252	3,431	8,218	1	4,566	300	0	40,8632	1,789
9,917	3,365	1,275	0,494	0,855	72,8571	0	31,2732	1,218
1,757	1,207	2,066	0,066	0,066	334,286	1	90,475	6,143
1,32	2,565	8,147	0	0,855	257,143	0	122,7604	0,932
4,638	1,427	5,207	0,428	1	308,571	4,5	117,565	0,932
5,427	6,078	0,932	0,428	0	240	0	46,13	4,566
3,064	0,143	14,132	0,246	0,066	291,429	1	6,1824	5,289
5,933	1,361	0,987	0,428	1	180	0	6,5016	4,566
7,075	2,139	7,57	0,428	0	411,429	0	199,948	4,643
6,935	1,493	0,484	0,428	0	351,429	1	14,952	4,643
4,72	0,979	1,198	0,428	0	325,714	1	94,4356	1,578
7,164	2,436	4,368	0,428	0,066	180	4,5	41,972	4,929
2,829	3,442	7,13	0,494	4,5	720	1	199,948	6,143
12,851	0,921	6,352	0,428	0	77,1429	0,789	9,324	4,643
4,363	0,998	0,275	0,123	0	231,429	4,5	0	0,066
4,858	2,493	7,853	0,786	0,789	214,286	1	151,312	6,789
2,9	2,642	4,275	0,786	0	300	4,5	235,928	1,066
6,983	1,284	4,367	0,428	0	171,429	1	270,8244	6,066
3,368	2,996	2,51	0,428	1	257,143	0	300,02	4,643
6,647	1,779	5,433	0,428	0	77,1429	1	116,62	5,289
3,996	1,075	1,855	0,123	0	377,143	1	53,3232	4,643
9,514	3,082	7,15	0,428	4,5	334,286	0	70,0952	0,789
5,205	2,499	4,653	0	0	60	1	0	1,143
7,453	3,51	8,352	0	0	274,286	4,5	51,1952	0,789
8,918	2,139	2,275	0,428	0	300	0,789	20,6052	6,143
3,271	3,367	4,77	0,123	0,143	342,857	4,5	263,242	0,143
10,426	2,719	7,499	0,428	4,5	231,429	4,5	14,686	0,789
5,939	3,433	2,429	0,428	0,789	111,429	0	0	0,789
4,564	6,353	8,855	0,428	4,5	514,286	4,5	136,612	5,289
5,28	5,707	4,207	0,428	0,066	214,286	0,143	65,2792	0,143
3,414	3,565	7,433	0,856	4,5	111,429	4,5	14,8932	5,289
2,317	1,787	5,341	0,123	0	445,714	1	116,62	6,143
5,706	2,565	4,71	0,551	0	497,143	1	51,7552	4,643
1,757	0,693	3,427	0,123	0,495	300	0	32,3736	4,643
3,489	3,651	2,141	0,786	0	240	0	233,24	6,066
1,79	1,13	14,143	0,428	4,5	325,714	6	50,1732	5,289

7,44	2,853	5,429	0,428	1	248,571	0,789	108,0072	1,578
4,414	2,932	4,007	0,856	1	205,714	4,5	151,5584	1,218
5,64	1,578	2,352	0,551	0	265,714	0,789	18,0012	6,143
5,44	3,431	4,495	0,428	4,5	480	1	30,002	5,289
6,59	2,293	4,143	0,786	0	180	0	123,298	0,143
4,278	5,718	10,945	0,428	4,5	437,143	4,5	0	6,789
7,517	2,645	2,721	0	0,143	240	1	93,24	5,289
6,517	1,985	4,653	0,428	4,5	317,143	0,066	36,134	5,289
4,201	2,645	11,998	0,786	0,789	351,429	4,5	9,2484	6,789
7,15	1,996	1,053	0,428	0,066	222,857	4,5	240,016	1,143
4,423	3,222	9,987	0,123	0	300	0,143	106,624	6,143
3,431	2,367	0,715	0,428	0	394,286	4,5	6,909	0,572
5,441	2,642	7,721	1	0,429	171,429	0,143	56,882	0,789
4,632	1,985	4,704	0,786	0,066	120	4,5	284,6088	5,289
6,073	1,273	2,429	0	0,429	94,2857	4,5	3,78	1,789
3,568	6,999	2,284	0,428	0,429	548,571	1	19,726	1,143
4,638	1,35	6,638	0,428	0,789	240	0,789	44,2484	0,932
5,282	1,858	3,286	0,428	0	100	0,789	0	4,643
5,203	1,853	8,075	0,786	0,429	274,286	4,5	257,1282	0,789
3,406	1,064	0,341	0,066	0	394,286	1	2,688	0,066
3,625	0,341	2,218	0,428	4,5	137,143	0	18,0012	6,066
3,128	3,862	8,066	0	0,066	360	1	300,02	4,643
9,677	7,219	0,286	0,856	0	180	0,789	2,688	1,789
4,124	1,13	0,855	1,642	0	411,429	0,789	96,0932	0,855
12,14	1,636	16,867	1,572	4,5	137,143	4,5	0	0,143
8,495	3,717	9,919	0	0	308,571	1	48,0032	0,789
3,34	3,007	0,998	1	6	600	0	83,762	4,643
10,848	2,787	3,998	0,428	1	180	0	189,056	0,143
2,689	2,864	10,418	0,551	4,643	274,286	1	43,442	5,289
10,081	1,93	3,644	0,428	0,066	231,429	0,789	106,3384	5,289
7,365	2,653	5,998	0,428	0,789	94,2857	0	38,066	5,289
5,344	2,064	0,693	0,428	0,789	282,857	0	94,5196	0,143
3,12	0,704	1,932	0	1,143	317,143	0	301,336	4,5
7,937	3,719	5,575	2,5	4,566	120	4,5	33,1072	5,289
5,858	3,222	10,209	0,428	0,143	214,286	6	0	1,789
7,069	1,776	1,578	0,428	1	291,429	0	17,2536	0,932
5,856	2,207	5,132	0,066	6	360	1	138,6672	1,789
4,704	1,196	2,71	0,123	0	222,857	1	31,542	0,143
2,939	1,207	2,209	0,428	0,789	22,1429	1	0	0,932
12,307	1,919	4,71	0,428	6,066	180	0	70,756	5,289
4,869	1,644	1,218	0,428	0	282,857	0	61,4628	4,5
5,572	1,427	0,209	0,189	0,855	394,286	0	37,3128	1,789
3,491	1,141	2,998	0,123	1	154,286	1	52,248	0,143
7,016	2,433	4,721	0,428	0	282,857	1	72,324	0,143
4,915	1,141	0,209	0,123	0	120	1	46,13	0,143
5,137	2,075	4,787	0,123	1	420	1	18,648	6,143
4,469	6,641	4,156	0,428	0	222,857	4,5	455,686	0,789
16,98	0,55	4,998	0,428	4,5	77,1429	4,5	0	6,789

4,904	0,998	6,921	0,428	1	222,857	4,5	5,4684	1,143
8,152	7,933	3,932	1	4,566	265,714	1	39,0922	1,789
3,7	2,787	0,352	0	1	120	4,5	667,226	1,143
4,64	1,218	5,075	0	4,566	411,429	0	19,74	1,143
4,051	1,427	3,429	0,428	1	522,857	4,5	23,058	1,066
5,229	6,364	8,51	0,428	4,643	257,143	1	279,86	5,289
3,291	2,576	2,855	0,428	0	445,714	1	323,232	6,789
2,889	1,644	0	0,066	0	300	4,5	52,7352	0,932
7,012	2,57	4,141	0,428	0	120	4,5	720,02	1,218
7,772	2,418	4,921	0,786	0	214,286	1	118,7452	0,143
5,572	1,207	1,064	0	1	120	1	21,4452	0,066
7,284	2,631	2,429	0,551	0,143	265,714	1	138,6532	1,143
5,717	0,987	6,932	0,428	1,066	205,714	0	22,6296	0,066
4,86	1,93	2,853	0,428	0	300	0	76,65	1,143
1,9	1,416	0,275	0,123	4,5	120	0	46,13	0,143
7,372	3,207	11,643	0,856	1,066	308,571	0	19,3704	0,286
13,017	4,499	1,721	1,428	0,789	120	0,066	140,9604	0,932
5,201	1,853	7,578	0,123	0	411,429	4,5	26,88	0,789
3,337	2,796	2,209	0,123	4,5	300	1	38,542	6,066
5,748	0,264	4,51	0,856	0	428,571	1	14,3892	0,932
7,864	1,864	4,418	0,786	0	565,714	1	13,818	1,218
8,857	1,636	10,987	0,428	1	514,286	4,5	44,7636	1,789
7,239	1,207	4,427	0,428	0	282,857	4,5	23,3016	5,289
2,469	1,284	8,576	0,428	4,5	214,286	0,066	0	0,932
3,893	1,776	4,853	1,214	1	214,286	4,5	325,1836	6,066
5,92	4,011	6,734	0,428	4,5	248,571	4,5	123,298	5,289
4,186	2,196	7,93	0	0	274,286	4,5	0	0,143
3,775	2,576	3,275	0,428	4,5	120	1	22,26	1,578
4,124	1,495	1,075	0,428	4,5	505,714	0,143	69,9384	6,143
1,93	0,55	0,495	0	0,066	180	1	15,8256	4,643
7,007	0,781	0,275	0,066	0	137,143	1	20,79	0
9,272	4,222	4,51	1,214	0,209	282,857	4,5	359,646	0
5,353	6,421	6,299	0,428	4,5	300	4,5	185,0716	4,643
5,069	2,064	9,209	0,066	0	300	4,5	58,5088	0,789
5,932	0,407	3,921	0,428	0	274,286	1	30,002	1,578
9,624	1,207	10,721	0,123	0	300	4,5	5,376	4,643
2,9	0,921	7,864	0	6,066	154,286	1	466,592	5,289
4,401	1,644	2,209	0,123	0	480	4,5	33,8436	0,143
4,135	1,207	3,429	0,123	1	360	0,066	91,322	6,143
5,074	2,139	12,139	0,428	6	282,857	4,5	27,594	4,643
7,161	3,508	6,429	1	0,789	102,857	4,5	51,296	0,932
3,186	0,638	0,275	0,066	0	445,714	0	20,9832	0
3,478	1,064	0,341	0,066	0	377,143	0	92,1284	0
2,623	1,064	2,132	0,786	0	402,857	4,5	32,69	0,932
2,485	2,352	9,418	0	4,566	120	0	58,9694	4,929
4,869	1,853	1,275	0,428	1,066	197,143	0	55,0116	1,143
1,966	0,704	0,132	0,123	0	300	0	25,9084	0,143
5,79	6,353	14,719	1	0,143	231,429	4,5	30,66	5,289

1,023	0,561	1,93	0,428	4,566	342,857	0,789	300,2412	0,789
2,988	2,139	7,79	0,428	4,566	128,571	4,5	144,522	2
3,128	4,222	2,341	0,123	1	137,143	0,789	11,1636	5,289
6,293	2,064	0,341	0,428	4,5	94,2857	0	2,8224	4,643
1,141	2,998	0,066	0,123	0,789	120	4,5	2,688	4,643
4,572	1,493	8,222	0,428	0,066	257,143	1	72,926	0,932
5,375	2,642	3,921	0	0	77,1429	0,789	0	0,932
2,625	1,207	2,921	0,066	4,566	360	0	76,622	0,789
11,856	2,57	7,709	0,428	1	214,286	0	179,984	0,143
3,615	1,853	5,196	0,123	0	274,286	4,5	49,1764	0,066
5,96	1,21	2,921	0,066	4,5	317,143	1	600,04	0,143
4,278	1,779	0,275	0,123	0	282,857	1	30,058	1,143
4,77	0	1,35	0,428	1	342,857	0	22,8522	1,143
5,86	3,359	3,264	0,856	0,789	351,429	1	60,004	4,643
5,071	2,642	2,284	0,123	4,643	137,143	4,5	19,726	1,143
2,623	1,196	4,275	0,856	0	265,714	1	99,974	0,932
5,715	1,787	3,644	1	4,5	265,714	4,5	40,754	4,643
4,069	2,576	2,433	0,428	4,5	411,429	1	29,2684	1,578
9,16	2,642	3,218	0,428	0	240	1	0	5,289
6,924	2,073	0,418	0,428	0,143	394,286	4,5	272,8152	0,066
3,918	1,284	1,781	1,572	1	197,143	1	26,1072	4,643
4,717	1,721	1,789	0,123	1	600	1	30,66	6,143
4,728	3,156	2,075	0	0,789	197,143	0	11,662	1,578
25,799	1,721	2,644	0,428	0	300	4,5	30,66	0,932
5,136	2,785	8,207	0,428	0,066	197,143	0,789	131,67	5,289
1,595	2,51	3,998	0,066	0,066	360	1	0	0,143
5,348	2,644	0,286	0,123	0	617,143	0	32,634	0,143
10,435	2,433	3,286	0,428	0	308,571	1	57,3272	1,789
6,98	1,13	1,064	1,856	0	240	1	30,8952	0,209
9,673	1,715	0,921	0,428	1	17,1429	1	288,372	1,789
5,5	1,13	3,638	0,428	0	171,429	1	53,984	5,289
6,658	1,35	3,284	0,428	4,566	282,857	1	182,8092	0,066
2,397	1,275	3,638	1	0	608,571	4,5	26,7876	0,143
5,127	2,71	1,864	1	0	180	0,066	0	0,143
5,5	0,781	0,418	0,428	0	197,143	4,5	93,296	0,143
2,186	2,062	1,559	0,123	1,143	360	0	72,982	4,643
5,208	4,007	3,075	0,428	0,143	257,143	0	23,324	1,429
5,495	5,641	5,35	0,786	4,643	257,143	1	179,984	4,643
4,511	1,001	4,286	0	0,429	137,143	1	81,606	0,932
13,93	2,853	4,352	0	1	0	0,789	0	6,143
6,418	5,83	1,141	0,428	0,066	291,429	4,5	0	0,143
3,416	1,636	3,484	0,428	0	300	0	61,32	6,789
5,638	2,073	9,13	0,428	0	154,286	0,143	18,648	4,643
14,22	1,273	1,713	0,428	0,143	214,286	0,143	36,5596	0,789
7,524	0,143	1,132	0,979	0	214,286	0,143	28,7784	0,143
5,069	1,559	10,932	1,214	0	180	1	60,606	6,789
5,489	3,005	7,842	0	5,289	248,571	1	179,984	0,789
9,569	1,284	3,367	0,856	1	60	1	89,3284	0,143

6,502	2,359	4,064	0,428	4,5	180	4,5	31,9872	1,218
6,087	0,341	3,066	0,123	4,566	300	0	156,9932	0,932
4,398	1,427	1,275	0,189	1	197,143	1	4,032	6,066
8,089	3,787	4,939	0,428	1,143	72,8571	1	90,006	1,143
4,071	6,498	0,429	0,786	1	265,714	1	116,62	4,643
6,137	2,207	6,073	0,428	0	240	1	60,004	5,289
4,405	2,57	1,284	0,428	0	317,143	0	360,024	0,143
4,775	1,79	3,721	1	0	257,143	1	33,166	0,143
2,469	0,209	12,855	0,428	0	222,857	6	48,426	5,289
1,617	1,35	9,218	0,786	1,066	368,571	4,5	34,5296	0,143
4,215	2,565	8,855	0	1	317,143	1	147,4032	6
13,702	1,93	1,864	0,428	4,5	102,857	0	138,6532	0,143
4,517	2,939	0,066	0,428	0	154,286	0	24,528	1,143
5,484	0,484	3,064	0,786	0	360	4,5	118,1992	6,066
6,477	1,493	5,341	0	0,143	171,429	0,143	22,9404	1,789
6,003	4,222	4,275	0	4,5	257,143	1	117,9752	5,289
2,986	0,627	1,418	0	1	308,571	0	2,1504	1,578
5,717	1,561	1,352	0,428	1	154,286	0,066	157,1192	0,066
4,935	5,707	4,864	0	0	428,571	4,5	27,594	0,143
2,821	2,359	5,341	1	4,5	480	4,5	94,591	4,643
3,337	1,985	5,998	0,066	0,143	600	4,5	67,452	4,566
3,698	1,064	0,198	0,428	0	137,143	1	27,72	1,143
4,126	2,276	3,855	0,786	5,289	300	1	40,4712	6,143
3,898	1,57	1,572	0,428	0,143	240	0,789	60,662	1,578
4,557	2,576	0,352	0,123	0	222,857	1	27,79	6,143
6,375	2,139	4,156	0,428	0	282,857	0,789	148,4616	5,289
5,225	1,286	3,198	0,066	0	85,7143	1	0	4,643
9,002	0,998	1,066	0,066	4,5	128,571	4,5	46,62	0,789
5,078	1,787	2,789	0,428	0,789	282,857	4,5	270,004	0,495
4,566	3,431	3,996	0,786	0,143	317,143	0,143	35,378	0,572
9,226	2,007	2,721	0	0,429	317,143	1	50,6576	6,789
3,772	2,062	5,284	0,428	0,066	248,571	4,5	59,4552	1,578
6,81	5,564	1,207	0,428	0,066	231,429	0,429	31,9704	0,789
6,304	2,642	1,284	0	4,5	342,857	0	43,1032	0,143
4,972	1,625	5,713	0,428	1,066	514,286	4,5	540,036	1,789
9,85	2,919	5,864	0,551	0	120	4,5	90,7732	5,289
3,054	1,416	3,284	0	4,5	420	1	130,312	0,143
8,58	3,442	2,367	0,428	4,5	171,429	6	1709,848	5,289
1,243	6,644	10,433	0,066	0	291,429	4,5	120,652	5,289
3,197	1,262	5,264	0	0	120	1	46,62	1,789
5,19	4,632	2,141	0,494	1	411,429	0,143	89,32	0,495
3,337	0,704	2,15	0,123	0	420	0	97,902	0,143
3,841	6,21	5,35	0,428	4,5	257,143	1	123,4492	0,789
6,216	2,787	13,51	0,428	4,5	137,143	4,5	20,2902	0,143
4,487	3,005	3,495	0	0	231,429	4,5	70,0952	1,789
4,558	2,073	10,998	0,428	4,5	574,286	4,5	117,964	4,643
12,58	2,71	10,51	0,428	0	308,571	4,5	127,4672	5,289
5,502	1,207	2,209	0,123	0	325,714	0	102,3904	1,143

5,006	1,218	8,341	0,428	1	120	0,789	30,002	1,143
4,849	1,787	0,275	0,428	1	180	0	60,662	4,643
5,943	4,995	2,998	0,786	0	317,143	0,789	130,2392	0,286
6,291	2,576	1,352	0,428	0,066	197,143	1	216,594	5,289
4,368	2,862	2,284	0,428	0	265,714	1	348,0092	0,789
2,098	2,642	14,864	0,123	9	265,714	1	251,678	4,643
5,29	3,354	1,996	0	0	454,286	4,5	143,9704	0,572
7,02	2,644	6,578	0,428	1,066	497,143	1	67,7152	0,789
10,002	5,707	3,51	0,246	0	257,143	4,5	183,232	6,143
5,93	2,576	6,842	0,428	0,066	197,143	4,5	41,958	4,643
4,209	5,498	0,132	0,428	0	274,286	1	18,102	6,143
6,658	3,431	9,207	0,551	0,143	300	1	61,4684	5,289
5,73	1,578	2,578	0,428	0	488,571	0	30,058	5,289
4,708	2,642	1,864	0,428	1	171,429	1	74,2952	5,289
4,986	2,282	2,132	0,786	1	300	0	256,5612	0,789
4,484	2,13	9,559	2,5	1,789	205,714	1	323,344	5,289
3,931	3,644	3,075	0,428	4,5	137,143	4,5	56,8764	1,578
6,412	0,858	5,641	0,428	1	300	4,5	14,5068	0,143
5,435	2,275	1,066	0,428	0	300	1	206,626	4,929
4,917	1,853	5,007	0,123	4,5	342,857	1	68,1044	0,066
3,841	2,064	6,286	1	0	308,571	4,5	18,0012	6,789
4,269	10,853	14,936	0	1,218	317,143	4,5	125,944	5,289
3,255	2,075	1,35	0,856	4,5	257,143	0,143	33,726	0,789
3,258	5,707	7,929	0,428	0,855	240	0,143	81,4604	4,643
6,941	2,719	3,93	0,428	0,429	180	1	53,984	6,143
5,146	1,141	0,352	0,428	1	462,857	0	41,72	0,066
2,703	1,207	3,13	0,428	1	154,286	1	30,002	4,643
4,436	1,143	3,578	0,428	0	154,286	0	0	1,578
5,349	2,785	11,784	0,428	0	180	0	121,324	4,643
5,704	1,93	9,855	0	0,429	522,857	4,5	904,722	0,932
5,583	6,078	12,998	0,428	1	300	4,5	11,662	0
5,792	1,416	1,484	0,428	1	154,286	0,789	0	1,578
4,621	2,853	9,341	0	0,143	342,857	4,5	123,298	5,289
2,131	2,062	4,418	0,786	0,855	300	0,789	209,6682	0,932
4,414	6,707	2,998	0,428	4,5	205,714	0	12,264	0,789
10,872	3,007	11,007	0,428	0,066	77,1429	4,5	0	6,143
3,917	2,996	4,264	0,428	4,566	420	1	97,902	6,143
5,418	2,073	9,196	0,428	0,066	180	1	82,4026	5,289
2,623	1,284	1,864	0,551	4,5	308,571	4,5	53,718	0,143
3,563	0,998	2,787	0,123	0,789	240	1	0	0,932
6,144	1,636	5,998	0,123	0,066	180	4,5	97,3	1,143
6,071	5,775	2,198	0,428	4,5	145,714	1	293,083	0,209
7,726	6,21	2,286	0,856	4,5	180	4,5	60,004	5,289
4,988	1,144	2,064	0,786	0,855	411,429	4,5	41,965	0,143
3,845	5,355	0,418	0,428	0,429	642,857	0	11,0208	0,143
6,001	2,568	9,852	0,856	4,5	77,1429	4,5	7,5264	4,643
5,284	1,702	10,627	0,786	0	522,857	4,5	95,27	0,066
2,197	1,273	7,996	0,551	4,566	180	4,5	78,1004	4,643

3,768	3,073	5,638	0,428	4,5	180	4,5	43,442	5,289
4,566	1,996	1,638	0,428	0,789	377,143	0,429	60,662	1,143
5,638	2,51	3,55	0,551	0	137,143	1	76,622	6,066
4,902	1,427	7,71	0,123	4,5	300	0	48,398	4,643
3,335	1,075	0,495	0,428	0	137,143	0	55,944	0,789
7,959	3,365	2,644	2,5	4,5	240	4,5	93,296	5,289
7,15	3,576	7,57	0,428	0,855	240	4,5	96,0372	0,143
5,195	2,776	11,286	1	1	240	4,5	4,662	6,789
7,068	1,93	1,578	0,428	4,5	342,857	1	25,2112	1,578
7,063	2,787	7,721	0,428	0,066	360	1	111,2412	1,578
4,491	2,51	0,286	0	0	428,571	0,789	15,414	5,289
4,649	1,133	1,704	0,551	4,5	360	0,143	18,102	1,578
2,483	0,704	4,293	0,066	1	274,286	1	76,622	4,643
4,926	2,93	11,287	0,786	0,066	94,2857	1	168,7784	5,289
3,855	3,361	1,287	0,428	0	162,857	0,143	30,66	0,932
3,772	2,433	10,867	0,428	0	240	4,5	240,016	5,289
5,849	2,13	3,919	0,428	0,066	197,143	1	40,9752	6,143
3,205	3,299	4,15	0,786	0	171,429	0,789	116,564	5,289
5,285	0,781	0,638	0,428	0,855	291,429	0	99,2628	0,143
6,928	3,288	3,433	0,428	1	334,286	0,066	764,1998	6,143
3,698	2,796	19,273	1	0,066	120	4,5	222,908	0,789
6,593	7,864	20,361	0,428	0	257,143	0	33,4964	1,789
5,223	1,71	1,198	0,551	4,5	162,857	4,5	19,2612	1,143
2,846	2,93	5,141	0,551	0	342,857	4,5	110,4292	0,066
3,425	3,51	15,143	0,428	1	497,143	4,5	26,88	5,289
5,3	1,636	2,075	0,428	1	248,571	4,5	30,3212	1,789
3,568	3,005	13,207	0,066	0,143	428,571	0	413,224	6,143
1,71	2,367	0,143	0,428	0,429	171,429	1	9,408	1,789
3,634	1,79	5,007	0,428	0	480	4,5	93,24	1,143
4,926	3,565	2,51	0,428	4,5	197,143	0,789	93,296	5,289
9,077	3,728	4,361	0,786	4,5	291,429	4,5	191,6712	4,643
3,434	1,504	1,075	0,856	4,5	377,143	1	330,008	0,143
2,131	3,071	8,932	0	0	222,857	4,5	780,024	6,789
11,738	5,995	2,427	0,428	1	120	1	13,7088	0,789
4,062	1,075	2,429	0,428	0	180	0	42,5292	0,143
4,66	6,364	10,141	0,123	0	197,143	0,066	55,944	4,643
5,793	3,354	2,576	0	1,066	617,143	0,789	189,2464	1,066
5,359	3,007	0	0,123	1,789	103,571	4,5	0	1,789
10,086	6,364	0,715	0,428	1	248,571	1	51,282	0
4,404	3,431	5,987	0,428	1	222,857	4,5	613,9504	0,789
3,84	1,647	6,352	0,786	0,066	240	4,5	37,296	0,143
1,987	1,71	20,286	0	0,789	120	0	169,176	0,143
7,332	1,064	2,218	0,786	6	274,286	0,789	38,136	0,066
11,086	3,299	5,299	0,428	0	411,429	1	30,66	5,289
5,131	0,209	0,209	0,428	0	480	0	0	0,143
7,741	2,642	6,855	0,123	0,143	120	4,5	32,69	1,143
3,854	2,721	6,787	0,786	0,143	274,286	4,5	46,62	0,789
14,28	0,407	3,275	0,428	4,5	420	4,5	199,6456	6,066

3,708	4,365	4,939	0,428	0	94,2857	0	46,62	4,643
6,788	3,442	4,51	0	0	94,2857	4,5	204,512	4,643
5,718	6,287	6,141	0,786	0	257,143	4,5	11,6592	0,143
5,434	2,15	11,218	0,786	4,5	231,429	4,5	74,592	6,143
4,7	3,299	9,644	0	1	480	6	74,592	5,289
3,335	1,776	0,855	0,428	0	608,571	4,5	4,662	0,143
4,432	2,425	4,225	0,123	1,066	480	1	138,0092	1,143
5,847	2,565	3,653	0,428	0	162,857	0,789	66,3124	1,578
6,726	1,207	3,433	0,246	1,066	282,857	0,066	0	1,218
4,48	2,631	3,721	0	0	411,429	4,5	10,2144	4,643
4,919	1,921	3,998	0,428	0	205,714	0	23,058	4,643
6,706	1,141	1	0,852	4,5	77,1429	1	555,2932	0,789
7,021	2,721	2,275	0,428	4,5	565,714	1	93,24	4,643
5,432	2,216	1,141	0,786	0,066	120	4,5	33,3816	0,789
7,012	2,708	4,796	0,428	4,5	334,286	1	2,7972	5,289
5,58	1,787	2,066	0,428	0,143	300	4,5	233,24	4,643
4,926	2,51	3,075	0,123	0	480	4,5	263,34	0,143
4,05	1,416	6,198	0,428	4,566	180	1	30,058	4,643
8,305	3,365	3,579	0,428	4,5	420	4,5	36,792	5,289
2,623	1,559	4,275	0,428	0,429	240	4,5	22,414	0,789
12,052	11,499	11,073	0	0,932	274,286	4,5	150,01	6,789
6,139	1,996	3,053	0,428	0,066	180	1	58,31	1,143
5,06	2,216	12,566	0,428	0,789	60	0	46,648	0,143
7,658	4,576	6,576	0,428	4,5	282,857	6	228,0012	4,643
5,581	1,853	5,064	0,674	0	462,857	4,5	23,5872	5,289
3,497	1,218	1,143	0,123	0	402,857	0,789	14,4396	0,132
5,574	1,13	3,51	0,428	0	360	1	30,3072	0
3,214	1,13	0,998	0,551	0,789	300	1	50,6492	4,643
4,78	1,787	1,141	0,428	0,429	162,857	0,143	46,648	0,143
7,468	0,484	7,075	0,428	0	77,1429	1	60,004	1,789
18,943	3,998	0,275	2,5	0,132	360	1	138,1604	0,066
7,049	5,927	6,638	1	0,429	317,143	4,5	65,982	6,143
6,078	1,218	3,286	0,428	1	300	1	244,678	0,143
6,513	2,653	6,284	0,428	0	240	4,5	63,4592	1,143
11,234	2,367	2	0,123	0	111,429	1	3,066	4,643
6,468	1,853	0,429	0,066	0	145,714	0	16,926	1,066
5,586	2,998	0,987	0,786	1,578	300	0,143	43,442	0,066
3,186	0,275	1,275	0,428	0	342,857	1	9,324	6,143
7,78	2,436	0,704	0,123	0	308,571	0	150,3012	6,143
7,449	2,361	0,209	0,123	0	360	4,5	13,986	4,643
2,939	2,367	12,209	0,123	0	394,286	4,5	41,2622	4,643
3,478	1,339	2,418	0,428	0	180	1	15,414	6,143
10,718	5,432	9,493	0,066	0,789	68,5714	0	0	0,932
10,431	1,35	0,789	0,246	0	548,571	1	45,9144	0,066
5,727	6,221	6,644	1	1,066	428,571	1	45,7212	1,218
6,939	2,141	2,286	0,428	0	300	0	66,136	0,789
1,647	1,998	1,143	0	0	420	0	9,0552	0,143
6,289	1,416	0,132	0,856	0,429	197,143	4,5	47,964	6,789

7,334	0,55	6,198	0,428	0	180	1	54,9892	0,143
2,139	1,416	12,198	0,428	0	420	4,5	23,31	4,643
7,794	3,998	7,432	0,428	4,5	274,286	4,5	69,972	0
3,172	1,559	6,632	0,428	0	325,714	1	95,368	1,789
6,048	2,719	4,721	0,428	0,143	282,857	4,5	60,662	6,143
2,851	1,721	0,855	0,123	0	120	0	0	6,066
21,366	4,644	3,222	0,856	4,643	102,857	0,789	199,9732	0,932
4,12	1,427	5,498	0	1	205,714	4,5	42,4032	0,572
3,904	1,13	0,572	0,856	0,066	600	4,5	12,726	1,578
3,851	1,275	16,495	0	0,789	505,714	0	607,033	5,289
11,36	9,987	7,293	0,428	0,066	257,143	0,789	140	6,789
10,387	1,787	2,075	0,428	0	171,429	4,5	2,688	0,143
2,35	4,918	7,79	0,123	0	197,143	4,5	0	6,143
7,732	0,921	6,71	0,428	0,789	180	0,143	9,1896	6,789
1,757	1,427	5,218	0,786	0,789	300	4,5	60,004	4,643
4,696	1,853	4,998	0	4,5	514,286	0	49,0392	4,643
7,917	1,495	0,209	0,428	1	394,286	0	46,2392	4,643
4,072	2,075	7,721	0,786	0	120	0	30,66	4,643
3,909	1,493	2,627	0,428	0,429	257,143	4,5	36,8844	0,066
7,771	0	10,5	0,132	1	102,857	4,5	0	1
7,287	1,352	1,921	0	4,5	531,429	1	54,0036	0,789
5,21	1,418	3,998	0,786	6	445,714	0,789	34,8012	4,643
5,224	2,367	6,064	0,428	0	222,857	4,5	233,24	5,289
7,629	3,352	7,144	0,786	1	180	4,5	171,4412	1,143
8,998	3,719	0,352	0	1,143	222,857	0	0	1,578
7,363	2,842	12,007	0,551	0,789	351,429	4,5	300,02	0,789
5,295	2,71	7,789	0,428	4,5	214,286	1	13,44	0,143
6,215	5,861	3,787	0,786	0	282,857	4,5	171,5812	5,289
1,955	1,559	1,064	0,066	0	342,857	4,5	4,662	4,643
5,278	1,996	1,075	0	0	360	0	33,1002	5,289
11,217	3,653	4,341	1,428	0	222,857	0	83,762	0,789
5,374	1,932	0,789	0,428	0	265,714	1	8,3916	1,789
5,009	2,51	4,132	0,786	1	197,143	1	42,5936	0,143
3,51	0,275	3,721	0,428	0	240	0	20,4372	4,643
3,395	0,932	0,998	0,123	0,789	300	0	0	6,143
5,073	1,998	0,132	0,428	1	531,429	1	316,2544	6,143
3,904	1,636	0,484	0,123	0,143	214,286	0	35,322	0,143
6,947	2,073	3,075	0,428	0	480	0	102,438	1,143
3,269	1,919	6,576	0,428	1	480	1	206,612	4,643
3,537	1,787	9,998	0,786	1	197,143	4,5	30,66	0,789
2,689	2,216	4,71	0	0	720	0,143	72,982	6,066
4,555	0,99	7,427	0,428	0	154,286	1	48,398	1,143
2,186	1,71	5,275	0,123	4,5	300	4,5	91,322	4,643
6,021	2,51	2,644	0,428	1	377,143	0	91,98	0,932
2,12	5,641	8,442	0,786	0,143	360	6	97,958	0,143
6,117	3,354	1,141	0,428	0	180	1	31,9872	6,789
3,898	1,196	1,264	2,428	1	72,8571	0	44,3072	0,789
6,568	2,007	8,143	0,856	4,5	180	0	36,19	0,143

7,125	1,93	3,275	0,428	1,143	325,714	0,789	18,396	6,143
3,489	2,15	8,209	0,428	4,5	548,571	4,5	67,298	0,143
3,197	2,073	5,209	0,123	0	120	4,5	136,626	4,643
5,84	2,642	2,207	0	1	274,286	0	84,532	4,643
3,713	3,222	10,143	1	0,429	231,429	4,5	69,944	2
3,629	1,721	8,572	0,428	4,5	274,286	4,5	0	1,578
5,552	1,776	3,572	0,428	1	411,429	0	55,2972	1,578
1,463	2,576	5,644	0,066	0	394,286	4,5	35,322	0,143
7,082	2,007	2	0,428	0	154,286	1	60,004	0,143
5,555	2,565	0,418	1,428	0,789	154,286	0	66,0044	0,143
4,997	1,713	4,418	0,428	0,143	197,143	1	199,948	1,143
5,851	0,473	7,776	1	4,5	111,429	1	42,2324	1,789
3,841	3,005	5,218	0,066	0,066	480	4,5	123,298	5,289
3,349	3,431	0,693	0,428	0	471,429	1	206,612	6,143
9,932	2,642	8,939	0,428	1	240	0,789	85,946	1,578
4,005	1,132	1,578	0,428	1	68,5714	1	24,528	6,143
5,864	1,207	5,367	0,428	0	360	1	0	1,143
4,652	1,996	3,001	0,786	0	231,429	4,5	0	0,932
2,986	3,005	1,064	0,066	0	402,857	4,5	142,443	4,643
4,566	0,495	5,473	1,214	0,789	222,857	1	127,3244	0,932
2,051	1,262	2,921	0	4,5	120	0	27,216	4,643
8,42	1,93	6,495	0,123	4,566	385,714	4,5	16,926	0,143
11,161	2,433	1,075	0,428	0	197,143	4,5	18,396	0,932
7,946	0,572	2	0,428	0	94,2857	1	0	1,789
7,794	8,719	1,143	0	0,429	248,571	0,143	0	1,578
6,66	1,864	0,132	1	0	274,286	0,066	0	0,143
8,485	2,218	4,218	0,428	4,5	248,571	0,789	29,946	4,643
5,726	1,864	5	1	0	360	0,429	0	6
7,368	6,289	2,143	0,428	4,5	120	4,5	25,6452	5,289
2,568	1,79	2,493	0,066	4,5	360	0	740,04	6,143
8,102	3,442	10,286	0,428	4,5	317,143	0	2,688	4,643
6,651	3,431	3,075	0,428	0,789	113,571	4,5	38,066	4,643
2,623	1,35	2,638	0,786	4,5	351,429	0	246,435	5,289
4,927	2,205	3,71	0	4,5	180	1	29,946	0,789
4,41	2,708	7,776	0	4,5	394,286	1	60,662	5,289
3,434	1,119	2,066	0,123	1	205,714	0	51,296	0,932
3,348	1,921	1,855	0,246	0	214,286	0	14,784	5,289
4,783	1,93	1,341	0,066	0	368,571	0	19,9668	5,289
4,172	1,864	5,855	0	1	274,286	4,5	63,6832	0,789
2,785	6,33	4,921	0,123	0,855	377,143	4,5	19,572	0,143
5,508	1,418	5,855	0,123	1,066	274,286	0	10,752	0,143
7,364	3,499	11,299	2,5	0	282,857	4,5	20,4372	0,143
8	1,218	3,218	0,066	1	257,143	0,789	18,396	0,932
5,926	2,853	2,787	0,428	1	180	0	390,012	0,932
8,086	1,787	1,275	0	4,566	240	4,5	661,0156	6,789
4,924	7,722	5,921	0,428	4,5	300	1	108,0072	0,066
5,043	5,916	0,987	1,428	0,066	197,143	0	43,442	4,643
2,744	1,064	12,073	0,066	0	394,286	6	72,982	5,289

4,049	1,13	6,198	0,428	0,855	120	0,143	45,4342	4,566
4,71	0,789	5,71	0,189	1	240	0,066	0	4,566
4,649	5,204	4,132	0,123	0	428,571	1	29,19	0,143
5,638	3,431	5,284	0,428	1	300	1	50,4896	0,932
5,491	1,207	1,275	0,123	0	274,286	4,5	25,326	0,143
7,649	1,064	6,998	0,856	1	282,857	0,066	52,4832	1,143
4,585	2,15	1,075	0,428	0,143	137,143	4,5	76,622	0
5,695	2,93	6,418	0,428	1	15	0	128,282	1,143
13,432	2,282	2,361	0,551	0	180	4,5	171,5588	6,143
5,658	1,427	0,855	0,428	4,5	514,286	4,5	128,282	4,643
4,72	1,075	11,429	1	4,5	308,571	4,5	2,688	0,143
3,623	1,286	4,352	0,123	0	162,857	1	7,35	1,143
3,346	3,365	11,284	0	4,5	240	4,5	44,9204	5,289
8,206	2,216	0,561	0,856	1	471,429	0,066	46,2392	5,289
5,489	1,996	1,35	1	0,495	548,571	0	146,076	5,289
6,847	2,708	5,282	0,428	1	300	0,066	65,982	1,143
3,775	1,57	7,51	0,786	4,5	274,286	0,066	30,002	5,289
3,621	2,216	4,565	0,066	4,5	394,286	0,143	73,7884	0,143
2,109	6,43	6,418	0,428	4,5	514,286	1	18,648	6,143
5,078	1,864	2,33	0,428	4,5	240	1	38,136	4,643
5,138	2,576	6,638	0,856	0,066	154,286	4,5	109,4212	0,143
5,57	1,361	10,932	0,428	0	120	4,5	12,264	5,289
2,34	0,627	3,418	1	1	77,1429	0,789	310,66	5,289
12,748	2,499	6,341	0,909	0	162,857	0	34,5324	0,209
2,189	2,51	10,264	0,066	0	291,429	4,5	79,058	0,143
7,084	2,642	8,284	0,428	0,789	411,429	1	30,002	4,643
2,535	0,704	4,924	0,428	0	342,857	0	124,67	1,789
3,913	2,719	6,709	0,428	0	120	4,5	240,016	5,289
3,708	2,064	3,264	0,123	0	231,429	1	30,66	4,643
6,577	2,862	4,075	0,428	0	205,714	1	111,51	4,643
7,368	6,43	8,075	0,428	1	437,143	1	76,622	0,143
16,014	2,653	11,853	0,551	0	214,286	4,5	55,944	1,789
4,85	7,219	0,855	0	6	300	1	206,612	4,643
4,139	1,93	5,93	0,428	0	514,286	1	51,506	1,143
7,427	2,853	3,429	0,428	0	162,857	1	238,3682	1,578
4,855	2,282	3,218	0,428	0,789	274,286	1	38,542	4,643
6,944	1,787	0,998	0,123	1	325,714	1	3,066	1,143
3,12	2,493	8,473	0,428	0	300	1	66,794	6,143
3,979	1,064	5,141	0,428	1	300	1	33,726	5,289
5,509	1,919	7,864	0,428	0	162,857	1	53,718	1,143
5,284	1,141	7,064	0,123	4,5	377,143	1	116,872	6,143
7,226	1,13	1,209	0,123	1,143	377,143	1	27,972	0,143
5,286	1,57	2,143	0,428	1	17,1429	0	18,648	1,789
4,291	6,65	6,787	1	0,789	128,571	4,5	140	5,289
6,438	5,85	2,644	0,428	0	214,286	0,789	26,4096	1,789
4,291	1,493	5,853	0,428	0	214,286	6	0	4,643
6,86	3,071	6,576	0,428	0	411,429	1	284,2336	5,289
5,99	2,218	7,932	1	4,5	60	0	176,624	5,289

3,713	2,007	10,787	0,428	0,932	317,143	4,5	186,62	5,289
5,289	1,273	1,93	0,551	4,5	300	0,143	99,6352	0,143
6,233	2,499	1,998	0,428	0	240	6	126,784	1,143
4,404	1,998	6,143	0	4,643	411,429	0,789	122,8052	0,143
5,26	2,205	1,209	0,428	1	497,143	1	51,1952	0,143
4,069	3,433	9,141	0,123	4,5	531,429	1	28,7532	5,289
7,15	1,064	11,141	0,428	0,066	154,286	4,5	139,944	1,143
4,927	1,361	2,075	0,428	1	137,143	0,143	57,0276	1,143
7,425	2,642	2,275	0,428	4,566	437,143	1	53,9952	0,143
3,97	1,218	1,644	0,369	0	214,286	4,5	43,1942	1,789
6,869	1,702	6,433	0,786	4,5	214,286	4,5	44,1266	0,789
6,921	9,855	1	0,428	0,143	222,857	0,429	143,5588	0
2,848	1,93	1,572	0,123	4,5	360	6	0	0,143
13,08	6,867	0,932	0,428	0	325,714	6	86,6376	0
7,409	2,644	6,073	0	0,495	197,143	0,429	128,226	0
8,385	5,523	1,429	0,786	0	162,857	4,5	0	5,289
7,882	4,088	2,864	0,428	0	214,286	4,5	179,984	1,578
4,005	1,273	0,275	0,856	0	342,857	4,5	54,9304	0,789
4,058	2,576	2,275	0,066	0,143	514,286	0,066	403,242	6,143
11,949	3,299	3,572	0,428	0	334,286	0,789	138,0092	4,643
5,337	2,998	5,932	0	0	120	1	89,992	0,143
2,835	1,932	3,578	1,123	0	428,571	1	28,5964	5,289
4,495	1,996	4,864	0,428	5,289	214,286	0	224	5,289
4,129	6,564	0,132	0,123	0	282,857	4,5	300,02	0,132
6,155	1,57	2,79	0,428	0	617,143	0	29,19	4,643
4,7	2,565	3,207	0,428	0,066	154,286	1	360,024	6,143
10,006	3,148	1,561	0,428	0	274,286	4,5	128,282	1,789
4,201	1,561	3,504	0,428	0,066	402,857	4,5	11,8776	0,789
6,163	3,216	10,198	0,428	4,5	257,143	0,066	46,62	4,643
6,163	1,932	1,787	0,428	4,5	154,286	4,5	60,662	4,643
5,215	2,062	3,578	0,428	0	240	4,5	106,624	0,855
6,497	2,207	2,429	0,428	1,066	308,571	1	30,002	1,789
8,915	7,153	10,987	0	0,066	480	0	46,62	0,789
2,983	0,921	0,352	0,123	4,5	445,714	4,5	26,166	0,066
5,845	2,71	12,066	0,428	0,143	171,429	4,5	11,662	6,789
3,481	1,273	3,139	0	0	120	1	273,3766	4,643
5,482	6,784	1,218	0,428	0,066	325,714	4,5	13,5492	5,289
4,355	0,77	2,55	0,066	0	531,429	4,5	88,312	0,789
6,221	2,721	8,367	0	0	394,286	0	62,5016	4,643
3,065	1,998	4,99	0	0,143	222,857	0,066	182,924	4,643
3,206	1,352	14,498	0,123	1	274,286	4,5	35,5964	6,066
3,86	0,825	6,418	0,786	0	334,286	0,066	76,132	5,289
5,199	1,908	1,919	0,428	0,143	625,714	0,066	147,4284	0,143
5,574	2,007	3,156	0,428	0,789	257,143	1	96,9892	5,289
7,351	1,996	15	0	0	154,286	0	359,968	0,066
5,515	2,785	4,407	0,856	0	360	1	228,4436	5,289
15,944	1,196	4,218	0,428	4,5	94,2857	1	0	1,789
6,216	3,222	4,284	0,428	0,066	214,286	0	54,53	1,143

5,852	7,296	5,575	0,786	0	582,857	4,5	120,008	1,218
3,416	2,064	1,143	0,123	1	600	1	102,9994	0,789
8,206	7,578	0,286	0,786	0	180	1	300,02	0,789
2,637	2,719	4,275	0,428	0	317,143	1	148,064	0,789
4,704	1,985	4,864	0,786	4,5	188,571	0,789	17,794	1
5,849	2,216	10,55	0,428	1,066	411,429	0,143	85,9642	1,143
2,559	0,275	0,286	0,123	4,5	600	1	6,132	0,143
3,842	1,196	17,499	0,786	1	300	4,5	240,016	0,789
5,438	5,641	0,429	0,428	0	428,571	6	0	0,572
6,288	2,51	0,286	0,428	0	257,143	0,143	0	1,789
5,77	1,493	7,264	0,428	0	257,143	0,143	61,32	6,143
5,707	1,721	1,284	0,428	0	214,286	0	141,2432	1,143
8,086	2,644	5,919	0,494	1	231,429	0,789	31,1164	0,789
9,177	0,352	4,721	0,428	0	480	0	2,688	0,789
3,5	3,717	0,264	0,066	0	360	0	4,662	6,143
3,5	0,921	2,066	0	0	72,8571	0,789	5,334	1,578
4,715	1,787	3,361	0,246	4,5	197,143	1	20,8488	1,578
4,64	2,367	8,275	0,066	1,066	171,429	1	8,9292	4,643
7,296	2,576	8,352	1	0,143	171,429	1	18,102	4,643
5,412	0,836	0,932	0,123	1,066	282,857	4,5	73,444	0,789
2,689	2,367	18,209	0,428	0	240	4,5	299,075	0,789
5,21	2,139	1,853	0,786	0	222,857	0,789	38,3964	0,143
9,574	2,93	1,921	1	4,5	222,857	1	83,6192	0,789
5,867	1,933	4,13	0,428	1,143	282,857	0,789	44,1616	4,643
2,997	2,064	5,998	0,786	4,5	317,143	4,5	89,992	4,643
2,678	5,138	5,921	0,123	4,5	428,571	4,5	0	0,143
4,414	2,367	4,066	0,428	1	68,5714	1	107,968	6,143
5,772	2,994	3,442	1	0	308,571	4,5	0	1,578
10,514	1,209	3,143	0,786	0	497,143	4,5	0	0,789
7,741	1,998	2,143	0	1	308,571	1	14,4522	1,143
6,375	2,561	14,073	0,428	0	214,286	4,5	51,506	1,143
1,77	1,93	2,066	0,428	0	197,143	4,5	380,016	0,143
5,142	1,275	5,407	0,123	1	514,286	0	37,296	6,789
4,577	5,498	5,644	0,428	4,5	531,429	0	23,926	6,789
4,194	7,439	1,578	0,428	0,789	60	4,5	0	0,143
4,28	4,841	1,495	0	1	325,714	0,066	58,282	4,566
7,655	3,156	1,361	0,428	0	171,429	0	89,992	0,572
8,835	1,35	9,156	0	0,495	154,286	0,066	30,303	5,289
2,746	2,139	12,55	0	0,789	514,286	1	22,5232	5,289
5,504	1,93	6,484	0,428	1	180	1	36,1872	0,932
4,258	6,355	4,484	0,786	0,066	308,571	1	120,652	0,789
1,823	1,787	0,341	0,428	1	77,1429	4,5	8,8704	0,932
6,63	1,495	5,572	0,786	1	162,857	1	0	4,643
2,975	1,218	6,418	0,246	1	334,286	FAŁSZ	0	5,289
2,403	4,077	4,561	0,428	0,789	197,143	1	56,3696	4,643
7,513	1,987	6,216	0,428	0,066	77,1429	1	18,648	0,572
2,549	1,133	2	0,786	0	308,571	4,5	114,471	0,143
3,929	3,299	7,361	0,428	1,578	102,857	4,5	26,88	1,789

5,715	0,407	0,209	0,428	1	360	1	1,9908	0,855
6,441	1,996	4,064	0,123	0,066	377,143	1	44,7552	1,066
3,052	1,056	3,275	0,786	0	274,286	0	22,638	0,855
4,935	2,565	0,132	1,214	0	282,857	4,5	145,5384	4,929
9,437	1,493	6,198	0,123	0,429	240	4,5	19,7232	5,289
9,785	2,13	6,066	0,786	0	274,286	1	13,44	5,289
4,063	1,342	4,484	2,5	1,066	120	1	144,1762	4,643
9,587	2,576	1,209	0,428	0	420	0	133,3024	4,643
4,858	1,13	9,932	1,066	4,5	282,857	4,5	305,5864	5,289
4,139	1,636	4,218	0,123	6	600	1	94,0464	5,289
5,35	1,93	6,704	0,428	4,566	214,286	4,5	86,884	4,643
7,005	4,433	7,864	0,428	0,789	257,143	1	108,388	4,643
6,368	1,572	2,572	0,428	0	342,857	1	60,004	2
5,575	1,932	7,35	0,428	0,789	394,286	1	32,69	4,643
5,803	2,719	8,576	0,428	4,566	265,714	0	38,1752	1,789
15,005	4,651	3,064	0,428	0,143	162,857	1	95,6004	0,789
1,606	1,286	3,075	0,123	1	462,857	1	19,222	6,143
4,564	1,57	7,141	0,494	0,143	300	4,5	18,2112	5,289
3,566	2,367	5,352	0,551	4,5	162,857	0,066	72,926	0,789
3,568	1,985	2,141	0,428	0	240	1	43,1872	4,643
4,133	2,216	10,715	0,428	0	120	1	240,016	6,789
2,834	1,207	0,407	0,066	4,5	291,429	0	440,02	0,143
7,095	2,073	2,572	1,428	0	107,143	1	0	1,143
5,138	1,273	0,286	0,786	6	480	0,143	19,3704	0,789
5,146	2,51	2,578	0,428	1	300	1	11,662	5,289
5,863	1,13	0,627	0,189	1,066	480	0,143	21,462	0,572
8,345	2,939	1,924	0,786	4,566	188,571	4,5	294,0196	0,789
5,792	2,433	7,284	0,123	4,5	334,286	0,143	0	0,143
10,485	1,787	3,143	0,428	4,5	514,286	0	116,872	0,143
2,535	1,864	14,073	2,928	1	377,143	4,5	23,4486	1,066
4,08	2,653	3,644	0,123	0	222,857	1	14,784	0,143
3,423	1,71	6,632	0,428	0	377,143	0	51,7104	0,572
4,99	2,13	14,407	0,428	0,066	197,143	1	216,594	5,289
4,058	0	2,998	0,428	6	368,571	0,066	26,7372	5,289
5,645	2,359	7,93	0,428	1,789	180	4,5	273,224	5,289
6,728	1,053	1,075	0,428	0	162,857	0,143	204,2796	0,789
5,581	1,787	3,71	0,123	1	171,429	0,066	0	4,643
3,403	1,787	0,484	0,428	4,5	342,857	0,429	17,2536	0,932
6,559	0,638	2,275	0,428	1,066	120	1	39,452	1,066
6,152	2,644	1,998	0,428	0	497,143	4,5	4,9056	1,143
2,493	0,987	2,704	0,123	1	368,571	4,5	3,1752	0,066
9,492	2,062	0,572	0,428	0	411,429	4,5	0	6,143
12,878	2,007	5,141	0,428	0,066	222,857	1	48,0032	0,932
6,853	2,499	4,484	0,066	0,066	154,286	4,5	18,6592	1,143
4,966	0,407	6,007	0,979	0,143	162,857	1	34,5324	0,572
4,146	2,007	3,007	0	0,143	137,143	0	153,1516	0,143
3,28	1,787	3,286	0,123	4,5	282,857	4,5	20,79	6,143
3,638	2,51	0,143	0,786	0	274,286	1	165,3064	0,932

5,708	0,407	0,789	0,123	0	257,143	0	46,2392	0,143
6,08	0,704	1,561	0,909	0,789	480	4,5	82,6504	0,066
3,78	0,77	0,066	0,123	4,5	420	1	37,296	5,289
3,647	2,209	2,998	0,123	0	171,429	4,5	12,096	6,143
2,924	2,064	0,275	0,123	4,5	137,143	4,5	618,688	4,643
2,604	2,864	3,638	4,5	0,495	265,714	6	20,986	1
14,502	0,627	0,998	0,123	0	137,143	0	99,372	0,789
7,588	0,484	4,728	0,428	0	480	1	34,3392	5,289
4,471	1,636	6,352	0,428	0	557,143	1	0	0,143
5,157	2,367	9,932	0,428	4,5	325,714	4,5	24,528	4,643
3,995	1,787	11,209	0,428	4,566	162,857	1	0	5,289
6,293	1,001	3,218	0,428	0,143	240	4,5	1211,742	4,643
1,603	1,919	1,064	0,428	0	462,857	1	9,324	1,066
20,928	1,919	6,932	0,786	0	128,571	0	6,72	5,289
4,689	2,785	3,482	0	0	197,143	0	0	1,789
6,299	4,156	4,367	0,428	0,789	514,286	0	240,016	1,218
4,993	1,996	1,644	1,642	0,495	214,286	0,789	37,296	6,066
3,852	2,141	1,853	0,123	0	257,143	0,143	74,592	0,932
3,34	1,427	0,998	0,786	1	394,286	0	19,7148	1,789
3,327	2,062	5,216	0	1	600	4,5	123,3204	4,643
9,22	2,502	11,156	0	0	180	0	2,1504	0,789
6,99	0,638	2,429	0,428	0	351,429	0	120,008	0,932
13,157	1,864	3,721	0,123	1	248,571	0	82,6224	1,143
4,206	5,852	7,79	0,428	0	171,429	4,5	182,924	0,789
3,91	2,216	12,218	0,428	4,5	720	4,5	85,3384	1,578
3,852	2,293	7,787	0,066	0,429	420	0	46,62	0,789
4,931	1,493	0,932	0,428	4,5	102,857	4,5	53,9784	0,143
1,232	1,702	8,341	0,123	0	300	4,5	146,622	6,143
7,717	1,273	4,207	0	4,566	90	1	59,6792	5,289
5,572	2,216	3,075	0,428	4,566	240	0	36,134	1,789
2,913	0,066	0,789	0,123	1	180	0	36,0024	4,643
5,644	3,585	6,218	1,428	0,143	180	1	265,0816	5,289
5,289	2,064	9,578	0	0,066	445,714	1	136,612	6,789
4,852	1,484	2,561	0,428	0	240	1	0	1,143
6,146	1,842	2,864	0,428	0	214,286	1	15,5232	5,289
6,363	1,71	0,921	0,428	1	0	4,5	89,992	0,066
3,625	2,075	1,998	0,428	1	480	1	53,718	6,143
6,561	1,504	7,141	0,856	0,066	222,857	0,066	29,946	0,066
3,911	1,13	0,921	0	0	162,857	0	10,2732	0,066
8,387	2,15	2,141	0,428	1	85,7143	1	11,662	0,789
4,917	1,787	2,484	0,428	0	351,429	0	263,242	0,855
2,535	0,913	3,484	0,066	0,789	274,286	4,5	99,974	1,143
2,689	2,433	13,495	0,066	0	377,143	1	37,562	4,643
3,436	2,141	5,367	0,428	0	411,429	0	153,44	4,643
4,548	2,996	14,644	0,428	4,5	351,429	1	78,4	4,643
6,869	2,15	11,218	0,786	6	531,429	0	14,9184	5,289
7,513	1,57	6,715	0,428	1,143	385,714	0,789	249,3932	0,932
5,414	0,627	1,275	0,123	4,5	240	0,789	16,128	0,143

12,518	1,776	1,998	0,551	0	480	4,5	11,0376	0,143
2,864	1,141	1,578	1	0	377,143	0,789	0	0
6,227	2,073	2,638	0,428	0	565,714	1	11,662	1,143
11,468	5,945	11,299	0,428	1	257,143	0	6,1824	1,789
3,706	3,945	2,275	0,428	4,5	240	0	128,282	1,143
6,719	1,787	3,064	0,428	0	180	0	64,9712	1,143
5,278	2,928	9,932	0,428	0,066	120	4,5	244,902	1,143
3,755	5,63	4,931	0	0,066	471,429	4,5	71,778	0,066
5,581	2,007	8,572	0,428	0,143	257,143	4,5	0	4,643
7,982	3,499	4,418	0	4,5	222,857	0	10,752	0,143
7,997	1,207	0,561	2	0	411,429	4,5	58,7412	0,209
7,089	1,001	0,352	0,786	0,789	240	0	0	4,643
5,726	3,148	4,998	0,428	0	462,857	0	35,322	0,789
14,794	2,776	5,864	0,428	4,5	257,143	0	61,32	0,789
2,777	6,287	11,064	1	0	274,286	4,5	106,624	6
5,798	2,796	1,715	0,428	0	274,286	0	5,376	0,789
7,78	1,644	0,855	0,428	0	145,714	0	13,9944	0,143
6,216	1,218	3,075	0,856	1	60	0	32,69	1,143
4,415	2,007	5,13	0,428	0,143	154,286	1	18,396	4,643
4,289	1,067	6,576	0,428	1,143	428,571	0	9,324	4,5
7,816	3,945	3,007	0,428	0,429	360	4,5	29,19	5,289
8,918	3,433	3,209	0,428	0,932	120	4,5	152,264	6,789
3,708	0,847	1,132	0,428	0	540	1	38,066	4,566
1,768	0,932	0,921	0,428	0	488,571	0	0	0,789
4,988	1,416	0,341	0,428	0	282,857	0	0	0,932
8,94	2,51	3,504	0,428	0,789	180	0	23,324	0,789
6,736	3,728	4,367	0,428	0,143	274,286	1	76,622	4,643
5,783	2,139	5,007	0,428	0	72,8571	4,5	40,8632	5,289
8,161	0,55	2,218	0,428	4,5	137,143	0,789	13,1712	0,143
9,78	5,421	7,064	0,123	0	248,571	4,5	28,462	0,143
6,218	3,075	7,273	0,428	4,566	197,143	1	67,606	6,143
4,792	0,99	0,275	0,123	0	360	1	29,19	0,143
2,843	1,919	9,853	0,123	4,5	342,857	1	22,414	5,289
3,992	4,775	6,275	0,066	0	282,857	4,5	31,2732	4,643
4,417	4,852	0	0,551	0	214,286	4,5	0	4,5
2,755	1,559	4,209	0,428	4,566	360	4,5	0	5,289
9,571	5,63	4,864	0,786	4,5	120	1	174,93	6,143
2,988	0,143	4,132	0,428	0,429	154,286	6	0	0,789
11,081	3,275	4,075	0	1	428,571	1	520,016	6,789
4,569	8,493	1,284	1,214	0,495	197,143	4,5	108,157	4,643
2,406	1,35	0,987	0,123	0	531,429	1	153,244	0,143
4,347	1,493	2,561	0,428	0,789	131,429	0	36,764	1,143
4,423	2,939	7,787	0,786	0	351,429	1	104,902	5,289
7,376	2,721	0,495	0,428	0	137,143	4,5	210,7532	0,143
8,229	2,418	4,429	1	0	300	1	55,3364	1,143
4,911	0,275	1,921	0,123	1	565,714	0,143	23,5872	0,143
15,362	7,065	0,998	0,786	0	540	1	141,2992	0,143
2,26	2,576	7,207	0,428	4,566	60	0	153,5492	5,289

6,203	5,85	4,209	0,066	4,5	325,714	0	238,7392	4,643
1,977	1,075	3,429	0,428	0	214,286	0	41,958	1,578
4,86	2,141	3,864	0,428	0	197,143	4,5	272,8152	5,289
2,9	1,427	1,207	0,428	4,566	325,714	0	346,6274	0,209
3,272	2,853	4,561	0,123	0	180	1	41,5604	4,643
4,638	1,284	6,998	0,428	4,5	257,143	4,5	91,322	6,789
12,573	2,51	1,495	0,123	0	0	1	6,132	0,932
12,03	0,418	1,067	0,428	0	334,286	0	46,7544	0,932
15,997	5,05	0,407	2	0	317,143	4,5	32,6256	0,066
5,137	2,576	0,132	0,123	0	265,714	0,143	360,024	0,143
4,126	3,071	2,776	0,123	0	617,143	1	361,368	0,143
2,331	5,641	1,198	0,428	1,143	471,429	0,789	334,6252	4,643
17,153	2,433	0,789	1	0	222,857	0	38,542	0
3,486	1,053	0,132	0	0,066	377,143	0,789	24,3418	4,566
3,623	2,216	7,698	0,428	0	180	0,789	256,62	5,289
3,186	2,996	5,789	1	0	480	0	42,5936	1,789
3,637	5,487	4,93	0,123	0,789	137,143	0,789	62,6892	0,855
3,401	1,339	1,853	0	0,789	325,714	0,789	18,102	0,066
6,523	2,348	2,341	1,214	0	154,286	4,5	47,7764	6,789
4,86	2,367	2,075	0,428	0	454,286	1	0	0
2,755	2,642	6,787	0,428	0,143	274,286	4,5	123,242	0,789
3,746	1,284	0,275	0	1	420	4,5	150,1304	4,566
2,865	2,367	0,198	0,123	0	205,714	4,5	18,648	1,218
5,629	4,83	3,064	0,428	0	205,714	4,5	39,6872	5,289
5,993	5,564	7,722	0,123	4,5	240	1	0,756	0,143
1,988	2,007	0,341	0,428	0	394,286	1	61,4684	5,289
4,137	0,352	1,721	0,123	1	480	0	0	0,143
6,79	2,642	8,418	0,786	6	205,714	4,5	52,5112	6,066
2,535	0,902	0,264	0,123	0	291,429	1	137,956	0,066
7,217	5,63	3,218	1,214	0	257,143	0,789	36,764	5,289
4,054	2,642	3,13	1,123	1	437,143	4,5	195,79	1,578
5,287	2,576	11,284	0,428	0,066	214,286	1	46,62	1,143
6,289	2,576	5,864	0,428	1	617,143	0,066	53,6284	0,789
4,203	1,416	8,787	0,428	0	385,714	1	39,3484	0,789
3,415	2,93	0,921	1	1	145,714	1	38,542	0,572
4,212	0,264	8,921	0,066	4,5	291,429	1	104,958	6,789
1,834	1,339	3,998	0,428	0	180	0	0	5,289
2,912	2,853	0,921	0,786	0	428,571	0	147,4284	6,143
5,083	2,864	5,575	0,428	1,789	385,714	0	0	4,5
1,9	1,996	4,218	0,066	0,066	360	0,789	323,232	0,789
3,906	1,985	2,932	0,786	0	240	0,066	57,974	0,932
4,915	1,273	2,776	0,428	1	137,143	4,5	11,662	6,066
3,054	2,139	1,715	0,123	4,5	377,143	1	24,7324	5,289
3,983	0,987	1,578	0,123	0	445,714	1	99,7136	0,789
4,423	2,139	1,721	0	0,066	308,571	0,066	123,242	4,643
5,584	4,088	6,921	0,428	4,5	428,571	0	30,66	0,066
1,528	1,71	0,143	0,123	0	291,429	0,066	57,3272	0,789
5,199	1,484	5,35	1	4,5	582,857	0,789	76,622	6,143

5,148	2,776	6,198	0,428	0	540	1	141,89	5,289
4,861	1,559	2,064	0	0,066	222,857	1	75,1968	1,789
3,56	1,416	3,073	2,5	0	180	0,143	279,944	1,143
3,709	2,636	2,653	0,123	0	445,714	1	440,02	6,143
16,137	0	1,066	2	0,789	282,857	0,789	42,2268	0,789
5,63	1,93	1,644	0,428	0	265,714	4,5	47,3172	0,143
6,216	2,499	5,93	0,428	1	154,286	0	55,3952	1,143
7,585	3,075	4,141	1,856	0	231,429	4,5	85,946	4,643
9,17	0,998	9,284	0,428	1	231,429	4,5	20,9832	1,789
3,566	2,565	7,495	0,856	4,5	214,286	0	22,26	1,578
8,653	5,011	10,864	1	0,429	120	1	48,0032	6,789
6,592	3,431	6,495	0,123	0,789	77,1429	4,5	17,038	0,789
7,57	1,275	0,198	2,214	0	394,286	0	224,6132	6
5,859	1,493	2,855	0,428	4,5	120	4,5	87,2004	4,643
7,95	3,719	5,93	0,123	1,066	180	4,5	0	1,789
4,858	0,847	0,495	0	0	565,714	4,5	26,7876	0,789
5,704	3,075	3,209	0,123	1	162,857	1	40,754	4,643
3,274	2,207	6,418	0,494	0,789	274,286	4,5	21,4116	6,789
5,616	2,495	5,075	0,123	4,5	471,429	1	46,62	1,143
6,08	2,653	13,852	0	4,5	162,857	1	81,6312	0,932
3,766	1,339	0,132	0,428	0	180	0,066	10,752	0,143
3,06	3,508	4	0,123	0,789	300	6	84,644	6,789
2,711	1,853	6,93	0,428	0	531,429	0	12,2304	0,789
5,495	2,862	7,484	0,428	4,5	257,143	1	273,224	1,789
4,479	2,359	0,352	0,786	0,066	274,286	1	93,653	0,789
5,35	2,565	2,367	0,428	0	154,286	1	42,322	6,143
7,212	1,35	1,064	1	0	480	0	25,6452	0,143
8,631	6,221	14,209	0	0	300	1	26,166	6,066
3,401	5,784	2,13	0,428	6	274,286	1	237,902	6,143
4,847	2,073	3,484	0,428	1	154,286	0	190,9376	5,289
4,579	2,216	4,51	0,428	4,5	171,429	0	61,32	4,643
12,199	6,498	2,429	1	0	154,286	0	85,2992	6,143
4,403	0,55	5,787	2,928	0,789	137,143	1	65,296	1,143
2,623	1,787	2,209	0,123	0	77,1429	4,5	153,44	0,143
5,284	1,57	5,075	0,428	0	317,143	0	53,0376	0,143
10,147	0	0,209	0,066	0	300	0	0	6,789
4,563	4,088	0,341	0,428	1	180	4,5	309,89	0,789
5,937	2,073	3,578	0	0,789	55,7143	1	32,004	4,643
5,273	0,572	4,141	0,428	1	162,857	1	67,606	1,143
2,986	1,482	5,367	0,428	6,066	240	0,789	136,612	6,143
5,42	2,141	3,132	0	4,5	274,286	4,5	9,324	4,643
5,994	2,842	6,275	0,428	1	94,2857	1	12,264	0,143
5,57	1,93	1,787	0	0	120	0,143	182,644	1,143
6,768	1,286	1,638	0,428	0	197,143	0	12,9696	0,572
7,23	3,787	4,853	0,428	4,5	411,429	0,789	155,2852	0,932
17,725	5,773	13,361	0	0,789	334,286	1	141,8452	1,143
4,28	1,066	2,209	0,428	1	171,429	1	20,9244	0,143
6,504	2,139	0,132	0,428	0,066	428,571	4,5	66,0044	0,143

4,484	2,216	6,132	0,428	0,066	300	1	153,44	4,643
8,4	0,924	4,704	0,786	0	428,571	0,429	130,7922	0,429
3,469	2,073	0,066	0,123	0,286	308,571	1	31,0296	4,566
14,119	1,853	6,559	0,856	4,5	462,857	4,5	182,6244	4,566
6,557	0,264	1,264	0,123	4,5	60	1	28,7532	0,143
3,403	1,141	3,853	0,066	0	514,286	0	61,32	5,289
10,654	1,787	11,367	0,428	4,566	265,714	4,5	186,48	0,789
5,852	2,653	3,638	0,428	0,066	111,429	4,5	151,312	0,143
3,559	2,209	9,073	0,428	0	94,2857	1	26,88	1,143
1,493	1,119	8,367	0,428	0,789	102,857	1	373,24	5,289
6,79	2,216	4,132	0,428	0,429	334,286	1	31,038	0,789
5,08	0,921	1,209	0,246	1	257,143	4,5	76,132	0,066
4,992	6,507	3,721	1	5,289	214,286	0	0	5,289
5,427	0,855	9,207	0,428	0	325,714	0	37,3072	0,143
7,076	1,93	4,053	0,428	1,143	197,143	4,5	64,6212	0,143
5,449	1,339	8,787	0,428	0,143	514,286	0,789	93,24	5,289
4,866	2,653	5,51	0	1,578	257,143	1	43,442	4,643
3,728	1,352	4,51	0	0	377,143	0	140	1,789
12,702	1,418	4,064	0,786	0	137,143	1	45,773	0,572
5,443	3,365	2,284	0,428	0	214,286	0,789	105,5684	5,289
3,836	2,284	4,066	0	0	557,143	1	0	6,066
3,489	0,781	3,77	0,066	1,218	394,286	0,066	539,952	1,789
3,203	1,493	3,998	0,066	0,066	214,286	0	0	4,643
3,115	2,708	9,264	0,428	4,5	77,1429	4,5	362,8212	5,289
4,563	3,51	8,63	0,428	1	68,5714	1	0	1,429
5,416	1,427	5,787	0,123	0	274,286	1	46,4408	1,578
4,997	1,721	1,776	0,123	1	317,143	0,066	34,1208	0,066
4,627	3,921	2,921	0,123	0,789	240	1	61,4684	5,289
12,151	4,299	5,93	0,786	1	351,429	0	450,03	4,643
9,319	2,35	1,932	0,428	0	102,857	4,5	11,3442	1,789
5,861	2,139	4,998	1,214	1	180	0,429	58,4164	4,643
4,768	2,853	4,653	1	4,5	377,143	0	201,32	4,566
7,374	1,864	0,286	1,214	0	608,571	4,5	98,8344	0,066
6,325	1,207	6,495	0	0,066	480	1	38,542	6,143
11,58	2,073	1,275	1	4,643	360	0	35,378	0,495
4,069	4,088	8,927	0,786	4,566	428,571	1	46,62	5,289
7,137	1,262	4,715	0,786	4,5	265,714	4,5	144,606	6,143
4,058	4,643	0,855	0,066	0	651,429	0,066	60,5612	0,143
4,203	1,636	11,853	0	1	197,143	4,5	4,662	0,789
6,873	2,719	5,713	0	4,566	102,857	4,5	100,758	0,789
3,896	6,353	4,565	0	0	685,714	1	76,622	0,143
15,286	0,715	1,075	2,5	0	180	FAŁSZ	96,0064	0,789
5,645	3,576	8,073	0,428	0	214,286	1	54,1072	5,289
7,453	2,433	1,789	0,428	0	240	0,429	330,022	5,289
4,412	5,85	5,198	1	1	274,286	1	13,986	1,218
3,412	1,504	2,796	0,428	4,5	248,571	1	63,9744	0,143
5,757	1,339	4,855	0,551	0,789	342,857	1	60,1244	0,789
2,546	1,921	0,855	0,428	4,566	394,286	4,5	180,0932	0,932

5,412	1,13	10,627	0,551	6	514,286	1	32,7992	0,932
13,722	3,075	3,367	0,246	0	394,286	0	29,0388	1,789
4,498	2,853	1,495	0,428	0	514,286	0	31,542	4,643
3,636	1,493	1,35	0,979	0	145,714	1	81,7852	0,932
4,352	1,93	5,275	0,428	0	308,571	4,5	48,9552	4,643
3,365	1,075	4,141	0,123	0	162,857	0,066	0	6,143
3,062	1,93	2,066	0,428	0	197,143	4,5	18,816	0,789
7,019	3,354	1,855	0,123	1	505,714	1	102,9952	4,643
3,573	6,364	10,007	1	1,789	128,571	4,5	0	5,289
6,491	1,141	2,132	0,428	0	325,714	0,789	9,324	0,789
3,713	1,721	3,578	0,123	0	317,143	0	0	0,209
8,17	1,636	3,721	0,428	0	154,286	0	49,686	4,643
6,849	5,707	5,776	0,786	0,143	445,714	4,5	57,974	6,789
5,518	2,425	1,143	1,428	4,5	342,857	1	111,9776	1,789
7,362	2,862	1,198	0,123	0	205,714	0	146,048	0,789
4,355	2,642	7,856	0,786	0,932	240	4,5	98,329	5,289
2,186	1,284	6,495	0,786	0	565,714	4,5	0	0,143
5,652	1,493	7,721	0,123	0,066	334,286	0,143	37,296	0,789
3,346	1,504	7,57	0,066	0	300	1	103,432	6,789
3,689	2,565	1,776	1	1,066	291,429	1	17,3124	1,143
2,898	0,921	8,198	0,066	0	308,571	0	0	6,143
5,001	1,141	1,284	0,428	1,143	222,857	4,5	23,058	0,932
4,866	2,642	11,919	2,5	4,5	257,143	1	131,0792	5,289
6,722	2,642	9,141	0,786	4,5	360	0	120,008	0,143
6,001	1,79	2,572	0,428	0	205,714	4,5	126,784	1,789
2,84	2,142	5,416	0,066	0	394,286	4,5	0	0,143
3,909	1,342	7,71	0,428	6	411,429	4,5	51,282	0,789
4,284	1,779	10,209	0,123	5,289	197,143	6	9,324	0,066
5,858	2,007	5,056	0	0	360	1	29,946	0,143
5,049	2,207	2,198	0,123	1	171,429	4,5	0	5,289
4,924	4,154	1,93	1,428	0,495	445,714	4,5	95,6004	1,218
4,423	0,132	0,066	0,123	0	154,286	0,789	11,9364	0
2,249	2,721	2,275	0,066	0	240	1	306,6	0,143
4,154	1,787	0,143	0,123	4,5	154,286	4,5	42,924	0
3,768	2,205	3,352	0,123	1	171,429	0,143	1979,824	6,066
4,495	1,636	6,921	0,428	6	497,143	4,5	146,622	1,578
4,506	5,575	7,064	0,428	1,789	334,286	4,5	58,282	6,143
6,985	3,156	6,932	0,428	4,5	360	1	116,62	6,143
4,278	2,075	14,073	0,066	0,429	240	4,5	104,902	4,643
4,366	2,711	8,644	0,123	0,789	231,429	1	170,66	4,643
4,872	2,216	0,143	0,428	0	120	0	44,1616	1,143
4,784	2,559	5,93	0	1	214,286	4,5	155,9502	5,289
5,423	2,427	3,132	0,428	1	120	0,143	27,9832	6,789
7,923	1,064	4,066	0,123	0	317,143	1	3,066	4,643
8,189	1,864	5,776	0,428	0,066	420	0,789	0	4,566
4,847	1,721	1,064	0,428	0,066	325,714	0	46,648	4,643
15,291	1,919	13,075	0	4,643	334,286	4,5	335,944	5,289
4,04	1,207	2,198	0,066	0	77,1429	1	23,324	6,789

5,627	2,273	1,064	0,428	1	77,1429	1	99,1396	0,932
7,803	0,132	1,066	0,856	0	120	0	0	0,066
4,566	2,13	0,275	0,428	0	120	0,143	11,5584	0,066
9,379	1,416	2,073	0,428	0	257,143	1	109,1468	4,643
4,214	2,721	4,998	1	4,5	265,714	0,789	183,232	5,289
3,349	2,636	1,864	0,786	0	85,7143	1	43,428	1,789
6,713	2,788	0,275	0,428	0	385,714	6	65,982	0,789
4,638	3,082	3,482	0,786	0	360	1	64,6212	6,789
7,95	2,576	2,721	0,786	0	360	0,789	600,04	1,578
4,763	6,21	5,13	0	0,143	240	0	128,282	5,289
7,082	1,141	0,286	0,428	0	120	0,789	117,978	0,789
3,634	1,495	3,932	0,428	0	480	0,789	68,7792	0,143
5,638	1,853	7,13	0,123	0	197,143	1	37,296	4,643
3,046	2,293	3,13	1	0,066	582,857	0,429	439,866	6,789
4,344	1,493	1,776	1	0	360	1	17,0688	4,643
4,44	2,787	9,787	0,428	6,143	240	0	186,62	6,789
7,524	1,853	0,715	0,428	0,429	300	0	48,398	5,289
4,289	3,442	10,273	0,856	0,066	582,857	4,5	316,8284	0,932
6,996	2,075	3,209	0,123	1	188,571	1	16,506	0,932
2,7	1,853	11,053	0,066	0	480	1	1,344	0,789
4,346	2,862	0,341	0,123	0,932	300	0,066	102,564	1,143
15,705	0,932	2,352	0,856	1	385,714	1	69,972	1,789
10,868	2,502	1,361	0,428	0	171,429	1	23,8644	4,643
8,583	3,073	4,864	0,428	0	265,714	4,5	390,012	5,289
4,858	2,139	4,704	0,428	0	137,143	0	255,3068	1,789
6,937	2,51	0,352	0,123	0,789	231,429	0,066	21,5656	5,289
1,812	1,207	1,715	0,428	0	420	1	62,5576	0,143
8,428	1,57	10,353	0,786	0,143	197,143	1	341,32	4,643
7,992	2,576	3,796	0,428	0	360	1	65,6572	1,789
8,583	6,63	3,721	0,428	0	137,143	4,5	0	1,789
4,157	2,352	0,143	0,428	1	265,714	1	33,7204	1,143
3,907	1,625	1,141	0	0	300	1	198,0132	0,143
5,357	1,561	4,932	0,786	0	257,143	4,5	41,972	5,289
4,06	1,644	1,209	0,123	0	282,857	4,5	60,606	0,143
4,984	0,473	3,71	0,428	1,066	480	0,143	94,3124	6,789
7,158	1,273	3,932	0,786	0,143	291,429	4,5	198,0132	1,789
4,917	2,073	5,216	0,428	1	291,429	0,789	17,1696	1,578
9,22	1,57	1,143	0,428	1	274,286	0	54,53	5,289
5,062	5,784	16,787	0,428	4,5	77,1429	4,5	79,4192	4,643
4,858	3,005	3,704	0,856	4,5	531,429	0,143	123,298	1,218
4,492	1,713	4,286	0,123	0	137,143	1	108,388	4,643
3,195	1,416	1,209	0	4,5	197,143	1	153,6584	4,643
5,504	2,576	3,427	2,5	4,566	497,143	1	104,594	5,289
6,598	2,433	6,789	0,428	1	257,143	1	0	1,578
4,794	3,51	2,143	1	1,066	205,714	4,5	186,62	4,643
4,214	1,218	4,418	0,428	0	480	0	48,398	6,143
5,142	2,51	4,209	0,123	4,5	394,286	1	0	1,066
6,44	1,93	0,998	0	0	154,286	1	600,04	4,643

4,052	3,216	11,288	1	0	214,286	4,5	23,478	0,143
4,426	2,719	3,141	0,428	4,5	154,286	1	108,9172	5,289
4,632	1,985	9,064	0,428	4,5	462,857	1	78,652	6,143
4,575	3,642	5,51	0,786	6	445,714	1	111,5912	5,289
5,724	2,207	5,789	0,428	4,5	420	4,5	153,244	0,789
10,07	4,156	1,361	1	6	342,857	0	0	0,789
6,356	2,998	9,721	0,551	4,5	342,857	0	0	6,143
6,005	1,787	1,572	0,428	0,789	145,714	0	141,2432	4,643
3,131	1,715	0,352	0,428	0,789	120	1	48,65	1,143
5,295	1,93	2,638	0,786	1	402,857	0	15,0528	0,932
5,069	0,275	0,209	0,428	0	102,857	0	18,0012	4,643
2,983	1,71	2,064	0,428	4,5	120	0,789	90,664	1,578
4,067	1,647	8,143	0	0,429	171,429	1	30,002	0,572
17,282	2,075	3,638	0,786	6	185,714	0	0	0,932
3,273	0,352	0,341	0,428	0	342,857	0	39,858	0,143
5,001	1,35	7,361	0,428	4,5	300	1	440,02	0,789
2,966	1,638	2,796	1	1,066	231,429	4,5	121,324	0,789
7,364	1,561	6,341	0,551	1	77,1429	0,066	137,8636	6,789
3,691	0,987	3,132	0,428	0	102,857	1	105,2856	0,143
4,28	2,285	2,858	0,066	0,066	351,429	1	233,24	0,143
10,146	1,561	17,15	0,551	1	222,857	4,5	93,296	0,143
3,643	0,924	7,427	0,123	4,5	137,143	0,143	11,13	5,289
7,361	2,576	11,641	0	0,789	240	1	115,948	0,143
6,924	2,996	2,073	0,123	0,066	291,429	4,5	22,0542	5,289
3,283	2,653	17,341	0,123	0	240	4,5	251,8964	5,289
3,918	1,924	7,073	0,786	4,5	300	4,5	294,6916	4,643
7,332	0,55	2,921	0,123	0	240	4,5	0	0,789
4,564	2,853	5,495	0,786	1	377,143	0	1260,084	0,143
2,462	2,064	4,13	0,066	0	360	4,5	11,662	6,066
3,918	1,636	5,064	0,123	0,143	240	1	43,7976	6,143
7,229	1,853	2,644	0	4,5	240	0	14,35	4,643
4,3	6,144	2,998	0,428	0	300	4,5	0	0,143
5,788	2,644	1,721	1,214	0	257,143	1	15,442	0,143
5,335	6,353	2,367	0,428	1	180	4,5	116,62	0,572
5,001	2,796	4,132	0,066	0	334,286	1	154,105	0,143
9,387	1,787	6,367	0,428	4,5	274,286	1	155,6044	5,289
6,508	1,495	1,218	0,246	0	171,429	6	0,2268	0,789
3,841	2,139	6,275	0,066	0,143	394,286	0	121,324	0,789
7,442	1,93	4,053	0,428	1,066	180	0	25,7656	4,643
2,68	2,216	3,218	0,428	0,932	428,571	0	12,1212	4,566
2,263	3,642	11,053	0,123	0,143	137,143	4,5	153,244	5,289
4,124	1,57	4,418	0	0	300	0	36,204	0,066
1,405	2,57	0,495	0	4,643	180	0	62,664	4,643
7,697	2,842	10,075	0	4,5	265,714	1	25,9084	5,289
5,205	2,13	3,572	0,189	0	240	0	14,35	6,143
1,768	1,561	6,855	0,428	0	377,143	1	4,6788	0,143
8,035	1,93	1,284	1	6,066	394,286	0,143	58,282	0,789
5,326	2,13	10,429	0,428	4,5	540	0	26,1212	4,566

10,977	1,644	6,847	0,428	0	317,143	4,5	37,562	0,143
5,004	2,785	16,71	0,856	4,5	240	4,5	43,6604	5,289
4,561	1,864	0,198	0,428	0	282,857	1	26,9416	5,289
4,548	5,707	7,864	0,428	1	120	0	123,242	0,789
5,792	1,787	4,864	0,428	0	154,286	1	38,066	0,143
7,221	4,222	9,722	0,428	0	385,714	1	41,3392	6,789
4,146	1,996	5,638	0,428	1	111,429	4,5	6,132	1,143
3,71	1,209	0,132	0,123	0	582,857	0,143	39,326	0,066
1,1	2,341	1,996	0	0	471,429	0	22,5204	4,643
4,429	1,57	5,141	0,123	0,143	282,857	0	0	1,143
5,586	1,933	4,864	0,786	0	240	0,789	6,72	0,789
6,209	2,15	8,578	0	0,789	205,714	0	4,662	1,789
7,084	1,284	2,429	0,189	0	102,857	1	78,652	0,932
1,198	2,864	3,396	0,066	0,143	394,286	1	96,796	6
4,99	1,067	11,421	0,123	0	420	1	60,3232	0,066
4,41	6,287	3,053	0,066	4,5	394,286	0	361,368	6,143
4,728	1,713	6,418	0,428	0	197,143	0,789	148,4476	5,289
8,694	2,996	6,638	0,428	4,5	180	1	39,984	4,643
4,484	3,365	10,704	0,428	0	360	4,5	193,4884	0,143
4,06	1,352	0,855	0,123	1	351,429	4,5	23,058	1,143
8,419	5,204	17,921	0,856	4,5	265,714	1	228,0012	4,643
3,497	2,207	0,286	0,123	0	274,286	4,5	16,884	6,143
7,436	2,51	2,341	0	0,789	240	0,429	189,2464	0,143
3,478	1,644	2,075	0,428	1	411,429	4,5	19,194	0,143
9,368	2,219	4,275	0,786	0	351,429	1	90,006	7
3,192	1,93	17,987	0,428	4,566	274,286	4,5	139,048	0,932
8,573	2,141	8,429	0,428	4,5	171,429	1	186,62	6,789
6,572	1,787	2,932	0,246	0,789	377,143	4,5	23,7552	0,932
3,392	1,196	3,418	0,123	4,5	274,286	1	31,388	5,289
7,735	2,788	8,007	0,123	0	317,143	1	32,4744	0,143
3,72	1,416	6,721	0,551	0	274,286	4,5	39,984	4,643
4,212	6,21	4,921	0,786	0,066	514,286	0	72,7944	6,143
3,915	1,141	0,998	0,428	4,566	317,143	4,5	0	0,286
4,26	0,33	10,566	1	0	171,429	1	81,508	0,143
3,104	0,998	0,998	0,428	1	360	1	155,2796	5,289
5,515	1,57	5,352	0,123	4,5	342,857	4,5	16,2372	0,572
12,804	2,073	2,075	0,428	0,429	282,857	4,5	4,662	0,572
6,06	2,776	2,484	0,856	4,5	240	1	540,036	1,066
4,42	1,842	3,13	0,428	0,789	171,429	0	16,128	0,932
2,689	1,141	3,075	0,123	1	351,429	0,066	67,3092	6,143
2,627	2,075	3,284	0,786	0	480	4,5	15,33	6,789
8,437	2,293	2,789	0,428	0,429	137,143	0,429	0	4,643
4,471	2,653	0,561	0,856	0	368,571	0,429	34,4232	1,218
2,403	1,996	2,855	0,428	0,143	240	0,789	61,3536	4,643
2,557	1,93	7,275	0,066	4,5	480	1	183,232	0,495
4,427	2,51	2,855	0,428	1	325,714	4,5	0	1,789
6,524	8,15	12,572	0,786	1,578	197,143	6	179,984	6,789
7,229	1,93	2,075	0,428	0	428,571	0	41,9272	5,289

6,781	2,719	1,141	0,428	4,566	60	4,5	131,0792	0,932
8,576	1,856	2,075	0,428	1	137,143	0	34,986	1,789
3,203	2,348	12,939	0,786	0	240	4,5	4,8384	6,789
7,653	1,908	1,075	0,428	1	300	1	114,8224	0,789
6,361	1,864	1,218	0,428	0,932	351,429	1	46,2728	0,789
2,849	1,996	2,644	0,428	0,066	171,429	1	145,936	4,643
4,421	1,352	2,836	0,428	1	351,429	4,5	35,378	4,643
3,051	1,996	6,066	0,123	0	720	1	48,65	6,143
5,906	2,064	0,132	0,428	0	240	1	47,3172	0,143
4,563	2,216	0,998	0,123	4,5	360	0	0	0,143
2,964	1,636	12,653	0,123	4,566	377,143	0,143	390,026	6,143
8,352	1,787	2,855	0	0	154,286	4,5	158,0012	0,789
9,53	1,715	2,998	0,856	0,789	265,714	4,5	38,9312	1,218
9,456	3,073	4,218	0,428	0,789	154,286	1	54,0792	1,218
6,732	1,787	3,218	0,428	0,143	94,2857	0	291,662	4,643
5,869	1,493	1,066	0	0	120	0,789	26,3676	0,143
13,946	2,367	2,932	0,428	0	197,143	4,5	0	0,789
4,006	1,921	4,284	0,428	1	282,857	4,5	35,322	1,578
2,339	3,139	5,198	0,428	5,289	300	0,066	37,3072	0,789
7,859	2,209	5,561	0,428	0	77,1429	0,789	57,3272	0,572
2,405	3,064	9,141	1,786	4,5	197,143	1	94,6512	0,143
2,755	1,71	3,066	0,123	4,5	360	0,429	173,278	0,143
10,578	1,427	11,864	0,066	6	274,286	0	27,72	0,789
5,711	1,273	5,484	0,066	6,143	120	1	47,4264	0,143
6,218	1,779	5,007	0,494	4,566	180	1	226,604	0,143
6,928	3,51	7,566	0,786	4,5	154,286	1	64,386	0,789
5,357	4,064	5,789	0,428	0,789	480	0	116,55	5,289
5,212	1,864	5,275	0	1	368,571	4,5	116,872	6,789
3,118	1,419	1,141	0	1	120	0,789	62,3924	0,572
3,909	2,565	2,064	0,123	4,5	317,143	1	3,738	6,789
6,884	1,064	0,418	0	0	120	4,5	64,1312	1,578
3,769	2,359	8,352	0	4,5	265,714	0	35,9156	6,789
4,148	0,987	1,198	0,786	0,143	325,714	1	52,0772	0,143
4,992	1,998	0,209	0,123	4,5	162,857	0,789	39,326	4,643
4,225	2,433	3,721	0	4,5	291,429	0,789	35,378	5,289
6,878	0,484	3,286	0,786	1	325,714	1	282,0188	0,789
3,763	1,638	6,987	0,428	4,5	120	0	380,016	4,643
9,925	0	0,209	0,786	0	565,714	0	2,1504	0,143
3,48	1,284	4,998	0,123	0	291,429	1	23,926	6,143
11,652	7,575	5,286	0,428	1	48,5714	4,5	30,912	5,289
14,714	5,564	6,721	0,428	1,143	154,286	0	204,1172	0,143
15,312	3,299	2,367	0,428	0	548,571	0	24,3964	0,789
4,127	1,57	4,064	0,786	0	360	0,066	55,2804	6,789
8,891	2,631	4,299	0	0	308,571	0,789	34,986	4,643
4,772	0,627	3,275	0,786	4,5	360	1	93,296	5,289
7,705	1,075	2,855	0,428	0,066	317,143	0,789	140	0,572
3,36	2,282	4,932	0,428	4,643	214,286	1	93,24	5,289
2,986	2,216	4,578	1	0	334,286	0	0	6,789

2,689	3,071	1,13	0,066	0,143	600	0,789	324,0384	0,789
3,335	1,787	0,789	0,428	0	514,286	1	130,6144	0,143
1,985	1,644	1,143	0	0,066	480	4,5	47,1912	4,5
6,941	2,139	7,776	0,428	1	565,714	0	181,328	0,143
5,952	1,207	3,787	0,428	0,143	240	4,5	153,6892	0,143
3,071	1,996	0,275	0,428	0	60	0	4,662	0,209
0,693	0,704	0,998	0,428	4,5	480	0	0,8064	0,932
3,346	2,842	2,787	0,428	4,643	428,571	0,789	35,378	5,289
1,768	5,424	4,71	0,786	0,429	514,286	0	93,24	0,789
9,132	1,93	4,484	0,786	0,143	411,429	4,5	38,9424	1,143
11,009	2,282	19,418	0,786	0,429	137,143	6	536,62	0,789
2,392	5,564	0,473	0,123	0,066	428,571	4,5	104,1768	1,143
8,696	1,416	10,638	1	1,143	102,857	1	0	4,643
5,724	1,35	1,119	0	4,566	377,143	0,066	120,8354	0,143
7,447	2,576	6,067	0,856	1	351,429	4,5	183,9796	1,066
2,703	1,856	3,858	0,123	0,429	214,286	1	65,6446	6,789
5,421	2,642	3,341	0,428	0,066	360	1	36,9992	1,143
1,977	0,275	3,209	0,123	0	600	4,5	263,242	6,066
6,447	2,13	4,484	0,428	4,5	162,857	1	126,0952	5,289
8,095	2,216	1,418	0,428	0	325,714	0	224,0084	0,143
4,654	0,495	5,418	0,428	0	137,143	4,5	4,662	1,143
5,003	1,996	0,924	0,494	0,429	214,286	1	41,958	4,566
8,638	1,647	1,998	0,066	1	154,286	1	84,6524	0,572
4,792	1,919	10,85	0,428	0,143	420	4,5	263,242	4,643
4,641	2,842	1,13	0	4,5	214,286	0,066	283,5742	0,789
5,918	1,427	2,209	0,428	0	205,714	0	0	0,143
4,403	0,484	4,207	0	0,789	94,2857	4,5	362,7932	0,286
6,072	1,71	6	0,428	0	120	1	0	4,643
13,054	3,776	2,427	1	0,429	600	0	158,6032	0,789
4,649	1,93	5,932	0,428	0	188,571	1	65,982	1,578
8,42	2,642	5,632	1	0	514,286	4,5	20,6556	1,578
2,417	2,216	4,35	0,066	0	514,286	4,5	40,4152	0,143
1,397	1,418	6,064	0	0	265,714	4,5	0	0,143
3,783	2,719	10,141	0,428	4,5	274,286	4,5	176,624	0,789
10,251	1,853	2,132	0,428	0	162,857	4,5	13,44	1,578
7,794	1,35	4,352	0,123	4,5	257,143	4,5	27,72	0,143
8,222	3,57	7,066	0,123	0,143	394,286	4,5	89,992	4,643
3,643	2,073	3,578	0,428	6	257,143	0,143	19,4572	6,789
5,57	3,796	4,627	0,246	4,5	394,286	4,5	122,2144	1,789
6,289	1,996	2,143	0,123	0	90	0	323,344	1,066
2,56	1,284	4,367	0	1	222,857	1	40,0932	4,643
5,894	0,275	0,132	0,066	0	360	4,5	26,0652	0,066
3,838	1,064	2,572	0,428	0,789	222,857	0,066	30,66	4,643
5,08	2,062	3,119	0,428	0	240	1	10,038	1,789
4,427	0,638	1,286	0,123	1	94,2857	1	63,084	6,066
3,431	0,998	1,853	0,428	0,066	360	4,5	122,1584	0,143
4,129	1,79	0,418	0,123	0	214,286	1	4,8384	6,789
5,201	0,627	5,209	0,132	6	428,571	4,5	109,3232	6,066

7,576	6,995	10,719	0,428	4,566	111,429	4,5	0	6,789
7,731	2,062	5,864	0,428	0	360	1	160,4442	5,289
12,939	1,141	3,341	0,123	0	282,857	1	122,2788	1,143
3,654	2,576	7,721	0	4,5	222,857	1	12,3816	6,789
4,854	1,561	7,275	0,066	4,5	300	1	152,1184	4,643
3,428	0,484	5,141	0,123	4,566	342,857	0	0	6,143
4,832	1,57	1,141	0,428	0	291,429	0,066	11,3484	1,578
4,574	0,855	0,132	0,123	1	120	4,5	31,514	0,932
4,267	1,919	8,222	0,786	0,789	411,429	0	0	1,578
7,649	0	0,275	0,123	0	651,429	0,789	15,2796	0,143
0,825	1,119	5,218	0,066	1	651,429	1	193,27	6,066
6,629	1,919	13,143	0,428	4,5	402,857	0,143	13,5492	4,643
5,366	0,33	1,141	0,428	0	102,857	0	1,344	0,143
4,983	1,196	2,932	0,428	0,789	308,571	0,143	4,662	5,289
3,195	0,462	1,644	1,428	0,789	411,429	4,5	67,1132	0,495
6,94	3,288	2,352	0,428	0	214,286	4,5	323,232	1,789
4,508	1,998	0,627	0,428	4,5	171,429	4,5	106,624	1,066
4,86	1,207	0,209	0,428	0,143	68,5714	0	121,324	0,143
7,953	2,862	2,075	0,428	0	300	4,5	29,316	0,143
5,861	1,787	6,921	0,428	1	214,286	1	48,958	4,643
10,206	2,293	2,007	0,786	0	394,286	1	66,794	1,218
2,502	2,367	4,921	0,428	0	240	4,5	269,976	5,289
3,052	0,429	5,418	0,428	4,5	480	4,5	102,8132	0,143
3,843	1,842	1,998	0,428	4,5	300	1	92,8032	4,566
6,508	1,57	3	0,428	0	188,571	1	300,02	1,578
4,348	3,13	4,864	0	0,429	214,286	4,5	55,972	0,143
15,291	1,218	0,855	0,428	0	257,143	0,789	12,1212	0,789
10,206	2,645	7,141	0,786	0	94,2857	4,5	16,5676	0,932
4,618	1,93	1,578	0,428	4,5	257,143	1	50,0052	5,289
3,706	2,719	12,867	0,786	1	120	4,5	182,5852	1,143
5,364	3,511	2,141	0,786	0,495	154,286	1	79,716	1,143
3,61	2,631	3,504	0,123	1,789	180	1	450,03	1,143
3,412	1,196	1,493	0,551	0,789	240	0,789	29,4336	5,289
3,972	2,51	4,13	1	0	205,714	0	16,128	0,789
8,653	1,93	8,93	0,428	1,143	171,429	4,5	233,24	1,789
9,14	1,067	1,418	0,123	0	102,857	1	165,6452	0,066
2,504	1,787	2,286	0,428	1	205,714	0	30,66	6,143
5,144	2,273	3,853	1	0	497,143	1	0	5,289
8,217	2,367	4,51	0,786	6	171,429	0	0	6,789
3,58	2,862	11,653	0	4,566	377,143	1	67,298	5,289
3,126	1,561	7,075	0,123	0,066	291,429	0	244,902	6,789
13,794	1,361	17,565	0	0	145,714	1	5,4684	6,143
6,367	7,864	5,578	1,214	0	231,429	1	176,624	6,789
4,918	1,864	1,853	0,428	0	445,714	0	74,592	0,143
5,291	3,431	3,719	0	0,066	171,429	1	39,921	6,143
9,943	2,427	15,156	0,786	0	685,714	4,5	263,242	5,289
3,494	2,139	4,207	0,246	0,066	462,857	0,789	32,1972	0,143
3,423	1,787	4,704	0,066	0,429	445,714	4,5	151,606	6,143

3,346	0,572	8,132	0,786	4,5	317,143	1	60,004	0,066
6,867	0,998	5,361	0,428	0,789	257,143	1	70,0952	0,143
5,563	1,776	0,998	0	0,789	420	0	7,1736	1,143
4,722	3,299	10,007	0,428	5,289	248,571	4,5	599,984	6,789
10,848	2,062	12,064	0,856	0	240	6	3,9312	0,066
3,933	2,207	9,361	0,123	4,5	445,714	0,789	61,866	4,643
3,783	2,073	2,196	0,428	0,789	180	0	30,002	4,643
3,772	3,222	7,853	0,066	4,5	394,286	4,5	201,32	0,143
3,504	2,576	2,932	0,123	0	120	0,789	76,622	5,289
4,278	1,93	2,789	0,428	0	265,714	4,5	87,7296	0,932
3,282	1,638	10,932	0,551	4,643	27,8571	0	27,6864	0,789
3,283	7,842	21,132	0,786	6	274,286	1	96,306	6,789
6,082	3,367	5,367	0,428	0,286	162,857	1	67,298	1,789
3,996	2,796	9,647	0,428	4,5	514,286	4,5	236,0372	0,789
3,772	2,282	9,853	0,066	4,5	351,429	4,5	66,493	6,143
4,258	1,787	1,352	0,123	1	205,714	1	25,452	6,143
9,776	1,141	11,504	0,123	4,5	394,286	4,5	22,6296	4,643
6,008	1,493	0,143	0,428	4,5	385,714	0	336,0224	0,572
8,42	6,575	9,721	1	0	300	1	26,88	0,143
6,584	4,156	9,864	0,428	5,289	437,143	4,5	39,858	6,789
5,353	2,642	3,13	0,123	0	62,8571	1	210	1,789
2,546	1,996	1,207	0,551	4,5	325,714	0,143	108,2746	0,143
3,272	5,916	5,787	0	0	300	1	630,042	4,566
5,212	2,216	6,644	0,428	1	180	4,5	6,9384	0,143
6,506	1,93	4,429	0	0,789	308,571	4,5	19,194	5,289
6,515	2,855	1,075	0	0	291,429	0	10,9788	5,289
2,321	5,927	8,864	0,428	0,066	180	4,5	126,6146	0,143
6,876	2,073	1,275	0,428	0,429	300	0	67,606	0,143
6,798	2,998	1,143	0,428	0	257,143	6	9,324	0,789
6,583	1,636	2,367	0,428	0	214,286	1	695,702	0,789
4,627	0,407	17,5	0,123	0,789	274,286	0	307,972	4,643
5,926	3,222	0,921	0,428	0	205,714	1	275,0412	0,066
5,843	2,205	14,786	0,786	4,5	394,286	4,5	37,0202	6,143
4,052	2,499	1,119	0,551	0,789	411,429	0	56,8764	1,578
7,651	2,141	2,495	0,856	1	445,714	4,5	71,666	4,643
5,935	3,945	1,864	0,428	0,143	428,571	0	84,0056	5,289
10,558	6,21	1,352	0,786	1	137,143	1	117,3172	5,289
4,421	1,284	2,218	0,428	0	231,429	1	33,166	6,789
9,211	4,154	1,284	0,428	4,5	154,286	4,5	326,62	0,789
5,63	1,93	7,352	0,428	1	214,286	0,429	84,532	4,643
5,774	1,427	6,932	0,123	0	180	1	390,8184	6,789
14,794	1,119	8,367	0	0	334,286	4,5	120,008	6,789
4,711	0,627	3,504	0,551	0,066	385,714	1	72,7664	0,932
2,711	2,653	6,064	0,428	0	248,571	0,789	184,0384	0
11,427	7,999	6,495	0,786	0,789	514,286	0	901,264	0,143
3,994	1,787	2,341	0,123	4,5	137,143	0	25,2112	4,566
6,218	2,642	1,919	0	0	437,143	0	145,2066	1,066
10,794	7,353	16,945	0	0,429	240	4,5	0	6,789

6,44	2,93	5,222	0,786	4,5	488,571	4,5	307,972	0,143
4,847	5,498	5,275	0,123	4,5	214,286	1	91,8456	1,143
5,291	3,222	5,143	0,786	4,5	248,571	0,066	7,9716	4,643
9,995	1,427	3,15	0,428	4,5	197,143	4,5	234,2956	1,578
3,423	1,35	5,781	0,428	0	94,2857	1	52,7772	5,289
3,94	2,653	3,275	0,066	0,066	171,429	0	17,1192	0,066
13,511	1,207	10,864	0,428	1	137,143	4,5	0	1,143
6,581	2,708	0,495	1,214	0,789	240	1	280,6804	0,932
4,472	6,782	9,787	0	0	257,143	1	46,8804	5,289
7,352	5,707	5,075	0,428	1	514,286	4,5	234,0632	5,289
6,504	1,996	7,064	0,428	0	171,429	4,5	57,974	0,143
8,209	2,996	6,141	0,428	6	411,429	0,066	48,3112	6,066
5,28	2,007	0,998	0,428	0,789	360	4,5	36,0024	0,143
3,918	2,796	9,578	0,786	0,789	428,571	4,5	236,306	6,143
4,926	2,576	1,495	0,428	0	222,857	1	88,6312	1,789
3,763	7,573	5,644	0,428	0,789	180	4,5	120,652	0,789
9,083	1,198	5,644	0,428	1,066	102,857	0	229,95	1,143
5,941	6,287	3,288	0,428	0,855	368,571	0	108,0072	4,643
3,139	1,57	5,275	0,123	0	334,286	4,5	10,752	4,643
4,992	0,407	0,341	0	4,5	411,429	0	94,9088	0,855
1,482	1,776	0,264	0,856	0	531,429	4,5	0	0
4,139	1,504	4,15	0	4,643	162,857	4,5	0	1,143
5,779	3,288	4,427	1,642	0	557,143	FAŁSZ	76,622	0,143
12,67	1,864	5,156	0,428	4,5	214,286	1	881,8796	4,643
6,515	2,719	3,196	0,428	1	240	0,789	25,8552	1,143
5,858	0,638	4,776	0	1	368,571	4,5	11,8776	0,789
5,943	3,787	6,721	0,428	1	257,143	4,5	167,972	1,789
6,071	1,998	1,143	0,123	0,143	102,857	0	44,8812	0,932
6,291	1,284	5,93	0,428	0	0	0	136,612	1,789
6,431	2,719	11,442	0,428	4,5	257,143	4,5	0	6,789
3,258	1,284	7,218	0,123	0	257,143	4,5	150,752	0,932
3,107	1,284	13,71	0,066	4,5	360	0,789	0	0,143
6,001	1,93	0,209	0,428	0,429	154,286	1	85,3384	1,143
5,155	0,616	4,653	0	1,578	171,429	4,5	77,4284	1,789
7,214	3,642	6,288	0,428	1	180	1	71,6464	0,143
5,423	1,33	1,143	0,123	0	162,857	4,5	76,2384	1,143
3,47	2,568	7,93	0,132	0,066	728,571	4,5	123,298	0,789
5,568	1,638	14,221	0,428	0	55,7143	4,5	327,0092	0,789
6,348	3,653	7,144	0,428	0	274,286	0	198,254	4,643
3,698	2,216	7,218	0,786	4,643	565,714	0	122,64	0,789
6,711	2,653	15,932	0	4,566	248,571	4,5	24,528	4,643
3,698	1,35	10,721	0,428	0	334,286	0,143	36,792	5,289
4,935	2,075	2,275	0	0,789	317,143	0,066	75,7232	1,143
5,192	2,631	1,921	0	4,566	240	6	984,5528	1,143
2,755	0,704	2,198	0,123	0	300	0,789	83,6192	6,143
4,135	1,702	9,418	0	0	514,286	4,5	202,9566	6,066
4,792	2,653	4,075	0	0,789	197,143	0,143	16,2708	4,643
4,858	1,064	8,932	0,428	0	497,143	1	77,1232	0,932

5,209	0,847	9,787	1	0	300	0	25,0992	4,566
2,99	0,209	1,286	0,123	0	462,857	0	21,7812	4,566
5,649	3,288	4,51	0,428	0	77,1429	1	108,0072	4,643
3,706	2,141	20,858	1	0	274,286	6	151,606	6,789
5,416	2,776	10,352	0,786	0	394,286	6	107,9932	4,643
9,492	2,493	9,576	0	0	188,571	1	135,8182	6,789
4,333	2,565	5,853	0,428	0	102,857	1	108,64	0,855
4,374	2,15	2,209	0,428	0,789	120	0	0	1,218
4,069	5,927	4,418	0,246	0	197,143	0	41,958	4,643
3,918	1,144	18,275	0	0	334,286	4,5	43,442	0,789
9,778	3,499	5,51	0	0,143	60	0	0	5,289
13,348	10,987	1,198	0,551	0	291,429	4,5	36,3888	0
10,076	1,418	2,561	0,428	0,789	222,857	1	3,78	2
4,186	1,13	5,218	0,428	0	77,1429	1	127,4392	1,143
4,632	6,353	0,704	0,428	4,5	531,429	0	123,242	6,143
6,706	1,908	4,653	0	1	240	1	0	1,143
4,344	2,721	11,286	0,428	1	274,286	0,789	69,6416	0,932
3,786	2,653	3,789	1	0,932	51,4286	6	36,288	1,143
6,799	2,13	0,855	0,428	0,789	540	4,5	177,5228	1,143
1,93	1,578	2,066	0,428	1	94,2857	1	89,992	1,143
6,299	1,284	2,578	0,428	0	411,429	1	93,24	4,643
2,785	1,93	1,998	0,428	1	45,7143	1	0	0,932
7,673	3,508	3,864	0,428	0	111,429	1	151,312	5,289
4,137	1,284	5,932	0,428	0,789	55,7143	0,143	61,4124	5,289
5,058	3,222	1,853	0,428	0,789	514,286	0	56,882	5,289
4,049	3,288	6,007	0,428	0,932	300	0,789	82,1324	1,578
8,077	1,493	12,841	0,856	4,5	300	0	25,095	5,289
3,481	2,065	1,919	0,494	0	274,286	1	84,6524	6,143
5,148	1,855	7,341	0,123	1	274,286	4,5	37,296	5,289
2,996	2,93	3,209	0,428	1	317,143	1	18,1188	4,566
3,777	1,262	0,418	0	1	360	0,066	12,9444	0,143
5,869	1,93	9,207	0,428	0	360	1	0	4,643
7,502	2,362	4,51	0,123	1,789	454,286	4,5	30,002	4,643
3,412	1,075	3,275	0,428	0	248,571	4,5	104,6584	0,066
4,72	0,715	4,007	0,428	0	325,714	1	53,4464	1,143
4,707	2,141	13,341	1	4,643	257,143	1	66,2004	5,289
3,426	1,075	0,066	0,066	0	282,857	6	0	0,066
5,367	1,79	9,427	1,786	1	214,286	4,5	28,7196	0,143
3,032	3,499	0,847	0,428	0	197,143	0	117,544	0,143
4,293	1,919	5,484	0,428	0	240	1	37,3072	1,143
6,86	3,367	0,998	1	1	222,857	1	233,24	0,789
8,946	2,062	1,864	0,428	0	231,429	1	85,2992	0,143
4,854	1,35	6,284	0,428	1,143	137,143	4,5	95,7922	1,143
11,523	7,144	7,299	0,123	0,789	317,143	4,5	2,688	1
4,779	2,053	5,498	1	1	188,571	1	116,62	0,066
10,129	1,842	0,855	0,786	1	274,286	1	204,6212	0,789
7,453	2,576	1,578	0,428	0	420	1	159,2612	0
6,069	2,062	0,209	0,428	0,066	445,714	0,789	21,4452	0,066

5,728	1,853	5,218	0,428	4,5	180	0	503,216	4,643
4,285	2,433	4,504	0,428	0	325,714	0	35,322	4,643
6,003	2,216	5,932	0,189	0	317,143	0	27,972	0,143
3,139	2,207	2,998	0,428	0	102,857	4,5	51,506	6,143
4,265	1,064	1,638	0,123	1,143	180	1	37,296	0,143
5,273	2,207	1,644	0,428	1,066	300	0,143	37,3184	5,289
2,997	2,499	6,207	0,428	0	205,714	4,5	32,634	5,289
3,632	4,643	7,075	0,428	6	240	4,5	0	6,143
2,911	1,284	4,367	0,786	0	420	4,5	43,442	1,789
9,596	7,284	9,433	0,428	0	180	1	182,924	0,789
4,935	2,51	2,218	0,428	0	165,714	1	34,1544	1,789
5,869	1,218	1,864	0,428	4,5	180	0	0	4,643
1,317	1,559	0,132	0,123	0,066	300	0	6,321	0,789
5,066	2,198	2,352	1	0	411,429	4,5	39,074	4,643
4,99	5,575	6,367	0,428	0	240	4,5	18,102	4,643
4,555	2,928	6,787	0,856	1	368,571	4,5	404,0064	0,789
12,205	1,064	0,132	1	4,5	120	4,5	2,7972	0,143
5,94	1,847	1,143	0,428	0	445,714	4,5	18,816	4,643
3,768	6,347	5,143	0	1,143	300	1	121,9484	0,066
2,249	2,499	3,572	0,428	0,066	514,286	1	43,2012	5,289
5,86	6,641	2,998	0,428	1	514,286	1	76,6612	4,643
5,221	1,79	2,864	0,428	0	308,571	1	253,274	1,143
6,487	6,573	5,361	0,909	0	137,143	1	57,6632	0,143
3,979	0,418	8,286	0,428	0	120	1	10,752	6,143
6,642	1,207	4,209	0,428	1,066	51,4286	4,5	17,7408	1,218
1,111	2,216	6,209	0,786	0	257,143	0,429	23,2848	6,066
3,117	6,364	6,71	0,428	6	274,286	4,5	0	0,143
4,851	2,075	2,209	0,123	0	308,571	0	43,442	6,143
5,775	1,919	10,341	0,428	0,855	377,143	4,5	83,3812	1,143
4,289	1,853	2,429	0,786	1	282,857	1	53,4464	0,209
3,412	0,847	6,644	0,066	0	651,429	0,789	169,946	4,643
2,691	2,796	6	0,786	0	368,571	0,429	36,9992	5,289
2,502	6,287	12,789	2,5	0,143	274,286	4,5	27,972	6
5,061	2,499	2,433	0,428	0	111,429	0	48,454	0,143
7,673	2,576	4,787	0,551	0,066	205,714	0	167,3252	0,789
9,022	7,788	3,143	0,428	4,5	137,143	6	62,8012	4,929
2,15	1,71	4,495	0,428	4,5	582,857	0,789	28,0224	0
7,161	0,473	4,341	0	1	68,5714	4,5	8,064	1,578
4,854	0,781	3,007	0,428	4,5	231,429	6	34,6248	6,143
10,518	1,075	5,075	0	4,5	180	0	10,0632	6,143
3,761	1,132	1,275	0,123	0	171,429	0	22,414	0,789
4,638	4,929	6,93	0,786	0	360	0,789	19,7232	5,289
4,555	2,721	2,367	0,428	0	600	1	2,688	0,789
4,776	2,721	2,209	0,123	1	248,571	4,5	18,648	1,789
7,572	1,207	7,198	0,428	0	197,143	1	25,2112	0,932
7,991	2,636	8,007	0,123	4,5	368,571	4,5	27,72	0,789
2,197	1,218	3,855	0,428	0	162,857	4,5	230,0312	4,643
4,192	2,919	8,082	0,428	0	325,714	1	171,4664	0,429

7,508	3,222	6,987	0,428	0,066	214,286	4,5	61,1072	0,932
5,364	3,508	4,907	0,856	0,209	445,714	4,5	40,7176	1,789
5,433	1,143	5,075	0	4,5	222,857	4,5	30,002	4,566
9,379	2,642	3,51	0,428	0	240	4,5	18,396	4,643
7,882	5,523	4,433	0,428	0	240	0,789	60,662	1,578
6,792	1,218	1,921	0,123	1	102,857	0	55,944	4,643
5,771	6,364	2,787	0,428	0	394,286	0	22,6632	0,789
13,139	3,578	11,218	0,428	5,289	411,429	1	67,298	0,143
2,183	2,216	11,719	0	1	480	4,5	233,24	1,143
6,35	0,99	3,35	0,786	1	360	0	110,8912	5,289
5,284	0,286	2,209	0,428	1	505,714	4,5	36,3636	1,578
6,23	1,644	3,715	0,428	1	111,429	0	61,32	1,143
3,557	3,352	5,55	2,5	1,066	77,1429	4,5	0	0,855
5,944	2,653	3,141	0,428	1,143	180	0	146,622	4,643
5,015	2,284	1,787	0	6	351,429	1	60,7712	0,143
2,381	1,427	9,921	0,066	4,5	420	4,5	20,0088	0,789
6,429	2,862	1,429	0	1	231,429	0,789	13,1292	0,932
4,063	1,933	5,638	0	0,789	60	4,5	166,628	1,789
3,907	3,354	2,141	0,428	0	531,429	4,5	639,968	6,143
5,146	6,993	12,793	1	0,066	334,286	6	0	0,143
5,781	0,638	6,495	0,428	0,066	240	1	49,4172	1,066
4,634	1,919	4,789	0,428	0	565,714	0	4,032	4,5
7,372	1,352	1,998	1	1	68,5714	4,5	48,0032	0,209
3,126	2,636	2,286	1	1	428,571	1	79,268	1,789
7,706	1,853	0,418	0,428	0	462,857	1	50,4308	0,066
2,548	1,284	4,987	0,786	4,5	514,286	0,789	68,2542	6,789
5,221	3,148	16,576	0	0,066	120	4,5	307,6836	1,789
5,716	6,496	11,651	1	4,5	548,571	4,5	632,8392	5,289
7,236	1,361	5,572	0,856	0,429	30	1	16,7832	0,932
7,289	1,93	0,855	0,123	1,143	31,4286	4,5	0	1,789
3,28	1,919	4,075	0,428	0	377,143	1	46,9364	5,289
10,855	11,643	9	0,428	0	205,714	0,429	27,0396	5,289
5,206	1,713	0,209	0	0,789	231,429	0,789	54,53	0,286
4,063	2,862	11,853	0,428	4,5	51,4286	4,5	229,04	6,143
2,043	3,288	11,218	0	4,566	402,857	0,789	153,244	5,289
1,911	1,627	6,064	0,856	1	308,571	1	105,7084	6,143
4,904	3,288	2,418	0,428	0	445,714	0	45,7352	1,066
5,273	2,282	6,264	0,786	0	197,143	4,5	61,4124	0,789
1,265	0,627	4,921	0,066	1	334,286	0	1430,002	0,143
11,586	1,721	0,275	0	0	282,857	0,143	210,5432	0
5,592	2,062	6,57	0,428	0,789	480	4,5	210,014	4,643
7,586	1,856	8,218	0,428	1	154,286	4,5	99,974	1,789
1,625	2,721	10,209	0	0,066	214,286	0	30,002	4,643
4,54	2,35	0,932	0,786	0,789	308,571	0	233,24	0,572
6,062	2,218	7,064	0,428	4,5	394,286	0	24,2592	5,289
5,21	1,919	5,996	0,428	0	137,143	1	19,817	0,143
5,004	2,284	4,064	0,786	4,5	411,429	4,5	60,6424	5,289
6,145	1,776	1,053	2,5	0,429	248,571	4,5	211,8536	1,143

5,08	2,987	2,13	1	1,066	300	4,5	120,008	1,578
4,336	1,207	6,264	0	4,5	240	1	263,242	6,789
8,706	1,493	7,284	0,123	0	154,286	1	25,6536	6,143
5,148	1,218	0,066	0,786	0	120	4,5	31,4412	0,066
7,362	1,275	8,721	0,428	0,789	282,857	0	309,5512	0,789
5,062	0,275	6,053	0,428	0	240	4,5	40,8632	1,143
5,92	2,141	4,264	1,214	0	531,429	4,5	76,7592	5,289
2,197	1,262	6,418	1,284	1,066	180	4,5	30,478	4,643
3,566	2,141	2,284	0,066	4,5	420	1	130,1216	6,789
8,848	2,576	6,141	0,428	0	137,143	1	54,53	5,289
3,832	2,576	4,565	0,066	4,5	540	0,066	103,5524	0,143
7,069	1,853	19,775	0,428	0,066	274,286	4,5	136,843	5,289
2,843	3,431	1,996	2	0,143	180	0	0	1,143
4,858	7,076	5,504	0,856	4,5	120	4,5	14,35	5,289
3,961	1,13	1,341	0,428	1,066	582,857	0	17,038	0,495
8,227	2,71	2,787	0,856	0,789	282,857	1	18,0012	0,143
1,625	0,55	3,064	0,428	0,143	240	0,789	630,042	0,855
4,992	2,216	6,998	0,428	0	394,286	4,5	154,7784	0,932
5,629	5,784	0,715	0,856	4,643	222,857	1	226,604	0,932
3,854	2,427	13,707	0	0	497,143	1	304,192	6,143
3,183	0,704	10,221	0,786	1	171,429	1	176,624	0,789
6,128	12,566	6,429	0,428	4,5	94,2857	1	17,038	5,289
7,926	5,641	4,209	0,428	0,066	351,429	4,5	0	0,143
4,071	0,998	4,132	0,428	0	214,286	4,5	13,44	6,143
6,442	1,218	0	0,428	1	75,7143	0	210,014	0
2,623	1,427	0,275	0,428	0	205,714	4,5	40,9332	1,578
10,861	5,718	0,066	1,284	0	317,143	1	27,972	1,789
5,432	2,919	1,198	0,786	6	437,143	0,789	188,8824	6,789
6,863	3,576	0,132	0,786	0	300	4,5	286,9384	0,143
2,843	1,864	4,141	0,551	0,855	300	0,066	40,0092	1,578
5,926	1,075	0,264	0,428	1	454,286	0	1,5624	0,143
5,067	2,073	9,141	0,428	0	180	4,5	33,852	1,143
3,489	1,35	2,264	0,428	0	257,143	1	158,648	0
6,611	2,271	4,499	0,786	0,789	214,286	0	164,8808	4,643
4,643	1,361	3,075	0,428	0	128,571	0	330,022	0,286
5,577	1,57	4,653	0	0,066	154,286	0,789	51,506	4,643
9,048	1,855	0,143	0,123	0	437,143	0	29,19	5,289
4,51	2,719	0,998	0,428	0	240	0,143	128,282	5,289
5,067	1,55	13,984	0,428	0	154,286	0	214,592	4,643
6,213	1,275	1	0,786	1	240	4,5	120,008	1,066
6,649	2,642	4,719	0,428	6	180	1	86,7524	4,643
2,12	1,119	4,484	0,428	4,5	497,143	0,066	10,8192	0,066
6,79	2,504	5,352	0	0,143	548,571	0,789	52,5364	0,789
4,146	2,862	0,066	0,428	0	240	4,5	90,006	0,932
3,706	5,204	6,209	0,123	0	120	4,5	18,102	1,143
6,847	2,638	10,998	0,428	0,066	180	4,5	200,2672	5,289
9,5	2,282	4,653	0,428	0	360	4,5	137,2924	4,643
7,364	1,141	1,132	0,123	0	300	0	74,8832	0,932

6,792	1,273	6,273	0,428	0	214,286	1	0	1,143
4,717	3,005	14,51	0	4,5	462,857	0	1091,566	4,643
4,865	2,862	3,864	0,428	0,143	154,286	1	41,454	4,643
4,7	2,642	0,209	0,428	0	411,429	4,5	179,3344	0,143
8,848	2,062	3,361	0,123	4,5	248,571	0,143	51,7552	4,566
5,148	2,578	2,644	0,123	0	154,286	1	13,986	0,789
5,148	1,988	6,198	0,428	4,566	300	0	0	4,643
4,357	0,638	2,429	1	1	180	1	0	0,572
3,425	5,421	9,064	0,123	1,066	505,714	4,5	25,3344	6,143
3,779	4,775	0,209	0,428	1	205,714	1	8,1732	4,643
4,212	0,341	1,286	0,428	0	240	1	18,0012	0,066
14,409	1,93	3,064	0,066	4,5	411,429	1	89,1436	0,066
5,711	0,704	4,066	0	1	317,143	1	373,24	6,789
9,17	3,642	6,576	0,428	6	257,143	1	32,7992	6,789
9,648	5,773	0,855	0,066	0,143	437,143	0,789	0	4,643
5,651	1,493	4,561	0,123	1	214,286	4,5	30,002	0,143
10,799	1,933	2,141	0,428	0	325,714	1	191,6236	0,789
3,214	1,352	1,352	0,428	0	325,714	0,066	16,128	0,932
1,54	1,919	0,352	0,428	0	154,286	6	33,8184	5,289
2,843	1,064	1,855	0,428	0	325,714	0	51,744	0,932
11,601	3,585	4,864	0,428	4,5	300	0,789	93,296	1,578
6,295	2,433	1,71	0	1	90	1	111,9552	4,643
7,494	2,493	17,498	0	0	197,143	FAŁSZ	45,7268	1,789
5,223	3,651	12,945	0,428	0,789	274,286	4,5	54,7484	0,789
6,219	1,919	1,141	0,428	0	120	0	39,4464	1,578
2,832	2,425	10,075	1,572	0,143	171,429	4,5	118,4596	0,789
6,212	2,218	1,504	0	0	240	1	318,0212	0,143
6,15	3,222	5,789	0,551	1	197,143	0	4,8174	0,855
3,282	1,847	10,987	0,428	4,5	120	4,5	400,008	6,143
7,218	2,719	0,429	0,786	0	240	1	632,8392	0,143
7,078	2,93	7,284	0,428	1,143	154,286	4,5	9,0048	0,932
3,64	1,713	12,293	0,786	0	94,2857	4,5	187,4264	0,143
5,44	1,218	0,429	0,428	0	351,429	4,5	233,24	0,143
4,348	0,998	0,198	0,428	1	85,7143	1	1,512	6,066
5,715	1,998	2,075	0,123	1	128,571	0,143	16,128	0,143
7,948	3,148	7,51	0,428	4,566	205,714	0,789	72,8924	4,566
2,271	5,498	6,341	0,428	0	257,143	0	17,1192	6,143
6,715	0,704	4,632	0,786	0	214,286	1	363,2244	0,143
9,853	2,062	4,141	0,123	1,066	214,286	1	0	6,789
2,274	0,99	4,353	0,066	4,5	394,286	0	27,79	0,143
4,578	2,576	3,853	0,428	1	480	1	0	4,643
7,275	4,929	1,721	0,428	1,143	402,857	0	0	4,5
3,979	1,842	0,638	0	0	128,571	1	14,3388	0,789
10,822	2,207	3,207	2,284	0,143	94,2857	1	473,9756	0,143
3,417	1,273	3,075	0,428	1	428,571	1	330,022	0,789
5,438	1,93	2,921	0,428	0,789	480	0,066	30,002	0,789
6,286	3,156	5,156	1,284	4,5	120	1	50,6352	0,789
6,996	1,207	3,359	0,123	0	205,714	1	79,0384	1,143

4,281	2,51	7,207	0,428	4,5	197,143	0,143	4,662	4,643
7,37	1,787	8,495	0,428	0	291,429	0	26,88	1,066
5,146	3,642	4,93	0,428	4,5	428,571	4,5	911,722	1,143
5	2,141	0	1	1	308,571	1	13,44	0
4,359	2,565	4,862	1	0	291,429	1	0	0,143
4,684	2,216	3,787	0,428	0,855	394,286	0	50,6912	0,789
9,996	2,502	9,072	0,786	0	180	0	180,012	5,289
6,726	2,719	6,429	0,786	0	274,286	4,5	33,4964	5,289
4,212	4,5	5,429	0	0	274,286	4,5	0	0,143
4,786	7,656	6,721	0,786	4,643	471,429	4,5	17,5812	0,789
10,714	2,93	3,653	0,123	0	428,571	1	107,3212	0,789
3,186	1,996	0,561	0,856	4,5	240	1	50,3342	4,643
3,775	1,207	6,653	0,786	4,5	411,429	4,5	66,6512	0,286
5,926	1,93	19,198	0,852	0	102,857	6	0	0,495
5,781	2,196	0,407	0,428	1	60	1	61,4432	0,789
3,489	1,847	13,572	0,066	0	420	1	97,902	6,143
3,063	1,625	5,075	0,856	0,066	582,857	4,5	31,9872	0,789
3,467	3,433	3,996	0,066	4,5	300	1	45,5784	6,789
5,353	5,564	12,998	0	6	471,429	0	7,623	0,143
9,723	2,919	0,921	0,856	0	300	6	33,6812	0,789
8,046	2,216	4,572	0,123	4,5	334,286	1	27,972	6,143
4,68	1,13	0,352	0,428	0	394,286	4,5	67,298	4,643
3,904	1,416	4,987	0	4,643	317,143	1	72,7202	0,932
5,19	1,627	7,71	0	0,495	600	1	55,4624	5,289
4,049	2,499	7,853	0	0,066	180	4,5	52,7268	5,289
8,511	3,367	0	0,428	0	300	6	2,4192	0
4,058	2,273	1,053	0,786	6,066	420	1	53,984	5,289
7,429	1,787	1	0,428	1,066	411,429	0	100,6236	4,643
2,996	1,141	0	0,123	0,066	137,143	0	122,4916	6,066
1,617	1,067	8,998	1,572	0	214,286	4,5	62,4708	0,789
4,258	2,359	3,13	1,786	0	248,571	1	169,8116	6,143
4,122	1,339	6,495	0,428	4,5	368,571	0	10,8444	4,566
7,989	2,073	6,561	0,123	0	180	0,429	38,4594	0,789
6,146	2,073	9,998	1,786	0	411,429	4,5	177,3184	0
3,86	0,704	10,647	0,786	1	231,429	4,5	64,1172	0,209
5,372	3,365	3,218	0	4,566	94,2857	4,5	40,754	5,289
6,069	2,139	7,918	1,123	1,066	291,429	1	132,5212	0,789
2,898	0,627	6,284	0,066	0	300	0	28,5768	0,143
5,561	1,776	5,007	0,428	0	214,286	1	94,6512	0,789
5,058	1,141	4,367	0,428	0	154,286	4,5	109,06	5,289
4,006	1,57	13,495	0,786	0	308,571	1	240,016	5,289
2,691	2,642	6,495	0	0	111,429	1	102,564	0,572
8,735	2,425	0,418	0,428	0	197,143	0,066	315,3612	0,932
7,862	1,493	10,429	0,428	4,5	531,429	1	24,1332	5,289
3,416	3,787	0,561	0,428	0	325,714	1	108,0072	0,143
11,664	2,139	3,864	0,428	4,5	154,286	4,5	15,33	5,289
6,649	1,416	9,198	0,856	0	291,429	4,5	384,4582	0,932
15,722	0,429	2,429	0,428	0,789	60	0,789	41,9832	0,143

6,941	1,996	3,13	0,428	0	265,714	4,5	119,9926	1,066
3,005	2,13	7,35	0,123	0,143	257,143	4,5	12,726	0,143
6,08	1,919	6,576	0	4,643	154,286	0,066	334,5888	6,789
4,506	2,51	4,286	0,428	0	145,714	6	146,1824	4,643
3,208	2,853	1,352	0,123	1,066	68,5714	1	15,7416	4,643
8,658	4,651	13,429	0,428	1,066	111,429	4,5	95,368	1,789
3,28	1,715	5,218	0	1	128,571	0	0	1,789
6,15	2,796	16,999	0,428	0	137,143	4,5	54,53	6,789
7,089	2,919	8,284	0,786	0,789	445,714	4,5	74,592	0,789
2,777	1,856	10,638	0,066	0,066	677,143	6	93,24	0,143
7,51	1,776	2,141	1	0	240	4,5	0	4,643
4,917	7,144	2,141	0,494	1	60	1	66,0744	6,789
3,86	2,853	6,209	0,428	1	214,286	4,5	20,076	0,143
6,155	2,653	9,71	0,428	0	120	4,5	30,66	0,789
6,937	1,704	9,853	0,551	0	480	4,5	60,2672	6,143
2,629	3,288	1,638	0,428	4,5	197,143	4,5	152,5664	0,932
5,917	5,061	6,341	0,428	0	188,571	4,5	400,008	0,066
2,612	1,273	6,275	0,066	4,5	454,286	0	39,0012	6,789
5,575	1,284	0,132	0,551	0	120	1	55,944	0,789
5,196	1,418	0,209	0,369	0,789	274,286	0	26,88	0,066
4,834	1,053	17,198	1	4,5	180	1	10,626	4,643
4,349	1,787	1,787	0	4,5	300	0,789	121,282	4,643
6,435	3,156	1,209	0,428	0	171,429	1	130,606	4,5
10	2,722	8,51	0,786	4,5	274,286	4,5	14,35	1,789
6,647	2,433	4,932	0,428	0	154,286	1	40,754	0,143
3,832	1,913	5,562	0,786	0,066	334,286	4,5	98,9464	6,789
4,135	3,499	2,576	0,428	4,5	77,1429	4,5	0	0,789
4,557	1,416	6,066	1	0,066	180	4,5	0	0,429
4,478	2,007	1,921	0,428	4,5	0	0,143	98,4242	1,143
9,787	3,442	10,566	0,856	0	137,143	0,789	92,4112	4,643
5,006	2,205	7,499	0,123	4,5	360	1	470,022	0,143
2,909	3,222	2,642	0,786	0	300	6	108,0492	0,789
5,135	3,207	13,427	0,428	0	154,286	FAŁSZ	65,646	0,143
5,493	1,996	6,139	0,066	0	548,571	4,5	33,4936	6,143
9,08	1,284	4,51	0,428	0	240	1	363,272	4,643
8,354	1,715	3,987	0,123	0	137,143	1	31,8976	1,218
3,841	0,77	2,075	0,428	0	480	1	170,3422	0,143
8,519	1,207	0,352	0,428	0,066	205,714	6	237,2412	0,789
4,708	2,57	1,064	0	0	360	1	900,06	0,572
3,271	3,222	4,578	0,428	1	68,5714	1	252,1372	4,643
4,359	1,93	1,141	0,428	4,5	291,429	4,5	78,6072	0,286
4,286	4,709	0,143	0,123	0	445,714	1	11,9952	1,066
5,495	1,495	0,132	0,123	0	300	4,5	15,1872	0,066
10,167	6,138	2,721	0,428	0,066	197,143	4,5	166,88	5,289
3,788	1,636	9,143	0,428	1,143	402,857	4,5	30,66	1,143
7,807	7,076	7,998	0,494	0,066	394,286	4,5	55,951	5,289
6,144	2,785	5,578	0	0	480	1	68,3354	1,143
5,221	3,945	5,361	0,428	0	300	0	0	4,566

9,651	1,35	6,075	0,428	0,789	377,143	1	44,828	1,218
4,845	2,559	7,218	1	0,066	531,429	4,5	480,004	0,932
2,964	2,139	0,704	1,214	4,566	265,714	4,5	67,6592	1,789
12,167	3,945	10,932	0,428	4,5	240	0	108,0072	0,572
4,278	3,576	6,275	0,123	0	300	0	24,528	1,578
3,359	2,776	6,341	0,066	0	240	0,066	76,8488	0,789
20,718	2,93	3,275	1	0,789	282,857	0	39,5892	6,789
7,274	1,721	0,066	0,786	4,5	600	4,5	86,884	6,143
5,72	1,93	2,429	0,428	0	214,286	1	48,958	1,143
6,22	1,141	3,787	0	1,066	137,143	4,5	13,44	5,289
4,564	2,073	3,207	0,428	0	197,143	0	96,0932	1,143
5,374	2,433	0,855	1,214	0	600	0	6,9972	0,789
9,925	2,855	0,429	0,123	1	265,714	0,789	1,344	4,643
7,082	2,216	12,998	0	0,066	360	0	183,288	5,289
7,969	2,35	9,787	0,428	0	197,143	6	91,4704	0,789
5,421	2,15	4,853	0,428	1	514,286	4,5	67,1006	1,789
4,77	0,143	1,064	0,123	4,5	240	0	1,344	0,789
3,063	2,653	1,578	0,428	4,5	300	4,5	9,324	5,289
6,06	2,502	5,218	0	0	137,143	1	116,6242	6,143
4,797	1,504	4,921	0,494	0,429	308,571	4,5	38,1024	1,578
4,403	2,348	0,407	0,428	0,066	274,286	4,5	30,002	4,566
7,304	6,215	4,143	0,428	0	180	4,5	18,102	5,289
3,337	6,916	11,218	0,428	0	282,857	0	0	0,143
4,715	1,196	4,578	0,428	0,789	205,714	0,789	22,3272	4,643
7,223	2,787	0,789	0,428	0,789	394,286	0	0	0,932
2,436	2,007	7,141	0,428	4,5	197,143	0	11,8356	1,578
4,858	1,561	5,143	0,428	1	180	4,5	32,6424	1,789
7,075	2,932	4,442	0,428	0	240	0	43,442	5,289
4,08	1,855	0,352	0,123	0	394,286	1	10,1472	4,643
4,907	1,919	0,998	0,428	0,789	428,571	4,5	103,3004	0,789
4,282	1,853	13,007	0	0	231,429	0	56,3696	4,643
8,596	1,416	5,367	0,428	0	274,286	0,789	94,6232	1,143
2,054	1,418	2,132	0,786	6	480	1	20,6556	6,143
5,944	2,367	2,143	0,428	1	377,143	0	4,662	4,643
5,651	2,631	5,13	0	0	137,143	4,5	102,8468	4,643
6,361	1,93	2,143	0,123	0	137,143	0	48,9272	4,643
5,794	3,071	4,132	0,428	1	180	1	127,288	1,578
7,862	2,864	4,075	0,428	1	180	0,789	180,012	0,858
6,726	1,853	1,198	0,428	0	300	0,789	628,0064	1,066
6,867	2,576	5,786	0,428	0	120	0,429	176,792	4,5
5,858	1,996	5,57	0,428	0	180	4,5	42,322	1,143
4,359	1,998	4,286	0,428	0	377,143	0	0	5,289
5,418	1,143	0,275	0,428	0	445,714	0,143	318,6792	0
3,779	1,209	3,286	0,428	1	102,857	0	18,0012	1,066
3,999	2,273	4,275	0,786	1	214,286	0	35,0392	5,289
3,082	1,075	1,578	0,428	1	411,429	0	8,6604	0,789
3,337	1,284	1,853	1,123	0	428,571	1	4,6788	1,143
4,585	0,847	3,275	0,428	0,789	180	4,5	43,05	4,643

3,06	0,627	13,064	0	0,143	385,714	4,5	81,5892	5,289
3,341	0,704	1,93	0,856	0	385,714	0,789	15,8256	6,789
9,512	2,922	2,209	0	0	180	1	91,98	5,289
2,777	1,55	12,638	0,066	0	514,286	1	4,662	4,643
8,302	5,523	4,076	0,428	0	240	4,5	123,298	1,578
2,777	1,053	7,141	0,123	0	205,714	1	155,5428	4,566
3,992	1,064	1,075	0,428	0,066	360	0,789	45,1192	0,143
7,227	2,499	2,361	0,428	0	111,429	1	10,752	4,643
4,502	0,429	7,075	0,786	0	197,143	0	0	4,5
4,282	3,578	0,998	0,786	0	180	4,5	1,6128	0,789
4,063	0,704	0,932	0,428	0,789	368,571	1	96,7344	0,143
5,506	2,053	10,284	0,066	4,566	214,286	0	256,55	6,143
4,992	2,776	6,066	0,856	1	222,857	4,5	48,7032	5,289
2,779	1,787	6,709	0,123	4,5	60	1	10,9788	0,143
6,438	2,785	4,418	0,786	0	120	1	78,6072	0,143
8,178	2,787	5,787	0,428	0	411,429	6	285,3564	0,143
6,726	3,576	2,561	0,123	0	480	4,5	117,3172	6,789
8,805	1,427	0,495	0,428	0	385,714	6	19,3368	0,143
6,139	1,985	5,433	1,428	0	222,857	1	89,3284	1,218
9,797	1,053	1,721	0,551	0	462,857	6	72,0048	5,289
3,997	1,495	1,493	0,428	1	445,714	0	54,0036	0,932
3,272	1,427	7,932	0,123	4,643	360	1	61,32	0,066
4,421	4,775	1,141	0,428	4,5	360	0	32,0992	1,578
4,216	1,79	5,5	0,428	1	68,5714	1	0	1,429
3,566	2,721	5,433	0,428	4,5	497,143	1	195,2636	4,643
3,783	6,353	6,352	0,123	0	214,286	1	11,662	6,143
1,397	1,985	6,075	0,123	4,5	334,286	1	9,408	4,643
5,843	2,139	5,719	0,428	1,066	334,286	0,789	6,9972	1,578
5,215	1,921	2,132	0,428	1	180	4,5	30,002	1,789
5,478	2,007	2,504	0,786	0	308,571	0,066	19,9122	0,143
3,283	2,284	11,495	0	1	385,714	6	0,2268	1,066
3,981	3,585	3,787	0	0,143	265,714	4,5	51,7552	5,289
4,495	6,342	5,064	0,428	0	445,714	1	74,8832	0,932
3,337	2,561	14,341	0,428	4,5	60	4,5	213,248	5,289
5,629	4,709	1,141	0,551	4,5	411,429	1	14,9184	0,143
5,792	1,57	8,073	0,428	0,066	154,286	0,143	34,7844	1,143
2,576	2,433	1,275	0	1,789	77,1429	6	3,066	1,143
2,832	6,496	6,855	0,428	0	274,286	1	15,8592	0,143
7,282	2,642	3,987	0,428	0,066	300	0,789	169,946	1,578
2,623	2,721	9,13	0,066	0	257,143	1	1,344	0,066
3,141	0,209	2,143	0,246	1	38,5714	1	134,8732	6,143
4,429	0,638	0,275	0,123	0	257,143	0,143	6,72	4,643
4,858	2,576	11,932	0,551	4,5	300	4,5	26,88	0,143
4,346	0,418	8,407	0,123	1	180	4,5	118,9524	1,143
5,077	2,928	2,572	0,428	0,066	77,1429	1	136,626	1,143
7,416	2,216	2,286	0	0,209	62,1429	0	160,0088	4,643
7,858	0,275	4,5	1,214	1	342,857	1	71,3286	1,143
4,205	2,352	2,855	0,123	1	137,143	1	46,62	4,643

3,703	0,352	6,998	0,428	0	634,286	0	173,873	0,789
3,981	1,996	1,418	0,551	0,789	428,571	0	39,984	1,578
6,592	1,787	2,638	0,428	0	94,2857	0,789	153,244	1,218
6,353	2,139	2,427	0,189	0,143	445,714	1	90,6444	0,789
6,339	3,431	2,064	0,428	1,143	317,143	1	553,9632	6,143
7,084	3,51	8,007	0,428	0	154,286	1	67,298	5,289
3,783	5,641	5,416	0,312	1	385,714	0	25,3344	0,143
0,066	0,495	4,709	0	0	0	4,5	28,224	0
8,665	2,708	4,585	0,428	0	128,571	4,5	0	5,289
3,055	2,796	4,864	0,428	0	411,429	0	85,946	1,143
10,218	1,064	2,367	0,189	4,5	120	0,066	66,6512	0,789
2,757	0,99	2,216	0,123	4,566	248,571	FAŁSZ	700	6,143
4,997	1,196	10,864	1	0	240	4,5	6,9972	0,789
5,203	1,119	2,939	0,428	4,5	445,714	1	145,2892	5,5
6,014	3,433	14,429	0,428	1,143	317,143	6	127,4672	1,578
18,852	0,789	0	0,428	0	60	0	172,5976	0,789
3,272	1,416	1,361	0	0,066	394,286	0	299,3424	1,143
3,559	1,779	5,284	0,786	0,143	317,143	0,143	38,542	0,143
7,313	1,284	1,075	0,066	0	265,714	1	87,976	0
6,361	1,35	1,13	0,856	1	214,286	0,789	11,6592	0,932
2,612	1,064	1,418	0,066	0	274,286	0,143	99,974	6,143
18,222	1,578	5,718	0,551	0	240	0,143	30,7272	0,789
3,335	0,561	0,418	0,428	0,066	180	0	60,004	5,289
3,772	4,011	5,222	1,428	1,789	428,571	1	136,626	1,578
3,104	1,93	6,216	0,066	4,5	360	1	80,6708	6,143
4,148	2,785	10,984	0,428	4,5	154,286	4,5	134,057	6,143
7,086	1,504	2,275	0,428	0	360	0	30,002	4,643
6,932	2,776	1,721	0,123	0	385,714	0	106,624	5,5
7,027	2,359	0,932	0,856	0	291,429	4,5	0	1,143
5,271	5,564	2,352	0,428	1	205,714	4,5	22,4028	0,143
5,726	1,218	7,361	0,428	0	394,286	1	1,344	1,143
1,735	1,93	3,35	0,428	0,789	274,286	1	29,5596	1,578
5,64	0,352	1,921	0,123	0,789	257,143	1	8,1732	0
8,135	8,57	9,781	1	1	600	1	116,886	4,643
3,398	0,913	7,198	0,123	4,5	385,714	4,5	30,002	6,143
3,911	3,508	10,361	1	0,143	214,286	4,5	125,0816	0,855
3,786	3,367	19,218	0,786	0,143	334,286	6	0,756	5,289
5,441	1,416	5,504	0,428	0,143	154,286	1	125,944	1,143
7,618	6,342	3,284	2,5	0	360	4,5	87,976	0,789
3,871	3,082	5,064	0,428	6	180	6	146,622	5,289
2,527	2,576	5,075	0,428	0,143	154,286	1	34,7844	0,932
6,08	2,787	9,721	0	0,429	94,2857	1	54,3032	0,143
6,374	0,998	2,275	0,428	0	197,143	1	104,902	4,643
4,183	0,987	3,141	0,123	0	265,714	4,5	58,7552	6,143
3,117	1,196	21,207	0,428	1	111,429	0	2,688	0,789
3,643	1,416	2,921	0,428	4,5	471,429	4,5	88,6452	0,143
5,271	2,565	1,207	0,066	0	60	0,143	0	0,143
4,781	2,499	4,998	0,428	0	240	4,5	59,6792	0,789

3,349	1,636	2,132	0,428	0	600	0,789	279,888	1,789
6,073	1,35	1,864	1	0,066	565,714	0	45,99	0
3,64	2,139	0,789	0,428	0	342,857	0	78,82	0,143
8,528	2,062	3,998	0,428	0,066	240	0,143	85,5568	0,143
5,135	2,708	12,15	0,428	1	154,286	4,5	43,442	6,789
5,553	2,433	5,132	0,066	1	480	4,5	513,24	6,789
5,364	1,996	6,484	0,428	0	325,714	4,5	55,2972	0,143
4,043	2,284	7,924	0,428	0	231,429	4,5	0	0,789
3,489	2,359	4,561	0,856	0,143	377,143	4,5	101,5868	1,789
8,009	1,789	1	1	0	205,714	4,5	115,374	0
6,996	0	3,847	0,428	1	394,286	4,5	114,3002	1,578
8,002	1,504	17,487	0,786	0	531,429	4,5	5,5944	0,066
4,071	1,853	0,209	0,246	1	0	4,5	43,442	0,932
11,017	4,365	9,35	0,428	0,143	180	0	273,224	6,789
4,425	0,275	4,653	0,428	0	317,143	4,5	12,726	0,789
8,442	4,077	6,284	0,123	0	120	0,789	126,8064	5,289
4,791	1,561	2,143	0,428	0,143	94,2857	1	360,024	1,789
5,221	2,721	4,578	0,428	6	368,571	0	56,882	4,643
2,109	2,433	6,196	0,123	0,429	265,714	4,5	163,268	1,218
5,632	1,209	5,787	0,428	0,429	128,571	0	49,5432	1,789
3,423	2,642	0,921	0,428	4,5	231,429	0,066	166,88	5,289
4,082	2,064	5,427	0,786	0	111,429	0	37,296	4,643
16,477	1,855	1,13	0,856	0	300	6	68,4068	4,643
6,207	2,075	2,932	0	0	77,1429	0	24,4692	5,289
3,627	0,921	0,209	0,123	0	120	1	35,378	6,143
5,287	3,139	7,932	2,5	1	385,714	4,5	134,6212	1,143
7,515	2,853	5,007	0,979	0	197,143	0,143	0	5,289
7,639	3,431	3,653	0,428	0	300	1	32,7992	0,066
4,764	1,71	5,13	0,066	6	497,143	1	31,0296	0,143
5,924	2,141	5,144	0,428	4,5	257,143	4,5	89,992	4,643
3,412	0,484	3,198	1	0	360	0,066	2,7972	4,643
4,579	2,207	2,853	0	0	480	4,5	5,6448	5,289
4,493	2,578	0,198	0,123	1	428,571	4,5	8,064	1,143
5,84	2,207	12,207	0	0	205,714	4,5	76,8488	5,289
2,612	1,853	1,787	0,428	0,789	180	4,5	35,8176	1,578
12,926	0,979	1,789	0,428	0	214,286	0	41,5604	0,789
7,571	0,572	1,93	0,428	0	300	0	19,0092	1,143
3,192	6,132	2,198	0,428	0	428,571	1	30,002	0,789
6,653	6,573	9,864	0,428	4,643	240	0,066	76,622	4,643
6,297	2,433	3,075	0,428	0	334,286	4,5	107,9904	0,789
8,708	1,93	3,275	0	1	162,857	1	13,7088	6,143
4,336	1,35	0,264	1,214	0,066	291,429	0,066	69,867	0,932
6,561	2,787	2,286	1,284	1	162,857	1	43,9964	0,789
4,99	1,713	1,075	0,428	0,429	411,429	4,5	95,27	6,789
3,478	1,493	3,15	0,428	0	171,429	6	96,9304	6,789
6,271	3,578	9,365	0,123	0	394,286	1	191,646	5,289
4,645	1,207	0,855	0,246	4,5	180	1	81,634	4,643
4,071	0,932	1,352	0,246	0	548,571	0,143	38,1192	1,143

4,564	0,55	8,566	1	4,566	308,571	1	85,946	1,066
3,922	1,57	8,367	0,428	0	162,857	4,5	481,32	0,143
6,43	2,482	15,153	0,856	1,066	162,857	4,5	0	0,429
4,341	1,207	14,209	0,856	1	454,286	6	250,0232	0,789
4,283	0,921	2,066	0,428	0,143	197,143	0,143	83,9664	0,932
4,863	3,082	3,721	0,428	1	214,286	4,5	0	1,143
5,414	3,288	11,293	0	0,429	197,143	1	390,012	6,143
3,717	3,156	2,495	0,428	0,789	248,571	1	109,6284	5,289
2,263	2,073	16,507	0	1	214,286	6	0	0,143
3,58	2,064	8,367	2,5	0,143	274,286	1	96,0372	5,289
3,425	1,855	2,855	0,551	1	377,143	1	35,378	4,566
7,218	2,495	6,578	0,786	4,929	154,286	0,789	0	5,289
4,442	1,932	5,429	0,428	6,066	240	4,5	78,652	0,143
3,052	1,064	0,855	0,066	0	600	4,5	37,3072	0,789
3,928	1,71	2,066	0,123	1	137,143	1	156,128	0,932
4,623	1,339	7,796	1	1	385,714	1	141,3328	5,289
5,249	1,196	2,721	1,123	1	240	0,789	590,3212	4,643
4,251	1,427	2,396	0,428	0	368,571	1	183,302	1,066
5,779	2,776	6,427	0,428	0,066	360	4,5	47,1912	0,789
4,347	2,064	3,932	0,786	0	214,286	4,5	139,3252	6,789
2,615	3,794	3,776	0,066	0,066	420	0	166,334	0,143
6,227	0,638	1,919	0	0	325,714	1	276,584	0
4,417	1,996	1,932	0,428	1	102,857	0,066	121,324	1,143
8,563	2,853	14,147	0,786	0,066	394,286	0,143	81,8076	0,143
4,924	3,156	2,218	0,428	0,789	702,857	0,066	94,9732	0,932
3,5	2,141	1,275	0,066	1	214,286	4,5	30,8084	6,143
7,37	3,499	1,715	1,123	0	205,714	1	261,6208	0,789
2,417	1,858	0,484	0,123	0,066	171,429	0	13,5324	0,143
4,418	0,921	0,066	0,428	0,066	282,857	0	30,2848	0,066
15,943	2,007	1,721	0,123	1,066	325,714	1	40,3452	5,289
3,654	2,15	11,075	0,123	4,5	240	4,5	43,442	1,578
7,001	2,073	7,141	0	1	180	4,5	39,858	0,143
5,067	2,862	6,484	0	0,066	600	4,5	151,606	5,289
4,285	3,15	4,064	1	1	317,143	1	40,754	4,643
2,348	1,853	10,498	0,428	0	240	4,5	54,0792	0,066
8,261	0,55	2,787	0	0	120	0,066	4,662	1,789
4,124	5,355	4,198	0,123	0	231,429	4,5	15,414	0,143
10,362	1,504	2,143	0,428	0	55,7143	1	13,986	1,143
5,661	2,576	3,721	0,428	0	257,143	4,5	22,414	0,143
5,138	1,416	1,919	0,123	0	197,143	1	54,53	0,932
5,429	2,642	4,075	0	0	111,429	1	13,5016	0,789
4,137	2,073	3,073	0,428	0	420	1	0	5,289
3,794	4,852	8,495	0,123	0	274,286	4,5	15,414	6,789
2,689	2,796	3,198	0,066	0	548,571	1	42,322	6,143
6,073	1,207	9,143	0,428	0	308,571	4,5	183,302	0,066
5,43	1,93	1,275	0,123	0,572	274,286	0,143	206,612	4,643
4,149	1,93	0,627	0,428	0	214,286	1	87,3292	1,143
10,011	2,367	4,429	0,786	0	300	0,066	0	5,289

5,126	6,276	1,286	0,428	1	514,286	4,5	52,528	0,066
7,123	6,342	6,273	0	0	325,714	6	109,4492	5,289
6,593	2,653	4,15	0,428	1	300	1	120,652	1,578
6,506	8,153	6,075	0,428	4,5	265,714	6	5,376	5,289
9,163	2,284	15,855	0,428	4,5	488,571	0,789	143,9872	0,143
6,148	1,853	1,275	0,428	0	351,429	4,5	32,69	4,643
6,504	2,499	10,073	0,428	1	480	4,5	83,6192	1,143
3,148	2,433	6,932	0,123	4,5	274,286	4,5	500,024	6,143
3,629	2,776	7,007	0,428	4,5	94,2857	4,5	19,9416	5,289
8,906	1,93	4,693	0,123	0,429	154,286	1	84,532	6,143
5,135	2,653	11,075	0,428	0	197,143	4,5	79,4192	6,789
5,074	5,63	4,218	0,428	0	351,429	0	15,414	5,289
8,17	2,939	0,855	0,428	0	720	0	93,24	5,289
4,491	13,786	2,504	0,428	0,789	162,857	1	158,8188	1,578
6,917	2,642	0,341	0,123	0,143	257,143	1	122,5952	4,643
6,715	1,864	1,429	0,428	4,5	180	FAŁSZ	10,038	1,578
3,904	1,207	0,132	0,428	0	454,286	4,5	147,3164	0,143
3,632	2,064	4,218	0	4,5	454,286	0	33,726	4,5
4,781	1,493	2,207	0,979	0,143	240	1	62,6444	1,578
6,732	3,222	3,51	0	4,5	111,429	1	29,6632	5,289
4,632	0,066	0,286	0	1	411,429	1	59,3264	1,143
6,592	5,707	3,286	0,428	4,5	325,714	1	110,4432	5,289
6,069	2,631	7,207	0,428	0	154,286	4,5	26,7232	1,066
4,442	0,855	2,429	0,246	0	565,714	0	51,282	0,143
5,704	2,996	11,075	0,428	4,5	377,143	4,5	279,3812	5,289
4,847	3,071	5,853	0,066	0	480	4,5	182,6244	0,143
9,006	1,776	1,715	0,428	0	300	1	71,176	0,855
3,572	2,721	1	0,428	0	94,2857	0,143	0	1,789
11,949	1,218	3,578	0,428	1	205,714	1	36,0024	1,143
5,267	6,43	1,578	0,428	1,143	257,143	4,5	118,6612	0
5,071	1,35	9,35	0,428	4,5	300	6	150,3572	5,289
5,205	1,352	1,578	0,428	0	334,286	0	0	5,289
4,838	7,432	2,787	0,428	4,5	480	1	164,6232	0,789
9,574	2,576	1,787	1,214	1,066	162,857	0,066	27,972	0,143
4,272	2,282	6,71	1,214	6,066	257,143	4,5	10,038	6,789
5,555	1,996	1,416	0,428	0,066	257,143	1	3,8724	1,143
5,924	1,853	6,141	0,428	0	197,143	4,5	6,132	1,143
5,911	6,353	8,998	2,5	0,143	120	1	27,972	4,643
3,508	4,643	0,209	0,123	0	79,2857	0	0	0
2,615	0,418	0,286	0,909	0,789	162,857	0	3,6792	0,932
4,994	1,064	2,141	1	0	205,714	1	0	4,643
3,86	0,132	0,932	0,123	0	197,143	1	6,5016	0,143
4,772	0,275	12,427	0,428	4,5	197,143	0	1,8396	0,932
6,365	1,075	3,71	0,428	1	265,714	4,5	40,6644	0,066
4,223	1,361	0,352	0,123	1	137,143	1	4,9056	0,143
3,458	1,787	4,075	0,066	0	360	1	5,4852	4,643
6,507	2,939	2,495	0,674	4,5	120	0	69,972	1,578
5,207	2,433	0,781	0,428	4,5	394,286	1	18,0012	0,789

7,917	0,341	1,93	0,428	0	240	0	0,8064	0,143
5,935	0,209	1,787	1,572	1	137,143	0,789	0	0,143
4,014	1,001	7,855	0,428	1	377,143	1	0	1,578
4,695	1,273	10,921	0,189	0,789	137,143	1	0	0,932
4,786	4,709	0,132	1	0	222,857	1	6,72	4,566
5,339	1,209	3,066	0,856	0	90	0	0	0,066
4,651	4,907	3,075	1	1	137,143	4,5	0	0
6,01	1,864	5,075	1,214	1	180	4,5	1,8396	0,143
2,994	4,643	2,855	0,428	0	257,143	1	0	0,789
4,787	0,855	1,361	0,428	1	257,143	1	1,6128	0,143
4,138	1,284	8,932	0,786	0	300	0	0,8064	6,066
7,456	1,495	3,075	1	1	360	1	6,9972	0,143
5,291	11,421	2,721	0,786	0	480	4,5	0	5,289
9,571	5,564	4,218	0,786	0,066	257,143	1	1,6128	5,289
2,616	1,341	5,852	0,189	1	205,714	0	1,8396	1,066
4,274	0,627	2,064	0,786	0,789	180	1	50,6352	1,066
3,9	1,776	0,998	0,428	0	180	1	18,0012	0,855
4,623	0,132	0,855	0,123	1	162,857	1	0	0,932
6,716	2,139	7,147	1,214	0	257,143	0,429	3,2256	0,789
3,7	1,141	0,132	0,123	0,066	360	0	0	6,789
7,566	0,627	3,721	0,246	4,5	120	0,789	2,3184	4,643
5,047	1,416	2,713	0,428	0	282,857	0	2,688	5,289
7,574	2,853	1	0,428	0	360	4,5	12,9696	1,066
4,994	1,286	3,209	0,428	4,5	377,143	1	2,688	0,143
6,293	1,418	4,286	0,786	0,143	317,143	4,5	0	4,643
5,649	1,064	7,578	0,066	1	342,857	1	0	0,932
9,573	1,286	4,209	0,428	0,429	420	1	57,974	5,289
4,368	2,653	8,789	0,428	0,143	317,143	4,5	0	4,643
3,621	0,627	1,71	0,428	0	240	1	0	0,143
5,724	2,565	2,064	0	0	265,714	4,5	55,2972	0,066
6,37	1,064	0,429	0,856	0	360	1	25,6452	0,066
12,024	3,299	1,864	2,5	0	154,286	4,5	14,2548	5,289
3,775	2,433	0,855	0,428	0,789	300	1	0,8064	1,143
5,014	1,262	0,781	0,551	0,429	180	1	72,5312	1,789
3,559	0,132	1,066	0,123	0	68,5714	4,5	0	0,143
8,716	0,561	1,286	0,786	4,5	411,429	0	0	0,789
7,148	4,841	13,632	2,928	1	240	0,429	7,476	4,566
8,125	1,71	0,789	0,551	0,789	137,143	1	0,672	0,143
4,357	1,273	3,367	0,617	1	154,286	1	5,1282	0,932
2,15	0,855	1,066	0,123	0	180	0,789	0,756	4,643
6,432	2,642	3,132	0,428	4,5	162,857	0	14,35	4,5
8,223	1,273	1,075	0,428	1	162,857	0	2,1504	0,789
13,709	1,352	1,418	0,198	0,789	137,143	1	19,74	0,932
1,68	1,853	1,721	0,428	0	420	0	0	5,289
8,648	1,427	2,855	0,428	4,5	282,857	1	26,663	0,789
4,058	0,781	1,075	0,428	1	282,857	1	0	4,643
4,993	2,216	0,627	0,428	1	214,286	4,5	0	5,289
8,89	2,367	4,367	0,428	0	300	1	0	1,578

1,693	0,932	0,143	0,066	0,066	162,857	1	4,032	4,643
9,514	1,143	1,493	1,428	4,5	214,286	1	5,5944	0
12,282	1,495	2,352	0,786	4,5	197,143	0,143	0	4,643
11,426	1,919	3,064	0	0,429	180	1	5,376	0,066
7,372	2,708	4,996	0,428	0,066	240	1	3,4944	1,218
7,438	1,198	0,066	0,123	0	120	1	0	0,143
0,825	0,341	2,561	0,428	0,789	240	1	8,064	1,578
7,296	0,781	4,436	0,428	1	600	1	19,2024	0,789
4,432	1,853	4,841	0,856	0	197,143	4,5	8,1732	4,643
5,06	2,785	2,218	0,428	0,789	137,143	1	0	1,143
10,151	1,787	1,855	0,123	0,789	240	0,789	0	0
4,638	1,559	1,416	0,123	4,5	360	4,5	1,344	6,789
4,638	0,286	5,57	0,428	0	428,571	4,5	9,863	1,143
2,491	1,207	3,275	0,123	0	205,714	1	6,132	1,143
7,366	1,207	0,998	0,428	0	214,286	1	6,9972	0,143
6,072	1,141	1,064	0,123	0	214,286	0,143	17,8584	1,143
5,941	0,638	0,789	0,123	0	325,714	4,5	2,688	0,143
3,64	1,578	0,209	0,123	0,789	60	0	2,5956	0,143
8,912	1,987	3,987	0,551	0,066	274,286	0	0	0,143
7,783	0,921	1,932	0,909	0	180	1	2,331	0,066
2,843	1,064	1,578	0,428	4,5	180	0,143	150,8164	0,932
4,992	0,77	4,275	0,551	0	240	1	5,6448	1,578
3,988	1,781	1,218	0,428	0	180	4,5	6,8292	0
5,221	1,141	0,352	0,428	0	300	0	12,3648	1,578
5,933	0,561	4,141	0,786	0,789	317,143	1	15,0024	0,143
9,149	1,361	4,647	0,428	0	300	0	0	4,566
9,888	1,427	1,13	0,123	0	265,714	1	39,326	0,143
6,641	2,143	0	0,123	0	222,857	4,5	0	4,643
4,117	1	0,921	0,123	1	300	4,5	0,756	1,066
5,566	1,352	5,207	0,123	0	240	1	0	1,143
3,557	1,064	2,286	0,428	4,5	180	0	4,9056	0,789
6,062	0,484	5,141	0,428	0,066	257,143	0	0	0,286
6,227	2,787	6,13	0,786	0	428,571	1	9,324	4,643
9,29	2,565	4,786	0,428	1	120	0	2,331	0,932
5,623	0,209	4,341	0,428	0	360	1	4,9056	0,789
6,223	1,842	3,789	0,856	0	334,286	1	8,9292	5,289
8,411	0,275	0,789	0,123	0	137,143	0	2,1504	0,066
2,205	1,787	0,286	0,551	0,789	240	1	30,0426	0,143
4,005	0,429	0,132	0	0	360	4,5	1,8396	0,143
10,292	0,264	1,284	0,246	0	282,857	0,789	13,9944	0,143
4,92	1,644	2,064	0,428	1	600	1	10,2816	1,578
4,48	0,429	1,132	0,123	1	162,857	1	18,0012	0,143
6,438	2,796	4,638	0,428	0	385,714	1	2,688	5,289
3,636	2,787	0,855	0,428	1	197,143	1	38,2284	0,789
6,054	1,559	3,787	1,572	0,066	282,857	0,429	3,8724	1,789
5,724	1,787	6,932	0,428	0,789	240	4,5	0	0,789
9,497	2,642	0,286	0,856	1,066	120	0,789	18,648	1,143
5,977	1,57	3	1	0	600	1	2,688	0,286

3,478	2,71	2,932	0,428	1	300	0,789	0	4,643
6,99	2,93	3,209	0,856	4,5	137,143	0	0	4,643
4,906	2,499	0,998	0,494	1	300	4,5	4,1412	0,143
25,507	1,353	3,644	0,909	0,429	162,857	1	0	0,932
6,509	1,721	0,998	0,428	4,5	411,429	1	9,6852	0,932
2,703	0,998	0,352	0,428	4,5	120	4,5	0	0
4,407	1,273	0,143	0,428	0,066	120	0,789	0,8064	0,143
5,906	1,721	4,932	0,428	1	368,571	1	12,1212	1,143
19,629	2,499	1,341	1,428	1	180	0,789	7,5264	0,066
2,48	1,427	3,341	0,123	1	420	4,5	3,8724	4,643
3,937	0,418	3,064	0,123	4,5	240	4,5	24,3852	0,143
4,704	1,429	3,352	0,066	0	257,143	0	0	4,643
3,719	4,709	3,209	0,123	1	334,286	0	0	0,143
3,772	1,427	0,352	0,066	0	360	0,066	0	0,789
6,634	5,63	1,71	0,428	0	222,857	0	0	6,143
5,429	1,13	8,073	0,123	0	120	1	11,1636	1,789
6,276	1,776	2,209	0,428	0,789	180	0,789	4,5276	0,789
9,935	2,062	4,787	2,5	0	360	0,066	14,35	5,289
9,422	1,416	0,209	0,428	1,066	60	0	18,095	4,643
2,975	4,995	0	1	0,789	360	1	3,6792	1,789
7,647	1,064	0,484	0,786	0	300	0	2,688	0,143
1,284	1,71	2,932	0,123	0,789	180	1	12,264	5,289
5,796	4,566	0,209	0,066	0	205,714	1	8,8704	0,143
5,998	1,141	1,286	0,123	0	137,143	0	2,7972	0,066
4,047	1,855	1,998	1,786	1	377,143	0	58,3072	0,209
18,004	0,921	0,932	0,066	4,5	325,714	0	0	4,643
3,632	1,919	2,064	0,428	0	420	1	12,1212	0,066
8,141	1,427	1,787	1	0,789	514,286	4,5	38,6904	0,066
6,349	0,198	1,198	0,123	0	300	1	1,5708	4,643
5,078	2,073	6,218	0,428	0	300	1	0	0,789
10,04	0,352	0,998	0,428	0	60	0,143	0	0,789
15,416	1,998	0,341	0,551	4,5	102,857	0	6,9972	1,066
6,498	1,284	1,143	0,428	0	342,857	1	4,5276	0,143
1,996	1,13	3,433	0,428	4,5	300	1	0	5,289
5,006	2,51	1,075	0,551	0	120	1	0	1,143
10,238	1,141	1,075	1,123	0,789	180	1	4,9056	0,143
14,003	2,073	2,284	0,428	0	120	0	36,0024	0,789
6,357	0,77	2,066	0,066	0	360	0	1,8396	0,143
0,737	0,341	3,141	0,428	0	480	0,789	27,972	1,578
6,48	3,148	5,996	0,428	0	274,286	1	13,44	4,643
6,374	1,853	3,855	1,642	1	240	1	30,002	4,643
4,977	1,996	3,862	0,428	1	420	1	68,9822	0,855
5,717	1,141	4,495	0,428	0	162,857	1	13,0704	0,143
4,654	1,066	0,789	0,428	0	188,571	1	0	0
7,704	2,216	4,578	0,856	4,5	317,143	0	4,8384	0,143
6,502	1,921	0,352	1,572	0	360	1	4,4352	0,789
3,404	1,209	8,565	0,786	0	214,286	1	4,032	0,495
8,08	3,365	9,643	1	0,932	171,429	1	11,662	5,289

4,539	2,576	0,418	1,123	1	222,857	1	37,2176	1,789
6,872	2,139	2,932	0,428	0	120	1	0	4,643
4,708	1,932	2,932	0,123	4,5	180	0	0,8064	4,643
7,359	1,853	1,275	0,428	0,143	480	0,789	9,3156	5,289
3,843	1,561	7,064	0,786	0,789	257,143	1	34,5324	5,289
1,757	1,064	1,132	0,786	0,429	282,857	1	14,1036	5,289
9,068	1,341	2,35	0,786	0	300	1	0	0,143
4,642	0,286	2,209	0,123	1	222,857	1	2,7972	1,143
2,994	0,932	10,275	0,428	0,789	300	0	0	5,289
6,282	2,062	2,132	0,428	0,789	154,286	1	0	5,289
7,429	1,064	1,721	0,494	1	240	0	4,5276	5,289
4,695	2,367	1,066	0,123	1	342,857	0	0	6,066
3,654	1,075	4,286	1	0	120	1	0	0,143
5,06	1,855	8,141	0,856	1	240	4,5	12,1212	1,789
3,423	1,198	4,352	0,123	0	651,429	1	9,0552	0,286
5,067	2,139	0,998	0,428	0	240	0,789	0	1,789
8,771	2,367	7,396	0,786	0	411,429	0,789	4,9056	4,643
13,365	1,787	2,998	0,856	0,789	102,857	1	197,9852	4,643
2,199	0,704	1,064	0	1	480	1	4,5276	1,789
5,287	1,275	0,286	0,428	0	197,143	4,5	43,3524	1,789
4,997	0,913	0,572	0,856	0,495	137,143	4,5	33,3144	0,143
4,135	2,139	2,987	0,123	4,5	240	0	1,8396	0,789
6,572	2,653	6,495	0,066	1	94,2857	1	5,5944	1,143
5,583	2,073	6,352	0,189	0	257,143	1	13,7172	1,143
8,08	0,855	0,429	0,428	1	120	0,789	2,688	0,143
3,86	0,341	0,998	0,066	0,789	334,286	1	7,5936	0
6,79	2,576	2,064	0,428	0	342,857	1	8,2824	0,932
2,623	1,064	7,053	0,786	0,789	180	0,066	18,2112	1,143
3,849	1,13	2,921	0,123	0,143	317,143	1	0	1,789
6,796	1,57	0,198	0,123	0	325,714	0	2,7972	1,578
5,358	0,987	0,209	0,428	0	240	1	0	4,643
3,06	0,987	1,429	0,123	0	154,286	1	46,7544	1,143
7,135	1,842	0,561	0,066	1,066	240	1	6,9888	4,643
10,008	1,71	2,209	0,428	1	240	1	2,7972	1,066
10,31	1,495	6,932	0,428	4,5	197,143	1	0	4,643
6,629	2,367	0,561	0,674	0,789	248,571	0	13,9944	0
5,051	1,996	3,93	0,856	4,5	240	0	4,1286	5,289
5,482	1,787	4,572	0,428	1	180	1	11,7264	0,789
9,435	1,273	2,141	0,856	4,5	317,143	0	45,9732	1,578
7,306	0,921	1	0,428	0,143	240	1	0	0,143
6,164	3,156	7,289	0,428	0	214,286	0,789	37,296	4,643
6,998	0,561	5,855	0,428	0,143	180	4,5	0	6,429
4,779	0,987	0,209	0,066	0	120	0	6,132	4,643
10,072	1,855	4,066	0,428	4,566	334,286	0	18,396	1,143
4,359	0,341	1,209	0,066	1	274,286	0	1,344	0,143
5,498	1,493	2,143	0,428	0	325,714	0	1,8396	1,143
7,576	2,073	1,286	0,428	1	394,286	4,5	3,6792	0,932
5,509	2,642	2,275	0,123	0	180	1	4,662	0,789

2,216	1,721	7,789	0,428	0	377,143	0,789	0	1,143
6,507	0,55	1,921	0,786	4,5	240	1	0,8064	0,789
2,612	0,998	1,264	0,066	1	480	0	1,8396	4,566
5,575	2,073	6,418	0,123	0	257,143	1	6,8292	1,143
7,084	1,864	1,644	0,428	1	197,143	0	2,7972	1,143
8,932	1,855	6,001	0,786	6,066	480	1	8,3412	4,643
6,642	1,418	0,275	0,856	1	180	4,5	2,7972	1,789
6,867	1,141	1,207	1	0,789	102,857	4,5	7,4592	1,578
4,484	1,789	4,264	0,066	0	360	4,5	3,4944	0,143
6,796	2,576	2,578	0,428	1	300	0	0	1,578
4,127	0,396	2,987	0,428	1,066	257,143	0	2,2344	5,289
4,284	1,578	3,275	0,066	1	394,286	4,5	3,4944	0,143
5,489	1,143	3,209	0,428	1	222,857	1	11,6592	0
3,689	0,572	3,864	0,786	0	282,857	4,5	0	5,289
8,639	1,196	4,075	0,428	0	420	0,789	8,1564	1,789
3,574	1,93	5,286	0,428	4,5	197,143	0,789	22,3314	0,286
6,491	0,473	3,418	1	1,066	180	1	8,9712	0,066
7,307	0,198	1,35	0,551	1	257,143	0,066	7,9716	1,578
1,996	1,141	0,352	0,123	0	60	1	3,1836	0,066
10,576	4,852	6,715	0	0	257,143	1	3,1248	5,289
8,227	2,853	6,847	1	0,066	120	4,5	0,8064	4,643
5,02	1,864	0,789	0,786	0	360	0	0	1
3,131	0,921	1,143	0,428	4,5	257,143	0	37,296	1,578
7,146	0,858	1,361	0,786	0,143	180	1	47,1212	0,143
10,369	1,644	0,209	0,123	0	342,857	1	13,6668	0,066
6,15	1,207	3,007	0,428	0,855	222,857	1	2,5116	1,578
4,546	1,407	1,207	0	0,429	102,857	4,5	16,7244	0,572
5,212	1,787	1,495	0,428	1	137,143	0	0	1,143
6,573	1,352	2,433	0,066	1	240	1	18,648	0,143
7,429	6,078	11,932	0,551	4,5	180	0	6,72	1,789
8,306	3,51	4,284	0,428	4,5	300	0	41,7284	0,932
7,862	2,13	0,921	0,786	0	240	1	78,4896	5,289
11,564	0,572	7,064	0,428	0,066	300	0	0	0,143
7,293	1,13	9,207	0,856	1,578	360	1	11,662	0,932
4,138	1,075	3,075	0,428	0	257,143	1	0	0,789
4,981	2,218	0,33	0,786	0	257,143	4,5	31,6512	1,143
7,706	1,427	2,132	0,123	1	102,857	4,5	10,752	0,143
4,575	0,987	1,209	0,428	0,066	342,857	0	3,066	0,789
3,483	1,143	6,064	0,786	0	240	1	24,528	1,143
5,801	3,082	2,79	0,428	0,066	368,571	0	7,2156	1,143
8,372	2,367	1,932	0,428	0	171,429	1	4,032	0,932
7,622	1,361	0,275	0,428	0,429	214,286	1	0	6,789
3,917	2,499	7	1	0	180	4,5	16,7832	4,566
2,557	2,721	9,647	0,428	0	342,857	4,5	12,726	4,643
2,777	1,218	0,429	0,428	0	257,143	0	0	0,143
3,996	2,367	10,932	0,428	4,5	222,857	0	0	6,143
2,647	1,789	3,921	0,123	0,789	222,857	4,5	0	0,932
0,737	1,066	6,198	0,428	1	420	1	0,8064	0,066

5,281	2,073	2,132	0,428	1	120	4,5	170,3212	0
6,434	1,855	11,5	0	0,429	0	0	4,8384	1,143
14,932	1,71	1,864	0,551	0	180	1	0	0,789
7,66	1,864	2,286	2	4,5	120	1	6,5016	0,143
5,21	0,998	0,286	0,189	1	300	4,5	3,066	0,789
7,935	1,218	1,789	0,428	4,5	120	4,5	22,6632	0,143
4,074	1,361	2,789	1	0	180	1	0	1,789
13,141	0,932	1,352	1,572	1	300	0,789	21,4452	0,572
5,86	1,141	3,998	1	0	180	4,5	8,064	5,289
5,286	0,209	3,921	0,428	0	394,286	4,5	88,6284	0,286
1,328	1,284	4,132	0,428	4,5	428,571	4,5	14,1036	4,643
2,843	0,932	4,776	0,066	4,5	420	1	8,2824	6,143
7,715	0,77	0,341	0,123	1	282,857	4,5	6,9972	0,066
5,078	0,858	3,064	0,551	0	317,143	1	0	1,143
4,058	1,93	1,787	0,786	0,789	351,429	1	0	0,143
4,192	1,987	1,429	0,428	0	180	1	18,648	0,932
6,839	1,921	0,143	0,856	0	240	1	10,0632	0,143
4,431	1,064	0,495	0,123	0,855	411,429	0	32,7992	0,789
9,223	3,299	2,932	0,428	0,429	334,286	1	5,5944	1,143
4,489	0,561	1,132	1	1	222,857	4,5	4,5276	0,789
6,155	1,361	0,209	0,428	4,5	145,714	1	0	4,643
4,435	0,066	1,143	0,066	4,5	282,857	0	8,064	4,643
5,135	1,853	4,427	0,856	0,789	282,857	1	28,7784	6,066
5,849	1,198	9,132	0,428	0	334,286	1	6,5604	1,789
6,427	1,721	7,853	0,428	4,5	248,571	1	4,9056	0,066
4,869	2,064	0,932	0,428	0	180	0	0	0,789
4,06	1,57	4,35	0,066	4,566	240	4,5	8,064	4,643
6,803	1,075	0,286	1	0	42,8571	0	4,662	0
6,914	1,341	2,721	0,428	0	120	0	23,324	1,143
3,786	2,71	4,721	0,066	0,143	154,286	1	46,62	4,643
15,64	0,396	2,921	1,428	0	120	1	9,324	0,066
4,053	2,141	3,647	0,428	0,789	282,857	1	2,688	1,789
3,428	0,429	1,864	0,246	0	137,143	1	7,4592	0,143
4,711	1,141	2,075	0,123	1	60	0,143	13,5492	0,286
4,157	1,196	0,932	0,369	0	300	0,789	6,1824	5,289
6,153	2,062	3,998	0,428	0	120	1	2,856	1,789
3,569	0,789	0,495	0,123	0,789	77,1429	1	3,066	0,789
3,329	0,209	1,864	0,428	4,5	137,143	0	4,8384	0,066
8,941	1,284	1,13	1,284	4,5	120	0,789	17,3922	1,789
14,955	2,653	3,71	0,123	4,566	145,714	0	2,7972	0,143
4,926	0,921	1,132	0,123	0	60	4,5	7,3332	0,209
4,368	0,33	4,209	0,428	4,566	214,286	1	2,688	0,143
10,844	0,132	0,921	0,494	1	240	1	0	0,143
13,52	0,987	2,132	1	4,5	180	0	0,378	1,143
5,869	1,284	2,141	0,123	0	240	0	4,5276	0,143
5,906	1,198	2,998	1	0	120	1	0	0,143
2,557	1,71	0,132	0,123	0	77,1429	1	2,1504	0,143
6,801	2,862	6,264	0,428	0	385,714	1	0	4,643

6,928	1,504	7,93	0,123	1	120	0,789	0,672	1,789
6,062	0,921	2,361	0,428	1	120	4,5	6,1824	0,789
12,785	0,932	1,578	0,066	4,5	60	4,5	0	0
6,935	1,93	3,433	0,123	1,066	137,143	0,789	5,4852	1,218
5,572	1,064	1,721	0,428	4,5	248,571	0,789	10,9704	0,789
8,785	1,996	2,921	1	1	222,857	4,5	5,376	0,932
12,595	1,075	6,71	1,428	0,789	197,143	1	2,1504	0,143
2,604	1,493	1,207	0,246	0,132	222,857	0,429	16,506	0,286
5,866	1,71	1,855	1	0	162,857	1	0	4,643
5,148	2,721	1,352	0,428	0	214,286	1	4,032	1,143
4,71	2,216	1,066	0,428	0	214,286	0	0	1,143
4,729	2,064	4,286	0,123	0	240	0,789	27,972	1,143
7,221	0,209	0,715	1,214	0	137,143	1	1,1844	4,643
3,054	0,572	2,15	0,428	1	222,857	1	5,8632	5,289
4,847	0,932	0,066	0,123	0	514,286	0	0	0,143
6,565	1,921	0,495	0,428	0	222,857	1	6,5016	5,289
5,997	1,198	0,855	0,123	1	102,857	1	33,0022	0,132
9,206	1,996	0,495	0,123	0,209	68,5714	0	8,064	0,143
6,801	0,561	1,644	0,428	4,5	60	4,5	18,648	0,143
4,283	2,631	0,418	1	0	102,857	4,5	14,784	0,932
5,917	1,053	0,418	0,428	0,855	291,429	1	22,0416	0,143
3,767	1,921	0,264	0,922	0	180	1	2,1504	0,572
3,706	0,495	0,143	0,428	0	154,286	1	0	4,643
6,412	1,13	0,209	0,428	0	180	1	4,5276	0,143
5,803	1,853	4,998	0,786	0	240	4,5	0	0
5,565	0,198	4,786	0,428	0	360	1	0	0,855
4,928	1,644	0,855	0,123	1	222,857	1	1,8396	4,643
5,493	1,13	6,418	0,428	0	480	1	24,7324	4,643
3,854	0,921	0,209	0,066	4,5	257,143	1	5,376	1,218
7,218	1,996	6,053	0,123	0	197,143	0	0	4,643
4,431	1,998	0,855	1	0	317,143	4,5	30,7692	1,143
6,792	4,907	9,064	0,428	0,932	222,857	0,789	5,9724	0,932
2,832	0,998	0,855	0,786	0	137,143	1	13,608	0,932
4,193	1,561	1,064	0,856	0	274,286	1	0	5,289
7,666	4,566	14,078	0,494	0	120	1	6,8544	1,789
4,435	1,064	9,284	0,428	0	214,286	1	6,9972	4,643
3,717	0,789	1	0,066	0,789	60	1	2,9148	4,643
4,508	1,776	9,841	0,123	4,566	385,714	4,5	17,7996	4,643
7,95	1,853	0,275	0,428	0	120	0	25,3932	0,789
5,861	1,71	3,433	0,786	0,789	137,143	1	3,4944	0,789
6,206	0,987	1,644	0,856	0	120	1	22,0332	1,578
5,195	2,207	0,484	0,786	0	282,857	1	11,8776	0,143
6,3	1,864	1,209	0,428	0	222,857	1	18,396	4,643
5,267	1,064	2,275	0,786	0,066	240	4,5	30,3212	0,066
10,292	2,15	3,367	0,123	0	240	4,5	42,266	0,066
9,304	1,561	0,275	1,214	0	120	1	8,6604	0,789
4,574	1,13	1,143	0,786	1,066	214,286	4,5	34,7844	0,932
3,142	1,427	7,932	0,786	0	154,286	4,5	56,3556	0,932

10,65	1	0,066	0	4,5	394,286	6	16,3212	0
4,489	1,853	1,721	0,428	0	180	0,789	21,4452	4,566
3,481	1,218	0,781	1,214	0,066	197,143	1	18,7572	0,066
5,715	0,998	0,275	0,123	0	180	4,5	10,9704	0,143
6,781	0,572	1,987	0,551	0	257,143	0,789	5,376	0,789
5,423	1,864	2,561	0	4,5	300	0	10,038	0,789
1,977	1,218	1,141	0,066	0	360	0,066	0	0,143
2,626	0,209	0	0,428	0	445,714	1	12,264	0,143
4,761	0,781	1,209	0,066	4,5	300	1	0	0,143
3,717	2,198	2,638	1,428	4,5	282,857	1	12,6336	0,143
4,77	1,71	6,787	0,123	4,5	162,857	1	0	5,289
16,075	6,364	4,566	0,123	0	480	1	83,6472	0,143
7,071	1,075	0,341	0,428	0	737,143	1	2,7972	0,143
11,918	2,007	7,281	1	0	480	0	4,032	5,289
11,147	7,142	5,132	0,428	1	548,571	1	17,0268	5,289
5,484	3,499	1,209	0,428	4,5	120	4,5	8,064	6,143
5,928	2,996	4,218	0,428	0,066	137,143	1	6,9384	4,643
5,212	1,119	0,921	0,551	0	240	0,143	9,3282	0,066
5,207	0,561	8,066	1	4,5	240	1	3,1752	1,066
2,84	0,998	4,644	0,428	4,5	180	0	9,9036	0,789
6,135	1,064	3,198	0,428	1	120	1	1,8396	1,789
4,869	2,065	2,429	0,123	4,5	137,143	0	0	1,218
5,786	0,572	0,693	0,123	1,066	257,143	1	14,1036	1,143
3,491	0,143	0,132	0,123	0	300	1	2,7972	0,066
2,403	0,921	0,275	0,066	0,429	334,286	0	0	5,289
5,203	0,495	2,998	0,856	4,5	60	0,789	0,8064	4,566
5,862	1,429	9,143	0,428	0	240	1	23,324	1,789
2,172	1,341	13,721	0,123	4,5	480	4,5	0	6,143
3,219	0,627	2,361	0,786	0	180	4,5	0	4,643
2,502	0,998	0,855	0,123	0	274,286	1	0	0,789
7,372	1,636	6,638	0,856	1,143	240	0,429	15,0612	1,789
6,992	1,275	4,352	0,551	1,143	214,286	0	3,5868	1,789
4,346	1,787	1,721	0,551	0	240	4,5	5,376	0,932
7,357	0,341	0,198	3,5	4,5	188,571	0,789	48,3112	0,495
9,741	4,786	1,721	0,428	1	180	0	0	0,789
4,704	0,913	0,932	0,066	6	334,286	0,143	0	1,066
7,295	1,493	1,93	0,428	1	60	4,5	182,6244	1,143
6,006	1,352	1,218	0,428	0	480	1	0	1,789
9,077	2,282	5,141	0,123	4,5	180	1	5,8716	5,289
7,857	1,922	3,998	0,428	4,5	342,857	4,5	2,688	5,289
5,563	0,921	1,578	0,132	0	257,143	0,789	1,5624	0,143
16,08	2,15	6,432	0,786	0,789	180	1	0	1,218
6,067	1,787	0,198	0,123	1	300	4,5	11,662	0,143
9,428	1,286	4,932	0,428	1	197,143	1	1,8396	0,143
7,946	9,066	3,644	0,428	0	600	1	12,8352	1,143
5,493	1,493	5,35	0,909	0	171,429	1	11,2476	6,789
4,054	1,207	1,864	0,428	0,429	257,143	4,5	30,66	0,143
3,544	0,998	0,209	0,123	0,429	300	0,143	8,1732	0,143

3,91	1,787	3,284	0,786	0	240	1	1,9404	5,289
4,992	2,216	1,921	0,123	4,566	274,286	1	15,8256	4,643
6,504	0,77	6,93	1,214	4,5	282,857	1	27,972	0
8,846	0,77	3,638	0,786	4,566	240	4,5	137,8062	0,932
4,704	1,787	2,284	0,066	0,429	342,857	4,5	0	0,932
2,909	1,996	1,998	0,123	6,143	162,857	1	0	4,643
5,779	1,143	3,299	0,123	0,789	222,857	1	0	0,789
5,355	1,064	1,132	0,428	0	240	0,789	4,662	0,789
4,204	1,864	7,053	1	1	162,857	0	0	0,789
2,493	0,715	0,789	0	0	248,571	0,143	0	0,143
4,997	1,987	2,499	0,979	0	282,857	0,789	3,4944	1,578
13,286	2,51	10,429	0,428	4,5	300	4,5	6,7452	5,289
5,715	1,776	1,418	0,786	0,066	300	0	0	1,143
4,574	0,921	0,132	0,123	1	180	1	0,2268	1,143
12,164	0,286	0,066	1	1	60	0	0	0,066
4,344	1,207	3,198	0,428	4,5	171,429	0	7,8876	1,578
5,353	1,064	4,132	0,428	0	325,714	1	23,9904	0,932
4,798	3,51	8,51	0,428	4,5	197,143	1	0	5,289
22,807	2,433	2,361	0,428	1	120	0	21,0672	0,789
4,706	0,352	2,998	0,428	0,143	257,143	1	13,9944	1,789
8,012	2,576	1,207	1	4,5	0	0	1,8396	1,143
3,482	4,5	0,209	0,428	0,789	222,857	0	16,3212	0,789
5,583	0,066	2,418	0,428	0	222,857	1	2,7972	0
7,069	1,855	1,721	0	0	240	0	0,8064	0,789
5,21	1,209	0,341	0,132	0,789	214,286	0	0	0,066
6,794	0,638	1,998	1	0	205,714	1	5,5944	0,932
5,014	5,641	0	1	1	300	1	0	0,143
4,126	0,987	3,93	0,428	1,066	120	1	6,1824	5,289
5,058	0,429	0,132	0,066	0	360	4,5	25,6452	0
6,366	0,209	0,066	0,123	0	300	1	2,7972	0,143
5,579	1,919	0,932	0,428	4,5	300	4,5	22,7892	1,789
2,175	1,416	2,987	0,428	0,855	240	1	9,2484	0,143
1,133	1,853	0,998	0,123	0	240	1	23,3772	0,932
2,425	1,132	0,066	0,123	0,789	445,714	4,5	22,0332	0,132
3,264	5,775	2,132	0	2	60	1	0	1
3,497	3,433	0,998	0,428	0	300	0	1,344	0,789
8,238	1,427	0,352	0,428	0,789	120	0,143	31,6512	4,643
8,521	1,064	2,275	0,428	1	180	0	0,8064	1,789
6,982	1,207	1,864	0,428	0	582,857	1	482,8292	0,932
10,071	1,064	4,921	0,428	0,789	240	1	2,688	1,578
5,863	1,996	6,275	0,428	0	180	0,429	0,8064	1,789
3,977	1,13	0,921	0,428	0	480	1	12,264	0,143
3,214	0,998	0,998	0,428	0	137,143	1	21,5796	0,143
5,58	0,998	1,286	0,428	0	222,857	1	6,132	5,289
4,918	1,484	1,35	0,551	0,066	197,143	0	13,5492	0,932
13,645	1,787	2,352	0,123	4,566	668,571	1	1,344	6,066
4,414	1,284	4,855	0,786	0	480	1	13,0704	1,789
2,579	0,789	0,429	0,428	0,789	300	0,789	4,5276	0,932

10,686	2,433	1,286	0,428	0	274,286	0	8,064	9
4,698	1,427	1,71	0,428	0,429	214,286	4,5	13,9272	0,066
4,349	4,775	11,361	0,428	0,429	197,143	4,5	32,7992	4,643
5,357	1,996	1,143	0,428	0	120	4,5	303,086	0,143
1,35	1,93	3,998	0,428	0	68,5714	0,143	0	0,286
3,72	3,367	4,15	0,428	1	222,857	1	0	5,289
3,185	0,352	0,066	0,123	1	120	1	0,2268	0,209
10,283	1,721	2,216	0,428	4,5	180	0	3,8724	1,789
3,493	1,418	2,198	0,428	4,5	180	4,5	0	4,566
10,863	6,998	3,075	0,428	1	360	4,5	13,1964	0,143
6,366	2,642	7,35	0,428	1,066	180	4,5	6,132	4,643
6,548	1,13	8,141	0,123	1	291,429	4,5	7,476	4,566
11,58	2,998	11,367	0,428	0,066	60	4,5	64,7556	6,789
3,988	0,132	0,132	0,066	0	282,857	1	0	0,143
2,713	1,855	0,495	0,123	0,789	214,286	0	0	5,289
3,871	1,075	0,855	0,786	0	38,5714	0	0,756	0,789
5,012	1,196	2,352	0,123	1	437,143	1	19,068	0,572
4,216	1,853	4,218	0	0,286	102,857	0	13,0872	1,578
11,732	0,921	2,284	0,428	0	437,143	4,5	18,396	0,066
4,495	1,636	4,429	0,786	1	420	1	6,132	1,789
4,915	2,576	1,644	0,856	0	274,286	1	5,376	0,789
2,614	0,998	2,209	0,123	0	120	1	0,2268	0,209
4,563	0,275	2,064	0,066	0	437,143	4,5	29,8256	6,143
5,775	1,143	0,855	0,428	0	274,286	0,429	0	0
3,99	1,341	1,855	0,428	4,643	420	1	2,688	1,066
3,218	1,789	6,352	0,786	0	137,143	4,5	0	4,643
5,691	4,973	2,075	0,428	0,855	240	4,5	16,4052	1,143
9,306	2,864	2,064	0,428	0,429	77,1429	0	2,5116	0,143
8,363	3,508	6,77	1	0	240	1	6,1824	4,643
4,286	0,561	0,855	0,066	0	642,857	0	0	0
2,987	0,209	3,132	0,066	1	394,286	4,5	0	0,143
1,999	0,572	5,075	0,551	0,789	342,857	0,143	23,31	0,143
8,589	1,209	10,721	1,214	0	300	1	6,1824	6,789
5,638	1,71	0,407	0,123	1	240	1	8,9292	0,932
12,217	1,053	3,855	0,123	1	197,143	0	0	1,143
5,086	1,853	0,352	0,856	0	180	0,789	17,0352	0,143
4,497	0,998	0,209	0,123	1	282,857	1	2,688	1,066
5,21	1,998	5,71	0,786	0	420	1	163,268	1,143
20,644	1,79	3,433	0,428	0	51,4286	0	0	0,143
7,218	1,919	2,207	0,066	0,429	214,286	0	0	0,143
6,194	1,064	0,275	0,123	1	180	0,143	3,4944	0,932
5,86	0,484	1,418	0,123	0	240	1	50,2768	1,578
8,051	2,719	0	0,428	4,5	248,571	1	9,9624	0,143
8,363	0,143	0,198	0,066	0	334,286	1	0	0,143
4,927	1,35	2,932	0,428	0	180	1	0	0,789
2,273	6,715	0,286	0,123	0	60	0	0	4,566
6,133	0,921	1,207	0,123	1	111,429	4,5	6,8586	1,143
6,44	2,139	4,13	0,428	0,789	120	0	3,6792	1,143

1,988	1,064	5,066	0,428	0,066	257,143	0,429	2,1504	0,789
5,004	5,641	2,286	0,856	1	300	4,5	9,1896	1,143
6,506	1,141	0,132	0,428	0	180	0	4,3008	0,789
6,44	3,945	3,367	0,428	1	257,143	1	7,5936	4,643
3,772	4,566	0,198	0,066	4,5	137,143	0	0,8064	1,143
3,558	1,075	0,275	1,214	0,789	300	4,5	39,1356	1,789
5,995	2,218	0,275	0,066	1	325,714	0	3,4944	0,789
4,583	1,13	0,932	0,428	0	282,857	0	18,648	0,932
2,054	5,432	3	0,123	0,789	394,286	1	1,512	0
13,511	1,578	5,289	0,428	1	265,714	0	14,658	0,066
8,137	1,13	0,286	0,786	0	300	1	7,4592	0,143
2,183	2,644	0,855	0,428	0	180	4,5	33,3312	0,932
6,86	1,141	3,264	1	1	462,857	1	2,688	0,143
5,578	1,721	2,143	0,428	0	300	1	0	1,789
6,274	0,341	1,71	0,428	0	180	4,5	6,1824	0,789
3,917	1,218	7,578	0,786	1	180	1	11,9112	1,789
5,138	4,852	1,644	0,428	0,143	231,429	4,5	0	0,789
3,348	0,998	0,921	0,123	4,5	360	1	0	4,643
5,202	1,932	3,638	0,428	4,5	240	0	4,5276	4,643
7,429	1,787	3,499	0,123	1	300	0	0	1,143
5,21	0,418	9,075	0,428	1	385,714	0,143	21,4452	4,5
8,313	0,921	1,284	0,246	0	197,143	4,5	2,7972	0,066
8,075	0,495	0,998	0,428	0	240	0	0	1,143
3,76	1,141	7,198	0,428	0	480	4,5	27,9804	0,066
2,073	0	0,932	0	0,429	37,1429	0,789	0	0,789
4,135	1,407	3,473	0,066	0,066	120	4,5	9,2484	1,143
9,143	1,13	4,578	0,428	0,789	102,857	4,5	14,1246	0,789
4,643	1,493	9,007	0,786	0,789	360	4,5	13,4652	1,789
6,147	0,693	1,066	0,428	6,066	240	1	6,9972	0,209
4,127	0,561	0,998	0,786	0	257,143	0	48,4876	4,643
6,223	1,064	4,998	0,428	0	197,143	0	0	1,143
4,28	2,653	1,352	0,428	0	300	4,5	47,5524	0,066
5,775	1,559	7,064	0,428	0,429	342,857	0,066	8,2824	5,289
4,203	1,418	1,427	0,786	0	222,857	4,5	0	1,578
10,088	0,143	1,132	1,123	0,789	180	0	0	0,789
4,06	1,853	2,932	0,123	0	180	0,789	0	4,643
6,695	1,985	4,721	0,428	4,5	257,143	0,143	18,774	1,789
10,334	2,141	0,572	1	0	120	1	5,376	0,143
7,227	1,776	6,561	0,428	0	240	0	0	1,143
4,289	1,493	1,141	0,123	0	240	1	0	0,572
5,194	2,433	4,638	0,428	1	154,286	4,5	14,1036	4,643
7,793	2,787	7,143	0,428	0	300	0	0	5,289
3,122	0,352	3,429	0,428	0,286	257,143	1	9,324	1,143
4,858	2,367	5,209	0,856	4,5	180	4,5	18,396	4,643
8,143	1,35	3,721	0,428	0	240	1	12,3732	0,789
7,307	0,855	3,504	0,428	0	240	4,5	0	4,643
9,17	4,365	0,407	0,428	1	240	1	7,9716	0,789
2,326	0,132	0,066	0,189	0,789	360	4,5	5,376	0,143

4,701	2,787	0,704	0,979	0,855	385,714	1	15,0612	0,066
4,348	0,858	1,721	0,786	0,429	274,286	1	18,648	1
7,935	2,218	1,864	0,428	0	428,571	0	58,7552	0,143
6,757	0,473	4,207	0,428	0,143	60	0	5,376	4,643
6,937	1,418	8,286	0,786	0	120	1	0	4,643
3,271	1,864	3,209	0,428	1	154,286	1	61,8436	5,289
4,773	1,93	7,064	0,786	0,789	154,286	4,5	11,1636	0,286
4,355	2,719	0,932	0,123	0,789	60	0,143	15,1074	0,143
7,227	1,864	2,721	0,428	0	197,143	0	20,2356	0,932
3,548	1,064	0,264	0,551	1	257,143	1	11,8146	0,066
11,803	2,139	0,132	0,786	0	300	0	9,324	0,143
4,205	1,143	0,066	0,123	0	180	0	1,6128	0
9,14	2,15	2,847	0,786	0	180	0	13,9944	4,643
4,911	1,141	7,432	0,428	1	317,143	1	74,592	1,429
3,353	0,855	0,132	0,123	1	205,714	0,789	0,8064	0,286
3,698	0,855	0,789	0,786	6	360	1	0	0,143
4,135	0,77	0,627	0,428	0,789	300	0	5,376	4,643
4,286	1,341	12,499	0,123	0,429	257,143	1	30,002	1,789
3,28	1,996	3,855	0,428	0	274,286	1	0	0,789
4,209	4,852	6,789	0,428	4,5	257,143	1	0	4,643
3,423	1,776	0,998	0,123	0	300	1	14,1036	1,789
7,447	0,77	4,066	0,856	0,143	214,286	4,5	85,6576	1,789
4,058	0,987	6,996	0,428	0	197,143	4,5	30,002	5,289
4,841	1,996	0,638	0,428	0,855	197,143	4,5	6,9384	1,143
6,854	2,708	3,209	1,428	1,066	394,286	0,066	8,3328	1,789
6,783	4,775	1,864	0	0	300	0,789	0	0,789
11,45	0,352	1,132	0,428	0	300	1	0,2268	1,066
6,067	0,407	0,855	0,123	0,789	420	1	7,35	0,066
4,779	2,207	5,93	0,428	0	180	1	13,181	0,143
4,126	2,053	1,075	0,428	0	180	1	19,1352	0,789
5,207	1,352	2,789	1,214	1	300	4,5	0	0,143
8,018	1,721	0,638	1	1	120	1	15,0612	0,932
6,482	0,286	2,132	0,123	1	197,143	0	0	1,143
6,428	2,719	6,638	0,428	1	394,286	4,5	13,1292	1,578
4,423	1,273	2,484	0,428	0,789	120	0	0	1,143
7,227	1,119	0,998	1,246	0,789	240	1	16,9428	0,143
4,368	1,141	3,932	0,428	4,5	300	1	18,648	5,289
5,28	5,564	5,284	1	4,5	154,286	1	12,264	5,289
3,844	1,427	1,064	1,786	0,066	180	1	30,1224	1,143
2,062	0,209	0,143	0,123	0	137,143	0	6,132	4,643
3,049	0,858	2,367	0,551	4,5	480	1	0	0,789
2,263	2,708	3,921	0,786	1	162,857	1	53,1496	4,643
7,306	1,429	0,286	0,428	1	377,143	1	27,972	0
6,998	0,855	4,932	1,123	4,5	334,286	1	0	0,143
4,413	1,218	2,066	0,786	6	420	0	0	6,143
9,208	1,075	0,987	0,428	0	120	0	0,8064	0,066
6,139	0,924	5,987	0	4,5	471,429	1	46,648	0,789
4,572	0,418	2,286	0,428	0	385,714	0,789	4,8636	0,932

4,118	4,786	7,715	0,856	0,789	214,286	1	20,2356	5,289
9,993	0,407	1,721	0,123	0	120	4,5	10,2144	0,066
5,928	1,075	1,075	0,123	4,5	180	0	0,8064	5,289
3,412	1,721	0,418	0,428	0,143	325,714	0,066	15,33	0,143
9,36	1,207	9,632	0,066	0	557,143	1	2,688	0,143
1,317	1,207	2,638	0,428	0,855	154,286	4,5	18,0012	0,932
3,706	1,416	10,352	0,428	4,5	222,857	1	0	4,643
10,506	3,299	4,218	0,428	1	120	4,5	8,064	1,789
5,856	1	1,361	0,066	0	300	0	0	0,143
3,994	3,508	5,141	0,428	5,289	222,857	0	3,4944	6,789
3,86	0,418	3,789	0,428	0	300	1	3,066	1,578
9,14	1,284	2,207	0,428	0	0	0,429	2,688	1,143
4,619	1,416	1,998	0,551	0,429	514,286	0,789	13,986	1,578
3,83	0,418	0,209	1	0,789	137,143	1	0	0,143
7,974	2,062	2,998	0,428	0	300	1	0,8064	1,789
6,792	1,207	0,418	0,428	0,066	137,143	1	33,1072	0,143
5,65	1,13	5,053	0,428	0	214,286	1	4,662	1,066
4,629	1,198	0,352	0,428	0	171,429	0	13,44	4,643
4,289	2,35	4,776	0,428	4,5	197,143	4,5	17,6652	1,143
7,232	0,932	2,286	0,428	0	274,286	0,789	5,376	4,643
6,074	2,499	1,644	0,428	0,066	265,714	0,143	18,0012	5,289
2,843	1,787	6,418	0,066	4,5	240	1	13,608	4,643
3,415	0,77	7,13	0,066	0,066	420	0	0	4,643
7,667	0,352	2,209	0,428	1	154,286	1	46,3232	0,143
3,981	1,141	0,638	0,428	0,789	385,714	4,5	10,9704	0,143
3,841	2,644	2,15	0,428	0	394,286	1	0	4,643
4,128	0,418	5,066	0,428	0,789	360	0	3,066	4,643
16,506	3,299	5,576	0,786	0,789	197,143	FAŁSZ	0	0,143
3,555	0,847	0,418	0,066	4,5	445,714	0	0	6,143
5,572	1,075	1,132	1	1	377,143	4,5	1,1844	0,855
2,131	4,852	4,286	0,123	0	274,286	0	0,8064	6,066
7,361	1,418	2,433	1	0	214,286	0,143	25,641	0,143
11,008	2,576	14,141	0,428	0	240	4,5	12,264	6,789
4,263	5,718	0,495	0	4,5	257,143	0,143	0	4,643
1,889	1,284	1,93	0,428	0,789	265,714	0,789	0	0,932
3,203	0,627	4,352	1	0,789	300	1	23,1924	5,289
4,061	0,998	0,855	0,428	0	197,143	1	10,2144	0,209
3,06	1,209	17,075	0,786	0	300	4,5	9,324	5,289
5,058	4,841	1,998	1	0	420	1	0,8064	6,789
3,778	1,93	0,341	0,428	0	257,143	0	4,5276	5,289
3,546	1,644	0,998	0,123	0	120	1	2,688	0,143
5,073	2,785	3,644	0	0	0	1	0	5,289
8,07	9,209	0,198	0,066	0,789	300	1	3,4944	0,066
5,995	1,493	5,789	0,786	0,789	197,143	1	7,5936	6,789
15,573	0,998	1,132	0,123	0,789	120	0	6,132	4,643
4,401	1,053	2,576	0,551	0,789	385,714	4,5	8,2824	0,143
3,78	0,693	5,361	1	4,5	360	4,5	1,8396	0,789
5,801	1,141	10,15	0,428	0	197,143	4,5	2,7972	1,143

10,789	5,355	1,341	0,428	0,143	162,857	0	0	0,789
5,431	0,561	8,066	0,123	0,789	360	4,5	0	1,066
4,564	0,932	1,132	0,494	0	154,286	1	4,8384	0,066
6,232	0,495	3,864	0,428	0	240	1	6,132	4,643
5,049	1,35	0,704	0,428	0	240	1	5,4852	0,932
2,766	3,156	1,207	0,428	4,643	420	1	0	0,066
4,478	4,775	9,198	0,428	4,5	300	1	37,9736	5,289
4,98	1,789	1,787	2,928	0	257,143	4,5	2,7972	6,143
5,08	1,853	4,721	0,428	0,789	180	0,066	2,1504	5,289
8,506	2,578	2,066	0,428	0	60	1	0	1,143
6,366	0,495	6,207	0,428	0	257,143	0	2,331	1,143
9,285	1,932	4,495	0,428	0	274,286	1	5,376	6,429
6,207	1,71	4,207	0,428	0,789	274,286	0,789	6,1824	4,643
4,357	1,864	0,198	0,428	0	102,857	0	7,9716	4,566
7,599	1,644	1,789	0,428	0	240	0,429	6,7452	0
10,788	2,418	1,075	0,428	0,495	300	4,5	2,688	1,143
4,915	0,932	6,132	0,189	4,5	360	0	0	0,789
5,144	1,998	3,075	0,428	0,143	240	1	0,8064	1,578
5,632	1,787	7,284	0,428	1	197,143	4,5	7,9716	0,066
18,637	0,341	0,352	1	0	300	0	2,688	0,066
3,346	0,572	1,864	0,428	0,789	342,857	4,5	21,9394	0,286
5,129	2,075	0,627	2	0	282,857	1	84,1008	0,143
11,377	2,275	0,932	0,428	4,5	102,857	4,5	1,344	0,789
4,355	0,847	0,132	0,428	0	257,143	1	46,648	1,578
5,289	2,216	9,93	0,428	0,789	180	1	2,2848	4,643
2,549	1,644	0,998	0,428	0,429	360	0,143	13,0032	1,218
8,387	1,427	12,862	0,428	0	137,143	1	3,4944	4,643
9,581	2,367	6,433	0,428	0	162,857	1	0,8064	1,578
6,071	0,198	0,209	0,066	0,789	360	1	9,3156	0,789
10,377	1,075	3,433	0	0,143	257,143	1	0	4,643
8,02	2,51	2,864	0,428	1	205,714	1	2,688	1,789
5,368	0,407	0,998	3	0	120	1	18,648	4,643
4,223	1,064	2,209	0,428	6	240	0,789	0	0,495
8,2	1,842	5,998	0,123	0	257,143	0	0,8064	0,932
8,071	1,361	8,141	0,428	0,066	60	4,5	4,032	1,143
5,155	1,919	0,429	0,428	1	274,286	0,143	9,324	1,143
6,164	2,139	2,855	0,123	1	334,286	1	53,984	4,643
5,575	1,919	0,132	0,428	0,066	197,143	0	7,35	0,789
5,278	1,064	4,352	0,428	1	197,143	0,143	9,324	1,143
2,559	1,141	6,275	0,428	0	120	0	1,512	5,289
3,7	0,693	1,198	1	6	420	1	12,39	0,143
5,995	0,77	0,066	0,494	4,643	205,714	1	0,8064	0,143
2,84	0,855	1,143	0,123	1	240	0	5,376	6,066
4,421	1,064	2,787	0	4,5	582,857	1	0	0,143
5,63	2,359	3,939	0,856	4,5	360	1	0	1,789
4,289	1,644	9,93	0,786	0	360	0,429	0	1,143
2,994	1,075	0,209	0,123	1	240	1	3,5532	4,566
3,92	1,13	1,396	0,428	1	120	0,066	6,5016	1,578

6,124	5,63	0,998	0,428	1	300	0,789	0	4,643
5,286	1,275	1,855	0,123	0	222,857	0	2,7972	4,643
9,571	2,15	2,721	0,786	0,429	102,857	4,5	5,5944	1,578
8,789	0,998	6,141	0,428	4,5	240	0,789	0	0,572
5,126	1,075	2,198	0,246	1,066	51,4286	4,5	2,688	6,143
2,208	1,713	2,499	0,786	0,429	360	1	49,4452	4,643
8,449	1,207	0,264	0,428	1	162,857	1	33,0022	0,143
7,711	1,209	2,132	0,428	0	180	1	18,648	0,789
8,632	3,368	18,073	1	1	531,429	4,5	27,594	4,643
4,061	1,196	0,418	0,428	0	300	0	0,8064	0,932
4,928	1,132	0	0,123	1,066	27,8571	1	0	1,789
3,258	0,484	3,429	0,428	0	317,143	1	13,0704	0,932
4,981	2,139	1,71	0,786	1,066	300	1	68,3452	0,932
4,924	2,433	2,864	0,428	0	308,571	1	15,0612	1,578
5,79	1,075	10,495	0,786	1	480	1	12,1212	1,789
4,419	1,066	2,275	0,428	1	102,857	1	7,9716	1,143
9,816	1,787	1,855	0,856	0,066	222,857	1	21,6048	0,143
4,487	1,721	3,932	0,494	0	214,286	0	24,7464	4,643
1,614	0,921	6,209	0,066	0	274,286	0	0	6,789
4,267	1,504	1,57	0,909	0	360	1	4,1412	0,932
6,86	0,998	0,352	0,428	1	240	0	11,13	0,789
5,564	2,644	2,484	0,856	0,789	222,857	0	0	1,578
6,355	2,205	2,499	0,428	0	240	0,789	3,1836	0,932
3,931	0,352	1,93	0,551	0,143	162,857	1	120,008	0
8,268	2,576	1,143	0,856	1	394,286	0,789	13,986	0,066
4,426	1,842	2,282	0,428	0,143	257,143	0	11,0292	0,143
5,995	0,55	1,998	0,066	0	437,143	1	0	0,143
3,142	1,218	10,418	0,123	0,789	325,714	6	65,0706	0,855
4,982	2,642	0,998	0,856	1	137,143	0,066	4,8384	0,932
5,284	1,427	4,998	0,786	1	300	1	0	1,789
5,861	1,864	8,198	0,428	0	300	0	0	0,143
2,906	2,284	0,341	0,428	0,143	377,143	0,143	10,1304	6,789
4,259	2,13	1,21	1,214	0	240	4,5	15,5568	1,066
4,649	2,367	3,141	0,428	5,289	360	0	18,396	5,289
5,998	2,007	8,367	0,428	4,5	180	4,5	0,8064	4,643
7,427	1,286	4,921	0,428	1	154,286	0	6,132	0,789
6,508	0	0,132	0,551	1	120	1	18,0012	0,789
5,647	1,864	1,855	1,572	4,643	282,857	4,5	31,8444	5,289
8,64	2,842	4,433	0,428	0,066	300	4,5	15,5232	1,789
4,408	2,207	6,275	0	0	300	1	7,476	5,289
7,93	2,499	0,407	1	0,066	240	1	0	0,789
5,278	0,429	2,132	2,5	0	368,571	1	2,7972	0,143
7,576	1,13	1,644	1,123	0,789	188,571	1	9,324	0,932
3,491	0,921	0,264	0,428	4,5	257,143	0	0	1,578
9,167	0,638	4,643	1,123	4,566	34,2857	1	6,9972	0,429
4,278	0,484	1,855	0,428	0	282,857	1	32,3904	0
6,126	0,998	0	0,856	0,429	30	1	9,324	0,143
4,838	2,361	5,427	0,428	0	214,286	4,5	19,992	0,789

3,458	1,064	0,286	0,123	1	120	4,5	18,0012	0,143
4,709	0,847	4,998	0,856	0	480	1	12,264	1,143
3,194	0,352	1,143	0,428	1	291,429	0,789	5,5188	0,066
5,568	0,275	4,932	0,428	1	480	4,5	0	1,066
4,638	0,847	0,932	0,428	0,429	282,857	4,5	6,132	1,789
3,778	1,853	2,264	0,428	0,789	120	1	19,2024	5,289
4,926	0,998	0,264	0,189	1	180	1	4,5276	0,932
2,483	1,132	1,998	0,428	4,5	162,857	0	0,8064	0,209
2,909	3,299	12,352	0,066	5,289	394,286	4,5	0	5,289
5,346	1,056	0,855	0,428	1	120	1	13,0704	0,066
5,146	1,427	1,207	0,428	4,5	240	4,5	0	0,143
3,626	1,921	1,998	0,428	0,789	240	1	23,058	5,289
3,635	1,853	6,644	0,428	0,429	282,857	4,5	18,396	6,143
6,708	1,578	6,704	0,123	0,789	120	4,5	11,9952	1,143
4,939	1,789	3,578	0,123	1	300	4,5	2,688	6,143
6,212	2,064	5,207	1	0,789	600	1	8,82	0,932
3,151	6,43	2,007	1	1	317,143	0	0	1,789
3,992	1,064	0,638	0,786	0	77,1429	1	3,4944	0,932
8,033	2,51	4,156	0	0	102,857	1	0	1,578
4,77	1,842	5,064	0,428	4,566	197,143	1	27,7116	1,578
4,194	0,855	1,578	0,123	1	60	4,5	0,8064	0,286
4,271	2,499	7,075	0,123	1	240	4,5	6,5604	1,066
5,423	2,576	4,418	0,856	1	154,286	4,5	2,1504	1,789
11,145	3,222	3,141	0,189	1	214,286	0,789	10,1934	1,143
10,154	1,504	3,001	0,428	4,5	171,429	0	24,528	4,643
5,871	0,715	1,644	0,786	1	120	4,5	12,3144	0,143
12,433	5,498	1,066	0,786	4,5	188,571	0	0	0,143
8,125	0,789	9,855	1	1	120	1	9,324	4,643
8,212	6,65	0,198	1	0	120	4,5	26,0652	0,132
6,504	1,987	0,855	0,428	0	240	0,789	2,7972	1,143
4,627	0,855	0,209	1,572	1	300	1	0	0
6,95	4,698	0,987	0,428	0	197,143	1	0	1,578
2,917	1,141	1,855	0,428	0,789	154,286	1	27,972	4,643
11,211	1,13	3,066	0,123	1	120	1	0,8064	1,143
3,496	2,721	3,352	0,066	4,5	471,429	4,5	42,1232	4,643
6,126	1,207	4,196	2,5	0,143	257,143	0,789	2,1504	1,578
5,715	1,35	5,71	0,428	4,5	197,143	4,5	15,0612	0,932
2,12	1,721	3,572	0,428	4,5	222,857	0	1,8396	4,643
5,691	1,284	0,352	0,551	0	180	1	0	0,932
2,309	0,77	5,561	0,123	4,5	325,714	0,429	4,8384	4,643
4,15	0,855	1,198	0,428	1	480	4,5	6,9384	4,643
4,34	0,561	3,427	1	4,5	300	4,5	3,8724	4,566
4,779	0,198	0,209	0,428	0,789	180	1	6,132	5,289
3,032	5,707	1,141	4,5	0	480	0,789	23,9904	0,932
5,366	2,576	1,418	0,428	0	257,143	1	16,7832	1,143
2,986	1,064	0,998	0,066	1	394,286	1	0	4,643
5,372	2,576	2,073	0,428	0	274,286	4,5	41,3784	5,289
6,137	1,341	1,352	0,123	1	102,857	1	8,442	4,643

4,643	1,842	6,367	0,428	0	0	0	0,756	5,289
2,043	2,642	8,939	0,066	0,789	360	1	0	5,289
3,983	2,642	4,484	0,428	0,066	102,857	1	27,972	4,566
2,194	0,704	6,71	0,428	0	377,143	4,5	18,396	4,566
5,702	2,433	1,638	0,856	0	180	4,5	39,4632	1,066
3,285	1,207	0,286	0,428	0	325,714	1	2,7972	0,932
8,294	1,721	3,275	0,428	0	60	1	0	5,289
0,88	1,064	5,352	0,428	0,789	360	1	0,378	4,5
5,652	2,576	1,71	0,428	0	300	0	34,986	1,578
4,278	0,484	2,352	0,856	0,789	120	1	22,7892	0,143
3,483	0,275	3,932	0,428	1,066	77,1429	1	67,298	1,066
8,351	0,932	1,209	0,066	0	574,286	0	2,688	0,932
5,5	1,075	6,641	0,428	1	154,286	1	19,74	0,932
15,009	2,653	1,218	0,786	0,143	162,857	0	0	0,789
6,159	1,075	2,864	0,428	0	154,286	0	13,6584	1,143
4,943	2,15	11,141	2,5	4,5	94,2857	1	0	0,572
4,706	0,132	0,855	0,428	1	282,857	1	7,35	0,066
5,783	0,693	3,352	0,066	4,5	360	0	3,6792	0,143
3,621	0,572	4,638	0,428	1,066	265,714	1	28,3332	4,566
9,208	1,064	0,275	0,428	0	480	0	0,8064	1,066
6,214	2,576	9,718	0,428	4,5	197,143	0,789	0	6,143
4,491	1,053	2,789	0,551	1	120	1	13,2244	0,143
6,795	0,561	0,275	1,428	0,789	222,857	0,429	17,0352	0,066
6,79	0,921	5,921	0,428	0	300	1	2,1504	0,143
5,155	1,919	2,207	0,428	1	154,286	1	0	0,932
5,348	1,141	0,143	0,428	0	137,143	0	0	1,143
6,911	1,427	6,715	0,246	4,5	102,857	0,789	2,7972	1,789
3,471	0,561	4,275	1	1	394,286	1	2,7972	0,855
7,15	0,921	1,275	0,428	0	385,714	1	10,0632	0,143
5,84	1,787	1,427	0,428	0	180	1	7,2156	5,289
12,94	2,073	2,367	0,551	4,5	205,714	4,5	13,986	4,643
3,508	0,921	3,855	2,5	0	548,571	0	11,1552	1,143
8,671	1,644	2,721	0,123	0	137,143	1	31,8416	0,789
8,657	1,787	1,064	0,123	1	265,714	1	61,4824	0
3,184	1,053	1,721	1,572	0	351,429	1	15,5568	0,143
3,489	0,352	0,77	0,066	1	300	0	0	5,289
5,99	1,418	5,987	0,786	1	240	1	0	4,643
5,498	0,484	11,207	0,428	4,5	120	0	9,9078	5,289
5,06	2,565	4,407	1,214	0,066	214,286	1	24,7324	1,218
2,271	4,643	0,132	0,066	0,143	300	4,5	2,688	0,143
2,612	1,064	9,998	0,123	0,789	162,857	4,5	18,396	0,789
4,906	2,433	3,352	3,286	0,789	240	1	10,752	4,643
3,26	0,484	0	0,428	0	265,714	1	10,752	0,143
8,814	4,877	2,855	0,428	4,5	257,143	4,5	10,08	0,429
12,015	1,064	2,987	1,214	0	180	4,5	11,2392	0,789
16,374	4,995	1	0,856	4,5	180	1	5,376	0,789
6,79	2,644	5,987	0,123	0,066	120	4,5	1,8396	0,143
3,687	2,367	6,361	0,428	0	351,429	0,143	10,9704	0,789

4,469	1,57	2,998	0,246	1	94,2857	1	24,7912	4,643
5,133	1,787	1,141	0,494	0	154,286	4,5	31,9872	0,789
2,785	0,998	6,132	0,428	4,5	317,143	0	14,9856	0,789
4,56	1,35	0,275	0,428	4,5	180	4,5	5,376	4,643
6,297	2,939	4,504	0,856	4,5	94,2857	1	0	5,289
5,634	2,499	1,787	0,123	0	240	4,5	4,662	4,643
3,361	1,644	2,209	0,856	1	180	1	2,688	5,289
10,861	2,367	1,932	0,428	0	240	4,5	101,3292	1,143
4,781	2,93	0,143	0,428	0	274,286	0	1,6128	0
5,357	0,789	0,132	0,428	1	308,571	0	3,066	0,932
4,621	2,644	10,064	0,786	6,066	197,143	4,5	5,376	4,566
4,261	2,271	0,921	1,214	0,789	557,143	1	15,8466	1,066
1,68	0,132	8,418	0,428	0,789	197,143	0	6,8544	4,643
4,212	1,264	0,407	1	4,5	240	1	0	0,143
2,142	2,361	0,143	1	1	317,143	0,789	36,0024	1
3,69	2,71	5,71	0,786	0,066	300	4,5	12,096	6,143
5,214	0,209	1,787	0,428	1	102,857	1	18,396	0,143
2,768	0,781	1,427	0,428	0	377,143	1	48,398	0,143
15,937	1,064	0,987	0,428	0,143	180	1	160,6724	0,789
3,33	2,139	1,504	1,284	0	240	1	10,8612	0,286
4,704	6,144	4,075	0,123	0	240	1	0	5,289
3,195	0,484	12,627	0	0	77,1429	0	2,688	1,143
6,577	0,572	7,939	0,428	1,789	385,714	0,066	2,331	0,789
4,927	1,284	4,781	0,979	0	240	1	0	0,143
25,095	0,932	1,075	0,786	0	300	4,5	5,8632	1,578
8,937	6,078	1,141	3,286	4,5	60	1	0,8064	0,143
2,854	0,987	2,275	0,123	4,5	222,857	1	7,5264	0,143
10,65	2,51	0,932	1,214	1	334,286	1	0	0,932
9,206	1,842	0,572	0,428	0	317,143	1	9,6264	1,789
9,537	2,862	0,715	0,428	0	240	4,5	37,296	4,643
5,937	0,987	0,143	0,428	1	120	0	23,324	1,143
4,472	1,218	1,141	0,428	1	197,143	1	1,8396	6,143
4,698	5,498	3,35	0,786	0	300	1	0,8064	0,066
4,994	1,776	3,572	0,428	0,789	154,286	4,5	4,662	1,066
9,574	1,141	4,143	2	0,789	437,143	4,5	161,9856	0,143
4,05	1,207	0,932	0,123	0	282,857	1	0	1,066
4,858	1,504	1,787	0,428	4,5	497,143	4,5	0	0,143
4,701	5,641	3,987	0,909	0,789	428,571	1	25,3344	0,789
2,68	1,71	6,143	0,066	4,566	651,429	4,5	9,324	5,289
6,869	1,286	0,132	0,123	0	102,857	0,789	111,7648	0,143
2,977	1,776	0,198	1	0	368,571	1	156,0104	0,143
7,583	1,13	0,198	0,123	0	111,429	4,5	2,1504	0,572
3,706	1,352	1,998	0,066	0	162,857	1	0	0
5,082	1,218	2,286	0,428	0	180	0	0,8064	6,143
5,063	1,075	2,418	0,428	0	240	0,429	8,442	0,143
16,291	2,721	6,505	0,786	0,143	16,4286	0,789	0	0,572
8,297	0,789	1,644	0,428	1	180	1	3,6792	0,789
5,867	1,218	0,847	0,551	0,789	282,857	4,5	21,4452	0,143

5,21	1,13	0,998	1,066	1,143	420	0,429	3,4944	1,143
4,497	1,064	2,075	0,428	1	197,143	1	0	0,143
3,996	5,63	5,998	1	0	120	1	75,1002	0,789
6,733	1,996	2,198	3,5	1	360	4,5	6,7452	0,143
10,257	1,273	2	0,428	0	385,714	0	13,9944	1,578
10,354	1,561	2,855	0,123	1	360	0	0	0,143
6,792	1,141	1,787	0,551	0,789	257,143	0	560,378	0,932
7,66	2,499	9,053	0,856	4,5	214,286	0	4,5276	0,286
18,582	1,075	0,495	0,856	0	180	1	5,8632	0,143
4,506	1,71	5,998	0,551	4,5	325,714	4,5	0	0,143
6,215	1,93	1,064	0,428	0,066	120	1	46,648	4,643
3,051	2,433	1,721	0,428	0,789	94,2857	0	0	1,789
3,92	0,715	2,407	0,066	0	574,286	0	24,0352	0,143
3,984	0,77	0,921	0,123	4,5	360	1	2,7972	4,643
3,849	1,064	0,987	0,428	0	120	1	0,378	0,066
3,931	1,196	4,855	0,428	0,286	214,286	0,066	0	1,789
3,632	0,781	1,075	0,856	0	480	1	52,8948	0,286
5,215	2,51	5,721	0,786	0,789	120	0,143	10,2564	1,578
3,832	2,433	1,207	0,066	0	377,143	1	6,1824	0,066
4,344	1,996	6,053	0,786	0	145,714	4,5	1,344	0,066
3,133	1,198	0,132	0,786	1	651,429	1	0,8064	0,789
3,772	1,064	7,222	0,856	0,789	274,286	0	9,5172	1,578
5,517	1,484	0,209	0,428	0	274,286	1	18,102	0,143
5,287	0,418	1,922	0,852	0,143	137,143	0,429	5,1072	0,789
5,995	1,787	6,858	0,428	0,429	137,143	1	14,2296	0,789
4,045	0,275	1,141	2	1	180	1	1,6128	0,143
5,22	1,495	2,198	0,428	0	600	1	24,528	5,289
5,065	2,578	7,286	0,123	4,5	325,714	4,5	0	5,289
14,578	5,289	2,429	0	0,066	85,7143	1	0	5,289
9,52	3,156	2,578	0,428	0	257,143	0	18,396	0,932
4,583	2,653	3,143	0,428	0	342,857	0	0	4,643
5,936	0,998	0,715	0,856	0	308,571	1	7,35	0,066
21,286	0,352	0,209	3,356	0	180	0,066	8,8704	0
4,212	0,921	1,418	0,066	4,5	514,286	1	2,7972	0,143
4,926	1,932	2,578	0,856	0,789	257,143	0,429	69,972	4,643
7,425	1,93	2,075	0,428	0,066	720	1	0	0,143
4,128	0,998	1,427	0,123	1	300	1	37,296	0,789
3,181	0,998	1,275	0,428	0,789	162,857	0,143	2,688	4,643
4,286	1,493	2,495	0,856	1	454,286	1	79,4472	0,143
9,646	2,921	1,209	0,786	4,566	240	1	20,2188	1,578
2,777	1,864	1,066	0,428	0	514,286	4,5	21,6888	0
12,307	0,759	3,218	0,428	4,5	197,143	0,789	29,5848	1,578
4,225	0,484	5,847	0,369	0,789	300	4,5	32,7992	0,143
4,858	1,721	2,075	0,786	1	222,857	1	23,324	1,143
4,99	1,141	0,987	0,786	0	94,2857	0	1,8396	0,143
6,084	1,075	5,007	0,428	1	282,857	0,066	8,148	5,289
8,376	1,493	1,218	0,428	0	137,143	4,5	3,36	0,789
3,789	1,787	10,998	0,428	1	162,857	4,5	5,2122	4,643

2,634	0,932	0,132	0,123	0	231,429	1	9,324	0
3,28	0,418	1,352	0,123	1	240	1	0,756	4,566
5,08	1,273	3,275	0,123	1	317,143	0,429	5,754	6,789
2,777	0,704	1,075	0,428	0,429	420	1	25,655	4,643
6,781	0,704	15,352	0	4,5	240	4,5	0,8064	6,143
8,884	1,207	6,715	0,428	4,5	342,857	4,5	23,324	5,289
6,372	1,721	0,715	0,428	1	300	1	7,4592	1,143
19,999	1,855	1,352	0,066	0,429	154,286	4,5	6,132	0,789
3,423	2,216	12,998	0,066	4,5	274,286	1	6,1824	0,789
4,417	7,144	3,71	2,5	0,789	180	1	0	4,643
6,069	2,576	5,853	0,428	0	180	1	0,8064	5,289
3,706	0,275	0,132	0,123	1	180	1	2,688	0,143
5,06	1,561	9,367	2,5	6	265,714	0	15,4896	0,572
9,716	1,209	1,644	0,551	0	257,143	1	6,132	0,143
3,126	1,644	6,855	0,856	0	300	0	23,3016	4,566
4,346	0,495	2,064	0,189	0,789	197,143	1	1,5624	1,578
4,489	0,987	5,264	0,428	0	214,286	1	10,2816	6,066
4,423	2,436	5,484	1	0,143	120	0	0	1,789
4,506	1,075	1,789	0,428	4,5	300	0	14,1036	0,789
4,149	2,719	1	1	0,066	120	4,5	15,6324	1,143
3,706	0,998	0,341	0,428	1	120	0	5,418	1,143
7,864	0,987	0	1	0	180	1	2,7972	0
3,282	2,642	0	0,428	1	120	4,5	2,7972	1,143
9,637	4,566	0	0,123	0	102,857	1	7,9716	0,066
9,483	0,572	1,275	0,189	4,5	282,857	1	12,1212	0,932
3,717	0,352	3,064	0,551	5,289	222,857	4,5	6,9972	5,289
4,629	1,075	1,429	0,856	0,143	377,143	0	0	4,643
5,992	0,847	4,367	0,786	0	300	4,5	0	4,643
8,266	1,066	3,998	0,123	0	437,143	1	2,9568	0,789
6,661	2,007	0	0,123	0	240	0	2,688	0,572
4,138	1,416	0	0,066	0	360	1	147,4284	0,789
0,979	0,418	0	0,123	0	257,143	1	368,4982	0,066
11,503	0,77	5,284	1	0,066	300	4,5	6,132	0,789
4,429	0,275	0,998	0,428	0,789	197,143	1	58,31	0,789
6,507	1,647	1,064	0,428	4,5	240	1	7,35	1,578
5,654	1,787	2,209	0,428	0	300	1	0	1,789
3,21	1,13	3,209	0,428	0	180	1	2,688	1,143
4,715	0	0	2	0	102,857	0	0	0
6,997	0,473	5,209	0,428	1	265,714	4,5	1,6128	4,643
9,381	2,367	4,998	0,428	1	120	1	0	1,789
12,493	1,35	2,207	2,5	1	257,143	0,066	4,5276	0,789
4,918	1,207	5,361	0,428	0	171,429	1	6,9972	1,143
5,86	5,707	1,066	0,428	4,5	420	0	4,5276	1,143
5,357	0,484	2,987	0,066	0	420	0,789	3,4944	0,143
5,449	2,51	1,789	0,428	0	145,714	0	0	5,289
4,154	1,504	5,286	0,428	0,066	214,286	1	5,376	0,932
5,278	2,216	0,352	0,786	4,566	437,143	1	43,1592	0,143
6,504	1,71	0	0,786	0	240	0	4,5276	0,066

7,721	1,776	9,198	0,428	0	77,1429	0,143	0	0,066
6,794	5,289	0,198	0,066	0,789	480	0,789	7,4592	0,066
3,478	2,205	20,275	0,428	4,5	137,143	4,5	0	5,289
5,063	1,341	0,286	0,428	0	282,857	1	27,972	0,789
4,706	0,998	0,352	0,246	4,5	274,286	0	2,688	1,066
3,06	1,787	6,352	0,123	1	120	1	8,064	1,143
5,421	1,998	4,924	0,856	0	282,857	1	7,5264	1,789
8,938	1,787	1,275	0,123	0	51,4286	0	5,1996	0,143
3,775	1,93	3,284	2,5	0,789	300	1	0	5,289
2,931	1,207	5,352	0,428	4,5	120	1	13,5492	0,932
1,683	1,141	3,13	0,551	0,066	257,143	0,429	10,8612	4,566
2,271	0,627	0,132	0,066	0	360	0,789	6,1824	6,066
2,065	1,207	3,352	0,123	4,5	360	0,789	1,8396	0,143
14,486	7,644	5,352	0,786	0	257,143	1	16,2708	5,289
6,95	1,284	1,715	0,428	0	214,286	0	0	4,643
3,133	3,365	1,721	0,428	0	77,1429	0,789	3,6792	1,578
3,992	2,565	3,064	0,856	0	197,143	1	14,2464	4,643
2,714	2,139	5,13	0,066	4,5	514,286	4,5	0	4,643
8,577	6,43	8,644	0,786	0,066	282,857	0	13,8026	6,143
6,012	1,853	3,429	0,786	0	180	1	12,264	0,143
3,283	1,484	11,578	1	4,5	445,714	0	10,1304	4,643
7,506	1,787	1,921	0,786	0	162,857	6	14,9184	1,143
1,493	1,13	13,284	0,428	0,855	342,857	0	63,4172	1,789
3,118	0,704	7,284	0,066	0	394,286	0,789	0	4,643
4,358	1,141	0,855	0,786	0,066	257,143	0,066	19,2024	0,143
4,267	1,064	1,429	0,123	0,066	360	1	7,5264	0,789
4,544	1,919	0,341	0,123	4,5	205,714	1	32,1804	0,132
5,706	0,33	8,007	0,786	1,143	111,429	0,789	7,476	1,218
7,222	1,71	1	0,428	0	42,8571	4,5	0,8064	0,932
3,7	2,576	4,921	0	0,209	360	1	7,4592	1,789
12,293	0,858	0,572	0,428	0,066	222,857	1	0	0,932
15,583	1,13	5,786	0,428	0	265,714	0,066	6,1824	0,143
3,109	0,561	4,141	0,066	1	360	0	0	4,566
4,993	1,064	2,284	0,123	0	214,286	1	2,016	4,643
5,906	3,642	10,284	0,428	0,789	137,143	0,143	6,132	4,643
6,779	1,482	2,998	1,214	0	377,143	1	18,396	0,066
9,517	2,787	1,855	1,551	0	120	0,143	20,9832	1,143
5,126	0,627	1,352	0,428	1,066	480	1	13,0704	1,143
7,15	1,064	0,561	0,856	1	222,857	1	23,324	4,643
7,227	0,638	0,495	0,428	1	368,571	1	30,31	0,143
12,06	1,352	0,132	0,551	0	111,429	0	0,6048	1,143
3,994	0,921	0,209	0,428	0,789	505,714	0,143	7,2156	4,566
2,045	0,55	0,198	0,123	0,789	180	1	99,974	0,855
2,348	1,075	2,51	0,123	4,5	257,143	0,789	2,7972	0,789
4,058	0,693	2,484	0,066	4,5	420	1	0	6,066
10,349	1,284	1,352	0,428	0	300	1	9,996	1,066
2,758	1,776	1,998	0,428	0	137,143	1	20,2356	4,643
8,592	0,539	2,066	0,428	4,5	102,857	1	0	1,789

6,064	1,853	5,064	0,786	0	300	1	0	1,143
3,992	5,575	2,361	0	5,289	197,143	1	30,002	6
6,348	2,216	3,141	1	0	180	1	36,3636	1,789
5,078	0,987	2,998	0,428	0,855	214,286	1	9,324	0,572
3,824	2,853	6,341	0,123	0,789	154,286	4,5	48,4876	4,643
4,574	0,932	0,341	0,066	1	188,571	4,5	9,408	4,643
4,289	1,842	4,787	0,551	0	377,143	1	14,1036	5,289
5,014	0,855	3,998	0,551	0,066	300	1	23,4024	0,789
3,205	0,998	1,209	0,123	1	300	1	12,2052	0,143
4,708	1,776	0,132	0,428	0	120	0,143	0	1,789
2,043	1,855	1,209	0,428	0	77,1429	0	0,8064	4,643
8,426	1,427	3,484	0,428	1	137,143	1	13,1964	5,289
8,995	0,781	0,352	0,066	1	257,143	1	1,8396	4,643
2,7	1,998	1,561	0,428	0	257,143	4,5	19,2024	6,143
4,27	1,504	3,064	0,786	0	222,857	1	11,2476	0,209
5,555	5,564	3,433	0,428	0	162,857	4,5	7,5264	0,143
5,649	1,13	0,418	0,551	0	428,571	0	0	0,789
11,503	1,207	0,998	0,123	0	300	4,5	8,519	0,789
3,709	1,71	0,132	0,428	0,143	257,143	1	10,752	4,643
4,621	1,284	7,721	0,123	0	360	1	0,8064	0,066
7,223	0,924	2,572	0,428	0	70	1	0	1,789
5,201	1,264	2,499	0,428	1	240	4,5	0	0,789
3,926	0,693	2,275	0,909	0	317,143	0	6,132	0,143
14,941	1,341	0,286	0,428	1	240	4,5	5,5944	1,789
8,477	1,064	1,064	0,856	0,143	197,143	0	15,1872	0,286
7,438	1,776	1,932	1,428	0,789	137,143	1	4,5276	0,143
4,491	1,787	4,855	0,428	4,5	154,286	1	65,1322	0,066
6,924	1,919	5,93	0,428	0,066	197,143	1	12,1212	4,643
4,355	2,576	1,987	0,428	0	180	0,789	0,8064	1,789
9,274	1,853	1,864	0,428	4,5	197,143	1	0	6,789
4,361	0,855	0	0,428	4,5	214,286	0	0	0,789
8,359	1,141	4,929	0,123	1	120	1	29,6632	0,932
5,937	1,207	1,721	0,428	0	120	1	6,9972	0,066
3,711	2,71	3,35	0,123	4,5	360	1	0	1,789
5,793	1,787	1,066	0,786	0,789	145,714	1	8,8704	0,932
9,775	0,998	0,352	1,494	0	137,143	1	36,6492	0,789
9,732	5,784	1,075	0,428	0	240	4,5	0,8064	5,289
4,99	2,13	5,787	0,428	0	154,286	1	1,8396	0,143
3,36	5,421	0,932	0,428	0,066	214,286	0	0	0,789
4,798	1,93	3,064	0,786	0,429	222,857	0,066	18,648	4,566
4,557	4,643	8,775	0,428	0	240	0	3,6792	0,143
2,579	1,93	1,932	0,428	0	137,143	1	0,8064	1,789
8,73	0,998	1,143	0,123	0	197,143	1	0	0,932
2,898	1,141	5,075	0,428	4,566	214,286	4,5	14,4354	1,578
9,229	7,078	3,075	0,428	0	162,857	4,5	0	0,789
3,753	2,576	1,998	0,428	0	137,143	1	9,2484	6,143
3,493	1,209	0,209	0,428	0	171,429	1	18,0012	0,066
2,414	1,787	3,284	0,066	0	351,429	0	14,686	6,789

8,102	0	1,207	0,428	0,429	282,857	0	0	0,789
1,471	1,721	3,275	0,123	0	428,571	0	11,2476	4,643
7,08	0,286	1,578	0,856	1	214,286	0	0,8064	0,143
9,708	1,196	3,264	1	0	300	1	8,2908	0,066
5,856	1,57	4,075	0,786	1,789	360	4,5	13,9944	4,643
2,919	1,075	0,066	0,428	0	120	1	18,396	1,066
5,058	1,064	4,216	1,428	0	120	1	2,1504	5,289
6,218	1,787	8,13	1	4,566	197,143	1	0,8064	1,143
6,28	0,495	6,5	0,428	1	257,143	4,5	0	0,932
2,274	3,139	14,853	0,066	0	351,429	1	0	5,289
6,293	1,075	4,418	0,123	0	257,143	0,429	5,376	0,066
9,925	1,996	3,352	1	0	137,143	0	0	5,289
4,05	2,284	6,007	0,909	0	77,1429	4,5	12,8352	0,143
4,559	1,853	0,132	0,428	0	394,286	0	9,0552	0,143
7,298	1,484	0,418	0,428	4,5	180	1	1,6128	0,286
3,827	1,13	0,429	0,551	0,789	300	0,143	0	0,932
5,344	1,273	4,198	0,123	0,143	180	0	66,6736	0,143
6,497	1,787	1,132	0,428	0	60	6	6,9972	0,789
7,43	2,207	4,51	0,123	0	180	0,143	1,4784	0,286
3,137	0,855	2,198	0,066	0,495	317,143	1	2,7972	4,643
5,584	1,644	1,71	0,123	0,143	180	1	1,8396	1,066
9,349	1,35	0,132	1	1	120	0,143	8,9292	4,643
5,041	0,855	3,51	0,428	0	308,571	1	30,31	1,066
4,201	1,066	1,064	0,066	1	240	1	12,9696	0,143
6,432	1,218	0,275	1	0	480	1	13,8684	0,143
4,928	0	0,143	0,123	0	68,5714	1	0	0,066
6,575	3,787	5,075	0,786	4,643	282,857	1	41,0592	5,289
7,012	0,921	0,209	0,428	0	102,857	4,5	52,9872	0,789
3,406	1,853	3,141	0,428	0,066	300	0,789	12,264	4,643
6,726	1,141	3,932	0,428	0	180	1	0	0,066
3,766	0,781	0,921	0,066	0	411,429	4,5	40,397	0,143
9,574	0,55	6,566	0,786	1	248,571	0	8,2824	0,066
4,858	1,075	0,781	0,617	0,789	317,143	4,5	0,672	0,789
7,86	1,71	6,066	0,428	4,5	154,286	0	0,2268	0,932
15,713	3,787	6,715	0,246	1	300	1	0	0,789
5,785	1,93	5,855	0	0	197,143	1	0	1,143
1,782	0,275	6,51	0,066	0,789	222,857	0	17,7492	0,789
5,493	1,998	1,075	2	4,5	162,857	0,143	11,8776	0,789
5,768	0,352	1,275	0,123	1	102,857	1	8,5512	0,209
9,079	5,641	1,075	0,428	0	120	0	0	0,932
5,117	1,504	1,352	0,786	0	300	0,429	21,4452	1,066
4,829	0,987	0,132	0,428	0	197,143	1	4,5276	0,789
5,144	2,282	4,13	0,428	0	360	1	0	5,289
7,084	1,71	2,576	0,551	1	137,143	0	12,8352	0,789
7,516	0,198	1,787	1,428	0	342,857	1	37,2176	0,143
8,81	1,93	0,495	1,284	0,789	411,429	1	27,1572	1,066
6,843	1,93	1,644	0,246	1	222,857	0,789	2,688	4,643
7,64	1,919	2,13	0,123	4,5	205,714	1	72,7692	0,143

5,374	1,864	5,284	0,428	0,789	180	1	0	1,143
5,998	1,13	0,418	0,428	1	325,714	4,5	0	1,143
4,412	3,354	0,998	1	0	214,286	0,789	41,0592	0,143
8,066	6,639	3,71	0,786	4,5	274,286	4,5	0	5,289
4,193	1,352	3,921	1,214	0	180	0,143	5,4852	4,566
5,849	1,996	12,51	0,551	0,429	385,714	4,5	6,5604	0,143
7,357	1,93	2,284	0,856	0,066	77,1429	4,5	5,376	0,855
4,127	1,998	2,858	0,786	0,789	257,143	1	13,0872	0,789
13,003	2,139	1,207	0,786	0,789	291,429	1	22,1088	1,143
7,269	1,066	1,921	1,214	0	222,857	0,789	3,1836	0,143
9,648	0,198	0,921	0,551	0	180	1	0	5,289
2,26	1,93	6,341	0,066	0	420	0	0	6,789
3,613	5,487	1,35	0,786	0	222,857	1	11,6004	0,286
3,269	1,427	6,007	0,428	0	137,143	4,5	2,4192	1,143
2,768	1,207	0,495	0,132	1	162,857	4,5	30,1224	0,143
3,623	0,495	0,286	0,428	6	360	1	0	0,789
7,629	0,572	3,644	0,066	6	480	1	15,33	6,066
7,51	1,853	1,418	0,551	0,789	120	1	12,5706	0,143
2,843	1,996	6,341	0,123	1,066	205,714	0,789	30,002	5,289
3,623	2,139	9,495	0,123	0,066	171,429	1	237,902	1,143
5,862	1,198	1,209	0,428	0,789	68,5714	1	14,5236	0,143
5,135	0,836	6,341	0,428	0	360	1	9,408	1,789
4,768	4,852	2,209	1,123	0,789	180	4,5	11,0376	0,066
3,205	1,064	1,075	0,428	0	214,286	1	4,2588	1,143
5,229	4,5	0,143	0,123	0,143	300	1	1,8396	1,218
6,506	1,57	4,93	0,428	1	154,286	0	0	0,932
5,204	5,421	0,132	0,494	6	385,714	4,5	1,6128	0,143
7,223	2,653	1,132	0,428	6	180	1	2,688	0,143
6,849	1,996	4,578	0,786	1,066	154,286	4,5	8,3916	0,789
4,5	1,932	1,209	0,123	0,789	531,429	0,143	0,8064	0,286
3,786	1,427	5,484	2	1	145,714	1	1,6128	0,572
5,875	1,644	2,789	0,856	0	214,286	0	3,4944	0,789
4,214	1,132	0,209	0,123	0	240	0	7,3668	0,066
6,862	0,855	1,932	0,123	1	428,571	4,5	1,8396	4,643
5,55	1,721	1,855	0,428	4,5	180	4,5	0	0,789
2,788	1,284	0,429	0,246	4,566	60	1	185,6064	5,289
1,32	1,064	0,286	0,428	0	180	1	37,6572	0,143
4,137	1,787	1,132	0,428	0	70	0	8,2824	1,578
8,453	0,781	5,284	0,428	1	180	1	0,8064	5,289
6,225	1,427	0,429	0,428	0	214,286	4,5	2,688	1,143
3,937	0,275	3,855	0,428	0,132	222,857	0,066	0	0,789
13,152	6,287	4,075	0,428	4,5	325,714	4,5	21,0672	5,289
8,506	1,776	2,198	0,123	0	180	4,5	0,672	1,789
6,562	1,141	2,352	0,123	1	368,571	1	5,4852	4,643
9,699	0,407	4,143	1	0	120	1	1,6128	4,643
5,559	2,216	2,001	0,786	0	120	0,066	2,7972	0,789
5,149	0,198	2,143	0,551	0,789	154,286	1	2,688	0,143
5,341	2,576	0,341	0,123	4,5	291,429	0	10,2816	6,143

7,959	2,51	1,352	0,428	0	428,571	0,789	0	4,643
7,429	2,073	2,207	0,856	0,855	120	0,143	33,1002	0,789
8,846	0,198	2,352	0,066	0	154,286	0	7,7028	0,789
6,666	1,578	0,987	0,066	0	334,286	1	5,376	1,578
8,084	1,787	0,495	0,428	0	497,143	4,5	57,3272	0,143
5,08	1,787	3,998	0,856	1	154,286	1	30,3212	0,143
4,331	1,71	19,365	4,5	0	240	4,5	8,5218	2
4,282	0,998	0,998	0,123	0	257,143	0,143	3,4944	4,643
5,201	1,853	0,921	0,428	4,5	300	1	6,9972	1,218
8,297	1,644	2,286	0,428	0	282,857	4,5	30,8084	1,143
6,726	1,561	0,143	1	1	188,571	1	36,0024	0,066
3,335	0,132	2,71	0,428	4,5	197,143	0	11,2392	0,789
7,003	1,504	3,51	0,786	0	300	4,5	30,7692	5,289
6,372	2,642	4,495	0,123	1	171,429	1	3,6792	5,289
3,625	1,141	2,932	0,786	4,5	205,714	1	18,396	0,789
7,724	1,776	1,064	0,551	0	300	4,5	8,8704	1,578
2,994	1,064	4,864	0,123	0	240	4,5	0,8064	5,289
3,717	1,218	2,864	0,428	0	282,857	1	0	4,643
5,223	1,064	0,209	0,428	4,5	180	1	2,6964	4,643
6,581	0,979	4,352	0,428	1,143	480	4,5	27,972	1,143
3,72	0,352	8,352	0,428	0,429	420	1	0	4,643
4,768	5,641	0,998	1	0	197,143	1	12,5076	0,143
9,56	1,284	4,132	0,428	0,789	60	1	8,8704	4,566
4,797	2,653	0,066	0,428	0	137,143	4,5	8,1564	1,578
17,372	4,775	1,275	0,674	0	162,857	1	17,1696	6,789
1,548	2,642	2,143	0,856	0,789	342,857	1	33,852	0,932
3,579	1,35	0,143	0,428	0	60	0,789	0	0,143
5,728	1,275	0	1,786	0	257,143	0	0	0,066
3,412	0,979	0,987	0,132	0	342,857	0,066	10,8612	0,932
12,28	1,853	8,286	0,123	1	240	0,789	2,688	6,143
3,988	1,218	1,855	0,428	1	205,714	0,143	31,038	4,643
4,535	2,644	1,13	0,428	0,789	540	1	3,066	0,789
5,847	1,064	1,075	0,428	4,5	411,429	1	30,5004	0,789
3,907	0,495	3,132	0	1	480	1	0	1,789
7,851	1,919	2,932	0,786	0	120	1	20,4876	4,643
5,988	2,007	0,572	0,123	0	240	4,5	28,8288	0,143
3,348	1,855	2,198	1	1	120	1	166,9472	0,789
4,223	1,644	2,495	0	0,932	197,143	0	0	5,289
4,346	2,842	2,286	0,428	4,5	300	1	62,8012	5,289
4,421	1,787	3,075	1	0	197,143	1	4,1076	4,643
4,421	1,141	0,264	0,066	0	420	0,429	19,698	4,643
4,132	0,715	0,987	0,786	0	257,143	1	14,1036	0,143
6,851	2,565	3,141	0,123	1	120	1	5,754	4,643
4,129	5,498	7,429	2,5	4,5	180	1	18,396	5,289
8,351	2,565	5,275	0,428	0	265,714	1	6,5604	6,143
6,423	1,064	0,066	0,856	1	480	1	5,6406	0,789
6,278	1,855	0,341	0,123	0,066	240	0,789	2,9568	1,578
3,478	0,352	2,286	0,428	0,209	462,857	0,143	18,396	0,143

4,214	2,436	8,645	0,066	4,5	420	1	18,396	6,789
7,001	2,576	1,13	0,551	0	240	0,429	3,4944	0,066
6,583	0,987	2,787	1,428	4,566	248,571	1	25,8552	0,143
7,387	1,644	1,504	0	0	137,143	0	0	4,643
5,287	1,207	5,352	0,428	4,5	180	1	4,9056	5,289
2,131	1,075	6,066	0,428	4,5	300	4,5	19,7232	5,289
3,689	1,352	0,638	0,123	0	462,857	4,5	17,794	4,643
15,154	1,284	0,429	1	0	120	0	5,0652	5,289
3,915	1,853	1,275	0,428	0	214,286	4,5	12,264	4,643
3,197	1,636	2,484	0,428	0	420	4,5	0,672	0,789
9,723	0,429	3,721	0,428	0	240	0,143	0	0,572
5,504	1,864	6,561	0,428	0	197,143	0,143	18,648	4,643
8,673	2,796	1,578	0,428	0	265,714	0	0	1,143
5,563	0,495	1,578	0,123	0	274,286	0	0	1,143
4,649	1,853	0,998	0,428	4,5	214,286	1	19,992	0,789
9,437	1,284	0,132	0,428	4,5	360	1	10,752	0,286
4,289	0,855	2,141	0,428	4,5	180	1	27,972	5,289
8,855	1,996	5,416	0,551	0,789	137,143	1	8,5512	0,855
5,928	1,787	0,066	0,189	0	274,286	1	0	0,143
3,783	0,275	0,286	0,428	0,789	257,143	4,5	5,5146	0,143
3,054	0,286	5,066	1	4,5	351,429	0	0	0,286
7,2	1,921	6,286	0,786	0	197,143	4,5	30,002	0,789
6,049	0,998	0,921	0,123	1	180	0	4,5276	0,855
9,828	1,196	0,352	0,428	0	411,429	4,5	6,048	1,143
3,423	1,496	1,578	0	0,789	317,143	1	7,35	1,789
4,986	1,075	0,286	0,428	1	162,857	4,5	2,688	0,789
16,78	2,787	5,787	0,856	5,289	142,857	0,066	270,6256	0,209
7,938	1,141	1,141	0,786	6	480	1	0	5,289
5,99	1,416	8,139	0,428	0	240	0,789	2,8224	1,143
2,26	1,35	0,627	0,066	0	480	4,5	0	5,289
4,852	2,271	2,653	0,428	4,5	385,714	1	7,4592	1,218
7,871	3,222	5,218	0,123	0,855	222,857	1	21,462	5,289
10,285	1,075	4,632	0,066	0	162,857	1	0	0
4,772	0,341	1,429	0,123	4,5	214,286	1	8,1732	4,643
5,585	2,064	0,855	0,428	0	180	0,789	9,324	6,066
4,007	0,484	6,275	0,428	1	120	4,5	9,324	1,578
5,861	0,638	0,638	0,494	0,143	180	0	13,9356	0,932
1,551	0,932	4,275	0,428	0	274,286	0,143	34,9272	6,143
7,372	1,284	2,418	0,428	4,5	240	1	31,038	1,578
6,873	0,77	4,572	0,428	0	300	4,5	18,0012	0,143
6,715	1,493	0,484	0,428	4,5	120	0,789	6,5604	0,143
4,849	1,207	0,484	0,428	0	265,714	1	7,8876	1,578
5,146	1,578	2,864	0,979	4,5	240	1	13,0704	4,643
4,937	1,71	1,644	0,428	1	102,857	1	2,7972	0,066
3,349	0,781	3,855	0,428	1	300	1	12,264	4,566
4,127	1,196	4,286	0,856	1,066	120	1	2,1504	1,143
5,561	1,787	3,293	0,428	0	257,143	1	2,5956	1,578
2,304	1,119	3,13	0,066	0	420	1	5,376	4,643

7,363	1,921	4,218	0,856	0,789	240	0	61,8436	1,789
5,777	0	0	1	0	60	1	1,6128	0,789
5,713	2,631	7,576	0,123	4,5	257,143	4,5	7,2156	0,572
10,204	1,418	0,855	0,123	1,066	94,2857	4,5	60,6424	0,932
5,223	1,064	8,429	0,428	0	368,571	4,5	0	1,789
4,781	1,721	4,132	0,786	0	240	1	99,6352	0,286
3,709	1,132	3,143	0,428	0	77,1429	0	6,132	1,066
4,201	5,696	6,578	0,428	0,789	325,714	1	1,5624	1,578
13,651	1,141	2,139	0,428	0,429	257,143	1	0	0,143
1,834	1,075	8,367	0,428	0	334,286	0	5,376	4,643
6,804	1,284	1,141	0,428	0,789	531,429	1	0	0,789
6,361	0,539	0,352	0,786	1	180	1	7,4592	0,143
7,53	3,156	3,789	0,428	0	257,143	1	65,5228	0,789
2,328	4,156	4,073	0,428	0	197,143	1	9,408	4,643
12,654	0,341	2,855	1	0	162,857	4,5	8,064	0,789
18,806	0	10,007	1	0	248,571	1	0	0
2,557	1,132	6,51	0,428	1	180	1	1,6128	0,143
8,066	2,719	4,482	0,428	0,066	360	4,5	14,0042	4,643
4,49	0,781	1,787	0,786	0	317,143	1	46,62	0,066
14,498	1,71	0,209	0,856	0	60	0,143	2,688	0,143
6,129	1,484	0,495	0,428	0	351,429	1	33,8352	1,143
9,648	2,719	8,855	0,428	1	205,714	1	5,0274	1,578
3,491	1,218	2,286	0,066	0,789	240	1	122,8052	0,932
2,823	1,787	1,644	0,856	1,066	514,286	0	3,8556	0,143
5,005	1,075	5,638	0,428	0	394,286	0	3,1836	0,143
9,664	1,71	1,275	0,428	1	180	1	3,4944	0,143
3,992	2,073	5,064	0,428	0	300	0	0	5,289
6,79	1,207	1,921	0,856	0	120	1	59,3124	1,578
5,512	0,932	0,932	0,428	0	274,286	0,789	2,7972	0
4,207	0,341	10,275	0,428	1,143	180	1	5,376	5,289
3,502	0,286	6,132	1	0,429	113,571	4,5	0	1,789
7,361	1,71	0,143	0,123	0	257,143	0	7,9716	0,143
11,006	0,407	4,932	0,066	1,143	68,5714	1	14,1736	0,143
4,137	1,57	4,51	0,428	0,429	240	1	2,688	0,143
12,955	1,143	1,855	0,428	0,789	368,571	0	0	1,789
4,94	1,13	1,638	1	0	240	1	29,4504	1,789
7,137	2,218	2,71	1,428	1	300	1	39,7152	0,143
5,793	2,284	4,924	0,428	4,5	171,429	4,5	25,6452	4,643
6,007	1,35	0,209	0,856	4,5	180	0	2,688	1,143
9,362	2,51	7,284	0,428	1,789	240	1	0	0,789
2,898	0,418	0,066	0,123	1	257,143	4,5	11,13	0,066
3,92	4,632	5,721	1	0	60	4,5	56,3234	0,143
5,995	2,141	1,275	0,856	0	214,286	0	13,9356	0,143
5,517	1,787	2,715	0,428	0,789	257,143	4,5	0	1,789
6,271	0,275	0,418	2,623	0,429	162,857	0,066	2,7972	0,143
5,787	2,141	2,143	0,428	4,5	240	0,789	2,688	0,143
3,137	2,499	11,776	1	1	282,857	4,5	0	1,789
4,072	1,842	1,207	0,428	0	257,143	1	16,7832	1,143

3,579	1,864	6,352	0,123	1,066	240	1	46,8636	1,143
4,289	1,13	0,921	0,428	1	85,7143	1	37,296	0,789
4,647	0,275	2,132	0,123	1	565,714	4,5	3,78	1
13,774	2,007	2,139	1,214	0	257,143	1	2,8224	0,932
6,504	2,433	6,776	0,551	1	257,143	1	2,7972	1,143
7,361	2,653	0,561	0,428	0,932	120	4,5	0,8064	5,289
10,934	1,075	0,275	0,428	4,5	128,571	0,143	37,3464	0,143
4,555	1,141	1,286	0,246	1	120	0,066	6,72	4,643
3,269	0,561	0,286	0,428	0,932	154,286	1	9,114	0,932
6,704	0,781	0,286	0,123	0	300	1	11,2728	0,066
4,563	2,796	6,498	0,428	0	240	0	0	6,143
6,304	3,071	8,721	1,428	4,5	548,571	4,5	36,0024	1,789
5,5	1,71	1,143	0,428	0,143	120	0,066	0	0,066
3,796	1,787	2,644	0,428	0,429	291,429	4,5	0	0,855
3,898	1,064	2,064	0,428	0	240	4,5	5,5944	0,789
7,214	1,864	2,433	0,428	0	120	1	0	0,143
3,423	1,427	3,275	0,428	0,789	214,286	1	23,7762	0,286
5,135	1,141	0,066	0,551	0,143	240	0	0	0,789
9,716	1,207	1,721	0,123	1	180	1	2,7972	0,143
5,434	5,564	4,787	0,066	1	325,714	1	39,6312	1,789
5,851	2,853	1,789	0,428	0	205,714	4,5	7,9716	1
4,803	3,565	2,932	0,066	1	317,143	4,5	0	0,066
6,089	2,361	2,286	0,428	0	265,714	1	1,8396	4,643
7,027	0,921	4,156	0,428	0	214,286	0,789	3,6792	1,578
4,918	2,853	7,075	1	4,5	360	0	0	5,289
6,842	2,576	2,275	1	1	342,857	0,789	4,1412	6,789
2,865	2,073	4,286	0,786	1	300	1	0	1,143
3,206	1,207	0,275	0,066	0	471,429	1	3,4944	0,143
4,214	0,341	0,286	0,189	0	300	0	0	0,789
3,203	0,209	0,209	0,123	4,5	222,857	1	4,3008	6,066
10,435	1,644	0,198	0,428	0,789	240	1	2,7972	0,143
6,291	1,141	4,218	1,214	0	145,714	4,5	6,8292	0,789
4,586	2,139	7,284	0,428	0	214,286	1	2,7972	1,143
9,663	5,355	2,578	0,428	1	274,286	1	0	4,643
7,598	2,787	6,429	0,428	1	282,857	1	30,66	0,143
4,41	2,075	1,987	1,214	0	197,143	1	13,0872	1,578
16,952	0,855	1,789	0,428	6	162,857	0,789	13,5912	0,789
2,194	0,484	1,864	0,428	0	120	1	10,2816	4,643
8,646	0,987	0,418	0,123	0,429	257,143	1	6,3672	0,066
16,222	0,932	0,789	0	0	257,143	1	5,5944	0
5,643	1,647	5,987	0,123	1	90	1	0,2268	1,143
5,684	1,93	0,198	1	4,566	180	0	0,2268	5,289
4,406	1,35	4,218	1,428	0,429	300	1	0,8064	0,286
6,412	1,064	6,222	0,856	0,789	377,143	4,5	5,376	0,066
4,271	1,141	6,855	0,428	0,789	180	1	0	4,566
4,781	0,789	0,132	0,123	0	240	4,5	13,9944	0,066
6,222	2,073	6	0,428	0	154,286	1	0	4,643
4,069	1,196	1,627	0,246	0	480	1	60,004	0,932

6,429	2,631	0,341	0,494	0	197,143	0,143	2,688	1,578
9,956	1,93	3,939	0,428	0	240	0	9,324	4,643
5,491	0,132	2,561	0,123	0	317,143	0	0	5,289
4,726	2,216	2,51	0,786	1	445,714	0	54,3564	0
10,364	2,007	0	0,428	0	60	4,5	0	0,572
1,702	1,35	0,066	0,066	0	325,714	4,5	0	5,289
6,372	0,932	0,998	0,123	0	214,286	0	2,7972	1,143
3,863	0,858	2,286	0,786	0	222,857	4,5	16,1364	6,789
4,542	1,847	7,064	1	0	317,143	0	22,7892	4,643
6,345	0,55	1,418	0,123	0,143	360	1	2,688	4,566
9,778	1,273	1,787	0,132	0	531,429	4,5	13,9944	0,789
4,849	1,064	1,418	0,428	0,789	180	0,789	25,1244	0,143
8,086	1,787	5,864	0,856	4,5	214,286	0	6,1824	0,143
5,438	1,35	6,429	0,066	0	120	1	8,8704	0,572
10,782	2,13	3,141	0,428	0,066	180	1	51,1952	0,572
6,709	0,396	0,987	0,856	1,066	120	0	18,0012	0,789
10,53	4,011	2,721	1	1,066	120	0	0	0,789
9,874	0,572	2,932	0,428	0,789	214,286	1	0	1,218
3,852	0,693	4,198	0,428	0,066	137,143	1	6,1824	4,643
4,858	1,066	6,075	0,428	4,5	377,143	0	6,1824	0,143
6,713	2,93	5,141	0,428	4,5	222,857	4,5	18,396	0,789
6,851	0,789	0,132	0,428	1,066	420	4,5	4,3008	0,143
6,728	0,495	0,066	0,123	0	120	1	1,8396	0,066
4,939	2,367	1,721	0,856	0	265,714	1	4,032	5,289
2,403	1,075	0,789	0,123	0,789	257,143	0,143	3,6792	4,566
17,163	1,864	1,143	0,428	0	214,286	1	1,8396	0,143
5,586	0,998	2,066	1	1	222,857	1	7,9716	0,932
5,566	5,498	3,855	1,428	0	120	1	16,7832	0,066
4,28	0,921	0,275	0,123	0	282,857	4,5	7,5264	0,143
4,871	2,787	0,998	0,246	1	180	0	0	1,789
2,133	1,787	4,286	0,428	2	480	4,5	10,9704	0,789
6,941	1,787	1,864	0,428	4,5	120	1	10,9704	0,789
9,363	1,064	2,998	0,428	0,066	265,714	4,5	0	0,066
5,21	1,218	2,053	0,123	1,143	197,143	1	0	1,066
4,133	1,787	3,578	0,246	1,066	300	4,5	0,2268	1,066
7,088	6,43	7,286	0,786	0	317,143	1	0	1,578
3,915	0,998	0,286	0,856	0,789	120	0,143	0,8064	0,143
7,44	2,862	0	0,428	0	377,143	1	25,1496	0,143
4,555	0	0,998	0,428	0	317,143	4,5	3,4944	0,789
6,935	3,361	5,275	0	1,789	60	1	60,606	1,789
4,924	0,932	4,429	0,856	6	394,286	4,5	12,264	0,789
6,381	2,653	6,932	0,428	1	197,143	1	2,7972	0,789
2,548	1,141	5,198	0,428	0,855	308,571	4,5	3,4944	1,143
5,553	1,93	1,132	0,786	0	411,429	4,5	29,4336	0,066
1,988	1,075	0,572	0,428	0	445,714	1	12,4782	0,932
5,063	3,51	1,572	0,428	1	222,857	1	0	0,143
5,005	2,293	5,721	0,428	0	317,143	1	4,5276	5,289
11,644	0,209	2,143	0,856	0,143	282,857	4,5	0	0,143

8,093	1,93	5,776	0,246	1	180	1	4,1706	1,218
4,119	0,264	4,132	0,428	4,5	171,429	1	12,4684	0,066
6,26	1,35	0,275	0,428	1	240	4,5	35,378	0,143
5,137	0,407	0,209	1,214	1	428,571	1	20,9916	0,066
9,349	0,286	0,275	0,066	1	240	1	0,672	0,143
4,412	0,921	1,352	0,428	5,289	162,857	0,066	5,5944	0,143
4,062	1	0,066	0,123	0	480	0,066	18,396	1,066
4,711	2,13	4,341	0,856	0	180	1	7,5264	5,289
1,985	1,644	0	0,856	4,5	454,286	0,789	13,8348	5,289
6,713	1,064	4,787	0,428	0	180	1	28,7952	0,066
12,101	1,93	2,418	2,5	0,429	145,714	4,5	0	0,429
5,638	1,075	5,638	0,428	1	257,143	4,5	1,8396	0,932
3,847	2,842	3,064	0,856	1	197,143	1	50,4896	4,643
9,866	0,627	0,998	0,123	1	171,429	0,789	6,048	1,789
6,485	2,708	3,209	0,428	1	300	4,5	0	6,143
9,925	1,998	0,286	0,428	1	145,714	1	5,5944	0,143
5,062	0,264	1,075	0,428	1	51,4286	0	10,0632	0,209
5,57	0,77	4,139	0,123	0	394,286	1	6,8544	5,289
3,684	0,921	0,132	1,066	1	282,857	1	1,6128	1,066
6,352	1,721	1,361	0,428	4,5	180	1	4,8384	0,143
3,772	3,222	1,572	0,123	0	240	1	0	0,143
3,917	3,499	8,418	0,428	0	257,143	0,789	7,5264	4,643
4,992	1,064	15,352	0,428	5,289	180	1	0	0,932
3,489	1,57	8,921	0,123	0	454,286	0,789	5,376	5,289
2,766	1,787	6,141	0,066	1	351,429	4,5	5,376	0,066
3,709	0,418	1,341	0,428	0	120	0	0	4,643
2,625	2,207	3,064	0,428	0,066	394,286	4,5	0	0,789
6,08	1,064	1,075	0,123	0	308,571	4,5	31,4664	0,143
13,565	1,361	0,275	0,428	0	240	1	0,8064	0,143
4,928	0	0,198	0,066	0	120	0	22,8186	0,143
2,92	5,498	9,987	0,856	1,789	420	4,5	36,0024	4,566
8,599	2,642	21,141	0,428	4,5	308,571	6	36,792	0,066
4,774	1,713	0,209	1	4,5	240	0	0	0,789
3,855	2,284	11,218	1	0,143	360	1	22,0752	5,289
5,155	0	2,721	0,979	4,5	154,286	0,789	0	0,789
10,071	1,144	2,864	0,123	0	514,286	0,789	15,0612	1,143
6,849	1,218	4,653	0,428	0	214,286	4,5	8,6282	4,643
3,172	1,198	1,855	0,066	0	480	4,5	6,1824	1,143
4,709	2,073	0,198	0,123	1	497,143	4,5	48,398	1,789
2,414	1,57	8,33	0,066	0	480	1	0,8064	6,789
5,144	1,71	0,198	0,428	4,5	240	0	0	0,066
2,845	4,775	1,932	0,123	4,5	102,857	1	23,3772	4,566
2,482	1,264	3,495	0,551	4,5	120	0,789	67,0012	4,643
5,798	1,578	0	0	0	317,143	4,5	6,1824	0
2,972	2,576	7,064	0,428	4,5	300	1	12,264	6,789
6,139	1,064	0	0,123	0	257,143	4,5	9,9036	0,143
4,497	3,071	7,721	1	6,789	300	4,5	0	6,789
5,203	2,565	5,284	0,428	1	240	4,5	17,9634	0,143

3,206	0,561	5,133	0,123	4,5	454,286	1	21,5796	4,643
3,849	1,853	0,998	0,428	0,143	411,429	4,5	1,8396	0,143
2,197	1,985	0,066	0,123	0	360	0,429	0	4,643
6,694	1,064	0,275	0,066	0,066	317,143	1	1,6128	0,066
8,069	0,473	0,352	0,066	0,143	428,571	4,5	18,0012	0,789
7,304	6,793	0,352	0,786	0	514,286	4,5	46,62	5,289
5,847	0	1,998	0,979	1	257,143	1	0	1,789
5,427	0,429	0,209	0,066	1	137,143	0	0	0,143
3,052	2,132	9,141	0,428	0	420	1	0,8064	4,566
7,449	1,429	2,572	0,123	1	257,143	0,143	20,6892	1,143
2,359	0,998	9,721	0,428	4,5	240	4,5	19,74	1,578
2,917	0,264	1,352	0,428	0,143	240	1	2,7972	0
6,726	1,429	16,286	0,428	0,143	240	0,789	0	0,789
8,432	1,064	0,132	1	0,429	180	0,789	9,3156	1,789
6,427	2,352	4,066	1	1	514,286	1	15,414	1,143
2,436	0,998	9,644	1	1	317,143	1	0	0,143
5,491	1,71	0,998	0,428	1	214,286	6	15,2208	0,143
4,346	1,985	3,578	1,572	0	420	0,143	9,2316	1,143
3,195	2,359	3,495	0,066	4,5	514,286	0	0	6,789
7,807	2,785	15,284	0,979	1	522,857	1	2,016	1,143
1,669	1,35	6,064	0,428	4,5	120	4,5	42,266	0,789
3,367	1,361	0,789	0,428	1	240	1	0	4,5
5,157	2,433	6,789	0,428	1	317,143	4,5	0	0,789
6,258	5,204	4,352	1	0	257,143	1	1,6128	5,289
5,008	0,572	12,572	0,123	0	120	1	8,442	6,143
5,491	1,776	2,132	0,066	0,066	480	0,789	0,8064	6,143
5,658	2,13	5,207	0,428	4,5	257,143	0	0	5,289
7,971	1,064	0,286	0,428	0	360	4,5	6,9888	0,066
1,023	2,433	5,064	0,428	0	240	1	0	0,932
4,555	0,638	6,429	0,123	0	360	4,5	7,9716	0,209
7,372	2,282	2,987	0,123	0,143	600	4,5	23,0748	1,578
3,7	1,064	0,855	0,428	0	222,857	0	6,9384	4,566
8,497	2,864	1,853	0,428	0	180	1	10,2816	0,789
7,581	1,578	0,275	1	0	137,143	1	0	0,066
8,3	1,207	2,932	1	1	240	6	13,608	0,143
9,277	7,416	0,341	0	0	222,857	0	1,8396	0,143
4,858	1,864	7,787	1	0	282,857	1	13,0704	0,572
5,289	3,653	5,864	0,428	0	154,286	0	26,7372	0,143
8,912	1,787	5,721	0,428	0	360	1	69,972	1,789
3,717	1,919	1,075	0,674	0	282,857	4,5	15,414	1,578
5,706	0,627	7,855	2,5	0	120	1	6,1824	5,289
10,628	2,053	0,132	0,428	0	102,857	4,5	6,5016	0,789
6,754	1,427	2,572	0,786	0	265,714	4,5	33,3144	5,289
3,481	1,141	1,207	0,856	0	394,286	4,5	19,7232	0,066
5,001	1,787	5,064	0,428	0,429	300	1	1,8396	5,289
4,299	0,932	8,143	0,428	0,429	77,1429	0,789	0	0,855
5,649	3,365	8,796	0,428	4,5	257,143	1	9,324	5,289
15,303	5,641	7,578	1,214	4,5	197,143	1	0	0,789

7,969	4,698	1,275	0,246	0	180	1	8,2824	1,578
6,982	1,71	2,561	0,123	1	300	1	0,8064	1,143
6,232	1,35	6,35	0,428	0,066	120	1	1,4784	1,066
2,821	0,638	4,341	0,066	1	411,429	1	10,668	1,789
3,122	1,141	2,561	0,123	0	162,857	1	1,8396	0,143
6,554	1,132	1,132	0,066	0	188,571	0	0	4,566
6,508	0,209	3,789	0,428	0	180	1	22,0332	4,643
5,5	1,339	2,776	0,428	0	180	1	0	0,789
4,851	1,93	2,209	0,674	0	205,714	4,5	5,376	5,289
15,291	1,075	2,855	0,428	0	222,857	1	4,662	4,643
6,146	0,858	1,341	0,123	0	282,857	1	2,7972	0,143
7,42	1,921	0	0,123	0	582,857	1	13,0536	0,143
4,135	2,062	0,921	0,428	4,5	120	1	7,9716	5,289
2,054	0,275	17,007	0,786	0	214,286	4,5	0	0,789
8,989	7,998	7,764	1,214	0,132	300	1	0	5,289
5,271	1,998	0	0,428	4,5	282,857	4,5	0,756	5,289
3,049	0,341	0,998	0,428	0,789	265,714	0	0	0,789
3,621	1,119	9,064	0,123	0	531,429	0	0,8064	0,143
16,959	3,508	1,998	0,428	0	240	0,789	22,0752	0,286
4,858	1,996	7,007	0,123	0	360	1	0	1,143
5,718	3,653	3,653	0,551	0	257,143	4,5	9,6768	1,578
4,869	0,275	2,209	0	1,066	300	1	33,2556	0,143
11,296	0,998	0,352	1	0	180	4,5	0	0,932
4,493	1,275	6,987	0,428	1	300	1	2,9148	1,789
4,617	6,21	2,493	1,214	1	300	0,066	25,102	4,643
4,774	1,141	1,143	0,123	1	120	1	36,0024	0,789
10,727	1,713	2,286	0,428	6	394,286	1	6,132	0,143
9,705	0,286	1,998	0,786	0	300	0	0	0,143
6,517	0,998	2	0,123	0	40	0	0	0
5,565	1,644	2,433	0,123	1	308,571	4,5	12,3732	0
3,77	0,998	1	1,428	1	231,429	1	23,324	4,643
4,352	2,776	2,998	0,428	1	180	4,5	0,378	4,643
5,352	1,853	0,352	0,428	1	240	1	17,6652	0,143
2,846	1,484	1,787	0,786	0	240	4,5	0,8064	0,932
8,224	1,636	2,578	0	1,143	454,286	0	0	0,143
5,651	1,776	7,066	0,066	0	120	1	3,4944	0,572
4,291	1,647	5,715	0,428	4,5	214,286	0,143	0	1,789
5,579	1,218	3	0,428	4,5	257,143	0	8,9292	4,643
2,777	1,644	2,141	0,123	1	300	0,066	194,9304	0,789
3,709	0,495	0,715	0,856	0	154,286	4,5	23,3772	0,789
10,589	2,075	3,51	0,428	0,789	437,143	1	1,8396	1,218
8,635	1,853	0,484	0,494	0	197,143	4,5	5,754	1,578
3,13	0,998	1	0,428	1	308,571	1	0	0,066
3,192	1,71	3,998	0,428	0	480	4,5	38,9312	0,143
16,576	7,721	6,929	0,786	0,429	47,1429	0	0	0,932
3,917	1,209	3,647	0,428	0	300	1	8,3412	4,643
1,702	1,776	0,132	1	0	300	1	0,2268	4,643
5,935	1,35	2,787	0,674	1	180	1	26,0652	1,143

8,994	0,998	3,284	1	0	668,571	4,5	4,032	0,932
3,478	1,559	2,636	1	0	317,143	4,5	29,1872	0,143
6,66	2,433	1,932	0,428	0	214,286	0	3,6792	1,143
7,161	0	0,209	0,123	1	514,286	0	0,8064	0,209
4,269	1,075	1,209	0,123	1	154,286	1	10,752	0,789
2,241	0,561	4,275	0,123	0	257,143	1	0	6,143
4,278	0,998	0,286	0,123	0	377,143	4,5	0	0,143
7,285	1,578	2,864	0,428	0	205,714	4,5	13,9944	0,143
3,489	2,367	3,064	0,786	0	214,286	1	17,8584	5,289
7,626	1,864	0,275	0,428	1,143	385,714	0	0	4,643
2,428	1,71	0,066	0,066	0	317,143	0,143	2,688	0,143
5,786	1,578	1,218	2,5	4,566	120	4,5	229,5482	1,143
1,196	1,855	4,143	0	1	120	4,5	2,688	0,066
6,128	0,855	2,932	0,856	1	240	4,5	10,8444	0
8,341	2,064	7,71	0,428	4,643	257,143	1	0	0,143
7,859	2,576	0,275	0,123	0	411,429	4,5	46,62	0
8,077	1,427	4,352	0,123	1,066	240	1	3,6792	6,143
2,774	1,141	0,921	0,428	1	120	1	1,8396	1,789
6,128	1,921	0,132	1,428	1	120	1	9,5172	0
8,497	0,352	3,433	0,189	0,066	205,714	4,5	15,1564	4,566
3,931	2,35	8,209	0,428	4,566	411,429	4,5	18,396	5,289
6,206	1,853	4,5	0,246	0	197,143	6	10,0548	0
8,855	1,416	4,073	0,189	0,932	120	1	125,4764	4,566
1,548	0,33	0,998	0,856	0,789	120	1	27,3252	0,932
12,22	0,066	4,5	1,214	0	300	0	5,8632	0,143
12,509	0,921	1,064	0,123	0	205,714	1	4,5276	0,143
5,429	1,787	2,578	0,428	0	180	4,5	9,324	1,143
3,775	6,221	4,561	0,428	0	300	1	0	4,643
4,348	4,5	0,352	0,123	1	171,429	1	0	4,643
9,727	2,864	0,572	0,428	0	257,143	0	0	0,572
0,77	0,407	2,198	0,123	1	240	1	8,3412	0,143
11,584	6,144	0,855	0,428	0	300	1	2,688	0,789
2,271	1,141	1,644	0,428	1	342,857	1	121,8476	0,143
8,285	1,207	7,221	0,428	0	51,4286	1	0	0,143
5,088	0,275	0,209	0,246	1	368,571	0	0	1,143
2,697	1,644	3,921	0,123	1,578	197,143	0	30,66	4,5
6,355	2,719	5,141	0,428	0	257,143	0	4,5276	5,289
6,86	2,433	9,853	1,786	4,5	120	4,5	0,8064	0,143
5,917	6,21	5,13	1,214	1	240	1	0	1,066
2,903	2,218	0,352	0,428	0	360	4,5	0	0,789
5,557	0,572	0	0,551	0	240	4,5	18,0012	0
4,775	0,998	1,132	0,428	0	162,857	1	9,324	0,932
5,286	1,93	2,15	0,428	4,5	282,857	1	13,7676	0,066
10,102	2,433	2,998	0,428	0	257,143	0	2,7972	4,643
7,785	1,996	1,787	0	0,789	77,1429	0	5,376	5,289
4,339	0,921	1,932	0,066	0	437,143	1	0,8064	0,789
6,735	2,433	0,209	0,428	0	274,286	0	1,8396	0,143
11,081	0,198	3,218	0,428	4,5	531,429	0	8,8704	0,789

3,128	0,932	0,209	0,066	1	180	4,5	2,688	4,566
15,798	1,93	3,064	0,856	0,789	205,714	1	10,9704	1,143
5,278	1,864	5,286	0,123	0	377,143	1	6,5604	4,566
9,785	1,57	5,075	2,5	0	300	4,5	8,2908	4,643
4,99	2,642	2,075	1,572	0,429	248,571	1	23,5536	0,066
4,5	1,787	1,286	0,428	0,143	300	0	0	1,143
5,059	0,704	7,275	0,786	6	420	1	0	0,143
1,749	1,864	1,275	0,066	4,5	240	0,143	6,132	4,643
6,078	1,209	3,209	0,428	1	205,714	1	5,4852	0,286
5,926	3,365	2,644	0,786	0	214,286	1	5,1282	1,143
4,083	0,998	6,789	0,428	0,429	437,143	1	0	0,143
5,349	1,352	1,787	2	0	265,714	1	30,0972	0,143
14,57	0,998	2,789	1	0,429	162,857	1	3,6792	1
3,621	1,864	1,638	0,123	1	360	0,789	38,1752	1,789
5,346	1,075	1,275	0,428	1	214,286	1	3,066	0,789
8,071	4,51	6,864	0,428	0	437,143	4,5	0,2268	5,289
6,795	1,141	2,284	0,189	1	514,286	4,5	12,264	0,143
17,136	0	2,495	1,214	4,5	180	0	0	0,066
6,642	2,642	6,855	1	0,789	231,429	4,5	0,756	1,578
3,918	1,482	2,341	0,786	0,429	265,714	1	2,1504	4,643
1,625	0,418	2,218	0,066	1	300	0,143	0	4,643
9,151	1,064	1,209	0,428	4,5	180	4,5	0	4,643
4,981	1,721	2,352	0,123	0	60	4,5	9,408	0,143
5,781	1,218	8,796	0,428	0,495	171,429	1	18,396	4,643
4,788	0,418	0,066	0,428	0	102,857	1	1,8396	0,132
5,801	2,007	6,721	0,428	4,643	120	1	25,1328	4,643
5,148	0,495	9,361	0,428	1,143	240	1	0	1,143
3,368	4,709	3,209	0,123	0	214,286	1	0,8064	0
4,271	0,627	10,275	0,428	0,066	394,286	4,5	8,5848	0,066
8,491	1,198	4,064	1,214	0	480	1	14,1036	6,789
6,495	1,35	4,51	0,979	0	317,143	1	29,316	0,932
5,201	1,141	0,275	0,123	0,789	437,143	0	12,1212	0,066
8,028	2,939	5,796	0,428	0,143	471,429	4,5	24,0576	1,578
4,498	1,218	6,561	0,428	1	171,429	4,5	12,1212	5,289
5,14	1,427	3,921	0,428	1	265,714	1	6,9468	0,932
6,148	0,418	1,286	0,428	0,143	68,5714	1	6,132	0,066
3,788	1,13	0,429	0,123	0	120	4,5	22,0752	0,789
9,703	0,987	0,495	0,066	0,789	180	0	8,3412	0,932
5,298	1,842	1,495	1,214	0	120	0	6,9972	0,932
6,278	2,853	3,284	0,428	0	240	0	19,2654	1,143
4,7	2,073	1,218	0,428	1	445,714	6	46,3232	0,932
4,138	1,273	2,644	1	1	445,714	1	39,7152	0,789
4,506	1,273	7,207	0,123	0	291,429	1	5,376	4,643
3,482	1,284	1,921	0,123	0	171,429	1	2,688	0,572
4,651	0,209	0	1	0	120	1	0	0,789
5,058	1,053	1,053	0,856	0	197,143	0	8,9544	1,066
11,362	1,275	0,998	0,428	1,066	180	1	2,3184	0,932
3,425	4,841	3,352	0,428	1	600	4,5	10,4832	0,066

4,065	*0,627*	*8,987*	*0,123*	*0,066*	*60*	*4,5*	28,4004	*4,643*
3,548	*1,064*	*2,998*	*0,428*	*1*	*548,571*	*1*	7,9716	*4,643*
3,717	*1,284*	*4,352*	*0,189*	*0*	*240*	*1*	2,7972	*1,143*
4,184	*1,853*	*1,776*	*0,551*	*1*	*214,286*	*1*	32,634	*0,143*
10,503	*1,141*	*2,855*	*0,428*	*4,5*	*60*	*0,789*	7,2156	*0,789*
7,221	*1,13*	*2,007*	*0,428*	*0,789*	*180*	*1*	31,6148	*0,932*
1,188	*3,442*	*2,33*	*0,123*	*0*	*480*	*4,5*	58,282	*6,066*
4,992	*2,216*	*0,418*	*0,428*	*1*	*240*	*0*	18,396	*0,789*
13,154	*0,855*	*2,721*	*0,123*	*0*	*120*	*4,5*	2,7972	*0,066*
4,566	*1,141*	*3,066*	*0,786*	*0*	*240*	*1*	0	*6,789*
5,214	*1,787*	*0,132*	*0,066*	*4,5*	*240*	*1*	23,058	*0,143*
5,581	*1,141*	*1,143*	*0,066*	*1,143*	*248,571*	*1*	0	*0,932*
3,854	*2,93*	*10,576*	*0,428*	*0,932*	*214,286*	*0*	12,264	*5,289*
5,272	*1,141*	*1,075*	*0,428*	*0*	*102,857*	*0,789*	11,2476	*4,643*
5,21	*1,001*	*1,644*	*0,428*	*1*	*360*	*4,5*	13,9944	*0,066*
4,403	*1,644*	*0,352*	*0,786*	*0,066*	*180*	*1*	2,688	*0,932*
6,515	*2,708*	*2,407*	*0,786*	*0*	*94,2857*	*1*	7,4592	*0,143*
3,579	*0,998*	*1,141*	*0,428*	*4,5*	*471,429*	*1*	24,528	*0,143*
4,495	*0,638*	*5,367*	*0,428*	*1*	*120*	*1*	560	*0,789*
10,643	*0,407*	*0,855*	*0,132*	*0*	*162,857*	*1*	1,8396	*0*
10,2	*0,275*	*2,352*	*0,786*	*4,5*	*600*	*1*	42,9464	*0,789*
4,723	*2,367*	*6,644*	*0,786*	*0,429*	*180*	*4,5*	14,2296	*0,789*
4,204	*2,493*	*2,361*	*1,428*	*0,066*	*240*	*1*	20,5464	*0,066*
6,661	*2,499*	*0,143*	*0,428*	*0*	*300*	*1*	139,944	*0,066*
4,703	*1,13*	*0,066*	*0,123*	*1*	*488,571*	*0*	9,324	*5,289*
5,078	*2,719*	*7,064*	*0,123*	*0*	*214,286*	*1*	5,1282	*4,643*
3,282	*1,721*	*1,143*	*0,123*	*0*	*240*	*0,143*	0	*4,643*
5,281	*0,484*	*1,855*	*0,428*	*0*	*282,857*	*1*	9,2484	*0,143*
8,869	*3,653*	*8,286*	*0,786*	*4,5*	*291,429*	*1*	0	*4,643*
8,796	*1,218*	*0,495*	*0,123*	*0*	*231,429*	*0,143*	0	*0*
8,857	*1,93*	*0,998*	*0,123*	*1*	*257,143*	*1*	3,6792	*6,789*
4,939	*2,576*	*2,066*	*1*	*1,789*	*411,429*	*1*	10,2144	*0*
3,214	*1,853*	*0,209*	*1,214*	*0*	*317,143*	*1*	0	*0,143*
4,058	*2,293*	*4,207*	*0,066*	*4,5*	*291,429*	*0,789*	0	*5,289*
5,133	*1,57*	*0,132*	*0,428*	*0,066*	*257,143*	*4,5*	1,89	*1,578*
7,519	*2,499*	*3,853*	*0*	*0,066*	*274,286*	*1*	0	*4,643*
2,843	*1,71*	*4,143*	*0,428*	*4,5*	*342,857*	*4,5*	34,1684	*4,643*
6,917	*1,141*	*18,156*	*4,5*	*0*	*300*	*1*	11,1888	*4,643*
6,137	*1,064*	*0,998*	*0,123*	*4,5*	*248,571*	*1*	3,5532	*0,132*
9,28	*1,352*	*6,075*	*0,066*	*0*	*180*	*4,5*	8,2824	*1,143*
1,254	*1,064*	*2,921*	*0,066*	*0*	*325,714*	*1*	0	*0,066*
2,757	*0,418*	*10,998*	*0,123*	*4,5*	*360*	*4,5*	0	*6,143*
9,857	*2,653*	*1,864*	*0,428*	*0*	*180*	*1*	0	*0,286*
14,48	*1,284*	*0,921*	*0,428*	*1,789*	*137,143*	*1*	19,2612	*4,643*
4,071	*0,998*	*3,493*	*0,066*	*1,066*	*282,857*	*1*	2,688	*0,143*
5,056	*1,273*	*2,407*	*0,123*	*4,5*	*360*	*1*	4,662	*6,789*
8,741	*1,644*	*1,858*	*0,428*	*1*	*480*	*1*	4,662	*5,289*
9,848	*1,075*	*1,352*	*0,066*	*1*	*137,143*	*0*	25,6452	*0,143*

10,428	1,35	1,71	0,428	0	248,571	1	25,1202	0,789
2,406	0,495	2,132	0,066	4,5	240	1	35,4312	0,066
4,267	1,985	1,207	1,786	0,789	480	1	186,9504	0,789
8,933	2,064	1,264	1,066	0	120	0,789	67,0012	0,789
4,073	1,93	4,198	0,066	0,066	137,143	0,789	6,132	4,643
7,835	3,367	1,998	0,428	4,5	300	1	30,9666	0,789
5,724	1,35	1,196	0,123	1	222,857	0,789	6,9972	0,286
4,118	1,119	10,627	1,786	0,429	162,857	1	15,3384	1,143
8,275	1,361	1,132	1,214	0,789	308,571	1	0	0,143
12,438	1,853	0,209	0,856	0,066	351,429	0,066	6,5604	0,572
2,395	2,719	11,996	0,428	4,5	180	4,5	3,4944	0,789
5,509	1,559	7,207	0,428	0	240	1	6,9972	1,143
8,721	0,932	1,286	0,428	0	77,1429	0,789	0	0,143
5,284	1,207	0,143	0,428	0	120	1	2,1504	4,566
7,653	2,576	6,638	0,428	1	257,143	1	30,7692	1,143
12,865	0,998	0,143	1,786	0,789	120	1	9,6264	0,143
5,364	1,71	7,361	0,428	4,5	92,8571	4,5	43,5512	4,643
8,159	1,493	6,484	0,428	1	197,143	1	30,9232	1,143
5,717	1,495	3,286	0,123	0	274,286	1	8,064	1,143
6,876	1,13	2,57	0,123	0	137,143	1	11,662	0,143
3,995	1,853	1,484	0,428	0	334,286	1	4,3092	5,289
10,861	3,288	10,218	0,551	1,789	60	4,5	15,6366	1,218
13,645	6,004	13,867	0,786	0	257,143	4,5	2,688	0,209
2,766	1,141	1,075	0,428	0	342,857	1	8,7486	1,578
2,777	0,352	0,495	0,551	0,429	240	1	11,6928	5,289
5,284	1,053	1,13	0,066	0	394,286	0	6,132	0,143
7,719	1,13	0	2,5	0	300	4,5	6,9468	0
2,997	0,998	0,143	0,428	0	180	4,5	17,2242	1,578
3,28	1,196	2,721	0,123	0	282,857	1	11,0208	0,789
3,977	1,987	9,064	0,428	0,789	454,286	4,5	32,634	4,5
7,851	2,719	3,864	0,551	4,5	240	1	0	1,143
4,511	4,5	4,143	0,786	0	137,143	4,5	0	6,143
2,904	1,921	2,433	0,856	0	180	1	38,9424	0,143
3,841	1,864	6,998	0,786	1,429	480	0	32,69	1,143
3,049	1,064	8,341	0,123	0	360	4,5	0	5,289
2,955	0,495	10,275	0,123	0	197,143	1	2,1504	4,643
6,928	1,361	3,352	0,428	0,143	120	1	4,1664	1,789
4,351	0,627	5,275	0,428	0	240	4,5	4,8384	5,289
4,862	1,064	3,864	0,786	0	120	4,5	6,132	4,643
3,776	2,71	9,787	0,428	6	300	0	0	5,289
3,979	0,275	3,341	0,123	1	120	1	12,264	0,932
6,078	2,139	4,433	0,786	0,429	214,286	1	21,3528	4,643
4,71	2,71	4,787	0,123	0,789	325,714	0	6,9888	1,143
4,852	1,855	5,71	0,428	1	257,143	1	16,926	1,143
10,782	1,218	1,921	0,123	0	60	1	13,9944	0,932
6,625	0,209	8,561	0,428	4,5	154,286	1	0,8064	0,932
4,281	2,075	4,198	0,786	6,789	531,429	0,429	0	6,143
3,917	0,987	2,352	0,123	0	274,286	0	0,756	4,643

3,621	1,93	0,341	0,428	0	411,429	0	5,5944	0,143
5,157	2,433	2,572	0,428	0	180	1	23,058	5,289
3,48	0,209	1,198	0,123	1	162,857	4,5	3,4944	6,143
2,326	1,35	6,721	0,428	1	360	1	0	0,855
7,849	1,427	0	0,428	0	240	1	18,0012	0,066
5,869	0,495	1,855	0,428	0	308,571	4,5	27,0396	0,066
3,109	0,561	6,141	0,428	1	222,857	1	0,672	1,143
5,651	2,721	2,198	0,551	1	222,857	1	6,9972	0,789
4,768	2,141	9,71	0,674	1	77,1429	4,5	6,9384	0,572
6,221	0	1,71	1,214	0	240	0,789	54,6504	0,066
7,003	1,71	4,143	1,428	4,5	180	0	0,8064	0
6,21	2,139	8,644	0,428	0	214,286	4,5	0	4,566
5,215	1,779	4,864	0,428	1	240	1	0,756	1,578
3,365	2,499	9,998	0,428	1	531,429	0	8,8704	1,143
3,775	1,13	0,066	0,428	0	120	1	29,19	0,143
11,362	3,299	7,141	0,786	0	300	1	36,8172	0,143
9,875	1,504	3,075	1,428	1	317,143	1	38,1752	1,218
5,506	2,293	6,787	0,428	0,789	248,571	0	2,016	1,143
6,425	1,218	4,275	1	1	205,714	0	3,4944	0,143
2,683	2,207	12,418	2,5	1	300	4,5	2,1	1,143
2,978	5,432	0,209	0,786	1	317,143	1	36,0024	0,789
3,713	1,787	5,064	0,428	0	171,429	1	0	5,289
2,777	1,218	2,932	0,856	0	300	0	0	0,932
6,012	0,495	1,075	0,066	0	454,286	4,5	25,1412	0,572
3,86	2,644	0,33	1,572	0	120	0,066	10,668	1,578
7,43	0,987	0,066	0,428	0	248,571	4,5	38,066	4,643
8,959	1,578	1,998	0,123	0	300	0	0	5,289
4,921	0,781	1,572	0,428	0	300	4,5	0	4,643
5,99	2,499	0,987	1,428	1	180	4,5	12,5076	0,789
8,523	1,644	0,286	0	0	102,857	4,5	23,8322	0
7,293	1,361	3,578	0,123	0	120	0	0	5,5
1,57	2	0	0,123	0	394,286	0,789	0	4,643
5,629	2,075	6,209	0,674	4,5	197,143	4,5	9,8532	0,143
5,006	1,842	5,284	0,428	0	171,429	0,789	1,4784	5,289
3,996	1,578	0,132	0,428	0	171,429	1	5,376	6,066
5,003	1,721	1,932	0,428	0	317,143	1	18,8776	1,578
5,782	1,207	6,864	0,428	4,5	342,857	4,5	11,6592	5,289
4,421	0,209	6,132	0,428	0	240	0	2,7972	4,643
11,795	1,198	3,064	0,428	0,789	248,571	1	22,6324	0,932
9,505	0,932	2,066	0,123	0	180	4,5	0	0,143
5,27	2,139	1,207	0,428	0	240	1	36,3972	1,789
5,643	1,416	2,064	1	1,066	240	1	22,3272	0,932
5,218	1,275	0,286	0,428	0	402,857	0,143	25,6564	0
3,214	1,853	23,789	1	4,5	60	6	0	4,643
5,493	0,855	1,066	0,428	1	171,429	4,5	5,8716	0,066
6,001	1,207	8,352	0,428	0,143	257,143	1	2,7972	1,143
4,509	2,499	6,13	0,369	0,789	360	1	5,8716	1,789
4,478	1,196	1,198	0,428	0	137,143	0	18,396	4,643

4,497	0,998	0,132	0,123	1	282,857	0,789	1,5624	4,643
8,782	2,642	5,643	0,856	0,066	180	1	9,324	0,932
4,698	1,787	6,998	0,856	4,5	257,143	1	2,688	5,289
8,374	1,787	1,987	1,428	0	180	1	2,688	0,932
20,939	2,433	3,787	1,786	0,429	171,429	0,429	0	0,066
8,266	1,787	1,578	0,428	0	240	1	0	0,789
3,338	0,759	4,264	0,786	0,789	240	0,429	20,4876	6,066
5,579	1,721	4	0,428	1	617,143	0	0	4,643
13,073	0,921	0,066	0,856	1	180	1	18,648	0,066
2,612	0,418	7,141	0,123	0,789	188,571	0	4,1412	1,578
3,577	1,218	9,209	0,428	0	171,429	1	6,1824	0,143
7,8	1,264	0,987	0,428	0	360	1	9,198	1,789
3,643	1,284	1,209	0,123	1	360	1	0	0,143
4,858	0,132	0,275	0,428	0	120	0,143	8,9292	0,143
10,362	5,498	0,198	0,189	1	591,429	1	4,8384	0,132
6,196	1,578	0	1,428	0	162,857	1	4,662	0,789
4,772	0,561	1,352	0,123	1	205,714	1	2,7972	4,566
10,15	5,852	2,71	0,428	4,5	180	1	0	5,289
6,568	1,996	3,207	1	0	300	1	3,4944	0,932
6,144	1,209	3,864	0,428	0,066	240	0,429	24,528	4,643
3,335	1,273	15,429	0,428	4,5	360	0	1,8396	4,643
6,574	1,141	1,064	0,428	0,789	240	0,143	1,512	0,143
5,913	1,13	1,855	0,428	0,789	197,143	1	6,1824	0,789
8,352	1,35	6,286	0,123	0	257,143	1	3,4944	6,143
3,15	2,653	3,721	0,066	0,789	180	1	0,8064	5,289
5,706	2,787	1,578	0,428	0	240	1	2,1504	5,289
1,837	1,644	0,209	0,189	4,5	428,571	0	20,9244	4,566
5,689	1,064	0,932	1,066	0	257,143	4,5	17,7828	0,066
5,709	0,561	2,075	0,786	0	214,286	1	15,4476	6,143
4,786	1,13	1,132	0,428	0	240	1	17,6652	0,789
5,082	1,93	0	1,428	4,643	360	0	0	0
7,007	1,209	0,932	0,123	0	222,857	6	7,4592	0,143
4,509	0,418	4,352	0,428	0,789	300	1	7,2156	1,578
7,06	1,853	3,853	1	0,789	180	4,5	15,8256	5,289
3,471	0,998	0,209	0,123	0	180	1	12,8352	1,143
10,006	1,141	2,066	0,856	0	120	1	46,648	0,143
6,082	4,929	4,209	0,428	4,5	420	4,5	21,4452	0,143
9,917	1,71	1,286	0,786	0	240	1	28,9184	6,143
9,848	2,776	1,284	2,5	4,5	240	0	6,9888	4,566
7,15	1,776	4,484	0,428	0	240	1	6,9972	0,143
7,449	0,921	2,433	0,428	4,5	360	1	0	0,789
5,155	2,007	1,578	0,786	0,855	162,857	0	0	0,429
3,7	1,209	1,998	0,123	1	154,286	4,5	32,69	4,643
3,405	0,855	1,132	0,123	1	111,429	4,5	60,004	0,066
10,498	1,218	2,576	0,428	4,5	111,429	0,789	8,064	0,789
3,917	8,511	0,998	0,123	1	565,714	4,5	8,8704	1,143
2,276	1,141	6,352	0,428	0	137,143	0	11,5584	1,789
4,48	1,141	2,572	0,123	0	240	1	0,8064	1,143

7,071	2,565	2,218	1,123	4,5	120	1	34,9832	0,789
4,269	1,853	6,064	0,428	0,789	360	1	10,794	4,643
14,363	0,855	5,643	0,551	4,5	180	0,789	0,8064	0,789
9,288	1,559	0,407	0,428	0,066	222,857	1	6,9888	0,286
12,882	0,932	4,156	0,428	4,5	240	0	0,8064	0,143
6,146	1,075	1,209	0,123	0,143	582,857	1	0	0,932
5,63	1,647	0,998	0,428	1	317,143	1	0,8064	1,789
3,192	0,638	2,209	0,066	0	360	0,789	1,5624	0,066
6,137	0,275	1,789	0,123	1	77,1429	1	12,4684	5,289
6,442	1,284	0,132	0,066	4,5	360	0	19,439	0,066
2,318	0,418	0,132	0,428	0	308,571	0,429	1,8396	0,143
4,703	0,341	0,627	0,856	0	308,571	0	0	0,572
4,06	0,921	2,578	0,551	1	240	1	10,1304	4,566
4,877	1,418	3,218	0	0	197,143	4,5	21,4452	4,643
8,732	1,064	1,493	1	0	180	1	2,1504	0,789
3,497	1,644	0,198	0,123	0	402,857	4,5	31,6652	0
7,207	0,627	0,921	0,856	1	394,286	4,5	30,4374	0,143
19,002	1,721	9,075	0,428	0	60	4,5	6,132	0,789
7,95	1,075	0,209	0,123	0	265,714	1	55,0116	0,143
8,132	4,643	0,264	0,428	0	180	0	0	0,932
4,995	1,864	5,066	0	1	531,429	1	5,376	6,143
4,491	0,275	0,998	0,428	1	634,286	0	8,9292	0,143
5,57	1,842	1,218	0,428	1	282,857	1	13,9944	0,143
5,5	1,493	8,932	0,428	1,578	300	1	0,2268	0,932
5,726	2,785	5,207	0,123	0	531,429	4,5	3,8724	5,289
4,564	0,781	6,418	0	0	240	4,5	0	0,143
5,429	1,262	1,864	0,428	0	240	4,5	0	0,789
8,846	0,913	1,855	0,852	4,566	120	4,5	9,2778	0,143
8,096	2,073	3,721	0,428	1	240	1	27,972	5,289
5,144	0,693	1,864	1	0	180	0	11,0376	5,289
8,148	6,364	2,218	0,428	0	197,143	4,5	42,1344	5,289
5,07	2,499	3,921	0,428	0	111,429	4,5	2,7972	1,578
3,908	0,627	1,066	0,428	5,289	428,571	4,5	0	1,789
4,785	0,286	6,632	0,123	0	205,714	1	2,7972	0,932
7,367	1,985	2,864	0,066	0	300	1	0	0,855
7,08	1,275	0,418	0,123	0,066	240	1	11,1552	1,578
8,558	2,578	10	2,5	4,5	197,143	4,5	47,6644	1,143
16,577	0,998	1,286	0,428	1	351,429	0	0	0,143
7,238	2,653	1,776	0,428	1	240	4,5	31,038	0,143
6,194	1,919	0,352	1	0	222,857	1	6,5604	5,289
6,293	1,996	7,284	0,428	0	197,143	4,5	6,9972	1,143
6,306	2,075	0,143	0,066	0	282,857	1	17,1528	0,066
7,448	3,508	1,352	0,428	0	240	1	22,0752	5,289
5,216	0	0,066	0,123	0,143	180	1	66,794	0,066
3,625	1,578	1,132	0,066	1	205,714	1	11,5584	0,855
5,854	1,067	7,721	0,428	0	145,714	4,5	0,189	1,143
4,853	6,287	9,15	0,856	0	240	6	31,1556	1,143
5,727	1,064	1,141	0,428	0	617,143	1	7,3962	0,143

1,408	1,284	1,495	0,428	0,789	180	1	10,0632	0,789
8,165	1,985	4,504	0,428	0,066	137,143	4,5	28,0392	4,643
4,7	1,284	0,209	0,428	1	240	0,789	79,4472	0,143
8,412	0,209	7,064	1,123	0,429	480	0,143	15,6282	0,143
6,219	2,273	0,275	0,428	0,855	402,857	1	0	0,143
5,849	1,273	6,407	0,551	1	120	1	11,4744	1,066
9,992	1,987	3,064	1,066	4,5	94,2857	1	48,9328	4,566
8,02	1,504	8,341	0	4,5	300	6	34,9272	4,643
19,776	0,198	1,789	0,428	0	300	0,789	0	0,789
3,552	1,141	7,286	0,428	0,789	154,286	4,5	10,752	0,789
9,275	1,273	4,075	0,428	0	214,286	1	0	4,643
3,401	1,075	0,352	0,428	0,789	300	4,5	5,6448	1,578
7,721	0,264	0,921	0,123	0,789	368,571	0,789	6,9972	0,789
4,932	1,998	8,132	0,428	0,066	257,143	4,5	0	5,289
4,772	2,919	3,209	0,428	1	360	1	0	5,289
2,636	0,921	0,209	0,123	0	171,429	4,5	0,2268	0,143
4,357	1,286	6,286	0,428	0	300	1	4,5444	0,789
7,207	0,132	1,855	1,428	1	265,714	1	6,132	0,143
3,64	1,787	0,932	0,856	0,789	360	1	1,8396	4,643
5,861	0	0,855	0,856	0	120	4,5	2,7972	0,932
5,082	4,786	5,066	0,428	0	214,286	0	0	0,789
4,777	6,078	5,207	0,428	0	180	4,5	23,926	0,789
5,429	0,924	3,367	0,428	1	411,429	0	0	0,789
2,634	0,715	3,418	0,786	1	120	1	2,7972	4,643
3,988	0,998	2,998	0,428	0	300	4,5	7,35	4,643
4,344	6,287	2,418	0,189	0	325,714	1	10,752	0,209
2,777	1,787	2,998	0,856	0	180	1	26,7372	0,789
14,359	1,35	2,484	0,428	0	334,286	1	15,9432	0,209
6,642	0,352	1,209	0,428	0	291,429	4,5	60,004	0,143
4,558	2,642	6,427	0,428	4,5	240	0,143	25,5864	6,789
3,863	0,572	0,921	0,132	0,143	70	1	1,8564	0,209
3,621	1,416	3,721	0,786	4,5	257,143	1	43,5512	0,143
6,216	1,207	0,209	0,428	0	300	1	9,324	4,566
3,931	1,35	2,561	0,123	1	317,143	1	11,6592	1,789
5,649	0,209	6,921	0,066	4,5	531,429	4,5	2,688	4,643
4,335	0,561	2,075	0,428	0	240	1	51,1952	1,578
2,425	1,275	3,209	0,123	0	197,143	0	0	0
5,14	1,987	7,264	1,572	4,5	240	4,5	19,3872	0,143
7,763	6,078	8,216	0,428	0	180	1	2,3184	6,789
3,632	1,853	1,075	0,428	0	360	1	0	1,578
8,164	0,932	0,638	0	1	197,143	1	0	0,066
3,537	0,998	1,209	0,123	1	274,286	1	6,5604	5,289
4,348	4,5	2,209	0,066	4,5	325,714	0	16,926	0,855
8,989	1,275	0,209	1,214	0	180	1	8,8704	0,143
4,349	1,141	5,207	0,786	0	360	1	4,662	0,143
3,258	1,93	7,576	0,123	4,5	300	1	0	0,143
4,131	2,139	6,143	0,428	0,572	137,143	4,5	18,396	0,932
3,338	5,498	1,209	0,246	0	265,714	0	1,8396	0,143

14,994	*1,418*	*2,286*	*0,786*	*0*	*257,143*	*1*	0	*0,143*		
5,075	*0,341*	*1,504*	*0,428*	*0*	*402,857*	*4,5*	9,5172	*0,066*		
4,843	*2,994*	*7,998*	*0,428*	*0,066*	*180*	*4,5*	34,9272	*4,566*		
6,363	*1,787*	*5,721*	*0,428*	*1*	*197,143*	*4,5*	0	*4,643*		
7,524	*2,51*	*2,218*	*0,428*	*0*	*300*	*1*	23,9904	*4,643*		
5,688	*0,264*	*1,066*	*0,066*	*0*	*360*	*4,5*	17,5812	*1,143*		
4,583	*2,367*	*3,945*	*0,428*	*0,066*	*240*	*0,789*	37,296	*0,066*		
6,992	*2,919*	*1,209*	*0,786*	*0*	*240*	*1*	4,5276	*6,143*		
5,5	*1,919*	*5,418*	*2,5*	*1,066*	*240*	*1*	9,9036	*1,789*		
6,273	*1,35*	*0,407*	*0,551*	*0*	*248,571*	*4,5*	17,6652	*0,932*		
4,425	*1,644*	*0,789*	*0*	*0*	*197,143*	*1*	23,9904	*0,789*		
9,349	*1,493*	*1,361*	*0,428*	*0,789*	*70*	*0*	7,644	*0,209*		
3,559	*2,644*	*6,284*	*0,428*	*4,5*	*257,143*	*4,5*	1,8396	*4,643*		
6,278	*3,156*	*1,787*	*0,856*	*0*	*531,429*	*4,5*	43,1312	*0,066*		
3,117	*1,064*	*0,418*	*0,189*	*4,5*	*385,714*	*0*	0	*0,143*		
8,66	*1,853*	*0,855*	*0,428*	*1*	*257,143*	*1*	0,8064	*1,143*		
3,621	*2,007*	*8,642*	*0,066*	*0*	*394,286*	*1*	8,8704	*6,789*		
14,293	*0,913*	*0,429*	*2,928*	*1*	*120*	*1*	4,9056	*0*		
4,918	*2,064*	*5,578*	*0,428*	*0*	*231,429*	*1*	0	*0,932*		
3,915	*2,139*	*2,627*	*0,428*	*1*	*317,143*	*1*	159,964	*0,789*		
5,788	*5,498*	*1,361*	*0,428*	*4,5*	*300*	*0,789*	77,4564	*0,143*		
4,285	*1,427*	*4,932*	*0,428*	*0*	*351,429*	*4,5*	49,4172	*1,066*		
4,555	*2,785*	*1,075*	*0,786*	*0,066*	*154,286*	*4,5*	18,6592	*1,789*		
2,9	*1,919*	*10,495*	*0,428*	*0,066*	*137,143*	*0,066*	12,264	*5,289*		
6,442	*1,985*	*5,299*	*0,856*	*0*	*171,429*	*4,5*	0	*4,643*		
8,736	*1,713*	*2,484*	*0,786*	*4,5*	*154,286*	*0,789*	25,3162	*0,855*		
3,937	*1,721*	*2,998*	*0,123*	*1*	*180*	*0*	0	*1,143*		
8,871	*3,222*	*7,787*	*0,428*	*0*	*120*	*4,5*	3,4944	*1,578*		
4,139	*0,932*	*0,998*	*0,123*	*0,789*	*317,143*	*4,5*	5,4852	*0,143*		
4,486	*1,066*	*0,209*	*0,428*	*0*	*240*	*1*	0	*0,066*		
5,067	*0,921*	*3,721*	*1,214*	*0*	*154,286*	*0,789*	12,264	*0,143*		
4,339	*4,918*	*9,789*	*1,428*	*0*	*394,286*	*1*	6,1824	*1,143*		
5,35	*1,361*	*4,864*	*0,856*	*0,789*	*137,143*	*1*	18,7656	*5,289*		
4,647	*1,284*	*0,286*	*0,428*	*0*	*240*	*4,5*	30,7692	*4,643*		
5,432	*1,141*	*0,352*	*0,551*	*0,066*	*180*	*4,5*	18,648	*0,143*		
5,291	*0,275*	*1,644*	*0,428*	*1*	*51,4286*	*0*	3,4944	*1,789*		
4,057	*2,721*	*2,932*	*0,428*	*1*	*60*	*4,5*	4,5276	*6,143*		
7,787	*1,853*	*0,341*	*0,551*	*0*	*222,857*	*1*	0,8064	*0,143*		
2,293	*0,789*	*0*	*0,428*	*1*	*257,143*	*0*	12,264	*4,643*		
5,717	*2,367*	*2,495*	*0,856*	*1,066*	*360*	*0*	0,8064	*5,289*		
5,836	*0,987*	*1,787*	*1,786*	*0*	*377,143*	*4,5*	3,066	*0,789*		
9,36	*1,919*	*4,576*	*0,428*	*0,143*	*317,143*	*1*	3,3516	*5,289*		
12,285	*2,141*	*3,218*	*0,551*	*0*	*360*	*4,5*	5,4852	*0*		
4,996	*0,932*	*0*	*0,856*	*1*	*231,429*	*1*	0	*0,789*		
10,825	*0,998*	*2,721*	*1*	*0,855*	*180*	*0*	0	*5,289*		
4,405	*1,284*	*2,721*	*1*	*0*	*77,1429*	*4,5*	2,9064	*1,789*		
8,866	*0,209*	*2,352*	*0,428*	*4,5*	*360*	*1*	16,6824	*0,143*		
3,698	*0,693*	*1,35*	*0,428*	*0,066*	*257,143*	*4,5*	100,6992	*1,789*		

6,223	0,987	1,141	0,428	1	205,714	4,5	13,9944	0,066
9,294	0,132	2,418	0,123	0,789	197,143	1	14,3724	0
4,467	2,644	10,216	1,284	0,789	462,857	1	11,9364	4,643
9,415	1,919	2,71	1	1	222,857	0,429	5,1996	4,643
9,771	0,143	4,066	0,066	0,789	120	1	1,8396	0
7,665	1,644	2,932	0,123	0	300	4,5	18,396	0,143
8,952	1,207	0,418	0,909	4,5	240	4,5	20,9916	1,143
17,809	1,207	1,418	0,428	0	180	1	0,672	0,143
5,157	6,221	5,075	1	0,143	274,286	4,5	0	0,143
3,841	1,998	2,998	0,428	0	257,143	0,789	8,8704	0,066
6,077	1,064	1,998	0,428	0	102,857	1	5,8632	0,143
2,112	1,132	2,787	0,428	0	300	1	2,7972	0,789
3,577	1,207	3,207	0,428	4,5	325,714	0	79,4192	4,643
5,133	2,787	1,578	0,786	4,5	360	4,5	57,974	0,066
5,294	1,427	2,284	0,551	0	257,143	1	22,2516	0,789
12,303	1,141	5,007	0,428	1	120	1	7,2156	4,643
5,42	2,796	17,578	0,428	1,143	257,143	FAŁSZ	13,9944	5,289
5,799	1,141	2,79	0,428	0	240	1	66,3992	4,643
3,851	4,632	3,286	0,551	0,143	214,286	0	0	0,143
4,128	5,487	9,418	0,428	4,566	120	0	0	0,066
10,31	5,632	1,352	0,428	4,5	180	0	2,7972	5,289
4,48	0,921	1,484	0,856	4,5	514,286	1	14,7084	1,143
7,583	0,066	0,561	0,066	0	51,4286	1	19,2612	0,066
6,142	0,715	7,919	0,856	1	154,286	4,5	30,9204	1,143
6,871	1,13	0,352	0,066	1	120	0	22,7892	0,932
5,99	1,418	1,35	0,786	1	102,857	4,5	8,1732	1,789
6,438	2,787	7,576	0,428	4,5	180	1	149,996	5,289
5,003	0,924	4,721	0,786	0,789	394,286	1	0,8064	0,429
6,766	2,787	3,638	0,786	1	240	1	0,8064	1,789
7,575	1,998	0,932	0,428	0	240	0,789	1,344	0,789
3,12	0,132	0,572	0,123	1	265,714	0,066	9,324	6,143
7,866	1,842	10,484	1,428	0	120	4,5	4,662	5,289
6,783	0,932	3,075	0,428	4,5	308,571	1	19,0092	0,932
8,506	0,627	1,352	0,856	0	222,857	1	9,5172	0,789
4,841	2,139	4,15	0,786	0,789	428,571	1	30,66	6,066
7,708	1,209	3,066	0,428	0,789	360	1	0,756	5,289
4,5	2,073	5,143	0	0	120	1	0	4,643
1,57	4,852	0	1	1	420	4,5	0,8064	1,789
5,5	1,57	7,998	0,428	0,143	197,143	1	85,0416	0,932
5,935	1,141	1,075	0,428	4,5	257,143	1	14,4592	4,643
4,499	2,644	5,286	0,786	4,5	240	1	2,688	5,289
8,268	1,13	1,286	0,123	4,5	317,143	0,789	3,4944	4,643
5,286	2,062	5,721	0,428	0,066	360	1	27,972	0,143
7,867	2,787	1,218	0,428	0,789	317,143	0	0	1,578
5,646	1,987	2,932	1,572	0,429	282,857	1	0	0,789
3,996	1,207	10,704	0,551	1,066	197,143	0	0	0,066
2,474	1,141	0,987	0,123	0	240	4,5	3,4944	1,789
4,483	1,644	6,264	0,428	0,143	180	1	16,4052	6,789

6,933	0,704	8,143	0,123	0	497,143	1	13,986	1,789
6,489	2,565	1,853	0,786	1	471,429	0,789	6,132	1,578
4,862	1,789	1,781	2,5	0,429	325,714	4,5	12,6882	1,578
7,018	1,787	3,352	0,428	0	180	0	0,8064	0,143
3,265	1,143	5,13	0,428	0	300	1	0	0,143
2,199	1,209	9,341	0,123	1,066	137,143	1	18,648	1,143
3,349	0,638	2,787	0,428	0	394,286	0	0	6,143
3,403	1,275	3,132	0,123	4,5	540	1	9,324	0,209
7,579	1,141	1,429	0,123	1	17,1429	1	2,9568	0,066
7,796	0,715	2,275	0,786	0	240	1	46,62	6,143
5,704	0,921	0,341	0,428	1	120	1	0	4,643
4,28	1,198	8,275	0,786	0	257,143	1	14,1036	5,289
5,776	0,627	4,209	0,494	1	497,143	1	10,2564	0,066
5,797	1,075	2,066	0,123	0	102,857	4,5	0	0,143
3,5	1,427	13,075	0,786	0	420	1	0	0,143
6,495	2,787	1,787	0,428	0	240	4,5	71,3272	5,289
5,866	2,644	1,787	0,428	0,429	360	1	0	0,789
7,071	1,987	2,855	1,214	1	120	4,5	35,0392	0,932
5,074	0,429	8,721	0,786	0,066	214,286	0,143	5,5944	1,143
2,917	1,93	0,066	0,123	1	274,286	1	10,038	4,643
5,394	1,132	0,132	0,123	0	77,1429	4,5	10,752	0,066
6,509	1,132	0,715	1	0	240	1	7,8876	0,789
7,879	2,576	2,367	0,786	1	180	0	30,002	0,932
6,221	2,367	6,007	0,786	0	162,857	0	13,5912	5,289
5,486	2,064	0,341	0,494	0	94,2857	0	12,264	0,932
4,209	1,721	5,198	0,428	0	480	1	8,8704	1,578
11,718	0,264	0,998	0,786	0,789	300	0,143	67,8132	0,286
8,868	2,862	7,996	0,551	0	257,143	0	0	4,643
9,571	1,919	2,352	0,132	1	565,714	4,5	23,3352	1,143
5,856	1,273	7,284	0,428	0,143	120	1	6,4722	0,932
10,292	1,207	3,51	0,856	0	257,143	1	6,5436	0,143
7,801	1,71	2,15	0,428	4,5	360	4,5	49,7644	0,789
4,78	1,196	7,853	0,786	0	377,143	1	10,4034	4,643
5,926	0,627	0,855	0,123	1	351,429	1	12,1212	4,643
5,005	1,787	5,578	0,428	0,789	454,286	1	13,5492	0,789
6,346	1,561	3,066	0,428	1	308,571	1	34,9692	1,789
3,911	1,548	2,418	0,428	0	171,429	4,5	23,324	5,289
6,012	5,861	6,143	0,428	0	214,286	1	0	0,932
5,636	1,493	4,787	0,786	0	197,143	1	8,064	4,643
10,654	1,853	2,143	0,066	0	60	4,5	8,064	5,289
23,571	6,209	5,207	0,428	0,789	214,286	1	0	0,789
4,915	1,13	0,352	1	1	265,714	4,5	0	4,643
5,394	2,433	1,141	0,428	0	342,857	0,789	180,012	0,143
2,994	0,998	0,132	0,786	1	197,143	0	0	4,566
5,421	2,642	0,998	1,551	0,429	360	4,5	33,7344	0,143
8,583	1,787	3	0,428	0	222,857	0	0	0,789
6,422	2,499	1,578	0,123	0,143	325,714	0,789	39,4464	0,932
9,855	0,561	6,561	0,551	0,066	145,714	1	14,3388	4,643

3,423	1,064	1,064	0,786	4,5	257,143	1	0	0,143
8,704	1,548	0,341	0,428	0,429	257,143	0	0	0,789
6,741	0,275	0,789	0,428	0	300	1	1,8396	0,143
4,917	2,433	2,275	0,428	4,5	274,286	0,143	0,8064	0,789
2,977	0,066	0,998	0,786	1	60	0	0	1,218
3,559	0,781	0,429	0,428	0	171,429	1	12,264	0
4,48	1,13	4,075	0,123	1	102,857	1	15,0612	0,932
6,506	2,565	11,075	0,428	1	197,143	1	15,1872	1,066
6,504	1,721	10,572	0,428	0	180	1	2,7972	1,143
6,069	1,71	1,921	0,856	1	180	1	24,1332	0
5,926	2,576	2,495	1,214	1,066	162,857	4,5	1,89	1,789
10,075	2,939	3,998	0,428	0	240	4,5	7,4592	5,289
2,898	1,141	4,141	0,066	4,5	420	4,5	0	4,643
5,917	1,71	2,143	2,5	4,5	471,429	1	51,31	0,066
10,731	5,575	0	0,428	0	617,143	1	15,3384	0
2,471	1,644	0,998	0,189	0,789	317,143	0,066	0	0,066
8,587	2,928	9,945	0,428	0,789	180	4,5	9,408	4,643
3,192	0,495	0,932	0,856	0	462,857	4,5	34,4736	0,143
7,152	1,864	3,222	1,428	0,066	420	4,5	1,8396	0,143
8,787	0,987	4,5	0,428	0	385,714	1	256,62	0,066
2,917	0,341	8,561	0,428	0,429	377,143	4,5	69,972	5,289
4,206	0,921	0,855	0,428	1	342,857	1	0,8064	1,143
4,851	1,284	6,352	0,428	0,143	600	4,5	0	6,143
6,065	0,921	12,352	1	4,5	137,143	1	12,264	5,289
2,986	6,287	5,721	0,123	0	377,143	1	0	6,789
5,203	1,141	0,132	0,428	1	180	4,5	30,9232	0,143
19,151	2,141	3,066	0,428	1	197,143	1	92,337	0,132
7,708	2,51	1,066	0,132	0,429	317,143	4,5	5,5944	0,143
7,304	2,645	1,286	0,428	0	480	0	719,936	1,789
7,853	0,998	1,284	1	1	257,143	1	26,9892	0,143
14,14	0,473	2,209	0,246	0	120	4,5	5,6028	1,578
12,794	4,995	0,921	0,428	0,429	162,857	0	0	0
6,197	5,784	3,51	0,551	0,143	180	4,5	1,344	0,143
5,416	1,141	1,713	1,214	0	197,143	1	17,6904	0,143
4,206	1,341	6,787	0,428	0,429	300	1	14,1036	0,143
14,151	0,704	3,286	1	0	514,286	1	5,5944	0,572
5,574	1,789	1,143	0,066	1	265,714	1	0,8064	0
5,919	1,71	0,932	0,428	0,789	428,571	4,5	21,1932	0,789
6,071	2,51	2,998	2,5	0	351,429	0	23,3772	0,572
4,34	0,858	0,998	1,214	0,143	137,143	0,066	10,0044	0,143
2,84	0,429	0,132	0,132	0	317,143	0,789	12,264	0,143
3,615	0,539	2,721	0,786	4,5	197,143	1	20,0088	0,066
8,134	1,262	1,418	1	0,066	137,143	0,789	0	1,789
2,983	1,196	10,075	0,856	0,066	377,143	6	25,5234	0,143
2,694	1,064	3,932	0,066	0	291,429	1	18,6592	0,066
7,926	5,204	6,781	1,572	4,5	282,857	1	7,476	4,643
7,346	1,776	1,275	1,428	0,789	51,4286	0	0	0,855
9,646	2,653	1,855	0,428	0	497,143	1	10,038	0,143

2,922	0,341	3,855	0,428	0	180	1	8,3412	1,789
4,994	1,71	4,998	0,428	0	240	1	22,4112	1,143
11,279	1,352	1,352	0,428	1	180	4,5	12,4684	5,289
6,944	1,572	3,143	0,066	0,789	180	1	2,7972	4,643
4,572	1,352	0,209	0,428	0,789	360	1	0	1,143
2,906	1,776	7,007	0,066	4,5	480	1	0	0,143
10,575	1,919	2,141	2,5	1	180	0,789	17,689	5,289
5,232	0,987	5,932	0,428	1	377,143	1	0	5,289
4,981	1,559	1,787	0,123	0	240	0,789	32,634	0,143
10,655	2,653	0,275	0,428	0	480	1	36,134	0,143
5,491	1,286	0,275	0,428	0,789	351,429	1	4,5276	4,643
5,429	1,919	3,15	0,856	4,5	411,429	1	5,376	1,143
6,278	2,853	6,361	1	4,5	300	4,5	0	1,578
7,214	2,207	0	0,428	0	180	1	0,2268	0,143
7,729	2,939	0,275	0,786	0,789	197,143	4,5	0,8064	0,789
5,869	1,198	2,132	0,123	0	145,714	1	9,2484	0,209
13,352	0,198	0,855	0,56	0	137,143	0,429	2,1504	0,143
12,642	0,132	2,066	0,066	1	85,7143	0	0	1,143
6,933	2,787	3,073	0,428	0	205,714	4,5	24,5112	0,789
4,129	5,641	2,352	0,856	0,066	137,143	0,789	0	0,932
3,893	1,207	4,064	0,428	0	240	0,789	12,5916	1,578
11,551	1,341	1,998	0,428	1	60	0,789	2,7972	0,143
6,152	0,198	1,132	0,428	0	197,143	1	3,4944	1,218
5,436	4,83	7,064	0,428	1	257,143	1	0	1,143
7,227	1,908	4,864	0,786	1	120	1	1,1844	0,789
4,144	1,141	4,141	1	1	300	0,789	265,4316	4,643
3,838	1,855	4,721	0	1	120	1	3,066	1,143
3,569	1,721	6,209	0,066	0	351,429	4,5	0	1,143
2,7	1,273	2,407	0,066	1	334,286	4,5	7,476	1,789
2,968	1,284	6,853	0,428	0,789	291,429	1	19,726	0,143
6,22	2,139	1,504	0,428	1	411,429	0	4,1412	0
4,778	0,693	0,418	0,551	0,789	180	1	3,1416	0,932
8,936	1,075	5,786	0,428	0	180	1	0	0,143
4,777	1,207	0,429	0,786	1	120	1	8,9292	0,143
2,175	0,627	1,996	2,214	4,5	180	1	5,376	0,572
5,917	1,13	1,787	1	1	222,857	1	38,64	0,789
7,204	3,141	5,209	0,123	0	222,857	0,789	9,324	4,643
6,583	3,299	1,998	0,428	1	454,286	4,5	0,8064	1,789
6,071	1,361	3,286	1,428	0	197,143	4,5	0,8064	0,066
3,616	1,064	2,855	0,123	1	162,857	1	4,1412	0,209
4,77	1,987	4,998	0,066	0	240	0,789	1,8396	4,643
4,28	0,921	2,209	0,123	4,5	445,714	1	5,4852	4,566
6,374	1,418	0,921	0,786	0	317,143	1	5,376	0,789
4,118	1,427	0,407	0,066	0,066	300	1	2,1504	6,143
4,803	2,708	2,998	0,428	0	317,143	1	0	4,566
2,678	1,286	3,71	0,786	0	197,143	1	6,9972	0,143
1,682	0,627	10,209	0,123	1	104,286	1	0	1,143
8,345	1,35	5,132	1	4,5	334,286	1	2,688	4,643

4,272	7,787	2,576	0	4,5	300	0	0,8064	6,789
3,919	2,719	4,209	0,428	1,143	257,143	1	18,648	1,143
3,851	1,572	2,209	0,066	1,789	334,286	1	0	0,143
3,537	1,064	1,075	0,428	4,5	154,286	1	11,1636	0,789
5,426	2,644	0,275	0,856	0,789	180	1	2,1504	0,572
9,735	1,13	3,864	0,428	4,5	77,1429	1	42,266	4,643
3,348	0,352	1,143	0,428	1	368,571	0	6,132	4,643
3,779	1,132	0,855	0,428	1	274,286	4,5	0	0,143
5,203	1,13	2,216	0,551	0	514,286	4,5	10,8948	0,066
10,228	6,507	3,853	1	4,5	257,143	1	0,8064	1,429
4,853	0,855	7,075	0,786	4,566	360	1	6,9468	1,789
9,575	1,207	2,721	0,428	1,578	77,1429	1	10,752	0,855
2,997	0,429	9,143	0,123	0	420	1	0	5,289
14,137	2,713	1,864	0,786	1	385,714	1	0	5,289
6,726	0,495	0,921	0,786	0	197,143	4,5	6,1824	0,789
0,99	0,924	8,132	0,551	4,5	265,714	1	30,8028	0,143
14,72	0,781	2,987	0,428	0,429	51,4286	1	0	1,578
4,926	1,559	1,644	0,786	0	402,857	4,5	14,8932	1,143
7,848	1,13	3,341	0,428	0	120	0	0	1,578
4,706	0,341	2,998	0,123	1	120	1	0	4,566
5,13	1,998	6,484	0,856	4,643	180	4,5	1,5498	4,643
17,345	1,787	5,644	0,856	1	77,1429	0	5,376	5,289
4,276	6,987	6,284	0,856	0,429	317,143	4,5	18,648	6,066
5,137	1,141	1,352	0,428	1	85,7143	0	122,2144	1,143
5,796	1,361	15,066	0,123	0	257,143	4,5	0	5,289
4,26	1,578	1,504	0,428	0	120	4,5	19,2024	1,143
2,186	1,787	1,132	0,066	4,5	480	1	4,8384	4,643
8,882	1,787	0,789	0,428	0	60	4,5	18,396	0,789
11,331	1,207	0,143	0,428	1	180	1	13,608	0
8,769	1,998	6,209	0,428	0	257,143	4,5	3,5532	1,789
6,926	1,561	0,352	0,428	0	317,143	1	1,512	0,143
5,792	1,427	0,855	0,123	1	240	4,5	27,972	0
11,571	2,13	2,418	0,786	0	111,429	4,5	6,5016	1,143
4,014	1,721	2,066	0,428	4,5	205,714	1	0	0,143
7,011	4,787	1,143	0,428	1	360	1	0,8064	0
7,361	2,93	5,275	1	0	180	4,5	0	1,066
4,558	1,864	7,209	0,428	4,5	257,143	1	3,4944	0,572
3,775	0,638	6,932	0,066	0	394,286	1	12,3732	5,289
12,006	2,862	3,585	0,786	0	248,571	4,5	7,35	0,789
8,003	1,996	1,418	0,494	0,429	240	0,789	5,6448	0,789
10,639	0,484	0,429	0	0	60	1	6,9972	0
10,564	1,644	1,721	0,428	1,066	240	4,5	8,5512	0,789
4,432	2,719	4,352	0,066	0	300	4,5	1,89	4,643
5,728	1,932	9,864	0	1	137,143	0	3,6792	4,643
6,154	4,786	4,209	0,428	0	102,857	4,5	8,064	5,289
4,633	1,286	3,141	0,786	0,429	325,714	0	0	0,209
7,726	0,932	1,075	0,428	0	240	0	0	0,143
5,437	0,998	2,209	0,428	1	385,714	4,5	2,7972	0,143

9,64	0,987	0,561	1,284	0,143	222,857	0,789	144,1244	0,143
4,473	1,998	0,855	0,428	0	317,143	1	6,1824	0,066
3,984	1,853	0,132	0,428	0	334,286	0,429	3,066	1,143
3,056	0,638	3,407	0,428	0	180	1	0	4,643
2,557	2,216	16,007	0,132	4,566	360	6	0	6,789
5,419	1,572	0,352	0,786	4,5	180	1	13,0704	1,143
5,706	5,63	6,638	0,428	0,429	180	4,5	23,058	0,932
4,511	2,367	3,721	0,123	4,5	308,571	0,066	8,82	0,143
2,252	1,075	4,852	0,428	4,5	265,714	1	5,5944	0,932
4,192	1,864	1,209	0,856	1	505,714	0,143	46,62	0,143
3,64	0,264	0,286	0,123	0	180	0	6,132	4,643
3,183	1,141	0,932	0,786	1,789	377,143	0	21,2562	0,572
3,346	0,264	2,77	0,066	1	325,714	0,789	0,2268	0,789
10,931	0,286	0,789	0,428	0,789	197,143	0	2,331	0,066
7,087	1,284	7,864	0,428	0	214,286	4,5	2,7972	0,143
4,201	1,776	7,933	0,066	0	300	1	7,7028	4,643
4,634	2,51	1,578	0,428	4,5	180	0,143	29,8116	5,289
6,581	1,482	0,396	0,428	1	480	1	13,9272	0,855
6,069	1,275	1,921	0,428	0	102,857	1	1,6128	0,143
7,003	1,921	5,787	0,428	0,143	240	0,789	46,3232	0,143
4,405	6,418	8,064	0,786	0,286	180	1	0,8064	6,143
3,995	0,781	0,932	0,856	0	351,429	0,429	15,9432	0,143
8,372	1,787	1,787	0,123	0	120	1	38,9312	0,932
4,421	1,284	2,51	0,246	1	300	4,5	0	0,143
7,062	1,919	0,132	0,428	0,789	257,143	1	14,9184	0,143
5,199	6,867	3,433	0,786	6,143	240	1	18,3456	1,143
8,013	0,781	1,141	0,428	6	737,143	0,789	0	0,789
5,935	2,93	3,721	0,856	0	145,714	1	61,8436	0,143
4,585	1,789	1,132	0,428	0	137,143	0	0,8064	0,066
9,142	2,361	6,787	0,428	0,789	180	4,5	6,5016	6,789
2,469	1,284	4,064	0,428	0	240	4,5	35,4872	0,789
7,568	1,429	0,921	0,428	4,5	231,429	1	0	4,643
6,143	1,855	1,053	0	1	228,571	0	17,1696	4,566
4,652	1,787	2,561	0,551	0	171,429	1	104,958	1,578
6,144	1,064	2,429	0,856	0	137,143	4,5	0,8064	1,578
6,511	0,858	1,143	0,428	0	334,286	1	7,5936	5,289
5,423	1,339	6,13	0,428	4,5	180	1	0	4,643
5,878	0,715	0,561	0,428	1	300	1	4,032	1,143
2,634	0,781	6,286	0,066	4,5	360	0	0	0,143
8,978	1,13	0,352	1,572	1	137,143	1	23,926	4,643
2,612	0,462	2,418	0,123	0,789	462,857	1	30,66	5,289
6,271	1,998	10,218	0,428	0	180	1	28,1988	0,789
4,215	1,284	5,075	0,428	1	317,143	1	0	0,143
3,073	0,855	2,286	0,123	0	180	1	1,6128	4,643
5,627	1,921	1,418	0,123	0	162,857	4,5	8,064	1,143
5,424	1,262	2,998	0,428	0	180	1	18,6004	1,578
5,718	2,51	2,15	0,856	0,789	368,571	0	2,1504	0,143
6,718	2,007	1,721	0,674	0,789	240	0	16,6824	1,789

7,912	2,367	1,198	0	5,289	282,857	1	5,4684	0,143
4,766	5,564	3,847	1,214	0,066	214,286	1	18,837	1,066
3,766	1,284	0,264	0,123	0,143	308,571	0	0	4,643
4,346	0,855	1,209	0,066	0	180	1	6,7452	1,066
5,784	1,416	3,572	0,428	0	240	0,143	21,4452	1,143
6,942	1,207	1,284	0,428	0,789	197,143	1	6,9972	1,143
5,784	1,842	3,341	1	0,789	377,143	4,5	26,4936	0,789
4,131	1,341	0,858	0,123	0	180	0,789	19,4544	6,143
4,572	1,209	0,066	0,123	0	60	0	6,1824	4,5
5,412	1,361	1,207	0,428	1	154,286	4,5	177,9652	1,789
5,284	3,433	3,141	0,786	1	197,143	1	0	0,143
10,006	1,996	1,352	0,123	1	180	1	0,2268	0,209
14,293	1,57	3,007	0,428	4,5	33,5714	1	0	1,218
10,358	4,011	7,789	0,123	4,5	60	4,5	5,376	4,566
10,575	2,073	3,71	0,428	4,5	274,286	0,066	32,8552	4,643
2,139	1,218	3,198	0,246	0,429	222,857	0,789	18,396	1,578
5,212	1,71	4,998	0,428	1	240	0	0	0,789
4,626	4,709	1,352	0,856	0	120	1	34,4736	0,143
9,021	1,57	0,286	0,428	4,5	300	1	0	5,289
4,775	5,421	5,853	0,786	0,789	454,286	1	22,5204	4,643
6,429	1,853	1,264	0,066	0	60	1	6,8292	0,932
7,925	1,141	2,218	0,428	4,5	205,714	0	14,2464	1,789
7,284	4,852	3,921	0	1	68,5714	1	19,1436	1,789
3,852	0,921	10,352	0,123	0,789	334,286	4,5	13,44	5,289
1,977	1,341	6,141	0,066	4,566	334,286	0	1,911	4,643
5,869	1,864	2,578	0,428	0	368,571	1	30,66	0,789
7,361	0,998	0,855	0,123	0	60	0	2,7972	0,066
5,496	1,284	3,064	0,428	0,143	325,714	4,5	2,7972	4,643
11,015	1,418	2,855	0,428	4,5	120	1	26,39	5,289
6,781	5,564	1,495	0,428	1	257,143	0,429	19,3452	4,643
2,348	2,207	2,427	0,428	0	480	1	5,376	0,789
17,769	5,707	2,075	0,428	0	120	4,5	2,1504	0,066
6,141	1,93	4,075	0,428	0,495	137,143	4,5	10,668	4,643
4,429	6,144	5,921	1	0	317,143	1	20,4876	4,643
9,565	2,787	7,141	0,909	4,5	317,143	1	10,1472	5,289
5,79	1,284	4,998	0,551	4,5	411,429	4,5	7,5936	6,789
4,434	1,35	8,075	0,786	4,5	445,714	4,5	0,8064	0,932
12,166	1,284	2,93	0,428	0	351,429	1	30,002	1,578
4,435	1,721	7,284	0,123	1	197,143	1	2,7972	0,143
4,204	1,35	3,13	0,856	0	180	4,5	38,2284	0,789
4,772	5,696	6,781	0,428	0	454,286	0	21,5656	5,289
3,834	0,418	1,198	0,066	0,789	120	1	0	0,143
4,214	2,141	1,275	0,428	0	308,571	4,5	17,0268	4,643
8,136	1,493	7,209	0,428	1	351,429	1	37,3044	1,143
2,568	1,141	5,218	0,428	4,5	300	1	11,1636	4,643
6,469	1,504	4,427	0,066	0	420	1	6,132	4,643
5,432	1,286	3,209	0,123	0	231,429	4,5	2,1504	4,643
3,926	0,572	0,998	0,066	0	600	1	7,9716	0,143

3,709	3,156	1,075	0,428	4,5	162,857	0	33,1072	5,289
2,823	2,499	4,572	0,786	1,066	377,143	0	0,2268	0,932
6,002	1,284	5,064	0,856	4,5	377,143	1	3,4944	1,789
4,861	2,073	1,209	0,428	0	300	1	37,296	0,143
8,359	0,209	1,075	0,856	1,143	180	1	1,8396	1,143
4,071	2,075	0,143	0,428	1	600	1	120,008	0,789
5,35	0,407	6,141	0,428	0,855	351,429	1	20,2356	0,932
16,365	2,853	0,066	0,856	1	205,714	0	0	0,066
2,34	1,143	0	0,066	0	214,286	1	8,064	4,643
3,86	1,996	4,075	0	4,566	248,571	0	0	1,789
5,062	1,427	0,572	0,428	1	377,143	4,5	38,066	0,143
4,858	1,35	0,855	0,123	1,066	557,143	0	17,0604	0,789
8,075	2,653	6,998	0,786	0	265,714	4,5	2,688	0,789
4,711	2,644	0,572	0,428	0	394,286	0	6,5604	0,789
4,206	1,776	5,066	0,428	1	300	4,5	0	0,143
3,054	1,998	1,143	0,428	1	60	0	5,376	1,143
3,849	1,71	7,143	0,428	0	197,143	1	30,1224	4,643
5,206	1,064	1,787	1	0	257,143	1	14,3472	0,932
4,712	1,064	1,284	0,428	0,066	94,2857	0,789	12,264	4,643
8,769	1,064	2,787	0,856	0,066	300	0,789	133,5096	0
5,088	2,576	8,653	0,428	5,289	137,143	4,5	2,688	5,289
5,351	1,985	0,932	1	1	180	0	30,002	0,066
6,152	1,35	3,653	0,123	1	214,286	4,5	0	1,143
3,282	1,864	4,286	2,928	0	565,714	1	6,9888	5,289
5,357	2,644	3,932	0,428	4,5	317,143	1	0	1,789
4,423	2,139	7,209	0,123	0	120	4,5	18,6508	1,578
5,48	2,642	2,066	0,428	1	334,286	0,789	65,3072	0,143
4,777	1,644	0,921	0,123	0,789	240	1	5,0232	0,855
8,73	3,156	1,93	0,428	0,429	334,286	1	2,688	0,789
8,583	0,066	2,932	2,5	0,789	154,286	4,5	36,0024	0,143
6,85	1,787	0,921	0,123	0	162,857	1	12,264	0,143
6,049	1,341	4,998	1	1	265,714	0,789	0	0,286
6,295	2,51	2,864	0,123	0,143	120	1	8,064	5,289
4,478	0,998	0,341	0,428	1	531,429	1	9,324	0,066
5,504	2,073	2,207	0,428	0	137,143	1	0	0,789
5,058	1,143	0,209	0,066	0	317,143	4,5	18,5556	4,643
2,339	2,576	0,132	0,428	0	377,143	4,5	8,442	5,289
7,574	0,855	1,644	1	0	300	1	12,1212	0,066
4,983	1,141	7,286	0,428	0	180	1	12,1212	0,572
1,845	0,341	2,51	0,428	1	240	0	0	4,566
1,889	0,418	0,789	0,428	0	240	4,5	4,5276	0,855
7,348	2,207	2,341	0,856	4,5	300	1	0,8064	1,578
7,005	2,576	3,075	0,428	0,789	240	4,5	15,1564	0,143
6,792	1,57	6,499	0,428	6	445,714	1	11,655	0,789
3,052	0,998	6,198	0,066	0	334,286	4,5	0	4,643
9,537	3,156	2,721	0,428	0,789	240	0,789	18,396	1,578
4,186	2,71	4,853	0,428	1	214,286	4,5	6,2034	0,789
7,086	1,998	1,789	0,123	4,643	154,286	4,5	2,688	1

6,209	1,919	2,367	0,428	1	300	4,5	19,2024	0
4,574	5,564	1,209	0,786	1	274,286	0,789	0	0,143
12,158	2,796	3,853	0,428	1	480	1	3,4944	1,218
5,994	2,216	6,299	0,428	1	360	4,5	18,396	1,578
10,134	3,853	2,719	1	0,429	120	4,5	2,7972	1,143
5,574	1,789	3,932	0,428	4,5	334,286	4,5	0	4,643
2,997	1,075	0,352	0,428	4,5	137,143	4,5	5,754	0,143
6,218	1,776	0,352	0,123	1	282,857	0,789	5,6448	1,789
9,144	2,433	3,939	0,428	0,143	77,1429	4,5	6,9888	0,143
5,869	1,141	1,13	0,428	1	222,857	1	25,6452	0,143
4,784	1,207	5,627	0,428	4,5	411,429	0	3,4944	6,789
4,622	6,144	6,073	1,572	0	257,143	1	13,0872	6,066
5,294	3,442	3,218	0,856	0,143	300	0,789	7,1274	1,218
8,505	0,855	0,143	0,786	4,5	180	4,5	0	0
4,491	0,921	1,998	0,428	4,5	360	0	4,032	4,643
3,139	6,276	4,352	0,551	4,5	334,286	1	27,972	4,643
7,649	2,007	6,284	0,428	1	282,857	4,5	32,7992	0,143
7,707	1,996	13,066	1	0	360	0	0	4,5
4,761	1,998	0,275	0,189	0,789	514,286	0	0,8064	0,932
6,504	2,708	1,627	0,428	0	137,143	1	6,132	4,643
6,352	1,207	1,644	0,428	1	240	0	0	1,789
10,006	1,721	1,998	0,428	1	120	1	2,7972	6
8,641	0,855	0,789	0,428	0,066	137,143	4,5	3,0744	0
5,563	1,853	0,352	0,428	0	334,286	1	167,972	5,289
3,051	2,205	7,57	0,066	1	454,286	4,5	17,1192	4,566
7,873	1,787	3,429	0,428	1	325,714	4,5	48,0032	0,932
6,641	1,93	2,218	0,428	0,143	317,143	1	4,032	1,143
6,146	1,55	4,561	0,428	1	137,143	1	17,8584	4,643
8,356	0,429	12,407	0	1	171,429	4,5	0	1,143
4,069	1,427	10,499	0,123	0	325,714	4,5	0	6,789
4,06	1,721	1,275	0,123	1	402,857	4,5	18,9252	4,643
5,801	2,51	5,284	0,428	0	214,286	1	18,648	4,643
4,071	0,495	1,275	0,856	0	274,286	1	0	4,643
9,167	1,644	0,855	0,786	0	308,571	0,789	4,9056	0,143
6,873	1,864	2,721	0,428	4,5	120	0	96,306	5,289
8,416	2,787	10,209	0,786	1	274,286	0,789	10,9704	0,932
6,566	0,998	2,341	2,5	1	120	4,5	36,3972	4,643
5,638	1,207	6,139	0	4,643	445,714	4,5	5,376	4,643
6,941	0,352	0,418	1	0	214,286	0,429	18,1944	0,286
6,57	0,913	0,855	1	0	214,286	1	37,296	0
3,777	0,998	0,998	0,856	4,5	360	1	21,8316	0,789
7,363	1,056	5,855	0,066	1,143	351,429	1	0	1,143
4,707	1,71	1,715	0,856	0	180	0	2,7972	0,066
3,918	0,638	12,352	0,428	6	342,857	4,5	13,986	0,143
3,502	1,855	0,921	0,428	4,5	300	0	7,5264	0,789
3,486	0,209	4,275	0,428	0	291,429	1	22,2684	5,289
9,21	1,789	0,066	0,428	4,5	360	1	5,376	1,789
5,5	2,216	1,075	0,428	0	394,286	0	10,2816	1,789

5,127	2,776	4,921	0,428	6	197,143	0,143	8,9292	1,143
7,66	2,71	0,143	0,786	4,5	300	4,5	24,325	0,143
5,924	2,644	4,775	0,066	0	180	0	0	0,789
5,592	1,638	1,341	0,428	1	197,143	1	25,3932	4,643
12,52	3,288	0,932	0,428	4,5	180	0,066	0	0,789
3,992	1,075	9,644	0,428	4,5	334,286	1	7,2156	1,789
9,44	0,495	3,075	0,428	1	300	4,5	2,7972	0,286
3,918	2,653	4,284	0,428	0	240	1	13,608	6,789
4,489	1,493	4,284	1	0	231,429	1	20,6052	6,066
4,071	1,996	0,561	0,428	4,5	137,143	1	0	4,643
10,272	5,498	8,221	0,856	0	197,143	0,789	7,3584	0,789
5,761	2,785	6,572	0,428	0,789	222,857	1	3,4944	6,789
6,572	1,578	4,429	0,123	0	240	0	0	0,143
7,694	1,853	0,855	0,428	0	300	1	0,8064	0,855
4,289	1,493	2,721	0,786	0	240	0,789	8,1732	1,143
6,837	2,264	0,209	1	0,789	42,8571	0	14,9184	1,143
7,148	1,93	2,51	0,428	1	162,857	1	6,9972	0,789
2,208	0,407	3,787	0,066	1	420	1	0	0,286
7,495	0,638	0,275	0,428	0	197,143	4,5	13,5912	0,066
7,27	0,561	6,066	2,5	0	308,571	1	0	4,643
7,337	2,576	0,209	0	0	265,714	4,5	1,6128	0,286
8,134	1,987	1,561	0,786	1	437,143	1	9,7062	0,209
6,069	1,93	3,13	0,428	0	180	1	26,7232	0,143
5,7	4,918	2,495	1,214	1	505,714	4,5	28,6524	4,643
3,772	1,842	0,066	0,551	0	300	1	6,1824	1,578
6,004	2,576	3,275	0,428	0	180	0	11,0376	0,572
8,238	6,496	0,407	0,551	1	180	1	6,1824	0,066
5,553	1,853	0,715	0,428	0,066	411,429	0	90,8124	0,789
3,823	1,053	1,064	0,856	0	522,857	0	2,688	0,143
9,998	5,355	4,132	0,428	1	300	1	77,6132	6,143
2,359	0,495	4,143	0,428	0	257,143	1	37,296	0,789
5,282	1,855	9,075	0,428	4,5	351,429	0	0	5,289
9,093	0,638	7,853	0,428	0,066	180	1	0	5,289
6,713	2,216	5,721	0,428	4,5	120	4,5	75,5664	1,578
11,996	1,064	0,987	0,428	0	480	4,5	2,7972	0,789
14,804	1,075	1,286	0,428	1	120	4,5	18,396	0,572
6,298	1,996	3,207	0,428	1,143	282,857	0,429	10,038	0,932
4,635	1,418	0,987	0,428	0	531,429	0,429	21,5796	0,789
5,493	0,55	1,361	0,428	1,143	462,857	1	23,3142	1,066
3,919	4,709	1,143	0,066	0	394,286	4,5	0	1,143
5,052	2,062	7,209	0,428	0	300	1	4,662	6,143
7,991	1,207	0,418	0	0	137,143	0,143	16,3212	4,643
3,994	1,787	8,632	0,786	0	137,143	4,5	6,048	0,572
4,5	1,776	1,075	0,786	4,5	180	1	0	0,143
2,111	1,495	3,141	0,428	0	180	4,5	13,5828	0
6,943	1,787	0	0,428	0	360	1	18,396	0,789
5,441	2,785	3,156	0,428	0	300	1	11,6508	4,643
7,163	1,93	4,578	1	1	145,714	0	6,9972	0,143

6,869	2,15	3,71	0,428	1	317,143	1	180,012	1,143
11,711	0,627	1,93	0,428	0	137,143	1	0	1,066
14,787	1,919	0,275	0,786	1	180	0	3,4944	1,578
1,691	2,51	1,429	0,428	6	137,143	4,5	12,8352	1,789
7,735	2,073	6,987	0,428	0	154,286	0,789	4,662	1,143
5,704	1,286	0,066	0	1	334,286	0	0	6,143
6,853	1,418	0,209	0,428	0	205,714	1	6,132	0,066
5,797	2,919	2,921	0,428	1	480	4,5	5,5944	0,143
4,26	2,073	5,218	0,123	0	300	4,5	7,5264	1,143
7,584	1,856	0,066	0,428	0	180	4,5	0,8064	0,789
3,329	0,693	2,921	0,428	0	60	1	23,9302	0,066
7,207	1,209	0,932	0,428	1	360	1	2,688	0,132
5,207	0,704	0,198	0,428	0	257,143	4,5	6,9972	0,789
6,065	2,51	2,932	0,428	0,429	265,714	1	28,7112	0,143
1,988	0,913	7,284	0,066	1	394,286	4,5	0	5,289
6,845	2,064	3,367	0,428	0	120	1	2,268	5,289
7,155	1,053	5,209	0,856	0,143	222,857	4,5	10,9704	1,789
4,649	1,207	0,352	0,428	0	240	1	4,662	0,143
7,525	2,565	0,209	0,123	0	102,857	1	8,064	0,789
8,306	1,93	6,789	0,617	1	480	4,5	9,0552	0,789
9,003	1,33	1,075	0,428	0	154,286	0	0,8064	0,789
6,208	1,999	5,638	0,123	1	360	4,5	6,132	5,289
6,23	1,985	4,361	0,123	0	154,286	1	4,7166	0,932
5,777	0,704	12,275	0,123	0	257,143	1	1,8396	4,643
4,344	1,647	0,921	0,066	0	257,143	4,5	3,1836	0,789
5,349	1,141	1,284	0,428	0	137,143	1	19,2612	4,643
6,28	3,431	13,63	0,786	1	180	4,5	16,926	5,289
6,652	1,787	1,141	0,428	0	420	1	4,8384	5,289
3,412	5,641	0,066	0,123	6	385,714	0	32,8552	4,566
13,432	1,702	3,921	1,786	0,789	480	1	1,8396	5,289
5,006	1,721	12,998	0,786	0	411,429	4,5	16,1896	5,289
2,546	1,064	13,429	1	0,066	180	1	16,6824	1,789
11,138	2,708	7,053	1,786	0	120	4,5	24,528	5,289
9,716	2,576	3,075	0,786	0	222,857	1	1,8396	6,143
0,627	1,644	6,143	0,428	0	120	0	23,7762	4,643
10,153	1,644	0	0,428	1	240	0	13,5492	0,789
4,575	0,913	1,361	0,123	0	180	4,5	49,4172	0,789
4,722	2,13	4,064	0,428	4,5	60	1	0,8988	6,789
9,312	1,361	2,361	0,428	0	214,286	0	27,972	5,289
4,849	1,066	2,855	0,066	4,5	257,143	4,5	0	0,066
1,661	1,416	1,198	0,066	4,5	574,286	4,5	7,2156	5,289
5,632	4,852	4,578	1	0	342,857	0	0,8064	4,5
4,937	1,218	3,143	0,428	1	540	1	31,4762	0,066
10,943	1,504	2,638	0,428	1	120	1	18,648	1,143
4,915	2,15	1,495	0,856	0	360	4,5	9,5172	0,932
1,908	1,075	2,209	0,428	0,789	257,143	4,5	22,2684	0,143
4,19	0,638	0,921	0,123	0	565,714	4,5	13,7172	0,789
3,994	0,921	4,721	0,246	0	351,429	4,5	0,8064	1,143

5,783	1,13	3,35	0,786	1	94,2857	0	18,0012	1,789
4,429	2,653	7,407	0,428	0	197,143	4,5	3,4944	4,643
13,774	5,432	2,066	0,551	0	342,857	1	5,4852	1,789
6,865	3,367	6,864	0,123	0	334,286	4,5	0	5,289
3,335	1,053	5,416	0,066	0	180	0,789	5,6028	0,143
4,871	0,638	1,143	0,428	0	240	4,5	30,0972	0,143
5,863	1,341	1,218	0,428	4,5	394,286	1	5,376	0,789
4,992	0,836	5,787	0,786	0	257,143	1	41,0592	0,932
4,851	1,704	4,561	0,123	1	240	0,789	0	1,789
9,259	0,55	4,209	2,5	0,066	120	1	4,4898	0,932
4,498	0,407	0,998	0,551	0	274,286	4,5	26,7372	0,932
3,635	0,704	2,361	1,214	0	162,857	4,5	7,9716	1,143
8,306	0,352	1,132	0,428	1	360	4,5	2,1504	0,855
5,185	2,007	0,132	0,428	0	480	1	0,8064	0
4,574	1,787	0,789	0,428	0	471,429	1	10,752	1,578
2,12	0,781	7,13	0,246	0	308,571	1	30,66	0,143
2,271	0,932	0,143	0,428	4,5	274,286	4,5	49,4452	0
3,713	3,288	6,275	0,428	0,789	180	1	8,1732	0,932
4,352	0,704	2,484	0,428	0	137,143	4,5	12,726	1,143
3,115	1,721	3,721	0,428	0,789	480	4,5	15,5904	4,643
7,051	1,064	3,198	0,123	1,066	137,143	1	0	0,932
7,007	1,787	0,855	0,428	0	222,857	1	18,396	1,143
9,376	4,907	2,429	0,428	0	394,286	4,5	2,688	5,289
11,729	1,93	0,275	0,123	1	514,286	1	3,6792	0,143
10,008	1,141	6,352	0,066	4,5	214,286	0	0	6,143
6,994	3,855	3,143	1,123	4,643	300	0	3,4944	6,789
1,614	0,781	6,352	0,123	0	420	4,5	1,344	6,789
4,368	1,064	5,141	0,428	0	154,286	0	2,7972	1,143
3,254	1,13	2,264	0,123	0,209	111,429	1	0	0,143
3,992	0,998	16,638	0,428	0	257,143	4,5	2,1504	5,289
2,779	2,433	1,789	0,066	0	480	0	0	6,789
4,212	1,141	0,77	0,856	0,429	180	0,143	9,324	0,209
3,987	6,353	5,864	0,786	0	257,143	4,5	16,926	5,289
4,054	0,987	3,644	0,428	0	360	0	9,5172	0,932
6,086	0,998	3,932	0,123	0	120	4,5	7,4592	4,643
16,562	2,642	10,286	0,786	4,5	77,1429	1	37,9176	4,643
5,06	1,853	8,341	0,428	0,789	120	1	7,9716	6,789
3,766	0,275	5,066	0,123	1	154,286	1	0	6,143
5,638	1,853	1,853	0,428	0	231,429	1	26,3676	1,143
3,557	0,341	0,998	0,123	0	282,857	1	6,132	0,209
4,64	1,218	6,13	0,066	0,066	514,286	0	6,132	4,643
5,797	1,842	8,143	0,428	4,5	308,571	4,5	6,5604	1,578
3,502	1,352	1,57	0,123	4,5	351,429	0	0	0,143
4,22	2,15	4,998	0,066	0	257,143	1	0	0,789
3,5	0,132	0,209	0,123	0	120	1	5,376	6,143
8,238	1,787	1,418	0,428	0	240	1	6,9972	0,143
4,051	1,932	1,578	0,123	1	162,857	1	3,4944	6,143
7,372	1,284	3,066	0,428	1	137,143	1	0	4,643

9,666	2,93	0,352	0,428	0	222,857	1	6,1824	0
3,557	2,787	0,789	0,786	1	651,429	4,5	30,8868	0,789
4,137	0,932	1,286	0,066	1	145,714	0	0	6,143
3,28	1,93	3,932	0,428	4,5	342,857	1	18,648	6,143
6,306	2,361	7,572	0,786	0	317,143	1	0	5,289
4,775	0,638	1,495	0,856	0	411,429	1	13,7172	0,855
6,945	1,693	1,418	1,428	0	257,143	4,5	20,5912	0,143
4,014	1,218	4,264	0,786	0	617,143	4,5	10,2564	0
3,555	1,339	4,066	0,856	4,5	154,286	1	12,3144	4,643
2,601	2,719	1,141	0,428	0	257,143	0,789	11,2476	0,932
7,664	0,781	1,064	0,428	0	257,143	0	7,9716	4,643
6,064	2,787	2,998	0,786	0,789	334,286	0	30,002	1,789
9,231	2,51	2,367	1	1	291,429	1	12,1212	1,143
5,634	1,842	2,286	0,428	0,789	377,143	1	46,648	0,143
5,709	1,207	0,921	0,856	0	514,286	1	10,2648	0,143
6,143	1,209	7,13	1,214	0	60	0,143	2,688	6,143
6,352	1,919	3,284	0,428	0	111,429	1	7,2156	0,143
4,344	1,57	8,207	0,786	0	214,286	1	1,6128	5,289
4,549	2,939	1,644	0,428	1	240	4,5	30,002	1,578
6,275	1,407	3,209	0,123	1	240	4,5	24,1164	0,143
3,7	2,787	9,064	0,856	1,143	257,143	4,5	0	6,143
2,84	1,284	0,132	0,428	1	411,429	1	75,2836	4,566
5,201	1,93	2,71	0,856	0,143	197,143	1	14,6244	1,578
5,374	1,064	1,429	0,428	0	205,714	1	1,8396	1,578
4,201	1,075	4,495	0,856	0	162,857	1	29,6562	0,932
4,928	0,932	1,644	0,428	0,789	51,4286	1	23,9904	4,566
5,634	3,51	3,855	0,123	1	600	0	2,7972	4,643
2,425	1,787	0,932	0,428	0	480	0	0	5,289
3,338	3,653	1,132	0,066	0	420	4,5	5,376	0,143
5,638	1,286	4,209	0,123	1,066	180	0	5,376	4,643
4,577	2,15	0,921	0,123	0	300	1	26,1212	0,143
5,267	3,288	2,407	0,856	0,789	540	4,5	36,3132	0,143
3,269	0,998	0,209	0	1	360	0	0	4,566
4,503	1,853	3,998	0,428	4,5	300	1	0,8064	5,289
11,125	0,627	0,495	0,428	0,789	137,143	1	6,132	1,578
6,296	1,141	7,71	0,428	0	531,429	1	0	4,5
5,421	2,062	6,638	0,428	0	180	0	42,266	0,143
4,706	0,209	0	0,066	1	180	1	5,6028	0,143
5,78	1,644	0,286	0,123	0	120	4,5	16,9932	0,789
4,061	1,064	4,721	1,214	0,789	282,857	1	18,3456	0,066
7,513	5,575	2,418	0,123	0	180	1	39,6592	5,289
8,311	0,704	3,064	0,123	0	171,429	0	2,7972	1,143
6,209	1,71	1,855	0,551	0	282,857	4,5	28,7532	0,066
5,575	0,407	2,627	1,066	0,789	411,429	4,5	14,4144	0,789
2,26	0,561	15,286	0,428	4,5	300	0	0	5,289
7,005	2,708	7,787	0,428	0	180	4,5	3,4944	0,143
3,992	1,427	3,721	0,428	1	428,571	0	18,396	0,932
2,392	1,647	3,132	0,123	0,789	240	1	10,752	5,289

4,115	1,559	4,787	0,066	1	360	1	0	1,143
8,365	1,273	8,855	0,189	0	454,286	4,5	13,0032	4,643
6,715	1,196	0,341	0,856	0,066	180	1	5,376	4,643
10,791	1,921	6,156	0,551	0	377,143	4,5	14,9184	0,932
2,565	0,987	1,921	0	0,855	394,286	0,789	12,264	0,066
3,651	0,998	0,275	0,786	0	300	4,5	13,9944	6,789
7,721	2,359	3,721	0,428	0,789	120	0	0,2268	5,289
5,21	1,427	3,493	0,856	0,789	300	4,5	19,3032	0,932
6,157	0,638	5,209	0,428	4,5	102,857	1	1,8396	0,572
12,217	1,143	3,075	0,428	0,789	214,286	0,789	12,3732	1,578
7,082	1,561	5,284	0,856	0	240	4,5	1,533	4,643
6,736	2,139	11,284	0,428	0,143	257,143	1	12,8352	0,932
12,791	0,932	1,207	0,246	0	197,143	0	7,3752	0,143
8,78	1,141	7,715	0,786	4,5	120	4,5	13,9944	6,143
2,546	1,853	0,998	0,428	0,143	420	0,066	0	1,143
1,691	0,932	2,209	0,066	0	480	1	13,818	4,643
11,691	1,853	1,644	0,066	1	240	0,143	6,9972	0,143
12,999	2,205	8,51	0,428	4,5	308,571	0,789	21,1932	1,578
15,58	2,075	8,355	0,428	0,932	240	1	46,13	0,932
2,483	0,627	14,499	0,428	0,789	317,143	0,066	18,648	6,789
3,338	1,864	2,073	0,856	0	540	4,5	23,058	0,143
9,08	2,719	7,361	1	4,5	180	4,5	39,634	1,143
7,956	4,154	2,367	0,428	0	240	4,5	0	0,789
3,126	1,919	4,721	0,066	0	282,857	1	0	5,289
5,568	2,196	3,418	0,123	0	420	4,5	7,2156	4,643
5,854	2,064	1,407	0,979	1	188,571	1	20,5464	0,143
5,13	1,71	0,264	0,856	0,066	480	0,789	10,668	0
6,77	2,064	5,361	0,856	1	180	1	23,3016	5,289
5,428	1,998	1,064	0,428	0,429	351,429	1	21,9156	1,066
5,137	0	0,209	0,123	1	497,143	0	1,8396	4,566
5,946	1,988	6,495	0,428	6	360	4,5	0	0,789
8,854	0,781	0,932	0,123	1	600	4,5	0	0,143
2,812	2,064	2,352	1	0	265,714	4,5	294,0196	0,855
5,561	2,996	1,853	0,189	1	197,143	1	5,4516	0,932
3,137	1,644	2,198	0,123	1	480	1	2,688	4,643
5,988	2,073	5,141	0,428	1	240	1	5,1282	4,643
5,641	1,416	2,13	0,428	0,855	317,143	4,5	30,31	5,289
4,856	0,418	2,352	0,428	1	377,143	4,5	46,62	4,643
3,697	1,284	6,352	0,066	1	180	4,5	78,0052	1,143
5,785	0,066	1,275	0,428	0	351,429	1	0	0,066
5,299	1,066	2,789	0	0,789	180	1	0	5,289
7,224	1,787	1,858	0,428	1	222,857	1	0	0,572
2,911	1,218	4,418	0,428	0,932	180	1	17,8584	0,855
3,917	1,776	0,209	0,123	0	282,857	0,066	0,756	6,143
3,338	1,713	6,367	0,856	0,143	154,286	1	18,396	0,143
2,719	2,721	0,429	0,428	1	300	4,5	0	5,289
6,926	1,064	6,721	0,428	0	274,286	0	13,4652	5,289
6,231	1,932	1,218	2	4,5	154,286	0	0	1,143

7,726	1,996	2,932	0,551	0	120	1	4,8384	0,066
4,85	1,196	1,064	0,123	0	342,857	4,5	0,4536	0,143
3,26	0,781	2,433	0,786	0,789	120	1	30,7692	0,066
11,009	0,998	0,855	0	0	257,143	0	0	1,578
6,93	1,721	3,572	1	0	137,143	4,5	5,376	0,572
6,493	1,207	1,064	0,132	0	240	1	5,6448	1,789
5,119	1,493	1,578	0,852	0,429	411,429	4,5	33,5804	0
2,471	4,5	1,141	0,066	1	300	4,5	0	0
6,59	1,864	3,855	0,123	1	300	1	0	5,289
11,14	1,919	5,998	0,428	0,143	60	4,5	12,9528	4,643
4,994	1,361	3,495	0,123	1	240	1	1,4784	1,789
2,623	1,93	4,275	0,123	0,066	300	4,5	11,13	1,143
5,425	4,841	4,998	0,123	0	180	1	2,1	0,143
4,86	1,855	6,855	1	1	60	1	33,4964	0,932
4,348	2,644	1,787	0,428	0,789	240	0	15,33	1,218
9,576	1,075	1,418	0,428	0	154,286	1	12,264	4,643
19,939	2,578	0,987	0,123	1	274,286	1	3,36	0,066
5,726	0,998	0,132	0,428	1	205,714	0,789	0	0,066
8,157	1,787	4,361	0,123	4,5	480	1	0	0,789
7,772	1,57	4,209	0,428	1,143	257,143	1	18,396	4,643
6,728	1,427	0,429	0,428	0	411,429	1	6,132	0,143
5,64	1,71	0,855	0,428	0	582,857	4,5	3,4944	0,789
5,08	1,273	6,565	0,428	4,566	240	1	6,5688	1,143
4,615	2,282	5,776	0,428	1	15	0	0	1,789
8,531	2,433	2,721	0,428	4,5	240	4,5	14,4592	4,643
5,937	1,284	0,132	0,428	0	334,286	1	7,4592	0,789
5,706	2,565	7,352	0,428	0,066	385,714	1	79,8294	5,289
4,137	1,996	5,57	1	0	308,571	0	11,8776	0,143
4,201	0,924	2,293	0,123	0	300	1	17,3124	0,143
4,352	1,721	1,209	0,428	0	360	4,5	2,688	6,789
5,493	2,073	4,075	0,428	0,789	222,857	4,5	28,7112	5,289
4,434	1,919	3,209	0,123	0	171,429	1	6,72	1,789
1,759	0,275	0,198	0,428	0	385,714	4,5	142,2764	0,143
4,424	1,71	1,209	0,123	0	51,4286	1	0	1,578
3,775	2,708	0	0,551	0	197,143	1	31,4804	0,495
4,19	2,062	5,273	0,786	0,066	154,286	1	38,066	1,218
2,689	1,284	2,139	0,123	1	428,571	0,789	0	4,643
10,208	5,355	3,286	0,428	4,5	180	1	14,4592	5,289
5,994	1,644	4,998	0,428	6,143	514,286	0	0	0,143
3,357	2,205	3,932	0,123	0	420	4,5	3,1836	0,789
4,194	0,198	3,921	0,066	0	300	4,5	9,324	1,578
6,348	1,273	11,264	0,428	0	120	1	0	0,572
6,203	2,576	6,352	1,856	1	102,857	4,5	0	0,789
5,414	1,919	7,561	0,428	0,429	240	0,789	16,1952	0,143
5,558	0,561	4,207	0,786	1	120	1	69,972	5,289
4,71	2,075	0,998	0,123	0	462,857	1	6,1824	0,066
7,006	2,787	0,495	0,786	4,5	274,286	1	0,8064	0,789
5,061	1,053	5,141	0,428	0	300	0,789	23,7832	1,143

5,783	0,921	0,132	1,123	0,789	342,857	0,143	10,3992	0,143
2,857	0,132	4,207	0	0,789	51,4286	0	28,7924	0,855
4,624	1,776	1,932	0,786	0,789	402,857	1	8,3412	0,855
11,911	1,996	1,998	0,428	0	180	4,5	51,716	0,066
2,832	1,93	3,064	0,066	1	360	0	0	4,643
5,625	2,216	9,864	0,786	0	120	1	0	5,289
9,732	3,275	2,638	0,428	1	120	1	7,5264	0,789
2,898	0,561	10,787	0,786	0	317,143	0,143	12,1212	4,566
6,487	1,93	1,141	0,856	0	257,143	1	0	0,143
3,546	2,275	2	0,428	0	514,286	0,789	9,324	0,066
2,474	5,498	11,21	0,551	1	480	0	0,8064	5,289
4,634	3,286	1,066	0,428	0	60	1	30,058	4,643
9,381	1,853	0,352	0,428	0	154,286	4,5	3,6792	0,132
3,186	1,264	3,367	0,786	0	137,143	1	32,7992	1,789
8,639	0,341	15,507	0,428	0	137,143	1	0	5,289
3,131	1,361	2,284	0,428	0	180	1	6,9972	5,289
5,135	1,141	3,721	0,428	0	257,143	4,5	0	4,643
5,789	0,781	0,198	1,572	0	240	0,429	12,5916	0,066
1,647	0,341	3,209	0,428	4,5	240	4,5	0	0,143
19,871	6,275	5,864	0,786	0	317,143	1	5,1282	1,789
8,221	2,93	1,427	0,428	1,066	257,143	0,143	6,2244	1,789
2,774	10,643	3,855	0,123	4,5	231,429	4,5	0	0,143
6,148	2,433	0,998	0,123	1	180	4,5	53,6256	0,789
3,489	1,35	5,644	0,551	0	240	1	23,058	1,578
7,162	1,064	5,293	0,856	1	514,286	4,5	4,5276	1,578
4,84	1,209	5,139	0,428	0	240	1	2,7972	0,932
7,216	0,921	5,007	0,428	1,143	222,857	0	7,4592	1,143
3,7	0,638	3,932	0,123	0	180	1	30,002	0,143
5,858	0,352	1,132	0,428	0	240	1	13,9944	0
4,784	0,495	5,207	0,551	0	171,429	1	1,8396	0,143
6,204	2,71	0,987	1,214	0	428,571	4,5	7,5264	0,143
5,592	1,996	0,715	0,786	1	317,143	1	27,972	0,572
4,627	1,919	7,71	0,428	0	360	1	0,8064	6,143
5,845	1,064	0,418	0,123	0,789	317,143	4,5	8,9292	0,143
4,983	1,053	2,998	0,123	1	154,286	4,5	68,9332	0,932
6,374	0,932	7,286	0,428	0	154,286	1	6,132	1,578
6,441	2,35	1,776	0,786	4,5	420	1	40,362	0,789
4,212	1,93	6,141	0,786	1	565,714	1	0	1,789
10,714	1,998	1,855	0,428	0	205,714	1	14,4592	0,143
3,42	0,418	5,066	0,123	0	240	0	6,7452	5,289
2,898	1,141	4,429	2	0,789	162,857	4,5	0	0,932
4,35	1,209	0,932	0,786	1	454,286	1	0	0,066
3,775	1,57	3,429	0,428	0	428,571	1	2,688	4,643
8,517	7,01	9,075	0,786	1	214,286	1	0	0,932
5,357	1,921	0,987	1,214	4,5	282,857	4,5	0	1,578
6,581	1,713	3,286	0,428	0	171,429	0	13,2902	1,578
3,049	1,864	11,141	0,428	0	360	1	5,376	6,143
4,625	1,064	3,578	0,123	1	377,143	1	2,7972	0,143

6,15	0,143	0,143	0,123	1	51,4286	4,5	18,0012	0
6,711	0,352	1,572	0,428	1	222,857	0,789	0	1,143
5,509	1,853	1,141	0,123	0	197,143	0	2,7972	1,789
4,849	0,715	4,13	0,428	0	274,286	1	0	6,789
4,5	1,71	6,787	0,428	4,5	120	1	50,3272	0,932
3,865	2,578	1,644	0,123	0	300	4,5	2,688	6,143
6,717	1,842	2,418	0,428	1	102,857	1	10,668	1,789
8,202	1,427	2,855	0,786	0	197,143	1	12,9108	0,143
3,137	1,218	0,932	0,428	1,143	325,714	1	2,7972	0,932
7,548	1,13	0,627	0,428	0	360	4,5	150,01	0,789
5,585	1,715	2	0,786	0	154,286	1	23,31	0,572
5,502	1,284	0,998	0,786	4,5	214,286	0,789	138,0092	0,143
13,348	1,427	2,209	0,428	4,5	180	1	0,9828	0,143
10,71	1,141	1,352	1,214	0	300	0	4,8384	4,643
4,289	1,284	1,352	2,623	0	240	1	3,4944	0,143
3,195	1,273	3,627	0,066	0	334,286	1	8,064	0,143
3,137	0,704	2,998	0,123	0	240	1	46,648	4,643
3,568	0,352	1,198	0,123	1	385,714	1	35,378	0,209
4,412	4,365	8,638	0,428	0	77,1429	4,5	0	5,289
4,275	1,075	0,932	0,428	0	385,714	4,5	0	1,578
5,135	0,275	2,132	0,123	1	205,714	4,5	1,344	4,643
5,421	2,425	4,71	0,786	1,143	257,143	1	45,1192	4,643
5,931	1,287	5,275	0,428	0	180	4,5	4,9056	0,789
7,515	1,864	2,572	0,551	4,5	180	1	8,1732	0,932
3,063	1,919	0,132	0,428	0,066	240	1	0	4,643
8,495	0,836	1,13	0,551	0	248,571	4,5	32,1132	0,932
3,84	0,352	3,352	0,123	0	180	1	15,33	0,143
7,128	2,653	1,075	0,428	0,855	360	1	49,2044	0,789
6,647	1,853	5,13	0,428	0,429	197,143	4,5	16,7832	0,143
5,779	5,718	2,007	0,856	0,789	240	1	32,0992	0,143
3,489	1,361	16,787	0,786	1	240	4,5	24,528	5,289
8,123	2,93	3,51	1,214	0,143	265,714	1	36,5568	4,643
1,056	0,495	3,209	0,066	0	240	4,5	0,8064	0,143
13,777	0,836	3,789	0,428	1,143	205,714	1	5,8632	4,643
6,572	1,71	4,284	1	0,429	145,714	4,5	18,0012	1,066
5,713	2,141	2,216	2	0	548,571	0,429	6,9384	0,855
3,423	1,416	4,932	0,428	1	240	0,789	5,376	4,643
5,715	1,721	2,921	0,786	4,5	300	0,789	0	0,789
5,651	1,638	0,209	0	1	308,571	4,5	21,0084	0,143
3,783	1,13	0,352	0,428	0,932	171,429	1	42,4956	4,643
3,48	0,275	0,352	0,856	0	274,286	4,5	25,3344	1,578
3,339	0,418	2,286	0,428	0	68,5714	4,5	20,2356	6,143
9,44	4,632	10,789	1	0	197,143	4,5	32,6424	0,066
6,157	1,853	2,932	0,123	1	0	1	5,376	1,789
6,491	7,207	9,787	0,428	4,5	471,429	1	5,4852	1,789
7,776	1	0,132	0,123	0	334,286	4,5	1,8396	0,932
3,717	2,15	3,51	0,428	0	137,143	0	0	1,789
7,282	0,987	1,143	0,428	0	360	1	41,1684	0,789

6,504	1,996	0,561	0,428	0	257,143	0,789	21,4452	1,578
7,218	1,996	4,13	0,856	0,789	180	1	52,479	1,143
5,559	2,576	0,921	0,428	0,789	411,429	0	22,0332	4,643
5,337	1,416	1,776	0,428	0,855	205,714	0	2,1504	1,789
2,353	0,429	2,286	0,551	0	420	4,5	8,2824	0,143
5,195	3,222	1,578	0,786	0,789	300	4,5	28,0224	0,286
7,475	1,427	1,341	0,066	0	480	0	0	6,143
2,559	0,429	5,51	0,428	0	214,286	0	2,7972	0,209
7,279	4,786	2,787	0,189	0	162,857	1	0,8064	0,932
5,206	2,785	0,429	0,428	4,5	360	4,5	5,754	0,855
6,651	1,998	3,209	0,428	1	351,429	0	0	1,578
4,352	1,35	8,864	0,428	4,5	154,286	1	28,6692	1,578
1,842	0,561	4,341	0,066	0,066	120	1	5,565	0,495
5,148	2,785	9,13	0,551	0	197,143	4,5	3,4944	0,143
6,152	1,275	0,638	0,786	0	342,857	0	26,3676	4,643
5,983	1,143	7,286	0,428	0	325,714	0	6,72	0,066
5,067	0,418	2,847	0,123	0,429	445,714	1	5,376	4,643
5,212	4,929	5,132	0,428	4,566	300	1	10,038	1,143
2,637	1,787	1,209	0,674	1	60	0	2,5956	0,932
11,14	0,561	1,638	0,856	4,5	360	1	4,8384	4,566
6,516	1,141	3,721	0,428	0	274,286	4,5	9,9036	1,789
6,871	1,141	0,209	0	0	77,1429	4,5	13,986	0,143
2,779	3,653	0	1	0	514,286	0,789	1,6128	0
2,483	1,644	1,721	1	0,789	180	0	1,8396	0,143
6,124	0,616	0,275	0,123	0	240	1	0,8064	1,143
4,061	1,141	3,93	0,428	0	360	1	23,3184	6,143
7,125	1,275	9,141	0	0,143	94,2857	4,5	13,174	0,143
11,863	0,473	1,284	0,428	0,789	360	1	5,376	0,066
4,838	1,93	0,998	0,428	0	600	4,5	36,0024	0,286
3,902	1,13	2,433	0,123	0	497,143	1	9,9036	4,643
3,915	0,352	7,789	0,551	4,5	137,143	1	8,1732	0,932
4,63	6,287	10,418	0,428	1	411,429	4,5	5,376	1,143
2,194	1,13	0,429	0,123	0,143	480	4,5	15,5232	5,289
5,482	1,644	1,209	0,123	1	120	0	0,8064	0,932
4,645	2,075	3,141	0,428	1	394,286	1	0	1,218
10,657	5,204	1,066	4,5	4,643	257,143	1	1,8396	4,643
3,995	1,93	2,789	0,428	0	240	1	14,6832	4,5
6,638	2,499	4,495	0,428	0	325,714	0	5,5944	0,932
4,003	1,787	3,341	0,428	1	300	4,5	46,62	1,578
5,574	1,352	2,284	0,123	0	162,857	0,789	5,754	0,143
5,715	1,207	3,284	0,428	0	257,143	4,5	5,5944	1,578
3,137	1,141	0,198	0,123	0,789	488,571	4,5	3,4944	6,143
5,775	0,704	3,987	0,428	1	360	1	71,316	1,789
5,075	2,576	4	0,428	0	197,143	4,5	5,376	0,932
4,348	2,433	6,209	0,428	0	180	4,5	7,476	1,143
4,946	2,073	1,864	0,428	0	240	4,5	10,752	0,789
3,635	1,985	2,987	0,428	0,789	197,143	0,429	0	0,143
6,013	2,139	2,418	0,428	1	162,857	1	19,0932	1,578

6,66	1,57	7,264	0,428	0,429	248,571	0	18,648	1,789
3,5	1,361	1,064	0,066	0	488,571	1	0	6,143
4,575	1,932	4,576	0,786	0	240	0,789	36,6492	0,495
7,155	2,642	4,073	0,786	4,566	317,143	4,5	3,6792	5,289
4,083	2,864	0,341	0,123	0	274,286	0,066	5,376	0,143
13,577	0,987	2,286	0,428	0	300	1	2,7972	1,143
5,518	1,787	0,286	0	0	197,143	0	0	1,143
7,447	1,218	2,504	0,428	0	180	0	13,5912	0,789
13,143	0,286	6,418	0,428	0	102,857	0	48,9804	0,789
5,704	1,853	2,352	0,123	1	60	4,5	30,66	6,143
4,347	1,284	0,341	0,123	0	180	4,5	7,2156	0,132
5,86	2,93	5,361	0,428	1	214,286	4,5	13,1922	0,932
4,996	0,789	0,066	0,428	0	240	4,5	5,376	0,789
7,346	2,578	0,789	0,123	0	180	4,5	1,8396	5,289
3,643	1,93	2,341	0,428	0	154,286	4,5	21,4452	4,566
7,799	0,932	2,007	1,214	0,066	300	1	36,0024	0,066
6,79	1,71	11,71	0,856	1,066	257,143	4,5	11,1636	1,578
3,434	2,218	1,578	0,428	0	257,143	0	5,376	1,143
3,893	0,132	0,429	0,428	0,789	197,143	0	16,7916	0,066
5,788	3,007	1,209	0,123	0	154,286	4,5	18,1272	0,143
3,416	1,064	1,787	0,428	4,5	351,429	1	40,0932	0,789
6,77	1,218	1	0,123	0	104,286	1	5,5944	0,143
12,301	1,93	2,781	0,428	0	34,2857	0,429	82,8002	0,858
3,629	1,787	0,286	0,123	0,143	274,286	1	8,8704	4,643
3,331	0,495	0,418	0,428	0,789	471,429	4,5	5,376	0,932
8,506	2,141	2,921	0,428	0	180	0,789	0	0,932
3,629	0,407	0,066	0,428	0	180	0,066	641,8636	0,066
13,533	0,429	2,715	0,428	1,066	180	0,143	0,8064	5,289
2,78	1,13	6,493	0,066	0,066	317,143	1	18,648	5,289
2,122	0,561	0,209	0,123	0	600	0	0	0,143
7,306	1,286	10,653	0,428	0,789	377,143	0,429	27,972	4,5
9,852	5,421	11,566	4,5	4,5	128,571	4,5	8,064	4,643
7,284	1,141	3,286	0,979	0	317,143	1	53,3792	0,143
3,778	2,578	5,71	0,428	1	514,286	1	24,1164	4,643
3,706	1,141	0,921	0,066	0	394,286	4,5	6,1824	4,643
4,126	0,638	2,198	0,428	0	222,857	4,5	18,1272	1,789
6,138	0,781	3,284	0,428	0	171,429	1	21,1932	0,789
2,909	5,564	0,275	0,428	0	257,143	0,143	30,3212	0,143
4,263	2,708	7,996	0,428	0	120	1	13,986	4,566
3,48	2,787	1,284	0,428	0,789	137,143	0	2,7972	0,143
5,214	1,207	8,418	0,123	0	94,2857	0,789	1,8396	1,789
3,926	1,93	1,721	0,551	4,5	394,286	1	19,2024	1,578
2,546	0,275	0,407	1,786	0	257,143	0	45,6372	0,143
5,003	2,499	2,198	0,246	4,5	102,857	4,5	45,1528	0
7,163	1,064	5,075	0,856	0	154,286	4,5	60,3232	1,143
6,067	0,99	0,836	0,856	0	531,429	4,5	3,0744	0,143
4,717	5,355	0,932	0,786	0	240	0,789	21,1932	4,643
5,568	0,484	0,998	0,123	0	265,714	4,5	7,4592	0

6,155	*3,433*	*2,286*	*0,428*	*1*	*265,714*	*4,5*	9,324	*1,789*
5,117	*1,625*	*7,789*	*0,428*	*0*	*214,286*	*1*	14,4522	*5,289*
12,381	*1,93*	*2,644*	*0,428*	*0*	*137,143*	*0,429*	0	*0,932*
6,515	*2,062*	*4,075*	*0,428*	*4,5*	*180*	*1*	41,958	*1,789*
6,509	*1,284*	*2,495*	*0,428*	*0*	*154,286*	*1*	8,1942	*1,789*
10,217	*0,855*	*1,132*	*0,856*	*0,789*	*180*	*6*	1,8396	*0*
4,336	*1,559*	*12,275*	*0,428*	*0,143*	*257,143*	*4,5*	46,648	*0,066*
5,215	*1,779*	*3,067*	*0,786*	*0,429*	*180*	*0,789*	40,9836	*0,066*
4,293	*1,842*	*9,341*	*0,123*	*4,5*	*120*	*1*	0	*5,289*
6,067	*1,853*	*5,987*	*0,428*	*0*	*308,571*	*4,5*	27,986	*1,143*
5,803	*4,918*	*4,787*	*0,428*	*0*	*197,143*	*0,429*	60,004	*1*
6,275	*1,209*	*5,286*	*0,428*	*0,429*	*300*	*0*	0	*0,143*
1,977	*0,638*	*4,132*	*0,428*	*0*	*411,429*	*4,5*	14,4144	*0,143*
6,854	*1,209*	*1,207*	*0,856*	*0,789*	*257,143*	*4,5*	8,5596	*0,066*
7,615	*1,352*	*0,198*	*1*	*0*	*222,857*	*4,5*	34,3532	*0,066*
2,854	*4,775*	*0,638*	*0,551*	*4,5*	*120*	*6*	0,8064	*4,566*
4,414	*0,286*	*3,721*	*0,123*	*0,066*	*342,857*	*1*	0	*0,066*
5,935	*2,719*	*7,932*	*0,428*	*0,143*	*291,429*	*1*	2,7972	*1,578*
5,506	*1,787*	*3,066*	*0*	*0*	*90*	*1*	5,5944	*4,643*
5,214	*0,998*	*0,132*	*0,123*	*0*	*300*	*1*	19,5132	*0,932*
6,352	*1,996*	*2,284*	*0,428*	*0*	*265,714*	*1*	21,1932	*1,143*
5,869	*0,572*	*3,141*	*0,428*	*1*	*154,286*	*1*	5,376	*4,643*
6,207	*2,139*	*8,282*	*0,428*	*0*	*180*	*4,5*	32,7992	*0,932*
1,768	*1,141*	*4,433*	*0,551*	*0,789*	*471,429*	*1*	21,4452	*1,578*
7,313	*2,576*	*1,864*	*0,428*	*0*	*360*	*0,789*	18,396	*4,643*
3,06	*0,352*	*4,286*	*0,428*	*4,5*	*360*	*4,5*	16,1364	*0,855*
3,17	*1,776*	*1,776*	*0,123*	*1*	*488,571*	*1*	6,1824	*0,789*
5,294	*1,93*	*0,715*	*0,428*	*0*	*360*	*1*	0	*5,289*
4,066	*0,77*	*2,561*	*0,123*	*0*	*514,286*	*1*	20,9916	*4,929*
12,865	*2,502*	*3,284*	*0,428*	*0,789*	*102,857*	*1*	5,4852	*1,218*
4,715	*3,367*	*3,644*	*0,428*	*4,5*	*300*	*4,5*	30,7692	*5,289*
7,524	*0,998*	*1,286*	*0,428*	*0*	*274,286*	*1*	0,8064	*4,643*
11,204	*0,855*	*3,921*	*0,856*	*0*	*222,857*	*1*	13,986	*0,855*
3,68	*0,693*	*6,576*	*0,551*	*0*	*205,714*	*1*	323,0374	*0,789*
3,983	*1,776*	*1,075*	*1,066*	*0,429*	*420*	*0*	3,4944	*0,143*
6,706	*0,715*	*4,864*	*0,123*	*0*	*94,2857*	*4,5*	45,3922	*1,143*
3,104	*0,209*	*0,352*	*0,428*	*0*	*454,286*	*FAŁSZ*	8,064	*0,286*
3,579	*1,352*	*0,998*	*0,856*	*1*	*257,143*	*0*	0	*0*
6,218	*1,845*	*3,361*	*0,123*	*0*	*480*	*4,5*	4,8384	*1,789*
17,086	*1,93*	*2,578*	*0,551*	*4,5*	*420*	*1*	6,132	*0,143*
5,203	*1,776*	*0,209*	*0,123*	*0*	*351,429*	*1*	0,8064	*1,143*
5,924	*1,196*	*7,207*	*0,428*	*0,066*	*274,286*	*0,143*	5,376	*4,643*
6,284	*2,433*	*2,286*	*0,428*	*0,143*	*120*	*4,5*	27,972	*0,932*
9,229	*2,653*	*6,209*	*0,856*	*0,066*	*480*	*4,5*	35,378	*0,789*
2,414	*1,13*	*2,198*	*0,428*	*0,066*	*437,143*	*1*	63,4592	*1,578*
3,489	*1,064*	*3,921*	*0,123*	*4,5*	*565,714*	*1*	2,688	*4,643*
8,855	*1,352*	*0,209*	*0,551*	*0,789*	*154,286*	*4,5*	7,5264	*0,066*
7,359	*1,493*	*7,284*	*0,428*	*0,066*	*411,429*	*4,5*	27,9832	*0,932*

4,919	1,207	1,638	0,428	0	205,714	1	2,688	6,143
5,94	2,064	0,132	0,428	4,5	180	1	21,3472	1,143
7,785	1,919	4,064	0,428	1,143	420	0,143	5,754	4,643
6,502	5,575	0,561	0,428	0	162,857	0	0	0,789
8,028	1,644	0,143	0,123	0	197,143	1	2,4192	0,143
3,841	1,842	6,418	0,786	0,855	197,143	4,5	0	0,932
5,346	0,987	0,932	0,551	0,789	240	0,789	7,5264	0,789
4,214	1,998	6,352	0,428	0,143	257,143	0	0	1,143
3,327	2,433	3,499	1	0	480	1	21,2184	1,066
10,154	1,352	1,286	0,856	0	420	1	19,7232	0
4,223	0,132	1,987	0,123	0	291,429	1	2,7972	1,429
4,643	1,207	3,921	0,189	0	274,286	1	8,064	0,143
2,546	1,93	0,352	0,123	0,789	240	4,5	9,1896	4,643
6,482	1,919	7,919	0,428	4,5	240	4,5	6,5604	4,643
7,071	0,484	5,275	0,428	0,789	231,429	1	5,376	0,143
7,802	2,576	1,998	0,428	0,789	180	1	1,4784	0,932
4,634	1,787	6,998	0,428	0	300	4,5	32,1496	0,143
7,298	1,35	0,066	0,066	0	240	1	21,462	0
8,944	0	14,284	0,786	1	265,714	1	53,9952	0,143
5,201	1,71	5,143	0,428	0,789	300	4,5	23,9232	5,289
4,983	2,139	3,429	0,428	4,5	428,571	1	13,0242	1,066
7,361	1,141	0,209	0,428	0,789	360	1	1,8396	0,143
3,912	1,704	1,787	0,428	0	120	0,789	6,72	0,789
5,012	1,559	5,853	0,428	4,5	257,143	0,789	2,7972	0,143
11,654	2,719	4,51	0	1,066	137,143	4,5	3,4944	4,643
12,158	2,576	2,647	0,428	0,429	120	4,5	0	4,643
7,667	2,433	1,932	0,428	0	274,286	0,789	18,648	1,789
14,082	1,93	0,987	0,246	0	411,429	1	6,72	0
3,915	1,209	2,407	0,066	0	394,286	1	34,5912	5,289
5,638	1,262	2,418	0,246	0,066	162,857	1	2,688	0,932
7,576	1,427	0,286	0,428	4,5	77,1429	1	26,3676	0,789
8,286	1,721	4,275	0,123	0	240	0	0,8064	0,066
3,981	3,365	7,576	2,5	0	240	1	43,442	1,578
2,535	1,284	1,987	0,428	0,429	342,857	1	25,1636	4,643
5,357	0,704	12,924	0,428	4,5	240	4,5	8,064	4,643
4,212	0,264	5,932	0,123	4,5	197,143	0,143	32,9994	0,789
6,486	1,919	2,721	0,428	1	102,857	0	0,672	0,789
3,492	1,93	3,284	0,123	0	154,286	0,789	2,7972	0,143
15,44	1,064	0,855	0,428	1	180	4,5	5,376	0,066
5,135	2,576	9,932	0,428	0	325,714	4,5	37,296	1,143
6,741	1,855	2,143	0,428	1	222,857	0,789	0	1,143
4,858	0,352	0,264	0,856	4,5	180	0	0	0,143
4,86	1,064	3,075	0,428	1	274,286	4,5	0	0
6,366	1,787	0,998	0,428	0	68,5714	1	11,9364	0,143
1,537	1,361	0,858	0,856	0,789	445,714	1	22,6632	0,143
5,269	1,559	1,921	0,428	4,5	300	1	1,344	1,218
9,592	1,789	0,998	0,786	1	342,857	0	0	0,789
3,926	1,35	2,209	0,198	0,789	591,429	4,5	3,6792	0,143

6,22	5,984	1,429	1,428	1	240	1	13,4652	1,789
7,576	1,064	2,264	0,123	0,143	180	1	8,064	0,143
4,847	1,273	1,13	1,284	0,789	420	4,5	44,3044	0,066
4,181	1,721	2,075	0,123	0,789	120	1	0,8064	0,855
9,809	1,71	6,855	1	1,066	180	4,5	0,8064	1,218
2,557	1,407	0,987	0,428	1	180	1	31,9872	0,143
2,843	1,218	0,132	0,123	0	300	4,5	26,7232	0,066
3,986	1,056	15,284	2,5	0,143	180	1	2,9064	0,066
4,071	1,264	5,578	0,428	0	137,143	4,5	42,3752	5,289
3,06	1,644	0,143	0,066	1	171,429	4,5	1,8396	0
12,214	2,073	2,484	0,428	4,5	471,429	4,5	18,5388	0,932
6,298	1,141	2,207	0,123	0,429	265,714	1	2,7972	1,143
6,212	5,27	4,35	0,428	0	214,286	1	29,19	5,289
4,271	0,418	3,578	0,428	1	240	0,789	67,298	0,066
8,996	2,796	2,787	1,428	4,5	120	1	4,9056	0,066
2,796	0,352	4,286	0,066	0,789	137,143	0,143	14,1036	5,289
6,654	1,996	4,209	0,428	0	120	1	7,6104	4,643
4,283	2,642	3,721	0	4,566	205,714	4,5	127,3524	1,143
6,221	1,93	1,572	0,786	0	377,143	1	5,1282	0,143
8,411	1,133	1,924	1	0,855	308,571	4,5	28,8414	1,143
12,376	3,368	4,51	0,428	0	274,286	4,5	30,1224	4,643
5,555	1,284	1,352	0,428	0	197,143	1	0,8064	1,143
7,851	1,35	0,495	0,551	4,5	120	1	8,5596	0,789
5,928	0,789	3,143	0,123	1	565,714	0,789	0,8064	0,143
4,994	2,13	2,064	1,428	1,066	257,143	1	110,6812	0,855
9,697	2,433	2,352	0,428	1	120	4,5	45,0632	0,286
5,287	1,273	1,776	0,428	0	437,143	4,5	5,4852	0,932
5,193	5,927	1,218	0,856	0	197,143	1	8,6604	6,789
8,208	0,209	4,721	0,428	1	180	0,789	0,8064	0,143
8,022	2,51	13,721	1,428	4,5	60	0	12,3984	5,289
2,57	1,721	3,132	0,428	1	68,5714	0	4,5276	4,643
5,938	2,565	13,651	0,428	4,5	257,143	1	0	5,289
5,562	2,576	2,998	1,214	0,429	214,286	4,5	12,8352	0,286
4,366	1,93	0,418	0,786	0	300	4,5	0	0,789
6,801	1,141	6,275	0,786	0	120	4,5	179,984	0,143
3,634	0,921	1,209	0,428	0	240	4,5	21,462	0
4,774	1,919	0,638	0,428	0	265,714	1	5,5944	0,789
13,427	2,853	3,721	0,428	4,5	77,1429	4,5	29,8116	4,643
5,203	0,987	1,429	0,786	0	660	1	13,44	0,143
1,834	1,864	7,064	0,428	4,5	411,429	0,789	59,1164	4,643
6,941	0,286	5,572	0,123	1	240	1	2,7972	0,143
4,638	0,704	0,495	0,123	0	282,857	4,5	16,3212	0,143
4,928	1,429	1,275	0,123	0	497,143	0	27,972	1,789
14,66	0,998	0	0,123	0	291,429	0	28,7784	0
8,884	1,196	6,264	1	0	180	4,5	2,1504	0,143
2,768	2,932	3,998	1	0,066	300	4,5	23,9232	1,143
4,781	0,429	3,064	0,786	0,429	180	1	0	0,855
3,772	1,71	8,853	0	0	480	4,5	8,064	5,289

6,225	1,141	2,143	0,428	1	72,8571	1	0	1,789
11,646	1,13	3,653	0,189	0	102,857	0	0	1,789
2,757	0,275	0,066	0,123	4,5	282,857	0,143	2,688	1,066
8,914	1,71	1,921	0,856	4,5	308,571	0	3,8724	4,566
5,784	1,361	0	1	0	300	1	34,3392	0
6,001	1,273	5,341	0,123	0	514,286	0	0	0,143
4,423	2,642	3,572	0,246	0	454,286	1	21,3864	1,143
6,078	1,064	1,864	0,428	0	240	4,5	18,6564	0
6,708	0,352	3,073	1	0	107,143	4,5	8,5596	0,143
8,141	1,352	2,998	0,428	0	111,429	4,5	0	0,143
2,469	0,638	5,198	0,123	0	360	0	0	4,566
5,148	2,495	10,789	0,786	0	394,286	4,5	46,62	0,143
5,44	1,064	3,715	0,428	1,789	180	4,5	0	1,789
4,06	1,209	6,218	0,428	0	66,4286	4,5	48,6766	1,789
11,577	2,062	4,855	0,428	0,789	574,286	6	13,0872	0
10,518	1,864	2,578	0,428	0	274,286	1	0	1,143
4,929	1,207	8,495	0,428	1	162,857	4,5	5,5944	1,066
5,421	1,998	0,209	0,123	1	300	4,5	1,6128	0,066
6,944	1,776	3,418	0,856	4,5	137,143	4,5	2,688	0,143
7,469	2,51	1,647	1	0,066	300	1	1,8396	4,643
5,289	1,207	10,209	0,428	1	240	4,5	0	4,643
3,983	2,13	0,921	0,428	6	411,429	1	123,298	0,066
10,206	1,262	2,418	0,428	1	222,857	1	68,5244	1,143
3,647	2,075	1,143	0,428	1	171,429	0	6,1824	0,143
5,496	1,855	1,855	0,123	0	282,857	4,5	13,608	4,643
5,79	2,141	9,073	0,428	0,572	257,143	1	7,6706	0,143
11,131	2,207	7,284	0,786	1	180	4,5	65,5984	1,066
5,058	1,998	0,143	0,066	0	282,857	0,789	8,1564	0,789
11,158	2,218	9,222	0,123	0,789	240	0,789	10,038	5,289
9,08	0,275	0,209	5	1	162,857	1	4,4184	0,789
6,803	1,361	6,715	0,428	1	377,143	1	12,1212	1,578
11,281	1,996	3,132	1,337	4,5	214,286	4,5	53,326	4,929
1,133	2,064	2,998	0,123	0	342,857	0	9,408	4,566
3,052	0,352	0,998	0,428	1	180	6	233,9792	0,143
3,997	2,578	2,132	0,786	0	411,429	1	10,2144	4,643
1,473	2,141	21,987	1	0	420	0	0	5,289
3,261	0,638	4,644	0,123	0	360	1	12,642	4,643
4,972	0,286	1,132	0,123	1	282,857	1	84	0,932
6,649	1,35	2,275	0,428	0,789	565,714	1	15,414	0,143
9,059	0,979	3,787	0,786	0	34,2857	1	8,064	1,789
3,569	0,561	8,222	0,428	0	162,857	0,066	49,4452	1,143
3,783	1,71	0,198	0,428	4,5	162,857	1	2,688	0,789
8,002	2,207	10,216	0	4,5	282,857	4,5	1,8396	0,143
4,418	0,987	2,143	0,123	0	68,5714	1	13,9188	0
10,998	0,704	1,143	0,551	0,066	60	1	6,9972	0,209
2,931	0,418	0,209	0,123	0,789	360	1	189,8022	0,789
6,926	1,427	1,264	0,786	1	60	1	78,0052	1,143
11,36	1,484	2,064	1,214	0	137,143	4,5	1,5624	5,289

6,086	2,642	10,715	0	0	505,714	4,5	0	5,289
7,007	2,15	0,209	0,856	0,066	377,143	1	0	0,066
11,804	1,996	2,352	1	0,066	514,286	4,5	6,9972	0,789
2,337	0,275	10,418	0,066	6	514,286	4,5	30,002	6,143
4,981	5,421	2,998	0,786	1	197,143	0,789	20,3322	0,066
4,083	1,141	1,286	0,123	4,5	257,143	1	45,0632	0,789
7,858	2,062	3,275	1	4,5	222,857	1	33,1128	0,143
8,875	5,63	7,075	0,428	4,566	240	1	8,064	5,289
5,944	1,644	0,352	0,123	0	480	1	18,396	0,789
5,702	1,064	0,264	0,551	4,5	411,429	0,789	1,8396	0,066
4,014	1,13	0,998	0,551	0	462,857	1	3,8724	0,789
8,437	0,932	4,5	0,786	6	120	6	0	6
2,504	1,075	6,352	0,428	0	214,286	1	12,264	1,143
12,013	5,355	1,561	0,428	0	394,286	4,5	66,9424	1,218
2,631	2,073	4,715	0,856	0	420	1	46,62	5,289
7,003	2,139	15,207	0,856	1	317,143	6	11,13	1,066
9,2	1,495	2,132	0,617	4,5	488,571	1	39,4464	1,789
3,625	0,855	2,066	0,428	0	137,143	4,5	2,7972	0,066
8,307	2,653	2,143	0,428	0	162,857	1	4,662	1,143
8,068	0,561	2,132	0,066	0	214,286	1	8,442	0,143
7,069	1,418	5,429	1,284	0	300	1	37,562	1,789
10,628	4,841	4,132	0,123	0,789	514,286	1	3,1836	0,789
9,351	2,075	2,787	0,428	0,789	454,286	1	10,1472	0,066
4,223	0,143	1,207	0,428	0,429	265,714	1	0	0,143
2,92	0,275	2,721	0,428	0	282,857	1	23,3016	0,143
13,937	1,493	5	0,428	1,066	171,429	1	34,986	0,855
3,64	1,141	0,932	0,428	0	368,571	1	0	0,143
3,273	5,498	11,275	0,786	6	102,857	4,5	28,7784	6,789
5,915	1,064	0,132	0,428	0	197,143	4,5	14,9184	0,143
2,744	0,561	5,864	0,428	0,789	257,143	4,5	5,376	1,578
7,093	1,35	1,207	0,786	0	274,286	1	17,2536	0,143
6,295	2,576	1,998	0,428	0	240	0,789	9,324	5,289
5,278	0,998	3,987	0,066	0	471,429	4,5	0	5,289
2,789	1,721	2,932	0,123	0,429	205,714	1	0	5,289
7,867	0,143	3,218	0,428	0	154,286	4,5	96,0064	0,789
2,917	1,578	5,218	0,428	4,5	180	0,789	12,264	4,643
8,694	0,855	1,495	0,856	0	197,143	1	2,688	4,643
1,603	2,141	1,341	4,5	1,066	411,429	1	0	4,643
3,063	4,852	5,275	0,123	4,5	214,286	1	0	1,143
4,48	2,919	1,418	0,551	0,066	480	1	6,9384	0,143
4,654	2,293	1,218	0,786	0,429	300	0,429	25,6564	4,643
5,64	0,209	1,352	0,123	0	214,286	1	23,324	0,932
9,521	1,207	1,644	0,428	0,789	377,143	1	114,2876	0,143
5,355	1,776	3,853	0,123	1	548,571	0	16,3464	0,143
12,219	2,499	0,998	0,551	1	548,571	1	18,648	6,143
3,052	1,787	0,066	0,786	0	197,143	1	5,5944	0,932
3,794	0,998	3,932	0,428	0	137,143	1	5,376	0,932
5,727	1,35	1,066	0	0,789	274,286	0,789	0	0,855

5,724	2,141	8,132	1	0	154,286	4,5	16,128	6,789
6,581	1,207	2,198	1,214	4,5	360	1	35,0392	0,066
5,783	1,053	9,143	0,428	0	385,714	4,5	0	1,218
8,205	2,207	1,638	0,428	0,143	120	1	0	1,789
5,205	1,132	4,929	0,428	1	137,143	1	14,1036	0,789
3,621	1,35	3,35	0,123	0,789	480	1	0	0,143
3,643	2,787	0,987	0,428	0	514,286	4,5	45,2284	0
2,777	6,078	0,132	0,123	1	428,571	4,5	8,064	0,143
5,927	1,644	4,578	0,786	0	325,714	4,5	22,414	4,643
8,993	0,352	0,921	0,551	1	300	1	2,9568	1,218
6,506	2,207	0,998	0,428	1	308,571	0	23,31	1,578
6,078	1,273	5,787	0,123	0,143	480	4,5	10,0548	0,143
5,003	1,919	10,561	0,123	0,789	240	4,5	9,324	0,572
7,916	1,493	2,275	0,428	1	582,857	1	6,132	4,643
6,429	1,119	1,284	0,786	4,5	660	4,5	1,8396	0,066
5,633	1,985	8,575	0,428	0	394,286	1	204,008	0,789
9,589	0,932	0,998	1	1	600	6	54,0036	0
5,144	1,996	9,275	0,428	0	137,143	1	18,648	1,143
5,278	2,499	0,209	0,428	4,566	180	4,5	3,4944	1,789
6,658	1,504	13,207	0,428	0	240	4,5	10,9704	0,932
5,777	1,35	0,209	0,123	0	600	0,429	10,752	0
7,073	1,71	0,209	0,428	0	342,857	1	7,4592	0,789
4,915	1,284	0,352	0,123	0,789	240	1	16,2372	4,643
5,995	1,998	5,156	0,428	0	180	1	22,0752	1,789
4,623	0,998	0,209	1,214	0,789	394,286	1	0	0,066
4,709	3,222	21,987	1	0	360	6	44,268	1,789
3,922	0,998	1,286	0,428	0	497,143	0	0	6,066
2,757	1,207	5,864	0,428	0	154,286	1	0	1,578
5,195	1,853	0,484	0,066	0	394,286	0,143	27,972	0,143
11,428	1,207	2,209	0,856	0	497,143	4,5	22,4112	0,143
4,772	2,644	3,132	0,428	1,066	120	1	9,6768	0,143
5,854	1,218	6,075	0,428	0,143	454,286	4,5	2,9568	0,143
4,99	0,638	0,341	1,428	0,143	180	4,5	54,6504	0,066
3,766	1,141	0,921	0,123	4,5	137,143	0	27,0032	0,066
5,575	2,367	4,576	0,123	0,143	240	1	7,35	1,578
6,719	3,93	12,007	1,284	1	445,714	0	17,661	1,143
8,019	1,284	4,218	0,428	0,429	154,286	1	3,6792	0,143
5,718	1,13	0,132	0,786	0	317,143	0,066	118,9496	0,495
5,643	1,218	1,286	0,123	4,5	137,143	4,5	0	0,143
5,775	1,855	1,921	0,428	0	197,143	0	0	1,066
7,073	1,495	1,855	0,786	1	120	0,789	10,794	1,143
9,851	2,433	0,209	0,246	0	317,143	4,5	76,65	0,143
13,837	0,352	0,132	0,189	0	265,714	1	54,0036	0
1,485	9,198	0,143	0,123	1	120	1	34,986	5,289
3,852	1,572	4,341	0,123	0	360	0	18,648	6,789
6,718	1,064	0,789	0,786	6	300	0,789	3,8724	4,643
7,664	2,064	0,066	0,428	0	137,143	1	6,1824	4,643
5,513	1,779	3,293	0,123	0,429	214,286	1	7,9716	4,643

8,2	5,575	6,075	0,428	4,5	265,714	0	6,9972	6,143
4,401	0,858	4,787	0,066	4,5	308,571	4,5	12,264	0,143
7,708	2,644	1,352	0,066	0,789	240	1	7,8162	4,643
6,614	3,433	3,284	0,428	0,429	8,57143	1	27,972	2
7,284	1,053	2,064	1	0	428,571	1	0	0,143
2,417	1,141	3,198	0,428	0	480	0	6,9888	1,578
4,489	0,55	1,064	0,123	1,143	411,429	0	0	0,572
5,493	0,855	0,143	0,428	1	180	1	5,4852	0,066
10,923	2,13	1,132	1	1	197,143	4,5	6,9888	0
8,869	1,998	2,198	1,428	1	420	1	44,2932	0,143
6,155	0,998	0,484	0,123	0,932	240	1	12,8352	0,143
6,013	2,062	1,781	0,428	0	317,143	1	24,2424	5,289
5,635	1,262	4,418	0,428	0,789	300	4,5	19,3872	0,789
4,623	0,341	1,284	0,123	1	548,571	0	2,5956	4,643
8,993	0,418	0,418	2,623	0	180	1	0	0,143
1,68	1,284	5,495	0,428	0	300	4,5	0	4,643
5,838	0,998	0,855	0,132	0	497,143	1	8,2824	0,143
2,557	0,286	0,143	0,246	0	282,857	0,789	1,8396	0
3,205	1,284	0,352	1	1	102,857	1	0	0,143
5,282	2,787	0,352	2,5	4,5	368,571	6	4,0656	1,143
2,898	1,053	6,576	0,428	0	300	4,5	27,3252	0,932
2,612	0,352	2,429	0,123	1	102,857	1	1,344	0,286
4,48	0,418	1,064	0,979	1	480	0,789	15,2208	0,789
10,646	1,341	4,209	0,786	4,5	308,571	4,5	5,376	0,572
8,443	1,361	1,209	0	0	360	4,5	13,44	0,143
7,275	1,864	5,71	0,428	4,5	557,143	1	30,8084	1,143
3,486	1,644	3,987	0,066	0,066	317,143	1	0	1,066
3,131	0,855	0,209	0,428	0	222,857	1	4,032	0,066
1,265	4,918	7,064	0,123	4,5	514,286	1	0	6,143
7,295	2,565	3,075	0,428	1,066	197,143	1	4,662	5,289
10,501	6,43	1,721	0,856	1	180	1	2,1504	1,578
8,703	0,484	8,51	0,428	4,5	180	4,5	18,396	5,289
5,919	1,053	0,198	0,786	1	497,143	1	27,972	1,066
5,422	1,416	5,341	0,428	0,429	257,143	1	0	0,143
2,906	0,286	0,132	0,428	0	240	1	12,264	0,932
5,851	1,985	2,987	2,5	1	300	4,5	0	0,066
5,128	1,286	0,286	0,428	0	162,857	4,5	14,3472	0,066
5,915	0,572	0,429	0,428	0	291,429	1	3,3726	0,143
2,34	3,431	5,053	0,428	0	300	4,5	7,5768	0,789
4,627	5,498	0,275	0,123	4,5	462,857	1	8,064	4,566
4,85	1,504	4,13	0,428	0,066	420	1	1,6128	4,643
5,42	1,275	0,352	0,123	1	205,714	1	18,396	0,209
3,48	1,932	10,275	0,123	0	377,143	1	7,9716	0,143
4,137	0,998	0,132	0,123	0,789	308,571	0,066	30,66	0,066
12,652	2,642	1,638	0,123	0	137,143	0	0	0,789
1,141	1,13	2,789	0,428	0,066	10,7143	0	4,662	4,643
14,575	1,35	0,987	0,494	0,143	205,714	4,5	12,7512	0,066
3,472	5,487	10,007	1,572	0,789	180	4,5	86,688	6,789

4,634	0,638	5,007	0,428	0	180	1	0	0,932
3,469	0,341	1,075	0,246	1	180	0,789	41,9804	0,066
8,794	2,062	4,427	0,786	0,789	180	4,5	11,7432	1,143
7,436	1,921	4,367	0,786	4,5	325,714	1	0	5,289
4,135	1,996	16,721	0,551	0,789	154,286	4,5	0	1,578
3,282	0,429	1,218	0,428	0	497,143	1	10,0464	0,789
4,489	0,561	1,578	0,786	0,429	137,143	1	27,7116	0,932
6,57	2,073	10,921	0,123	0,429	300	1	13,8768	0,143
7,212	2,644	1	0,123	1	462,857	1	1,8396	1,066
2,194	1,578	4,075	0,428	0	522,857	1	32,8552	5,289
4,5	8,073	6,789	0,786	0	274,286	1	0	4,643
8,204	6,633	3	0,428	0,429	231,429	6	0	0,143
4,203	2,853	8,572	0,786	0	342,857	4,5	16,926	4,643
8,787	1,341	2,578	0,428	0	120	4,5	0	1,578
6,149	1,842	1,864	0,551	1,789	240	0	0,2268	1,218
3,401	1,141	2,275	0,246	0,066	120	4,5	0	1,143
11,729	1,71	2,075	1,428	0,789	120	4,5	2,688	0,789
5,772	1,35	1,352	0,428	0,789	240	4,5	10,038	4,643
4,931	1,416	3,987	0,428	4,5	531,429	0	0	1,578
7,835	1,352	1,275	0,551	0	231,429	4,5	2,688	0,143
4,994	0,275	2,286	0,428	1	248,571	4,5	5,376	0,855
4,997	2,073	3,71	0,428	0,789	274,286	1	29,19	0,572
2,571	2,007	1,932	0,786	0	120	1	13,44	4,643
5,214	2,653	3,007	0,428	1	197,143	1	0	1,143
4,041	1,996	3,561	0,189	1	557,143	0	12,264	0,495
7,366	1,638	2,066	0,428	1	308,571	4,5	0	0,789
6,917	1,339	4,264	1	0	214,286	4,5	12,726	4,643
8,079	1,284	1,998	0,786	0,143	217,857	4,5	30,002	0
5,354	4,852	2,132	0,066	1	214,286	0	0,756	4,643
4,403	2,062	12,209	0,909	6	514,286	1	5,8632	4,566
3,492	1,361	1,218	0,428	0,789	60	4,5	0	1,789
4,849	1,273	3,141	0,428	1	162,857	0,789	0	1,218
5,364	2,282	3,855	0,786	0	77,1429	0,789	14,1036	5,289
5,932	1,141	7,132	1	0,143	77,1429	0	0,756	1,143
9,236	3,945	5,156	0,428	0	300	4,5	46,62	5,289
4,706	1,787	1,209	0,123	4,5	385,714	0,789	8,8704	4,643
7,642	2,062	10,789	0,428	1	394,286	0	47,964	0,143
6,297	2,433	3,644	0,428	4,643	240	1	0	0,855
7,719	1,93	1,578	0,428	1	240	1	25,3204	4,643
6,365	2,576	18,353	0,428	4,5	137,143	1	0	1,143
8,866	1,495	0,715	0,428	1,066	360	1	9,7902	1,143
5,429	1,919	6,576	0,428	0,789	68,5714	4,5	18,648	1,143
3,832	2,433	6,572	0,786	0	257,143	0	0	1,789
4,84	1,275	0,921	0,428	0	342,857	1	7,9716	0,789
4,649	0,998	3,064	0,428	0	360	1	28,2072	0,572
5,781	2,216	5,644	0,428	0	360	1	60,004	5,289
8,238	1,864	1,284	0,856	0	102,857	1	11,6592	0,789
14,638	1,855	0,143	0,797	0	351,429	1	14,1036	0,143

5,503	1,218	2,721	0,786	0,789	162,857	0	2,7972	1
2,92	1,141	1,721	0,856	4,5	180	4,5	6,1824	0,066
7,062	1,776	4,207	0,123	1	240	1	9,408	0,143
6,559	1,209	1,066	0,066	0	377,143	1	0,8064	0,855
17,158	2,796	4,496	0,786	0,789	34,2857	0,429	0	0,932
7,297	3,222	3,361	0,428	0	480	4,5	8,9292	0,143
4,796	2,576	12,578	0,786	0,143	180	4,5	2,688	0,789
5,417	0,341	0,286	0,551	4,5	137,143	4,5	182,7812	0,932
5,139	1,921	2,075	0,428	4,5	291,429	1	11,4744	5,289
10,222	1,361	1,429	0,786	0	342,857	1	18,648	5,289
2,572	1,352	2,789	0,428	0	497,143	1	0	0
9,191	1,13	2	1	0	180	1	116,0544	0,932
4,709	1,218	8,352	0,428	0	214,286	1	9,324	1,143
4,053	2,644	2,367	1	0,066	154,286	1	0,8064	4,566
9,64	2,51	6,221	1,214	0,429	651,429	4,5	6,8292	0,143
10,521	1,987	1,066	0,428	1	120	0	9,324	1,789
6,871	2,073	1,143	0,428	0	128,571	0,789	0	4,643
4,493	0,209	1,998	0,123	1	51,4286	1	37,296	0
5,561	1,284	2,275	0,428	1	497,143	4,5	8,1732	4,643
4,506	1,144	0,572	0,428	0,066	445,714	4,5	18,648	4,643
4,423	2,51	2,341	4,5	0,429	102,857	1	9,2484	0,572
4,625	2,433	4,427	0,123	1,143	531,429	0	10,038	1,066
5,423	1,787	0,418	0,246	0	120	1	0	0,143
6,57	2,433	0,407	1	4,5	222,857	1	11,0208	0,143
6,065	1,273	1,132	0,123	0	514,286	1	20,6052	0,143
3,412	1,339	1,273	0,066	4,5	325,714	1	31,9872	0,932
6,59	1,143	0,132	1,214	4,5	180	1	8,2824	1,143
9,668	2,499	2,141	0,428	0	180	1	29,8116	4,643
7,449	2,576	4,51	0,428	1	137,143	0,789	0	0,789
3,928	0,572	5,286	0,123	0	102,857	0,429	0	1,143
4,696	2,216	3,484	0,786	0	197,143	1	8,3328	4,643
5,012	2,71	8,499	1	1,143	257,143	1	18,7656	1,143
7,001	0,286	3,367	0,494	0	257,143	1	13,0704	0,143
4,983	1,273	0,352	0,428	1	274,286	4,5	0	0,789
6,051	2,13	2,007	0,551	1	300	4,5	0	1,218
5,067	0,704	7,638	0,198	0	377,143	1	3,8724	6,143
2,92	1,13	10,919	0,066	0,429	334,286	1	9,408	0,143
11,729	1,141	1,075	0,428	0	265,714	1	0,8064	0,789
8,442	2,721	12,653	0,428	0,789	240	1	100,2932	4,643
4,509	5,432	4,429	0,123	0	514,286	4,5	6,132	0,789
2,9	0,198	0,855	0,428	0	120	0	0	4,643
4,711	1,218	0,275	0,428	1	462,857	1	8,9292	4,566
10,223	1,209	0,132	0,428	0	197,143	1	0	0,143
4,917	1,13	0,495	0,428	0	282,857	1	12,1212	1,143
7,478	2,075	3,719	0,909	0,495	180	0	38,1024	0,789
7,653	0,198	2,367	0	1	445,714	1	48,0032	0,143
7,001	1,919	2,561	0,786	0,429	240	1	11,9742	0,789
3,06	1,13	1,196	0,066	4,5	531,429	1	2,5956	4,643

3,222	0,066	0	0	0	60	0	0	1
6,563	0,352	2,789	0	0	360	1	0	1,143
9,778	4,984	10,001	0,428	1,143	300	4,5	6,1824	5,289
13,439	1,721	0,132	0,428	0,789	142,857	0	0	0,789
7,411	2,207	1,998	0,852	0,429	197,143	0,789	3,066	0,789
4,491	2,141	5	2,5	1	180	4,5	0	0,143
4,79	1,864	2,864	0,428	4,5	317,143	4,5	0	0,572
3,485	1,493	4,998	0,428	4,5	231,429	1	15,33	4,643
3,64	0,264	0,352	0,428	0	231,429	4,5	59,3712	0,143
7,928	2,576	5,156	0,428	4,5	171,429	1	2,688	1,578
2,207	2,284	7,007	0,428	0	240	4,5	10,752	5,289
5,139	1,286	1,143	0,428	0,429	308,571	0	0	0,143
8,629	1,996	9,352	0,123	0,495	471,429	4,5	12,264	0,932
5,572	3,71	8,51	2,5	0	154,286	0	0	6,789
6,082	1,064	4,429	0,123	1,066	85,7143	4,5	3,4944	1,789
4,981	1,066	3,143	0,123	0,789	317,143	1	0,2268	1
13,062	0,932	0,998	0,428	0	274,286	4,5	9,324	0,789
3,414	1,119	7,853	1	0,066	120	1	191,6768	0,932
5,869	1,276	8,007	0,428	4,5	428,571	4,5	7,5936	0,932
6,438	2,282	7,141	0,123	0	428,571	4,5	7,5264	6,789
5,074	1,493	5,218	0,428	0	214,286	4,5	12,264	1,143
5,269	5,63	7,644	0,428	4,5	360	1	0	4,643
6,623	2,853	4,644	0,428	0	377,143	0	9,1266	5,289
3,932	1,35	5,644	0,428	0,143	274,286	1	23,926	4,643
4,216	1,932	1,132	0,123	1	480	1	2,688	0,143
5,796	6,275	7,643	0,428	1	205,714	1	100,2932	4,643
7,139	0,286	0,132	0,856	0	274,286	1	25,3512	0,789
3,118	0,998	10,209	0,123	4,5	240	1	0	5,289
4,718	2,367	1,429	0,428	0,066	334,286	1	13,7424	1,143
7,071	1,721	1,932	0,428	0	180	1	0	0,143
12,02	3,288	7,156	0,428	0	334,286	4,5	35,833	0,789
6,504	1,13	11,284	0,428	0	214,286	0,789	4,662	4,643
3,994	1,721	2,638	0,428	0	180	4,5	0	4,643
2,186	7,141	1,493	2	0	120	0,429	1,8396	0,932
10,505	7,01	6,572	0,786	0	531,429	1	16,3212	4,643
7,524	1,864	2,796	0,428	4,5	240	4,5	15,9432	5,289
9,356	4,698	0,924	1	4,5	840	1	0	0,932
4,05	1,93	2,572	0,786	1	137,143	1	0	4,643
5,858	1,636	3,299	0,428	4,566	205,714	0,143	21,7924	1,578
4,784	0,781	0,495	0,428	0	360	0	20,986	1,066
4,418	1,636	4,209	0,786	0	180	1	20,0004	4,566
5,419	1,275	0,198	0,123	0	120	0	0	0,143
6,427	0,998	0,209	0,123	0,143	231,429	1	18,0012	0,143
3,918	1,196	1,064	0,786	0	282,857	1	1,8396	0,932
11,217	0,209	1,218	1,428	4,5	368,571	4,5	19,7736	1,789
5,781	2,576	5,786	0,786	0,209	197,143	4,5	69,972	1,143
5,205	1,998	0,209	0,428	0	394,286	4,5	6,132	0,143
4,916	0,638	0,998	0,066	0	300	4,5	12,1212	0,066

6,572	1,842	5,352	0,979	4,5	240	0,066	11,8776	0,855
5,288	5,498	0,638	0	0	154,286	1	23,058	0,143
4,509	4,154	1,932	0,428	0,066	180	4,5	3,1836	0,789
6,291	1,864	2,209	0,428	4,5	137,143	4,5	0	1,789
2,546	1,721	1,721	0,428	0,789	385,714	0,066	7,4592	1,578
4,137	1,853	3,064	0,428	0	308,571	4,5	27,972	6,143
7,295	1,13	4,286	0,123	1	257,143	6	7,4592	0,143
7,8	1,418	4,218	0,428	0,429	480	0	0	1,143
5,926	1,35	1,275	0,428	1	300	1	18,648	0,932
5,946	1,35	0,418	0,786	4,5	180	6	41,9664	0,143
2,713	1,352	2,075	0,428	1	51,4286	4,5	45,0632	4,643
7,486	0,704	8,715	0,551	1,143	342,857	4,5	28,1106	1,143
9,508	3,141	3,053	1	0	120	0	6,0606	5,289
6,201	2,143	0,998	0,428	1	360	1	2,1504	4,643
13,586	4,852	5,498	0,123	1	145,714	1	2,7972	0
8,59	2,853	3,862	0,856	0	222,857	1	20,3952	0,143
3,937	0,572	1,352	0,123	0	117,857	4,5	0	0,789
5,922	1,998	7,787	0,428	4,643	360	0	5,376	1,578
5,503	2,579	0,286	0,856	0	162,857	4,5	46,62	0,143
3,049	0,921	1,064	0,428	4,5	180	0	0	5,289
3,258	2,785	11,787	1,284	0	462,857	1	148,4616	0,789
4,357	1,776	0,286	0,066	4,5	420	4,5	13,9944	6,143
2,722	1,864	0	0,428	0	360	1	0	5,289
7,828	0,132	6,198	1,428	0	300	1	57,0416	0
13,517	1,075	1,504	0,428	1	137,143	4,5	3,6792	0,143
4,704	1,853	0,781	0,428	0,429	154,286	1	2,688	1,143
4,983	1,776	0,987	1	0,066	102,857	0,066	0,8064	0,066
5,584	0,627	1,13	0,428	4,5	445,714	1	0	0,143
6,936	2,708	5,007	0,428	0	282,857	1	0	4,643
8,455	5,355	5,066	0,428	4,5	240	1	6,1824	5,289
3,511	2,796	10,143	0,786	0	240	4,5	0	4,5
6,366	5,564	2,932	0,428	0	214,286	4,5	17,038	0,932
11,574	1,921	0,855	0,428	0	394,286	6	110,3564	0,132
6,925	0,066	2,932	0,428	0	197,143	0	4,1706	0,209
3,72	0,847	0,789	0,856	4,5	428,571	1	33,8562	1,578
4,047	1,998	0,352	0,428	0	360	0,429	30,1224	1,789
7,37	2,007	3,721	0,428	4,5	171,429	0	0	5,289
12,074	2,51	1,987	0,246	0,143	282,857	1	11,662	5,289
8,857	4,995	6,789	0,786	0	222,857	4,5	0	0,789
10,807	1,064	1,578	0,428	4,5	480	0	0	0,143
8,089	2,293	1,064	0,428	1	137,143	1	0	1,143
2,359	1,352	1,286	0,123	0,429	214,286	1	10,752	0,143
8,37	1,196	1,209	0,428	0,143	180	4,5	7,4592	1,578
3,2	1,578	1,427	0,428	0	120	4,5	9,324	1,789
2,565	1,721	1,721	0,189	0	257,143	0,789	21,5796	0,789
6,711	0,418	4,852	0,856	0,429	394,286	0,429	16,3884	0,143
5,761	1,284	1,053	1,428	0	171,429	0,789	34,9832	0,066
6,073	1,427	1,93	0,856	4,5	531,429	1	11,7516	0,932

5,137	0,998	1,066	0,428	1	205,714	4,5	2,7972	0,789
3,84	0,998	2,495	0,551	1,066	120	0	6,321	0,143
2,678	0,275	0,855	0,786	0,789	368,571	0,789	22,3272	0,066
5,718	0,998	0,921	0,428	1	197,143	1	0	1,789
5,133	2,576	0,341	0,123	0	617,143	0	8,064	0,789
5,04	1,919	2,932	1	0	231,429	4,5	0	0,143
1,408	0,352	2,932	0,551	4,5	197,143	1	10,2564	0,143
8,959	1,064	4,51	0,428	0	600	1	5,5944	5,289
5,117	0,921	4,495	0,551	4,5	334,286	1	2,688	0
5,088	2,348	0,132	0,123	4,5	411,429	4,5	43,6688	0,143
6,36	0,275	0,855	0,123	0	120	1	0	5,289
6,48	2,644	6,5	0,428	0	360	1	17,038	1,143
7,499	1,93	2,286	0,551	0,429	137,143	4,5	0,8064	0,143
5,983	0,998	2,209	1	1	385,714	4,5	28,5852	0,789
3,788	1,559	6,132	0,428	4,5	420	1	12,6336	4,643
6,273	0,789	10,921	0,786	0	351,429	0	6,5604	0
4,283	0,998	1,075	0,123	0,143	308,571	0	2,7972	6,143
6,201	1,93	2,653	0,551	0,286	137,143	4,5	3,4944	1,143
7,304	1,996	4,638	0,428	0	308,571	1	2,7972	0,143
6,638	1,141	0,418	0,123	0,789	240	4,5	150,3292	0,066
4,038	0,286	1,429	0,123	1	102,857	4,5	1,8396	0,209
4,058	6,43	1,721	0,428	0	394,286	1	0	0,143
5,781	1,919	1,141	0,428	0	240	0	0	0
4,414	1,644	0,638	0,428	4,5	360	0,066	28,9044	4,643
6,849	2,644	1,075	0,428	1	402,857	4,5	34,9272	0,066
6,209	2,367	1,932	0,123	1	188,571	1	5,4852	0,932
3,577	0,341	0,932	0,246	0	180	1	0	0,143
8,447	1,504	6,299	0,428	0,429	137,143	0,143	0	1,218
5,618	1,495	7,864	0,786	1,066	385,714	4,5	1,533	0,066
5,497	0,715	2,998	0,428	1	420	0,789	0,8064	1,143
9,14	1,075	0,286	0,132	0,789	214,286	4,5	0	0,932
1,265	2,139	17,927	0,123	1	291,429	4,5	24,528	4,643
8,352	1,998	2,721	0,428	0,789	180	4,5	2,688	5,289
6,919	1,341	2,721	0,428	1	162,857	0	1,344	1,143
9,932	1,418	4,209	0,428	0	137,143	0,066	45,7352	0,143
5,66	1,427	2,286	0,428	0,789	257,143	0	24,528	0,143
5,491	1,339	1,429	1,214	4,566	368,571	0,066	0	0,789
5,425	1,196	6,275	0,428	1	197,143	6	9,5508	0,932
1,812	1,262	2,141	0,428	0	394,286	1	18,7656	1,578
8,765	0,484	9,644	0	4,5	274,286	0,066	107,9904	4,566
6,214	1,644	0,998	0,856	0	240	1	0,2268	0,066
8,85	0,572	6,627	0,123	0,789	360	1	0	0,789
3,203	2,51	8,141	0,123	1	377,143	1	0	1,578
7,73	1,218	1,704	0,428	0	180	4,5	1,6128	0,789
2,075	1,209	0,932	0,123	1	162,857	4,5	10,2816	4,566
6,524	6,707	4,286	0,428	0,066	360	1	14,4312	5,289
6,095	1,43	0,932	0,786	0	48,5714	0,429	0	0,429
4,06	1,853	0,781	0,551	1	154,286	1	9,0888	1,066

4,792	2,565	1,141	0,066	0,789	471,429	0	3,8724	4,643
4,113	5,138	5,284	0,786	0	300	4,5	30,058	6,789
7,871	1,919	1,853	1	1	531,429	1	6,4722	4,566
6,506	3,082	1,284	0,428	4,5	180	1	12,264	6,143
5,22	0,572	2,075	0,123	1	428,571	4,5	6,8292	0,495
2,832	0,539	6,653	0,428	4,5	514,286	1	7,1736	0,572
3,063	2,502	0,921	0,786	0,789	257,143	1	0	0,143
7,53	2,433	3,578	0,428	0	300	0,789	0	5,289
4,127	1,207	0,429	0	0	137,143	0,066	36,9908	0,143
5,291	1,93	4,209	0,786	1	497,143	1	6,132	4,643
5,344	1,198	9,352	0,428	1	197,143	4,5	0	1,143
20,567	5,432	0,855	0,428	0,429	351,429	0,143	24,528	0,789
3,975	2,51	1,561	0,123	0,143	240	4,5	5,376	1,578
5,357	1,064	1,998	0,123	1	222,857	0,789	0,8064	0,143
6,484	1,999	0,638	0,428	4,5	180	1	8,064	0,143
6,273	2,284	4,064	0,786	1,066	334,286	1	0,378	5,289
10,585	1,504	1,578	0,786	0	334,286	0	0	0,143
4,194	1,932	0,132	0,786	1	120	0	3,4944	0,066
6,719	1,644	3,787	0,428	0	420	0,789	6,132	0,143
2,898	2,565	8,561	1	1	180	4,5	5,4852	4,643
6,142	1,207	1,57	0,123	4,566	428,571	4,5	19,7232	0
6,069	0,858	6,218	0,428	0	231,429	1	61,7512	5,289
6,642	0,198	2	0,246	0	180	1	4,662	4,566
5,782	1,493	1,264	0,189	0	162,857	1	10,0044	1,143
6,365	0,352	1,921	0,428	0	102,857	4,5	11,662	1,789
6,926	2,075	5,787	0,428	1	282,857	4,5	15,33	1,789
8,844	1,284	1,275	0,428	0	180	1	5,8632	0,143
12,595	1,416	0,998	2,5	0	265,714	4,5	8,1564	0,066
5,585	4,775	0,132	0,428	0	342,857	1	84,532	0,066
3,118	1,284	1,704	0,428	1,066	282,857	1	21,714	0,932
5,994	2,862	4,341	0,856	0,143	522,857	0,789	10,773	6,789
5,427	1,842	5,51	0,428	1	497,143	1	0	1,143
7,34	0,264	1,066	0,123	0	445,714	0,789	2,9568	0,143
3,555	2,642	6,053	0,979	4,5	180	4,5	23,3772	0,932
6,505	1,919	3,407	0,786	0,066	145,714	0,789	35,588	1,143
4,852	0,921	1,776	0,551	0,855	282,857	4,5	120,8144	0,855
8,638	0,932	1,644	0,551	0,789	240	1	2,1504	0,789
9,58	1,504	0,921	0,786	4,5	257,143	4,5	0	0,066
4,992	0,352	1,264	0,428	4,5	257,143	1	6,1824	1,578
7,096	1,286	7	0,132	0	394,286	0	2,7972	0,789
9,102	0,781	2,578	0,428	0	377,143	4,5	2,7972	0,143
9,077	1,339	12,865	0,428	0,855	257,143	4,5	16,1196	5,289
4,794	0,998	0	0,428	0	282,857	0	2,7972	0,143
3,339	0,352	0,921	0,428	0	325,714	1	3,1836	0
5,157	1,143	2,143	1,214	1	214,286	4,5	18,0012	0
6,137	0,998	1,787	1,214	0	240	1	0,8064	0,143
3,414	2,062	1,361	0,428	0	617,143	1	6,132	0,143
15,506	1,559	0,998	0,786	0	334,286	4,5	6,8628	5,289

5,355	2,576	1,275	0,428	4,5	120	0	3,3726	4,643
4,354	1,207	2,429	0,123	1	454,286	0	0	4,643
5,271	1,996	1,13	0,786	0	685,714	4,5	11,2476	0,143
7,719	1,787	4,053	0,428	0	197,143	1	2,7972	1,143
5,553	1,908	2,433	0,428	0,789	180	0,789	33,1884	5,289
5,786	2,15	0,789	0,428	0	85,7143	0,789	69,972	0,932
4,418	1,207	2,286	0,428	0	471,429	0,789	0	1,578
5,355	2,216	5,218	0,123	0,143	257,143	1	2,688	4,643
4,994	1,141	0,286	0,428	0	411,429	1	15,0612	5,289
9,075	3,299	3,284	0,123	4,5	111,429	4,5	22,6632	1,789
5,44	2,576	5,576	0	0,143	154,286	1	10,8948	6,143
7,742	2,216	0,998	0,428	0	360	1	24,528	4,643
9,089	1,919	3,578	0,428	0	411,429	1	27,972	4,643
7,06	2,064	6,218	0,428	4,5	514,286	1	12,4684	4,566
4,877	1,504	9,207	0,428	1	428,571	4,5	0	6,143
9,934	0,638	0,998	0,246	0	120	4,5	6,132	0,143
6,697	3,354	2,15	0,428	0,066	214,286	0	5,4852	4,566
3,346	1,721	2,495	0,674	0	300	4,5	23,058	0,932
3,052	2,007	0,132	0,123	0,429	428,571	4,5	5,5944	5,289
14,783	2,504	5,219	1	0,789	240	0,143	2,688	4,643
6,64	0,998	8,855	0,428	0	154,286	4,5	0	4,643
7,368	5,553	2,787	0,856	0	334,286	1	43,3524	0,143
3,275	1	2,066	0,123	0	342,857	1	0,8064	1,789
7,908	0,921	0,855	0,551	0,789	120	1	10,794	0
8,635	1,141	1,787	1	0	565,714	4,5	18,648	5,289
11,083	0,847	5,218	0,428	4,5	274,286	0	8,9292	0,143
5,643	1,427	4,721	0,428	0	360	4,5	1,8396	1,578
4,567	1,418	1,287	0,786	0	240	1	4,032	0,932
4,137	2,642	4,275	0,428	0	411,429	4,5	93,24	1
4,721	2,864	4,578	0	4,566	531,429	1	12,264	4,566
2,403	0,572	0,198	0,066	0	514,286	1	7,5264	0
2,843	1,864	2,418	0,428	4,5	420	0,429	2,1504	4,643
4,495	2,341	0,066	0,428	0	94,2857	1	0	1,143
6,427	2,642	1,273	0,856	0,143	265,714	4,5	37,7622	0,209
3,79	1,998	8,198	1	0	257,143	0	1,8396	4,643
7,873	6,21	9,853	0,428	0,789	171,429	4,5	7,476	5,289
4,711	1,644	0,932	0,123	1	300	0	1,5624	4,643
5,421	2,207	6,275	0,428	0,209	197,143	1	12,8352	1,218
6,003	1,853	2,932	0,428	4,5	180	0,066	60,8104	0,143
6,726	3,51	5,066	1	1	514,286	4,5	70,0952	1,143
4,261	2,271	1,932	0,066	0	300	1	5,831	0,789
4,768	0,932	1,789	0,428	0,789	471,429	1	23,31	0,143
8,332	1,207	8,721	0,123	0,789	274,286	4,5	8,442	1,218
4,432	0,341	0,932	0,551	0	394,286	1	36,3636	4,643
4,632	5,707	0,998	0,428	0	411,429	1	18,0012	1,789
5,289	0,638	0,429	0,551	0	214,286	1	14,952	0,143
4,994	3,064	1,064	0,123	0,132	351,429	4,5	24,528	0,932
7,875	1,495	4,143	0,428	1	111,429	1	6,1824	0,143

2,917	0,855	5,13	0,246	4,5	120	0,066	7,7154	1,143
4,412	1,985	5,207	0,428	1	265,714	1	9,3282	0,932
6,218	1,853	2,341	0,428	1	180	4,5	18,2112	0,143
3,625	1,286	2,644	0,428	1	171,429	1	7,9716	1,143
4,643	0,979	6,578	0,786	0,143	394,286	1	0	5,289
5,555	1,864	0,352	0,674	0,429	282,857	1	6,1824	0
6,865	1,13	1,13	0,428	0	214,286	4,5	23,3184	0,143
5,719	1,075	0,932	0,123	1	548,571	1	5,376	0,143
10,283	1,064	1,427	0,246	1	120	0,143	8,5512	0,495
4,86	2,653	6,367	0,428	4,643	197,143	4,5	0	1,143
5,265	1,787	0,275	0,123	1	565,714	4,5	30,3212	0,132
15,506	5,289	2,578	0,428	4,5	308,571	4,5	33,6812	0
5,347	2,504	6,796	1,214	0	137,143	1	34,9272	0,789
3,032	3,357	2,787	0	4,5	274,286	0,143	0	4,643
9,58	2,719	1,132	0,428	0	282,857	0	0	4,643
3,58	0,352	2,367	0,428	0	180	0	0	4,643
4,484	1,493	0,495	0,428	1	291,429	1	15,1872	0,789
6,271	1,352	2,932	0,428	4,5	77,1429	4,5	1,8396	1,066
4,363	1,141	3,075	0,428	0	300	0,789	7,4592	0,143
9,283	0,987	1,781	0,856	0	120	1	1,344	0,789
4,486	2,996	7,139	0,123	6	317,143	1	1,8396	5,289
4,994	2,367	4,787	0,428	0	94,2857	1	1,8396	1,143
4,282	1,721	10,078	0,786	0	197,143	4,5	18,648	1,143
7,135	2,994	1,35	0,428	1	480	0	49,4172	0,932
8,524	2,51	4,075	0,428	0	394,286	0	24,528	5,289
2,131	1,361	9,064	0,123	0	360	1	0	5,289
4,561	0,836	3,429	0,066	4,5	300	1	13,44	0,143
6,139	1,35	6,638	0,428	0,066	240	1	2,688	4,643
4,258	1,416	4,284	1,428	4,5	300	0,066	22,0542	4,566
5,498	1,427	3,007	0,786	0	462,857	1	8,6604	0
7,011	1,075	5,075	0,428	0,429	60	4,5	0	4,643
17,786	6,43	0,209	0,428	1	360	6	0,378	5,289
4,583	1,207	2,644	0,786	1	385,714	1	0	5,289
15,878	2,576	2,075	0,786	0	240	0	23,1924	5,289
6,135	1,93	2,576	0,551	0	240	1	9,1476	0,932
4,205	2,721	1,284	0,786	0	180	0	0	5,289
5,495	2,062	0,781	0,428	4,5	180	4,5	8,3412	0,066
5,289	0,789	0,143	0,123	0,789	548,571	0	0	1,066
2,689	3,075	1,921	0,551	0,429	222,857	4,5	2,688	6,789
4,332	6,21	6,075	0,856	0	240	1	13,9776	5,289
5,067	2,15	11,864	0,551	1,789	480	4,5	9,6012	4,643
5,784	0,715	12,286	0,786	4,929	411,429	1	5,376	4,643
7,44	0,143	2,066	0,123	1	140	4,5	0	0,066
3,717	1,275	4,209	0,123	0	274,286	1	8,064	4,643
7,101	2,433	5,156	0,428	0	274,286	4,5	29,5848	1,578
5,146	2,075	0,932	1	1	360	4,5	2,7972	5,289
5,431	1,853	1,209	0,428	0	377,143	1	9,324	4,643
5,298	0,789	0,275	0,123	0	300	1	14,3472	0

11,521	2,007	9,352	0,428	0	60	4,5	4,662	1,789
7,66	2,15	8,796	0,856	0	325,714	1	0	0,143
5,34	1,13	3,721	0,123	0	102,857	1	18,0012	0,066
4,924	5,421	1,275	0,428	0	240	1	28,4004	0,572
4,689	1,207	3,196	0,551	0,132	197,143	1	26,6784	0,066
4,709	2,073	1,284	1,572	0	411,429	4,5	11,6508	0,495
7,807	2,053	4,073	0,428	0,789	222,857	1	2,9736	1,578
5,787	4,643	3,156	0,428	1	240	1	2,688	0,143
7,572	2,576	3,51	0,123	1,789	385,714	0	23,324	4,643
4,011	0,998	0,352	0,123	0	171,429	4,5	2,688	1,066
7,436	1,284	6,275	0,428	0	248,571	0	18,6592	1,143
5,927	2,218	7,71	0,428	1	265,714	1	2,7972	4,643
12,298	0,627	1,066	1	1	360	1	48,0032	0,855
7,804	1,93	1,932	0,428	0	334,286	4,5	11,1888	0,143
3,06	0,209	0,484	0,551	0	180	1	19,8352	0
9,732	1,13	3,007	0,856	4,566	102,857	1	0	5,289
2,414	1,853	1,066	0,428	4,566	531,429	0	18,7362	4,566
3,118	1,119	1,418	0,066	0	420	1	0	6,143
6,282	1,284	3,066	0,428	1	300	4,5	2,7972	0
5,783	2,785	0,998	0,428	1	248,571	0	0	0,143
3,843	1,064	1,218	0,123	1	205,714	0,789	0,378	4,643
7,431	1,853	4,932	0,428	4,5	180	4,5	0	4,643
1,815	1,922	6,064	0,786	0	137,143	4,5	0	5,289
4,06	2,653	3,288	0,428	4,5	137,143	4,5	5,0946	4,643
19,654	2,433	4,715	0,786	0	317,143	1	0	0,789
4,781	1,561	4,433	0,428	1	531,429	1	0	5,289
1,746	6,564	5,284	2,5	1	300	4,5	111,9524	4,566
6,935	2,284	4,053	2,5	0,066	334,286	4,5	15,0864	1,218
6,069	1,853	2,209	0,428	0,143	94,2857	1	0	5,289
7,073	1,71	2,198	0,856	0	137,143	4,5	1,8396	0,143
3,889	1,209	0,855	0,428	1	360	0	0,8064	0,066
8,486	2,073	2,352	0,786	4,5	257,143	4,5	13,9944	0,066
5,79	0,847	2,139	1,214	1	222,857	1	33,8436	0,932
3,504	0,932	2,143	0,428	1	90	4,5	1,8396	1,143
7,728	1,495	4,864	0,428	1	77,1429	0,789	0,9954	0,789
5,704	1,207	1,429	0,066	0	180	1	0,8064	0,143
4,347	5,707	2,275	0,428	0,789	437,143	0,143	8,8704	4,643
3,367	1,853	1,286	0,123	0,789	60	0,066	0	1
4,502	2,367	2,429	0,123	0	240	0	0	0,143
5,649	1,427	0,286	0,428	0	60	0	0	4,643
7,06	0,341	0,275	0,428	4,5	180	1	36,1032	0
4,489	0,55	2,715	0,123	0	162,857	1	18,396	1,143
6,872	1,141	1,864	0,428	1	180	4,5	4,662	0,132
4,696	2,367	5,932	0,428	1	300	0,789	3,4944	0,855
6,003	2,433	1,578	0,428	0,429	394,286	1	4,8384	0,789
5,774	0,932	1,132	0,123	1	342,857	4,5	0	0,143
4,775	2,719	0,998	0,428	0	300	1	7,5936	0,143
5,695	1,13	0,341	0,123	0	257,143	4,5	1,7976	0,066

4,48	1,473	6,064	0,428	1,066	154,286	1	2,688	1,143
5,858	1,504	1,35	0,428	0	342,857	4,5	0	1,789
13,144	0,932	6	0,428	4,5	171,429	4,5	27,972	0,143
4,946	1,644	8,358	0,786	0	162,857	0	0	0,789
3,757	1,787	1	0,123	1	137,143	1	1,8396	0,789
5,627	1,996	1,075	0,123	1	180	1	13,44	4,643
3,9	1,196	0,407	0,428	0	462,857	1	21,1932	0,143
6,368	1,207	2,284	0,428	0	94,2857	4,5	12,2346	4,643
9,848	1,064	1,286	0,786	0	282,857	4,5	0	0,932
10,151	1,864	1	0,123	0	351,429	1	84,0056	0,143
3,933	1,275	0	0,786	1	411,429	4,5	75,005	0,143
3,537	2,642	10,433	0,428	4,5	420	4,5	41,9272	1,578
3,794	1,853	3,218	0	0,066	394,286	4,5	9,2484	1,143
6,355	6,144	2,644	0,551	0,066	197,143	1	78,4616	0,143
5,148	1,996	10,064	0,428	0	154,286	1	15,5232	4,643
5,57	1,572	1,209	0,428	0	360	1	0	0,143
8,202	0,407	2,13	0,123	0,789	282,857	4,5	1,5708	4,566
7,233	1,427	0,932	0,123	1	334,286	4,5	18,396	0,789
6,782	0,987	6,495	0,123	1	411,429	0,789	0,8064	0,572
2,994	0,132	1,341	0,066	0	257,143	1	0	4,566
5,642	2,066	8,275	1	0	77,1429	4,5	0,8064	5,289
3,917	2,139	6,645	0,066	4,5	334,286	1	18,648	4,643
3,783	0,704	1,075	0,428	4,5	77,1429	1	7,4592	1,578
3,794	3,222	2,998	0,428	4,5	180	0,143	9,9204	0,143
4,99	1,559	0,855	0,428	0,429	531,429	4,5	35,5656	4,643
5,271	1,218	0,275	0,123	0,789	205,714	1	12,8352	0,143
6,849	1,919	2,352	1	0	154,286	1	5,8632	1,143
2,416	2,067	5,787	0,123	0	454,286	4,5	19,068	6,143
3,988	0,198	3,141	0,428	0,066	317,143	4,5	2,7972	0,066
10,303	1,578	1,721	0,428	4,5	240	4,5	3,066	5,289
6,869	2,721	7,644	0,428	4,5	497,143	4,5	0	0,143
5,647	0,572	2,932	0,123	1	462,857	1	0,8064	4,643
7,708	0,495	0,998	0,856	0,855	300	4,5	6,9972	0,143
4,273	4,5	0,209	0,123	0	600	1	0	0,143
7,506	2,578	1,361	0,428	0,143	377,143	1	0	1,066
4,926	2,13	9,367	0,551	0	197,143	0,789	0	5,289
7,804	1,856	1,79	0,428	0	300	1	26,9472	4,643
6,733	6,933	5,499	0,856	0	162,857	1	116,2644	0,789
6,484	1,075	5,721	1,428	0	317,143	1	67,9812	1,066
6,862	1,853	1,427	0,428	0	154,286	1	16,7832	0,143
3,568	2,139	4,361	0,428	0	120	1	11,655	1,143
5,08	1,361	1,352	0,066	6	351,429	1	6,1824	6,143
3,841	4,088	8,864	0,066	0	334,286	1	6,132	4,643
5,983	1,416	2,064	0,428	1	180	4,5	24,115	0,066
11,375	3,429	3,939	0,786	1,066	360	1	1,8396	1,218
6,789	0,484	3,132	0,428	0,789	300	4,5	8,3916	0,143
4,649	1,13	0,998	0,066	0	308,571	4,5	98,784	0,143
13,715	1,416	4,13	0,428	0,789	325,714	0,789	14,7924	0,143

2,337	1,787	5,198	0,066	1	317,143	4,5	30,31	4,643
7,516	1,064	1,075	0,786	0	317,143	1	0,8064	0,143
12,339	1,644	0,143	0,786	1	180	0,066	16,3884	0,789
5,498	1,064	1,284	0,856	0,789	445,714	1	13,9944	0,143
4,698	1,57	5,275	0,123	1	377,143	1	4,5276	0,789
3,603	0,495	0,209	0,428	0	514,286	1	6,7872	0
7,449	0	2,218	0	0,789	377,143	0	1,8396	0,066
13,819	3,299	3,361	0,428	0	274,286	0	12,264	5,289
4,794	0,781	0,143	0,428	0	222,857	0,143	5,5944	0,932
6,795	0,998	2,578	0,428	1	222,857	0	5,376	0,143
5,432	1,064	0,932	0,428	0	197,143	0	9,8112	1,143
4,853	0,198	0,932	0,123	1	145,714	0	0,8064	1,143
8,769	2,273	1,064	0,123	0,789	462,857	0,143	8,5596	1,143
3,772	2,282	4,433	0,066	0	274,286	1	14,784	4,643
3,639	1,207	5,066	0,123	0	94,2857	4,5	0,8064	0,143
5,923	0,998	3,15	0,909	0,789	377,143	1	39,9056	1,066
4,641	1,484	0,132	0,428	6,066	282,857	4,5	0	0,143
3,775	2,51	4,141	0,786	1	240	0,143	21,8316	5,289
10,949	0,143	1	0,786	0	548,571	4,5	21,0672	0
6,304	3,365	2,578	0,428	0	300	0,789	30,0552	0,572
6,442	2,433	0,286	0,066	0	102,857	1	0	1,143
3,862	1,286	0,209	0,123	1	317,143	0	5,4684	1,143
12,054	1,644	0,495	1	0	308,571	0	26,4432	0,143
8,933	0	0,143	0,123	0	411,429	1	8,1732	0,066
6,438	1,141	4,284	0,428	0	257,143	1	14,4592	0,572
13,299	1,864	5,075	0,428	4,643	282,857	0	0	1,143
6,295	1,93	12,638	0,428	0	154,286	4,5	4,662	1,143
6,562	1,856	2,855	1	0,066	265,714	1	93,296	4,643
7,518	1,864	3,15	0,428	0	257,143	4,5	12,264	5,289
5,495	1,932	6,433	0,428	4,5	154,286	1	36,19	1,143
6,796	1,721	4,218	0,786	1	31,4286	1	0	0
5,878	1,495	5,284	0,909	0	120	1	20,8992	1,143
6,86	1,339	4,209	0,428	6	154,286	1	24,528	1
7,216	2,642	4,429	0,428	0	308,571	4,5	12,096	0,209
4,216	0,418	2,789	0,123	0,789	171,429	4,5	6,9384	0,066
4,703	1,93	1,71	0,909	0,789	240	1	18,0292	1,143
5,432	1,053	1,429	0,428	0	214,286	4,5	3,6792	0,143
6,335	1,985	0,638	0,123	1,143	300	0	8,6604	0,132
10,741	1,644	0,987	0,786	1	428,571	1	0	0
6,945	1,853	5,299	0,856	0	248,571	1	5,4852	0,209
13,726	0,704	2,352	0,123	0,429	94,2857	0,789	11,7432	0,066
8,673	2,51	3,064	0,428	0	308,571	1	26,7232	0,143
7,238	0,418	6,352	0,123	1	325,714	1	21,4452	0,932
5,927	0,921	5,341	0,428	0	334,286	1	12,1212	1,143
5,641	0,572	0,418	0,123	0,429	145,714	4,5	0	0,143
7,704	2,425	1,064	0,786	0	377,143	4,5	3,6792	0,572
4,687	1,275	2,71	0,428	1	480	1	7,5264	1,143
4,929	1,196	3,156	0,123	0	300	0,066	10,0842	4,643

9,137	0,55	5,064	0,428	0	154,286	1	0,8064	1,578
5,785	2,719	4,495	0,123	0,789	402,857	4,5	6,1824	1,143
11,582	1,493	2	0,786	0	120	4,5	6,5016	0,932
2,188	0,627	3,998	0	0	377,143	1	60,004	0,143
5,478	0,77	0,066	0,066	0,066	300	1	10,668	0,143
4,192	1,207	0,275	0,123	1	111,429	1	6,3672	0,286
4,109	0,275	2,352	0,123	1	145,714	1	5,4852	0,286
3,864	0,932	0,132	0,428	1	334,286	0	0,8064	4,566
3,489	1,647	0,209	0,066	4,5	531,429	1	5,8212	0,066
8,679	1,71	0,921	0,428	0	51,4286	1	14,35	1,066
2,986	2,053	4,932	0,786	1	411,429	1	6,9972	1,143
4,08	1,787	7,066	0,123	4,5	248,571	0	18,648	0
6,724	2,642	9,578	1	1	240	1	2,7972	4,643
1,625	1,71	8,418	0,066	0	394,286	4,5	32,004	6,143
2,961	1,787	2,572	0,856	0,066	282,857	1	26,6784	1,578
4,071	1,427	1,209	0,428	4,5	222,857	4,5	4,0572	1,578
6,509	1,721	11,504	0	0	222,857	4,5	12,264	6,789
8,932	1,35	0,352	0,189	1,066	531,429	0,429	13,0704	1,143
9,574	0,264	2,209	0,123	0	94,2857	1	5,376	1,143
3,192	3,576	6,066	0,786	0	240	1	6,132	0,789
8,372	0,704	0,484	1,428	1	334,286	4,5	3,0744	0,789
6,557	0,704	1,264	0,428	0	334,286	4,5	15,6324	1,143
6,506	2,644	1,064	0,123	4,5	385,714	1	4,662	0,855
7,306	2,062	8,359	0,428	1	154,286	4,5	6,132	5,289
5,012	3,156	0,132	0,428	0,132	428,571	4,5	23,31	1,066
1,985	0,429	9,787	0,123	0	154,286	4,5	40,0932	5,289
5,707	1,79	1,275	0,428	0,066	180	4,5	91,322	5,289
7,161	1,075	2,418	0,786	0	240	1	1,8396	1,143
4,559	1,196	13,643	0,428	4,5	402,857	1	69,972	1,789
1,111	1,427	1,721	0,123	4,643	334,286	0,429	14,1036	6,066
10,72	1,998	3,209	1	0	171,429	0	13,0704	4,643
5,992	3,365	6,365	0,428	4,5	205,714	0	13,986	4,643
5,492	5,707	0,352	0,123	0	180	1	0	5,289
2,15	0,932	9,209	0,786	0,789	317,143	0	54,0036	0,789
6,078	0,638	9,787	0,428	1	180	4,5	12,1212	1,143
6,575	2,139	3,064	0,428	0	454,286	0,789	8,064	1,789
10,002	3,71	7,852	1	0	257,143	1	2,016	5,289
7,769	1,484	4,495	1,856	1	248,571	4,5	18,6396	1,789
4,772	1,075	5,141	0,428	0	325,714	0	3,4944	4,643
4,77	1,853	0,352	0,123	1	265,714	0	0,8064	4,643
3,985	0	0,066	0,428	0	51,4286	1	2,688	4,566
6,139	2,787	4,853	0,494	4,566	282,857	4,5	2,7972	0,789
8,011	0,132	3,71	0,428	0,429	197,143	4,5	0	0,855
7,436	1,35	3,781	0,428	1	265,714	0	12,1212	1,143
2,766	0,341	3,141	0,066	4,5	617,143	1	6,9384	4,643
4,489	5,421	8,218	0,428	4,5	282,857	1	8,3412	4,566
4,28	0,77	8,495	0,428	1,066	394,286	4,5	6,1824	4,643
8,141	0,539	1,075	0,551	1	445,714	1	5,5944	0,066

3,566	1,787	5,361	0,066	0	394,286	1	21,462	0,143
6,788	2,139	4,064	0,428	0,789	394,286	4,5	11,6508	0,932
7,649	2,493	6,367	0,428	4,5	180	4,5	0,8064	1,578
5,366	2,644	1,427	0,551	0	180	0	9,324	1,066
5,983	0,998	1,209	0,123	1	102,857	1	2,688	0,932
8,728	1,13	0,998	0,551	0	0	1	0	0,789
5,64	1,35	1,198	0,123	0	334,286	1	2,7972	4,643
7,229	1,075	1,93	0,246	1	180	4,5	2,7972	0,932
5,926	2,862	6,418	0,428	4,5	291,429	4,5	29,5512	0,143
3,052	0,264	2,987	0,066	6	300	0	0	0,495
5,935	2,359	2,352	0,123	4,566	154,286	1	58,7664	1,218
5,141	1,418	7,286	0,786	0	240	1	0	4,643
5,779	1,93	0,275	0,123	0	394,286	1	3,4944	0,143
6,792	1,644	1,209	0,066	0	257,143	0,429	29,6632	0,143
13,276	0,704	0,352	0,066	0,789	420	4,5	27,986	0,066
5,713	1,919	0,855	0,428	0	257,143	1	0,8064	1,789
14,791	0,275	0,418	0,428	0	111,429	1	37,4962	0,143
5,056	1,71	1,207	0,494	1	428,571	1	15,0612	0,143
1,979	1,143	12,215	1,066	0	205,714	1	0,8064	1,143
8,07	2,073	1,418	0,428	0,429	428,571	1	65,646	0,143
5,93	2,79	3,864	0,123	0	120	1	3,024	4,643
4,718	0,781	4,198	0,066	0	351,429	1	6,132	4,643
2,007	5,784	0,352	0,123	0	274,286	0	0	4,643
14,867	2,132	1,429	0,066	0	728,571	1	10,038	0,789
4,706	0,998	1,71	0,123	0	505,714	1	1,8396	0
6,577	1,853	3,429	0,786	0,789	385,714	1	1,5624	0,932
8,64	1,418	1,075	0,856	1	265,714	4,5	9,6768	0,789
13,18	0,275	1,921	0,066	0,143	197,143	1	2,1504	0,132
6,495	2,719	0,418	0,786	0	557,143	4,5	10,8108	0,789
7,152	0,209	6,075	0,551	0	188,571	1	0	1,789
6,726	1,864	4,352	0,123	0	565,714	4,5	0	4,643
5,938	0,847	1,284	1,214	1	514,286	0,789	59,3124	0,143
5,924	2,433	0,561	0,428	1	300	0	4,8384	0,932
6,43	3,073	7,93	0,786	4,5	342,857	0	3,4944	5,289
6,353	1,853	1,71	0,856	0	205,714	4,5	5,0232	0,143
6,293	1,207	3,341	0,428	1	300	1	18,396	0,143
3,706	1,064	1,207	0,066	4,5	334,286	0,066	4,9056	4,643
7,914	1,119	5,796	0,123	0	497,143	4,5	27,972	0,066
2,928	1,864	1,275	0,551	0	351,429	4,5	24,1332	0,143
4,834	0,418	1,418	1	4,5	240	1	32,6382	4,643
3,769	1,341	2,073	1	0	557,143	0,789	0	0,932
4,203	0,352	0,275	0,246	0,789	265,714	1	0	0,143
5,795	2,007	2,429	1,214	0	257,143	1	34,986	0,143
4,206	0,858	6,132	0,428	0	197,143	4,5	12,1212	0,789
4,052	1,196	0,198	0,428	0	548,571	0,429	6,7452	0,572
1,54	0,275	11,853	0,123	0	300	4,5	12,726	1,066
6,638	1,484	3,864	2,07	4,5	437,143	4,5	24,7212	0,143
19,498	0	1,066	0,428	0	180	1	5,5944	0,143

5,84	1,853	4,207	0,856	1	162,857	0	0,8064	0,789
4,222	1,787	2,855	0,428	0	77,1429	1	0	5,289
3,557	0,484	3,275	0,123	1	205,714	1	8,1942	0,855
7,081	1,864	8,51	0,856	0	120	4,5	10,038	1,789
5,206	0,275	0,132	0,123	4,5	0	4,5	16,6824	1,143
8,232	3,222	0,198	0,856	0	205,714	4,5	113,9684	1,218
11,08	2,15	4,13	0,428	4,5	342,857	0,066	0	0,286
4,537	1,207	0,209	0,428	1	351,429	4,5	66,3992	1,143
7,862	1,855	1,396	0,428	1	240	0,789	7,9716	5,289
6,365	0,572	3,433	0,428	1	231,429	4,5	0,8064	4,643
6,078	6,144	6,284	0,428	0	162,857	1	48,0592	0,789
3,995	2,642	6,921	0,428	1	162,857	1	20,5464	1,789
5,215	0,627	9,284	0,428	1,143	222,857	1	1,8396	4,643
5,059	2,433	4,284	0,428	0	240	1	54,6504	0,286
2,788	1,93	8,51	0,066	0,789	274,286	1	0	0,789
4,852	1,427	1,218	0,428	1	240	1	8,1732	0,143
4,291	2,499	3,275	0,428	0	137,143	1	0,8064	1,789
6,506	1,853	5,51	0,428	0	214,286	1	0	1,789
9,523	1,13	3,143	0,856	0	120	1	25,6564	0,789
7,075	1,133	5,504	0,428	0	377,143	1	23,324	0,143
5,498	0,715	0,998	0,246	1	98,5714	0	20,2902	0,789
5,993	4,907	1,352	0,856	4,5	360	4,5	8,3328	4,566
8,581	0,704	3,998	1,214	0	265,714	0,143	22,1592	0,932
3,623	0,998	3,141	0	0,789	300	1	12,6336	1,143
5,217	1,482	2,293	0,123	0	617,143	4,5	12,4698	1,143
14,218	1,93	3,141	1,856	1	462,857	1	7,4592	0,143
5,08	1,93	1,998	0,428	4,5	214,286	4,5	18,396	4,643
3,282	1,71	0	1,123	0	360	1	12,264	0,066
2,537	0,998	1,352	0,123	1	428,571	1	9,5172	0,209
2,996	1,427	7,998	0,428	0	120	1	5,376	4,643
6,726	0,616	4,51	0,428	4,566	120	0,143	0,8064	0,789
4,783	2,073	8,367	0,123	4,5	77,1429	0,789	0,8064	5,289
6,717	0,209	3	0,066	0	0	1	3,4944	4,643
6,08	1,284	0,286	0,428	0	282,857	1	13,993	0,789
4,157	2,216	1,075	0,428	0	274,286	1	0	1,789
5,075	1,644	3,921	0,123	0	471,429	1	0,8064	0,143
5,361	1,93	0,066	0,428	0	300	4,5	16,926	0
3,467	1,209	9,493	0,551	0,789	197,143	1	25,872	0,932
4,575	1,559	9,578	0,786	0	300	4,5	30,66	5,289
4,509	1,921	7,789	0,428	4,5	162,857	0	0	0,143
6,495	0,913	1,218	0,123	0,143	565,714	4,5	6,132	6,143
4,586	1,066	0,209	0,066	0	480	1	4,032	0
6,803	1,275	1,998	0,428	1	325,714	1	0	4,643
6,566	0	0,275	1	1	291,429	0,429	0	0,789
4,063	2,139	8,647	0,786	0,143	445,714	1	55,9524	4,643
5,63	2,207	2,064	0,428	0,855	240	4,5	16,1364	0,143
5,99	1,985	11,71	0,428	0,789	180	4,5	60,662	1,578
6,414	1,141	1,275	0,428	1,066	445,714	1	0,8064	1,143

2,994	1,207	6,141	0,123	0	300	0,143	2,331	0,932
12,126	2,719	5,209	0,428	0,789	282,857	0,789	94,6232	4,643
4,63	1,35	9,987	0,123	4,5	360	4,5	6,132	6,143
9,008	1,493	5,209	0	0,789	162,857	4,5	18,6592	0,789
4,915	0,921	7,13	0,428	0	565,714	4,5	12,264	6,143
4,282	0,561	0,495	0,428	0	385,714	4,5	6,132	0,066
11,495	1,075	6,143	0,428	0,789	274,286	4,5	15,8508	0,429
4,271	1,275	0,855	0,428	0	60	0,789	0,8064	0,789
6,438	1,93	0,561	0,428	0	308,571	0	26,1072	1,143
5,858	1,853	5,218	0,428	0	180	4,5	46,648	1,143
15,132	2,653	3,007	0,428	0	240	4,5	94,0982	1,578
4,638	2,216	1,141	0,123	0	377,143	0	333,3946	4,643
7,003	3,653	1,141	0,428	0,066	94,2857	1	23,5704	5,289
5,915	1,053	1,71	0,428	1	145,714	1	20,5464	5,289
7,021	1,864	1,352	0,428	4,643	154,286	1	21,462	1,578
8,956	4,8	0,932	0,428	0,429	420	4,5	24,528	1,789
3,64	5,641	9,352	0,123	0,789	197,143	0	0	4,643
4,06	2,216	3,352	0,428	1	360	1	0	1,143
7,649	5,553	7,987	0,786	0	180	4,5	3,066	0,209
5,783	1,275	2,352	0,428	1	265,714	1	16,3212	0,066
4,992	1,209	0,932	0,428	0	102,857	1	5,4852	0,286
4,442	4,786	0,198	0,123	1	411,429	4,5	8,5848	1,143
4,555	1,064	7,787	0,428	0	214,286	1	28,9044	1
4,647	1,578	3,932	0,428	1	197,143	1	156,0104	0,143
5,434	1,493	3,855	0,786	0	60	0	2,9064	0,932
5,28	0,495	1,066	0,246	1	60	1	180,012	0,209
9,055	1,339	3,776	0,674	0,066	342,857	4,5	6,3672	0,143
4,48	2,073	1,561	0,428	4,5	377,143	1	7,476	1,789
11,81	1,787	9,275	0,786	0	102,857	0,143	78,0052	0,143
7,661	1,341	3,427	0,428	0	248,571	1	1,8396	5,289
4,214	0,352	0,209	0	0	420	0,066	7,4592	0
9,225	1,075	1,644	0,786	0	317,143	1	14,1036	0,789
4,544	1,196	1,998	0,066	6,066	420	0	79,4472	4,643
5,133	1,13	0,998	0,123	0,789	582,857	4,5	23,5662	4,566
4,55	1,93	1,075	0,786	0	60	1	0	0,143
3,544	1,273	6,93	0,428	0	300	1	0	4,643
2,254	1,064	2,93	0,428	1	102,857	1	23,9904	4,643
7,447	0,473	3,855	0,312	1,066	120	4,5	151,2602	1,143
4,48	1,13	3,796	0,428	0,789	205,714	1	64,7136	0,855
15,018	1,919	1,93	0,428	0,429	411,429	1	19,3872	0,789
6,37	0,932	0,429	0,428	0,789	454,286	1	11,7432	0,143
4,418	0,932	1,429	0,123	1	428,571	0,143	2,7972	4,566
3,701	3,073	4,218	1	4,5	60	4,5	20,2356	6,789
4,632	1,427	3,644	0,428	0	205,714	0,789	39,326	4,566
5,215	1,504	2,141	0	4,5	120	1	18,396	0,066
3,412	1,064	6,721	0,856	4,5	197,143	1	11,5584	0,932
3,912	1,264	0,998	0,428	4,5	240	0	2,7972	0,066
3,772	2,644	4,433	0,428	0	377,143	1	0,8064	6,143

5,841	6,144	6,51	0,856	0	265,714	1	29,0556	5,289
6,504	1,985	2,264	0,786	0,143	300	1	25,1412	0,855
5,856	1,286	0	0,123	0	300	4,5	0	1,143
2,823	1,352	1,075	0,123	0	180	1	23,3772	4,643
5,98	0,198	3,066	1,494	1	137,143	0,789	0	0,143
3,786	2,776	1,075	0,123	0,789	600	4,5	25,102	0,789
5,053	0,638	4,218	0,428	4,5	334,286	4,5	3,1836	1,578
6,135	2,367	0,932	1	0,143	411,429	0	10,038	0,789
7,724	3,299	4,578	0,428	0	317,143	1	10,8948	1,578
6,141	6,43	3,361	0,123	1,143	291,429	1	0	1,578
9,923	1,418	2,284	0,551	0	162,857	1	28,1988	0,286
4,146	2,996	0,638	0,123	1,066	480	4,5	13,44	1,143
3,197	2,785	6,007	0,428	1	514,286	4,5	14,1036	0,143
7,429	2,631	7,273	0,428	4,566	137,143	1	8,9292	6,789
2,898	1,644	2,921	0,123	0	420	1	0	4,643
6,212	1,57	6,639	0,428	5,289	282,857	4,5	0	0,066
6,713	1,427	7,432	0,428	1	360	0,143	14,1722	0,932
3,335	1,35	1,427	0,856	4,5	514,286	4,5	382,8748	0,789
9,703	1,919	0,484	0,123	1	282,857	1	18,648	0,143
6,724	0,341	5,13	0,428	0	265,714	1	10,2564	0,932
7,934	1,218	0,132	0,428	0	265,714	1	4,9056	0
8,509	1,141	0,341	0,428	1	77,1429	1	10,0632	0,143
8,503	0,987	1,141	0,123	0,066	334,286	1	32,9196	0,932
5,715	2,987	0,921	0,428	1	197,143	FAŁSZ	3,6792	6,143
4,917	1,284	3,418	0,428	4,5	462,857	1	0	1,066
5,269	2,642	4,352	0,428	4,5	102,857	1	4,8384	5,289
5,86	1,361	4,284	0,428	1,789	197,143	0,429	9,324	4,643
6,433	1,853	2,499	1,214	0,066	162,857	4,5	7,9296	0,855
13,071	3,51	2,275	0,428	0,789	437,143	1	3,4944	1,143
6,214	2,075	4,429	0,123	0	437,143	4,5	49,2044	0,143
4,836	1,352	2,209	0,123	0	120	1	2,688	0,066
7,724	3,499	9,93	1	0	282,857	4,5	12,2304	6,789
5,286	1,776	0,264	0,856	0	411,429	1	27,972	0
4,829	2,207	1	0,786	1	137,143	4,5	0	4,643
4,854	2,073	0,572	0,123	0	377,143	0	2,9064	0,143
6,365	1,996	5,576	0,428	1,066	120	4,5	64,9096	4,643
3,194	2,433	9,361	0,428	6	248,571	4,5	24,528	0,855
3,632	2,719	5,141	0,066	1	334,286	1	2,1504	4,566
9,057	1,996	5,429	0,786	4,5	180	0	2,7972	0,789
5,797	2,073	5,427	1	1	205,714	4,5	53,9952	0,789
8,648	3,141	0,407	0,786	1	180	4,5	25,5612	4,643
8,809	1,998	6,209	1	1	257,143	0	12,9696	1,789
3,258	2,198	5,932	2,566	1	582,857	1	29,0556	0,855
3,981	0,998	0,209	0,428	4,5	360	1	3,6792	0
7,236	3,156	3,789	0,428	0	300	4,5	67,298	0,789
6,153	0,998	0,286	0,909	0	462,857	4,5	31,1556	4,643
11,705	1,053	1,855	0,786	0,789	480	1	37,3072	1,066
4,506	0,275	1,218	0,428	0	222,857	1	0	0,932

6,012	1,493	12,561	0,123	0	214,286	4,5	18,648	1,789
6,552	2,205	8,139	0,428	0	214,286	4,5	10,1052	0,495
4,713	1,141	3,864	0,123	1	162,857	1	5,418	1,143
5,585	0,209	3,504	0,428	1	377,143	4,5	50,3342	1,143
10,77	1,493	1,286	0,428	0	522,857	0	12,264	0
3,548	1,853	3,264	0,428	0,143	420	1	6,1824	5,289
5,038	2,576	4,418	0,428	0	248,571	4,5	4,5276	0,143
5,146	1,864	3,284	0,428	4,566	77,1429	1	0	0,143
7,801	2,576	3,367	0,428	0	274,286	1	0,8064	4,643
10,663	7,153	2,209	0,428	0	77,1429	4,5	0	5,289
8,077	2,853	1,932	1	4,5	222,857	1	1,8396	0,143
4,632	2,15	1,218	0,786	1,066	317,143	1	22,7892	5,289
5,346	4,231	6,495	0,066	0,066	480	0	11,662	0,789
8,128	1,339	3,286	0,856	0	94,2857	1	6,1824	1,789
9,306	1,787	2,932	0,428	1	248,571	1	30,8084	1,789
5,002	7,01	5,418	0,123	4,5	411,429	4,5	27,986	4,643
3,283	0,209	0	0,428	6	394,286	4,5	0	0
15,128	0,495	1,132	0,428	0	360	1	18,6564	0,143
3,917	1,207	2,932	0,428	4,5	300	4,5	2,688	0,209
3,653	2,776	7,998	0,428	1	574,286	1	23,3184	0,932
3,832	2,939	10,218	0	0,429	351,429	1	41,1908	0,572
4,858	0,638	0,561	0,428	1	300	0	55,2972	1,143
7,705	1,064	2,198	0,786	0,429	60	1	40,8632	0,143
2,469	0,715	2,787	0,066	0	428,571	1	2,688	6,143
5,933	3,565	8,787	1,428	0,789	360	6	27,972	0,143
1,625	1,776	9,341	0,123	0	291,429	4,5	3,4944	0,932
4,346	1,71	3,275	0,428	1	300	0,789	7,5936	1,143
6,135	0,55	3,264	0,674	0,066	128,571	0,143	20,9832	0,932
5,584	0,998	0	0,428	1,066	137,143	4,5	18,6564	0,789
2,53	1,416	8,064	0,786	0,066	240	1	8,442	6,789
4,842	0,275	0,209	0,428	0	360	1	5,754	0,143
3,983	0,789	0,855	0,123	1	282,857	1	2,688	0,209
4,939	2,51	3,218	0,428	0	180	1	2,688	6,143
8,912	0,924	1,064	0,123	1,143	154,286	4,5	26,7148	0,066
3,709	1,644	0,789	0	0	480	0	41,972	0,572
2,54	0,627	6,209	0,428	0,143	77,1429	4,5	0	0,932
5,335	1,853	1,407	0,428	0,132	197,143	0,066	26,1408	4,643
4,709	1,13	7,427	0,786	0	214,286	0,143	46,62	6,789
6,005	4,064	4,275	1	6	77,1429	1	2,688	5,5
4,995	2,15	1,064	1	0	197,143	4,5	8,8704	5,289
6,869	1,93	0,429	0,428	0	300	4,5	51,31	0,066
5,443	1,644	0,932	0,428	0	137,143	4,5	9,324	0
3,28	1,787	0,198	0,066	0	300	0,789	0	5,289
5,723	0,979	2,647	0,428	0	240	0,143	7,9716	1,578
6,276	0,693	2,275	1,572	0,789	351,429	0	28,4592	0,066
3,283	2,007	0,998	0,123	0	180	0,143	8,9292	0,066
5,357	2,15	2,427	0,123	1	291,429	4,5	19,0344	0,066
6,79	0,932	11,064	0,123	0	154,286	0	2,7972	0,143

5,075	1,427	4,275	0,428	0	137,143	1	0	4,643
2,837	0,836	1,361	0,428	0	437,143	1	13,1712	6,143
6,212	6,793	6,572	0,786	4,5	214,286	1	2,7972	4,643
8,579	1,71	3,286	0,428	1	120	1	0	0,143
5,493	0,998	0,998	0,123	4,5	120	1	2,7972	4,643
2,194	2,919	15,075	0,428	1	351,429	4,5	0	4,643
4,489	0,855	1,209	0,123	0	540	0	13,9944	0
5,704	1,921	7,51	0,428	0,066	531,429	4,5	24,7324	0,143
7,656	1,93	3,367	0,786	0,789	137,143	4,5	7,4592	4,566
5,64	1,141	2,998	0,428	1	411,429	0	30,9204	1,066
5,215	1,998	6,218	0,428	0,143	154,286	1	6,9972	1,143
14,43	5,85	10,355	0,786	0,143	197,143	1	5,5188	5,289
7,167	3,442	2,361	0,428	4,566	111,429	4,5	5,376	5,289
8,78	1,495	3,433	0,786	0	240	1	4,032	4,643
5,493	1,284	1,561	0,428	0	497,143	0,789	0	0,855
6,946	1,644	1,855	0,428	0	360	0,789	46,62	0,066
4,932	1,418	7,561	0,428	1	394,286	0,789	0	5,289
9,406	1,855	3,787	0,428	4,5	462,857	4,5	5,0232	0,143
8,164	0,998	2,644	0,428	0	240	1	4,662	0,143
4,497	0,429	1,987	0,066	0	240	4,5	6,7452	0,286
6,23	1,218	0,418	0,428	0	154,286	0,143	21,4452	0,932
2,779	0,638	6,064	0,066	1	445,714	1	0	4,643
1,309	0,836	8,264	0,066	4,5	480	1	38,066	4,643
4,972	2,073	8,784	0,856	0,066	265,714	4,5	16,3212	1,066
3,849	5,707	1,361	0,428	0	317,143	1	8,1732	0,932
15,155	1,57	8,853	0,428	0	180	4,5	6,7452	4,643
4,484	1,35	2,141	0,123	0	102,857	0,789	6,4176	5,289
5,203	0,987	3,855	0,123	0	51,4286	1	28,7784	4,566
1,757	1,075	0,429	0,428	4,5	351,429	0,789	23,9904	0,209
4,869	2,499	3,987	0,551	0	90	4,5	8,1564	0,932
6,713	1,35	0,352	1,189	0	162,857	1	26,1212	4,643
6,352	1,548	7,484	0,428	0,855	180	0,789	18,7656	1,578
4,84	2,642	5,352	0,246	4,566	137,143	4,5	3,4944	4,643
5,432	4,709	0,132	0,428	0,429	137,143	1	2,1504	1,789
5,632	2,576	1,286	0,551	1	394,286	0,789	0	1,143
18,565	1,636	4,429	1	0,429	34,2857	0	0	0,286
9,22	2,51	7,566	1,214	1	300	1	180,012	0,143
19,083	3,721	6,578	0,856	0	120	4,5	4,9056	1,789
4,201	1,361	7,998	0,066	4,5	394,286	0	2,688	4,643
9,907	1,35	0,352	0,428	0,066	120	4,5	33,1072	1,143
4,977	0	1,209	0,123	0	282,857	1	0,8064	0,143
5,871	2,713	11,864	0,428	0	240	0	9,324	4,643
5,566	1,93	0,704	0,786	1,066	420	1	7,9044	1,143
8,644	1,638	5,141	0,786	0	60	1	37,296	6,143
8,206	0,352	2,352	0	0,066	180	0	0,8064	1,066
5,415	0,847	2,064	0,428	0	137,143	1	9,7692	5,289
9,861	2,708	2,51	1,786	4,5	171,429	0,789	93,9764	4,643
16,082	1,141	2,998	1	1	240	1	9,8112	0,066

4,281	0,352	1,495	0,428	0,429	360	4,5	0,8064	0,932
2,902	1,198	0,209	0,123	1	205,714	0	2,688	0,286
2,977	2,576	6,066	0,132	0	420	0,143	0	0,789
2,177	0,495	6,504	0,428	0	634,286	0	3,4944	4,643
1,823	2,565	9,064	0,123	0	180	0	1,7388	5,289
5,063	1,93	6,51	0,428	1,066	334,286	4,5	106,624	1,143
5,727	1,207	3,341	0,123	0,209	540	1	54,53	0,143
5,498	1,13	1,721	0,428	0,789	180	0,066	6,132	1,578
8,664	2,719	4,576	0,786	0,932	120	4,5	12,8352	1,143
3,258	1,273	6,495	0,428	4,5	480	0	0	6,143
7,649	1,919	2,075	0,428	1	368,571	0,143	17,0352	0,066
3,309	5,775	1,578	0,786	1	368,571	4,5	36,3636	0,143
2,254	3,787	0,33	0,066	0	274,286	4,5	0	1,143
3,838	0,932	6,987	0,123	0	360	1	0	6,143
2,535	0,484	2,367	0,428	4,643	480	4,5	6,1824	0,932
8,721	1,996	2,198	0,428	0	240	1	3,4944	0,495
6,003	0,341	0,286	0,123	0	102,857	1	0	0,932
4,491	1,427	2,275	0,428	1,066	240	1	13,4652	0,789
5,291	2,576	0	0,856	1	428,571	1	8,9292	0
4,849	2,642	0,407	0,786	4,5	462,857	4,5	147,3332	0,143
14,728	1,35	4,156	0,428	0	60	4,5	57,974	0,789
5,65	0,495	1,075	0,066	0	428,571	1	14,9184	0,143
4,149	6,496	2,578	0,428	6	214,286	1	22,3272	5,289
6,656	1,933	2,264	0,786	0,066	197,143	4,5	27,4848	1,578
7,418	3,007	1,998	2	0,143	634,286	4,5	30,058	1,789
4,759	1,339	3,352	0,066	1	497,143	4,5	5,376	1,143
5,372	2,721	1,066	1	0	514,286	1	54,0036	0,143
6,724	2,293	7,218	2,5	0	162,857	4,5	21,0084	4,643
2,12	1,921	0,143	0,428	0	394,286	0	8,064	0,143
3,478	1,644	0,998	0,369	0	162,857	1	12,2052	0,066
6,223	1,996	0,143	0,428	0	282,857	4,5	13,86	0
4,346	0,418	2,143	0,428	1	582,857	4,5	7,9716	0,143
2,755	0,561	0,352	0,066	0	471,429	4,5	14,3472	0,789
4,006	2,576	7,361	0,428	0	154,286	1	0	4,643
4,715	1,196	3,998	0,428	1	368,571	0	74,634	0,932
10,351	1,141	0,209	0,428	4,5	505,714	1	0,2268	1,143
5,001	1,207	2,855	0,428	0	197,143	1	1,8396	1,143
3,838	2,721	1,855	0,123	0	368,571	4,5	7,5264	1,789
2,862	1,77	3,864	0,428	6	420	1	45,9732	1,218
2,48	1,075	3,275	0,428	4,566	317,143	0	5,376	0,143
6,926	1,93	2,075	0,428	0	111,429	0	10,038	0,143
8,717	1,781	6,644	2,5	0,789	180	1	15,6576	0,286
6,359	2,708	4,207	0,123	0,789	265,714	0,143	8,8704	1,143
3,928	0,855	3,075	0,123	1	120	1	2,7972	4,643
7,816	2,499	0,484	1	0	120	1	12,8352	0,143
5,715	4,907	1,71	0,428	0,789	334,286	1	8,6436	6,143
6,304	1,559	7,864	1	1,789	531,429	4,5	33,5664	5,289
6,999	2,919	4,775	0,428	0	240	4,5	27,972	4,643

3,335	1,647	4,341	0,066	4,5	360	0	81,634	0,789
1,735	1,196	7,207	0,856	0,789	531,429	4,5	35,6972	0,932
4,434	5,487	0,418	0,786	0,789	111,429	0,789	5,8716	4,566
7,668	2,15	2,932	0,428	0	274,286	0	5,5944	5,289
7,495	1,407	6,198	0,856	1	462,857	0,789	13,986	0,143
6,352	1,341	2,075	0,123	1	394,286	4,5	0,8064	0,143
3,957	0,495	2,13	0,428	1	291,429	1	5,376	4,643
4,572	3,005	0,55	0,551	0	411,429	4,5	3,6792	0,932
5,566	2,433	1,141	0,551	0	248,571	1	19,3872	5,289
2,812	5,487	0,132	0,428	1	428,571	1	26,1212	1,143
8,143	2,644	3,644	0,066	0	257,143	1	8,1732	0,572
4,43	2,51	4,209	0	0	68,5714	1	0	1,143
2,612	0,627	4,132	1	0	257,143	1	3,066	1,143
6,726	1,427	1,143	0,428	0	82,8571	4,5	0	4,643
3,838	2,561	6,638	0,428	0,132	531,429	4,5	10,4832	0,209
4,421	2,653	6,576	0,786	0	257,143	1	13,608	0,789
13,005	2,35	3,653	0,428	4,5	300	1	9,7902	1,578
5,792	1,427	4,352	0,428	4,643	240	0,789	44,7552	0,143
5,064	2,073	2,57	1,214	0,143	162,857	1	81,1608	0,066
4,336	1,779	2,286	0,066	1	360	4,5	8,8704	1,143
12,919	0,341	0,407	0,428	0	334,286	1	16,3212	0,932
8,596	1,93	3,007	0,428	0	300	4,5	24,528	0,429
2,77	0,066	1,143	0,123	0	317,143	6	0	0
3,566	1,998	1,427	1,786	1	300	1	11,1888	0,143
5,79	2,073	3,284	0,428	4,5	334,286	1	13,9944	0,143
5,636	1,132	3,286	0,123	1	180	1	4,1412	0,932
3,365	2,007	6,075	0,428	1	428,571	1	18,0012	4,643
8,871	0,429	2,864	0,246	4,5	411,429	1	50,6912	1,578
3,489	1,996	14,796	0,428	0	317,143	4,5	5,376	0,066
3,918	2,931	14,579	0,123	4,5	360	4,5	2,688	6,143
6,661	1,427	2,495	0,428	0	240	4,5	75,6896	5,289
4,803	1,93	8,853	0,123	5,289	428,571	4,5	27,6654	4,643
6,504	2,352	15,299	0,428	4,5	222,857	4,5	6,8544	0,932
9,286	1,064	5,141	0,066	1	257,143	1	4,6788	4,643
6,064	1,207	2,864	0,246	0,855	368,571	1	5,6448	0,143
5,005	1,996	1,141	0,123	0	317,143	1	17,0268	0,855
7,864	7,13	0,418	0,786	0,789	137,143	1	14,4592	1,578
7,135	1,198	5,578	0,428	4,5	180	1	5,8716	1,066
4,871	1,418	3,352	0,428	0	94,2857	1	14,6034	0,143
3,917	1,921	6,418	0,786	0	300	1	6,72	6,143
4,394	1,064	9,653	0,428	4,5	120	4,5	6,9384	4,566
1,768	1,198	0,921	0,428	0,286	325,714	1	8,064	0,143
4,847	2,565	5,064	0,428	0,066	428,571	1	13,4484	0,932
2,604	0,847	14,15	0,123	4,5	334,286	1	2,688	6,066
7,571	1,264	7,141	0,428	4,5	420	0	0,2268	5,289
1,605	0,198	1,275	0,428	0	514,286	4,5	28,7532	0,066
5,518	1,721	2,721	0,123	0	257,143	4,5	12,264	4,643
15,15	1,996	0,429	2,5	0	325,714	4,5	7,9716	0,143

7,06	0,561	9,141	0,428	4,643	137,143	0,789	0	4,643
8,363	0,572	0,143	0,123	0,789	377,143	4,5	15,1872	0
3,414	0,418	1,352	0,123	4,5	360	0	2,688	4,643
5,502	0,998	0	0,123	0	300	1	0	0
5,64	2,719	11,141	0,856	0,789	197,143	4,5	17,0352	0,143
4,429	0,987	0,066	0	0	257,143	1	24,7912	4,643
7,792	1,352	1,855	0,189	6	240	4,5	12,264	0
5,638	1,053	15,719	0,428	0	325,714	4,5	12,4684	1,143
4,701	4,995	1,713	1,428	0	394,286	4,5	0	6,143
6,144	1,416	11,207	0,428	0,855	240	4,5	6,9384	6,143
2,491	1,218	0,132	0,428	0	257,143	0,789	1,8396	1,578
5	1,787	10,429	0,786	1	325,714	4,5	9,6852	5,289
3,568	5,564	5,93	0,123	0	188,571	4,5	0	4,643
5,274	3,642	5,288	1	0,429	411,429	4,5	9,8616	1,218
4,49	1,702	0,987	0,066	1	351,429	0	9,0552	0,143
12,509	1,853	2,998	0,428	0	240	1	23,31	4,643
5,653	4,929	9,789	0,428	0	180	4,5	2,4192	6,789
4,915	2,13	7,355	0,428	1	565,714	6	42,1232	1,143
9,27	0,418	1,932	0,428	1	248,571	1	5,376	0,132
5,919	1,864	6,644	1	0	197,143	4,5	0,756	6,789
6,355	1,141	2,367	1	0	351,429	4,5	11,6592	0,143
1,603	2,433	2,275	0,066	0	514,286	0	0	6,143
4,773	1,853	4,011	0,428	0	180	0,143	2,1	5,289
10,468	1,721	1,064	0,428	0,066	154,286	0,789	1,512	0,789
2,829	0,561	4,064	0,066	0,066	514,286	1	13,818	6,066
5,001	2,073	2,218	0,428	0	274,286	1	0	5,289
5,508	2,565	1,207	0,189	1	437,143	0,066	1,4784	4,643
1,768	0,352	10,921	0,066	4,5	480	4,5	4,662	0,789
6,434	1,143	0,132	0,428	0	77,1429	4,5	0	0,066
6,147	2,073	6,141	0,428	1,143	222,857	1	8,064	1,143
2,414	1,647	6,264	0,428	0	180	4,5	8,064	5,289
2,403	2,433	13,218	0,123	4,5	240	1	0	4,643
4,851	0,341	7,286	0,428	6	120	4,5	30,66	6,066
3,495	1,209	0,066	0,123	1	171,429	4,5	67,298	0,143
6,724	1,919	10,499	0,494	0	120	4,5	3,4944	1,143
4,774	1,864	1,932	0,786	0,429	120	1	10,9704	1,218
4,579	2,644	10,088	0,786	0	317,143	4,5	1,344	4,643
2,92	1,13	12,998	0,786	1	197,143	4,5	0,8064	5,289
5,84	0,855	1,132	0,123	0	60	4,5	0,756	0,572
3,489	1,075	8,99	0,123	0	505,714	4,5	8,064	4,643
10,218	0,855	0,855	0,551	1	351,429	0	7,0182	0,143
2,928	1,064	0,275	0,123	0,789	214,286	0	0,8064	0,572
4,195	0,998	5,141	0,428	0	171,429	0	20,9832	5,289
9,136	4,698	0,198	1,214	1	222,857	4,5	23,31	0,066
11,653	0,998	3,79	0,428	1	300	1	2,4192	5,289
4,772	4,852	6,209	0,428	0,789	257,143	1	0,8064	4,643
3,208	0,77	4,433	0,123	4,5	145,714	4,5	4,8384	0,286
6,213	1,218	4,143	0,428	0	317,143	0,789	7,2156	0,286

5,627	2,576	0,495	0	0,066	222,857	1	8,8704	0,855
9,283	0,932	2,064	0,428	0,789	428,571	0,789	0	0,789
5,481	1,787	1,644	0,856	0,789	120	0,066	4,0572	0,066
3,994	5,498	9,209	0,428	0	334,286	4,5	0,8064	4,643
5,201	1,864	6,416	0,428	1	377,143	0	15,6366	5,289
6,793	2,565	10,576	0,428	0	257,143	4,5	2,7972	1,143
2,986	0,998	2,932	0,428	0	180	1	12,2304	1,578
6,867	1,207	4,561	0,428	0,066	94,2857	4,5	46,648	0
7,574	0,561	2,921	0,428	0	411,429	1	2,7972	1,789
5,724	7,142	2,407	0,428	0,855	385,714	1	42,1232	0,932
8,44	5,852	3,721	0,428	4,5	120	4,5	10,2564	5,289
2,92	0,55	6,141	0,428	0	180	4,5	13,0872	0,572
9,857	0,275	4,207	1	0	360	4,5	5,5944	0,143
8,012	1,644	0,495	0,856	0	162,857	0,066	66,0044	0,789
5,491	2,218	0,932	0,428	0	180	4,5	10,1304	0,143
6,082	0,847	4,132	0,428	4,5	240	1	3,4944	0,143
5,651	1,141	2,787	0,123	1	385,714	0	16,506	0,143
5,917	1,919	2,341	0,428	4,5	428,571	1	6,9972	0,143
3,337	1,196	3,578	0,428	0	394,286	4,5	0,8064	0,143
2,691	0,352	0,209	0,123	1	394,286	1	0,8064	4,566
6,372	1,864	2,407	0,123	4,5	445,714	4,5	23,3772	0,789
7,794	3,51	17,789	0,428	0	300	4,5	24,668	5,289
8,146	1,207	7,064	0,428	0,143	180	1	12,4782	1,789
5,424	1,418	1,644	0,856	0,066	180	1	37,9008	0,143
5,287	2,15	3,207	0,428	0,789	197,143	0,789	36,1032	0,143
5,507	1,57	6,275	0,786	0,429	240	1	34,5912	4,566
5,869	5,641	3,77	0,428	0,143	360	4,5	15,1872	5,289
7,229	1,141	1,286	1,066	4,5	17,1429	4,5	2,688	1,143
5,92	1,996	2,35	1	0	90	4,5	0	0,932
5,915	1,209	1,284	0,428	0	368,571	6	12,6336	0,789
6,598	0,858	2,198	0,428	0,143	240	1	108,0072	1,578
2,78	1,504	0,693	0,123	0	531,429	0,789	9,9036	0,143
6,011	3,864	1,932	0,428	1	205,714	1	1,6128	5,289
3,335	2,073	5,921	0,123	5,289	274,286	0	0	6,789
3,775	0,352	0,341	0,428	0,143	325,714	0	240,016	0,066
4,014	2,284	9,71	0,428	1	600	4,5	5,376	5,289
4,961	0,407	1,561	0,428	0,789	154,286	0,789	30,1224	1,143
4,563	1,644	2,361	0,428	0,789	120	4,5	28,7532	0,143
6,151	1,855	0,209	0,428	0	300	0,429	0	0,143
7,17	1,864	2,722	0,428	1	445,714	1	30,1224	0,789
6,293	1,064	0,418	0,494	0	102,857	1	10,794	0,143
8,793	2,286	2,998	0,428	1	180	4,5	6,72	1,143
6,84	0,495	0,932	0,428	0	145,714	4,5	2,7972	1,143
16,431	1,352	11,275	0,428	1	274,286	1	53,9952	5,289
6,772	2,853	2,141	0,123	1,066	600	4,5	87,2984	1,066
7,216	2,418	1,932	1	4,5	120	1	0	5,289
3,769	0,55	1,493	0,428	6	531,429	1	35,7084	6,143
7,062	2,433	1,776	0,066	0,143	428,571	0,143	3,6792	1,789

5,933	2,642	1,93	0,786	1	300	1	8,8704	0
6,447	2,642	2,787	0,786	0,855	240	1	12,264	4,5
8,087	1,636	0,572	1,123	1	137,143	1	30,9288	0,066
5,482	3,719	3,141	0,428	1	197,143	4,5	27,972	6,789
1,748	4,984	9,432	0,428	0,066	300	4,5	4,8384	1,143
6,43	0,932	0,143	0,786	6	360	1	0	6,143
6,568	1,93	3,998	0,428	0	274,286	0,789	14,4144	4,643
6,924	1,416	3,284	0,551	0	480	1	10,1808	0,143
5,269	0,638	1,998	0,428	0	394,286	1	0	0,143
3,713	1,284	6,796	0,123	1,143	274,286	1	8,9292	5,289
5,515	0	2,066	2,5	0	334,286	4,5	8,064	0
10,132	2,565	7,561	2,5	1,066	617,143	4,5	6,2664	5,289
5,346	1,218	1,053	0,428	0,789	300	1	5,4684	1
6,003	0,264	0,998	0,428	1	120	1	0	1,143
4,352	2,075	1,064	0,428	0	77,1429	0	0	0,143
4,7	1,919	0,572	0,428	0	240	0,789	2,688	0,066
9,099	1,932	0,789	0,428	0	282,857	1	0	5,289
5,141	1,427	1,264	0,428	0,789	368,571	1	57,974	6,143
3,691	0,495	5,207	0,123	0,066	137,143	0	49,3724	6,066
7,084	1,721	4,352	0,123	0	462,857	1	9,324	0,143
4,986	4,852	6,367	1,786	0,066	342,857	4,5	0,672	0,143
7,729	1,787	0,286	0,428	0,789	45,7143	1	0	1,789
7,935	0,132	1,429	0,123	0	445,714	4,5	168	0,789
6,656	1,196	1,996	0,428	0	325,714	1	27,3252	0,932
8,642	1,35	4,275	0,123	0,429	214,286	1	6,132	5,289
8,699	1,625	5,355	0,786	1	120	4,5	12,8352	0,789
7,794	1,352	1,93	0,428	1	257,143	4,5	0	0,066
14,22	1,284	2,066	1,428	0	60	1	0	1,429
4,28	5,553	5,198	0,428	1	308,571	1	0	5,289
4,486	0,143	1,352	0,123	0	240	1	8,1732	1,066
7,937	1,71	5,275	0,428	0,789	171,429	1	2,7972	0,066
5,004	4,852	0,924	0,123	1	300	4,5	15,9432	0,932
6,421	6,144	7,864	0,786	1	240	4,5	6,1824	0,143
6,785	1,495	1,141	0,066	0	514,286	1	13,986	4,643
2,274	1,35	2,286	0,428	1	317,143	1	5,4852	0,143
10,705	1,853	1,578	1	0	111,429	1	4,4184	0,066
5,124	2,141	0,572	0,428	0	394,286	1	2,9064	1,143
4,911	1,064	9,132	0,123	4,5	137,143	0	10,1472	1,143
6,058	1,787	2,578	0,123	0	291,429	4,5	6,9972	0,789
7,572	2,93	4,156	0,428	0,789	180	1	0,8064	0,932
5,647	2,719	21,207	0,428	1,143	257,143	0	89,992	4,643
6,517	0,855	2,932	0,551	0	180	1	20,0088	0,932
7,139	2,218	1,209	0,428	0	188,571	1	18,6564	0,143
10,231	2,51	2,075	1	1	77,1429	0	2,7972	5,289
8,025	3,945	3,218	0,428	0,789	300	1	37,296	5,289
22,294	3,288	2,43	0,852	6	48,5714	0	0	1,789
3,634	1,504	6,864	0,132	0	531,429	1	23,926	4,643
5,223	0,418	3,284	0,428	0	180	1	8,1732	4,643

6,785	1,141	13,141	0,428	0	197,143	4,5	69,972	1,143
3,065	0,998	0,341	0,428	0	591,429	1	18,396	0,066
13,93	5,564	4,862	0,246	0,143	222,857	1	6,1824	0,286
3,917	0,473	0,143	0,123	1	265,714	1	1,5624	4,643
6,293	2,064	8,864	0,428	1,066	428,571	4,5	23,31	1,789
6,706	0,998	5,066	0,428	0	454,286	4,5	26,7372	0
3,425	1,864	7,93	0,551	0,789	240	1	14,0042	1,578
3,834	2,205	1,075	1	0	497,143	0	9,324	0,932
5,717	1,273	2,141	0,428	0	240	0	12,264	4,643
14,085	1,43	1,143	0,786	0	42,8571	4,5	0	0,429
5,363	1,853	2,286	0,428	0	120	1	6,5604	5,289
4,994	1,919	4,207	0,428	0,789	531,429	0,789	20,5464	0,932
4,28	4,775	3,064	0,123	0,066	402,857	0	13,0704	1,143
4,993	1,13	0,998	0,551	0,143	428,571	1	7,2156	0,066
4,217	1,416	4,932	0,551	1	180	4,5	23,324	0
6,284	1,561	1,141	0,428	4,566	325,714	4,5	27,3084	0,789
4,061	1,559	7,57	0,786	0,066	420	1	0	5,289
9,514	1,218	1,789	0,428	1	154,286	4,5	0	1,578
2,988	0,858	0,987	0,066	0	137,143	0,143	1,344	0,789
1,911	0,572	10,407	0,066	0	282,857	0	4,5276	6,789
17,631	4,852	5,504	0,428	0	240	4,5	111,1852	4,643
3,635	1,198	2,132	0,428	0	240	4,5	2,7972	4,566
5,643	2,348	2,998	1,214	0,066	257,143	1	4,662	0,143
6,063	0,924	5,998	0,551	4,5	325,714	4,5	11,6592	4,643
8,658	1,284	7,286	0,428	1,066	265,714	1	44,3044	1,789
11,052	1,13	1,141	1,428	4,5	120	1	5,5944	1
4,638	2,282	2,578	0,856	4,5	411,429	1	57,288	1,789
5,412	2,196	4,998	0,428	0	531,429	4,5	33,726	1,143
7,579	1,985	3,198	0,428	2	300	1	9,2484	1,143
10,655	1,853	0,429	0,428	0	180	4,5	2,688	1,789
7,137	1,93	2,787	0,428	1	334,286	4,5	3,066	4,643
2,759	0,715	1,721	1,428	0	300	4,5	6,2748	4,566
3,56	1,853	4,57	0,123	0	171,429	1	5,4684	4,643
5,869	2,062	6,064	0,428	0	197,143	1	2,7972	0,143
5,577	2,144	2,143	0,123	0	55,7143	1	49,9884	1,789
5,781	3,431	4,064	0,428	0,789	180	4,5	46,648	1,789
5,511	2,642	1,864	0,123	0,429	120	1	24,528	1,578
4,28	1,275	3,998	0,123	1	540	4,5	49,336	0,855
12,214	0,855	3,352	0,123	1	488,571	0	0	0,789
20,779	2,502	5,007	0,428	0,789	162,857	1	0	0,855
4,706	0,789	2,855	0,246	1	377,143	4,5	0,8064	1,143
4,634	1,789	2,855	0,428	1	60	0	0,8064	0,932
8,428	6,364	0	0,428	0	394,286	4,5	1,344	0
5,148	1,143	4,209	0,428	0	0	4,5	2,688	1,143
5,861	2,073	3,776	0,786	0,209	94,2857	0,789	14,8932	0,143
1,68	1,064	0,495	0,428	0	222,857	4,5	1,344	1,066
5,348	0,275	1,064	0,551	0	351,429	4,5	13,9944	0,143
4,204	1,207	2,578	0,428	0,789	300	1	6,1824	4,643

7,647	1,921	0,998	0,066	0	514,286	4,5	16,3212	0,932
4,061	2,075	10,644	0	0,789	154,286	1	0	5,289
2,271	3,299	0,198	0,786	1,218	360	6	41,3252	0
3,854	1,141	1,198	0,066	0,429	514,286	0,143	24,528	1,578
7,352	2,919	3,352	0,428	0	291,429	1	18,396	0,066
2,395	0,998	18,845	0,123	0	317,143	4,5	23,5536	6,066
6,572	1,578	0,143	0,428	1	531,429	1	11,662	0,143
4,649	1,141	2,998	0,123	1	137,143	1	9,324	6,143
7,774	1,339	1,921	0,189	0	60	1	16,926	0,143
5,777	3,156	9,578	0,428	0	402,857	4,5	14,4592	0,143
5,783	1,341	1,209	0,428	0,789	137,143	1	6,72	4,643
2,045	1,93	4,132	0,428	1,066	394,286	0	0	5,289
3,92	2,062	2,638	0,123	1	480	1	3,8724	4,643
4,566	1,638	0,998	0,123	4,5	497,143	4,5	70,4802	4,566
6,43	1,787	4,352	0,428	0	111,429	1	11,6928	0,286
4,054	1,998	3,776	1,214	0,429	222,857	1	0	1,066
12,927	5,289	0,998	0,428	4,5	80	1	0	1,218
5,355	0,418	2,998	0,428	0,143	351,429	4,5	88,6312	0,789
5,732	1,644	3,721	0,428	1	411,429	1	8,1564	0,143
5,935	1,13	8,418	0,123	4,566	488,571	4,5	7,2156	5,289
3,57	0,572	5,066	0,428	1	214,286	1	1,512	1,143
4,412	2,13	11,061	0,786	0,066	300	1	5,5944	0,572
6,293	2,708	3,787	0,856	4,5	291,429	1	18,396	5,289
3,555	0,987	6,998	1	4,5	197,143	4,5	27,972	1,143
7,508	0,932	2,644	0,123	6,789	171,429	4,5	0,567	0,066
8,807	1,864	3,721	0,428	0,789	402,857	4,5	30,002	0,066
9,728	1,429	0,209	0,428	4,5	540	1	0,8064	0,143
4,065	1,075	2,064	0,123	0	471,429	4,5	1,5624	1,143
16,023	0,473	2,141	1,123	0	171,429	1	4,5276	0,932
1,713	1,35	6,578	0,428	0,143	77,1429	4,5	2,688	5,289
5,794	1,273	7,141	0,123	0	222,857	1	5,376	0,143
6,73	1,787	5,286	0,428	4,5	240	0,789	5,4852	1,578
1,911	1,93	0,932	0,428	1,578	394,286	0	36,3132	1,578
8,814	2,499	3,578	0,428	0	240	4,5	27,972	5,289
2,472	1,075	4,064	0,123	4,5	300	0	0	6,143
6,306	1,141	8,704	0,123	1	214,286	4,5	32,7992	0,066
2,683	0,998	0,352	0,428	0,143	300	1	2,7972	0
5,794	1,284	2,275	0,428	4,5	62,1429	1	0	5,289
4,058	4,995	0,987	0,909	0	197,143	4,5	15,0612	0,066
2,271	1,495	4,132	0,123	0,143	214,286	1	2,1504	4,643
2,853	1,207	1,209	1	0	102,857	4,5	5,0778	4,643
2,051	0,341	8,132	0,066	0,143	462,857	4,5	10,038	4,566
3,634	0,484	2,932	0,428	0	120	1	0	0,932
1,133	0,198	1,35	0,428	0,066	325,714	4,5	13,993	4,643
3,489	0,781	5,13	0,786	0	154,286	4,5	5,376	0,143
6,704	2,853	2,578	0,786	4,5	154,286	4,5	0	5,289
3,196	2,499	4,075	0,428	0	651,429	1	5,5944	0
4,366	2,064	2,572	0,428	1,143	240	1	0	0,932

5,922	2,721	5,007	0,786	0	428,571	4,5	7,3626	1,218
4,483	0,638	2,207	0,428	0,789	394,286	1	48,65	0,286
5,645	0,198	3,998	0,856	4,5	222,857	1	0	0
3,192	2,073	24,995	0,428	0	651,429	1	7,35	0,932
5,577	1,273	2,484	0,856	1	308,571	0	0,8064	1,143
4,341	1,207	1,787	0,428	0,789	197,143	0	12,1212	0,143
11,15	2,359	3,924	0,428	1	94,2857	1	36,1844	1,578
5,212	2,15	9,57	1,428	1	445,714	1	46,62	0,143
2,425	1,284	5,198	0,066	1	300	1	6,132	4,643
3,483	2,71	4,284	0,786	4,5	600	4,5	7,9716	4,643
3,511	3,299	1,143	0,428	0,429	222,857	0	0	0,572
4,638	2,928	7,427	0,066	4,566	248,571	1	6,72	6,143
3,206	1,721	4,361	0,856	0,789	411,429	4,5	56,9044	4,643
9,637	6,221	0,209	2,566	0	120	4,5	46,648	0
5,784	1,353	3,064	0,786	0	197,143	4,5	8,3916	1,578
12,937	1,218	8,221	0,066	0	120	4,5	17,8752	5,289
10,213	2,642	3,361	0,428	0,143	60	1	0	4,566
6,791	1,416	4,143	0,246	1	334,286	0	20,9832	0,932
12,72	1,636	5,786	0,428	4,5	411,429	0	8,6282	5,289
3,478	2,433	2,776	0,123	1	240	0,429	3,1374	1,578
4,849	1,075	1,075	0,428	0	300	1	7,4592	0,143
7,668	2,51	3,51	0,428	0	180	1	6,9972	5,289
4,996	0,143	0,209	0,123	0	180	1	2,7972	0,143
7,003	1,352	4,721	0,428	4,5	197,143	1	0	0,789
5,79	1,427	1,352	0,066	0	531,429	4,5	2,688	0,286
1,331	0,987	1,207	0,123	1	351,429	1	3,1836	5,289
4,929	1,721	0,132	0,123	0	385,714	1	2,688	0
4,069	0,781	4,853	0	1	360	0,429	0,8064	5,289
6,926	1,275	1,561	0,428	0,789	377,143	4,5	34,9272	1,578
3,653	0,143	0	0,123	6	471,429	1	0	0,143
4,792	0,473	4,576	0,428	4,5	120	1	6,5016	1,143
5,275	1,429	1,644	0,189	0	377,143	1	7,9716	1,578
4,709	1,419	10,576	0,786	1,066	180	4,5	39,634	5,289
5,29	2,284	2,286	0,551	0,789	248,571	1	6,9972	1,143
8,382	2,636	3,361	0,428	1,066	240	1	1,8396	1,789
5,366	1,933	6,572	0,066	4,5	51,4286	0,143	0	0,932
4,836	1,996	0,275	1	4,5	120	0	25,4772	0,143
5,566	2,719	4,945	0,428	4,5	300	1	0	0,143
14,629	1,13	1,286	0,123	1	180	1	7,2156	0,066
3,632	2,565	9,864	0,428	0	222,857	4,5	0	0,789
7,504	2,13	1,721	0,428	0	231,429	1	12,264	0,789
4,126	1,644	3,198	0,551	0	72,8571	4,5	27,972	4,643
7,497	1,93	10,352	0,428	0,429	120	0	0	0,789
6,717	0	1,855	1	1	300	1	42,7224	0,789
4,761	1,196	5,13	0,428	0	480	1	7,5264	4,643
4,508	0,998	1,644	1	0	248,571	0	0	0,066
6,719	3,007	1,855	1	0	334,286	1	5,376	5,289
5,99	2,565	3,638	0,428	0,143	154,286	1	14,1036	1,143

4,357	2,499	6,198	0,428	0,066	480	1	9,5424	0,209
2,928	1,218	7,578	0,428	0	540	4,5	0	5,289
4,566	1,284	4,429	0,428	0,855	171,429	4,5	26,2542	0,143
3,071	2,141	4,066	0,428	0,143	282,857	4,5	0	6,789
7,479	1,495	4,275	0,428	0	197,143	4,5	32,7992	6,143
6,081	1,407	19,35	1,284	0	411,429	1	12,1212	0,789
8,143	4,995	4,198	0,066	0	197,143	4,5	0,8064	4,566
5,572	1,787	5,93	0,428	0	180	4,5	0	1,143
6,414	1,141	4,644	1,214	4,5	480	1	12,8772	0,066
6,007	2,218	1,495	0,428	0	240	0	10,038	0
4,139	0,352	1,352	0,123	4,5	180	1	3,4944	4,643
3,64	3,431	14,141	0,786	0,789	240	4,5	0	5,289
7,137	2,565	8,075	0,428	4,5	291,429	1	10,2732	1,143
3,901	2,282	2,053	0,856	0	360	4,5	10,752	0,143
7,914	1,998	6,51	0,428	0,429	222,857	1	4,5276	1,143
2,813	1,493	1,284	0,066	4,5	240	0,429	0	1,789
11,44	5,432	9,644	0,786	4,5	257,143	4,5	7,9716	5,289
8,504	1,35	0,998	0,856	0	282,857	0	2,7972	0,932
7,076	2,653	3,864	0,428	4,5	360	4,5	34,5296	1,578
5,287	1,427	6,352	0,856	0	420	0,066	10,1808	0,932
3,629	0,998	3,789	0,428	0	154,286	0	0	1,143
4,506	5,424	4,275	0,428	0,855	317,143	1	8,064	1,143
10,062	1,842	2,132	0,428	0	214,286	1	59,0884	4,643
6,652	1,71	5,007	0,428	1	308,571	4,5	34,9692	1,578
5,052	0,77	1,71	0,428	0	548,571	1	7,7112	0,143
3,118	0,638	0,998	0,066	1	514,286	0	23,058	1,143
4,135	1,286	1,721	0,786	0	342,857	1	8,2824	4,643
6,047	1,919	6,719	0,428	0,789	120	1	6,5604	1,143
4,772	0,627	2,132	0,551	0	222,857	1	66,0044	0,855
5,081	2,576	3,367	0,428	0	120	4,5	67,6396	0,789
5,429	2,15	16,561	0,428	0	445,714	1	2,1504	1,578
5,287	2,787	1,578	0,428	0	240	1	0	1,218
5,7	1,075	1,064	0,428	0,143	480	1	3,6792	0
1,911	1,207	4,932	0,428	1	385,714	4,5	0	0,789
4,482	0,341	0,921	0,123	0	137,143	1	0,8064	0,066
7,225	1,787	7,284	0,428	0,066	180	1	2,7972	1,578
12,354	3,921	2	0,428	1,066	445,714	1	78,0052	1,218
6,148	2,642	5,064	0,428	0	197,143	1	32,3904	1,143
3,991	2,141	3,13	1,214	0	214,286	1	57,974	1,789
8,664	0,484	1,132	0,123	1	180	1	4,662	0,932
2,917	0,275	0,132	0,066	4,5	600	4,5	3,6792	4,566
6,786	2,71	3,361	0,428	0	514,286	1	29,6632	6,789
11,437	0,638	4,566	0,428	0	377,143	0	79,4584	0,855
3,632	1,71	2,407	0,428	4,5	171,429	1	7,4592	0,066
4,214	2,51	0,066	0,123	1,429	600	4,5	4,3008	0,143
4,546	6,353	10,433	0,786	0,066	257,143	1	47,292	5,289
5,508	1,855	2,721	0,123	0	94,2857	0,789	0	1,143
11,149	2,787	1,572	0,428	0	565,714	4,5	0,2268	1,789

9,22	2,284	1,141	0,428	1	351,429	4,5	120,008	1,789
8,944	1,143	1,075	0,428	0	411,429	1	4,5276	4,643
5,124	6,364	6,286	1	0,143	300	1	18,1272	1,143
5,144	2,721	0,209	1,214	0,429	214,286	1	7,4592	4,643
2,491	1,284	0,198	0,123	4,566	531,429	4,5	56,6664	5,289
3,115	0,998	2,352	0,123	1	428,571	4,5	1,512	4,643
3,544	1,919	2,064	0,123	4,5	274,286	1	10,752	4,643
5,768	0,341	7,647	0,428	0,066	120	1	17,038	4,643
4,005	0,987	5,141	0,123	1	274,286	1	0	0,572
4,415	1,218	1,578	0,428	0	222,857	4,5	151,606	0,932
6,788	1,064	0,789	0,428	0	360	0,429	270,018	0,143
4,297	0,132	1,855	0,123	0	77,1429	0	3,4944	5,289
5,269	1,482	9,998	0,856	4,5	300	1	1,1844	1,143
7,86	1,55	8,141	0,428	0	94,2857	1	7,4592	4,643
5,346	1,352	0,066	0,428	0	171,429	0	3,1836	0,066
7,155	2,862	4,638	0,123	0	257,143	0	18,102	4,643
5,126	0,55	1,93	0,428	1	488,571	4,5	14,4144	0,932
6,579	0,998	1,207	0,428	0	180	4,5	12,8352	1,143
5,269	1,644	0,572	0,428	0,789	308,571	0,143	2,7972	0,572
7,658	0,781	6,429	0,909	0,789	240	1	24,6652	0,066
6,713	1,908	5,864	0,428	6	411,429	0,066	36,3972	0,066
6,137	1,427	1,284	0,428	0,143	300	4,5	15,0612	0,143
2,073	0	6,289	0,123	0	428,571	1	0	0
8,848	0,77	8,638	0,428	4,566	248,571	1	7,5264	4,643
6,003	1,996	10,721	1,428	4,5	171,429	4,5	30,7692	0,789
5,935	0,998	2,275	0,428	0	38,5714	1	2,1504	4,643
6,86	2,642	1,286	0,786	1	120	1	0,8064	0,143
7,636	1,855	5,075	1	0	154,286	1	0	0,066
5,504	5,861	8,987	0,428	6,143	197,143	4,5	23,31	5,289
3,623	0,693	0,998	0,428	0,932	265,714	1	8,022	0,932
13,431	0,264	3,787	0,189	1	154,286	1	53,7964	0,789
6,876	1,141	3,368	0,428	0	248,571	1	87,9732	0,789
3,777	0,913	4,132	0,428	0	120	4,5	1,8396	0,066
9,152	2,51	5,578	0,856	0,066	180	1	36,0024	0,789
5,138	1,275	0,275	0,856	0	437,143	1	9,5172	0,789
7,44	2,075	2,51	1	4,5	34,2857	4,5	45,7212	1,578
5,203	0	6,341	0,428	0,789	240	4,5	27,5772	0,789
10,426	1,919	7,075	0,428	1,066	360	1	191,674	4,643
3,36	0,484	5,939	0,123	0	197,143	1	11,662	1,578
4,729	1,056	0,495	0,123	0	180	1	28,2072	0,932
2,55	0,341	1,275	0,123	1	497,143	1	0	4,566
5,57	1,064	8,064	0,428	0,789	171,429	4,5	43,1032	0,143
9,075	1,578	2,352	0,551	0	514,286	0,429	4,1412	0,932
4,487	2,776	1,71	0,428	4,5	600	4,5	48,0032	0,143
1,493	1,787	10,264	0,066	0,066	480	4,5	5,376	4,643
7,592	2,939	7,361	0,428	0	360	4,5	1,8396	5,289
3,769	1,504	6,15	2,5	0	77,1429	1	0	4,643
4,071	2,433	0,484	1	0,143	120	4,5	15,5904	0,143

7,436	1,721	6,721	0,551	6	428,571	4,5	0	0,143
5,229	0,77	6,284	0,123	4,5	385,714	0,429	20,2216	0,066
4,852	1,119	3,913	0,786	0,066	300	1	30,7272	0,855
9,062	1,196	0,352	0,428	1	240	0	3,4944	0,143
3,64	4,632	2,209	0,123	0	471,429	1	5,376	4,643
9,447	1,864	0,998	0,428	1	240	4,5	5,4684	1,789
9,798	5,564	2,932	0,428	4,5	325,714	1	7,35	5,289
8,637	1,141	0,132	0,428	1	102,857	1	3,066	1,143
8,45	1,856	2,275	0,428	1	120	1	0	5,289
5,926	1,218	4,286	0,551	0	154,286	6	18,0012	0,789
3,922	0,932	0	0	0	0	4,5	5,754	0,066
7,649	1,853	0,198	0,428	4,5	120	0,789	2,7972	0,143
8,579	2,062	3,787	0,123	0	180	1	0	1,066
7,081	2,708	5,299	0,428	4,566	411,429	4,5	68,775	1,578
4,432	1,418	7,855	0,428	4,5	437,143	1	3,6792	4,643
1,573	0,341	3,855	1	1	402,857	0	0	0
4,071	1,919	7,075	0,428	1	120	0	0	0,572
3,783	1,418	6,932	0,123	0,789	214,286	4,5	9,6768	4,643
4,629	1,919	1,429	0	0	94,2857	0,789	24,2536	1,789
5,496	1,207	0,561	0,123	0	514,286	1	38,066	0,066
4,706	1,218	2,286	0,123	1	437,143	4,5	0,8064	0,143
3,926	1,207	0,495	0,123	0,066	325,714	4,5	0,8064	0,066
10,571	0,286	4,998	0,123	0	137,143	4,5	60,004	0,143
4,28	1,35	4,484	0,428	0	557,143	1	24,5112	5,289
2,406	2,653	7,196	0,066	0	240	1	15,0612	1,578
9,735	0,429	0,638	0,428	0	137,143	1	1,8396	0,143
6,926	1,996	2,141	1	0	514,286	1	0,756	1,143
4,911	0,407	4,484	0,428	0	180	0,143	4,8384	5,289
3,778	0,572	5,585	0,428	0	137,143	4,5	8,064	0,572
5,99	2,708	0,275	0,428	1,066	137,143	1	13,608	0,789
2,26	0,704	6,341	0,123	0	351,429	0,066	48,454	4,643
7,436	0,209	0,932	0,428	4,5	145,714	1	1,8396	0,789
6,146	2,642	22,932	0,856	4,5	180	FAŁSZ	0	6,066
4,403	0,341	1,132	0,189	0	317,143	4,5	17,794	0,066
9,932	1,636	3,789	0,428	0,789	428,571	4,5	1,344	0,143
8,007	2,499	4,653	0,428	0	94,2857	1	24,5112	1,143
4,68	0,855	1,209	0,428	1	497,143	4,5	33,3312	4,643
4,709	1,776	1,787	0,428	1	514,286	4,5	23,324	6,789
2,568	1,075	9,075	0,428	0,789	240	4,5	5,5944	5,289
5,623	1,776	1,001	1,428	0	171,429	4,5	27,5394	0,789
4,641	0,781	6,075	0,786	0,429	411,429	0,143	31,6708	6,143
4,071	1,787	1,853	0,428	1	248,571	4,5	37,296	0,789
4,269	1,196	6,066	0	1,066	197,143	4,5	16,926	1,143
6,293	2,062	3,218	0,428	0,066	454,286	4,5	2,7972	1,789
4,572	1,842	2,789	0,428	0,066	300	4,5	0	0,143
6,144	2,064	5,352	0,494	0	420	4,5	56,6412	0,143
3,851	1,644	3,987	0	0	428,571	4,5	6,5604	0,855
6,864	2,642	12,15	0,428	0	265,714	4,5	5,376	6,789

8,115	0,693	2,352	0,123	0,066	240	4,5	1,8396	0,066
7,903	0,352	2,209	0,066	4,5	222,857	4,5	0	0,066
3,206	1,853	4,13	0,428	0,066	180	1	0	0,143
12,39	2,51	2,064	0,123	0	300	4,5	0	1,578
4,258	1,493	3,987	0,428	0,429	394,286	1	13,0704	0,066
4,717	2,433	0,341	0,189	1	300	0,789	0,8064	4,643
6,07	1,13	3,495	0,428	0,789	197,143	4,5	8,1942	0,932
6,376	1,71	2,504	0,428	0	180	1	2,7972	0,932
9,933	0,341	2,939	0,428	0	222,857	4,5	23,0664	0,286
9,866	1,842	5,644	0,428	0	180	4,5	30,6964	6,789
3,984	1,996	1,35	0,123	1	462,857	1	76,622	0,143
4,566	1,361	4,073	1	0	205,714	0	11,13	4,643
13,142	2,073	1,572	1,428	0,143	437,143	4,5	33,4404	0,789
3,624	0,198	5,841	0,123	0	351,429	0,143	63,8484	0,066
10,408	0,264	0	0,786	0	437,143	4,5	46,648	0,143
14,297	0,209	0,429	0,786	1	377,143	1	2,1504	0,143
5,133	1,864	2,205	0,066	0,429	377,143	0	1,8396	0,066
5,206	0,847	2,998	1	0	420	1	2,688	0,143
3,131	1,361	3,578	0,786	0,143	274,286	4,5	0	0,932
5,298	2,721	4,918	0,786	0	257,143	1	12,1212	0,932
10,855	3,776	3,156	0,428	0,429	120	4,5	0	1,789
4,48	1,141	1,352	0,551	0	205,714	4,5	0,8064	4,643
6,86	1,064	1,921	0,123	0,789	368,571	4,5	19,2024	0,143
3,205	0,341	0,209	0,123	0	282,857	1	12,264	0,143
4,625	2,642	1,275	1,214	0,066	240	4,5	1,8396	0,789
3,566	1,853	8,864	0,428	0	222,857	1	19,1142	1,143
7,482	1,987	5,367	0,428	0	154,286	4,5	0	0,932
3,472	1,13	6,847	0,428	0	137,143	1	2,7972	0,932
4,564	2,565	5,075	0,123	0,429	394,286	1	4,8384	6,789
3,359	1,864	3,286	0,066	0	531,429	4,5	0	0,143
3,183	0,855	0,286	0,123	0	265,714	4,5	3,4944	0,789
7,082	1,57	2,638	0,428	0,429	497,143	1	2,7972	0,143
5,502	1,35	5,064	1,428	0	222,857	4,5	0	0,789
12,851	0,987	0,352	0,428	0,789	120	4,5	25,6564	0,143
7,525	2,367	3,495	0,428	0	162,857	1	1,8396	5,289
5,917	0,998	7,932	0,428	0	360	0	2,7972	0,789
5,223	1,207	3,352	0,428	0,143	197,143	1	12,1212	1,143
6,146	1,209	2,132	0,123	0	582,857	1	25,3512	0,209
5,43	1,273	0,418	0,428	4,5	137,143	1	20,4876	4,643
5,441	2,073	3,787	0,428	0	240	4,5	0	1,218
6,925	1,064	0,209	0,428	0	142,857	4,5	0	0,789
9,68	0,198	5,787	0,428	0	162,857	1	97,902	0
4,502	1,141	6,51	0,428	1	411,429	1	10,2816	1,789
6,471	1,996	2,198	1,284	4,5	180	4,5	48,398	0,789
4,792	1,493	2,638	0,246	0,789	128,571	4,5	27,3252	0,932
6,521	2,51	0	0	0	342,857	1	38,64	0
3,827	5,498	8,721	1,214	0	334,286	4,5	5,0232	0,789
4,551	3,576	6,576	1,572	4,566	514,286	4,5	8,1732	1,143

10,077	0,704	1,921	0,856	0	180	1	64,1102	0,932
5,276	0,715	2,275	0,428	0	428,571	4,5	47,4264	0
5,212	2,708	7,073	0,786	1	480	1	20,9328	4,643
6,545	1,053	1,132	0,856	1	351,429	1	7,4592	0,066
6,223	2,576	0,264	0,428	0,066	351,429	1	13,9944	0,066
4,572	1,207	5,143	0	0,066	180	4,5	20,1264	0,932
2,986	1,482	4,13	1	0,066	197,143	4,5	2,1504	6,789
3,871	1,864	3,721	0,786	0,429	282,857	1	6,9972	0,572
3,851	2,062	0,132	0,786	0,429	488,571	0	0	0,789
6,871	2,932	4,578	0,428	0	377,143	4,5	0	1,789
2,917	1,207	1,141	0,428	0	480	4,5	33,5664	0,066
1,869	2,578	2,361	0,428	4,5	317,143	1	48,0032	1,066
9,631	2,921	0	0,428	4,5	411,429	1	13,5912	1,143
4,421	1,284	7,064	0,428	1	154,286	4,5	33,166	0,143
5,421	1,064	6,284	1	0,789	480	0,429	19,726	0,143
5,412	2,644	5,493	0,786	0,429	257,143	1	19,2024	5,289
3,075	6	0	0,066	0	351,429	0	1,6128	0
8,866	1,075	0,932	0,428	4,5	325,714	0	0	0,932
4,348	1,207	3,35	2,5	0,429	171,429	4,5	8,3328	0,789
4,517	2,073	5,064	0,428	0	274,286	0,429	7,4592	0,789
8,923	0,539	1,053	0,856	0,066	120	4,5	8,8704	0,789
2,614	0,921	4,064	0,123	4,5	617,143	4,5	53,9952	0,066
4,915	2,653	2,572	0,428	1	257,143	1	6,9972	5,289
6,003	2,565	2,57	0,123	4,566	394,286	1	7,5936	0,932
5,289	1,559	1,855	0,428	1	282,857	1	5,8716	1,066
8,841	1,921	3,789	0,428	0	480	4,5	2,7972	0,066
7,425	2,719	2,864	1	0,066	137,143	4,5	42,63	4,566
4,64	0,932	3,71	0,428	0	248,571	1	37,296	1,578
4,326	1,998	5,13	0,428	4,5	394,286	0,789	1,344	4,566
3,269	2,293	6,924	0,428	5,289	342,857	4,5	1,8396	1,218
4,484	2,293	9,427	0,786	4,5	274,286	0,789	0	4,643
4,983	2,284	7,427	0,428	4,5	317,143	0,429	8,2824	5,289
7,652	3,156	5,787	0,246	0,429	411,429	1	0	1,218
4,337	0,921	1,064	0,428	0	102,857	1	6,132	0,209
5,291	1,275	0,066	1,284	0	120	0	0,8064	0,143
7,706	1,341	1,921	1	0	180	1	23,058	1,789
5,374	1,721	3,855	0,428	0	257,143	4,5	0	5,289
5,142	1,864	1,064	0,786	0,789	231,429	1	8,8452	1,143
6,998	2,433	0,286	0,123	0	222,857	0	0,2268	0,143
5,356	2,789	3,429	0,856	0,066	411,429	4,5	69,972	1,218
5,348	1,13	0,275	0,428	0,789	437,143	4,5	10,0464	0,143
5,862	2,93	3,71	0,856	0	120	1	6,9384	1,143
10,639	1,644	3,787	1	1	77,1429	1	1,344	0,066
4,141	0,418	6,064	0,428	1	377,143	1	21,4452	4,643
6,513	1,141	3,924	0	0	265,714	0	17,8584	5,289
4,13	0,789	0,143	0,123	0	162,857	1	6,1824	0
6,216	1,495	2,429	0,856	4,5	68,5714	1	28,7532	1
6,421	0	1,075	0,428	1	154,286	1	0	0,143

4,284	1,493	2,209	0,066	0	137,143	4,5	6,1824	0,066
8,785	1,284	0,987	1	0,789	265,714	1	48,9804	0,932
3,216	1,218	4,789	0,123	4,5	68,5714	1	12,264	4,566
3,984	0,55	1,932	0,428	0,789	437,143	4,5	14,259	0,143
7,518	2,713	1,504	0,123	0	214,286	1	18,396	1,143
3,28	1,207	3,721	0,428	4,5	385,714	4,5	6,132	5,289
11,14	1,998	1,853	0,856	0	325,714	1	2,9568	0,143
5,566	5,498	3,864	0,428	4,5	120	1	0	5,289
4,355	1,418	2,921	0,066	1	600	4,5	4,1412	0,143
9,306	2,053	1,855	1,214	0	162,857	4,5	2,688	5,289
7,665	4,011	5,929	0,786	0	342,857	4,5	40,9752	4,643
2,774	1,578	6,433	0,428	0,789	291,429	0,066	1,1844	6,066
7,706	2,425	1,275	0,856	0,429	214,286	1	18,0264	1,066
4,355	2,073	1,996	0,428	0,789	300	1	9,408	4,643
5,294	1,284	11,075	0,786	6	334,286	1	0	6,789
7,73	2,433	2,35	0,856	0,789	214,286	1	0,8064	1,578
4,994	1,996	3,495	1	0	240	0,789	2,7972	6,789
4,497	1,853	5,759	0,123	4,5	334,286	0	0	6,143
4,621	1,636	1,787	0,428	1	480	0,789	35,4312	4,643
4,709	1,473	3,064	0,428	0,789	248,571	1	10,9704	0,572
1,273	1,644	2,352	0,123	0,789	300	0,789	2,1504	0,932
3,197	1,572	4,222	0,123	6	180	0,789	4,8384	6,789
7,3	2,721	3,418	0,428	4,643	137,143	1	6,132	4,643
7,628	2,998	11,273	0,428	4,566	231,429	0,789	10,3992	0,495
4,058	1,35	5,853	0,428	0,066	411,429	1	6,8544	1,218
2,282	1,721	0,143	0,123	1	77,1429	4,5	2,688	4,643
5,718	7,722	8,779	1	0,066	274,286	4,5	0,8064	6,143
5,55	1,13	0,341	0,979	0	162,857	1	18,7684	0,066
4,212	1,919	5,578	0,428	0,429	257,143	1	0	0,143
4,709	1,638	2,932	0,786	0	488,571	0,789	0	0,789
9,108	4,8	3,644	0,786	0	197,143	0,789	13,44	0,789
4,546	0,998	0,429	0,428	0	497,143	1	4,5276	0,066
13,777	1,919	0,275	0,123	0	334,286	1	81,634	0,789
7,313	2,785	0,715	0,123	0,858	162,857	4,5	72,2764	4,643
6,286	3,225	100,284	1	4,5	300	1	0	5,289
5,809	1,787	1,143	0,428	1	94,2857	6	0	0,789
7,357	1,721	0,198	1,284	4,5	334,286	1	14,3808	5,289
8,667	2,433	6,218	0,428	0	240	1	4,662	4,643
3,071	1,275	1,352	0,123	0,789	317,143	1	5,376	4,643
3,702	1,143	3,209	0,123	1	265,714	4,5	4,1412	0,855
6,338	1,71	0,789	0,428	0	300	4,5	11,8776	4,643
4,992	1,987	10,572	0,123	0,932	171,429	0	10,8612	0,066
5,64	0,855	0,209	0,066	0	282,857	1	4,9056	0,066
4,711	0,418	3,132	0,428	0	171,429	0,066	37,709	1,789
4,857	1,286	5,143	0,123	4,5	222,857	4,5	0	6,143
3,906	0,561	9,209	0,494	1,066	265,714	4,5	8,9292	5,289
6,283	6,21	0,572	0,428	0	471,429	1	281,8396	6,143
3,986	1,787	1,209	0,428	0,789	497,143	1	17,8584	0

1,834	1,064	0,143	0,123	0	77,1429	1	5,376	0,286
5,076	3,499	10,495	0,428	0	240	4,5	34,9104	5,289
6,407	1,921	5,998	0,428	4,5	385,714	1	15,0612	5,289
5,977	0,418	2,198	1	4,5	180	0	7,4592	0,143
6,003	2,15	1,218	0,428	0	257,143	4,5	40,5804	0,143
10,943	0,418	5,499	0,551	4,5	300	1	3,4944	4,566
5,7	1,207	6,998	0,428	0	334,286	0	3,1836	0,789
4,603	0,987	1,855	0,428	4,5	651,429	1	5,8716	0,572
9,266	3,293	6,787	0,428	4,5	240	0	4,578	4,566
6,58	2,51	2,79	0,428	0	540	4,5	12,264	5,289
5,054	0,561	1,853	0,786	0,066	274,286	1	0	0,286
9,297	0,638	1,855	1	0	120	1	8,442	1,789
13,947	2,642	1,855	0,428	0	180	4,5	2,9568	0
5,629	2,776	8,572	0,428	0	197,143	4,5	94,6232	4,643
13,285	2,35	4,429	1	0	325,714	4,5	2,7972	1,789
7,708	3,576	3,075	0,123	0	437,143	1	0,8064	1,218
5,224	3,431	1,644	0,428	1	360	1	54,3984	4,643
5,209	1,842	2,132	0,856	0	154,286	0,789	0	1,143
5,478	3,222	2,578	0,428	4,5	197,143	1	10,0632	0,932
3,86	1,644	0,066	0,428	0,789	497,143	0,789	8,2824	0
9,578	1,93	4,064	0,428	1	257,143	0	0	0,572
5,419	1,985	0,341	0,786	0	471,429	4,5	18,0012	0,143
5,295	0,429	1,853	0,066	4,5	445,714	0	19,8352	6,143
1,842	1,853	5,209	0,189	0,066	308,571	1	4,032	0,932
12,438	2,576	8,275	1	1	368,571	1	3,6792	0,143
2,348	1,919	5,352	0,856	0	265,714	0	3,4944	0,789
5,57	1,273	3,862	0,428	6	325,714	1	26,3844	0,789
5,772	1,864	0,998	0,428	0	222,857	1	4,9056	0,143
3,109	1,275	1,93	0,856	0,789	248,571	1	1,344	1,066
4,146	2,367	1,578	0,786	6	171,429	0	0	0,789
9,514	4,566	0,286	0,428	0	317,143	1	0	0,789
14,139	0,066	1	1	1	180	1	300,02	0
3,772	0,781	9,207	0,123	1	420	1	6,72	1,218
4,549	5,564	1,721	0,786	0,429	394,286	1	0	5,289
8,372	2,719	4,796	0,852	0	291,429	4,5	41,7452	0,143
9,58	2,13	2,578	0,428	4,5	214,286	1	2,688	1,789
3,863	1,853	2,787	0,428	0,789	35,7143	1	0	1,789
4,337	6,33	0,275	2,5	0	300	1	251,678	0,066
3,703	1,71	1,71	0,786	0	197,143	1	7,4592	0,495
7,838	0,286	3,275	0,428	0	480	1	18,0012	0,143
4,415	5,861	6,721	0,428	1	120	1	6,1824	1,789
12,636	2,064	9,641	0,428	0	514,286	1	0	0,789
2,917	0,352	15,143	0,428	0,789	420	4,5	37,296	0,132
3,098	0	1,064	0,312	0,789	205,714	1	140	0,143
7,506	0,198	1,932	0,428	4,5	394,286	4,5	7,35	0,789
5,86	2,642	2,721	0,428	0,066	137,143	1	36,134	1,789
3,849	2,499	1,286	0,369	0	240	0	0,378	0,066
6,062	1,578	3,209	0,428	0	480	4,5	4,032	0,143

5,001	0,99	3,075	0,428	0	488,571	4,5	22,4112	5,289
4,069	1,864	4,286	0,123	1	480	1	38,6442	0,932
9,297	1,484	3,789	0,856	0	420	4,5	14,2548	0,143
5,768	0,286	8,078	1	0	240	1	0	0,209
5,005	2,15	0,275	0,066	4,5	197,143	1	0	0,932
4,988	0,495	2,644	0,551	4,5	240	4,5	18,6592	1,143
5,147	0,921	0,932	0,979	0	282,857	4,5	9,2484	0,143
3,646	3,365	4,644	1	0,066	411,429	4,5	0,8064	4,643
6,363	0,998	2,064	0,428	0	102,857	4,5	7,476	4,643
4,118	0,495	0,998	0,551	0,789	548,571	0	2,4192	1,143
3,192	1,13	7,007	0,428	0,789	428,571	1	10,0632	0,572
6,552	2,007	1,721	0,428	1	68,5714	4,5	24,2424	0
2,68	1,93	8,286	0,428	0,143	531,429	4,5	12,264	1,789
4,779	1,132	1,721	0,551	0	137,143	1	6,132	0,143
3,348	1,284	8,495	0,066	4,5	394,286	1	18,648	4,643
5,92	1,141	1,143	1	0	240	1	4,6788	1,143
3,28	1,578	6,715	0,428	0	497,143	0,143	7,3584	0,495
4,192	2,433	7,284	0,428	0	240	1	6,132	0,143
3,974	1,143	3,209	1	1	600	0	19,6224	4,643
3,331	1,416	7,284	1,284	4,5	497,143	4,5	88,2896	1,143
5,783	0,638	0,209	0,123	0	60	0	13,5912	4,643
3,992	1,13	3,924	0,428	0	214,286	1	4,9056	4,643
3,269	0,987	2,998	0,428	4,643	257,143	4,5	34,3392	4,643
13,946	0,998	1,853	0,428	0	291,429	0	6,3672	0,143
7,161	2,642	5,218	0,428	0,789	437,143	1	18,0012	5,289
7,209	2,282	5,264	0,428	1,066	222,857	0	30,828	4,643
6,427	1,276	2,286	1	0	360	1	27,972	0,066
7,262	2,919	9,998	0,856	4,5	480	4,5	21,4452	6,789
7,018	5,641	1,207	0,428	4,5	394,286	0,143	30,3072	1,578
9,866	1,273	0,495	1,066	0	514,286	4,5	17,8584	0,066
7,742	1,721	3,644	0,428	1,143	137,143	0,429	0	4,643
9,855	5,498	9,575	1	1,066	257,143	4,5	10,2564	6,789
5,135	1,578	6,275	0,786	4,5	668,571	1	1,6128	4,643
7,236	2,719	16,429	0,856	0	240	4,5	4,662	1,578
5,93	1,721	0,066	0,428	0,789	0	0	0,8064	0,143
2,414	1,493	9,864	0,428	0,066	317,143	4,5	0,378	4,643
6,849	1,644	2,007	1	0	197,143	1	3,4944	1,789
5,559	2,864	3,275	0,123	4,5	360	1	12,2052	0,932
3,672	1,998	1,064	1,428	0,789	514,286	0	23,31	4,643
1,636	1,93	0,066	0,123	0	325,714	1	0	0,143
2,994	2,007	1,352	0	0	480	4,5	10,2732	0,066
11,14	1,919	3,789	3,356	0,143	265,714	4,5	138,0092	0,143
3,832	1,644	1,064	0,123	4,5	300	0,143	3,4944	4,643
8	1,644	7,407	0,856	0	120	4,5	4,662	1,789
4,28	1,787	4,721	0,428	4,5	334,286	1	18,0012	5,289
5,946	2,708	2,141	0,428	1	137,143	1	3,8724	4,643
8,345	1,864	1,855	0,428	0,143	377,143	1	38,2844	0,066
3,148	0,352	5,787	0	0	154,286	0	13,8768	0,789

5,075	2,436	3,218	0,428	0	274,286	1	0	4,643
6,199	1,209	3,141	0,428	0	300	4,5	0,8064	0,429
3,205	1,644	7,721	0,123	4,5	334,286	4,5	8,064	6,789
4,207	0,209	0,932	0,123	1	497,143	1	5,6616	0,066
5,418	1,273	1,207	0,428	0	154,286	4,5	35,3388	4,643
4,26	1,209	2,721	0,123	0	137,143	1	0,8064	0,143
9,855	1,787	2,218	0,428	4,5	480	0	6,9972	0,789
4,71	3,945	3,864	0,066	4,5	257,143	1	0	0,789
4,033	5,061	2,073	1,214	0,429	137,143	4,5	15,8088	0,789
3,544	4,852	12,995	1,572	0	282,857	4,5	22,848	0,066
3,986	1,075	1,787	0,428	0	291,429	1	42,1134	0,143
9,455	2,576	3,578	0,856	0	60	1	0	1,143
4,926	2,35	0,209	0,066	6	360	0,143	0	0,066
11,283	1,853	0,209	0,786	0	265,714	1	21,1932	0,286
2,546	7,01	2,561	0,123	0,209	420	1	0	5,289
3,642	0,638	9,284	0,123	0	197,143	1	0	4,643
5,937	1,644	2,51	0,428	1	154,286	0	14,728	5,289
7,447	1,93	3,51	1,214	0,789	308,571	4,5	4,032	1,578
4,643	1,141	3,638	0,246	1	497,143	4,5	1,8396	0,143
4,405	1,143	0,132	0,123	0	120	0	3,4944	0,066
8,917	1,407	6,198	0,428	0	505,714	4,5	22,3356	5,289
5,203	1,919	12,576	1	0	120	4,5	6,132	0,789
9,789	0,209	2,789	1	0	582,857	1	19,5804	0,066
5,067	2,284	3,789	0,123	0,855	325,714	1	8,064	4,643
9,438	3,433	9,218	1,428	6	120	0	18,648	5,289
6,495	1,416	5,284	0,428	0,143	197,143	4,5	1,8396	1,143
7,197	0,066	1,578	1	0	231,429	0	0	0,066
3,766	2,653	3,638	0,428	0	411,429	1	21,462	0,789
4,634	1,787	4,007	0,428	0,143	162,857	4,5	56,3696	0,286
7,801	2,642	4,352	0,494	0,789	180	4,5	80,7884	4,643
6,429	2,653	4,13	0,428	0	454,286	1	12,1212	1,143
5,062	4,929	0,143	0,132	0	282,857	4,5	9,408	0
5,566	1,053	0,132	1	0	514,286	0,066	93,296	0,789
14,438	0,759	12,638	1,214	1	565,714	1	25,8552	0,572
7,447	1,218	3,853	0,123	1	77,1429	4,5	33,1072	1,143
5,229	0,484	7,064	0,856	1	205,714	1	0	0,066
5,72	4,786	2,216	0,786	1	171,429	0,143	7,8876	5,289
6,724	1,787	1,647	0,428	4,5	257,143	4,5	74,8776	0,789
4,641	0,209	0,987	1,123	0,429	120	1	3,6792	0,066
5,144	2,15	3,93	0,066	1	420	1	20,7984	0,789
7,509	5,289	2,789	0,428	0	282,857	0,789	18,648	0,789
5,55	1,996	3,361	1,428	0,066	154,286	0,789	0	0,789
4,357	1,776	1,075	0,786	1	360	0	0	0,495
6,156	2,864	0,066	1,428	1	377,143	1	5,376	1,789
4,489	1,418	6,156	0,786	1	180	0,143	0	0,143
6,645	0,932	1,286	0,123	0	214,286	1	4,662	4,643
6,071	1,71	4,504	0,066	0	197,143	4,5	7,5642	1,578
8,939	1,578	1,284	0,123	0	35	0,789	0	0

6,293	2,499	4,945	0,428	0,789	325,714	1	0,567	1,578
3,541	0,341	2,15	1	0	265,714	0,429	22,4322	0,066
5,506	2,15	6,781	1	0,789	257,143	0	0	5,289
5,661	1,919	2,216	0,786	0,789	480	0,066	21,4452	0,286
9,8	2,565	0,998	1,428	1	505,714	4,5	10,752	0,855
9,221	0,998	0,484	0,786	0	120	4,5	8,1312	0,143
6,726	2,218	4,79	0,428	4,5	291,429	4,5	21,714	5,289
4,071	2,499	2,789	0,428	0	120	1	0	0,143
2,634	1,207	8,275	0,428	4,5	137,143	4,5	12,5328	1,578
3,416	1,066	1	0	0	0	1	13,8348	4,566
8,492	1,919	2,286	0,428	0	282,857	1	30,9204	0,143
8,486	5,927	7,198	1,428	0	308,571	4,5	22,0752	4,643
6,871	1,71	5,275	0,856	0,789	454,286	4,5	0	4,643
7,66	1,064	0,418	1	1	351,429	1	93,408	0,066
7,214	0,704	0,198	0,428	1	248,571	1	3,6792	0,789
4,069	0,341	2,998	0,551	1	497,143	0,066	0	0,286
5,715	0,352	1,066	0	0	222,857	1	46,13	0,572
4,803	1,647	5,143	0,494	1	197,143	4,5	30,66	0,932
4,14	2,284	9,998	0,428	0	180	0,789	0,8064	0,143
11,994	6,138	3,436	0,428	0,143	300	1	18,0012	6,429
3,911	1,067	0,429	0,428	0	411,429	1	0,8064	1,143
5,195	1,987	1,572	0,246	1	582,857	4,5	13,9944	0,143
9,651	1,93	1,644	0,428	0,429	120	1	0	0,143
4,706	3,653	4,853	0,066	1	300	1	3,6624	5,289
3,768	6,287	2,787	0,428	1	360	1	17,038	1,789
4,722	2,218	3	1	0,789	93,5714	0	0	1,218
4,643	1,853	4,275	0,428	0	257,143	1	40,0932	0,143
4,568	1,207	10,007	0,428	0	360	1	8,1564	4,643
4,278	0	2,075	0,428	1	531,429	0	29,5848	1,578
1,471	1,864	5,075	0,428	0	137,143	1	23,6712	4,643
6,229	3,585	2,427	0,428	0	240	1	18,396	1,578
8,705	2,284	15,564	0,856	0	214,286	4,5	0,8064	5,289
4,993	1,644	5,198	0,123	0,789	334,286	1	0	0,789
5,19	1,721	4,644	0,979	4,5	360	0,143	19,3872	1,789
4,854	6,43	1,075	0,428	0	437,143	1	42,266	0,143
6,946	2,642	4,853	1	0,789	197,143	1	8,064	4,643
4,871	0,473	0,286	0,428	0,286	325,714	4,5	22,3776	0,066
5,495	2,007	7,218	0,428	1	77,1429	1	0,8064	4,643
3,849	0,352	9,418	0,428	4,5	120	4,5	26,2584	0,143
4,99	1,853	1,578	0,428	4,566	257,143	1	9,324	1,578
4,636	1,93	1,352	0,428	0	205,714	1	2,1504	0,066
5,724	0,627	5,13	0,786	1	205,714	0	0	1,066
4,489	2,436	5,576	1	4,5	497,143	4,5	35,7924	5,289
5,575	1,427	0,495	0,428	0	222,857	1	87,2284	0,932
4,78	6,353	3,638	0,786	1	394,286	4,5	16,7832	1,789
5,146	2,073	3,341	0,428	0,066	240	4,5	10,9704	1,578
4,715	2,708	3,853	0,123	4,5	557,143	1	10,2144	4,643
3,797	2,653	10,495	0,428	0	497,143	0	218,337	4,643

4,698	2,789	0,132	0,786	6	428,571	1	5,376	6
3,715	0,143	1,143	0	0	137,143	0	0	0,143
3,623	4,775	3,35	0,856	0,789	214,286	4,5	6,9384	0,143
5,495	0,924	0,143	1,428	0	471,429	4,5	7,9716	0,066
4,922	1,141	1,207	0,428	0	428,571	0	0	5,289
8,286	1,985	3,853	0,428	1,066	34,2857	1	5,4852	1,143
7,284	0	13,932	0,551	1	377,143	4,5	3,4944	1,578
6,852	3,493	1,776	1	0,132	385,714	0	0,8064	6,789
3,434	1,075	6,207	0,786	0	162,857	1	7,4592	0,932
5,924	2,708	6,141	0,428	1	197,143	1	0	6,789
4,851	2,864	8,218	0,428	1	265,714	4,5	5,376	1,143
4,843	4,367	6,789	0,428	0	180	0	27,972	5,289
4,907	1,207	2,565	0,786	1	394,286	1	24,5112	0,572
11,795	1,273	3,998	1,428	4,5	582,857	4,5	105,3136	0,789
5,994	1,064	1,286	1	1	282,857	0,789	6,132	0,789
4,621	1,787	0,998	0,856	0,429	462,857	0	9,0888	0,789
4,77	6,144	0,264	1	0	300	0	0	5,289
11,548	1,908	0,132	0,123	0	360	0,789	0	0
6,289	1,218	3,075	0,428	0	111,429	1	0,756	1,218
6,932	2,576	9,718	0,428	0,932	214,286	4,5	13,44	1,066
7,638	1,361	0,209	0,123	0,066	214,286	0	0	4,643
8,428	1,787	0,341	0,066	0	197,143	0,789	18,648	4,643
3,775	0,627	2,264	0,428	0,789	351,429	1	27,9006	0,932
3,48	1,339	0,352	0,428	4,5	394,286	1	9,3156	0,066
4,225	6,867	6,638	0,786	0	325,714	0	5,376	5,289
4,946	1,075	3,998	0,428	4,5	325,714	0,429	0	0,143
7,862	1,053	1,341	0,066	0	394,286	1	2,688	0,066
2,628	1,493	0,987	0,123	1	317,143	1	0	5,289
3,915	1,713	1,998	0,856	4,5	582,857	1	5,8716	6,789
5,867	1,996	2,721	0,123	4,5	120	0,789	6,1824	4,643
5,364	2,064	0,143	0,428	0	154,286	1	13,986	1,143
8,485	1,273	2,341	0	0,143	38,5714	1	4,9056	5,289
5,355	2,075	13,787	0,428	0	497,143	4,5	5,5944	1,789
6,158	1,578	2,429	0,428	0	274,286	1	24,528	1,143
6,807	0,921	1,987	0,428	1	360	4,5	13,986	1,143
5,572	0,932	5,864	0,428	4,5	394,286	1	8,064	4,643
6,455	0,998	0,921	0,428	0	79,2857	0,789	4,662	4,643
5,496	2,576	1,721	0,428	0	222,857	1	41,4736	1,789
6,735	1,361	5,939	0,123	0	102,857	4,5	0	1,218
7,016	2,207	4,352	0,428	4,5	600	1	35,5964	4,643
3,625	1,853	3,207	2,5	0,429	300	0,143	10,038	1,143
5,728	1,484	12,998	0,428	1,789	300	4,5	3,36	4,643
5,499	1,35	3,064	0,428	0,789	497,143	1	42,8904	0,572
4,363	1,864	1,198	0,856	0,789	454,286	1	58,31	0,143
5,93	1,198	1,998	0,428	0,789	505,714	4,5	5,4852	4,643
4,561	1,064	2,064	0,428	0,932	214,286	1	15,9432	4,643
9,16	1,864	2,789	0,428	4,5	214,286	0	0	0,143
6,479	4,995	2,064	0,123	0	651,429	4,5	36,8256	0,143

5,786	0,473	0,264	0,123	0,855	120	4,5	23,982	1,143
5,574	1,864	4,998	0,428	1	240	1	3,4944	1,789
4,44	2,141	4,209	0,428	0,932	214,286	4,5	4,3008	1,143
3,86	1,141	1,787	0,428	0	282,857	4,5	93,296	0,143
9,517	0,932	8,921	2	0	60	1	0	0,789
4,634	2,642	5,132	1,428	0	180	4,5	13,986	1,143
5,506	0,998	1,998	0,428	0	72,8571	1	15,6576	4,643
3,977	2,282	5,653	0,428	1	428,571	1	49,4452	1,789
4,069	1,721	0,286	0,123	1	514,286	0	13,44	6,143
4,278	2,653	12,495	0,428	0,066	120	4,5	76,65	4,643
2,197	4,918	0,855	0,189	0	197,143	4,5	0	0
3,709	2,653	2,064	0,123	0	334,286	0	5,376	4,643
2,906	0,286	3,198	0,123	0	360	1	36,19	0,143
4,992	1,787	2,998	0,428	0	154,286	1	9,324	0,143
3,401	2,007	0,998	0,428	4,5	385,714	0,789	0	4,643
6,504	1,919	1,053	0,428	0,143	394,286	1	12,8282	0,143
12,908	1,853	4,143	0,428	0,789	180	4,5	0,2268	0,789
6,94	1,218	2,578	0,428	0,789	265,714	0	0	0,143
6,93	2,644	1,064	0,123	1	300	1	14,973	1,143
3,414	1,218	2,218	0,428	0,789	257,143	1	3,8724	1,789
1,977	0,418	0,638	0,428	4,5	411,429	4,5	45,0408	0,932
5,506	3,222	3,789	0,428	1	214,286	1	47,964	0,143
5,858	2,433	5,576	0,428	0	291,429	4,5	24,1332	0,789
3,852	0,198	2,066	0,428	1	274,286	0,789	0	0,143
6,788	1,218	7,075	1	1	180	4,5	0	5,289
8,653	1,416	1,721	0,856	0,789	300	4,5	65,3072	4,643
3,214	0,855	1,286	0,123	1	385,714	4,5	9,324	4,566
5,137	1,855	1,141	0,428	1	514,286	0	20,986	6,066
6,01	1,71	3,209	0,123	1	231,429	0,789	9,324	0,143
7,227	1,93	2,484	0,428	1	308,571	1	26,7232	1,143
6,563	1,93	1,286	0,123	1	205,714	0,789	4,3008	0,143
3,359	0,921	1,075	0,551	0	257,143	4,5	0	5,289
4,056	1,855	2,93	0,123	0	411,429	1	16,261	6,789
5,212	1,075	2,484	0,066	0	360	4,5	2,7972	4,643
5,972	4,995	7,421	1	0	282,857	1	0	4,643
6,284	1,284	6,495	0,246	0	137,143	1	2,688	0,932
2,546	1,273	5,853	0,123	1	360	1	11,8776	5,289
8,2	2,15	2,855	0,428	0	514,286	4,5	2,2848	0,789
10,201	0,418	0,132	0,428	0	60	4,5	179,984	1,143
5,781	1,218	1,418	0,428	0	240	0	2,688	0,143
2,901	5,498	4,924	1,214	0,789	197,143	4,5	6,7872	0,066
5,684	1,987	1,275	0,428	4,566	282,857	0,066	21,1932	0,286
9,935	1,504	2,715	0,428	0,789	171,429	4,5	0,8064	4,643
6,598	1,284	3,921	0,786	0	240	1	46,8636	1,789
4,838	1,141	6,275	0,123	4,5	565,714	1	93,296	5,289
4,774	2,433	0,275	0,428	0	608,571	1	28,4004	0,066
6,139	3,156	6,561	0,428	4,5	120	4,5	3,4944	4,643
4,94	5,432	0,198	0,428	0	548,571	4,5	2,7972	0,143

11,477	2,13	6,275	0,428	0,066	557,143	4,5	25,3302	4,566
7,012	6,782	8,073	0,786	4,5	325,714	4,5	2,688	5,289
11,075	1,93	1,207	0,856	1	180	4,5	23,324	0,143
2,84	1,427	4,709	0,428	0,789	154,286	1	19,2528	1,218
3,403	1,35	5,998	0,066	0	360	1	5,6028	6,066
4,985	0,998	0,209	0,123	4,5	471,429	1	3,8724	0,143
1,1	1,853	5,218	0,428	4,566	197,143	6	42,8148	5,289
10,176	1,273	0,209	0,428	4,5	265,714	1	9,198	4,643
5,641	1,638	3,275	0,428	4,5	428,571	0	11,6592	1,789
6,155	1,996	3,066	1,786	1,066	308,571	4,5	7,35	1,789
7,642	0,638	0,924	0,551	0	274,286	4,5	14,4144	0,143
5,201	1,141	6,13	0,428	1	197,143	0	6,9972	4,643
4,788	1,996	0,418	0,428	0,143	102,857	4,5	0	0,143
5,583	1,998	6,156	0,428	4,5	394,286	0	3,4944	5,289
3,508	0,418	3,264	0,856	1	137,143	0,789	30,8028	1,143
3,755	0,693	2,361	0,786	0	514,286	4,5	5,5944	0,143
7,769	0,998	0,429	0,428	4,5	385,714	1	28,2072	0,143
2,139	1,855	0,066	0,428	0	360	4,5	45,0632	0,066
9,455	5,718	3,075	0,428	0	240	1	3,4944	5,289
2,909	0,781	3,713	1,214	0	385,714	1	22,0752	0,143
8,004	1,143	2,143	0,123	0,789	300	1	0	0,143
17,374	1,721	0,789	2	1	137,143	1	1,4784	0,789
4,138	2,064	6,721	0,428	0	531,429	0	8,442	0,143
5,072	1,636	3,005	0,428	5,289	334,286	4,5	27,972	4,643
4,763	0,998	3,286	0,428	0	0	1	2,688	1,789
3,909	0,275	0,132	0,786	0,789	308,571	1	32,004	0,143
6,352	1,93	0,998	0,123	0	180	0	2,7972	0,209
7,431	2,198	1,787	0,428	1	265,714	4,5	25,6564	5,289
4,344	1,13	4,776	0,428	0	411,429	0,789	0	0,143
6,727	1,776	0,638	0,428	0	154,286	0	13,0704	0,572
5,992	1,064	1,209	0,066	0,429	497,143	0,789	3,6792	6,143
7,574	5,421	1,209	0,551	0,789	154,286	1	13,2594	0,066
3,26	2,141	0,638	0,428	1	180	1	38,9312	0,789
11,363	6,507	10,007	0,786	0	394,286	4,5	6,9384	5,289
3,852	1,71	5,286	0,428	0,789	462,857	1	18,0012	1,578
6,489	1,001	1,352	1,123	0,143	488,571	1	79,3016	0,066
7,438	2,499	1,998	0,428	4,5	514,286	4,5	11,0796	0,789
9,363	5,498	7,218	0,786	0	531,429	1	66,5056	0,789
3,478	1,053	4,561	1,284	0,789	531,429	4,5	12,9024	0,932
3,137	1,141	1,218	0,132	4,5	257,143	0	0	6,789
3,414	1,787	3,847	1	0	377,143	0	4,8384	0,066
8,011	1,776	11,787	0,123	1	514,286	4,5	18,396	1,789
4,412	1,427	4,367	0,123	0	411,429	0	0,8064	1,143
2,392	2,653	1,064	0,428	0,932	360	4,5	43,442	1,578
8,481	0,704	1,644	0,123	0	137,143	1	4,2588	1,143
6,425	2,576	0,561	0,428	1	325,714	4,5	20,5912	0,932
6,572	1,996	1,644	0,428	4,5	282,857	1	7,4592	0,789
3,478	1,93	8,429	0,428	4,566	402,857	1	45,3306	0,932

8,155	2,853	6,064	0,428	1	282,857	4,5	93,296	4,643
7,223	2,359	2,71	0,123	0	154,286	1	41,958	0,066
7,732	1,484	6,653	0,428	0	308,571	0	6,8292	0,572
10,002	1,93	2,064	0,428	4,5	274,286	4,5	21,5656	0,143
3,772	1,35	3,207	0,856	0	334,286	4,5	14,5656	6,789
6,647	1,559	10,995	0,428	6	240	4,5	0	6,143
2,043	1,064	3,855	0,123	0	180	0,143	0	0,932
6,95	0,704	6,207	0,428	0,066	154,286	1	12,1212	1,143
3,579	0,429	0,209	0,428	1	300	4,5	7,4592	0,143
5,704	1,636	7,57	1	4,643	445,714	1	128,6684	0,143
7,499	0,407	5,418	1	4,5	60	1	10,794	0,789
5,711	1,787	1	0,123	0	171,429	4,5	0	5,289
5,28	2,13	4,141	0,428	0	300	0	0,8064	1,789
6,221	0,836	8,796	1,428	6	300	0	0	4,643
6,232	0,495	4,578	0,428	0	180	0,789	0,6804	1,143
6,223	2,293	0,921	0,786	0	617,143	1	107,9904	0,789
5,363	0,407	8,495	0,428	4,566	514,286	1	12,726	6,789
1,603	0,495	1,275	0,123	0	300	1	29,925	0,209
5,347	5,784	0,847	0,786	6	480	0,429	28,6692	6,789
4,423	0,921	2,71	0,551	4,5	480	4,5	22,7892	0,143
5,935	2,007	0,209	0,123	0	240	1	22,8256	0,143
5,795	1,13	11,789	0,123	0	205,714	1	4,8384	0,789
4,135	2,933	3,064	0	4,929	480	6	18,648	0,143
5,915	2,367	2,719	0,428	0	334,286	1	16,4052	1,143
6,933	2,928	12,784	0,428	1	497,143	4,5	46,7222	0,143
4,757	0,55	2,561	1,246	0	248,571	0	48,9272	1,143
9,651	2,576	0,132	0,786	0,066	411,429	1	29,6632	0,789
5,992	5,498	1,721	0,856	1	420	4,5	26,6966	0,572
3,851	1,493	3,855	0,428	1	394,286	1	3,4944	0,143
3,917	1,787	0,132	0,123	0	274,286	0	3,3726	0,572
6,355	1,776	1,855	0,428	0	411,429	1	1,1844	0,932
5,482	1,35	2,209	0,428	0	497,143	1	0,2268	4,566
8,989	0,627	0,209	1,428	0	257,143	1	26,9892	6,143
9,557	2,71	0,429	0,428	0,429	377,143	4,5	18,648	0,789
5,502	1,921	3,286	0,123	0	94,2857	4,5	2,7972	1,143
15,437	2,51	9,578	0,786	0,429	137,143	6	0	6,789
3,852	1,93	3,156	0,428	1	77,1429	1	46,648	4,643
4,564	1,273	3,855	0,856	0,066	180	0,789	1,8396	0
4,786	1,636	1,284	1,214	0,789	308,571	4,5	53,3232	0,286
6,561	0,561	3,341	1,428	0,789	325,714	0,789	91,8904	0,143
2,427	1,064	2,143	0,123	0	68,5714	1	6,132	4,643
5,137	1,644	0,143	0,123	0	222,857	6	1,5624	0
3,775	1,93	1,922	0,123	0	540	0	37,4052	0,789
4,564	2,576	0,572	1	0	180	1	13,6584	1,789
4,073	4,5	5,075	0,786	0	240	1	42,322	0
4,423	2,642	4,572	2,5	0	240	4,5	1,8396	6,143
7,506	1,996	6,864	0,066	4,5	377,143	1	37,9764	1,578
13,296	10,218	1,275	0,786	0,429	68,5714	4,5	0	1,218

3,995	2,361	1,132	0,786	0	497,143	6	8,6604	0,143
5,977	9,352	14,221	2,5	0,143	317,143	1	15,8592	6,789
6,078	1,262	7,998	0,428	1,143	42,8571	4,5	13,44	1,143
5,35	1,056	1,853	1	0	171,429	0,066	15,0612	4,643
5,583	1,787	6,141	0,428	0,789	171,429	1	18,4632	1,578
6,792	1,196	0,198	1	0,429	231,429	1	26,3676	1,066
8,093	1,493	1,064	1,066	1	197,143	0,789	70,1988	0,143
3,852	1,207	2,007	0,856	1,578	180	4,5	24,2424	0,932
6,792	0,998	2,275	0,428	0	137,143	4,5	14,1036	0,143
4,838	1,71	1,341	0,123	0	377,143	1	4,032	0,855
5,08	1,207	6,418	0,551	1	248,571	4,5	19,726	1,578
12,651	0,715	1,932	1,123	0	214,286	4,5	2,1504	0,789
5,015	1,578	2,209	0,428	0	214,286	4,5	18,396	1,143
13,86	1,644	4,561	0,786	0	342,857	1	14,7756	5,289
3,137	5,498	0,209	0,123	1	248,571	1	8,8704	4,643
2,845	9,704	5,721	0,786	1	394,286	4,5	67,0012	4,643
6,715	0,987	5,427	0,123	4,5	111,429	1	26,3676	0,143
12,509	4,632	4,643	0,856	0	180	0	0,8064	0,143
17,793	3,299	13,341	0,428	0	77,1429	1	6,1824	4,643
5,86	2,785	17,216	0,428	0	180	4,5	94,64	4,643
4,632	2,713	4,352	0,428	0	171,429	4,5	46,648	6,143
5,927	5,707	0,781	0,428	0,429	240	0,789	10,2144	1,789
3,128	2,499	7,864	0,428	0,066	265,714	1	11,1636	5,289
7,914	0,55	4,198	0,123	2	334,286	1	4,662	0,143
6,79	2,51	4,352	0,428	1	180	1	26,1072	0,932
3,846	0,998	1,064	0,428	1	411,429	0,789	9,6348	0,143
4,421	5,641	1,847	0,786	6	214,286	1	27,594	6,143
6,087	1,57	7,504	0,428	0	180	4,5	23,7832	1,143
2,482	0,561	6,789	0,123	1	317,143	0	5,376	4,5
6,864	1,143	2	0,428	0	317,143	1	0	4,643
6,876	2,642	1,572	0,428	0	240	1	46,62	4,643
4,495	0,998	3,218	0,428	4,5	377,143	1	32,8314	4,643
3,183	1,064	2,275	0,066	1	180	1	124,1044	1,066
7,021	2,273	4,572	0,856	1,143	394,286	1	21,1932	1,143
12,151	1,064	2,073	1,428	0	214,286	1	61,8436	0,143
5,924	1,35	3,998	0,909	0	437,143	4,5	8,3328	1,578
5,915	1,93	7,284	0,428	0,143	197,143	0	2,7972	1,143
5,641	1,075	5,924	0,428	0	394,286	0	12,1212	1,143
7,005	1,064	0,066	0,856	0,066	411,429	0	46,648	0,066
5,986	2,862	0,998	0,428	0,066	394,286	1	180,012	1,789
5,93	1,644	0,286	0,428	1,578	137,143	1	7,2156	0,932
4,641	1,427	1,71	0,786	0	257,143	1	32,4996	0,143
14,572	1,998	6,855	2,5	0,789	162,857	4,5	6,72	4,5
4,348	2,064	2,352	0,428	4,5	274,286	1	9,0552	4,643
6,732	0,921	1,855	0,123	4,5	180	4,5	300,02	4,566
8,571	1,13	2,286	0,428	0,066	205,714	1	18,3204	1,143
4,83	2,71	1,787	0,428	4,5	497,143	1	5,376	0,066
9,239	2,293	0,429	0,246	0	154,286	0	179,984	0,143

5,344	1,864	3,286	0,123	0,572	154,286	1	2,7972	0,572
6,221	1,996	6,141	0,123	0	214,286	1	0	1,143
2,469	1,987	6,275	0,786	0	282,857	4,5	19,6336	6,143
4,627	0,987	4	0,428	4,5	462,857	0	27,972	5,289
3,197	1,776	6,264	0,428	0	480	0	16,926	0,066
7,914	1,207	4,275	1,786	0,789	300	4,5	22,5232	0,143
2,109	2,276	5,275	0,123	6	334,286	0,789	0	6,789
6,994	1,132	3,264	0,428	0	377,143	6	27,2454	0,143
9,809	2,433	1,075	1	0	291,429	0,789	14,4592	1,143
6,117	0,998	1,066	0,123	0	454,286	1	1,6128	0,066
6,724	1,075	1,352	0,189	0	197,143	1	6,132	0,143
3,483	6,21	7,053	0,428	0,066	214,286	4,5	29,0724	6,789
2,48	1,93	5,429	0,428	4,5	300	1	28,6944	5,289
7,447	1,207	13,361	0,428	0,143	522,857	4,5	9,324	0,932
2,414	2,787	1,361	0,123	0	557,143	0	31,6512	4,643
3,195	1,198	0,209	0,428	0	171,429	4,5	24,3852	0,855
9,8	1,13	4,433	0,428	1,066	137,143	1	26,0652	0,143
2,546	0,998	5,998	0,428	4,5	360	1	23,324	4,643
1,836	2,282	3,787	2,5	0	197,143	1	27,986	6,143
10,949	0,932	0,495	0,066	0	94,2857	1	7,9716	0,143
5,441	2,642	2,141	0,428	0	360	4,5	73,6596	1,143
3,437	2,218	8,075	0,428	0,789	497,143	6	46,62	4,643
5,79	1,853	4,499	0,428	0,789	154,286	4,5	11,1636	0,143
20,357	4,841	15,858	0,852	4,566	171,429	4,5	5,1744	6,143
4,272	1,275	5,064	0,123	0	120	4,5	31,1164	6,143
8,015	2,15	7,429	0,428	4,5	402,857	1	8,9292	1,218
2,766	0,484	2,787	0,123	1	480	1	3,6792	6,143
4,129	1,93	2,066	0,428	0,789	351,429	1	26,4432	0,066
7,864	2,139	2,627	0,786	0,143	94,2857	4,5	0	0,932
6,566	3,064	0,572	0,246	4,5	445,714	4,5	5,5944	1,789
5,426	1,361	0,143	2,623	0	377,143	0	64,386	0,066
3,489	1,842	1,71	0,123	1	600	0	54,0792	0,066
3,983	2,207	8,51	0,428	0	240	1	0	4,566
2,471	1,066	0,132	0,123	0,143	445,714	1	8,064	0
6,084	1,996	2,653	0,428	0,143	137,143	4,5	10,2144	6,143
7,021	1,141	1,075	0,066	0,789	531,429	4,5	6,804	0,143
4,212	1,218	6,998	0,428	0,429	188,571	1	6,132	0,143
8,332	1,787	1,352	0,123	1	505,714	0,429	1,8396	4,643
6,155	1,141	1,286	0,428	0,789	137,143	1	0	5,289
5,573	2,51	7,141	0,428	1	154,286	4,5	30,6964	5,289
5,429	1,064	11,222	0,428	0,789	180	0	7,476	4,643
6,653	2,93	5,352	0,428	0	377,143	4,5	18,648	0,789
5,994	0,275	1,495	0,123	0	480	1	1,8396	4,643
11,919	0,987	12,341	0,123	4,5	462,857	FAŁSZ	53,9952	4,643
3,329	2,141	12,282	0,786	0,066	360	6	13,818	0,209
3,864	0,132	0,066	0,066	0	428,571	4,5	0	0,789
5,638	2,499	4,361	0,428	0,143	102,857	4,5	5,5944	0,932
6,231	1,361	3,578	0,428	1	180	1	36,0024	0,932

4,429	*1,636*	*1,787*	*0,428*	*0*	*471,429*	*1*	30,66	*6,143*
5,649	*0,561*	*6,207*	*0,428*	*6*	*137,143*	*1*	2,7972	*1,143*
3,041	*1,196*	*9,864*	*0,123*	*4,5*	*420*	*4,5*	5,376	*4,643*
7,229	*3,367*	*6,286*	*0,428*	*0*	*325,714*	*1*	0	*1,218*
5,348	*0,275*	*5,566*	*1*	*0*	*300*	*1*	6,9972	*0,143*
7,502	*0,561*	*1,286*	*1*	*1*	*291,429*	*4,5*	5,5944	*0,143*
9,712	*1,207*	*0,429*	*1*	*1,066*	*480*	*4,5*	32,0992	*1,789*
4,484	*1,493*	*14,642*	*0,123*	*0,143*	*300*	*4,5*	18,102	*4,643*
4,994	*1,075*	*4,998*	*0,786*	*0,429*	*51,4286*	*0,429*	0	*0,932*
7,941	*2,576*	*7,361*	*0,856*	*0,209*	*162,857*	*4,5*	0,8064	*5,289*
5,124	*0,561*	*6,998*	*0,123*	*0*	*471,429*	*4,5*	23,3772	*1,143*
4,643	*2,433*	*0,209*	*0,428*	*0*	*197,143*	*1*	0,8064	*0,286*
6,275	*0,198*	*1,998*	*0,551*	*0*	*162,857*	*1*	2,1504	*0,429*
12,342	*2,216*	*8,132*	*0,786*	*0,209*	*411,429*	*4,5*	9,2484	*4,566*
8,56	*1,141*	*2,578*	*0,428*	*0*	*51,4286*	*4,5*	8,2824	*4,643*
4,918	*2,073*	*2,71*	*1,214*	*0*	*180*	*4,5*	6,9384	*1,066*
5,28	*1,919*	*3,578*	*0,428*	*0,789*	*102,857*	*1*	0	*0,143*
4,473	*2,51*	*7,576*	*0,428*	*0,066*	*351,429*	*4,5*	10,0548	*6,143*
2,703	*2,139*	*11,15*	*0,428*	*0,209*	*342,857*	*1*	6,8544	*0,495*
5,766	*0,495*	*5,13*	*0,856*	*0,789*	*300*	*4,5*	12,8352	*0,855*
2,395	*2,139*	*2,416*	*0,066*	*0*	*420*	*1*	3,066	*1,218*
3,972	*1,482*	*0*	*0,428*	*0*	*377,143*	*1*	0	*0,789*
11,353	*2,787*	*1,864*	*0,428*	*1*	*428,571*	*4,5*	37,0524	*0,143*
3,195	*2,275*	*4,484*	*0,852*	*1*	*497,143*	*1*	28,7784	*0,572*
6,37	*2,642*	*8,713*	*0,856*	*0,066*	*514,286*	*4,5*	30,3212	*0,932*
3,337	*0,484*	*0,638*	*0,428*	*0*	*214,286*	*0*	32,634	*6,143*
9,285	*1,416*	*3,998*	*0,123*	*0*	*402,857*	*4,5*	0	*0*
3,708	*1,361*	*1,001*	*0,428*	*0*	*145,714*	*4,5*	8,064	*0,789*
2,634	*1,853*	*5,352*	*0,551*	*1*	*257,143*	*0,789*	33,8562	*1,578*
6,005	*0,352*	*0,286*	*0,856*	*4,5*	*334,286*	*1*	8,6604	*0,143*
5,859	*1,987*	*2,51*	*0,428*	*1*	*360*	*4,5*	54,53	*0,143*
3,471	*1,721*	*13,198*	*0,428*	*0*	*120*	*4,5*	3,4944	*4,643*
10,051	*1,418*	*6,275*	*0,428*	*1*	*394,286*	*0*	0,2268	*5,289*
6,572	*1,996*	*0,495*	*0,428*	*0,429*	*205,714*	*1*	12,4684	*1,789*
6,57	*3,293*	*6,787*	*0,428*	*4,566*	*480*	*1*	18,648	*0,932*
3,834	*1,908*	*4,407*	*0,428*	*0,066*	*120*	*4,5*	0,756	*0,932*
1,768	*2,216*	*0,066*	*0,428*	*0*	*137,143*	*1*	31,038	*4,643*
7,592	*1,721*	*8,715*	*0,428*	*0*	*531,429*	*4,5*	4,662	*5,289*
5,572	*2,216*	*7,284*	*0,428*	*0*	*180*	*4,5*	5,4852	*5,289*
9,349	*6,221*	*7,841*	*0,428*	*0,143*	*154,286*	*0*	15,4476	*1,789*
4,768	*1,35*	*0,264*	*0,123*	*0,066*	*582,857*	*1*	5,5944	*0,066*
6,804	*2,433*	*3,939*	*0,123*	*0,789*	*325,714*	*4,5*	3,6792	*1,789*
5,203	*1,207*	*1,264*	*0,123*	*0*	*454,286*	*1*	7,2156	*0,572*
8,202	*1,781*	*0,209*	*2,5*	*0,066*	*360*	*1*	55,2972	*4,643*
4,203	*0,932*	*0,209*	*0,428*	*1*	*257,143*	*1*	318,0212	*0,143*
4,786	*1,787*	*2,284*	*0,428*	*0,429*	*257,143*	*0*	0	*1,143*
3,126	*1,209*	*3,209*	*0,123*	*0*	*300*	*1*	0,8064	*1,143*
7,509	*2,939*	*2,932*	*0,123*	*1*	*85,7143*	*0,066*	0	*1,789*

4,366	2,796	5,141	0,428	1	120	4,5	3,822	4,643
4,154	1,209	0,932	0,786	1	0	4,5	0	1,143
5,278	0,495	6,998	1,428	0,789	180	1	1,6128	0,143
8,869	3,653	6,218	1	4,5	180	4,5	12,3732	4,566
3,261	1,57	0,286	0,428	0,789	300	4,5	30,1224	0,143
6,81	1,721	2,789	0,786	1	17,1429	4,5	19,4782	0,066
5,915	0,998	1,998	0,123	1	77,1429	1	6,1824	1,143
6,284	1,853	5,484	0,428	0,429	240	1	0	1,143
3,915	1,207	4,998	0,428	0	257,143	0,789	26,3676	1,578
5,569	2,073	1,787	0,428	0	214,286	0	0	0,789
11,215	1,79	4,341	0,428	0	428,571	1	30,002	0,855
7,44	1,284	1,352	0	1	120	4,5	22,6632	0,932
4,352	1,644	0,209	0,123	0	180	1	2,688	6,066
6,148	3,073	9,721	0,428	0,789	334,286	4,5	13,0704	0,932
7,587	1,644	1,053	0,428	0	154,286	4,5	15,0612	0,143
7,59	2,216	5,284	0,428	0	257,143	1	10,1472	0,143
6,372	1,93	7,864	0,856	4,5	120	4,5	15,0612	0,932
12,728	1,284	9,929	0,428	0	360	1	10,0548	0,143
3,412	1,998	10,209	0,123	0	445,714	4,5	5,376	6,789
5,005	1,578	0,286	0,123	1	282,857	0	5,1282	0,932
3,695	0,132	8,644	0,428	0	197,143	4,5	0	5,289
13,351	0,932	15,5	1	0	385,714	4,5	5,5944	0
4,291	1,71	5,289	0,786	1	85,7143	4,5	3,8724	0,143
3,566	1,218	3,275	0,786	0	300	6	24,668	0
2,348	1,855	1,075	0,123	0	222,857	4,5	13,5324	0,132
11,085	3,082	8,218	0,428	4,5	257,143	4,5	10,794	5,289
3,772	0,209	9,932	0,428	0	720	1	8,5596	5,289
6,64	2,348	4,365	0,786	0,143	437,143	1	43,6604	1,218
4,634	2,361	0,143	0,123	0	154,286	4,5	0	0,932
10,923	2,062	2,504	1,428	4,5	128,571	1	89,6672	0,789
6,592	1,141	0,998	0,428	1	377,143	1	0	0,789
12,008	1,647	0,998	0,856	0	120	1	0,8064	1,143
11,93	0,998	1,932	0,856	0	240	1	93,324	0,789
2,177	5,707	9,647	1	0,066	180	0,789	5,5188	1
6,214	1,627	1,561	1	0	617,143	1	35,5964	5,289
2,348	1,504	13,13	0,123	4,5	531,429	4,5	6,5604	0,143
6,346	2,631	0,998	1,428	0,789	317,143	4,5	24,1332	0,143
3,203	1,93	2,273	0,428	1	180	0	4,032	0,932
2,975	0,341	1,141	0,428	4,5	60	4,5	1,8396	0,789
4,423	2,275	5,418	1	0	222,857	4,5	35,5964	5,289
12,652	1,919	2,064	0,428	0	265,714	1	5,4852	1,578
10,503	1,864	1,853	0,123	0,789	102,857	4,5	88,9756	1,789
4,699	1,919	1,284	0,428	0	325,714	0	12,9696	0,789
3,845	1,93	2,855	0,428	0	162,857	0	6,132	0,066
5,506	0,855	1,644	0	0	60	0	1,8396	0,143
5,64	0,418	12,218	0,246	0	840	1	0	0,286
7,515	2,565	2,064	0,856	4,5	454,286	1	8,2824	4,643
4,917	2,433	9,641	0,428	4,5	85,7143	4,5	6,132	4,643

2,625	1,209	2,932	0,123	4,5	385,714	4,5	3,4944	4,643
6,935	6,432	5,721	0,428	0	137,143	1	279,0844	0,932
12,006	3,071	4,484	0,428	4,5	180	1	4,662	5,289
6,427	0,858	5,429	0,786	6	600	1	25,4744	6,143
6,337	0,704	1,198	0,856	0,066	205,714	1	18,5052	0,066
8,81	1,13	0,484	0,428	0,789	282,857	1	23,8322	0,143
7,504	1,636	1,853	0,856	0	274,286	4,5	121,8476	1,066
4,291	2,007	9,064	0,123	0	171,429	1	4,662	5,289
5,137	0,066	0,209	0,123	0	428,571	4,5	6,9384	0,143
2,755	1,064	3,207	0,132	0	300	1	6,9468	0,143
4,192	0,473	3,273	0,494	0,066	411,429	1	20,9832	0,855
3,853	0,495	2,998	0,428	0	480	4,5	7,9716	6,789
4,644	0,561	2,367	0,428	1	240	1	10,038	0
5,346	2,644	3,132	0,123	1	248,571	1	53,3792	0,209
5,493	2,71	3,15	0,428	4,566	531,429	0,789	17,8584	0,789
4,985	1,064	1,209	0,123	1	300	0,789	4,3008	4,566
6,368	0,921	1,066	0,428	0	137,143	4,5	33,4908	0,143
4,775	1,647	0,418	1,642	0,789	145,714	4,5	48,9272	0,143
10,004	1,842	1,13	0	0	180	1	90,006	4,643
7,375	2,359	0,998	0,428	0,429	420	4,5	1,8396	1,789
9,36	2,51	1,987	0,856	0	274,286	4,5	10,2144	0,789
5,715	2,216	8,218	0,428	0	257,143	4,5	6,9972	0,932
6,563	2,141	4,644	0,123	0,066	291,429	0,789	9,1896	0,789
11,226	3,576	0,275	0	0	102,857	0	30,66	0,932
4,355	2,141	4,787	0,428	4,643	428,571	0	27,3252	1,789
7,593	2,073	4,862	0,428	0	180	0,066	46,648	5,289
2,823	1,559	5,627	1	4,5	420	1	30,66	4,643
5,078	2,576	4,987	0,428	0	162,857	1	10,2144	0,855
9,084	2,708	2,286	0,123	1	162,857	1	1,6128	0,143
2,777	1,075	14,407	0,066	1	274,286	4,5	8,064	4,643
6,003	2,141	0,341	0,428	0	325,714	0	5,565	0,789
5,796	2,855	2,429	0,428	1	282,857	1	8,064	1,143
3,797	2,293	6,789	0,428	0	214,286	4,5	16,1364	4,5
3,898	5,773	2,484	0,786	0,066	342,857	4,5	32,0488	4,643
4,546	2,433	1,198	0,428	0	120	4,5	17,7982	0,143
4,701	0,341	1,218	0,786	0,429	197,143	0,429	17,0352	0,789
4,851	0,987	2,493	0,428	1	154,286	4,5	9,408	0,143
5,301	1,218	1	0,428	0	248,571	4,5	5,376	0
2,983	1,207	10,987	0,264	1	531,429	4,5	4,368	4,566
3,783	5,072	0,921	0,123	1	342,857	0	13,8768	0,143
4,062	0,759	1,198	1,786	1	454,286	4,5	30,3072	1,789
1,853	0,789	6,064	0,123	0	154,286	4,5	123,9	0,572
6,226	2,864	2,352	0,428	1,789	154,286	1	1,8396	5,289
5,847	1,416	4,998	0,428	0	480	0,789	8,3328	0,572
4,344	2,578	5,576	1,572	0	77,1429	1	22,5232	0,143
6,218	1,284	2,932	0,428	0,789	257,143	4,5	27,986	0,789
3,907	5,718	0,572	0,909	0,066	257,143	1	30,1224	0,209
7,774	1,919	2,715	0,428	0	445,714	4,5	0	0,286

5,21	5,421	3,13	0,786	0,789	162,857	1	7,2156	4,566
6,584	3,075	4,638	0,428	6	214,286	4,5	0	6,143
6,133	0,418	0,495	1	0,789	180	1	24,2424	0,143
5,223	1,853	8,075	0,856	0	462,857	4,5	36,3636	0,143
3,404	1,352	1,71	0,428	0	214,286	1	18,648	4,643
6,438	2,139	4,853	0,123	1	162,857	1	16,1896	1,143
8,857	1,273	4,132	0,066	0	257,143	4,5	9,198	0,143
7,268	0,921	2,787	0,369	0	55,7143	4,5	0	1,789
7,735	2,787	2,143	0,428	0	137,143	1	0,8064	1,578
5,366	2,788	5,787	0,428	0	111,429	1	30,5368	5,289
4,5	1,921	2,433	0,428	0	531,429	1	23,3016	0,066
5,715	1,559	3,075	1,786	1	291,429	4,5	24,528	1,143
12,31	0,932	8,078	0,428	1	214,286	1	0	1,578
8,419	1,132	0,209	0,246	0	377,143	0	0,8064	0,143
5,269	3,216	5,216	1,214	0	214,286	1	69,972	6,143
6,715	1,787	0,286	0,551	0,789	462,857	1	40,5776	1,143
3,071	4,786	0,209	0,066	0	240	1	29,19	0
6,508	1,209	0,352	0,428	0	205,714	0	0	4,643
4,992	1,141	3,209	0,123	1	154,286	1	18,396	0,789
6,138	1,572	6,143	0	1	77,1429	4,5	19,2024	4,643
5,267	6,353	2,407	0,123	0,066	385,714	0,789	15,4476	6,789
2,766	0,638	2,141	0,066	0	180	1	7,9716	1,578
5,269	0,855	2,286	0,123	1	360	1	2,7972	0,143
5,726	1,57	3,007	0,786	0,066	411,429	1	8,1732	1,143
4,852	1,721	0,987	0,123	0	385,714	0	9,5172	0,143
4,568	0,998	2,275	0,123	0	120	1	5,4852	4,643
4,282	2,796	1,864	0,428	0,066	180	0	0	1,143
4,86	2,429	0,341	0,428	0	300	1	0	0,932
5,774	2,075	0,352	0,428	4,5	445,714	1	0	0,789
10,192	0,55	0,998	0,428	0,143	282,857	1	48,0032	0,066
3,404	1,35	6,721	0,428	1	248,571	4,5	35,7924	4,643
5,432	2,073	6,561	0,428	0,143	291,429	0,066	18,102	0,143
12,637	6,204	15,355	2,623	0,429	317,143	1	55,944	5,289
4,148	0,998	2,209	0,123	1	102,857	4,5	12,264	6,066
5,695	2,642	2,216	0,428	1	77,1429	1	0,672	0,932
9,244	2,51	2,286	0,428	0	240	4,5	13,986	1,789
3,841	2,855	4,275	2,5	1	300	0,429	38,542	5,289
2,252	1,559	4,864	0,066	0	360	FAŁSZ	7,2156	0,789
4,651	1,418	2,286	0,123	1	282,857	1	0	1,143
4,338	2,559	8,853	0,428	0,066	771,429	1	12,642	6,789
8,846	2,631	6,119	0,428	0	308,571	1	12,6336	0,066
7,209	2,284	2,721	0,428	4,5	411,429	1	10,2144	0,789
5,427	1,998	10,066	0	1,066	214,286	0	0	1,143
2,472	4,698	2,141	0,428	1	205,714	0,429	0,8064	0,066
4,137	0,198	3,286	0,428	0	102,857	1	10,0044	0,143
6,135	1,93	5,638	0,123	0,143	308,571	0,789	18,648	1,143
7,227	1,787	1,33	0,551	4,5	411,429	4,5	12,7428	0,143
4,278	2,998	5,35	0,428	4,566	180	4,5	37,296	5,289

6,559	1,985	2,282	0,428	0,429	600	1	38,0296	4,566
5,135	0,418	0,275	0,123	0	342,857	4,5	3,8724	0,143
3,783	1,842	2,352	0,428	4,566	437,143	4,5	83,9664	4,643
4,634	0,484	4,632	0,428	0	51,4286	0	1,8396	0,789
6,304	0,143	0,789	0,428	0,429	240	0,789	39,634	0,143
3,704	5,707	1,067	0,922	0,429	197,143	4,5	19,74	1,143
4,135	1,053	0,407	0,428	0	557,143	6	67,3204	0,143
4,289	5,432	1,218	1,214	0	197,143	4,5	13,4484	6,143
3,766	1,416	6,352	0,428	0,066	480	4,5	11,0292	6,143
4,869	0,932	2,075	0,066	1,143	154,286	0,429	8,8704	1,143
6,447	1,218	7,156	0,786	0,789	180	4,5	94,43	0,789
5,08	2,367	5,064	0,123	1	300	4,5	0	0,143
9,925	0,627	4,855	0,428	1	222,857	4,5	4,5276	5,289
4,709	5,85	2,576	0,428	0	197,143	0	5,754	4,643
2,337	0,855	3,578	0,428	4,5	120	1	13,4652	4,643
9,874	6,147	1,789	0,786	0,855	162,857	6	0	5,289
6,796	2,71	10,576	0,428	1,066	180	0	0	0,789
6,443	1,361	8,007	3,5	1	180	1	2,688	0,932
4,63	1,427	7,71	0,123	1	197,143	1	8,064	4,566
4,992	2,141	7,996	0,428	0,143	257,143	1	12,264	4,643
8,814	1,141	0,789	0,428	0	300	0	30,002	0,143
5,849	2,51	8,007	1	0,789	300	0,143	8,1732	6,143
7,361	1,847	1,075	1	1	120	4,5	3,4524	1,143
3,64	2,064	0,921	0,123	0,143	240	0	0,8064	4,643
2,249	0,627	3,198	0,551	0	214,286	0	7,2156	0,932
5,223	1,776	1,853	0,428	0	77,1429	0,789	12,3732	5,289
7,007	2,719	15,156	0,428	1,578	257,143	4,5	24,7324	6,789
4,715	0,33	2,499	0,123	0,429	360	4,5	31,9872	4,643
4,072	2,433	11,493	0,123	4,5	334,286	4,5	23,926	6,789
7,993	0,998	0,932	0,428	0,789	411,429	0	8,4252	0,143
4,649	1,35	2,932	0	0	214,286	4,5	0	4,643
10,639	2,367	2,855	0,123	0	265,714	4,5	20,9832	0
4,363	1,93	2,418	0,428	0	394,286	4,5	8,5512	0,143
5,204	5,421	5,781	1	0	282,857	0	9,6852	6
4,062	1,053	8,286	0,123	0	231,429	0,789	6,132	1,066
5,163	1,064	11,35	0,786	1	257,143	4,5	16,7832	1,789
5,284	1,13	0,998	0,123	0,789	437,143	4,5	25,6536	1,578
6,069	0,275	2,578	0,123	1	205,714	4,5	12,1212	4,643
5,638	2,642	6,284	0,428	4,5	120	1	7,9716	4,566
4,262	3,299	3,638	0,428	0	137,143	1	3,4944	6,143
7,781	1,921	2,862	0,428	0	428,571	0	0	1,789
4,431	0,921	2,429	0,428	0	411,429	1	16,3884	0,932
6,43	1,207	0,286	0,123	4,5	445,714	0	20,6892	0,143
5,44	5,575	4,198	0,428	0	317,143	4,5	0,8064	1,143
4,346	2,007	0,352	0,428	0	274,286	1	35,7952	4,643
7,726	5,498	3,075	0,428	4,5	265,714	1	36,9992	5,289
6,214	1,053	6,341	1	4,5	120	4,5	53,6172	5,5
7,796	1,207	0,921	1,428	0	308,571	1	17,1696	0

7,506	2,141	0,921	0,428	0	120	4,5	65,6572	0,143
5,651	4,929	4,932	0,428	0	72,8571	4,5	20,6892	5,289
2,332	1,93	3,636	0,428	1	162,857	0,066	12,1212	0,209
7,287	1,853	1,075	0,856	1	180	1	23,3184	0,789
8,86	2,416	2	0,428	0,066	8,57143	1	93,296	1,789
8,851	1,996	1,789	0,428	0,066	231,429	4,5	36,0024	0,066
2,098	0,264	20,921	0,066	1	385,714	4,5	0	4,566
4,909	2,427	1,141	0,246	0	205,714	4,5	29,6632	0,143
5,641	0,77	1,495	0,428	1	180	4,5	12,3648	0,932
13,14	2,787	0,495	0,786	0	257,143	4,5	57,3272	0,143
5,781	2,51	5,156	0,123	1	60	4,5	2,7972	0,143
2,351	7,867	5,921	0,123	0	257,143	4,5	4,662	0,143
2,262	1,209	2,998	0,123	1	282,857	0	5,4684	0,066
4,502	1,721	4,286	0	0	411,429	0	22,5204	5,289
5,491	2,13	3,275	0,428	1	377,143	4,5	8,9292	4,643
4,918	0,341	8,053	0,428	1	317,143	4,5	6,9384	0,789
4,909	0,627	1,132	0,066	0	274,286	4,5	15,0612	0
3,783	0,209	4,786	0,123	1	222,857	1	62,1544	4,566
4,509	1,075	0,132	0,428	0,429	445,714	0,789	0	0,429
6,069	1,13	9,299	0,428	4,5	257,143	0	1,344	4,643
4,148	1,998	11,275	0,428	0	257,143	4,5	0	5,289
3,504	2,207	15,855	0,856	1	205,714	1	8,9292	6,789
4,843	1,853	19,209	0,123	0	120	4,5	4,662	0,932
2,491	1,427	0,495	0,066	0	480	0	49,2632	0,066
4,77	0,209	0,998	0,856	0	334,286	1	18,2448	0,789
3,2	2,064	2,427	0	0	385,714	0	12,264	4,566
6,726	1,721	5,585	0,428	4,5	360	0	0	4,643
5,577	1,196	6,418	0,123	1	154,286	4,5	7,9716	0,143
4,346	1,638	1,218	0,066	1	557,143	1	0,756	4,643
2,469	1,776	3,207	0,428	0,789	180	4,5	17,038	5,289
9,426	5,498	0,352	0,856	0,143	462,857	1	24,7464	0,143
1,988	1,141	3,721	0,123	1	462,857	4,5	22,5204	4,643
4,984	1,932	0,55	1,214	0,789	308,571	1	16,0272	0,789
3,863	5,432	16,293	0,428	1	368,571	4,5	13,9944	0,066
4,058	5,641	4,209	0,123	1	514,286	1	2,9064	5,289
7,486	1,787	1,143	0,428	4,5	351,429	1	7,5264	5,289
1,034	0,484	6,576	0,123	0	420	1	8,064	4,643
6,146	0,704	1,143	0,428	0	180	6	77,3192	0,143
3,137	2,568	11,856	0,123	0	531,429	4,5	15,0612	6,143
7,946	1,57	4,075	0,428	0,066	120	4,5	37,7832	5,289
6,075	1,209	0,429	0,428	0	300	4,5	31,9872	0,066
4,368	1,141	4,275	0,123	1	85,7143	1	9,4332	6,143
5,502	2,776	0,998	0,428	0	274,286	0	2,688	0,789
6,43	1,787	6,141	0,428	1	162,857	4,5	21,4452	1,143
3,555	1,342	1,13	0,551	0,066	565,714	1	23,8644	1,143
8,712	1,578	0,932	0,786	0	240	4,5	0	5,289
8,259	2,565	2,789	0,428	0	308,571	1	8,1732	0,066
1,207	0,143	6,275	0,428	0	154,286	4,5	6,0606	5,289

5,726	2,642	1,141	1	0	180	0	18,8776	0,066
10,655	2,631	5,71	0,428	1	180	4,5	3,1836	4,643
9,912	0,921	0,132	1,246	0	437,143	0,143	67,6032	0,143
7,959	2,367	3,51	0,428	0	840	1	46,648	0,789
2,142	0	2,932	0,428	4,5	240	1	16,2582	0,143
11,446	2,433	3,433	0,428	0,143	360	1	11,662	4,643
3,933	0,855	0,143	0,123	0	428,571	4,5	5,418	0,066
4,981	0,998	1,638	0,551	0	205,714	1	3,1836	0
3,893	0,561	0,209	0,066	0	480	4,5	0	0,066
7,07	0,998	1,209	0,123	1	68,5714	4,5	2,7972	6,066
3,217	2,216	12,855	0,786	4,5	240	4,5	5,4852	0,932
12,16	0,924	9,218	1	1	214,286	4,5	13,44	1,789
6,495	1,853	5	0,428	1	222,857	1	11,4744	1,143
13,586	0,352	1,932	0,428	0	300	0	46,62	0,066
10,149	0,55	3,921	0,428	0,066	94,2857	4,5	30,0552	0,932
4,498	6,21	3,066	0,428	4,566	351,429	1	3,738	4,643
2,205	2,075	1,132	0,428	0,143	377,143	1	93,296	0,932
11,224	1,284	3,284	0,428	4,5	385,714	0,143	12,1212	0,932
5,717	1,064	0,932	0,786	0,789	257,143	4,5	69,3252	1,789
3,709	1,35	0,998	0,786	4,5	334,286	0	36,0024	0,143
9,872	0,275	15,075	0,786	4,5	360	1	29,3244	5,289
4,788	2,075	1,998	1,428	0	360	0	2,7972	0,066
4,616	1,985	0,132	0,066	0	368,571	4,5	112,6244	0,143
11,154	2,075	2,218	0,428	1	411,429	1	26,859	1,143
4,568	2,062	1,352	0,428	0,066	428,571	1	124,1044	0,932
2,702	1,284	7,216	0,246	0	514,286	4,5	19,7232	4,643
9,083	1,998	0,998	0,856	0,789	480	4,5	0	0,789
4,065	0,77	6,919	0,428	4,5	334,286	4,5	18,0012	1,578
8,005	3,141	4	0	1,143	248,571	4,5	2,7972	0,789
4,201	1,119	1,71	0,786	1	514,286	4,5	19,2612	0,495
6,643	2,073	0,781	0,428	0,143	385,714	1	39,326	1,143
5,655	2,499	1,715	0,428	4,5	428,571	4,5	24,528	4,643
5,656	2,433	2,796	0,123	0	291,429	4,5	3,6792	5,289
5,574	1,855	5,218	0	0	68,5714	4,5	5,376	0,789
4,135	1,504	5,939	0,123	1	514,286	1	115,948	5,289
4,566	5,289	1,209	0,428	0,066	445,714	0,143	25,4632	0,132
5,64	2,719	10,653	0,428	0,855	137,143	1	11,13	4,643
4,614	0,902	5,93	1	0,789	231,429	1	24,528	4,643
2,546	1,053	1,284	0,123	4,5	137,143	0,143	10,2816	0,789
5,566	1,196	5,418	0,123	1,143	222,857	4,5	0	1,218
5,216	0,921	0,286	0,123	1	162,857	0	2,7972	4,643
4,201	2,928	8,35	0,123	4,5	300	1	67,298	0,143
3,429	0,715	4,721	3,286	0	197,143	1	16,2204	0,066
5,355	2,994	11,566	2,5	0	685,714	1	93,296	0,143
3,7	0,198	1,209	0,123	1	0	1	2,7972	0,143
10,655	2,51	4,361	1	1	55,7143	1	7,8162	1,578
6,442	5,061	0,998	0,786	0	214,286	0	0	4,643
8,218	5,63	1,789	0,428	1	308,571	1	7,35	0,789

3,77	0,407	0,209	0,428	0	497,143	4,5	32,69	0,789
4,575	1,561	1,143	0,428	0,789	120	1	27,972	0,789
5,078	1,787	3,367	0,428	4,5	120	4,5	71,505	0,143
2,986	1,932	8,495	1,856	1	385,714	1	18,648	0
7,004	1,196	5,066	0,856	0	120	4,5	11,508	0,932
4,487	5,564	2,998	0,786	0,066	240	1	27,972	0,143
6,273	1,998	0,275	0,428	4,5	265,714	4,5	8,442	0,286
5,069	2,064	4,638	0,428	4,5	377,143	1	33,8352	1,578
7,524	2,007	1,932	0,428	0,789	411,429	4,5	10,794	0,932
1,834	0,352	5,207	0,428	0,789	137,143	4,5	25,872	0,143
8,02	4,852	5,932	0,428	1	300	4,5	45,738	5,289
7,871	4,709	1	0,428	1	308,571	1	0,8064	0,789
4,709	2,293	10,51	0,428	0,789	351,429	4,5	13,4484	5,289
6,444	0,638	2,075	0,428	0	214,286	1	6,132	4,643
5,275	1,644	4,495	0,428	0	180	4,5	4,1412	1,578
6,078	1,482	5,139	0,428	0	565,714	1	152,8072	4,929
4,335	1,855	1,932	0,123	1	240	0	5,376	4,643
4,503	1,427	1,064	0,428	1	265,714	1	12,096	0,932
3,999	1,855	0,143	0,428	0	471,429	4,5	46,62	5,289
10,982	1,908	2,352	0,856	4,5	120	1	14,5656	0,143
2,983	0,789	1,721	0,786	0,143	120	1	0	0,143
2,482	2,785	3,209	0,428	1	377,143	1	24,2424	1,789
5,845	5,553	4,13	0,786	0	300	1	4,8888	5,289
6,232	1,504	3,299	0,428	0	548,571	4,5	0	4,643
5,638	0,77	0,932	0,428	0	154,286	4,5	26,663	0,143
2,15	2,642	2,429	0,428	4,5	137,143	1	1,344	5,289
3,871	1,001	10,286	0,428	0	514,286	0	49,4172	0,143
4,638	1,713	4,433	0,786	4,643	291,429	1	1,6128	0,789
5,294	1,141	10,218	1,572	4,5	514,286	1	5,4852	4,5
3,841	1,787	2,998	0,786	0,855	514,286	1	301,364	0,789
2,634	1,721	0,932	0,428	0	300	1	10,9704	4,643
5,864	2,075	1	0,856	0	240	1	0	0,143
3,69	0,418	0,55	0,909	0,789	265,714	1	24,2424	0,066
5,366	1,339	0,561	0,856	0	231,429	1	6,5016	0,143
7,551	1,207	0,352	0,428	0	180	1	8,064	4,566
5,436	1,35	3,218	0,428	1	154,286	4,5	0,8064	1,789
3,544	0,693	2,275	0,123	0	334,286	1	12,8352	0,066
8,363	1,284	1,275	0,428	1	685,714	4,5	0,8064	1,066
3,901	2,284	3,572	0,856	0	445,714	4,5	18,6592	0,143
4,946	2,51	2,143	0,428	0	368,571	1	98,9212	5,289
9,598	1,93	0,286	0,428	1	205,714	1	5,376	0,789
9,455	1,418	1,572	0,856	1,066	274,286	0,143	63,679	1,789
8,084	1,141	1,341	0,551	0	411,429	4,5	5,1282	0,143
6,867	1,141	1,855	0,551	1	497,143	1	1,8396	0,066
6,85	6,144	6,275	0,428	0	231,429	4,5	8,8704	6,143
10,578	0,847	6,156	0,428	6,066	222,857	0,143	5,376	1,578
4,192	1,352	1,855	0,123	0	394,286	4,5	15,0612	1,578
5,774	1,284	4,132	0,066	1	222,857	4,5	6,132	0,143

4,709	2,053	5,209	0,123	0	325,714	1	9,2484	0,789
3,359	1,93	10,864	0,428	0	317,143	4,5	2,7972	5,289
1,878	1,284	12,418	0,428	0	411,429	4,5	19,2024	0,066
10,718	2,207	3,207	0,786	0,066	120	4,5	57,974	4,643
4,487	1,644	0,998	0,428	4,5	428,571	4,5	17,8584	1,789
11,002	1,985	2,141	1,214	0,066	162,857	1	5,6448	4,643
3,926	4,852	2,998	0,123	0,789	214,286	1	2,1504	1,143
6,087	2,642	10,864	0,428	0,066	231,429	1	18,396	5,289
4,003	1,644	0,429	0,428	0,789	514,286	0,789	10,8444	0,932
8,587	1,196	0,572	0,428	0	248,571	0	0	1,143
6,283	2,284	3,218	0,428	4,5	205,714	1	3,066	5,289
2,931	1,075	4,156	0,551	0	317,143	0,143	26,3214	1,578
3,272	1,275	0,987	0,428	0	300	0,429	8,064	6,143
9,362	5,566	1,286	0,786	0	102,857	4,5	38,066	0,143
6,728	1,721	3,143	0,428	0	265,714	4,5	0	1,218
3,275	0,066	6,225	0,428	0	197,143	0	0	0,789
10,257	4,011	11	0,428	4,5	308,571	6	25,3344	0,858
3,907	1,998	2,495	0,786	0,429	480	4,5	24,528	0,932
4,348	2,367	7,207	0,428	0	428,571	1	34,8012	0,143
6,216	2,787	3,715	0,551	0,143	360	4,5	8,5512	0,932
11,134	2,359	0,286	0,428	4,5	162,857	4,5	0	0,789
9,215	1,787	5,427	0,786	0,066	120	4,5	12,726	4,643
1,691	1,13	2,209	0,428	1	102,857	0	3,6792	5,289
3,84	0,396	1,275	0,123	0,209	497,143	1	19,362	0,066
3,39	0,561	1,141	0,428	1	497,143	1	6,9972	0,143
4,481	1,787	2,998	0,246	1	454,286	0,429	12,264	1,218
7,574	2,433	5,341	0,856	0	291,429	1	7,4466	1,143
3,634	1,987	2,075	0,123	0	291,429	1	10,8612	0,143
21,863	1,636	1,143	4,5	0,066	162,857	0,789	0	0,143
8,062	1,207	0,341	0,856	0,429	120	1	20,9916	5,289
3,36	0,715	1,789	0,123	0	317,143	4,5	6,1824	0,143
6,005	1,196	1,987	0	0	257,143	0	16,926	0,789
5,99	1,064	0,561	0,428	0	282,857	4,5	46,648	1,143
3,926	2,939	1,578	0,428	0	497,143	4,5	90,5324	1,578
3,709	1,141	4,789	0,551	0,143	265,714	4,5	20,5464	0,932
3,77	1	2,572	0,428	1,066	48,5714	0	0	1
6,286	1,853	2,787	0,123	0	240	0,066	18,7572	1,143
6,142	1,218	3,064	0,428	0,789	325,714	1	34,9272	0,286
9,703	1,919	3,427	1,066	0,789	385,714	4,5	15,0528	0,855
2,689	1,416	4,141	0,123	0	214,286	4,5	8,2824	0,932
6,071	2,007	0,627	0,786	0	651,429	0	0,8064	1,143
6,932	1,352	0,998	0,428	1	300	1	21,0672	4,643
10,206	2,367	0,275	0,786	1	402,857	0,143	2,7972	0,143
8,551	2,576	5,787	0,428	4,566	300	4,5	13,9944	4,643
5,585	1,864	2,51	0,428	0	180	4,5	0	1,578
5,355	2,51	3,064	0,428	1	308,571	1	6,9972	0,143
7,449	1,853	1,352	0,428	0,143	197,143	1	0	5,289
4,924	2,139	0,484	0,856	0	154,286	4,5	7,6188	0,286

1,548	0,275	0,352	0,428	0	325,714	1	14,9268	0,143
4,997	2,565	1,493	0,551	1	222,857	0	8,3412	0,932
7,442	2,642	1,207	0	0	162,857	0,789	6,9972	0,789
5,346	2,642	1,141	1,214	0	180	0,789	8,3412	0,143
6,062	1,35	8,495	0,428	1	317,143	4,5	0	6,143
4,781	1,473	8,847	0,066	1	317,143	1	31,843	6,143
2,548	1,053	10,855	0,428	0	300	1	76,622	4,643
4,281	2,073	5,007	0,123	4,5	514,286	1	19,7232	6,789
3,964	1,341	0,921	0,856	1	282,857	1	19,3452	0,789
5,067	2,15	1,644	0,428	0,066	154,286	4,5	44,7552	1,143
2,548	1,721	2,418	0,123	0	197,143	1	35,378	4,566
3,691	2,209	4	0,428	1	325,714	4,5	120,008	6,143
6,855	0,495	1	0,428	0	274,286	0	12,264	0,429
4,509	3,064	5,075	0,066	0	0	4,5	140,3976	0,143
6,706	0,855	0,998	0,856	4,5	248,571	0,789	0,2268	0,143
7,155	1,57	6,299	0,856	6	360	4,5	6,9972	0,789
7,365	1,141	0,143	0,123	0	87,1429	0	0	4,643
5,489	1,262	4,921	0,428	1	171,429	4,5	2,7972	4,643
3,64	1,207	7,932	2,928	0	317,143	0	0	1,143
5,203	2,93	4,33	0,428	0	300	4,5	36,9992	0,066
8,456	2,631	1,13	0,428	1	120	4,5	18,648	1,578
9,134	1,853	7,352	0,428	4,5	180	1	5,376	0,789
6,067	1,998	2,284	0,428	0,066	308,571	1	8,1732	0,572
7,583	1,996	1,932	0,428	0	291,429	4,5	0	0,789
3,651	1,721	0,715	0,786	4,5	480	0,143	13,9944	4,5
12,505	2,15	1,644	0	0,789	385,714	4,5	18,396	0,143
8,646	2,064	0,638	2,5	0,429	102,857	1	42,266	5,289
6,795	2,499	5,638	0,428	4,5	282,857	0,789	19,7232	0,932
5,695	1,57	5,218	0,428	4,643	137,143	1	10,1304	4,643
5,779	3,576	1,856	0,856	0	180	1	22,6632	0,789
8,486	1,713	0,132	0,123	0	385,714	6	12,264	4,643
5,231	0,495	6,51	0,066	1	377,143	1	5,4684	0,143
3,269	1,141	2,075	0,123	1	214,286	1	5,376	4,643
9,349	0,921	0,352	0,856	4,5	240	4,5	14,1036	0,143
9,076	5,784	1,429	0,428	0,066	154,286	6	0	0,789
3,574	1,93	1,132	0,428	0,789	274,286	1	25,6452	0,572
4,357	1,352	2,352	0,428	1	102,857	0	8,9292	4,643
3,983	1,71	0,998	0,066	6	360	1	8,442	6,789
2,337	0,352	1,932	0,123	1	120	4,5	13,986	1,143
5,203	0,352	2,286	0,123	0	497,143	4,5	3,4944	0,143
3,917	0,998	1,352	0,123	1	505,714	1	120,008	0,209
3,838	1,71	2,921	0,066	0	325,714	4,5	1,8396	0,143
7,079	6,144	2,066	1,123	0	411,429	1	5,0232	0,789
4,715	1,075	4,93	0,551	4,5	428,571	4,5	76,9412	0,789
5,192	1,787	1,198	0,428	1	514,286	1	6,3672	0,143
5,337	1,787	3,143	1	1	257,143	4,5	2,9568	1,143
2,494	0,715	6,786	0,428	4,5	411,429	1	36,0024	0,143
6,3	2,007	2,653	1,428	0	325,714	1	6,5016	0,143

4,374	0,143	0	0	0	300	4,5	18,396	0
6,049	0,921	2,715	0,551	0,789	497,143	1	18,0012	0,932
12,414	1,218	0,932	0,922	1	411,429	6	30,3212	0
3,467	1,13	1,864	0,123	0	445,714	0	31,038	0,143
7,046	1,075	2,075	0,428	4,566	240	1	3,8724	0,855
4,84	0,484	2,075	1	0	480	6	0	0,143
3,005	0,132	0,998	0,428	0,789	505,714	1	0	1,789
2,48	1,075	4,141	0,856	4,5	180	1	22,5372	0,932
5,994	1,71	0,132	0,428	0,789	188,571	4,5	72,282	0,143
2,472	3,156	7,21	0,066	0	420	4,5	21,462	4,643
7,019	1,864	2,43	0,123	0	240	1	9,8112	0,789
9,521	0,789	1,644	2	0,789	205,714	0,066	69,972	0,932
7,444	1,985	4,222	0,428	0,855	111,429	1	12,9696	4,643
5,438	0,132	2,352	0,428	1	68,5714	1	16,926	0,143
3,328	4,566	1,209	0,786	0,066	274,286	0	0	4,643
11,142	2,998	4	0	0	60	1	57,974	0,143
5,724	0,473	4,132	0,428	0	282,857	4,5	6,7452	0,143
4,57	2,921	3,143	0,786	1	300	0	18,396	0,143
10,798	3,156	2,429	0,428	0	265,714	4,5	4,662	5,289
6,548	0,932	4,932	0,428	1	317,143	1	58,31	4,566
4,795	0,987	1,141	0,428	0,429	154,286	1	16,6824	0,789
10,221	3,365	4,721	0,428	1,066	480	1	18,2112	4,643
6,928	1,132	2,789	0,428	0	240	0	12,264	0,143
6,944	4,929	3	0,428	0,789	317,143	1	0	1,143
6,282	1,93	7,156	2,5	0	60	4,5	3,066	6,143
4,643	1,075	0,855	0,066	1	471,429	0,789	3,8724	0,066
5,847	1,064	7,51	1	0	265,714	0	40,8002	1,066
2,994	1,284	4,35	0,066	1	420	0,066	0	5,289
5,515	2,359	0,132	0,428	1	548,571	4,5	46,6312	4,643
6,509	1,141	1,93	0,786	0	214,286	1	2,7972	1,789
4,146	1,636	7,932	0,428	1	462,857	0	36,0024	0,932
20,994	7,855	1,284	0,551	4,5	462,857	4,5	35,322	4,643
5,849	1,93	8,216	0,428	0,789	497,143	1	2,5956	1,143
13,632	6,287	13,575	0,786	0	214,286	1	122,2144	5,289
6,056	1,427	0,638	0,428	4,5	171,429	1	30,002	0,143
7,012	1,273	2,721	0,428	0	137,143	4,5	5,376	0,932
4,206	2,139	4,075	0	4,5	300	4,5	6,72	1,578
5,849	1,504	10,932	0,428	0	600	4,5	6,9888	4,5
4,558	2,273	6,218	0,123	4,5	214,286	1	4,9056	0,143
16,427	1,561	0,209	0,428	1,143	420	1	12,4236	0,143
5,344	2,576	4,998	0,428	0,143	291,429	4,5	46,648	1,143
5,707	1,996	2,704	0,428	1	505,714	1	57,974	0,932
6,434	1,715	12,864	0,551	0	480	1	11,1636	5,289
4,784	1,853	6,578	0,123	0,066	197,143	4,5	21,6888	4,566
5,498	2,719	9,429	0,428	0	394,286	4,5	36,9992	5,289
3,918	1,493	1,429	0,066	0	274,286	1	30,058	6,789
21,994	3,71	11	0,428	0	180	4,5	0,756	2
3,624	2,787	10,647	0	0,143	368,571	4,5	0	4,643

6,376	2,51	1,361	0,428	4,5	180	4,5	62,9524	0,143
13,008	0,998	6,287	1,786	0,143	180	4,5	36,9992	0,066
2,117	2,57	0,429	1	0,132	102,857	1	12,1212	0,932
2,81	2,282	6,576	0,428	4,643	428,571	0,066	19,4544	4,566
4,852	1,572	1,209	0,428	1	428,571	1	113,9992	0,143
8,921	2,499	6,853	0,551	0	497,143	1	5,6448	1,143
4,183	0,33	0,627	0,066	0	248,571	4,5	294,0196	0,066
6,221	1,207	1,064	1,214	1	137,143	0,143	0,2268	0,855
5,438	1,919	7,998	0,428	0,789	394,286	1	26,7232	4,643
11,018	2,862	5,007	0,428	1	334,286	4,5	27,9832	1,789
7,649	1,638	6,132	0,428	4,5	111,429	1	10,0548	0,789
6,274	2,143	5,352	0,428	0,066	72,8571	4,5	0	1,789
5,724	1,721	9,275	0,428	0	385,714	1	15,33	6,789
2,972	1,853	4,572	0,428	0,143	154,286	4,5	27,7116	0,932
3,492	3,13	6,352	1	4,5	197,143	4,5	60,662	1,143
8,499	0,143	0,209	0,123	0	248,571	1	0	0,066
8,319	1,787	1	0,428	0	197,143	0,789	24,528	4,643
11,017	1,864	0,789	0,428	0	360	0	0	0,066
2,035	2,15	5,495	0,428	0	531,429	1	0,8064	5,289
11,807	2,578	1,209	0,428	0	222,857	1	2,688	1,143
3,986	1,218	0,286	0,123	0	497,143	0	3,066	0,572
6,078	2,062	3,141	1	4,5	162,857	0	4,1076	1,143
5,726	0,987	14	1	2	188,571	1	18,648	4,643
6,507	1,427	4,561	0,428	0	154,286	1	17,7912	0,143
5,061	2,205	4,407	0,428	4,5	137,143	1	53,613	4,5
5,348	1,853	9,064	1,428	0	360	4,5	4,8384	4,643
2,986	1,93	2	0,428	1	154,286	1	33,9528	4,643
5,638	1,71	2,264	0,551	0	300	1	9,0552	0,066
5,574	1,853	5,209	0,428	0	120	1	14,35	4,643
9,503	2,139	5,075	0,428	1	325,714	1	39,3204	0,789
5,352	1,141	10,352	0,428	0	291,429	4,5	0	0,789
7,019	1,275	2,209	0,428	0	265,714	4,5	10,752	0,143
5,497	2,864	1,064	0,428	0	300	4,5	46,62	0
6,298	3,719	10,93	0,428	0	514,286	1	170,7524	0,143
5,193	0,913	4,789	0,066	0	60	0,143	16,7902	1,789
4,775	0,704	4,721	1,214	1,143	257,143	0	18,648	1,578
12,864	2,647	3,721	1	0	128,571	1	10,752	0,143
9,006	2,576	1,429	0,123	1,789	214,286	1	28,6692	0,789
5,577	2,139	9,139	0,428	5,289	497,143	4,5	10,8612	5,289
5,64	4,852	3,787	0,428	4,5	222,857	0,143	4,3008	5,289
3,857	1,207	3,858	0,123	1,066	317,143	1	0,8064	5,289
2,634	3,431	3,93	0,066	4,5	334,286	1	11,2056	4,643
5,067	1,284	0,275	0,856	0,789	282,857	0,429	11,1552	0,209
2,972	2,721	8,499	0,066	1	317,143	0,429	2,1504	5,289
8,374	3,365	2,132	0,428	0	351,429	0	62,8012	1,143
8,227	4,231	9,932	0,428	1	180	4,5	0,8064	1,143
4,348	1,273	3,284	0,428	0	360	1	8,8704	5,289
6,57	1,141	1,504	0,856	0	205,714	4,5	18,0012	0

4,135	0,418	6,286	1	0,789	85,7143	1	13,0536	0,143
6,207	1,35	8,853	0,428	0,066	282,857	4,5	30,058	0,143
3,687	3,354	1,196	0,428	0,066	394,286	4,5	84,0056	0,789
2,469	0,638	3,932	0,123	0	428,571	0	5,1282	1,143
3,205	1,064	3,141	0,428	1	685,714	1	12,264	4,643
3,417	1,644	1,218	0,428	0,429	222,857	4,5	0	0,143
6,985	0,789	5,789	0,123	1	265,714	4,5	8,8704	0,143
2,966	0,561	2,996	1,428	1	394,286	1	21,3472	0,789
10,295	3,222	3,721	0,428	1	214,286	4,5	70,9772	5,289
7,644	4,775	2,143	0,428	0,143	102,857	1	36,0024	0,143
5,348	1,407	1,704	0,786	1	120	1	0	0,932
8,209	1,578	9,075	0,123	0	540	1	4,9056	0,066
5,148	1,721	0,855	1	0,143	102,857	0,066	4,662	0,143
6,646	1,932	2,504	0,856	0	222,857	4,5	0	0,143
5,647	2,073	9,924	0,428	4,5	94,2857	FAŁSZ	38,3712	4,643
5,051	0,921	11,93	1	1	377,143	1	15,4308	5,289
3,778	1,721	5,987	0,856	0	154,286	1	42,7644	0,932
7,284	1,209	2,209	0,123	0	77,1429	4,5	0	1,066
4,496	1,864	4,787	0,428	0,143	471,429	4,5	0,8064	1,143
4,774	1,998	2,132	0,428	0	120	4,5	8,5512	4,643
6,365	0,352	1,275	2	0,143	248,571	1	0,8064	5,289
5,074	1,996	4,57	1,428	1,066	480	1	32,8692	0,855
4,418	5,916	11,718	0,786	0	257,143	1	3,2256	4,643
4,772	1,284	1,987	0,428	0	171,429	1	37,296	1,578
5,08	2,13	6,132	0,428	1	137,143	4,5	48,0256	4,643
2,109	0,704	3,053	0,123	0	437,143	0	15,33	0,066
3,984	1,504	2,787	0,123	0	334,286	1	24,528	0,143
4,275	0,341	11,216	0,123	4,5	608,571	4,5	7,2156	0,143
5,647	1,207	5,578	0,551	4,5	308,571	0,143	50,6688	0,932
5,146	2,207	0,704	0,428	1	445,714	1	21,7686	0,932
3,26	2,71	8,564	0,551	0	180	0	5,376	1,066
3,326	1,284	6,141	0	0	51,4286	0	0,8064	0,932
7,293	2,273	4,284	0,428	4,5	377,143	4,5	6,9972	1,066
3,142	0	0,286	0,551	4,5	300	1	18,0012	0
4,544	1,218	9,075	0,786	1,143	308,571	1	0	1,218
5,726	0,341	10,275	0,786	0	480	4,5	254,0104	0,143
3,832	1,987	1,064	0,123	4,5	420	0,066	1,8396	4,643
6,251	2,93	0,921	0,428	0,789	154,286	0,143	5,754	4,643
6,841	1,721	1,198	0,428	4,5	308,571	0	2,688	0,143
4,206	2,007	3,075	0,123	0,143	214,286	4,5	4,8384	0,932
3,28	1,064	3,141	0,123	0	145,714	1	14,35	4,566
5,058	2,359	4,484	0,786	4,5	231,429	0,789	8,1564	5,289
4,197	0,561	4,638	0,428	0	102,857	4,5	5,4852	0,855
6,076	1,504	4,647	0,123	0	394,286	1	25,8552	6,789
11,581	2,073	4,939	0,428	0	325,714	0	2,688	0,572
2,917	0,781	3,495	0,189	4,566	94,2857	1	10,1304	4,643
7,363	2,576	1,275	0,551	0,429	162,857	1	0	1,789
3,702	0,484	9,141	0,786	0,066	38,5714	4,5	12,264	1,143

2,975	3,359	9,776	1	0	497,143	4,5	69,972	4,5
5,137	2,853	1,998	0,428	1	180	1	8,064	6,789
5,566	2,293	3,932	0,428	0	214,286	1	0	5,289
4,928	0,066	2,932	0,123	1	462,857	1	8,1732	4,643
6,278	2,576	8,341	0,428	0	137,143	4,5	23,926	1,143
6,774	2,576	5,945	0,428	0	120	0	35,4872	5,289
2,271	0,921	0,132	0,428	0,143	180	0,789	28,7784	5,289
5,496	2,207	8,073	0,786	4,5	300	1	123,6172	5,289
3,783	2,064	2,064	0,123	1	445,714	4,5	4,5276	4,643
7,698	2,787	4,776	0,428	0,066	497,143	4,5	19,4376	4,643
4,926	1,93	10,987	0,428	1,066	197,143	4,5	15,5232	1,578
1,691	1,776	1,264	0,428	0,143	600	0,789	63,4592	0,143
5,06	6,573	2,855	0,428	6	240	0	300,02	6,066
5,583	2,207	1,429	0,856	1	120	1	0,8064	4,643
3,118	1,864	8,216	0,066	4,5	317,143	4,5	5,0232	6,143
5,344	1,13	1,71	0,428	4,5	462,857	1	126,6832	4,643
4,408	1,209	0,286	0,123	1	120	4,5	0,8064	4,643
5,489	1,141	3,13	0,428	0	368,571	1	48,3112	4,643
7,952	1,721	1,789	0,428	4,5	257,143	0	32,9168	0,789
4,443	5,575	0,998	0,428	0	377,143	6	36,0024	0,789
2,48	0,352	3,495	0,066	0	317,143	4,5	5,4684	4,643
5,443	2,007	4,429	1	1	60	1	0	0,932
7,935	2,645	9,293	0,428	0,855	385,714	1	24,2536	0,143
1,606	0,561	13,787	0,428	1,066	574,286	0	33,3872	5,289
3,86	3,51	3,781	0,856	0	257,143	1	18,648	4,929
8,679	2,51	4,367	0,428	0	282,857	0,789	21,714	0,789
4,66	1,57	1,787	0,786	4,5	240	0,429	14,4522	0,143
5,364	2,499	1,932	0,428	4,5	445,714	4,5	16,7244	0,789
7,807	0,998	2,853	0,428	0,789	437,143	1	30,7692	0,143
8,146	1,352	3,504	0,428	1	72,8571	1	32,634	1,789
5,063	1,853	5,418	0,856	0	377,143	4,5	19,5804	4,643
5,009	1,985	1,998	0,428	4,5	308,571	1	29,6632	5,289
6,203	2,064	2,919	0,428	0	214,286	1	3,4944	4,643
3,32	2,141	1,132	0,428	1	548,571	0	4,5276	4,643
4,073	1,286	0,143	0,123	1	240	0	2,9148	0,066
0,891	2,064	2,064	0,123	1	360	0	0,8064	5,289
2,832	0,704	3,198	0,066	0	360	1	0	6,143
21,589	10,721	1,79	0,909	0,429	62,1429	0	0	1,578
13,266	1,13	6,495	1,214	4,5	120	1	6,9384	0,789
7,012	2,787	1,864	0,428	0	308,571	4,5	2,688	1,789
15,295	1,493	3,075	0,428	0	154,286	1	67,298	0,143
7,438	1,361	6,198	0,786	0	171,429	1	12,1212	5,289
3,623	0,638	2,367	0,856	0,429	214,286	4,5	41,4204	5,289
7,302	1,919	10,998	0,786	4,5	137,143	4,5	6,2748	4,643
7,164	1,57	2,075	0,428	0,429	514,286	1	0	4,643
6,545	1,853	4,427	0,786	0,429	214,286	4,5	38,311	1,218
6,149	0,429	1,504	0,786	0	617,143	4,5	12,264	5,289
5,423	0,847	1,858	1,428	0,789	368,571	4,5	2,688	0,932

4,834	1,998	3,418	0,428	0	480	4,5	17,038	1,143
2,601	2,367	5,299	0,428	0	420	0,429	0	5,289
4,212	2,141	1,35	0,428	0	291,429	1	14,35	4,643
8,772	1,93	1,921	1,066	4,5	77,1429	1	31,0296	0,143
3,258	1,141	5,275	0,551	0	522,857	6	8,5596	0,932
14,778	1,275	0,352	0,551	0,066	385,714	1	249,34	0
4,071	1,075	1,075	1,066	1	411,429	4,5	12,1212	0,066
5,271	1,198	4,789	1	0	248,571	1	6,1824	0,143
6,203	2,062	0,932	0,428	1	274,286	1	30,002	4,643
4,632	2,062	3,284	0,428	0,789	180	4,5	30,3212	1,789
13,008	0,275	1	0,856	4,5	248,571	1	60,1356	0,143
4,851	1,644	1,429	0,123	1	368,571	1	15,2796	0,209
6,511	1,075	0,066	0,123	0	300	1	5,5944	0,143
6,508	1,644	1,484	1,214	0,143	60	4,5	4,032	1,143
2,075	2,209	5,572	0,428	0,143	214,286	4,5	2,688	4,643
5,07	1,864	3,064	0,428	1,143	342,857	0	5,376	1,789
4,72	0,932	1,218	0,428	0	214,286	1	16,128	0,789
6,067	2,207	9,564	0,856	0,789	257,143	1	4,5276	4,643
13,586	0	1,644	1,428	1	180	4,5	29,6632	0,789
3,208	1,067	12,704	0,369	1	51,4286	0,429	0	6,143
6,498	2,565	6,933	0,428	0,429	240	1	49,686	5,289
11,088	4,566	3,143	0,123	1	94,2857	1	2,7972	0,789
5,869	2,15	3,653	0,428	4,5	120	0,429	23,5536	1,143
4,557	2,93	14,578	0,428	4,5	317,143	1	32,7992	5,289
4,124	1,218	12,007	0,066	0,789	334,286	4,5	14,1036	0,286
3,846	1,416	1,987	1	0	497,143	1	27,3084	0,855
9,216	3,365	6,143	0,428	0	411,429	4,5	15,3384	5,289
2,12	1,987	4,13	0,123	4,5	488,571	1	2,1504	5,289
8,522	1,284	3,064	1,786	0,143	197,143	1	10,4328	0,286
5,557	1,985	4,495	0,123	0,066	540	4,5	16,4052	0,143
5,869	1,636	8,066	0,428	0,789	180	1	0	5,289
8,839	0,987	10,864	0,428	4,5	180	1	3,4944	6,066
4,632	1,207	4,132	0,066	1	428,571	6	6,9972	0,066
8,209	1,427	1,075	1,214	0	257,143	1	1,6128	0,143
6,649	0,704	3,572	0,428	0,143	180	0,789	0	1,143
4,487	1,987	4,407	0,123	0	317,143	0	2,688	0,143
4,469	1,853	1,286	0,551	0	300	1	23,058	1,218
5,795	1,996	3,636	1,214	0	197,143	1	125,2902	0,932
3,994	0,286	1	0,786	0,066	445,714	4,5	0	0,789
5,289	1,284	1,001	0,428	0	257,143	4,5	42,1344	4,643
4,06	1,919	7,073	0,428	0,789	197,143	4,5	3,066	0,789
7,363	1,776	2,361	0,856	0	60	4,5	1,8396	0,143
5,79	1,787	2,418	0,428	0	154,286	4,5	6,9972	0,143
6,849	3,288	3,853	0,909	0,143	94,2857	4,5	47,8604	1,578
4,286	1,853	5,93	0,428	1	257,143	4,5	7,2156	1,789
4,559	2,433	3,075	0,428	0	94,2857	1	15,0612	1,143
7,363	1,853	5,209	0,428	1	77,1429	4,5	6,132	1,218
3,489	3,442	6,786	0,428	4,5	162,857	1	41,958	5,289

6,518	1,93	4,209	0,428	0	180	1	11,6592	4,643
5,138	1,93	7,284	0,428	0,789	154,286	4,5	2,7972	1,143
3,184	1,207	3,418	0,066	0	394,286	1	0	4,643
3,417	1,13	1,143	2,5	0,209	137,143	4,5	0,9828	0,572
3,052	5,193	3,416	0,428	4,5	411,429	1	0	4,643
6,928	2,565	9,215	0,428	0	77,1429	4,5	0	0,789
4,071	1,218	6,286	0,428	4,643	240	0	0,8064	5,289
8,121	5,784	8,704	0,123	0	317,143	1	5,4852	0,855
4,506	1,275	4,932	0,551	0	214,286	1	24,528	0,143
1,746	1,787	7,367	0,428	0	257,143	0,789	30,0552	0,143
3,423	4,566	0,143	0,123	0	334,286	0	0	4,643
8,58	1,864	9,996	0,428	0	317,143	4,5	8,064	5,289
4,924	2,71	2,578	0,428	4,5	428,571	4,5	37,296	1,143
6,797	2,073	1,721	0,428	0	222,857	1	18,396	5,289
7,884	1,71	5,218	0,428	0	240	4,5	2,7972	0,932
6,695	0,693	0,341	0,428	0	300	4,5	4,1412	0,789
7,869	2,284	3,433	0,123	1,066	102,857	0	60,004	1,143
4,995	1,484	0,627	1,428	0	411,429	0	47,964	0,143
6,566	0,352	0,418	2,5	0,789	188,571	6	36,134	0,143
6,161	2,499	6,433	1	0	300	4,5	0	4,643
5,553	2,13	2,064	0,246	0,066	437,143	1	33,0834	0,066
1,548	2,653	6,561	0,428	0	471,429	1	3,4944	0,143
8,306	3,005	7,064	1,284	1	462,857	1	17,7492	1,066
5,059	1,919	0,921	0,551	1	240	0	37,0552	4,643
4,561	2,642	6,93	1	4,5	497,143	1	41,958	0,143
2,562	1,721	1,352	0,123	0,143	102,857	4,5	28,7784	0,143
10,866	2,425	6,073	0,786	0,495	231,429	1	12,1212	5,289
6,651	2,578	8,064	0,428	0	120	4,5	2,688	5,289
2,92	1,787	3,418	0,123	0	360	1	15,33	0,143
6,943	1,787	5,932	0,428	5,289	17,1429	4,5	5,376	0,789
7,708	1,855	8,064	1	1	480	4,5	7,5264	0,143
3,247	0,858	1,932	0,428	4,5	360	6	5,5944	0
4,777	0,924	0,275	0,123	1	317,143	0	3,4944	0,143
4,935	1,35	6,998	0,123	0,066	445,714	1	0	4,643
8,137	2,787	0,561	0,428	0	222,857	4,5	76,622	0,789
2,689	1,133	3,207	0,132	0	102,857	1	7,2156	0,572
4,795	7,284	0,143	0,255	0	617,143	6	21,5656	0,143
8,102	3,299	9,734	0,786	4,5	411,429	4,5	2,9148	5,289
9,793	2,141	4,561	0,123	1,143	240	0,789	22,9824	1,143
4,915	2,367	1,919	0,494	0	222,857	4,5	34,4792	0,789
6,075	0,407	3,299	0,123	1	428,571	4,5	3,8808	1,066
5,851	1,996	1,789	0,428	0	471,429	1	17,6652	6,143
5,269	2,141	4,495	0,428	1	248,571	1	8,463	1,143
6,782	1,284	6,429	0,786	0	231,429	4,5	11,2602	0,143
6,552	1,13	14,355	1	0,143	162,857	4,5	0,8064	1,578
4,124	1,71	6,704	0,066	0	274,286	4,5	29,19	6,143
6,355	1,853	3,066	0,617	0,143	300	0,066	40,754	1,218
5,203	2,073	1,218	0,123	1	377,143	4,5	4,032	0,143

15,791	1,57	1,066	0,551	0	60	4,5	15,0612	0,066
6,506	1,842	1,495	0	0,066	90	1	1,8396	4,643
5,489	1,13	9,288	1	0,066	94,2857	4,5	8,064	0,932
5,056	1,721	5,855	0,428	4,5	445,714	4,5	0	1
3,335	1,919	7,995	0,856	0	471,429	1	26,3214	0,143
6,374	2,286	1,132	0,123	1	180	1	0,8064	1,789
5,561	1,341	11,561	0,979	0	351,429	4,5	8,3412	1,789
2,711	5,641	10,632	0,551	0	334,286	0	24,528	0,143
5,197	1,985	5,207	2,5	4,643	120	0	3,066	4,643
3,926	5,927	7,787	0,786	0	282,857	4,5	72,7692	5,289
5,155	2,007	3,064	0,123	1	197,143	0	5,376	1,789
3,568	1,721	7,284	0,428	0	180	0	2,688	0,143
6,509	1,996	6,284	0,428	1,143	214,286	4,5	13,9944	0,143
3,346	1,284	4,064	0,123	4,5	274,286	0,429	20,5464	6,143
4,08	1,273	8,364	0,428	0	480	4,5	19,0344	1,578
4,702	2,721	1	0,856	1,143	317,143	0	0	1,143
7,003	2,141	4,484	0,551	0	222,857	1	100,002	1,789
4,283	2,928	4,787	0,786	0	197,143	0	47,5524	1,218
3,005	1,721	1,855	0,856	0,789	265,714	1	35,378	0,143
4,366	1,493	11,207	0,123	0	214,286	6	10,038	6,429
5,715	1,196	0,275	0,428	0	360	1	0	1,143
3,478	2,499	4,352	0,786	4,5	248,571	4,5	1,8396	4,643
6,801	1,57	2,075	0,786	1	505,714	4,5	23,1924	0,143
3,416	4,775	0,932	0,428	0	137,143	1	15,0612	0,143
3,265	1,853	6,579	0,428	1	582,857	1	8,064	0,572
3,983	1	10,218	0,123	0	265,714	4,5	6,9888	0,789
9,89	2,007	3,286	0,428	1	0	0	0,8064	1,789
2,898	6,352	4,416	0,066	0	480	4,5	19,7232	4,643
1,71	2,653	6,209	0	0,789	325,714	4,5	10,038	4,643
7,361	1,932	2,286	0,428	0	51,4286	1	9,324	1,789
3,968	0,913	6,341	0,123	0,789	377,143	1	36,5232	4,643
4,271	1,35	3,561	2,5	0	162,857	0	17,6106	1,143
4,201	1,427	6,721	0,428	0,789	368,571	4,5	75,9752	0,143
9,775	0,484	13,286	0,428	0	548,571	6	10,836	0,066
7,447	0,341	7,141	0,123	4,5	214,286	1	14,0364	1,143
5,195	1,35	4,207	1,786	4,5	180	0,429	8,3412	4,643
5,788	0,638	3,209	0,123	0,429	300	1	2,7972	5,289
4,469	1,132	0,484	0,494	0	300	6	15,0108	1,066
3,274	1,644	0,638	0,428	1	240	4,5	5,1282	0,143
3,687	1,207	2,352	1	0,495	180	4,5	49,4172	0,143
4,357	0,429	0,638	0,123	0	222,857	1	7,2156	0,066
4,561	3,354	4,847	0,428	0,066	214,286	1	81,1972	4,5
10,149	0,561	1,341	0,786	0,143	300	1	91,35	0,789
6,513	1,141	6,921	0,428	0	240	0	24,528	1,143
6,213	0,987	2,007	0,551	1	162,857	1	0,756	0,143
3,406	2,139	6,275	0,786	6	471,429	1	13,9944	6,066
12,852	3,431	7,919	0,856	0	497,143	0,066	20,4372	6,143
2,689	1,264	2,484	0,066	4,5	548,571	4,5	18,396	6,143

4,634	1,855	1,286	0,428	0	420	1	6,9384	0,932
7,15	1,13	8,284	0,123	0,143	180	4,5	9,324	1,143
5,614	1,273	1,484	0,428	1	120	0	5,376	1,789
10,086	3,288	5,352	0,786	0,789	102,857	1	13,6668	1,578
13,017	6,144	2,066	0,066	4,5	334,286	0,143	58,6572	0,789
6,223	1,064	1,075	0,856	0	60	4,5	10,2564	0,143
5,058	1,427	7,71	0,428	1	300	1	20,2356	1,143
1,053	1,075	3,286	0,428	0	205,714	4,5	0	0,789
6,157	2,785	2,066	0,428	0	180	1	43,442	4,643
6,627	1,921	6,209	0,428	0,789	248,571	4,5	18,0012	0,066
4,215	0,275	0,132	0,246	0	514,286	0,143	51,2904	0,066
15,297	2,207	2,998	0,123	1	300	4,5	2,7972	0,066
5,632	0,495	2,132	0,189	6	265,714	1	57,0612	6,789
2,9	1,493	4,495	0,123	1	497,143	1	23,4192	4,566
3,269	1,284	1,075	0,856	0,789	180	1	38,738	1,578
11,429	2,708	3,853	0,428	1	94,2857	4,5	130,6088	1,143
5,553	1,787	1,075	0,494	4,5	120	1	6,5016	0,143
5,443	1,57	1,064	0,428	1	120	1	7,3962	0,143
4,564	1,35	15,644	0,066	0	360	4,5	8,2824	0,932
4,919	1,35	2,066	0,856	1	471,429	1	5,4684	0,066
6,697	1,427	3,075	0	1	402,857	4,5	4,0068	5,289
4,412	2,053	15,284	0,123	6	180	4,5	11,382	4,566
10,639	5,995	6,418	0,123	0	274,286	4,5	13,0704	1,789
6,575	2,132	1,998	0,246	0	257,143	0,789	10,9704	1
6,563	1,93	0,987	0,428	4,5	445,714	4,5	8,9712	1,143
4,073	1,064	4,787	0,123	4,5	360	0,789	2,5956	4,643
8,697	2,13	5,196	0,428	0	428,571	0,066	13,9272	0,143
9,566	1,853	3,721	0,123	0,495	137,143	1	4,662	1,578
3,766	1,196	3,93	0,428	4,5	265,714	0,143	3,4944	1,789
7,953	1,35	5,919	0,856	1	180	1	61,4432	0,143
3,503	2,293	1,853	0,428	1,066	265,714	0	36,3972	0,066
5,355	2,576	4,207	0,428	0,066	240	0,143	0	1,218
5,205	1,776	0,855	0,428	0	300	1	0	0,789
4,632	1,001	0,275	0,066	0,789	514,286	0,143	3,6792	0,066
7,504	1,559	6,299	0,428	4,5	77,1429	1	57,6352	1,789
5,931	2,13	6,932	0,428	0	85,7143	1	0	5,289
7,227	2,787	6,007	0,551	0,789	282,857	1	2,7972	1,789
5,717	1,921	1,998	0,123	1	120	0	12,096	0,066
6,003	2,576	0,352	0,428	0	222,857	0,429	7,9716	0,143
1,924	2,644	8,789	0,856	0	79,2857	1	0	5,289
5,086	2,51	5	0,428	0	222,857	4,5	7,9716	5,289
5,489	2,075	12,367	1,786	4,5	282,857	4,5	0	0,143
5,861	2,708	0,932	0,428	4,566	497,143	4,5	36,0024	1,789
5,344	1,284	0,132	0,123	0	514,286	1	7,9716	0,143
12,081	6,364	3,352	1	0,066	300	1	0	4,643
5,576	2,576	5,495	0,428	0	180	1	1,8396	0,143
3,052	1,55	0,209	0,428	4,5	411,429	4,5	24,528	0,789
2,821	2,293	1,418	0,066	0,286	274,286	1	27,594	6,789

5,566	1,919	5,484	0,428	0	55,7143	4,5	9,5508	4,643
5,212	1,273	1,429	0,428	0	334,286	4,5	21,6566	0,066
4,638	1,141	8,293	0,123	0	300	4,5	0	4,643
5,054	1,119	2,13	0,428	0	300	4,5	24,2424	1,143
2,975	1,504	2,275	0,123	0,143	531,429	1	14,9184	0,932
22,942	3,433	6,361	0,123	2	154,286	4,5	5,4684	1,143
3,489	1,853	8,209	0,856	0,855	274,286	1	49,686	1,789
10,936	4,918	4,141	0,856	4,5	411,429	1	33,4404	4,643
12,307	0	1,143	3,5	0,789	377,143	0,789	1,8396	0,789
11,417	1,341	1,143	1	0,789	141,429	1	93,5452	0,143
8,64	2,708	2,001	0,428	0,143	120	4,5	24,2424	5,289
10,598	1,064	1,132	0,428	1	480	1	0,8064	0,209
5,195	1,932	3,075	0,786	1	514,286	1	27,594	1,789
4,783	1,787	1,064	0,123	1	102,857	1	55,944	0,143
4,993	1,427	1,721	0,428	1	411,429	4,5	2,7972	0
5,403	1,064	1,644	0,123	0	480	1	17,8752	0,209
8,785	1,853	0,932	0,428	0	360	4,5	20,6052	0
4,282	1,35	0,198	0,428	4,5	471,429	4,5	55,944	0,143
4,794	4,775	7,286	0,123	0	480	1	3,066	1,143
2,502	1,075	1,209	0,428	0,789	102,857	1	0	0,789
3,632	1,998	7,932	0,428	1	428,571	1	61,8072	1,789
12,647	5,575	7,078	0,428	0,429	68,5714	1	72,0048	1,578
2,634	1,075	0,132	0,066	0	222,857	0	1,8396	0
3,142	0,495	2,198	0,123	0	197,143	1	6,132	0,932
5,781	2,787	0,352	1	1	445,714	4,5	27,9888	4,643
4,715	0,484	1,064	0,494	0	180	4,5	46,3232	1,143
8,363	2,141	4,653	0	4,5	120	1	61,8436	0,789
1,748	0,396	1,064	0,066	0	334,286	4,5	0	0,066
6,355	1,207	8,218	0,428	0	257,143	1	41,958	0,932
4,717	0,781	1,284	0,428	1	411,429	4,5	27,3252	1,143
11,083	1,57	0,855	0,428	0,143	514,286	0,143	22,764	0,066
8,653	6,573	6,578	0,428	4,566	240	4,5	5,376	5,289
6,496	1,141	1,787	0,246	0	334,286	4,5	0,672	0,066
2,623	0,913	6,855	0,066	0	342,857	4,5	12,096	6,143
5,337	1,273	1,93	0,551	1,789	317,143	1	15,5232	1,143
4,269	1,207	0,495	0,428	1	385,714	4,5	5,5944	5,289
8,773	1,275	1,789	0,123	0	128,571	1	0	0,066
6,304	2,499	7,275	0,551	0,429	420	4,5	1,8564	0,932
10,686	1,919	4,156	0,428	0	171,429	4,5	16,506	5,289
7,649	3,642	3,495	0,551	0,143	377,143	4,5	107,9932	4,643
4,915	1,339	0,473	0,551	0	154,286	4,5	7,3164	0,066
7,552	2,064	6,361	0,551	1	180	1	24,1304	0,789
5,354	1,13	1,064	0,428	0,789	368,571	1	0	6,789
4,966	0,759	0,855	0,123	4,5	445,714	1	49,4452	5,289
7,225	2,576	3,361	0,428	0	351,429	4,5	54,1352	0,572
4,997	1,352	3,361	0,428	0	394,286	1	16,0986	1,143
6,794	1,996	3,361	0,428	0,066	222,857	1	2,1504	5,289
7,289	5,432	1,998	0,246	0,429	411,429	1	6,1824	1,578

3,434	1,144	3,001	0,123	0	308,571	4,5	0	4,643
3,703	1,842	2,998	0,856	0	480	1	17,8444	1,789
3,632	1,919	3,721	0,428	4,5	488,571	0	31,038	0,143
5,434	3,499	0,418	0,428	0	582,857	4,5	6,132	0,143
4,706	1,71	5,644	0,428	0,789	257,143	0,789	24,1332	0,143
8,735	2,367	1,932	0,551	0	137,143	4,5	84	1,429
5,005	3,431	1,286	0,428	1	154,286	0	18,0012	0,143
4,612	1,787	0,286	0,428	0	377,143	1	30,66	0,143
4,142	1,075	1,286	0,123	0	274,286	4,5	6,132	0,932
5,48	1,207	8,341	0,132	0,789	77,1429	4,5	4,5276	1,066
10,349	1,776	5,141	0,551	4,5	205,714	1	2,7972	0,932
9,89	1,789	2	0,428	0,789	171,429	1	10,5042	0,143
5,548	2,996	4,495	0,428	0	137,143	0	316,596	1,789
6,001	0,932	1,721	0,123	0	120	4,5	30,002	5,289
10,22	2,864	7,218	0,786	4,5	222,857	1	5,4852	4,643
7,438	2,576	6,867	0,428	1	77,1429	0	0,8064	0,789
3,428	1,35	11,789	0,551	0	377,143	1	39,984	5,289
4,127	2,93	1,787	0,786	0	497,143	4,5	21,462	0,143
7,688	2,216	1,93	0,428	0	214,286	0	0,378	5,289
3,064	0,066	0,921	0,428	0	154,286	1	4,662	4,5
5,28	3,499	0,921	1	4,5	300	1	18,102	0,143
5,195	2,787	1,776	1,428	0,429	394,286	1	5,4684	0,066
4,212	1,207	7,35	0,066	4,5	334,286	4,5	16,926	4,643
3,368	1,361	11,721	0,123	4,5	411,429	1	21,0084	4,5
5,63	3,148	2,998	0,428	4,5	325,714	1	84,6552	1,143
9,371	0,429	0	0,428	0	437,143	4,5	27,3252	0,143
2,978	1,141	2,921	0,066	1	240	4,5	0	4,643
6,649	3,642	0	0,428	4,5	240	4,5	39,634	5,289
7,647	2,631	4,93	0,494	0	197,143	4,5	0	0,789
6,638	2,787	1,864	0,856	4,5	282,857	1	30,002	0,789
3,917	1,644	1,776	0,428	0	420	1	13,9944	0,066
4,615	2,787	10,073	0,428	4,5	402,857	0,143	8,064	4,643
4,557	1,919	6,196	0,551	1,789	385,714	1	17,2564	5,289
6,286	1,064	2,218	0,428	0	308,571	0	0,2268	5,289
9,646	1,218	0,561	0,428	0	120	0	53,6452	0,143
6,223	0,352	2	1,551	0	154,286	0	0	0,143
5,135	1,71	3,787	0,856	0,789	137,143	4,5	5,8716	1,218
6,35	1,132	4,286	0,428	0,066	137,143	4,5	10,4622	1,143
6,86	1,284	1,864	0,428	0	274,286	1	252,0168	0,789
5,7	0,33	13,207	1	0	385,714	4,5	20,986	6,789
5,863	2,642	3,293	0,428	1	197,143	0	0,8064	1,218
6,293	2,721	1,141	0,786	1	480	0	0	0,932
4,792	1,495	2,33	0,428	0	120	1	0	0,143
5,798	1,855	4,198	0,551	0	120	0,066	25,1412	1,789
3,063	2,216	12,576	0,066	4,5	274,286	1	8,064	0,143
5,858	1,209	5,429	0,428	1	197,143	0	9,324	1,143
8,674	2,51	2,721	0,123	1	265,714	1	9,9036	1,143
3,425	0,99	1,207	0,428	4,5	531,429	1	46,648	1,143

5,847	2,282	5,713	0,428	0	514,286	1	5,754	0,789
6,579	1,996	3,787	0,428	0	257,143	1	0	1,066
2,986	2,433	4,066	0,428	0	282,857	0,429	0	0,932
1,251	1,218	0,198	0,123	0	420	6	60,004	0,143
7,858	1,207	4,064	0,428	0	214,286	1	7,4592	4,643
4,564	1,625	5,209	0	1	197,143	4,5	18,648	1,143
6,269	5,498	1,143	1,346	0	240	4,5	28,4004	0,143
4,772	1,064	6,341	0,428	4,5	240	1	0,8064	0,143
3,28	1,996	5,498	0,856	0	291,429	4,5	33,0022	1,578
11,244	3,431	3,275	0,123	0,132	120	1	11,9364	1,578
1,122	2,271	5,064	0,066	0	317,143	1	5,376	0,066
6,078	1,787	0,932	0,428	0	180	0	4,662	0
5,772	0,418	1,715	0,428	0,789	411,429	1	0,8064	4,643
9,94	1,352	10,787	0,428	0	514,286	4,5	5,376	0,143
5,643	2,367	9	0,786	0	282,857	1	8,3412	1,789
4,863	1,284	1,55	0,428	0	102,857	1	41,958	0,932
6,708	2,853	5,442	0,428	1	240	0,066	28,2296	0,932
6,952	1,35	5,141	0,123	0,789	300	1	14,539	0,286
5,642	4,5	6	0	0	385,714	1	0	0,066
4,5	1,141	7,218	0,428	1,218	171,429	4,5	1,9908	1,143
6,193	2,284	0,855	1	4,5	600	4,5	2,1504	0,143
3,197	2,119	2,484	0,856	1	102,857	1	11,2392	1,143
5,935	1,71	3,218	0,428	1	197,143	1	46,648	0,572
3,052	0,418	7,71	0,066	0	317,143	4,5	24,7212	0,143
4,911	0,921	1,275	0,123	0	454,286	4,5	0,8064	0,789
4,285	1,636	1,987	0	0,143	94,2857	0	0	0,932
3,983	1,141	2,855	0,123	0,143	360	0	14,7924	5,289
5,704	1,141	0,561	0,428	0	180	1	6,9972	4,643
3,283	1,493	2,13	0,123	4,5	437,143	1	14,5656	0,789
8,087	4,576	3,79	0,428	1	111,429	4,5	93,24	4,643
7,694	1,352	4,143	0,428	0	137,143	1	9,324	0,572
5,856	1,207	1,064	0,428	0	411,429	4,5	5,8632	0,143
16,651	2,796	10,585	0,786	0	120	4,5	1,1844	6,789
3,772	0,913	4,918	0,123	0	291,429	0	5,376	0,143
3,043	2,642	6,561	0,494	4,643	385,714	1	54,1072	1,143
2,254	1,913	9,429	0,428	0	180	4,5	8,1564	0,932
1,614	1,352	1,855	0,066	1	480	0,066	0	4,566
4,869	2,576	1,352	0,428	1	497,143	1	54,0792	0,932
4,292	2,007	2,132	0,428	0,429	137,143	0,429	18,648	0,495
4,401	1,776	1,064	0,551	4,5	205,714	0	4,5864	0,132
4,568	1,218	1,352	0,123	0	531,429	1	15,2208	0,143
10,228	0,572	3	0,428	1	257,143	0	36,0024	0,789
6,269	3,433	5,578	0,786	0	480	4,5	13,5016	4,643
7,427	1,218	7,119	0,856	1	120	1	2,688	1,218
6,87	1,713	5,284	0,123	0	137,143	0,429	5,376	4,643
4,78	6,867	17,007	0,428	0	514,286	1	2,688	5,289
5,722	2,51	2,789	0,551	0,143	171,429	1	0	1,789
8,641	1,275	1,066	0,066	0	360	4,5	6,1824	0

3,041	3,651	3,13	0,123	1	531,429	4,5	43,1284	0,143
2,403	1,341	0,341	0,428	1,143	274,286	4,5	16,128	4,643
3,491	1,207	7,207	0,428	1	128,571	1	20,979	1,143
7,55	2,15	2,484	0,428	1,066	197,143	1	2,268	1,143
3,412	2,644	1,578	0,786	1	514,286	4,5	8,064	5,289
4,049	1,71	0,341	0,428	0	505,714	1	13,5282	1,578
6,571	1,864	7,367	0,428	4,5	480	0	1,89	1,578
3,7	2,433	0,209	0,428	4,5	120	1	3,4944	4,643
4,984	1,482	4,853	0,066	0,429	514,286	1	8,9628	4,643
1,966	1,141	0,132	0,123	0	360	1	0	0,143
4,786	1,71	1,864	0,551	0,143	265,714	0	6,3672	6,789
9,229	2,062	2,578	0,428	1,143	437,143	4,5	31,9872	0,932
5,493	2,141	1,209	0,123	0	120	1	8,1732	1,143
6,284	1,218	1,284	0,123	1	360	4,5	23,926	1,143
6,142	2,062	1,053	0,428	0	214,286	1	9,3282	1,578
4,338	6,144	0,341	0,123	0	214,286	1	4,4184	6,143
5,069	1,721	4,066	0,786	0	360	0	2,688	0,789
4,632	1,853	0,209	0,123	0	197,143	1	2,7972	0,066
5,423	2,064	4,007	0,428	1	120	4,5	40,8632	0,932
7,474	5,784	7,51	0,428	0	445,714	4,5	46,648	6,789
5,498	1,427	0,921	0,551	0,143	514,286	1	6,132	0,066
4,139	0,855	5,578	0,856	0,066	480	1	4,662	1,789
6,152	1,286	1,218	0,428	1	300	0	0,8064	4,643
8,98	0,987	1,066	0,856	0,209	308,571	0,789	268,6376	0,066
4,5	3,005	7,066	0,428	1,789	214,286	4,5	19,32	1,789
5,575	1,987	8,218	0,428	0,066	334,286	1	0	5,289
5,346	3,007	9,284	0,786	0	480	0	21,7686	1,789
3,979	1,143	0,286	0,123	0	480	1	300,02	0
4,414	1,644	1,198	0,428	1	548,571	0	4,032	0,932
11,999	1,262	5,352	1	1	240	1	93,9708	5,289
2,249	1,064	5,286	0,428	0,789	300	0,789	8,8704	0,932
5,928	1,864	0,855	0,428	1	137,143	0	25,8552	0,143
6,666	2,644	0,286	0	1	282,857	4,5	0	1,143
13,086	2,51	1,721	0,246	0	257,143	FAŁSZ	35,5964	0,789
4,418	1,429	12,495	0,428	4,5	180	0	6,132	4,643
6,429	1,985	1,196	0,551	0	197,143	4,5	33,8142	0,143
4,333	0,627	6,427	0,066	1	394,286	4,5	8,1984	4,643
1,9	1,493	0,352	0,428	0	274,286	0	27,972	4,643
7,506	1,493	4,93	0,551	4,566	531,429	4,5	34,9692	1,143
9,066	0,987	0,198	0,123	1	531,429	4,5	18,396	0,789
5,364	1,636	4,139	0,428	4,5	428,571	4,5	13,195	0,143
6,636	2,842	3,653	0,428	4,5	385,714	1	10,2144	1,789
5,128	2,367	9,653	0,428	4,5	317,143	0	28,9044	6,143
6,803	1,284	1,932	0,428	0	137,143	1	29,6632	1,789
1,721	0	8,932	0	4,5	497,143	1	12,264	1,789
7,875	1,93	3,141	0,428	1	162,857	0	13,986	1,143
5,992	1,702	6,132	0,856	1	222,857	4,5	1,1844	1,066
6,221	2,207	1,715	0,428	0	360	1	0	0,143

6,153	1,352	9,284	0,428	0	300	4,5	4,8384	4,643
6,484	2,996	5,996	0,428	0	522,857	4,5	114,6544	0,789
10,283	2,073	11,553	1	0	120	1	0	1,789
8,275	1,996	3,987	0,428	4,5	240	0	28,3108	4,643
5,193	1,493	0	0,428	1	497,143	1	8,064	4,566
3,994	0,998	0,341	0,428	0	240	1	8,5512	0,066
2,414	1,066	1,855	0,123	0	582,857	0	0,8064	0,143
6,273	2,064	0,932	0	0,789	51,4286	4,5	2,688	0,932
5,926	1,638	4,367	0,786	0	222,857	4,5	0	5,289
10,143	3,359	12,51	0,856	0	137,143	4,5	6,72	5,289
7,007	3,576	5,299	0,428	1	180	0,789	0,8064	1,143
6,238	1,71	1,776	0,551	0	317,143	0,789	3,8724	0,789
3,643	2,853	2,264	0,786	0	240	1	12,8352	1,143
5,917	0,407	7,998	1,428	1	445,714	6	0,8064	1,066
7,596	1,789	8,361	1	0	445,714	4,5	120,008	1,143
5,05	2,71	14,073	0,428	1	171,429	4,5	6,72	0,789
3,073	1,141	11,284	0,123	0	120	1	0	5,289
2,928	1,35	8,132	0	0	514,286	4,5	2,016	5,289
2,329	0,715	14,576	0	4,5	394,286	1	17,8444	0,789
8,082	1,361	2,143	0,428	1	325,714	1	5,376	0,789
4,36	6,63	0,429	0,428	0	197,143	0	0	1,143
2,909	1,996	1,855	1	1	325,714	1	95,662	1,143
6,429	0,627	4,709	0,428	0	120	1	0	0,066
3,919	2,056	8,638	0,428	4,566	377,143	1	2,1504	0,932
6,474	0,275	0,921	0,066	0	334,286	4,5	0,4536	0,143
7,161	2,51	8,93	0,428	1,066	154,286	4,5	26,1072	1,143
5,35	1,919	1,776	0,428	0	445,714	1	30,058	4,643
3,348	0,209	10,209	0	0	180	4,5	19,7232	0,789
4,124	1,064	2,776	0,123	1	325,714	1	12,9024	0,789
7,662	1,053	4,932	0,066	0,132	411,429	4,5	16,4808	0,855
3,928	2,007	3,932	0	1	274,286	1	0	0,932
8,099	2,51	2	0,428	0,789	16,4286	0,789	0	0,789
7,003	0,55	6,209	0,246	0,429	240	1	0	5,289
32,216	7,222	8,864	0,786	0,429	98,5714	0,789	0	1,578
5,63	1,13	0,209	0,856	1	27,8571	4,5	41,9804	0,143
5,632	1,416	3,053	0,551	0,429	222,857	1	36,9992	0,789
7,073	1,996	6,781	0,428	0	248,571	4,5	23,7832	1,143
6,509	1,143	3,715	0,856	0	248,571	0	6,9972	1,789
3,409	2,15	8,987	0,428	6	197,143	1	16,506	4,5
4,482	2,787	5,864	0,428	1,143	445,714	1	42,1232	4,643
2,766	1,064	5,216	0,123	4,5	300	1	13,5324	4,643
6,518	1,218	5,932	0,856	0	428,571	4,5	26,3676	1,143
5,711	4,154	12,789	0,786	4,5	300	4,5	0	6,789
4,061	1,57	0,066	0,123	1	257,143	1	0	0,143
5,205	3,286	6,852	0,428	4,5	120	1	0,2268	1,143
4,129	0,759	0,495	0,856	1	154,286	1	11,2476	0
3,922	0,77	0,921	0,428	0,209	137,143	1	11,1636	0
4,139	1,218	1,789	0,428	0	102,857	0,066	20,6052	0,855

5,493	1,853	5,053	0,428	0	154,286	0	2,7972	0,932
5,43	2,862	5,082	1	0	102,857	0	7,7952	5,289
7,17	1,209	0,341	0,428	0,143	240	1	12,264	0
2,348	1,207	1,284	0,428	4,5	265,714	4,5	16,2582	0,932
4,431	2,51	1,352	0,428	0	394,286	0,143	20,2356	5,289
6,291	1,864	2,264	0,066	1,143	265,714	1	18,0012	0,932
4,065	1,853	1,578	0,428	4,5	437,143	1	78,0052	1,578
1,54	2,073	8,341	0,066	0	480	4,5	0	0,066
5,634	6,355	2,93	1	4,5	248,571	1	13,6584	0,572
1,702	0,561	4,998	0,066	0	360	1	0	5,289
5,795	0,286	0,855	0,123	0	565,714	4,5	6,9384	0,143
5,283	2,073	6,51	0,786	4,566	600	4,5	29,9964	1,143
5,568	1,638	1,275	0,123	0	394,286	1	16,926	0,143
7,563	2,787	1,787	0,428	1,066	480	4,5	30,002	0
4,435	1,141	8,352	0,786	1	214,286	1	30,002	1,143
4,781	1,93	4,13	0,123	1	480	1	11,3988	4,643
4,478	1,776	6,775	0,428	1	385,714	1	25,4744	0,932
10,176	0,715	0,352	0,428	0	180	0,066	1,6128	0,143
3,557	0,264	2,352	0,428	1	428,571	4,5	34,5296	0,132
4,708	0,987	1,853	0,123	4,643	334,286	4,5	13,1292	4,643
2,832	0,627	10,367	0,066	1	531,429	4,5	0	4,566
4,795	3,288	5,71	0,551	4,5	214,286	4,5	0,8064	4,643
6,001	2,216	7,493	0,428	0,789	120	1	0	4,643
8,374	1,921	2,572	0,123	0,789	360	0,066	6,7452	0,789
7,72	1,853	1,504	0,428	0	222,857	4,5	13,0704	0,143
7,649	1,71	10,429	2	0	240	1	13,2762	1,143
10,872	1,776	4,429	0	0,789	617,143	1	6,1824	1,789
5,715	1,776	0,638	0,428	1	308,571	4,5	24,8976	1,143
6,425	1,842	0,418	0,428	0	120	1	12,264	6,143
4,269	4,643	1,352	0,066	1	222,857	0	8,3412	4,643
2,315	2,642	1,921	0,123	1	308,571	0	300,02	6,143
2,865	1,218	1,864	0,786	0,789	248,571	0,429	0	0,789
11,435	2,864	5,361	0,786	4,5	360	0,429	5,376	1,789
4,861	1,429	6,209	0,123	0,789	214,286	1	2,1504	1,066
6,928	4,918	2,932	0,786	0	197,143	0,143	2,1504	1,143
4,126	0,921	1,855	0,123	0	180	4,5	16,506	4,643
3,775	1,702	2,067	0,066	4,5	351,429	1	0	0,143
7,438	0,275	0,932	0,428	4,5	360	1	84	0,789
6,628	1,721	11,007	0,428	5,289	70	4,5	101,4272	4,643
4,586	1,064	3,075	0,066	1	154,286	1	51,282	0,143
10,503	2,141	4,787	0,066	0	428,571	1	4,662	1,143
8,071	2,141	0,341	1,572	4,5	325,714	1	27,426	0,932
5,364	1,998	6,275	0,428	0,789	257,143	1	10,2144	4,643
3,415	1,064	0,638	0,428	0,429	180	6	16,3212	1,143
7,991	2,796	2,789	0,123	0,143	94,2857	6	0	0,789
9,709	4,852	0,921	0,786	0	445,714	1	2,7972	5,289
3,915	1,064	0,998	0,428	0	102,857	0	18,648	4,643
4,861	1,998	0,638	1,214	0	240	1	33,3872	0,066

2,972	1,787	2,921	0,123	0	574,286	1	9,1896	4,643
5,726	2,708	1,864	0,428	1	197,143	4,5	7,4592	1,789
9,866	1,921	1,932	2	4,5	385,714	1	4,5276	6,789
4,832	2,499	6,789	0,428	0	240	1	0	0,789
6,7	2,064	1,418	1,572	0	291,429	1	67,6032	4,643
4,7	2,35	3,352	2,5	0,789	222,857	1	32,8608	4,643
6,293	1,932	7,218	0,428	1,789	394,286	6	17,7912	1,578
5,203	0,264	7,209	0,428	1	325,714	4,5	0,8064	1,143
8,167	1,998	0,429	0,428	1	248,571	1	0	0,143
5,983	1,996	3,064	1,214	4,5	205,714	4,5	3,7632	1,143
1,548	1,341	10,198	0,786	1	600	1	6,5016	4,566
10,085	1,578	3,209	0,123	1	437,143	1	6,3672	4,566
9,938	0,407	0,495	0,428	0	282,857	4,5	53,9952	0,143
5,496	1,919	0,998	0,428	0	257,143	1	3,6792	4,643
12,45	2,073	7,721	0,786	0	137,143	4,5	28,4662	1,789
3,832	0,264	0,209	0,428	0,143	514,286	4,5	77,9296	0,789
4,548	2,216	4,572	0,066	0	300	4,5	6,72	4,643
3,995	2,576	4,644	0,123	1	360	4,5	27,986	6,143
8,725	2,007	2,286	0,428	0,143	72,8571	4,5	0	1,218
3,401	0,429	5,721	0,123	1	514,286	1	9,3156	4,643
5,935	1,57	9,284	0,428	0,143	300	4,5	33,1072	1,789
7,59	2,064	6,284	0,428	1	197,143	1	19,7232	0,143
4,434	2,576	5,064	0,428	0,789	240	1	0	0,789
5,489	1,996	0,341	0,428	0	257,143	1	22,6632	0,143
5,784	1,864	5,218	0,428	0	154,286	4,5	7,9254	1,143
7,284	2,708	3,218	0,856	4,5	351,429	1	26,7876	4,566
5,225	2,218	8,998	0,123	1,143	171,429	4,5	10,2564	1,143
5,207	1,075	2,209	0,123	1	282,857	1	5,4852	0,143
10,083	6,507	6,432	0,428	0	548,571	4,5	13,5912	0,932
4,923	2,576	4,51	0,428	0,066	154,286	4,5	20,9076	5,289
4,707	1,853	1,787	0,856	0	514,286	1	14,1036	4,643
5,86	0,987	4,932	0,856	1	274,286	1	1,8396	0,789
6,352	5,707	2,352	0,428	4,5	394,286	4,5	11,6592	4,643
5,491	2,776	2,075	0,428	0	154,286	0	110,5776	0,789
4,717	1,075	0,998	0,428	0	257,143	1	11,13	1,066
5,365	0,921	1,495	0,123	0	135,714	1	0	0,429
9,44	2,644	1,352	1	0	248,571	1	57,6352	0,143
3,272	1,35	5,418	0,786	0	282,857	4,5	18,1272	0,143
6,524	2,007	0,209	0,123	0,143	97,1429	0,789	8,6604	0,143
5,712	1,864	5,284	0,428	0,495	282,857	4,5	12,39	1,143
2,491	0,998	5,987	0,066	0	462,857	0	2,688	4,643
5,917	1,985	1,13	0,428	0	394,286	1	43,5708	4,643
5,368	0,132	9,932	0,428	0	120	1	0,2268	1
3,69	0,495	2,139	0,617	0,429	308,571	4,5	6,3672	0,855
5,789	2,207	2,075	0,786	0,143	240	1	16,6152	5,289
4,788	1,781	0,638	0,428	1	257,143	4,5	19,4782	1,143
2,032	2,199	1,361	0,123	1	274,286	4,5	18,648	4,643
7,706	2,062	6,196	1	1	72,8571	4,5	5,5944	0,286

4,939	0,781	2,789	0,428	0,789	225,714	1	0	0
6,586	0,987	1,71	0,428	1	514,286	1	0,8064	0,143
6,508	2,15	1,578	0,428	1	154,286	0,066	24,2424	1,789
9,066	0,352	7,221	0,428	0,132	102,857	1	17,038	1,143
3,348	0,418	1,932	0,123	0	317,143	0	3,4944	4,643
4,498	2,15	9,355	1,214	0,066	180	1	19,74	4,643
4,706	2,216	5,418	0,428	4,5	240	1	9,324	4,643
5,555	1,853	6,987	0,428	0	325,714	4,5	138,0092	0,143
5,069	1,493	0,132	0,428	0	265,714	0	21,0672	0,066
4,915	2,642	0,855	0,786	4,566	77,1429	4,5	13,1292	1,218
6,007	1,93	7,218	0,428	1	180	4,5	15,0612	1,143
6,726	0,429	1,284	0,123	0,143	214,286	0	12,3942	1,143
7,239	3,431	5,222	0,428	0	428,571	1	86,2708	4,5
2,546	1,855	0,209	0,189	0	180	4,5	0	0
7,66	2,139	1,578	0,428	1	90	1	27,972	1,143
4,35	3,293	10,293	1	4,5	360	4,5	0,8064	0,789
8,66	0,572	1,987	1	1	480	1	141,9796	1,578
1,757	6,144	4,789	1	0	651,429	4,5	55,9524	6,143
4,704	2,499	3,576	0,428	4,5	79,2857	1	17,0352	4,643
4,635	0,132	4,066	0,123	0	420	0	1,344	0,143
6,566	1,075	0,132	1	0	240	0,789	18,0012	0,789
3,574	5,641	2,066	0,123	1	317,143	4,5	6,2748	4,643
9,562	1,855	2,427	0,428	0	240	4,5	2,688	0,855
10,996	2,565	3,495	1	1	317,143	1	551,614	4,643
5,138	2,141	1,998	1,572	0	411,429	0,429	1,8396	0,143
7,664	2,433	0	0,428	0	300	0,789	46,62	0,789
2,054	2,367	1,71	0,428	1	360	4,5	3,6624	6,143
3,275	2,642	0,275	0,428	0	300	1	8,5596	0,789
4,722	2,653	6,987	0,786	0	111,429	1	13,0704	1,789
11,929	1,416	2,998	0,428	1	120	4,5	5,8632	0,066
2,623	1,284	2,495	0,428	0,789	377,143	1	11,13	1,578
6,925	1,578	2,207	0,856	4,5	120	4,5	0	0,143
8,409	1,35	4,704	0,123	4,5	514,286	1	4,662	5,289
3,973	2,293	6,559	0,066	0	257,143	4,5	3,8724	5,289
3,546	6,144	4,361	0,786	0,066	205,714	4,5	2,1504	0,143
7,071	0,209	1,064	0,428	0	300	1	48,0032	0,143
5,155	2,785	10,293	0,428	0	137,143	4,5	11,8776	5,289
6,572	3,508	5,864	0,428	1	274,286	1	18,0012	0,789
1,46	2,073	1,064	0,123	4,5	334,286	0	15,0612	6,789
3,187	1,341	5,418	1	0	214,286	1	19,74	4,643
1,482	2,578	1,855	0,123	0,066	471,429	1	12,5916	1,066
5,423	2,053	4,71	0,428	0	334,286	4,5	5,0232	0,789
3,86	1,578	0	0,428	0	274,286	0	1,8396	0
4,13	1,427	7,067	1,066	0	394,286	1	19,7232	0,066
9,415	0,561	5,921	0,428	0,855	180	1	5,5944	0
6,507	1,787	9,864	0,856	4,5	17,1429	4,5	18,648	5,289
6,352	2,293	4,143	0,428	1	265,714	1	0	0,143
1,812	0,715	4,064	0,066	0	574,286	1	75,7792	0,143

4,841	1,207	12,932	0,56	4,5	300	1	5,5944	5,289
7,156	1,998	3,219	0,428	0,429	300	4,5	0	1,789
5,353	1,196	1,71	0,123	4,5	325,714	4,5	25,9084	0,066
8,801	1,284	1,921	0,428	1	162,857	1	2,4192	1,143
2,603	5,487	6,578	0,428	0,429	582,857	4,5	3,1836	4,643
4,717	1,776	1,064	0,428	0,789	300	4,5	42,3668	0,789
4,143	1,93	1,35	0,428	0	411,429	0	8,064	0,143
9,848	1,33	6,484	0,856	0	120	4,5	10,6092	0,789
5,803	1,578	2,284	0,786	1	334,286	1	189,9576	1,789
8,78	1,853	0,429	0,123	0	197,143	4,5	12,264	0,143
9,5	0,789	1,644	0,123	1	171,429	4,5	1,8396	0,143
3,291	4,852	4,198	0,428	0	445,714	1	0	0
3,775	1,721	6,484	0,428	1	137,143	1	0	5,289
3,137	5,498	5,143	0,123	4,5	300	4,5	3,6792	0,143
6,284	2,71	8,075	1	1	480	4,5	733,9304	1,143
5,157	0,572	0,921	0,123	1,143	120	1	7,4592	1,578
8,094	2,51	3,367	0,123	0	360	1	0	4,929
4,706	0,352	0,275	0,428	1	197,143	0	45,0632	0,143
12,773	0,341	0,198	0,551	0,143	282,857	4,5	25,6564	0,066
5,359	2,216	3,361	0,428	0,066	154,286	0	0	1,143
4,201	2,785	1,787	1	4,5	600	1	122,2144	4,643
11,051	2,499	12,71	0,123	0,132	47,8571	4,5	36,5022	4,643
9,773	1,141	1,429	0,066	0	240	1	6,9972	0,143
5,071	2,207	13,064	0,428	4,5	77,1429	4,5	44,6544	6,066
7,807	1,787	1,075	0,428	1	274,286	1	23,31	4,643
9,503	2,425	1,721	0,428	0,855	411,429	4,5	20,9832	0,789
3,299	0,286	5,218	0,428	0	300	4,5	8,064	1,143
3,841	2,273	3,853	0,786	0	582,857	4,5	2,7972	4,643
4,86	1,064	1	0,428	0	300	1	360,024	4,566
3,06	0,561	4,209	0,428	0	240	4,5	54,53	0,143
5,212	1,787	4,352	0,428	1	214,286	1	10,5756	0,932
4,614	1,864	0,407	0,066	0	231,429	4,5	16,1896	0,143
5,779	2,93	4,495	1	0	325,714	0,066	18,1272	6,789
6,728	1,847	0,066	0,428	0	394,286	4,5	0	1,578
4,223	0,286	0	0,066	0	445,714	1	29,6632	0
4,916	0,55	1,141	0,066	0	420	1	0	0,066
4,869	3,442	4,945	0,428	1	428,571	4,5	5,1282	1,789
12,881	1,787	0,998	0,066	0	180	4,5	2,9568	0,143
2,046	2,576	5,921	0,123	0,789	480	4,5	6,72	1,143
3,338	1,493	3,638	0,428	0	197,143	1	5,376	4,643
12,903	3,222	1,721	0,856	0	300	4,5	20,4582	5,289
3,937	4,918	5,361	0,786	0	291,429	4,5	126,5096	4,643
3,28	2,433	9,299	0,856	0,789	522,857	4,5	19,2024	1,578
13,173	1,71	3,367	0,428	0	402,857	1	22,8508	0,143
2,436	1,075	2,143	0,123	0	308,571	1	0	0
6,142	1,352	5,721	1,428	0,789	205,714	4,5	18,6592	0,143
8,692	3,299	3,796	0,428	1	137,143	0,789	27,972	1,578
12,14	3,365	6,921	1	1,143	1028,57	0	22,6632	6,789

11,736	1,908	2,642	0,428	0	180	4,5	42,8904	1,218
4,48	0,407	1,998	0,123	0	282,857	6	4,8384	0,143
4,346	1,352	2,209	0,123	0	68,5714	1	3,4944	0
2,263	2,796	8,987	0,123	1	445,714	4,5	0	4,643
3,911	1,418	14,275	0,428	4,5	257,143	0	6,9384	4,566
10,004	0,627	2,433	2	1	102,857	4,5	78,0052	0,066
7,645	2,13	2,787	0,786	0	60	4,5	5,0232	1,578
5,711	3,299	6,141	0,428	0	240	1	0	0,789
11,782	1,407	2,484	1,214	4,5	222,857	1	25,6564	0,143
3,493	1,341	2,932	0,428	1	102,857	4,5	2,688	0,066
4,922	0,407	3,352	0,246	1,789	291,429	4,5	0	4,643
3,926	0,627	7,721	0,428	0,789	420	1	4,221	1,066
4,058	1,339	3,704	0,551	1	240	1	26,1212	4,643
8,786	1,578	2,578	0,123	0,789	445,714	1	30,1224	0,143
4,212	1,93	0,932	0,856	0	600	4,5	69,972	0,143
3,97	2,073	1,787	0,428	0	420	1	9,9288	1,789
5,352	1,13	3,218	0,428	0	171,429	1	9,0552	0,143
5,157	0,921	0,143	0,428	0,789	120	4,5	36,134	1,218
6,216	0,998	5,5	0,428	4,5	300	0	0	0,572
4,709	1,985	8,218	0,428	0	420	4,5	1,533	4,643
7,402	1,578	0,066	0,123	4,5	480	1	9,2484	0,143
8,007	1,921	2,921	0,428	4,5	274,286	4,5	17,7912	0,143
19,299	2,708	1,33	0,428	1	162,857	4,5	5,4684	0,789
10,143	3,367	1,286	0,428	0	308,571	4,5	14,6664	0
4,855	1,427	2,293	0,428	4,5	197,143	0,789	1,8396	4,643
4,005	1,13	4,647	0,428	0	154,286	4,5	0	1,789
2,337	1,207	6,286	0,428	0,789	300	4,5	5,376	4,643
2,774	1,064	4,484	0,856	0	240	4,5	1,344	4,643
3,341	1,075	2,644	0,428	0,429	445,714	4,5	20,6766	2
5,704	2,644	0,932	0,786	4,566	437,143	0	11,9364	4,643
2,056	0,066	0,921	0,066	0	162,857	4,5	24,528	0
6,798	0,77	2,484	0,428	1	454,286	1	16,3212	4,643
5,786	1,493	2,644	0,852	0,429	325,714	0,143	64,666	4,643
4,847	3,365	0,715	0,979	4,566	462,857	0	34,9272	0,789
5,339	1,776	1,853	0,786	0,429	257,143	1	5,5944	1,143
7,429	2,282	3,932	0,428	0,066	137,143	1	6,5016	0,143
3,497	2,576	6,787	0,123	4,5	420	0	8,8704	0,143
5,012	1,864	2,075	0,428	4,5	180	4,5	51,31	0,789
5,924	1,141	4,638	0,856	0	257,143	1	0	4,643
3,489	0,858	1,71	0,123	0,789	222,857	1	7,5264	1,789
5,19	1,35	4,932	0,786	0,429	222,857	1	13,9272	4,643
5,289	0,855	0,275	0,123	0	102,857	4,5	10,2816	0,066
5,001	1,273	4,284	0,786	0,855	162,857	1	14,8932	1,143
8,732	1,787	5,653	0,428	1	180	1	0	1,143
5,139	3,354	3,13	0,428	4,5	77,1429	1	14,9184	1,143
4,715	1,985	7,141	0,066	0	351,429	1	8,064	6,789
6,238	2,51	5,776	0,132	1	480	0	78,4896	1,143
12,292	1,119	3,644	1,066	0	197,143	1	4,5276	1,143

3,071	1,218	0,638	0,123	0,429	31,4286	0	0	0
7,212	2,359	3,93	1,284	1	411,429	4,5	25,6452	0,066
3,189	1,996	3,987	0,428	0	257,143	1	85,3104	0,932
4,922	1,561	0,352	0,786	1,066	308,571	1	30,758	0,286
5,931	1,787	1,998	0,909	1	60	1	2,1504	0,143
18,349	5,553	4,064	0,856	0,789	240	1	4,1412	0,286
2,469	1,064	0,429	0,066	1	377,143	0,789	40,8632	4,643
7,943	1,578	3,932	1	0	154,286	4,5	0	2
5,836	6,221	3,495	0,786	0	617,143	4,5	37,3072	0,789
5,651	1,572	8,341	0,786	0	94,2857	4,5	0	6,143
3,937	1,218	1,209	0,428	0	120	0	0	0,932
4,92	0,998	5,5	1,284	0	111,429	4,5	8,3916	0,143
5,572	1,429	6,51	0,428	0	120	1	3,7464	1,143
3,768	1,787	6,221	0,428	0	548,571	0	2,7972	0,286
2,823	1,196	0,484	0,428	0	377,143	1	35,4312	0,855
4,568	2,064	2,218	0,428	0	368,571	4,5	21,0672	0,932
4,794	1,908	10,999	2,5	0	102,857	0,789	0	4,643
4,135	6,43	5,141	0,428	0	342,857	1	0	5,289
3,48	0,561	3,644	0,123	0	360	1	19,3452	0,066
4,838	4,154	3,196	0,856	0,066	317,143	1	129,3796	1,789
6,95	2,653	1,075	1,123	1	308,571	4,5	59,3264	0,143
5,337	0,561	5,996	1	1	480	4,5	83,9664	1
3,206	1,361	1,064	0,856	4,5	480	4,5	103,6812	0,066
12,209	0,913	0,132	0,428	0	240	1	1,6128	0,855
3,359	1,066	0,132	0,428	1	102,857	0	6,132	0,143
9,943	1,71	1,218	0,428	0	154,286	4,5	13,0704	4,643
3,717	1,721	2,638	0,428	4,5	240	1	72,7692	1,578
1,9	1,053	8,15	0,786	4,5	214,286	1	10,0632	0,286
4,003	1,559	2,418	0	0	94,2857	1	7,3164	1,789
6,572	2,271	1,759	0,428	1	480	1	6,5604	1,578
6,065	2,064	6,561	0,123	0,066	428,571	4,5	49,7644	0,495
5,938	0,198	3,064	0,856	0	180	1	4,662	0,932
1,955	1,842	5,273	0,123	0	274,286	1	10,752	5,289
12,294	1,853	10,93	0,428	4,5	205,714	4,5	51,296	0,932
4,715	1,493	0,209	0,428	1	282,857	4,5	42,5936	4,5
5,154	2,776	5,075	0,856	1	377,143	1	9,5046	4,566
7,864	0,858	4,418	0,428	0	90	4,5	30,31	0,932
2,689	0,352	10,264	0,132	0	480	4,5	2,7972	0,143
6,789	2,785	1,075	0,856	0	240	1	18,396	4,643
9,857	3,433	10,433	0,786	0	154,286	0	73,6512	0,143
3,918	2,93	4,352	0,428	0	197,143	4,5	51,282	5,289
7,005	5,487	1,13	0,428	0	214,286	4,5	31,5252	0,789
7,794	2,13	0,209	1	0	360	1	37,296	1,789
7,431	1,141	4,576	0,428	4,5	300	1	26,4096	4,643
7,007	1,198	0,286	0,428	1	291,429	1	18,0012	0,789
4,28	0,932	2,275	0,428	0	180	0,789	0	0,932
1,977	0,704	18,143	0,786	1	231,429	4,5	47,4264	1,143
4,129	1,273	3,141	0,428	0	342,857	1	30,5172	4,643

4,783	1,998	0,264	0,066	0	394,286	4,5	32,9196	0,066
6,232	3,007	13,721	0,428	4,5	137,143	4,5	12,264	5,289
4,786	1,196	0,638	0,856	0,789	77,1429	4,5	35,0392	0,932
6,516	1,493	1,781	0,428	1,429	205,714	1	13,44	4,643
3,552	1,864	0,341	1,214	0,429	454,286	0,789	32,7992	0,855
6,276	2,13	6,559	0,551	1	102,857	0,789	69,972	5,289
6,287	2,139	1,853	0,786	1	300	4,5	10,752	0,209
6,948	3,222	2,075	0,428	0	240	4,5	18,396	5,289
6,795	2,644	5,647	0,428	0	317,143	0	0	5,289
3,691	3,499	4,075	0,428	1	154,286	0	2,1504	1,789
5,207	1,787	10,855	0,066	4,5	291,429	1	0	0,789
3,335	2,282	6,499	0,132	4,5	480	1	12,2304	5,289
7,796	2,216	5,576	0,428	0,066	471,429	0	0,8064	4,643
4,289	1,141	5,932	0,551	0	411,429	1	52,7422	1,578
3,896	2,007	3,921	0,066	6	514,286	1	0,8064	0,143
5,124	5,498	2,073	0,428	0	265,714	4,5	31,5252	0,143
9,56	2,631	6,418	0,428	0,429	120	0	6,72	0,789
4,992	2,053	1,141	0,856	0	462,857	0,066	36,0024	0,143
3,348	1,13	3,132	0,428	0	180	1	0,8064	0,209
4,423	2,928	1,789	0,786	1,143	368,571	4,5	44,0412	0,143
7,774	0,987	1,132	0,189	0,789	325,714	1	2,688	0,143
4,201	1,207	1,987	0,066	0	257,143	1	13,818	6,066
4,86	2,282	5,075	0,428	0	308,571	4,5	36,0024	0,932
7,617	1,35	4,572	2,5	4,5	240	1	19,74	0,572
1,493	0,998	2,789	0,551	0	68,5714	0	0	0
6,293	3,205	6,433	0,428	0	368,571	4,5	48,7284	6
5,632	1,561	7,284	0,786	4,5	265,714	4,5	5,6448	0,932
8,851	1,207	6,855	0,786	0,789	137,143	1	1,344	0,143
10,881	3,286	4,864	0,428	0,789	154,286	4,5	31,8402	1,578
3,843	2,141	1,209	0	0	120	4,5	0	5,289
6,87	2,568	3,721	0,428	0	317,143	4,5	8,5848	1,789
4,348	1,064	5,51	0,428	1	317,143	0	21,4452	1,143
5,57	0,987	1,919	1,786	0	240	1	43,3132	5,289
6,35	0,209	1,578	0,428	0,066	385,714	1	5,5944	0,143
1,669	1,721	0,209	0,856	0,789	60	4,5	30,1224	0,143
5,787	1,842	1,286	0,428	0,789	85,7143	4,5	81,634	4,566
7,282	1,284	0,495	0,123	0	591,429	0	6,132	6,143
6,561	1,913	7,853	0,428	0,066	120	1	0	1,789
3,288	2,433	2,064	0,428	0	300	4,5	13,5492	0,789
4,047	2,282	3,064	0,786	1	111,429	0,789	27,9804	1,143
2,997	2,568	12,921	0,123	0	274,286	4,5	21,1932	0,143
3,776	1,561	2,853	0,123	0	77,1429	1	0	0,066
7,583	2,642	1,284	0,674	0	77,1429	4,5	6,9972	0,143
3,478	0,143	11,119	0,428	0	120	6	3,36	4,566
3,557	2,51	1,644	0,428	0,143	222,857	1	19,0344	1,578
7,81	2,007	1,504	0,428	0	300	4,5	10,9368	1,143
4,834	6,144	6,341	0,494	0,066	214,286	1	22,0542	5,289
4,766	2,853	10,35	0,786	1	154,286	1	21,168	4,566

4,259	1,207	11,064	0,786	0,855	282,857	6	18,2406	4,643
5,276	1,93	11,996	0,123	4,5	600	4,5	11,662	5,289
6,583	0,275	3,578	0,428	1	171,429	1	6,7452	0,789
7,925	1,787	2,77	0,246	0,143	205,714	4,5	23,324	1,578
10,347	5,432	9,856	2,928	0,789	214,286	1	1,344	5,289
5,638	1,787	6,781	0,428	1	162,857	0	2,7972	0,286
4,847	0,998	1,209	0,551	0	480	4,5	6,9384	4,643
3,192	2,141	8,504	0,428	0	265,714	4,5	13,0872	4,643
4,871	2,51	4,433	0,428	0	94,2857	1	4,032	1,143
3,907	6,276	3,429	0,428	1	240	1	53,1272	0,789
5,87	0,858	7,221	0,428	4,5	231,429	4,5	0	4,643
11,159	3,288	1,644	0,428	1,218	240	0,789	30,66	4,643
5,764	2,207	6,719	1,786	0,143	334,286	4,5	34,4736	0,286
14,509	2,862	6,932	0,428	4,5	351,429	1	36,9992	5,289
4,278	1,636	11,919	0,786	0	214,286	4,5	18,396	5,289
10,432	3,502	3,429	0,428	0	394,286	4,5	13,9944	0,209
5,842	2,708	1,787	0,428	0	214,286	1	26,7372	0,143
4,071	3,789	10,075	1	0,066	317,143	4,5	16,6488	0,789
4,006	1,864	6,572	0,428	0	240	4,5	5,5944	1,578
4,273	3,998	5,361	1	4,5	120	4,5	0	4,643
12,439	0,998	2,064	0,428	1	102,857	4,5	39,0684	1,143
3,932	1,13	2,429	0,428	0	514,286	1	48,0032	0,143
8,882	1,853	4,284	0,428	1	265,714	0,429	14,4592	0,572
5,498	0,275	0,341	0,428	0,789	120	1	10,8612	0,855
8,594	3,071	1,218	1	1,143	762,857	1	12,7428	1,143
2,197	1	3,796	0,123	4,5	600	1	19,4544	4,643
7,009	5,564	1,13	0,428	0	411,429	4,5	37,3072	1,143
6,785	1,855	15,073	0,428	0	214,286	4,5	4,032	5,289
6,795	2,359	16,932	0,909	4,5	471,429	0	5,5944	6,143
4,838	1,361	5,495	1	0	300	4,5	0	0,789
4,566	1,853	4,721	0,123	0	300	1	2,688	0,855
6,867	1,141	3,361	0,123	0,789	257,143	1	20,7172	1,143
5,577	1,504	9,218	0,428	0	368,571	4,5	8,9292	0
8,664	2,073	3,284	0,428	0,789	102,857	1	0	1,143
6,507	1,418	2,196	1,214	0,789	265,714	1	12,9948	0,143
3,626	1,493	1,987	1	1	385,714	1	21,714	5,289
10,36	2,073	0,286	0,786	0,066	120	4,5	2,9064	0,789
6,715	1,273	3,638	0,428	0	154,286	0	18,6592	0,286
5,346	1,57	2,13	0,551	4,5	240	4,5	0	1,143
5,926	0,484	0,932	0,428	1	282,857	1	30,9232	0,066
8,801	2,576	2,932	0,428	0	120	4,5	59,6736	5,289
6,998	2,776	0,855	0,856	0	377,143	1	60,2308	1,066
5,491	1,77	0,932	1	0	300	4,5	0	0,932
4,843	1,207	6,352	0,123	1	334,286	4,5	15,33	5,289
6,067	1,636	3,053	0,428	0,789	257,143	4,5	15,8592	0,286
3,708	1,198	9,864	0,428	0	402,857	1	2,7972	0,143
5,214	1,064	2,576	0,428	0,143	471,429	4,5	0,8064	0,572
5,781	1,71	4,207	0,551	0,066	197,143	1	14,4592	1,143

2,485	1,053	6,141	0,428	0,286	274,286	4,5	8,064	0,932
9,803	1,493	1,066	0,856	1	180	1	30,8644	1,789
2,922	2,708	4,572	0,428	0	411,429	4,5	18,396	1,143
1,779	0,998	5	0,617	0	437,143	0	0,8064	0,932
4,649	2,576	6,064	0,922	4,5	162,857	1	5,376	4,643
4,931	0,998	2,341	0,123	1	154,286	4,5	9,2484	0,932
4,333	0,77	1,776	0,066	0	360	4,5	18,7656	0,066
1,996	0,561	0,418	0,428	0	120	1	45,6372	0,932
4,489	2,71	5,641	2,5	0,066	317,143	1	38,066	4,643
3,478	0,473	8,719	0	4,5	360	1	0,8064	0,143
6,15	5,63	3,352	0,786	1	608,571	1	23,058	1,143
6,726	1,853	2,275	3,5	0,932	102,857	4,5	36,0024	0,143
6,073	1,132	0,209	0,428	4,5	222,857	1	1,6128	0,143
5,271	2,787	8,781	0,428	1	600	1	15,0612	4,643
9,664	2,653	3,075	0,786	0	113,571	1	18,102	1,143
4,272	5,553	0,855	0,428	0,429	600	4,5	33,348	5,289
6,952	2,51	1,495	0,428	0,143	60	4,5	10,038	5,289
3,555	2,862	0,429	0,066	0	360	1	60,004	5,289
7,304	1,93	5,218	0,856	0	222,857	0,143	7,2282	1,578
5,004	1,053	5,141	1,066	0,429	377,143	1	5,4852	4,566
6,495	1,427	0,495	0,856	1	274,286	0,789	0,8064	1,143
7,114	1,919	2,855	0,428	1,143	94,2857	4,5	7,9716	1,789
11,351	2,207	1,998	1	4,5	497,143	1	10,038	4,643
10,005	2,578	6,864	0,428	4,5	240	4,5	46,3232	1,143
8,785	1,721	0,066	1	0	265,714	1	55,9482	0,143
5,72	1,787	3,13	0,786	0	162,857	4,5	21,1932	4,643
4,919	2,653	5,576	0,123	1,578	162,857	1	2,688	5,289
2,207	0,921	0,066	0,123	4,5	180	0	1,8396	4,643
4,849	2,719	5,644	0,428	4,5	300	0	5,376	4,643
9,839	1,418	6,352	0,428	4,5	180	4,5	13,9272	1,143
5,28	3,141	3,858	0,856	0	540	4,5	11,382	5,289
4,718	1,493	5,653	0,428	1	72,8571	0,789	1,512	1,789
4,907	1,416	1,075	1	0	394,286	1	0	0,143
2,186	2,642	5,484	0,066	0	282,857	4,5	1,512	6,143
6,229	1,35	6,721	0,123	0	317,143	4,5	0,8064	5,289
8,449	1,647	2,429	0,428	0	197,143	0	4,5276	0,143
7,374	0,198	5,998	0,428	1	522,857	4,5	42,3164	0
6,839	2,207	1,493	0,189	0	394,286	0,789	18,396	0,143
6,706	2,644	5,932	0,428	4,5	214,286	4,5	23,1924	4,643
3,419	1,787	1,998	0,428	1	77,1429	1	14,2464	4,643
2,186	2,578	5,001	1	0	94,2857	1	13,5492	0,143
7,615	1,341	8,275	0,786	0,789	265,714	1	15,414	4,643
2,342	0,352	0,921	0,132	0	351,429	1	2,688	0
7,297	2,502	8,721	0,428	1	531,429	0	0	4,643
5,284	2,93	9,075	0,786	6	497,143	1	59,3264	4,643
5,718	2,647	8,566	0,786	0	300	1	0	0,932
6,502	3,216	10,218	0,428	0,789	274,286	4,5	10,1472	0
5,431	1,864	5,141	0,428	0,789	77,1429	4,5	2,331	4,643

6,218	6,564	0,286	0,856	4,5	120	0,066	0	5,289		
9,86	1,787	0	2,5	0	12,8571	1	93,324	0,789		
4,641	1,864	0,998	0,786	0	214,286	4,5	24,2424	0,932		
6,128	1,207	3,93	2	1	77,1429	4,5	2,7972	0,932		
4,856	1,133	4,776	0	5,289	214,286	4,5	12,1212	5,289		
6,207	1,196	2,073	0,551	1,066	137,143	4,5	10,2816	0,143		
9,075	1,33	0,921	0,428	0	94,2857	4,5	33,5202	1,143		
8,701	1,273	5,77	0,428	1	394,286	4,5	84,4312	1,066		
5,926	1,141	3,644	0,066	1,218	137,143	0	0	0,789		
14,225	2,13	5,361	0,856	4,5	214,286	1	4,1412	0,855		
2,183	2,996	1,998	0,428	1,143	411,429	0,789	47,586	0,932		
4,99	5,498	7,275	0,909	4,5	565,714	1	20,6052	0,789		
9,505	0,704	1,721	0,123	0	180	0,429	0	0,143		
4,985	0,407	1,264	0,551	0	445,714	1	5,8632	0,143		
10,086	2,708	4,653	0,428	0	77,1429	1	24,528	5,289		
8,728	1,35	3,721	0,428	1	265,714	0	14,7	1,578		
4,117	1,853	0,921	0,428	0	514,286	1	6,1824	0,932		
5,289	1,627	1,561	1	1	137,143	0,789	18,0012	1,143		
7,087	1,001	4,932	0,428	1	377,143	4,5	0	0,932		
3,845	3,073	6,13	0,856	0,066	642,857	1	20,7984	6,143		
4,434	1,864	4,286	0,428	0	274,286	4,5	121,6208	1,143		
9,372	1,504	5,932	0,428	1	394,286	1	0	0,495		
4,572	2,293	0,066	0,428	1	317,143	1	83,6472	0,066		
2,612	1,493	9,075	0,428	4,5	437,143	4,5	16,1364	4,643		
6,361	2,436	1,361	0,428	4,5	514,286	1	30,9232	4,643		
11,663	2,864	1,352	0,428	0	120	0,066	7,5264	1,789		
5,14	1,864	0,352	0,428	0,429	240	4,5	41,958	0,286		
7,078	0	1,198	0,786	0,789	60	1	38,7506	1,789		
6,144	2,216	4,572	0,428	1	240	1	42,3752	1,143		
9,952	1,416	1,286	0,428	4,5	385,714	4,5	2,7972	0,286		
5,572	1,075	1,209	1,189	0,789	222,857	4,5	6,9972	0,789		
4,494	1,853	2,427	0,428	0	351,429	4,5	17,493	1,143		
4,781	1,776	9,656	0,786	0	420	0,066	0	1,218		
6,14	3,365	8,209	1,428	0,066	120	0	4,3008	0,143		
4,937	2,51	1,218	0,428	4,5	137,143	4,5	18,9168	4,643		
4,082	0,932	0,418	0,428	0,143	497,143	1	27,972	0,143		
3,854	2,15	1,644	0,428	4,5	214,286	0	0	0,789		
7,28	1,787	3,007	0,856	0	162,857	1	4,4352	0,286		
3,361	1,207	3,352	1	0,789	497,143	4,5	96,0064	1,789		
8,089	1,721	3,433	0,428	0	582,857	0,143	29,19	1,143		
4,157	0,789	0,286	0,428	0	214,286	4,5	2,7972	0		
5,867	2,073	5,713	0,856	1	180	4,5	48,398	0,143		
5,559	0,066	0,921	0,856	0	308,571	1	12,1212	0,143		
1,273	1,853	5,209	0,066	0	180	1	0	4,643		
3,536	0,352	0,998	1	0	445,714	1	9,6768	0,066		
4,192	1,275	2,064	0,428	1	231,429	4,5	8,82	0,209		
4,203	1,064	1,429	0,123	0	162,857	1	7,2156	4,643		
2,285	0,855	1,789	0,428	0	141,429	1	0	4,5		

5,772	1,416	0,429	0,856	0	308,571	0	5,8716	4,643
11,215	2,064	1,429	0,428	1	325,714	1	10,8444	0,143
4,34	1,284	5,787	0,786	4,5	317,143	4,5	0	4,566
5,418	3,365	5,579	0,428	0,429	385,714	1	33,4964	5,289
5,06	0,693	9,073	0,428	0	120	1	6,5604	5,289
9,563	1,644	1,578	0,428	1	120	4,5	2,1504	0,789
4,915	6,287	2,066	0,123	0	317,143	1	50,7486	1,143
3,571	2,721	4,367	0,428	0	154,286	4,5	52,78	1,789
5,783	1,853	5,218	0,428	0,932	240	1	7,35	4,643
2,779	0	0,932	0,123	4,5	565,714	1	0,8064	0,066
3,778	0,847	2,075	0,066	0	360	0	2,688	6,143
6,414	2,367	4,484	2	0	205,714	1	2,1504	0,066
8,084	1,284	0,998	1,123	0,066	377,143	1	2,7972	0,066
8,492	1,919	3,864	0,428	0	180	4,5	37,3184	1,066
5,146	0,998	4,495	0,428	0	240	1	69,972	1,789
3,64	0,921	1,132	0,123	0	282,857	1	0,8064	0,143
4,05	1,636	4,222	0,428	1	428,571	1	49,315	1,143
4,344	0,847	5,407	0,066	0	394,286	1	23,9232	5,289
7,506	6,289	0,209	0	0,143	94,2857	4,5	8,064	5,289
7,218	0,921	5,987	0,428	0	334,286	4,5	25,6452	0,789
5,498	1,262	0,484	0,123	0	222,857	0	15,4476	0,066
8,771	0,932	0,998	0,494	0	282,857	4,5	2,688	4,643
7,64	1,141	1,932	0,123	1,066	257,143	1	10,794	4,643
10,661	2,928	3,367	0,428	0	497,143	1	12,264	5,289
3,981	1,284	3,352	0,428	0	85,7143	4,5	17,8584	0,143
3,42	0,341	2,143	0,428	0	102,857	4,5	67,298	0,932
4,282	2,921	3,787	1	0,789	445,714	4,5	10,038	5,289
2,911	1,273	1,352	0,123	0	411,429	1	12,8772	0,066
3,551	1,561	0,275	0,428	0	60	4,5	1,512	1,143
7,513	2,139	7,207	0,428	1	120	4,5	39,9056	0,932
7,016	0,77	4,653	0,856	0	300	0	25,1244	1,789
4,335	1,35	1,141	0,428	0	291,429	4,5	20,6052	0,932
5,293	2,787	4,51	0,428	0,789	154,286	0,789	45,5924	4,643
5,358	2,007	6,561	0,428	0,143	197,143	4,5	13,9944	0,143
3,489	1,996	3,207	0,428	4,5	240	1	37,296	4,643
2,645	1,286	2,352	0	0,429	300	1	11,0292	0,143
10,083	1,218	1,429	0,428	0	120	1	21,4452	1,143
6,07	1,93	8,436	0,428	0,789	454,286	1	18,6592	1,143
6,862	0,921	5,561	0,189	1	325,714	4,5	0	0,066
3,834	0,275	1,132	0,123	1	188,571	4,5	7,9716	0,066
5,067	2,119	12,987	0,786	0,143	180	4,5	0	4,566
4,555	2,93	8,862	0,786	4,566	197,143	1	5,376	5,289
9,206	0,855	1,998	0,494	0,789	180	4,5	10,3992	0,066
5,85	2,853	6,207	0,786	4,5	445,714	4,5	36,1872	1,578
2,625	1,132	9,066	0,123	0	180	4,5	18,396	0,789
13,717	4,852	0,638	0,856	1	437,143	1	2,7972	0,143
6,649	3,776	9,13	0,428	0	222,857	1	18,2112	1,789
8,093	1,864	5,998	0,428	0	154,286	0	13,986	1,143

5,779	2,864	1,93	0,123	4,5	377,143	4,5	7,2324	5,289
5,005	1,209	2,352	0,428	1,066	51,4286	4,5	18,2532	6,143
6,156	1,218	4,367	0,428	0,789	180	1	36,1872	4,643
2,997	1,853	0,998	0,856	0,132	180	0	1,4784	0,143
11,063	2,57	0,275	0,428	0	205,714	4,5	8,8704	1,143
12,059	1,921	2,998	0,123	0,143	137,143	4,5	55,944	1,578
5,707	1,71	1,119	0,066	0	600	4,5	40,8156	1,578
2,928	1,075	4,143	0,551	0,789	394,286	0,789	32,004	5,289
4,201	0,998	6,416	0,066	4,5	274,286	0	0	4,643
11,006	1,053	0,275	0,428	1	325,714	4,5	10,4832	0,789
2,273	1,141	2,855	0,428	1	85,7143	1	2,688	1,143
4,34	2,785	7,132	1	6	222,857	0,143	4,9056	5,289
3,489	1,504	6,576	0,066	0	394,286	0,066	0	4,643
6,148	1,341	1,998	0,132	0,789	368,571	0,429	1,8396	4,643
2,987	0,932	0,209	0,123	0	137,143	4,5	0	0
3,335	2,653	4,638	1	0	94,2857	4,5	43,4966	0,932
5,647	1,504	0,209	0,428	0	342,857	4,5	0	0,143
3,511	0,789	7,721	0,786	4,5	197,143	4,5	4,662	4,643
14,517	0,932	4,484	0,123	1	325,714	4,5	2,7972	0,789
5,416	1,93	1,064	0,428	0	325,714	0	32,6424	4,643
8,798	2,143	2,367	0,246	1	428,571	1	5,1072	1,143
8,161	1,064	0,932	1	1	282,857	4,5	6,72	0,789
4,697	0,998	1,275	0,428	4,5	334,286	1	3,4944	0,855
6,141	0,341	2,644	0,246	0,789	360	4,5	10,038	1,066
3,337	0,855	0,132	0,123	1	471,429	0	0,8064	0,855
5,869	1,352	4,721	0,123	1	368,571	1	12,264	4,643
16,8	2,495	0,132	2,5	0	120	1	29,6632	0,932
3,284	0,932	2,066	0,123	0	402,857	1	1,344	6,143
6,221	1,339	1,284	0,428	0	180	0,143	13,9944	0,932
2,823	1,275	2,576	0,428	1	351,429	0,789	0	1,066
9,477	0,561	5,352	0,428	0	94,2857	4,5	2,1504	1,143
16,017	2,433	16,864	0,786	0	274,286	1	0,8064	5,289
8,712	4,995	9,35	0,786	0,789	248,571	1	0	1,578
8,229	2,216	1,932	0,123	1	214,286	4,5	54,53	4,643
3,502	2,141	1,932	0,428	1	222,857	4,5	2,7972	1,789
5,427	5,564	5,064	0,428	1	514,286	4,5	49,3192	4,643
7,089	2,93	4,653	0,428	0	137,143	0,789	0,672	5,289
9,635	1,064	4,932	0,428	0	282,857	1	20,0088	0,209
6,928	2,642	1,504	0,428	0,789	94,2857	1	0	0,143
4,126	2,499	5,198	0,123	0,143	102,857	4,5	18,648	4,643
3,642	1,644	0,352	0,428	1	60	0	0	0,143
5,359	2,853	4,864	0,786	0,789	154,286	4,5	9,5172	0,143
5,798	0,638	1,787	0,123	1	214,286	6	0	0,143
4,273	2,13	8,998	0,428	0	171,429	1	27,972	4,643
5,416	2,207	5,71	0,786	0,789	282,857	1	15,0024	4,643
6,868	0	3,578	2	0,789	120	4,5	8,3412	0,143
4,442	1,721	2,721	0,786	0,789	240	FAŁSZ	46,3484	4,643
4,214	3,071	3,627	0,428	0,789	300	0	36,792	0,143

6,441	4,995	1,218	0,428	0,789	42,8571	4,5	0	0
2,131	0,275	2,427	0,428	0	300	0	0	4,643
2,99	1,141	0,921	0,428	1	257,143	0,143	28,6692	0,932
4,058	3,51	7,842	0,428	0	300	1	7,5264	6,143
5,215	2,787	3,207	1,572	0,789	385,714	4,5	26,5804	0,789
6,003	2,293	10,007	0,428	0	308,571	1	10,1472	4,643
2,834	2,642	7,352	1	0	171,429	4,5	71,8116	4,643
7,274	2,987	1,141	4,5	0,789	428,571	0	18,6564	6,789
3,335	0,33	2,275	0,066	0	300	4,5	12,9696	6,143
3,843	2,062	2,141	0,428	0,143	531,429	4,5	86,6292	0,789
8,08	2,708	7,51	0,428	0,789	351,429	4,5	5,376	1,789
4,863	1,561	1,064	1,428	0	231,429	1	43,7976	0,066
6,137	2,218	2,433	0,856	0,429	300	4,5	5,376	0,789
4,214	1,141	2,352	0,123	1	282,857	1	10,038	4,643
4,344	1,053	1,493	0,066	0	248,571	1	6,9972	4,643
4,278	1,644	0,932	0,786	0	154,286	1	20,4876	0,143
5,924	2,216	5,855	0,786	4,5	60	0,789	16,9428	0,789
5,137	1,855	2,13	0	4,5	308,571	4,5	10,2816	5,289
6,794	1,064	0,198	0,428	4,5	180	4,5	1,344	0,066
5,614	2,71	1,141	0,786	0	300	1	23,3016	0,143
6,214	1,427	5,627	0,123	1,066	197,143	0	4,662	4,566
4,781	1,416	7,418	0,123	0	274,286	1	2,7972	0,789
6,873	1,286	9,064	0,428	0	180	4,5	25,8552	4,643
6,357	2,721	7,93	1	4,5	377,143	1	24,528	0
3,634	1,504	9,427	0,551	1	240	4,5	26,1072	5,289
7,007	2,789	5,578	0,428	1	38,5714	1	63,07	6,143
2,697	1,493	1,141	0,428	0	145,714	1	10,8444	0
7,673	0,715	1,572	0,066	0	34,2857	0,143	0	0,572
2,625	2,644	9,216	0,428	4,5	240	1	30,8084	4,566
2,604	1,789	1,198	0,909	0	411,429	1	53,6284	4,643
6,511	1,842	0,987	0,428	0	154,286	6	18,648	0,066
3,351	1,787	13,495	0,786	0	180	1	1,344	1,218
8,923	1,721	0,418	1	1	94,2857	1	0	1,143
2,318	1,776	0,286	0,786	4,5	205,714	1	6,132	0,932
8,094	2,576	2,647	0,123	4,566	300	4,5	35,322	0,429
7,152	2,207	10,576	0,123	1,578	274,286	4,5	67,298	1,218
6,583	2,499	1,721	1	0,789	377,143	0,143	4,5276	1,578
2,832	2,13	7,787	0,066	4,566	240	4,5	0,8064	4,643
5,372	1,787	1,064	0,428	0	531,429	4,5	1,6128	1,143
11,427	5,432	6,864	0,786	0	120	1	0	4,643
7,164	2,433	0,209	0,428	0	325,714	1	30,66	4,643
2,997	1,001	10,073	0,428	0,066	248,571	4,5	4,8384	5,289
6,001	2,642	13,365	0,428	1,066	497,143	4,5	142,8924	1,143
2,504	0,855	2,209	0,428	0	102,857	0,066	0,8064	4,566
5,858	1,853	5,636	0,428	0,143	257,143	1	97,958	4,643
5,711	1,864	1,144	0,428	0	154,286	0	83,9664	0,286
5,004	0,407	1,207	1,572	0	685,714	1	9,954	0,789
2,56	1,141	4,209	0,123	0,143	180	1	18,396	0,143

7,45	2,499	2,855	0,123	0	240	4,5	60,662	4,643
5,507	1,853	1,998	1,214	0,066	137,143	0,429	18,7656	0,143
4,766	0,33	3,352	1	0	265,714	1	6,1824	0,143
4,043	1,779	1,561	0,428	0	120	1	31,9452	1,143
1,977	2,282	4,064	0,066	0	385,714	1	128,282	5,289
2,922	0,143	0,286	0,123	0	368,571	0	0,8064	4,643
7,144	1,93	4,075	0,786	0	394,286	4,5	12,8688	0,143
3,702	1,998	7,776	0,428	1	282,857	4,5	19,3914	4,643
4,555	1,196	5,781	0,066	4,5	205,714	4,5	7,5264	0,789
3,054	1,141	0,209	0,066	0	60	1	33,5664	0,429
11,08	1,787	0,924	0,428	4,5	222,857	1	43,7976	0,789
5,117	0,418	0,132	0,428	1	462,857	0	1,8396	0,132
2,403	1,427	6,499	0,123	4,5	471,429	1	2,9148	4,643
11,017	3,075	7,578	1,572	4,5	282,857	4,5	15,6324	1,143
4,19	0	0,198	0,551	4,5	540	0	0	0,789
7,937	5,784	0,352	0,428	1	222,857	1	8,1732	1,789
5,223	2,062	4,284	0,428	0	497,143	1	5,4852	1,143
3,225	0,932	1,066	1	0	60	1	84	0,143
7,221	2,141	9,13	2,5	4,566	360	4,5	158,6032	0,066
9,995	1,985	4,787	0,786	0,143	214,286	4,5	1,8396	1,218
2,526	1,853	8,141	0,123	4,5	411,429	4,5	10,8612	1,578
5,702	1,625	10,218	0,786	1	240	1	18,648	6,789
5,44	3,156	2	0,428	0,429	497,143	4,5	30,002	5,289
5,444	0,429	0,789	1	0,429	30	0	32,3904	0
4,48	1,064	1,721	0,123	0	265,714	4,5	2,7972	4,643
4,337	1,987	0,352	0,123	0,143	102,857	4,5	96,0064	0,209
2,845	0,847	4,433	0,428	0	257,143	4,5	6,9972	0,143
5,915	1,932	2,064	0,786	0,429	154,286	4,5	20,6556	0,143
3,115	1,644	3,141	0,428	4,5	325,714	0,789	55,9552	0,855
6,652	5,575	1,001	0,428	0	531,429	4,5	15,1872	1,143
10,505	1,713	8,222	1	1,143	445,714	1	58,31	1,578
5,141	1,429	14,867	0,123	4,5	420	0,789	6,9972	0,143
4,913	1,996	1,71	0,428	0,066	531,429	0	0	0,932
6,792	2,565	2,55	1,214	0	428,571	4,5	26,1072	0,066
3,766	1,416	1,064	0,428	0	608,571	1	163,268	0,066
6,443	2,433	1,064	0,428	1	317,143	1	30,002	1,143
6,082	1,853	0,789	0,856	0	274,286	4,5	8,3622	0,572
5,221	1,713	2,721	0,066	0	420	0,066	4,6788	6,066
3,632	2,15	0,998	0,786	0	317,143	1	33,8352	6,789
7,943	2,565	1,207	0,428	1	351,429	4,5	11,0376	0,789
5,48	2,653	3,064	0,428	1	445,714	1	82,6504	4,643
7,022	3,222	2,075	0,428	0	60	4,5	0	0,143
4,621	4,011	2,787	0,551	0,132	325,714	1	5,4852	0,932
6,43	2,559	1,493	0,312	0,429	411,429	4,5	45,9872	1,143
2,062	0,855	0,143	0,066	1	274,286	0	0	0,143
7,08	1,71	2,853	0,428	0	265,714	4,5	108,0072	0,143
6,702	1,484	3,367	0,428	0	162,857	1	3,4944	0,286
5,003	0,855	0,998	0,428	0	462,857	1	11,2392	0,143

5,335	*2,073*	*4,787*	*2,5*	*0,429*	*497,143*	*0,789*	69,93	*4,643*
5,922	*5,355*	*0,132*	*2,5*	*0*	*394,286*	*4,5*	11,13	*6,066*
9,387	*5,432*	*3,932*	*0,428*	*1*	*265,714*	*0*	3,4944	*5,289*
5,506	*1,218*	*1,93*	*0,428*	*0*	*394,286*	*0*	21,2352	*6,789*
7,809	*2,361*	*3,143*	*0,428*	*1*	*248,571*	*0*	32,7992	*1,143*
5,495	*2,073*	*7,504*	*0,428*	*6*	*480*	*4,5*	3,6792	*6,143*
3,77	*2,207*	*3,286*	*0,123*	*0*	*334,286*	*4,5*	5,4852	*4,643*
4,792	*2,007*	*3,209*	*0,428*	*0*	*240*	*0,789*	38,6316	*5,289*
3,853	*1,644*	*7,35*	*0,856*	*1*	*180*	*0*	34,3392	*5,289*
4,992	*1,636*	*1,919*	*0,123*	*0,143*	*334,286*	*1*	4,5276	*6,066*
4,7	*1,77*	*13,209*	*0,066*	*6*	*334,286*	*1*	2,1168	*6,143*
4,916	*1,13*	*9,196*	*0,428*	*0,789*	*120*	*4,5*	4,5276	*0,932*
6,592	*1,075*	*5,578*	*0,428*	*0*	*137,143*	*4,5*	4,3302	*5,289*
4,44	*2,007*	*2,998*	*0,786*	*0*	*11,4286*	*4,5*	0	*4,5*
5,649	*2,216*	*5,93*	*1*	*6*	*325,714*	*4,5*	287,2604	*6,143*
4,272	*1,198*	*7,139*	*0,428*	*4,5*	*240*	*1*	4,032	*5,289*
5,869	*0,998*	*1,218*	*0,856*	*1*	*274,286*	*4,5*	24,7884	*0,286*
8,336	*1,13*	*8,064*	*2,5*	*0*	*394,286*	*1*	12,726	*4,643*
5,724	*3,064*	*6,007*	*0,786*	*0*	*257,143*	*1*	2,688	*0,143*
7,798	*1,284*	*6,064*	*0,428*	*0*	*154,286*	*1*	7,4592	*0,143*
11,283	*2,578*	*0,932*	*0,428*	*1*	*265,714*	*4,5*	114,0076	*0,789*
3,258	*1,075*	*2,998*	*0,123*	*0*	*162,857*	*0*	5,4852	*6,143*
4,937	*1,275*	*1,998*	*0,428*	*0*	*137,143*	*0*	6,132	*4,566*
14,869	*1,776*	*1,273*	*1,214*	*0*	*282,857*	*4,5*	19,1352	*0,066*
4,135	*1,275*	*1,284*	*0,428*	*0*	*120*	*0,429*	0,8064	*4,643*
9,195	*1,207*	*14,218*	*0,428*	*4,5*	*274,286*	*4,5*	10,794	*4,566*
2,626	*1,996*	*1,561*	*0,123*	*1*	*471,429*	*1*	5,5944	*0,143*
13,203	*4,841*	*1,209*	*1*	*0*	*257,143*	*0,066*	2,7972	*1,143*
8,81	*3,367*	*9,575*	*0,428*	*1*	*454,286*	*4,5*	6,9384	*1,578*
2,834	*1,932*	*1,789*	*0,786*	*0,066*	*454,286*	*0*	0,8064	*0,066*
4,219	*0,627*	*5,576*	*0,428*	*1*	*111,429*	*1*	9,324	*1,143*
5,581	*1,721*	*5,578*	*0,786*	*4,5*	*257,143*	*0,066*	2,7972	*1*
4,574	*1,996*	*5,35*	*0,786*	*0,066*	*274,286*	*0*	5,376	*4,643*
5,407	*0,286*	*0,132*	*1*	*0*	*300*	*4,5*	18,6564	*0*
6,796	*1,855*	*0,066*	*0,123*	*1*	*462,857*	*1*	5,4852	*0,143*
3,855	*1,71*	*2,141*	*0,428*	*0,429*	*137,143*	*0,789*	9,3282	*0,932*
5,355	*1,196*	*2,864*	*0,909*	*1*	*291,429*	*1*	7,2282	*0,932*
5,349	*2,139*	*3,064*	*0,856*	*0,789*	*154,286*	*1*	49,6104	*0,932*
2,994	*1,721*	*2,998*	*0,123*	*0,789*	*308,571*	*0*	13,44	*1,218*
2,407	*0,855*	*4,275*	*0,123*	*0,789*	*77,1429*	*0*	12,264	*1*
5,049	*1,842*	*1,275*	*0,428*	*0*	*411,429*	*4,5*	7,707	*0,143*
3,217	*1,996*	*3,273*	*0,856*	*0,429*	*154,286*	*0*	24,528	*0,143*
14,216	*1,286*	*9,207*	*0,856*	*0*	*180*	*4,5*	45,003	*6,789*
1,966	*1,284*	*3,075*	*0,123*	*0*	*274,286*	*0,143*	32,634	*6,143*
6,506	*1,789*	*1,932*	*0,123*	*0*	*180*	*4,5*	2,7972	*0,209*
3,994	*1,064*	*0,429*	*0,123*	*1*	*180*	*4,5*	12,264	*4,643*
5,84	*2,576*	*21,064*	*1*	*0*	*385,714*	*4,5*	3,1836	*1,789*
3,192	*1,207*	*2,921*	*0,066*	*4,5*	*300*	*4,5*	8,064	*6,143*

7,438	1,713	7,218	0,123	0	197,143	4,5	23,926	1,143
4,104	1,064	8,352	0	0,789	162,857	4,5	0,8064	0,209
2,906	1,787	8,286	0,123	0	85,7143	4,5	20,2356	1,143
3,787	1,132	8,286	0,786	0	90	1	5,376	6,789
3,796	2,787	18,643	0,428	1	317,143	4,5	46,648	0,143
6,072	2,565	1,864	1	1,218	240	0	13,8642	0,932
2,755	1,064	2,721	0,856	0	394,286	1	31,9872	0,789
10,94	1,71	4,576	0,551	1	77,1429	4,5	0	1,143
9,431	1,853	2,341	0,246	0,789	342,857	4,5	26,8184	0,789
5,845	1,339	6,572	0	0	120	4,5	14,9184	0,143
3,118	2,721	9,653	0,066	0	300	1	16,9344	4,566
5,493	0,921	0,209	0,066	0,789	334,286	1	23,324	0,789
6,095	1,427	0,286	0,123	0,429	52,1429	0	0	0
10,791	1,132	1,132	2,5	1	462,857	1	66,0044	1,066
6,796	0,704	3,001	0,428	0,855	180	0	22,1242	1,143
5,28	1,919	6,709	0,428	0	240	1	57,974	1,789
7,218	1,855	2,359	0,856	0	162,857	0	3,6792	0,143
8,571	0,987	2,998	0,123	0,789	480	0	1,8396	0,932
4,838	1,13	2,858	0,494	0,066	471,429	0	51,2512	0,789
5,634	1,787	5,341	0,428	0	137,143	1	0	1,143
5,149	2,007	7,284	0,428	1	214,286	0	12,1212	1,143
6,298	1,209	3,218	0,428	0	454,286	1	18,0012	0,143
3,757	1,71	1,064	0,428	1	514,286	0	9,408	0,143
4,642	2,141	11,499	0,428	4,5	257,143	4,5	36,134	4,643
4,489	2,075	7,796	0,428	1,143	214,286	1	13,986	1,143
6,849	0,132	2,721	0,246	1	154,286	0	0,8064	4,643
6,078	3,576	1	0,909	6,789	514,286	0	2,1504	6
1,889	0,198	0,264	0,066	0,429	454,286	0,789	6,9972	0,066
5,926	0,418	1,275	0,123	1	308,571	1	27,3252	0,132
4,55	5,421	4,495	0,428	0	214,286	4,5	13,9944	0,143
10,211	0,921	0,352	0,786	0	120	1	4,5276	0
5,785	2,075	4,576	0,428	0,143	240	4,5	13,9944	1,789
3,613	0,561	1,998	0,551	4,566	265,714	1	1,8396	1,143
4,5	2,71	3,638	0,428	0	120	4,5	3,4944	1,143
1,757	1,141	0,855	0,856	0,789	180	4,5	9,2484	0,066
5,005	0,275	1,066	0,123	1	240	0	18,396	0,209
2,194	1,427	5,132	0,428	0	222,857	4,5	57,5582	0,932
6,801	1,855	0,418	0,856	0,066	420	1	16,4052	0,143
4,986	1,207	1,352	0,132	1	317,143	4,5	10,794	0,143
5,287	1,196	0,987	0,123	1	102,857	1	2,688	1,143
9,991	2,776	4,132	2	1	120	6	93,3296	1,143
6,35	1,284	3,787	0,428	1	60	1	18,396	4,643
13,429	1,776	1,218	0,428	0	188,571	1	0,8064	0,143
4,344	1,133	4,787	0,123	1	334,286	4,5	4,599	4,643
2,117	1,075	8,066	0,428	0	240	1	4,8384	5,289
3,482	1,473	8,429	0,428	0	360	0	0	5,289
3,137	0,352	1,418	0,428	0	514,286	0	0	0,932
2,983	0,627	6,275	0,428	0,143	77,1429	1	8,064	1,143

4,137	1,644	6,209	0,428	0,429	171,429	4,5	14,6076	1,789
7,794	2,653	5,998	1	1	120	1	6,132	5,289
5,48	1,996	6,719	0,189	0	257,143	1	7,5264	0,932
4,84	1,209	1,132	0,428	0	240	1	8,064	0,789
3,632	0,638	0,066	0,428	4,5	342,857	1	0	0,143
5,574	1,638	2,998	0,428	1	462,857	1	1,8396	4,566
6,849	1,864	2,264	0,551	0,066	197,143	1	22,7556	0,143
7,361	3,365	4,932	0,428	0	137,143	0,789	37,296	9
5,135	1,998	1,133	0	0	617,143	4,5	18,648	0,143
10,929	0,407	3,787	0,123	0,855	120	0	4,662	0,932
5,9	3,365	3,93	0,428	0	265,714	1	24,0744	1,789
4,137	1,141	3,273	0,786	0	480	1	39,984	0,143
4,562	2,139	0,198	0,786	4,5	154,286	4,5	3,066	4,643
4,567	1,93	8,715	0,428	0,143	197,143	4,5	37,296	1,143
4,5	0,998	6,638	0,428	0	214,286	1	0	0,143
2,482	1,207	6,352	0,428	0,143	60	6	0	1,789
7,152	1,853	1,341	0,856	4,5	257,143	4,5	0	4,643
8,939	2,141	10,209	2,5	0	351,429	4,5	30,5116	1,789
4,774	0,418	0,921	0,123	0	222,857	1	1,8396	1,143
3,58	1,35	4,064	1	0,789	154,286	1	1,89	1,143
3,415	2,075	1,284	0,786	0	505,714	0	1,8396	0,495
4,127	1,644	5,132	0,856	0	180	0,429	15,5232	1,789
4,413	2,719	5,484	0,428	4,929	394,286	1	1,8396	6,143
8,788	1,273	7,855	0,428	0,789	154,286	0	0	4,566
6,226	2,576	4,721	0,428	0,789	274,286	0	27,972	0,932
9,637	1,572	4,572	1	0	205,714	1	23,324	1,789
3,72	2,139	14,561	0,123	4,5	180	1	24,5952	4,643
4,346	1,416	4,864	0,674	0	274,286	1	11,9952	0,932
5,583	1,787	0,847	0,123	0	197,143	0,143	99,974	5,289
7,366	2,51	3,864	0,428	0	197,143	1	15,6324	0,932
9,22	1,996	4,932	0,189	4,5	274,286	4,5	5,5944	0,143
10,65	2,282	3,075	0,856	4,5	257,143	0,789	35,378	4,643
6,074	1,787	2,132	1,066	0	120	4,5	0	0,066
4,355	1,064	1,141	1	0	257,143	1	2,7972	1,789
4,344	2,436	3,787	0,066	1	231,429	1	20,4372	4,643
4,565	2,719	11,499	0,123	0,789	180	4,5	9,408	1,066
5,298	2,939	1,644	1	1,143	514,286	0	0	0,066
3,904	0,627	6,561	0,123	4,5	154,286	1	3,1836	6,143
7,511	2,71	4,642	0,428	0	107,143	4,5	60,662	5,289
4,058	1,064	3,495	0,428	0	137,143	4,5	2,688	4,643
7,216	1,776	2,998	1	1	154,286	0	7,5264	1,066
8,921	2,719	8,284	0,428	0	317,143	4,5	6,9972	1,578
9,057	1,842	1,998	0,428	4,5	60	0,789	3,5784	4,643
3,139	1,275	4,776	0,428	0	394,286	4,5	23,9232	0,066
7,363	1,787	3,721	0,428	0,789	90	1	0	5,289
3,651	1,721	0,132	0,123	0	445,714	4,5	7,2156	0,143
6,144	1,196	9,484	0,428	0	411,429	4,5	54,1352	0,855
6,142	1,921	2,209	0,428	0	411,429	1	11,6592	0,143

4,344	1,141	2,638	0,369	0,789	205,714	0	15,9894	0,286
5,702	2,853	2,15	0,428	0	248,571	1	49,4452	1,066
11,874	2,075	10,209	0,246	0	394,286	1	0	0,789
5,992	3,576	1,578	0,852	0	480	4,5	156,0104	1,578
8,925	1,207	4,787	0,856	0	265,714	4,5	10,1304	1,789
9,422	1,13	0,341	0,123	1	411,429	1	48,7592	0,143
13,983	1,998	13,078	0,856	0	291,429	1	13,9944	4,566
12,577	2,139	14,355	0,123	0,789	368,571	1	10,8948	1,578
4,355	1,493	4,578	0,428	0	231,429	1	540,036	0,143
2,403	2,502	7,855	0,066	0	480	4,5	233,24	5,289
6,432	2,284	16,775	0,428	0	214,286	4,5	12,264	5,289
4,478	1,341	3,141	0,066	4,5	445,714	0	7,5264	4,643
5,427	1,427	1,864	0,428	0,789	257,143	0	60,8104	0,932
5,424	2,578	2,341	1,786	0	282,857	1	13,608	5,289
5,724	0,429	2,207	0,428	0	180	4,5	53,984	1,143
4,981	1,999	9,361	0,428	1	137,143	0,789	11,662	6,789
6,295	2,139	10,007	0,428	0	377,143	4,5	26,1072	5,289
8,506	5,861	2,932	0,428	0	94,2857	4,5	9,408	5,289
4,124	0,855	0,132	0,428	0	205,714	1	7,0392	0,932
13,498	0	1,075	0,123	1	497,143	4,5	23,926	1
3,544	1,636	6,007	0,066	0	394,286	1	12,9696	6,143
11,655	2,15	4,585	0,428	4,5	214,286	0	38,0184	5,289
7,856	1,352	2,578	0,856	0	248,571	4,5	97,4372	0,789
3,898	1,35	3,493	0,123	4,5	420	1	12,7512	4,566
3,766	2,271	0,495	0,428	0,429	514,286	1	13,0872	6,789
3,775	1,273	5,787	0	0,429	240	1	0	5,289
5,506	1,787	3,141	0,428	0	197,143	1	0,8064	4,643
3,623	2,994	3,064	0,786	0	385,714	0,789	13,0704	6,143
14,209	2,653	11,13	0,428	0,066	265,714	4,5	29,5204	5,289
2,834	1,787	1,71	0,856	4,5	120	1	2,688	1,789
14,341	2,499	1,932	2,928	0	120	4,5	13,9944	4,566
4,715	1,075	4,721	0,246	0	274,286	1	51,2904	6,143
5,783	1,919	2,418	0,428	4,566	257,143	1	89,3732	0,209
3,258	1,864	1,919	0	0	197,143	4,5	22,6632	1,143
6,131	5,289	5,721	0,856	1	300	0,789	8,6688	4,566
7,088	2,286	0,429	0,428	0	205,714	1	10,038	0,143
6,627	2,064	2,578	1	0	514,286	1	30,002	0,789
4,204	1,998	1,721	0,786	0,143	205,714	0	8,2824	4,643
3,634	1,57	2,075	0,123	1	222,857	4,5	2,688	4,643
6,572	1,853	2,352	0,428	0	137,143	0	9,1896	5,289
3,129	0,858	3,299	0,428	0	480	4,5	29,6716	0,143
12,417	1,919	2,787	0,428	0	428,571	1	39,6396	0,143
7,021	1,864	2,001	0,428	0	222,857	4,5	31,6512	0,789
3,68	2,644	0,561	1,572	0,789	565,714	4,5	148,3356	0,495
5,061	1,427	0,132	0,428	0,143	394,286	4,5	8,3916	0,143
6,201	3,365	1,273	1,428	0,143	180	4,5	75,3396	1,789
10,033	2,787	2,352	0,428	4,5	248,571	1	5,376	1,789
7,202	0,484	2,341	0,428	4,5	411,429	4,5	9,6768	1,066

6,847	1,273	5,061	0,428	1	214,286	0	4,9056	1,218
5,144	1,284	1,853	1,214	0	222,857	0	4,5696	6,789
1,757	1,13	2,921	0,123	0	360	4,5	22,2684	4,643
4,861	2,862	3,13	0,856	0,855	180	6	30,0846	0,932
5,291	1,064	4,484	0,428	1	325,714	0	69,3112	1,143
7,576	3,365	7,007	0,428	1	137,143	1	152,2164	1,578
5,223	5,575	8,007	0,786	0	377,143	1	0	0,286
6,146	1,853	3,495	0,428	1	240	1	17,7912	0,143
5,584	1,35	9,427	0,428	0,066	368,571	1	6,9972	0,932
4,355	2,51	0,932	0,428	4,5	411,429	1	37,5984	0,932
6,93	1,141	3,141	0,494	1	197,143	1	0	5,289
13,352	1,064	0,132	0,246	0,789	265,714	0	1,6128	0,143
4,109	1,13	0,198	0,551	0	265,714	4,5	71,8116	0,143
3,766	1,996	2,352	0,428	1	137,143	1	26,7232	1,143
5,071	3,367	13,361	0,428	0	360	4,5	76,3112	5,289
8,506	5,061	2,286	0,428	4,5	180	4,5	3,7632	5,289
4,786	0,198	1,066	0,786	1	317,143	1	5,5944	0,066
3,621	0,704	0,275	0,066	0	660	1	12,1212	0,066
10,281	6,573	9,427	0,786	0,789	120	4,5	1,8396	1,789
13,57	2,433	0,429	0,428	0	214,286	0	9,5508	0,143
6,518	3,365	3,436	0,428	0	171,429	4,5	33,726	5,289
7,863	1,853	8,855	0,066	0	480	0	6,132	0,855
4,058	1,427	3,352	0,123	4,5	214,286	4,5	8,3328	4,643
6,147	1,988	24,143	0,428	4,5	171,429	4,5	63,4172	0,789
4,249	1,275	3,855	0,428	0	248,571	1	1,8396	1,218
4,124	1,067	1,13	0,066	0	360	1	14,4592	0,789
6,637	2,007	1,053	0,551	1	368,571	1	12,264	0,932
4,135	0,352	4,998	0,428	0	154,286	4,5	0	0,855
5,992	0,561	9,35	0,428	0	342,857	4,5	1,344	1,143
7,232	1,647	7,482	0,551	0,286	437,143	4,5	20,4876	6,789
6,506	3,365	1,13	2,5	0,789	480	4,5	95,6592	0,932
16,993	4,995	13,075	0,428	0	120	4,5	0	9
7,521	2,367	1,921	0	1	154,286	1	6,4008	4,643
4,344	0,99	1,572	0,428	0	480	4,5	7,4592	1,143
2,062	6,352	19,561	0,551	0	437,143	4,5	21,5922	5,289
4,774	0,33	3,13	0,123	0	300	0	0	1,143
3,273	1,644	0,209	0,123	1	582,857	1	7,9716	4,566
3,423	2,578	4,427	0,428	1,789	394,286	4,5	39,326	0,572
5,216	1,141	10,275	0,428	0	240	4,5	0	1
6,15	2,064	5,427	0,428	4,5	317,143	6	0,8064	0
7,585	1,93	9,153	0,551	0,132	317,143	1	6,1824	4,643
5,649	1,57	6,007	0,123	0,429	197,143	4,5	9,3156	4,643
3,915	2,708	11,93	0,428	0	411,429	4,5	18,396	4,643
11,929	2,642	5,996	0,551	1	197,143	1	25,3512	0,932
5,14	1,776	1,921	1,428	1	137,143	4,5	25,7656	1,789
3,206	1,207	3,561	0,123	0	291,429	1	46,648	1,143
4,834	0,855	0,275	0,428	0	240	4,5	56,7112	0
6,23	2,719	5,427	0,909	0,789	265,714	4,5	16,9764	0,572

6,708	*2,141*	*8,141*	*1,979*	*4,5*	*120*	*4,5*	13,0536	*0,143*
7,655	*2,93*	*4,51*	*0,428*	*0*	*77,1429*	*4,5*	4,5276	*4,643*
6,867	*4,852*	*3,284*	*0,428*	*4,566*	*282,857*	*4,5*	0	*5,289*
7,945	*4,077*	*3,864*	*0,428*	*0*	*77,1429*	*4,5*	42,2016	*4,643*
4,781	*2,787*	*0,932*	*0,786*	*4,5*	*471,429*	*1*	180,012	*4,643*
5,926	*1,776*	*7,284*	*0,428*	*0*	*214,286*	*4,5*	2,688	*1,143*
7,161	*2,642*	*3,864*	*0,428*	*0*	*162,857*	*4,5*	53,6284	*0,143*
2,329	*1,987*	*5,141*	*0,428*	*4,5*	*154,286*	*4,5*	0	*1,143*
5,426	*2,853*	*7,284*	*0,428*	*0*	*325,714*	*1*	0,8064	*5,289*
5,006	*2,499*	*0,143*	*0,428*	*1*	*265,714*	*0*	2,7972	*1,066*
6,427	*1,787*	*1,284*	*0,428*	*1*	*308,571*	*4,5*	30,002	*0,143*
3,469	*0,921*	*2,484*	*0,786*	*0,789*	*471,429*	*0*	49,0392	*4,643*
6,212	*1,932*	*1,286*	*0,428*	*1*	*145,714*	*1*	2,7972	*0,286*
4,355	*1,35*	*5,638*	*0,066*	*0*	*274,286*	*4,5*	17,038	*0,143*
3,128	*2,141*	*2,209*	*0,123*	*0*	*77,1429*	*4,5*	30,758	*5,289*
2,986	*1,853*	*4,064*	*0,123*	*0*	*360*	*1*	12,264	*0,143*
4,847	*1,207*	*9,855*	*0,066*	*4,5*	*394,286*	*1*	20,2356	*6,143*
6,194	*2,13*	*0,495*	*0,856*	*1*	*180*	*1*	9,5172	*4,566*
4,843	*2,064*	*4,787*	*0,428*	*0*	*171,429*	*4,5*	13,44	*6,789*
5,423	*1,209*	*0,638*	*0,428*	*1*	*34,2857*	*1*	5,1282	*0,143*
5,981	*0,77*	*2,644*	*0,428*	*0*	*411,429*	*4,5*	3,4944	*5,289*
7,229	*0,855*	*1,066*	*0,123*	*0,789*	*240*	*1*	4,1412	*0,143*
6,141	*3,288*	*0,932*	*0,428*	*0,143*	*385,714*	*4,5*	23,9232	*0,495*
6,284	*1,702*	*1,495*	*0,428*	*1*	*68,5714*	*1*	30,5004	*0,789*
11,729	*2,93*	*1,855*	*0,428*	*0*	*334,286*	*4,5*	60,6424	*4,643*
2,196	*1,853*	*0,132*	*0,123*	*0*	*771,429*	*4,5*	159,964	*4,566*
4,41	*2,359*	*6,71*	*0,066*	*0,789*	*342,857*	*1*	35,322	*4,643*
1,537	*0,341*	*0,921*	*0,428*	*0*	*68,5714*	*0*	24,528	*1,066*
4,634	*1,132*	*0,638*	*0,123*	*0*	*411,429*	*4,5*	12,3144	*4,566*
8,63	*2,853*	*2,275*	*2,5*	*1*	*120*	*1*	0	*1,066*
10,868	*2,367*	*10,864*	*0,428*	*4,5*	*274,286*	*1*	11,0292	*1,578*
6,361	*2,864*	*0,572*	*1,214*	*0,429*	*300*	*0*	14,35	*0,789*
3,785	*1,919*	*1,075*	*0,428*	*0,066*	*728,571*	*1*	28,7532	*0,855*
6,137	*2,499*	*3,222*	*0,428*	*1*	*325,714*	*6*	22,6632	*1,789*
1,416	*0,932*	*2,209*	*0,123*	*1*	*240*	*0,789*	66,794	*1,143*
5,566	*2,064*	*0,789*	*0,428*	*0*	*257,143*	*0*	21,462	*1,789*
4,865	*1,713*	*8,147*	*0,428*	*0,789*	*334,286*	*0,789*	8,442	*0,789*
4,726	*1,721*	*3,93*	*0,428*	*0*	*308,571*	*1*	46,1496	*0,143*
10,065	*1,218*	*2,075*	*0,123*	*0*	*257,143*	*1*	7,35	*0,429*
8,3	*6,221*	*5,132*	*0,428*	*0,789*	*240*	*1*	13,2244	*0,932*
1,561	*0,921*	*7,15*	*0,551*	*0*	*77,1429*	*4,5*	35,378	*5,289*
5,858	*1,196*	*4,001*	*0,674*	*0*	*282,857*	*1*	84,6524	*0,286*
2,791	*1,427*	*3,578*	*0,428*	*0*	*154,286*	*4,5*	0	*1,143*
4,586	*0,418*	*3,578*	*0,066*	*0,429*	*197,143*	*0,789*	22,0752	*0,286*
6,146	*1,996*	*5,864*	*0,856*	*0,789*	*325,714*	*1*	27,986	*1,143*
4,981	*1,141*	*1,721*	*0,123*	*1*	*77,1429*	*1*	2,688	*4,643*
3,849	*1,921*	*4,998*	*0,909*	*0*	*128,571*	*1*	0,8064	*6,066*
4,693	*1,932*	*5*	*1*	*1*	*265,714*	*4,5*	18,396	*4,643*

4,434	1,71	1	0,428	0	428,571	1	64,9712	4,643
5,777	1,493	0,275	0,428	0	531,429	1	5,8632	0,143
5,328	0,921	0,264	0,428	0	497,143	1	49,3192	0,143
6,858	0,789	4,855	0,123	0	240	1	51,4472	0,143
4,489	1,427	2,273	0,494	0	360	4,5	50,3496	1,143
4,073	1,207	0,418	0	4,5	180	0	17,2564	0,789
10,228	2,064	5,721	0,786	4,5	300	0	23,926	6,143
6,719	0	3,132	0,123	1	120	4,5	5,8632	0,789
4,335	1,141	2,998	1,214	0	462,857	1	15,4392	0,143
6,381	2,367	1	0,428	0	214,286	0,789	22,0752	0,789
4,269	1,93	3,864	0,428	4,566	394,286	4,5	0,2268	1,789
4,928	1,787	3,341	0,428	0	197,143	1	0,8064	1,143
8,157	2,939	5,286	0,786	1,578	394,286	1	18,0012	0,932
9,488	3,222	7,064	0,428	0,066	248,571	0,429	23,436	1,143
10,356	1,209	10,209	0,123	1	257,143	1	2,7972	1,143
6,438	1,93	8,073	0,428	1,066	197,143	0	2,7972	1,143
5,07	2,708	1,57	0,428	1	197,143	4,5	60,004	1,218
9,143	2,853	6,15	0,786	0	531,429	4,5	9,324	0,789
9,481	1,776	6,781	0,123	0,429	214,286	4,5	16,926	6,066
4,722	1,053	2,998	0,428	0	360	4,5	0	0,143
10,158	1,787	2,367	0,428	0	300	1	5,5188	4,5
10,075	1,996	1,855	0,428	1	265,714	4,5	6,3672	4,643
9,728	3,442	3,286	1	0	180	1	5,4852	5,289
5,291	1,644	1,075	0,123	1	162,857	4,5	48,398	0,066
3,115	0,495	6,341	0,123	0	274,286	4,5	36,6492	0,932
2,403	1,57	1,33	0,494	1	351,429	1	25,2182	1,789
2,843	4,929	1,418	0,123	1	325,714	1	6,5604	6,143
3,2	1,207	7,275	0,428	1	522,857	1	120,8144	1,429
4,069	0,781	2,638	0,428	0	317,143	4,5	48,398	0,855
15,565	1,053	3,51	1,066	1	171,429	4,5	0	5,289
5,152	0,704	1,493	0,189	0	300	4,5	20,5464	0,066
5,272	0,484	0,132	0,428	0	377,143	0	10,1304	0,066
4,572	2,708	3,365	0,786	0,143	94,2857	4,5	7,2156	4,643
3,926	1,71	5,275	0,123	0,789	282,857	4,5	0	0,066
5,94	2,064	0,781	0,428	4,5	180	4,5	5,4852	5,289
4,154	0,998	1,132	0,246	1	325,714	4,5	12,8352	0,143
10,217	2,51	2,998	0,428	4,5	222,857	0	7,2156	0,143
5,916	1,776	9,207	0,428	0	154,286	1	18,6592	4,643
5,001	2,433	7,218	1	4,5	240	4,5	0	4,643
6,629	2,719	4,919	0,066	1	454,286	4,5	61,1072	1,143
3,645	1,787	2,075	0,428	1	248,571	0,789	8,5512	0,932
4,434	2,853	2,367	0,428	4,5	308,571	0	8,9292	0,789
3,708	0,789	1,286	0,123	0	402,857	1	6,5604	1,143
5,346	2,064	2,075	1,066	4,5	531,429	1	3,4944	1,789
9,588	2,007	1,578	0,428	0	180	4,5	0	0,932
4,862	1,196	2,132	0	1,143	60	1	19,726	0,286
3,423	1,57	0,998	0,066	0	651,429	1	23,5536	0,143
5,215	2,576	4,141	0,066	4,5	248,571	1	0,8064	1,789

5,576	*2,499*	*2,578*	*0,428*	*0*	*411,429*	*4,5*	16,3884	*0,143*
3,489	*2,996*	*2,721*	*0,428*	*0,209*	*317,143*	*4,5*	180,012	*4,643*
4,267	*1,842*	*7,996*	*0,123*	*1*	*600*	*4,5*	41,1824	*0,932*
7,726	*5,421*	*2,427*	*0,494*	*0,789*	*162,857*	*1*	0,8064	*1,578*
15,503	*0*	*2,367*	*1,712*	*0*	*411,429*	*1*	46,648	*1,578*
6,058	*1,207*	*0,143*	*1,123*	*1,066*	*197,143*	*0*	10,2732	*1,578*
5,87	*1,141*	*3,578*	*0,428*	*0*	*480*	*4,5*	11,662	*5,289*
3,557	*1,35*	*7,51*	*1,428*	*0,789*	*360*	*0,789*	22,1592	*0,143*
3,052	*2,644*	*9,996*	*0,428*	*0*	*214,286*	*0*	29,5512	*4,643*
6,661	*1,721*	*1,789*	*0,428*	*0*	*300*	*0,789*	9,198	*5,289*
6,726	*2,433*	*1,638*	*0,066*	*0*	*685,714*	*4,5*	18,0012	*0,789*
5,492	*1,341*	*4,207*	*0,856*	*0*	*497,143*	*1*	4,8384	*4,643*
7,004	*0,932*	*0*	*0,428*	*1*	*180*	*0*	3,4944	*0,143*
5,778	*3,51*	*2,99*	*0,123*	*0,789*	*197,143*	*1*	12,012	*0,789*
6,586	*1,141*	*2,789*	*0,428*	*1*	*205,714*	*4,5*	0	*0,789*
6,117	*0,616*	*5,644*	*0,551*	*1*	*51,4286*	*4,5*	21,0672	*1,789*
3,197	*0,561*	*4,207*	*0,428*	*0,066*	*257,143*	*1*	0	*0,932*
5,695	*2,139*	*4,561*	*0,428*	*0*	*428,571*	*1*	25,2112	*6,066*
3,748	*1,207*	*7,396*	*0,856*	*6*	*240*	*1*	0	*0,143*
3,843	*1,284*	*0,209*	*0,123*	*0*	*154,286*	*4,5*	11,0796	*1,143*
5,425	*2,499*	*4*	*0,428*	*1*	*214,286*	*0,789*	26,1212	*1,143*
5,559	*2,51*	*0,855*	*0,786*	*6,066*	*94,2857*	*6*	25,3344	*0,572*
4,574	*1,855*	*0,932*	*1*	*1*	*300*	*0,789*	0,8064	*1,578*
5,12	*1,919*	*1,341*	*0,123*	*0*	*222,857*	*1*	3,6792	*0,143*
7,92	*1,987*	*3,209*	*0,428*	*1*	*162,857*	*4,5*	12,8352	*0,789*
4,99	*1,93*	*3,864*	*0,428*	*0*	*411,429*	*0,789*	15,4308	*5,289*
9,576	*2,853*	*3,495*	*0,428*	*1,066*	*180*	*1*	5,5944	*0,789*
4,214	*1,13*	*2,418*	*0,123*	*1*	*514,286*	*1*	1,8396	*0,495*
3,346	*1,776*	*8,786*	*0,428*	*4,5*	*471,429*	*4,5*	7,4592	*5,289*
8,571	*1,787*	*3,352*	*0,123*	*0,789*	*197,143*	*4,5*	5,376	*6,143*
8,129	*1,504*	*6,429*	*0,428*	*0*	*205,714*	*4,5*	11,662	*5,289*
4,572	*1,71*	*3,921*	*0,123*	*1*	*394,286*	*4,5*	5,754	*5,289*
10,303	*2,855*	*2,075*	*0,428*	*0*	*265,714*	*4,5*	27,72	*5,289*
9,796	*1,361*	*0*	*0,428*	*1*	*180*	*4,5*	18,0012	*0*
2,417	*1,787*	*1,71*	*0,123*	*1*	*325,714*	*0*	0	*0,789*
5,217	*0,704*	*9,132*	*0,123*	*0*	*428,571*	*4,5*	8,064	*0,143*
10,993	*3,13*	*2,275*	*0,856*	*1*	*497,143*	*4,5*	46,648	*6,143*
2,972	*0,275*	*6,77*	*0,066*	*1*	*420*	*1*	0	*4,643*
3,698	*1,776*	*6,209*	*0,123*	*6*	*360*	*4,5*	0,8064	*6,143*
6,139	*1,141*	*0,198*	*0,856*	*0,789*	*497,143*	*4,5*	183,9796	*5,289*
11,653	*2,787*	*10,864*	*0,428*	*1*	*317,143*	*4,5*	7,35	*5,289*
7,988	*0,198*	*0,132*	*0,066*	*1*	*162,857*	*4,5*	0,8064	*0,143*
2,823	*3,053*	*7,198*	*0,428*	*0,132*	*240*	*4,5*	9,408	*0,495*
3,335	*0,572*	*10,715*	*0,123*	*4,5*	*360*	*4,5*	36,0024	*1,143*
6,141	*2,998*	*11,787*	*0,428*	*1*	*137,143*	*4,5*	6,132	*4,643*
2,032	*0,341*	*8,341*	*0,066*	*0*	*308,571*	*4,5*	0	*0,855*
7,375	*1,429*	*0,638*	*1,214*	*4,5*	*214,286*	*1*	0	*0,143*
3,717	*2,075*	*0,209*	*0,066*	*1*	*317,143*	*0,789*	6,9888	*6,789*

4,906	*1,787*	*3,141*	*0,428*	*0*	*351,429*	*4,5*	30,002	*1,143*
5,777	*1,495*	*8,853*	*0,123*	*0*	*154,286*	*4,5*	4,662	*5,289*
7,227	*2,787*	*7,632*	*0,428*	*0*	*411,429*	*0*	34,9832	*0,143*
8,346	*1,864*	*5,855*	*0,123*	*0*	*617,143*	*1*	0	*0,143*
8,922	*1,787*	*3,075*	*0,786*	*1*	*480*	*1*	2,7972	*1,789*
3,192	*1,209*	*1,198*	*0,428*	*0*	*282,857*	*4,5*	15,0024	*1,789*

var 32
0,789
1,209
0,855
0,066
0,143
0,789
1,066
0,143
0,855
1,066
0,066
4,786
0,789
0,143
0,066
0
0,495
0,789
6,072
5,355
1,789
4,643
[truncated: 124,355 more chars]
