# Supplementary material for: Lifestyle elements and risk of metabolic syndrome in adults
Source: PLoS One. 2022 Sep 30;17(9):e0275510. doi: 10.1371/journal.pone.0275510 (PMC9524662; doi:10.1371/journal.pone.0275510)
Supplement: Attachment — Submitted filename: Response to Reviewers.docx [file pone.0275510.s002.docx]

Response to Reviewers

Comments Reviewer 1

| Section | Comments and Suggestions for Author(s) | Our answers |
| --- | --- | --- |
| Anthropometric measurements, assessment of biomarkers, blood pressure and lifestyle data | Typo at bottom of page 3, 'Due differences' probably should be 'Due to differences'. | The typo has been corrected. |
| Anthropometric measurements, assessment of biomarkers, blood pressure and lifestyle data | Need more information in the methods about how the specifics of coffee and food was collected. Was the coffee black or did it include creamers? Response would be very different and potentially contribute to sugar contact. | The respondents were only asked about the amount and frequency of coffee consumption in general. The consumption of sugar and other products was assessed in separate questions. |
| Family history of cardiovascular disease | I have concerns with the language related to family history of cardiovascular disease throughout. Poor lifestyle often contributes to an older family member being diagnosed with disease. This needs to be better clarified and the claims about CV disease being a strong determinant of metabolic health needs to be fixed to be stated as family history being a risk factor and not a determinant. | Information concerning the family history of CVD has been corrected. |
| Results | Tables 3, 4 , and 5 are too big with too much data that isn't necessary. | We agree that these tables are too big. Their size results from the fact that they contain the OR with 95% CI obtained across groups with different numbers of components of MetS. The aim of the backward stepwise analysis used in our study was to present the final model, which consisted of the best possible set of predictors affecting the participants’ metabolic health, without losing the significance of the model. We decided to include all analyzed predictors, even when the ORs were statistically insignificant. In our opinion, this presentation of data is clearer to the readers. However, if the Reviewer believes that the tables should be revised, we can, for example, delete the 95% CI or the variables for which *p* >0.05 in order to reduce the amount of data shown in the tables; instead, we can provide these data somewhere else, for instance in the table legends. |
| Discussion | Missing a reference in the Discussion section, page 15, third paragraph about those who quit smoking tend to increase calorie intake - include citation for this. Also in the Discussion, paragraph 4 states that this study confirms.... but it can't because it all associations and looking at risk for metabolic syndrome. Fix this claim. | As per the Reviewers suggestion, the missing references were added. |
| Discussion | Page 16 needs a sentence re-worded - 'women who limited animal fats were more likely not to have metabolic disorders.' This is difficult to read, re-word to a positive flow. | The sentence has been corrected. |
| Discussion | Page 16 needs a reference for the statement that people who sleep longer, in a lying position longer, have a higher risk. This seems like a stretch, were is the reference? | As per the Reviewers suggestion, the missing references have been added. |
| Title | I also recommend changing the title to include the word 'risk' somewhere as the methods and data are all risk of development. | We decided not to include the word ‘risk’ in the title, because the primary aim of our study was to investigate which elements of lifestyle are associated with metabolic health (absence of MetS components) in adults aged 40-65 years. Therefore, our aim was to find health determinants, rather than risk factors.  Our approach, which focuses on elements of lifestyle that help to maintain metabolic health, follows the concept of ideal cardiovascular health promoted by the American Heart Association [Lloyd-Jones et al. 2010; Labarthe2012; Tsao et al. 2022]. This approach can also be used as the leading strategy in the design of innovative prophylactic and educational programs aimed at reinforcing healthy behavior among society. |
| Conclusions | Would be good to have more connection to why this work is needed and how it adds to the literature as it is already well-known that healthy behaviors can prevent and treat diseases. | As per the Reviewer’s suggestion, we have added more information to the summary about why the work is needed and its contribution to the literature. |

Comments Reviewer 2

| Section | Comments and Suggestions for Author(s) | Our answers |
| --- | --- | --- |
| Statistical methods/  Results | All the statistical models are unadjusted? Potential confounders, and interactions between the different components should be addressed. In the case that the models are adjusted, the adjustment variables and the description of the variables used should be included. | Univariate analyses showed the effect of individual factors on a group of participants with 0, 1, 2, 3 or more MetS components, and were not adjusted. Conversely, the aim of the backward stepwise analysis was to present the final model, which consisted of the best possible set of predictors that affected the participants’ metabolic health, without losing the significance of the model. The vast number of variables and cases allowed us to use the aforementioned procedure. This is why all potential variables that may have constituted the group of covariates were shown in both types of regression models. |
| Anthropometric measurements, assessment of biomarkers, blood pressure and lifestyle data | Dietary assessment should be described. How nutritional data was collected? Using FFQ? 24R? 3-days register? Please, provide this information in the methods section. | Description of the dietary assessment has been added, as per the Reviewer’s suggestion. The description is also provided below.  Information on smoking, consumption of alcohol, coffee and other food products, physical activity, sedentary behavior, and sleep duration was collected during face-to-face interviews. Dietary data were collected using the Frequency Questionnaire (FFQ), which consisted of a list of 67 products. The questionnaire was constructed based on a previously designed and validated FFQ for the Polish arm of the PURE study. The questionnaire was highly accurate and repeatable compared to the reference method [Dehghan, M.; Ilow, R.; Zatonska, K.; Szuba, A.; Zhang, X.; Mente, A.; Regulska-Ilow, B. Development, reproducibility and validity of the food frequency questionnaire in the Poland arm of the Prospective Urban and Rural Epidemiological (PURE) study. J. Hum. Nutr. Diet. 2012, 25, 225–232.]. The respondents declared how frequently they consumed a standard portion of each product over the preceding year. |
| Study population/  The definition of metabolic health/  Results | Baseline characteristics of the study participants should be included, including also medication status | The Study section describes the participants’ baseline characteristics, including medication status. This information was also taken into account when the patients were qualified for the presence of each MetS component, in accordance with the IDF definition of MetS. |
| Anthropometric measurements, assessment of biomarkers, blood pressure and lifestyle data/  Limitations | Is it possible to include nutritional data (caloric intake, fiber, fatty acids profile)? Total energy intake should be included as a covariate in the adjustment. | The questionnaire we used in our study did not allow for a precise calculation of the calorie content or the content of other nutrients in the participants’ diet. This information has been provided in the limitations of the study. |
| Results | Tables are not easily readable. Maybe it is necessary to add borders in order to clarify the data (each factor, quartiles, etc.). | As per the Reviewer’s suggestion, for better readability, borders between the rows and columns with data have been added to Tables 1 and 2. |
| Results | Table 3: Use the same order to present data (Q4, Q3, Q2, Q1 or Q1 to Q4). | The order of the data has been changed according to the Reviewer’s suggestion. |
| Anthropometric measurements, assessment of biomarkers, blood pressure and lifestyle data | Limitations: only physical activity was self-reported? Please add if dietary data, smoking status and other health outcomes were self-reported. | All lifestyle data were self-reported. The relevant information has been added, as per the Reviewer’s suggestion. |
