# Supplementary material for: Lifestyle elements and risk of metabolic syndrome in adults
Source: PLoS One. 2022 Sep 30;17(9):e0275510. doi: 10.1371/journal.pone.0275510 (PMC9524662; doi:10.1371/journal.pone.0275510)
Supplement: Attachment — Submitted filename: Response to Reviewers.docx [file pone.0275510.s003.docx]

Response to the Reviewers

Reviewer #1:

**Comments and Suggestions for Author(s):** Most reviewer comments were addressed. There are few that I recommend be further considered before publication.
More inclusion of risk needs to be considered. The purpose or aim isstated to be about association but then the conclusion is about risk of developing metabolic syndrome. Consider adjusting the title or at least adding the work risk to the key words list.
Title suggestion, Lifestyle elements and risk of metabolic syndrome in adults

**Our answer:**

We have changed the title. Now the title is ‘Lifestyle elements and risk of metabolic syndrome in adults’. We have also added ‘risk factors’ to the list of key words.

**Comments and Suggestions for Author’s (Discussion):** Consider the following suggestion for this section
The results of our study confirmed that a healthy lifestyle may significantly increase the odds of being metabolically healthy, with the absence of family history of cardiovascular disease also strongly determining metabolic health.
Suggestion, instead of study confirmed consider changing to study add support that

**Our answer:**

We have tailored the Discussion section to the Reviewer’s suggestion.

The sentence currently reads: The results of our study show that a healthy lifestyle may significantly increase the odds of being metabolically healthy, with the absence of a family history of cardiovascular disease also strongly determining metabolic health.

**Comments and Suggestions for Author’s (Conclusion)
Consider the following suggestion for this section:**

However, the results of our study clearly show that a healthy lifestyle increased the odds of being metabolically healthy, defined as the absence of any component of MetS. Suggestion, if your study aim was to find association, don't conclude
that your study clearly show that a healthy lifestyle increased the odds…should be clearly show an association.

**Our answer:**

The sentence has been corrected. It now reads: However, the results of our study clearly show an association between a healthy lifestyle and being metabolically healthy, defined as the absence of any component of MetS.

Reviewer #2:

**Comments and Suggestions for Author’s:**

Thank you for your answer. I'm not familiar with the statistical approach that you are using. However, i don't think that
these 5 long tables help us to understand the association between life- style factors and MetS severity. Two of them are only frequencies, not relationships. In the case of table 3, the strongest association is observed in alcohol intake (OR: 5.49 (2.23-13.52) <0.001). So, in this OR and 95% CI, you are also considering the other significant factors such as family history of CVD, smoking status, or sweet beveragesintake?? Probably, multiple comparison correction should be applied.

**Our answer:**

According to the reviewer’s suggestions, we showed only multivariate analyses with backward stepwise selection. Univariate analyses were performed, but the data were not shown in order to make the tables more readable and much shorter.
